# Global research trend of esophageal squamous cell carcinoma from 2012 to 2022: a bibliometric analysis

**DOI:** 10.3389/fonc.2022.977935

**Published:** 2022-08-11

**Authors:** Zehua Zhang, Zhuyun Leng, Kang Fang, Mingchuang Sun, Zhaoxing Li, Le Kang, Tao Chen, Meidong Xu

**Affiliations:** ^1^ Endoscopy Center, Department of Gastroenterology, Shanghai East Hospital, School of Medicine, Tongji University, Shanghai, China; ^2^ Department of Gastroenterology, Changhai Hospital, Naval Medical University, Shanghai, China

**Keywords:** esophageal squamous cell carcinoma, ESCC, bibliometric analysis, global trend, hotspot

## Abstract

**Background:**

Esophageal cancer is currently a worldwide health problem. Esophageal squamous cell carcinoma (ESCC) is the most common pathological type of esophageal cancer, and its treatment methods and therapeutic effects are relatively limited, so it also requires the unremitting efforts of basic and clinical researchers to overcome difficulties. Bibliometric analysis can help sort out global research trends and hotspots, but no bibliometric analysis of ESCC has been reported. Therefore, we performed this study to analyze the global trends and potential hotspots of ESCC to indicate future research directions.

**Methods:**

The articles related to ESCC research were collected from the WoS Core Collection SCI-EXPANDED database from 2012 to 2022. The article information was analyzed by BiblioShiny and VOSviewer. Results were presented as bar and network visualization to describe the current trend of ESCC research. This was a retrospective study evaluating data that is publicly available online and at libraries and institutional review board approval, as such, was not demanded.

**Results:**

The global publication trend illustrated a strong growth in the ESCC research field (annual growth rate of 11.4%) and the citation trend increased from an average of 2.98 citations per article per year in 2012 to an average of 3.84 citations per article per year in 2019. With the corresponding author’s country, China contributed the largest number (5,063 articles). The scholars from China and USA had the most collaboration (427 times). China had the largest number of institutions conducting ESCC research. Oncotarget, Oncology Letters, and Annals of Surgical Oncology published the most articles, while Cancer Research, International Journal of Cancer, and Journal of Clinical Oncology had the most local citations. Furthermore, the clinical research hotspots involved in the treatment of ESCC and the basic research hotspots involved in tumor malignant phenotype have received the most attention in recent years.

**Conclusion:**

Our study demonstrated that the research of ESCC has developed rapidly in recent years, and the academic institutions in China have played a decisive role in this field. The global research purpose is to find effective therapies against ESCC, so some emerging hotspots related to ESCC treatment, such as endoscopic therapy, chemoradiotherapy, immunotherapy, tumor microenvironment, and the epithelial-mesenchymal transition will receive more attention and develop rapidly in the future.

## Introduction

Esophageal cancer (EC) is an aggressive malignancy with an overall 5-year survival rate of less than 20%, accounting for over 400,000 deaths worldwide annually (1, 2). EC mainly includes two epidemiologically and pathologically distinct subtypes: esophageal squamous cell carcinoma (ESCC) and esophageal adenocarcinoma (EAC) (3). Among these two subtypes, ESCC’s incidence accounts for 70% of EC (4) and has various risk factors, such as smoking, alcohol, thermal damage, and micronutrient deficiency (5). Currently, the treatment options for ESCC are limited, including surgery, chemotherapy, and radiotherapy (5). Endoscopic treatment of early cancer has received more attention in recent years and has been considered to be the main development direction of EC treatment, such as endoscopic submucosal dissection (ESD) and endoscopic mucosal resection (EMR) (6). In the future, more basic or clinical research is still needed to improve the therapeutic effect of ESCC.

ESCC develops from the esophageal squamous epithelial cells, experiences basal cell hyperplasia and dysplasia (low to high grade), and finally to carcinoma *in situ* (Tis) (5). The molecular mechanism of ESCC development is still unclear, but *TP53* (encodes P53) and other genes involved in cell cycle regulation are abnormal expressions in ESCC, such as *CDKN2A* (encodes cyclin-dependent kinase inhibitor 2A) and *RB1* (encodes retinoblastoma-associated protein) (7). Recently, several large-scale sequencing and multiplatform studies have demonstrated some genes closely related to the occurrence and development of ESCC, such as *TNFAIP3* (encodes tumor necrosis factor-induced protein 3), *CHN1* (encodes chimerin 1), *KMT2D* (encodes lysine methyltransferase 2D) and *NFE2L2* (encodes nuclear factor erythroid 2-like 2), *EGFR* (encodes epidermal growth factor receptor), etc. (8–10). Furthermore, some cellular signaling pathways regulate the growth and invasion of ESCC and are considered potential targets for drug therapy, such as the Hippo signaling pathway, Notch signaling pathway, and EGFR signaling pathway (9, 11). But so far, no molecule or pathway-targeted drug can provide a truly effective treatment for ESCC.

Bibliometric analysis is a quantitative analysis of global academic publications at the level of countries, institutions, individuals, author keywords, etc. (12, 13). With the continuous academic output of various countries in the world, the number of existing literatures in different disciplines are very huge, and the traditional review articles are difficult to summarize the overall development trend and research hotspots of a certain discipline. Recently, bibliometric analysis has been employed in different disciplines to analyze research development, global trends, and hotspots (14, 15). The advantage of bibliometric analysis is that it can analyze a large number of publications in the same research field, extract information such as countries, institutions, authors, etc. to clarify their influence in the field, and it also can analyze the author keywords that represent the core of these studies to identify changes in research hotspots. The results can illustrate potential research hotspots in this field and point out future research directions for scholars. The Web of Science database is one of the most commonly used databases in scientific research and covers a large number of high-quality literatures worldwide (16, 17). Therefore, it is an ideal choice for bibliometric analysis in various research fields. BiblioShiny (Bibliometrix) and VOSviewer are two software that has been used for bibliometric analysis to visualize and network the analysis results and show the global trend and hotspots in a particular research field (18, 19).

In recent years, ESCC research has developed rapidly, and there are thousands of related publications in the Web of Science database. However, no bibliometric analysis of ESCC has been published. Existing bibliometric analysis literature involving ESCC mostly focuses on EC. Powell A.G. et al. analyzed the 100 most influential publications on EC (20). Miao Y. et al. analyzed esophageal and esophagogastric junction cancer from 2007 to 2016 (21). Because these analyses include EAC-related publications, they cannot accurately show global trends and hotspots in ESCC research. Therefore, we performed a bibliometric analysis to outline the global trends of relevant publications in this research field. Our findings will indicate the main research directions for future work.

## Materials and methods

### Literature search strategy

This was a retrospective study evaluating data that is publicly available online and at libraries and institutional review board approval, as such, was not demanded. The Web of Science (WoS) Core Collection Science Citation Index Expanded (SCI-EXPANDED) database was used to comprehensively search relevant publications from January 1, 2012, to May 19, 2022. “Esophageal squamous cell carcinoma” or “oesophageal squamous cell carcinoma” was searched as the topic (available publications = 9,188). The languages other than English were excluded (available publications = 9,173) and the document types other than articles were excluded (available publications = 7,274) (22).

### Data collection

Three authors independently checked the remaining articles and determined their quality. All basic information of the collected articles, including author, title, abstract, keyword, journal, address, citation, etc., was downloaded from the WoS Core Collection SCI-EXPANDED database with text formation (22, 23). In addition, three authors independently inspected all the information, and the articles with missing items have been excluded (available publications=7,153).

### Bibliometric analysis

The data of qualified articles were imported to BiblioShiny (the bibliometrix package in R 4.2.0) to automatically analyze the bibliographic information of these articles, including global publishing trend, global citation trend, distributions of country/institution/journal, historical direct citation network, thematic map of author keywords, international collaborations, most local cited journals/articles/references, and most global cited articles. All figures and tables were directly exported from BiblioShiny. VOSviewer (Version 1.6.16, Leiden, Netherlands) was employed to perform a bibliometric analysis and create the co-occurrence networks of author keywords (18, 24): author keywords occurred more than or equal to 50 times (230 keywords in total). The results were shown as three visualizations: network with clusters, network with timeline, and density visualizations. The occurrence number of author keywords was displayed as the circle size, and the link strength was displayed as the thickness of the line. In the network with a timeline, different colors indicated different average publication years. In the density map, yellow indicated the most frequently occurred keyword.

## Results

### The global research trend in ESCC research

**Figure 1:**
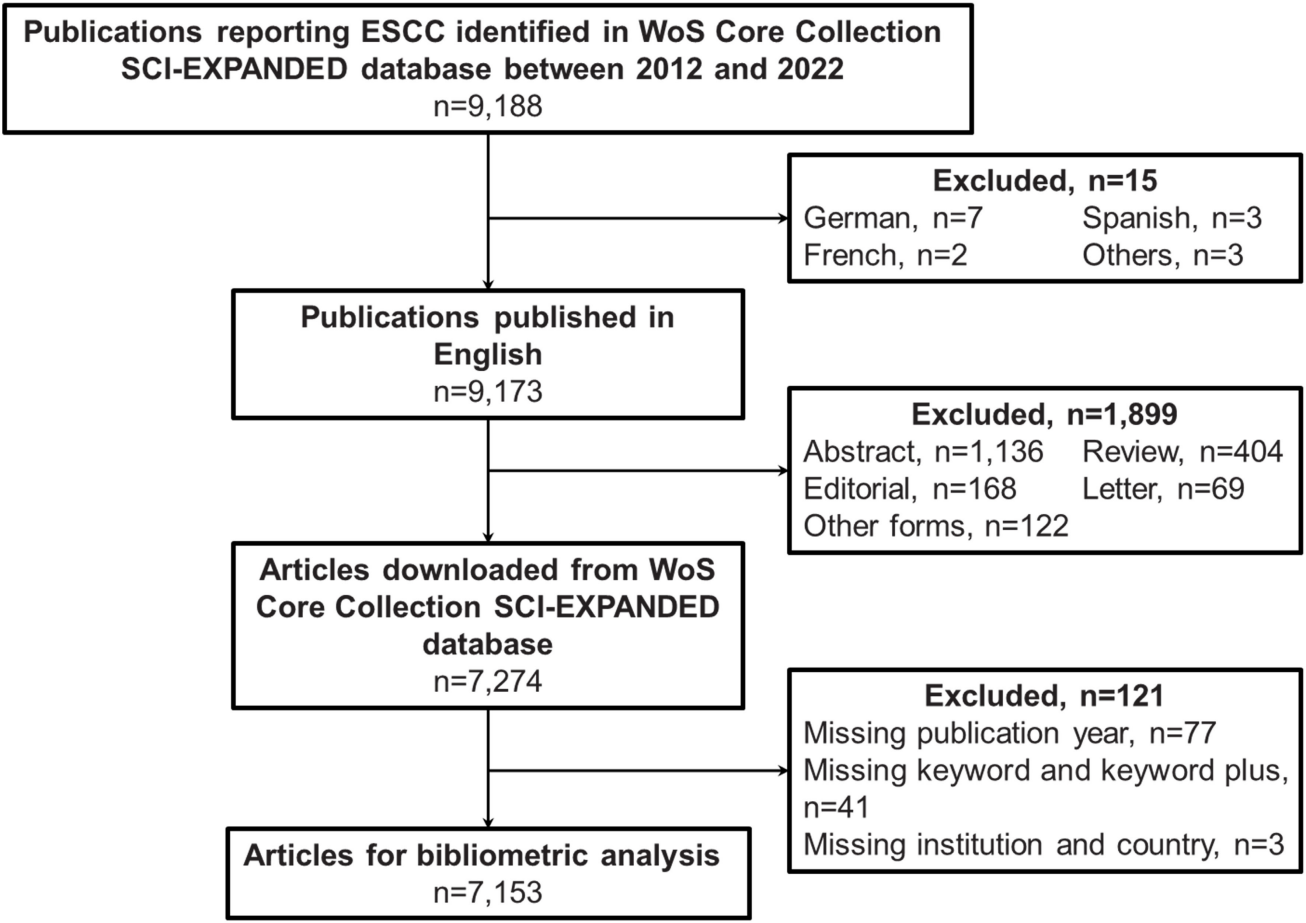
Flowchart for screening eligible articles. WoS, Web of Science; SCI-EXPANDED, Science Citation Index Expanded.

A total number of 9,188 publications on the ESCC topic in the WoS Core Collection SCI-EXPANDED database was identified from 2012 to 2022, 15 publications were removed due to languages other than English (**Figure 1**). Additional 1,899 publications with non-target article types or missing items were excluded, including 1,136 meeting abstracts, 404 review articles, 168 editorial materials, 69 letters, 122 other types of publications, and 121 publications with missing items (**Figure 1**). Finally, 7,153 articles were qualified to perform the bibliometric analysis.

**Figure 2:**
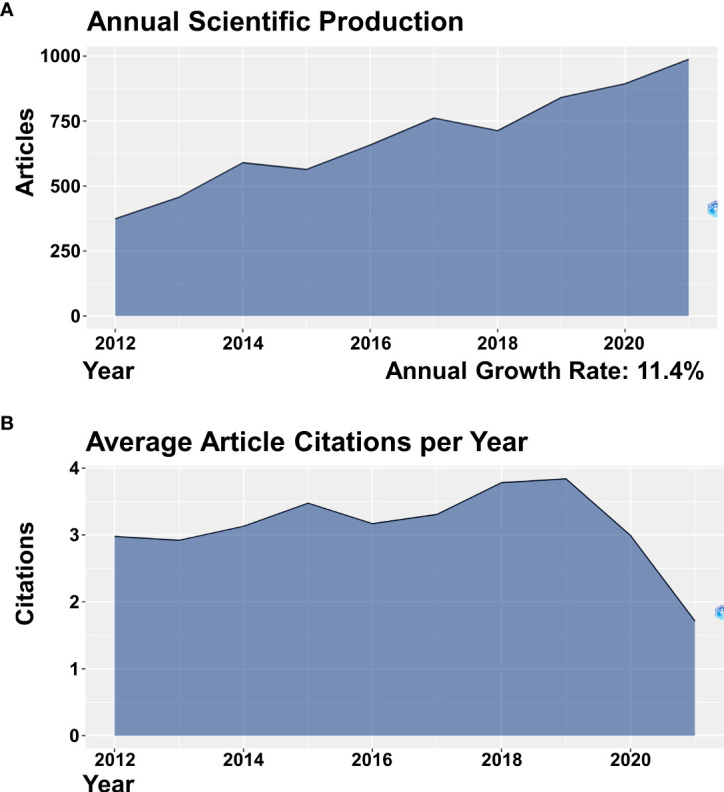
**(A)** Annual scientific production in ESCC research from 2012 to 2021. **(B)** Average article citations per year in ESCC research from 2012 to 2021.

A total of 7,153 finely qualified articles on ESCC over the past decade were analyzed by BiblioShiny and the results showed that the publication number increased from 374 articles in 2012 to 988 articles in 2021, and the annual growth rate arrived at 11.4% (**Figure 2A**). The global publication trend illustrated a strong growth in the ESCC research field. In addition, the citation trend of articles increased from an average of 2.98 citations per article per year in 2012 to an average of 3.84 citations per article per year in 2019 and decreased from an average of 3.84 citations per article per year in 2019 to an average of 1.71 citations per article per year in 2021 (**Figure 2B**). Since the citation of an article is affected by the year of publication, it is normal for the citation frequency to drop in recent years.

### The national and institutional research trends in ESCC research

**Figure 3:**
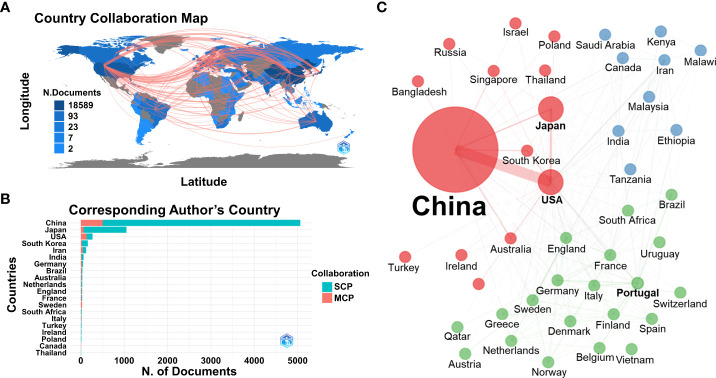
**(A)** World map showing the national collaborations and the volume of related articles. **(B)** Top 20 corresponding author’s countries with the highest number of related articles. SCP, Single country publications; MCP, Multiple country publications. **(C)** Network of national collaborations (color indicated clusters, circle size indicated publication number, thickness of lines indicated collaboration strength).

**Figure 4:**
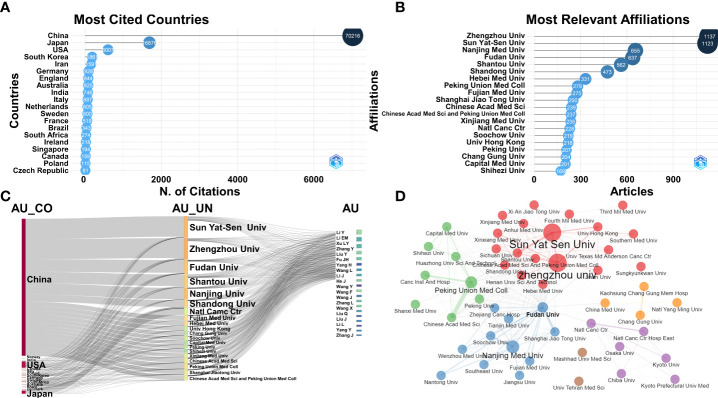
**(A)** Top 20 countries with the most global citations. **(B)** Top 20 institutions with the highest publication number. **(C)** Three-fields plot among countries (AU_CO), institutions (AU_UN), and authors (AU). **(D)** Network of institutional collaborations.

The national and institutional contributions related to ESCC research were analyzed and exported by BiblioShiny as a world map with international collaboration lines (**Figure 3A**). A total of 74 countries published articles in ESCC research from 2012 to 2022. With the corresponding author’s country, China contributed the largest number (5,063 articles), followed by Japan (1,052 articles), the USA (269 articles), South Korea (161 articles), and Iran (123 articles) (**Figure 3B**; **Table S1**). Articles contributed by China were cited the most (70,216 times), followed by Japan (16,876 times), the USA (6,007 times), South Korea (1,861 times), and Iran (1,591 times) (**Figure 4A**). China had the largest number of publications and citations, therefore, contributed the most to ESCC research. The scholars in China played a leading role in this field. Japan, the USA, South Korea, and Iran were ranked in the same number of publications and citations, indicating that these countries have relatively balanced research development related to ESCC. Regarding international collaborations, scholars from China and USA had the most collaboration (427 times), followed by Japan and USA (68 times), USA and Iran (53 times), China and Japan (46 times), and USA and France (42 times) (**Figures 3A–C**; **Table S2**). The results demonstrated that the international collaboration between China and USA was far greater than that of other countries, and the two countries had a large impact on the global research trend of ESCC.

A total of 3,903 institutions published articles related to ESCC from 2012 to 2022. Zhengzhou University and Sun Yat-sen University contributed the largest numbers (1,137 and 1,123 articles), followed by Nanjing Medical University (655 articles), Fudan University (637 articles), Shantou University (562 articles), and Shandong University (473 articles) (**Figure 4B**). China has the largest number of institutions conducting ESCC research (**Figure 4C**). Regarding institutional collaborations, Zhengzhou University, Sun Yat-sen University, and Fudan University had the most collaborations (**Figure 4D**). The global trend of institutional collaborations was dominated by Chinese institutions (**Figure 4D**).

### The distributional trend of journals and articles in ESCC research

**Figure 5:**
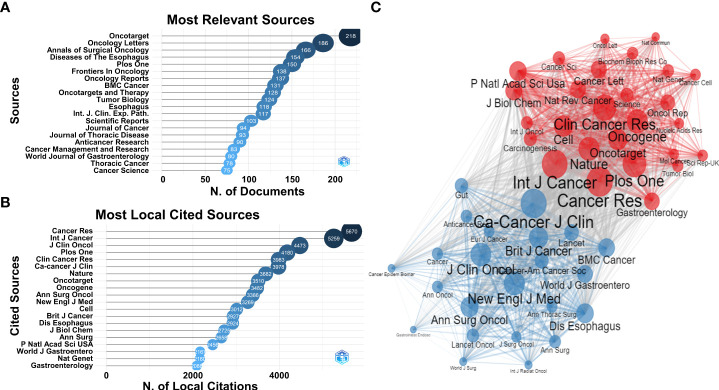
**(A)** Top 20 journals with the highest publication number. **(B)** Top 20 local cited journals from reference lists of eligible articles. **(C)** Network of the co-citation journals cited by eligible articles.

All 7,153 articles related to ESCC research were published by a total of 820 journals. Oncotarget published the most (218 articles), followed by Oncology Letters (186 articles), Annals of Surgical Oncology (166 articles), Diseases of the Esophagus (154 articles), PLOS ONE (150 articles), and Frontiers in Oncology (138 articles) (**Figure 5A**). All 7,153 articles cited a total of 247,879 references (7,906 journals or other sources). Cancer Research had the most local citations (5,670 times), followed by International Journal of Cancer (5,259 times), Journal of Clinical Oncology (4,473 times), PLOS ONE (4,180 times), and Clinical Cancer Research (3,983 times) (**Figure 5B**). Furthermore, we represented a network of the top 50 co-citation journals. Results showed that most of these journals belonged to oncology journals and a small part belonged to gastroenterology journals and top comprehensive journals (**Figure 5C**).

**Figure 6:**
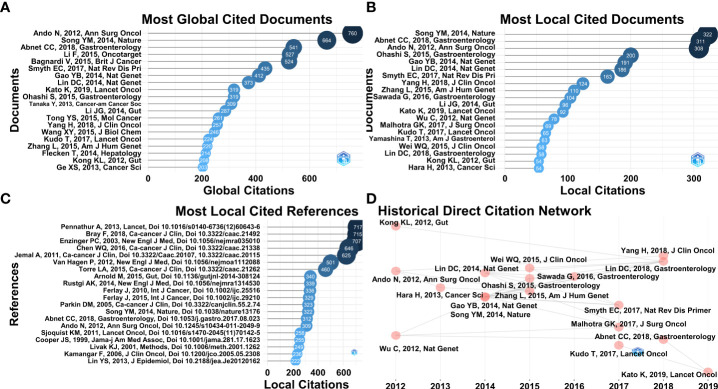
**(A)** Top 20 articles with the most global citations. **(B)** Top 20 articles with the most local citations. **(C)** Top 20 locally cited references in eligible articles. **(D)** Historical direct citation network of top 20 most local cited articles from 2012 to 2022.

All qualified articles were globally cited a total number of 104,428 times and local cited a total number of 23,745 times. The most globally cited article was Japanese clinical research “A randomized trial comparing postoperative adjuvant chemotherapy with cisplatin and 5-fluorouracil versus preoperative chemotherapy for localized advanced squamous cell carcinoma of the thoracic esophagus (JCOG9907)” (760 global citations in total), published in Annals of Surgical Oncology (2012) by Nobutoshi Ando, et al. and correspondence to Nobutoshi Ando (**Figure 6A**) (25). The most local cited article was a Chinese whole-genome sequencing research “Identification of genomic alterations in oesophageal squamous cell cancer” (322 local citations in total), published in Nature (2014) by Yongmei Song, et al. and correspondence to Qimin Zhan (**Figure 6B**) (9). The top 20 most globally or locally cited articles were detailed in **Table S3** and **S4**. Among the total of 247,879 references cited in all screened articles, the most local cited reference was a review article from the USA, “Oesophageal carcinoma” (717 local citations in total), published in The Lancet (2013) by Arjun Pennathur, et al. and correspondence to James D. Luketich (**Figure 6C**) (26). The historical direct citation network of the top 20 local cited articles from 2012 to 2022 was displayed with time annotations (**Figure 6D**).

### The distributional trend of articles and author keywords in ESCC research

**Figure 7:**
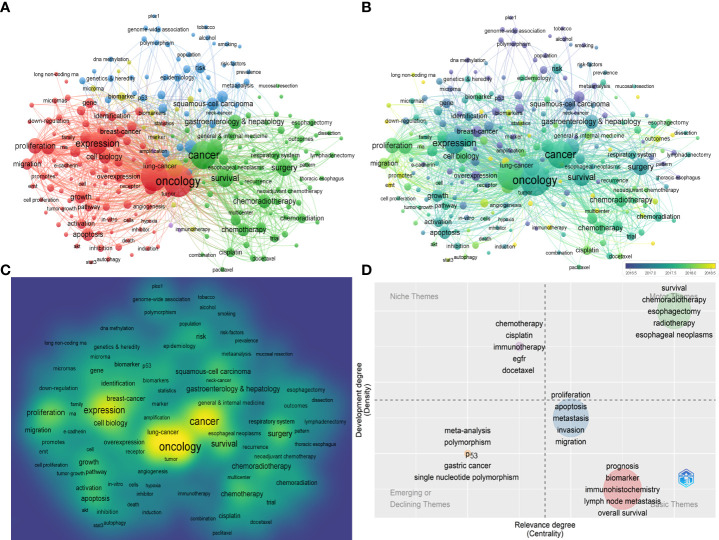
**(A)** Author keywords co-occurrence network with different clusters. **(B)** Author keywords co-occurrence network with average publication year. **(C)** Author keywords density map according to the mean frequency of occurrences (yellow indicated the highest frequency). **(D)** Author keywords thematic map with different clusters (bubble size: the occurrences of cluster keywords) and four domains: Emerging or declining themes; Niche themes; Basic themes; Motor themes. The most common author keywords were used as cluster labels in cluster bubbles.

We employed VOSviewer to analyze the co-occurrence author keywords from the 7,153 articles. 230 keywords that occurred 50 times or more were filtered from a total of 16,617 keywords and displayed as network maps with clusters, average publication year, and density visualization (**Figure 7A–C**). Among the 230 author keywords, the top 10 most frequently occurred keywords were oncology (3,700 times), esophageal squamous cell carcinoma (3,087 times), cancer (2,845 times), expression (1,801times), survival (1,197 times), prognosis (1,088 times), esophageal cancer (1,049 times), surgery (1,030 times), metastasis (870 times) and proliferation (866 times) (**Table S5**). The top 10 most co-occurred author keywords were oncology (total link strength, 25,315), esophageal squamous cell carcinoma (21,170), cancer (19,073), expression (13,503), survival (9,061), prognosis (8,208), surgery (7,209), esophageal cancer (7,191), proliferation (6,915), and metastasis (6,833) (**Table S5**).

Furthermore, we also performed a thematic analysis of the author keywords and represented the results as a two-dimensional matrix with two types of measurements: relevance degree or centrality indicated the strength of external ties to other themes and development degree or density indicated the strength of internal ties among all author keywords describing the research theme (**Figure 7D**) (27). The coupling map has four quadrants: emerging or declining themes (both weakly developed and marginal for a research field), niche themes (highly developed but isolated with marginal importance for the field), motor themes (well developed and important for the structuring of the field), basic themes (important but not developed for the field) (27). Notably, the cluster related to survival, chemoradiotherapy, esophagectomy, radiotherapy, and esophageal neoplasms was positioned in the motor themes field and demonstrated that some clinical research hotspots involved in the treatment of ESCC have received the most attention in recent years (**Figure 7D**). The cluster related to proliferation, apoptosis, metastasis, invasion, and migration nearly entered the motor themes from basic themes and indicated that the basic research hotspots involved in tumor malignant phenotype of ESCC have received more attention recently (**Figure 7D**). In addition, the cluster related to prognosis, biomarker, immunohistochemistry, lymph node metastasis, and overall survival was positioned in the basic themes field; the cluster related to meta-analysis, polymorphism, P53, gastric cancer, and single nucleotide polymorphism was positioned in the emerging or declining themes field; the cluster related to chemotherapy, cisplatin, immunotherapy, EGFR, and docetaxel was positioned in the niche themes field (**Figure 7D**).

## Discussion

EC is a worldwide health problem, especially in Southeast Asian countries (1, 2). ESCC has the highest incidence of EC, and the therapeutic methods have been limited until now (4, 5). Bibliometric analysis has been widely used in various disciplines to assess global trends and hotspots in this research area (28, 29). However, no bibliometric analysis of ESCC has been reported up to now. In our study, we collected and analyzed 7,153 articles from the WoS database related to ESCC over the past decade and described its current academic landscape through bibliometric analysis combined with the bar or network visualization. Our results illustrate a rapid increase in the number of articles on ESCC from 2012 to 2022, and the global trend of article citation also increased from 2012 to 2019. Considering the uncertain difficulty of searching and reading the full text of recently published articles, it is normal for the citation number of articles to decrease from 2019 to 2021 (30).

Regarding national contributions, China has absolute advantages in the number of publications, citations, collaborations, and institutions in this field, and it is currently regarded as a global leader in ESCC research. This research advantage may be due to the high incidence of ESCC in China, and another country with a high incidence of ESCC, Japan, ranks second in the number of publications and citations but ranks third in the number of international collaborations, which still needs to be improved (31, 32). This situation shows that the global research trend of ESCC is mainly oriented by clinical needs and the most popular hotspot is the clinical treatment of ESCC, including survival, chemoradiotherapy, esophagectomy, and radiotherapy, which also verifies this conclusion. However, the incidence of ESCC in the USA is not very high, but it ranks third in the number of publications and citations and ranks second in the number of international collaborations, which proves that the ESCC research in the USA is not oriented by clinical needs, but more academic needs (5). The articles related to the ESCC research field are mainly published in the professional journal of oncology, such as Oncology Letters, Annals of Surgical Oncology, and Frontiers in Oncology. The journals cited by these articles are also dominated by professional oncology journals, such as Cancer Research, International Journal of Cancer, and Journal of Clinical Oncology. These results illustrate that the academic research of ESCC may be relatively closed, and the interdisciplinary research with other disciplines still needs to be improved. Thirteen of the 20 most globally cited articles are duplicated with the 20 most locally cited articles. Among these articles, 13 are clinical and epidemiological studies and 12 are basic research studies. However, some basic research is related to the diagnosis, prognosis, and genetic analysis of ESCC patients, and the ultimate purpose is to guide clinical treatment (33–35). At present, several large-scale genomic and epigenetic analyses of ESCC patients have been published (9, 33, 36). The basic research of ESCC is fertile and has clear directions, so the number of basic studies may be greatly increased in the future.

Our keyword co-occurrence analysis indicates that current ESCC research mainly focuses on clinical treatment (survival, prognosis, surgery, chemoradiotherapy, therapy, and risk) and malignant phenotype of ESCC cells (metastasis, proliferation, invasion, apoptosis, migration, and overexpression), and mainly involves the disciplines of Gastroenterology and Hepatology, Cell Biology, Research and Experimental Medicine, Biochemistry and Molecular Biology. These results present the characteristics of basic studies to assist the clinical practice with the purpose to address ESCC patient needs. Our thematic analysis also verifies the above conclusions. Some clinical research hotspots related to the treatment of ESCC are characterized by being well-developed and important for the structuring of this field, such as survival, chemoradiotherapy, and esophagectomy. Esophagectomy is the most important treatment for ESCC. Several studies have compared the therapeutic effects of right-sided, left-sided thoracoabdominal, and transhiatal approaches with open or minimally invasive techniques (37). The transhiatal approach with minimally invasive technique showed fewer pulmonary complications, but no significant difference in survival between these approaches or techniques (37). Chemotherapy and radiotherapy are often used in adjuvant esophagectomy to obtain curative treatment or prolong the survival of patients with ESCC, among them, preoperative neoadjuvant therapy has been widely used due to its remarkable curative effect (37, 38). In recent years, the combination of chemotherapy and radiotherapy has received more attention in ESCC treatment, called chemoradiotherapy, which is more effective than chemotherapy and radiotherapy alone (32). Our study found some basic research hotspots related to tumor malignant phenotype of ESCC and clinical research hotspots related to patient prognosis and diagnosis are characterized by important but non-developed for the field, such as proliferation, apoptosis, prognosis, biomarker, and overall survival. The focus of tumor treatment is early detection and early resection, the degree of tumor development at the time of diagnosis is closely related to the prognosis of ESCC patients (37). Biomarkers are widely used in the diagnosis of different tumors due to their simplicity and efficiency, such as alpha-fetoprotein (AFP) in hepatocellular carcinoma (39) and CA19-9 in pancreatic cancer (40). However, biomarkers for ESCC diagnosis are insufficient, and some targets are still experimental, such as ADAR1, β-catenin, LTB4R, and TRAP1 (41, 42). Therefore, we need more research to identify effective biomarkers that can be used for the clinical diagnosis of ESCC. Some other clinical research hotspots related to chemotherapy and immunotherapy of ESCC are characterized by highly developed but isolated with marginal importance for the field, such as cisplatin, docetaxel, and EGFR. Immunotherapy is one of the important directions of tumor treatment. Monoclonal antibodies (mAbs) are the main component of tumor immunotherapy and have been widely used in clinical practice (43). However, no effective mAb is available to treat ESCC other than the HER2-targeting drug trastuzumab (5). Recently, chimeric antigen receptor T cell (CAR-T) therapy has entered clinical trials for multiple tumors, such as hematological malignancies (44), gastric cancer (45), and colorectal cancer (46), and has shown great potential in tumor treatment (47). A Prospective pilot study reported the benefit of anti-CD3-activated autologous αβT cell therapy in the treatment of ESCC (48). Furthermore, several clinical trials of CAR-T therapy for ESCC are ongoing, and the results may hopefully change the current treatment status of ESCC (49). Some clinical and basic research hotspots are characterized by emerging or declining hotspots in this field, such as meta-analysis, polymorphism, P53, and single nucleotide polymorphism. Furthermore, our time-dependent keyword co-occurrence analysis identifies some research hotspots that have emerged in recent years, such as complications, endoscopic submucosal dissection (ESD), neoadjuvant chemoradiotherapy and concurrent chemoradiotherapy, immunotherapy, tumor microenvironment, epithelial-mesenchymal transition (EMT), and long non-coding RNAs. In recent years, more patients with early-stage ESCC have been diagnosed due to the popularity of esophageal endoscopy in physical examinations. ESD is the primary treatment option for early-stage ESCC (50), and its surgical criteria, prognosis, and complications are the focus of future ESCC research. Tumor microenvironment, epithelial-mesenchymal transition, and long non-coding RNAs are hotspots in basic ESCC research, and their findings help to determine the possible efficacy of different clinical therapies, tumor metastatic ability, and potential therapeutic targets (51–53). All of these emerging hotspots, persistent hotspots, and declining hotspots are tightly centered around ESCC patient needs, and the global research trends of ESCC have not changed over the past decade.

Our study exists some inherent limitations. Firstly, we only collected articles from the WoS database, without other databases. However, the WoS database is the most commonly used in scientometrics and records high-quality publications. Secondly, we didn’t present the authors’ contributions and the co-authorships network map because most of the authors were from China, and the abbreviations of names in the downloaded data cannot accurately identify them. Thirdly, selection bias may exist in the literature screening process.

## Conclusion

We searched and analyzed 7,153 articles in the ESCC research area from 2012 to 2022. Despite some limitations, our study demonstrated that the publications related to ESCC research around the world are increasing rapidly. The academic institutions in China have an absolute advantage in this field. The global research trend of ESCC over the past decade is basic research assisting clinical studies with the purpose to find effective therapies. Therefore, the emerging hotspots related to ESCC treatment, such as endoscopic therapy, chemoradiotherapy, immunotherapy, tumor microenvironment, and epithelial-mesenchymal transition are the focus of future research and should be supported by more scholars and funds.

## Data availability statement

The raw data supporting the conclusions of this article will be made available by the authors, without undue reservation.

## Author contributions

ZZ, ZhuL: study concept and design, data acquisition and analysis, critical revision of the manuscript, writing of the manuscript. KF, MS, ZhaL, LK: critical revision of the manuscript. MX, TC: study concept and design, critical revision of the manuscript, writing of the manuscript, study supervision. All authors contributed to the article and approved the submitted version.

## Funding

This study was funded by medical discipline Construction Project of Pudong Health Committee of Shanghai (Grant No. PWYgf2021-02) and Shanghai Committee of Science and Technology (Grant No. 22YF1436400).

## Conflict of interest

The authors declare that the research was conducted in the absence of any commercial or financial relationships that could be construed as a potential conflict of interest

## Publisher note

All claims expressed in this article are solely those of the authors and do not necessarily represent those of their affiliated organizations, or those of the publisher, the editors and the reviewers. Any product that may be evaluated in this article, or claim that may be made by its manufacturer, is not guaranteed or endorsed by the publisher.
