# Supplementary material for: Global research trend of esophageal squamous cell carcinoma from 2012 to 2022: a bibliometric analysis
Source: Front Oncol. 2022 Aug 11;12:977935. doi: 10.3389/fonc.2022.977935 (PMC9403081; doi:10.3389/fonc.2022.977935)
Supplement: Supplementary file 1 [file DataSheet_1.docx]

**Supplementary Material**

**Table S1** Top 20 corresponding author's countries with the most publications in ESCC research.

| **Rank** | **Region** | **Articles** |  | **Rank** | **Region** | **Articles** |
| --- | --- | --- | --- | --- | --- | --- |
| 1 | CHINA | 5063 |  | 11 | ENGLAND | 31 |
| 2 | JAPAN | 1052 |  | 12 | FRANCE | 30 |
| 3 | USA | 269 |  | 13 | SWEDEN | 28 |
| 4 | SOUTH KOREA | 161 |  | 14 | SOUTH AFRICA | 20 |
| 5 | IRAN | 123 |  | 15 | ITALY | 19 |
| 6 | INDIA | 64 |  | 16 | TURKEY | 16 |
| 7 | GERMANY | 51 |  | 17 | IRELAND | 14 |
| 8 | BRAZIL | 35 |  | 18 | POLAND | 13 |
| 9 | AUSTRALIA | 34 |  | 19 | CANADA | 8 |
| 10 | NETHERLANDS | 33 |  | 20 | THAILAND | 6 |

**Table S2** Top 20 international collaborations in ESCC research.

| **Rank** | **From** | **To** | **Freq** |  | **Rank** | **From** | **To** | **Freq** |
| --- | --- | --- | --- | --- | --- | --- | --- | --- |
| 1 | CHINA | USA | 427 |  | 11 | IRAN | FRANCE | 27 |
| 2 | JAPAN | USA | 68 |  | 12 | CHINA | CANADA | 26 |
| 3 | USA | IRAN | 53 |  | 13 | USA | SOUTH KOREA | 25 |
| 4 | CHINA | JAPAN | 46 |  | 14 | CHINA | GERMANY | 24 |
| 5 | USA | FRANCE | 42 |  | 15 | FRANCE | ENGLAND | 24 |
| 6 | CHINA | AUSTRALIA | 37 |  | 16 | USA | NETHERLANDS | 24 |
| 7 | ENGLAND | SWEDEN | 34 |  | 17 | USA | GERMANY | 22 |
| 8 | USA | ENGLAND | 33 |  | 18 | CHINA | SWEDEN | 21 |
| 9 | CHINA | SOUTH KOREA | 32 |  | 19 | USA | INDIA | 21 |
| 10 | CHINA | ENGLAND | 29 |  | 20 | CHINA | NETHERLANDS | 19 |

**Table S3** Top 20 articles with the most global citations.

| **Rank** | **Paper** | **DOI** | **Total Citations** | **TC per Year** | **Normalized TC** |
| --- | --- | --- | --- | --- | --- |
| 1 | ANDO N, 2012, ANN SURG ONCOL | 10.1245/S10434-011-2049-9 | 760 | 69.09 | 25.50 |
| 2 | SONG YM, 2014, NATURE | 10.1038/NATURE13176 | 664 | 73.78 | 26.50 |
| 3 | ABNET CC, 2018, GASTROENTEROLOGY | 10.1053/J.GASTRO.2017.08.023 | 541 | 108.20 | 35.73 |
| 4 | LI F, 2015, ONCOTARGET | 10.18632/ONCOTARGET.3469 | 527 | 65.88 | 21.65 |
| 5 | BAGNARDI V, 2015, BRIT J CANCER | 10.1038/BJC.2014.579 | 524 | 65.50 | 21.52 |
| 6 | SMYTH EC, 2017, NAT REV DIS PRIMERS | 10.1038/NRDP.2017.48 | 435 | 72.50 | 26.28 |
| 7 | GAO YB, 2014, NAT GENET | 10.1038/NG.3076 | 412 | 45.78 | 16.44 |
| 8 | LIN DC, 2014, NAT GENET | 10.1038/NG.2935 | 373 | 41.44 | 14.89 |
| 9 | KATO K, 2019, LANCET ONCOL | 10.1016/S1470-2045(19)30626-6 | 319 | 79.75 | 27.68 |
| 10 | OHASHI S, 2015, GASTROENTEROLOGY | 10.1053/J.GASTRO.2015.08.054 | 319 | 39.88 | 13.10 |
| 11 | TANAKA Y, 2013, CANCER-AM CANCER SOC | 10.1002/CNCR.27895 | 309 | 30.90 | 11.74 |
| 12 | LI JG, 2014, GUT | 10.1136/GUTJNL-2013-305806 | 287 | 31.89 | 11.45 |
| 13 | TONG YS, 2015, MOL CANCER | 10.1186/1476-4598-14-3 | 261 | 32.63 | 10.72 |
| 14 | YANG H, 2018, J CLIN ONCOL | 10.1200/JCO.2018.79.1483 | 257 | 51.40 | 16.97 |
| 15 | WANG XY, 2015, J BIOL CHEM | 10.1074/JBC.M114.596866 | 246 | 30.75 | 10.11 |
| 16 | KUDO T, 2017, LANCET ONCOL | 10.1016/S1470-2045(17)30181-X | 224 | 37.33 | 13.53 |
| 17 | ZHANG L, 2015, AM J HUM GENET | 10.1016/J.AJHG.2015.02.017 | 220 | 27.50 | 9.04 |
| 18 | FLECKEN T, 2014, HEPATOLOGY | 10.1002/HEP.26731 | 214 | 23.78 | 8.54 |
| 19 | KONG KL, 2012, GUT | 10.1136/GUTJNL-2011-300178 | 208 | 18.91 | 6.98 |
| 20 | GE XS, 2013, CANCER SCI | 10.1111/CAS.12296 | 203 | 20.30 | 7.72 |

**Table S4** Top 20 articles with the most local citations.

| **Rank** | **Paper** | **DOI** | **Local Citations** | **LC/GC Ratio (%)** | **Normalized Local Citations** |
| --- | --- | --- | --- | --- | --- |
| 1 | SONG YM, 2014, NATURE | 10.1038/NATURE13176 | 322 | 48.49 | 52.68 |
| 2 | ABNET CC, 2018, GASTROENTEROLOGY | 10.1053/J.GASTRO.2017.08.023 | 311 | 57.49 | 89.92 |
| 3 | ANDO N, 2012, ANN SURG ONCOL | 10.1245/S10434-011-2049-9 | 308 | 40.53 | 42.4 |
| 4 | OHASHI S, 2015, GASTROENTEROLOGY | 10.1053/J.GASTRO.2015.08.054 | 200 | 62.7 | 38.96 |
| 5 | GAO YB, 2014, NAT GENET | 10.1038/NG.3076 | 191 | 46.36 | 31.25 |
| 6 | LIN DC, 2014, NAT GENET | 10.1038/NG.2935 | 186 | 49.87 | 30.43 |
| 7 | SMYTH EC, 2017, NAT REV DIS PRIMERS | 10.1038/NRDP.2017.48 | 163 | 37.47 | 41.93 |
| 8 | YANG H, 2018, J CLIN ONCOL | 10.1200/JCO.2018.79.1483 | 124 | 48.25 | 35.85 |
| 9 | ZHANG L, 2015, AM J HUM GENET | 10.1016/J.AJHG.2015.02.017 | 110 | 50 | 21.43 |
| 10 | SAWADA G, 2016, GASTROENTEROLOGY | 10.1053/J.GASTRO.2016.01.035 | 104 | 56.83 | 23.95 |
| 11 | LI JG, 2014, GUT | 10.1136/GUTJNL-2013-305806 | 96 | 33.45 | 15.71 |
| 12 | KATO K, 2019, LANCET ONCOL | 10.1016/S1470-2045(19)30626-6 | 92 | 28.84 | 39.8 |
| 13 | WU C, 2012, NAT GENET | 10.1038/NG.2411 | 78 | 42.62 | 10.74 |
| 14 | MALHOTRA GK, 2017, J SURG ONCOL | 10.1002/JSO.24592 | 69 | 43.4 | 17.75 |
| 15 | KUDO T, 2017, LANCET ONCOL | 10.1016/S1470-2045(17)30181-X | 65 | 29.02 | 16.72 |
| 16 | YAMASHINA T, 2013, AM J GASTROENTEROL | 10.1038/AJG.2013.8 | 63 | 40.91 | 9.71 |
| 17 | WEI WQ, 2015, J CLIN ONCOL | 10.1200/JCO.2014.58.0423 | 58 | 38.93 | 11.3 |
| 18 | LIN DC, 2018, GASTROENTEROLOGY | 10.1053/J.GASTRO.2017.06.066 | 58 | 52.25 | 16.77 |
| 19 | KONG KL, 2012, GUT | 10.1136/GUTJNL-2011-300178 | 54 | 25.96 | 7.43 |
| 20 | HARA H, 2013, CANCER SCI | 10.1111/CAS.12274 | 54 | 43.2 | 8.32 |

**Table S5** Top 30 keywords with the most occurrences or total link strength.

|  | **Occurrences** |  |  |  | **Total link strength** |  |
| --- | --- | --- | --- | --- | --- | --- |
| **Rank** | **Keyword** | **Times** |  | **Rank** | **Keyword** | **Strength** |
| 1 | oncology | 3700 |  | 1 | oncology | 25315 |
| 2 | esophageal squamous cell carcinoma | 3087 |  | 2 | esophageal squamous cell carcinoma | 21170 |
| 3 | cancer | 2845 |  | 3 | cancer | 19073 |
| 4 | expression | 1801 |  | 4 | expression | 13503 |
| 5 | survival | 1197 |  | 5 | survival | 9061 |
| 6 | prognosis | 1088 |  | 6 | prognosis | 8208 |
| 7 | esophageal cancer | 1049 |  | 7 | surgery | 7209 |
| 8 | surgery | 1030 |  | 8 | esophageal cancer | 7191 |
| 9 | metastasis | 870 |  | 9 | proliferation | 6915 |
| 10 | proliferation | 866 |  | 10 | metastasis | 6833 |
| 11 | gastroenterology & hepatology | 718 |  | 11 | invasion | 5670 |
| 12 | invasion | 686 |  | 12 | chemoradiotherapy | 5342 |
| 13 | chemoradiotherapy | 682 |  | 13 | chemotherapy | 4823 |
| 14 | cell biology | 650 |  | 14 | cell biology | 4671 |
| 15 | carcinoma | 620 |  | 15 | growth | 4357 |
| 16 | research & experimental medicine | 615 |  | 16 | carcinoma | 4319 |
| 17 | chemotherapy | 607 |  | 17 | apoptosis | 4317 |
| 18 | squamous-cell carcinoma | 568 |  | 18 | research & experimental medicine | 4280 |
| 19 | apoptosis | 567 |  | 19 | gastroenterology & hepatology | 4050 |
| 20 | growth | 545 |  | 20 | squamous-cell carcinoma | 3977 |
| 21 | adenocarcinoma | 496 |  | 21 | migration | 3863 |
| 22 | biochemistry & molecular biology | 478 |  | 22 | adenocarcinoma | 3673 |
| 23 | migration | 468 |  | 23 | progression | 3669 |
| 24 | progression | 456 |  | 24 | breast-cancer | 3417 |
| 25 | breast-cancer | 447 |  | 25 | biochemistry & molecular biology | 3387 |
| 26 | therapy | 435 |  | 26 | therapy | 3379 |
| 27 | risk | 418 |  | 27 | radiotherapy | 3189 |
| 28 | lung-cancer | 392 |  | 28 | lung-cancer | 2911 |
| 29 | radiotherapy | 390 |  | 29 | overexpression | 2863 |
| 30 | activation | 366 |  | 30 | activation | 2831 |
