# Supplementary material for: Global research trend of esophageal squamous cell carcinoma from 2012 to 2022: a bibliometric analysis
Source: Front Oncol. 2022 Aug 11;12:977935. doi: 10.3389/fonc.2022.977935 (PMC9403081; doi:10.3389/fonc.2022.977935)
Supplement: Supplementary file 2 [file DataSheet_2.docx]

FN Clarivate Analytics Web of Science

VR 1.0

PT J

AU Liu, B

Li, XH

Li, C

Xu, RX

Sun, XR

AF Liu, Bing

Li, Xuhua

Li, Chuan

Xu, Ruixia

Sun, Xuerong

TI miR-25 mediates metastasis and epithelial-mesenchymal-transition in

human esophageal squamous cell carcinoma via regulation of E-cadherin

signaling

SO BIOENGINEERED

LA English

DT Article

DE Esophageal squamous cell carcinoma; lymph node metastasis;

epithelial-mesenchymal transition; miR-25; E-cadherin

ID EXPRESSION; INVASION; REVERSES

AB MiR-25 is a well-documented oncogenic miRNA implicated in esophageal squamous cell carcinoma (ESCC) development, progression and metastasis. However, whether and how miR-25 is involved in the development and metastasis of ESCC remain un-addressed. By using qRT-PCR analysis to compare levels of miR-25 in ESCC tissues with or without lymph node metastasis (LNM), it showed that ESCC tissues with LNM had increased levels of miR-25, which was correlated with tumor metastasis and poor prognosis. Gain- and loss-of-function assays indicated that targeting miR-25 could reverse EMT, and reduce in vitro cell migration and invasion, but not apoptosis and proliferation of ESCC. Furthermore, targeting miR-25 inhibited in vivo lung metastasis, and vice versa. And E-cadherin was a direct target of miR-25 through which affected EMT process and metastasis of ESCC. It is therefore indicated that miR-25 promotes metastasis of ESCC through E-cadherin and EMT events, thus may serves as a negative prognostic factor and possible target for treatment of ESCC patients.

C1 [Liu, Bing] Cent Hosp Linyi, Dept Thorac Surg, Linyi, Shandong, Peoples R China.

[Li, Xuhua; Xu, Ruixia] Qingdao Univ, Dept Thyroid Surg, Affiliated Hosp, Shandong, Peoples R China.

[Li, Chuan; Sun, Xuerong] Qingdao Univ, Dept Thorac Surg, Affiliated Hosp, Shandong, Peoples R China.

RP Liu, B (corresponding author), Cent Hosp Linyi, Dept Thorac Surg, Linyi, Shandong, Peoples R China.

EM zxl82915870@126.com

FU National Natural Science Foundation of China [81872417]

FX This work was supported by the grants from the National Natural Science

Foundation of China [grant numbers: 81872417].

CR Abnet CC, 2018, GASTROENTEROLOGY, V154, P360, DOI 10.1053/j.gastro.2017.08.023

Calin GA, 2006, NAT REV CANCER, V6, P857, DOI 10.1038/nrc1997

Cavallaro U, 2014, NAT REV CANCER, V4, P118

Chen WQ, 2016, CA-CANCER J CLIN, V66, P115, DOI 10.3322/caac.21338

Dong PX, 2016, ONCOTARGET, V7, P20260, DOI 10.18632/oncotarget.7754

Enzinger PC, 2003, NEW ENGL J MED, V349, P2241, DOI 10.1056/NEJMra035010

Gloushankova NA, 2018, BIOCHEMISTRY-MOSCOW+, V83, P1469, DOI 10.1134/S0006297918120052

Guarino M, 2007, PATHOLOGY, V39, P305, DOI 10.1080/00313020701329914

He L, 2004, NAT REV GENET, V5, P522, DOI 10.1038/nrg1379

Jia YX, 2017, AM J TRANSL RES, V9, P4458

Karatzas G, 1999, HEPATO-GASTROENTEROL, V46, P232

Lu W, 2019, DEV CELL, V49, P361, DOI 10.1016/j.devcel.2019.04.010

Maretzky T, 2005, P NATL ACAD SCI USA, V102, P9182, DOI 10.1073/pnas.0500918102

Pecina-Slaus Nives, 2012, Front Biosci (Elite Ed), V4, P889, DOI 10.2741/E427

Riihimaki M, 2013, BMC CANCER, V13, DOI 10.1186/1471-2407-13-36

Song J, 2017, CANCER SCI, V108, P23, DOI 10.1111/cas.13104

Syrigos KN, 1995, INT J CANCER, V64, P367, DOI 10.1002/ijc.2910640603

Tang Q, 2014, EUR J PHARMACOL, V741, P97, DOI 10.1016/j.ejphar.2014.07.051

Wang M, 2018, EXP THER MED, V15, P440, DOI 10.3892/etm.2017.5358

Wu CY, 2014, PLOS ONE, V9, DOI 10.1371/journal.pone.0092292

Xu XH, 2012, BIOCHEM BIOPH RES CO, V421, P640, DOI 10.1016/j.bbrc.2012.03.048

NR 21

TC 16

Z9 16

U1 2

U2 3

PU TAYLOR & FRANCIS INC

PI PHILADELPHIA

PA 530 WALNUT STREET, STE 850, PHILADELPHIA, PA 19106 USA

SN 2165-5979

EI 2165-5987

J9 BIOENGINEERED

JI Bioengineered

PD JAN 1

PY 2019

VL 10

IS 1

BP 679

EP 688

DI 10.1080/21655979.2019.1687391

PG 10

WC Biotechnology & Applied Microbiology

WE Science Citation Index Expanded (SCI-EXPANDED)

SC Biotechnology & Applied Microbiology

GA JP7FU

UT WOS:000498427700001

PM 31679450

OA Green Published, gold

DA 2022-05-19

ER

PT J

AU Huang, HR

Xu, Y

Guo, Z

Chen, X

Ji, SG

Xu, ZY

AF Huang, Hairong

Xu, Yang

Guo, Zhong

Chen, Xi

Ji, Shaiguang

Xu, Zhiyun

TI MicroRNA-133b inhibits cell proliferation and promotes apoptosis by

targeting cullin 4B in esophageal squamous cell carcinoma

SO EXPERIMENTAL AND THERAPEUTIC MEDICINE

LA English

DT Article

DE microRNA-133b; esophageal squamous cell carcinoma; proliferation;

apoptosis; cullin 4B

ID IN-VITRO; EXPRESSION; CANCER; PATHOGENESIS; INTERACTS; PATHWAY

AB microRNAs (miRs) serve a role as modulators during carcinogenesis. It has been demonstrated that the expression of miR-133b is decreased in a variety of tumor tissues and cell lines and serves a suppressive role in the proliferation and apoptosis of different types of tumor cells. However, its effect on esophageal squamous cell carcinoma (ESCC) cells remains unclear. In the current study, the expression of mature miR-133b was measured using reverse transcription-quantitative polymerase chain reaction and the results indicated that miR-133b was significantly downregulated in ESCC tissues and various ESCC cell lines. The overexpression of miR-133b significantly inhibited the proliferation and promoted the apoptosis of KYSE150 and Eca-109 cells. Furthermore, it was demonstrated that cullin 4B (CUL4B) promotes ESCC cell proliferation and inhibits apoptosis by activating the protein kinase B/glycogen synthase kinase 3/-catenin pathway. Taken together, these results demonstrate that miR-133b/CUL4B serves a tumor suppressive role during ESCC progression and may therefore be used as a potential target to treat patients with ESCC.

C1 [Huang, Hairong; Xu, Yang; Guo, Zhong; Chen, Xi; Ji, Shaiguang] Nanjing Univ, Sch Med, Jinling Hosp, Dept Cardiothorac Surg, Nanjing 210002, Jiangsu, Peoples R China.

[Xu, Zhiyun] Second Mil Med Univ, Changhai Hosp, Dept Cardiothorac Surg, 168 Changhai Rd, Shanghai 200433, Peoples R China.

RP Xu, ZY (corresponding author), Second Mil Med Univ, Changhai Hosp, Dept Cardiothorac Surg, 168 Changhai Rd, Shanghai 200433, Peoples R China.

EM xzy_changhai@163.com

CR Ambros V, 2004, NATURE, V431, P350, DOI 10.1038/nature02871

Chen Z, 2014, ONCOL REP, V32, P2047, DOI 10.3892/or.2014.3465

Clough E, 2016, METHODS MOL BIOL, V1418, P93, DOI 10.1007/978-1-4939-3578-9_5

Dong J, 2015, EUR REV MED PHARMACO, V19, P1013

Dumbliauskas E, 2011, EMBO J, V30, P731, DOI 10.1038/emboj.2010.359

Edge SB, 2010, ANN SURG ONCOL, V17, P1471, DOI 10.1245/s10434-010-0985-4

Feber A, 2008, J THORAC CARDIOV SUR, V135, P255, DOI 10.1016/j.jtcvs.2007.08.055

Gu J, 2013, CURR PHARM DESIGN, V19, P1292

He B, 2012, MOL MED REP, V6, P459, DOI 10.3892/mmr.2012.975

Higa LA, 2006, CELL CYCLE, V5, P71, DOI 10.4161/cc.5.1.2266

Higa LA, 2006, NAT CELL BIOL, V8, P1277, DOI 10.1038/ncb1490

Hu G, 2010, CANCER BIOL THER, V10, P190, DOI 10.4161/cbt.10.2.12186

Jia ST, 2005, NAT CELL BIOL, V7, P1007, DOI 10.1038/ncb1300

Jiang T, 2013, MED ONCOL, V30, DOI 10.1007/s12032-013-0534-7

Kano M, 2010, INT J CANCER, V127, P2804, DOI 10.1002/ijc.25284

Kerzendorfer C, 2011, MECH AGEING DEV, V132, P366, DOI 10.1016/j.mad.2011.02.003

Kirby TJ, 2013, FREE RADICAL BIO MED, V64, P95, DOI 10.1016/j.freeradbiomed.2013.07.004

Li P, 2013, DIGEST DIS SCI, V58, P3483, DOI 10.1007/s10620-013-2854-z

Lin RJ, 2012, J SURG ONCOL, V105, P175, DOI 10.1002/jso.22066

Lin SB, 2015, NAT REV CANCER, V15, P321, DOI 10.1038/nrc3932

Livak KJ, 2001, METHODS, V25, P402, DOI 10.1006/meth.2001.1262

Napier KJ, 2014, WORLD J GASTRO ONCOL, V6, P112, DOI 10.4251/wjgo.v6.i5.112

Nishitani H, 2008, J BIOL CHEM, V283, P29045, DOI 10.1074/jbc.M806045200

Qian YY, 2015, CANCER RES, V75, P5070, DOI 10.1158/0008-5472.CAN-15-0898

Siegel RL, 2015, CA-CANCER J CLIN, V65, P5, DOI [10.3322/caac.21254, 10.1136/bmj.g1502, 10.1001/jamaoto.2014.2530, 10.3322/caac.21208]

Song YM, 2014, NATURE, V509, P91, DOI 10.1038/nature13176

Sugimura K, 2012, CLIN CANCER RES, V18, P5144, DOI 10.1158/1078-0432.CCR-12-0701

Toh Y, 2013, GEN THORAC CARDIOVAS, V61, P262, DOI 10.1007/s11748-013-0235-3

Williams AH, 2009, CURR OPIN CELL BIOL, V21, P461, DOI 10.1016/j.ceb.2009.01.029

Zhao HF, 2013, PLOS ONE, V8, DOI 10.1371/journal.pone.0083571

Zhao Y, 2014, BMC CANCER, V14, DOI 10.1186/1471-2407-14-34

Zhou K, 2014, MED ONCOL, V31, DOI 10.1007/s12032-014-0017-5

NR 32

TC 14

Z9 15

U1 0

U2 1

PU SPANDIDOS PUBL LTD

PI ATHENS

PA POB 18179, ATHENS, 116 10, GREECE

SN 1792-0981

EI 1792-1015

J9 EXP THER MED

JI Exp. Ther. Med.

PD APR

PY 2018

VL 15

IS 4

BP 3743

EP 3750

DI 10.3892/etm.2018.5906

PG 8

WC Medicine, Research & Experimental

WE Science Citation Index Expanded (SCI-EXPANDED)

SC Research & Experimental Medicine

GA GB3GV

UT WOS:000428945200082

PM 29581734

OA Green Submitted, gold, Green Published

DA 2022-05-19

ER

PT J

AU Liu, SY

Chen, W

Chughtai, EA

Qiao, Z

Jiang, JT

Li, SM

Zhang, W

Zhang, J

AF Liu, Shi-Yuan

Chen, Wei

Chughtai, Ehtesham Annait

Qiao, Zhe

Jiang, Jian-Tao

Li, Shao-Min

Zhang, Wei

Zhang, Jin

TI PIK3CA gene mutations in Northwest Chinese esophageal squamous cell

carcinoma

SO WORLD JOURNAL OF GASTROENTEROLOGY

LA English

DT Article

DE PIK3CA gene mutations; Esophageal squamous cell carcinoma; Northwest

Chinese; Prognostic significance

ID COLORECTAL CANCERS; BREAST-CANCER; ESOPHAGOGASTRIC JUNCTION; PI3K/AKT

PATHWAY; LUNG CANCERS; STATISTICS; BIOMARKERS; PROGNOSIS; OVARIAN;

GROWTH

AB AIM

To evaluate PIK3CA gene mutational status in Northwest Chinese esophageal squamous cell carcinoma (ESCC) patients, and examine the associations of PIK3CA gene mutations with clinicopathological characteristics and clinical outcome.

METHODS

A total of 210 patients with ESCC who underwent curative resection were enrolled in this study. Pyrosequencing was applied to investigate mutations in exons 9 and 20 of PIK3CA gene in 210 Northwest Chinese ESCCs. The associations of PIK3CA gene mutations with clinicopathological characteristics and clinical outcome were examined.

RESULTS

PIK3CA gene mutations in exon 9 were detected in 48 cases (22.9%) of a non-biased database of 210 curatively resected Northwest Chinese ESCCs. PIK3CA gene mutations were not associated with sex, tobacco use, alcohol use, tumor location, stage, or local recurrence. When compared with wild-type PIK3CA gene cases, patients with PIK3CA gene mutations in exons 9 experienced significantly better disease-free survival and overall survival rates.

CONCLUSION

The results of this study suggest that PIK3CA gene mutations could act as a prognostic biomarker in Northwest Chinese ESCC patients.

C1 [Liu, Shi-Yuan; Qiao, Zhe; Jiang, Jian-Tao; Li, Shao-Min; Zhang, Wei; Zhang, Jin] Xi An Jiao Tong Univ, Affiliated Hosp 2, Dept Thorac Surg, 157 Xi Wu Lu, Xian 710004, Shaanxi Provinc, Peoples R China.

[Chen, Wei] Lanzhou Univ, Sch Basic Med Sci, Dept Med Biochem & Mol Biol, Lanzhou 730000, Gansu Province, Peoples R China.

[Chughtai, Ehtesham Annait] Xi An Jiao Tong Univ, Sch Med, Xian 710061, Shaanxi Provinc, Peoples R China.

RP Zhang, J (corresponding author), Xi An Jiao Tong Univ, Affiliated Hosp 2, Dept Thorac Surg, 157 Xi Wu Lu, Xian 710004, Shaanxi Provinc, Peoples R China.

EM mansteinxjtu@163.com

FU National Natural Science Foundation of China [81602023]

FX Supported by National Natural Science Foundation of China, No. 81602023.

CR Abubaker J, 2008, ONCOGENE, V27, P3539, DOI 10.1038/sj.onc.1211013

Barbareschi M, 2007, CLIN CANCER RES, V13, P6064, DOI 10.1158/1078-0432.CCR-07-0266

Broderick DK, 2004, CANCER RES, V64, P5048, DOI 10.1158/0008-5472.CAN-04-1170

Campbell IG, 2004, CANCER RES, V64, P7678, DOI 10.1158/0008-5472.CAN-04-2933

Chen WQ, 2016, CA-CANCER J CLIN, V66, P115, DOI 10.3322/caac.21338

Engelman JA, 2006, NAT REV GENET, V7, P606, DOI 10.1038/nrg1879

Enzinger PC, 2003, NEW ENGL J MED, V349, P2241, DOI 10.1056/NEJMra035010

Fang WL, 2016, ONCOTARGET, V7, P6201, DOI 10.18632/oncotarget.6641

Gertler R, 2011, ANN SURG, V253, P689, DOI 10.1097/SLA.0b013e31821111b5

Jelovac D, 2014, HUM PATHOL, V45, P880, DOI 10.1016/j.humpath.2013.10.016

Kalinsky K, 2009, CLIN CANCER RES, V15, P5049, DOI 10.1158/1078-0432.CCR-09-0632

Lai YL, 2008, ANN SURG ONCOL, V15, P1064, DOI 10.1245/s10434-007-9751-7

Lee SE, 2016, J KOREAN MED SCI, V31, P1224, DOI 10.3346/jkms.2016.31.8.1224

Li VSW, 2005, BMC CANCER, V5, DOI 10.1186/1471-2407-5-29

Maeng CH, 2012, PLOS ONE, V7, DOI 10.1371/journal.pone.0041655

Manning BD, 2007, CELL, V129, P1261, DOI 10.1016/j.cell.2007.06.009

Miyaki M, 2007, INT J CANCER, V121, P1627, DOI 10.1002/ijc.22829

Mori R, 2008, J SURG RES, V145, P320, DOI 10.1016/j.jss.2007.03.044

Muc-Wierzgon M, 2014, WORLD J GASTROENTERO, V20, P9759, DOI 10.3748/wjg.v20.i29.9759

Murugan AK, 2013, CANCER LETT, V338, P193, DOI 10.1016/j.canlet.2013.04.005

Nam SK, 2016, PLOS ONE, V11, DOI 10.1371/journal.pone.0151865

Rahman M, 2012, HUM PATHOL, V43, P2197, DOI 10.1016/j.humpath.2012.03.011

Rice TW, 2010, ANN SURG ONCOL, V17, P1721, DOI 10.1245/s10434-010-1024-1

Saal LH, 2005, CANCER RES, V65, P2554, DOI 10.1158/0008-5472-CAN-04-3913

Samuels Y, 2005, CANCER CELL, V7, P561, DOI 10.1016/j.ccr.2005.05.014

Samuels Y, 2004, CELL CYCLE, V3, P1221, DOI 10.4161/cc.3.10.1164

Samuels Y, 2004, SCIENCE, V304, P554, DOI 10.1126/science.1096502

Sasaki Y, 2016, WORLD J GASTROENTERO, V22, P2284, DOI 10.3748/wjg.v22.i7.2284

Shigaki H, 2013, CLIN CANCER RES, V19, P2451, DOI 10.1158/1078-0432.CCR-12-3559

Sutcliffe P, 2009, HEALTH TECHNOL ASSES, V13, P1, DOI 10.3310/hta13050

Trape J, 2012, INT J BIOL MARKER, V27, P257, DOI 10.5301/JBM.2012.9314

Wang L, 2014, PLOS ONE, V9, DOI 10.1371/journal.pone.0103021

Wang WF, 2013, ACTA PHARMACOL SIN, V34, P1560, DOI 10.1038/aps.2013.163

Whitehall VLJ, 2012, INT J CANCER, V131, P813, DOI 10.1002/ijc.26440

Xie GH, 2015, J EXP CLIN CANC RES, V34, DOI 10.1186/s13046-015-0179-9

Yamamoto H, 2008, CANCER RES, V68, P6913, DOI 10.1158/0008-5472.CAN-07-5084

Zeng HM, 2016, THORAC CANCER, V7, P232, DOI 10.1111/1759-7714.12322

Zhou ZY, 2006, J CLIN MICROBIOL, V44, P3900, DOI 10.1128/JCM.01209-06

NR 38

TC 7

Z9 7

U1 1

U2 5

PU BAISHIDENG PUBLISHING GROUP INC

PI PLEASANTON

PA 8226 REGENCY DR, PLEASANTON, CA 94588 USA

SN 1007-9327

EI 2219-2840

J9 WORLD J GASTROENTERO

JI World J. Gastroenterol.

PD APR 14

PY 2017

VL 23

IS 14

BP 2585

EP 2591

DI 10.3748/wjg.v23.i14.2585

PG 7

WC Gastroenterology & Hepatology

WE Science Citation Index Expanded (SCI-EXPANDED)

SC Gastroenterology & Hepatology

GA ES4BW

UT WOS:000399475100015

PM 28465643

OA hybrid, Green Published, Green Submitted

DA 2022-05-19

ER

PT J

AU Jin, ZX

Feng, W

Ji, Y

Jin, LY

AF Jin, Zixuan

Feng, Wei

Ji, Ying

Jin, Longyu

TI Resveratrol mediates cell cycle arrest and cell death in human

esophageal squamous cell carcinoma by directly targeting the EGFR

signaling pathway

SO ONCOLOGY LETTERS

LA English

DT Article

DE esophageal squamous cell carcinoma; resveratrol; epidermal growth factor

receptor

ID GROWTH-FACTOR RECEPTOR; GENOME-WIDE ASSOCIATION; NUCLEAR EGFR; GENE

AMPLIFICATION; PROGNOSTIC VALUE; BREAST-CANCER; DNA-PK; MECHANISMS;

EXPRESSION; NETWORK

AB Resveratrol is a small polyphenol that has been intensively studied in a wide spectrum of therapeutic fields. More recently, resveratrol has been demonstrated to exert its antitumor activity in numerous tumor models. The present study reported that resveratrol exhibited a marked anti-proliferative effect on human esophageal squamous cell carcinoma (ESCC) cells by inducing cell cycle Go/G, phase arrest and cell death, which was associated with a decrease in the expression levels of cyclin Dl and an increase in cleaved PARP/cleaved caspase-3 expression levels. The mechanisms underlying the antitumor potency of resveratrol were principally attributed to the downregulation of epidermal growth factor receptor (EGFR) signaling. The western blotting results showed that exposure of ESCC cells to resveratrol inhibited EGF-induced EGFR activation in addition to decreasing the total protein levels of EGFR and membrane/nuclear localization. In summary, the results suggested that resveratrol, or an associated analog, may have a role in the management of human ESCC.

C1 [Jin, Zixuan] Hunan Normal Univ, High Sch, Dept Biochem, Changsha 410006, Hunan, Peoples R China.

[Feng, Wei; Ji, Ying; Jin, Longyu] Cent S Univ, Xiangya Hosp 3, Dept Cardiothorac Surg, 138 Tongzipo Rd, Changsha 410013, Hunan, Peoples R China.

RP Jin, LY (corresponding author), Cent S Univ, Xiangya Hosp 3, Dept Cardiothorac Surg, 138 Tongzipo Rd, Changsha 410013, Hunan, Peoples R China.

EM jinlongyu1123@163.com

FU National Nature Science Foundation of China [81472774]

FX This study was supported by the National Nature Science Foundation of

China (grant no. 81472774).

CR Aichler M, 2014, ONCOTARGET, V5, P6620, DOI 10.18632/oncotarget.2268

Brand TM, 2013, RADIOTHER ONCOL, V108, P370, DOI 10.1016/j.radonc.2013.06.010

Britton RG, 2015, ANN NY ACAD SCI, V1348, P124, DOI 10.1111/nyas.12796

Cheung WY, 2009, GASTROENTEROL CLIN N, V38, P75, DOI 10.1016/j.gtc.2009.01.009

Dittmann K, 2005, RADIOTHER ONCOL, V76, P157, DOI 10.1016/j.radonc.2005.06.022

Dittmann K, 2007, RADIOTHER ONCOL, V84, P328, DOI 10.1016/j.radonc.2007.07.006

Dittmann K, 2008, MOL CANCER, V7, DOI 10.1186/1476-4598-7-69

Enzinger PC, 2003, NEW ENGL J MED, V349, P2241, DOI 10.1056/NEJMra035010

Ettenberg SA, 1999, ONCOGENE, V18, P1855, DOI 10.1038/sj.onc.1202499

Fichter CD, 2014, J MOL MED, V92, P1209, DOI 10.1007/s00109-014-1187-5

Frazzi R, 2014, INT J MOL SCI, V15, P4977, DOI 10.3390/ijms15034977

Gan HK, 2012, CANCER RES, V72, P2924, DOI 10.1158/0008-5472.CAN-11-3898

Gao YB, 2014, NAT GENET, V46, P1097, DOI 10.1038/ng.3076

Gescher A, 2013, ANN NY ACAD SCI, V1290, P12, DOI 10.1111/nyas.12205

Han W, 2012, CANCER LETT, V318, P124, DOI 10.1016/j.canlet.2012.01.011

Hanawa M, 2006, INT J CANCER, V118, P1173, DOI 10.1002/ijc.21454

Hongo M, 2009, J GASTROEN HEPATOL, V24, P729, DOI 10.1111/j.1440-1746.2009.05824.x

Hoshino M, 2007, PATHOBIOLOGY, V74, P15, DOI 10.1159/000101047

Jaganathan S, 2011, PLOS ONE, V6, DOI 10.1371/journal.pone.0019605

Kolch W, 2010, NAT REV CANCER, V10, P618, DOI 10.1038/nrc2900

Kulkarni SS, 2015, BBA-MOL BASIS DIS, V1852, P1114, DOI 10.1016/j.bbadis.2014.10.005

Lacouture ME, 2006, NAT REV CANCER, V6, P803, DOI 10.1038/nrc1970

Levkowitz G, 1999, MOL CELL, V4, P1029, DOI 10.1016/S1097-2765(00)80231-2

Li A, 2013, ONCOGENE, V32, P2273, DOI 10.1038/onc.2012.253

Liccardi G, 2014, CLIN CANCER RES, V20, P3496, DOI 10.1158/1078-0432.CCR-13-2695

Lin SY, 2001, NAT CELL BIOL, V3, P802, DOI 10.1038/ncb0901-802

Lin YL, 2014, BRIT J NUTR, V112, P2002, DOI 10.1017/S0007114514003055

Lo HW, 2010, DISCOV MED, V10, P44

Lo HW, 2005, CANCER RES, V65, P338

Mitsudomi T, 2010, FEBS J, V277, P301, DOI 10.1111/j.1742-4658.2009.07448.x

Novelle MG, 2015, AGEING RES REV, V21, P1, DOI 10.1016/j.arr.2015.01.002

Olayioye MA, 2000, EMBO J, V19, P3159, DOI 10.1093/emboj/19.13.3159

Park EJ, 2015, BBA-MOL BASIS DIS, V1852, P1071, DOI 10.1016/j.bbadis.2015.01.014

Russo GL, 2014, CANCER TREAT RES, V159, P185, DOI 10.1007/978-3-642-38007-5_11

Schramm Laura, 2013, J Carcinog Mutagen, V4, P1000142

Singh CK, 2015, BBA-MOL BASIS DIS, V1852, P1178, DOI 10.1016/j.bbadis.2014.11.004

Singh CK, 2013, ANN NY ACAD SCI, V1290, P113, DOI 10.1111/nyas.12160

Taguchi Takahide, 2014, Kokubyo Gakkai Zasshi, V81, P45

Tebbutt N, 2013, NAT REV CANCER, V13, P663, DOI 10.1038/nrc3559

Umemura S, 2005, ONCOL REP, V14, P337

van Hagen P, 2012, NEW ENGL J MED, V366, P2074, DOI 10.1056/NEJMoa1112088

Wang J, 2014, ASIAN PAC J CANCER P, V15, P5889, DOI 10.7314/APJCP.2014.15.14.5889

Wu C, 2013, NAT GENET, V45, P632, DOI 10.1038/ng.2638

Wu C, 2011, NAT GENET, V43, P679, DOI 10.1038/ng.849

Xue YQ, 2014, OXID MED CELL LONGEV, V2014, DOI 10.1155/2014/765832

Yang X, 2014, ANTI-CANCER AGENT ME, V14, P806, DOI 10.2174/1871520614666140521121722

NR 46

TC 3

Z9 4

U1 1

U2 8

PU SPANDIDOS PUBL LTD

PI ATHENS

PA POB 18179, ATHENS, 116 10, GREECE

SN 1792-1074

EI 1792-1082

J9 ONCOL LETT

JI Oncol. Lett.

PD JAN

PY 2017

VL 13

IS 1

BP 347

EP 355

DI 10.3892/ol.2016.5391

PG 9

WC Oncology

WE Science Citation Index Expanded (SCI-EXPANDED)

SC Oncology

GA EJ2CV

UT WOS:000393018400053

PM 28123566

OA Green Published, gold

DA 2022-05-19

ER

PT J

AU Wang, YY

Zhou, S

Zhao, R

Hai, P

Zhe, H

AF Wang, Yan-Yang

Zhou, Shun

Zhao, Ren

Hai, Ping

Zhe, Hong

TI The therapeutic response of CDDO-Me in the esophageal squamous cell

carcinoma (ESCC) cells is mediated by CaMKII alpha

SO AMERICAN JOURNAL OF TRANSLATIONAL RESEARCH

LA English

DT Article

DE CDDO-Me; CaMKII alpha; esophageal squamous cell carcinoma; SILAC

ID QUANTITATIVE PROTEOMICS; BARDOXOLONE METHYL; CULTURE SILAC; AMINO-ACIDS;

KINASE-II; CANCER; PREVENTION; AUTOPHAGY

AB CDDO-Me has exhibited a potent anticancer effect in human esophageal squamous cell carcinoma (ESCC) cells in our previous study, but the molecular interactome remains elusive. We applied the approach of stable-isotope labeling by amino acids in cell culture (SILAC) to assess the proteomic responses of CDDO-Me treatment in human ESCC Ec109 cells. The data were subsequently validated using Western blot assay. The results of our study revealed that CDDO-Me increased the expression level of 543 protein molecules, but decreased the expression level of 709 protein molecules in Ec109 cells. Among these modulated protein molecules, calcium/calmodul-independent protein kinase type II subunit alpha (CaMKII alpha) was highly expressed in all tested ESCC cell lines, whereas its expression levels were substantially lower in normal control cell line. Its silencing by small interfering RNA inhibited CDDO-Me induced apoptosis and autophagy in ESCC cells. Collectively, these data demonstrate that the therapeutic response of CDDO-Me in the human ESCC cells is mediated by CaMKII alpha.

C1 [Wang, Yan-Yang; Zhao, Ren; Hai, Ping; Zhe, Hong] Ningxia Med Univ, Gen Hosp, Dept Radiat Oncol, Ningxia 750004, Peoples R China.

[Wang, Yan-Yang; Zhao, Ren; Hai, Ping; Zhe, Hong] Ningxia Med Univ, Inst Canc, Ningxia 750004, Peoples R China.

[Zhou, Shun] Ningxia Med Univ, Grad Sch, Ningxia 750004, Peoples R China.

RP Wang, YY (corresponding author), Ningxia Med Univ, Gen Hosp, Dept Radiat Oncol, Ningxia 750004, Peoples R China.; Wang, YY (corresponding author), Ningxia Med Univ, Inst Canc, Ningxia 750004, Peoples R China.

EM fdwyy1981@hotmail.com

FU National Natural Science Foundation of China [81502-094]; Scientific

Program for Overseas Chinese Scholar of MOHRSS of China

FX This work was supported by the National Natural Science Foundation of

China (No. 81502-094) and Scientific Program for Overseas Chinese

Scholar of MOHRSS of China (2015). They also appreciate the guidance and

technical assistance for this study from Shu-Feng Zhou, PhD, Zhi-Wei

Zhou, PhD, and Kent Seeley, PhD, University of South Florida, Tampa, FL

33612, USA.

CR Belkhiri A, 2015, ONCOTARGET, V6, P1348, DOI 10.18632/oncotarget.2752

Chen XL, 2015, PROTEOMICS, V15, P3175, DOI 10.1002/pmic.201500108

D'Journo XB, 2014, J THORAC DIS, V6, pS253, DOI 10.3978/j.issn.2072-1439.2014.04.16

Daly JM, 2000, J AM COLL SURGEONS, V190, P573, DOI 10.1016/S1072-7515(00)00275-1

Daly JM, 2000, J AM COLL SURGEONS, V190, P562, DOI 10.1016/S1072-7515(00)00238-6

Enzinger PC, 2003, NEW ENGL J MED, V349, P2241, DOI 10.1056/NEJMra035010

Fitzmaurice C, 2015, JAMA ONCOL, V1, P505, DOI 10.1001/jamaoncol.2015.0735

Gaertner TR, 2004, J BIOL CHEM, V279, P12484, DOI 10.1074/jbc.M313597200

Hoedt E, 2014, ADV EXP MED BIOL, V806, P93, DOI 10.1007/978-3-319-06068-2_5

Hoelz A, 2003, MOL CELL, V11, P1241, DOI 10.1016/S1097-2765(03)00171-0

Hong DS, 2012, CLIN CANCER RES, V18, P3396, DOI 10.1158/1078-0432.CCR-11-2703

Lagergren J, 2013, CA-CANCER J CLIN, V63, P232, DOI 10.3322/caac.21185

Liang XH, 1999, NATURE, V402, P672, DOI 10.1038/45257

Liby KT, 2012, PHARMACOL REV, V64, P972, DOI 10.1124/pr.111.004846

Llambi F, 2011, CURR OPIN GENET DEV, V21, P12, DOI 10.1016/j.gde.2010.12.001

Mariette C, 2015, LANCET ONCOL, V16, P1008, DOI 10.1016/S1470-2045(15)00127-8

Nakatogawa H, 2007, CELL, V130, P165, DOI 10.1016/j.cell.2007.05.021

Ong Shao-En, 2007, Methods Mol Biol, V359, P37

Pan ST, 2015, DRUG DES DEV THER, V9, P937, DOI 10.2147/DDDT.S76021

Siegel RL, 2016, CA-CANCER J CLIN, V66, P7, DOI 10.3322/caac.21332

Wang YY, 2015, ONCOTARGET, V6, P11725, DOI 10.18632/oncotarget.3955

Wang YY, 2015, DRUG DES DEV THER, V9, P993, DOI 10.2147/DDDT.S73493

Wang YY, 2014, MOL CANCER, V13, DOI [10.1186/1476-4598-13-30, 10.1186/s12934-014-0151-y]

NR 23

TC 3

Z9 3

U1 0

U2 1

PU E-CENTURY PUBLISHING CORP

PI MADISON

PA 40 WHITE OAKS LN, MADISON, WI 53711 USA

SN 1943-8141

J9 AM J TRANSL RES

JI Am. J. Transl. Res.

PY 2016

VL 8

IS 4

BP 1695

EP 1707

PG 13

WC Oncology; Medicine, Research & Experimental

WE Science Citation Index Expanded (SCI-EXPANDED)

SC Oncology; Research & Experimental Medicine

GA DK9QI

UT WOS:000375265800005

PM 27186293

DA 2022-05-19

ER

PT J

AU Wang, X

Li, XJ

Zhang, XQ

Fan, RT

Gu, H

Shi, YG

Liu, HT

AF Wang, Xin

Li, XiaoJuan

Zhang, XiaQing

Fan, RuiTai

Gu, Hao

Shi, YongGang

Liu, HongTao

TI Glucose-6-phosphate dehydrogenase expression is correlated with poor

clinical prognosis in esophageal squamous cell carcinoma

SO EJSO

LA English

DT Article

DE Glucose-6-phosphate dehydrogenase; Pentose phosphate pathway; Esophageal

squamous cell carcinoma; Prognostic factor

ID PENTOSE-PHOSPHATE PATHWAY; CANCER; TUMORS; METABOLISM; OVEREXPRESSION;

PROGRESS; THERAPY; TARGET

AB Increasing evidence has demonstrated that glucose-6-phosphate dehydrogenase (G6PD), a key metabolic enzyme, participating in pentose phosphate pathway (PPP), is tightly associated with development and progression of a variety of tumors. Here, we reported expression of G6PD and its association with the prognosis of the patients with esophageal squamous cell carcinoma (ESCC). The results revealed significantly elevated G6PD mRNA and protein expressions in ESCC tissues compared with normal tissues (P < 0.05). Furthermore, high G6PD expression was tightly associated with histological grade, TNM staging and lymph node metastasis (P < 0.05), but not related to the patients' age and gender (P > 0.05). Importantly, the survival time of G6PD-positive patients was markedly lower than that of G6PD-negative patients (P < 0.05). Most notably, Cox multivariate assay demonstrated that G6PD was an independent prognostic factor for the patients with ESCC. In conclusion, G6PD may be a novel predictor for the prognosis of the patients with ESCC. (C) 2015 Elsevier Ltd. All rights reserved.

C1 [Wang, Xin; Fan, RuiTai; Gu, Hao; Shi, YongGang] Zhengzhou Univ, Affiliated Hosp 1, Dept Radiotherapy, Zhengzhou 450052, Henan, Peoples R China.

[Li, XiaoJuan] Henan Univ Tradit Chinese Med, Sch Basic Med Sci, Zhengzhou 450000, Henan, Peoples R China.

[Zhang, XiaQing; Liu, HongTao] Zhengzhou Univ, Sch Life Sci, Cell Biol Lab, Zhengzhou 450001, Henan, Peoples R China.

RP Liu, HT (corresponding author), Zhengzhou Univ, Sch Life Sci, Cell Biol Lab, 100 Kexue Ave, Zhengzhou 450001, Henan, Peoples R China.

EM liuht1230@126.com

OI Shi, Yonggang/0000-0003-2701-2522

FU National Natural Science Foundation of China [81272691]

FX This work was supported by the National Natural Science Foundation of

China (No. 81272691).

CR BABA M, 1989, INT J CANCER, V43, P892, DOI 10.1002/ijc.2910430526

Boros LG, 1997, CANCER RES, V57, P4242

Cheng AJ, 2001, JPN J CANCER RES, V92, P576, DOI 10.1111/j.1349-7006.2001.tb01132.x

Cheong H, 2012, NAT BIOTECHNOL, V30, P671, DOI 10.1038/nbt.2285

Crane LMA, 2007, EUR J CANCER, V43, P1445, DOI 10.1016/j.ejca.2007.03.024

Devi G. Sandhya, 1995, Indian Journal of Experimental Biology, V33, P829

Dong LP, 2015, TUMOR BIOL, V36, P2169, DOI 10.1007/s13277-014-2827-7

Elf SE, 2014, CANCER-AM CANCER SOC, V120, P774, DOI 10.1002/cncr.28501

Enzinger PC, 2003, NEW ENGL J MED, V349, P2241, DOI 10.1056/NEJMra035010

Ferlay J, 2010, INT J CANCER, V127, P2893, DOI 10.1002/ijc.25516

Ganapathy-Kanniappan S, 2013, MOL CANCER, V12, DOI 10.1186/1476-4598-12-152

Hofstetter W, 2002, ANN SURG, V236, P376, DOI 10.1097/00000658-200209000-00014

Hong XH, 2014, GUT, V63, P1635, DOI 10.1136/gutjnl-2013-305302

Hu HD, 2014, AM J PHYSIOL-GASTR L, V307, pG611, DOI 10.1152/ajpgi.00160.2014

Hu T, 2015, EXP BIOL MED MAYWOOD

JAVLE M, 2006, J SUPPORT ONCOL, V4, P379

Javle Milind, 2006, J Support Oncol, V4, P365

Jemal A, 2011, CA-CANCER J CLIN, V61, P69, DOI [10.3322/caac.20107, 10.3322/caac.20115]

Jiang P, 2014, PROTEIN CELL, V5, P592, DOI 10.1007/s13238-014-0082-8

Jones NP, 2012, DRUG DISCOV TODAY, V17, P232, DOI 10.1016/j.drudis.2011.12.017

KOUDSTAAL J, 1975, EUR J CANCER, V11, P111, DOI 10.1016/0014-2964(75)90188-7

Kuo WY, 1998, FREE RADICAL BIO MED, V24, P1130, DOI 10.1016/S0891-5849(97)00413-9

Kuo WY, 2000, INT J CANCER, V85, P857, DOI 10.1002/(SICI)1097-0215(20000315)85:6<857::AID-IJC20>3.0.CO;2-U

Lam KY, 1996, J CLIN PATHOL, V49, P124, DOI 10.1136/jcp.49.2.124

Liu HT, 2010, J SURG ONCOL, V102, P683, DOI 10.1002/jso.21627

Liu Y, 2012, J HISTOCHEM CYTOCHEM, V60, P512, DOI 10.1369/0022155412444437

Livak KJ, 2001, METHODS, V25, P402, DOI 10.1006/meth.2001.1262

Mathupala SP, 2011, RECENT PAT ANTI-CANC, V6, P6

Mocanu A, 2015, J Med Life, V8, P150

Moestue SA, 2013, BREAST CANCER RES, V15, DOI 10.1186/bcr3391

Patra KC, 2014, TRENDS BIOCHEM SCI, V39, P347, DOI 10.1016/j.tibs.2014.06.005

Pennathur A, 2013, LANCET, V381, P400, DOI 10.1016/S0140-6736(12)60643-6

Razungles J, 2013, M S-MED SCI, V29, P1026, DOI 10.1051/medsci/20132911020

Saarikoski ST, 2005, J HISTOCHEM CYTOCHEM, V53, P549, DOI 10.1369/jhc.4C6576.2005

Seyfried TN, 2015, CANCER LETT, V356, P289, DOI 10.1016/j.canlet.2014.07.015

Siegel R, 2012, CA-CANCER J CLIN, V62, P10, DOI 10.3322/caac.20138

Urschel JD, 2002, AM J SURG, V183, P274, DOI 10.1016/S0002-9610(02)00795-X

Vizcaino AP, 2002, INT J CANCER, V99, P860, DOI 10.1002/ijc.10427

Wang JX, 2012, TUMOR BIOL, V33, P95, DOI 10.1007/s13277-011-0251-9

Wang L, 2004, STROKE, V35, P1732, DOI 10.1161/01.STR.0000132196.49028.a4

Wikman H, 2004, J PATHOL, V203, P584, DOI 10.1002/path.1552

Zeng HM, 2006, J HISTOCHEM CYTOCHEM, V54, P889, DOI 10.1369/jhc.5A6881.2006

Zhang CH, 2014, ANTI-CANCER AGENT ME, V14, P280, DOI 10.2174/18715206113136660337

NR 43

TC 19

Z9 20

U1 0

U2 8

PU ELSEVIER SCI LTD

PI OXFORD

PA THE BOULEVARD, LANGFORD LANE, KIDLINGTON, OXFORD OX5 1GB, OXON, ENGLAND

SN 0748-7983

EI 1532-2157

J9 EJSO-EUR J SURG ONC

JI EJSO

PD OCT

PY 2015

VL 41

IS 10

BP 1293

EP 1299

DI 10.1016/j.ejso.2015.08.155

PG 7

WC Oncology; Surgery

WE Science Citation Index Expanded (SCI-EXPANDED)

SC Oncology; Surgery

GA CU2HW

UT WOS:000363345600004

PM 26329784

DA 2022-05-19

ER

PT J

AU Du, Q

Yan, WS

Burton, VH

Hewitt, SM

Wang, LM

Hu, N

Taylor, PR

Armani, MD

Mukherjee, S

Emmert-Buck, MR

Tangrea, MA

AF Du, Qiang

Yan, Wusheng

Burton, Victoria H.

Hewitt, Stephen M.

Wang, Lemin

Hu, Nan

Taylor, Philip R.

Armani, Michael D.

Mukherjee, Sumana

Emmert-Buck, Michael R.

Tangrea, Michael A.

TI Validation of esophageal squamous cell carcinoma candidate genes from

high-throughput transcriptomic studies

SO AMERICAN JOURNAL OF CANCER RESEARCH

LA English

DT Article

DE Expression microdissection; esophageal squamous cell carcinoma;

RNAscope; immunoblot

ID EXPRESSION MICRODISSECTION; CANCER; CORNULIN

AB In a recent study, a unique gene expression signature was observed when comparing esophageal squamous cell carcinoma (ESCC) epithelial cells to normal esophageal epithelial cells using laser capture microdissection (LCM) and cDNA microarray technology. To validate the expression of several intriguing genes from that study (KRT17, cornulin, CD44, and EpCAM), we employed two new technologies, expression microdissection (xMD) for high-throughput microdissection facilitating protein analysis and RNAscope for the evaluation of low abundant transcripts in situ. For protein measurements, xMD technology was utilized to specifically procure sufficient tumor and normal epithelium from frozen human tissue for immunoblot analysis of KRT17 (CK17) and cornulin. A novel in situ hybridization method (RNAscope) was used to determine the transcript level of two relatively low expressed genes, CD44 and EpCAM in both individual formalin-fixed paraffin-embedded (FFPE) tissue sections and in an ESCC tissue microarray (TMA). The results successfully confirmed the initial expression pattern observed for all four genes, potentially implicating them in the pathogenesis of ESCC. Additionally, the study provides important methodological information on the overall process of candidate gene validation.

C1 [Du, Qiang; Yan, Wusheng; Armani, Michael D.; Mukherjee, Sumana; Emmert-Buck, Michael R.; Tangrea, Michael A.] NCI, Pathogenet Unit, Pathol Lab, Ctr Canc Res,NIH, Bethesda, MD 20892 USA.

[Burton, Victoria H.; Hewitt, Stephen M.] NCI, Tissue Array Res Program, Appl Mol Pathol Lab, NIH, Bethesda, MD 20892 USA.

[Wang, Lemin; Hu, Nan; Taylor, Philip R.] NCI, Genet Epidemiol Branch, Div Canc Epidemiol & Genet, NIH, Bethesda, MD 20892 USA.

RP Emmert-Buck, MR (corresponding author), NCI, Pathogenet Unit, Pathol Lab, Ctr Canc Res,NIH, Bethesda, MD 20892 USA.

EM buckm@mail.nih.gov

FU Center for Cancer Research, National Cancer Institute, National

Institutes of Health; NATIONAL CANCER INSTITUTE [ZICBC010923] Funding

Source: NIH RePORTER

FX This work was supported by the intramural program of the Center for

Cancer Research, National Cancer Institute, National Institutes of

Health. M.R.E.-B. and M.A.T. are inventors on NIH held patents covering

expression microdissection and are entitled to receive royalty-based

payments through the NIH technology transfer program.

CR Contzler R, 2005, J INVEST DERMATOL, V124, P990, DOI 10.1111/j.0022-202X.2005.23694.x

DePianto D, 2010, NAT GENET, V42, P910, DOI 10.1038/ng.665

Hanson JC, 2011, NAT PROTOC, V6, P457, DOI 10.1038/nprot.2010.202

Ide M, 2012, ANN SURG ONCOL, V19, P3506, DOI 10.1245/s10434-012-2437-9

Jemal A, 2010, CA-CANCER J CLIN, V60, P277, DOI 10.3322/caac.20073

Kamangar F, 2006, J CLIN ONCOL, V24, P2137, DOI 10.1200/JCO.2005.05.2308

Morra L, 2011, VIRCHOWS ARCH, V459, P465, DOI 10.1007/s00428-011-1151-5

Mukherjee S, 2009, AM J PATHOL, V175, P2277, DOI 10.2353/ajpath.2009.090013

Pawar H, 2013, ACTA HISTOCHEM, V115, P89, DOI 10.1016/j.acthis.2012.04.003

Richardson AM, 2007, DIAGN MOL PATHOL, V16, P189, DOI 10.1097/PDM.0b013e3180de20ac

Roye GD, 1996, INT J CANCER, V69, P254, DOI 10.1002/(SICI)1097-0215(19960822)69:4<254::AID-IJC2>3.0.CO;2-W

Shou JZ, 2008, CANCER EPIDEM BIOMAR, V17, P1424, DOI 10.1158/1055-9965.EPI-06-0666

Stoecklein NH, 2006, BMC CANCER, V6, DOI 10.1186/1471-2407-6-165

Tangrea MA, 2004, DIAGN MOL PATHOL, V13, P207, DOI 10.1097/01.pdm.0000135964.31459.bb

Went P, 2008, J CANC MOL, V3, P169

Yan Wusheng, 2012, BMC Res Notes, V5, P73, DOI 10.1186/1756-0500-5-73

Zhao JS, 2011, PLOS ONE, V6, DOI 10.1371/journal.pone.0021419

NR 17

TC 13

Z9 14

U1 0

U2 1

PU E-CENTURY PUBLISHING CORP

PI MADISON

PA 40 WHITE OAKS LN, MADISON, WI 53711 USA

SN 2156-6976

J9 AM J CANCER RES

JI Am. J. Cancer Res.

PY 2013

VL 3

IS 4

BP 402

EP 410

PG 9

WC Oncology

WE Science Citation Index Expanded (SCI-EXPANDED)

SC Oncology

GA AA4MJ

UT WOS:000331069400006

PM 23977449

DA 2022-05-19

ER

PT J

AU Hui, MKC

Lai, KKY

Chan, KW

Luk, JM

Lee, NP

Chung, Y

Cheung, LC

Srivastava, G

Tsao, SW

Tang, JC

Law, S

AF Hui, Marco K. C.

Lai, Kenneth K. Y.

Chan, Kwok Wah

Luk, John M.

Lee, Nikki P.

Chung, Yvonne

Cheung, Leo C.

Srivastava, Gopesh

Tsao, Sai Wah

Tang, Johnny C.

Law, Simon

TI Prognostic significance of phosphorylated RON in esophageal squamous

cell carcinoma

SO MEDICAL ONCOLOGY

LA English

DT Article

DE Esophageal squamous cell carcinoma; Phosphorylated RON; Overexpression;

Poor prognostic marker; Median survival after surgery

ID RECEPTOR TYROSINE KINASE; GROWTH-FACTOR RECEPTOR; NUCLEAR-LOCALIZATION;

PANCREATIC-CANCER; ESTABLISHMENT; EXPRESSION; PRODUCT; SIGNALS; LINE;

MET

AB Esophageal squamous cell carcinoma (ESCC) is the most common type of esophageal cancer. RON is a transmembrane receptor overexpressed in various cancers; however, the clinical significance of its phosphorylated form (pRON) is not fully deciphered. This report is the first to investigate the expression and clinical significance of pRON in human ESCC. Quantitative polymerase chain reaction revealed an up-regulation of RON mRNA in 70% (7/10) of ESCC tissues when compared to the adjacent nontumor tissues. An overexpression of pRON protein was found in most of the ESCC cell lines studied (4/5) when compared to two non-neoplastic esophageal epithelial cells using immunoblot. In 64 ESCC tissues, pRON was localized at the cell membrane, cytoplasm and nucleus in 15 (23.4%), 63 (98.4%) and 61 (95.3%) cases using immunohistochemistry. Patients having high expression of cytoplasmic pRON significantly associated with shorter median survival when compared to those with low expression (25.41 months vs. 14.43 months), suggesting cytoplasmic pRON as a potential marker for poor prognosis in ESCC patients.

C1 [Hui, Marco K. C.; Lai, Kenneth K. Y.; Lee, Nikki P.; Chung, Yvonne; Cheung, Leo C.; Law, Simon] Univ Hong Kong, Queen Mary Hosp, Dept Surg, Pokfulam, Hong Kong, Peoples R China.

[Chan, Kwok Wah; Srivastava, Gopesh] Univ Hong Kong, Dept Pathol, Pokfulam, Hong Kong, Peoples R China.

[Luk, John M.] Natl Univ Hlth Syst, Dept Pharmacol & Surg, Singapore, Singapore.

[Tsao, Sai Wah] Univ Hong Kong, Dept Anat, Pokfulam, Hong Kong, Peoples R China.

[Tang, Johnny C.] Hong Kong Polytech Univ, Dept Appl Biol & Chem Technol, Pokfulam, Hong Kong, Peoples R China.

RP Law, S (corresponding author), Univ Hong Kong, Queen Mary Hosp, Dept Surg, Pokfulam, Hong Kong, Peoples R China.

EM slaw@hkucc.hku.hk

RI Law, Simon/C-4324-2009; Luk, John/A-4085-2008; Tang, Johnny C.

O./M-9639-2014; Tang, Johnny Cheuk-on/W-7214-2019

OI Law, Simon/0000-0002-6518-5806; Luk, John/0000-0002-6323-7940; Tang,

Johnny C. O./0000-0002-4261-3206; Tang, Johnny

Cheuk-on/0000-0002-4261-3206

CR ANDERSON LL, 1982, CANCER, V50, P1587, DOI 10.1002/1097-0142(19821015)50:8<1587::AID-CNCR2820500820>3.0.CO;2-S

Camp ER, 2007, CANCER-AM CANCER SOC, V109, P1030, DOI 10.1002/cncr.22490

Chen YQ, 2000, EXP CELL RES, V261, P229, DOI 10.1006/excr.2000.5012

Cheng HL, 2005, BRIT J CANCER, V92, P1906, DOI 10.1038/sj.bjc.6602593

Cheung LCM, 2007, CANCER GENET CYTOGEN, V178, P17, DOI 10.1016/j.cancergencyto.2007.05.026

Chung Y, 2007, ANN SURG ONCOL, V14, P3260, DOI 10.1245/s10434-007-9511-8

Hsu PY, 2006, J UROLOGY, V176, P2262, DOI 10.1016/j.juro.2006.07.048

Hu YC, 2000, CANCER GENET CYTOGEN, V118, P112, DOI 10.1016/S0165-4608(99)00193-4

Hu YC, 2002, CANCER GENET CYTOGEN, V135, P120, DOI 10.1016/S0165-4608(01)00580-5

Iwama A, 1996, EMBO J, V15, P5866, DOI 10.1002/j.1460-2075.1996.tb00973.x

Jemal A, 2011, CA-CANCER J CLIN, V61, P69, DOI [10.3322/caac.20107, 10.3322/caac.20115]

Kaku N, 2008, ENDOCRINOLOGY, V149, P3960, DOI 10.1210/en.2008-0137

Lange A, 2007, J BIOL CHEM, V282, P5101, DOI 10.1074/jbc.R600026200

Lee HJ, 2002, J BIOL CHEM, V277, P6318, DOI 10.1074/jbc.M110371200

Lee NP, 2010, INT J CANCER, V127, P968, DOI 10.1002/ijc.25100

Lee NP, 2009, J PROTEOME RES, V8, P1293, DOI 10.1021/pr800637z

Lee NPY, 2008, PROTEOMICS, V8, P2136, DOI 10.1002/pmic.200700590

Lee NPY, 2006, FEBS LETT, V580, P923, DOI 10.1016/j.febslet.2006.01.019

Lee WY, 2005, CLIN CANCER RES, V11, P2222, DOI 10.1158/1078-0432.CCR-04-1761

Lin SY, 2001, NAT CELL BIOL, V3, P802, DOI 10.1038/ncb0901-802

Liu LX, 2009, HEPATOLOGY, V50, P1453, DOI 10.1002/hep.23143

Nakai K, 1999, TRENDS BIOCHEM SCI, V24, P34, DOI 10.1016/S0968-0004(98)01336-X

Ni CY, 2001, SCIENCE, V294, P2179, DOI 10.1126/science.1065412

Okino T, 1999, INT J ONCOL, V15, P709

Peace BE, 2001, ONCOGENE, V20, P6142, DOI 10.1038/sj.onc.1204836

Pozner-Moulis S, 2006, CANCER RES, V66, P7976, DOI 10.1158/0008-5472.CAN-05-4335

Rampino T, 2003, AM J SURG PATHOL, V27, P779, DOI 10.1097/00000478-200306000-00008

Schlessinger J, 2006, CELL, V127, P45, DOI 10.1016/j.cell.2006.09.013

Tang JCO, 2001, CANCER GENET CYTOGEN, V124, P36, DOI 10.1016/S0165-4608(00)00317-4

Thomas RM, 2007, CANCER RES, V67, P6075, DOI 10.1158/0008-5472.CAN-06-4128

Wang MH, 2007, J PATHOL, V213, P402, DOI 10.1002/path.2245

Wang MH, 1996, EXP CELL RES, V226, P39, DOI 10.1006/excr.1996.0200

Wang MH, 2000, CARCINOGENESIS, V21, P1507, DOI 10.1093/carcin/21.8.1507

WANG MH, 1994, SCIENCE, V266, P117, DOI 10.1126/science.7939629

Zhang H, 2006, CANCER GENET CYTOGEN, V165, P25, DOI 10.1016/j.cancergencyto.2005.07.016

Zhang H, 2007, CANCER LETT, V245, P184, DOI 10.1016/j.canlet.2006.01.005

NR 36

TC 6

Z9 7

U1 0

U2 3

PU HUMANA PRESS INC

PI TOTOWA

PA 999 RIVERVIEW DRIVE SUITE 208, TOTOWA, NJ 07512 USA

SN 1357-0560

EI 1559-131X

J9 MED ONCOL

JI Med. Oncol.

PD SEP

PY 2012

VL 29

IS 3

BP 1699

EP 1706

DI 10.1007/s12032-011-0112-9

PG 8

WC Oncology

WE Science Citation Index Expanded (SCI-EXPANDED)

SC Oncology

GA 994WE

UT WOS:000307964100044

PM 22086736

DA 2022-05-19

ER

PT J

AU Lin, GS

Luo, HC

Fu, ZC

Liao, SG

Cai, LJ

Zhu, JF

AF Lin, Gui-Shan

Luo, Hua-Chun

Fu, Zhi-Chao

Liao, Shao-Guang

Cai, Lv-Juan

Zhu, Jin-Feng

TI Does brain radiotherapy improve survival in patients with esophageal

squamous cell carcinoma with brain metastasis?

SO ANNALS OF PALLIATIVE MEDICINE

LA English

DT Article

DE Esophageal squamous cell carcinoma (ESCC); brain metastases (BM); risk

factors; prognosis; whole-brain radiotherapy

ID WHOLE-BRAIN; CANCER STATISTICS; RADIOSURGERY; THERAPY

AB Background: To investigate the clinical characteristics, influencing factors, and their impact on survival in patients with brain metastases from esophageal squamous cell carcinoma (BM-ESCC).

Methods: A total of 67 patients with patients with newly diagnosed BM-ESCC were retrospectively analyzed from December 2000 to December 2016, in order to examine the correlation between clinicopathological characteristics and brain metastases, and between brain metastases and survival.

Results: The number of BM-ESCC was positively correlated with T and N stages (P<0.05). The higher the T and N stages, the higher the incidence. The median survival time was 9.65 months. N stage was an independent risk factor for BM-ESCC. N0 + N1 was associated with a lower risk of brain metastases (P<0.05). Patients with 1 brain metastasis had a significantly longer survival than those with 2 and 3 brain metastases. N stage-stratified analysis revealed that N0 + N1 patients had a longer survival than N2 and N3 patients (P<0.05). Cox regression analysis revealed that mortality in T3 + T4 patients was 2.337 times that of Tis + T1 patients; mortality in N3 patients was 3.486 times that of N0 + N1 patients; and mortality in untreated patients was 2.772 times that of those treated with whole brain radiotherapy.

Conclusions: The number of BM-ESCC is correlated to T and N stages. The higher the N stage, the higher risk of brain metastases. The higher of T and N stages in ESCC, the worse in prognosis. Whole brain radiotherapy could offer greater survival benefits. Keywords: Esophageal squamous cell carcinoma (ESCC); brain metastases (BM); risk factors; prognosis; whole-brain radiotherapy

C1 [Lin, Gui-Shan; Zhu, Jin-Feng] Fujian Med Univ, Fujian Prov Hosp, Dept Med Oncol, Prov Clin Coll, Fuzhou 350000, Peoples R China.

[Luo, Hua-Chun; Fu, Zhi-Chao; Liao, Shao-Guang; Cai, Lv-Juan] Fujian Med Univ, Joint Logist 900 Hosp, Dept Radiat Oncol, Fuzhou 350000, Peoples R China.

RP Fu, ZC (corresponding author), Fujian Med Univ, Joint Logist 900 Hosp, Dept Radiat Oncol, Fuzhou 350000, Peoples R China.

EM fauster1112@126.com

FU Medical innovation project of Fujian Health Committee [2018-CX-3]

FX This study is supported by Medical innovation project of Fujian Health

Committee (Grant No. 2018-CX-3).

CR Abu Hejleh T, 2012, WORLD J GASTRO ONCOL, V4, P103, DOI 10.4251/wjgo.v4.i5.103

Aoyama H, 2006, JAMA-J AM MED ASSOC, V295, P2483, DOI 10.1001/jama.295.21.2483

Cavanna L, 2018, ONCOL LETT, V15, P5787, DOI 10.3892/ol.2018.8054

Chen WQ, 2016, CA-CANCER J CLIN, V66, P115, DOI 10.3322/caac.21338

Chen XH, 2015, ANN TRANSL MED, V3, DOI 10.3978/j.issn.2305-5839.2015.11.05

Feng W, 2014, J CANCER RES THER, V10, pC296, DOI 10.4103/0973-1482.151536

He LR, 2016, RADIOTHER ONCOL, V121, P70, DOI 10.1016/j.radonc.2016.08.005

Hu Y, 2010, ANN SURG ONCOL, V17, P784, DOI 10.1245/s10434-009-0818-5

Huang CM, 2014, SURG ONCOL, V23, P5, DOI 10.1016/j.suronc.2014.01.003

Kanemoto A, 2011, J RADIAT RES, V52, P509, DOI 10.1269/jrr.10184

Kocher M, 2011, J CLIN ONCOL, V29, P134, DOI 10.1200/JCO.2010.30.1655

Owen S, 2014, FRONT ONCOL, V4, DOI 10.3389/fonc.2014.00248

Parvin S, 2003, Med J Malaysia, V58, P429

Rades D, 2007, INT J RADIAT ONCOL, V69, P1509, DOI 10.1016/j.ijrobp.2007.05.014

Rades D, 2014, ANTICANCER RES, V34, P6763

Rades D, 2012, CANCER-AM CANCER SOC, V118, P3852, DOI 10.1002/cncr.26680

Rodrigues G, 2013, RADIOTHER ONCOL, V106, P206, DOI 10.1016/j.radonc.2012.10.014

Siegel RL, 2017, CA-CANCER J CLIN, V67, P7, DOI 10.3322/caac.21387

Smith RS, 2011, WORLD J GASTROENTERO, V17, P2407, DOI 10.3748/wjg.v17.i19.2407

Spallone A, 2013, ONCOL LETT, V6, P722, DOI 10.3892/ol.2013.1436

Tsao MN, 2012, PRACT RADIAT ONCOL, V2, P210, DOI 10.1016/j.prro.2011.12.004

Wadhwa R, 2013, ONCOLOGY-BASEL, V85, P204, DOI 10.1159/000354736

Weinberg JS, 2003, CANCER-AM CANCER SOC, V98, P1925, DOI 10.1002/cncr.11737

Yoshida S, 2007, SURG NEUROL, V67, P288, DOI 10.1016/j.surneu.2006.05.065

Zu HL, 2013, PLOS ONE, V8, DOI 10.1371/journal.pone.0054502

宋欣, 2017, [中华消化杂志, Chinese Journal of Digestion], V37, P106

NR 26

TC 1

Z9 1

U1 0

U2 0

PU AME PUBL CO

PI SHATIN

PA FLAT-RM C 16F, KINGS WING PLAZA 1, NO 3 KWAN ST, SHATIN, HONG KONG

00000, PEOPLES R CHINA

SN 2224-5820

EI 2224-5839

J9 ANN PALLIAT MED

JI Ann. Pallliat. Med.

PD MAY

PY 2020

VL 9

IS 3

BP 637

EP 643

DI 10.21037/apm.2020.04.23

PG 7

WC Health Care Sciences & Services

WE Science Citation Index Expanded (SCI-EXPANDED)

SC Health Care Sciences & Services

GA LU9DE

UT WOS:000538046500004

PM 32312072

OA gold

DA 2022-05-19

ER

PT J

AU Dobashi, A

Goda, K

Furuhashi, H

Matsui, H

Hara, Y

Kamba, S

Kobayashi, M

Sumiyama, K

Hirooka, S

Hamatani, S

Rajan, E

Ikegami, M

Tajiri, H

AF Dobashi, Akira

Goda, Kenichi

Furuhashi, Hiroto

Matsui, Hiroaki

Hara, Yuko

Kamba, Shunsuke

Kobayashi, Masakuni

Sumiyama, Kazuki

Hirooka, Shinichi

Hamatani, Shigeharu

Rajan, Elizabeth

Ikegami, Masahiro

Tajiri, Hisao

TI Diagnostic efficacy of dual-focus endoscopy with narrow-band imaging

using simplified dyad criteria for superficial esophageal squamous cell

carcinoma

SO JOURNAL OF GASTROENTEROLOGY

LA English

DT Article

DE Narrow-band imaging; Esophageal squamous cell carcinoma; Diagnosis;

Dual-focus; Intrapapillary capillary loop

ID MAGNIFYING ENDOSCOPY; NEOPLASIA; HEAD; SYSTEM; CANCER; DEPTH

AB BackgroundOur previous studies have shown the diagnostic utility of a newly developed dual-focus endoscope with narrow-band imaging (DF-NBI) and simplified dyad criteria for detection of superficial esophageal squamous cell carcinoma (SESCC). This clinical trial aimed to study the diagnostic efficacy of DF-NBI with dyad criteria for detecting SESCC compared to white light imaging (WLI).MethodsThis was a single-arm prospective comparative trial. We enrolled 170 consecutive high-risk patients for esophageal squamous cell carcinoma. Patients were initially examined with WLI by one independent endoscopist and then the other performed DF-NBI blinded to the WLI diagnosis to avoid a carry-over effect. Lesions showing proliferation and/or various shapes of intrapapillary capillary loops (IPCL) under DF-NBI (i.e., dyad criteria) were endoscopically diagnosed as SESCC including high-grade intraepithelial neoplasia. The primary endpoint was sensitivity of WLI and DF-NBI for detecting SESCC. The secondary endpoints were the diagnostic performance (i.e., specificity and accuracy) and inter/intra-observer concordance of DF-NBI with dyad criteria.ResultsA total 77 SESCCs were detected. The sensitivity of DF-NBI for SESCC was significantly higher than that of WLI (91% vs. 51%, P<0.001). The specificity and accuracy of WLI and DF-NBI using dyad criteria were 100% vs. 84%, and 86% vs. 86%, respectively. Various shapes and proliferation of IPCL showed the highest value in inter-observer and intra-observer agreements (=0.77 and 0.82, respectively).ConclusionDF-NBI combined with dyad criteria may be a promising technique with a high sensitivity for diagnosis of SESCC and high inter/intra-observer agreement.

C1 [Dobashi, Akira; Goda, Kenichi; Furuhashi, Hiroto; Matsui, Hiroaki; Hara, Yuko; Kamba, Shunsuke; Kobayashi, Masakuni; Sumiyama, Kazuki] Jikei Univ, Sch Med, Dept Endoscopy, Tokyo, Japan.

[Goda, Kenichi] Dokkyo Med Univ, Dept Gastroenterol, Mibu, Tochigi, Japan.

[Hirooka, Shinichi; Hamatani, Shigeharu; Ikegami, Masahiro] Jikei Univ, Sch Med, Dept Pathol, Tokyo, Japan.

[Dobashi, Akira; Rajan, Elizabeth] Mayo Clin, Div Gastroenterol & Hepatol, Dev Endoscopy Unit, Rochester, MN 55905 USA.

[Tajiri, Hisao] Jikei Univ, Sch Med, Dept Innovat Intervent Endoscopy Res, Tokyo, Japan.

RP Dobashi, A (corresponding author), Jikei Univ, Sch Med, Dept Endoscopy, Tokyo, Japan.; Dobashi, A (corresponding author), Mayo Clin, Div Gastroenterol & Hepatol, Dev Endoscopy Unit, Rochester, MN 55905 USA.

EM akira.dobashi1980@gmail.com

RI Kamba, Shunsuke/AAE-4111-2021

CR [Anonymous], 2007, JAP CLASS ES CANC

Arima H., 2005, ESOPHAGUS, V2, P191, DOI DOI 10.1007/S10388-005-0060-6

Dobashi A, 2016, WORLD J GASTROENTERO, V22, P9196, DOI 10.3748/wjg.v22.i41.9196

Enzinger PC, 2003, NEW ENGL J MED, V349, P2241, DOI 10.1056/NEJMra035010

Ferlay J, 2015, INT J CANCER, V136, pE359, DOI 10.1002/ijc.29210

Gabbert HE, 2000, PATHOLOGY GENETICS T, P1

Goda K, 2016, ENDOSCOPY, V48, P321, DOI 10.1055/s-0035-1569644

Goda K, 2015, GASTROENT RES PRACT, V2015, DOI 10.1155/2015/639462

Goda K, 2014, DIGEST ENDOSC, V26, P1, DOI 10.1111/den.12220

Gono K, 2003, OPT REV, V10, P211, DOI 10.1007/s10043-003-0211-8

Gwet, 2002, SERIES STAT METHODS, DOI DOI 10.1007/SPRINGERREFERENCE_183942]

Inoue H., 1997, DIGEST ENDOSC, V9, P16, DOI 10.1111/J.1443-1661.1997.TB00453.X

Ishihara R, 2010, J GASTROEN HEPATOL, V25, P1410, DOI 10.1111/j.1440-1746.2010.06378.x

Kaga M, 2011, ONCOL REP, V26, P1063, DOI 10.3892/or.2011.1398

Kanzaki H, 2013, J GASTROEN HEPATOL, V28, P274, DOI 10.1111/jgh.12059

Kodama M, 1998, SURGERY, V123, P432, DOI 10.1016/S0039-6060(98)70165-5

Kumagai Y, 2014, JPN J CLIN ONCOL, V44, P526, DOI 10.1093/jjco/hyu039

LANDIS JR, 1977, BIOMETRICS, V33, P159, DOI 10.2307/2529310

Lee CT, 2010, ENDOSCOPY, V42, P613, DOI 10.1055/s-0030-1255514

Lv J, 2013, WORLD J GASTROENTERO, V19, P8391, DOI 10.3748/wjg.v19.i45.8391

Minami H, 2012, GASTROENT RES PRACT, V2012, DOI 10.1155/2012/529782

Mochizuki Yosuke, 2012, J Gastrointest Cancer, V43, P599, DOI 10.1007/s12029-012-9395-0

Muto M, 2002, CARCINOGENESIS, V23, P1759, DOI 10.1093/carcin/23.10.1759

Muto M, 2010, J CLIN ONCOL, V28, P1566, DOI 10.1200/JCO.2009.25.4680

Nagami Y, 2014, AM J GASTROENTEROL, V109, P845, DOI 10.1038/ajg.2014.94

Ono S, 2009, GASTROINTEST ENDOSC, V70, P860, DOI 10.1016/j.gie.2009.04.044

Oyama T, 2017, ESOPHAGUS-TOKYO, V14, P105, DOI 10.1007/s10388-016-0527-7

Prueksapanich P, 2015, ENDOSC INT OPEN, V3, pE281, DOI 10.1055/s-0034-1391903

Sato H, 2015, ENDOSCOPY, V47, P122, DOI 10.1055/s-0034-1390858

Sharma P, 2007, GASTROENTEROLOGY, V133, P454, DOI 10.1053/j.gastro.2007.06.006

Shimizu Y, 2008, J GASTROEN HEPATOL, V23, P546, DOI 10.1111/j.1440-1746.2007.04990.x

Szura M, 2016, SURG ENDOSC, V30, P204, DOI 10.1007/s00464-015-4186-x

Takahashi M, 2014, J GASTROEN HEPATOL, V29, P762, DOI 10.1111/jgh.12477

Wallace MB, 2014, GASTROINTEST ENDOSC, V80, P1072, DOI 10.1016/j.gie.2014.05.305

Yoshida T, 2004, GASTROINTEST ENDOSC, V59, P288, DOI 10.1016/S0016-5107(03)02532-X

NR 35

TC 8

Z9 8

U1 1

U2 3

PU SPRINGER JAPAN KK

PI TOKYO

PA CHIYODA FIRST BLDG EAST, 3-8-1 NISHI-KANDA, CHIYODA-KU, TOKYO, 101-0065,

JAPAN

SN 0944-1174

EI 1435-5922

J9 J GASTROENTEROL

JI J. Gastroenterol.

PD JUN

PY 2019

VL 54

IS 6

BP 501

EP 510

DI 10.1007/s00535-018-1527-2

PG 10

WC Gastroenterology & Hepatology

WE Science Citation Index Expanded (SCI-EXPANDED)

SC Gastroenterology & Hepatology

GA IA0DQ

UT WOS:000469227400003

PM 30406847

DA 2022-05-19

ER

PT J

AU Lyu, SS

Lu, JB

Chen, WD

Huang, WY

Huang, HQ

Xi, SY

Yan, SM

AF Lyu, Shanshan

Lu, Jiabin

Chen, Wendan

Huang, Weiye

Huang, Haoqi

Xi, Shaoyan

Yan, Shumei

TI High expression of eIF4A2 is associated with a poor prognosis in

esophageal squamous cell carcinoma

SO ONCOLOGY LETTERS

LA English

DT Article

DE eukaryotic initiation factor 4A-II; immunohistochemistry; esophageal

squamous cell carcinoma; prognosis

ID TRANSLATION INITIATION; CANCER; SURVIVAL; RNA

AB Eukaryotic initiation factor 4A-II (eIF4A2) is an ATP-dependent RNA helicase involved in mRNA translation. Abnormal expression of eIF4A2 has been reported as a prognostic factor in different types of cancer. However, little is known regarding the function of eIF4A2 in esophageal squamous cell carcinoma (ESCC). In the present study, 253 samples were collected from patients diagnosed with ESCC, and the expression of eIF4A2 was detected by immunohistochemical staining. The clinicopathological and prognostic significance of eIF4A2 expression in ESCC were then statistically analyzed. The results demonstrated that eIF4A2 was specifically localized to the cytoplasm. Kaplan-Meier analysis also revealed that eIF4A2 expression was associated with the clinical prognosis of patients with ESCC. The median disease-free and overall survival times were 40 and 48 months for patients with low eIF4A2 expression, compared with 16 and 25 months in the high eIF4A2 expression group, respectively. In conclusion, high expression levels of eIF4A2 are associated with a poor prognosis and may be used as a potential prognostic indicator in patients with ESCC.

C1 [Lyu, Shanshan; Chen, Wendan; Huang, Weiye; Huang, Haoqi] Guangdong Acad Med Sci, Guangdong Prov Peoples Hosp, Dept Pathol, Guangzhou 510080, Guangdong, Peoples R China.

[Lu, Jiabin; Xi, Shaoyan; Yan, Shumei] Collaborat Innovat Ctr Canc Med, State Key Lab Oncol South China, 651 Dong Feng Rd East, Guangzhou 510060, Guangdong, Peoples R China.

[Lu, Jiabin; Xi, Shaoyan; Yan, Shumei] Sun Yat Sen Univ, Dept Pathol, Canc Ctr, Guangzhou 510060, Guangdong, Peoples R China.

RP Xi, SY; Yan, SM (corresponding author), Collaborat Innovat Ctr Canc Med, State Key Lab Oncol South China, 651 Dong Feng Rd East, Guangzhou 510060, Guangdong, Peoples R China.

EM xishy@sysucc.org.cn; yanshm@sysucc.org.cn

CR Aitken CE, 2012, NAT STRUCT MOL BIOL, V19, P568, DOI 10.1038/nsmb.2303

Bao Y, 2015, CANCER CELL INT, V15, DOI 10.1186/s12935-015-0250-9

Bhat M, 2015, NAT REV DRUG DISCOV, V14, P261, DOI 10.1038/nrd4505

Bitterman PB, 2015, BBA-GENE REGUL MECH, V1849, P774, DOI 10.1016/j.bbagrm.2014.09.007

Bray F, 2018, CA-CANCER J CLIN, V68, P394, DOI 10.3322/caac.21492

Chan CC, 2004, RNA, V10, P200, DOI 10.1261/rna.5230104

Chan K, 2019, NAT COMMUN, V10, DOI 10.1038/s41467-019-13086-5

Chen MF, 2013, ANN SURG ONCOL, V20, P3023, DOI 10.1245/s10434-013-2935-4

Chen ZH, 2019, J EXP CLIN CANC RES, V38, DOI 10.1186/s13046-019-1178-z

Cheng YF, 2018, CANCER MED-US, V7, P4193, DOI 10.1002/cam4.1499

Dancau AM, 2010, METHODS MOL BIOL, V576, P49, DOI 10.1007/978-1-59745-545-9_4

DONZE O, 1995, EMBO J, V14, P3828, DOI 10.1002/j.1460-2075.1995.tb00052.x

Gholipour C, 2008, DIS ESOPHAGUS, V21, P322, DOI 10.1111/j.1442-2050.2007.00776.x

Hinnebusch AG, 2014, ANNU REV BIOCHEM, V83, P779, DOI 10.1146/annurev-biochem-060713-035802

Hinnebusch AG, 2012, CSH PERSPECT BIOL, V4, DOI 10.1101/cshperspect.a011544

Ji P, 2003, ONCOGENE, V22, P8031, DOI 10.1038/sj.onc.1206928

Li QY, 1999, MOL CELL BIOL, V19, P7336

Liang SH, 2014, INT J GYNECOL CANCER, V24, P908, DOI 10.1097/IGC.0000000000000152

Lomnytska MI, 2012, BRIT J CANCER, V106, P1297, DOI 10.1038/bjc.2012.67

Meijer HA, 2013, SCIENCE, V340, P82, DOI 10.1126/science.1231197

Meijer HA, 2019, NUCLEIC ACIDS RES, V47, P8224, DOI 10.1093/nar/gkz509

Modelska A, 2015, CELL DEATH DIS, V6, DOI 10.1038/cddis.2014.542

Oblinger JL, 2016, NEURO-ONCOLOGY, V18, P1265, DOI 10.1093/neuonc/now032

Pickens A, 2003, ANN THORAC SURG, V76, pS1367, DOI 10.1016/S0003-4975(03)01202-5

Rice TW., 2017, AJCC CANC STAGING MA, V8th, P185

Shahbazian D, 2010, MOL CELL BIOL, V30, P1478, DOI 10.1128/MCB.01218-09

Smyth EC, 2017, NAT REV DIS PRIMERS, V3, DOI 10.1038/nrdp.2017.48

Spilka R, 2013, CANCER LETT, V340, P9, DOI 10.1016/j.canlet.2013.06.019

Wilczynska A, 2019, GENOME BIOL, V20, DOI 10.1186/s13059-019-1857-2

Wolfe AL, 2014, NATURE, V513, P65, DOI 10.1038/nature13485

Xi SY, 2013, CLIN LUNG CANCER, V14, P658, DOI [10.1016/j.cllc.2013.04.011, 10.1016/j.dlc.2013.04.011]

Yan LX, 2011, BREAST CANCER RES, V13, DOI 10.1186/bcr2803

NR 32

TC 2

Z9 2

U1 1

U2 5

PU SPANDIDOS PUBL LTD

PI ATHENS

PA POB 18179, ATHENS, 116 10, GREECE

SN 1792-1074

EI 1792-1082

J9 ONCOL LETT

JI Oncol. Lett.

PD NOV

PY 2020

VL 20

IS 5

AR 177

DI 10.3892/ol.2020.12038

PG 8

WC Oncology

WE Science Citation Index Expanded (SCI-EXPANDED)

SC Oncology

GA OS4ZJ

UT WOS:000590173300054

PM 32934744

OA Green Published, gold

DA 2022-05-19

ER

PT J

AU He, SJ

Peng, J

Li, L

Xu, Y

Wu, XX

Yu, J

Liu, JL

Zhang, JG

Zhang, RY

Wang, W

AF He, Shujin

Peng, Jie

Li, Lei

Xu, Ying

Wu, Xiaoxiao

Yu, Juan

Liu, Jianli

Zhang, Jinguo

Zhang, Renya

Wang, Wei

TI High expression of cytokeratin CAM5.2 in esophageal squamous cell

carcinoma is associated with poor prognosis

SO MEDICINE

LA English

DT Article

DE CAM5.2; esophageal squamous cell carcinoma; prognosis

ID TISSUE MICROARRAY TECHNOLOGY; MONOCLONAL-ANTIBODY; CANCER; REAGENT;

CK18; CK8

AB Esophageal cancer is a common human malignant tumor with high mortality. Glandular epithelial markers, such as CAM5.2, can be expressed in esophageal squamous cell carcinoma (ESCC), but the clinical significance of these cells in ESCC remains elusive.

Immunohistochemical analysis of CAM5.2 was performed on 604 ESCC specimens using tissue microarray. Our study design and study population used retrospective cohorts based on the hospital information system and pathological information management system which included medical information, date of admission, procedures undergone, registration, examinations, and medication.

In total, positive staining of CAM5.2 was 145 of 604 (24%). Statistical analysis showed that the expression of CAM5.2 had no relationship with sex, age, tumor differentiation, tumor size, tumor-node-metastasis (TNM) classification, and lymph node metastasis, but it was significantly associated with poor prognosis of overall survival (P=.0041) and disease-free survival (P=.0048) in ESCC patients.

Herein, we report for the first time that the high expression of the CAM 5.2 is an independent predictor of poor prognosis in patients with ESCC.

C1 [He, Shujin; Peng, Jie; Li, Lei; Xu, Ying; Wu, Xiaoxiao; Yu, Juan; Liu, Jianli; Zhang, Renya; Wang, Wei] Jining Med Univ, Affiliated Hosp, Dept Pathol, Jining 272029, Shandong, Peoples R China.

[Zhang, Jinguo] Jining Med Univ, Affiliated Hosp, Dept Cardiovasol, Jining 272029, Shandong, Peoples R China.

RP Zhang, RY; Wang, W (corresponding author), Jining Med Univ, Affiliated Hosp, Dept Pathol, Jining 272029, Shandong, Peoples R China.

EM zhangrenya@mail.jnmc.edu.cn; wangwei1985@csu.edu.cn

FU National Natural Science Foundation of China [81802945]; Natural Science

Foundation of Shandong Province, China [ZR2016HQ28]; Science and

Technology Development Project of Jining [2016-56-28]; PhD Research

Foundation Affiliated Hospital of Jining Medical University

[2016-BS-002]; cultivation project of the National Natural Science

Foundation of Jining Medical University [JYP201733, JYP201731]; Research

support found for young teachers of Jining Medical University

[JYFC2018FKJ036, JY2017FS007]; Jining Science and Technology Key

Research and Development Plan [2018SMNS006]; Shandong Traditional

Chinese Medicine Science and Technology Development Plan Project

[2017-277]

FX This research was supported by the National Natural Science Foundation

of China (81802945); the Natural Science Foundation of Shandong

Province, China (ZR2016HQ28); the Science and Technology Development

Project of Jining (2016-56-28); the PhD Research Foundation Affiliated

Hospital of Jining Medical University (2016-BS-002); the cultivation

project of the National Natural Science Foundation of Jining Medical

University (JYP201733, JYP201731); Research support found for young

teachers of Jining Medical University (JYFC2018FKJ036, JY2017FS007);

Jining Science and Technology Key Research and Development Plan

(2018SMNS006); Shandong Traditional Chinese Medicine Science and

Technology Development Plan Project (2017-277).

CR Arnold M, 2017, AM J GASTROENTEROL, V112, P1247, DOI 10.1038/ajg.2017.155

Boone J, 2008, VIRCHOWS ARCH, V452, P507, DOI 10.1007/s00428-008-0602-0

Bray F, 2018, CA-CANCER J CLIN, V68, P394, DOI 10.3322/caac.21492

Chang KL, 2014, CHEST, V145, P1441, DOI 10.1378/chest.14-0168

Chen PJ, 2010, ENDOCR PATHOL, V21, P69, DOI 10.1007/s12022-010-9108-9

Chu PG, 2009, ENDOCR PATHOL, V20, P1, DOI 10.1007/s12022-009-9061-7

Dadlchah E, 2013, ARCH IRAN MED, V16, P463, DOI 013168/AIm.006

Forghanifard MM, 2014, MED ONCOL, V31, DOI 10.1007/s12032-014-0191-5

Goyal R, 2015, J NEUROSCI RURAL PRA, V6, P499, DOI 10.4103/0976-3147.168426

Hoff PM, 2017, SURG ONCOL CLIN N AM, V26, P57, DOI 10.1016/j.soc.2016.07.013

Hu N, 2010, BMC GENOMICS, V11, DOI 10.1186/1471-2164-11-576

Irizarry RA, 2003, NUCLEIC ACIDS RES, V31, DOI 10.1093/nar/gng015

Kallioniemi OP, 2001, HUM MOL GENET, V10, P657, DOI 10.1093/hmg/10.7.657

Koolen BB, 2014, ANN SURG ONCOL, V21, P700, DOI 10.1245/s10434-013-3273-2

LAM KY, 1995, VIRCHOWS ARCH, V426, P345

Leei CK, 2010, INT J CLIN EXP PATHO, V3, P742

Moghbeli M, 2016, J CELL COMMUN SIGNAL, V10, P129, DOI 10.1007/s12079-016-0320-3

Moghbeli M, 2016, J CELL COMMUN SIGNAL, V10, P49, DOI 10.1007/s12079-015-0314-6

Moll R, 2008, HISTOCHEM CELL BIOL, V129, P705, DOI 10.1007/s00418-008-0435-6

Niu YJ, 2011, PLOS ONE, V6, DOI 10.1371/journal.pone.0020202

Oue N, 2012, ANN SURG ONCOL, V19, P1902, DOI 10.1245/s10434-011-2175-4

Qian X, 2016, ONCOTARGETS THER, V9, P2247, DOI 10.2147/OTT.S103179

Shimoyama M, 2003, DIS COLON RECTUM, V46, P333, DOI 10.1007/s10350-004-6552-y

Wang H, 2011, ORAL ONCOL, V47, P775, DOI 10.1016/j.oraloncology.2010.08.012

Wang W, 2017, J CELL PHYSIOL, V232, P2829, DOI 10.1002/jcp.25706

Wang W, 2016, ONCOTARGET, V7, P16745, DOI 10.18632/oncotarget.7778

Wang W, 2014, CANCER LETT, V348, P109, DOI 10.1016/j.canlet.2014.03.005

Xu Y, 2018, NEOPLASMA, V65, P469, DOI 10.4149/neo_2018_170529N384

Yamada A, 2008, ONCOL REP, V20, P1021, DOI 10.3892/or_00000104

NR 29

TC 13

Z9 13

U1 1

U2 5

PU LIPPINCOTT WILLIAMS & WILKINS

PI PHILADELPHIA

PA TWO COMMERCE SQ, 2001 MARKET ST, PHILADELPHIA, PA 19103 USA

SN 0025-7974

EI 1536-5964

J9 MEDICINE

JI Medicine (Baltimore)

PD SEP

PY 2019

VL 98

IS 37

AR e17104

DI 10.1097/MD.0000000000017104

PG 6

WC Medicine, General & Internal

WE Science Citation Index Expanded (SCI-EXPANDED)

SC General & Internal Medicine

GA JB7ZM

UT WOS:000488784400033

PM 31517842

OA Green Published, gold

DA 2022-05-19

ER

PT J

AU Dai, FQ

Mei, LY

Meng, SL

Ma, Z

Gao, W

Zhou, JH

Zhang, JG

AF Dai Fuqiang

Mei Longyong

Meng Shenglan

Ma Zheng

Gao Wei

Zhou Jinghai

Zhang Jingge

TI The global expression profiling in esophageal squamous cell carcinoma

SO GENOMICS

LA English

DT Article

DE Long no-coding RNAs; microRNAs; mRNAs; Expression profiling; Esophageal

squamous cell carcinoma

ID GASTRIC-CANCER; INVASION; RNA; PROGRESSION; PROMOTES; PROLIFERATION;

MIGRATION; MICRORNA; GROWTH; METASTASIS

AB Esophageal squamous cell carcinoma (ESCC) is the dominant subtype of esophageal cancer worldwide. This study aimed to explore the aberrant global expression profiling and construct regulatory network in ESCC for understanding tumorigenesis of ESCC. The expression pattern of long non-coding RNA (lncRNA), microRNA (miRNA) and mRNA was measured by RNA-sequencing in ESCC. Differentially expressed lncRNAs/miRNAs/mRNAs (DELs/DEMs/DEMIs) were identified in ESCC. DEMIs-DEMs network was constructed; hsa-miR-424-5p and hsa-miR-450b-5p were the hub miRNAs in the network, which negatively regulated 19 and 17 DEMs. DEMs targeted by DEMIs were significantly enriched in MAPK signaling pathway, pathways in cancer and focal adhesion signaling pathway. The expression of candidate DEMs and DEMIs in ESCC were validated through quantitative real-time polymerase chain reaction and microarray expression profiling analyses, and the results were generally consistent with our bioinformatics analysis. Our results might provide useful information for exploring the tumorigenesis mechanism and potentially therapeutic targets in ESCC. (C) 2017 Elsevier Inc. All rights reserved.

C1 Third Mil Med Univ, Daping Hosp, Inst Surg Res, Dept Thorac Surg, Chongqing, Peoples R China.

RP Ma, Z (corresponding author), Third Mil Med Univ, Daping Hosp, Dept Thorac Surg, Chongqing 400042, Peoples R China.

EM daidphospital@163.com

CR Bartel DP, 2009, CELL, V136, P215, DOI 10.1016/j.cell.2009.01.002

Carmona-Saez P, 2007, GENOME BIOL, V8, DOI 10.1186/gb-2007-8-1-r3

Che CL, 2013, INT J CLIN EXP PATHO, V6, P1538

Chen GM, 2014, MED ONCOL, V31, DOI 10.1007/s12032-014-0263-6

Chen PN, 2017, BIOMED PHARMACOTHER, V86, P541, DOI 10.1016/j.biopha.2016.12.050

Cheng LN, 2016, GENE, V595, P31, DOI 10.1016/j.gene.2016.09.028

Arnal MJD, 2015, WORLD J GASTROENTERO, V21, P7933, DOI 10.3748/wjg.v21.i26.7933

Dweep H, 2015, NAT METHODS, V12, P697, DOI 10.1038/nmeth.3485

Enzinger PC, 2003, NEW ENGL J MED, V349, P2241, DOI 10.1056/NEJMra035010

Feng H, 2016, PANCREAS, V45, P1263, DOI 10.1097/MPA.0000000000000677

Friedlander MR, 2012, NUCLEIC ACIDS RES, V40, P37, DOI 10.1093/nar/gkr688

Fu HL, 2013, CELL BIOCHEM BIOPHYS, V67, P657, DOI 10.1007/s12013-013-9554-3

Ghosh S, 2016, METHODS MOL BIOL, V1374, P339, DOI 10.1007/978-1-4939-3167-5_18

Hezova R, 2015, DIAGN PATHOL, V10, DOI 10.1186/s13000-015-0280-6

Hong L, 2015, EXPERT REV GASTROENT, V9, P887, DOI 10.1586/17474124.2015.1041507

Hu CC, 2014, ASIAN PAC J CANCER P, V15, P9439, DOI 10.7314/APJCP.2014.15.21.9439

Huang Y, 2015, ONCOTARGETS THER, V8, P795, DOI 10.2147/OTT.S77378

Kano M, 2010, INT J CANCER, V127, P2804, DOI 10.1002/ijc.25284

Komatsu S, 2016, AM J CANCER RES, V6, P1511

Li X, 2014, EUR REV MED PHARMACO, V18, P2031

Li XF, 2014, CELL BIOCHEM BIOPHYS, V70, P1369, DOI 10.1007/s12013-014-0066-6

Lin XX, 2014, MED ONCOL, V31, DOI 10.1007/s12032-014-0042-4

Liu FX, 2013, MOL CELL BIOCHEM, V381, P115, DOI 10.1007/s11010-013-1693-8

Liu H, 2012, DIS ESOPHAGUS, V25, P656, DOI 10.1111/j.1442-2050.2011.01284.x

Liu LY, 2014, INT J ONCOL, V45, P1257, DOI 10.3892/ijo.2014.2516

Liu M, 2016, CANCER LETT, V377, P97, DOI 10.1016/j.canlet.2016.04.034

LIU R, 2012, PLOS ONE, V7, P33987

Muller S, 2014, BIOMED RES INT, V2014, DOI 10.1155/2014/948408

Nie JH, 2015, ONCOL REP, V34, P311, DOI 10.3892/or.2015.3962

Qi Y, 2015, NEOPLASMA, V62, P384, DOI 10.4149/neo_2015_046

Ren KW, 2016, TRANSL ONCOL, V9, P489, DOI 10.1016/j.tranon.2016.09.005

Schmittgen TD, 2008, NAT PROTOC, V3, P1101, DOI 10.1038/nprot.2008.73

Shannon P, 2003, GENOME RES, V13, P2498, DOI 10.1101/gr.1239303

Shi XJ, 2016, ONCOTARGET, V7, P36407, DOI 10.18632/oncotarget.9239

Shin WS, 2016, ONCOTARGET, V7, P73242, DOI 10.18632/oncotarget.12303

Siegel R, 2012, CA-CANCER J CLIN, V62, P10, DOI 10.3322/caac.20138

Song W, 2016, CLIN CHIM ACTA, V463, P169, DOI 10.1016/j.cca.2016.10.035

Tan D., 2016, DIS ESOPHAGUS, V30, P1

Trapnell C, 2009, BIOINFORMATICS, V25, P1105, DOI 10.1093/bioinformatics/btp120

Truong A, 2016, FEBS J, V283, P704, DOI 10.1111/febs.13625

Wang C, 2016, BRIT J CANCER, V114, P290, DOI 10.1038/bjc.2015.463

Wang ML, 2016, INT J CANCER, V139, P65, DOI 10.1002/ijc.29978

Wang W, 2016, ONCOTARGET, V7, P25668, DOI 10.18632/oncotarget.8257

Wang XJ, 2014, CELL BIOCHEM FUNCT, V32, P625, DOI 10.1002/cbf.3034

Wang YY, 2015, CELL PHYSIOL BIOCHEM, V36, P1587, DOI 10.1159/000430321

Wu TY, 2016, ONCOTARGET, V7, P11553, DOI 10.18632/oncotarget.7270

Xia T, 2016, AM J CANCER RES, V6, P2031

Yan RY, 2016, SCI REP-UK, V6, DOI 10.1038/srep20001

Yang F, 2016, ONCOTARGETS THER, V9, P761, DOI 10.2147/OTT.S97664

Yang YB, 2016, TUMOR BIOL, V37, P1183, DOI 10.1007/s13277-015-3903-3

Yu BQ, 2016, PLOS ONE, V11, DOI 10.1371/journal.pone.0158961

Yu JH, 2016, GENE, V589, P20, DOI 10.1016/j.gene.2016.05.005

Zeng R, 2016, CELL BIOCHEM FUNCT, V34, P133, DOI 10.1002/cbf.3170

Zhang WB, 2015, CELL PHYSIOL BIOCHEM, V37, P1123, DOI 10.1159/000430237

Zheng ST, 2011, MOL BIOL REP, V38, P865, DOI 10.1007/s11033-010-0178-z

NR 55

TC 20

Z9 22

U1 0

U2 6

PU ACADEMIC PRESS INC ELSEVIER SCIENCE

PI SAN DIEGO

PA 525 B ST, STE 1900, SAN DIEGO, CA 92101-4495 USA

SN 0888-7543

EI 1089-8646

J9 GENOMICS

JI Genomics

PD JUL

PY 2017

VL 109

IS 3-4

BP 241

EP 250

DI 10.1016/j.ygeno.2017.04.005

PG 10

WC Biotechnology & Applied Microbiology; Genetics & Heredity

WE Science Citation Index Expanded (SCI-EXPANDED)

SC Biotechnology & Applied Microbiology; Genetics & Heredity

GA EZ2XY

UT WOS:000404575600015

PM 28442363

OA Bronze

DA 2022-05-19

ER

PT J

AU Ohashi, T

Komatsu, S

Ichikawa, D

Miyamae, M

Okajima, W

Imamura, T

Kiuchi, J

Nishibeppu, K

Kosuga, T

Konishi, H

Shiozaki, A

Fujiwara, H

Okamoto, K

Tsuda, H

Otsuji, E

AF Ohashi, Takuma

Komatsu, Shuhei

Ichikawa, Daisuke

Miyamae, Mahito

Okajima, Wataru

Imamura, Taisuke

Kiuchi, Jun

Nishibeppu, Keiji

Kosuga, Toshiyuki

Konishi, Hirotaka

Shiozaki, Atsushi

Fujiwara, Hitoshi

Okamoto, Kazuma

Tsuda, Hitoshi

Otsuji, Eigo

TI Overexpression of PBK/TOPK Contributes to Tumor Development and Poor

Outcome of Esophageal Squamous Cell Carcinoma

SO ANTICANCER RESEARCH

LA English

DT Article

DE PBK/TOPK; esophageal squamous cell carcinoma; prognosis; oncogene

ID ORIGINATED PROTEIN-KINASE; PDZ-BINDING KINASE; CANCER; EXPRESSION; TOPK;

PROLIFERATION; GENE; PBK; P53

AB Background: PDZ-binding kinase/T-celloriginated protein kinase (PBK/TOPK) is a serine-threonine kinase and overexpressed in various types of cancer. PBK/TOPK is associated with tumor cell development and progression through suppression of p53 function. In this study, we tested whether PBK acts as a cancer-promoting factor by being overexpressed in esophageal squamous cell carcinoma (ESCC). Materials and Methods: We analyzed PBK/TOPK expression in 15 ESCC cell lines, and 54 primary ESCC tumors that were curatively resected between 1994 and 2007. Results: Overexpression of the PBK/TOPK protein was detected in 93% (14/15) ESCC cell lines and 19% (10/54) primary ESCC tumor samples, and significantly correlated with macroscopic appearance and tumor depth. PBK/TOPK positivity was independently associated with worse outcome in multivariate analysis (p=0.0235, hazard ratio=3.58). Knockdown of PBK/TOPK using specific siRNAs inhibited the cell proliferation, invasion/migration of PBK/TOPK-overexpressing ESCC cell lines. Conclusion: These findings suggest that PBK/TOPK plays a crucial role in tumor malignant potential through its overexpression in ESCC.

C1 [Ohashi, Takuma; Komatsu, Shuhei; Ichikawa, Daisuke; Miyamae, Mahito; Okajima, Wataru; Imamura, Taisuke; Kiuchi, Jun; Nishibeppu, Keiji; Kosuga, Toshiyuki; Konishi, Hirotaka; Shiozaki, Atsushi; Fujiwara, Hitoshi; Okamoto, Kazuma; Otsuji, Eigo] Kyoto Prefectural Univ Med, Div Digest Surg, Dept Surg, Kyoto, Japan.

[Tsuda, Hitoshi] Natl Canc Ctr, Dept Pathol, Tokyo, Japan.

[Tsuda, Hitoshi] Natl Def Med Coll, Dept Basic Pathol, Tokorozawa, Saitama, Japan.

RP Komatsu, S (corresponding author), Kyoto Prefectural Univ Med, Div Digest Surg, Dept Surg, Kamigyo Ku, 465 Kajii Cho, Kyoto 6028566, Japan.

EM skomatsu@koto.kpu-m.ac.jp

RI Kiuchi, Jun/AAJ-6202-2021; Kosuga, Toshiyuki/AAE-6892-2020

CR Abe Y, 2000, J BIOL CHEM, V275, P21525, DOI 10.1074/jbc.M909629199

ADELAIDE J, 1995, BRIT J CANCER, V71, P64, DOI 10.1038/bjc.1995.13

Ayllon V, 2007, ONCOGENE, V26, P3451, DOI 10.1038/sj.onc.1210142

Gaudet S, 2000, P NATL ACAD SCI USA, V97, P5167, DOI 10.1073/pnas.090102397

Gratas C, 1998, CANCER RES, V58, P2057

He FR, 2010, HUM PATHOL, V41, P415, DOI 10.1016/j.humpath.2009.05.016

Herrero-Martin D, 2009, BRIT J CANCER, V101, P80, DOI 10.1038/sj.bjc.6605104

HOLLSTEIN MC, 1990, P NATL ACAD SCI USA, V87, P9958, DOI 10.1073/pnas.87.24.9958

Hu F, 2010, ONCOGENE, V29, P5464, DOI 10.1038/onc.2010.275

Ikeda Y, 2016, CLIN CANCER RES, V22, P6110, DOI 10.1158/1078-0432.CCR-16-0207

Joel M, 2015, MOL CANCER, V14, DOI 10.1186/s12943-015-0398-x

Kamangar F, 2006, J CLIN ONCOL, V24, P2137, DOI 10.1200/JCO.2005.05.2308

Kashimoto K, 2012, CANCER SCI, V103, P2021, DOI 10.1111/j.1349-7006.2012.02407.x

Kim DJ, 2012, CANCER RES, V72, P3060, DOI 10.1158/0008-5472.CAN-11-3851

Komatsu S, 2015, BRIT J CANCER, V112, P357, DOI 10.1038/bjc.2014.543

Komatsu S, 2009, CARCINOGENESIS, V30, P1139, DOI 10.1093/carcin/bgp116

Matsumoto S, 2004, BIOCHEM BIOPH RES CO, V325, P997, DOI 10.1016/j.bbrc.2004.10.133

Matsuo Y, 2014, SCI TRANSL MED, V6, DOI 10.1126/scitranslmed.3010277

Meng XY, 2011, DIS ESOPHAGUS, V24, P444, DOI 10.1111/j.1442-2050.2010.01159.x

Nandi AK, 2006, LEUKEMIA RES, V30, P437, DOI 10.1016/j.leukres.2005.08.011

Nandi AK, 2007, BIOCHEM BIOPH RES CO, V358, P181, DOI 10.1016/j.bbrc.2007.04.125

Nishimura Y, 2013, BRIT J CANCER, V108, P1324, DOI 10.1038/bjc.2013.65

O'Leary PC, 2013, BMC CANCER, V13, DOI 10.1186/1471-2407-13-175

Park JH, 2006, CANCER RES, V66, P9186, DOI 10.1158/0008-5472.CAN-06-1601

Park JH, 2010, CANCER SCI, V101, P403, DOI 10.1111/j.1349-7006.2009.01400.x

Shi HY, 2011, CANCER LETT, V309, P19, DOI 10.1016/j.canlet.2011.05.010

Shih MC, 2012, ONCOGENE, V31, P2389, DOI 10.1038/onc.2011.419

SHIMADA Y, 1992, CANCER, V69, P277, DOI 10.1002/1097-0142(19920115)69:2<277::AID-CNCR2820690202>3.0.CO;2-C

Singh PK, 2014, IMMUNOBIOLOGY, V219, P469, DOI 10.1016/j.imbio.2014.02.003

Sobin LH, 2010, CANCER-AM CANCER SOC, V116, P5336, DOI 10.1002/cncr.25537

Tsuda H, 2008, BREAST CANCER-TOKYO, V15, P121, DOI 10.1007/s12282-008-0032-5

Zhu F, 2007, GASTROENTEROLOGY, V133, P219, DOI 10.1053/j.gastro.2007.04.048

NR 32

TC 29

Z9 31

U1 0

U2 5

PU INT INST ANTICANCER RESEARCH

PI ATHENS

PA EDITORIAL OFFICE 1ST KM KAPANDRITIOU-KALAMOU RD KAPANDRITI, PO BOX 22,

ATHENS 19014, GREECE

SN 0250-7005

EI 1791-7530

J9 ANTICANCER RES

JI Anticancer Res.

PD DEC

PY 2016

VL 36

IS 12

BP 6457

EP 6466

DI 10.21873/anticanres.11244

PG 10

WC Oncology

WE Science Citation Index Expanded (SCI-EXPANDED)

SC Oncology

GA EG3LW

UT WOS:000390946700030

PM 27919968

OA Bronze

DA 2022-05-19

ER

PT J

AU Hu, JM

Li, L

Chen, YZ

Pang, LJ

Yang, L

Liu, CX

Zhao, J

Chang, B

Zou, H

Qi, Y

Liang, WH

Li, F

AF Hu, J. M.

Li, L.

Chen, Y. Z.

Pang, L. J.

Yang, L.

Liu, C. X.

Zhao, J.

Chang, B.

Zou, H.

Qi, Y.

Liang, W. H.

Li, F.

TI Human papillomavirus type 16 infection may be involved in esophageal

squamous cell carcinoma carcinogenesis in Chinese Kazakh patients

SO DISEASES OF THE ESOPHAGUS

LA English

DT Article

DE carcinogenesis; esophageal squamous cell carcinoma; HPV16; Kazakh; PCR

ID HIGH-RISK REGION; CERVICAL-CANCER; HPV; AREA; MEN; DNA

AB The aim of this study was to investigate human papillomavirus type 16 (HPV16) prevalence in esophageal squamous cell carcinoma (ESCC) in Xinjiang Kazakh patients and its role in ESCC carcinogenesis. One hundred and fifty cases of ESCC and 150 cases of corresponding normal esophageal mucosa (CNGM) samples were collected from north Xinjiang where the Kazakh ethnic group has lived since ancient times. HPV16 infection in ESCC and CNGM was detected by genotype-specific polymerase chain reaction. HPV16 DNA was detected in 55 of 150 ESCC samples (36.7%) and 24 of 150 corresponding normal esophageal mucosa samples (16%) with significant differences (P < 0.001, odds ratio = 3.039, 95% confidence interval: 1.756-5.260). No statistically significant correlations were found between HPV16 infection and the age or gender of patients, tumor site, tumor cell differentiation, or lymph node metastasis (P > 0.05). HPV16 infection is common in cases of ESCC in the Kazakh ethnic group in Xinjiang and may be involved in ESCC carcinogenesis.

C1 [Hu, J. M.; Chen, Y. Z.; Pang, L. J.; Yang, L.; Liu, C. X.; Zhao, J.; Chang, B.; Zou, H.; Qi, Y.; Liang, W. H.; Li, F.] Shihezi Univ, Sch Med, Dept Pathol, Minist Educ, Shihezi 832003, Peoples R China.

[Hu, J. M.; Chen, Y. Z.; Pang, L. J.; Yang, L.; Liu, C. X.; Zhao, J.; Chang, B.; Zou, H.; Qi, Y.; Liang, W. H.; Li, F.] Shihezi Univ, Sch Med, Key Lab Xinjiang Endem & Ethn Dis, Minist Educ, Shihezi 832003, Peoples R China.

[Li, L.] Shihezi Univ, Sch Life Sci, Shihezi 832003, Peoples R China.

RP Li, F (corresponding author), Shihezi Univ, Sch Med, Dept Pathol, Shihezi 832003, Peoples R China.

EM lifeng7855@yahoo.com.cn

FU Ministry of Science and Technology of China [2012AA02A503, 2009BAI82B03,

2010DFB34100]; National Natural Science Foundation [81160301]

FX Ministry of Science and Technology of China (Nos. 2012AA02A503,

2009BAI82B03 and 2010DFB34100), and The National Natural Science

Foundation, No. 81160301.

CR Antonsson A, 2010, CANCER EPIDEM BIOMAR, V19, P2080, DOI 10.1158/1055-9965.EPI-10-0033

Brown LM, 2001, AM J EPIDEMIOL, V153, P114, DOI 10.1093/aje/153.2.114

Chang FJ, 2000, ANTICANCER RES, V20, P3935

Federschneider JM, 2004, AM J OBSTET GYNECOL, V191, P757, DOI 10.1016/j.ajog.2004.03.077

Gao GF, 2006, INT J CANCER, V119, P1354, DOI 10.1002/ijc.21980

Gillison ML, 2008, CANCER-AM CANCER SOC, V113, P3036, DOI 10.1002/cncr.23764

Gillison Maura L, 2003, J Natl Cancer Inst Monogr, P57

Hamilton S R, 2000, WHO CLASSIFICATION T, V9-11

Harris M, 2011, HEAD NECK ONCOL, V3, DOI 10.1186/1758-3284-3-9

Hou C, 2000, HEBEI WORKERS J MED, V17, P27

Hubbard RA, 2003, ARCH PATHOL LAB MED, V127, P940

Iyer A, 2011, J CLIN VIROL, V50, P205, DOI 10.1016/j.jcv.2010.11.015

Kamangar F, 2006, INT J CANCER, V119, P579, DOI 10.1002/ijc.21871

Koshiol J, 2010, INT J CANCER, V127, P93, DOI 10.1002/ijc.25023

Parkin DM, 2005, CA-CANCER J CLIN, V55, P74, DOI 10.3322/canjclin.55.2.74

Peixoto GD, 2001, CANC LETT, V162, P231

Schmitt M, 2010, CANCER RES, V70, P249, DOI 10.1158/0008-5472.CAN-09-2514

Shi SR, 2002, J HISTOCHEM CYTOCHEM, V50, P1005, DOI 10.1177/002215540205000802

Shuyama K, 2007, BRIT J CANCER, V96, P1554, DOI 10.1038/sj.bjc.6603765

Sitas F, 2012, JNCI-J NATL CANCER I, V104, P147, DOI 10.1093/jnci/djr499

Smith JS, 2007, INT J CANCER, V121, P621, DOI 10.1002/ijc.22527

Syrjanen KJ, 2002, J CLIN PATHOL, V55, P721, DOI 10.1136/jcp.55.10.721

Wang XQ, 2010, BMC CANCER, V10, DOI 10.1186/1471-2407-10-19

Zhang DH, 2010, BMC CANCER, V10, DOI 10.1186/1471-2407-10-650

Zou XN, 2002, INT J VITAM NUTR RES, V72, P375, DOI 10.1024/0300-9831.72.6.375

zur Hausen H, 2002, NAT REV CANCER, V2, P342, DOI 10.1038/nrc798

NR 26

TC 10

Z9 10

U1 0

U2 4

PU OXFORD UNIV PRESS INC

PI CARY

PA JOURNALS DEPT, 2001 EVANS RD, CARY, NC 27513 USA

SN 1120-8694

EI 1442-2050

J9 DIS ESOPHAGUS

JI Dis. Esophagus

PD SEP

PY 2013

VL 26

IS 7

BP 703

EP 707

DI 10.1111/dote.12009

PG 5

WC Gastroenterology & Hepatology

WE Science Citation Index Expanded (SCI-EXPANDED)

SC Gastroenterology & Hepatology

GA 227CR

UT WOS:000325086500009

PM 23607265

DA 2022-05-19

ER

PT J

AU Guo, JC

Lin, CC

Lino, CY

Hsieh, MS

Ku, HY

Lien, MY

Shao, YY

Huang, TC

Hsu, CH

AF Guo, Jhe-Cyuan

Lin, Chia-Chi

Lino, Chen-Yuan

Hsieh, Min-Shu

Ku, Hung-Yang

Lien, Ming-Yu

Shao, Yu-Yun

Huang, Ta-Chen

Hsu, Chih-Hung

TI Neutrophil-to-lymphocyte Ratio and Use of Antibiotics Associated With

Prognosis in Esophageal Squamous Cell Carcinoma Patients Receiving

Immune Checkpoint Inhibitors

SO ANTICANCER RESEARCH

LA English

DT Article

DE Antibiotic; esophageal squamous cell carcinoma; neutrophil-to-lymphocyte

ratio; prognosis; programmed cell death protein-1

AB Background/Aim: This study explored the prognostic significance of the neutrophil-to-lymphocyte ratio (NLR) and use of antibiotics in advanced esophageal squamous cell carcinoma (ESCC) patients receiving immune checkpoint inhibitors (ICIs). Patients and Methods: Patients were enrolled from two referral centers in Taiwan. Clinical benefit was defined as complete response, partial response, or a stable disease for >= 6 months via Response Evaluation Criteria In Solid Tumors 1.1. Clinicopathological factors' impact on overall survival (OS) and progression-free survival (PFS) was analyzed via Cox proportional hazards model. Results: Forty-nine patients were enrolled. The median PFS and OS were 1.8 and 6.1 months, respectively. The median NLR at baseline was 6.40, and 21 patients received antibiotics. Both high NLR and use of antibiotics were associated with inferior PFS (p=0.028 and p<0.001, respectively) and OS (p<0.001 and p<0.001, respectively) in multivariate analysis. Conclusion: High NLR and use of antibiotics were associated with inferior survival in advanced ESCC patients receiving ICIs.

C1 [Guo, Jhe-Cyuan; Lin, Chia-Chi; Ku, Hung-Yang; Shao, Yu-Yun; Huang, Ta-Chen; Hsu, Chih-Hung] Natl Taiwan Univ Hosp, Dept Oncol, 7 Chungshan S Rd, Taipei 10002, Taiwan.

[Guo, Jhe-Cyuan; Lin, Chia-Chi] Natl Taiwan Univ, Grad Inst Clin Med, Coll Med, Taipei, Taiwan.

[Guo, Jhe-Cyuan; Shao, Yu-Yun; Hsu, Chih-Hung] Natl Taiwan Univ, Dept Med Oncol, Canc Ctr, Taipei, Taiwan.

[Lino, Chen-Yuan; Lien, Ming-Yu] China Med Univ Hosp, Div Hematol & Oncol, Taichung, Taiwan.

[Hsieh, Min-Shu] Natl Taiwan Univ Hosp, Dept Pathol, Taipei, Taiwan.

[Ku, Hung-Yang; Shao, Yu-Yun; Huang, Ta-Chen; Hsu, Chih-Hung] Natl Taiwan Univ, Grad Inst Oncol, Coll Med, 1,Sect 1 Jen Rd, Taipei 10051, Taiwan.

RP Huang, TC (corresponding author), Natl Taiwan Univ Hosp, Dept Oncol, 7 Chungshan S Rd, Taipei 10002, Taiwan.; Huang, TC; Hsu, CH (corresponding author), Natl Taiwan Univ, Grad Inst Oncol, Coll Med, 1,Sect 1 Jen Rd, Taipei 10051, Taiwan.

EM e360215@gmail.com; chihhunghsu@ntu.edu.tw

RI Shao, Yu-Yun/K-6434-2012

OI Shao, Yu-Yun/0000-0001-7334-1912; HSU, CHIH-HUNG/0000-0003-0495-973X;

KUO, HUNG-YANG/0000-0002-0573-2636; Guo, Jhe-Cyuan/0000-0002-6689-9640

FU Ministry of Science and Technology, R.O.C. [MOST 105-2314-B-002-186-MY3,

MOST 106-2314-B-002-135-, MOST 107-2314-B-002-199-, MOST

1082314-B-002-096-, MOST 108-2314-B-002-076-MY3]; Ministry of Health and

Welfare, R.O.C [MOHW 107-TDU-B-211-114017, MOHW108-TDU-B-211-124017]

FX This work was supported in part by the Ministry of Science and

Technology, R.O.C. (MOST 105-2314-B-002-186-MY3, MOST

106-2314-B-002-135-, MOST 107-2314-B-002-199-, MOST 1082314-B-002-096-,

and MOST 108-2314-B-002-076-MY3), and the Ministry of Health and

Welfare, R.O.C (MOHW 107-TDU-B-211-114017 and MOHW108-TDU-B-211-124017).

CR Arnold M, 2015, GUT, V64, P381, DOI 10.1136/gutjnl-2014-308124

Chen WW, 2013, ANTICANCER RES, V33, P4123

Derosa L, 2018, ANN ONCOL, V29, P1437, DOI 10.1093/annonc/mdy103

Diem S, 2017, LUNG CANCER, V111, P176, DOI 10.1016/j.lungcan.2017.07.024

Eisenhauer EA, 2009, EUR J CANCER, V45, P228, DOI 10.1016/j.ejca.2008.10.026

Ferrucci PF, 2016, ANN ONCOL, V27, P732, DOI 10.1093/annonc/mdw016

Guo JC, 2015, J THORAC ONCOL, V10, P1481, DOI 10.1097/JTO.0000000000000651

HERSH EM, 1965, J AMER MED ASSOC, V193, P105, DOI 10.1001/jama.1965.03090020019005

Hiramoto S, 2018, INT J CLIN ONCOL, V23, P466, DOI 10.1007/s10147-018-1239-x

Hooper LV, 2012, SCIENCE, V336, P1268, DOI 10.1126/science.1223490

Huang AC, 2017, NATURE, V545, P60, DOI 10.1038/nature22079

Jeyakumar G, 2017, J IMMUNOTHER CANCER, V5, DOI 10.1186/s40425-017-0287-5

Kaderbhai C, 2017, ANTICANCER RES, V37, P3195, DOI 10.21873/anticanres.11680

Kojima T, 2019, J CLIN ONCOL, V37, DOI 10.1200/JCO.2019.37.4_suppl.2

Lin CS, 2017, J THORAC DIS, V9, P3193, DOI 10.21037/jtd.2017.08.108

Pflug N, 2016, ONCOIMMUNOLOGY, V5, DOI 10.1080/2162402X.2016.1150399

Routy B, 2018, SCIENCE, V359, P91, DOI 10.1126/science.aan3706

Schreiber RD, 2011, SCIENCE, V331, P1565, DOI 10.1126/science.1203486

Sivan A, 2015, SCIENCE, V350, P1084, DOI 10.1126/science.aac4255

Smyth EC, 2017, NAT REV DIS PRIMERS, V3, DOI 10.1038/nrdp.2017.48

Suh KJ, 2018, CANCER IMMUNOL IMMUN, V67, P459, DOI 10.1007/s00262-017-2092-x

Taur Y, 2016, GENOME MED, V8, DOI 10.1186/s13073-016-0306-z

Templeton AJ, 2014, JNCI-J NATL CANCER I, V106, DOI 10.1093/jnci/dju124

Torre LA, 2015, CA-CANCER J CLIN, V65, P87, DOI 10.3322/caac.21262

Vetizou M, 2015, SCIENCE, V350, P1079, DOI 10.1126/science.aad1329

NR 25

TC 16

Z9 17

U1 0

U2 4

PU INT INST ANTICANCER RESEARCH

PI ATHENS

PA EDITORIAL OFFICE 1ST KM KAPANDRITIOU-KALAMOU RD KAPANDRITI, PO BOX 22,

ATHENS 19014, GREECE

SN 0250-7005

EI 1791-7530

J9 ANTICANCER RES

JI Anticancer Res.

PD OCT

PY 2019

VL 39

IS 10

BP 5675

EP 5682

DI 10.21873/anticanres.13765

PG 8

WC Oncology

WE Science Citation Index Expanded (SCI-EXPANDED)

SC Oncology

GA JB4GA

UT WOS:000488512600046

PM 31570466

OA Bronze

DA 2022-05-19

ER

PT J

AU Xu, J

Hu, ZD

AF Xu, Jing

Hu, Zhendong

TI Y-box-binding protein 1 promotes tumor progression and inhibits

cisplatin chemosensitivity in esophageal squamous cell carcinoma

SO BIOMEDICINE & PHARMACOTHERAPY

LA English

DT Article

DE Y-box-binding protein 1; Esophageal squamous cell carcinoma; Drug

resistance; Prognosis; Tumor progression

ID NUCLEAR EXPRESSION; P-GLYCOPROTEIN; BREAST-CANCER; TRANSLATIONAL

ACTIVATION; PROGNOSTIC MARKER; YB-1 PROMOTES; PROLIFERATION; APOPTOSIS;

GROWTH

AB Y-box-binding protein 1 (YB-1) has been identified as a pleotropic oncoprotein in multiple human malignancies and is involved in the regulation of various DNA/RNA-dependent events, including transcription and translation. However, little is known about expression pattern and underlying functions of YB-1 in esophageal squamous cell carcinoma (ESCC). In this study, we report that up-regulated YB-1 is closely correlated with TNM stage and tumor size in ESCC specimens and predicts a poor prognosis in ESCC patients. Genetic silencing of YB-1 inhibits cell proliferation and invasive potential by regulating its targeted genes associated with tumor growth and metastasis. In particular, we also show that silencing of YB-1 enhances chemotherapy-induced cytotoxicity in EC109 and TE-1 cells by targeting multidrug resistance 1 (MDR1). Our findings demonstrate the oncogenic roles of YB-1 in ESCC and support it as a target for ESCC therapy. (C) 2016 Elsevier Masson SAS. All rights reserved.

C1 [Xu, Jing; Hu, Zhendong] Nanjing Med Univ, Affiliated Canc Hosp, Dept Thorac Surg, 42 Baizi Pavil, Nanjing 210000, Jiangsu, Peoples R China.

[Xu, Jing] Nanjing Med Univ, Affiliated Hosp 1, Dept Oncol, Nanjing 210000, Jiangsu, Peoples R China.

RP Hu, ZD (corresponding author), Nanjing Med Univ, Affiliated Canc Hosp, Dept Thorac Surg, 42 Baizi Pavil, Nanjing 210000, Jiangsu, Peoples R China.

EM hzd8124@sina.com

CR Allum WH, 2009, J CLIN ONCOL, V27, P5062, DOI 10.1200/JCO.2009.22.2083

El-Naggar AM, 2015, CANCER CELL, V27, P682, DOI 10.1016/j.ccell.2015.04.003

Enzinger PC, 2003, NEW ENGL J MED, V349, P2241, DOI 10.1056/NEJMra035010

Evdokimova V, 2009, CANCER CELL, V15, P402, DOI 10.1016/j.ccr.2009.03.017

Gimenez-Bonafe P, 2004, PROSTATE, V59, P337, DOI 10.1002/pros.20023

Guo TT, 2015, HISTOPATHOLOGY, V66, P491, DOI 10.1111/his.12570

Ha B, 2015, BIOCHEM BIOPH RES CO, V458, P347, DOI 10.1016/j.bbrc.2015.01.114

Jung K, 2014, BMC CANCER, V14, DOI 10.1186/1471-2407-14-328

Lasham A, 2013, BIOCHEM J, V449, P11, DOI 10.1042/BJ20121323

Lee C, 2008, CANCER RES, V68, P8661, DOI 10.1158/0008-5472.CAN-08-1082

Lovett DH, 2010, BIOCHEM BIOPH RES CO, V398, P482, DOI 10.1016/j.bbrc.2010.06.104

Nishio S, 2014, GYNECOL ONCOL, V132, P703, DOI 10.1016/j.ygyno.2014.01.045

Oda Y, 1998, CLIN CANCER RES, V4, P2273

Oda Y, 2003, J PATHOL, V199, P251, DOI 10.1002/path.1282

Panupinthu N, 2014, ONCOGENE, V33, P2846, DOI 10.1038/onc.2013.259

Schittek B, 2007, INT J CANCER, V120, P2110, DOI 10.1002/ijc.22512

Shibahara K, 2001, CLIN CANCER RES, V7, P3151

Shiota M, 2011, BJU INT, V108, pE142, DOI 10.1111/j.1464-410X.2010.09810.x

Siegel RL, 2015, CA-CANCER J CLIN, V65, P5, DOI [10.3322/caac.21254, 10.1136/bmj.g1502, 10.1001/jamaoto.2014.2530, 10.3322/caac.21208]

Sinnberg T, 2012, EXP DERMATOL, V21, P265, DOI 10.1111/j.1600-0625.2012.01448.x

Thallinger CMR, 2011, J CLIN ONCOL, V29, P4709, DOI 10.1200/JCO.2011.36.7599

Wang X, 2015, INT J CLIN EXP MED, V8, P1780

Wu Y, 2015, CLIN TRANSL ONCOL, V17, P152, DOI 10.1007/s12094-014-1208-4

Yan XB, 2014, INT J CLIN EXP PATHO, V7, P8715

NR 24

TC 7

Z9 8

U1 0

U2 4

PU ELSEVIER FRANCE-EDITIONS SCIENTIFIQUES MEDICALES ELSEVIER

PI ISSY-LES-MOULINEAUX

PA 65 RUE CAMILLE DESMOULINS, CS50083, 92442 ISSY-LES-MOULINEAUX, FRANCE

SN 0753-3322

EI 1950-6007

J9 BIOMED PHARMACOTHER

JI Biomed. Pharmacother.

PD APR

PY 2016

VL 79

BP 17

EP 22

DI 10.1016/j.biopha.2016.01.037

PG 6

WC Medicine, Research & Experimental; Pharmacology & Pharmacy

WE Science Citation Index Expanded (SCI-EXPANDED)

SC Research & Experimental Medicine; Pharmacology & Pharmacy

GA DI5GO

UT WOS:000373527100003

PM 27044807

OA hybrid

DA 2022-05-19

ER

PT J

AU Chu, J

Jia, JL

Yang, LJ

Qu, YH

Yin, HQ

Wan, JH

He, FC

AF Chu, Jie

Jia, Jinlin

Yang, Lijun

Qu, Yunhui

Yin, Huiqing

Wan, Junhu

He, Fucheng

TI LncRNA MIR31HG functions as a ceRNA to regulate c-Met function by

sponging miR-34a in esophageal squamous cell carcinoma

SO BIOMEDICINE & PHARMACOTHERAPY

LA English

DT Article

DE Esophageal squamous cell carcinoma; Long non-coding RNA; MIR31HG;

MiR-34a; c-Met

ID NONCODING RNA MIR31HG; CANCER; METASTASIS; GROWTH

AB Accumulating evidence has demonstrated that long non-coding RNAs (lncRNAs) function as essential regulators in the development and progression of multiple tumors. However, the molecular mechanisms of MIR31HG in regulating ESCC progression remain unknown. Here, we confirmed that MIR31HG facilitated ESCC cells proliferation in vivo. Besides, MIR31HG knockdown increases the percentage of cells at the G1 phase, along with reduced arrest in S phase and MIR31HG overexpression exhibits the opposite effects. Overexpressed MIR31HG decreases the percentage of apoptotic ESCC cells. Interestingly, MIR31HG can function as a competing endogenous RNA by sponging miR-34a. The rescue experiments demonstrated that MIR31HG function is partially reversed by inhibiting miR-34a. In addition, we found c-Met is a target gene of miR-34a and is indirectly regulated by MIR31HG. Taken together, our findings revealed that MIR31HG promotes ESCC progression by regulating miR-34a/ c-Met axis and may provide a new prospective for exploration and understanding of the biological effects of esophageal squamous cell carcinoma.

C1 [Chu, Jie; Jia, Jinlin; Yang, Lijun; Qu, Yunhui; Yin, Huiqing; Wan, Junhu; He, Fucheng] Zhengzhou Univ, Affiliated Hosp 1, Dept Med Lab, Zhengzhou, Henan, Peoples R China.

RP Wan, JH; He, FC (corresponding author), Zhengzhou Univ, Affiliated Hosp 1, Jianshe Dong Rd 1, Zhengzhou, Henan, Peoples R China.

EM 504017950@qq.com; 2632943581@qq.com; 1907529740@qq.com; zdqyh09@126.com;

yinhuiqing12@126.com; wanjh2009@163.com; hefucheng@zzu.edu.cn

OI He, Fucheng/0000-0002-9024-1144

FU Medical Scientific Research Foundation of Health Department of Henan

Province of China [201403077]; National Natural Science Foundation of

China [81702780]; Science and Technology Project of Henan province of

China [202102310112]; Academy of Medical Sciences of Zhengzhou

University Translational Medicine platform

FX The study was supported by grants from Medical Scientific Research

Foundation of Health Department of Henan Province of China (Grant No.

201403077), the National Natural Science Foundation of China grants

(Grant No. 81702780), and the Science and Technology Project of Henan

province of China (Grant No. 192102310133), the Science and Technology

Project of Henan province of China (Grant No. 202102310112). The authors

also thank for the support from Academy of Medical Sciences of Zhengzhou

University Translational Medicine platform.

CR Adenis A, 2019, J CLIN ONCOL, V37, P2379, DOI 10.1200/JCO.19.00963

Arnold M, 2015, GUT, V64, P381, DOI 10.1136/gutjnl-2014-308124

Blokhin I, 2018, EXPERT OPIN DRUG DIS, V13, P837, DOI 10.1080/17460441.2018.1501024

Chu J, 2019, BIOMED PHARMACOTHER, V116, DOI 10.1016/j.biopha.2019.109029

Cotton RGH, 2014, ANN NY ACAD SCI, V1325, P138, DOI 10.1111/nyas.12522

Dong Y, 2019, BIOMED PHARMACOTHER, V109, P2035, DOI 10.1016/j.biopha.2018.08.017

El Bezawy R, 2017, J HEMATOL ONCOL, V10, DOI 10.1186/s13045-016-0387-6

Feng D, 2019, CLIN CHIM ACTA, V495, P326, DOI 10.1016/j.cca.2019.04.060

Haghi M, 2019, J CELL BIOCHEM, V120, P13342, DOI 10.1002/jcb.28608

Jing HY, 2019, BIOMED PHARMACOTHER, V110, P825, DOI 10.1016/j.biopha.2018.12.024

Jing ZF, 2019, MOL ONCOL, V13, P2079, DOI 10.1002/1878-0261.12545

Kapranov P, 2007, SCIENCE, V316, P1484, DOI 10.1126/science.1138341

Khalil AM, 2009, P NATL ACAD SCI USA, V106, P11667, DOI 10.1073/pnas.0904715106

Le Bras GF, 2016, PHARMACOL RES, V113, P236, DOI 10.1016/j.phrs.2016.08.021

Li YS, 2018, NUCLEIC ACIDS RES, V46, P1113, DOI 10.1093/nar/gkx1311

Liang X, 2019, CANCER MANAG RES, V11, P8923, DOI 10.2147/CMAR.S222708

Liu HY, 2019, BIOMED PHARMACOTHER, V119, DOI 10.1016/j.biopha.2019.109396

MA GL, 2019, INT J CANCER, V14, DOI DOI 10.1177/1934578X19857902

Nie FQ, 2016, TUMOR BIOL, V37, P7693, DOI 10.1007/s13277-015-4644-z

Pennathur A, 2013, LANCET, V381, P400, DOI 10.1016/S0140-6736(12)60643-6

Prensner JR, 2011, CANCER DISCOV, V1, P391, DOI 10.1158/2159-8290.CD-11-0209

Quinn JJ, 2016, NAT REV GENET, V17, P47, DOI 10.1038/nrg.2015.10

Shao RL, 2019, EPJ APPL METAMATERIA, V6, DOI 10.1051/epjam/2019009

Shih JW, 2017, NAT COMMUN, V8, DOI 10.1038/ncomms15874

Sun KY, 2018, LIFE SCI, V202, P124, DOI 10.1016/j.lfs.2018.03.050

Sun YJ, 2019, CANCER MANAG RES, V11, P8055, DOI 10.2147/CMAR.S214569

Talebi A, 2020, J CELL PHYSIOL, V235, P848, DOI 10.1002/jcp.29083

Wan JH, 2019, BIOMED PHARMACOTHER, V111, P976, DOI 10.1016/j.biopha.2018.12.148

Wan JH, 2018, CELL DEATH DIS, V9, DOI 10.1038/s41419-018-0556-3

Wang N, 2019, FRONT MICROBIOL, V10, P1

Wang R, 2018, MOL CANCER, V17, DOI 10.1186/s12943-018-0916-8

Wu DD, 2019, GENE, V692, P223, DOI 10.1016/j.gene.2018.12.077

Xuan Y, 2019, CELL DEATH DIS, V10, DOI 10.1038/s41419-019-1940-3

Yan SY, 2018, J EXP CLIN CANC RES, V37, DOI 10.1186/s13046-018-0853-9

Yang H, 2016, ONCOGENE, V35, P3647, DOI 10.1038/onc.2015.430

Yang LJ, 2018, BIOMED PHARMACOTHER, V106, P1570, DOI 10.1016/j.biopha.2018.07.129

NR 36

TC 10

Z9 10

U1 1

U2 4

PU ELSEVIER FRANCE-EDITIONS SCIENTIFIQUES MEDICALES ELSEVIER

PI ISSY-LES-MOULINEAUX

PA 65 RUE CAMILLE DESMOULINS, CS50083, 92442 ISSY-LES-MOULINEAUX, FRANCE

SN 0753-3322

EI 1950-6007

J9 BIOMED PHARMACOTHER

JI Biomed. Pharmacother.

PD AUG

PY 2020

VL 128

AR 110313

DI 10.1016/j.biopha.2020.110313

PG 7

WC Medicine, Research & Experimental; Pharmacology & Pharmacy

WE Science Citation Index Expanded (SCI-EXPANDED)

SC Research & Experimental Medicine; Pharmacology & Pharmacy

GA ND2MV

UT WOS:000561740200004

PM 32502839

OA gold

DA 2022-05-19

ER

PT J

AU Liu, ZY

Zhang, Y

Chen, YL

Lin, YB

Lin, Z

Wang, H

AF Liu, Zhaoyong

Zhang, Ying

Chen, Yelong

Lin, Youbin

Lin, Zhen

Wang, Hu

TI STAT1 inhibits STAT3 activation in esophageal squamous cell carcinoma

SO CANCER MANAGEMENT AND RESEARCH

LA English

DT Article

DE STAT1; STAT3; esophageal squamous cell carcinoma; transcription activity

ID CANCER; EXPRESSION

AB Background: Signal transducer and activator of transcription (STAT) 1 is an important transcription factor and has been reported to be a tumor suppressor in many types of cancer. However, another STAT family member, STAT3, is considered to be an oncogene. The cross-talk between STAT1 and STAT3 in cancer has not been fully demonstrated.

Materials and methods: Esophageal squamous cell carcinoma (ESCC) was used as a model to examine STAT1-STAT3 cross-regulation in cancer. We detected STAT1-STAT3 binding by co-immunoprecipitation (co-IP) and measured the transcription activity by using a luciferase reporter gene. DNA binding was detected by a DNA probe. Expression of STAT1 and STAT3 in ESCC was detected by immunohistochemistry.

Results: We found that STAT1 attenuated STAT3 activity upon oncostatin M treatment by decreasing STAT3 transcription activity and DNA binding ability of STAT3. Furthermore STAT3 downregulation increased the phosphorylation and transcriptional activation of STAT1. Finally, STAT1 expression and STAT3 expression were negatively correlated in ESCC cases.

Conclusion: Altogether, this paper demonstrated STAT I and STAT3 cross-regulation in ESCC and proposed that STAT3 downregulation and/or STAT1 accumulation may be a therapeutic approach to treat ESCC.

C1 [Liu, Zhaoyong; Chen, Yelong; Lin, Youbin; Wang, Hu] Shantou Univ, Affiliated Hosp 1, Dept Orthopaed, Med Coll, 57 Changping Rd, Shantou 515041, Guangdong, Peoples R China.

[Zhang, Ying; Lin, Zhen] Shantou Univ, Dept Pathol, Med Coll, 22 Xinling Rd, Shantou, Guangdong, Peoples R China.

RP Wang, H (corresponding author), Shantou Univ, Affiliated Hosp 1, Dept Orthopaed, Med Coll, 57 Changping Rd, Shantou 515041, Guangdong, Peoples R China.; Zhang, Y (corresponding author), Shantou Univ, Dept Pathol, Med Coll, 22 Xinling Rd, Shantou, Guangdong, Peoples R China.

EM yzhangmimazi@stu.edu.cn; wanghu0754@163.com

FU Natural Science Foundation of Guangdong [2018A030310219]

FX This work was supported by Natural Science Foundation of Guangdong

(2018A030310219).

CR Chai EZP, 2016, PHARMACOL THERAPEUT, V162, P86, DOI 10.1016/j.pharmthera.2015.10.004

Costa-Pereira AP, 2002, P NATL ACAD SCI USA, V99, P8043, DOI 10.1073/pnas.122236099

Dimberg LY, 2012, BMC CANCER, V12, DOI 10.1186/1471-2407-12-318

Ehret GB, 2001, J BIOL CHEM, V276, P6675, DOI 10.1074/jbc.M001748200

Friedrich K, 2017, BIOSCI TRENDS, V11, P1, DOI 10.5582/bst.2016.01250

Gujam FJA, 2016, ONCOTARGET, V7, P77607, DOI 10.18632/oncotarget.12730

Han W, 2013, MOL CARCINOGEN, V52, P959, DOI 10.1002/mc.21936

Kim HS, 2007, CELL SIGNAL, V19, P454, DOI 10.1016/j.cellsig.2006.09.003

Meissl K, 2017, CYTOKINE, V89, P12, DOI 10.1016/j.cyto.2015.11.011

Nivarthi H, 2016, ONCOTARGET, V7, P51096, DOI 10.18632/oncotarget.9315

Ramana CV, 2002, TRENDS IMMUNOL, V23, P96, DOI 10.1016/S1471-4906(01)02118-4

Su M, 2007, EUR J EPIDEMIOL, V22, P43, DOI 10.1007/s10654-006-9086-x

Yu H, 2014, NAT REV CANCER, V14, P736, DOI 10.1038/nrc3818

Zhang Y, 2017, J EXP MED, V1, P1, DOI DOI 10.3727/096504017X14957939026111

Zhang Y, 2017, DISCOV MED, V24, P19

Zhang Y, 2016, ONCOTARGET, V7, P54973, DOI 10.18632/oncotarget.10362

Zhang Y, 2014, BMC CANCER, V14, DOI 10.1186/1471-2407-14-791

NR 17

TC 5

Z9 5

U1 2

U2 3

PU DOVE MEDICAL PRESS LTD

PI ALBANY

PA PO BOX 300-008, ALBANY, AUCKLAND 0752, NEW ZEALAND

SN 1179-1322

J9 CANCER MANAG RES

JI Cancer Manag. Res.

PY 2018

VL 10

BP 6517

EP 6523

DI 10.2147/CMAR.S182105

PG 7

WC Oncology

WE Science Citation Index Expanded (SCI-EXPANDED)

SC Oncology

GA HC4VW

UT WOS:000451803900001

PM 30555257

OA Green Submitted, gold, Green Published

DA 2022-05-19

ER

PT J

AU Liu, XP

Yan, YL

Ma, WP

Wu, SG

AF Liu, Xiaoping

Yan, Yanli

Ma, Weiping

Wu, Suge

TI Knockdown of frizzled-7 inhibits cell growth and metastasis and promotes

chemosensitivity of esophageal squamous cell carcinoma cells by

inhibiting Wnt signaling

SO BIOCHEMICAL AND BIOPHYSICAL RESEARCH COMMUNICATIONS

LA English

DT Article

DE Esophageal squamous cell carcinoma; FZD7; Drug resistance; Wnt

ID MESENCHYMAL TRANSITION; CATENIN PATHWAY; DOWN-REGULATION; EXPRESSION;

INVASION; PROLIFERATION; FZD7; OVEREXPRESSION; TARGET; PROGNOSIS

AB A growing body of evidence suggests that frizzled-7 (FZD7) is an important oncogene in multiple human malignancies and is involved in regulating sensitivity to chemotherapeutic drugs. However, little is known about the expression and function of FZD7 in esophageal squamous cell carcinoma (ESCC). In this study, we aimed to investigate the functional significance of FZD7 in ESCC. Here, we found that both mRNA and protein expression levels of FZD7 were highly upregulated in ESCC cell lines. The knockdown of FZD7 inhibited cell growth, induced cell apoptosis, and suppressed cell migration and invasion of ESCC cells in vitro. Furthermore, knockdown of FZD7 promoted chemosensitivity to cisplatin in ESCC cells and suppressed the expression of multidrug resistance protein 1. Moreover, the silencing of FZD7 also significantly impeded the activation of Wnt signaling in ESCC cells. Taken together, our study reveals an oncogenic role of FZD7 in the progression of ESCC and suggests that FZD7 might serve as a potential therapeutic target for treatment of ESCC. (C) 2017 Elsevier Inc. All rights reserved.

C1 [Liu, Xiaoping; Yan, Yanli; Ma, Weiping; Wu, Suge] Xi An Jiao Tong Univ, Affiliated Hosp 1, Canc Ctr, 277 Yanta West Rd, Xian 710061, Shaanxi, Peoples R China.

RP Liu, XP (corresponding author), Xi An Jiao Tong Univ, Affiliated Hosp 1, Canc Ctr, 277 Yanta West Rd, Xian 710061, Shaanxi, Peoples R China.

EM liu_xiaopingxp@163.com

CR Ai RN, 2016, CANCER BIOL THER, V17, P943, DOI 10.1080/15384047.2016.1210734

Anastas JN, 2014, J CLIN INVEST, V124, P2877, DOI 10.1172/JCI70156

Chen ZL, 2013, CELL SIGNAL, V25, P2693, DOI 10.1016/j.cellsig.2013.08.032

Deng BY, 2015, TUMOR BIOL, V36, P8065, DOI 10.1007/s13277-015-3483-2

Deng BY, 2015, MED ONCOL, V32, DOI 10.1007/s12032-015-0552-8

Fu HL, 2013, CELL BIOCHEM BIOPHYS, V67, P657, DOI 10.1007/s12013-013-9554-3

Kim M, 2008, J HEPATOL, V48, P780, DOI 10.1016/j.jhep.2007.12.020

King TD, 2012, CELL SIGNAL, V24, P846, DOI 10.1016/j.cellsig.2011.12.009

Kirikoshi H, 2001, INT J ONCOL, V19, P111

Li XX, 2014, INT J CLIN EXP PATHO, V7, P2729

Liu N, 2016, ONCOTARGET, V7, P6175, DOI 10.18632/oncotarget.6742

Mealey KL, 2015, J VET INTERN MED, V29, P1, DOI [10.1111/jvim.12482, 10.1111/jvim.12525]

Merle P, 2005, J HEPATOL, V43, P854, DOI 10.1016/j.jhep.2005.05.018

Merle P, 2004, GASTROENTEROLOGY, V127, P1110, DOI 10.1053/j.gastro.2004.07.009

Miyata H, 2009, CANCER, V115, P3324, DOI 10.1002/cncr.24390

Moghbeli M, 2016, J CELL COMMUN SIGNAL, V10, P129, DOI 10.1007/s12079-016-0320-3

Phesse T, 2016, CANCERS, V8, DOI 10.3390/cancers8050050

Qiu X, 2016, ONCOTARGET, V7, P85987, DOI 10.18632/oncotarget.13292

Ren W, 2016, BIOCHEM BIOPH RES CO, V469, P633, DOI 10.1016/j.bbrc.2015.12.054

Sagara N, 1998, BIOCHEM BIOPH RES CO, V252, P117, DOI 10.1006/bbrc.1998.9607

Sanford NN, 2017, DIS ESOPHAGUS, V30, DOI 10.1093/dote/dox025

Siegel RL, 2016, CA-CANCER J CLIN, V66, P7, DOI 10.3322/caac.21332

Su HF, 2015, J TRANSL MED, V13, DOI 10.1186/s12967-015-0464-6

Takahashi H, 2010, J THORAC ONCOL, V5, P122, DOI 10.1097/JTO.0b013e3181c1ffd5

Ueno K, 2009, BRIT J CANCER, V101, P1374, DOI 10.1038/sj.bjc.6605307

Ueno K, 2008, NEOPLASIA, V10, P697, DOI 10.1593/neo.08320

van Hagen P, 2012, NEW ENGL J MED, V366, P2074, DOI 10.1056/NEJMoa1112088

Wang P, 2016, J CANCER RES THER, V12, P19, DOI 10.4103/0973-1482.191622

Wei W, 2011, MOL CANCER, V10, DOI 10.1186/1476-4598-10-16

Wu WH, 2017, BIOCHEM BIOPH RES CO, V482, P100, DOI 10.1016/j.bbrc.2016.10.136

Xu HP, 2015, ONCOTARGET, V6, P39908, DOI 10.18632/oncotarget.5516

Yang L, 2011, ONCOGENE, V30, P4437, DOI 10.1038/onc.2011.145

Zhang JX, 2013, INT J CANCER, V132, P2567, DOI 10.1002/ijc.27930

NR 33

TC 16

Z9 16

U1 0

U2 3

PU ACADEMIC PRESS INC ELSEVIER SCIENCE

PI SAN DIEGO

PA 525 B ST, STE 1900, SAN DIEGO, CA 92101-4495 USA

SN 0006-291X

EI 1090-2104

J9 BIOCHEM BIOPH RES CO

JI Biochem. Biophys. Res. Commun.

PD AUG 26

PY 2017

VL 490

IS 3

BP 1112

EP 1118

DI 10.1016/j.bbrc.2017.06.185

PG 7

WC Biochemistry & Molecular Biology; Biophysics

WE Science Citation Index Expanded (SCI-EXPANDED)

SC Biochemistry & Molecular Biology; Biophysics

GA FC4OQ

UT WOS:000406820600079

PM 28669726

DA 2022-05-19

ER

PT J

AU Chen, HI

Tsai, HP

Chen, YT

Tsao, SC

Chai, CY

AF Chen, Hsin-I

Tsai, Hung-Pei

Chen, Yi-Ting

Tsao, Shu-Chuan

Chai, Chee-Yin

TI Autophagy and Apoptosis Play Opposing Roles in Overall Survival of

Esophageal Squamous Cell Carcinoma

SO PATHOLOGY & ONCOLOGY RESEARCH

LA English

DT Article

DE Autophagy; Apoptosis; Esophageal squamous cell carcinoma

ID MEDIATED CLEAVAGE; EXPRESSION; CANCER; CASPASE-3; SERVES

AB Esophageal cancer is among the most aggressive gastrointestinal tract malignancies, and squamous cell carcinoma is the most common subtype. Although both autophagy and apoptosis involve programmed cell death, autophagy also maintains cell survival by recycling cellular waste. The relationship between autophagy and apoptosis in esophageal squamous cell carcinoma (ESCC) is unclear. Autophagic and apoptotic markers of ESCC were detected by immunohistochemical staining (IHC) in 43 ESCC patients treated during 2007-2011. Chi-square test and Kaplan-Meier method were used to determine how clinicopathological parameters were related to IHC results for LC3B, Beclin-1 and caspase-3 (CASP-3). Correlations among Beclin-1, LC3B, and CASP-3 were analyzed by Spearman rho. The statistical analyses revealed no clinicopathological parameters that significantly correlated with expressions of Beclin-1, LC3B, and CASP-3. However, low CASP-3 expression and high LC3B expression revealed by IHC were predictors of a poor prognosis. Additionally, LC3B expression had a significant negative correlation with CASP-3 expression. Autophagy is antagonistic to apoptosis and predicts poor overall survival in ESCC.

C1 [Chen, Hsin-I; Chen, Yi-Ting; Tsao, Shu-Chuan; Chai, Chee-Yin] Kaohsiung Med Univ, Kaohsiung Med Univ Hosp, Dept Pathol, 100 Tzyou 1st Rd, Kaohsiung 807, Taiwan.

[Tsai, Hung-Pei; Chai, Chee-Yin] Kaohsiung Med Univ, Grad Inst Med, Coll Med, Kaohsiung, Taiwan.

[Chai, Chee-Yin] Kaohsiung Med Univ, Dept Pathol, Coll Med, Fac Med, Kaohsiung, Taiwan.

[Chai, Chee-Yin] Natl Sun Yat Sen Univ, Inst Biomed Sci, Kaohsiung, Taiwan.

RP Chai, CY (corresponding author), Kaohsiung Med Univ, Kaohsiung Med Univ Hosp, Dept Pathol, 100 Tzyou 1st Rd, Kaohsiung 807, Taiwan.; Chai, CY (corresponding author), Kaohsiung Med Univ, Grad Inst Med, Coll Med, Kaohsiung, Taiwan.; Chai, CY (corresponding author), Kaohsiung Med Univ, Dept Pathol, Coll Med, Fac Med, Kaohsiung, Taiwan.; Chai, CY (corresponding author), Natl Sun Yat Sen Univ, Inst Biomed Sci, Kaohsiung, Taiwan.

EM cychai@kmu.edu.tw

OI Chen, yiting/0000-0002-0817-1539; Chai, Chee-Yin/0000-0003-0486-9742

CR Apel A, 2008, CANCER RES, V68, P1485, DOI 10.1158/0008-5472.CAN-07-0562

Bosman FT, 2010, WHO TUMORS DIGESTIVE

Chen S, 2013, CLIN CANCER RES, V19, P6853, DOI 10.1158/1078-0432.CCR-13-1617

Chen YS, 2013, PATHOL RES PRACT, V209, P562, DOI 10.1016/j.prp.2013.06.006

Choi KS, 2012, EXP MOL MED, V44, P109, DOI 10.3858/emm.2012.44.2.033

Del Bello B, 2013, PLOS ONE, V8, DOI 10.1371/journal.pone.0057236

Hanna RA, 2012, J BIOL CHEM, V287, P19094, DOI 10.1074/jbc.M111.322933

Hu YL, 2012, CANCER RES, V72, P4294, DOI 10.1158/0008-5472.CAN-12-1076

Huang H, 2010, CANCER SCI, V101, P1314, DOI 10.1111/j.1349-7006.2010.01524.x

Huang X, 2010, J CLIN NEUROSCI, V17, P1515, DOI 10.1016/j.jocn.2010.03.051

INA H, 1994, CANCER-AM CANCER SOC, V73, P2038, DOI 10.1002/1097-0142(19940415)73:8<2038::AID-CNCR2820730804>3.0.CO;2-X

Kung CP, 2011, CRIT REV EUKAR GENE, V21, P71, DOI 10.1615/CritRevEukarGeneExpr.v21.i1.50

Lai SW, 2012, LIBYAN J MED, V7

Li J, 2010, EUR J CANCER, V46, P1900, DOI 10.1016/j.ejca.2010.02.021

Li WW, 2012, CELL MOL LIFE SCI, V69, P1125, DOI 10.1007/s00018-011-0865-5

Liang XH, 2001, CANCER RES, V61, P3443

Liu DL, 2011, MED ONCOL, V28, P105, DOI 10.1007/s12032-009-9397-3

Liu EY, 2012, J CELL SCI, V125, P2349, DOI 10.1242/jcs.093708

Melguizo C, 2012, J TRANSL MED, V10, DOI 10.1186/1479-5876-10-250

Ni Chonghaile T, 2011, MOL CELL, V44, P844, DOI 10.1016/j.molcel.2011.12.007

Shrivastava S, 2012, J VIROL, V86, P8705, DOI 10.1128/JVI.00616-12

Su MF, 2013, J ONCOL, V2013, DOI 10.1155/2013/102735

Wirawan E, 2010, CELL DEATH DIS, V1, DOI 10.1038/cddis.2009.16

Yorimitsu T, 2005, CELL DEATH DIFFER, V12, P1542, DOI 10.1038/sj.cdd.4401765

Young MM, 2012, J BIOL CHEM, V287, P12455, DOI 10.1074/jbc.M111.309104

Yousefi S, 2006, NAT CELL BIOL, V8, P1124, DOI 10.1038/ncb1482

Zheng HC, 2003, WORLD J GASTROENTERO, V9, P1415, DOI 10.3748/wjg.v9.i7.1415

NR 27

TC 11

Z9 11

U1 0

U2 6

PU SPRINGER

PI DORDRECHT

PA VAN GODEWIJCKSTRAAT 30, 3311 GZ DORDRECHT, NETHERLANDS

SN 1219-4956

EI 1532-2807

J9 PATHOL ONCOL RES

JI Pathol. Oncol. Res.

PD OCT

PY 2016

VL 22

IS 4

BP 699

EP 705

DI 10.1007/s12253-016-0051-z

PG 7

WC Oncology; Pathology

WE Science Citation Index Expanded (SCI-EXPANDED)

SC Oncology; Pathology

GA DX7OQ

UT WOS:000384578200007

PM 26980476

DA 2022-05-19

ER

PT J

AU Koh, HK

Park, Y

Koo, T

Park, HJ

Lee, MY

Chang, AR

Hong, S

Bae, H

AF Koh, Hyeon Kang

Park, Younghee

Koo, Taeryool

Park, Hae Jin

Lee, Me Yeon

Chang, Ah Ram

Hong, Semie

Bae, Hoonsik

TI Neutrophil-to-Lymphocyte Ratio After Definitive Concurrent

Chemoradiotherapy Predicts Survival in Patients With Esophageal Squamous

Cell Carcinoma

SO IN VIVO

LA English

DT Article

DE Chemoradiotherapy; esophageal squamous cell carcinoma;

neutrophil-to-lymphocyte ratio; inflammation

ID TUMOR MICROENVIRONMENT; RADIATION-THERAPY; PROGNOSTIC-FACTOR; CANCER;

INFLAMMATION; TRIAL

AB Background/Aim: Lymphocyte-to-monocyte ratio, neutrophil-to-lymphocyte ratio, and platelet-to-lymphocyte ratio represent systemic immune-inflammatory responses. We evaluated the association between immune-inflammatory cell ratios and prognosis in esophageal squamous cell carcinoma (ESCC) patients who underwent definitive concurrent chemoradiotherapy (dCCRT). Patients and Methods: Medical records of 68 ESCC patients in three institutions who underwent dCCRT between 2006 and 2017 were reviewed. The immune-inflammatory cell ratios were calculated before and after dCCRT. Results: The median follow-up time was 11.4 months. The 3-year overall survival (OS) rate was 21.6%. Among the immune-inflammatory cell ratios, lower post-dCCRT neutrophil-to-lymphocyte ratio (NLRpost) was associated with better OS (median 15.2 vs. 9.7 months, p=0.030). Patients with lower NLRpost had more improved OS when adjuvant chemotherapy was administered following dCCRT (median 16.6 vs. 4.8 months, p<0.001). Conclusion: NLRpost may be useful in predicting OS in ESCC patients after dCCRT. Furthermore, NLRpost might play a role in establishing adjuvant therapy plans following dCCRT.

C1 [Koh, Hyeon Kang; Hong, Semie] Konkuk Univ, Dept Radiat Oncol, Sch Med, Seoul, South Korea.

[Koh, Hyeon Kang; Hong, Semie] Konkuk Univ, Med Ctr, Seoul, South Korea.

[Park, Younghee; Chang, Ah Ram] Soonchunhyang Univ, Dept Radiat Oncol CyberKnife Ctr, Seoul Hosp, Seoul, South Korea.

[Koo, Taeryool; Lee, Me Yeon; Bae, Hoonsik] Hallym Univ, Sacred Heart Hosp, Dept Radiat Oncol, Coll Med, Anyang, South Korea.

[Park, Hae Jin] Hanyang Univ, Dept Radiat Oncol, Coll Med, Seoul, South Korea.

RP Koo, T (corresponding author), Hallym Univ, Sacred Heart Hosp, Dept Radiat Oncol, Coll Med, 22 Gwanpyeong Ro 170Beon-gil, Anyang Si 14068, Gyeonggi Do, South Korea.

EM kootaeryool@hallym.or.kr

OI Koh, Hyeon Kang/0000-0002-6855-1832

CR Cannon NA, 2015, J THORAC ONCOL, V10, P280, DOI 10.1097/JTO.0000000000000399

Coussens LM, 2013, SCIENCE, V339, P286, DOI 10.1126/science.1232227

Crawford J, 2004, CANCER-AM CANCER SOC, V100, P228, DOI 10.1002/cncr.11882

Fan CY, 2018, SCI REP-UK, V8, DOI 10.1038/s41598-018-31302-y

Galdiero MR, 2013, IMMUNOBIOLOGY, V218, P1402, DOI 10.1016/j.imbio.2013.06.003

Gooden MJM, 2011, BRIT J CANCER, V105, P93, DOI 10.1038/bjc.2011.189

Gregory AD, 2011, CANCER RES, V71, P2411, DOI 10.1158/0008-5472.CAN-10-2583

Grivennikov SI, 2010, CELL, V140, P883, DOI 10.1016/j.cell.2010.01.025

Hu PP, 2014, PLOS ONE, V9, DOI 10.1371/journal.pone.0108062

Kato K, 2011, INT J RADIAT ONCOL, V81, P684, DOI 10.1016/j.ijrobp.2010.06.033

Koh HK, 2020, ANTICANCER RES, V40, P1771, DOI 10.21873/anticanres.14131

Lee HW, 2013, BBA-REV CANCER, V1835, P170, DOI 10.1016/j.bbcan.2012.12.007

Mantovani A, 2008, NATURE, V454, P436, DOI 10.1038/nature07205

Martinez-Lostao L, 2015, CLIN CANCER RES, V21, P5047, DOI 10.1158/1078-0432.CCR-15-0685

Mason M, 2017, J NEURO-ONCOL, V132, P463, DOI 10.1007/s11060-017-2395-y

Minsky BD, 2002, J CLIN ONCOL, V20, P1167, DOI 10.1200/JCO.20.5.1167

Ou D, 2020, STRAHLENTHER ONKOL, V196, P252, DOI 10.1007/s00066-019-01529-3

Pitt JM, 2016, ANN ONCOL, V27, P1482, DOI 10.1093/annonc/mdw168

Ray-Coquard I, 2009, CANCER RES, V69, P5383, DOI 10.1158/0008-5472.CAN-08-3845

Sherry AD, 2020, INT J RADIAT ONCOL, V108, P268, DOI 10.1016/j.ijrobp.2019.11.398

Sung S, 2017, PLOS ONE, V12, DOI 10.1371/journal.pone.0173955

Team R.C, 2013, C R LANGUAGE ENV STA

Van Cutsem Eric, 2005, Eur J Oncol Nurs, V9 Suppl 2, pS51, DOI 10.1016/j.ejon.2005.09.007

Walsh Sr, 2005, J SURG ONCOL, V91, P181, DOI 10.1002/jso.20329

Wang DY, 2020, FUTURE ONCOL, V16, P439, DOI 10.2217/fon-2019-0837

Wu SX, 2017, SCI REP-UK, V7, DOI 10.1038/s41598-017-17254-9

Yan MJ, 2016, BBA-MOL CELL RES, V1863, P392, DOI 10.1016/j.bbamcr.2015.07.008

Yodying H, 2016, ANN SURG ONCOL, V23, P646, DOI 10.1245/s10434-015-4869-5

Zachariah B, 2001, INT J RADIAT ONCOL, V50, P465, DOI 10.1016/S0360-3016(00)01587-X

NR 29

TC 1

Z9 2

U1 0

U2 0

PU INT INST ANTICANCER RESEARCH

PI ATHENS

PA EDITORIAL OFFICE 1ST KM KAPANDRITIOU-KALAMOU RD KAPANDRITI, PO BOX 22,

ATHENS 19014, GREECE

SN 0258-851X

EI 1791-7549

J9 IN VIVO

JI In Vivo

PD MAR-APR

PY 2021

VL 35

IS 2

BP 1133

EP 1139

DI 10.21873/invivo.12359

PG 7

WC Medicine, Research & Experimental

WE Science Citation Index Expanded (SCI-EXPANDED)

SC Research & Experimental Medicine

GA RZ9GH

UT WOS:000648906100025

PM 33622911

OA Green Published, gold

DA 2022-05-19

ER

PT J

AU Qi, B

Wang, Y

Chen, ZJ

Li, XN

Qi, Y

Yang, Y

Cui, GH

Guo, HZ

Li, WH

Zhao, S

AF Qi, Bo

Wang, Yan

Chen, Zhi-Jun

Li, Xiang-Nan

Qi, Yu

Yang, Yang

Cui, Guang-Hui

Guo, Hai-Zhou

Li, Wei-Hao

Zhao, Song

TI Down-regulation of miR-30a-3p/5p promotes esophageal squamous cell

carcinoma cell proliferation by activating the Wnt signaling pathway

SO WORLD JOURNAL OF GASTROENTEROLOGY

LA English

DT Article

DE miR-30a-3p/5p; Proliferation; Esophageal squamous cell carcinoma; Wnt

signaling pathway; Wnt2; Fzd2

ID WNT/BETA-CATENIN PATHWAY; LONG-TERM SURVIVAL; QUALITY-OF-LIFE;

MESENCHYMAL TRANSITION; COLORECTAL-CANCER; TUMOR-SUPPRESSOR; MICRORNAS;

EXPRESSION; INVASION; INVASIVENESS

AB AIM

To investigate the potential role of microRNA-30a (miR30a) in esophageal squamous cell carcinoma (ESCC).

METHODS

Expression of miR-30a-3p/5p was analyzed using microarray data and fresh ESCC tissue samples. Both in vitro and in vivo assays were used to investigate the effects of miR-30a-3p/5p on ESCC cell proliferation. Furthermore, Kyoto Encyclopedia of Genes and Genomes analysis was performed to explore underlying mechanisms involved in ESCC, and then, assays were carried out to verify the potential molecular mechanism of miR-30a in ESCC.

RESULTS

Low expression of miR-30a-3p/5p was closely associated with advanced ESCC progression and poor prognosis of patients with ESCC. Knock-down of miR-30a-3p/5p promoted ESCC cell proliferation. Increased miR-30a3p/ 5p expression inhibited the Wnt signaling pathway by targeting Wnt2 and Fzd2.

CONCLUSION

Down-regulation of miR-30a-3p/5p promotes ESCC cell proliferation by activating the Wnt signaling pathway through inhibition of Wnt2 and Fzd2.

C1 [Qi, Bo; Li, Xiang-Nan; Qi, Yu; Yang, Yang; Cui, Guang-Hui; Guo, Hai-Zhou; Li, Wei-Hao; Zhao, Song] Zhengzhou Univ, Affiliated Hosp 1, Dept Thorac Surg, Zhengzhou 450052, Henan, Peoples R China.

[Qi, Bo; Chen, Zhi-Jun] Xinxiang Med Univ, Affiliated Hosp 1, Dept Thorac Surg, Weihui 453100, Henan, Peoples R China.

[Wang, Yan] Xinxiang Med Univ, Period Publishing House, Xinxiang 453003, Henan, Peoples R China.

RP Zhao, S (corresponding author), Zhengzhou Univ, Affiliated Hosp 1, Dept Thorac Surg, 1 Jianshe East Rd, Zhengzhou 450000, Henan, Peoples R China.

EM zhaosong@zzu.edu.cn

FU Youth Fund of the First Affiliated Hospital of Xinxiang Medical

University [Type A-4]

FX Supported by the Youth Fund of the First Affiliated Hospital of Xinxiang

Medical University (Type A-4).

CR Ai RN, 2016, CANCER BIOL THER, V17, P943, DOI 10.1080/15384047.2016.1210734

Baffa R, 2009, J PATHOL, V219, P214, DOI 10.1002/path.2586

Bartel DP, 2009, CELL, V136, P215, DOI 10.1016/j.cell.2009.01.002

Bockhorn J, 2013, BREAST CANCER RES TR, V137, P373, DOI 10.1007/s10549-012-2346-4

Chen WQ, 2016, CA-CANCER J CLIN, V66, P115, DOI 10.3322/caac.21338

Cheng CW, 2012, BREAST CANCER RES TR, V134, P1081, DOI 10.1007/s10549-012-2034-4

Deng FY, 2015, INT J CLIN EXP PATHO, V8, P3045

Gao YB, 2014, NAT GENET, V46, P1097, DOI 10.1038/ng.3076

He J, 2016, J EXP CLIN CANC RES, V35, DOI 10.1186/s13046-016-0378-z

He L, 2004, NAT REV GENET, V5, P522, DOI 10.1038/nrg1379

Headrick JR, 2002, ANN THORAC SURG, V73, P1697, DOI 10.1016/S0003-4975(02)03496-3

Heinzelmann J, 2011, WORLD J UROL, V29, P367, DOI 10.1007/s00345-010-0633-4

Ichimi T, 2009, INT J CANCER, V125, P345, DOI 10.1002/ijc.24390

Igci YZ, 2015, GENET TEST MOL BIOMA, V19, P418, DOI 10.1089/gtmb.2015.0062

Jia ZF, 2013, PLOS ONE, V8, DOI 10.1371/journal.pone.0055008

Kano M, 2010, INT J CANCER, V127, P2804, DOI 10.1002/ijc.25284

Kim NH, 2013, CELL CYCLE, V12, P1578, DOI 10.4161/cc.24739

Kumarswamy R, 2012, INT J CANCER, V130, P2044, DOI 10.1002/ijc.26218

Liao WT, 2014, J PATHOL, V232, P415, DOI 10.1002/path.4309

Liu Z, 2014, MATH PROBL ENG, V2014, P10, DOI DOI 10.1155/2014/829597

Liz J, 2016, BBA-GENE REGUL MECH, V1859, P169, DOI 10.1016/j.bbagrm.2015.06.015

Luketich JD, 2002, ANN THORAC SURG, V73, P1702

Quintavalle C, 2013, ONCOGENE, V32, P4001, DOI 10.1038/onc.2012.410

Rustgi AK, 2014, NEW ENGL J MED, V371, P2499, DOI 10.1056/NEJMra1314530

Saad MA, 2015, MOL CANCER, V14, DOI 10.1186/s12943-015-0452-8

Sand M, 2012, J DERMATOL SCI, V68, P119, DOI 10.1016/j.jdermsci.2012.09.004

Saydam O, 2009, MOL CELL BIOL, V29, P5923, DOI 10.1128/MCB.00332-09

Schepeler Troels, 2013, Critical Reviews in Oncogenesis, V18, P357

Siegel RL, 2016, CA-CANCER J CLIN, V66, P7, DOI 10.3322/caac.21332

Tan XG, 2011, CLIN CANCER RES, V17, P6802, DOI 10.1158/1078-0432.CCR-11-0419

Tang J, 2012, INT J MOL SCI, V13, P13414, DOI 10.3390/ijms131013414

Teo JL, 2010, ADV DRUG DELIVER REV, V62, P1149, DOI 10.1016/j.addr.2010.09.012

Tsukasa K, 2016, HUM CELL, V29, P130, DOI 10.1007/s13577-016-0137-7

Wang HY, 2014, EXP BIOL MED, V239, P891, DOI 10.1177/1535370214532758

Wang JJ, 2017, BIOSCI BIOTECH BIOCH, V81, P119, DOI 10.1080/09168451.2016.1238294

Wang K, 2013, PATHOL ONCOL RES, V19, P405, DOI 10.1007/s12253-012-9593-x

Wang P, 2016, J CANCER RES THER, V12, P19, DOI 10.4103/0973-1482.191622

Wang W, 2014, EJSO-EUR J SURG ONC, V40, P1586, DOI 10.1016/j.ejso.2013.11.008

Wang XX, 2016, KSII T INTERNET INF, V10, P1016, DOI 10.3837/tiis.2016.03.004

Wang X, 2013, CANCER LETT, V331, P211, DOI 10.1016/j.canlet.2013.01.005

Wang ZY, 2015, BIOCHEM BIOPH RES CO, V465, P374, DOI 10.1016/j.bbrc.2015.08.007

Xie KP, 2016, ONCOTARGET, V7, P47966, DOI 10.18632/oncotarget.10299

Yang CS, 2016, GASTROINTEST TUMORS, V3, P3, DOI 10.1159/000443155

Yang Y, 2010, CANCER GENET CYTOGEN, V200, P127, DOI 10.1016/j.cancergencyto.2010.03.014

Zeng HM, 2015, INT J CANCER, V136, P1921, DOI 10.1002/ijc.29227

Zhang Q, 2015, PLOS ONE, V10, DOI 10.1371/journal.pone.0120698

Zhao JJ, 2014, CANCER RES, V74, P1801, DOI 10.1158/0008-5472.CAN-13-3311-T

Zhong K, 2014, BMC CANCER, V14, DOI 10.1186/1471-2407-14-703

Zhong M, 2013, CELL PHYSIOL BIOCHEM, V31, P209, DOI 10.1159/000343362

Zhou J, 2015, ONCOL REP, V33, P2915, DOI 10.3892/or.2015.3937

NR 50

TC 36

Z9 37

U1 0

U2 9

PU BAISHIDENG PUBLISHING GROUP INC

PI PLEASANTON

PA 7041 Koll Center Parkway, Suite 160, PLEASANTON, CA, UNITED STATES

SN 1007-9327

EI 2219-2840

J9 WORLD J GASTROENTERO

JI World J. Gastroenterol.

PD DEC 7

PY 2017

VL 23

IS 45

BP 7965

EP 7977

DI 10.3748/wjg.v23.i45.7965

PG 13

WC Gastroenterology & Hepatology

WE Science Citation Index Expanded (SCI-EXPANDED)

SC Gastroenterology & Hepatology

GA FO5UW

UT WOS:000416927800003

PM 29259372

OA hybrid, Green Published, Green Submitted

DA 2022-05-19

ER

PT J

AU Guo, ZJ

Yang, H

Zhang, FB

Zhang, RX

Wang, CJ

AF Guo, Zhanjun

Yang, Hua

Zhang, Fengbin

Zhang, Ruixing

Wang, Cuiju

TI Single nucleotide polymorphisms in the mitochondrial displacement loop

and age-at-onset of esophageal squamous cell carcinoma

SO ONCOLOGY LETTERS

LA English

DT Article

DE esophageal squamous cell carcinoma; single nucleotide polymorphisms;

displacement loop; age-at-onset

ID HEPATOCELLULAR-CARCINOMA; SOMATIC MUTATIONS; PANCREATIC-CANCER;

OXIDATIVE STRESS; DNA; REGION; IDENTIFICATION; TRANSCRIPTION;

MAINTENANCE; FREQUENT

AB The accumulation of single nucleotide polymorphisms (SNPs) in the displacement loop (D-loop) of mitochondrial DNA (mtDNA) has been described for various types of cancer, and their association with cancer risk and disease outcome has been extensively identified. In the present study, we investigated the association between age-at-onset and SNPs in the mitochondrial D-loop using a population-based series of esophageal squamous cell carcinoma (ESCC) patients. The SNP sites of nucleotides 16266 C/T were identified for their association with age-at-onset using the log-rank test. The age-at-onset of patients with the minor allele T genotype was significantly lower than that of patients with the C genotype at the 16266 site (p=0.036). Genetic polymorphisms in the D-loop are predictive markers for age-at-onset in ESCC patients. Accordingly, the analysis of genetic polymorphisms in the mitochondrial D-loop may help identify ESCC patient subgroups at high risk of early onset.

C1 [Wang, Cuiju] Hebei Med Univ, Hosp 4, Dept Gynaecol Ultrasound, Shijiazhuang 050011, Peoples R China.

[Guo, Zhanjun; Yang, Hua; Zhang, Fengbin; Zhang, Ruixing] Hebei Med Univ, Hosp 4, Dept Gastroenterol & Hepatol, Shijiazhuang 050011, Peoples R China.

[Wang, Cuiju] Hebei Med Univ, Hebei Key Lab Lab Anim Sci, Shijiazhuang 050011, Peoples R China.

RP Wang, CJ (corresponding author), Hebei Med Univ, Hosp 4, Dept Gynaecol Ultrasound, Shijiazhuang 050011, Peoples R China.

EM irenguo@yahoo.cn

FU National Natural Science Foundation of P.R. China [30801384]; Natural

Science Foundation of Hebei Province [C2008000958]

FX This work was supported by the National Natural Science Foundation of

P.R. China No. 30801384. The research was supported in part by the

Natural Science Foundation of Hebei Province No. C2008000958.

CR Abnet CC, 2004, BMC CANCER, V4, DOI 10.1186/1471-2407-4-30

Bai RK, 2007, CANCER RES, V67, P4687, DOI 10.1158/0008-5472.CAN-06-3554

BANDY B, 1990, FREE RADICAL BIO MED, V8, P523, DOI 10.1016/0891-5849(90)90152-9

Beal MF, 1996, CURR OPIN NEUROBIOL, V6, P661, DOI 10.1016/S0959-4388(96)80100-0

Blanchard P, 2009, B CANCER, V96, P379, DOI 10.1684/bdc.2008.0778

Blot W J, 1985, Natl Cancer Inst Monogr, V69, P29

Dement GA, 2007, EXP CELL RES, V313, P77, DOI 10.1016/j.yexcr.2006.09.014

Dimauro S, 2001, AM J MED GENET, V106, P18, DOI 10.1002/ajmg.1392

GILLE JJP, 1992, MUTAT RES, V275, P405, DOI 10.1016/0921-8734(92)90043-O

Lee CH, 2009, INT J CANCER, V125, P1134, DOI 10.1002/ijc.24357

Lee HC, 2004, MUTAT RES-FUND MOL M, V547, P71, DOI 10.1016/j.mrfmmm.2003.12.011

Navaglia F, 2006, AM J CLIN PATHOL, V126, P593, DOI 10.1309/GQFCCJMH5KHNVX73

Nishikawa M, 2001, CANCER RES, V61, P1843

Sanchez-Cespedes M, 2001, CANCER RES, V61, P7015

Shadel GS, 1997, ANNU REV BIOCHEM, V66, P409, DOI 10.1146/annurev.biochem.66.1.409

SHIGENAGA MK, 1994, P NATL ACAD SCI USA, V91, P10771, DOI 10.1073/pnas.91.23.10771

Taanman JW, 1999, BBA-BIOENERGETICS, V1410, P103, DOI 10.1016/S0005-2728(98)00161-3

Wang CJ, 2011, BIOCHEM BIOPH RES CO, V406, P493, DOI 10.1016/j.bbrc.2011.02.088

Wang L, 2007, CANCER EPIDEM BIOMAR, V16, P1455, DOI 10.1158/1055-9965.EPI-07-0119

Wang L, 2008, CANCER EPIDEM BIOMAR, V17, P3558, DOI 10.1158/1055-9965.EPI-08-0434

Yoneyama H, 2005, MOL CANCER RES, V3, P14

Zhang RX, 2010, J EXP CLIN CANC RES, V29, DOI 10.1186/1756-9966-29-155

Zhang RX, 2010, J EXP CLIN CANC RES, V29, DOI 10.1186/1756-9966-29-130

NR 23

TC 8

Z9 8

U1 0

U2 4

PU SPANDIDOS PUBL LTD

PI ATHENS

PA POB 18179, ATHENS, 116 10, GREECE

SN 1792-1074

EI 1792-1082

J9 ONCOL LETT

JI Oncol. Lett.

PD FEB

PY 2012

VL 3

IS 2

BP 482

EP 484

DI 10.3892/ol.2011.494

PG 3

WC Oncology

WE Science Citation Index Expanded (SCI-EXPANDED)

SC Oncology

GA 880KL

UT WOS:000299405900047

PM 22740936

OA Green Published, Green Submitted, gold

DA 2022-05-19

ER

PT J

AU Shen, JF

Ge, JF

Zheng, SY

Jiang, D

AF Shen, Jiang-Feng

Ge, Jin-Feng

Zheng, Shi-Ying

Jiang, Dong

TI Integrative analysis of differential circular RNA and long non-coding

RNA profiles and associated competing endogenous RNA networks in

esophageal squamous cell carcinoma

SO FUNCTIONAL & INTEGRATIVE GENOMICS

LA English

DT Article

DE circRNA; lncRNA; Esophageal squamous cell carcinoma; RNA sequencing;

ceRNA network

ID CANCER; PROLIFERATION; INHIBITORS; LNCRNA; STAT1

AB Long non-coding RNAs (lncRNAs) and circular RNAs (circRNAs) play vital roles in the tumorigenesis of esophageal squamous cell carcinoma (ESCC). Nevertheless, the mechanism and regulatory network associated with this process remain largely unknown. In this study, we performed a comprehensive analysis of the expression of mRNAs, lncRNAs, and circRNAs by RNA-seq. A total of 3265 mRNAs, 1084 lncRNAs, and 38 circRNAs were found to be differentially expressed. Among these, 269 mRNAs were found to encode transcription factors (TFs). Functional enrichment analysis indicated that the dysregulated TFs are associated with the Hedgehog, Jak-STAT, TGF-beta, and MAPK signaling pathways. Furthermore, we constructed co-expression networks to screen the core lncRNAs and circRNAs involved in the regulation of transcription factors in these four pathways. Finally, we constructed a competing endogenous RNA (ceRNA) network of ESCC based on the abovementioned pathways. Our findings provide important insight into the role of lncRNAs and circRNAs in ESCC; the differentially expressed lncRNAs and circRNAs may represent potential targets for ESCC diagnosis and therapy.

C1 [Shen, Jiang-Feng; Ge, Jin-Feng; Zheng, Shi-Ying; Jiang, Dong] Soochow Univ, Affiliated Hosp 1, Dept Thorac Surg, Suzhou, Peoples R China.

[Shen, Jiang-Feng] Nanjing Med Univ, Taizhou Peoples Hosp, Taizhou Clin Med Sch, Dept Thorac Surg, Taizhou, Peoples R China.

RP Zheng, SY; Jiang, D (corresponding author), Soochow Univ, Affiliated Hosp 1, Dept Thorac Surg, Suzhou, Peoples R China.

EM syzheng571025@163.com; jd159@163.com

FU National Science Foundation of China [81972800]

FX This work was supported by the National Science Foundation of China

(grant number 81972800).

CR Abnet CC, 2018, GASTROENTEROLOGY, V154, P360, DOI 10.1053/j.gastro.2017.08.023

Anastasiadou E, 2018, NAT REV CANCER, V18, P5, DOI 10.1038/nrc.2017.99

Ashburner M, 2000, NAT GENET, V25, P25, DOI 10.1038/75556

Batlle E, 2019, IMMUNITY, V50, P924, DOI 10.1016/j.immuni.2019.03.024

Chan IS, 2014, ALCOHOL CLIN EXP RES, V38, P787, DOI 10.1111/acer.12279

Chen Ru, 2019, Zhonghua Yufang Yixue Zazhi, V53, P1094, DOI 10.3760/cma.j.issn.0253-9624.2019.11.004

Cheng ZA, 2019, NAT COMMUN, V10, DOI 10.1038/s41467-019-11162-4

Darvin P, 2018, EXP MOL MED, V50, DOI 10.1038/s12276-018-0191-1

Fu XL, 2017, FUTURE MED CHEM, V9, P1227, DOI 10.4155/fmc-2017-0068

Grixti JM, 2020, NON-CODING RNA RES, V5, P77, DOI 10.1016/j.ncrna.2020.04.003

Grund-Groschke S, 2019, CELL COMMUN SIGNAL, V17, DOI 10.1186/s12964-019-0459-7

Hu CY, 2021, CURR PHARM DESIGN, V27, P1211, DOI 10.2174/1381612826666201029100151

Hu QH, 2019, PATHOL RES PRACT, V215, P843, DOI 10.1016/j.prp.2019.01.033

Huilgol D, 2019, GENES-BASEL, V10, DOI 10.3390/genes10100794

Jarroux J, 2017, ADV EXP MED BIOL, V1008, P1, DOI 10.1007/978-981-10-5203-3_1

Jiang L, 2021, J BIOCHEM, V169, P535, DOI 10.1093/jb/mvaa134

Kanehisa M, 2004, NUCLEIC ACIDS RES, V32, pD277, DOI 10.1093/nar/gkh063

Kim D, 2015, NAT METHODS, V12, P357, DOI [10.1038/NMETH.3317, 10.1038/nmeth.3317]

Krzywinski M, 2009, GENOME RES, V19, P1639, DOI 10.1101/gr.092759.109

Lagergren J, 2017, LANCET, V390, P2383, DOI 10.1016/S0140-6736(17)31462-9

Lambert M, 2018, MOLECULES, V23, DOI 10.3390/molecules23061479

Lee CJ, 2020, EXP MOL MED, V52, P1526, DOI 10.1038/s12276-020-00506-6

Li Y, 2016, TUMOR BIOL, V37, P13091, DOI 10.1007/s13277-016-5227-3

Lin RH, 2016, ONCOTARGET, V7, P24564, DOI 10.18632/oncotarget.8356

Liu B, 2014, BRIEF BIOINFORM, V15, P1, DOI 10.1093/bib/bbs075

Liu Y, 2018, J EXP CLIN CANC RES, V37, DOI 10.1186/s13046-018-0959-0

Lu CW, 2017, ONCOIMMUNOLOGY, V6, DOI 10.1080/2162402X.2017.1291106

Ma JL, 2017, CANCER LETT, V398, P37, DOI 10.1016/j.canlet.2017.04.006

Ma XL, 2006, INT J CANCER, V118, P139, DOI 10.1002/ijc.21295

Mao Y S, 2020, Zhonghua Zhong Liu Za Zhi, V42, P228, DOI 10.3760/cma.j.cn112152-20191112-00729

Matsui M, 2017, NAT REV DRUG DISCOV, V16, P167, DOI 10.1038/nrd.2016.117

Meissl K, 2017, CYTOKINE, V89, P12, DOI 10.1016/j.cyto.2015.11.011

Meng SJ, 2017, MOL CANCER, V16, DOI 10.1186/s12943-017-0663-2

Mitra P, 2018, ANN TRANSL MED, V6, DOI 10.21037/atm.2018.09.62

Pancione M, 2017, CURR MED CHEM, V24, P1383, DOI 10.2174/0929867324666170227114356

Papavassiliou KA, 2016, J CELL BIOCHEM, V117, P2693, DOI 10.1002/jcb.25605

Pencik J, 2016, CYTOKINE, V87, P26, DOI 10.1016/j.cyto.2016.06.017

Quirit JG, 2017, BIOCHEM PHARMACOL, V127, P13, DOI 10.1016/j.bcp.2016.12.007

Romano G, 2017, CARCINOGENESIS, V38, P485, DOI 10.1093/carcin/bgx026

Sheng WQ, 2018, CELL, V174, P549, DOI 10.1016/j.cell.2018.05.052

Shi XY, 2020, EUR REV MED PHARMACO, V24, P9532, DOI 10.26355/eurrev_202009_23038

Sui GP, 2006, J SURG RES, V134, P1, DOI 10.1016/j.jss.2005.12.029

Sun S, 2010, J PROTEOME RES, V9, P70, DOI 10.1021/pr9002118

Tay Y, 2014, NATURE, V505, P344, DOI 10.1038/nature12986

Wang CC, 2020, J CELL PHYSIOL, V235, P8224, DOI 10.1002/jcp.29477

Yang Z, 2019, AUTOPHAGY, V15, P668, DOI 10.1080/15548627.2018.1539590

Yeh JE, 2013, CURR OPIN ONCOL, V25, P652, DOI 10.1097/01.cco.0000432528.88101.1a

Yoshikawa R, 2008, BRIT J CANCER, V98, P1670, DOI 10.1038/sj.bjc.6604361

You XT, 2015, NAT NEUROSCI, V18, P603, DOI 10.1038/nn.3975

Zhang L, 2020, BMC PULM MED, V20, DOI 10.1186/s12890-020-01180-0

Zhang L, 2019, J EXP CLIN CANC RES, V38, DOI 10.1186/s13046-019-1059-5

Zhang Y, 2017, DISCOV MED, V24, P19

NR 52

TC 1

Z9 1

U1 0

U2 4

PU SPRINGER HEIDELBERG

PI HEIDELBERG

PA TIERGARTENSTRASSE 17, D-69121 HEIDELBERG, GERMANY

SN 1438-793X

EI 1438-7948

J9 FUNCT INTEGR GENOMIC

JI Funct. Integr. Genomics

PD JAN

PY 2021

VL 21

IS 1

BP 125

EP 138

DI 10.1007/s10142-020-00765-6

EA JAN 2021

PG 14

WC Genetics & Heredity

WE Science Citation Index Expanded (SCI-EXPANDED)

SC Genetics & Heredity

GA PU7QU

UT WOS:000605910000001

PM 33415515

DA 2022-05-19

ER

PT J

AU Zheng, YX

Chen, ZY

Han, YC

Han, L

Zou, X

Zhou, BQ

Hu, R

Hao, J

Bai, SH

Xiao, HB

Li, WV

Bueker, A

Ma, YH

Xie, GH

Yang, JY

Chen, SY

Li, HC

Cao, J

Shen, LS

AF Zheng, Yingxia

Chen, Zheyi

Han, Yichao

Han, Li

Zou, Xin

Zhou, Bingqian

Hu, Rui

Hao, Jie

Bai, Shihao

Xiao, Haibo

Li, Wei Vivian

Bueker, Alex

Ma, Yanhui

Xie, Guohua

Yang, Junyao

Chen, Shiyu

Li, Hecheng

Cao, Jian

Shen, Lisong

TI Immune suppressive landscape in the human esophageal squamous cell

carcinoma microenvironment

SO NATURE COMMUNICATIONS

LA English

DT Article

ID T-CELLS; LYMPHOCYTES; MACROPHAGES; EXPRESSION; INFERENCE; DYNAMICS;

SURVIVAL; MARKERS; FATE

AB Cancer immunotherapy has revolutionized cancer treatment, and it relies heavily on the comprehensive understanding of the immune landscape of the tumor microenvironment (TME). Here, we obtain a detailed immune cell atlas of esophageal squamous cell carcinoma (ESCC) at single-cell resolution. Exhausted T and NK cells, regulatory T cells (Tregs), alternatively activated macrophages and tolerogenic dendritic cells are dominant in the TME. Transcriptional profiling coupled with T cell receptor (TCR) sequencing reveal lineage connections in T cell populations. CD8 T cells show continuous progression from pre-exhausted to exhausted T cells. While exhausted CD4, CD8 T and NK cells are major proliferative cell components in the TME, the crosstalk between macrophages and Tregs contributes to potential immunosuppression in the TME. Our results indicate several immunosuppressive mechanisms that may be simultaneously responsible for the failure of immuno-surveillance. Specific targeting of these immunosuppressive pathways may reactivate anti-tumor immune responses in ESCC. Understanding the tumour microenvironment is essential for the efficacy of immunotherapies. Here the authors describe the immune landscape in esophageal squamous cell carcinoma and suggest several immunosuppressive mechanisms, which upon targeting may restore anti-tumour immune response.

C1 [Zheng, Yingxia; Chen, Zheyi; Han, Li; Zhou, Bingqian; Ma, Yanhui; Xie, Guohua; Yang, Junyao; Chen, Shiyu; Shen, Lisong] Shanghai Jiao Tong Univ, Xin Hua Hosp, Dept Lab Med, Sch Med, Shanghai, Peoples R China.

[Zheng, Yingxia] Shanghai Jiao Tong Univ, Inst Biliary Tract Dis Res, Sch Med, Shanghai, Peoples R China.

[Han, Yichao; Li, Hecheng] Shanghai Jiao Tong Univ, Ruijin Hosp, Dept Thorac Surg, Sch Med, Shanghai, Peoples R China.

[Zou, Xin; Hao, Jie; Bai, Shihao] Shanghai Jiao Tong Univ, Shanghai Ctr Syst Biomed, Minist Educ, Key Lab Syst Biomed, Shanghai, Peoples R China.

[Hu, Rui; Xiao, Haibo] Shanghai Jiao Tong Univ, Xin Hua Hosp, Dept Thorac Surg, Sch Med, Shanghai, Peoples R China.

[Li, Wei Vivian] Rutgers Sch Publ Hlth, Dept Biostat & Epidemiol, New Brunswick, NJ USA.

[Bueker, Alex; Cao, Jian] Rutgers Canc Inst New Jersey, New Brunswick, NJ 08901 USA.

[Cao, Jian] Rutgers State Univ, Robert Wood Johnson Med Sch, Dept Med, New Brunswick, NJ 08854 USA.

[Shen, Lisong] Shanghai Jiao Tong Univ, Fac Med Lab Sci, Sch Med, Shanghai, Peoples R China.

RP Zheng, YX; Shen, LS (corresponding author), Shanghai Jiao Tong Univ, Xin Hua Hosp, Dept Lab Med, Sch Med, Shanghai, Peoples R China.; Zheng, YX (corresponding author), Shanghai Jiao Tong Univ, Inst Biliary Tract Dis Res, Sch Med, Shanghai, Peoples R China.; Li, HC (corresponding author), Shanghai Jiao Tong Univ, Ruijin Hosp, Dept Thorac Surg, Sch Med, Shanghai, Peoples R China.; Cao, J (corresponding author), Rutgers Canc Inst New Jersey, New Brunswick, NJ 08901 USA.; Cao, J (corresponding author), Rutgers State Univ, Robert Wood Johnson Med Sch, Dept Med, New Brunswick, NJ 08854 USA.; Shen, LS (corresponding author), Shanghai Jiao Tong Univ, Fac Med Lab Sci, Sch Med, Shanghai, Peoples R China.

EM zhengyingxia@xinhuamed.com.cn; lihecheng2000@hotmail.com;

jian.cao@cinj.rutgers.edu; lisongshen@hotmail.com

RI Han, Li/AGZ-0451-2022

OI Han, Li/0000-0001-7303-5739; Li, Wei Vivian/0000-0002-2087-2709; Hu,

Rui/0000-0002-9765-9922; HAN, YICHAO/0000-0002-9691-3028

FU National Natural Science Foundation of China [81571525, 81873863,

81672363, 81871882]; Shanghai Municipal Education Commission-Gaofeng

Clinical Medicine Grant Support [20161315]; Shanghai Pujiang Talent Plan

[17PJD027]; Key Specialty Development Program of Xin Hua Hospital;

Shanghai Municipal Health Commission; Clinical Research Plan of SHDC

[16CR3057A]; Medicine and Engineering Cross Research Foundation of

Shanghai Jiao Tong University [YG2017ZD02]; Rutgers Cancer Institute of

New Jersey (State of NJ appropriation); Rutgers School of Public Health

Pilot Grant; New Jersey Alliance for Clinical and Translational Science

Mini-methods Grant; GorgeosAI (Gu Ai) Biotechnology; Program of

Innovative Research Team of High-level Local Universities in Shanghai;

Rutgers Cancer Institute of New Jersey (National Institutes of Health)

[P30CA072720]

FX We thank Prof. Bin Li for his valuable scientific advice and discussion.

This work was supported by grants from the National Natural Science

Foundation of China [81571525 and 81873863 to Y.Z., 81672363 to L.S.,

81871882 to H.L.], the Shanghai Municipal Education Commission-Gaofeng

Clinical Medicine Grant Support [20161315 to Y.Z.], and the Shanghai

Pujiang Talent Plan [17PJD027 to Y.Z.], the Key Specialty Development

Program of Xin Hua Hospital and Shanghai Municipal Health Commission (to

L.S.), the Clinical Research Plan of SHDC (Project No. 16CR3057A, to

L.S.), the Medicine and Engineering Cross Research Foundation of

Shanghai Jiao Tong University (Project No. YG2017ZD02 to L.S.), the

Start-up Fund and a New Investigator Award (both to J.C.) provided by

Rutgers Cancer Institute of New Jersey (State of NJ appropriation and

National Institutes of Health grant P30CA072720), the Rutgers School of

Public Health Pilot Grant and the New Jersey Alliance for Clinical and

Translational Science Mini-methods Grant to W.V.L. The single cell

RNA-seq was supported by Genergy Biotechnology of Shanghai. The data

analysis was supported by GorgeosAI (Gu Ai) Biotechnology and the

Program of Innovative Research Team of High-level Local Universities in

Shanghai.

CR Aibar S, 2017, NAT METHODS, V14, P1083, DOI [10.1038/NMETH.4463, 10.1038/nmeth.4463]

Alexandrov LB, 2013, NATURE, V500, P415, DOI 10.1038/nature12477

Andre P, 2018, CELL, V175, P1731, DOI 10.1016/j.cell.2018.10.014

Andreas N, 2018, EUR J IMMUNOL, V48, P923, DOI 10.1002/eji.201747332

[Anonymous], [No title captured]

Azizi E, 2018, CELL, V174, P1293, DOI 10.1016/j.cell.2018.05.060

Bai AP, 2015, NAT COMMUN, V6, DOI 10.1038/ncomms9819

Barkal AA, 2018, NAT IMMUNOL, V19, P76, DOI 10.1038/s41590-017-0004-z

Briseno CG, 2017, P NATL ACAD SCI USA, V114, P3957, DOI 10.1073/pnas.1619863114

Chen ZC, 2016, J PATHOL, V239, P450, DOI 10.1002/path.4742

Condamine T, 2016, SCI IMMUNOL, V1, DOI 10.1126/sciimmunol.aaf8943

De Simone M, 2016, IMMUNITY, V45, P1135, DOI 10.1016/j.immuni.2016.10.021

Duhen T, 2018, NAT COMMUN, V9, DOI 10.1038/s41467-018-05072-0

Ghoneim HE, 2017, CELL, V170, DOI 10.1016/j.cell.2017.06.007

Griss J, 2019, NAT COMMUN, V10, DOI 10.1038/s41467-019-12160-2

Guo XY, 2018, NAT MED, V24, P1628, DOI 10.1038/s41591-018-0167-7

Guo XY, 2018, NAT MED, V24, P978, DOI 10.1038/s41591-018-0045-3

Gyorffy B, 2010, BREAST CANCER RES TR, V123, P725, DOI 10.1007/s10549-009-0674-9

Hao J, 2019, BIOINFORMATICS, V35, P2602, DOI 10.1093/bioinformatics/bty1016

Jarjour NN, 2019, NAT IMMUNOL, V20, P687, DOI 10.1038/s41590-019-0382-5

Jerby-Arnon L, 2018, CELL, V175, P984, DOI 10.1016/j.cell.2018.09.006

Kato K, 2019, LANCET ONCOL, V20, P1506, DOI 10.1016/S1470-2045(19)30626-6

Kim J, 2017, NATURE, V541, P169, DOI 10.1038/nature20805

Kim N, 2020, NAT COMMUN, V11, DOI 10.1038/s41467-020-16164-1

Kumar MP, 2018, CELL REP, V25, P1458, DOI 10.1016/j.celrep.2018.10.047

Kuo HY, 2020, J FORMOS MED ASSOC, V119, P565, DOI 10.1016/j.jfma.2019.10.010

Lambrechts D, 2018, NAT MED, V24, P1277, DOI 10.1038/s41591-018-0096-5

Lavin Y, 2017, CELL, V169, P750, DOI 10.1016/j.cell.2017.04.014

Li HJ, 2019, CELL, V176, P775, DOI 10.1016/j.cell.2018.11.043

Li Y, 2017, ONCOIMMUNOLOGY, V6, DOI 10.1080/2162402X.2017.1356147

Liu JY, 2015, BRIT J CANCER, V113, P747, DOI 10.1038/bjc.2015.290

Macosko EZ, 2015, CELL, V161, P1202, DOI 10.1016/j.cell.2015.05.002

Maier B, 2020, NATURE, V580, P257, DOI 10.1038/s41586-020-2134-y

Mantovani A, 2002, TRENDS IMMUNOL, V23, P549, DOI 10.1016/S1471-4906(02)02302-5

McLane LM, 2019, ANNU REV IMMUNOL, V37, P457, DOI 10.1146/annurev-immunol-041015-055318

Mercer F, 2010, PLOS ONE, V5, DOI 10.1371/journal.pone.0008639

Navarro-Barriuso J, 2018, FRONT IMMUNOL, V9, DOI 10.3389/fimmu.2018.02062

Oh DY, 2020, CELL, V181, P1612, DOI 10.1016/j.cell.2020.05.017

Pauken KE, 2016, SCIENCE, V354, P1160, DOI 10.1126/science.aaf2807

Penter L, 2019, ONCOIMMUNOLOGY, V8, DOI 10.1080/2162402X.2019.1586409

Prat Q, 2019, FRONT PSYCHOL, V10, DOI 10.3389/fpsyg.2019.01072

Ritvo PGG, 2017, SCI IMMUNOL, V2, DOI 10.1126/sciimmunol.aan0368

Rustgi AK, 2014, NEW ENGL J MED, V371, P2499, DOI 10.1056/NEJMra1314530

Sade-Feldman M, 2018, CELL, V175, P998, DOI 10.1016/j.cell.2018.10.038

Savas P, 2018, NAT MED, V24, P986, DOI 10.1038/s41591-018-0078-7

Scott AC, 2019, NATURE, V571, P270, DOI 10.1038/s41586-019-1324-y

Sen DR, 2016, SCIENCE, V354, P1165, DOI 10.1126/science.aae0491

Simoni Y, 2018, NATURE, V557, P575, DOI 10.1038/s41586-018-0130-2

Stojanovic A, 2018, NAT IMMUNOL, V19, P650, DOI 10.1038/s41590-018-0142-y

Street K, 2018, BMC GENOMICS, V19, DOI 10.1186/s12864-018-4772-0

Stubbington MJT, 2016, NAT METHODS, V13, P329, DOI [10.1038/NMETH.3800, 10.1038/nmeth.3800]

Tirosh I, 2016, SCIENCE, V352, P189, DOI 10.1126/science.aad0501

Trapnell C, 2014, NAT BIOTECHNOL, V32, P381, DOI 10.1038/nbt.2859

Whitfield ML, 2006, NAT REV CANCER, V6, P99, DOI 10.1038/nrc1802

Wu TD, 2020, NATURE, V579, P274, DOI 10.1038/s41586-020-2056-8

Xu HX, 2012, NAT IMMUNOL, V13, P642, DOI 10.1038/ni.2304

Yao JC, 2020, NAT COMMUN, V11, DOI 10.1038/s41467-020-17492-y

Yost KE, 2019, NAT MED, V25, P1251, DOI 10.1038/s41591-019-0522-3

Zhang L, 2020, CELL, V181, P442, DOI 10.1016/j.cell.2020.03.048

Zhang L, 2018, NATURE, V564, P268, DOI 10.1038/s41586-018-0694-x

Zhang QM, 2019, CELL, V179, P829, DOI 10.1016/j.cell.2019.10.003

Zhao JM, 2019, SCAND J IMMUNOL, V90, DOI 10.1111/sji.12804

Zheng CH, 2017, CELL, V169, P1342, DOI 10.1016/j.cell.2017.05.035

Zilionis R, 2019, IMMUNITY, V50, P1317, DOI 10.1016/j.immuni.2019.03.009

NR 64

TC 40

Z9 40

U1 15

U2 34

PU NATURE PORTFOLIO

PI BERLIN

PA HEIDELBERGER PLATZ 3, BERLIN, 14197, GERMANY

EI 2041-1723

J9 NAT COMMUN

JI Nat. Commun.

PD DEC 8

PY 2020

VL 11

IS 1

AR 6268

DI 10.1038/s41467-020-20019-0

PG 17

WC Multidisciplinary Sciences

WE Science Citation Index Expanded (SCI-EXPANDED)

SC Science & Technology - Other Topics

GA PT8CC

UT WOS:000608837700005

PM 33293583

OA Green Published, gold

DA 2022-05-19

ER

PT J

AU Koh, HK

Park, Y

Koo, T

Park, HJ

Lee, MY

Chang, AR

Hong, S

Bae, H

AF Koh, Hyeon Kang

Park, Younghee

Koo, Taeryool

Park, Hae Jin

Lee, Me Yeon

Chang, Ah Ram

Hong, Semie

Bae, Hoonsik

TI Adjuvant Chemotherapy and Dose Escalation in Definitive Concurrent

Chemoradiotherapy for Esophageal Squamous Cell Carcinoma

SO ANTICANCER RESEARCH

LA English

DT Article

DE Chemoradiotherapy; esophageal squamous cell carcinoma; adjuvant

chemotherapy; radiotherapy dose escalation

ID RADIATION-THERAPY; RESPONSE RELATIONSHIP; RANDOMIZED-TRIAL; CANCER;

RADIOTHERAPY; STANDARD; CISPLATIN; CETUXIMAB

AB Background/Aim: To validate the effect of treatment intensification on survival in esophageal squamous cell carcinoma (ESCC) patients undergoing definitive concurrent chemoradiotherapy (dCCRT). Patients and Methods: We reviewed the medical records of 73 ESCC patients who underwent dCCRT between 2006 and 2017 in 3 institutions. Results: The median follow-up time was 13.3 months. The median overall survival ( OS) and locoregional recurrence-free survival (LRFS) were 13.3 and 11.2 months, respectively. The median radiotherapy dose was 55.8 Gy, and the median biologically effective dose (BED) was 65.8 Gy. Chemotherapy was given in all patients during dCCRT, and adjuvant chemotherapy was administered in 56 patients (76.7%). Adjuvant chemotherapy improved OS (3-year, 24.2% vs. 11.8%, p=0.004). Higher BED >= 70 Gy improved LRFS (3-year, 41.7% vs. 23.6%, p=0.035). Conclusion: The addition of chemotherapy after dCCRT improves OS. A higher radiotherapy dose improved LRFS, but not OS. Adjuvant chemotherapy should be considered after dCCRT for better outcomes.

C1 [Koh, Hyeon Kang] Konkuk Univ, Sch Med, Dept Radiat Oncol, Seoul, South Korea.

[Koh, Hyeon Kang; Hong, Semie] Konkuk Univ, Med Ctr, Seoul, South Korea.

[Park, Younghee; Chang, Ah Ram] Soonchunhyang Univ Seoul Hosp, CyberKnife Ctr, Dept Radiat Oncol, Seoul, South Korea.

[Koo, Taeryool; Lee, Me Yeon; Bae, Hoonsik] Hallym Univ, Coll Med, Sacred Heart Hosp, Dept Radiat Oncol, Gyeonggi Do, South Korea.

[Park, Hae Jin] Hanyang Univ, Coll Med, Dept Radiat Oncol, Seoul, South Korea.

RP Koo, T (corresponding author), Hallym Univ, Sacred Heart Hosp, Coll Med, Dept Radiat Oncol, 22,Gwanpyeong Ro,170beon Gil, Anyang Si 14068, Gyeonggi Do, South Korea.

EM kootaeryool@hallym.or.kr

OI Koh, Hyeon Kang/0000-0002-6855-1832

CR Abnet CC, 2018, GASTROENTEROLOGY, V154, P360, DOI 10.1053/j.gastro.2017.08.023

Ajani JA, 2008, J CLIN ONCOL, V26, P4551, DOI 10.1200/JCO.2008.16.6918

[Anonymous], 2019, ES ES JUNCT CANC VER

Bese NS, 2007, INT J RADIAT ONCOL, V68, P654, DOI 10.1016/j.ijrobp.2007.03.010

Bray F, 2018, CA-CANCER J CLIN, V68, P394, DOI 10.3322/caac.21492

Cao CN, 2015, HEAD NECK-J SCI SPEC, V37, P151, DOI 10.1002/hed.23572

Chen MQ, 2018, RADIAT ONCOL, V13, DOI 10.1186/s13014-018-1086-y

Chen NB, 2020, CANCER RES TREAT, V52, P31, DOI 10.4143/crt.2018.624

Conroy T, 2014, LANCET ONCOL, V15, P305, DOI 10.1016/S1470-2045(14)70028-2

Cooper JS, 1999, JAMA-J AM MED ASSOC, V281, P1623, DOI 10.1001/jama.281.17.1623

Crosby T, 2013, LANCET ONCOL, V14, P627, DOI 10.1016/S1470-2045(13)70136-0

Fan CY, 2018, SCI REP-UK, V8, DOI 10.1038/s41598-018-31302-y

Grills IS, 2003, INT J RADIAT ONCOL, V57, P875, DOI 10.1016/S0360-3016(03)00743-0

Jung KW, 2019, CANCER RES TREAT, V51, P417, DOI 10.4143/crt.2019.138

Kim DE, 2013, CANCER RES TREAT, V45, P276, DOI 10.4143/crt.2013.45.4.276

Kim HJ, 2017, CANCER RES TREAT, V49, P669, DOI 10.4143/crt.2016.354

Kim TH, 2019, HEAD NECK-J SCI SPEC, V41, P146, DOI 10.1002/hed.25483

Li CC, 2019, ANTICANCER RES, V39, P511, DOI 10.21873/anticanres.13142

Lordick F, 2016, ANN ONCOL, V27, pv50, DOI 10.1093/annonc/mdw329

Minsky BD, 2002, J CLIN ONCOL, V20, P1167, DOI 10.1200/JCO.20.5.1167

Minsky BD, 1999, INT J RADIAT ONCOL, V43, P517, DOI 10.1016/S0360-3016(98)00463-5

R Development Core Team, 2019, R LANG ENV STAT COMP

Shin A, 2018, J GASTROEN HEPATOL, V33, P1961, DOI 10.1111/jgh.14289

Smith TJ, 1998, INT J RADIAT ONCOL, V42, P269, DOI 10.1016/S0360-3016(98)00232-6

Suntharalingam M, 2017, JAMA ONCOL, V3, P1520, DOI 10.1001/jamaoncol.2017.1598

van Hagen P, 2012, NEW ENGL J MED, V366, P2074, DOI 10.1056/NEJMoa1112088

Welsh J, 2012, INT J RADIAT ONCOL, V82, P468, DOI 10.1016/j.ijrobp.2010.10.023

Wu SX, 2017, SCI REP-UK, V7, DOI 10.1038/s41598-017-17254-9

Zhang Z, 2005, INT J RADIAT ONCOL, V61, P656, DOI 10.1016/j.ijrobp.2004.06.022

NR 29

TC 5

Z9 5

U1 0

U2 1

PU INT INST ANTICANCER RESEARCH

PI ATHENS

PA EDITORIAL OFFICE 1ST KM KAPANDRITIOU-KALAMOU RD KAPANDRITI, PO BOX 22,

ATHENS 19014, GREECE

SN 0250-7005

EI 1791-7530

J9 ANTICANCER RES

JI Anticancer Res.

PD MAR

PY 2020

VL 40

IS 3

BP 1771

EP 1778

DI 10.21873/anticanres.14131

PG 8

WC Oncology

WE Science Citation Index Expanded (SCI-EXPANDED)

SC Oncology

GA KY7CX

UT WOS:000522732700071

PM 32132086

DA 2022-05-19

ER

PT J

AU Staudenmaier, AM

Simone-Freilicher, EA

Noonan, BP

Ewing, PJ

AF Staudenmaier, Anne M.

Simone-Freilicher, Elisabeth A.

Noonan, Brendan P.

Ewing, Patty J.

TI Antemortem diagnosis of esophageal squamous cell carcinoma in a

blue-fronted Amazon parrot (Amazona aestiva) and a mealy Amazon parrot

(Amazona farinosa)

SO JAVMA-JOURNAL OF THE AMERICAN VETERINARY MEDICAL ASSOCIATION

LA English

DT Article

ID OROPHARYNX; THYMOMA; CANCER

AB CASE DESCRIPTION

A 28-year-old blue-fronted Amazon parrot (Amazona aestiva) with a 1-week history of regurgitation-like movement of the neck and a 34-year-old mealy Amazon parrot (Amazona farinosa) with a 1-to 2-day history of regurgitation and crop distention were evaluated.

CLINICAL FINDINGS

Full-body radiography of the blue-fronted Amazon parrot revealed a soft tissue structure in the right side of the neck, cranial to the crop. Results of cytologic examination of a fine-needle aspirate specimen of the mass were diagnostic for an epithelial sarcoma involving the esophagus. For the mealy Amazon parrot, CT revealed diffuse esophageal thickening that was most consistent with esophageal squamous cell carcinoma on the basis of its location and appearance. Both parrots had secondary bacterial ingluvitis.

TREATMENT AND OUTCOME

Given the location and extent of the neoplasm in each bird, surgical excision was not undertaken. Options of chemotherapy and radiation therapy were explored but declined by the owners owing to concerns regarding the parrots' quality of life. Both parrots were euthanized, and necropsy findings confirmed the diagnosis of esophageal squamous cell carcinoma in both cases.

C1 [Staudenmaier, Anne M.; Simone-Freilicher, Elisabeth A.; Noonan, Brendan P.] Angell Anim Med Ctr, Dept Avian & Exot Anim Med, 350 S Huntington Ave, Boston, MA 02120 USA.

[Ewing, Patty J.] Angell Anim Med Ctr, Dept Pathol, 350 S Huntington Ave, Boston, MA 02120 USA.

RP Staudenmaier, AM (corresponding author), Angell Anim Med Ctr, Dept Avian & Exot Anim Med, 350 S Huntington Ave, Boston, MA 02120 USA.

EM astaudenmaier@mspca.org

CR Allen JW, 1997, SURG ONCOL, V6, P193, DOI 10.1016/S0960-7404(98)00003-6

ANDERSON WI, 1989, AVIAN DIS, V33, P827, DOI 10.2307/1591169

Bertone ER, 2003, J VET INTERN MED, V17, P557, DOI 10.1892/0891-6640(2003)017&lt;0557:EALRFF&gt;2.3.CO;2

Higuchi Katsuhiko, 2009, Gastrointest Cancer Res, V3, P153

IDE H, 1994, WORLD J SURG, V18, P321, DOI 10.1007/BF00316810

Kato K., 2015, ESOPHAGEAL SQUAMOUS, P197

Kaur Gurkiran, 2009, Indian J Dent Res, V20, P71

Khan N, 2013, J CYTOL, V30, P263, DOI 10.4103/0970-9371.126661

KOLBUSZ R, 1988, ACTA CYTOL, V32, P399

Latimer KS, 2001, J AVIAN MED SURG, V15, P37, DOI 10.1647/1082-6742(2001)015[0037:TIAF]2.0.CO;2

Latimer KS, 2009, AVIAN MED PRINCIPLES, P640

Manucy TK, 1998, J AVIAN MED SURG, V12, P158

Matsuda S, 2016, ANN THORAC CARDIOVAS, V22, P275, DOI 10.5761/atcs.ra.16-00111

MURTAUGH RJ, 1986, J AM VET MED ASSOC, V188, P872

Nair V., 2013, DIS POULTRY, P513

Nordio L, 2016, AVIAN DIS, V60, P694, DOI 10.1637/11374-011516-Case.1

PRIESTER WA, 1975, AVIAN DIS, V19, P213, DOI 10.2307/1588974

Stathopoulos GP, 2003, ONCOL REP, V10, P449

STRAFUSS AC, 1970, AVIAN DIS, V14, P406, DOI 10.2307/1588486

Vazquez S, 2003, AVIAN DIS, V47, P215, DOI 10.1637/0005-2086(2003)047[0215:SCCOTO]2.0.CO;2

Zehnder A., 2016, CURRENT THERAPY AVIA, V1st ed, P107

ZUBAIDY AJ, 1980, AVIAN PATHOL, V9, P575, DOI 10.1080/03079458008418445

NR 22

TC 1

Z9 1

U1 0

U2 2

PU AMER VETERINARY MEDICAL ASSOC

PI SCHAUMBURG

PA 1931 N MEACHAM RD SUITE 100, SCHAUMBURG, IL 60173-4360 USA

SN 0003-1488

EI 1943-569X

J9 JAVMA-J AM VET MED A

JI JAVMA-J. Am. Vet. Med. Assoc.

PD JUN 1

PY 2019

VL 254

IS 11

BP 1324

EP 1328

PG 5

WC Veterinary Sciences

WE Science Citation Index Expanded (SCI-EXPANDED)

SC Veterinary Sciences

GA HX4LD

UT WOS:000467366400027

PM 31067188

DA 2022-05-19

ER

PT J

AU Hou, GQ

Zhao, Q

Zhang, MY

Wang, P

Ye, H

Wang, Y

Ren, YD

Zhang, JY

Lu, ZM

AF Hou, Guiqin

Zhao, Qi

Zhang, Mengying

Wang, Peng

Ye, Hua

Wang, Yang

Ren, Yandan

Zhang, Jianying

Lu, Zhaoming

TI LSD1 regulates Notch and PI3K/Akt/mTOR pathways through binding the

promoter regions of Notch target genes in esophageal squamous cell

carcinoma

SO ONCOTARGETS AND THERAPY

LA English

DT Article

DE LSD1; Notch; PI3K/Akt/mTOR; esophageal squamous cell carcinoma

ID HISTONE DEMETHYLASE LSD1; INHIBITION; ACTIVATION; EXPRESSION

AB Background: The aberrant activation of Lysine-specific demethylase 1(LSD1), Notch and PI3K/Akt/mTOR signaling pathways were frequently happened in many cancers, including esophageal squamous cell carcinoma (ESCC). However, the regulatory relationship between LSD1 and Notch as well as PI3K/Akt/mTOR pathways is still unclear.

Purpose: This study aimed to explore the regulatory effects and mechanisms of LSD1 on Notch and PI3K/Akt/mTOR pathway in ESCC.

Results: Firstly, we demonstrated that LSD1 and proteins in Notch and PI3K/Akt/mTOR pathway were expressed in ESCC cells. Secondly, inhibition of LSD1 by tranylcypromine (TCP) or shRNA could decrease the expressions of related proteins in Notch and PI3K/Akt/ mTOR signaling pathways in ESCC cells. Finally, we found that LSD1 could bind to the promoter regions of Notch3, Hes1 and CR2, and the combinations between them were reduced by TCP in ESCC.

Conclusion: Summarily, this study indicated that LSD1 might positively regulate Notch and PI3K/Akt/mTOR pathways through binding the promoter regions of related genes in Notch pathway in ESCC.

C1 [Hou, Guiqin; Zhao, Qi; Zhang, Mengying; Wang, Yang; Ren, Yandan; Lu, Zhaoming] Zhengzhou Univ, Sch Pharmaceut Sci, 100 Kexue Ave, Zhengzhou 450001, Henan, Peoples R China.

[Wang, Peng; Ye, Hua; Zhang, Jianying] Zhengzhou Univ, Coll Publ Hlth, Zhengzhou, Henan, Peoples R China.

[Zhang, Jianying] Zhengzhou Univ, Henan Acad Med & Pharmaceut Sci, 40 Daxue Rd, Zhengzhou 450052, Henan, Peoples R China.

RP Lu, ZM (corresponding author), Zhengzhou Univ, Sch Pharmaceut Sci, 100 Kexue Ave, Zhengzhou 450001, Henan, Peoples R China.; Zhang, JY (corresponding author), Zhengzhou Univ, Henan Acad Med & Pharmaceut Sci, 40 Daxue Rd, Zhengzhou 450052, Henan, Peoples R China.

EM jianyingzhang@hotmail.com; lzm310@zzu.edu.cn

FU Natural Science Foundation of Henan Province, China [182300410312];

National Science and Technology Major projects of China [2018ZX10302205]

FX This study is supported by the Natural Science Foundation of Henan

Province, China (No. 182300410312) and the National Science and

Technology Major projects of China (No: 2018ZX10302205).

CR Augert A, 2019, SCI SIGNAL, V12, P567, DOI [10.1126/scisignal.aat8595, DOI 10.1126/SCISIGNAL.AAT8595]

Capaccione KM, 2013, CARCINOGENESIS, V34, P1420, DOI 10.1093/carcin/bgt127

Dawson MA, 2012, CELL, V150, P12, DOI 10.1016/j.cell.2012.06.013

Di Stefano L, 2011, GENE DEV, V25, P17, DOI 10.1101/gad.1983711

Efferson CL, 2010, CANCER RES, V70, P2476, DOI 10.1158/0008-5472.CAN-09-3114

Feng SJ, 2016, MED SCI MONITOR, V22, P4742, DOI 10.12659/MSM.898825

Ferrari-Amorotti G, 2014, NEOPLASIA, V16, P1047, DOI 10.1016/j.neo.2014.10.006

Forghanifard MM, 2015, PATHOL ONCOL RES, V21, P463, DOI 10.1007/s12253-014-9849-8

Guo SC, 2011, BBA-REV CANCER, V1815, P197, DOI 10.1016/j.bbcan.2010.12.002

Hales EC, 2014, CELL SIGNAL, V26, P149, DOI 10.1016/j.cellsig.2013.09.021

Haring M, 2007, PLANT METHODS, V3, DOI 10.1186/1746-4811-3-11

Hassan B, 2013, SURG ONCOL CLIN N AM, V22, P641, DOI 10.1016/j.soc.2013.06.008

Hayami S, 2011, INT J CANCER, V128, P574, DOI 10.1002/ijc.25349

Hoshino I, 2016, ANN SURG ONCOL, V23, P312, DOI 10.1245/s10434-015-4488-1

Hou GQ, 2018, BIOMED PHARMACOTHER, V106, P1348, DOI 10.1016/j.biopha.2018.07.075

Jambhekar A, 2017, CSH PERSPECT MED, V7, DOI 10.1101/cshperspect.a026484

Kim J, 2017, NATURE, V541, P169, DOI 10.1038/nature20805

Li Y, 2019, CANCER LETT, V454, P179, DOI 10.1016/j.canlet.2019.03.052

Lin YW, 2014, CELL CYCLE, V13, DOI 10.4161/cc.28619

Lopez CI, 2016, DEV NEUROBIOL, V76, P1360, DOI 10.1002/dneu.22397

Lu ZM, 2018, BIOMED PHARMACOTHER, V107, P1370, DOI 10.1016/j.biopha.2018.08.140

Mittal S, 2009, MOL CANCER, V8, DOI 10.1186/1476-4598-8-128

Moghbeli M, 2016, J CELL COMMUN SIGNAL, V10, P129, DOI 10.1007/s12079-016-0320-3

Mulligan P, 2011, MOL CELL, V42, P689, DOI 10.1016/j.molcel.2011.04.020

Murphy G, 2017, ANN ONCOL, V28, P2086, DOI 10.1093/annonc/mdx279

Pace BS, 2003, EXP HEMATOL, V31, P1089, DOI 10.1016/S0301-472X(03)00235-2

Palomero T, 2007, NAT MED, V13, P1203, DOI 10.1038/nm1636

Peng KZ, 2017, BIOCHEM PHARMACOL, V140, P16, DOI 10.1016/j.bcp.2017.05.013

Rubenstein JH, 2015, GASTROENTEROLOGY, V149, P302, DOI 10.1053/j.gastro.2015.04.053

Shi YJ, 2004, CELL, V119, P941, DOI 10.1016/j.cell.2004.12.012

Vanorny DA, 2017, REPRODUCTION, V153, pR187, DOI 10.1530/REP-16-0689

Venkatesh Vandana, 2018, Stem Cell Investig, V5, P5, DOI 10.21037/sci.2018.02.02

Wang YL, 2016, CANCER LETT, V374, P12, DOI 10.1016/j.canlet.2016.02.004

Weng AP, 2006, GENE DEV, V20, P2096, DOI 10.1101/gad.1450406

Yatim A, 2012, MOL CELL, V48, P445, DOI 10.1016/j.molcel.2012.08.022

NR 35

TC 10

Z9 12

U1 9

U2 14

PU DOVE MEDICAL PRESS LTD

PI ALBANY

PA PO BOX 300-008, ALBANY, AUCKLAND 0752, NEW ZEALAND

SN 1178-6930

J9 ONCOTARGETS THER

JI OncoTargets Ther.

PY 2019

VL 12

BP 5215

EP 5225

DI 10.2147/OTT.S207238

PG 11

WC Biotechnology & Applied Microbiology; Oncology

WE Science Citation Index Expanded (SCI-EXPANDED)

SC Biotechnology & Applied Microbiology; Oncology

GA IF9GQ

UT WOS:000473401100007

PM 31308693

OA Green Published, Green Submitted

DA 2022-05-19

ER

PT J

AU Bai, QZ

Yu, J

Li, YS

Ma, JL

Gou, YJ

AF Bai, Qizhou

Yu, Jun

Li, Yongshun

Ma, Jilong

Gou, Yunjiu

TI MicroRNA-182 promoted esophageal squamous cell carcinoma cell growth and

metastasis via targeting YWHAG

SO JOURNAL OF BUON

LA English

DT Article

DE esophageal squamous cell carcinoma; metastasis; miR-182; proliferation;

YWHAG

ID CANCER; EXPRESSION; BIOMARKERS; INVASION; PROLIFERATION; MIR-182;

RELEVANCE; APOPTOSIS; FEATURES; OUTCOMES

AB Purpose: To study the functioning mode of miR-182 on esophageal squamous cell carcinoma (ESCC) cell growth and metastasis and provide therapeutic targets for ESCC.

Methods: miR-182 expression level in ESCC cell lines was examined by quantitative real-time polymerase chain reaction (qRT-PCR). Using miR-182 inhibitor, we analyzed the effects of miR-182 down-expression on cell proliferation, invasion, cell apoptosis and cell cycle. Dual-luciferase activity assay was used to examine the potential target gene YWHAG which was predicted by several databases. Protein level was studied using western blotting.

Results: Decreased expression of miR-182 in ESCC cells was accompanied by decreased cell invasion and proliferation, promotion of cell apoptosis and cell cycle arrest at GO/GI phase. Dual-luciferase and western blot confirmed YWHAG as a target gene of miR-182. Furthermore, silence of YWHAG counteracted the suppressive effect of miR-182 down-expression on cell growth and metastasis.

Conclusions: miR-182 could suppress ESCC cell proliferation and metastasis via regulating YWHAG, which might provide a new target for ESCC diagnosis and therapy.

C1 [Bai, Qizhou; Yu, Jun; Li, Yongshun; Ma, Jilong; Gou, Yunjiu] Gansu Prov Peoples Hosp, Dept Thorac Surg, 204 Donggang West Rd, Lanzhou 730000, Gansu, Peoples R China.

RP Gou, YJ (corresponding author), Gansu Prov Peoples Hosp, Dept Thorac Surg, 204 Donggang West Rd, Lanzhou 730000, Gansu, Peoples R China.

EM gouyunjiu@163.com

FU Gansu Province People's Hospital [16GYSY3-1]

FX This study was supported by the Research Fund of Gansu Province People's

Hospital (No. 16GYSY3-1).

CR Amaral FC, 2009, J CLIN ENDOCR METAB, V94, P320, DOI 10.1210/jc.2008-1451

Chen L, 2015, TUMOR BIOL, V36, P7439, DOI 10.1007/s13277-015-3430-2

Chen QJ, 2014, ONCOL REP, V31, P1863, DOI 10.3892/or.2014.3004

Dantoc MM, 2012, J GASTROINTEST SURG, V16, P486, DOI 10.1007/s11605-011-1792-3

Dikken JL, 2012, CANCER-AM CANCER SOC, V118, P349, DOI 10.1002/cncr.26301

Fu JH, 2018, EUR REV MED PHARMACO, V22, P351, DOI 10.26355/eurrev_201801_14179

Hosokawa Y, 2012, ANN SURG ONCOL, V19, P677, DOI 10.1245/s10434-011-1983-x

Jia J, 2016, CLIN TRANSL ONCOL, V18, P592, DOI 10.1007/s12094-015-1406-8

Koshy M, 2004, ONCOLOGIST, V9, P137, DOI 10.1634/theoncologist.9-2-137

Li Y, 2013, DIAGN PATHOL, V8, DOI 10.1186/1746-1596-8-207

Ma LF, 2017, J BUON, V22, P1533

Moldovan L, 2014, J CELL MOL MED, V18, P371, DOI 10.1111/jcmm.12236

Niu GC, 2018, J BUON, V23, P157

Schreurs LMA, 2014, ANN SURG ONCOL, V21, P3751, DOI 10.1245/s10434-014-3848-6

Smyth LA, 2015, IMMUNOLOGY, V144, P197, DOI 10.1111/imm.12390

Sun JB, 2015, INT J CLIN EXP PATHO, V8, P4755

Tang SW, 2016, SCI REP-UK, V6, DOI 10.1038/srep24466

Wang HB, 2017, CYTOKINE, V92, P93, DOI 10.1016/j.cyto.2016.12.013

Winther M, 2015, ACTA ONCOL, V54, P1582, DOI 10.3109/0284186X.2015.1064161

Xia ZK, 2012, DIS ESOPHAGUS, V25, P427, DOI 10.1111/j.1442-2050.2011.01261.x

Yan D, 2012, PLOS ONE, V7

Yan JW, 2014, INT J CANCER, V135, P1011, DOI 10.1002/ijc.28563

Yang H, 2017, EUR REV MED PHARMACO, V21, P3200

Yoo JO, 2016, BBA-MOL CELL RES, V1863, P1601, DOI 10.1016/j.bbamcr.2016.04.016

Yu YJ, 2015, MOL CANCER, V14, DOI 10.1186/s12943-015-0372-7

Zhang GJ, 2015, J EXP CLIN CANC RES, V34, DOI 10.1186/s13046-015-0189-7

Zhang K, 2017, GENE, V619, P10, DOI 10.1016/j.gene.2017.03.038

Zhao H, 2017, J BUON, V22, P1540

Zhao JY, 2015, MED SCI MONITOR, V21, P3222, DOI 10.12659/MSM.895001

NR 29

TC 8

Z9 8

U1 0

U2 5

PU IMPRIMATUR PUBLICATIONS

PI ATHENS

PA 30 DEM POLIORKETES ST, ATHENS, 136 76, GREECE

SN 1107-0625

EI 2241-6293

J9 J BUON

JI J. BUON

PD SEP-OCT

PY 2018

VL 23

IS 5

BP 1439

EP 1447

PG 9

WC Oncology

WE Science Citation Index Expanded (SCI-EXPANDED)

SC Oncology

GA GS5US

UT WOS:000443737600032

PM 30570871

DA 2022-05-19

ER

PT J

AU Xiong, T

Wang, MY

Zhao, J

Liu, Q

Yang, C

Luo, W

Li, XC

Yang, HM

Kristiansen, K

Roy, B

Zhou, Y

AF Xiong, Teng

Wang, Mengyao

Zhao, Jing

Liu, Qing

Yang, Chao

Luo, Wen

Li, Xiangchun

Yang, Huanming

Kristiansen, Karsten

Roy, Bhaskar

Zhou, Yong

TI An esophageal squamous cell carcinoma classification system that reveals

potential targets for therapy

SO ONCOTARGET

LA English

DT Article

DE esophageal squamous cell carcinoma (ESCC); tumor microenvironment; gene

set enrichment analysis (GSEA); RNA expression

ID BREAST-CANCER METASTASIS; GENE-EXPRESSION; T-CELLS; KINASE;

IDENTIFICATION; PROGRESSION; SUBTYPES; GENOMICS; NETWORK; NUMBER

AB ESCC (Esophageal squamous cell carcinoma) is a heterogeneous cancer with diverse prognosis. Here, to explore the biological diversity of ESCC, we employed gene expression profiles from 360 ESCC tumors from East Asians to establish a comprehensive molecular classification and characterization of ESCC. Using the specific 185-gene signature generated by unsupervised consensus clustering of gene expression data, we defined four subtypes associated with distinct clinical metrics: tumors with high metastasis associated with EMT (epithelial to mesenchymal transition) and active MAP4K4/JNK signaling pathway; tumors with high chromosomal instability with up regulated MYC targes; well differentiated tumors with less aggressive and moderated tumors. The clinical relevance of these subtypes was stated by significant differences in prognosis. Importantly, 24% of all ESCCs (n = 360) were classified into the high metastasis subtype associated with poorly differentiation and unfavorable prognosis. We provided evidence that this subtype relates to tumor microenvironment. Collectively, these results might contribute to more precise personalized therapeutic strategies for each subtype of ESCC patients in the near future.

C1 [Xiong, Teng; Wang, Mengyao] Univ Chinese Acad Sci, BGI Educ Ctr, Shenzhen, Peoples R China.

[Xiong, Teng; Wang, Mengyao; Zhao, Jing; Yang, Chao; Luo, Wen; Li, Xiangchun; Yang, Huanming; Kristiansen, Karsten; Roy, Bhaskar; Zhou, Yong] BGI Shenzhen, Shenzhen, Peoples R China.

[Zhao, Jing; Kristiansen, Karsten] Univ Copenhagen, Dept Biol, Copenhagen, Denmark.

[Liu, Qing] Xi An Jiao Tong Univ, Key Lab, Coll Forens Sci, Minist Publ Hlth Forens Sci, Xian, Shaanxi, Peoples R China.

[Yang, Huanming] James D Watson Inst Genome Sci, Hangzhou, Zhejiang, Peoples R China.

RP Roy, B; Zhou, Y (corresponding author), BGI Shenzhen, Shenzhen, Peoples R China.

EM roy@genomics.cn; zhouyong@genomics.cn

RI Kristiansen, Karsten/J-5148-2014

OI Kristiansen, Karsten/0000-0002-6024-0917

FU Natural Science Foundation of China [81672818]; Science Technology and

Innovation Commmittee of Shenzhen municipality [JCYA 20160331190123578]

FX This work was financially supported by the Natural Science Foundation of

China (81672818) and Science Technology and Innovation Commmittee of

Shenzhen municipality under grant No. JCYA 20160331190123578.

CR Bandla S, 2012, ANN THORAC SURG, V93, P1101, DOI 10.1016/j.athoracsur.2012.01.064

Benito M, 2004, BIOINFORMATICS, V20, P105, DOI 10.1093/bioinformatics/btg385

Chen JQ, 2011, CANCER CELL, V19, P541, DOI 10.1016/j.ccr.2011.02.006

Chen Q, 2011, CANCER CELL, V20, P538, DOI 10.1016/j.ccr.2011.08.025

Collins CS, 2006, P NATL ACAD SCI USA, V103, P3775, DOI 10.1073/pnas.0600040103

Dang CV, 2006, SEMIN CANCER BIOL, V16, P253, DOI 10.1016/j.semcancer.2006.07.014

Gentles AJ, 2015, NAT MED, V21, P938, DOI 10.1038/nm.3909

Green MR., 2016, NAT METHODS, V21, P193, DOI [10.1016/j.molmed.2014.11.008.Mitochondria, DOI 10.1016/J.MOLMED.2014.11.008.MITOCHONDRIA]

Groger CJ, 2012, PLOS ONE, V7, DOI 10.1371/journal.pone.0051136

Gupta GP, 2006, CELL, V127, P679, DOI 10.1016/j.cell.2006.11.001

Harada K, 2016, J GASTROENTEROL, V51, P520, DOI 10.1007/s00535-016-1161-9

Herskovic A, 2012, ANN ONCOL, V23, P1095, DOI 10.1093/annonc/mdr433

Hu N, 2015, BMC GENOMICS, V16, DOI 10.1186/s12864-015-1919-0

Hu N, 2010, BMC GENOMICS, V11, DOI 10.1186/1471-2164-11-576

Hudson TJ, 2010, NATURE, V464, P993, DOI 10.1038/nature08987

Huh SJ, 2010, CANCER RES, V70, P6071, DOI 10.1158/0008-5472.CAN-09-4442

Isella C, 2015, NAT GENET, V47, P312, DOI 10.1038/ng.3224

Kamangar F, 2006, J CLIN ONCOL, V24, P2137, DOI 10.1200/JCO.2005.05.2308

Kim J, 2017, NATURE, V541, P169, DOI 10.1038/nature20805

Kitamura T, 2015, NAT REV IMMUNOL, V15, P73, DOI 10.1038/nri3789

Lee HW, 2013, BBA-REV CANCER, V1835, P170, DOI 10.1016/j.bbcan.2012.12.007

Li JG, 2014, GUT, V63, P1700, DOI 10.1136/gutjnl-2013-305806

Liu W., 2016, ADV MATER SCI ENG, V2016, P1, DOI DOI 10.1109/ECCE.2016.7855083

Machida N, 2004, J BIOL CHEM, V279, P15711, DOI 10.1074/jbc.C300542200

McCall MN, 2011, NUCLEIC ACIDS RES, V39, pD1011, DOI 10.1093/nar/gkq1259

McCall MN, 2010, BIOSTATISTICS, V11, P242, DOI 10.1093/biostatistics/kxp059

McDermott U, 2007, P NATL ACAD SCI USA, V104, P19936, DOI 10.1073/pnas.0707498104

Miller JA, 2011, BMC BIOINFORMATICS, V12, DOI 10.1186/1471-2105-12-322

Novitskiy SV, 2011, CANCER DISCOV, V1, P430, DOI 10.1158/2159-8290.CD-11-0100

Olkhanud PB, 2011, CANCER RES, V71, P3505, DOI 10.1158/0008-5472.CAN-10-4316

Polyak K, 2011, J CLIN INVEST, V121, P3786, DOI 10.1172/JCI60534

Roth MJ, 1997, CANCER-AM CANCER SOC, V80, P2047, DOI 10.1002/(SICI)1097-0142(19971201)80:11<2047::AID-CNCR3>3.0.CO;2-U

Sawada G, 2015, PLOS ONE, V10, DOI 10.1371/journal.pone.0139808

Sebastiani P, 2003, MACH LEARN, V52, P91

Stevenson RP, 2012, J CELL SCI, V125, P1073, DOI 10.1242/jcs.093799

Su H, 2011, CLIN CANCER RES, V17, P2955, DOI 10.1158/1078-0432.CCR-10-2724

Subramanian A, 2005, P NATL ACAD SCI USA, V102, P15545, DOI 10.1073/pnas.0506580102

Tan MCB, 2009, J IMMUNOL, V182, P1746, DOI 10.4049/jimmunol.182.3.1746

Tibshirani R, 2002, P NATL ACAD SCI USA, V99, P6567, DOI 10.1073/pnas.082099299

Tibshirani R, 2001, J ROY STAT SOC B, V63, P411, DOI 10.1111/1467-9868.00293

Tothill RW, 2008, CLIN CANCER RES, V14, P5198, DOI 10.1158/1078-0432.CCR-08-0196

Tusher VG, 2001, P NATL ACAD SCI USA, V98, P5116, DOI 10.1073/pnas.091062498

Verhaak RGW, 2010, CANCER CELL, V17, P98, DOI 10.1016/j.ccr.2009.12.020

Wen J, 2014, ANN ONCOL, V25, P1769, DOI 10.1093/annonc/mdu201

Yan Wusheng, 2012, BMC Res Notes, V5, P73, DOI 10.1186/1756-0500-5-73

Yang S, 2016, ONCOTARGET, V7, P85728, DOI 10.18632/oncotarget.13828

Yao J, 2016, INT J ONCOL, V48, P2543, DOI 10.3892/ijo.2016.3457

Yoshihara K, 2013, NAT COMMUN, V4, DOI 10.1038/ncomms3612

NR 48

TC 12

Z9 14

U1 1

U2 8

PU IMPACT JOURNALS LLC

PI ORCHARD PARK

PA 6666 E QUAKER ST, STE 1, ORCHARD PARK, NY 14127 USA

EI 1949-2553

J9 ONCOTARGET

JI Oncotarget

PD JUL 25

PY 2017

VL 8

IS 30

BP 49851

EP 49860

DI 10.18632/oncotarget.17989

PG 10

WC Oncology; Cell Biology

WE Science Citation Index Expanded (SCI-EXPANDED)

SC Oncology; Cell Biology

GA FB6DV

UT WOS:000406232400111

PM 28591712

OA Green Submitted, Green Published, gold

DA 2022-05-19

ER

PT J

AU Chang, WL

Wang, WL

Chung, TJ

Lin, FC

Yen, CJ

Lai, WW

Yang, HB

Sheu, BS

AF Chang, Wei-Lun

Wang, Wen-Lun

Chung, Ta-Jung

Lin, Forn-Chia

Yen, Chia-Jui

Lai, Wu-Wei

Yang, Hsiao-Bai

Sheu, Bor-Shyang

TI Response evaluation with endoscopic ultrasound and computed tomography

in esophageal squamous cell carcinoma treated by definitive

chemoradiotherapy

SO JOURNAL OF GASTROENTEROLOGY AND HEPATOLOGY

LA English

DT Article

DE definitive chemoradiotherapy; esophageal squamous cell carcinoma;

miniprobe endoscopic ultrasound (EUS); prognostic factors; response

evaluation

ID POSITRON-EMISSION-TOMOGRAPHY; NEOADJUVANT CHEMORADIATION; CANCER

INCIDENCE; TUMOR RESPONSE; FDG-PET; THERAPY; CHEMOTHERAPY; SURVIVAL;

EUS; CT

AB Background and AimWe assessed the feasibility of combined endoscopic ultrasound and computed tomography on response evaluation in patients with esophageal squamous cell carcinoma treated by definitive chemoradiotherapy, and the impact of response on prognosis.

MethodsSixty patients treated by definitive chemoradiotherapy were followed by miniprobe endoscopic ultrasound and computed tomography. The post-treatment esophageal wall thickness was measured by miniprobe endoscopic ultrasound. Metastatic tumors were evaluated by computed tomography. The correlation between post-treatment image findings and prognosis was evaluated.

ResultsTwenty-four patients (40%) had esophageal stricture after chemoradiotherapy, which limited complete evaluation by endoscopy. Miniprobe successfully penetrated all strictures to measure post-treatment esophageal wall thickness. Both post-treatment esophageal wall thickness <8mm measured by endoscopic ultrasound and no enlargement of metastatic tumor foci on computed tomography predicted good prognosis (P=0.001). Combined evaluation with these two modalities improved survival prediction (P<0.001). Patients who met the above two criteria after chemoradiotherapy had the longest survival compared with those who met only one or none of the criteria. The corresponding median survivals were >30 months, 16.8 months and 7.1 months, respectively (P<0.001). On multivariate analysis, treatment response is the strongest independent prognostic factor (hazard ratio 3.65, P=0.006) regardless of baseline tumor-node-metastasis staging and chemoradiation regimen.

ConclusionsResponse evaluation by miniprobe endoscopic ultrasound and computed tomography can predict the prognosis of esophageal squamous cell carcinoma patients treated by definitive chemoradiotherapy. Those who were judged as poor responder should receive additional treatment to improve outcome.

C1 [Chang, Wei-Lun; Wang, Wen-Lun; Lin, Forn-Chia; Sheu, Bor-Shyang] Natl Cheng Kung Univ, Coll Med, Inst Clin Med, Natl Cheng Kung Univ Hosp, Tainan 70101, Taiwan.

[Chang, Wei-Lun; Yen, Chia-Jui; Sheu, Bor-Shyang] Natl Cheng Kung Univ, Coll Med, Dept Internal Med, Natl Cheng Kung Univ Hosp, Tainan 70101, Taiwan.

[Chung, Ta-Jung] Natl Cheng Kung Univ, Coll Med, Dept Diagnost Radiol, Natl Cheng Kung Univ Hosp, Tainan 70101, Taiwan.

[Lin, Forn-Chia] Natl Cheng Kung Univ, Coll Med, Dept Radiat Oncol, Natl Cheng Kung Univ Hosp, Tainan 70101, Taiwan.

[Lai, Wu-Wei] Natl Cheng Kung Univ, Coll Med, Dept Surg, Natl Cheng Kung Univ Hosp, Tainan 70101, Taiwan.

[Wang, Wen-Lun] I Shou Univ, Dept Internal Med, E Da Hosp, Kaohsiung, Taiwan.

[Yang, Hsiao-Bai] Ton Yen Gen Hosp, Dept Pathol, Hsinchu, Taiwan.

RP Sheu, BS (corresponding author), Natl Cheng Kung Univ, Coll Med, Dept Internal Med, Natl Cheng Kung Univ Hosp, 138 Sheng Li Rd, Tainan 70101, Taiwan.

EM sheubs@mail.ncku.edu.tw

RI , 賴吾為/T-8615-2019; Wang, Wen-Lun/AAJ-1281-2020

OI , 賴吾為/0000-0001-9174-8310;

FU National Science Council [NSC 99-2314-B-006-045-MY2]; Department of

Health [DOH101-TD-PB-111-TM003]; National Cheng Kung University

Hospital, Taiwan [NCKUH-10204004]

FX This study was supported by grants from National Science Council (NSC

99-2314-B-006-045-MY2), Department of Health (DOH101-TD-PB-111-TM003),

and National Cheng Kung University Hospital (NCKUH-10204004), Taiwan.

CR Bedenne L, 2007, J CLIN ONCOL, V25, P1160, DOI 10.1200/JCO.2005.04.7118

Bowrey DF, 1999, J GASTROINTEST SURG, V3, P462, DOI 10.1016/S1091-255X(99)80098-5

Cerfolio RJ, 2005, J THORAC CARDIOV SUR, V129, P1232, DOI 10.1016/j.jtcvs.2004.12.042

Chak A, 2000, CANCER, V88, P1788

Chang WL, 2011, DIS ESOPHAGUS, V24, P590, DOI 10.1111/j.1442-2050.2011.01195.x

Enzinger PC, 2003, NEW ENGL J MED, V349, P2241, DOI 10.1056/NEJMra035010

Ferlay J, 2010, INT J CANCER, V127, P2893, DOI 10.1002/ijc.25516

Giovannini M, 1999, ITAL J GASTROENTEROL, V31, P593

Health Promotion Administration Minitry of Health and Welfare Executive Yuan Taiwan, 2013, TAIW CANC REG ANN RE

Isenberg G, 1998, GASTROINTEST ENDOSC, V48, P158, DOI 10.1016/S0016-5107(98)70157-9

Jemal A, 2010, CANCER EPIDEM BIOMAR, V19, P1893, DOI 10.1158/1055-9965.EPI-10-0437

Kamangar F, 2006, J CLIN ONCOL, V24, P2137, DOI 10.1200/JCO.2005.05.2308

Lu CL, 2010, CANCER CAUSE CONTROL, V21, P269, DOI 10.1007/s10552-009-9458-0

Mesenas S, 2008, DIS ESOPHAGUS, V21, P37, DOI 10.1111/j.1442-2050.2007.00731.x

Miyata H, 2011, ANN SURG, V253, P279, DOI 10.1097/SLA.0b013e318206824f

Monjazeb AM, 2010, J CLIN ONCOL, V28, P4714, DOI 10.1200/JCO.2010.30.7702

NOUSBAUM JB, 1992, GUT, V33, P1459, DOI 10.1136/gut.33.11.1459

Ota M, 2005, DIG ENDOSC, V17, P59, DOI DOI 10.1111/J.1443-1661.2005.00458.X

Owaki T, 2012, AM J SURG, V203, P191, DOI 10.1016/j.amjsurg.2011.01.027

Ribeiro A, 2006, AM J GASTROENTEROL, V101, P1216, DOI 10.1111/j.1572-0241.2006.00692.x

Sarkaria IS, 2009, ANN SURG, V249, P764, DOI 10.1097/SLA.0b013e3181a38e9e

Schwartz LH, 2006, ANN ONCOL, V17, P1018, DOI 10.1093/annonc/mdl058

Stahl M, 2005, J CLIN ONCOL, V23, P2310, DOI 10.1200/JCO.2005.00.034

Swisher SG, 2004, ANN THORAC SURG, V78, P1152, DOI 10.1016/j.athoracsur.2004.04.046

van Hagen P, 2012, NEW ENGL J MED, V366, P2074, DOI 10.1056/NEJMoa1112088

Westerterp M, 2005, RADIOLOGY, V236, P841, DOI 10.1148/radiol.2363041042

Willis J, 2002, GASTROINTEST ENDOSC, V55, P655, DOI 10.1067/mge.2002.123273

NR 27

TC 7

Z9 8

U1 0

U2 5

PU WILEY-BLACKWELL

PI HOBOKEN

PA 111 RIVER ST, HOBOKEN 07030-5774, NJ USA

SN 0815-9319

EI 1440-1746

J9 J GASTROEN HEPATOL

JI J. Gastroenterol. Hepatol.

PD MAR

PY 2015

VL 30

IS 3

BP 463

EP 469

DI 10.1111/jgh.12683

PG 7

WC Gastroenterology & Hepatology

WE Science Citation Index Expanded (SCI-EXPANDED)

SC Gastroenterology & Hepatology

GA CC1YE

UT WOS:000350140500010

PM 25088453

DA 2022-05-19

ER

PT J

AU Shen, SS

Zhang, XQ

Li, ZL

Zou, XP

Ling, TS

AF Shen, Shan-Shan

Zhang, Xiao-Qi

Li, Zhen-Lei

Zou, Xiao-Ping

Ling, Ting-Sheng

TI Over-the-scope clip to close a gastrocutaneous fistula after

esophagectomy

SO WORLD JOURNAL OF GASTROENTEROLOGY

LA English

DT Article

DE Over-the-scope clip; Gastrocutaneous fistula; Digestive endoscopy;

Esophageal squamous cell carcinoma

AB Over-the-scope clip (OTSC) system is becoming a new reliable technique which is available for the endoscopic closure of fistulas, bleeding, perforations and so on. We describe the case of a patient with a non-healing gastrocutaneous fistula after esophagectomy for esophageal squamous cell carcinoma which was successfully closed using an OTSC system. This is the first report of the use of OTSC to treat a non-healing gastrocutaneous fistula successfully after esophagectomy. We believe our experience will give such patients an ideal way to cure the fistula without suffering too much and also explore new application of OTSC.

C1 [Shen, Shan-Shan; Zhang, Xiao-Qi; Li, Zhen-Lei; Zou, Xiao-Ping; Ling, Ting-Sheng] Nanjing Univ, Sch Med, Affiliated Drum Tower Hosp, Dept Gastroenterol, Nanjing 210008, Jiangsu, Peoples R China.

RP Ling, TS (corresponding author), Nanjing Univ, Sch Med, Affiliated Drum Tower Hosp, Dept Gastroenterol, 321 Zhongshan Rd, Nanjing 210008, Jiangsu, Peoples R China.

EM chinalts@126.com

FU National Natural Science Foundation of China [81201908]

FX Supported by National Natural Science Foundation of China, No. 81201908.

CR Aly A, 2013, J GASTROINTEST SURG, V17, P606, DOI 10.1007/s11605-012-2062-8

Donatelli G, 2014, ENDOSCOPY, V46, pE128, DOI 10.1055/s-0034-1364889

Kumar Nitin, 2013, Gastrointest Endosc Clin N Am, V23, P123, DOI 10.1016/j.giec.2012.10.002

Nishiyama N, 2013, WORLD J GASTROENTERO, V19, P2752, DOI 10.3748/wjg.v19.i18.2752

Sandmann M, 2011, Z GASTROENTEROL, V49, P981, DOI 10.1055/s-0029-1245972

NR 5

TC 2

Z9 2

U1 1

U2 2

PU BAISHIDENG PUBLISHING GROUP INC

PI PLEASANTON

PA 8226 REGENCY DR, PLEASANTON, CA 94588 USA

SN 1007-9327

EI 2219-2840

J9 WORLD J GASTROENTERO

JI World J. Gastroenterol.

PD DEC 21

PY 2015

VL 21

IS 47

BP 13396

EP 13399

DI 10.3748/wjg.v21.i47.13396

PG 4

WC Gastroenterology & Hepatology

WE Science Citation Index Expanded (SCI-EXPANDED)

SC Gastroenterology & Hepatology

GA CZ1TZ

UT WOS:000366889600023

PM 26715825

OA Green Published, hybrid

DA 2022-05-19

ER

PT J

AU Liu, Q

Chen, JQ

Li, BS

Ye, JJ

Wei, SH

Wang, YH

Yang, HJ

Zhu, ZF

Lai, ST

Li, L

Chen, Y

Wang, JL

Xiang, JQ

Zhao, KL

AF Liu, Qi

Chen, Junqiang

Li, Baosheng

Ye, Jinjun

Wei, Shihong

Wang, Yaohui

Yang, Huanjun

Zhu, Zhengfei

Lai, Songtao

Li, Ling

Chen, Yun

Wang, Jialei

Xiang, Jiaqing

Zhao, Kuaile

TI Local therapy for oligometastatic esophageal squamous cell carcinoma: a

prospective, randomized, Phase II clinical trial

SO FUTURE ONCOLOGY

LA English

DT Article

DE esophageal squamous cell carcinoma; local therapy; oligometastasis

AB For patients with oligometastatic esophageal squamous cell carcinoma, the efficacy of local therapy is still controversial because of patient selection and lack of adequate controls in most studies. Here the authors design the ESO-Shanghai 13 trial, a prospective, multicenter, randomized, Phase II trial, to assess the impact of combined local therapy and systemic therapy on progression and survival compared with systemic therapy alone for patients with four or less metastases. A total of 102 patients will be recruited over 3 years from approximately five centers and randomized in a 1:1 ratio to receive either systemic therapy alone or systemic therapy and local therapy, such as radiation, surgery and thermal ablation. The primary endpoint is progression-free survival. The secondary endpoints are overall survival, local control, toxicity and quality of life.

Lay abstract: The ESO-Shanghai 13 trial is a prospective, multicenter, randomized, Phase II trial to assess the impact of combined local treatment (such as radiotherapy, surgery and thermal ablation) and chemical drugs for patients with esophageal squamous cell carcinoma. Patients with four or less metastases and controlled esophageal lesion will be enrolled. The authors will recruit a total of 102 patients over 3 years from approximately five centers. All patients will be randomized and receive either chemical drugs alone or chemical drugs plus local treatment with the same probability. Patients will then be observed after treatment until disease progression or death or the end of the trial. Patients will need to report their symptoms and physical status and fill out quality of life scales during the treatment and follow-up period.

C1 [Liu, Qi; Yang, Huanjun; Zhu, Zhengfei; Lai, Songtao; Li, Ling; Chen, Yun; Zhao, Kuaile] Fudan Univ, Shanghai Canc Ctr, Dept Radiat Oncol, 270 Dongan Rd, Shanghai 200032, Peoples R China.

[Liu, Qi; Wang, Yaohui; Yang, Huanjun; Zhu, Zhengfei; Lai, Songtao; Li, Ling; Chen, Yun; Wang, Jialei; Xiang, Jiaqing; Zhao, Kuaile] Fudan Univ, Shanghai Med Coll, Dept Oncol, Shanghai 200032, Peoples R China.

[Chen, Junqiang] Fujian Med Univ, Fujian Prov Canc Hosp, Teaching Hosp, Dept Radiat Oncol, Fuzhou 350011, Peoples R China.

[Li, Baosheng] Shangdong Canc Hosp, Dept Radiat Oncol, Jinan 250117, Peoples R China.

[Ye, Jinjun] Jiangsu Canc Hosp, Dept Radiat Oncol, Nanjing 210009, Peoples R China.

[Wei, Shihong] Gansu Prov Canc Hosp, Dept Radiat Oncol, Lanzhou 730050, Peoples R China.

[Wang, Yaohui] Fudan Univ, Shanghai Canc Ctr, Dept Intervent Therapy, Shanghai 200032, Peoples R China.

[Wang, Jialei] Fudan Univ, Dept Med Oncol, Shanghai Canc Ctr, Shanghai 200032, Peoples R China.

[Xiang, Jiaqing] Fudan Univ, Dept Thorac Surg, Shanghai Canc Ctr, Shanghai 200032, Peoples R China.

RP Zhao, KL (corresponding author), Fudan Univ, Shanghai Canc Ctr, Dept Radiat Oncol, 270 Dongan Rd, Shanghai 200032, Peoples R China.; Zhao, KL (corresponding author), Fudan Univ, Shanghai Med Coll, Dept Oncol, Shanghai 200032, Peoples R China.

EM kuaile_z@sina.com

FU Medical Innovation Research Foundation of Shanghai Science and

Technology Committee [20Y11908700]

FX The research was supported by the Medical Innovation Research Foundation

of Shanghai Science and Technology Committee (no. 20Y11908700). The

authors have no other relevant affiliations or financial involvement

with any organization or entity with a financial interest in or

financial conflict with the subject matter or materials discussed in the

manuscript apart from those disclosed.

CR AARONSON NK, 1993, J NATL CANCER I, V85, P365, DOI 10.1093/jnci/85.5.365

Benedict SH, 2010, MED PHYS, V37, P4078, DOI 10.1118/1.3438081

Blazeby JM, 2003, EUR J CANCER, V39, P1384, DOI 10.1016/S0959-8049(03)00270-3

Chen WQ, 2016, CA-CANCER J CLIN, V66, P115, DOI 10.3322/caac.21338

Gomez DR, 2019, J CLIN ONCOL, V37, DOI 10.1200/JCO.19.00201

Huang J, 2020, LANCET ONCOL, V21, P832, DOI 10.1016/S1470-2045(20)30110-8

Ichida H, 2013, WORLD J SURG, V37, P398, DOI 10.1007/s00268-012-1830-7

Iyengar P, 2018, JAMA ONCOL, V4, DOI 10.1001/jamaoncol.2017.3501

Kanamori J, 2017, JPN J CLIN ONCOL, V47, P25, DOI 10.1093/jjco/hyw141

Kato K, 2019, LANCET ONCOL, V20, P1506, DOI 10.1016/S1470-2045(19)30626-6

Kobayashi N, 2014, ANN SURG ONCOL, V21, P365, DOI 10.1245/s10434-014-3677-7

Kojima T, 2020, J CLIN ONCOL, V38, DOI 10.1200/JCO.20.01888

Liu Q, 2020, INT J RADIAT ONCOL, V108, P707, DOI 10.1016/j.ijrobp.2020.05.003

National Comprehensive Cancer Network, 2020, ESOPHAGEAL ESOPHAGOG

Palma DA, 2019, LANCET, V393, P2051, DOI 10.1016/S0140-6736(18)32487-5

Parkin DM, 2005, CA-CANCER J CLIN, V55, P74, DOI 10.3322/canjclin.55.2.74

POCOCK SJ, 1975, BIOMETRICS, V31, P103, DOI 10.2307/2529712

Timmerman RD, 2008, SEMIN RADIAT ONCOL, V18, P215, DOI 10.1016/j.semradonc.2008.04.001

NR 18

TC 0

Z9 0

U1 1

U2 1

PU FUTURE MEDICINE LTD

PI LONDON

PA UNITEC HOUSE, 3RD FLOOR, 2 ALBERT PLACE, FINCHLEY CENTRAL, LONDON, N3

1QB, ENGLAND

SN 1479-6694

EI 1744-8301

J9 FUTURE ONCOL

JI Future Oncol.

PD APR

PY 2021

VL 17

IS 11

BP 1285

EP 1293

DI 10.2217/fon-2020-0873

EA FEB 2021

PG 9

WC Oncology

WE Science Citation Index Expanded (SCI-EXPANDED)

SC Oncology

GA QZ1HJ

UT WOS:000621387800001

PM 33626929

OA hybrid

DA 2022-05-19

ER

PT J

AU Liu, YT

Wang, XJ

Jiang, XS

Yan, PW

Zhan, LL

Zhu, HF

Wang, TT

Wen, J

AF Liu, Yatian

Wang, Xiaojun

Jiang, Xuesong

Yan, Pengwei

Zhan, Liangliang

Zhu, Huanfeng

Wang, Tingting

Wen, Jing

TI Tumor-suppressive microRNA-10a inhibits cell proliferation and

metastasis by targeting Tiam1 in esophageal squamous cell carcinoma

SO JOURNAL OF CELLULAR BIOCHEMISTRY

LA English

DT Article

DE esophageal squamous cell carcinoma (ESCC); metastasis; miR-10a;

proliferation; Tiam1

ID EPITHELIAL-MESENCHYMAL TRANSITION; INVASION; EXPRESSION; PROMOTES; GENE;

RAC; OVEREXPRESSION; MIGRATION

AB Aberrant microRNAs (miRNAs) expressions could contribute to the progression of numerous cancers, including esophageal squamous cell carcinoma, while miR-10a participates in multiple biological processes on cancers. However, the molecular mechanism of miR-10a in esophageal squamous cell carcinoma (ESCC) has not been investigated. Herein, miR-10a was significantly reduced in ESCC clinical tissues and ESCC cell lines (EC109 and TE-3). In addition, immunohistochemistry indicated that the expressions of alpha-SMA, Ki-67, and PCNA in tumor tissues were higher than that of controls. In vitro, overexpression of miR-10a dramatically suppressed cell proliferation and enhanced cell apoptosis, while the decrease of miR-10a expressed the opposite outcome. Specially, overexpression of miR-10a caused a G0/G1 peak accumulation. Moreover, miR-10a also negatively regulated ESCC cell migration and invasion. Furthermore, targetscan bioinformatics predictions and the dual-luciferase assay confirmed that Tiam1 was a direct target gene of miR-10a. The statistical analysis showed Tiam1 was negatively in correlation with miR-10a in ESCC patient samples. And silencing Tiam1 could lead to a decline on cell growth, invasion, and migration in ESCC cell lines, while it could enhance cell apoptosis and cause a G0/G1 peak accumulation. In vivo, it revealed that miR-10a notably decreased the tumor growth and metastasis in xenograft model and pulmonary metastasis model. And it showed a lower expressions of Tiam1 in the miR-10a mimics group by immunohistochemistry. Taken together the results, they indicated that miR-10a might function as a novel tumor suppressor in vitro and in vivo via targeting Tiam1, suggesting miR-10a to be a candidate biomarker for the ESCC therapy.

C1 [Liu, Yatian; Jiang, Xuesong; Yan, Pengwei; Zhan, Liangliang; Zhu, Huanfeng; Wang, Tingting; Wen, Jing] Nanjing Med Univ, Jiangsu Canc Hosp, Dept Radiotherapy, Nanjing, Jiangsu, Peoples R China.

[Liu, Yatian; Wang, Xiaojun; Jiang, Xuesong; Yan, Pengwei; Zhan, Liangliang; Zhu, Huanfeng; Wang, Tingting; Wen, Jing] Nanjing Med Univ, Jiangsu Inst Canc Res, Affiliated Canc Hosp, Nanjing, Jiangsu, Peoples R China.

[Wang, Xiaojun] Nanjing Med Univ, Jiangsu Canc Hosp, Dept Thorac Surg, Nanjing, Jiangsu, Peoples R China.

RP Wen, J (corresponding author), Nanjing Med Univ, Jiangsu Canc Hosp, Dept Radiotherapy, Jiangsu Inst Canc Res, Nanjing 210009, Jiangsu, Peoples R China.; Wen, J (corresponding author), Nanjing Med Univ, Affiliated Canc Hosp, Nanjing 210009, Jiangsu, Peoples R China.

EM wenjing201711@163.com

RI wang, tingting/AAK-2640-2020

FU Jiangsu provincial health and Family Planning Commission Project

[H2017036]

FX Jiangsu provincial health and Family Planning Commission Project,

Grant/Award Number: H2017036

CR Abbas G, 2017, ANN CARDIOTHORAC SUR, V6, P131, DOI 10.21037/acs.2017.03.03

Abell AN, 2004, J CELL SCI, V117, P243, DOI 10.1242/jcs.00853

Agirre X, 2008, MOL CANCER RES, V6, P1830, DOI 10.1158/1541-7786.MCR-08-0167

Buchsbaum RJ, 2002, MOL CELL BIOL, V22, P4073, DOI 10.1128/MCB.22.12.4073-4085.2002

CHEN HM, 1995, GENOMICS, V30, P123, DOI 10.1006/geno.1995.0025

Croce CM, 2009, NAT REV GENET, V10, P704, DOI 10.1038/nrg2634

Crompton AM, 2000, J BIOL CHEM, V275, P25751, DOI 10.1074/jbc.M002050200

Engers R, 2006, BRIT J CANCER, V95, P1081, DOI 10.1038/sj.bjc.6603385

Fan Q, 2016, PROTEIN CELL, V7, P899, DOI 10.1007/s13238-016-0316-z

Fareed KR, 2009, GUT, V58, P127, DOI 10.1136/gut.2008.155861

Feber A, 2008, J THORAC CARDIOV SUR, V135, P255, DOI 10.1016/j.jtcvs.2007.08.055

Guo XJ, 2013, MOL CANCER RES, V11, P230, DOI 10.1158/1541-7786.MCR-12-0632

Gurtan AM, 2013, J MOL BIOL, V425, P3582, DOI 10.1016/j.jmb.2013.03.007

HABETS GGM, 1995, CYTOGENET CELL GENET, V70, P48, DOI 10.1159/000133989

Hou M, 2004, ACTA BIOCH BIOPH SIN, V36, P537, DOI 10.1093/abbs/36.8.537

Jia HY, 2014, PLOS ONE, V9, DOI 10.1371/journal.pone.0088057

Li ZL, 2016, BMC CANCER, V16, DOI 10.1186/s12885-016-2724-0

Liu SG, 2014, DIAGN PATHOL, V9, DOI 10.1186/1746-1596-9-69

Liu Y, 2017, CELL DEATH DIS, V8, pe2379

Long MJ, 2012, CANCER LETT, V324, P186, DOI 10.1016/j.canlet.2012.05.022

Mathe EA, 2009, CLIN CANCER RES, V15, P6192, DOI 10.1158/1078-0432.CCR-09-1467

Matsushima K, 2010, DIGESTION, V82, P138, DOI 10.1159/000310918

Mertens AE, 2003, FEBS LETT, V546, P11, DOI 10.1016/S0014-5793(03)00435-6

MICHIELS F, 1995, NATURE, V375, P338, DOI 10.1038/375338a0

Mu HQ, 2019, J CELL BIOCHEM, V120, P4987, DOI 10.1002/jcb.27774

Otsuki Y, 2001, P NATL ACAD SCI USA, V98, P4385, DOI 10.1073/pnas.071411598

Qi HW, 2001, CELL GROWTH DIFFER, V12, P603

Sanmartin E, 2017, ONCOTARGET, V8, P45286, DOI 10.18632/oncotarget.16787

Song YM, 2014, NATURE, V509, P91, DOI 10.1038/nature13176

Stiles BM, 2012, EUR J CARDIO-THORAC, V42, P659, DOI 10.1093/ejcts/ezs105

Wang S, 2015, ONCOL REP, V33, P1807, DOI 10.3892/or.2015.3785

Whalley HJ, 2015, NAT COMMUN, V6, DOI 10.1038/ncomms8437

Yan Y, 2015, FEBS LETT, V589, P756, DOI 10.1016/j.febslet.2015.02.005

Yan Y, 2013, HEPATOLOGY, V57, P667, DOI 10.1002/hep.26071

Yang MH, 2014, J TRANSL MED, V12, DOI 10.1186/1479-5876-12-109

Zhu M, 2014, FEBS LETT, V588, P600, DOI 10.1016/j.febslet.2013.12.028

NR 36

TC 7

Z9 8

U1 0

U2 5

PU WILEY

PI HOBOKEN

PA 111 RIVER ST, HOBOKEN 07030-5774, NJ USA

SN 0730-2312

EI 1097-4644

J9 J CELL BIOCHEM

JI J. Cell. Biochem.

PD MAY

PY 2019

VL 120

IS 5

BP 7845

EP 7857

DI 10.1002/jcb.28059

PG 13

WC Biochemistry & Molecular Biology; Cell Biology

WE Science Citation Index Expanded (SCI-EXPANDED)

SC Biochemistry & Molecular Biology; Cell Biology

GA HO6VK

UT WOS:000461070700101

PM 30426564

DA 2022-05-19

ER

PT J

AU Zheng, ST

Yang, CC

Lu, M

Liu, Q

Liu, T

Dai, F

Gao, XP

Sheyhidin, I

Lu, XM

AF Zheng, Shutao

Yang, Chenchen

Lu, Mang

Liu, Qing

Liu, Tao

Dai, Fang

Gao, Xiangpeng

Sheyhidin, Ilyar

Lu, Xiaomei

TI PIK3CA promotes proliferation and motility but is unassociated with

lymph node metastasis or prognosis in esophageal squamous cell carcinoma

SO HUMAN PATHOLOGY

LA English

DT Article

DE Esophageal squamous cell carcinoma; PIK3CA; Proliferation; Metastasis;

Motility; Prognosis

ID NORTHWESTERN CHINA; SAMPLE-SIZE; CANCER; AMPLIFICATION; EXPRESSION;

INVASION; PLACE; LEARN

AB The PIK3CA mutation has been extensively reported in the setting of cancers; however, the clinicopathological significance of PIK3CA expression has rarely been discussed in esophageal squamous cell carcinoma. In the present study, to confirm the significance of PIK3CA expression in association with metastasis and prognosis, which has been somewhat controversial in esophageal squamous cell carcinoma (ESCC), the relationship between clinicopathological features of ESCC and PIK3CA expression was analyzed using immunohistochemistry with a tissue microarray. Meanwhile, as additional verification and an ethnic control, another independent small cohort of Kazakh ESCC were analyzed by immunohistochemistry. To investigate the pilot role of PIK3CA in ESCC cells, ESCC cell lines ECa109 and EC9706 were transiently transfected with specific siRNA against PIK3CA. The silencing effect was detected by Western blot. Cell proliferation was examined using the 3-(4,5-dirnethyl-2-thiazolyl)-2,5diphenyl-2-H-tetrazolium bromide assay; apoptosis and the cell cycles were analyzed by flow cytometry. Furthermore, the migratory and invasive ability were evaluated by wound healing and transwell invasion assay, respectively. Expression of PIK3CA was significantly higher in ESCC than in paired normal controls and was ethnicity independent; no statistically significant difference was observed between PIK3CA expression and sex, age, depth of invasion, tumor differentiation, lymph node metastasis, or prognosis. Proliferation, migration, and invasion were all markedly reduced after knockout of PIK3CA. Moreover, the cell cycle was arrested at the S phase, and the apoptosis rate was significantly increased, suggesting that PIK3CA plays a key role in promoting the proliferation and motility of ESCC cells. (C) 2016 Elsevier Inc. All rights reserved.

C1 [Zheng, Shutao; Yang, Chenchen; Lu, Mang; Liu, Qing; Liu, Tao; Dai, Fang; Gao, Xiangpeng; Lu, Xiaomei] Xinjiang Med Univ, Affiliated Hosp 1, Clin Med Res Inst, Urumqi 830054, Xinjiang Uygur, Peoples R China.

[Zheng, Shutao; Yang, Chenchen; Lu, Mang; Liu, Qing; Liu, Tao; Dai, Fang; Gao, Xiangpeng; Sheyhidin, Ilyar; Lu, Xiaomei] Xinjiang Med Univ, Affiliated Hosp 1, State Key Lab Incubat Base Xinjiang Major Dis Res, Urumqi 830054, Xinjiang Uygur, Peoples R China.

RP Sheyhidin, I (corresponding author), Xinjiang Med Univ, Affiliated Hosp 1, State Key Lab Incubat Base Xinjiang Major Dis Res, Urumqi 830054, Xinjiang Uygur, Peoples R China.; Lu, XM (corresponding author), Xinjiang Med Univ, Affiliated Hosp 1, Clin Med Res Inst, State Key Lab Breeding Base Xinjiang Major Dis Re, Urumqi 830054, Xinjiang Uygur, Peoples R China.

EM ilyarsha@hotmail.com; luxiaomei88@163.com

OI Zheng, Shutao/0000-0002-9000-0148

FU Natural Science Foundation of China [81160303, 81260359, 81201891,

U1303321, 81360357]; Major Science and Technology Projects of the

Xinjiang Uygur Autonomous Region [201430123-1]; Xinjiang Key Laboratory

of Major Diseases [2014Y3, 2012-Y2]; Xinjiang Medical University

Scientific Research Innovation Foundation [201311]

FX This work was supported by the Natural Science Foundation of China (nos.

81160303, 81260359, 81201891, U1303321, and 81360357). Support also was

obtained from Major Science and Technology Projects of the Xinjiang

Uygur Autonomous Region (no. 201430123-1), Xinjiang Key Laboratory of

Major Diseases Grant (nos. 2014Y3 and 2012-Y2), and Xinjiang Medical

University Scientific Research Innovation Foundation (no. 201311).

CR Akagi I, 2009, INT J ONCOL, V34, P767, DOI 10.3892/ijo_00000202

Arnaud F, 2010, J VIROL, V84, P4415, DOI 10.1128/JVI.00029-10

Baker M, 2015, NATURE, V521, P274, DOI 10.1038/521274a

Burry RW, 2011, J HISTOCHEM CYTOCHEM, V59, P6, DOI 10.1369/jhc.2010.956920

German S, 2013, HERED CANCER CLIN PR, V11, DOI 10.1186/1897-4287-11-5

Helsby Matthew A, 2013, F1000Res, V2, P153, DOI 10.12688/f1000research.2-153.v1

Helsby Matthew A, 2014, F1000Res, V3, P241, DOI 10.12688/f1000research.5405.2

Huang WS, 2012, WORLD J GASTROENTERO, V18, P3458, DOI 10.3748/wjg.v18.i26.3458

Iijima Y, 2015, INT J ONCOL, V46, P505, DOI 10.3892/ijo.2014.2742

Kaplan RM, 2014, CTS-CLIN TRANSL SCI, V7, P342, DOI 10.1111/cts.12178

Kolasa IK, 2009, CANCER BIOL THER, V8, P21, DOI 10.4161/cbt.8.1.7209

Konopka B, 2011, HUM PATHOL, V42, P1710, DOI 10.1016/j.humpath.2010.01.030

Li J, 2013, CARCINOGENESIS, V34, P658, DOI 10.1093/carcin/bgs384

Lin DC, 2014, NAT GENET, V46, P467, DOI 10.1038/ng.2935

Linderoth J, 2007, EUR J HAEMATOL, V79, P146, DOI 10.1111/j.1600-0609.2007.00892.x

Matarrese P, 2010, MOL CANCER, V9, DOI 10.1186/1476-4598-9-207

Mees ST, 2011, J SURG RES, V171, P136, DOI 10.1016/j.jss.2010.03.034

Meier DT, 2015, J HISTOCHEM CYTOCHEM, V63, P663, DOI 10.1369/0022155415585995

Napier KJ, 2014, WORLD J GASTRO ONCOL, V6, P112, DOI 10.4251/wjgo.v6.i5.112

Samuels Y, 2011, CURR TOP MICROBIOL, V347, P21, DOI 10.1007/82_2010_68

Shi J, 2012, BMC CANCER, V12, DOI 10.1186/1471-2407-12-50

Smits AJJ, 2015, APPL IMMUNOHISTO M M, V23, P416, DOI 10.1097/PAI.0000000000000099

Song YM, 2014, NATURE, V509, P91, DOI 10.1038/nature13176

VOLINIA S, 1994, GENOMICS, V24, P472, DOI 10.1006/geno.1994.1655

Wada S, 2006, ANN SURG ONCOL, V13, P961, DOI 10.1245/ASO.2006.08.006

Wang L, 2014, PLOS ONE, V9, DOI 10.1371/journal.pone.0103021

Weiss T, 2010, EPIGENET CHROMATIN, V3, DOI 10.1186/1756-8935-3-7

Wu XH, 2012, J TRANSL MED, V10, DOI 10.1186/1479-5876-10-180

Yang CC, 2015, TRANSL RES, V166, P614, DOI 10.1016/j.trsl.2015.05.004

Zheng ST, 2011, EUR J GASTROEN HEPAT, V23, P1087, DOI 10.1097/MEG.0b013e32834a14d9

Zheng ST, 2010, EUR J GASTROEN HEPAT, V22, P917, DOI 10.1097/MEG.0b013e3283313d8b

Zhou XK, 2011, WORLD J GASTROENTERO, V17, P3700, DOI 10.3748/wjg.v17.i32.3700

NR 32

TC 7

Z9 7

U1 1

U2 7

PU W B SAUNDERS CO-ELSEVIER INC

PI PHILADELPHIA

PA 1600 JOHN F KENNEDY BOULEVARD, STE 1800, PHILADELPHIA, PA 19103-2899 USA

SN 0046-8177

EI 1532-8392

J9 HUM PATHOL

JI Hum. Pathol.

PD JUL

PY 2016

VL 53

BP 121

EP 129

DI 10.1016/j.humpath.2015.11.013

PG 9

WC Pathology

WE Science Citation Index Expanded (SCI-EXPANDED)

SC Pathology

GA DP7GW

UT WOS:000378668200016

PM 27001433

DA 2022-05-19

ER

PT J

AU Nobel, TB

Barbetta, A

Hsu, M

Tan, KS

Pinchinat, T

Schlottmann, F

Bains, MS

Ku, GY

Wu, AJ

Patti, MG

Jones, DR

Molena, D

AF Nobel, Tamar B.

Barbetta, Arianna

Hsu, Meier

Tan, Kay See

Pinchinat, Tiffany

Schlottmann, Francisco

Bains, Manjit S.

Ku, Geoffrey Y.

Wu, Abraham J.

Patti, Marco G.

Jones, David R.

Molena, Daniela

TI Outcomes of Radiation-Associated Esophageal Squamous Cell Carcinoma: The

MSKCC Experience

SO JOURNAL OF GASTROINTESTINAL SURGERY

LA English

DT Article; Proceedings Paper

CT Annual Meeting of the American-Society-for-Gastrointestinal-Endoscopy /

Digestive Disease Week (DDW) / 59th Annual Meeting of the

Society-for-Surgery-of-the-Alimentary-Tract (SSAT)

CY JUN 02-05, 2018

CL Washington, DC

SP Amer Soc Gastrointestinal Endoscopy, Soc Surg Alimentary Tract, Pancreas Club

DE Esophageal squamous cell carcinoma; Reirradiation; Treatment selection;

Neoadjuvant therapy

ID BREAST-CANCER; INCREASED RISK; THERAPY; RADIOTHERAPY; SURVIVORS; FIELD

AB ObjectiveEsophageal squamous cell carcinoma (ESCC-R) is a rarely encountered sequela of chest radiation. Treatment is limited by toxicity with reirradiation and complex surgical dissection in a previously radiated field. The clinical presentation, prognosis, and treatment selection of ESCC-R remain undefined.MethodsA retrospective review of patients with esophageal squamous cell carcinoma at a single institution between 2000 and 2017 was performed to identify patients with previous radiation therapy (5years delay). Clinicopathologic characteristics, treatment, and outcomes of ESCC-R (n=69) patients were compared to patients with primary esophageal squamous cell carcinoma (ESCC) (n=827). Overall survival (OS) and cumulative incidence of recurrence (CIR) were compared using log-rank and Gray's tests, respectively.ResultsMedian time from radiation to ESCC-R was 18.2years. The majority of ESCC-R patients werefemale and presented with earlier disease and decreased behavioral risk factors. ESCC-R treated with surgery alone had worse OS than ESCC (5-year 15 vs 33%; p=0.045). Patients with ESCC-R who received neoadjuvant treatment had higher risk of postoperative in-house mortality (16.7 vs 4.2%; p=0.032). Patients with ESCC-R treated with surgery alone and definitive chemoradiation had higher recurrence risk than those with neoadjuvant+surgery (5-year recurrence 55 and 45 vs 15%; p=0.101).ConclusionNeoadjuvant chemotherapy or chemoradiation should be used whenever possible for ESCC-R as it is associated with lower risk of recurrence. The improved survival benefits of aggressive treatment must be weighed against the higher associated postoperative risks.

C1 [Nobel, Tamar B.; Barbetta, Arianna; Bains, Manjit S.; Jones, David R.; Molena, Daniela] Mem Sloan Kettering Canc Ctr, Dept Surg, 1275 York Ave, New York, NY 10065 USA.

[Nobel, Tamar B.] Mt Sinai Hosp, Dept Surg, New York, NY 10029 USA.

[Hsu, Meier; Tan, Kay See] Mem Sloan Kettering Canc Ctr, Dept Epidemiol & Biostat, New York, NY 10021 USA.

[Pinchinat, Tiffany] Brookdale Univ Hosp, Dept Surg, Brooklyn, NY USA.

[Schlottmann, Francisco; Patti, Marco G.] Univ N Carolina, Dept Med & Surg, Chapel Hill, NC 27515 USA.

[Ku, Geoffrey Y.; Wu, Abraham J.] Mem Sloan Kettering Canc Ctr, Dept Oncol, 1275 York Ave, New York, NY 10021 USA.

RP Molena, D (corresponding author), Mem Sloan Kettering Canc Ctr, Dept Surg, 1275 York Ave, New York, NY 10065 USA.

EM molenad@mskcc.org

OI Nobel, Tamar/0000-0002-4519-3008; Jones, David/0000-0002-3318-0146

FU National Institutes of Health/National Cancer Institute Cancer Support

Grant [P30 CA008748]; American Cancer Society; NATIONAL CANCER INSTITUTE

[P30CA008748] Funding Source: NIH RePORTER

FX This study was supported, in part, by the National Institutes of

Health/National Cancer Institute Cancer Support Grant P30 CA008748.

Tamar Nobel is supported, in part, by a grant from the American Cancer

Society.

CR Ahsan H, 1998, ANN INTERN MED, V128, P114, DOI 10.7326/0003-4819-128-2-199801150-00007

Bledsoe TJ, 2017, CLIN CHEST MED, V38, P201, DOI 10.1016/j.ccm.2016.12.004

Bryant AK, 2017, CANCER EPIDEM BIOMAR, V26, P963, DOI 10.1158/1055-9965.EPI-16-1023

de Gonzalez AB, 2013, INT J RADIAT ONCOL, V86, P224, DOI 10.1016/j.ijrobp.2012.09.001

Farjah F, 2018, DEBBIES DREAM FDN CU

Grantzau T, 2015, RADIOTHER ONCOL, V114, P56, DOI 10.1016/j.radonc.2014.10.004

Hull MC, 2003, JAMA-J AM MED ASSOC, V290, P2831, DOI 10.1001/jama.290.21.2831

Markar SR, 2017, EUR J CANCER, V75, P179, DOI 10.1016/j.ejca.2016.12.036

Matheson JB, 2002, RADIOTHER ONCOL, V65, P159, DOI 10.1016/S0167-8140(02)00135-4

Miller KD, 2016, CA-CANCER J CLIN, V66, P271, DOI 10.3322/caac.21349

Morton LM, 2012, ANN ONCOL, V23, P3081, DOI 10.1093/annonc/mds144

Morton LM, 2014, HAEMATOLOGICA, V99, pE193, DOI 10.3324/haematol.2014.108258

Schena M, 2017, J THORAC DIS, V9, P1767, DOI 10.21037/jtd.2017.06.45

Scholl B, 2001, AM J SURG, V182, P476, DOI 10.1016/S0002-9610(01)00749-8

Stubblefield MD, 2017, MUSCLE NERVE, V56, P1031, DOI 10.1002/mus.25778

Zablotska LB, 2005, AM J EPIDEMIOL, V161, P330, DOI 10.1093/aje/kwi050

NR 16

TC 1

Z9 1

U1 0

U2 0

PU SPRINGER

PI NEW YORK

PA 233 SPRING ST, NEW YORK, NY 10013 USA

SN 1091-255X

EI 1873-4626

J9 J GASTROINTEST SURG

JI J. Gastrointest. Surg.

PD JAN

PY 2019

VL 23

IS 1

BP 11

EP 22

DI 10.1007/s11605-018-3958-8

PG 12

WC Gastroenterology & Hepatology; Surgery

WE Science Citation Index Expanded (SCI-EXPANDED); Conference Proceedings Citation Index - Science (CPCI-S)

SC Gastroenterology & Hepatology; Surgery

GA HI1LD

UT WOS:000456206000002

PM 30215197

OA Green Accepted

DA 2022-05-19

ER

PT J

AU Liu, D

Zhou, KM

Li, QX

Deng, FY

Ma, YQ

AF Liu, Dong

Zhou, Keming

Li, Qiaoxin

Deng, Feiyan

Ma, Yuqing

TI Expression of Wnt11 and Rock2 protein with clinical characteristics of

esophageal squamous cell carcinoma in Kazakh and Han patients

SO INTERNATIONAL JOURNAL OF CLINICAL AND EXPERIMENTAL PATHOLOGY

LA English

DT Article

DE Esophageal squamous cell carcinoma; Wnt11; Rock2; immunohistochemistry;

clinicopathologic factors

ID BETA-CATENIN; RHO/ROCK PATHWAY; INVASION; CANCER; RHO; KINASE;

METASTASIS; GROWTH; OVEREXPRESSION; PROGRESSION

AB Background: Esophageal squamous cell carcinoma (ESCC) is one of the most malignancies with a very poor outcome in China. Wnt11 and Rock2, new identified proteins highly associated with metastasis of many cancers, which were never reported in esophageal squamous cell carcinoma (ESCC). Here we measured the expression levels of Wnt11 and Rock2 in tissues from 265 patients with ESCC. Immunohistochemical staining was employed to detect the correlation of Wnt11 and Rock2 expression with clinicopathological features. Methods: The expression of Wnt11 and Rock2 was detected by immunohistochemistry in esophageal squamous cell carcinomas and normal esophageal tissues. A chi-square test was used to assess the statistical significance of the correlations between Wnt11, Rock2 expression and different clinicopathological parameters, respectively. Results: The high-expression of Wnt11 and Rock2 was observed in ESCCs. Seventy-five cases of ESCC (51.7%) showed a positive expression of Wnt11, which indicated a significant association with the AJCC stage (P=0.007). Ninety-eight cases of ESCC (65.5%) showed a positive expression of Rock2, which indicated a significant association with ethnic background. There were no close correlations between Rock2 expression and gender, tumor location, AJCC stage, lymph node metastasis. Specifically, the expression of Rock2 was significantly different between Hans and Kazaks ethnicities (P=0.000). In Kaplan-Meier curve analysis, no significant correlation was observed between the expression of Wnt11, Rock-2 and the poor prognosis of ESCCs. Conclusion: Our finding suggests that the over-expression of Rock2 may play an important role in the carcinogenesis and progression, and may become a new underlying molecular marker in the diagnosis and treatment in ESCC.

C1 [Liu, Dong; Li, Qiaoxin; Deng, Feiyan; Ma, Yuqing] Xinjiang Med Univ, Dept Pathol, Affiliated Hosp 1, Urumqi 830054, Peoples R China.

[Zhou, Keming] Peoples Hosp Xinjiang Uygur Autonomous Reg, Hypertens Ctr, Hypertens Inst Xinjiang Uygur Autonomous Reg, Urumqi 830001, Peoples R China.

RP Ma, YQ (corresponding author), Xinjiang Med Univ, Dept Pathol, Affiliated Hosp 1, Urumqi 830054, Peoples R China.

EM mayuqing-patho@hotmail.com

FU National Natural Science Foundation of China [81260308]; State Key Lab

Incubation Base of Xinjiang Major Disease [SKLIB-XJMDR-2014-12]

FX This study was supported by the grant from "The National Natural Science

Foundation of China" (81260308) and "State Key Lab Incubation Base of

Xinjiang Major Disease" (SKLIB-XJMDR-2014-12).

CR Borisoff JF, 2003, MOL CELL NEUROSCI, V22, P405, DOI 10.1016/S1044-7431(02)00032-5

Cha SW, 2009, CURR BIOL, V19, P1573, DOI 10.1016/j.cub.2009.07.062

Cui XB, 2014, J EXP CLIN CANC RES, V33, DOI 10.1186/1756-9966-33-20

Cui XB, 2014, J MED VIROL, V86, P608, DOI 10.1002/jmv.23775

Dwyer MA, 2010, CANCER RES, V70, P9298, DOI 10.1158/0008-5472.CAN-10-0226

Eisenberg CA, 1999, DEV DYNAM, V216, P45, DOI 10.1002/(SICI)1097-0177(199909)216:1<45::AID-DVDY7>3.3.CO;2-C

Hsu PK, 2010, ANN THORAC SURG, V89, P1024, DOI 10.1016/j.athoracsur.2010.01.017

Huang LJ, 2012, DIAGN PATHOL, V7, DOI 10.1186/1746-1596-7-180

Imamura F, 2000, JPN J CANCER RES, V91, P811, DOI 10.1111/j.1349-7006.2000.tb01018.x

Itoh K, 1999, NAT MED, V5, P221, DOI 10.1038/5587

Kamai T, 2003, CLIN CANCER RES, V9, P2632

Kamai T, 2004, CLIN CANCER RES, V10, P4799, DOI 10.1158/1078-0432.CCR-0436-03

Katoh M, 2009, INT J MOL MED, V24, P247, DOI 10.3892/ijmm_00000227

Kirikoshi H, 2001, INT J MOL MED, V8, P651

Komagome R, 2000, JPN J VET RES, V47, P127

Kuwano Hiroyuki, 2003, Ann Thorac Cardiovasc Surg, V9, P6

Lane J, 2008, INT J ONCOL, V33, P585, DOI 10.3892/ijo_00000044

Lock FE, 2009, PLOS ONE, V4, DOI 10.1371/journal.pone.0008190

Majumdar A, 2003, DEVELOPMENT, V130, P3175, DOI 10.1242/dev.00520

Mikels AJ, 2006, PLOS BIOL, V4, P570, DOI 10.1371/journal.pbio.0040115

Monnier PP, 2003, MOL CELL NEUROSCI, V22, P319, DOI 10.1016/S1044-7431(02)00035-0

Nagy II, 2010, CARDIOVASC RES, V85, P100, DOI 10.1093/cvr/cvp254

Ouko L, 2004, J BIOL CHEM, V279, P26707, DOI 10.1074/jbc.M402877200

Parkin DM, 2005, CA-CANCER J CLIN, V55, P74, DOI 10.3322/canjclin.55.2.74

Rice TW, 2009, DIS ESOPHAGUS, V22, P1, DOI 10.1111/j.1442-2050.2008.00901.x

Riento K, 2003, NAT REV MOL CELL BIO, V4, P446, DOI 10.1038/nrm1128

Rosel D, 2008, MOL CANCER RES, V6, P1410, DOI 10.1158/1541-7786.MCR-07-2174

Shimada Temiko, 2007, Kobe J Med Sci, V53, P125

Shimizu Y, 2005, J CELL BIOL, V168, P941, DOI 10.1083/jcb.200411179

Tada M, 2002, SEMIN CELL DEV BIOL, V13, P251, DOI 10.1016/S1084-9521(02)00052-6

Thumkeo D, 2003, MOL CELL BIOL, V23, P5043, DOI 10.1128/MCB.23.14.5043-5055.2003

Ueno K, 2009, BRIT J CANCER, V101, P1374, DOI 10.1038/sj.bjc.6605307

Uysal-Onganer P, 2012, ACTA PHYSIOL, V204, P52, DOI 10.1111/j.1748-1716.2011.02297.x

Van Eyk JE, 1998, J BIOL CHEM, V273, P23433, DOI 10.1074/jbc.273.36.23433

Vishnubhotla R, 2007, LAB INVEST, V87, P1149, DOI 10.1038/labinvest.3700674

Wang GQ, 2005, GUT, V54, P187, DOI 10.1136/gut.2004.046631

Xue F, 2008, HEPATOL RES, V38, P810, DOI 10.1111/j.1872-034X.2008.00333.x

Yoshioka K, 1999, CANCER RES, V59, P2004

Yoshioka K, 1998, J BIOL CHEM, V273, P5146, DOI 10.1074/jbc.273.9.5146

Zhu HN, 2004, CANCER RES, V64, P7918, DOI 10.1158/0008-5472.CAN-04-2704

NR 40

TC 3

Z9 3

U1 1

U2 1

PU E-CENTURY PUBLISHING CORP

PI MADISON

PA 40 WHITE OAKS LN, MADISON, WI 53711 USA

SN 1936-2625

J9 INT J CLIN EXP PATHO

JI Int. J. Clin. Exp. Pathol.

PY 2015

VL 8

IS 6

BP 7122

EP 7130

PG 9

WC Oncology; Pathology

WE Science Citation Index Expanded (SCI-EXPANDED)

SC Oncology; Pathology

GA CO6PD

UT WOS:000359277700128

PM 26261605

DA 2022-05-19

ER

PT J

AU Yang, F

Fu, Y

Kumar, A

Chen, MS

Si, L

Rojanasarot, S

AF Yang, Fan

Fu, Yu

Kumar, Arun

Chen, Mingsheng

Si, Lei

Rojanasarot, Sirikan

TI Cost-effectiveness analysis of camrelizumab in the second-line treatment

for advanced or metastatic esophageal squamous cell carcinoma in China

SO ANNALS OF TRANSLATIONAL MEDICINE

LA English

DT Article

DE Advanced; metastatic esophageal squamous cell carcinoma (advanced;

metastatic ESCC); cost-effectiveness analysis; camrelizumab; second-line

treatment

ID CLINICAL-PRACTICE GUIDELINES; CANCER; CHEMOTHERAPY; EPIDEMIOLOGY; JAPAN

AB Background: This study aimed to evaluate the cost-effectiveness of camrelizumab versus chemotherapy as second-line treatment for patients with advanced/metastatic esophageal squamous cell carcinoma (ESCC) from the perspective of the Chinese healthcare system. Methods: A trial-based Markov model was constructed using Excel to integrate clinical and economic data in a hypothetical cohort of advanced/metastatic ESCC patients with a 5-year time horizon. Clinical inputs were derived directly from the ESCORT trial (NCT03099382). Weibull distribution was used to fit transition probabilities extracted from the Kaplan-Meier curves. Cost inputs were estimated from the Beijing Medicine Sunshine Purchasing official website, local charges, publications and expert opinions. Deterministic and probabilistic sensitivity analyses were performed to test the robustness of the model results. Results: At 5 years, camrelizumab had higher quality-adjusted life years (QALYs) (0.782 vs. 0.499) and higher cost (US$31,537 vs. US$6,998) than chemotherapy. The incremental cost-effectiveness ratio (ICER) was estimated to be US$86,745 per QALY gained. The two primary parameters upon which this result was most sensitive were median overall survival of camrelizumab and camrelizumab cost. At a willingness-to-pay threshold of three times per capita gross domestic product (US$30,094 per QALY gained), the probability of camrelizumab being cost-effective was 33.7%. Conclusions: Camrelizumab was not cost-effective as a second-line treatment for advanced/metastatic ESCC patients in China compared with chemotherapy. Keywords: Advanced/metastatic esophageal squamous cell carcinoma (advanced/metastatic ESCC); costeffectiveness analysis; camrelizumab; second-line treatment

C1 [Yang, Fan; Fu, Yu; Chen, Mingsheng] Nanjing Med Univ, Sch Hlth Policy & Management, 101 Longmian Ave, Nanjing 211166, Peoples R China.

[Yang, Fan; Fu, Yu; Chen, Mingsheng] Nanjing Med Univ, Creat Hlth Policy Res Grp, Nanjing, Peoples R China.

[Yang, Fan; Fu, Yu; Chen, Mingsheng] Nanjing Med Univ, Ctr Global Hlth, Nanjing, Peoples R China.

[Kumar, Arun] Univ Minnesota, Coll Pharm, Minneapolis, MN 55455 USA.

[Si, Lei] UNSW Sydney, George Inst Global Hlth, Kensington, NSW, Australia.

[Si, Lei] UNSW Australia, UNSW Med, Sydney, NSW, Australia.

[Rojanasarot, Sirikan] Boston Sci, Marlborough, MA USA.

RP Yang, F (corresponding author), Nanjing Med Univ, Sch Hlth Policy & Management, 101 Longmian Ave, Nanjing 211166, Peoples R China.

EM yangfan512@njmu.edu.cn

CR Abnet CC, 2018, GASTROENTEROLOGY, V154, P360, DOI 10.1053/j.gastro.2017.08.023

Al-Batran SE, 2016, ANN ONCOL, V27, P673, DOI 10.1093/annonc/mdv625

[Anonymous], 2021, INTEGRATED MANAGEMEN

[Anonymous], 2020, NAT EC WAS GEN STABL

Bray F, 2018, CA-CANCER J CLIN, V68, P394, DOI 10.3322/caac.21492

Chen WQ, 2016, CA-CANCER J CLIN, V66, P115, DOI 10.3322/caac.21338

Committee of Guidelines of Chinese society of clinical oncology, 2020, GUID CHIN SOC CLIN O

Gu XH, 2019, LUNG CANCER, V127, P84, DOI 10.1016/j.lungcan.2018.11.029

Guo LW, 2017, CHIN J CANCER, V36, DOI 10.1186/s40880-017-0242-3

Hollman C, 2017, PHARMACOECONOMICS, V35, P817, DOI 10.1007/s40273-017-0510-8

Huang J, 2020, LANCET ONCOL, V21, P832, DOI 10.1016/S1470-2045(20)30110-8

Jimenez P, 2011, J GASTROINTEST ONCOL, V2, P240, DOI 10.3978/j.issn.2078-6891.2011.027

Jinglin H, 2020, COMPREHENSIVELY DEEP

Kato K, 2019, LANCET ONCOL, V20, P1506, DOI 10.1016/S1470-2045(19)30626-6

Kitagawa Y, 2019, ESOPHAGUS-TOKYO, V16, P25, DOI 10.1007/s10388-018-0642-8

Lin YS, 2013, J EPIDEMIOL, V23, P233, DOI 10.2188/jea.JE20120162

Liu CY, 2018, WORLD J SURG, V42, P2522, DOI 10.1007/s00268-018-4501-5

Liu G., 2020, CHINA GUIDELINES PHA, V2st .

Mario C, 2010, INTRO SURVIVAL ANAL, V3rd

Muro K, 2004, ANN ONCOL, V15, P955, DOI 10.1093/annonc/mdh231

National Institute for Health and Care Excellence, 2013, GUID METH TECHN APPR

Rustgi AK, 2014, NEW ENGL J MED, V371, P2499, DOI 10.1056/NEJMra1314530

Salcedo J, 2020, CANCER MED-US, V9, P440, DOI 10.1002/cam4.2721

Shah MA, 2019, J CLIN ONCOL, V37, DOI 10.1200/JCO.2019.37.15_suppl.4010

Stahl M, 2013, ANN ONCOL, V24, P51, DOI 10.1093/annonc/mdt342

Sun DQ, 2020, CHINESE J CANCER RES, V32, P129, DOI 10.21147/j.issn.1000-9604.2020.02.01

Tan CQ, 2014, PHARMACOECONOMICS, V32, P235, DOI 10.1007/s40273-013-0065-2

Wu B, 2014, BMC CANCER, V14, DOI 10.1186/1471-2407-14-984

Wu B, 2012, CLIN THER, V34, P468, DOI 10.1016/j.clinthera.2012.01.012

Yang J, 2012, WORLD J GASTROENTERO, V18, P2493, DOI 10.3748/wjg.v18.i20.2493

Zeng HM, 2018, LANCET GLOB HEALTH, V6, pE555, DOI 10.1016/S2214-109X(18)30127-X

Zhan M, 2019, RADIOTHER ONCOL, V141, P27, DOI 10.1016/j.radonc.2019.07.031

Zhang PF, 2020, FUTURE ONCOL, V16, P1189, DOI 10.2217/fon-2019-0821

Zhou MG, 2019, LANCET, V394, P1145, DOI 10.1016/S0140-6736(19)30427-1

NR 34

TC 3

Z9 3

U1 7

U2 12

PU AME PUBL CO

PI SHATIN

PA FLAT-RM C 16F, KINGS WING PLAZA 1, NO 3 KWAN ST, SHATIN, HONG KONG

00000, PEOPLES R CHINA

SN 2305-5839

EI 2305-5847

J9 ANN TRANSL MED

JI ANN. TRANSL. MED.

PD AUG

PY 2021

VL 9

IS 15

AR 1803

DI 10.21037/atm-21-1803

PG 11

WC Oncology; Medicine, Research & Experimental

WE Science Citation Index Expanded (SCI-EXPANDED)

SC Oncology; Research & Experimental Medicine

GA UA6LY

UT WOS:000685272900009

PM 34532363

OA Green Published, gold

DA 2022-05-19

ER

PT J

AU Zhang, R

Liu, J

Zhang, W

Hua, L

Qian, LT

Zhou, SB

AF Zhang, Rui

Liu, Jing

Zhang, Wei

Hua, Lei

Qian, Li-Ting

Zhou, Shao-Bing

TI EphA5 knockdown enhances the invasion and migration ability of

esophageal squamous cell carcinoma via epithelial-mesenchymal transition

through activating Wnt/beta-catenin pathway

SO CANCER CELL INTERNATIONAL

LA English

DT Article

DE Esophageal squamous cell carcinoma; EphA5; Epithelial-mesenchymal

transition; Wnt; beta-catenin

ID DOWN-REGULATION; EXPRESSION; PROGNOSIS

AB Background The erythropoietin-producing hepatocellular (Eph) receptor A5 (EphA5) has been found to be overexpressed in some malignant tumors and is associated with disease prognosis. However, the role of EphA5 in esophageal squamous cell carcinoma (ESCC) is not clear. Methods In the present study, we measured the expression of EphA5 in ESCC tissues and cell lines including KYSE150 and KYSE450 cells. siRNA transfection was used to interfere with EphA5 expression in ESCC cell lines. Cell viability, colony formation, scratch and invasion assays were performed to explore the roles of EphA5 in ESCC cell lines. Flow cytometry analysis was performed to investigate whether EphA5 could affect the cell apoptosis and cycle. The biomarkers related to epithelial-mesenchymal transition (EMT) and molecules associated with Wnt/beta-catenin signaling were also measured by western blot and immunofluorescence. Results The protein and mRNA expression of EphA5 were significantly higher in fresh ESCC tissues and cell lines compared with normal control groups and human normal esophageal epithelial cells (HEEC). The cell viability assay and colony formation assay revealed that EphA5 knockdown enhanced the proliferation of KYSE150 and KYSE450 cells in vitro. The invasion and migration of ESCC cells were accelerated after EphA5 knockdown. The expression of EMT biomarkers was altered in ESCC cells transfected with siRNA targeting EphA5. Moreover, EphA5 downregulation enhanced the protein levels of beta-catenin and p-GSK-3 beta(Ser9), which play a key role in the Wnt/beta-catenin pathway. Conclusions EphA5 knockdown promotes the proliferation of esophageal squamous cell carcinoma,enhances invasion and migration ability via epithelial-mesenchymal transition through activating Wnt/beta-catenin pathway.

C1 [Zhang, Rui] Shan Dong Univ, Sch Clin Med, Jinan 250000, Shandong, Peoples R China.

[Zhang, Rui; Liu, Jing] Fudan Univ, Zhongshan Hosp, Qing Pu Branch, Dept Oncol, Shanghai 201799, Peoples R China.

[Zhang, Wei] Taixing Peoples Hosp, Dept Pathol, Taixing 225400, Jiangsu, Peoples R China.

[Hua, Lei] Anhui Med Univ, Anhui Prov Hosp, Dept Prov Clin Coll, Hefei 230031, Anhui, Peoples R China.

[Qian, Li-Ting] Univ Sci & Technol China, Affiliated Hosp 1, Div Life Sci & Med, Dept Radiat Oncol, Hefei 230001, Anhui, Peoples R China.

[Zhou, Shao-Bing] Taixing Peoples Hosp, Dept Radiat Oncol, Taixing 225400, Jiangsu, Peoples R China.

RP Qian, LT (corresponding author), Univ Sci & Technol China, Affiliated Hosp 1, Div Life Sci & Med, Dept Radiat Oncol, Hefei 230001, Anhui, Peoples R China.; Zhou, SB (corresponding author), Taixing Peoples Hosp, Dept Radiat Oncol, Taixing 225400, Jiangsu, Peoples R China.

EM emailqlt@163.com; zzrr19840227@163.com

FU Science and Technology Foundation of Anhui Province [1704a0802157];

Shanghai Qingpu Science and Technology Development Foundation (CN) [QKY

2018-07]

FX This work was supported by the Science and Technology Foundation of

Anhui Province (No. 1704a0802157) and Shanghai Qingpu Science and

Technology Development Foundation (CN) (QKY 2018-07).

CR Nieto MA, 2016, CELL, V166, P21, DOI 10.1016/j.cell.2016.06.028

[Anonymous], 1997, CELL, V90, P403, DOI DOI 10.1016/S0092-8674(00)80500-0

Aponte PM, 2017, STEM CELLS INT, V2017, DOI 10.1155/2017/5619472

Brabletz T, 2012, NAT REV CANCER, V12, P425, DOI 10.1038/nrc3265

Bray F, 2018, CA-CANCER J CLIN, V68, P394, DOI 10.3322/caac.21492

Chen X, 2016, J OVARIAN RES, V9, DOI 10.1186/s13048-016-0292-1

Fu DY, 2010, HUM PATHOL, V41, P48, DOI 10.1016/j.humpath.2009.06.007

Gao YP, 2017, J EXP CLIN CANC RES, V36, DOI 10.1186/s13046-017-0622-1

Giaginis C, 2010, PATHOL ONCOL RES, V16, P267, DOI 10.1007/s12253-009-9221-6

Gu SD, 2017, HISTOL HISTOPATHOL, V32, P491, DOI 10.14670/HH-11-815

Huang J, 2014, ONCOGENE, V33, P2737, DOI 10.1038/onc.2013.238

Lagergren J, 2017, LANCET, V390, P2383, DOI 10.1016/S0140-6736(17)31462-9

Li SB, 2015, BMC CANCER, V15, DOI 10.1186/s12885-015-1025-3

Li YF, 2019, FASEB J, V33, P4851, DOI 10.1096/fj.201701561RRRR

Mirza MK, 2010, J EXP MED, V207, P1675, DOI 10.1084/jem.20091857

Niwa Y, 2014, J SURG ONCOL, V110, P764, DOI 10.1002/jso.23694

Pasquale EB, 2008, CELL, V133, P38, DOI 10.1016/j.cell.2008.03.011

Peng Q, 2018, CELL DEATH DIS, V9, DOI 10.1038/s41419-018-1164-y

Puisieux A, 2014, NAT CELL BIOL, V16, P488, DOI 10.1038/ncb2976

Qin TT, 2018, BIOMED PHARMACOTHER, V103, P308, DOI 10.1016/j.biopha.2018.04.032

SHIMADA Y, 1992, CANCER, V69, P277, DOI 10.1002/1097-0142(19920115)69:2<277::AID-CNCR2820690202>3.0.CO;2-C

Singh M, 2018, PHARMACOL THERAPEUT, V182, P80, DOI 10.1016/j.pharmthera.2017.08.009

Staquicini FI, 2015, J BIOL CHEM, V290, P7345, DOI 10.1074/jbc.M114.630525

Su HF, 2019, ONCOL REP, V41, P599, DOI 10.3892/or.2018.6799

Sun Bingsheng, 2008, Cancer Biol Ther, V7, P387

Tejeda-Munoz N, 2015, IUBMB LIFE, V67, P914, DOI 10.1002/iub.1454

Wu JC, 2010, J CANCER RES CLIN, V136, P595, DOI 10.1007/s00432-009-0695-0

Zhang W, 2017, ONCOGENE, V36, P3890, DOI 10.1038/onc.2017.15

Zhang W, 2019, ONCOL LETT, V17, P5147, DOI 10.3892/ol.2019.10167

Zhu XM, 2019, EXP CELL RES, V375, P41, DOI 10.1016/j.yexcr.2019.01.002

NR 30

TC 19

Z9 19

U1 0

U2 3

PU BMC

PI LONDON

PA CAMPUS, 4 CRINAN ST, LONDON N1 9XW, ENGLAND

EI 1475-2867

J9 CANCER CELL INT

JI Cancer Cell Int.

PD JAN 13

PY 2020

VL 20

IS 1

AR 20

DI 10.1186/s12935-020-1101-x

PG 12

WC Oncology

WE Science Citation Index Expanded (SCI-EXPANDED)

SC Oncology

GA KL8WS

UT WOS:000513699500005

PM 31956298

OA Green Published, gold

DA 2022-05-19

ER

PT J

AU Danese, M

Gricar, J

Abraham, P

AF Danese, Mark

Gricar, Joseph

Abraham, Pranav

TI Real-world outcomes with second-line therapy in advanced esophageal

squamous cell carcinoma using SEER-Medicare data

SO FUTURE ONCOLOGY

LA English

DT Article

DE esophageal squamous cell carcinoma; locally advanced; overall survival;

recurrent or metastatic; second line; treatment patterns; unresectable

ID CANCER; EPIDEMIOLOGY

AB Lay abstract This study assessed how US physicians have been treating a common type of esophageal cancer, known as squamous cell carcinoma, which has spread from the esophagus to other parts of the body (advanced/metastatic cancer). We looked at information from US cancer registry data on 756 people who were 66 years and older and diagnosed between 2010 and 2015. Only 14% of people received a second kind of chemotherapy after their first chemotherapy was stopped. People received their second chemotherapy for a short period (approximately 6 weeks) and lived for approximately 6 months on average from start of treatment. This research highlights that more effective treatments are needed for older people with advanced/metastatic esophageal squamous cell carcinoma.

Aim: To characterize real-world patterns of second-line treatment and outcomes in older patients with advanced/metastatic esophageal squamous cell carcinoma (ESCC). Patients and methods: Patients aged >= 66 years diagnosed with advanced/metastatic ESCC between 2010 and 2015 and followed through 2016 were included in this retrospective analysis using SEER-Medicare data. Results: Of 756 patients with advanced/metastatic ESCC, 104 (14%) received second-line therapy; median duration of treatment was 1.5 months. Median overall survival was 5.7 months for all patients receiving second-line treatment, and 4.5, 5.6 and 8.5 months, respectively, for patients receiving taxane monotherapy, taxane combination therapy and nontaxane therapy. Conclusion: A small proportion of patients with advanced/metastatic ESCC received second-line therapy, which was associated with short duration of treatment and poor overall survival.

C1 [Danese, Mark] Outcomes Insights Inc, Agoura Hills, CA 91361 USA.

[Gricar, Joseph; Abraham, Pranav] Bristol Myers Squibb, Princeton, NJ USA.

RP Danese, M (corresponding author), Outcomes Insights Inc, Agoura Hills, CA 91361 USA.

EM mark@outins.com

OI Danese, Mark/0000-0002-7068-9603

FU Bristol Myers Squibb

FX This study was funded by Bristol Myers Squibb, which was involved in

study design; in the collection, analysis and interpretation of data; in

the writing of the report; and in the decision to submit the article for

publication. Employees from Bristol Myers Squibb participated as authors

in this publication, in compliance with authorship guidelines from the

International Committee of Medical Journal Editors. All authors

contributed to and approved the article. M Danese received funding from

Bristol Myers Squibb to conduct this research. J Gricar and P Abraham

were employees of Bristol Myers Squibb when this research was conducted.

The authors have no other relevant affiliations or financial involvement

with any organization or entity with a financial interest in or

financial conflict with the subject matter or materials discussed in the

manuscript apart from those disclosed.

CR Abnet CC, 2018, GASTROENTEROLOGY, V154, P360, DOI 10.1053/j.gastro.2017.08.023

Abraham P, 2020, ADV THER, V37, P3392, DOI 10.1007/s12325-020-01394-y

Berry MF, 2014, J THORAC DIS, V6, pS289, DOI 10.3978/j.issn.2072-1439.2014.03.11

Bray F, 2018, CA-CANCER J CLIN, V68, P394, DOI 10.3322/caac.21492

Danese MD, 2017, LEUKEMIA LYMPHOMA, V58, P1094, DOI 10.1080/10428194.2016.1228924

Davidoff AJ, 2010, J CLIN ONCOL, V28, P2191, DOI 10.1200/JCO.2009.25.4052

Edge SB, 2010, AJCC CANC STAGING MA, V7th ed

Enewold Lindsey, 2020, Journal of the National Cancer Institute Monographs, P3, DOI 10.1093/jncimonographs/lgz029

Engel LS, 2003, J NATL CANCER I, V95, P1404, DOI 10.1093/jnci/djg047

Kato K, 2019, LANCET ONCOL, V20, P1506, DOI 10.1016/S1470-2045(19)30626-6

Klabunde CN, 2007, ANN EPIDEMIOL, V17, P584, DOI 10.1016/j.annepidem.2007.03.011

Kojima T, 2020, J CLIN ONCOL, V38, DOI 10.1200/JCO.20.01888

Thallinger CMR, 2011, J CLIN ONCOL, V29, P4709, DOI 10.1200/JCO.2011.36.7599

Then EO, 2020, WORLD J ONCOL, V11, P55, DOI 10.14740/wjon1254

Torre LA, 2016, CANCER EPIDEM BIOMAR, V25, P16, DOI 10.1158/1055-9965.EPI-15-0578

NR 15

TC 0

Z9 0

U1 0

U2 0

PU FUTURE MEDICINE LTD

PI LONDON

PA UNITEC HOUSE, 3RD FLOOR, 2 ALBERT PLACE, FINCHLEY CENTRAL, LONDON, N3

1QB, ENGLAND

SN 1479-6694

EI 1744-8301

J9 FUTURE ONCOL

JI Future Oncol.

PD MAR

PY 2022

VL 18

IS 8

BP 927

EP 936

DI 10.2217/fon-2021-0460

EA JAN 2022

PG 10

WC Oncology

WE Science Citation Index Expanded (SCI-EXPANDED)

SC Oncology

GA ZK4AO

UT WOS:000747187100001

PM 35081734

OA hybrid

DA 2022-05-19

ER

PT J

AU Mohammadi, F

Javid, H

Afshari, AR

Mashkani, B

Hashemy, SI

AF Mohammadi, Fariba

Javid, Hossein

Afshari, Amir Reza

Mashkani, Baratali

Hashemy, Seyed Isaac

TI Substance P accelerates the progression of human esophageal squamous

cell carcinoma via MMP-2, MMP-9, VEGF-A, and VEGFR1 overexpression

SO MOLECULAR BIOLOGY REPORTS

LA English

DT Article

DE Esophageal squamous cell carcinoma; Substance P; Aprepitant; Matrix

metalloproteinase; Vascular endothelial growth factor

ID 1 RECEPTOR; CANCER; EXPRESSION; INVASION; SECRETION; PEPTIDES; BOMBESIN

AB Tachykinins such as Substance P (SP) are a group of neuropeptides that are involved in cancer development. Neurokinin-1 receptor (NK-1R) is the main tachykinin receptor mediating the effects of SP, which is overexpressed in human esophageal squamous cell carcinoma (ESCC) and other malignant tissues. However, the effects of SP/NK-1R system on the migration of esophageal cancer cells and angiogenesis is not clear yet. This study seeks to obtain data to address these research gaps. In order to assess the effects of the FDA-approved aprepitant drug, a commercially available NK-1R antagonist, on the viability of KYSE-30 ESCC cells, resazurin assay was performed. The influence of SP/NK-1R system on the migration potential of these cells was examined using scratch assay. The effects of this system on the expression levels of metastatic factors were also examined by RT-PCR and western blot analyses. The half-maximal inhibitory concentration (IC50) value for KYSE-30 cells treated with aprepitant found to be 29.88 mu M. Treatment with SP significantly promoted KYSE-30 esophageal cancer cell migration, and aprepitant blocked this effect. In addition, SP significantly induced the expression of matrix metalloproteinase-2 (MMP-2), MMP-9, vascular endothelial growth factor-A (VEGF-A), and VEGF receptor1 (VEGFR1) in the cells, whereas aprepitant inhibited the up-regulation effects caused by SP. SP plays important roles in the development of human esophageal squamous cell carcinoma by promoting cancer cell invasion and enhancing the expression of factors involved in cellular migration and angiogenesis, which can be blocked by the NK-1R antagonist, aprepitant.

C1 [Mohammadi, Fariba; Javid, Hossein; Mashkani, Baratali; Hashemy, Seyed Isaac] Mashhad Univ Med Sci, Fac Med, Dept Clin Biochem, Mashhad, Razavi Khorasan, Iran.

[Mohammadi, Fariba; Javid, Hossein; Afshari, Amir Reza] Mashhad Univ Med Sci, Fac Med, Student Res Comm, Mashhad, Razavi Khorasan, Iran.

[Mashkani, Baratali; Hashemy, Seyed Isaac] Mashhad Univ Med Sci, Surg Oncol Res Ctr, Mashhad, Razavi Khorasan, Iran.

RP Hashemy, SI (corresponding author), Mashhad Univ Med Sci, Fac Med, Dept Clin Biochem, Mashhad, Razavi Khorasan, Iran.; Hashemy, SI (corresponding author), Mashhad Univ Med Sci, Surg Oncol Res Ctr, Mashhad, Razavi Khorasan, Iran.

EM hashemyi@mums.ac.ir

RI Hashemy, Seyed Isaac/A-2693-2017

OI Hashemy, Seyed Isaac/0000-0002-1323-5250; Afshari, Amir

R./0000-0001-9989-9818; Javid, Dr. Hossein/0000-0002-2248-7708

FU Research Council, Mashhad University of Medical Sciences, Mashhad, Iran

FX This study was financially supported by the Research Council, Mashhad

University of Medical Sciences, Mashhad, Iran. This manuscript is

written based on the MSc thesis of Fariba Mohammadi.

CR Amirchaghmaghi Maryam, 2016, J Dent (Shiraz), V17, P21

Borra Ricardo Carneiro, 2009, Braz. oral res., V23, P255, DOI 10.1590/S1806-83242009000300006

BOSSALLER C, 1992, HERZ, V17, P284

Bray F, 2018, CA-CANCER J CLIN, V68, P394, DOI 10.3322/caac.21492

Chen Xia-bin, 2009, Zhonghua Yufang Yixue Zazhi, V43, P559, DOI 10.3760/cma.j.issn.0253-9624.2009.07.006

Dalirsani Z, 2019, INT J CANCER MANAG, V12, DOI 10.5812/ijcm.90249

Davoodian M, 2019, MOL BIOL REP, V46, P1285, DOI 10.1007/s11033-019-04599-9

De Felipe C, 1998, NATURE, V392, P394, DOI 10.1038/32904

Demeester Steven R, 2009, Gastrointest Cancer Res, V3, pS2

Dong JQ, 2015, GENE, V560, P205, DOI 10.1016/j.gene.2015.02.002

Ebner K, 2006, AMINO ACIDS, V31, P251, DOI 10.1007/s00726-006-0335-9

Ebrahimi S, 2020, CLIN GENET, V98, P322, DOI 10.1111/cge.13750

Enzinger PC, 2003, NEW ENGL J MED, V349, P2241, DOI 10.1056/NEJMra035010

Fadaee J, 2020, IRAN J ALLERGY ASTHM, V19, P18, DOI 10.18502/ijaai.v19i1.2414

Feistritzer C, 2003, REGUL PEPTIDES, V116, P119, DOI 10.1016/S0167-0115(03)00193-9

Gharaee N, 2018, MOL BIOL REP, V45, P2257, DOI 10.1007/s11033-018-4387-1

Ghasemi A, 2019, MOL CELL BIOCHEM, V458, P207, DOI 10.1007/s11010-019-03544-5

Ghasemi A, 2019, CELL ONCOL, V42, P243, DOI 10.1007/s13402-019-00428-0

Ghasemi A, 2018, J CELL BIOCHEM, V119, P2333, DOI 10.1002/jcb.26396

Ghasemi A, 2017, CELL SIGNAL, V32, P104, DOI 10.1016/j.cellsig.2017.01.020

Gonzalez N, 2008, CURR OPIN ENDOCRINOL, V15, P58, DOI 10.1097/MED.0b013e3282f3709b

Hashemian P, 2020, INT J CANC MANAG, V13, pe100717

Javid H, 2020, MOL BIOL REP, V47, P2253, DOI 10.1007/s11033-020-05330-9

Javid H, 2019, J PHYSIOL BIOCHEM, V75, P415, DOI 10.1007/s13105-019-00697-1

Jiang WG, 2015, SEMIN CANCER BIOL, V35, pS244, DOI 10.1016/j.semcancer.2015.03.008

Jin Z, 2007, CLIN CANCER RES, V13, P6293, DOI 10.1158/1078-0432.CCR-07-0818

Juchniewicz A, 2017, ACTA BIOCHIM POL, V64, P295, DOI 10.18388/abp.2016_1408

Kai F, 2019, DEV CELL, V49, P332, DOI 10.1016/j.devcel.2019.03.026

Kast RE, 2014, ONCOTARGET, V5, P8052, DOI 10.18632/oncotarget.2408

Kataoka M, 1996, INT J ONCOL, V8, P773

KECK PJ, 1989, SCIENCE, V246, P1309, DOI 10.1126/science.2479987

Khorasani S, 2020, J CELL BIOCHEM, V121, P3031, DOI 10.1002/jcb.29668

Li XQ, 2013, MOL CANCER RES, V11, P294, DOI 10.1158/1541-7786.MCR-12-0609

Liu L, 2005, PEPTIDES, V26, P1369, DOI 10.1016/j.peptides.2005.03.027

Lorestani S, 2020, MOL BIOL REP

Meshki J, 2009, J BIOL CHEM, V284, P9280, DOI 10.1074/jbc.M808825200

Morgat C, 2014, J NUCL MED, V55, P1650, DOI 10.2967/jnumed.114.142000

Munoz M, 2013, PEPTIDES, V48, P1, DOI 10.1016/j.peptides.2013.07.024

Palma C, 1998, J NEUROIMMUNOL, V81, P127, DOI 10.1016/S0165-5728(97)00167-7

Pennathur A, 2013, LANCET, V381, P400, DOI 10.1016/S0140-6736(12)60643-6

RUFF MR, 1985, PEPTIDES, V6, P107, DOI 10.1016/0196-9781(85)90142-1

Schratzberger P, 1997, J IMMUNOL, V158, P3895

Shay G, 2015, MATRIX BIOL, V44-46, P200, DOI 10.1016/j.matbio.2015.01.019

Song HJ, 2012, INT J BIOCHEM CELL B, V44, P1410, DOI 10.1016/j.biocel.2012.04.014

Steinhoff MS, 2014, PHYSIOL REV, V94, P265, DOI 10.1152/physrev.00031.2013

Stewart JM, 2002, CAN J PHYSIOL PHARM, V80, P275, DOI 10.1139/Y02-030

Theoharides TC, 2010, P NATL ACAD SCI USA, V107, P4448, DOI 10.1073/pnas.1000803107

Thrumurthy SG, 2019, BMJ-BRIT MED J, V366, DOI 10.1136/bmj.l4373

Wang F, 2019, BIOCHEM BIOPH RES CO, V514, P1210, DOI 10.1016/j.bbrc.2019.05.092

[吴凤鹏 WU Fengpeng], 2008, [中华放射肿瘤学杂志, Chinese Journal of Radiation Oncology], V17, P269

Wu Zherui, 2012, Front Endocrinol (Lausanne), V3, P184, DOI 10.3389/fendo.2012.00184

Xie LN, 2010, CANCER SCI, V101, P2570, DOI 10.1111/j.1349-7006.2010.01735.x

Yu CY, 2004, CANCER RES, V64, P7622, DOI 10.1158/0008-5472.CAN-04-1521

NR 53

TC 14

Z9 15

U1 0

U2 7

PU SPRINGER

PI DORDRECHT

PA VAN GODEWIJCKSTRAAT 30, 3311 GZ DORDRECHT, NETHERLANDS

SN 0301-4851

EI 1573-4978

J9 MOL BIOL REP

JI Mol. Biol. Rep.

PD JUN

PY 2020

VL 47

IS 6

BP 4263

EP 4272

DI 10.1007/s11033-020-05532-1

EA MAY 2020

PG 10

WC Biochemistry & Molecular Biology

WE Science Citation Index Expanded (SCI-EXPANDED)

SC Biochemistry & Molecular Biology

GA MJ4KD

UT WOS:000534469600003

PM 32436041

DA 2022-05-19

ER

PT J

AU Hsu, PK

Huang, CS

Wang, BY

Wu, YC

Hsu, WH

AF Hsu, Po-Kuei

Huang, Chien-Sheng

Wang, Bing-Yen

Wu, Yu-Chung

Hsu, Wen-Hu

TI Survival Benefits of Postoperative Chemoradiation for Lymph

Node-Positive Esophageal Squamous Cell Carcinoma

SO ANNALS OF THORACIC SURGERY

LA English

DT Article

ID SURGERY; CANCER; CHEMORADIOTHERAPY; NEOADJUVANT; ADJUVANT; THERAPY;

TRIAL

AB Background. Little is known about the efficacy of chemoradiation therapy after surgery for patients with esophageal squamous cell carcinoma. This retrospective study aimed to determine whether postoperative chemoradiation improves survival compared with surgery alone.

Methods. Of 290 patients with esophageal squamous cell carcinoma, 104 received postoperative chemoradiation therapy (CRT group) and 186 underwent surgery alone (S group). Propensity score matching analysis was used to identify 56 well-balanced pairs of patients to compare outcomes.

Results. For N0 patients, overall survival (OS) and disease-free survival (DFS) were similar in both groups. For N+ patients, the median OS (31.0 versus 16.0 months) and the 3-year OS rate (45.8% versus 14.1%) were significantly higher in the CRT group than in the S group (p < 0.001). Similarly, the median DFS (16.0 versus 9.0 months) and the 3-year DFS rate (24.1% versus 11.5%) were significantly higher in the CRT group than in the S group (p = 0.002). In propensity-matched patients, a survival benefit was observed for N+ patients receiving postoperative chemoradiation (CRT versus S group: median OS 29.0 versus 16.0 months, 3-year OS rate 48.6% versus 16.8%; p = 0.003). Disease-free survival (median DFS 11.0 versus 8.0 months, 3-year DFS rate 21.3% versus 12.5%) tended to be better in the CRT group than in the S group (p = 0.057).

Conclusions. Postoperative chemoradiation therapy provided a survival benefit for patients with lymph node-positive esophageal squamous cell carcinoma. (C) 2014 by The Society of Thoracic Surgeons

C1 [Hsu, Po-Kuei; Huang, Chien-Sheng; Wu, Yu-Chung; Hsu, Wen-Hu] Taipei Vet Gen Hosp, Dept Surg, Div Thorac Surg, Taipei, Taiwan.

[Hsu, Po-Kuei; Huang, Chien-Sheng; Wu, Yu-Chung] Natl Yang Ming Univ, Sch Med, Taipei 112, Taiwan.

[Hsu, Po-Kuei; Huang, Chien-Sheng] Natl Yang Ming Univ, Inst Clin Med, Taipei 112, Taiwan.

[Wang, Bing-Yen] Changhua Christian Hosp, Div Thorac Surg, Dept Surg, Taichung, Taiwan.

RP Hsu, PK (corresponding author), Taipei Vet Gen Hosp, Dept Surg, Div Thorac Surg, 201,Sec 2,Shih Pai Rd, Taipei, Taiwan.

EM hsupokuei@yahoo.com.tw

CR Bedard ELR, 2001, CANCER-AM CANCER SOC, V91, P2423, DOI 10.1002/1097-0142(20010615)91:12<2423::AID-CNCR1277>3.3.CO;2-T

Dexter SPL, 2001, GUT, V48, P667, DOI 10.1136/gut.48.5.667

Dittrick GW, 2012, ANN SURG ONCOL, V19, P1678, DOI 10.1245/s10434-011-2078-4

Edge S B, 2010, AM JOINT COMMITTEE C

Hsu PK, 2013, ANN THORAC SURG, V96, P995, DOI 10.1016/j.athoracsur.2013.04.098

Hsu PK, 2011, J GASTROINTEST SURG, V15, P558, DOI 10.1007/s11605-011-1458-1

Macdonald JS, 2001, NEW ENGL J MED, V345, P725, DOI 10.1056/NEJMoa010187

Mariette C, 2010, J CLIN ONCOL, V28

National Comprehensive Cancer Network, 2014, ES CANC CLIN PRACT G

Pennathur A, 2013, LANCET, V381, P400, DOI 10.1016/S0140-6736(12)60643-6

Rice TW, 2003, J THORAC CARDIOV SUR, V126, P1590, DOI 10.1016/S0022-5223(03)01025-0

Tepper J, 2008, J CLIN ONCOL, V26, P1086, DOI 10.1200/JCO.2007.12.9593

Urba SG, 2001, J CLIN ONCOL, V19, P305, DOI 10.1200/JCO.2001.19.2.305

van Hagen P, 2012, NEW ENGL J MED, V366, P2074, DOI 10.1056/NEJMoa1112088

Visser BC, 2003, SURG ONCOL, V12, P1, DOI 10.1016/S0960-7404(02)00072-5

Xu YP, 2012, WORLD J SURG ONCOL, V10, DOI 10.1186/1477-7819-10-173

NR 16

TC 52

Z9 66

U1 0

U2 11

PU ELSEVIER SCIENCE INC

PI NEW YORK

PA STE 800, 230 PARK AVE, NEW YORK, NY 10169 USA

SN 0003-4975

EI 1552-6259

J9 ANN THORAC SURG

JI Ann. Thorac. Surg.

PD MAY

PY 2014

VL 97

IS 5

BP 1734

EP 1741

DI 10.1016/j.athoracsur.2013.12.041

PG 8

WC Cardiac & Cardiovascular Systems; Respiratory System; Surgery

WE Science Citation Index Expanded (SCI-EXPANDED)

SC Cardiovascular System & Cardiology; Respiratory System; Surgery

GA AG5HX

UT WOS:000335451200064

PM 24612702

DA 2022-05-19

ER

PT J

AU Liu, YW

Yan, FW

Tsai, DL

Li, HP

Lee, YL

Chiang, HH

Hsu, HT

Chuang, HY

Chou, SH

AF Liu, Yu-Wei

Yan, Fan-Wei

Tsai, Dong-Lin

Li, Hsien-Pin

Lee, Yen-Lung

Chiang, Hung-Hsing

Hsu, Hung-Te

Chuang, Hung-Yi

Chou, Shah-Hwa

TI Expedite recovery from esophagectomy and reconstruction for esophageal

squamous cell carcinoma after perioperative management protocol

reinvention

SO JOURNAL OF THORACIC DISEASE

LA English

DT Article

DE Recovery; perioperative management; esophagectomy; esophageal squamous

cell carcinoma

ID ENHANCED RECOVERY; CANCER; OUTCOMES; EXTUBATION; RESECTION

AB Background: Surgery for esophageal cancer is invasive and challenging, and always to be followed with arduous post-operative care and recovery. This study, maybe one of the first in Asian populations, is to determine whether a reinvented protocol for perioperative management for esophageal cancer surgery which is being implemented in our department, will lead to a faster convalescence and also significantly decrease financial burdens garnered by patients during hospitalization.

Methods: Operated on by the same surgeon and team in the same hospital, consecutive patients who had received esophagectomy and reconstruction for esophageal squamous cell carcinoma were retrospectively reviewed. On the basis of two different treatment periods, patients were divided into two groups: A and B. Group A was patients who had received the new reinvented protocol between 2012 and 2016, while group B patients were those having received the previous protocol between 2008 and 2011. Their demographics, post-operative outcome, and hospital charges were collected and compared. Results: There were 64 patients in group A, and 69 in group B. Ventilator days (P<0.001), ICU stay (P<0.001), and post-operative stay (P<0.001) were significantly shorter in group A patients. Complication rates were similar between the two groups. No hospital mortality was noted in either group. Hospital charges in group A were found to be perceptively lower, although not statistically significant (P value =0.078).

Conclusions: The current protocol of perioperative care effectively ameliorated convalescence after esophagectomy and reconstruction for esophageal squamous cell carcinoma without increasing complication rate or mortality. It is also potentially more practical in future health care policies during this era of financial shortage.

C1 [Liu, Yu-Wei; Yan, Fan-Wei; Tsai, Dong-Lin; Li, Hsien-Pin; Lee, Yen-Lung; Chiang, Hung-Hsing; Chou, Shah-Hwa] Kaohsiung Med Univ, Kaohsiung Med Univ Hosp, Dept Surg, Kaohsiung, Taiwan.

[Hsu, Hung-Te] Kaohsiung Municipal Tatung Hosp, Dept Anesthesia, Kaohsiung, Taiwan.

[Chuang, Hung-Yi] Kaohsiung Med Univ, Kaohsiung Med Univ Hosp, Dept Environm & Occupat Med, Kaohsiung, Taiwan.

[Chou, Shah-Hwa] Kaohsiung Med Univ, Dept Resp Therapy, Coll Med, Kaohsiung, Taiwan.

RP Chou, SH (corresponding author), Kaohsiung Med Univ Hosp, Dept Surg, 100,Tzyou 1st Rd, Kaohsiung 80756, Taiwan.

EM shhwch@cc.kmu.edu.tw

RI Chuang, Hung-Yi/C-9143-2009; Chuang, H. Y./AAM-3118-2021; Liu,

Yu-Wei/AAV-7334-2021

OI Chuang, Hung-Yi/0000-0002-8321-8720; Chuang, H. Y./0000-0002-8321-8720;

Liu, Yu-Wei/0000-0001-9908-2322; Hsu, Hung-Te/0000-0002-2140-3146

CR [Anonymous], CANC REG ANN REP 201

Blom RLGM, 2013, WORLD J SURG, V37, P2372, DOI 10.1007/s00268-013-2135-1

Chandrashekar MV, 2003, BRIT J ANAESTH, V90, P474, DOI 10.1093/bja/aeg091

Chou SH, 2010, WORLD J SURG, V34, P1832, DOI 10.1007/s00268-010-0595-0

Decker G, 2009, EUR J CARDIO-THORAC, V35, P13, DOI 10.1016/j.ejcts.2008.09.024

Edge SB, 2010, ANN SURG ONCOL, V17, P1471, DOI 10.1245/s10434-010-0985-4

Findlay JM, 2015, DIS ESOPHAGUS, V28, P567, DOI 10.1111/dote.12234

Gemmill EH, 2007, BRIT J SURG, V94, P1461, DOI 10.1002/bjs.6015

Guo W, 2016, SURG ENDOSC, V30, P3873, DOI 10.1007/s00464-015-4692-x

Hjort Jakobsen D, 2004, Scand J Surg, V93, P24

Kehlet H, 2002, AM J SURG, V183, P630, DOI 10.1016/S0002-9610(02)00866-8

Lanuti M, 2006, ANN THORAC SURG, V82, P2037, DOI 10.1016/j.athoracsur.2006.07.024

Lazar G, 2003, SURGERY, V134, P799, DOI 10.1016/S0039-6060(03)00254-X

Liu QX, 2014, WORLD J GASTROENTERO, V20, P17218, DOI 10.3748/wjg.v20.i45.17218

Markar SR, 2015, DIS ESOPHAGUS, V28, P468, DOI 10.1111/dote.12214

Okuyama M, 2007, SURG TODAY, V37, P947, DOI 10.1007/s00595-007-3541-5

Safranek PM, 2010, BRIT J SURG, V97, P1845, DOI 10.1002/bjs.7231

Shewale JB, 2015, ANN SURG, V261, P1114, DOI 10.1097/SLA.0000000000000971

Thiele RH, 2015, CAN J ANESTH, V62, P169, DOI 10.1007/s12630-014-0265-z

Watkins A C, 2001, J Perianesth Nurs, V16, P379, DOI 10.1053/jpan.2001.28887

Weijs TJ, 2016, ANN THORAC SURG, V102, P1141, DOI 10.1016/j.athoracsur.2016.04.067

NR 21

TC 3

Z9 3

U1 0

U2 1

PU PIONEER BIOSCIENCE PUBL CO

PI HONG KONG

PA 9A GOLD SHINE TOWER, 346-348 QUEEN'S RD CENTRAL, SHEUNG WAN, HONG KONG,

00000, PEOPLES R CHINA

SN 2072-1439

EI 2077-6624

J9 J THORAC DIS

JI J. Thorac. Dis.

PD JUL

PY 2017

VL 9

IS 7

BP 2029

EP 2037

DI 10.21037/jtd.2017.06.13

PG 9

WC Respiratory System

WE Science Citation Index Expanded (SCI-EXPANDED)

SC Respiratory System

GA FE2AD

UT WOS:000408017800069

PM 28840003

OA Green Published

DA 2022-05-19

ER

PT J

AU Komatsu, Y

Orita, H

Sakurada, M

Maekawa, H

Hoppo, T

Sato, K

AF Komatsu, Yoshihiro

Orita, Hajime

Sakurada, Mutsumi

Maekawa, Hiroshi

Hoppo, Toshitaka

Sato, Koichi

TI Intraoperative Blood Transfusion Contributes to Decreased Long-Term

Survival of Patients With Esophageal Cancer

SO WORLD JOURNAL OF SURGERY

LA English

DT Article

ID SQUAMOUS-CELL CARCINOMA; DISEASE-FREE SURVIVAL; PROGNOSTIC-FACTORS;

EXTENDED LYMPHADENECTOMY; HEMORRHAGIC-SHOCK; RESECTION; ADENOCARCINOMA;

RECURRENCE; NUMBER; IMPACT

AB Several prognostic factors for patients who have undergone esophagectomy owing to esophageal squamous cell carcinoma have been suggested, including intraoperative blood loss. There are few data, however, suggesting such an association with the prognosis following radical esophagectomy.

Patients with esophageal squamous cell carcinoma who underwent radical esophagectomy were divided into two groups based on the median value of the intraoperative blood loss (510 g). A multivariate Cox proportional-hazard regression analysis was performed to determine if intraoperative blood loss could be an independent prognostic factor for long-term survival following radical esophagectomy. Kaplan-Meier survival analysis with a log-rank test was performed between the groups.

From April 2005 to May 2009, a total of 37 patients underwent radical esophagectomy for the treatment of esophageal squamous cell carcinoma at the Juntendo Shizuoka Hospital and were assigned either to one of two groups: those with >= 510 g blood loss [bleeding group (BG), n = 19] or of those with < 510 g blood loss [less bleeding group (LBG), n = 18]. The distribution of the stage of disease, the number of positive lymph nodes, and the presence of lymphatic and vascular invasion was comparable between the groups, but the Kaplan-Meier survival analysis demonstrated that survival was significantly worse in the BG group than in the LBG group (p = 0.00295). This was supported by the multivariate analysis, which indicated that intraoperative blood loss was independently associated with long-term survival after radical esophagectomy.

Intraoperative blood loss could be a useful prognostic factor following radical esophagectomy in patients with esophageal squamous cell carcinoma.

C1 [Komatsu, Yoshihiro; Orita, Hajime; Sakurada, Mutsumi; Maekawa, Hiroshi; Sato, Koichi] Juntendo Univ, Sch Med, Juntendo Shizuoka Hosp, Dept Surg, Shizuoka 4102295, Japan.

[Hoppo, Toshitaka] Univ Pittsburgh, Med Ctr, Dept Cardiothorac Surg, Pittsburgh, PA 15232 USA.

RP Sato, K (corresponding author), Juntendo Univ, Sch Med, Juntendo Shizuoka Hosp, Dept Surg, 1129 Nagaoka, Shizuoka 4102295, Japan.

EM kou-sato@chive.ocn.ne.jp

CR Altorki N, 2002, ANN SURG, V236, P177, DOI 10.1097/00000658-200208000-00005

Bruns C J, 1996, Langenbecks Arch Chir Suppl Kongressbd, V113, P146

Cohen DJ, 2011, EXPERT OPIN PHARMACO, V12, P225, DOI 10.1517/14656566.2010.517748

CUE JI, 1992, J TRAUMA, V32, P613, DOI 10.1097/00005373-199205000-00013

Demeester Steven R, 2009, Gastrointest Cancer Res, V3, pS2

Dhar DK, 2000, WORLD J SURG, V24, P588, DOI 10.1007/s002689910099

Dresner SM, 2000, EUR J SURG ONCOL, V26, P492, DOI 10.1053/ejso.1999.0929

Higuchi Katsuhiko, 2009, Gastrointest Cancer Res, V3, P153

Hu Yi, 2010, Chin J Cancer, V29, P178

Jemal A, 2009, CA-CANCER J CLIN, V59, P225, DOI 10.3322/caac.20006

Kamei T, 2009, WORLD J SURG, V33, P1240, DOI 10.1007/s00268-009-9979-4

KAPLAN J, 1984, BLOOD, V64, P308

Katz SC, 2009, ANN SURG, V249, P617, DOI 10.1097/SLA.0b013e31819ed22f

Kitagawa Yuko, 2008, Nihon Geka Gakkai Zasshi, V109, P333

Kobayashi S, 2001, HEPATO-GASTROENTEROL, V48, P372

Lerut T, 2004, ANN SURG, V240, P962, DOI 10.1097/01.sla.0000145925.70409.d7

LIEBERMAN MD, 1990, J SURG RES, V48, P498, DOI 10.1016/0022-4804(90)90020-3

Mariette C, 2008, ANN SURG, V247, P365, DOI 10.1097/SLA.0b013e31815aaadf

Nagai S, 2011, PANCREAS, V40, P3, DOI 10.1097/MPA.0b013e3181f7147a

Nishihira T, 1998, AM J SURG, V175, P47, DOI 10.1016/S0002-9610(97)00227-4

OEFELEIN MG, 1995, J UROLOGY, V154, P442, DOI 10.1016/S0022-5347(01)67070-8

Ojima T, 2009, J GASTROINTEST SURG, V13, P1821, DOI 10.1007/s11605-009-0973-9

Osugi H, 2003, ONCOL REP, V10, P81

Osugi H, 2002, BRIT J SURG, V89, P909, DOI 10.1046/j.1365-2168.2002.02109.x

Peyre CG, 2008, ANN SURG, V248, P979, DOI 10.1097/SLA.0b013e3181904f3c

ROUMEN RMH, 1993, ANN SURG, V218, P769, DOI 10.1097/00000658-199312000-00011

Tachibana M, 1999, J SURG ONCOL, V72, P88, DOI 10.1002/(SICI)1096-9098(199910)72:2<88::AID-JSO9>3.0.CO;2-V

Tachibana M, 1999, ARCH SURG-CHICAGO, V134, P50, DOI 10.1001/archsurg.134.1.50

Tachibana M, 1999, AM J GASTROENTEROL, V94, P757

Takemura Masashi, 2005, Ann Thorac Cardiovasc Surg, V11, P293

TARTTER PI, 1992, ANN SURG, V216, P633, DOI 10.1097/00000658-199212000-00004

Tsurumaru M, 2001, Ann Thorac Cardiovasc Surg, V7, P325

Ydy LRA, 2007, WORLD J SURG, V31, P2044, DOI 10.1007/s00268-007-9159-3

YOUNES RN, 1991, ANN SURG, V214, P107, DOI 10.1097/00000658-199108000-00003

NR 34

TC 34

Z9 37

U1 0

U2 4

PU SPRINGER

PI NEW YORK

PA ONE NEW YORK PLAZA, SUITE 4600, NEW YORK, NY, UNITED STATES

SN 0364-2313

EI 1432-2323

J9 WORLD J SURG

JI World J.Surg.

PD APR

PY 2012

VL 36

IS 4

BP 844

EP 850

DI 10.1007/s00268-012-1433-3

PG 7

WC Surgery

WE Science Citation Index Expanded (SCI-EXPANDED)

SC Surgery

GA 909UR

UT WOS:000301591200024

PM 22350472

DA 2022-05-19

ER

PT J

AU Nomoto, D

Baba, Y

Akiyama, T

Okadome, K

Iwatsuki, M

Iwagami, S

Miyamoto, Y

Yoshida, N

Watanabe, M

Baba, H

AF Nomoto, Daichi

Baba, Yoshifumi

Akiyama, Takahiko

Okadome, Kazuo

Iwatsuki, Masaaki

Iwagami, Shiro

Miyamoto, Yuji

Yoshida, Naoya

Watanabe, Masayuki

Baba, Hideo

TI Adapted systemic inflammation score as a novel prognostic marker for

esophageal squamous cell carcinoma patients

SO ANNALS OF GASTROENTEROLOGICAL SURGERY

LA English

DT Article

DE adapted systemic inflammation score; esophageal squamous cell carcinoma;

prognosis

ID LYMPHOCYTE RATIO; CANCER; NEUTROPHIL

AB Background The adapted systemic inflammation score (aSIS), calculated from serum albumin and the lymphocyte-to-monocyte ratio, has been reported to be a novel prognostic marker for some types of cancers. However, the prognostic impact of aSIS in patients with esophageal squamous cell carcinoma (ESCC) remains controversial. This study aimed to examine the prognostic effects of aSIS in a large cohort of 509 ESCC patients.

Methods Preoperative aSIS was retrospectively calculated for 509 ESCC patients who underwent curative resection. Time-dependent receiver operating characteristics (t-ROC) curves were used for comparing the prognostic impact.

Results Patients with high aSIS showed significantly poorer overall survival (OS) than patients with low aSIS (log rank P < .001). The multivariate analysis revealed that aSIS was an independent prognostic factor for overall survival (multivariate hazard ratio 1.76; 95% confidence interval 1.13-2.75; P = .013). The t-ROC analysis showed that aSIS was more sensitive than other nutritional prognostic factors (controlling for nutritional status, systemic inflammation score, and the neutrophil-to-lymphocyte ratio).

Conclusion Preoperative aSIS may be a useful prognostic biomarker in ESCC patients who underwent curative resection.

C1 [Nomoto, Daichi; Baba, Yoshifumi; Akiyama, Takahiko; Okadome, Kazuo; Iwatsuki, Masaaki; Iwagami, Shiro; Miyamoto, Yuji; Yoshida, Naoya; Baba, Hideo] Kumamoto Univ, Grad Sch Med Sci, Dept Gastroenterol Surg, Kumamoto, Japan.

[Baba, Yoshifumi] Kumamoto Univ, Grad Sch Med Sci, Dept Next Generat Surg Therapy Dev, Kumamoto, Japan.

[Watanabe, Masayuki] Japanese Fdn Canc Res, Canc Inst Hosp, Dept Gastroenterol Surg, Tokyo, Japan.

[Baba, Hideo] Kumamoto Univ, Ctr Metab Regulat Hlth Aging, Kumamoto, Japan.

RP Baba, H (corresponding author), Kumamoto Univ, Grad Sch Med Sci, Dept Gastroenterol Surg, Chuo Ku, 1-1-1 Honjo, Kumamoto 8608556, Japan.

EM hdobaba@kumamoto-u.ac.jp

OI Miyamoto, Yuji/0000-0003-3948-5044; Watanabe,

Masayuki/0000-0003-0298-1597

CR Bray F, 2018, CA-CANCER J CLIN, V68, P394, DOI 10.3322/caac.21492

CAMP RL, 2004, CLIN CANCER RES, V10, P7252, DOI DOI 10.1158/1078-0432.CCR-04-0713

Chang Y, 2015, BRIT J CANCER, V113, P626, DOI 10.1038/bjc.2015.241

Feng YR, 2019, ONCOTARGETS THER, V12, P6617, DOI 10.2147/OTT.S213720

Fu XB, 2019, BMC CANCER, V19, DOI 10.1186/s12885-019-5940-6

Han L, 2016, DIS ESOPHAGUS, V29, P740, DOI 10.1111/dote.12401

Han LH, 2016, TUMOR BIOL, V37, P3081, DOI 10.1007/s13277-015-4152-1

Ignacio de Ulíbarri J., 2005, Nutr. Hosp., V20, P38

Kanda M, 2019, ANN SURG ONCOL, V26, P4773, DOI 10.1245/s10434-019-07914-7

Lin JX, 2019, GASTRIC CANCER, V22, P403, DOI 10.1007/s10120-018-0854-6

Ma MZ, 2019, ANZ J SURG, V89, P377, DOI 10.1111/ans.15103

Mantovani A, 2008, NATURE, V454, P436, DOI 10.1038/nature07205

McShane LM, 2005, JNCI-J NATL CANCER I, V97, P1180, DOI 10.1093/jnci/dji237

Okadome K, 2020, ANN SURG, V271, P693, DOI 10.1097/SLA.0000000000002985

Quail DF, 2013, NAT MED, V19, P1423, DOI 10.1038/nm.3394

Sato B, 2018, WORLD J SURG, V42, P3277, DOI 10.1007/s00268-018-4597-7

Sharaiha RZ, 2011, ANN SURG ONCOL, V18, P3362, DOI 10.1245/s10434-011-1754-8

Sobin L.H., 2010, TNM CLASSIFICATION M, Vseventh

Suzuki Y, 2018, ANN SURG, V267, P527, DOI 10.1097/SLA.0000000000002115

Yagi T, 2019, EUR J CANCER, V111, P38, DOI 10.1016/j.ejca.2019.01.018

Yagi T, 2019, ANN SURG, V269, P471, DOI 10.1097/SLA.0000000000002616

Yodying H, 2016, ANN SURG ONCOL, V23, P646, DOI 10.1245/s10434-015-4869-5

NR 22

TC 0

Z9 0

U1 0

U2 0

PU WILEY-V C H VERLAG GMBH

PI WEINHEIM

PA POSTFACH 101161, 69451 WEINHEIM, GERMANY

SN 2475-0328

J9 ANN GASTROENT SURG

JI Ann. Gastroent. Surg.

PD SEP

PY 2021

VL 5

IS 5

BP 669

EP 676

DI 10.1002/ags3.12464

EA JUN 2021

PG 8

WC Gastroenterology & Hepatology

WE Science Citation Index Expanded (SCI-EXPANDED)

SC Gastroenterology & Hepatology

GA US2NG

UT WOS:000661677800001

PM 34585051

OA Green Published, gold

DA 2022-05-19

ER

PT J

AU Hu, XL

Zhai, YF

Shi, RY

Qian, Y

Cui, HY

Yang, J

Bi, YH

Yan, T

Yang, J

Ma, YC

Zhang, L

Liu, YQ

Li, GD

Zhang, MS

Cui, YP

Kong, PZ

Cheng, XL

AF Hu, Xiaoling

Zhai, Yuanfang

Shi, Ruyi

Qian, Yu

Cui, Heyang

Yang, Jie

Bi, Yanghui

Yan, Ting

Yang, Jian

Ma, Yanchun

Zhang, Ling

Liu, Yiqian

Li, Guodong

Zhang, Mingsheng

Cui, Yongping

Kong, Pengzhou

Cheng, Xiaolong

TI FAT1 inhibits cell migration and invasion by affecting cellular

mechanical properties in esophageal squamous cell carcinoma

SO ONCOLOGY REPORTS

LA English

DT Article

DE esophageal squamous cell carcinoma; FAT1; migration; invasion; cell

mechanical properties

ID ATOMIC-FORCE MICROSCOPY; EPITHELIAL-MESENCHYMAL TRANSITION; GENOMIC

CHARACTERIZATION; SIGNALING PATHWAY; CANCER GENOMICS; BREAST-CANCER;

IN-SITU; CADHERIN; GENES; BIOMECHANICS

AB FAT atypical cadherin 1 (FAT1) belongs to the cadherin superfamily and has been reported to regulate cell-cell adhesion and other cell behaviors, suggesting its pivotal roles in human cancers. We previously identified FAT1 as one of the significant mutant genes in esophageal squamous cell carcinoma (ESCC). In the present study, the knockdown of FAT1 expression in YSE2 and Colo680N cell lines was carried out by lentivirus, and we found that knockdown of FAT1 led to acceleration of cell migration and invasion. Furthermore, we detected the cell adhesive force and cell elasticity force by atomic force microscopy (AFM) and found that the suppression of endogenous expression of FAT1 led to a decrease in the cell adhesive force and increase in the cell elasticity force compared with the control groups. In conclusion, our study demonstrated that FAT1 altered cellular mechanical properties leading to deregulation of cell migration and invasion of ESCC, which may be a novel target for ESCC therapy.

C1 [Hu, Xiaoling; Zhai, Yuanfang; Shi, Ruyi; Qian, Yu; Cui, Heyang; Yang, Jie; Bi, Yanghui; Yan, Ting; Yang, Jian; Ma, Yanchun; Zhang, Ling; Liu, Yiqian; Cui, Yongping; Kong, Pengzhou; Cheng, Xiaolong] Shanxi Med Univ, Translat Med Res Ctr, 56 Xin Jian Nan Rd, Taiyuan 030001, Shanxi, Peoples R China.

[Hu, Xiaoling; Zhai, Yuanfang; Shi, Ruyi; Qian, Yu; Cui, Heyang; Yang, Jie; Bi, Yanghui; Yan, Ting; Yang, Jian; Ma, Yanchun; Zhang, Ling; Liu, Yiqian; Cui, Yongping; Kong, Pengzhou; Cheng, Xiaolong] Shanxi Med Univ, Minist Educ, Key Lab Cellular Physiol, Taiyuan 030001, Shanxi, Peoples R China.

[Hu, Xiaoling; Zhang, Mingsheng] Shanxi Med Univ, Dept Pharmacol, Taiyuan 030001, Shanxi, Peoples R China.

[Zhai, Yuanfang; Cheng, Xiaolong] Shanxi Med Univ, Dept Anat, Taiyuan 030001, Shanxi, Peoples R China.

[Li, Guodong] Shanxi Prov Peoples Hosp, Dept Otorhinolaryngol, Taiyuan 030001, Shanxi, Peoples R China.

RP Kong, PZ; Cheng, XL (corresponding author), Shanxi Med Univ, Translat Med Res Ctr, 56 Xin Jian Nan Rd, Taiyuan 030001, Shanxi, Peoples R China.

EM kongpengzhou@sxmu.edu.cn; chengxl@sxmu.edu.cn

OI Qian, Yu/0000-0003-4759-4697

FU National Natural Science Foundation of China [81330063, 81672768,

81602176, 81602458, 81502135]; Natural Science Foundation of Shanxi

Province [201601D202107, 201701D221249]; Startup Foundation for Doctors

of Shanxi Medical University [03201006]; Youth Foundation of Shanxi

Medical University [057487]; Shanxi Medical University [201219]

FX The present study was supported through funding from the National

Natural Science Foundation of China (81330063 to YC, 81672768 to XC,

81602176 to RS, 81602458 to CC and 81502135 to PK), Natural Science

Foundation of Shanxi Province (201601D202107 to RS and 201701D221249 to

XH), Startup Foundation for Doctors of Shanxi Medical University

(03201006 to XH), Youth Foundation of Shanxi Medical University (057487

to XH) and The 331 Early Career Researcher Grant of Shanxi Medical

University (201219 to XH).

CR Bennett FC, 2006, CURR BIOL, V16, P2101, DOI 10.1016/j.cub.2006.09.045

Cai XF, 2009, SCANNING, V31, P83, DOI 10.1002/sca.20144

Cerami E, 2012, CANCER DISCOV, V2, P401, DOI 10.1158/2159-8290.CD-12-0095

Chen WQ, 2016, CA-CANCER J CLIN, V66, P115, DOI 10.3322/caac.21338

Cheng CX, 2016, AM J HUM GENET, V98, P256, DOI 10.1016/j.ajhg.2015.12.013

Cross SE, 2007, NAT NANOTECHNOL, V2, P780, DOI 10.1038/nnano.2007.388

Ferlay J, 2015, INT J CANCER, V136, pE359, DOI 10.1002/ijc.29210

Ferlay J, 2010, INT J CANCER, V127, P2893, DOI 10.1002/ijc.25516

Fletcher DA, 2010, NATURE, V463, P485, DOI 10.1038/nature08908

Fuchs E, 1998, SCIENCE, V279, P514, DOI 10.1126/science.279.5350.514

Gao JJ, 2013, SCI SIGNAL, V6, DOI 10.1126/scisignal.2004088

Gao YB, 2014, NAT GENET, V46, P1097, DOI 10.1038/ng.3076

Guck J, 2005, BIOPHYS J, V88, P3689, DOI 10.1529/biophysj.104.045476

Hammerman PS, 2012, NATURE, V489, P519, DOI 10.1038/nature11404

Hu XL, 2017, CANCER LETT, V397, P83, DOI 10.1016/j.canlet.2017.03.033

Katoh M, 2012, INT J ONCOL, V41, P1913, DOI 10.3892/ijo.2012.1669

Kaul-Ghanekar R, 2009, BMC CANCER, V9, DOI 10.1186/1471-2407-9-350

Khalili AA, 2015, INT J MOL SCI, V16, P18149, DOI 10.3390/ijms160818149

Kim KT, 2016, HEAD NECK-J SCI SPEC, V38, pE2021, DOI 10.1002/hed.24372

Kumar S, 2009, CANCER METAST REV, V28, P113, DOI 10.1007/s10555-008-9173-4

Lee GYH, 2007, TRENDS BIOTECHNOL, V25, P111, DOI 10.1016/j.tibtech.2007.01.005

Lee S, 2012, CANCER RES, V72, P4574, DOI 10.1158/0008-5472.CAN-12-0636

Lian SZ, 2016, ONCOL REP, V36, P984, DOI 10.3892/or.2016.4847

Lin DC, 2014, NAT GENET, V46, P467, DOI 10.1038/ng.2935

Morris LGT, 2013, NAT GENET, V45, P253, DOI 10.1038/ng.2538

Pennathur A, 2013, LANCET, V381, P400, DOI 10.1016/S0140-6736(12)60643-6

Pickering CR, 2013, CANCER DISCOV, V3, P770, DOI 10.1158/2159-8290.CD-12-0537

Reddy BVVG, 2008, DEVELOPMENT, V135, P2827, DOI 10.1242/dev.020974

Sadeqzadeh E, 2011, J BIOL CHEM, V286, P28181, DOI 10.1074/jbc.M111.234419

Settakorn J, 2005, J CLIN PATHOL, V58, P1249, DOI 10.1136/jcp.2005.026575

Shi RY, 2015, ONCOL LETT, V9, P2249, DOI 10.3892/ol.2015.2982

Shi XL, 2012, NANOMEDICINE-UK, V7, P1625, DOI [10.2217/NNM.12.130, 10.2217/nnm.12.130]

Siamantouras E, 2016, NANOMED-NANOTECHNOL, V12, P1013, DOI 10.1016/j.nano.2015.12.362

Song YM, 2014, NATURE, V509, P91, DOI 10.1038/nature13176

Suresh S, 2007, ACTA BIOMATER, V3, P413, DOI 10.1016/j.actbio.2007.04.002

Tang J, 2015, CLIN CHIM ACTA, V444, P264, DOI 10.1016/j.cca.2015.02.042

Tanoue T, 2005, J CELL SCI, V118, P2347, DOI 10.1242/jcs.02398

Valletta D, 2014, CARCINOGENESIS, V35, P1407, DOI 10.1093/carcin/bgu054

Wang DD, 2012, MOL MED REP, V6, P591, DOI 10.3892/mmr.2012.945

Wheeler JB, 2012, SURG CLIN N AM, V92, P1077, DOI 10.1016/j.suc.2012.07.008

Yao YF, 2016, MOL CARCINOGEN, V55, P1714, DOI 10.1002/mc.22421

Zhang F, 2016, CELL PHYSIOL BIOCHEM, V40, P172, DOI 10.1159/000452535

Zhang L, 2015, AM J HUM GENET, V96, P597, DOI 10.1016/j.ajhg.2015.02.017

Zhang L, 2014, BIOSENS BIOELECTRON, V56, P271, DOI 10.1016/j.bios.2014.01.024

NR 44

TC 15

Z9 16

U1 4

U2 14

PU SPANDIDOS PUBL LTD

PI ATHENS

PA POB 18179, ATHENS, 116 10, GREECE

SN 1021-335X

EI 1791-2431

J9 ONCOL REP

JI Oncol. Rep.

PD MAY

PY 2018

VL 39

IS 5

BP 2136

EP 2146

DI 10.3892/or.2018.6328

PG 11

WC Oncology

WE Science Citation Index Expanded (SCI-EXPANDED)

SC Oncology

GA GB6TG

UT WOS:000429205600009

PM 29565465

OA Green Submitted, Green Published, hybrid

DA 2022-05-19

ER

PT J

AU Song, L

Wang, XH

Feng, Z

AF Song, Liang

Wang, Xiaohang

Feng, Zhen

TI Overexpression of FOXM1 as a target for malignant progression of

esophageal squamous cell carcinoma

SO ONCOLOGY LETTERS

LA English

DT Article

DE esophageal squamous cell carcinoma; forkhead box protein M1; short

interfering RNA; targeted therapy

ID CANCER

AB Esophageal squamous cell carcinoma (ESCC) is one of the most common types of malignancies globally. Forkhead box M1 (FOXM1) has important functions in cancer progression. However, the function of FOXM1 signaling in ESCC remains unknown. The aim of the present study was to evaluate the expression level and function of FOXM1 in human ESCC. The present study first detected the FOXM1 expression level in 78 cases of primary ESCC tissues and matched normal tissue samples by immunohistochemistry, western blot analysis and reverse transcription-quantitative polymerase chain reaction. Subsequently, the present study investigated the impact of FOXM1 knockdown on the ability of cell proliferation and migration of ESCC cells by MTT, clonogenic and Transwell assays. The results revealed that the mRNA and protein expression levels of FOXM1 were upregulated in a series of ESCC tissue samples. Silencing of FOXM1 inhibited the proliferation and migration of ESCC cells. In conclusion, FOXM1 was significantly increased in cancerous tissue samples of patients with ESCC. It may serve as a selective target for the treatment of ESCC.

C1 [Song, Liang; Wang, Xiaohang; Feng, Zhen] Shandong Univ, Shandong Prov Hosp, Dept Thorac Surg, 324 Jingwuweiqi Rd, Jinan 250021, Shandong, Peoples R China.

RP Wang, XH (corresponding author), Shandong Univ, Shandong Prov Hosp, Dept Thorac Surg, 324 Jingwuweiqi Rd, Jinan 250021, Shandong, Peoples R China.

EM drwangxh666@163.com

CR Ahmed M, 2012, J CLIN ENDOCR METAB, V97, pE1, DOI 10.1210/jc.2011-1506

Balli D, 2011, CANCER RES, V71, P40, DOI 10.1158/0008-5472.CAN-10-2004

Bray F, 2013, INT J CANCER, V132, P1133, DOI 10.1002/ijc.27711

Calvisi DF, 2009, GUT, V58, P679, DOI 10.1136/gut.2008.152652

Chai JY, 2012, WORLD J GASTROENTERO, V18, P6521, DOI 10.3748/wjg.v18.i45.6521

Ferlay J, 2010, INT J CANCER, V127, P2893, DOI 10.1002/ijc.25516

Laoukili J, 2007, BBA-REV CANCER, V1775, P92, DOI 10.1016/j.bbcan.2006.08.006

Livak KJ, 2001, METHODS, V25, P402, DOI 10.1006/meth.2001.1262

Raychaudhuri P, 2011, CANCER RES, V71, P4329, DOI 10.1158/0008-5472.CAN-11-0640

Sun HC, 2011, CANCER LETT, V306, P214, DOI 10.1016/j.canlet.2011.03.009

Tejani MA, 2012, CURR TREAT OPTION ON, V13, P390, DOI 10.1007/s11864-012-0193-5

Yu JS, 2011, CLIN CANCER RES, V17, P1924, DOI 10.1158/1078-0432.CCR-10-1551

NR 12

TC 12

Z9 12

U1 0

U2 0

PU SPANDIDOS PUBL LTD

PI ATHENS

PA POB 18179, ATHENS, 116 10, GREECE

SN 1792-1074

EI 1792-1082

J9 ONCOL LETT

JI Oncol. Lett.

PD APR

PY 2018

VL 15

IS 4

BP 5910

EP 5914

DI 10.3892/ol.2018.8035

PG 5

WC Oncology

WE Science Citation Index Expanded (SCI-EXPANDED)

SC Oncology

GA GD9HA

UT WOS:000430822500247

PM 29552222

OA Green Published, gold, Green Submitted

DA 2022-05-19

ER

PT J

AU Kong, DM

Long, DL

Liu, B

Pei, DK

Cao, N

Zhang, GH

Xia, ZK

Luo, M

AF Kong, Demiao

Long, Dali

Liu, Bo

Pei, Dengke

Cao, Na

Zhang, Guihua

Xia, Zhenkun

Luo, Meng

TI Downregulation of long non-coding RNA LOC101928477 correlates with tumor

progression by regulating the epithelial-mesenchymal transition in

esophageal squamous cell carcinoma

SO THORACIC CANCER

LA English

DT Article

DE epithelial&#8208; mesenchymal transition; esophageal squamous cell

carcinoma; LOC101928477

AB Background Esophageal squamous cell carcinoma (ESCC) is one of the deadliest malignancies. There is a growing body of evidence showing that long non-coding RNAs (lncRNAs) play critical roles in ESCC oncogenesis. The present study aimed to explore the role of LOC101928477, a newly discovered lncRNA, in the development and metastasis of ESCC.

Methods In this study, real-time PCR, western blotting, cell counting kit-8 (CCK-8), flow cytometry, colony formation, wound healing, transwell migration/invasion assay, immunofluorescence, and immunohistochemistry were used. We also applied an in situ xenograft mouse model and a lung metastasis mouse model to verify our findings.

Results We determined that LOC101928477 expression was inhibited in ESCC tissue and ESCC cell lines when compared with controls. Moreover, forced expression of LOC101928477 effectively inhibited ESCC cell proliferation, colony formation, migration, and invasion via suppression of epithelial-mesenchymal transition (EMT). Furthermore, LOC101928477 overexpression inhibited in situ tumor growth and lung metastasis in a mouse model.

Conclusions Together, our results suggested that LOC101928477 could be a novel suppressor gene involved in ESCC progression.

C1 [Kong, Demiao; Liu, Bo; Pei, Dengke; Zhang, Guihua; Xia, Zhenkun; Luo, Meng] Guizhou Prov Peoples Hosp, Dept Thorac Surg, 83 East Zhongshan Rd, Guiyang 550001, Guizhou, Peoples R China.

[Long, Dali] Guizhou Prov Peoples Hosp, Dept Intens Care Unit, Guiyang, Peoples R China.

[Cao, Na] Guizhou Prov Peoples Hosp, Dept Logist, Guiyang, Guizhou, Peoples R China.

RP Luo, M (corresponding author), Guizhou Prov Peoples Hosp, Dept Thorac Surg, 83 East Zhongshan Rd, Guiyang 550001, Guizhou, Peoples R China.

EM luomeng1225@163.com

FU National Natural Science Foundation of China [81301810]; Guizhou

Highlevel Talents Training Program [GZSYQCC [2014] 003]

FX the National Natural Science Foundation of China, Grant/Award Number:

81301810; Guizhou Highlevel Talents Training Program, Grant/Award

Number: GZSYQCC [2014] 003

CR Chen S, 2017, CELL DEATH DIS, V8, DOI 10.1038/cddis.2017.486

Chen ZY, 2018, EXP CELL RES, V362, P90, DOI 10.1016/j.yexcr.2017.11.006

Cui YB, 2018, ONCOTARGET, V9, P783, DOI 10.18632/oncotarget.23153

Dai FQ, 2017, GENOMICS, V109, P241, DOI 10.1016/j.ygeno.2017.04.005

Demeester Steven R, 2009, Gastrointest Cancer Res, V3, pS2

Feng FY, 2017, ONCOTARGET, V8, P29091, DOI 10.18632/oncotarget.16196

Feng YF, 2018, CELL DEATH DIS, V9, DOI 10.1038/s41419-018-0979-x

Guo J, 2017, ONCOL RES, V25, P1189, DOI 10.3727/096504017X14865126670075

Guo QY, 2016, BIOMED PHARMACOTHER, V82, P589, DOI 10.1016/j.biopha.2016.02.049

Guohong Zhang, 2010, PLoS One, V5, pe9668, DOI 10.1371/journal.pone.0009668

Hong YL, 2018, INT J ONCOL, V52, P901, DOI 10.3892/ijo.2018.4247

Jin YJ, 2017, VIROL J, V14, DOI 10.1186/s12985-017-0903-5

Kang M, 2018, J EXP CLIN CANC RES, V37, DOI 10.1186/s13046-018-0845-9

Kong YG, 2018, GENE, V639, P77, DOI 10.1016/j.gene.2017.10.006

Kudo-Saito C, 2009, CANCER CELL, V15, P195, DOI 10.1016/j.ccr.2009.01.023

Lan XT, 2019, J CELL MOL MED, V23, P2351, DOI 10.1111/jcmm.13497

Li ZW, 2017, CELL DEATH DIS, V8, DOI 10.1038/cddis.2017.245

Liang Y, 2018, CELL DEATH DIFFER, V25, P1980, DOI 10.1038/s41418-018-0084-9

Liu, 2021, THORAC CANCER, P1

Liu RM, 2018, CANCER LETT, V412, P289, DOI 10.1016/j.canlet.2017.10.018

Pan YF, 2017, BIOMED PHARMACOTHER, V87, P669, DOI 10.1016/j.biopha.2017.01.018

Qin Y, 2017, ACTA BIOCH BIOPH SIN, V49, P138, DOI 10.1093/abbs/gmw127

Shi XJ, 2016, ONCOTARGET, V7, P36407, DOI 10.18632/oncotarget.9239

Tamaoki M, 2018, CANCER SCI, V109, P1121, DOI 10.1111/cas.13531

Thiery JP, 2009, CELL, V139, P871, DOI 10.1016/j.cell.2009.11.007

Upadhyay P, 2017, ORAL ONCOL, V73, P56, DOI 10.1016/j.oraloncology.2017.08.003

Wang L, 2018, INT J ONCOL, V52, P861, DOI 10.3892/ijo.2018.4253

Wang W, 2016, ONCOTARGET, V7, P25668, DOI 10.18632/oncotarget.8257

Wen SW, 2018, DIGEST DIS SCI, V63, P2285, DOI 10.1007/s10620-018-5119-z

Wu YY, 2017, MOL CANCER, V16, DOI 10.1186/s12943-017-0715-7

Xie FJ, 2011, BMC CANCER, V11, DOI 10.1186/1471-2407-11-86

Xin ZW, 2018, THORAC CANCER, V9, P1687, DOI 10.1111/1759-7714.12883

Ye YB, 2018, J CELL BIOCHEM, V119, P2951, DOI 10.1002/jcb.26509

Yu XF, 2017, EBIOMEDICINE, V26, P100, DOI 10.1016/j.ebiom.2017.10.030

Zang WQ, 2016, ONCOTARGET, V7, P19960, DOI 10.18632/oncotarget.6963

Zhang C, 2017, ONCOL REP, V37, P2942, DOI 10.3892/or.2017.5539

Zhang CY, 2016, CELL BIOL TOXICOL, V32, P391, DOI 10.1007/s10565-016-9341-1

Zhang HF, 2015, ONCOTARGET, V6, P6218, DOI 10.18632/oncotarget.3358

Zhang W, 2017, ONCOGENE, V36, P3890, DOI 10.1038/onc.2017.15

Zheng XW, 2018, J CELL BIOCHEM, V119, P3247, DOI 10.1002/jcb.26481

Zhou M, 2017, BIOMED PHARMACOTHER, V85, P348, DOI 10.1016/j.biopha.2016.11.035

Zhu GQ, 2017, MOL CARCINOGEN, V56, P2210, DOI 10.1002/mc.22674

Zhu HC, 2017, RADIOTHER ONCOL, V124, P439, DOI 10.1016/j.radonc.2017.04.001

Zhu WY, 2017, MOL MED REP, V16, P4694, DOI 10.3892/mmr.2017.7175

NR 44

TC 1

Z9 1

U1 1

U2 1

PU WILEY

PI HOBOKEN

PA 111 RIVER ST, HOBOKEN 07030-5774, NJ USA

SN 1759-7706

EI 1759-7714

J9 THORAC CANCER

JI Thorac. Cancer

PD MAY

PY 2021

VL 12

IS 9

BP 1303

EP 1311

DI 10.1111/1759-7714.13858

EA MAR 2021

PG 9

WC Oncology; Respiratory System

WE Science Citation Index Expanded (SCI-EXPANDED)

SC Oncology; Respiratory System

GA RV7PG

UT WOS:000628329800001

PM 33713583

OA Green Published, gold

DA 2022-05-19

ER

PT J

AU Li, CC

Fang, HY

Lin, CY

Shen, WC

Chien, CR

AF Li, Chia-Chin

Fang, Hsin-Yuan

Lin, Chen-Yuan

Shen, Wei-Chih

Chien, Chun-Ru

TI Outcomes of Localized Esophageal Squamous Cell Carcinoma Patients

Treated With Definitive Concurrent Chemoradiotherapy Using Either

Standard or High Radiotherapy Dose: A Retrospective Study Controlling

for Organ at Risk Dose

SO ANTICANCER RESEARCH

LA English

DT Article

DE Esophageal squamous cell carcinoma; concurrent chemoradiotherapy;

radiotherapy dose

ID RESPONSE RELATIONSHIP; RADIATION-THERAPY; CANCER; STAGE; CHEMORADIATION;

CHEMOTHERAPY; GUIDELINES

AB Background/Aim: The optimal radiotherapy dose for localized esophageal squamous cell carcinoma (ESqCC) patients treated with definitive concurrent chemoradiotherapy (CCRT) is debated. The aim of our study was to compare patient outcomes using either standard or high radiotherapy dose. Materials and Methods: Eligible patients diagnosed between 2011 and 2015 from the cancer registry of our Institute were identified and a propensity score (PS)matched cohort (1: 1 for high vs. standard dose) was constructed to balance observable potential confounders (including organ at risk dose). The hazard ratio (HR) of death between high and standard dose was compared. Results: Our study population included 73/36 patients before/after PS matching. The HR of death at the high dose compared to the standard dose was 0.554 (95% confidence interval (CI)=0.308-0.998, p=0.049). Conclusion: Definitive CCRT using a high radiotherapy dose showed improved survival outcomes for localized ESqCC patients compared to standard dose.

C1 [Li, Chia-Chin; Chien, Chun-Ru] China Med Univ Hosp, Dept Radiat Oncol, Taichung, Taiwan.

[Fang, Hsin-Yuan] China Med Univ Hosp, Dept Chest Surg, Taichung, Taiwan.

[Fang, Hsin-Yuan; Chien, Chun-Ru] China Med Univ, Coll Med, Sch Med, 91 Hsueh Shih Rd, Taichung 40402, Taiwan.

[Lin, Chen-Yuan] China Med Univ Hosp, Div Hematol & Oncol, Taichung, Taiwan.

[Shen, Wei-Chih] Asia Univ, Dept Comp Sci & Informat Engn, Taichung, Taiwan.

[Chien, Chun-Ru] China Med Univ, Hsinchu Hosp, Dept Radiat Oncol, Hsinchu, Taiwan.

RP Chien, CR (corresponding author), China Med Univ, Coll Med, Sch Med, 91 Hsueh Shih Rd, Taichung 40402, Taiwan.

EM d16181@gmail.com

FU Ministry of Science and Technology [MOST 107-2314-B-039-026-]; China

Medical University Hospital [DMR-108-054]

FX This work was partly supported by Ministry of Science and Technology

(MOST 107-2314-B-039-026-) and China Medical University Hospital

(DMR-108-054). The corresponding Author would like to thank Dr. Ya Chen

Tina Shih for her mentoring in health services research.

CR Ali MS, 2015, J CLIN EPIDEMIOL, V68, P122, DOI 10.1016/j.jclinepi.2014.08.011

[Anonymous], 2018, NATL COMPREHENSIVE C

[Anonymous], 2011, NATL COMPREHENSIVE C

Austin PC, 2014, STAT MED, V33, P1242, DOI 10.1002/sim.5984

Chen CY, 2018, WORLD J SURG ONCOL, V16, DOI 10.1186/s12957-018-1444-0

Chen CY, 2016, RADIOTHER ONCOL, V120, P136, DOI 10.1016/j.radonc.2016.04.042

Chien CR, 2009, JNCI-J NATL CANCER I, V101, P1428, DOI 10.1093/jnci/djp304

Deist TM, 2018, MED PHYS, V45, P3449, DOI 10.1002/mp.12967

Deng W, 2018, ANN TRANSL MED, V6, DOI 10.21037/atm.2017.11.28

Fan BJ, 2017, ONCOTARGET, V8, P34498, DOI 10.18632/oncotarget.15606

Ferlay J, 2015, INT J CANCER, V136, pE359, DOI 10.1002/ijc.29210

Garrido MM, 2014, HEALTH SERV RES, V49, P1701, DOI 10.1111/1475-6773.12182

Jackson John W, 2017, Curr Epidemiol Rep, V4, P271, DOI 10.1007/s40471-017-0131-y

Jagsi R, 2014, INT J RADIAT ONCOL, V90, P11, DOI 10.1016/j.ijrobp.2014.05.013

Kim HJ, 2017, CANCER RES TREAT, V49, P669, DOI 10.4143/crt.2016.354

Kuwano H, 2015, ESOPHAGUS-TOKYO, V12, P1, DOI 10.1007/s10388-014-0465-1

LI CC, 2018, MEDICINE, V97

Li CC, 2016, ONCOTARGET, V7, P71548, DOI 10.18632/oncotarget.12250

Lin CY, 2016, THORAC CANCER, V7, P288, DOI 10.1111/1759-7714.12326

Lordick F, 2016, ANN ONCOL, V27, pv50, DOI 10.1093/annonc/mdw329

Matloff N., 2017, STAT REGRESSION CLAS

Minsky BD, 2002, J CLIN ONCOL, V20, P1167, DOI 10.1200/JCO.20.5.1167

Pennathur A, 2013, LANCET, V381, P400, DOI 10.1016/S0140-6736(12)60643-6

Stahl M, 2013, ANN ONCOL, V24, P51, DOI 10.1093/annonc/mdt342

Suh YG, 2014, JPN J CLIN ONCOL, V44, P534, DOI 10.1093/jjco/hyu047

Urschel JD, 2003, DIS ESOPHAGUS, V16, P60, DOI 10.1046/j.1442-2050.2003.00296.x

VanderWeele TJ, 2017, ANN INTERN MED, V167, P268, DOI 10.7326/M16-2607

von Elm E, 2014, INT J SURG, V12, P1495, DOI 10.1016/j.ijsu.2014.07.013

Xu YJ, 2018, J CLIN ONCOL, V36, DOI 10.1200/JCO.2018.36.15_suppl.4013

Yamoah K, 2015, INT J RADIAT ONCOL, V93, P737, DOI 10.1016/j.ijrobp.2015.07.2284

Yao XXI, 2017, JNCI-J NATL CANCER I, V109, DOI 10.1093/jnci/djw323

Yuan H, 2016, NUCL MED COMMUN, V37, P947, DOI 10.1097/MNM.0000000000000527

Zhang Z, 2005, INT J RADIAT ONCOL, V61, P656, DOI 10.1016/j.ijrobp.2004.06.022

NR 33

TC 6

Z9 6

U1 0

U2 4

PU INT INST ANTICANCER RESEARCH

PI ATHENS

PA EDITORIAL OFFICE 1ST KM KAPANDRITIOU-KALAMOU RD KAPANDRITI, PO BOX 22,

ATHENS 19014, GREECE

SN 0250-7005

EI 1791-7530

J9 ANTICANCER RES

JI Anticancer Res.

PD JAN

PY 2019

VL 39

IS 1

BP 511

EP 517

DI 10.21873/anticanres.13142

PG 7

WC Oncology

WE Science Citation Index Expanded (SCI-EXPANDED)

SC Oncology

GA HF7IS

UT WOS:000454413400069

PM 30591503

OA Bronze

DA 2022-05-19

ER

PT J

AU Takemoto, A

Tanimoto, K

Mori, S

Inoue, J

Fujiwara, N

Noda, T

Inazawa, J

AF Takemoto, Akira

Tanimoto, Kousuke

Mori, Seiichi

Inoue, Jun

Fujiwara, Naoto

Noda, Tetsuo

Inazawa, Johji

TI Integrative genome-wide analyses reveal the transcriptional aberrations

in Japanese esophageal squamous cell carcinoma

SO CANCER SCIENCE

LA English

DT Article

DE allelic expression imbalance; DNA methylation; esophageal squamous cell

carcinoma; imprinted gene; integrative analysis

ID GENETIC LANDSCAPE; ALLELIC IMBALANCE; DNA METHYLATION; CANCER;

MUTATIONS; EXPRESSION; PROLIFERATION; SIGNATURES; PEG1/MEST; IGF2

AB Esophageal squamous cell carcinoma (ESCC) is a malignant disease. At present, the genomic profiles of ESCC are known to a considerable extent, and DNA methylation and gene expression profiles have been mainly used for the classification of ESCC subtypes, but integrative genomic, transcriptomic, and epigenomic analyses remain insufficient. Therefore, we performed integrative analyses using whole-exome sequencing, DNA methylation, and RNA sequencing (RNA-seq) analyses of Japanese patients with ESCC. In cancer-related genes, such as NOTCH family genes, RTK/PI3K pathway genes, and NFE2L2 pathway genes, variants and copy number amplification were detected frequently. Japanese ESCC cases were clustered into two mutational signatures: an APOBEC-associated signature and an age-related signature. In imprinted genes, DNA methylation was aberrant in gene promoter regions and correlated well with gene expression profiles. Nonsynonymous single-nucleotide variants and allelic expression imbalance were detected frequently in FAT family genes. Our integrative genome-wide analyses, including DNA methylation and allele-specific gene expression profiles, revealed altered gene regulation of imprinted genes and FAT family genes in ESCC.

C1 [Takemoto, Akira; Inazawa, Johji] Tokyo Med & Dent Univ TMDU Yushima, Bioresource Res Ctr, Tokyo, Japan.

[Tanimoto, Kousuke] TMDU, Med Res Inst, Genome Lab, Tokyo, Japan.

[Tanimoto, Kousuke] TMDU, Res Core, Gen Res Support Unit, Tokyo, Japan.

[Mori, Seiichi] Japanese Fdn Canc Res JFCR, Div Canc Gen, Inst Canc, Tokyo, Japan.

[Inoue, Jun; Inazawa, Johji] TMDU, Med Res Inst, Dept Mol Cytogenet, Tokyo, Japan.

[Fujiwara, Naoto] TMDU, Dept Gastrointestinal Surg, Tokyo, Japan.

[Noda, Tetsuo] Japanese Fdn Canc Res JFCR, Canc Inst, Tokyo, Japan.

RP Tanimoto, K; Inazawa, J (corresponding author), Tokyo Med & Dent Univ TMDU, Unkyo Ku, 1-5-45 Yushima, Tokyo 1138519, Japan.

EM ktani.nri@mri.tmd.ac.jp; johinaz.cgen@mri.tmd.ac.jp

OI Tanimoto, Kousuke/0000-0002-0826-2940

FU Conquering cancer through NEO-dimensional systems understandings

[15H05908]; Japan Agency for Medical Research and Development (AMED);

Nanken-Kyoten, TMDU; [16K14630]

FX Grants--in--Aid for Scientific Research, Grant/Award Number: 16K14630;

Conquering cancer through NEO-dimensional systems understandings,

Grant/Award Number: 15H05908; Japan Agency for Medical Research and

Development (AMED); Nanken-Kyoten, TMDU

CR Agrawal N, 2011, SCIENCE, V333, P1154, DOI 10.1126/science.1206923

Aihara T, 1998, HEPATOLOGY, V28, P86, DOI 10.1002/hep.510280113

Alexandrov LB, 2013, NATURE, V500, P415, DOI 10.1038/nature12477

Baba Y, 2013, SURG TODAY, V43, P1355, DOI 10.1007/s00595-012-0451-y

Babak T, 2015, NAT GENET, V47, P544, DOI 10.1038/ng.3274

Chen XW, 2008, HUM MOL GENET, V17, P1336, DOI 10.1093/hmg/ddn022

Chosdol K, 2009, BMC CANCER, V9, DOI 10.1186/1471-2407-9-5

Cui HM, 2002, CANCER RES, V62, P6442

Dutta M, 2020, PEERJ, V8, DOI 10.7717/peerj.9294

Ferlay J, 2010, INT J CANCER, V127, P2893, DOI 10.1002/ijc.25516

Friedenreich CM, 2002, INT J EPIDEMIOL, V31, P86, DOI 10.1093/ije/31.1.86

Gao YB, 2014, NAT GENET, V46, P1097, DOI 10.1038/ng.3076

Hosokawa Y, 1998, GENE CHROMOSOME CANC, V22, P66, DOI 10.1002/(SICI)1098-2264(199805)22:1<66::AID-GCC9>3.0.CO;2-5

Inoue A, 2017, NATURE, V547, P419, DOI 10.1038/nature23262

Kaneda A, 2007, P NATL ACAD SCI USA, V104, P20926, DOI 10.1073/pnas.0710359105

Katoh M, 2012, INT J ONCOL, V41, P1913, DOI 10.3892/ijo.2012.1669

Kim J, 2017, NATURE, V541, P169, DOI 10.1038/nature20805

Lin DC, 2014, NAT GENET, V46, P467, DOI 10.1038/ng.2935

Lin YS, 2013, J EPIDEMIOL, V23, P233, DOI 10.2188/jea.JE20120162

Love C, 2012, NAT GENET, V44, P1321, DOI 10.1038/ng.2468

Matsuura N, 2021, CANCER MED-US, V10, P3986, DOI 10.1002/cam4.3933

McKenna A, 2010, GENOME RES, V20, P1297, DOI 10.1101/gr.107524.110

Milani L, 2007, NUCLEIC ACIDS RES, V35, DOI 10.1093/nar/gkl1152

Morris LGT, 2013, NAT GENET, V45, P253, DOI 10.1038/ng.2538

Nagata H, 2017, ONCOTARGET, V8, P37740, DOI 10.18632/oncotarget.17147

Nakanishi H, 2004, ONCOL REP, V12, P1273

Nomoto S, 1998, CANCER RES, V58, P1380

Ntziachristos P, 2014, CANCER CELL, V25, P318, DOI 10.1016/j.ccr.2014.02.018

Pancewicz J, 2010, P NATL ACAD SCI USA, V107, P16619, DOI 10.1073/pnas.1010722107

Pedersen IS, 1999, CANCER RES, V59, P5449

Pennathur A, 2013, LANCET, V381, P400, DOI 10.1016/S0140-6736(12)60643-6

Qizilbash N, 1995, LANCET, V346, P1647

Roberts SA, 2013, NAT GENET, V45, P970, DOI 10.1038/ng.2702

Robinson JT, 2017, CANCER RES, V77, pE31, DOI 10.1158/0008-5472.CAN-17-0337

Robinson JT, 2011, NAT BIOTECHNOL, V29, P24, DOI 10.1038/nbt.1754

Sawada G, 2016, GASTROENTEROLOGY, V150, P1171, DOI 10.1053/j.gastro.2016.01.035

Song YM, 2014, NATURE, V509, P91, DOI 10.1038/nature13176

Stransky N, 2011, SCIENCE, V333, P1157, DOI 10.1126/science.1208130

Thorvaldsdottir H, 2013, BRIEF BIOINFORM, V14, P178, DOI 10.1093/bib/bbs017

Tuch BB, 2010, PLOS ONE, V5, DOI 10.1371/journal.pone.0009317

Wang NJ, 2011, P NATL ACAD SCI USA, V108, P17761, DOI 10.1073/pnas.1114669108

Weng AP, 2004, SCIENCE, V306, P269, DOI 10.1126/science.1102160

Yokoyama A, 2019, NATURE, V565, P312, DOI 10.1038/s41586-018-0811-x

Zhang L, 2015, AM J HUM GENET, V96, P597, DOI 10.1016/j.ajhg.2015.02.017

Zhao RH, 2009, CARCINOGENESIS, V30, P2117, DOI 10.1093/carcin/bgp254

Zhou X, 2016, NAT GENET, V48, P4, DOI 10.1038/ng.3466

NR 46

TC 0

Z9 0

U1 0

U2 4

PU WILEY

PI HOBOKEN

PA 111 RIVER ST, HOBOKEN 07030-5774, NJ USA

SN 1347-9032

EI 1349-7006

J9 CANCER SCI

JI Cancer Sci.

PD OCT

PY 2021

VL 112

IS 10

BP 4377

EP 4392

DI 10.1111/cas.15063

EA AUG 2021

PG 16

WC Oncology

WE Science Citation Index Expanded (SCI-EXPANDED)

SC Oncology

GA UZ9DZ

UT WOS:000684153900001

PM 34263978

OA Green Published, gold

DA 2022-05-19

ER

PT J

AU Wang, MQ

Liu, D

Huang, YCX

Jiang, ZY

Wu, F

Cen, Y

Ma, L

AF Wang, Meiqi

Liu, Dan

Huang, Yunchuanxiang

Jiang, Ziyi

Wu, Feng

Cen, Yu

Ma, Lan

TI Identification of Key Genes Related to the Prognosis of Esophageal

Squamous Cell Carcinoma Based on Chip Re-Annotation

SO APPLIED SCIENCES-BASEL

LA English

DT Article

DE esophageal squamous cell carcinoma; chip re-annotation; DEGs;

bioinformatics analyses; prognostic biomarkers

AB Esophageal cancer (EC) is one of the deadliest cancers worldwide. However, reliable biomarkers for early diagnosis, or those for the prognosis of therapy, remain unfulfilled goals for its subtype esophageal squamous cell carcinoma (ESCC). The purpose of this study was to identify reliable biomarkers for the diagnosis and prognosis of ESCC by gene chip re-annotation technique and downstream bioinformatics analysis. In our research, the GSE53624 dataset was downloaded from the GEO database. Then, we reannotated the gene expression probe and obtained the gene expression matrix. Differential expressed genes (DEGs) were found by R packages and they were subjected to Gene Ontology enrichment analysis and protein-protein interaction (PPI) network construction. As a result, a total of 28,885 mRNA probes were reannotated, among which 210 down-regulated and 80 up-regulated DEGs were screened out. By combining these genes set in clinical prognosis information and Western blot analysis, we found four genes with diagnostic and prognostic significance, including MMP13, SPP1, MMP10, and COL1A1. Furthermore, markers of infiltrating immune cells exhibited different DEG-related immune infiltration patterns.

C1 [Wang, Meiqi; Ma, Lan] Tsinghua Univ, Dept Chem, Beijing 100084, Peoples R China.

[Wang, Meiqi; Ma, Lan] Tsinghua Univ, State Key Lab Chem Oncogen, Tsinghua Shenzhen Int Grad Sch, Shenzhen 518055, Peoples R China.

[Wang, Meiqi; Liu, Dan; Huang, Yunchuanxiang; Jiang, Ziyi; Wu, Feng; Cen, Yu; Ma, Lan] Tsinghua Univ, Inst Biopharmaceut & Hlth Engn, Tsinghua Shenzhen Int Grad Sch, Shenzhen 518055, Peoples R China.

[Huang, Yunchuanxiang; Ma, Lan] Tsinghua Univ, Tsinghua Berkeley Shenzhen Inst, Shenzhen 518055, Peoples R China.

[Wu, Feng; Ma, Lan] Tsinghua Univ, Sch Life Sci, Beijing 100084, Peoples R China.

[Ma, Lan] Shenzhen Bay Lab, Inst Biomed Hlth Technol & Engn, Shenzhen 518132, Peoples R China.

RP Ma, L (corresponding author), Tsinghua Univ, Dept Chem, Beijing 100084, Peoples R China.; Ma, L (corresponding author), Tsinghua Univ, State Key Lab Chem Oncogen, Tsinghua Shenzhen Int Grad Sch, Shenzhen 518055, Peoples R China.; Ma, L (corresponding author), Tsinghua Univ, Inst Biopharmaceut & Hlth Engn, Tsinghua Shenzhen Int Grad Sch, Shenzhen 518055, Peoples R China.; Ma, L (corresponding author), Tsinghua Univ, Tsinghua Berkeley Shenzhen Inst, Shenzhen 518055, Peoples R China.; Ma, L (corresponding author), Tsinghua Univ, Sch Life Sci, Beijing 100084, Peoples R China.; Ma, L (corresponding author), Shenzhen Bay Lab, Inst Biomed Hlth Technol & Engn, Shenzhen 518132, Peoples R China.

EM wmq18@mails.tsinghua.edu.cn; danieliu091@aliyun.com;

hycx19@mails.tsinghua.edu.cn; jiang-zy20@mails.tsinghua.edu.cn;

wf19@mails.tsinghua.edu.cn; cen.yu@sz.tsinghua.edu.cn;

malan@sz.tsinghua.edu.cn

FU Shenzhen Strategic Emerging Industry Development Special Funds

[JCYJ20170816143646446]; Shenzhen Science and Technology Research and

Development Funds [JCYJ20200109143018683]

FX This research was funded by Shenzhen Strategic Emerging Industry

Development Special Funds, grant number JCYJ20170816143646446, and

Shenzhen Science and Technology Research and Development Funds, grant

number JCYJ20200109143018683.

CR Ahmed M, 2010, MOL CANCER, V9, DOI 10.1186/1476-4598-9-101

Awerkiew S, 2003, MED MICROBIOL IMMUN, V192, P137, DOI 10.1007/s00430-002-0128-z

Bray F, 2020, CA-CANCER J CLIN, V70, P313, DOI 10.3322/caac.21609

Chen PF, 2019, J CELL BIOCHEM, V120, P9117, DOI 10.1002/jcb.28187

Chen WQ, 2016, CA-CANCER J CLIN, V66, P115, DOI 10.3322/caac.21338

Chin CH, 2014, BMC SYST BIOL, V8, DOI 10.1186/1752-0509-8-S4-S11

Chiu TJ, 2018, BIOMED RES INT, V2018, DOI 10.1155/2018/9098215

Davis CA, 2018, NUCLEIC ACIDS RES, V46, pD794, DOI 10.1093/nar/gkx1081

De Silva RA, 2012, CANCER IMMUNOL IMMUN, V61, P581, DOI 10.1007/s00262-012-1205-9

Dunham I, 2012, NATURE, V489, P57, DOI 10.1038/nature11247

El-Tanani MK, 2008, FRONT BIOSCI, V13, P4276, DOI 10.2741/3004

Ginestet C, 2011, J R STAT SOC A STAT, V174, P245, DOI 10.1111/j.1467-985X.2010.00676_9.x

He XX, 2016, ADV EXP MED BIOL, V936, P73, DOI 10.1007/978-3-319-42023-3_4

Hiyama T, 2007, INT J CANCER, V121, P1643, DOI 10.1002/ijc.23044

Hu Z, 2005, CLIN CANCER RES, V11, P4646, DOI 10.1158/1078-0432.CCR-04-2013

Jiang H, 2008, BIOINFORMATICS, V24, P2395, DOI 10.1093/bioinformatics/btn429

Johansson N, 2000, CELL MOL LIFE SCI, V57, P5, DOI 10.1007/s000180050495

Kolls JK, 2004, IMMUNITY, V21, P467, DOI 10.1016/j.immuni.2004.08.018

Leeman MF, 2002, CRIT REV BIOCHEM MOL, V37, P149, DOI 10.1080/10409230290771483

Li JCA, 2003, SOCIOL METHOD RES, V32, P117, DOI 10.1177/0049124103031004005

Li JG, 2014, GUT, V63, P1700, DOI 10.1136/gutjnl-2013-305806

Li J, 2016, WORLD J SURG ONCOL, V14, DOI 10.1186/s12957-016-1056-5

Li T, 2001, CARCINOGENESIS, V22, P929, DOI 10.1093/carcin/22.6.929

Lin DC, 2014, NAT GENET, V46, P467, DOI 10.1038/ng.2935

Liu H, 2012, DIS ESOPHAGUS, V25, P656, DOI 10.1111/j.1442-2050.2011.01284.x

Liu J, 2018, DISCOV MED, V25, P211

Malhotra GK, 2017, J SURG ONCOL, V115, P564, DOI 10.1002/jso.24592

Martin MD, 2007, CANCER METAST REV, V26, P717, DOI 10.1007/s10555-007-9089-4

Mendonsa AM, 2015, MOL CANCER, V14, DOI 10.1186/s12943-014-0282-0

Oleksiewicz U, 2017, J CANCER RES CLIN, V143, P1133, DOI 10.1007/s00432-017-2381-y

Ritchie ME, 2015, NUCLEIC ACIDS RES, V43, DOI 10.1093/nar/gkv007

Rizki A, 2008, CANCER RES, V68, P1378, DOI 10.1158/0008-5472.CAN-07-2225

Rothberg JM, 2011, NATURE, V475, P348, DOI 10.1038/nature10242

Rudland PS, 2002, CANCER RES, V62, P3417

Rustgi AK, 2014, NEW ENGL J MED, V371, P2499, DOI 10.1056/NEJMra1314530

Shah KV, 1997, NEW ENGL J MED, V337, P1386, DOI 10.1056/NEJM199711063371911

Shannon P, 2003, GENOME RES, V13, P2498, DOI 10.1101/gr.1239303

Shi XJ, 2016, ONCOTARGET, V7, P36407, DOI 10.18632/oncotarget.9239

SYRJANEN K, 1982, DIAGN HISTOPATHOL, V5, P291

SYRJANEN KJ, 1982, ARCH GESCHWULSTFORSC, V52, P283

Szklarczyk D, 2019, NUCLEIC ACIDS RES, V47, pD607, DOI 10.1093/nar/gky1131

Tan MY, 2015, SCI REP-UK, V5, DOI 10.1038/srep13996

Vargas AC, 2012, BREAST CANCER RES TR, V135, P153, DOI 10.1007/s10549-012-2123-4

Walaszek K, 2018, BRIT J CANCER, V119, P1259, DOI 10.1038/s41416-018-0228-1

Wang L, 2012, ACTA BIOCHIM POL, V59, P593

Wang Q, 2020, CANCER GENET-NY, V240, P33, DOI 10.1016/j.cancergen.2019.11.001

Xu ST, 2015, MOL MED REP, V11, P3701, DOI 10.3892/mmr.2015.3202

Yin YW, 2019, J CELL PHYSIOL, V234, P12757, DOI 10.1002/jcp.27896

Yu GC, 2015, BIOINFORMATICS, V31, P608, DOI 10.1093/bioinformatics/btu684

Yu GC, 2012, OMICS, V16, P284, DOI 10.1089/omi.2011.0118

Zeng B, 2018, ONCOTARGETS THER, V11, P1333, DOI 10.2147/OTT.S154215

Zhang JJ, 2014, MOL CANCER, V13, DOI 10.1186/1476-4598-13-130

NR 52

TC 0

Z9 0

U1 5

U2 9

PU MDPI

PI BASEL

PA ST ALBAN-ANLAGE 66, CH-4052 BASEL, SWITZERLAND

EI 2076-3417

J9 APPL SCI-BASEL

JI Appl. Sci.-Basel

PD APR

PY 2021

VL 11

IS 7

AR 3229

DI 10.3390/app11073229

PG 14

WC Chemistry, Multidisciplinary; Engineering, Multidisciplinary; Materials

Science, Multidisciplinary; Physics, Applied

WE Science Citation Index Expanded (SCI-EXPANDED)

SC Chemistry; Engineering; Materials Science; Physics

GA RK5TA

UT WOS:000638357300001

OA Green Published, gold

DA 2022-05-19

ER

PT J

AU Qian, X

Tan, C

Yang, B

Wang, F

Ge, Y

Guan, Z

Cai, J

AF Qian, X.

Tan, C.

Yang, B.

Wang, F.

Ge, Y.

Guan, Z.

Cai, J.

TI Astaxanthin increases radiosensitivity in esophageal squamous cell

carcinoma through inducing apoptosis and G2/M arrest

SO DISEASES OF THE ESOPHAGUS

LA English

DT Article

DE apoptosis; astaxanthin; cell cycle; esophageal squamous cell carcinoma;

radiosensitization

ID INHIBITS PROLIFERATION; DOWN-REGULATION; UP-REGULATION; IN-VITRO;

CANCER; MECHANISMS; BAX

AB Nowadays esophageal squamous cell carcinoma (ESCC) is primarily treated by a comprehensive approach combining surgical resection and neoadjuvant chemo-or radiotherapy. However, ESCC is resistant to radiation therapy, resulting in its invasion, infiltration, and metastasis. It usually has rapidly progressed and has a poor outcome clinically. The purpose of this study is to determine the potential radiosensitizing effect of astaxanthin (ATX) and explore the underlying mechanisms in ESCC cells in vitro. ESCC cell lines were exposure to irradiation, in the presence or absence of ATX treatment. Cell viability and radiosensitization were tested by CCK8 assay and clonogenic survival assay, respectively. Cell apoptosis and the changes of cell cycle distribution were observed by flow cytometry. The protein expression of Bcl2, Bax, CyclinB1, and Cdc2 was examined by western blot analysis. It was shown that ATX improved radiosensitivity of ESCC cells and induced apoptosis and G2/M arrest via inhibiting Bcl2, CyclinB1, Cdc2, and promoting Bax expression. In conclusion, ATX might function as a promising radiosensitizer in ESCC cells by leading to apoptosis and G2/M arrest.

C1 [Qian, X.; Tan, C.; Yang, B.; Wang, F.; Ge, Y.; Guan, Z.; Cai, J.] Nantong Univ, Affiliated Tumor Hosp, Dept Radiat Oncol, Nantong Tumor Hosp, Nantong 226321, Peoples R China.

RP Cai, J (corresponding author), Nantong Univ, Affiliated Tumor Hosp, Dept Radiat Oncol, Nantong Tumor Hosp, Nantong 226321, Peoples R China.

EM cj7227@sina.com

CR Alshatwi AA, 2016, EXP TOXICOL PATHOL, V68, P89, DOI 10.1016/j.etp.2015.10.002

Ambati RR, 2014, MAR DRUGS, V12, P128, DOI 10.3390/md12010128

Chen H, 2008, BMC CANCER, V8, DOI 10.1186/1471-2407-8-29

Cui YH, 2012, ASIAN PAC J CANCER P, V13, P1693, DOI 10.7314/APJCP.2012.13.4.1693

Ding Y Q, 2015, DIS ESOPHAGUS

Ikeda Y, 2008, J NEUROCHEM, V107, P1730, DOI 10.1111/j.1471-4159.2008.05743.x

Ishikawa C, 2008, INT J CANCER, V123, P2702, DOI 10.1002/ijc.23860

Jemal A, 2011, CA-CANCER J CLIN, V61, P69, DOI [10.3322/caac.20107, 10.3322/caac.20115]

Kavitha K, 2013, BBA-GEN SUBJECTS, V1830, P4433, DOI 10.1016/j.bbagen.2013.05.032

Kossmehl P, 2006, APOPTOSIS, V11, P347, DOI 10.1007/s10495-006-4350-9

Kowshile J, 2014, PLOS ONE, V9, DOI 10.1371/journal.pone.0109114

Li JJ, 2015, MAR DRUGS, V13, P6064, DOI 10.3390/md13106064

Liu J, 2014, ASIAN PAC J CANCER P, V15, P6649, DOI 10.7314/APJCP.2014.15.16.6649

Nagendraprabhu P, 2011, INVEST NEW DRUG, V29, P207, DOI 10.1007/s10637-009-9342-5

Naseri MH, 2015, CANCER CELL INT, V15, DOI 10.1186/s12935-015-0204-2

Qian X, 2016, ONCOTARGETS THER, V9, P2247, DOI 10.2147/OTT.S103179

Schweigert M, 2013, NAT REV GASTRO HEPAT, V10, P230, DOI 10.1038/nrgastro.2012.236

Siegel RL, 2015, CA-CANCER J CLIN, V65, P5, DOI [10.3322/caac.21254, 10.1136/bmj.g1502, 10.1001/jamaoto.2014.2530, 10.3322/caac.21208]

Song XD, 2012, TOXICOL MECH METHOD, V22, P679, DOI 10.3109/15376516.2012.717119

Tan C, 2016, SPRINGERPLUS, V5, DOI 10.1186/s40064-016-2119-3

Wang ZM, 2016, DIS ESOPHAGUS, V29, P54, DOI 10.1111/dote.12255

Yang X, 2013, CANCER BIOL THER, V14, P1068, DOI 10.4161/cbt.26426

Yuan JP, 2011, MOL NUTR FOOD RES, V55, P150, DOI 10.1002/mnfr.201000414

Zhang L, 2015, MAR DRUGS, V13, P4310, DOI 10.3390/md13074310

Zhang X, 2011, ARCH BIOCHEM BIOPHYS, V512, P96, DOI 10.1016/j.abb.2011.05.004

Zhang YQ, 2016, TUMOR BIOL, V37, P305, DOI 10.1007/s13277-015-3730-6

NR 26

TC 17

Z9 19

U1 1

U2 12

PU OXFORD UNIV PRESS INC

PI CARY

PA JOURNALS DEPT, 2001 EVANS RD, CARY, NC 27513 USA

SN 1120-8694

EI 1442-2050

J9 DIS ESOPHAGUS

JI Dis. Esophagus

PD JUN

PY 2017

VL 30

IS 6

DI 10.1093/dote/dox027

PG 7

WC Gastroenterology & Hepatology

WE Science Citation Index Expanded (SCI-EXPANDED)

SC Gastroenterology & Hepatology

GA EZ2IK

UT WOS:000404532800015

PM 28475750

DA 2022-05-19

ER

PT J

AU Luo, J

Xie, K

Gao, X

Yao, Y

Wang, GM

Shao, CY

Li, XK

Xu, Y

Ren, BH

Hu, LW

Shen, Y

AF Luo, Jing

Xie, Kai

Gao, Xiang

Yao, Yu

Wang, Gaoming

Shao, Chenye

Li, Xiaokun

Xu, Yang

Ren, Binhui

Hu, Liwen

Shen, Yi

TI Long Noncoding RNA Nuclear Paraspeckle Assembly Transcript 1 Promotes

Progression and Angiogenesis of Esophageal Squamous Cell Carcinoma

Through miR-590-3p/MDM2 Axis

SO FRONTIERS IN ONCOLOGY

LA English

DT Article

DE nuclear paraspeckle assembly transcript 1 (NEAT1); esophageal squamous

cell carcinoma (ESCC); angiogenesis; MDM2; miR-590-3p

AB Angiogenesis has been identified as one of the hallmarks of cancer and aggravates cancer development and progression. Accumulating evidence indicated that long noncoding RNAs (lncRNAs) are powerful factors in regulating various cancer behaviors. The aim of this study is to verify the function and potential mechanisms of lncRNA NEAT1 in progression and angiogenesis of esophageal squamous cell carcinoma (ESCC). We found that NEAT1 was overexpressed in ESCC tissues and correlated with clinical characteristics of patients. Silence of NEAT1 inhibited proliferation, migration, invasion and angiogenesis of ESCC cells. High throughput sequencing and western blotting revealed that NEAT1 regulated MDM2/p53 pathway. Rescue of MDM2 restored the effect of NEAT1 on progression and angiogenesis of ESCC cells. Nude mice xenograft models further validated the role of NEAT1 in vivo. Importantly, NEAT1 functioned as a competing endogenous RNA for miR-590-3p to regulate MDM2 expression and miR-590-3p acted as a tumor suppressor in ESCC progression and angiogenesis. These findings suggested that NEAT1/miR-590-3p/MDM2 axis might serve as potential therapeutic targets for ESCC patients.

C1 [Luo, Jing; Xie, Kai; Gao, Xiang; Ren, Binhui] Nanjing Med Univ, Affiliated Canc Hosp, Dept Thorac Surg, Nanjing, Peoples R China.

[Luo, Jing; Xie, Kai; Gao, Xiang; Ren, Binhui] Jiangsu Canc Hosp, Nanjing, Peoples R China.

[Luo, Jing; Xie, Kai; Gao, Xiang; Ren, Binhui] Jiangsu Inst Canc Res, Jiangsu Key Lab Mol & Translat Canc Res, Nanjing, Peoples R China.

[Luo, Jing; Xie, Kai; Wang, Gaoming; Shao, Chenye; Li, Xiaokun; Xu, Yang; Hu, Liwen; Shen, Yi] Nanjing Univ, Med Sch, Jinling Hosp, Dept Cardiothorac Surg, Nanjing, Peoples R China.

[Yao, Yu] Nanjing Second Hosp, Dept Resp Med, Nanjing, Peoples R China.

[Wang, Gaoming] Xuzhou Cent Hosp, Dept Thorac Surg, Xuzhou, Jiangsu, Peoples R China.

RP Ren, BH (corresponding author), Nanjing Med Univ, Affiliated Canc Hosp, Dept Thorac Surg, Nanjing, Peoples R China.; Ren, BH (corresponding author), Jiangsu Canc Hosp, Nanjing, Peoples R China.; Ren, BH (corresponding author), Jiangsu Inst Canc Res, Jiangsu Key Lab Mol & Translat Canc Res, Nanjing, Peoples R China.; Hu, LW; Shen, Y (corresponding author), Nanjing Univ, Med Sch, Jinling Hosp, Dept Cardiothorac Surg, Nanjing, Peoples R China.

EM renbinhui@jszlyy.com.cn; lw_hoo@163.com; dryishen@nju.edu.cn

FU Basic Research Program of Jiangsu Province [BK20160606]; National

Natural Science Foundation for Youth of China [81902354, 81702444];

Natural Science Foundation of Jiangsu Province [BK20181239]; National

Natural Science Foundation of China [81672869]; Jiangsu Provincial

Medical Outstanding Talent; Talents Program of Jiangsu Cancer Hospital

(Lin Xu) [YC201814]; "333" Talent Project [BRA2019325]

FX This research was supported by the Basic Research Program of Jiangsu

Province (BK20160606), National Natural Science Foundation for Youth of

China (nos. 81902354 and 81702444), Natural Science Foundation of

Jiangsu Province (BK20181239), National Natural Science Foundation of

China (81672869), Jiangsu Provincial Medical Outstanding Talent (Lin

Xu), Jiangsu Provincial Medical Youth Talent (BR, QNRC2016657), the

Talents Program of Jiangsu Cancer Hospital YC201814 and the "333" Talent

Project: BRA2019325.

CR Barry G, 2017, SCI REP-UK, V7, DOI 10.1038/srep40127

Bray F, 2018, CA-CANCER J CLIN, V68, P394, DOI 10.3322/caac.21492

Carmeliet P, 2000, NATURE, V407, P249, DOI 10.1038/35025220

Chen XJ, 2015, AM J CANCER RES, V5, P2808

Chen XX, 2019, CELL DEATH DIS, V10, DOI 10.1038/s41419-018-1188-3

Cheng HP, 2020, J ENVIRON PATHOL TOX, V39, P101, DOI 10.1615/JEnvironPatholToxicolOncol.2020032508

Dong PX, 2019, J EXP CLIN CANC RES, V38, DOI 10.1186/s13046-019-1306-9

Ebrahim M, 2015, HISTOL HISTOPATHOL, V30, P1271, DOI 10.14670/HH-11-636

Enzinger PC, 2003, NEW ENGL J MED, V349, P2241, DOI 10.1056/NEJMra035010

Gao M, 2020, ONCOTARGETS THER, V13, P3977, DOI 10.2147/OTT.S245557

Ge X, 2017, TUMOR BIOL, V39, DOI 10.1177/1010428317695947

Hanahan D, 2011, CELL, V144, P646, DOI 10.1016/j.cell.2011.02.013

Hayashi Y, 2016, CARCINOGENESIS, V37, P972, DOI 10.1093/carcin/bgw085

Khorkova O, 2015, ADV DRUG DELIVER REV, V87, P15, DOI 10.1016/j.addr.2015.05.012

Li TM, 2017, INT J MOL SCI, V18, DOI 10.3390/ijms18050920

Li X, 2018, CANCER RES, V78, P4138, DOI 10.1158/0008-5472.CAN-18-0688

Li Y, 2017, DIS MARKERS, V2017, DOI 10.1155/2017/5314649

Lin XJ, 2019, MOL CARCINOGEN, V58, P2286, DOI 10.1002/mc.23117

Luo J, 2019, CANCER CELL INT, V19, DOI 10.1186/s12935-019-0800-7

Luo J, 2019, CANCER LETT, V453, P45, DOI 10.1016/j.canlet.2019.03.045

Marme D, 2018, ONCOL RES TREAT, V41, P164, DOI 10.1159/000488340

Morchikh M, 2017, MOL CELL, V67, P387, DOI 10.1016/j.molcel.2017.06.020

Pang HY, 2015, BIOCHEM BIOPH RES CO, V468, P739, DOI 10.1016/j.bbrc.2015.11.025

Patterson DM, 2011, ANGIOGENESIS, V14, P255, DOI 10.1007/s10456-011-9210-8

Qi XL, 2015, J MED GENET, V52, P710, DOI 10.1136/jmedgenet-2015-103334

Rajabi M, 2017, BIOMEDICINES, V5, DOI 10.3390/biomedicines5020034

Ramjiawan RR, 2017, ANGIOGENESIS, V20, P185, DOI 10.1007/s10456-017-9552-y

Richard JLC, 2018, SLAS TECHNOL, V23, P493, DOI 10.1177/2472630318780639

Rohini M, 2018, BIOCHIMIE, V154, P10, DOI 10.1016/j.biochi.2018.07.023

Siegel RL, 2020, CA-CANCER J CLIN, V70, P7, DOI 10.3322/caac.21590

Song JP, 2019, J CELL BIOCHEM, V120, P6937, DOI 10.1002/jcb.27743

Song RP, 2015, INT J SURG, V20, P80, DOI 10.1016/j.ijsu.2015.04.050

Tay Y, 2014, NATURE, V505, P344, DOI 10.1038/nature12986

Venkatesan T, 2018, CELLS-BASEL, V7, DOI 10.3390/cells7050041

Wade M, 2013, NAT REV CANCER, V13, P83, DOI 10.1038/nrc3430

Wang WT, 2018, BIOMED PHARMACOTHER, V107, P1763, DOI 10.1016/j.biopha.2018.06.124

Wang ZQ, 2020, CELL MOL LIFE SCI, V77, P3769, DOI 10.1007/s00018-020-03503-0

Wang ZQ, 2017, CELL MOL LIFE SCI, V74, P1117, DOI 10.1007/s00018-016-2398-4

Xiong J, 2014, ANGIOGENESIS, V17, P37, DOI 10.1007/s10456-013-9376-3

Xue F, 2020, AGING-US, V12, P14174, DOI 10.18632/aging.103427

Yan L, 2020, ONCOTARGETS THER, V13, P2449, DOI 10.2147/OTT.S240769

Yang GD, 2014, BBA-GENE REGUL MECH, V1839, P1097, DOI 10.1016/j.bbagrm.2014.08.012

Yang XS, 2018, ONCOGENE, V37, P6399, DOI 10.1038/s41388-018-0416-8

Yu X, 2017, CELL PROLIFERAT, V50, DOI 10.1111/cpr.12329

Zhang YW, 2013, WORLD J GASTROENTERO, V19, P5598, DOI 10.3748/wjg.v19.i34.5598

Zhao XX, 2020, MOL MED REP, V21, P2560, DOI 10.3892/mmr.2020.11064

Zhou ZW, 2019, BRAIN RES, V1707, P90, DOI 10.1016/j.brainres.2018.10.031

NR 47

TC 3

Z9 3

U1 2

U2 3

PU FRONTIERS MEDIA SA

PI LAUSANNE

PA AVENUE DU TRIBUNAL FEDERAL 34, LAUSANNE, CH-1015, SWITZERLAND

SN 2234-943X

J9 FRONT ONCOL

JI Front. Oncol.

PD FEB 19

PY 2021

VL 10

AR 618930

DI 10.3389/fonc.2020.618930

PG 12

WC Oncology

WE Science Citation Index Expanded (SCI-EXPANDED)

SC Oncology

GA QS2XU

UT WOS:000625768200001

PM 33680941

OA Green Published, gold

DA 2022-05-19

ER

PT J

AU Takao, K

Konishi, H

Fujiwara, H

Shiozaki, A

Shoda, K

Kosuga, T

Kubota, T

Arita, T

Morimura, R

Murayama, Y

Kuriu, Y

Ikoma, H

Nakanishi, M

Okamoto, K

Otsuji, E

AF Takao, Koji

Konishi, Hirotaka

Fujiwara, Hitoshi

Shiozaki, Atsushi

Shoda, Katsutoshi

Kosuga, Toshiyuki

Kubota, Takeshi

Arita, Tomohiro

Morimura, Ryo

Murayama, Yasutoshi

Kuriu, Yoshiaki

Ikoma, Hisashi

Nakanishi, Masayoshi

Okamoto, Kazuma

Otsuji, Eigo

TI Clinical Significance of Prognostic Nutritional Index in the Treatment

of Esophageal Squamous Cell Carcinoma

SO IN VIVO

LA English

DT Article

DE Prognostic nutritional index; esophageal squamous cell carcinoma;

neoadjuvant chemotherapy; esophagectomy; prognosis

ID NEOADJUVANT CHEMOTHERAPY; PREOPERATIVE CHEMOTHERAPY; POSTOPERATIVE

MORTALITY; ENTERAL NUTRITION; CANCER-PATIENTS; CHEMORADIOTHERAPY;

COMPLICATIONS; MORBIDITY; OUTCOMES; 5-FLUOROURACIL

AB Background/Aim: The prognostic nutritional index (PNI) is reported to affect postoperative complications and survival of patients with esophageal squamous cell carcinoma (ESCC). The aim of this study is to investigate the clinical significance of PNI in treatment of ESCC. Patients and Methods: Two hundred and sixty-three patients who underwent radical esophagectomy were retrospectively analyzed. PNI was calculated in the pretreatment (pre-Tx), post-neoadjuvant chemotherapy (post-NAC), and postoperative periods. Results: Pre-Tx PNI positively correlated with prognosis irrespective of undergoing NAC (p<0.05). In the patients with NAC, pre-Tx PNI was one of the independent prognostic factors (p=0.04). In patients with low pre-Tx PNI, the prognosis was improved by increase of PNI after NAC (p=0.08), and two cycles of NAC significantly correlated with high post-NAC PNI (p=0.04). Conclusion: Pre-Tx PNI is an independent prognostic factor irrespective of NAC. Patients in whom the post-NAC PNI can be improved have a high probability of obtaining a good prognosis.

C1 [Takao, Koji; Konishi, Hirotaka; Fujiwara, Hitoshi; Shiozaki, Atsushi; Shoda, Katsutoshi; Kosuga, Toshiyuki; Kubota, Takeshi; Arita, Tomohiro; Morimura, Ryo; Murayama, Yasutoshi; Kuriu, Yoshiaki; Ikoma, Hisashi; Nakanishi, Masayoshi; Okamoto, Kazuma; Otsuji, Eigo] Kyoto Prefectural Univ Med, Dept Surg, Div Digest Surg, Kyoto, Japan.

RP Konishi, H (corresponding author), Kyoto Prefectural Univ Med, Dept Surg, Div Digest Surg, Kamigyo Ku, 465 Kajii Cho, Kyoto 6028566, Japan.

EM h-koni7@koto.kpu-m.ac.jp

RI Arita, Tomohiro/AAQ-1339-2021

CR Ando N, 2012, ANN SURG ONCOL, V19, P68, DOI 10.1245/s10434-011-2049-9

Bancewicz J, 2002, LANCET, V359, P1727

Bray F, 2018, CA-CANCER J CLIN, V68, P394, DOI 10.3322/caac.21492

Caccialanza R, 2016, J CANCER, V7, P131, DOI 10.7150/jca.13818

Clavien PA, 2009, ANN SURG, V250, P187, DOI 10.1097/SLA.0b013e3181b13ca2

Cools-Lartigue J, 2015, ANN SURG ONCOL, V22, P1858, DOI 10.1245/s10434-014-4270-9

Di Fiore A, 2014, DIGEST LIVER DIS, V46, P270, DOI 10.1016/j.dld.2013.10.016

Feng JF, 2014, THER CLIN RISK MANAG, V10, P1, DOI 10.2147/TCRM.S56159

Fietkau R, 2013, CANCER-AM CANCER SOC, V119, P3343, DOI 10.1002/cncr.28197

Hara H, 2013, CANCER SCI, V104, P1455, DOI 10.1111/cas.12274

Ho JWC, 2015, CLIN NUTR, V34, P679, DOI 10.1016/j.clnu.2014.07.012

Hu WH, 2015, NUTR J, V14, DOI 10.1186/s12937-015-0081-5

Huddy JR, 2018, DIS ESOPHAGUS, V31, DOI 10.1093/dote/dox110

Japan Esophageal Society, 2017, JAP CLASS ES CANC, V14, P37, DOI [10.1007/s10388-016-0556-2, DOI 10.1007/S10388-016-0556-2]

Kanda M, 2011, BRIT J SURG, V98, P268, DOI 10.1002/bjs.7305

Migita K, 2013, ANN SURG ONCOL, V20, P2647, DOI 10.1245/s10434-013-2926-5

Mohri Y, 2013, WORLD J SURG, V37, P2688, DOI 10.1007/s00268-013-2156-9

Motoori M, 2012, ONCOLOGY-BASEL, V83, P91, DOI 10.1159/000339694

National Institute of Health Cancer Therapy Evaluation Program, 2017, COMM TERM CRIT ADV E

Nozoe T, 2002, EUR J SURG ONCOL, V28, P396, DOI 10.1053/ejso.2002.1257

Onodera T, 1984, Nihon Geka Gakkai Zasshi, V85, P1001

Pinato DJ, 2012, BRIT J CANCER, V106, P1439, DOI 10.1038/bjc.2012.92

Reisinger KW, 2015, ANN SURG ONCOL, V22, P4445, DOI 10.1245/s10434-015-4558-4

Sakurai K, 2016, ANN SURG ONCOL, V23, P525, DOI 10.1245/s10434-015-4814-7

Schwegler I, 2010, BRIT J SURG, V97, P92, DOI 10.1002/bjs.6805

Shapiro J, 2015, LANCET ONCOL, V16, P1090, DOI 10.1016/S1470-2045(15)00040-6

Sjoquist KM, 2011, LANCET ONCOL, V12, P681, DOI 10.1016/S1470-2045(11)70142-5

Watanabe M, 2014, ANN SURG ONCOL, V21, P2838, DOI 10.1245/s10434-014-3684-8

Yoshida N, 2014, DIS ESOPHAGUS, V27, P374, DOI 10.1111/dote.12117

Yoshida N, 2016, WORLD J SURG, V40, P1910, DOI 10.1007/s00268-016-3549-3

NR 30

TC 3

Z9 3

U1 0

U2 1

PU INT INST ANTICANCER RESEARCH

PI ATHENS

PA EDITORIAL OFFICE 1ST KM KAPANDRITIOU-KALAMOU RD KAPANDRITI, PO BOX 22,

ATHENS 19014, GREECE

SN 0258-851X

EI 1791-7549

J9 IN VIVO

JI In Vivo

PD NOV-DEC

PY 2020

VL 34

IS 6

BP 3451

EP 3457

DI 10.21873/invivo.12184

PG 7

WC Medicine, Research & Experimental

WE Science Citation Index Expanded (SCI-EXPANDED)

SC Research & Experimental Medicine

GA OP2QN

UT WOS:000587929100044

PM 33144453

OA Green Published, gold

DA 2022-05-19

ER

PT J

AU Yao, Q

Yang, J

Liu, T

Zhang, JJ

Zheng, YB

AF Yao, Qi

Yang, Jun

Liu, Ting

Zhang, Jianjiang

Zheng, Yibo

TI Long noncoding RNA MALAT1 promotes the stemness of esophageal squamous

cell carcinoma by enhancing YAP transcriptional activity

SO FEBS OPEN BIO

LA English

DT Article

DE esophageal squamous cell carcinoma; MALAT1; migration; stemness; YAP

ID UP-REGULATION; PROLIFERATION; MIGRATION; INVASION; PATHWAY; PROGRESSION;

SURVIVAL; THOR

AB The tumor promoting roles of long noncoding RNA (lncRNA) MALAT1 have been revealed in various cancers; however, its roles in esophageal squamous cell carcinoma (ESCC) have not previously been disclosed. In this study, we found that MALAT1 expression was remarkably increased in ESCC cells compared to normal human esophageal epithelial cells. In addition, knockdown of MALAT1 attenuated the stemness of ESCC cells, as evidenced by a decrease in spheroid formation capacity, stemness marker expression and aldehyde dehydrogenase 1 activity. Moreover, MALAT1 knockdown decreased the migration ability of ESCC cells. Notably, knockdown of MALAT1 enhanced the radiosensitivity and chemosensitivity of ESCC cells. We also established that MALAT1 binds directly to Yes-associated protein (YAP), thereby enhancing YAP protein expression and increasing YAP transcriptional activity. Overexpression of YAP partially rescued the effect of MALAT1 knockdown on stemness and radiosensitivity of ESCC cells. Overall, this study has identified that a novel MALAT1-YAP axis promotes the stemness of ESCC cells, and thus could be a potential target for treatment of ESCC.

C1 [Yao, Qi; Yang, Jun; Liu, Ting; Zhang, Jianjiang; Zheng, Yibo] Ningbo First Hosp, Dept Geriatr Med, 59 Liuting St, Ningbo 315010, Zhejiang, Peoples R China.

RP Yao, Q (corresponding author), Ningbo First Hosp, Dept Geriatr Med, 59 Liuting St, Ningbo 315010, Zhejiang, Peoples R China.

EM qiyao_qy@163.com

CR Abulwerdi FA, 2019, ACS CHEM BIOL, V14, P223, DOI 10.1021/acschembio.8b00807

Bray F, 2018, CA-CANCER J CLIN, V68, P394, DOI 10.3322/caac.21492

Cao XG, 2015, CANCER BIOMARK, V15, P717, DOI 10.3233/CBM-150513

Cazet AS, 2018, NAT COMMUN, V9, DOI 10.1038/s41467-018-05220-6

Chen XN, 2017, CELL DISCOV, V3, DOI 10.1038/celldisc.2017.2

Duan GC, 2019, INT J ONCOL, V54, P17, DOI 10.3892/ijo.2018.4600

Fu Y, 2019, GENE, V704, P91, DOI 10.1016/j.gene.2019.04.019

Gong NQ, 2019, ACS APPL MATER INTER, V11, P37, DOI 10.1021/acsami.8b18288

Gordon MA, 2019, MOL CARCINOGEN, V58, P196, DOI 10.1002/mc.22919

Graham RP, 2017, MODERN PATHOL, V30, P1443, DOI 10.1038/modpathol.2017.68

Guan ZB, 2018, CANCER BIOMARK, V21, P557, DOI 10.3233/CBM-170431

Han Y, 2016, CELL DEATH DIS, V7, DOI 10.1038/cddis.2015.407

He WM, 2019, ONCOGENE, V38, P4637, DOI 10.1038/s41388-019-0747-0

Hosono Y, 2017, CELL, V171, P1559, DOI 10.1016/j.cell.2017.11.040

Hu JG, 2018, CELL PHYSIOL BIOCHEM, V47, P654, DOI 10.1159/000490020

Hu LW, 2015, J EXP CLIN CANC RES, V34, DOI 10.1186/s13046-015-0123-z

Kim J, 2018, NAT GENET, V50, P1705, DOI 10.1038/s41588-018-0252-3

Kim T, 2015, NAT COMMUN, V6, DOI 10.1038/ncomms10186

Kwok ZH, 2018, INT J CANCER, V143, P668, DOI 10.1002/ijc.31386

Li CL, 2017, NAT CELL BIOL, V19, P106, DOI 10.1038/ncb3464

Li ZK, 2018, CELL PROLIFERAT, V51, DOI 10.1111/cpr.12504

Liu K, 2018, ONCOTARGETS THER, V11, P8729, DOI 10.2147/OTT.S182993

Ni W, 2017, J HEMATOL ONCOL, V10, DOI 10.1186/s13045-017-0449-4

Ohgushi M, 2015, CELL STEM CELL, V17, P448, DOI 10.1016/j.stem.2015.07.009

Ouyang SR, 2019, CANCER CELL INT, V19, DOI 10.1186/s12935-019-0784-3

Pollyea DA, 2018, NAT MED

Qin JH, 2018, BIOMED PHARMACOTHER, V105, P758, DOI 10.1016/j.biopha.2018.06.028

Song H, 2018, BIOMED PHARMACOTHER, V108, P338, DOI 10.1016/j.biopha.2018.09.057

Spiniello M, 2018, J PROTEOME RES, V17, P3022, DOI 10.1021/acs.jproteome.8b00189

Sun ZQ, 2019, ONCOGENE, V38, P2627, DOI 10.1038/s41388-018-0628-y

Tanaka K, 2017, CANCER LETT, V385, P215, DOI 10.1016/j.canlet.2016.10.020

Tang DX, 2019, J CELL PHYSIOL, V234, P20816, DOI 10.1002/jcp.28687

Tian J, 2018, BRIT J CANCER, V119, P1495, DOI 10.1038/s41416-018-0287-3

Torre LA, 2015, CA-CANCER J CLIN, V65, P87, DOI 10.3322/caac.21262

Wang LH, 2019, ONCOGENE, V38, P2042, DOI 10.1038/s41388-018-0476-9

Xiao YW, 2019, FEBS OPEN BIO, V9, P1212, DOI 10.1002/2211-5463.12649

Yao WJ, 2016, TUMOR BIOL, V37, P4305, DOI 10.1007/s13277-015-4223-3

Zhang M, 2018, J HEMATOL ONCOL, V11, DOI 10.1186/s13045-018-0656-7

NR 38

TC 19

Z9 19

U1 0

U2 5

PU WILEY

PI HOBOKEN

PA 111 RIVER ST, HOBOKEN 07030-5774, NJ USA

SN 2211-5463

J9 FEBS OPEN BIO

JI FEBS Open Bio

PD AUG

PY 2019

VL 9

IS 8

BP 1392

EP 1402

DI 10.1002/2211-5463.12676

PG 11

WC Biochemistry & Molecular Biology

WE Science Citation Index Expanded (SCI-EXPANDED)

SC Biochemistry & Molecular Biology

GA IM4PL

UT WOS:000477976800005

PM 31116509

OA Green Published, gold

DA 2022-05-19

ER

PT J

AU Zhu, R

Liu, YS

Zhou, HH

Li, L

Li, Y

Ding, F

Cao, XF

Liu, ZH

AF Zhu, Rui

Liu, Yongshuo

Zhou, Honghong

Li, Lei

Li, Yi

Ding, Fang

Cao, Xiufeng

Liu, Zhihua

TI Deubiquitinating enzyme PSMD14 promotes tumor metastasis through

stabilizing SNAIL in human esophageal squamous cell carcinoma

SO CANCER LETTERS

LA English

DT Article

DE Esophageal squamous cell carcinoma; PSMD14; SNAIL; Deubiquitination;

Tumor metastasis

ID E-CADHERIN REPRESSION; EPITHELIAL-MESENCHYMAL TRANSITION; BREAST-CANCER;

REGULATES P53; MYC STABILITY; C-MYC; UBIQUITINATION; PROTEASOME; POH1;

DEGRADATION

AB The epithelial-mesenchymal transition (EMT) transcription factor SNAIL is associated with distant metastasis and poor prognosis of esophageal squamous cell carcinoma (ESCC) patients. The proteolysis of SNAIL is mediated by the ubiquitin-proteasome system. Several E3 ligases have been characterized to promote SNAIL ubiquitination and degradation. However, the reverse process - deubiquitination of SNAIL remains largely unknown. In this study, we performed a mass spectrometry to examine the interaction between SNAIL and deubiquitinating enzyme(s). Subsequently, the deubiquitinating enzyme PSMD14 was identified to target SNAIL for deubiquitination and stabilization. Furthermore, knockdown of PSMD14 significantly blocks SNAIL-induced EMT and then suppresses tumor cell migration and invasion in vitro and tumor metastasis in vivo. In addition, the high expression level of PSMD14 predicts poor prognosis for esophageal cancer patients. These findings suggest PSMD14 as a bona fide deubiquitinating enzyme to regulate SNAIL at the post-translational level and provide a promising therapeutic strategy against tumor metastasis of esophageal cancer. (C) 2018 Elsevier B.V. All rights reserved.

C1 [Zhu, Rui; Liu, Yongshuo; Zhou, Honghong; Li, Lei; Li, Yi; Ding, Fang; Liu, Zhihua] Chinese Acad Med Sci, Natl Canc Ctr, Canc Hosp, State Key Lab Mol Oncol, Beijing 100021, Peoples R China.

[Zhu, Rui; Liu, Yongshuo; Zhou, Honghong; Li, Lei; Li, Yi; Ding, Fang; Liu, Zhihua] Peking Union Med Coll, Beijing 100021, Peoples R China.

[Liu, Yongshuo] Binzhou Med Univ Hosp, Dept Clin Lab, Binzhou 256603, Shandong, Peoples R China.

[Cao, Xiufeng] Nanjing Med Univ, Nanjing Hosp 1, Dept Surg Oncol, Nanjing 210029, Jiangsu, Peoples R China.

RP Liu, ZH (corresponding author), Chinese Acad Med Sci, Natl Canc Ctr, Canc Hosp, State Key Lab Mol Oncol, Beijing 100021, Peoples R China.; Liu, ZH (corresponding author), Peking Union Med Coll, Beijing 100021, Peoples R China.

EM liuzh@cicams.ac.cn

RI Liu, Yongshuo/AGZ-8274-2022

OI Liu, Yongshuo/0000-0001-8190-0139

FU National Key R&D Program of China [2016YFC1302100]; National Science

Foundation of China [81420108025]; CAMS Initiative for Innovative

Medicine [2016-I2M-1-001]; Shandong Provincial Natural Science

Foundation, China [ZR2017BH058]

FX This work was supported by National Key R&D Program of China

(2016YFC1302100), National Science Foundation of China (81420108025),

CAMS Initiative for Innovative Medicine (2016-I2M-1-001) and Shandong

Provincial Natural Science Foundation, China (ZR2017BH058). We thank Dr.

Shimada Y (Kyoto University, Kyoto, Japan) for kindly providing the ESCC

cell lines.

CR Amodio N, 2010, AM J PATHOL, V177, P2622, DOI 10.2353/ajpath.2010.091075

Anaya J, 2016, PEERJ COMPUT SCI, DOI 10.7717/peerj-cs.67

Nieto MA, 2016, CELL, V166, P21, DOI 10.1016/j.cell.2016.06.028

Baulida J, 2015, BBA-REV CANCER, V1856, P55, DOI 10.1016/j.bbcan.2015.05.005

Buckley SM, 2012, CELL STEM CELL, V11, P783, DOI 10.1016/j.stem.2012.09.011

Butler LR, 2012, EMBO J, V31, P3918, DOI 10.1038/emboj.2012.232

Byrne A, 2010, EXP CELL RES, V316, P258, DOI 10.1016/j.yexcr.2009.08.018

Chen WQ, 2016, CA-CANCER J CLIN, V66, P115, DOI 10.3322/caac.21338

Das CM, 2013, ONCOGENE, V32, P1691, DOI 10.1038/onc.2012.182

Dong CF, 2012, J CLIN INVEST, V122, P1469, DOI 10.1172/JCI57349

Ferrari-Amorotti G, 2013, CANCER RES, V73, P235, DOI 10.1158/0008-5472.CAN-12-1739

Gallery M, 2007, MOL CANCER THER, V6, P262, DOI 10.1158/1535-7163.MCT-06-0542

Gyorffy B, 2010, BREAST CANCER RES TR, V123, P725, DOI 10.1007/s10549-009-0674-9

Hao R, 2013, MOL CELL, V51, P819, DOI 10.1016/j.molcel.2013.08.016

Herranz N, 2008, MOL CELL BIOL, V28, P4772, DOI 10.1128/MCB.00323-08

Herrera A, 2016, CLIN TRANSL ONCOL, V18, P872, DOI 10.1007/s12094-015-1474-9

Jacquemont C, 2007, CANCER RES, V67, P7395, DOI 10.1158/0008-5472.CAN-07-1015

Jin Y, 2015, CANCER LETT, V362, P70, DOI 10.1016/j.canlet.2015.03.037

Kaufhold S, 2014, J EXP CLIN CANC RES, V33, DOI 10.1186/s13046-014-0062-0

Kim D, 2017, J CELL PHYSIOL, V232, P3664, DOI 10.1002/jcp.25841

King B, 2013, CELL, V153, P1552, DOI 10.1016/j.cell.2013.05.041

Li J, 2017, NAT CHEM BIOL, V13, P486, DOI [10.1038/NCHEMBIO.2326, 10.1038/nchembio.2326]

Li MY, 2002, NATURE, V416, P648, DOI 10.1038/nature737

Li Y, 2016, BIOCHEM BIOPH RES CO, V473, P278, DOI 10.1016/j.bbrc.2016.03.093

Liu H, 2009, PLOS ONE, V4, DOI 10.1371/journal.pone.0005544

Liu LP, 2017, BIOCHEM BIOPH RES CO, V493, P20, DOI 10.1016/j.bbrc.2017.09.091

Liu TZ, 2017, NAT COMMUN, V8, DOI 10.1038/ncomms13923

Liu Y.S, 2017, ONCOGENE

Ma J, 2015, BIOCHEM BIOPH RES CO, V464, P147, DOI 10.1016/j.bbrc.2015.06.104

Maddika S, 2011, NAT CELL BIOL, V13, P728, DOI 10.1038/ncb2240

Nabhan JF, 2006, J BIOL CHEM, V281, P16099, DOI 10.1074/jbc.M512086200

Old JB, 2010, MOL CANCER RES, V8, P353, DOI 10.1158/1541-7786.MCR-09-0232

Pan J, 2015, ONCOGENE, V34, P3957, DOI 10.1038/onc.2014.327

Paul I, 2013, ONCOGENE, V32, P1284, DOI 10.1038/onc.2012.144

Peinado H, 2004, MOL CELL BIOL, V24, P306, DOI 10.1128/MCB.24.1.306-319.2004

Peinado H, 2007, NAT REV CANCER, V7, P415, DOI 10.1038/nrc2131

Pennathur A, 2013, LANCET, V381, P400, DOI 10.1016/S0140-6736(12)60643-6

Perez C, 2017, J MED CHEM, V60, P1343, DOI 10.1021/acs.jmedchem.6b01379

Rustgi AK, 2014, NEW ENGL J MED, V371, P2499, DOI 10.1056/NEJMra1314530

Schwarz T, 2010, J CELL BIOCHEM, V109, P967, DOI 10.1002/jcb.22475

SHIMADA Y, 1992, CANCER, V69, P277, DOI 10.1002/1097-0142(19920115)69:2<277::AID-CNCR2820690202>3.0.CO;2-C

Shin G, 2011, CANCER INFORM, V10, P149, DOI 10.4137/CIN.S7226

Steeg PS, 2016, NAT REV CANCER, V16, P201, DOI 10.1038/nrc.2016.25

Verma R, 2002, SCIENCE, V298, P611, DOI 10.1126/science.1075898

Vinas-Castells R, 2014, NUCLEIC ACIDS RES, V42, P1079, DOI 10.1093/nar/gkt935

Vinas-Castells R, 2010, J BIOL CHEM, V285, P3794, DOI 10.1074/jbc.M109.065995

Wang BS, 2015, NAT COMMUN, V6, DOI 10.1038/ncomms9704

Wang YF, 2013, CURR CANCER DRUG TAR, V13, P963, DOI 10.2174/15680096113136660102

Wu C. E., 2017, BR J CANC

Wu YD, 2017, NAT COMMUN, V8, DOI 10.1038/ncomms14228

Yao TT, 2002, NATURE, V419, P403, DOI 10.1038/nature01071

Yuan L, 2015, NAT CELL BIOL, V17, P1169, DOI 10.1038/ncb3218

Zhang JD, 2017, CELL DEATH DIS, V8, DOI 10.1038/cddis.2017.149

Zhang JS, 2013, NAT CELL BIOL, V15, P1486, DOI 10.1038/ncb2874

Zheng HQ, 2014, CANCER CELL, V26, P358, DOI 10.1016/j.ccr.2014.07.022

Zhou BHP, 2004, NAT CELL BIOL, V6, P931, DOI 10.1038/ncb1173

Zhou ZC, 2014, GASTROENTEROLOGY, V147, P1043, DOI 10.1053/j.gastro.2014.07.021

NR 57

TC 39

Z9 41

U1 0

U2 15

PU ELSEVIER IRELAND LTD

PI CLARE

PA ELSEVIER HOUSE, BROOKVALE PLAZA, EAST PARK SHANNON, CO, CLARE, 00000,

IRELAND

SN 0304-3835

EI 1872-7980

J9 CANCER LETT

JI Cancer Lett.

PD APR 1

PY 2018

VL 418

BP 125

EP 134

DI 10.1016/j.canlet.2018.01.025

PG 10

WC Oncology

WE Science Citation Index Expanded (SCI-EXPANDED)

SC Oncology

GA FX5SL

UT WOS:000426139800013

PM 29331416

DA 2022-05-19

ER

PT J

AU Hu, DX

Zhang, MY

Wang, S

Wang, Z

AF Hu, Dongxin

Zhang, Mingyan

Wang, Shuai

Wang, Zhou

TI High expression of cyclooxygenase 2 is an indicator of prognosis for

patients with esophageal squamous cell carcinoma after Ivor Lewis

esophagectomy

SO THORACIC CANCER

LA English

DT Article

DE COX2; esophageal squamous cell carcinoma; Ku80; prognosis; recurrence

ID CANCER; RECURRENCE; OVEREXPRESSION; KU80; RISK

AB Background: The poor prognosis of esophageal squamous cell carcinoma (ESCC) is attributed to a high recurrence rate after surgery. Cyclooxygenase 2 (COX2) is an important regulator of cell growth, differentiation, apoptosis, and transformation. COX2 overexpression is significantly associated with the tumorigenesis and progression of diverse cancers; however, its expression and significance in ESCC remains unclear.

Methods: We enrolled 118 patients with ESCC who had undergone Ivor-Lewis esophagectomy. The expression profile of COX2 was examined by immunohistochemistry.

Results: A high expression of COX2 correlated with a higher T staging (P = 0.014), lower differentiation degree (P = 0.002), lymph node metastasis (P = 0.009), recurrence status (P = 0.004), and tumor node metastasis (TNM) stage (P = 0.001). Cox regression analysis showed that TNM stage (P = 0.001), differentiation degree (P = 0.001), and high COX2 expression (P = 0.004) were independent risk factors of prognosis.

Conclusion: Our data indicated that COX2 expression level is associated with key clinicopathological features and could be an effective biomarker to predict ESCC prognosis.

C1 [Hu, Dongxin; Wang, Shuai; Wang, Zhou] Shandong Univ, Prov Hosp Affiliated, Dept Thorac Surg, 324 Jing 5 Rd, Jinan 250021, Shandong, Peoples R China.

[Zhang, Mingyan] Shandong Univ, Prov Hosp Affiliated, Dept Gastroenterol, 324 Jing 5 Rd, Jinan 250021, Shandong, Peoples R China.

RP Wang, Z (corresponding author), Shandong Univ, Prov Hosp Affiliated, Dept Thorac Surg, 324 Jing 5 Rd, Jinan 250021, Shandong, Peoples R China.

EM 43572850@qq.com

OI Wang, Shuai/0000-0001-8503-9024

CR Ferlay J, 2010, INT J CANCER, V127, P2893, DOI 10.1002/ijc.25516

Gaffney DK, 2001, INT J RADIAT ONCOL, V49, P1213, DOI 10.1016/S0360-3016(00)01583-2

Hao Q, 2014, CELL SIGNAL, V26, P564, DOI 10.1016/j.cellsig.2013.11.030

Hulscher JBF, 2000, J AM COLL SURGEONS, V191, P143, DOI 10.1016/S1072-7515(00)00349-5

Kamangar F, 2006, J CLIN ONCOL, V24, P2137, DOI 10.1200/JCO.2005.05.2308

Lagergren J, 1999, NEW ENGL J MED, V340, P825, DOI 10.1056/NEJM199903183401101

Mariette C, 2007, LANCET ONCOL, V8, P545, DOI 10.1016/S1470-2045(07)70172-9

Perez AA, 2015, J HISTOCHEM CYTOCHEM, V63, P408, DOI 10.1369/0022155415576540

Ranelletti FO, 2001, INT J CANCER, V95, P343, DOI 10.1002/1097-0215(20011120)95:6<343::AID-IJC1060>3.0.CO;2-D

Saviozzi S, 2009, CANCER RES, V69, P3390, DOI 10.1158/0008-5472.CAN-08-2981

Song YM, 2012, ANN SURG ONCOL, V19, P3606, DOI 10.1245/s10434-012-2419-y

Thoen H, 2015, RADIOTHER ONCOL, V115, P16, DOI 10.1016/j.radonc.2015.03.012

Wang S, 2015, TUMOR BIOL, V36, P6181, DOI 10.1007/s13277-015-3302-9

Wang S, 2015, WORLD J SURG, V39, P1773, DOI 10.1007/s00268-015-3023-7

Wolff H, 1998, CANCER RES, V58, P4997

Yamauchi T, 2002, DIS COLON RECTUM, V45, P98, DOI 10.1007/s10350-004-6120-5

Zhou LP, 2012, ASIAN PAC J CANCER P, V13, P3637, DOI 10.7314/APJCP.2012.13.8.3637

NR 17

TC 14

Z9 15

U1 0

U2 0

PU WILEY-BLACKWELL

PI HOBOKEN

PA 111 RIVER ST, HOBOKEN 07030-5774, NJ USA

SN 1759-7706

EI 1759-7714

J9 THORAC CANCER

JI Thorac. Cancer

PD MAY

PY 2016

VL 7

IS 3

BP 310

EP 315

DI 10.1111/1759-7714.12329

PG 6

WC Oncology; Respiratory System

WE Science Citation Index Expanded (SCI-EXPANDED)

SC Oncology; Respiratory System

GA DK9EV

UT WOS:000375234500006

PM 27148416

OA Green Published, gold

DA 2022-05-19

ER

PT J

AU Wu, XY

Wang, ZT

Jiang, YA

Zhou, H

Li, A

Wei, YX

Bao, Z

Wang, DH

Zhao, JM

Chen, XH

Guo, YP

Dong, ZG

Liu, KD

AF Wu, Xiangyu

Wang, Zitong

Jiang, Yanan

Zhou, Hao

Li, Ang

Wei, Yaxing

Bao, Zhuo

Wang, Donghao

Zhao, Jimin

Chen, Xinhuan

Guo, Yaping

Dong, Zigang

Liu, Kangdong

TI Tegaserod Maleate Inhibits Esophageal Squamous Cell Carcinoma

Proliferation by Suppressing the Peroxisome Pathway

SO FRONTIERS IN ONCOLOGY

LA English

DT Article

DE esophageal squamous cell carcinoma; tegaserod maleate; proteome;

peroxisome; peroxisome membrane protein 11B; peroxisome membrane protein

13; patient-derived xenograft

ID CANCER; EXPRESSION

AB Esophageal squamous cell carcinoma (ESCC) and esophageal adenocarcinoma (EAC) are the two major types of esophageal cancer (EC). ESCC accounts for 90% of EC. Recurrence after primary treatment is the main reason for poor survival. Therefore, recurrence prevention is a promising strategy for extending the 5-year survival rate. Here, we found tegaserod maleate could inhibit ESCC proliferation both in vivo and in vitro. Proteomics analysis revealed that tegaserod maleate suppressed the peroxisome signaling pathway, in which the key molecules peroxisome membrane protein 11B (PEX11B) and peroxisome membrane protein 13 (PEX13) were downregulated. The immunofluorescence, catalase activity assay, and reactive oxygen species (ROS) confirmed that downregulation of these proteins was related to impaired peroxisome function. Furthermore, we found that PEX11B and PEX13 were highly expressed in ESCC, and knockout of PEX11B and PEX13 further demonstrated the antitumor effect of tegaserod maleate. Importantly, tegaserod maleate repressed ESCC tumor growth in a patient-derived xenograft (PDX) model in vivo. Our findings conclusively demonstrated that tegaserod maleate inhibits the proliferation of ESCC by suppressing the peroxisome pathway.

C1 [Wu, Xiangyu; Wang, Zitong; Jiang, Yanan; Zhou, Hao; Li, Ang; Wei, Yaxing; Bao, Zhuo; Wang, Donghao; Zhao, Jimin; Chen, Xinhuan; Guo, Yaping; Dong, Zigang; Liu, Kangdong] Zhengzhou Univ, Sch Basic Med Sci, Pathophysiol Dept, Zhengzhou, Peoples R China.

[Jiang, Yanan; Li, Ang; Bao, Zhuo; Wang, Donghao; Dong, Zigang; Liu, Kangdong] China US Henan Hormel Canc Inst, Zhengzhou, Peoples R China.

[Jiang, Yanan; Zhao, Jimin; Chen, Xinhuan; Guo, Yaping; Liu, Kangdong] Zhengzhou Univ, Prov Cooperat Innovat Ctr Canc Chemoprevent, Zhengzhou, Peoples R China.

[Jiang, Yanan; Zhao, Jimin; Dong, Zigang; Liu, Kangdong] State Key Lab Esophageal Canc Prevent & Treatment, Zhengzhou, Peoples R China.

[Dong, Zigang; Liu, Kangdong] Canc Chemoprevent Int Collaborat Lab, Zhengzhou, Peoples R China.

RP Dong, ZG; Liu, KD (corresponding author), Zhengzhou Univ, Sch Basic Med Sci, Pathophysiol Dept, Zhengzhou, Peoples R China.; Dong, ZG; Liu, KD (corresponding author), China US Henan Hormel Canc Inst, Zhengzhou, Peoples R China.; Liu, KD (corresponding author), Zhengzhou Univ, Prov Cooperat Innovat Ctr Canc Chemoprevent, Zhengzhou, Peoples R China.; Dong, ZG; Liu, KD (corresponding author), State Key Lab Esophageal Canc Prevent & Treatment, Zhengzhou, Peoples R China.; Dong, ZG; Liu, KD (corresponding author), Canc Chemoprevent Int Collaborat Lab, Zhengzhou, Peoples R China.

EM dongzg@zzu.edu.cn; kdliu@zzu.edu.cn

FU National Science & Technology Major Project Key New Drug Creation and

Manufacturing Program, China [2018ZX09711002]; National Natural Science

Foundations of China [81872335]; National Natural Science Youth

Foundation [81902486]; Natural Science Foundation of Henan

[161100510300]; Science and Technology Project of Henan Province

[212102310187]

FX This research was supported by the National Science & Technology Major

Project Key New Drug Creation and Manufacturing Program, China (No.

2018ZX09711002), National Natural Science Foundations of China (grant

number 81872335), National Natural Science Youth Foundation (grant

number 81902486), the Natural Science Foundation of Henan (grant number

161100510300), the Science and Technology Project of Henan Province

(grant number 212102310187).

CR Agrawal G, 2016, BBA-MOL CELL RES, V1863, P892, DOI 10.1016/j.bbamcr.2015.09.014

Ashburn TT, 2004, NAT REV DRUG DISCOV, V3, P673, DOI 10.1038/nrd1468

Ataee R, 2010, ARCH IRAN MED, V13, P120

Bonekamp NA, 2013, PLOS ONE, V8, DOI 10.1371/journal.pone.0053424

Cao JJ, 2021, CURR PROB CANCER, V45, DOI 10.1016/j.currproblcancer.2020.100638

Chandrashekar DS, 2017, NEOPLASIA, V19, P649, DOI 10.1016/j.neo.2017.05.002

Deori NM, 2018, BIOGERONTOLOGY, V19, P303, DOI 10.1007/s10522-018-9761-9

Emmanouilidis L, 2016, BBA-MOL CELL RES, V1863, P804, DOI 10.1016/j.bbamcr.2015.09.034

Evans BW, 2007, COCHRANE DB SYST REV, DOI 10.1002/14651858.CD003960.pub3

Glorieux C, 2017, BIOL CHEM, V398, P1095, DOI 10.1515/hsz-2017-0131

GOLDFISCHER S, 1973, SCIENCE, V182, P62, DOI 10.1126/science.182.4107.62

Gould SJ, 1996, J CELL BIOL, V135, P85, DOI 10.1083/jcb.135.1.85

Grancher A, 2018, B CANCER, V105, P171, DOI 10.1016/j.bulcan.2017.09.013

Heckman-Stoddard BM, 2017, DIABETOLOGIA, V60, P1639, DOI 10.1007/s00125-017-4372-6

Huang JJ, 2021, CANCERS, V13, DOI 10.3390/cancers13010141

Lismont C, 2015, FRONT CELL DEV BIOL, V3, DOI 10.3389/fcell.2015.00035

Liu W, 2020, J EXP CLIN CANC RES, V39, DOI 10.1186/s13046-020-1539-7

Lupberger J, 2019, GASTROENTEROLOGY, V157, P537, DOI 10.1053/j.gastro.2019.04.003

Marciniak TA, 2019, AM J THER, V26, pE417, DOI 10.1097/MJT.0000000000000947

Montague TG, 2014, NUCLEIC ACIDS RES, V42, pW401, DOI 10.1093/nar/gku410

Nagy A, 2018, SCI REP-UK, V8, DOI 10.1038/s41598-018-27521-y

Pennathur A, 2013, LANCET, V381, P400, DOI 10.1016/S0140-6736(12)60643-6

Rayan A, 2017, PLOS ONE, V12, DOI 10.1371/journal.pone.0187925

Schrader M, 2012, BBA-MOL BASIS DIS, V1822, P1343, DOI 10.1016/j.bbadis.2011.12.014

Shen XZ, 2021, INT J CANCER, V148, P1323, DOI 10.1002/ijc.33323

Sung HH, 2022, CLIN EXP HYPERTENS, V44, P146, DOI 10.1080/10641963.2021.2007943

Wanders RJA, 2006, ANNU REV BIOCHEM, V75, P295, DOI 10.1146/annurev.biochem.74.082803.133329

Xie YF, 2021, CANCER LETT, V510, P1, DOI 10.1016/j.canlet.2021.03.029

Xie YF, 2020, FRONT ONCOL, V10, DOI 10.3389/fonc.2020.01217

Xu ZK, 2017, PLOS PATHOG, V13, DOI 10.1371/journal.ppat.1006360

Yuan Q, 2019, AGING-US, V11, P9530, DOI 10.18632/aging.102402

Zhang L, 2020, INVEST NEW DRUG, V38, P311, DOI 10.1007/s10637-019-00790-8

NR 32

TC 2

Z9 2

U1 4

U2 7

PU FRONTIERS MEDIA SA

PI LAUSANNE

PA AVENUE DU TRIBUNAL FEDERAL 34, LAUSANNE, CH-1015, SWITZERLAND

SN 2234-943X

J9 FRONT ONCOL

JI Front. Oncol.

PD AUG 4

PY 2021

VL 11

AR 683241

DI 10.3389/fonc.2021.683241

PG 12

WC Oncology

WE Science Citation Index Expanded (SCI-EXPANDED)

SC Oncology

GA UD9XQ

UT WOS:000687553700001

PM 34422635

OA gold, Green Published

DA 2022-05-19

ER

PT J

AU Liu, HS

Zhang, J

Luo, XY

Zeng, M

Xu, LQ

Zhang, QX

Liu, H

Guo, JL

Xu, LL

AF Liu, Huasong

Zhang, Jun

Luo, Xiangyu

Zeng, Min

Xu, Liqiang

Zhang, Qunxian

Liu, Hua

Guo, Jialong

Xu, Lanlan

TI Overexpression of the Long Noncoding RNA FOXD2-AS1 Promotes Cisplatin

Resistance in Esophageal Squamous Cell Carcinoma Through the

miR-195/Akt/mTOR Axis

SO ONCOLOGY RESEARCH

LA English

DT Article

DE FOXD2-AS1; Esophageal squamous cell carcinoma (ESCC); Cisplatin

resistance; miR-195; Akt/mTOR signal pathway

ID CANCER CELLS; LNCRNA; PROLIFERATION; MIR-195

AB Emerging evidence has demonstrated that long noncoding RNAs (lncRNAs) mediate the development of esophageal squamous cell carcinoma (ESCC) via various pathophysiological pathways. This study explored the impact of the lncRNA FOXD2-AS1 on cisplatin resistance in ESCC and its possible mechanisms. Upregulation of FOXD2-AS was detected in patients with ESCC and ESCC cells that are resistant to cisplatin. In an in vitro assay, knockdown of FOXD2-AS1 noticeably inhibited cell invasion and growth, triggered cell death, and repressed the stimulation of the Akt/mTOR axis in cisplatin-resistant ESCC cells (TE-1/DDP). Conversely, the overexpression of FOXD2-AS1 remarkably increased cell invasion and growth, repressed cell death, and triggered the stimulation of the Akt/mTOR axis in TE-1/DDP cells. These findings, along with bioinformatics and validation tests, showed that FOXD2-AS1 targeted miR-195 by acting as a competing endogenous RNA. FOXD2-AS1/miR-195/Akt/mTOR axis plays a crucial role in resistance to cisplatin in ESCC cells, offering an innovative strategy to treat ESCC.

C1 [Liu, Huasong; Zhang, Jun; Luo, Xiangyu; Zeng, Min; Xu, Liqiang; Zhang, Qunxian; Liu, Hua; Guo, Jialong] Hubei Univ Med, Dept Cardiothorac Surg, Taihe Hosp, 30 Renmin South Rd, Shiyan, Hubei, Peoples R China.

[Xu, Lanlan] Hubei Univ Med, 30 Renmin South Rd, Shiyan, Hubei, Peoples R China.

RP Guo, JL (corresponding author), Hubei Univ Med, Dept Cardiothorac Surg, Taihe Hosp, 30 Renmin South Rd, Shiyan, Hubei, Peoples R China.; Xu, LL (corresponding author), Hubei Univ Med, 30 Renmin South Rd, Shiyan, Hubei, Peoples R China.

EM gj19988@163.com; 215518542@qq.com

FU Youth Talent Project of Health Commission of Hubei Province [WJ2019Q014]

FX This work was supported by the Youth Talent Project of Health Commission

of Hubei Province (WJ2019Q014). The authors declare no conflicts of

interest.

CR An Q, 2018, BIOMED PHARMACOTHER, V103, P415, DOI 10.1016/j.biopha.2018.03.138

Chabre O, 2013, ENDOCR-RELAT CANCER, V20, P579, DOI 10.1530/ERC-13-0051

Chang YH, 2018, ONCOL REP, V40, P3625, DOI 10.3892/or.2018.6752

Che Y, 2018, CELL DEATH DIS, V9, DOI 10.1038/s41419-018-0808-2

Chen G, 2018, GENE, V645, P76, DOI 10.1016/j.gene.2017.12.026

Chen ZZ, 2019, BIOMED PHARMACOTHER, V109, P2228, DOI 10.1016/j.biopha.2018.11.049

Denaro N, 2019, MOL ONCOL, V13, P61, DOI 10.1002/1878-0261.12413

Fanelli Giuseppe Nicolo, 2018, Noncoding RNA Res, V3, P195, DOI 10.1016/j.ncrna.2018.10.001

Fanucchi S, 2019, NAT GENET, V51, P138, DOI 10.1038/s41588-018-0298-2

Fu MG, 2013, FEBS LETT, V587, P3471, DOI 10.1016/j.febslet.2013.08.036

Gong J, 2019, J CELL PHYSIOL, V234, P12884, DOI 10.1002/jcp.27953

Guo XF, 2018, THORAC CANCER, V9, P1801, DOI 10.1111/1759-7714.12882

Hsiao YT, 2018, INDIAN J OPHTHALMOL, V66, P1215, DOI 10.4103/ijo.IJO_259_18

Huang XJ, 2018, ONCOGENE, V37, P6316, DOI 10.1038/s41388-018-0410-1

Jiang D, 2018, CELL DEATH DIS, V10, DOI 10.1038/s41419-018-1236-z

Kang K, 2018, ARCH MED SCI, V14, P752, DOI 10.5114/aoms.2018.73713

Kang M, 2018, J EXP CLIN CANC RES, V37, DOI 10.1186/s13046-018-0845-9

Li J, 2018, MOL NUTR FOOD RES, V20

Liang RB, 2017, SCI REP-UK, V7, DOI 10.1038/s41598-017-06634-w

Lin Y, 2018, EUR REV MED PHARMACO, V22, P8690, DOI 10.26355/eurrev_201812_16634

Liu JG, 2018, ONCOL REP, V39, P1631, DOI 10.3892/or.2018.6268

Lyu JH, 2018, RADIAT ONCOL, V13, DOI 10.1186/s13014-018-1183-y

McKinsey TA, 2018, F1000RES, V7

Murakami Y, 2018, J RADIAT RES, V59, P616, DOI 10.1093/jrr/rry047

Qiao Y, 2018, ONCOGENE, V37, P873, DOI 10.1038/onc.2017.387

Ren WQ, 2019, J CELL BIOCHEM, V120, P5417, DOI 10.1002/jcb.27820

Sadeghpour S, 2019, MOL BIOL REP, V46, P991, DOI 10.1007/s11033-018-4556-2

Song X, 2019, EPIGENOMICS-UK, V11, P381, DOI 10.2217/epi-2018-0182

Su F, 2018, CELL DEATH DIS, V9, DOI 10.1038/s41419-018-0275-9

Sun NN, 2014, INT J CLIN EXP PATHO, V7, P6871

Wang BY, 2019, ANN THORAC SURG, V107, P1060, DOI 10.1016/j.athoracsur.2018.11.036

Wang HJ, 2017, APOPTOSIS, V22, P1157, DOI 10.1007/s10495-017-1395-x

Wang YX, 2018, GENE, V667, P25, DOI 10.1016/j.gene.2018.05.030

Xu CH, 2018, CELL BIOSCI, V8, DOI 10.1186/s13578-018-0260-0

Xu QY, 2019, J CELL BIOCHEM, V120, P8300, DOI 10.1002/jcb.28113

Xu TP, 2018, ONCOGENE, V37, P5020, DOI 10.1038/s41388-018-0308-y

Yang X, 2017, EUR REV MED PHARMACO, V21, P3586

Yu J, 2019, CANCER MED-US, V8, P21, DOI 10.1002/cam4.1731

Yu S, 2017, ONCOTARGET, V8, P98014, DOI 10.18632/oncotarget.20439

Zhang Y, 2018, J FUNCT FOODS, V40, P573, DOI 10.1016/j.jff.2017.11.045

Zhao JJ, 2018, CANCER MANAG RES, V10, P6457, DOI 10.2147/CMAR.S181949

Zhou B, 2017, CELL PHYSIOL BIOCHEM, V44, P1867, DOI 10.1159/000485876

Zhou Y, 2018, NEUROPSYCH DIS TREAT, V14, P3219, DOI 10.2147/NDT.S178435

NR 43

TC 7

Z9 7

U1 2

U2 6

PU COGNIZANT COMMUNICATION CORP

PI PUTNAM VALLEY

PA 18 PEEKSKILL HOLLOW RD, PO BOX 37, PUTNAM VALLEY, NY 10579 USA

SN 0965-0407

EI 1555-3906

J9 ONCOL RES

JI Oncol. Res.

PY 2020

VL 28

IS 1

BP 65

EP 73

DI 10.3727/096504019X15656904013079

PG 9

WC Oncology

WE Science Citation Index Expanded (SCI-EXPANDED)

SC Oncology

GA KK8NR

UT WOS:000512993600008

PM 31558183

OA hybrid, Green Published

DA 2022-05-19

ER

PT J

AU Liu, Y

Ren, ZH

Yuan, L

Xu, SN

Yao, ZH

Qiao, L

Li, K

AF Liu, Ying

Ren, Zhonghai

Yuan, Long

Xu, Shuning

Yao, Zhihua

Qiao, Lei

Li, Ke

TI Paclitaxel plus cisplatin vs. 5-fluorouracil plus cisplatin as

first-line treatment for patients with advanced squamous cell esophageal

cancer

SO AMERICAN JOURNAL OF CANCER RESEARCH

LA English

DT Article

DE Paclitaxel; cisplatin; 5-fluorouracil; squamous cell esophageal cancer;

first-line treatment

ID RANDOMIZED PHASE-II; CARCINOMA; CHEMOTHERAPY; TRIAL; NEDAPLATIN;

RECURRENT; ADENOCARCINOMA; INFUSION; PATTERN

AB Paclitaxel plus cisplatin and 5-fluorouracil plus cisplatin treatments are effective strategies for patients with advanced esophageal squamous cell carcinoma. This study was to evaluate the safety and efficacy of paclitaxel plus cisplatin and 5-fluorouracil plus cisplatin as first-line chemotherapy for patients with advanced esophageal squamous cell carcinoma. A total of 398 patients with advanced esophageal squamous cell carcinoma who received chemotherapy were included and divided into 2 groups: paclitaxel plus cisplatin group and 5-fluorouracil plus cisplatin group. 195 patients received paclitaxel plus cisplatin and 203 patients received 5-fluorouracil plus cisplatin. The objective response rates were 42.5% and 38.4% for paclitaxel plus cisplatin group and 5-fluorouracil plus cisplatin group, respectively (P=0.948). The median progression-free survival was 7.85 months (95% CI, 6.77-8.94 months) for the paclitaxel plus cisplatin group and 6.53 months (95% CI, 5.63-7.43 months) for the 5-fluorouracil plus cisplatin group with significant difference (P=0.02). The median overall survival was 13.46 months (95% CI, 12.01-14.91 months) for the paclitaxel plus cisplatin group and 12.67 months (95% CI, 11.87-13.47 months) for the 5-fluorouracil plus cisplatin group (P=0.204). The first-line chemotherapy of paclitaxel plus cisplatin had better median progression-free survival than 5-fluorouracil plus cisplatin in patients with advanced esophageal squamous cell carcinoma with tolerable toxicities.

C1 [Liu, Ying; Xu, Shuning; Yao, Zhihua; Qiao, Lei; Li, Ke] Zhengzhou Univ, Affiliated Canc Hosp, Henan Canc Hosp, Dept Med Oncol, 127th Dongming Rd, Zhengzhou 450003, Henan, Peoples R China.

[Yuan, Long] Zhengzhou Univ, Affiliated Canc Hosp, Henan Canc Hosp, Dept Surg Oncol, Zhengzhou, Henan, Peoples R China.

[Ren, Zhonghai] Nanyang City Ctr Hosp, Dept Med Oncol, Nanyang, Henan, Peoples R China.

RP Liu, Y (corresponding author), Zhengzhou Univ, Affiliated Canc Hosp, Henan Canc Hosp, Dept Med Oncol, 127th Dongming Rd, Zhengzhou 450003, Henan, Peoples R China.

EM yaya7207@126.com

FU National Natural Science Foundation of China [81201954]

FX This work was supported by National Natural Science Foundation of China,

(grant number 81201954). We wish to thank Dr. Peng Liu for assistance in

preparation of the manuscript.

CR AJANI JA, 1994, J NATL CANCER I, V86, P1086, DOI 10.1093/jnci/86.14.1086

Bleiberg H, 1997, EUR J CANCER, V33, P1216, DOI 10.1016/S0959-8049(97)00088-9

Cao WG, 2009, JPN J CLIN ONCOL, V39, P582, DOI 10.1093/jjco/hyp058

Du JP, 2015, ANTI-CANCER DRUG, V26, P101, DOI 10.1097/CAD.0000000000000170

Hayashi K, 2001, JPN J CLIN ONCOL, V31, P419, DOI 10.1093/jjco/hye090

He YF, 2013, WORLD J GASTROENTERO, V19, P5910, DOI 10.3748/wjg.v19.i35.5910

Herszenyi L, 2010, EUR REV MED PHARMACO, V14, P249

Huang J, 2013, MED ONCOL, V30, DOI 10.1007/s12032-012-0343-4

Ilson DH, 2007, ANN ONCOL, V18, P898, DOI 10.1093/annonc/mdm004

Ilson DH, 2000, CANCER J, V6, P316

Lorenzen S, 2009, ANN ONCOL, V20, P1667, DOI 10.1093/annonc/mdp069

Mariette C, 2003, CANCER-AM CANCER SOC, V97, P1616, DOI 10.1002/cncr.11228

Nakagawa S, 2004, J AM COLL SURGEONS, V198, P205, DOI 10.1016/j.jamcollsurg.2003.10.005

Petrasch S, 1998, BRIT J CANCER, V78, P511, DOI 10.1038/bjc.1998.524

Vestermark Lene Weber, 2008, Ugeskr Laeger, V170, P633

Wang An-Hui, 2014, World J Gastrointest Pathophysiol, V5, P335, DOI 10.4291/wjgp.v5.i3.335

Wilke H, 2014, LANCET ONCOL, V15, P1224, DOI 10.1016/S1470-2045(14)70420-6

Zhang XD, 2008, AM J CLIN ONCOL-CANC, V31, P29, DOI 10.1097/COC.0b013e3181131ca9

NR 18

TC 22

Z9 25

U1 0

U2 3

PU E-CENTURY PUBLISHING CORP

PI MADISON

PA 40 WHITE OAKS LN, MADISON, WI 53711 USA

SN 2156-6976

J9 AM J CANCER RES

JI Am. J. Cancer Res.

PY 2016

VL 6

IS 10

BP 2345

EP 2350

PG 6

WC Oncology

WE Science Citation Index Expanded (SCI-EXPANDED)

SC Oncology

GA EB8CJ

UT WOS:000387618300018

PM 27822423

DA 2022-05-19

ER

PT J

AU Nishiumi, S

Fujigaki, S

Kobayashi, T

Kojima, T

Ito, Y

Daiko, H

Kato, K

Shoji, H

Kodama, Y

Honda, K

Yoshida, M

AF Nishiumi, Shin

Fujigaki, Seiji

Kobayashi, Takashi

Kojima, Takashi

Ito, Yoshinori

Daiko, Hiroyuki

Kato, Ken

Shoji, Hirokazu

Kodama, Yuzo

Honda, Kazufumi

Yoshida, Masaru

TI Metabolomics-based Discovery of Serum Biomarkers to Predict the

Side-effects of Neoadjuvant Chemoradiotherapy for Esophageal Squamous

Cell Carcinoma

SO ANTICANCER RESEARCH

LA English

DT Article

DE Esophageal squamous cell carcinoma; metabolomics; biomarker;

side-effect; neoadjuvant chemoradiotherapy; 5-flurouracil; cisplatin

ID IN-VITRO; CHEMOTHERAPY; SURGERY; CANCER

AB Background/Aim: Neoadjuvant chemoradiotherapy has side-effects that adversely affect patients' quality of life. The aim of this study was to identify serum metabolite biomarkers that might be used to predict the side-effects of neoadjuvant chemoradiotherapy for esophageal squamous cell carcinoma (ESCC). Patients and Methods: Metabolomic analysis of serum samples from 26 patients with ESCC that were collected before neoadjuvant chemoradiotherapy was performed. The metabolites associated with hematological toxicity or nephrotoxicity were evaluated. Results: Serum levels of glutaric acid, glucuronic acid, and cystine were significantly higher in hematological toxicity, and phosphatidylcholines and phosphatidylethanolamines exhibited a tendency to be higher in those with hematological toxicity. The serum level of pyruvic acid was significantly lower in nephrotoxicity, and lysophosphatidylcholines and lysophosphatidylethanolamines tended to be lower in those with nephrotoxicity. Conclusion: Our study found that serum levels of some metabolites differed significantly between patients with and without hematological or renal side-effects. These metabolites may be useful biomarkers for predicting hematological toxicity or nephrotoxicity after neoadjuvant chemoradiotherapy for ESCC.

C1 [Nishiumi, Shin; Fujigaki, Seiji; Kobayashi, Takashi; Kodama, Yuzo; Yoshida, Masaru] Kobe Univ, Grad Sch Med, Dept Internal Med, Div Gastroenterol, Kobe, Hyogo, Japan.

[Kojima, Takashi] Natl Canc Ctr Hosp East, Dept Gastrointestinal Oncol, Chiba, Japan.

[Ito, Yoshinori] Natl Canc Ctr, Dept Radiat Oncol, Tokyo, Japan.

[Ito, Yoshinori] Showa Univ, Sch Med, Dept Radiat Oncol, Tokyo, Japan.

[Daiko, Hiroyuki] Natl Canc Ctr, Dept Esophageal Surg, Tokyo, Japan.

[Kato, Ken; Shoji, Hirokazu] Natl Canc Ctr, Dept Gastrointestinal Med Oncol, Tokyo, Japan.

[Honda, Kazufumi] Natl Canc Ctr, Dept Biomarkers Early Detect Canc, Tokyo, Japan.

[Honda, Kazufumi; Yoshida, Masaru] Japan Agcy Med Res & Dev, AMED CREST, Tokyo, Japan.

[Yoshida, Masaru] Kobe Univ, Grad Sch Med, Dept Internal Related, Div Metabol Res, Kobe, Hyogo, Japan.

RP Nishiumi, S; Yoshida, M (corresponding author), Kobe Univ, Grad Sch Med, Dept Internal Med, Div Gastroenterol,Chuo Ku, 7-5-1 Kusunoki Cho, Kobe, Hyogo 6500017, Japan.

EM nishiums@med.kobe-u.ac.jp

RI Nishiumi, Shin/ABD-5775-2020; KOBAYASHI, Takashi/AAM-7549-2021

OI Nishiumi, Shin/0000-0003-2126-3013; KOBAYASHI,

Takashi/0000-0002-9202-7643

FU Japan Society for the Promotion of Science [16H05227]; AMED-CREST by the

Japan Agency for Medical Research and Development [18gm0710013h0005]

FX This study was supported in part by a Grant-in-Aid for Scientific

Research (B) from the Japan Society for the Promotion of Science

(16H05227) (M.Y.) and the AMED-CREST by the Japan Agency for Medical

Research and Development (18gm0710013h0005) (M.Y.).

CR Allum WH, 2009, J CLIN ONCOL, V27, P5062, DOI 10.1200/JCO.2009.22.2083

Arany I, 2003, SEMIN NEPHROL, V23, P460, DOI 10.1016/S0270-9295(03)00089-5

BAJORIN DF, 1986, CANCER RES, V46, P5969

Bueschl C, 2013, ANAL BIOANAL CHEM, V405, P27, DOI 10.1007/s00216-012-6375-y

Ferreira GD, 2005, NEUROCHEM RES, V30, P1123, DOI 10.1007/s11064-005-7711-9

Fitzmaurice C, 2018, JAMA ONCOL, V4, P1553, DOI 10.1001/jamaoncol.2018.2706

Fujigaki S, 2018, BIOMARK MED, V12, P827, DOI 10.2217/bmm-2017-0449

Harmon RC, 2006, TOXICOL APPL PHARM, V213, P179, DOI 10.1016/j.taap.2005.10.008

Hashimoto J, 2019, INT J CLIN ONCOL, V24, P60, DOI 10.1007/s10147-018-1336-x

Hishinuma E, 2018, DRUG METAB DISPOS, V46, P1083, DOI 10.1124/dmd.118.081737

Kelsen DP, 2007, J CLIN ONCOL, V25, P3719, DOI 10.1200/JCO.2006.10.4760

Marques FD, 2003, BRAIN RES, V964, P153, DOI 10.1016/S0006-8993(02)04118-5

Ozkok A, 2014, BIOMED RES INT, V2014, DOI 10.1155/2014/967826

Pabla N, 2008, KIDNEY INT, V73, P994, DOI 10.1038/sj.ki.5002786

Parry K, 2015, ANN SURG ONCOL, V22, pS1292, DOI 10.1245/s10434-015-4840-5

SALAHUDEEN AK, 1991, J CLIN INVEST, V88, P1886, DOI 10.1172/JCI115511

Shapiro J, 2015, LANCET ONCOL, V16, P1090, DOI 10.1016/S1470-2045(15)00040-6

Sjoquist KM, 2011, LANCET ONCOL, V12, P681, DOI 10.1016/S1470-2045(11)70142-5

Sobin LH, 2011, TNM CLASSIFICATION M, P1

Spratlin JL, 2009, CLIN CANCER RES, V15, P431, DOI 10.1158/1078-0432.CCR-08-1059

van Hagen P, 2012, NEW ENGL J MED, V366, P2074, DOI 10.1056/NEJMoa1112088

Zhang P, 2017, J PROTEOME RES, V16, P1753, DOI 10.1021/acs.jproteome.7b00014

NR 22

TC 5

Z9 6

U1 0

U2 9

PU INT INST ANTICANCER RESEARCH

PI ATHENS

PA EDITORIAL OFFICE 1ST KM KAPANDRITIOU-KALAMOU RD KAPANDRITI, PO BOX 22,

ATHENS 19014, GREECE

SN 0250-7005

EI 1791-7530

J9 ANTICANCER RES

JI Anticancer Res.

PD JAN

PY 2019

VL 39

IS 1

BP 519

EP 526

DI 10.21873/anticanres.13143

PG 8

WC Oncology

WE Science Citation Index Expanded (SCI-EXPANDED)

SC Oncology

GA HF7IS

UT WOS:000454413400070

PM 30591504

OA Bronze

DA 2022-05-19

ER

PT J

AU Wang, WW

Wei, CG

Li, P

Wang, L

Li, WC

Chen, KS

Zhang, JY

Zhang, WJ

Jiang, GZ

AF Wang, Weiwei

Wei, Chengguo

Li, Pan

Wang, Li

Li, Wencai

Chen, Kuisheng

Zhang, Jianying

Zhang, Weijia

Jiang, Guozhong

TI Integrative analysis of mRNA and lncRNA profiles identified pathogenetic

lncRNAs in esophageal squamous cell carcinoma

SO GENE

LA English

DT Article

DE Genome-wide screening; Esophageal squamous cell carcinoma; mRNA; IncRNA

ID LONG NONCODING RNA; BREAST-CANCER; HEPATOCELLULAR-CARCINOMA;

PROGRESSION; METASTASIS; EXPRESSION; SIGNATURE; MIGRATION; PREDICTS;

REVEALS

AB Systems biology approaches can help understand pathogenesis of complex human diseases like cancers for identification of potential new therapeutic targets. Here in this study, we performed genome-wide screening for mRNA and IncRNA profiles in esophageal cancer to identify the novel cancer-related mRNA and IncRNA in esophageal squamous cell carcinoma (ESCC). We identified 1260 up-regulated/1445 down-regulated mRNAs and 402 up-regulated/741 down-regulated lncRNAs. Further functional analysis revealed differentially expressed genes (DEGs) of mRNA and IncRNA are related to different pathways. mRNA pathways are mainly involved in cell cycles while 1ncRNA pathways are for regulation and metabolic procession. Differentially expressed mRNAs/lncRNAs were validated with qPCR. At last, mRNA and IncRNA co-expression network were built and highly-connected networks were identified, which may provide a mechanism of mRNA expression regulation by 1ncRNA. In together, we used next generation sequencing data to explore the co-expression networks of 1ncRNA and mRNA, which may elucidate the functions and mechanisms of IncRNA in ESCC.

C1 [Wang, Weiwei; Li, Pan; Wang, Li; Li, Wencai; Chen, Kuisheng; Jiang, Guozhong] Zhengzhou Univ, Affiliated Hosp 1, Dept Pathol, 1 Jianshe East Rd, Zhengzhou 450052, Henan, Peoples R China.

[Wang, Weiwei; Li, Pan; Wang, Li; Li, Wencai; Chen, Kuisheng; Jiang, Guozhong] Zhengzhou Univ, Sch Basic Med, Dept Pathol, Zhengzhou 450052, Henan, Peoples R China.

[Wang, Weiwei; Li, Pan; Wang, Li; Li, Wencai; Chen, Kuisheng; Jiang, Guozhong] Henan Key Lab Tumor Pathol, Zhengzhou 450052, Henan, Peoples R China.

[Wei, Chengguo; Zhang, Weijia] Icahn Sch Med Mt Sinai, Dept Med, Div Nephrol, 1468 Madison Ave,Annenberge 23-16, New York, NY 10029 USA.

[Zhang, Jianying] Zhengzhou Univ, Inst Med & Pharmaceut Sci, Zhengzhou 450052, Henan, Peoples R China.

RP Jiang, GZ (corresponding author), Zhengzhou Univ, Affiliated Hosp 1, Dept Pathol, 1 Jianshe East Rd, Zhengzhou 450052, Henan, Peoples R China.; Zhang, WJ (corresponding author), Icahn Sch Med Mt Sinai, Dept Med, Div Nephrol, 1468 Madison Ave,Annenberge 23-16, New York, NY 10029 USA.

EM weijia.zhang@mssm.edu; guozhongjiang@zzu.edu.cn

FU National Natural Science Foundation of China [81272371]; Major Project

of Science and Technology in Henan Province [161100311400]; Key Research

Projects of Henan Higher Education [18A310033]; Youth Innovation Fund of

The First Affiliated Hospital of Zhengzhou University

FX This study was supported by grant from National Natural Science

Foundation of China (No. 81272371), and the Major Project of Science and

Technology in Henan Province (No.161100311400), and the Key Research

Projects of Henan Higher Education (No.18A310033), and the Youth

Innovation Fund of The First Affiliated Hospital of Zhengzhou University

to Weiwei Wang & Pan Li.

CR Beermann J, 2016, PHYSIOL REV, V96, P1297, DOI 10.1152/physrev.00041.2015

Beltran AS, 2014, ONCOGENE, V33, P4767, DOI 10.1038/onc.2013.422

Bhan A, 2017, CANCER RES, V77, P3965, DOI 10.1158/0008-5472.CAN-16-2634

Cao W, 2013, INT J GENOMICS, V2013, DOI 10.1155/2013/480534

Chen DH, 2013, CANCER RES, V73, P5821, DOI 10.1158/0008-5472.CAN-13-1080

Chen ZL, 2014, CANCER LETT, V350, P34, DOI 10.1016/j.canlet.2014.04.013

Dai DN, 2017, J MOL MED, V95, P873, DOI 10.1007/s00109-017-1537-1

Douet-Guilbert N, 2017, HEMATOL ONCOL, V35, P385, DOI 10.1002/hon.2268

Evans JR, 2016, J CLIN INVEST, V126, P2775, DOI 10.1172/JCI84421

Ferlay J, 2015, INT J CANCER, V136, pE359, DOI 10.1002/ijc.29210

Guo LY, 2017, ONCOTARGET, V8, P68483, DOI 10.18632/oncotarget.19590

Han D, 2017, HEPATOLOGY, V66, P1151, DOI 10.1002/hep.29270

Huang MD, 2015, MOL CANCER, V14, DOI 10.1186/s12943-015-0431-0

Huarte M, 2015, NAT MED, V21, P1253, DOI 10.1038/nm.3981

Ito T, 2008, CANCER RES, V68, P3214, DOI 10.1158/0008-5472.CAN-07-3043

Li H, 2009, BIOINFORMATICS, V25, P1754, DOI 10.1093/bioinformatics/btp324

Li W, 2014, GASTROENTEROLOGY, V146, P1714, DOI 10.1053/j.gastro.2014.03.002

Li X, 2013, BRIT J CANCER, V109, P2266, DOI 10.1038/bjc.2013.548

Liu H, 2012, BMC CANCER, V12, DOI 10.1186/1471-2407-12-583

Ma S, 2012, GASTROENTEROLOGY, V143, P675, DOI 10.1053/j.gastro.2012.06.007

Ohashi S, 2015, GASTROENTEROLOGY, V149, P1700, DOI 10.1053/j.gastro.2015.08.054

Pan ZW, 2016, CANCER MED-US, V5, P2442, DOI 10.1002/cam4.770

Pandey GK, 2014, CANCER CELL, V26, P722, DOI 10.1016/j.ccell.2014.09.014

PASTUSZAKLEWANDOSK, 2016, MED ONCOL, V33, DOI DOI 10.1007/S12032-016-0791-3

Saeed AI, 2003, BIOTECHNIQUES, V34, P374, DOI 10.2144/03342mt01

Sawada G, 2016, GASTROENTEROLOGY, V150, P1171, DOI 10.1053/j.gastro.2016.01.035

Schmitt AM, 2016, CANCER CELL, V29, P452, DOI 10.1016/j.ccell.2016.03.010

Song YM, 2014, NATURE, V509, P91, DOI 10.1038/nature13176

Srijakotre N, 2017, BIOCHEM SOC T, V45, P963, DOI 10.1042/BST20160269

Su H, 2011, CLIN CANCER RES, V17, P2955, DOI 10.1158/1078-0432.CCR-10-2724

Tai IT, 2008, DRUG RESIST UPDATE, V11, P231, DOI 10.1016/j.drup.2008.08.005

Tanaka M, 2017, HUM PATHOL, V66, P177, DOI 10.1016/j.humpath.2017.06.013

Wu YY, 2017, MOL CANCER, V16, DOI 10.1186/s12943-017-0715-7

Xu XP, 2005, CANCER RES, V65, P130

Yang Y, 2018, J NATL CANC I, V110

Yao J, 2016, INT J ONCOL, V48, P2543, DOI 10.3892/ijo.2016.3457

NR 36

TC 20

Z9 20

U1 1

U2 21

PU ELSEVIER SCIENCE BV

PI AMSTERDAM

PA PO BOX 211, 1000 AE AMSTERDAM, NETHERLANDS

SN 0378-1119

EI 1879-0038

J9 GENE

JI Gene

PD JUN 30

PY 2018

VL 661

BP 169

EP 175

DI 10.1016/j.gene.2018.03.066

PG 7

WC Genetics & Heredity

WE Science Citation Index Expanded (SCI-EXPANDED)

SC Genetics & Heredity

GA GG2GH

UT WOS:000432507700019

PM 29604464

DA 2022-05-19

ER

PT J

AU Salehi, S

Jafarian, AH

Forghanifard, MM

AF Salehi, Somayeh

Jafarian, Amir Hossein

Forghanifard, Mohammad Mandi

TI Expression analysis of BRUCE protein in esophageal squamous cell

carcinoma

SO ANNALS OF DIAGNOSTIC PATHOLOGY

LA English

DT Article

DE Bruce; IAP; Esophageal squamous cell carcinoma; Immunohistochemistry;

Immunocytochemistry; Apoptosis

ID APOPTOSIS PROTEINS; APOLLON; GENE; IAP; INHIBITOR; PATHWAY; P53

AB Apoptosis is a form of cell death in response to diverse stressful physiological or pathological stimuli. One of the most important gene families involved in apoptosis is inhibitors of apoptosis. As a member of inhibitors of apoptosis, BRUCE can suppress apoptosis and promote cell division. Because esophageal squamous cell carcinoma (ESCC) cells, as well as other cancer cells, are immortal, our aim in this study was to analyze BRUCE protein expression in ESCC and evaluate its correlation with tumoral clinicopathologic features. Fifty ESCC specimens were examined for BRUCE protein expression using immunohistochemistry. A defined scoring method was applied. BRUCE protein was detected in 82% of tumors. Tumor progression stage and invasion depth correlated significantly with BRUCE protein expression (P = .019 and .005, respectively). Furthermore, association of BRUCE expression with tumor location was near significant (P = .058). The correlation of BRUCE overexpression in ESCC and disease aggressiveness may confirm the importance of BRUCE in ESCC progression and invasiveness. Therefore, BRUCE protein may be a molecular marker for aggressive ESCC and, thus, a potential therapeutic target to inhibit tumor cell progression and invasion. (C) 2016 Elsevier Inc. All rights reserved.

C1 [Salehi, Somayeh; Forghanifard, Mohammad Mandi] Islamic Azad Univ, Damghan Branch, Dept Biol, Damghan, Iran.

[Jafarian, Amir Hossein] Mashhad Univ Med Sci, Fac Med, Ghaem Hosp, Canc Mol Res Ctr, Mashhad, Iran.

RP Forghanifard, MM (corresponding author), Islamic Azad Univ, Cell & Mol Biol, Dept Biol, Damghan Branch, POB 3671639998,Cheshmeh Ali Blvd,Sadel Sq, Damghan, Iran.

EM forghanifard@gmail.com

RI Forghanifard, Mohammad Mahdi/J-9410-2014

OI Forghanifard, Mohammad Mahdi/0000-0002-7691-6897; salehi,

somayeh/0000-0002-4328-4029

CR Ando N, 1997, J THORAC CARDIOV SUR, V114, P205, DOI 10.1016/S0022-5223(97)70146-6

Bartke T, 2004, MOL CELL, V14, P801, DOI 10.1016/j.molcel.2004.05.018

Bianchini M, 2006, INT J ONCOL, V29, P83

Ch W, 2002, UICC TNM CLASSIFICAT

Charles Brunicardi F, 2010, SHWARTZS PRINCIPLES, V2, P862

Chen ZH, 1999, BIOCHEM BIOPH RES CO, V264, P847, DOI 10.1006/bbrc.1999.1585

Chu L, 2008, GENE THER, V15, P484, DOI 10.1038/gt.2008.6

Ghodsi M, 2015, INT J SURG, V13, P193, DOI 10.1016/j.ijsu.2014.11.036

Hanahan D, 2011, CELL, V144, P646, DOI 10.1016/j.cell.2011.02.013

Hao YY, 2004, NAT CELL BIOL, V6, P849, DOI 10.1038/ncb1159

Lopergolo A, 2009, BRIT J CANCER, V100, P739, DOI 10.1038/sj.bjc.6604927

Lopez J, 2010, CURR OPIN CELL BIOL, V22, P872, DOI 10.1016/j.ceb.2010.08.025

Pohl C, 2008, CELL, V132, P832, DOI 10.1016/j.cell.2008.01.012

Qiu XB, 2004, EMBO J, V23, P800, DOI 10.1038/sj.emboj.7600075

Qiu XB, 2005, J BIOL CHEM, V280, P174, DOI 10.1074/jbc.M411430200

Ren JY, 2005, P NATL ACAD SCI USA, V102, P565, DOI 10.1073/pnas.0408744102

Sekine K, 2005, BIOCHEM BIOPH RES CO, V330, P279, DOI 10.1016/j.bbrc.2005.02.165

Sung KW, 2007, CLIN CANCER RES, V13, P5109, DOI 10.1158/1078-0432.CCR-07-0693

Vucic D, 2007, CLIN CANCER RES, V13, P5995, DOI 10.1158/1078-0432.CCR-07-0729

Zhou SL, 2010, WORLD J GASTROENTERO, V16, P2348, DOI 10.3748/wjg.v16.i19.2348

NR 20

TC 3

Z9 3

U1 0

U2 3

PU ELSEVIER SCIENCE INC

PI NEW YORK

PA 360 PARK AVE SOUTH, NEW YORK, NY 10010-1710 USA

SN 1092-9134

EI 1532-8198

J9 ANN DIAGN PATHOL

JI Ann. Diagn. Pathol.

PD OCT

PY 2016

VL 24

BP 47

EP 51

DI 10.1016/j.anndiagpath.2016.07.003

PG 5

WC Pathology

WE Science Citation Index Expanded (SCI-EXPANDED)

SC Pathology

GA DX1WJ

UT WOS:000384157400011

PM 27649954

DA 2022-05-19

ER

PT J

AU Wang, W

Wang, ZW

Zhao, J

Wei, M

Zhu, XH

He, Q

Ling, TL

Chen, XY

Cao, Z

Zhang, YX

Liu, L

Shi, MX

AF Wang, Wei

Wang, Zhiwei

Zhao, Jun

Wei, Min

Zhu, Xinghua

He, Qi

Ling, Tianlong

Chen, Xiaoyan

Cao, Ziang

Zhang, Yixin

Liu, Lei

Shi, Minxin

TI A novel molecular and clinical staging model to predict survival for

patients with esophageal squamous cell carcinoma

SO ONCOTARGET

LA English

DT Article

DE esophageal squamous cell carcinoma; UBE2C; MGP; survival

ID MATRIX GLA-PROTEIN; UBIQUITIN-CONJUGATING ENZYME; POOR-PROGNOSIS;

BREAST-CANCER; UBCH10; EXPRESSION; OVEREXPRESSION; SIGNATURE;

ADENOCARCINOMA; IDENTIFICATION

AB Current prognostic factors fail to accurately determine prognosis for patients with esophageal squamous cell carcinoma (ESCC) after surgery. Here, we constructed a survival prediction model for prognostication in patients with ESCC. Candidate molecular biomarkers were extracted from the Gene Expression Omnibus (GEO), and Cox regression analysis was performed to determine significant prognostic factors. The survival prediction model was constructed based on cluster and discriminant analyses in a training cohort (N= 205), and validated in a test cohort (N= 207). The survival prediction model consisting of two genes (UBE2C and MGP) and two clinicopathological factors (tumor stage and grade) was developed. This model could be used to accurately categorize patients into three groups in the test cohort. Both disease- free survival and overall survival differed among the diverse groups (P< 0.05). In summary, we have developed and validated a predictive model that is based on two gene markers in conjunction with two clinicopathological variables, and which can accurately predict outcomes for ESCC patients after surgery.

C1 [Wang, Wei; Zhang, Yixin; Liu, Lei; Shi, Minxin] Nantong Univ, Affiliated Tumor Hosp, Dept Surg, Nantong, Jiangsu, Peoples R China.

[Wang, Zhiwei; Wei, Min; He, Qi] Shanghai Jiao Tong Univ, Int Peace Matern & Child Hlth Hosp, Dept Breast, Shanghai, Peoples R China.

[Wang, Zhiwei; Ling, Tianlong] Shanghai Jiao Tong Univ, Sch Med, Shanghai Renji Hosp, Dept Thorac Surg, Shanghai, Peoples R China.

[Zhao, Jun] Soochow Univ, Coll Med, Affiliated Hosp 1, Dept Thorac & Cardiovasc Surg, Suzhou, Jiangsu, Peoples R China.

[Zhu, Xinghua] Nantong Univ, Affiliated Tumor Hosp, Dept Pathol, Nantong, Jiangsu, Peoples R China.

[Chen, Xiaoyan] Shanghai Jiao Tong Univ, Sch Med, Shanghai Ruijin Hosp, Dept Pathol, Shanghai, Peoples R China.

RP Liu, L; Shi, MX (corresponding author), Nantong Univ, Affiliated Tumor Hosp, Dept Surg, Nantong, Jiangsu, Peoples R China.

EM liulei5602@126.com; Docshimx@126.com

FU NSFC (Natural Science Foundation of China) [81101847]; Shanghai

Committee of Science and Technology, China [124119a4801]; Nantong

Science and Technology Commission [HS149127]; Scientific Fund of

International Peace Maternity and Child Health Hospital

FX This study was supported in part by funding from NSFC (Natural Science

Foundation of China, 81101847), Shanghai Committee of Science and

Technology, China (124119a4801), Nantong Science and Technology

Commission (grant no.: HS149127), and Scientific Fund of International

Peace Maternity and Child Health Hospital (2014).

CR [Anonymous], 2010, INT UNION AGAINSTCAN

Berlingieri MT, 2007, ONCOGENE, V26, P2136, DOI 10.1038/sj.onc.1210010

Bianchi F, 2004, CLIN CANCER RES, V10, P6023, DOI 10.1158/1078-0432.CCR-04-0619

Camp RL, 2004, CLIN CANCER RES, V10, P7252, DOI 10.1158/1078-0432.CCR-04-0713

Cao HH, 2014, PLOS ONE, V9, DOI 10.1371/journal.pone.0106007

Castro C, 2014, ANN ONCOL, V25, P283, DOI 10.1093/annonc/mdt486

Chang WJ, 2014, GUT, V63, P1457, DOI 10.1136/gutjnl-2013-305475

Chen MF, 2013, ANN SURG ONCOL, V20, P3023, DOI 10.1245/s10434-013-2935-4

Chen WQ, 2015, CHINESE J CANCER RES, V27, P2, DOI 10.3978/j.issn.1000-9604.2015.01.06

Fan CW, 2001, CANCER LETT, V165, P63, DOI 10.1016/S0304-3835(01)00416-5

Fristrup N, 2013, AM J PATHOL, V182, P339, DOI 10.1016/j.ajpath.2012.10.017

Fujita T, 2009, BMC CANCER, V9, DOI 10.1186/1471-2407-9-87

Gavin AT, 2012, CANCER EPIDEMIOL, V36, P505, DOI 10.1016/j.canep.2012.07.009

Guo Lei, 2010, Chinese Journal of Pathology, V39, P436, DOI 10.3760/cma.j.issn.0529-5807.2010.07.002

Hoshida Y, 2008, NEW ENGL J MED, V359, P1995, DOI 10.1056/NEJMoa0804525

Hough CD, 2001, CANCER RES, V61, P3869

Kim SM, 2010, PLOS ONE, V5, DOI 10.1371/journal.pone.0015074

Kuzontkoski PM, 2010, ONCOGENE, V29, P3793, DOI 10.1038/onc.2010.147

LEVEDAKOU EN, 1992, INT J CANCER, V52, P534, DOI 10.1002/ijc.2910520406

Liang WH, 2015, J CLIN ONCOL, V33, P861, DOI 10.1200/JCO.2014.56.6661

Lin J, 2006, NEOPLASIA, V8, P1062, DOI 10.1593/neo.05832

Matsumoto A, 2014, ANTICANCER RES, V34, P955

MCCARTY KS, 1985, ARCH PATHOL LAB MED, V109, P716

Mertsch S, 2009, BMC CANCER, V9, DOI 10.1186/1471-2407-9-302

Micke P, 2007, J INVEST DERMATOL, V127, P1516, DOI 10.1038/sj.jid.5700714

Okamoto Y, 2003, CANCER RES, V63, P4167

Ong CAJ, 2013, J CLIN ONCOL, V31, P1576, DOI 10.1200/JCO.2012.45.9636

Paik S, 2004, NEW ENGL J MED, V351, P2817, DOI 10.1056/NEJMoa041588

Pallante P, 2013, EUR J CANCER, V49, P1117, DOI 10.1016/j.ejca.2012.09.033

Parris TZ, 2014, INT J CANCER, V134, P1617, DOI 10.1002/ijc.28497

Peng ZM, 2014, INT J CLIN EXP PATHO, V7, P5950

Schurgers LJ, 2008, THROMB HAEMOSTASIS, V100, P593, DOI 10.1160/TH08-02-0087

Summers MK, 2008, MOL CELL, V31, P544, DOI 10.1016/j.molcel.2008.07.014

Sun LL, 2015, INT J CANCER, V136, pE569, DOI 10.1002/ijc.29211

Sveen A, 2012, CLIN CANCER RES, V18, P6001, DOI 10.1158/1078-0432.CCR-11-3302

Torre LA, 2015, CA-CANCER J CLIN, V65, P87, DOI 10.3322/caac.21262

Trivers KF, 2008, INT J CANCER, V123, P1422, DOI 10.1002/ijc.23691

van Ree JH, 2010, J CELL BIOL, V188, P83, DOI 10.1083/jcb.200906147

Williamson A, 2009, P NATL ACAD SCI USA, V106, P18213, DOI 10.1073/pnas.0907887106

Yoshimura K, 2009, MOL MED REP, V2, P549, DOI 10.3892/mmr_00000135

NR 40

TC 6

Z9 6

U1 0

U2 3

PU IMPACT JOURNALS LLC

PI ORCHARD PARK

PA 6666 E QUAKER ST, STE 1, ORCHARD PARK, NY 14127 USA

EI 1949-2553

J9 ONCOTARGET

JI Oncotarget

PD SEP 27

PY 2016

VL 7

IS 39

BP 63526

EP 63536

DI 10.18632/oncotarget.11362

PG 11

WC Oncology; Cell Biology

WE Science Citation Index Expanded (SCI-EXPANDED)

SC Oncology; Cell Biology

GA EB2DN

UT WOS:000387167800055

PM 27556859

OA Green Published, gold, Green Submitted

DA 2022-05-19

ER

PT J

AU Ma, J

Zhang, JN

Ning, T

Chen, ZP

Xu, CQ

AF Ma, Juan

Zhang, Jianna

Ning, Tao

Chen, Ziping

Xu, Changqing

TI Association of genetic polymorphisms in MDM2, PTEN and P53 with risk of

esophageal squamous cell carcinoma

SO JOURNAL OF HUMAN GENETICS

LA English

DT Article

DE esophageal squamous cell carcinoma; MDM2; P53; polymorphism; PTEN

ID SINGLE NUCLEOTIDE POLYMORPHISM; CHINESE POPULATION; NASOPHARYNGEAL

CARCINOMA; LUNG-CANCER; PROMOTER; SUSCEPTIBILITY; VARIANTS; GSTP1; TP53

AB Genetic variations in MDM2, PTEN and P53 might be involved in cancer susceptibility. To assess the contribution of polymorphisms in these three genes to the risk of esophageal squamous cell carcinoma (ESCC) in a Chinese population, we genotyped MDM2 T309G, Del1518, PTEN rs701848, rs2735343 and P53 Arg72Pro polymorphisms using PCR-restriction fragment length polymorphism analysis in 226 ESCC cases and 226 cancer-free controls. Here we showed that the risk of ESCC was elevated in subjects with any of the variant genotypes of PTEN rs2735343 and P53 Arg72Pro polymorphisms, but not any genotype of MDM2 or PTEN rs701848. Moreover, multiplicative interactions were observed between PTEN rs2735343 and P53 Arg72Pro or smoking status on risk of ESCC. Our study firstly indicated that PTEN rs2735343 might be a susceptibility factor for ESCC and reaffirmed the role of P53 Arg72Pro in ESCC in this Chinese population, but did not replicate the positive association between MDM2 T309G and ESCC found previously. Journal of Human Genetics (2012) 57, 261-264; doi:10.1038/jhg.2012.15; published online 16 February 2012

C1 [Ma, Juan; Zhang, Jianna; Chen, Ziping; Xu, Changqing] Shandong Univ, Dept Gastroenterol, Qianfoshan Hosp, Jinan 250014, Shandong Provin, Peoples R China.

[Ning, Tao] Peking Univ, Beijing Canc Hosp & Inst, Key Lab Carcinogenesis & Translat Res, Minist Educ,Lab Genet,Sch Oncol, Beijing 100871, Peoples R China.

RP Xu, CQ (corresponding author), Shandong Univ, Dept Gastroenterol, Qianfoshan Hosp, 66 JingShi Rd, Jinan 250014, Shandong Provin, Peoples R China.

EM xcqys@126.com

RI wang, bing/F-5528-2011

FU Program of Shandong Province's Pharmaceutical Health and Technology

Development [HZ068]

FX This paper was supported by the Program of Shandong Province's

Pharmaceutical Health and Technology Development (No HZ068).

CR Bond GL, 2005, CANCER RES, V65, P5481, DOI 10.1158/0008-5472.CAN-05-0825

Bond GL, 2004, CELL, V119, P591, DOI 10.1016/j.cell.2004.11.022

Cai L, 2006, CANCER EPIDEM BIOMAR, V15, P294, DOI 10.1158/1055-9965.EPI-05-0680

Chang D, 2007, DIS ESOPHAGUS, V20, P491, DOI 10.1111/j.1442-2050.2007.00695.x

CHANG FJ, 1992, GASTROENTEROLOGY, V103, P1336, DOI 10.1016/0016-5085(92)91526-A

Ding J, 2011, DNA CELL BIOL, V30, P229, DOI 10.1089/dna.2010.1126

Dumont P, 2003, NAT GENET, V33, P357, DOI 10.1038/ng1093

Ge H, 2008, DIS ESOPHAGUS, V21, P409, DOI 10.1111/j.1442-2050.2007.00786.x

Haiman CA, 2006, CANCER EPIDEM BIOMAR, V15, P1021, DOI 10.1158/1055-9965.EPI-05-0896

Haupt Y, 1997, NATURE, V387, P296, DOI 10.1038/387296a0

Hiyama T, 2007, INT J CANCER, V121, P1643, DOI 10.1002/ijc.23044

Hong Y, 2005, CANCER RES, V65, P9582, DOI 10.1158/0008-5472.CAN-05-1460

Hu ZB, 2006, INT J CANCER, V118, P1275, DOI 10.1002/ijc.21463

Lee JM, 2000, INT J CANCER, V89, P458, DOI 10.1002/1097-0215(20000920)89:5<458::AID-IJC10>3.0.CO;2-R

Lindsey D. M., 2002, TRENDS BIOCHEM SCI, V27, P9

Ma HX, 2006, CANCER LETT, V240, P261, DOI 10.1016/j.canlet.2005.09.019

Mayo LD, 2002, J BIOL CHEM, V277, P5484, DOI 10.1074/jbc.M108302200

MOMAND J, 1992, CELL, V69, P1237, DOI 10.1016/0092-8674(92)90644-R

Ohmiya N, 2006, J CLIN ONCOL, V24, P4434, DOI 10.1200/JCO.2005.04.1459

Phillips LS, 2009, WORLD J GASTROENTERO, V15, P3771, DOI 10.3748/wjg.15.3771

Sholom W., 1992, AM J EPIDEMIOL, V135, P1029

Stephens M, 2001, AM J HUM GENET, V68, P978, DOI 10.1086/319501

Tachibana M, 2002, CANCER-AM CANCER SOC, V94, P1955, DOI 10.1002/cncr.0678

Treloar SA, 2007, MOL HUM REPROD, V13, P587, DOI 10.1093/molehr/gam023

Xiao M, 2010, BMC CANCER, V10, DOI 10.1186/1471-2407-10-147

Xing DY, 2003, ONCOL REP, V10, P1615

Yin Y, 2008, ONCOGENE, V27, P5443, DOI 10.1038/onc.2008.241

Zhang Lei, 2002, Zhonghua Yi Xue Yi Chuan Xue Za Zhi, V19, P10

Zhang XM, 2006, HUM MUTAT, V27, P110, DOI 10.1002/humu.20277

Zhou GQ, 2007, CLIN CANCER RES, V13, P2627, DOI 10.1158/1078-0432.CCR-06-2281

NR 30

TC 32

Z9 34

U1 0

U2 6

PU NATURE PUBLISHING GROUP

PI NEW YORK

PA 75 VARICK ST, 9TH FLR, NEW YORK, NY 10013-1917 USA

SN 1434-5161

EI 1435-232X

J9 J HUM GENET

JI J. Hum. Genet.

PD APR

PY 2012

VL 57

IS 4

BP 261

EP 264

DI 10.1038/jhg.2012.15

PG 4

WC Genetics & Heredity

WE Science Citation Index Expanded (SCI-EXPANDED)

SC Genetics & Heredity

GA 934CA

UT WOS:000303416800008

PM 22336889

OA Bronze

DA 2022-05-19

ER

PT J

AU Wang, G

Ye, MJ

Zheng, SK

Wu, KS

Geng, H

Liu, CX

AF Wang, Geng

Ye, Meijie

Zheng, Shukai

Wu, Kusheng

Geng, Hui

Liu, Caixia

TI Cigarette Smoke Extract induces H19 in Esophageal Squamous Cell

Carcinoma in Smoking Patients: Based on A Chronic Exposed Cell Model

SO TOXICOLOGY LETTERS

LA English

DT Article

DE cigarette smoking; esophageal squamous cell carcinoma (ESCC); chemical

carcinogenesis; long non-coding RNA (lncRNA)

ID EPITHELIAL-CELLS; MALIGNANT-TRANSFORMATION; UP-REGULATION; EXPRESSION;

CANCER; CARCINOGENESIS; CONTRIBUTES; GENE

AB Cigarette smoking is a factor capable of inducing esophageal squamous cell carcinoma (ESCC). However, the biological pathways that are responsible for tumor development and are directly affected by cigarette smoking remain unknown. To explore the role of cigarette smoking in ESCC, we developed a long-term cigarette smoke extract (CSE) exposed cell model using the normal immortalized SHEE esophageal epithelial cell line, which would malignantly transform after long-term cultivation without carcinogens. CSE-exposed cells displayed higher malignancy and differently expressed several lncRNAs. Among them, H19, a lncRNA responsible for proliferation and invasion, was upregulated in CSE-exposed SHEE cells. In tumors from ESCC patients, H19 was significantly increased in smoking ESCC patients compared to non-smoking patients, and H19 was overexpressed and correlated with pathological tumor size in smokers. These results indicated that cigarette smoking lead to a different biological change from non-smoking induced ESCC and H19 related to cancer development during CSE-induced carcinogenesis.

C1 [Ye, Meijie; Zheng, Shukai; Wu, Kusheng; Geng, Hui; Liu, Caixia] Shantou Univ, Dept Prevent Med, Med Coll, 22 Xinling Rd, Shantou 515041, Guangdong, Peoples R China.

[Wang, Geng] Shantou Univ, Dept Thorac Surg, Med Coll, Canc Hosp, Shantou, Guangdong, Peoples R China.

RP Liu, CX (corresponding author), Shantou Univ, Dept Prevent Med, Med Coll, 22 Xinling Rd, Shantou 515041, Guangdong, Peoples R China.

EM gengwangst@foxmail.com; 1353841204@qq.com; 386791448@qq.com;

kswu@stu.edu.cn; genghui186@163.com; liucx@stu.edu.cn

RI Wu, Kusheng/O-5141-2019

OI Wu, Kusheng/0000-0001-5163-2524; Liu, Caixia/0000-0001-5473-4315

FU Natural Science Foundation of China [81602878]; Guangdong Esophageal

Cancer Institute Science and Technology Program [Q201804]; Department of

Education of Guangdong Province, China [2015KQNCX049]

FX The work was supported the Natural Science Foundation of China (grant

numbers 81602878, 2016); Guangdong Esophageal Cancer Institute Science

and Technology Program (grant numbers, Q201804, 2015) and Department of

Education of Guangdong Province, China ((grant numbers, 2015KQNCX049,

2015). We thank Prof. LY Xu (Shantou University Medical College, China)

for providing the immortal SHEE cell line and Mr. CX Zeng (Shenzhen

Hospital of Southern Medical University, China) and Prof. CM Lin

(Shantou University Medical College, China) for the writing suggestions.

CR Bray F, 2018, CA-CANCER J CLIN, V68, P394, DOI 10.3322/caac.21492

Chen MJ, 2019, INT J BIOCHEM CELL B, V113, P27, DOI 10.1016/j.biocel.2019.05.011

Chen XJ, 2015, AM J CANCER RES, V5, P2808

Fu Y, 2018, CHEMOSPHERE, V207, P84, DOI 10.1016/j.chemosphere.2018.05.048

Hu HB, 2016, CELL PHYSIOL BIOCHEM, V40, P117, DOI 10.1159/000452529

Kaplan R, 2003, CANCER RES, V63, P1475

Kaur G, 2019, ARCH TOXICOL, V93, P2715, DOI 10.1007/s00204-019-02562-y

Ke S, 2018, AGING-US, V10, P56, DOI 10.18632/aging.101359

Lee CH, 2015, ONCOTARGET, V6, P25188, DOI 10.18632/oncotarget.4512

Li DC, 2012, TOXICOL SCI, V125, P382, DOI 10.1093/toxsci/kfr299

Li JG, 2014, GUT, V63, P1700, DOI 10.1136/gutjnl-2013-305806

Liang Y, 2018, CELL DEATH DIFFER, V25, P1980, DOI 10.1038/s41418-018-0084-9

Lin YS, 2013, J EPIDEMIOL, V23, P233, DOI 10.2188/jea.JE20120162

Liu F, 2010, ONCOGENE, V29, P3650, DOI 10.1038/onc.2010.129

Liu Y, 2016, TOXICOL LETT, V240, P60, DOI 10.1016/j.toxlet.2015.10.016

Lu L, 2015, TOXICOL APPL PHARM, V289, P276, DOI 10.1016/j.taap.2015.09.016

Mohapatra P, 2014, TOXICOL APPL PHARM, V274, P168, DOI 10.1016/j.taap.2013.09.028

Oze I, 2012, JPN J CLIN ONCOL, V42, P63, DOI 10.1093/jjco/hyr170

Radojicic J, 2012, INT J BIOL MARKER, V27, P1, DOI 10.5301/JBM.2011.8737

Shen ZY, 2004, ONCOL REP, V11, P647

Shen ZY, 2002, WORLD J GASTROENTERO, V8, P976, DOI 10.3748/wjg.v8.i6.976

Shen ZY, 2001, INT J MOL MED, V8, P633

Shen ZY, 2000, J CANCER RES CLIN, V126, P589, DOI 10.1007/PL00008469

Sobus SL, 2014, CANCER-AM CANCER SOC, V120, P3617, DOI 10.1002/cncr.28904

Toh Y, 2010, INT J CLIN ONCOL, V15, P135, DOI 10.1007/s10147-010-0057-6

Wang MH, 2019, LANCET RESP MED, V7, P35, DOI [10.1016/S2213-2600(18)30432-6, 10.1016/s2213-2600(18)30432-6]

Wang Q, 2011, ONCOGENE, V30, P3875, DOI 10.1038/onc.2011.103

Wang QL, 2017, JNCI-J NATL CANCER I, V109, DOI 10.1093/jnci/djx115

Wu I.-C., 2012, PLOS ONE, V8

WU Y, 2017, J CHINESE SOCIOLOGY, V4, P9, DOI DOI 10.1186/S40711-017-0057-2

Xie JJ, 2018, GASTROENTEROLOGY, V154, P2137, DOI 10.1053/j.gastro.2018.02.018

Yang XZ, 2018, CELL PHYSIOL BIOCHEM, V48, P251, DOI 10.1159/000491724

Zeng N, 2019, J CELL MOL MED, V23, P5532, DOI 10.1111/jcmm.14436

Zhang ED, 2017, 2017 14TH WEB INFORMATION SYSTEMS AND APPLICATIONS CONFERENCE (WISA 2017), P1, DOI 10.1109/WISA.2017.16

Zhang EB, 2017, NUCLEIC ACIDS RES, V45, P3086, DOI 10.1093/nar/gkw1247

Zhang JL, 2019, NAT COMMUN, V10, DOI 10.1038/s41467-019-09712-x

Zhao Y, 2013, TOXICOL SCI, V135, P265, DOI 10.1093/toxsci/kft150

Zheng YZ, 2015, PLOS ONE, V10, DOI 10.1371/journal.pone.0123246

Zhou YW, 2016, ONCOTARGET, V7, P30730, DOI 10.18632/oncotarget.8950

Zhuang Z., 2020, CHEM RES TOXICOL

Zou GR, 2019, EXP THER MED, V17, P3671, DOI 10.3892/etm.2019.7356

NR 41

TC 0

Z9 0

U1 3

U2 10

PU ELSEVIER IRELAND LTD

PI CLARE

PA ELSEVIER HOUSE, BROOKVALE PLAZA, EAST PARK SHANNON, CO, CLARE, 00000,

IRELAND

SN 0378-4274

EI 1879-3169

J9 TOXICOL LETT

JI Toxicol. Lett.

PD OCT 15

PY 2020

VL 333

BP 62

EP 70

DI 10.1016/j.toxlet.2020.07.030

PG 9

WC Toxicology

WE Science Citation Index Expanded (SCI-EXPANDED)

SC Toxicology

GA OD5PY

UT WOS:000579907000008

PM 32739445

DA 2022-05-19

ER

PT J

AU Deng, JY

Zhou, DZ

Zhang, JH

Chen, Y

Wang, CY

Liu, Y

Zhao, KL

AF Deng, Jiaying

Zhou, Daizhan

Zhang, Junhua

Chen, Yun

Wang, Chunyu

Liu, Yun

Zhao, Kuaile

TI Aberrant methylation of the TERT promoter in esophageal squamous cell

carcinoma

SO CANCER GENETICS

LA English

DT Article

DE Telomerase reverse transcriptase; esophageal squamous cell carcinoma;

upstream of the transcription start site (UTSS); methylation

ID TELOMERASE-REVERSE-TRANSCRIPTASE; CATALYTIC SUBUNIT; CANCER;

HYPERMETHYLATION; DNA; HYPOMETHYLATION; EPIDEMIOLOGY; EPIGENETICS;

EXPRESSION; DISEASE

AB A recent study indicated that upstream of the transcription start site (UTSS) hypermethylation of the telomerase reverse transcriptase (TERT) gene was associated with tumor progression and poor prognosis in pediatric brain tumors. The potential for methylation-mediated regulation of the UTSS region of the TERTgene in esophageal squamous cell carcinoma (ESCC) has not yet been investigated. Here, TERT methylation was investigated in tumor and paired adjacent noncancerous tissues (ANT) from 185 ESCC patients, and the expression of TERT was investigated in 26 tumors paired with ANTs selected from the same cohort. The methylation level of TERT was analyzed in three different regions: region 1, region 2, and the UTSS region. Comparison and correlation of methylation level and clinical features were analyzed in the abovementioned regions. The results showed that the methylation level of TERT was significantly elevated in the tumor relative to the ANT in ESCC. TERT RNA expression was significantly reduced in primary tumors. Tumor stage was the major determinant of survival. The UTSS region may not be an accessible biomarker for ESCC.

C1 [Deng, Jiaying; Zhang, Junhua; Chen, Yun; Wang, Chunyu; Zhao, Kuaile] Fudan Univ, Shanghai Canc Ctr, Dept Radiat Oncol, Shanghai 200433, Peoples R China.

[Deng, Jiaying; Zhao, Kuaile] Fudan Univ, Dept Oncol, Shanghai Med Coll, Shanghai 200433, Peoples R China.

[Zhou, Daizhan] Shanghai Jiao Tong Univ, BioX Ctr, Minist Educ, Key Lab Genet Dev & Neuropsychiat Disorders, Shanghai 200030, Peoples R China.

[Liu, Yun] Fudan Univ, Inst Biomed Sci, Shanghai 200433, Peoples R China.

RP Liu, Y (corresponding author), Fudan Univ, Inst Biomed Sci, Shanghai 200433, Peoples R China.

EM superliuyun@gmail.com; kuaile_z@sina.com

FU National Natural Science Foundation of China [21172043]; National

Natural Science Foundation of China (Beijing, PR China) [31200954];

China Postdoctoral Science Foundation (Beijing, PR China) [2012M510110,

2013T60440]

FX This study was financially supported by the National Natural Science

Foundation of China (grant number 21172043), the National Natural

Science Foundation of China (31200954 Beijing, PR China) and the China

Postdoctoral Science Foundation Funded Project (2012M510110, 2013T60440

Beijing, PR China).

CR Baba Y, 2010, MOL CANCER, V9, DOI 10.1186/1476-4598-9-125

Barrow TM, 2014, BIOCHEM BIOPH RES CO, V455, P70, DOI 10.1016/j.bbrc.2014.08.002

Beleford D, 2010, CLIN CANCER RES, V16, P398, DOI 10.1158/1078-0432.CCR-09-1677

Castelo-Branco P, 2013, LANCET ONCOL, V14, P534, DOI 10.1016/S1470-2045(13)70110-4

Cordaux R, 2009, NAT REV GENET, V10, P691, DOI 10.1038/nrg2640

Dai W, 2011, CLIN CANCER RES, V17, P4052, DOI 10.1158/1078-0432.CCR-10-3021

Das PM, 2004, J CLIN ONCOL, V22, P4632, DOI 10.1200/JCO.2004.07.151

Dessain SK, 2000, CANCER RES, V60, P537

Egger G, 2004, NATURE, V429, P457, DOI 10.1038/nature02625

Ferlay J, 2010, INT J CANCER, V127, P2893, DOI 10.1002/ijc.25516

Guilleret I, 2002, INT J CANCER, V101, P335, DOI 10.1002/ijc.10593

Hamilton JP, 2006, CLIN GASTROENTEROL H, V4, P701, DOI 10.1016/j.cgh.2006.03.007

Herman JG, 2003, NEW ENGL J MED, V349, P2042, DOI 10.1056/NEJMra023075

Hoque MO, 2004, CANCER RES, V64, P5511, DOI 10.1158/0008-5472.CAN-04-0799

Horikawa I, 1999, CANCER RES, V59, P826

Hu CY, 2014, CLIN CANCER RES, V20, P4349, DOI 10.1158/1078-0432.CCR-14-0494

Iwagami S, 2012, ANN SURG ONCOL, V19, P2726, DOI 10.1245/s10434-011-2176-3

Jemal A, 2011, CA-CANCER J CLIN, V61, P69, DOI [10.3322/caac.20107, 10.3322/caac.20115]

Safont MJ, 2011, CLIN TRANSL ONCOL, V13, P396, DOI 10.1007/s12094-011-0673-2

Kitkumthorn N, 2011, CLIN EPIGENETICS, V2, P315, DOI 10.1007/s13148-011-0032-8

Kumari A, 2009, ANN SURG ONCOL, V16, P1051, DOI 10.1245/s10434-009-0333-8

Lee HS, 2009, CLIN CANCER RES, V15, P812, DOI 10.1158/1078-0432.CCR-08-0266

Liu L, 2004, GENE CHROMOSOME CANC, V41, P26, DOI 10.1002/gcc.20058

Martin JA, 2002, CELL GROWTH DIFFER, V13, P397

Metzger R, 2009, ANTICANCER RES, V29, P1157

Ogino S, 2013, MODERN PATHOL, V26, P465, DOI 10.1038/modpathol.2012.214

Peters I, 2014, PLOS ONE, V9, DOI 10.1371/journal.pone.0091440

Poole JC, 2001, GENE, V269, P1, DOI 10.1016/S0378-1119(01)00440-1

Secretan B, 2009, LANCET ONCOL, V10, P1033, DOI 10.1016/S1470-2045(09)70326-2

Shigaki H, 2012, ANN SURG ONCOL, V19, P4238, DOI 10.1245/s10434-012-2488-y

Shin KH, 2003, BRIT J CANCER, V89, P1473, DOI 10.1038/sj.bjc.6601291

Suzuki H, 2014, BIOCHEM BIOPH RES CO, V455, P35, DOI 10.1016/j.bbrc.2014.07.007

Wang ZH, 2010, ARCH MED RES, V41, P1, DOI 10.1016/j.arcmed.2009.11.001

Zhang Xiao-Mei, 2010, Front Med China, V4, P378, DOI 10.1007/s11684-010-0230-3

NR 34

TC 5

Z9 5

U1 0

U2 8

PU ELSEVIER SCIENCE INC

PI NEW YORK

PA STE 800, 230 PARK AVE, NEW YORK, NY 10169 USA

SN 2210-7762

EI 2210-7770

J9 CANCER GENET-NY

JI Cancer Genet.

PD DEC

PY 2015

VL 208

IS 12

BP 602

EP 609

DI 10.1016/j.cancergen.2015.10.004

PG 8

WC Oncology; Genetics & Heredity

WE Science Citation Index Expanded (SCI-EXPANDED)

SC Oncology; Genetics & Heredity

GA CZ3UQ

UT WOS:000367029900004

PM 26669682

DA 2022-05-19

ER

PT J

AU Kwon, J

Lee, TS

Lee, HW

Kang, MC

Yoon, HJ

Kim, JH

Park, JH

AF Kwon, Junhye

Lee, Tae-Sup

Lee, Hae Won

Kang, Moon Chul

Yoon, Hyeon-Joon

Kim, Ji-Hee

Park, Jong Ho

TI Integrin alpha 6: A novel therapeutic target in esophageal squamous cell

carcinoma

SO INTERNATIONAL JOURNAL OF ONCOLOGY

LA English

DT Article

DE esophageal squamous cell carcinoma; integrin alpha 6; tumorigenesis;

biodistribution; xenograft model

ID HUMAN BREAST-CARCINOMA; CANCER; EXPRESSION; PROTEIN; KINASE;

METAANALYSIS; PROMOTES; SURVIVAL; INVASION; GROWTH

AB Esophageal squamous cell carcinoma (ESCC), the most common subtype of esophageal cancer in East Asian countries, is a devastating disease characterized by distinctly high incidence and mortality rates. Our previous expression profile analysis showed that integrin alpha 6 (ITGA6) is highly expressed in ESCC tissues. To validate cell surface expression of ITGA6 as a novel target in ESCC, we investigated ITGA6 expression in tumor tissue samples and cell lines of ESCC and found that ITGA6 was upregulated in these cells. In vitro knockdown of ITGA6 in ESCC cells resulted in inhibition of cell proliferation, invasion and colony formation. In addition, we demonstrated that ITGA6 associates with integrin beta 4 (ITGB4), and that this heterodimer complex is upregulated in both ESCC tissues and cell lines. Moreover, our biodistribution results in an ESCC xenograft model indicated that ITGA6 is a possible target for antibody-related diagnostic and therapeutic modalities in ESCC. Thus, our findings suggest that ITGA6 plays an important role in tumorigenesis in ESCC and represents a potential therapeutic target in the treatment of ESCC.

C1 [Kwon, Junhye; Yoon, Hyeon-Joon; Kim, Ji-Hee] Korea Inst Radiol & Med Sci, Korea Canc Ctr Hosp, Dept Translat Res, Seoul 139706, South Korea.

[Kwon, Junhye] Sookmyung Womens Univ, Res Inst Womens Hlth, Seoul, South Korea.

[Lee, Tae-Sup] Korea Inst Radiol & Med Sci, Mol Imaging Res Ctr, Seoul 139706, South Korea.

[Lee, Hae Won; Kang, Moon Chul; Park, Jong Ho] Korea Inst Radiol & Med Sci, Korea Canc Ctr Hosp, Dept Thorac Surg, Seoul 139706, South Korea.

RP Park, JH (corresponding author), Korea Inst Radiol & Med Sci, Korea Canc Ctr Hosp, Dept Thorac Surg, Seoul 139706, South Korea.

EM jhpark@kcch.re.kr

RI LEE, TAE SUP/K-9638-2019

OI Lee, Tae Sup/0000-0002-8266-7380

FU National R&D Program for Cancer Control [1120260]; Ministry of Health

and Welfare, Republic of Korea; Radiological Translational Research

Program (RTR), Korea Institute of Radiological & Medical Sciences

[50455-2012]

FX This work was supported by grants from the National R&D Program for

Cancer Control (1120260 to J.H.P.), Ministry of Health and Welfare,

Republic of Korea, and from the Radiological Translational Research

Program (RTR), Korea Institute of Radiological & Medical Sciences

(50455-2012).

CR Avraamides CJ, 2008, NAT REV CANCER, V8, P604, DOI 10.1038/nrc2353

Beaulieu Jean-Francois, 2010, World J Gastrointest Pathophysiol, V1, P3, DOI 10.4291/wjgp.v1.i1.3

Bon G, 2007, BREAST CANCER RES, V9, DOI 10.1186/bcr1651

Capdevila J, 2009, CANCER TREAT REV, V35, P354, DOI 10.1016/j.ctrv.2009.02.001

Cariati M, 2008, INT J CANCER, V122, P298, DOI 10.1002/ijc.23103

Chiu PWY, 2005, J GASTROINTEST SURG, V9, P794, DOI 10.1016/j.gassur.2005.05.005

Chung J, 2004, MOL CELLS, V17, P203

Cruz-Monserrate Z, 2008, NEOPLASIA, V10, P408, DOI 10.1593/neo.07868

Darling G, 2009, J SURG ONCOL, V99, P189, DOI 10.1002/jso.21209

FRIEDRICHS K, 1995, CANCER RES, V55, P901

Giancotti FG, 2003, ANNU REV CELL DEV BI, V19, P173, DOI 10.1146/annurev.cellbio.19.031103.133334

HEMLER ME, 1989, J BIOL CHEM, V264, P6529

Hu YC, 2001, CLIN CANCER RES, V7, P3519

Hwang R, 2004, HEMATOL ONCOL CLIN N, V18, P991, DOI 10.1016/j.hoc.2004.09.010

HYNES RO, 1992, CELL, V69, P11, DOI 10.1016/0092-8674(92)90115-S

Jemal A, 2011, CA-CANCER J CLIN, V61, P69, DOI [10.3322/caac.20107, 10.3322/caac.20115]

Kranzfelder M, 2011, BRIT J SURG, V98, P768, DOI 10.1002/bjs.7455

Kwon YJ, 2009, ONCOL RES, V18, P141, DOI 10.3727/096504009790217407

Lathia JD, 2010, CELL STEM CELL, V6, P421, DOI 10.1016/j.stem.2010.02.018

Lu XJ, 2008, PERSPECT MED CHEM, V2, P57

Mercurio AM, 2001, SEMIN CANCER BIOL, V11, P129, DOI 10.1006/scbi.2000.0364

Mizejewski GJ, 1999, P SOC EXP BIOL MED, V222, P124, DOI 10.1046/j.1525-1373.1999.d01-122.x

Mukhopadhyay R, 1999, CLIN EXP METASTAS, V17, P325

Noh TW, 2010, ANTICANCER RES, V30, P4485

Su JM, 2002, WORLD J GASTROENTERO, V8, P613, DOI 10.3748/wjg.v8.i4.613

Tew WP, 2005, ONCOLOGIST, V10, P590, DOI 10.1634/theoncologist.10-8-590

Vanhoefer U, 2004, J CLIN ONCOL, V22, P175, DOI 10.1200/JCO.2004.05.114

VARNER JA, 1995, MOL BIOL CELL, V6, P725, DOI 10.1091/mbc.6.6.725

Wewer UM, 1997, AM J PATHOL, V151, P1191

Xu XH, 2012, ASIAN PAC J CANCER P, V13, P103, DOI 10.7314/APJCP.2012.13.1.103

Yang XWH, 2008, CANCER RES, V68, P3204, DOI 10.1158/0008-5472.CAN-07-2949

NR 31

TC 28

Z9 31

U1 0

U2 7

PU SPANDIDOS PUBL LTD

PI ATHENS

PA POB 18179, ATHENS, 116 10, GREECE

SN 1019-6439

EI 1791-2423

J9 INT J ONCOL

JI Int. J. Oncol.

PD NOV

PY 2013

VL 43

IS 5

BP 1523

EP 1530

DI 10.3892/ijo.2013.2097

PG 8

WC Oncology

WE Science Citation Index Expanded (SCI-EXPANDED)

SC Oncology

GA 225SN

UT WOS:000324982700023

PM 24042193

OA Green Submitted, hybrid

DA 2022-05-19

ER

PT J

AU Yang, XY

Wang, L

Du, H

Lin, BW

Yi, J

Wen, XM

Geng, LD

Du, XB

AF Yang, Xiyue

Wang, Lei

Du, Huan

Lin, Binwei

Yi, Jie

Wen, Xuemei

Geng, Lidan

Du, Xiaobo

TI Prognostic impact of eosinophils in peripheral blood and tumor site in

patients with esophageal squamous cell carcinoma treated with concurrent

chemoradiotherapy

SO MEDICINE

LA English

DT Article

DE concurrent chemoradiotherapy; eosinophil; esophageal squamous cell

carcinoma; peripheral blood; prognosis; tumor site

ID TISSUE EOSINOPHILIA; CANCER; INFILTRATION; CHEMOTHERAPY; SURVIVAL; TRIAL

AB To date, no effective biological markers have been identified for predicting the prognosis of esophageal cancer patients. Recent studies have shown that eosinophils are independent prognostic factors in some cancers. This study aimed to identify the prognostic impact of eosinophils in esophageal squamous cell carcinoma patients treated with concurrent chemoradiotherapy (CCRT). This study enrolled 136 patients who received CCRT for locally advanced unresectable esophageal squamous cell carcinoma (ESCC). We evaluated the survival time and clinical pathological characteristics of eosinophils. The Kaplan-Meier method was used to estimate survival data. The log-rank test was used for univariate analysis and the Cox proportional hazards regression model was used to conduct a multivariate analysis. Kaplan-Meier analysis revealed that high eosinophil infiltration correlated with better overall survival (OS) (P = .008) and better progression-free survival (PFS) (P = .015). The increase in absolute eosinophil count after CCRT also enhanced OS (P = .005) and PFS (P = .007). The PFS and OS in patients with high blood eosinophil count before CCRT (>2%) was better than those with low blood eosinophil count(<2%) (P = .006 and P = .001, respectively). Additionally, the multivariate analysis revealed that disease stage and high eosinophil infiltration, increased peripheral blood absolute eosinophil count after CCRT, and high peripheral blood eosinophil count before CCRT were independent prognostic indicators. High eosinophil count of tumor site, increased peripheral blood absolute eosinophil count after CCRT, and high peripheral blood eosinophil count before CCRT are favorable prognostic factors for patients with ESCC treated with CCRT.

C1 [Yang, Xiyue; Du, Huan; Lin, Binwei; Wen, Xuemei; Geng, Lidan; Du, Xiaobo] Mianyang Cent Hosp, Dept Oncol, Mianyang 621000, Sichuan, Peoples R China.

[Yang, Xiyue; Du, Huan] North Sichuan Med Coll, Affiliated Hosp, Dept Oncol, Nanchong, Peoples R China.

[Wang, Lei; Yi, Jie] Mianyang Cent Hosp, Pathol Dept, Mianyang, Sichuan, Peoples R China.

RP Du, XB (corresponding author), Mianyang Cent Hosp, Dept Oncol, Mianyang 621000, Sichuan, Peoples R China.

EM duxiaobo2005@126.com

FU Mianyang Science and Technology Bureau [15-S01-3]

FX This work was supported by the Mianyang Science and Technology Bureau

(15-S01-3).

CR CAINENG C, 2015, HEAD NECK-J SCI SPEC, V37, P151, DOI DOI 10.1002/hed.23572

Carretero R, 2015, NAT IMMUNOL, V16, P609, DOI 10.1038/ni.3159

Cooper JS, 1999, JAMA-J AM MED ASSOC, V281, P1623, DOI 10.1001/jama.281.17.1623

Cuschieri A, 2002, BRIT J CANCER, V86, P674, DOI 10.1038/sj.bjc.6600161

Dorta RG, 2002, HISTOPATHOLOGY, V41, P152, DOI 10.1046/j.1365-2559.2002.01437.x

Enzinger PC, 2003, NEW ENGL J MED, V349, P2241, DOI 10.1056/NEJMra035010

Ferlay J, 2015, INT J CANCER, V136, pE359, DOI 10.1002/ijc.29210

Fernandez-Acenero MJ, 2000, CANCER, V88, P1544, DOI 10.1002/(SICI)1097-0142(20000401)88:7<1544::AID-CNCR7>3.0.CO;2-S

Fujii M, 2002, AURIS NASUS LARYNX, V29, P175, DOI 10.1016/S0385-8146(01)00135-3

Harbaum L, 2015, MODERN PATHOL, V28, P403, DOI 10.1038/modpathol.2014.104

HERSKOVIC A, 1992, NEW ENGL J MED, V326, P1593, DOI 10.1056/NEJM199206113262403

Hu GM, 2020, BMC CANCER, V20, DOI 10.1186/s12885-020-06966-3

Ishibashi S, 2006, ANTICANCER RES, V26, P1419

IWASAKI K, 1986, CANCER, V58, P1321, DOI 10.1002/1097-0142(19860915)58:6<1321::AID-CNCR2820580623>3.0.CO;2-O

Jain Megha, 2014, Patholog Res Int, V2014, P507512, DOI 10.1155/2014/507512

Jemal A, 2011, CA-CANCER J CLIN, V61, P69, DOI [10.3322/caac.20107, 10.3322/caac.20115]

Jemal A, 2010, CA-CANCER J CLIN, V60, P277, DOI 10.3322/caac.20073

LAURA SA, 2018, ACTA MEDICA PORT, V31, P329, DOI DOI 10.20344/amp.10112

Liu YN, 2014, WORLD J SURG ONCOL, V12, DOI 10.1186/1477-7819-12-43

Lucarini V, 2017, ONCOIMMUNOLOGY, V6, DOI 10.1080/2162402X.2017.1317420

Mattes J, 2003, J EXP MED, V197, P387, DOI 10.1084/jem.20021683

Minsky BD, 2002, J CLIN ONCOL, V20, P1167, DOI 10.1200/JCO.20.5.1167

Moreira A, 2017, IMMUNOTHERAPY-UK, V9, P115, DOI 10.2217/imt-2016-0138

Nielsen HJ, 1999, J PATHOL, V189, P487, DOI 10.1002/(SICI)1096-9896(199912)189:4<487::AID-PATH484>3.0.CO;2-I

Ohashi Y, 2000, ANTICANCER RES, V20, P3025

Onesti Concetta Elisa, 2018, Oncotarget, V9, P33719, DOI 10.18632/oncotarget.26120

PRETLOW TP, 1983, CANCER RES, V43, P2997

Prizment AE, 2016, MODERN PATHOL, V29, P516, DOI 10.1038/modpathol.2016.42

Stahl M, 2005, J CLIN ONCOL, V23, P2310, DOI 10.1200/JCO.2005.00.034

Tajima K, 1998, HUM PATHOL, V29, P1024, DOI 10.1016/S0046-8177(98)90212-X

THOMPSON AC, 1994, AM J SURG, V168, P469, DOI 10.1016/S0002-9610(05)80102-3

Varricchi G, 2018, ONCOIMMUNOLOGY, V7, DOI 10.1080/2162402X.2017.1393134

Wu YF, 2019, BIOMED RES INT, V2019, DOI 10.1155/2019/1263050

YULING Z, 2014, INT J MOL SCI, V15, P9718, DOI DOI 10.3390/ijms15069718

Zhu LL, 2015, PLOS ONE, V10, DOI 10.1371/journal.pone.0128616

NR 35

TC 2

Z9 2

U1 2

U2 4

PU LIPPINCOTT WILLIAMS & WILKINS

PI PHILADELPHIA

PA TWO COMMERCE SQ, 2001 MARKET ST, PHILADELPHIA, PA 19103 USA

SN 0025-7974

EI 1536-5964

J9 MEDICINE

JI Medicine (Baltimore)

PD JAN 22

PY 2021

VL 100

IS 3

AR e24328

DI 10.1097/MD.0000000000024328

PG 7

WC Medicine, General & Internal

WE Science Citation Index Expanded (SCI-EXPANDED)

SC General & Internal Medicine

GA PZ6FV

UT WOS:000612835100144

PM 33546064

OA gold, Green Published

DA 2022-05-19

ER

PT J

AU Hu, LJ

Kong, Z

Meng, QH

Wang, JL

Zhou, MY

Yu, JP

Jiang, XD

AF Hu, Lijun

Kong, Ze

Meng, Qinghong

Wang, Jianlin

Zhou, Mengyun

Yu, Jingping

Jiang, Xiaodong

TI The Safety and Efficacy of Apatinib Treatment in Addition to Concurrent

Chemoradiotherapy in Patients with Nonoperative Locally Advanced

Esophageal Squamous Cell Carcinoma

SO MEDICAL SCIENCE MONITOR

LA English

DT Article

DE Chemoradiotherapy; Drug-Related Side Effects and Adverse Reactions;

Esophageal Neoplasms

ID PHASE-III TRIAL; CANCER

AB Background: Esophageal cancer is a common gastrointestinal malignancy in China. We evaluated the efficacy and safety of adding Apatinib to concurrent chemoradiotherapy in patients with locally advanced esophageal squamous cell carcinoma.

Material/Methods: In this single-center retrospective study, we compared short-term efficacy, long-term efficacy, and adverse events between patients who received Apatinib and concurrent chemoradiotherapy (Apatinib group), and those who received only concurrent chemoradiotherapy (CCRT group).

Results: Sixty-five patients with stage II and III esophageal squamous cell carcinoma were enrolled (31 in the Apatinib group, 34 in the CCRT group). After treatment, the therapy response rate (the sum of the complete and partial remission rates) was significantly higher in the Apatinib group than in the CCRT group (P=0.045); the complete remission rate was particularly higher in the Apatinib group. Median progression-free survival in the Apatinib group (12 months) was higher than that of the CCRT group (7 months), and the 1- and 2-year progression-free survival rates were significantly higher in the Apatinib group than in the CCRT group (47.0% vs. 30.3% and 20.2% vs. 12.1%, respectively; P=0.040). The main adverse effects of Apatinib treatment were elevated blood pressure, proteinuria, hand-foot syndrome, fatigue, and oral mucositis, all of which were level 1-2. Cox multivariate regression analysis indicated T stage and short-term efficacy were independent prognostic factors for overall and progression-free survival.

Conclusions: For patients with locally advanced esophageal squamous cell carcinoma, combining Apatinib with concurrent chemoradiotherapy can improve patient survival and significantly prolong progression-free survival, with tolerable adverse reactions.

C1 [Hu, Lijun; Kong, Ze; Meng, Qinghong; Wang, Jianlin; Zhou, Mengyun; Yu, Jingping] Nanjing Med Univ, Affiliated Changzhou Peoples Hosp 2, Dept Radiat Oncol, Changzhou, Jiangsu, Peoples R China.

[Jiang, Xiaodong] Nanjing Med Univ, Affiliated Lianyungang Clin Med Coll, Dept Radiat Oncol, Lianyungang, Jiangsu, Peoples R China.

RP Yu, JP (corresponding author), Nanjing Med Univ, Affiliated Changzhou Peoples Hosp 2, Dept Radiat Oncol, Changzhou, Jiangsu, Peoples R China.; Jiang, XD (corresponding author), Nanjing Med Univ, Affiliated Lianyungang Clin Med Coll, Dept Radiat Oncol, Lianyungang, Jiangsu, Peoples R China.

EM yujingping700420@sina.com; jxdysy1970@163.com

FU Jiangsu Province "333 Project" Second Level Talent Scientific Research

Funding Project [BRA2019025]; Changzhou High Level Health Talent Project

[2016C2BJ007]; Key Project of Science and Technology Development Fund of

Nanjing Medical University [2017NJMUZD039]

FX This study was supported by the Jiangsu Province "333 Project" Second

Level Talent Scientific Research Funding Project (No. BRA2019025),

Changzhou High Level Health Talent Project (No. 2016C2BJ007), and Key

Project of Science and Technology Development Fund of Nanjing Medical

University (No. 2017NJMUZD039)

CR Cooper JS, 1999, JAMA-J AM MED ASSOC, V281, P1623, DOI 10.1001/jama.281.17.1623

Feng Y, 2019, CLIN THER, V41, P2263, DOI 10.1016/j.clinthera.2019.08.022

Hu LJ, 2019, ONCOL LETT, V17, P1609, DOI 10.3892/ol.2018.9803

Ishida K, 2004, JPN J CLIN ONCOL, V34, P615, DOI 10.1093/jjco/hyh107

Li J, 2016, J CLIN ONCOL, V34, P1448, DOI 10.1200/JCO.2015.63.5995

Liao JB, 2019, J EXP CLIN CANC RES, V38, DOI 10.1186/s13046-019-1419-1

Manzo A, 2017, INT J MOL SCI, V18, DOI 10.3390/ijms18102021

Minsky BD, 2002, J CLIN ONCOL, V20, P1167, DOI 10.1200/JCO.20.5.1167

[孙可欣 Sun Kexin], 2019, [中国肿瘤, Bulletin of Chinese Cancer], V28, P1

Sun NN, 2018, DIAGN INTERV RADIOL, V24, P195, DOI 10.5152/dir.2018.17369

Tang J, 2019, ONCOL RES, V27, P635, DOI 10.3727/096504018X15288447760357

Teleanu RI, 2020, J CLIN MED, V9, DOI 10.3390/jcm9010084

Wang J, 2016, Zhonghua Zhong Liu Za Zhi, V38, P589, DOI 10.3760/cma.j.issn.0253-3766.2016.08.005

[王鑫 Wang Xin], 2018, [中华放射肿瘤学杂志, Chinese Journal of Radiation Oncology], V27, P959

Welsh J, 2012, CANCER-AM CANCER SOC, V118, P2632, DOI 10.1002/cncr.26586

Yang AL, 2020, J GROUNDW SCI ENG, V8, P1, DOI 10.19637/j.cnki.2305-7068.2020.01.001

Yanwei L, 2020, ONCOLOGIST

NR 17

TC 3

Z9 3

U1 1

U2 2

PU INT SCIENTIFIC INFORMATION, INC

PI MELVILLE

PA 150 BROADHOLLOW RD, STE 114, MELVILLE, NY 11747 USA

SN 1643-3750

J9 MED SCI MONITOR

JI Med. Sci. Monitor

PD NOV 27

PY 2020

VL 26

AR e927221

DI 10.12659/MSM.927221

PG 8

WC Medicine, Research & Experimental

WE Science Citation Index Expanded (SCI-EXPANDED)

SC Research & Experimental Medicine

GA OX6UW

UT WOS:000593698100001

PM 33243967

OA Green Published

DA 2022-05-19

ER

PT J

AU Wang, J

Qiu, MT

Xu, YT

Li, M

Dong, GC

Mao, QX

Yin, R

Xu, L

AF Wang, Jie

Qiu, Mantang

Xu, Youtao

Li, Ming

Dong, Gaochao

Mao, Qixing

Yin, Rong

Xu, Lin

TI Long noncoding RNA CCAT2 correlates with smoking in esophageal squamous

cell carcinoma

SO TUMOR BIOLOGY

LA English

DT Article

DE CCAT2; Long noncoding RNA; Esophageal squamous cell carcinoma;

Expression

ID COLORECTAL-CANCER; HEPATOCELLULAR-CARCINOMA; POLYMORPHISM RS6983267;

EPIGENETIC REGULATION; GASTRIC-CANCER; EXPRESSION; GENE; 8Q24;

ASSOCIATION; VARIANTS

AB Esophageal cancer is one of the leading causes of cancer-related mortality, and most esophageal squamous cell carcinoma (ESCC) cases are located in Asian area. Recent studies about long noncoding RNAs (lncRNA) have offered a new perspective for cancer research and provided new approaches to understand the complex regulation network in cancer. Our group has reported that the novel lncRNA colon cancer-associated transcript 2 (CCAT2) has important biological function and could be a potential biomarkers in lung cancer. Here, we performed in silico analysis and characterized the expression profile of CCAT2 in a cohort of esophageal squamous cell carcinoma (ESCC) patients and cell lines. In silico analysis showed that no CpG island is found in the chromosome region of CCAT2, indicating that the expression of CCAT2 is possibly not regulated by DNA methylation. Compared with paired adjacent normal esophageal tissues, CCAT2 was significantly overexpressed in ESCC tissues with an average fold of 7.18. In ESCC cell lines, CCAT2 was mostly upregulated in KYSE410 cell (24.7-fold upregulation) when normalized to normal esophageal epithelium cell line (HEEC) and most CCAT2 transcripts were located in nucleus (>95 %). Statistical analysis showed that CCAT2 expression level was significantly associated with smoking status (P=0.036). Receiver operative curve and the area under curve were calculated to assess the diagnostic potential of CCAT2. Measured by area under curve (AUC), CCAT2 showed higher diagnostic performance than conventional serum biomarkers, like AFP, CA153, and NSE. In this study, we firstly characterized the expression profile of CCAT2 in ESCC and evaluated its potential diagnostic value as a biomarker.

C1 [Wang, Jie; Qiu, Mantang; Xu, Youtao; Li, Ming; Dong, Gaochao; Mao, Qixing; Yin, Rong; Xu, Lin] Nanjing Med Univ, Affiliated Canc Hosp, Canc Inst Jiangsu Prov, Dept Thorac Surg,Jiangsu Key Lab Mol & Translat C, Nanjing 210009, Jiangsu, Peoples R China.

[Wang, Jie; Dong, Gaochao] Nanjing Med Univ, Affiliated Canc Hosp, Canc Inst Jiangsu Prov, Dept Sci Res, Nanjing 210009, Jiangsu, Peoples R China.

[Qiu, Mantang; Mao, Qixing] Nanjing Med Univ, Clin Coll 4, Nanjing 210000, Jiangsu, Peoples R China.

[Xu, Youtao] Nanjing Med Univ, Clin Coll 1, Nanjing 210000, Jiangsu, Peoples R China.

RP Yin, R (corresponding author), Nanjing Med Univ, Affiliated Canc Hosp, Canc Inst Jiangsu Prov, Dept Thorac Surg,Jiangsu Key Lab Mol & Translat C, Baiziting 42, Nanjing 210009, Jiangsu, Peoples R China.

EM yinhero001@126.com; xulin83cn@gmail.com

RI Qiu, Mantang/L-8937-2019

FU Natural Science Foundation of China [81372321, 81201830, 81472200];

Natural Science Foundation for High Education of Jiangsu Province

[13KJB320010]; Jiangsu Provincial Special Program of Medical Science

[BL2012030]

FX We thank Professor Xinchen Sun (Department of Radiology, the First

Affiliated Hospital of Nanjing Medical University) for gifting ESCC

cells. This study is funded by the Natural Science Foundation of China

(81372321 to Lin Xu; 81201830 and 81472200 to Rong Yin), Natural Science

Foundation for High Education of Jiangsu Province (13KJB320010 to Rong

Yin), and Jiangsu Provincial Special Program of Medical Science

(BL2012030 to Lin Xu).

CR Enzinger PC, 2003, NEW ENGL J MED, V349, P2241, DOI 10.1056/NEJMra035010

Guo Y, 2011, WORLD J GASTROENTERO, V17, P1759, DOI 10.3748/wjg.v17.i13.1759

Gupta RA, 2010, NATURE, V464, P1071, DOI 10.1038/nature08975

Gutschner T, 2013, CANCER RES, V73, P1180, DOI 10.1158/0008-5472.CAN-12-2850

Jemal A, 2011, CA-CANCER J CLIN, V61, P69, DOI [10.3322/caac.20107, 10.3322/caac.20115]

Jendrzejewski J, 2012, P NATL ACAD SCI USA, V109, P8646, DOI 10.1073/pnas.1205654109

Kim T, 2014, P NATL ACAD SCI USA, V111, P4173, DOI 10.1073/pnas.1400350111

Lee JT, 2012, SCIENCE, V338, P1435, DOI 10.1126/science.1231776

Ling H, 2013, GENOME RES, V23, P1446, DOI 10.1101/gr.152942.112

Nan H, 2013, JNCI-J NATL CANCER I, V105, P1852, DOI 10.1093/jnci/djt331

Panzitt K, 2007, GASTROENTEROLOGY, V132, P330, DOI 10.1053/j.gastro.2006.08.026

Park SL, 2008, CANCER EPIDEM BIOMAR, V17, P3193, DOI 10.1158/1055-9965.EPI-08-0523

Pohl H, 2005, J NATL CANCER I, V97, P142, DOI 10.1093/jnci/dji024

Ponting CP, 2009, CELL, V136, P629, DOI 10.1016/j.cell.2009.02.006

Qiu MT, 2013, TUMOR BIOL, V34, P613, DOI 10.1007/s13277-013-0658-6

Real LM, 2014, PLOS ONE, V9, DOI 10.1371/journal.pone.0101178

Redis RS, 2013, ONCOTARGET, V4, P1748, DOI 10.18632/oncotarget.1292

Rinn JL, 2007, CELL, V129, P1311, DOI 10.1016/j.cell.2007.05.022

Salmena L, 2011, CELL, V146, P353, DOI 10.1016/j.cell.2011.07.014

Smits BMG, 2013, PLOS GENET, V9, DOI 10.1371/journal.pgen.1003549

Sur I, 2013, CANCER RES, V73, P4180, DOI 10.1158/0008-5472.CAN-13-0789

Tripathi V, 2010, MOL CELL, V39, P925, DOI 10.1016/j.molcel.2010.08.011

Tsai MC, 2011, CANCER RES, V71, P3, DOI 10.1158/0008-5472.CAN-10-2483

Tsai MC, 2010, SCIENCE, V329, P689, DOI 10.1126/science.1192002

Xiang JF, 2014, CELL RES, V24, P513, DOI 10.1038/cr.2014.35

Xing Z, 2014, CELL, V159, P1110, DOI 10.1016/j.cell.2014.10.013

Yang F, 2011, HEPATOLOGY, V54, P1679, DOI 10.1002/hep.24563

Yang Z, 2011, ANN SURG ONCOL, V18, P1243, DOI 10.1245/s10434-011-1581-y

Zaratiegui M, 2007, CELL, V128, P763, DOI 10.1016/j.cell.2007.02.016

Zhang XJ, 2014, CARCINOGENESIS, V35, P2062, DOI 10.1093/carcin/bgu103

Zhou CP, 2014, GENET MOL RES, V13, P3673, DOI 10.4238/2014.May.9.10

NR 31

TC 65

Z9 71

U1 2

U2 25

PU SAGE PUBLICATIONS LTD

PI LONDON

PA 1 OLIVERS YARD, 55 CITY ROAD, LONDON EC1Y 1SP, ENGLAND

SN 1010-4283

EI 1423-0380

J9 TUMOR BIOL

JI Tumor Biol.

PD JUL

PY 2015

VL 36

IS 7

BP 5523

EP 5528

DI 10.1007/s13277-015-3220-x

PG 6

WC Oncology

WE Science Citation Index Expanded (SCI-EXPANDED)

SC Oncology

GA CP0MI

UT WOS:000359569000079

PM 25677908

DA 2022-05-19

ER

PT J

AU Tang, H

Tan, LJ

Shen, YX

Wang, H

Lin, M

Feng, MX

Xu, ST

Guo, WG

Qian, C

Liu, TS

Zeng, ZC

Hou, YY

Yu, ZT

Jiang, HJ

Li, ZG

Chen, C

Lian, CH

Du, M

Li, HC

Xie, DY

Yin, J

Zhao, NQ

Wang, Q

AF Tang, Han

Tan, Lijie

Shen, Yaxing

Wang, Hao

Lin, Miao

Feng, Mingxiang

Xu, Songtao

Guo, Weigang

Qian, Cheng

Liu, Tianshu

Zeng, Zhaochong

Hou, Yingyong

Yu, Zhentao

Jiang, Hongjing

Li, Zhigang

Chen, Chun

Lian, Changhong

Du, Ming

Li, Hecheng

Xie, Deyao

Yin, Jun

Zhao, Naiqing

Wang, Qun

TI CMISG1701: a multicenter prospective randomized phase III clinical trial

comparing neoadjuvant chemoradiotherapy to neoadjuvant chemotherapy

followed by minimally invasive esophagectomy in patients with locally

advanced resectable esophageal squamous cell carcinoma

(cT(3-4a)N(0-1)M(0)) (NCT03001596)

SO BMC CANCER

LA English

DT Article

DE Esophageal esophageal squamous carcinoma; Neoadjuvant chemoradiation;

Neoadjuvant chemotherapy; Minimally invasive esophagectomy

ID CANCER; OUTCOMES; SURGERY; SURVIVAL

AB Background: Neoadjuvant chemoradiation is not recommended as an approach for treatment of esophageal squamous cell carcinoma due to its significant postoperative mortality. However, it is assumed the combination of neoadjuvant chemoradiation with minimally invasive esophagectomy (MIE) may reduce postoperative mortality, which can revive preoperative chemoradiation. No randomized controlled studies comparing neoadjuvant chemoradiation plus MIE with neoadjuvant chemotherapy plus MIE have been performed so far. The present trial is initiated to obtain valid information whether neoadjuvant chemoradiation plus MIE yields better survival without worse postoperative morbidity and mortality in the treatment of locally advanced resectable esophageal squamous cell carcinoma(cT(3-4a)N(0-1)M(0)).

Methods/design: CMISG1701 is a multicenter, prospective, randomized, phase III clinical trial, investigating the safety and efficacy of neoadjuvant chemoradiation plus MIE compared with neoadjuvant chemotherapy plus MIE. Patients with locally advanced resectable esophageal squamous cell carcinoma (cT(3-4a)N(0-1)M(0)) are eligible for the study. A total of 264 patients are randomly assigned to neoadjuvant chemoradiation (arm A) or neoadjuvant chemotherapy (arm B) with a 1:1 allocation ratio. The primary outcome is overall survival assessed with a minimum follow-up of 36 months. Secondary outcomes are progression-free survival, recurrence-free survival, postoperative pathologic stage, treatment-related complications, postoperative mortality as well as quality of life.

Discussion: The objective of this trial is to identify the superior protocol with regard to patient survival, treatment morbidity/mortality and quality of life between neoadjuvant chemoradiation plus MIE and neoadjuvant chemotherapy plus MIE.

C1 [Tang, Han; Tan, Lijie; Shen, Yaxing; Wang, Hao; Lin, Miao; Feng, Mingxiang; Xu, Songtao; Guo, Weigang; Qian, Cheng; Wang, Qun] Fudan Univ, Zhongshan Hosp, Dept Thorac Surg, 180 Fenglin Rd, Shanghai 200032, Peoples R China.

[Liu, Tianshu] Fudan Univ, Zhongshan Hosp, Dept Med Oncol, Shanghai 200032, Peoples R China.

[Zeng, Zhaochong] Fudan Univ, Zhongshan Hosp, Dept Radiotherapy, Shanghai 200032, Peoples R China.

[Hou, Yingyong] Fudan Univ, Zhongshan Hosp, Dept Pathol, Shanghai 200032, Peoples R China.

[Yu, Zhentao; Jiang, Hongjing] Tianjin Med Univ, Canc Inst & Hosp, Key Lab Canc Prevent & Therapy Tianjin City, Dept Esophageal Canc, Tianjin 300060, Peoples R China.

[Li, Zhigang] Shanghai Jiao Tong Univ, Shanghai Chest Hosp, Dept Thorac Surg, Shanghai 200032, Peoples R China.

[Chen, Chun] Fujian Med Univ, Fujian Union Hosp, Dept Thorac Surg, Fuzhou 350001, Fujian, Peoples R China.

[Lian, Changhong] Changzhi Med Coll, Heping Hosp, Dept Gen Surg, Changzhi 046000, Shanxi, Peoples R China.

[Du, Ming] Chongqing Med Univ, Affiliated Hosp 1, Dept Cardiothorac Surg, Chongqing 400016, Peoples R China.

[Li, Hecheng] Shanghai Jiao Tong Univ, Sch Med, Ruijin Hosp, Dept Thorac Surg, Shanghai 200025, Peoples R China.

[Xie, Deyao] Wenzhou Med Univ, Affiliated Hosp 1, Dept Cardiothorac Surg, Wenzhou 325035, Zhejiang, Peoples R China.

[Yin, Jun] Jiangsu Univ, Affiliated Peoples Hosp, Dept Cardiothorac Surg, Zhenjiang 212002, Jiangsu, Peoples R China.

[Zhao, Naiqing] Fudan Univ, Sch Publ Hlth, Dept Biostat, Shanghai 200032, Peoples R China.

RP Tan, LJ (corresponding author), Fudan Univ, Zhongshan Hosp, Dept Thorac Surg, 180 Fenglin Rd, Shanghai 200032, Peoples R China.

EM tan.lijie@zs-hospital.sh.cn

FU Foundation of Science and Technology Commission of Shanghai Municipality

[16411965900]; Clinical Research Foundation of Zhongshan Hospital

[2016ZSLC15]

FX The study is supported by Foundation of Science and Technology

Commission of Shanghai Municipality (16411965900) and Clinical Research

Foundation of Zhongshan Hospital (2016ZSLC15).

CR Ando N, 2012, ANN SURG ONCOL, V19, P68, DOI 10.1245/s10434-011-2049-9

Baba Y, 2014, WORLD J GASTRO ONCOL, V6, P121, DOI 10.4251/wjgo.v6.i5.121

Bakhos C, 2014, J LAPAROENDOSC ADV S, V24, P688, DOI 10.1089/lap.2014.0118

Becker K, 2003, CANCER-AM CANCER SOC, V98, P1521, DOI 10.1002/cncr.11660

Biere SSAY, 2012, LANCET, V379, P1887, DOI 10.1016/S0140-6736(12)60516-9

Burmeister BH, 2011, EUR J CANCER, V47, P354, DOI 10.1016/j.ejca.2010.09.009

Chen WQ, 2016, CA-CANCER J CLIN, V66, P115, DOI 10.3322/caac.21338

Edge SB, 2010, ANN SURG ONCOL, V17, P1471, DOI 10.1245/s10434-010-0985-4

Feng MX, 2012, J AM COLL SURGEONS, V214, P838, DOI 10.1016/j.jamcollsurg.2011.12.047

Gebski V, 2007, LANCET ONCOL, V8, P226, DOI 10.1016/S1470-2045(07)70039-6

Jang R, 2015, J NATL COMPR CANC NE, V13, P229, DOI 10.6004/jnccn.2015.0029

Jemal A, 2011, CA-CANCER J CLIN, V61, P69, DOI [10.3322/caac.20107, 10.3322/caac.20115]

Kumagai K, 2014, BRIT J SURG, V101, P321, DOI 10.1002/bjs.9418

Low DE, 2015, ANN SURG, V262, P286, DOI 10.1097/SLA.0000000000001098

Luketich JD, 2012, ANN SURG, V256, P95, DOI 10.1097/SLA.0b013e3182590603

Luketich JD, 2003, ANN SURG, V238, P486, DOI 10.1097/01.sla.0000089858.40725.68

Mariette C, 2014, J CLIN ONCOL, V32, P2416, DOI 10.1200/JCO.2013.53.6532

Shapiro J, 2015, LANCET ONCOL, V16, P1090, DOI 10.1016/S1470-2045(15)00040-6

Shen YX, 2014, J AM COLL SURGEONS, V218, P108, DOI 10.1016/j.jamcollsurg.2013.09.014

Shen YX, 2012, J GASTROINTEST SURG, V16, P715, DOI 10.1007/s11605-012-1824-7

Shimoda Tadakazu, 2011, Nihon Rinsho, V69 Suppl 6, P109

Sjoquist KM, 2011, LANCET ONCOL, V12, P681, DOI 10.1016/S1470-2045(11)70142-5

Stahl M, 2009, J CLIN ONCOL, V27, P851, DOI 10.1200/JCO.2008.17.0506

Tepper J, 2008, J CLIN ONCOL, V26, P1086, DOI 10.1200/JCO.2007.12.9593

van Hagen P, 2012, NEW ENGL J MED, V366, P2074, DOI 10.1056/NEJMoa1112088

Warner S, 2014, ANN THORAC SURG, V97, P439, DOI 10.1016/j.athoracsur.2013.09.042

Zinner MJ, 2003, ANN SURG, V238, P494

NR 27

TC 18

Z9 25

U1 1

U2 9

PU BMC

PI LONDON

PA CAMPUS, 4 CRINAN ST, LONDON N1 9XW, ENGLAND

EI 1471-2407

J9 BMC CANCER

JI BMC Cancer

PD JUN 28

PY 2017

VL 17

AR 450

DI 10.1186/s12885-017-3446-7

PG 8

WC Oncology

WE Science Citation Index Expanded (SCI-EXPANDED)

SC Oncology

GA EZ8WZ

UT WOS:000405011200003

PM 28659128

OA Green Published, gold

DA 2022-05-19

ER

PT J

AU Liu, HM

Wang, X

Shi, GR

Jiang, LF

Liu, XL

AF Liu, Huaimin

Wang, Xin

Shi, Guirong

Jiang, Lifeng

Liu, Xiaoli

TI Tiam1 siRNA enhanced the sensitivity of sorafenib on esophageal squamous

cell carcinoma in vivo

SO TUMOR BIOLOGY

LA English

DT Article

DE Tiam1; Sorafenib; Esophageal squamous cell carcinoma; Nude mice; Cell

proliferation; Apoptosis

ID METASTASIS-INDUCING FACTOR-1; GASTRIC-CANCER CELLS;

HEPATOCELLULAR-CARCINOMA; INVASIVE CAPACITY; LYMPHOMA INVASION;

LUNG-CANCER; T-LYMPHOMA; APOPTOSIS; VITRO; EXPRESSION

AB Our previous study demonstrated that Tiam1 was highly expressed in esophageal squamous cell carcinoma (ESCC) tissues. In the present study, we investigated the therapeutic role of Tiam1 siRNA in combination with sorafenib in xenografted human ESCC. Our results demonstrated that expression of Tiam1 protein in EC9706 cells was significantly higher than those in ESCC cells (Eca109 and EC1) and normal esophageal epithelial cells Het-1A (P < 0.05). Tiam1 siRNA markedly suppressed Tiam1 protein expression in tumor tissues of nude mice, but sorafenib did not alter Tiam1 level. In addition, Tiam1 siRNA or sorafenib alone evidently inhibited tumor growth, reduced Ki-67 proliferation index, and induced cell apoptosis in xenografted nude mice, and their combinations had the strongest effect. Notably, Tiam1 siRNA or sorafenib alone obviously increased p27 level, but reduced Mcl-1 and bcl-2 levels in xenografted nude mice, and their combinations reached the best effect. These findings suggest that combination of Tiam1 siRNA with sorafenib may be the novel molecular therapy target for the patients with ESCC.

C1 [Liu, Huaimin; Jiang, Lifeng; Liu, Xiaoli] Zhengzhou Univ, Affiliated Tumor Hosp, Integrated TCM & Western Med Dept, Zhengzhou 450008, Henan, Peoples R China.

[Wang, Xin] Zhengzhou Univ, Affiliated Hosp 1, Dept Radiotherapy, Zhengzhou 450052, Henan, Peoples R China.

[Shi, Guirong] Shangqiu Med Coll, Dept Nursing, Shangqiu 476100, Henan, Peoples R China.

RP Liu, XL (corresponding author), Zhengzhou Univ, Affiliated Tumor Hosp, Integrated TCM & Western Med Dept, 127 Dongming Rd, Zhengzhou 450008, Henan, Peoples R China.

EM liu_xiaoli2011@126.com

CR Abou-Alfa GK, 2006, J CLIN ONCOL, V24, P4293, DOI 10.1200/JCO.2005.01.3441

Bose Prithviraj, 2013, Leuk Res Rep, V2, P12

Buchsbaum RJ, 2002, MOL CELL BIOL, V22, P4073, DOI 10.1128/MCB.22.12.4073-4085.2002

Carlo-Stella C, 2013, PLOS ONE, V8, DOI 10.1371/journal.pone.0061603

Chang YS, 2007, CANCER CHEMOTH PHARM, V59, P561, DOI 10.1007/s00280-006-0393-4

Chen G, 2013, PLOS ONE, V8, DOI 10.1371/journal.pone.0057870

Edwards BK, 2005, JNCI-J NATL CANCER I, V97, P1407, DOI 10.1093/jnci/dji289

Ekman S, 2008, EXPERT REV ANTICANC, V8, P1433, DOI 10.1586/14737140.8.9.1433

Enzinger PC, 1999, SEMIN ONCOL, V26, P12

Fleming IN, 2000, BIOCHEM J, V351, P173, DOI 10.1042/0264-6021:3510173

Galmiche A, 2014, CANCER LETT, V346, P159, DOI 10.1016/j.canlet.2013.12.028

Gridelli C, 2007, ONCOLOGIST, V12, P191, DOI 10.1634/theoncologist.12-2-191

HABETS GGM, 1994, CELL, V77, P537, DOI 10.1016/0092-8674(94)90216-X

HOCKENBERY DM, 1993, CELL, V75, P241, DOI 10.1016/0092-8674(93)80066-N

Hou GQ, 2010, CANCER LETT, V290, P248, DOI 10.1016/j.canlet.2009.09.015

Huang CY, 2013, INT J RADIAT ONCOL, V86, P456, DOI 10.1016/j.ijrobp.2013.01.025

Huang J, 2013, INT J CANCER, V132, P90, DOI 10.1002/ijc.27627

Huynh H, 2006, CLIN CANCER RES, V12, P4306, DOI 10.1158/1078-0432.CCR-05-2568

Ji ZY, 2006, CANCER LETT, V244, P182, DOI 10.1016/j.canlet.2005.12.010

Kang MR, 2012, ONCOL LETT, V3, P113, DOI 10.3892/ol.2011.430

Kusano H, 2013, INT J ONCOL, V42, P1897, DOI 10.3892/ijo.2013.1904

Lam AKY, 2000, CRIT REV ONCOL HEMAT, V33, P71, DOI 10.1016/S1040-8428(99)00054-2

LAM M, 1994, P NATL ACAD SCI USA, V91, P6569, DOI 10.1073/pnas.91.14.6569

Li K, 2002, INT J CANCER, V102, P271, DOI [10.1002/ijc.10706, 10.1002/jhc.10706]

Liu HM, 2011, ONCOL REP, V25, P841, DOI 10.3892/or.2010.1122

Liu L, 2006, NEOPLASIA, V8, P917, DOI 10.1593/neo.06364

Liu L, 2006, CANCER RES, V66, P11851, DOI 10.1158/0008-5472.CAN-06-1377

Liu Y, 2014, PLOS ONE, V9, DOI 10.1371/journal.pone.0088393

Lloyd RV, 1997, AM J PATHOL, V150, P401

Lu ZM, 2008, INT J ONCOL, V32, P643

Mertens AE, 2003, FEBS LETT, V546, P11, DOI 10.1016/S0014-5793(03)00435-6

Minard ME, 2006, CLIN EXP METASTAS, V23, P301, DOI 10.1007/s10585-006-9040-z

Parkin DM, 2001, EUR J CANCER, V37, pS4, DOI 10.1016/S0959-8049(01)00267-2

Pawaskar DK, 2013, CANCER CHEMOTH PHARM, V71, P1231, DOI 10.1007/s00280-013-2117-x

Rygiel TP, 2008, J CELL SCI, V121, P1183, DOI 10.1242/jcs.017194

Shi YL, 2013, CHINESE MED J-PEKING, V126, P640, DOI 10.3760/cma.j.issn.0366-6999.20122167

Strumberg D, 2007, ONCOLOGIST, V12, P426, DOI 10.1634/theoncologist.12-4-426

Subramanian N, 2013, PLOS ONE, V8, DOI 10.1371/journal.pone.0070422

Tsihlias J, 1999, ANNU REV MED, V50, P401

Wang S, 2014, EUR J CANCER, V50, P379, DOI 10.1016/j.ejca.2013.10.003

Wu Yuan-qing, 2012, Zhonghua Zhong Liu Za Zhi, V34, P831, DOI 10.3760/cma.j.issn.0253-3766.2012.11.007

YANG CS, 1980, CANCER RES, V40, P2633

Yu CR, 2006, MOL CANCER THER, V5, P2378, DOI 10.1158/1535-7163.MCT-06-0235

Yu LN, 2013, PLOS ONE, V8, DOI 10.1371/journal.pone.0073077

Zhang YN, 2014, MOL MED REP, V9, P365, DOI 10.3892/mmr.2013.1782

Zhu JM, 2013, MOL MED REP, V8, P425, DOI 10.3892/mmr.2013.1513

Zhu JM, 2013, ASIAN PAC J CANCER P, V14, P201, DOI 10.7314/APJCP.2013.14.1.201

NR 47

TC 4

Z9 6

U1 0

U2 5

PU SAGE PUBLICATIONS LTD

PI LONDON

PA 1 OLIVERS YARD, 55 CITY ROAD, LONDON EC1Y 1SP, ENGLAND

SN 1010-4283

EI 1423-0380

J9 TUMOR BIOL

JI Tumor Biol.

PD AUG

PY 2014

VL 35

IS 8

BP 8249

EP 8258

DI 10.1007/s13277-014-2083-x

PG 10

WC Oncology

WE Science Citation Index Expanded (SCI-EXPANDED)

SC Oncology

GA AP2DU

UT WOS:000341883500119

PM 24852430

DA 2022-05-19

ER

PT J

AU Jiang, L

Wang, CF

Sun, CL

Xu, YM

Ding, ZQ

Zhang, XL

Huang, JX

Yu, H

AF Jiang, Lin

Wang, Chaofu

Sun, Canlin

Xu, Yumin

Ding, Zhongqi

Zhang, Xueling

Huang, Junxing

Yu, Hong

TI The impact of pri-miR-218 rs11134527 on the risk and prognosis of

patients with esophageal squamous cell carcinoma

SO INTERNATIONAL JOURNAL OF CLINICAL AND EXPERIMENTAL PATHOLOGY

LA English

DT Article

DE Esophageal squamous cell carcinoma; miR-218; laminin 5; single

nucleotide polymorphism; susceptibility

ID POOR-PROGNOSIS; CANCER; MICRORNA-218; EXPRESSION; INVASION; ASSOCIATION;

MIGRATION; POLYMORPHISMS; MATURATION; BIOMARKERS

AB MicroRNA-218 (miR-218) acts as a tumor suppressor in numerous types of cancer by regulation of the expression of target genes. The aim of this study was to investigate whether polymorphisms in miR-218 LAMB3 pathway were associated with the risk and prognosis of esophageal squamous cell carcinoma (ESCC). Pri-mir-218 rs11134527 and LAMB3 rs2566 were genotyped in ESCC patients and 745 controls to assess their associations with cancer risk and overall survival. Pri-mir-218 rs11134527 was significantly associated with a decreased risk of ESCC under codominant, recessive and additive models. Although there was a significant association between rs11134527 and better survival of ESCC patients under codominant, recessive and additive models, the association disappeared after adjustment for TNM and LNM. However, further stratified analysis revealed that the association remained significant in patients with TNM stages I and II or non-LNM. Our data suggest that pri-miR-218 rs11134527 may contribute to the genetic susceptibility and prognosis for ESCC in Chinese Han population.

C1 [Jiang, Lin; Sun, Canlin; Xu, Yumin] Taizhou Peoples Hosp, Dept Anesthesiol, Taizhou 225300, Jiangsu, Peoples R China.

[Wang, Chaofu] Fudan Univ, Shanghai Canc Ctr, Dept Pathol, Shanghai 200433, Peoples R China.

[Ding, Zhongqi] Taizhou Peoples Hosp, Dept Nucl Med, Taizhou 225300, Jiangsu, Peoples R China.

[Zhang, Xueling] Taizhou Cent Hosp, Dept Thorac Surg, Taizhou 318000, Zhejiang, Peoples R China.

[Huang, Junxing; Yu, Hong] Taizhou Peoples Hosp, Dept Oncol, 210 Yingchun Rd, Taizhou 225300, Jiangsu, Peoples R China.

[Yu, Hong] Taizhou Peoples Hosp, Dept Pathol, Taizhou 225300, Jiangsu, Peoples R China.

RP Yu, H (corresponding author), Taizhou Peoples Hosp, Dept Oncol, 210 Yingchun Rd, Taizhou 225300, Jiangsu, Peoples R China.

EM hjxtz@sina.cn; yuhongmiao-miao@163.com

FU Health Scientific Research Foundation, Jiangsu, China [H201260]

FX This work was supported by the Health Scientific Research Foundation,

Jiangsu, China (grant No. H201260).

CR Bosman FT, 2003, J PATHOL, V200, P423, DOI 10.1002/path.1437

Chen CR, 2014, TOHOKU J EXP MED, V233, P89, DOI 10.1620/tjem.233.89

Ding SL, 2013, J BIOL CHEM, V288, P26865, DOI 10.1074/jbc.M112.440453

Gao XB, 2013, GENE, V523, P173, DOI 10.1016/j.gene.2013.03.119

Guo H, 2010, FAM CANCER, V9, P599, DOI 10.1007/s10689-010-9370-5

Han GH, 2014, INT J CLIN EXP PATHO, V7, P751

Hayes J, 2014, TRENDS MOL MED, V20, P460, DOI 10.1016/j.molmed.2014.06.005

He XQ, 2012, MOL MED, V18, P1491, DOI 10.2119/molmed.2012.00304

Hesse M, 2014, METHODS MOL BIOL, V1095, P11, DOI 10.1007/978-1-62703-703-7_2

Hu ZB, 2008, J CLIN INVEST, V118, P2600, DOI 10.1172/JCI34934

Huang J, 2014, CELL PROLIFERAT, V47, P277, DOI 10.1111/cpr.12109

Jemal A, 2011, CA-CANCER J CLIN, V61, P69, DOI [10.3322/caac.20107, 10.3322/caac.20115]

Kinoshita T, 2012, ONCOTARGET, V3, P1386, DOI 10.18632/oncotarget.709

Kita Y, 2009, EJSO-EUR J SURG ONC, V35, P52, DOI 10.1016/j.ejso.2008.01.025

Lewis BP, 2005, CELL, V120, P15, DOI 10.1016/j.cell.2004.12.035

Li JR, 2012, INT J MOL SCI, V13, P16053, DOI 10.3390/ijms131216053

Lin JH, 2014, PEDIATR NEPHROL, V29, P1979, DOI 10.1007/s00467-014-2818-3

Miranda KC, 2006, CELL, V126, P1203, DOI 10.1016/j.cell.2006.07.031

Nishikawa R, 2014, CANCER SCI, V105, P802, DOI 10.1111/cas.12441

Shi TY, 2013, BMC CANCER, V13, DOI 10.1186/1471-2407-13-19

Wang N, 2014, BIOMARKERS, V19, P43, DOI 10.3109/1354750X.2013.866164

Wang Y, 2014, INT J MED SCI, V11, P691, DOI 10.7150/ijms.8880

Wu CH, 2014, TOHOKU J EXP MED, V232, P85, DOI 10.1620/tjem.232.85

Wu CH, 2014, CANCER CHEMOTH PHARM, V73, P335, DOI 10.1007/s00280-013-2364-x

Xin SY, 2014, WORLD J GASTROENTERO, V20, P6906, DOI 10.3748/wjg.v20.i22.6906

Yamamoto N, 2013, INT J ONCOL, V42, P1523, DOI 10.3892/ijo.2013.1851

Yu H, 2013, INT J CLIN EXP PATHO, V6, P2904

Yuan J, 2013, PLOS ONE, V8, DOI [10.1371/journal.pone.0059885, 10.1371/journal.pone.0061047]

Zhang JJ, 2014, PLOS ONE, V9, DOI 10.1371/journal.pone.0100055

Zhang XL, 2013, PATHOL RES PRACT, V209, P510, DOI 10.1016/j.prp.2013.06.005

Zhang XL, 2012, PLOS ONE, V7, DOI 10.1371/journal.pone.0041930

Zhi H, 2012, CLIN RES CARDIOL, V101, P289, DOI 10.1007/s00392-011-0391-3

Zhou XY, 2010, GYNECOL ONCOL, V117, P287, DOI 10.1016/j.ygyno.2010.01.020

Zhu ZM, 2014, J SURG ONCOL, V109, P89, DOI 10.1002/jso.23475

NR 34

TC 16

Z9 16

U1 0

U2 7

PU E-CENTURY PUBLISHING CORP

PI MADISON

PA 40 WHITE OAKS LN, MADISON, WI 53711 USA

SN 1936-2625

J9 INT J CLIN EXP PATHO

JI Int. J. Clin. Exp. Pathol.

PY 2014

VL 7

IS 9

BP 6206

EP 6212

PG 7

WC Oncology; Pathology

WE Science Citation Index Expanded (SCI-EXPANDED)

SC Oncology; Pathology

GA AT7PH

UT WOS:000345129400090

PM 25337271

DA 2022-05-19

ER

PT J

AU Zhang, CY

Luo, Y

Cao, JJ

Wang, XY

Miao, ZW

Shao, GQ

AF Zhang, Chunyu

Luo, Yan

Cao, Jingjing

Wang, Xiaoyu

Miao, Zhiwei

Shao, Guoqing

TI Exosomal lncRNA FAM225A accelerates esophageal squamous cell carcinoma

progression and angiogenesis via sponging miR-206 to upregulate NETO2

and FOXP1 expression

SO CANCER MEDICINE

LA English

DT Article

DE angiogenesis; esophageal squamous cell carcinoma; exosomal; FAM225A

ID COMPETING ENDOGENOUS RNA; LONG NONCODING RNAS; INVASION; PROLIFERATION;

MIGRATION; TARGET

AB Esophageal cancer is one of the leading causes of cancer-related deaths worldwide. FAM225A is a novel lncRNA, only has been explored in nasopharyngeal carcinoma tumorigenesis. This study aims to investigate the regulatory mechanism of FAM225A in esophageal squamous cell carcinoma (ESCC). We discovered that FAM225A exhibited higher expression in ESCC. The silence of FAM225A attenuated cell viability, migration, and invasion, but facilitated cell apoptosis in ESCC. Exosome-mediated transfer of lncRNA FAM225A could participate in ESCC progression. In addition, we found that miR-206 bound to FAM225A. Moreover, we further demonstrated that FAM225A absorbed miR-206 to upregulate NETO2 and FOXP1 expression, and FOXP1 acted as a transcription factor to enhance FAM225A expression. Eventually, it was revealed that the overexpression of NETO2 or FOXP1 rescued the effects of FAM225A repression on ESCC progression. Our results suggested that FAM225A upregulated NETO2 and FOXP1 expression by sponging miR-206 to accelerate ESCC progression and angiogenesis. These results determined the biological role of lncRNA FAM225A in ESCC tumorigenesis, and FAM225A may be a promising biomarker for ESCC treatment.

C1 [Zhang, Chunyu; Luo, Yan; Cao, Jingjing; Wang, Xiaoyu; Miao, Zhiwei; Shao, Guoqing] Nanjing Univ Chinese Med, Zhangjiagang TCM Hosp, 77 Changan South Rd, Zhangjiagang 215600, Peoples R China.

RP Miao, ZW; Shao, GQ (corresponding author), Nanjing Univ Chinese Med, Zhangjiagang TCM Hosp, 77 Changan South Rd, Zhangjiagang 215600, Peoples R China.

EM zhiweimiao871003@163.com; guoqingshao0420@163.com

RI Miao, Zhiwei/AFA-9607-2022

CR Ambros V, 2004, NATURE, V431, P350, DOI 10.1038/nature02871

Arnold M, 2015, GUT, V64, P381, DOI 10.1136/gutjnl-2014-308124

Bartel DP, 2009, CELL, V136, P215, DOI 10.1016/j.cell.2009.01.002

Cheng Y, 2019, J ONCOL, V2019, DOI 10.1155/2019/2476175

Dai CX, 2018, BIOMED PHARMACOTHER, V104, P763, DOI 10.1016/j.biopha.2018.05.077

Gibb EA, 2011, MOL CANCER, V10, DOI 10.1186/1476-4598-10-38

He AR, 2019, BIOCHEM BIOPH RES CO, V513, P494, DOI 10.1016/j.bbrc.2019.03.061

He L, 2004, NAT REV GENET, V5, P522, DOI 10.1038/nrg1379

Hu L, 2015, BMC CANCER, V15, DOI 10.1186/s12885-015-2018-y

Jiang Z, 2018, BIOCHEM BIOPH RES CO, V495, P238, DOI 10.1016/j.bbrc.2017.11.012

Kumar MM, 2017, CURR TOP MED CHEM, V17, P1750, DOI 10.2174/1568026617666161116144744

Lang HL, 2017, ONCOL REP, V38, P785, DOI 10.3892/or.2017.5742

Li X-Y, 2019, AGING ALBANY NY, V11

Li X, 2019, 2019 2ND INTERNATIONAL CONFERENCE ON MECHANICAL ENGINEERING, INDUSTRIAL MATERIALS AND INDUSTRIAL ELECTRONICS (MEIMIE 2019), P44, DOI 10.23977/meimie.2019.43009

Li ZW, 2017, CELL DEATH DIS, V8, DOI 10.1038/cddis.2017.245

Li ZY, 2018, BIOMED PHARMACOTHER, V104, P181, DOI 10.1016/j.biopha.2018.05.047

Lian Y, 2018, J EXP CLIN CANC RES, V37, DOI 10.1186/s13046-018-0918-9

Liu JG, 2016, ASIA-PAC J CLIN ONCO, V12, P308, DOI 10.1111/ajco.12503

Liu JY, 2019, CELL DEATH DIS, V10, DOI 10.1038/s41419-019-1388-5

Liu ML, 2018, WORLD J GASTROENTERO, V24, P1004, DOI 10.3748/wjg.v24.i9.1004

Qiu JJ, 2018, INT J BIOL SCI, V14, P1960, DOI 10.7150/ijbs.28048

Samaeekia R, 2017, CLIN CANCER RES, V23, P1091, DOI 10.1158/1078-0432.CCR-16-0943

Schmitt AM, 2016, CANCER CELL, V29, P452, DOI 10.1016/j.ccell.2016.03.010

Sheng SR, 2017, FUTURE ONCOL, V13, P1551, DOI 10.2217/fon-2017-0149

Shima H, 2018, BREAST CANCER RES TR, V170, P507, DOI 10.1007/s10549-018-4793-z

Smyth EC, 2017, NAT REV DIS PRIMERS, V3, DOI 10.1038/nrdp.2017.48

Tay Y, 2014, NATURE, V505, P344, DOI 10.1038/nature12986

Wang LX, 2019, MED SCI MONITOR, V25, P2896, DOI 10.12659/MSM.913881

Wang Y, 2018, PROSTATE, V78, P479, DOI 10.1002/pros.23468

Wang YY, 2018, ONCOL RES, V26, P923, DOI 10.3727/096504017X15143731031009

Wang Y, 2018, CANCER LETT, V434, P172, DOI 10.1016/j.canlet.2018.07.016

Wang ZZ, 2019, EUR REV MED PHARMACO, V23, P5206, DOI 10.26355/eurrev_201906_18185

Yuan RB, 2018, EUR REV MED PHARMACO, V22, P7265, DOI 10.26355/eurrev_201811_16261

Zhang YW, 2013, WORLD J GASTROENTERO, V19, P5598, DOI 10.3748/wjg.v19.i34.5598

Zhang ZH, 2019, ACTA BIOCH BIOPH SIN, V51, P826, DOI 10.1093/abbs/gmz069

Zhao J, 2019, AM J CANCER RES, V9, P1367

Zheng ZQ, 2019, CANCER RES, V79, P4612, DOI 10.1158/0008-5472.CAN-19-0799

Zhong YB, 2018, EUR REV MED PHARMACO, V22, P8288, DOI 10.26355/eurrev_201812_16526

NR 38

TC 12

Z9 14

U1 1

U2 2

PU WILEY

PI HOBOKEN

PA 111 RIVER ST, HOBOKEN 07030-5774, NJ USA

SN 2045-7634

J9 CANCER MED-US

JI Cancer Med.

PD NOV

PY 2020

VL 9

IS 22

BP 8600

EP 8611

DI 10.1002/cam4.3463

EA OCT 2020

PG 12

WC Oncology

WE Science Citation Index Expanded (SCI-EXPANDED)

SC Oncology

GA OR0QH

UT WOS:000574177300001

PM 33006432

OA gold, Green Published

DA 2022-05-19

ER

PT J

AU Mao, XL

Zhu, HY

Luo, DH

Ye, LP

Yin, HF

Zhang, JS

Zhang, Y

Zhang, Y

AF Mao, Xinli

Zhu, Hongyuan

Luo, Dinghai

Ye, Liping

Yin, Huifei

Zhang, Jinshun

Zhang, Yan

Zhang, Yu

TI Capsaicin inhibits glycolysis in esophageal squamous cell carcinoma by

regulating hexokinase-2 expression

SO MOLECULAR MEDICINE REPORTS

LA English

DT Article

DE capsaicin; esophageal squamous cell carcinoma; glycolysis; hexokinase-2;

phosphatase and tensin homolog

ID TOTAL LESION GLYCOLYSIS; COLON-CANCER CELLS; HEPATOCELLULAR-CARCINOMA;

OVARIAN-CANCER; APOPTOSIS; OVEREXPRESSION; 5-FLUOROURACIL; MITOCHONDRIA;

PATHWAY; STRESS

AB Capsaicin is a principal component of hot red peppers and chili peppers. Previous studies have reported that capsaicin exhibits antitumor functions in a variety of tumor models. Although various mechanisms underlying the capsaicin-mediated inhibition of tumor growth have been demonstrated, the impact of capsaicin on tumor metabolism has rarely been reported. The present study demonstrated that capsaicin exhibited an inhibitory effect on tumor glycolysis in esophageal squamous cell carcinoma (ESCC) cells. Following treatment with capsaicin, glucose consumption and lactate production in ESCC cells was decreased. Capsaicin resulted in a decrease of hexokinase-2 (HK-2) expression, which is known for its important role in tumor glycolysis. Further investigations demonstrated that phosphatase and tensin homolog (PTEN) expression was increased in ESCC cells treated with capsaicin, and that the RAC- serine threonine-protein kinase signaling pathway was downregulated. In PTEN-knockdown KYSE150 cells, the decrease in HK-2 and inhibition of glycolysis caused by capsaicin was attenuated, which suggested that the impact of capsaicin on tumor metabolism was associated with its effect on PTEN.

C1 [Mao, Xinli; Zhu, Hongyuan; Luo, Dinghai; Ye, Liping; Yin, Huifei; Zhang, Jinshun; Zhang, Yan; Zhang, Yu] Taizhou Hosp Zhejiang Prov, Wenzhou Med Coll, Dept Gastroenterol, 150 Ximen St, Linhai 317000, Zhejiang, Peoples R China.

RP Zhang, Y (corresponding author), Taizhou Hosp Zhejiang Prov, Wenzhou Med Coll, Dept Gastroenterol, 150 Ximen St, Linhai 317000, Zhejiang, Peoples R China.

EM yuzhang201601@126.com

OI Zhu, HongYuan/0000-0001-8645-3833

FU Health Bureau of Zhejiang Province [2015KYB433]

FX The present study was funded by the Health Bureau of Zhejiang Province

(grant no. 2015KYB433).

CR Bhattacharya B, 2016, BRIT J PHARMACOL, V173, P970, DOI 10.1111/bph.13422

Burness CB, 2016, DRUGS, V76, P123, DOI 10.1007/s40265-015-0520-9

Chang D, 2007, DIS ESOPHAGUS, V20, P491, DOI 10.1111/j.1442-2050.2007.00695.x

Clark R, 2016, ANTICANCER RES, V36, P837

DELAPAZ RL, 1983, AM J NEURORADIOL, V4, P826

Diaz-Laviada I, 2010, FUTURE ONCOL, V6, P1545, DOI [10.2217/fon.10.117, 10.2217/FON.10.117]

Enzinger PC, 2003, NEW ENGL J MED, V349, P2241, DOI 10.1056/NEJMra035010

Guzman G, 2015, DIGEST DIS SCI, V60, P420, DOI 10.1007/s10620-014-3364-3

Hong JH, 2016, J KOREAN MED SCI, V31, P39, DOI 10.3346/jkms.2016.31.1.39

Hu F, 2012, BIOCHEM BIOPH RES CO, V424, P196, DOI 10.1016/j.bbrc.2012.06.115

Huang SP, 2009, ANTICANCER RES, V29, P165

Ito K, 2004, CANCER RES, V64, P1071, DOI 10.1158/0008-5472.CAN-03-1670

Jiang JX, 2014, MOL MED REP, V10, P995, DOI 10.3892/mmr.2014.2271

Jin JZ, 2014, INT J BIOL SCI, V10, P285, DOI 10.7150/ijbs.7730

Jin Z, 2014, EUR J GYNAECOL ONCOL, V35, P519

Lau JK, 2014, APOPTOSIS, V19, P1190, DOI 10.1007/s10495-014-1007-y

Lee HK, 2009, BIOCHEM BIOPH RES CO, V382, P445, DOI 10.1016/j.bbrc.2009.03.046

Li YM, 2014, ASIAN PAC J CANCER P, V15, P1369, DOI 10.7314/APJCP.2014.15.3.1369

Matharu M, 2010, BMJ CLIN EVID, V02, P1212

Mathupala SP, 2006, ONCOGENE, V25, P4777, DOI 10.1038/sj.onc.1209603

Mathupala SP, 2009, SEMIN CANCER BIOL, V19, P17, DOI 10.1016/j.semcancer.2008.11.006

Ogawa H, 2015, MOL CLIN ONCOL, V3, P563, DOI 10.3892/mco.2015.490

Patra KC, 2013, CANCER CELL, V24, P213, DOI 10.1016/j.ccr.2013.06.014

Peng QP, 2009, HEPATO-GASTROENTEROL, V56, P355

Richards BL, 2012, COCHRANE DB SYST REV, DOI 10.1002/14651858.CD008921.pub2

Sarkar Arnab, 2015, Asian Pac J Cancer Prev, V16, P6753

Schreurs LMA, 2014, ANN SURG ONCOL, V21, P3751, DOI 10.1245/s10434-014-3848-6

Suh DH, 2014, CLIN EXP MED, V14, P345, DOI 10.1007/s10238-013-0250-9

Wang L, 2014, CELL REP, V8, P1461, DOI 10.1016/j.celrep.2014.07.053

Wilson JE, 2003, J EXP BIOL, V206, P2049, DOI 10.1242/jeb.00241

NR 30

TC 7

Z9 9

U1 2

U2 12

PU SPANDIDOS PUBL LTD

PI ATHENS

PA POB 18179, ATHENS, 116 10, GREECE

SN 1791-2997

EI 1791-3004

J9 MOL MED REP

JI Mol. Med. Rep.

PD APR

PY 2018

VL 17

IS 4

BP 6116

EP 6121

DI 10.3892/mmr.2018.8574

PG 6

WC Oncology; Medicine, Research & Experimental

WE Science Citation Index Expanded (SCI-EXPANDED)

SC Oncology; Research & Experimental Medicine

GA GA9PA

UT WOS:000428672100161

PM 29436634

OA hybrid

DA 2022-05-19

ER

PT J

AU Takata, A

Takiguchi, S

Okada, K

Takahashi, T

Kurokawa, Y

Yamasaki, M

Miyata, H

Nakajima, K

Mori, M

Doki, Y

AF Takata, Akihiro

Takiguchi, Shuji

Okada, Kaoru

Takahashi, Tsuyoshi

Kurokawa, Yukinori

Yamasaki, Makoto

Miyata, Hiroshi

Nakajima, Kiyokazu

Mori, Masaki

Doki, Yuichiro

TI Clinicopathological and Prognostic Significance of FOXM1 Expression in

Esophageal Squamous Cell Carcinoma

SO ANTICANCER RESEARCH

LA English

DT Article

DE FOXM1; esophageal squamous cell carcinoma; immunohistochemistry

ID TRANSCRIPTION FACTOR FOXM1; FORKHEAD BOX M1B; COLORECTAL-CANCER;

BREAST-CANCER; DOWNSTREAM TARGET; OXIDATIVE STRESS; OVER-EXPRESSION;

POOR-PROGNOSIS; GASTRIC-CANCER; GLIOMA-CELLS

AB Background: Esophageal squamous cell carcinoma (ESCC) has a poor prognosis because invasion and metastasis are prevalent. To improve diagnosis, it is important to identify and characterize tumor-specific molecular markers in ESCC. FOXM1 is overexpressed and correlates with pathogenesis in a variety of human malignancies. We aimed to investigate the clinical significance of FOXM1 overexpression in ESCC. Patients and Methods: FOXM1 expression was assessed in ESCC specimens from 174 curatively-resected cases. The relationships between FOXM1 expression, clinicopathological parameters, and prognoses were examined. Results: Immunohistochemical analysis showed that 94 (54.0%) tumors were positive for FOXM1 expression. FOXM1 positivity did not correlate with any clinicopathological parameter. However, FOXM1-positive cases had poorer prognoses than FOXM1-negative ones (p=0.0037, log-rank test). In multivariate analysis, the following were independent prognostic factors: pT, pN, neoadjuvant chemotherapy, and FOXM1 expression (hazard ratio-1.69, 95% confidence interval=1.06-2.75, p=0.027). Conclusion: FOXM1 may be a novel prognostic factor in patients with ESCC who undergo curative resection.

C1 [Takata, Akihiro; Takiguchi, Shuji; Takahashi, Tsuyoshi; Kurokawa, Yukinori; Yamasaki, Makoto; Miyata, Hiroshi; Nakajima, Kiyokazu; Mori, Masaki; Doki, Yuichiro] Osaka Univ, Grad Sch Med, Dept Surg Gastroenterol, Suita, Osaka 5650871, Japan.

[Okada, Kaoru] Nishinomiya Municipal Cent Hosp, Dept Surg, Nishinomiya, Hyogo, Japan.

RP Takiguchi, S (corresponding author), Osaka Univ, Grad Sch Med, Dept Surg Gastroenterol, 2-2,E2 Yamadaoka, Suita, Osaka 5650871, Japan.

EM stakiguchi@gesurg.med.osaka-u.ac.jp

CR Ahmad A, 2010, BREAST CANCER RES TR, V122, P337, DOI 10.1007/s10549-009-0572-1

Bektas N, 2008, BMC CANCER, V8, DOI 10.1186/1471-2407-8-42

Calvisi DF, 2009, GUT, V58, P679, DOI 10.1136/gut.2008.152652

Chan DW, 2008, J PATHOL, V215, P245, DOI 10.1002/path.2355

Chu XY, 2012, ACTA HISTOCHEM, V114, P755, DOI 10.1016/j.acthis.2012.01.002

Douard R, 2006, SURGERY, V139, P665, DOI 10.1016/j.surg.2005.10.012

Francis RE, 2009, INT J ONCOL, V35, P57, DOI 10.3892/ijo_00000313

Hui MKC, 2012, WORLD J SURG, V36, P90, DOI 10.1007/s00268-011-1302-5

Kalin TV, 2006, CANCER RES, V66, P1712, DOI 10.1158/0008-5472.CAN-05-3138

Kalinichenko VV, 2004, GENE DEV, V18, P830, DOI 10.1101/gad.1200704

Karin M, 2006, MOL CARCINOGEN, V45, P355, DOI 10.1002/mc.20217

Kim IM, 2006, CANCER RES, V66, P2153, DOI 10.1158/0008-5472.CAN-05-3003

Laoukili J, 2005, NAT CELL BIOL, V7, P126, DOI 10.1038/ncb1217

Leung TWC, 2001, FEBS LETT, V507, P59, DOI 10.1016/S0014-5793(01)02915-5

Li Q, 2009, CANCER RES, V69, P3501, DOI 10.1158/0008-5472.CAN-08-3045

Li SKM, 2008, J BIOL CHEM, V283, P16545, DOI 10.1074/jbc.M709604200

Liu MG, 2006, CANCER RES, V66, P3593, DOI 10.1158/0008-5472.CAN-05-2912

Madureira PA, 2006, J BIOL CHEM, V281, P25167, DOI 10.1074/jbc.M603906200

Major ML, 2004, MOL CELL BIOL, V24, P2649, DOI 10.1128/MCB.24.7.2649-2661.2004

Okada K, 2013, ANN SURG ONCOL, V20, P1035, DOI 10.1245/s10434-012-2680-0

Park HJ, 2009, EMBO J, V28, P2908, DOI 10.1038/emboj.2009.239

Raychaudhuri P, 2011, CANCER RES, V71, P4329, DOI 10.1158/0008-5472.CAN-11-0640

Shimada H, 2003, SURGERY, V133, P486, DOI 10.1067/msy.2003.139

Sun HC, 2011, ONCOL REP, V25, P1533, DOI 10.3892/or.2011.1230

Tamoto E, 2004, CLIN CANCER RES, V10, P3629, DOI 10.1158/1078-0432.CCR-04-0048

Tan YJ, 2007, MOL CELL BIOL, V27, P1007, DOI 10.1128/MCB.01068-06

Teh MT, 2002, CANCER RES, V62, P4773

Uddin S, 2011, AM J PATHOL, V178, P537, DOI 10.1016/j.ajpath.2010.10.020

Wang IC, 2008, ONCOGENE, V27, P4137, DOI 10.1038/onc.2008.60

Wang IC, 2008, J BIOL CHEM, V283, P20770, DOI 10.1074/jbc.M709892200

Wang IC, 2005, MOL CELL BIOL, V25, P10875, DOI 10.1128/MCB.25.24.10875-10894.2005

Wang ZW, 2007, CANCER RES, V67, P8293, DOI 10.1158/0008-5472.CAN-07-1265

Wierstra I, 2007, BIOL CHEM, V388, P1257, DOI 10.1515/BC.2007.159

Wierstra I, 2006, FEBS J, V273, P4645, DOI 10.1111/j.1742-4658.2006.05468.x

Wierstra I, 2007, BIOCHEM BIOPH RES CO, V352, P61, DOI 10.1016/j.bbrc.2006.10.151

Wonsey DR, 2005, CANCER RES, V65, P5181, DOI 10.1158/0008-5472.CAN-04-4059

Xia LM, 2009, J CELL BIOCHEM, V106, P247, DOI 10.1002/jcb.21996

Zhang YJ, 2008, CANCER RES, V68, P8733, DOI 10.1158/0008-5472.CAN-08-1968

NR 38

TC 20

Z9 23

U1 0

U2 0

PU INT INST ANTICANCER RESEARCH

PI ATHENS

PA EDITORIAL OFFICE 1ST KM KAPANDRITIOU-KALAMOU RD KAPANDRITI, PO BOX 22,

ATHENS 19014, GREECE

SN 0250-7005

EI 1791-7530

J9 ANTICANCER RES

JI Anticancer Res.

PD MAY

PY 2014

VL 34

IS 5

BP 2427

EP 2432

PG 6

WC Oncology

WE Science Citation Index Expanded (SCI-EXPANDED)

SC Oncology

GA AG6RK

UT WOS:000335545900049

PM 24778055

DA 2022-05-19

ER

PT J

AU Shen, WT

Tang, DR

Wan, P

Peng, ZY

Sun, MJ

Guo, XX

Liu, R

AF Shen, Weitao

Tang, Derong

Wan, Ping

Peng, Zhenyan

Sun, Mingjun

Guo, Xinxin

Liu, Ran

TI Identification of tissue-specific microbial profile of esophageal

squamous cell carcinoma by full-length 16S rDNA sequencing

SO APPLIED MICROBIOLOGY AND BIOTECHNOLOGY

LA English

DT Article

DE Esophageal squamous cell carcinoma; 16S rDNA full-length sequencing;

Microbial profile; In situ microorganism

ID SP NOV.; APOPTOSIS; RISK; GENE

AB It was previously believed that the microbial community in the esophagus was relatively stable, but it has been reported that different esophageal diseases have different microbial community characteristics. In this study, we recruited patients with esophageal squamous cell carcinoma (ESCC) and collected 51 pairs of tumor and adjacent non-tumor tissues for full-length 16S rDNAsequencing and qPCR to compare the differences in microbial community structure. The results of sequencing in 19 pairs of tissues showed that Proteobacteria, Firmicutes, Bacteroidetes, Deinococcus-Thermus, and Actinobacteria were the main bacteria in tumor and adjacent non-tumor tissues. At the genus level, the bacteria with the highest relative proportion in tumor and adjacent non-tumor tissues were Streptococcus and Labrys, respectively. At the same time, it was observed that the complexity of microbial interactions in tumor tissues was weaker than that of adjacent non-tumor tissues. The results also found that the relative abundance of 24 taxa was statistically different between tumor and adjacent non-tumor tissues. The findings of qPCR in 32 pairs of tissues further evidence that the relative proportions of Blautia, Treponema, Lactobacillus murinus, Peptoanaerobacter stomatis, and Fusobacteria periodonticum were statistically different in tumor and adjacent non-tumor tissues. The findings of PIRCUSt2 indicated the lipopolysaccharide biosynthesis and biotin metabolism in the microbiome of cancer tissues are more significant. This study supplements the existing information on the structure, function, and interaction of microorganisms in the esophagus in situ and provides a direction for the further exploration of the relationship between esophageal in situ microorganisms and esophageal squamous cell carcinoma.

C1 [Shen, Weitao; Wan, Ping; Peng, Zhenyan; Sun, Mingjun; Guo, Xinxin; Liu, Ran] Southeast Univ, Sch Publ Hlth, Key Lab Environm Med Engn, Minist Educ, Nanjing 210009, Peoples R China.

[Tang, Derong] Nanjing Med Univ, Dept Thorac Surg, Affiliated Huaian Peoples Hosp 1, Huaian 223300, Jiangsu, Peoples R China.

RP Liu, R (corresponding author), Southeast Univ, Sch Publ Hlth, Key Lab Environm Med Engn, Minist Educ, Nanjing 210009, Peoples R China.

EM ranliu@seu.edu.cn

FU National Natural Science Foundation of China [81872579, 82173479];

Ministry of Education [NCET-13-012]

FX National Natural Science Foundation of China grants,81872579, Ran

Liu,82173479,Ran Liu,New Century Excellent Talents in University from

Ministry of Education, NCET-13-012,Ran Liu

CR Abnet CC, 2018, GASTROENTEROLOGY, V154, P360, DOI 10.1053/j.gastro.2017.08.023

Albert RA, 2010, INT J SYST EVOL MICR, V60, P1570, DOI 10.1099/ijs.0.014977-0

Alvarez L, 2014, APPL ENVIRON MICROB, V80, P19, DOI 10.1128/AEM.02594-13

Bessa VS, 2019, SCI TOTAL ENVIRON, V690, P739, DOI 10.1016/j.scitotenv.2019.06.461

Binda C, 2018, DIGEST LIVER DIS, V50, P421, DOI 10.1016/j.dld.2018.02.012

Callahan BJ, 2019, NUCLEIC ACIDS RES, V47, DOI 10.1093/nar/gkz569

Carvalho MF, 2008, INT J SYST EVOL MICR, V58, P692, DOI 10.1099/ijs.0.65472-0

Chen WG, 2012, PLOS ONE, V7, DOI 10.1371/journal.pone.0039743

Coker OO, 2018, GUT, V67, P1024, DOI 10.1136/gutjnl-2017-314281

Cryan JF, 2019, PHYSIOL REV, V99, P1877, DOI 10.1152/physrev.00018.2018

De Luca F, 2019, CLIN EXP IMMUNOL, V195, P74, DOI 10.1111/cei.13158

Duan HY, 2014, CANCER CELL INT, V14, DOI 10.1186/s12935-014-0135-3

Duan HY, 2014, CANCER LETT, V354, P447, DOI 10.1016/j.canlet.2014.08.018

Edge SB, 2010, AJCC CANC STAGING MA, V7th ed

FINLAY IG, 1982, THORAX, V37, P181, DOI 10.1136/thx.37.3.181

Freedberg DE, 2014, CLIN LAB MED, V34, P771, DOI 10.1016/j.cll.2014.08.008

Gessner AL, 2021, CANCER CAUSE CONTROL, V32, P1347, DOI 10.1007/s10552-021-01482-6

Goldstein SL, 2020, FRONT MICROBIOL, V11, DOI 10.3389/fmicb.2020.621041

Goncalves LFH, 2012, J PERIODONTAL RES, V47, P711, DOI 10.1111/j.1600-0765.2012.01485.x

Greathouse KL, 2018, GENOME BIOL, V19, DOI 10.1186/s13059-018-1501-6

Huang B, 2014, INT J CLIN EXP PATHO, V7, P584

Johnson CH, 2015, CELL METAB, V21, P891, DOI 10.1016/j.cmet.2015.04.011

Kamarajan P, 2020, PLOS PATHOG, V16, DOI 10.1371/journal.ppat.1008881

Kovaleva O, 2021, BIOMEDICINES, V9, DOI 10.3390/biomedicines9070743

Kubo Y, 2022, ESOPHAGUS-TOKYO, V19, P39, DOI 10.1007/s10388-021-00892-4

Ling ZX, 2019, FRONT IMMUNOL, V10, DOI 10.3389/fimmu.2019.00533

Luu TH, 2017, NUTR CANCER, V69, P267, DOI 10.1080/01635581.2017.1263750

Lv ZL, 2019, MUCOSAL IMMUNOL, V12, P1164, DOI 10.1038/s41385-019-0184-y

Maruvada P, 2017, CELL HOST MICROBE, V22, P589, DOI 10.1016/j.chom.2017.10.005

Mitchell J, 2011, MOL ORAL MICROBIOL, V26, P89, DOI 10.1111/j.2041-1014.2010.00601.x

Mwachiro MM, 2021, ENVIRON INT, V152, DOI 10.1016/j.envint.2021.106485

Nobel YR, 2018, CLIN TRANSL GASTROEN, V9, DOI 10.1038/s41424-018-0067-7

Park CH, 2020, J NEUROGASTROENTEROL, V26, P171, DOI 10.5056/jnm19240

Peters BA, 2017, CANCER RES, V77, P6777, DOI 10.1158/0008-5472.CAN-17-1296

Pinzauti D., 2022, MICROB GENOMICS, V8

Poosari A, 2021, EUR J MED RES, V26, DOI 10.1186/s40001-021-00561-3

Poppleton DI, 2017, FRONT MICROBIOL, V8, DOI 10.3389/fmicb.2017.01215

Quast Christian, 2013, Nucleic Acids Res, V41, pD590, DOI 10.1093/nar/gks1219

R Core Team, 2021, R LANG ENV STAT COMP

Riquelme E, 2019, CELL, V178, P795, DOI 10.1016/j.cell.2019.07.008

Salaemae W, 2016, MICROBIOL SPECTR, V4, DOI 10.1128/microbiolspec.VMBF-0008-2015

Sawada G, 2016, GASTROENTEROLOGY, V150, P1171, DOI 10.1053/j.gastro.2016.01.035

Shannon P, 2003, GENOME RES, V13, P2498, DOI 10.1101/gr.1239303

Shao DT, 2019, CANCER-AM CANCER SOC, V125, P3993, DOI 10.1002/cncr.32403

Sun HY, 2020, BIOMED RES INT, V2020, DOI 10.1155/2020/4712498

Sung HH, 2022, CLIN EXP HYPERTENS, V44, P146, DOI 10.1080/10641963.2021.2007943

Umar S, 2012, CURR COLORECT CANC R, V8, P298, DOI 10.1007/s11888-012-0143-4

Vashishta A, 2019, PATHOGENS, V8, DOI 10.3390/pathogens8020059

Wang G, 2022, J CANCER, V13, P413, DOI 10.7150/jca.65400

Wang HN, 2017, GENOME MED, V9, DOI 10.1186/s13073-017-0405-5

Wang JB, 2012, PLOS ONE, V7, DOI 10.1371/journal.pone.0042281

Wang YJ, 2020, CANCER MANAG RES, V12, P11553, DOI 10.2147/CMAR.S275316

Xiao CH, 2021, SUPPORT CARE CANCER, V29, P3173, DOI 10.1007/s00520-020-05820-3

Xu FK, 2020, CELL COMMUN SIGNAL, V18, DOI 10.1186/s12964-020-0510-8

You M, 2017, MOL ORAL MICROBIOL, V32, P455, DOI 10.1111/omi.12185

Yue LD, 2021, OXID MED CELL LONGEV, V2021, DOI 10.1155/2021/6633419

Zhang T, 2021, APPL MICROBIOL BIOT, V105, P5785, DOI 10.1007/s00253-021-11453-1

Zhang T, 2019, MICROB BIOTECHNOL, V12, P1109, DOI 10.1111/1751-7915.13410

Zhao C, 2019, CHEMOSPHERE, V235, P288, DOI 10.1016/j.chemosphere.2019.06.124

Zhong L, 2011, ASIAN PAC J CANCER P, V12, P1771

NR 60

TC 0

Z9 0

U1 2

U2 2

PU SPRINGER

PI NEW YORK

PA ONE NEW YORK PLAZA, SUITE 4600, NEW YORK, NY, UNITED STATES

SN 0175-7598

EI 1432-0614

J9 APPL MICROBIOL BIOT

JI Appl. Microbiol. Biotechnol.

PD APR

PY 2022

VL 106

IS 8

BP 3215

EP 3229

DI 10.1007/s00253-022-11921-2

EA APR 2022

PG 15

WC Biotechnology & Applied Microbiology

WE Science Citation Index Expanded (SCI-EXPANDED)

SC Biotechnology & Applied Microbiology

GA 0Y3GY

UT WOS:000784385900007

PM 35435458

DA 2022-05-19

ER

PT J

AU Lubin, DJ

Mick, R

Shroff, SG

Stashek, K

Furth, EE

AF Lubin, Daniel J.

Mick, Rosemarie

Shroff, Stuti G.

Stashek, Kristen

Furth, Emma E.

TI The notch pathway is activated in neoplastic progression in esophageal

squamous cell carcinoma

SO HUMAN PATHOLOGY

LA English

DT Article

DE Surgical pathology; Notch signaling; Esophageal squamous cell carcinoma;

Carcinogenesis; Overall survival

ID CHROMOSOMAL TRANSLOCATIONS; LYMPHOBLASTIC NEOPLASMS; GENE;

ADENOCARCINOMA; CANCER

AB The Notch signaling pathway is integral to normal human development and homeostasis and has a deterministic function on cell differentiation. Recent studies suggest aberrant Notch signaling may contribute to neoplastic progression by an increase in stem cell survival, chemoresistance, and the promotion of epithelial-to-mesenchymal transition. The goals of our study were to determine, utilizing quantitative technologies, the expression of activated Notch 1 in esophageal squamous cell carcinoma (SCC) and to determine the relationship between Notch 1 expression and various clinicopathologic parameters. Immunohistochemical staining for Notch intracellular domain (NICD) was performed on 60 consecutive cases of esophageal squamous cell carcinoma, 42 cases of benign esophageal squamous epithelium, and 13 cases of eosinophilic esophagitis diagnosed in our department from 2007 through 2015, and exact nuclear staining and nuclear characteristics were graded using the Vectra imaging system. Clinicopathologic data (gender, age at diagnosis, smoking status, tumor grade, tumor stage, tumor location, and survival) were collected for each SCC case and these were correlated with NICD staining. Cases of esophageal SCC demonstrated significantly higher NICD staining compared to cases of benign and reactive esophageal epithelium (P = .003 and .005, respectively). Among cases of esophageal SCC, nuclear NICD staining was significantly correlated with both tumor grade and stage. Following classification and regression tree analysis, esophageal SCC patients with increased NICD expression were found to be more likely to die from their disease than those with lower levels of expression. Taken together, the findings suggest that increased Notch 1 may contribute to the development and aggressiveness of esophageal SCC. (C) 2017 Elsevier Inc. All rights reserved.

C1 [Lubin, Daniel J.; Shroff, Stuti G.; Stashek, Kristen; Furth, Emma E.] Hosp Univ Penn, Dept Pathol, Philadelphia, PA 19104 USA.

[Mick, Rosemarie] Univ Penn, Perelman Sch Med, Dept Biostat & Epidemiol, Philadelphia, PA 19104 USA.

RP Lubin, DJ (corresponding author), Hosp Univ Penn, 6 Founders,3400 Spruce St, Philadelphia, PA 19104 USA.

EM Daniel.Lubin@uphs.upenn.edu

OI Lubin, Danny/0000-0002-0409-2374

CR Agrawal N, 2012, CANCER DISCOV, V2, P899, DOI 10.1158/2159-8290.CD-12-0189

Balint K, 2005, J CLIN INVEST, V115, P3166, DOI 10.1172/JCI25001

ELLISEN LW, 1991, CELL, V66, P649, DOI 10.1016/0092-8674(91)90111-B

Forghanifard MM, 2012, ANN SURG ONCOL, V19, P743, DOI 10.1245/s10434-011-2074-8

Gao YB, 2014, NAT GENET, V46, P1097, DOI 10.1038/ng.3076

Huang JY, 2013, J EXP CLIN CANC RES, V32, DOI 10.1186/1756-9966-32-84

Morgan TH, 1917, AM NAT, V51, P513, DOI 10.1086/279629

Park JT, 2010, AM J PATHOL, V177, P1087, DOI 10.2353/ajpath.2010.100316

Penton AL, 2012, SEMIN CELL DEV BIOL, V23, P450, DOI 10.1016/j.semcdb.2012.01.010

Poulson DF, 1937, P NATL ACAD SCI USA, V23, P133, DOI 10.1073/pnas.23.3.133

Proweller A, 2006, CANCER RES, V66, P7438, DOI 10.1158/0008-5472.CAN-06-0793

REYNOLDS TC, 1987, CELL, V50, P107, DOI 10.1016/0092-8674(87)90667-2

Taleb Shaghayegh, 2014, J Gastrointest Cancer, V45, P466, DOI 10.1007/s12029-014-9648-1

Wang T, 2013, TUMOUR BIOL J INT SO, V35, P1193

Westhoff B, 2009, P NATL ACAD SCI USA, V106, P22293, DOI 10.1073/pnas.0907781106

NR 15

TC 13

Z9 16

U1 0

U2 5

PU W B SAUNDERS CO-ELSEVIER INC

PI PHILADELPHIA

PA 1600 JOHN F KENNEDY BOULEVARD, STE 1800, PHILADELPHIA, PA 19103-2899 USA

SN 0046-8177

EI 1532-8392

J9 HUM PATHOL

JI Hum. Pathol.

PD FEB

PY 2018

VL 72

BP 66

EP 70

DI 10.1016/j.humpath.2017.11.004

PG 5

WC Pathology

WE Science Citation Index Expanded (SCI-EXPANDED)

SC Pathology

GA FX4EC

UT WOS:000426024500008

PM 29137934

DA 2022-05-19

ER

PT J

AU Suzuki, T

Yajima, S

Ishioka, N

Nanami, T

Oshima, Y

Washizawa, N

Funahashi, K

Otsuka, S

Nemoto, T

Shimada, H

AF Suzuki, Takashi

Yajima, Satoshi

Ishioka, Nobuki

Nanami, Tatsuki

Oshima, Yoko

Washizawa, Naohiro

Funahashi, Kimihiko

Otsuka, Seiko

Nemoto, Tetsuo

Shimada, Hideaki

TI Prognostic significance of high serum p53 antibody titers in patients

with esophageal squamous cell carcinoma

SO ESOPHAGUS

LA English

DT Article

DE Serum p53 antibody; Serum titer; Esophageal squamous cell carcinoma;

Prognosis

ID NEOADJUVANT CHEMOTHERAPY; CANCER; RECURRENCE; PREDICTOR; SURVIVAL

AB Background The p53 protein overexpression that usually results from genetic alterations reportedly induces serum antibodies against p53. However, little information is available about the prognostic significance of perioperative serum p53 antibody (s-p53-Abs) titers in patients with esophageal squamous cell carcinoma.

Methods In this study, we retrospectively evaluated the clinical significance of perioperative s-p53-Abs in 135 patients with esophageal squamous cell carcinoma. Of these, 58 patients received neoadjuvant chemotherapy comprising 5-FU and CDDP. While the cutoff level at 1.3 U/ml indicated seropositive patients, level of 13.4 U/ml was used to identify high-titer patients. We monitored serum titers seropositive patients after surgery and evaluated the prognostic significance by the univariate and multivariate analyses.

Results In this study, 29 patients (21.5%) were positive for s-p53-Abs before treatment. The frequency of both seropositive patients and high-titer patients (> 13.4 U/ml) was not significantly associated with tumor progression. While seropositive patients did not demonstrate significant poor overall survival, high-titer patients demonstrated significant poor overall survival based on the multivariate analysis (P < 0.001). Moreover, the s-p53-Abs titer did not correlate with the response to neoadjuvant chemotherapy. Among seropositive patients, the negative conversion of s-p53-Abs more likely led to be long-term survival.

Conclusions This study determined that the high-titer of s-p53-Abs was an independent risk factor to reduce the overall survival of patients with esophageal cancer patients. The negative conversion of s-p53-Abs could be a good indicator of favorable prognosis.

C1 [Suzuki, Takashi; Yajima, Satoshi; Ishioka, Nobuki; Nanami, Tatsuki; Oshima, Yoko; Washizawa, Naohiro; Funahashi, Kimihiko; Otsuka, Seiko; Shimada, Hideaki] Toho Univ, Sch Med, Dept Surg, Ota Ku, 6-11-1 Omori Nishi, Tokyo 1428541, Japan.

[Ishioka, Nobuki; Shimada, Hideaki] Toho Univ, Grad Sch Med, Dept Clin Oncol, Tokyo, Japan.

[Nemoto, Tetsuo] Toho Univ, Sch Med, Dept Pathol, Tokyo, Japan.

RP Shimada, H (corresponding author), Toho Univ, Sch Med, Dept Surg, Ota Ku, 6-11-1 Omori Nishi, Tokyo 1428541, Japan.; Shimada, H (corresponding author), Toho Univ, Grad Sch Med, Dept Clin Oncol, Tokyo, Japan.

EM hideaki.shimada@med.toho-u.ac.jp

RI Oshima, Yoko/AGP-3054-2022; Shimada, Hideaki/H-9313-2019

OI Shimada, Hideaki/0000-0002-1990-8217

FU JSPS KAKENHI [JP16K10520]

FX This work was supported by JSPS KAKENHI grant number JP16K10520.

CR Ando N, 2012, ANN SURG ONCOL, V19, P68, DOI 10.1245/s10434-011-2049-9

Hiyoshi Y, 2017, WORLD J SURG, V41, P480, DOI 10.1007/s00268-016-3649-0

Kanda Y, 2013, BONE MARROW TRANSPL, V48, P452, DOI 10.1038/bmt.2012.244

Kubota Y, 2017, TOHO J MED, V3, P58

Makino T, 2010, ANN SURG ONCOL, V17, P804, DOI 10.1245/s10434-009-0786-9

Okumura H, 2014, SURG TODAY, V44, P421, DOI 10.1007/s00595-013-0580-y

Saito F, 2017, ESOPHAGUS-TOKYO, V14, P366, DOI 10.1007/s10388-016-0564-2

Shimada H, 2003, SURGERY, V133, P24, DOI 10.1067/msy.2003.31

Shimada H, 2002, SURGERY, V132, P41, DOI 10.1067/msy.2002.125307

Shimada H, 2001, SURG TODAY, V31, P591, DOI 10.1007/s005950170092

Shimada H, 2008, J AM COLL SURGEONS, V206, P48, DOI 10.1016/j.jamcollsurg.2007.06.013

Shimada H, 2009, WORLD J SURG, V33, P272, DOI 10.1007/s00268-008-9821-4

Sobin L. H., 2009, UICC TNM CLASSIFICAT

Takahashi K, 2007, DIS ESOPHAGUS, V20, P117, DOI 10.1111/j.1442-2050.2007.00656.x

Yamasaki M, 2010, ANN SURG ONCOL, V17, P634, DOI 10.1245/s10434-009-0851-4

Yamashita K, 2017, WORLD J SURG, V41, P1566, DOI 10.1007/s00268-017-3894-x

NR 16

TC 13

Z9 15

U1 0

U2 1

PU SPRINGER JAPAN KK

PI TOKYO

PA CHIYODA FIRST BLDG EAST, 3-8-1 NISHI-KANDA, CHIYODA-KU, TOKYO, 101-0065,

JAPAN

SN 1612-9059

EI 1612-9067

J9 ESOPHAGUS-TOKYO

JI Esophagus

PD OCT

PY 2018

VL 15

IS 4

BP 294

EP 300

DI 10.1007/s10388-018-0629-5

PG 7

WC Gastroenterology & Hepatology

WE Science Citation Index Expanded (SCI-EXPANDED)

SC Gastroenterology & Hepatology

GA GT7RC

UT WOS:000444725300012

PM 29959634

DA 2022-05-19

ER

PT J

AU Qiu, B

Li, JX

Wang, B

Wang, ZQ

Liang, Y

Cai, PQ

Chen, ZL

Liu, MZ

Fu, JH

Yang, H

Liu, H

AF Qiu, Bo

Li, JiaXiang

Wang, Bin

Wang, ZhiQiang

Liang, Ying

Cai, Peiqiang

Chen, ZhaoLin

Liu, MengZhong

Fu, JianHua

Yang, Hong

Liu, Hui

TI Adjuvant Therapy for a Microscopically Incomplete Resection Margin after

an Esophagectomy for Esophageal Squamous Cell Carcinoma

SO JOURNAL OF CANCER

LA English

DT Article

DE Esophageal squamous cell carcinoma; R1 resection; adjuvant chemotherapy;

adjuvant radiotherapy; prognosis

ID THORACIC ESOPHAGUS; POSTOPERATIVE RADIATION; PROGNOSTIC-SIGNIFICANCE;

MULTIVARIATE-ANALYSIS; CANCER STATISTICS; CHEMOTHERAPY; RADIOTHERAPY;

SURVIVAL; SURGERY; RECURRENCE

AB Purpose: To investigate the prognosis of esophageal squamous cell carcinoma with a microscopically incomplete (R1) resection margin following an esophagectomy, as well as the impact of adjuvant treatment on survival.

Methods: Data obtained from 124 patients with R1-resected ESCC were reviewed. The impact of clinicopathological factors and adjuvant treatment on the overall survival, locoregional recurrence, and distant recurrence were explored.

Results: For a median follow-up time of 16.8 months, the median overall survival of 124 patients was 25.6 months. The 1, 3, and 5-year overall survival rates were 75.6%+/- 4.0%, 35.9%+/- 5.1%, and 23.2%+/- 5.0%, respectively. Adjuvant therapy was administered in 78 patients. In the univariate analyses, patients with a pN0 stage (log rank, p=0.028) and adjuvant chemotherapy (log rank, p=0.032) exhibited more favorable overall survival. In the multivariate analyses, the pN stage (HR=2.192, p=0.004) and adjuvant chemotherapy (HR=0.032, p=0.004) were independent prognostic factors for overall survival. Locoregional recurrence was the main failure pattern after R1 resection. The pN stage (HR=2.567, p=0.009) and adjuvant radiotherapy (HR=0.278, p=0.000) were independent prognostic factors for locoregional recurrence.

Conclusion: In R1-resected esophageal squamous cell carcinoma, adjuvant radiotherapy reduced locoregional recurrence; however, it did not improve overall survival. Adjuvant chemotherapy demonstrated benefits for overall survival. The pN stage was an independent prognostic factor for locoregional recurrence and overall survival.

C1 [Qiu, Bo; Wang, Bin; Wang, ZhiQiang; Liang, Ying; Cai, Peiqiang; Chen, ZhaoLin; Liu, MengZhong; Fu, JianHua; Yang, Hong; Liu, Hui] State Key Lab Oncol South China, Guangzhou, Guangdong, Peoples R China.

[Qiu, Bo; Wang, Bin; Wang, ZhiQiang; Liang, Ying; Cai, Peiqiang; Chen, ZhaoLin; Liu, MengZhong; Fu, JianHua; Yang, Hong; Liu, Hui] Guangdong Esophageal Canc Res Inst, Guangzhou, Guangdong, Peoples R China.

[Qiu, Bo; Chen, ZhaoLin; Liu, MengZhong; Liu, Hui] Sun Yat Sen Univ, Ctr Canc, Dept Radiat Oncol, 651 Dongfengdong Rd, Guangzhou 510060, Guangdong, Peoples R China.

[Wang, ZhiQiang; Liang, Ying] Sun Yat Sen Univ, Ctr Canc, Dept Med Oncol, Guangzhou, Guangdong, Peoples R China.

[Cai, Peiqiang] Sun Yat Sen Univ, Ctr Canc, Dept Med Imaging, Guangzhou, Guangdong, Peoples R China.

[Fu, JianHua; Yang, Hong] Sun Yat Sen Univ, Ctr Canc, Dept Thorac Oncol, Guangzhou, Guangdong, Peoples R China.

[Li, JiaXiang] First Peoples Hosp Zhaoqing City, Dept Oncol, Zhaoqing, Guangdong, Peoples R China.

RP Yang, H; Liu, H (corresponding author), State Key Lab Oncol South China, Guangzhou, Guangdong, Peoples R China.; Yang, H; Liu, H (corresponding author), Guangdong Esophageal Canc Res Inst, Guangzhou, Guangdong, Peoples R China.; Liu, H (corresponding author), Sun Yat Sen Univ, Ctr Canc, Dept Radiat Oncol, 651 Dongfengdong Rd, Guangzhou 510060, Guangdong, Peoples R China.; Yang, H (corresponding author), Sun Yat Sen Univ, Ctr Canc, Dept Thorac Oncol, Guangzhou, Guangdong, Peoples R China.

EM liuhui@sysucc.org.cn

CR Ando N, 2003, J CLIN ONCOL, V21, P4592, DOI 10.1200/JCO.2003.12.095

Ando N, 1997, J THORAC CARDIOV SUR, V114, P205, DOI 10.1016/S0022-5223(97)70146-6

Bao Y, 2013, RADIAT ONCOL, V8, DOI 10.1186/1748-717X-8-241

Chen G, 2009, ANN THORAC SURG, V87, P1697, DOI 10.1016/j.athoracsur.2009.03.060

Chen WQ, 2016, CA-CANCER J CLIN, V66, P115, DOI 10.3322/caac.21338

Debove C, 2015, COLORECTAL DIS, V17, P197, DOI 10.1111/codi.12849

Deeter M, 2009, ARCH SURG-CHICAGO, V144, P618, DOI 10.1001/archsurg.2009.115

Gilbert S, 2015, J THORAC CARDIOV SUR, V149, P548, DOI 10.1016/j.jtcvs.2014.10.040

Kelsen DP, 1998, NEW ENGL J MED, V339, P1979, DOI 10.1056/NEJM199812313392704

Lad NL, 2013, ANN SURG ONCOL, V20, P3626, DOI 10.1245/s10434-013-3080-9

Mariette C, 2004, ANN THORAC SURG, V78, P1177, DOI 10.1016/j.athoracsur.2004.02.068

Mirnezami R, 2010, INT J SURG, V8, P58, DOI 10.1016/j.ijsu.2009.11.001

Munasinghe A, 2015, ANN SURG, V262, P79, DOI 10.1097/SLA.0000000000000805

Nakagawa S, 2004, J AM COLL SURGEONS, V198, P205, DOI 10.1016/j.jamcollsurg.2003.10.005

Riquet M, 2010, ANN THORAC SURG, V89, P870, DOI 10.1016/j.athoracsur.2009.11.052

Schreiber D, 2010, J THORAC ONCOL, V5, P244, DOI 10.1097/JTO.0b013e3181c5e34f

Shapiro J, 2015, LANCET ONCOL, V16, P1090, DOI 10.1016/S1470-2045(15)00040-6

Siegel R, 2012, CA-CANCER J CLIN, V62, P10, DOI 10.3322/caac.20138

TENIERE P, 1991, SURG GYNECOL OBSTET, V173, P123

van Hagen P, 2012, NEW ENGL J MED, V366, P2074, DOI 10.1056/NEJMoa1112088

Wu J, 2014, ANN THORAC SURG, V97, P446, DOI 10.1016/j.athoracsur.2013.10.043

Xiao ZF, 2005, INT J RADIAT ONCOL, V62, P82, DOI 10.1016/j.ijrobp.2004.08.046

Xiao ZF, 2003, ANN THORAC SURG, V75, P331, DOI 10.1016/S0003-4975(02)04401-6

Zafirellis K, 2002, DIS ESOPHAGUS, V15, P155, DOI 10.1046/j.1442-2050.2002.00230.x

NR 24

TC 9

Z9 9

U1 0

U2 5

PU IVYSPRING INT PUBL

PI LAKE HAVEN

PA PO BOX 4546, LAKE HAVEN, NSW 2263, AUSTRALIA

SN 1837-9664

J9 J CANCER

JI J. Cancer

PY 2017

VL 8

IS 2

BP 249

EP 257

DI 10.7150/jca.16409

PG 9

WC Oncology

WE Science Citation Index Expanded (SCI-EXPANDED)

SC Oncology

GA EO3EU

UT WOS:000396578500011

PM 28243329

OA Green Published, Green Submitted, gold

DA 2022-05-19

ER

PT J

AU Huang, CY

Wang, L

Yang, XB

Lai, L

Chen, D

Duan, CY

AF Huang, Chuying

Wang, Li

Yang, Xibiao

Lai, Lin

Chen, Dian

Duan, Chunyan

TI Expression of activated signal transducer and activator of

transcription-3 as a predictive and prognostic marker in advanced

esophageal squamous cell carcinoma

SO WORLD JOURNAL OF SURGICAL ONCOLOGY

LA English

DT Article

DE Esophageal squamous cell carcinoma; p-Stat3 expression; Chemotherapy

ID STAT3; CANCER; DOCETAXEL; ADENOCARCINOMA; ANGIOGENESIS; FLUOROURACIL;

CHEMOTHERAPY; CISPLATIN; RESECTION; PROTEINS

AB Background: Signal transducer and activator of transcription-3 (STAT3) is an oncogenic transcription factor constitutively active and aberrantly expressed in various types of malignancies, and the expression of p-STAT3 has been recognized as a predictor of poor survival. It remains unclear how variations in p-STAT3 expression influence clinical outcomes in esophageal squamous cell carcinoma (ESCC).

Methods: Between 1 January 2008 and 1 November 2013, 153 advanced esophageal squamous cell carcinoma patients (stage IV) from two cancer centers in West China were treated with paclitaxel and cisplatin. We retrospectively analyzed the clinical outcomes of patients with ESCC and examined the correlation between p-STAT3 levels and clinical outcomes in esophageal cancer patients.

Results: Among the 153 patients, positive p-STAT3 expression was observed in 73 of 153 (47.7 %) cases. The median PFS for patients with positive expression of p-STAT3 and negative expression of p-STAT3 was 5.0 months and 6.9 months, respectively (P < 0.001). The median overall survival was significantly higher in patients with p-STAT3 negative tumors than in those with p-STAT3 positive tumors (9.9 vs 8.9 months, P = 0.026). Kaplan-Meier survival analysis showed that p-STAT3 expression was statistically indicative of a poor prognosis for progression-free survival.

Conclusions: These data showed that p-STAT3 expression was significantly associated with poor prognosis in patients with esophageal cancer and could be used as a predictive and prognostic marker in esophageal cancer.

C1 [Huang, Chuying] Hubei Inst Nationalities, Affiliated Hosp, Dept Respirat, Enshi, Hubei Province, Peoples R China.

[Huang, Chuying; Lai, Lin; Chen, Dian; Duan, Chunyan] Enshi Tujia & Miao Autonomous Prefecture Cent Hos, Dept Med Oncol, Enshi, Hubei Province, Peoples R China.

[Wang, Li] Enshi Tujia & Miao Autonomous Prefecture Cent Hos, Dept Dermatol, Enshi, Hubei Province, Peoples R China.

[Yang, Xibiao] Sichuan Univ, West China Hosp, Dept Radiol, Chengdu 610064, Sichuan, Peoples R China.

RP Duan, CY (corresponding author), Enshi Tujia & Miao Autonomous Prefecture Cent Hos, Dept Med Oncol, 158 Wu Yang Ave, Enshi, Hubei Province, Peoples R China.

EM 724249691@qq.com

CR Ajani JA, 2005, J CLIN ONCOL, V23, P5660, DOI 10.1200/JCO.2005.17.376

Allum WH, 2009, J CLIN ONCOL, V27, P5062, DOI 10.1200/JCO.2009.22.2083

Benekli M, 2003, BLOOD, V101, P2940, DOI 10.1182/blood-2002-04-1204

Burtness B, 2009, ANN ONCOL, V20, P1242, DOI 10.1093/annonc/mdn787

Campbell CL, 2001, AM J PATHOL, V158, P25, DOI 10.1016/S0002-9440(10)63940-5

Chen M., 2013, MATH PROBL ENG, V2013, P1

Chen MF, 2013, MOL CANCER, V12, DOI 10.1186/1476-4598-12-26

Chen W, 2012, LIVER INT, V32, P70, DOI 10.1111/j.1478-3231.2011.02631.x

Cortas T, 2007, LUNG CANCER, V55, P349, DOI 10.1016/j.lungcan.2006.11.003

DARNELL JE, 1994, SCIENCE, V264, P1415, DOI 10.1126/science.8197455

Feng XS, 2014, ASIAN PAC J CANCER P, V15, P1981, DOI 10.7314/APJCP.2014.15.5.1981

Huang M, 2000, GYNECOL ONCOL, V79, P67, DOI 10.1006/gyno.2000.5931

Hulscher JBF, 2002, NEW ENGL J MED, V347, P1662, DOI 10.1056/NEJMoa022343

Li HY, 2014, INT J CLIN EXP PATHO, V7, P7989

Li R, 2013, PLOS ONE, V8, DOI 10.1371/journal.pone.0074670

Niu GL, 2002, ONCOGENE, V21, P2000, DOI 10.1038/sj.onc.1205260

Real PJ, 2002, ONCOGENE, V21, P7611, DOI 10.1038/sj.onc.1206004

Scholz A, 2003, GASTROENTEROLOGY, V125, P891, DOI 10.1016/S0016-5085(03)01064-3

Sjoquist KM, 2011, LANCET ONCOL, V12, P681, DOI 10.1016/S1470-2045(11)70142-5

Song JI, 2000, ONCOGENE, V19, P2489, DOI 10.1038/sj.onc.1203483

Van Cutsem E, 2006, J CLIN ONCOL, V24, P4991, DOI 10.1200/JCO.2006.06.8429

Wang TH, 2004, NAT MED, V10, P48, DOI 10.1038/nm976

Wei LH, 2003, ONCOGENE, V22, P1517, DOI 10.1038/sj.onc.1206226

Xuan XY, 2015, MOL BIOL REP, V42, P907, DOI 10.1007/s11033-014-3828-8

NR 24

TC 7

Z9 7

U1 0

U2 3

PU BIOMED CENTRAL LTD

PI LONDON

PA 236 GRAYS INN RD, FLOOR 6, LONDON WC1X 8HL, ENGLAND

SN 1477-7819

J9 WORLD J SURG ONCOL

JI World J. Surg. Oncol.

PD NOV 9

PY 2015

VL 13

AR 314

DI 10.1186/s12957-015-0726-z

PG 7

WC Oncology; Surgery

WE Science Citation Index Expanded (SCI-EXPANDED)

SC Oncology; Surgery

GA CV8UQ

UT WOS:000364563900001

PM 26553224

OA gold, Green Published

DA 2022-05-19

ER

PT J

AU Xu, YY

Peng, Z

Li, ZW

Lu, M

Gao, J

Li, YL

Li, YY

Shen, L

AF Xu, Yingying

Peng, Zhi

Li, Zhongwu

Lu, Ming

Gao, Jing

Li, Yilin

Li, Yanyan

Shen, Lin

TI Expression and clinical significance of c-Met in advanced esophageal

squamous cell carcinoma

SO BMC CANCER

LA English

DT Article

DE Esophageal squamous cell carcinoma; c-Met; Chemotherapy; Overall

survival

ID RECEPTOR; RESISTANCE; CANCER; AMPLIFICATION; OVEREXPRESSION; ACTIVATION;

HGF/SF; GROWTH; TRIAL; GENE

AB Background: c-Met, one of current potential hot targets, has been suggested as a potential tumor marker in the development of esophageal squamous cell carcinoma (ESCC). Our aim was to investigate the expression of c-Met in advanced esophageal squamous cell carcinoma in four phase II trials who had tumor tissues from archival in our center and analyze the correlations between c-Met expression and clinical features.

Methods: Ninety patients with advanced ESCC who were admitted to the phase II clinical trials in the Department of Gastrointestinal Oncology, Peking University Cancer Hospital and Institute from March 2007 to March 2014 were finally eligible for present study and the corresponding tissues and clinical data were collected. The expression of c-Met in the tissue samples was detected by immunohistochemistry (IHC). c-Met overexpression was defined as >= the median value of H-score. Kaplan-Meier and Cox multivariate regression were conducted to evaluate the relationship between c-Met expression and ESCC survival.

Results: The overexpression of c-Met is 43.3% in advanced ESCC. There was no statistical difference between c-Met expression and clinical features except sex and tumor location. Survival analysis documented that the overexpression of c-Met predicted a worse prognosis (OS: 253 d vs 422 d, P = 0.011). In the group treated with chemotherapy combined with anti-EGFR drugs, patients with lowexpression of c-Met had a better OS than those with overexpression of c-Met (OS: 577 d vs 232 d, P = 0.007).

Conclusions: c-Met may be an independent prognostic factor in advanced ESCC. The overexpression of c-Met may predict a worse efficacy of anti-EGFR therapy.

C1 [Xu, Yingying; Peng, Zhi; Li, Zhongwu; Lu, Ming; Gao, Jing; Li, Yilin; Li, Yanyan; Shen, Lin] Peking Univ, Canc Hosp & Inst, Dept Gastrointestinal Oncol, Key Lab Carcinogenesis & Translat Res,Minist Educ, Beijing 100871, Peoples R China.

[Li, Zhongwu] Peking Univ, Canc Hosp & Inst, Dept Pathol, Key Lab Carcinogenesis & Translat Res,Minist Educ, Beijing 100871, Peoples R China.

RP Shen, L (corresponding author), Peking Univ, Canc Hosp & Inst, Dept Gastrointestinal Oncol, Key Lab Carcinogenesis & Translat Res,Minist Educ, FuCheng Rd 52, Beijing 100871, Peoples R China.

EM lin100@medmail.com.cn

RI Li, Yilin/AAW-9533-2021

FU National Natural Science Foundation of China [81172110]; National High

Technology Research and Development Program [2012AA02A504]; Beijing

Municipal Science & Technology Commission Program [Z11110706730000];

Beijing Natural Science Foundation [7142034]; China Postdoctoral Science

Foundation [2013 M530494]

FX This work was supported by National Natural Science Foundation of China

(No. 81172110), National High Technology Research and Development

Program (No. 2012AA02A504), Beijing Municipal Science & Technology

Commission Program (No. Z11110706730000), Beijing Natural Science

Foundation (7142034), and China Postdoctoral Science Foundation (2013

M530494).

CR Andermarcher E, 1996, DEV GENET, V18, P254, DOI 10.1002/(SICI)1520-6408(1996)18:3<254::AID-DVG6>3.0.CO;2-8

Anderson MR, 2006, CLIN CANCER RES, V12, P5936, DOI 10.1158/1078-0432.CCR-06-1208

Bardelli A, 2013, CANCER DISCOV, V3, P658, DOI 10.1158/2159-8290.CD-12-0558

BLADT F, 1995, NATURE, V376, P768, DOI 10.1038/376768a0

Boccaccio C, 2006, NAT REV CANCER, V6, P637, DOI 10.1038/nrc1912

Chen CT, 2012, MOL CANCER THER, V11, P660, DOI 10.1158/1535-7163.MCT-11-0754

Comoglio PM, 2008, NAT REV DRUG DISCOV, V7, P504, DOI 10.1038/nrd2530

DIRENZO MF, 1995, CLIN CANCER RES, V1, P147

Dong G, 2004, ONCOGENE, V23, P6199, DOI 10.1038/sj.onc.1207851

Engelman JA, 2007, SCIENCE, V316, P1039, DOI 10.1126/science.1141478

Jemal A, 2011, CA-CANCER J CLIN, V61, P69, DOI [10.3322/caac.20107, 10.3322/caac.20115]

Lee JH, 2000, ONCOGENE, V19, P4947, DOI 10.1038/sj.onc.1203874

Mesteri I, 2014, EUR J CANCER, V50, P1354, DOI 10.1016/j.ejca.2014.01.022

Minuti G, 2012, BRIT J CANCER, V107, P793, DOI 10.1038/bjc.2012.335

Peng Z, 2014, PLOS ONE, V9, DOI [10.1371/journal.pone.0084502, 10.1371/journal.pone.0092924, 10.1371/journal.pone.0085663]

[秦艳茹 QIN Yanru], 2006, [郑州大学学报. 医学版, Journal of Zhengzhou University.Medical Sciences], V41, P34

Shattuck DL, 2008, CANCER RES, V68, P1471, DOI 10.1158/0008-5472.CAN-07-5962

Spigel DR, 2012, CLIN LUNG CANCER, V13, P500, DOI 10.1016/j.cllc.2012.05.009

STREIT A, 1995, DEVELOPMENT, V121, P813

Zhang XD, 2008, AM J CLIN ONCOL-CANC, V31, P29, DOI 10.1097/COC.0b013e3181131ca9

NR 20

TC 24

Z9 27

U1 0

U2 9

PU BMC

PI LONDON

PA CAMPUS, 4 CRINAN ST, LONDON N1 9XW, ENGLAND

EI 1471-2407

J9 BMC CANCER

JI BMC Cancer

PD JAN 15

PY 2015

VL 15

AR 6

DI 10.1186/s12885-014-1001-3

PG 7

WC Oncology

WE Science Citation Index Expanded (SCI-EXPANDED)

SC Oncology

GA CA3MZ

UT WOS:000348812200003

PM 25588551

OA Green Published, gold

DA 2022-05-19

ER

PT J

AU Cui, F

Wu, DG

He, XT

Wang, WJ

Xi, JL

Wang, MH

AF Cui, Fei

Wu, Duoguang

He, Xiaotian

Wang, Wenjian

Xi, Jingle

Wang, Minghui

TI Long noncoding RNA SPRY4-IT1 promotes esophageal squamous cell carcinoma

cell proliferation, invasion, and epithelial-mesenchymal transition

SO TUMOR BIOLOGY

LA English

DT Article

DE SPRY4-IT1; Long noncoding RNA; Esophageal squamous cell carcinoma; EMT

ID GASTRIC-CANCER; METASTASIS

AB The biology of esophageal squamous cell carcinoma (ESCC) remains poorly understood. Long noncoding RNAs (lncRNAs) are found to be dysregulated in a variety of cancers, including ESCC. SPRY4-IT1 has been recently revealed as oncogenic regulator or tumor suppressors in different cancers; however, whether SPRY4-IT1 is involved in ESCC remains poorly understood. To investigate the role of SPRY4-IT1 in ESCC, we evaluated the SPRY4-IT1 expression levels in a series of ESCC patients and a panel of ESCC cell line using qRT-PCR. CCK8 and colony formation assay were performed to assess the effect of SPRY4-IT1siRNA on cell proliferation, migration, and invasion of ESCC cell lines. SPRY4-IT1 expression was upregulated in ESCC tissues and the higher expression of SPRY4-IT1 was significantly correlated with tumor grade, depth of invasion, and lymph node metastasis. Moreover, silencing of SPRY4-IT1 expression inhibited ESCC cell proliferation, colony formation, migration, and invasion. Therefore, our study indicates that SPRY4-IT1 promotes proliferation and migration of ESCC cells and is a potential oncogene of ESCC.

C1 [Cui, Fei; Xi, Jingle] Southern Med Univ, Nanfang Hosp, Dept Oncol, Guangzhou 510515, Guangdong, Peoples R China.

[Wu, Duoguang; He, Xiaotian; Wang, Wenjian; Wang, Minghui] Sun Yat Sen Univ, Sun Yat Sen Mem Hosp, Dept Thorac Surg, Guangdong Prov Key Lab Malignant Tumor Epigenet &, Guangzhou 510120, Guangdong, Peoples R China.

RP Wang, MH (corresponding author), Sun Yat Sen Univ, Sun Yat Sen Mem Hosp, Dept Thorac Surg, Guangdong Prov Key Lab Malignant Tumor Epigenet &, Guangzhou 510120, Guangdong, Peoples R China.

EM wangmh6767@163.com

FU Key Laboratory of Malignant Tumor Molecular Mechanism and Translational

Medicine of Guangzhou Bureau of Science and information Technology

[[2013] 163]; Key Laboratory of Malignant Tumor Gene Regulation and

Target Therapy of Guangdong Higher Education institutes [KLB09001];

National Natural Science Foundation of China [81372567]; Natural Science

Foundation of Guangdong Province, China [2014A030310450]; Director of

Nanfang Hospital Foundation [2013B018]

FX This work was supported by Grant [2013] 163 from the Key Laboratory of

Malignant Tumor Molecular Mechanism and Translational Medicine of

Guangzhou Bureau of Science and information Technology; Grant KLB09001

from the Key Laboratory of Malignant Tumor Gene Regulation and Target

Therapy of Guangdong Higher Education institutes, National Natural

Science Foundation of China grant (No. 81372567), the Natural Science

Foundation of Guangdong Province, China (No. 2014A030310450), and

Director of Nanfang Hospital Foundation (No. 2013B018)

CR Chen FJ, 2013, MOL CARCINOGEN, V52, P908, DOI 10.1002/mc.21944

Guohong Zhang, 2010, PLoS One, V5, pe9668, DOI 10.1371/journal.pone.0009668

Gupta RA, 2010, NATURE, V464, P1071, DOI 10.1038/nature08975

Guttman M, 2011, NATURE, V477, P295, DOI 10.1038/nature10398

Khaitan D, 2011, CANCER RES, V71, P3852, DOI 10.1158/0008-5472.CAN-10-4460

Matsushima K, 2011, J TRANSL MED, V9, DOI 10.1186/1479-5876-9-30

Matsushima K, 2010, DIGESTION, V82, P138, DOI 10.1159/000310918

Mazar J, 2014, ONCOTARGET, V5, P8959, DOI 10.18632/oncotarget.1863

Nagaraja V, 2014, J GASTROINTEST ONCOL, V5, P67, DOI 10.3978/j.issn.2078-6891.2013.054

Peng W, 2015, TUMOR BIOL, V36, P6751, DOI 10.1007/s13277-015-3376-4

Qiu MT, 2013, TUMOR BIOL, V34, P613, DOI 10.1007/s13277-013-0658-6

Sun M, 2014, CELL DEATH DIS, V5, DOI 10.1038/cddis.2014.256

Tsai MC, 2010, SCIENCE, V329, P689, DOI 10.1126/science.1192002

Wang KC, 2011, MOL CELL, V43, P904, DOI 10.1016/j.molcel.2011.08.018

Wu JX, 2014, TUMOR BIOL, V35, P3709, DOI 10.1007/s13277-013-1490-8

Xie M, 2015, J TRANSL MED, V13, DOI 10.1186/s12967-015-0595-9

Zou YF, 2013, PLOS ONE, V8, DOI 10.1371/journal.pone.0079598

NR 17

TC 33

Z9 34

U1 1

U2 12

PU SPRINGER

PI DORDRECHT

PA VAN GODEWIJCKSTRAAT 30, 3311 GZ DORDRECHT, NETHERLANDS

SN 1010-4283

EI 1423-0380

J9 TUMOR BIOL

JI Tumor Biol.

PD AUG

PY 2016

VL 37

IS 8

BP 10871

EP 10876

DI 10.1007/s13277-016-4962-9

PG 6

WC Oncology

WE Science Citation Index Expanded (SCI-EXPANDED)

SC Oncology

GA DV1HW

UT WOS:000382672900087

PM 26883252

DA 2022-05-19

ER

PT J

AU Qiao, GN

Zhang, WJ

Dong, K

AF Qiao, Guanen

Zhang, Wenjuan

Dong, Kui

TI Regulation of ferroptosis by noncoding RNAs: a novel promise treatment

in esophageal squamous cell carcinoma

SO MOLECULAR AND CELLULAR BIOCHEMISTRY

LA English

DT Article; Early Access

DE Esophageal squamous cell carcinoma; ncRNAs; Ferroptosis; GSH metabolism;

Keap1; Nfr2; p53

ID PROLIFERATION; METASTASIS; MIGRATION; INVASION; PATHWAY; STRESS; NRF2

AB Esophageal squamous cell carcinoma (ESCC) is a highly prevalent tumor that requires extensive research. Ferroptosis is a unique cell death modality driven by iron-dependent phospholipid peroxidation manifested as an accumulation of lipid-reactive oxygen species. With further understanding of noncoding RNAs (ncRNAs), numerous studies have demonstrated an important regulatory role of ncRNAs in ESCC through ferroptosis, including microRNAs, long ncRNAs, and circular RNAs. These ncRNAs influence the expression of the target gene to regulate ESCC progression by involving the ferroptosis signaling pathway. However, the specific regulatory mechanism of ncRNAs on ferroptosis in ESCC remains largely unknown. This review summarized the current knowledge on the relation between ferroptosis regulators, such as glutathione synthesis/metabolism, Keap1/Nfr2, and p53, by ncRNAs and ESCC. This review also proposed the possible therapeutic approaches for ncRNAs targeting ferroptosis in ESCC. This is the latest and most effective summary of recent research achievements of ncRNAs on ferroptosis in ESCC. These ncRNAs based on ferroptosis merit further investigation in preclinical research of ESCC.

C1 [Qiao, Guanen; Zhang, Wenjuan; Dong, Kui] First Hosp Handan City, Dept Gastroenterol, 25 Congtai Rd, Handan 056002, Hebei, Peoples R China.

RP Qiao, GN (corresponding author), First Hosp Handan City, Dept Gastroenterol, 25 Congtai Rd, Handan 056002, Hebei, Peoples R China.

EM 627559246@qq.com

FU Medical Science Research Project of Hebei Province [20220013]

FX This study was supported by Medical Science Research Project of Hebei

Province (20220013).

CR Abnet CC, 2018, GASTROENTEROLOGY, V154, P360, DOI 10.1053/j.gastro.2017.08.023

Akdemir B, 2017, CANCER RES, V77, DOI 10.1158/1538-7445.AM2017-2532

Akdemir B, 2017, MOL CANCER RES, V15, P1570, DOI 10.1158/1541-7786.MCR-17-0232

Bai T, 2020, J CELL PHYSIOL, V235, P5637, DOI 10.1002/jcp.29496

Bellezza I, 2018, BBA-MOL CELL RES, V1865, P721, DOI 10.1016/j.bbamcr.2018.02.010

Braga EA, 2020, INT J MOL SCI, V21, DOI 10.3390/ijms21228855

Bure IV, 2018, MOL BIOL+, V52, P787, DOI 10.1134/S0026893318060031

Chen JL, 2019, THER ADV MED ONCOL, V11, DOI 10.1177/1758835919838958

Choi Y-W., 2013, TRANSGLUTAMINASE 2 A

Di Bella S, 2020, BRIEF BIOINFORM, V21, P1987, DOI 10.1093/bib/bbz110

Dixon SJ, 2012, CELL, V149, P1060, DOI 10.1016/j.cell.2012.03.042

Elofey SGH, 2020, J EGYPT NATL CANCER, V32, DOI 10.1186/s43046-020-00049-x

Gao MH, 2015, MOL CELL, V59, P298, DOI 10.1016/j.molcel.2015.06.011

Ge MH, 2021, CNS NEUROSCI THER, V27, P1023, DOI 10.1111/cns.13657

Han M, 2020, HUM CELL, V33, P641, DOI 10.1007/s13577-020-00329-7

He ZS, 2021, J CELL PHYSIOL, V236, P107, DOI 10.1002/jcp.29631

Hong T, 2021, REDOX BIOL, V42, DOI 10.1016/j.redox.2021.101928

Hsu JL, 2020, EMBO MOL MED, V12, DOI 10.15252/emmm.201809386

Hu HB, 2016, CELL PHYSIOL BIOCHEM, V40, P117, DOI 10.1159/000452529

Huang FL, 2018, ASIAN J SURG, V41, P210, DOI 10.1016/j.asjsur.2016.10.005

Jaramillo MC, 2013, GENE DEV, V27, P2179, DOI 10.1101/gad.225680.113

Jeon BN, 2009, J BIOL CHEM, V284, P17935, DOI 10.1074/jbc.M809559200

Jiang B, 2020, DIGEST DIS SCI, V65, P1999, DOI 10.1007/s10620-019-05929-4

Jiang L, 2015, NATURE, V520, P57, DOI 10.1038/nature14344

Jiang XJ, 2021, NAT REV MOL CELL BIO, V22, P266, DOI 10.1038/s41580-020-00324-8

Koppula P, 2018, CANCER COMMUN, V38, DOI 10.1186/s40880-018-0288-x

Lai X, 2019, NUCLEIC ACIDS RES, V47, P7753, DOI 10.1093/nar/gkz638

Li WJ, 2016, CHEM RES TOXICOL, V29, P694, DOI 10.1021/acs.chemrestox.6b00016

Li XY, 2020, CANCER CELL INT, V20, DOI 10.1186/s12935-020-01617-w

Liu B, 2018, CANCER CELL INT, V18, DOI 10.1186/s12935-018-0537-8

Liu JM, 2020, FRONT ONCOL, V10, DOI 10.3389/fonc.2020.552236

Liu M, 2015, BIOSCIENCE REP, V35, DOI 10.1042/BSR20150092

Lu JJ, 2020, LIFE SCI, V260, DOI 10.1016/j.lfs.2020.118305

Luo J, 2021, FRONT ONCOL, V10, DOI 10.3389/fonc.2020.618930

Luo JD, 2013, CANCER SCI, V104, P1544, DOI 10.1111/cas.12248

Luo YF, 2021, REDOX BIOL, V42, DOI 10.1016/j.redox.2021.101908

Lv DS, 2016, TUMOR BIOL, V37, P16259, DOI 10.1007/s13277-016-5426-y

Ma SH, 2018, ANN NY ACAD SCI, V1434, P164, DOI 10.1111/nyas.13681

Meng LJ, 2018, CELL DEATH DIS, V9, DOI 10.1038/s41419-018-1119-3

Nie JH, 2015, ONCOL REP, V34, P311, DOI 10.3892/or.2015.3962

Ou Y, 2016, P NATL ACAD SCI USA, V113, pE6806, DOI 10.1073/pnas.1607152113

Rios-Luci C, 2020, CANCER LETT, V470, P161, DOI 10.1016/j.canlet.2019.11.026

Salzman J, 2013, PLOS GENET, V9, DOI 10.1371/journal.pgen.1003777

Shi ZY, 2021, FRONT ONCOL, V11, DOI 10.3389/fonc.2021.579286

Tarangelo A, 2018, CELL REP, V22, P569, DOI 10.1016/j.celrep.2017.12.077

Tong KI, 2006, MOL CELL BIOL, V26, P2887, DOI 10.1128/MCB.26.8.2887-2900.2006

Venkatesh D, 2020, GENE DEV, V34, P526, DOI 10.1101/gad.334219.119

Villacorta L, 2007, AM J PHYSIOL-HEART C, V293, pH770, DOI 10.1152/ajpheart.00261.2007

Wang PL, 2016, MOL MED REP, V13, P3074, DOI 10.3892/mmr.2016.4913

Wang Y, 2020, APOPTOSIS, V25, P786, DOI 10.1007/s10495-020-01638-w

Wen Y, 2021, FREE RADICAL BIO MED, V173, P41, DOI 10.1016/j.freeradbiomed.2021.07.019

Wu S, 2021, FRONT CELL DEV BIOL, V9, DOI 10.3389/fcell.2021.733751

Xiao S, 2021, FREE RADICAL BIO MED, V164, P58, DOI 10.1016/j.freeradbiomed.2020.11.029

Yamamoto S, 2014, MOL CANCER RES, V12, P58, DOI 10.1158/1541-7786.MCR-13-0246-T

Yan S, 2021, BIOENGINEERED, V12, P682, DOI 10.1080/21655979.2021.1887645

Yang WS, 2014, CELL, V156, P317, DOI 10.1016/j.cell.2013.12.010

Yang Y, 2019, J CELL BIOCHEM, V120, P18702, DOI 10.1002/jcb.29178

Yao J, 2020, IUBMB LIFE, V72, P401, DOI 10.1002/iub.2196

Ye JC, 2021, FRONT GENET, V12, DOI 10.3389/fgene.2021.697524

Ye JC, 2019, BIOCHEM PHARMACOL, V166, P93, DOI 10.1016/j.bcp.2019.05.013

Zhang HY, 2020, MOL CANCER, V19, DOI 10.1186/s12943-020-01168-8

Zhang J, 2014, INT J CLIN EXP PATHO, V7, P602

Zhang MJ, 2019, J MATER CHEM B, V7, P2421, DOI 10.1039/c9tb00170k

Zhang Y, 2020, EUR REV MED PHARMACO, V24, P3674, DOI 10.26355/eurrev_202004_20830

Zhang Y, 2019, CANCER MANAG RES, V11, P6201, DOI 10.2147/CMAR.S197557

Zhang ZH, 2019, ACTA BIOCH BIOPH SIN, V51, P826, DOI 10.1093/abbs/gmz069

Zhao ZH, 2019, IUBMB LIFE, V71, P1492, DOI 10.1002/iub.2069

Zheng YZ, 2020, BIOMED PHARMACOTHER, V129, DOI 10.1016/j.biopha.2020.110442

Zhu Y, 2021, MOL MED REP, V23, DOI 10.3892/mmr.2021.12110

Zuo J, 2020, THORAC CANCER, V11, P738, DOI 10.1111/1759-7714.13326

NR 70

TC 0

Z9 0

U1 1

U2 1

PU SPRINGER

PI DORDRECHT

PA VAN GODEWIJCKSTRAAT 30, 3311 GZ DORDRECHT, NETHERLANDS

SN 0300-8177

EI 1573-4919

J9 MOL CELL BIOCHEM

JI Mol. Cell. Biochem.

DI 10.1007/s11010-022-04441-0

EA APR 2022

PG 10

WC Cell Biology

WE Science Citation Index Expanded (SCI-EXPANDED)

SC Cell Biology

GA 0Q0EC

UT WOS:000784598000002

PM 35449482

DA 2022-05-19

ER

PT J

AU Davakis, S

Syllaios, A

Mpaili, E

Liakakos, T

Charalabopoulos, A

AF Davakis, Spyridon

Syllaios, Athanasios

Mpaili, Efstratia

Liakakos, Theodoros

Charalabopoulos, Alexandros

TI Laparoscopic-assisted Esophageal Bypass for T4b Esophageal Tumor as a

Bridge to Definitive Therapy

SO IN VIVO

LA English

DT Article

DE Esophageal bypass; squamous cell carcinoma; esophagobrochial fistula; 3D

laparoscopy

ID RETROSTERNAL BYPASS; KIRSCHNER OPERATION; GASTRIC BYPASS; CANCER;

PALLIATION; FISTULA; CHEMORADIOTHERAPY; SURGERY

AB Background/Aim: Esophagobronchial fistula is a common complication of advanced esophageal cancer, related to respiratory distress and mortality. Esophageal bypass has been successfully utilized for palliation, as bridging to definitive chemoradiotherapy. The aim of this study is to present an extremely difficult case of a mid-esophageal squamous cell carcinoma complicated with aerodigestive fistula that was treated using 3D laparoscopic-assisted esophageal bypass with curative intent. Case Report: A 49-year-old female patient presented with T4b esophageal-squamous cell carcinoma and esophagobronchial fistula. Laparoscopic-assisted V-shaped retrosternal esophageal bypass using a gastric conduit was started, which was converted to open surgery due to respiratory distress. The patient was able to undergo chemoradiotherapy treatment. Conclusion: 3D laparoscopic-assisted esophageal bypass can be a safe and feasible approach in patients with advanced mid-esophageal squamous cell carcinoma and esophagobroncial fistula. Additionally to the advantages of laparoscopic surgery, this operation permits oral feeding, and can be used with possible curative intent in patients with adequate response to chemoradiotherapy.

C1 [Davakis, Spyridon; Syllaios, Athanasios; Mpaili, Efstratia; Liakakos, Theodoros; Charalabopoulos, Alexandros] Natl & Kapodistrian Univ Athens, Laiko Gen Hosp, Dept Surg 1, Upper Gastrointestinal & Gen Surg Unit, Athens, Greece.

[Charalabopoulos, Alexandros] Mid Essex Hosp Serv NHS Trust, Broomfield Hosp, Dept Upper Gastrointestinal Surg, Reg Oesophagogastr Canc Ctr, Chelmsford, Essex, England.

RP Davakis, S (corresponding author), Natl & Kapodistrian Univ Athens, Laiko Gen Hosp, Upper Gastrointestinal & Gen Surg Unit, 17 Agiou Thoma St, Athens 11527, Greece.

EM spdavakis@gmail.com

RI Baili, Efstratia/AAE-3367-2019

OI Baili, Efstratia/0000-0001-8745-2269; Charalabopoulos,

Alexandros/0000-0003-1835-4723

CR Bakshi A, 2017, ANN CARDIOTHORAC SUR, V6, P137, DOI 10.21037/acs.2017.03.07

CONLAN AA, 1983, ANN THORAC SURG, V36, P396, DOI 10.1016/S0003-4975(10)60476-6

Diamantis G, 2011, WORLD J GASTROENTERO, V17, P144, DOI 10.3748/wjg.v17.i2.144

Dobrucali A, 2010, WORLD J GASTROENTERO, V16, P5739, DOI 10.3748/wjg.v16.i45.5739

Halpern AL, 2020, SURG ENDOSC, V34, P3470, DOI 10.1007/s00464-019-07124-y

Hanagiri T, 2011, INT SURG, V96, P189, DOI 10.9738/CC38.1

Hihara J, 2014, ANN THORAC SURG, V97, P290, DOI 10.1016/j.athoracsur.2013.08.060

Javed A, 2013, SURG ENDOSC, V27, P3726, DOI 10.1007/s00464-013-2956-x

Javed A, 2012, SURG ENDOSC, V26, P3344, DOI 10.1007/s00464-012-2307-3

Kimura M, 2015, INT J SURG CASE REP, V9, P115, DOI 10.1016/j.ijscr.2015.02.053

Kitagawa Y, 2019, ESOPHAGUS-TOKYO, V16, P25, DOI 10.1007/s10388-018-0642-8

MANNELL A, 1988, BRIT J SURG, V75, P283, DOI 10.1002/bjs.1800750332

Meunier B, 1996, ANN THORAC SURG, V62, P373, DOI 10.1016/0003-4975(96)00236-6

Moore JM, 2016, ANN THORAC SURG, V102, P215, DOI 10.1016/j.athoracsur.2016.02.039

Motoori M, 2012, ANN SURG ONCOL, V19, P2135, DOI 10.1245/s10434-012-2231-8

Nakajima Y, 2016, WORLD J SURG, V40, P1158, DOI 10.1007/s00268-015-3391-z

Ohsawa M, 2019, ANTICANCER RES, V39, P4399, DOI 10.21873/anticanres.13610

ONG GB, 1973, BRIT J SURG, V60, P221, DOI 10.1002/bjs.1800600315

ORRINGER MB, 1975, J THORAC CARDIOV SUR, V70, P836

Reim D, 2015, GASTROINTEST TUMORS, V2, P166, DOI 10.1159/000442907

Sdralis E, 2020, AM J SURG, V219, P123, DOI 10.1016/j.amjsurg.2019.06.013

Shin S, 2011, IEEE INTERNATIONAL CONFERENCE ON CONSUMER ELECTRONICS (ICCE 2011), P133, DOI 10.1109/ICCE.2011.5722501

Sinha RY, 2017, J MINIM ACCESS SURG, V13, P165, DOI 10.4103/0972-9941.181761

Siosaki MD, 2013, J SURG CASE REP, DOI 10.1093/jscr/rjt017

Whooley BP, 2002, ARCH SURG-CHICAGO, V137, P1228, DOI 10.1001/archsurg.137.11.1228

NR 25

TC 0

Z9 0

U1 0

U2 1

PU INT INST ANTICANCER RESEARCH

PI ATHENS

PA EDITORIAL OFFICE 1ST KM KAPANDRITIOU-KALAMOU RD KAPANDRITI, PO BOX 22,

ATHENS 19014, GREECE

SN 0258-851X

EI 1791-7549

J9 IN VIVO

JI In Vivo

PD JUL-AUG

PY 2020

VL 34

IS 4

BP 2163

EP 2168

DI 10.21873/invivo.12024

PG 6

WC Medicine, Research & Experimental

WE Science Citation Index Expanded (SCI-EXPANDED)

SC Research & Experimental Medicine

GA NH3ZY

UT WOS:000564612800026

PM 32606199

OA Green Published, gold

DA 2022-05-19

ER

PT J

AU Cheng, R

Zhu, ST

Guo, SL

Min, L

Xing, J

Guo, QD

Li, P

Zhang, ST

AF Cheng, Rui

Zhu, Shengtao

Guo, Shuilong

Min, Li

Xing, Jie

Guo, Qingdong

Li, Peng

Zhang, Shutian

TI Downregulation of NONO induces apoptosis, suppressing growth and

invasion in esophageal squamous cell carcinoma

SO ONCOLOGY REPORTS

LA English

DT Article

DE NONO; esophageal squamous cell carcinoma; apoptosis; migration; invasion

ID NUCLEAR-MATRIX; CANCER; PROTEIN; PSF; P54(NRB); RNA; PROMOTES;

EPIDEMIOLOGY; PROGRESSION; ACTIVATION

AB Esophageal squamous cell carcinoma (ESCC) is one of the most common malignancies in China, and is associated with high morbidity and mortality. However, the molecular mechanisms that control ESCC tumorigenicity and metastasis remain unclear. Here, we report that the RNA splicing factor, NONO, is an important regulator of ESCC growth, apoptosis and invasion. NONO protein levels were dramatically upregulated in ESCC when compared with that in adjacent benign esophageal squamous epithelium. Particularly, NONO expression was statistically higher in tumors with greater tumor invasion depth. Using multiple ESCC cell models, we further showed that NONO depletion using siRNA significantly inhibited proliferation, invasion, and promoted apoptosis of ESCC cells. In addition we found that knockdown of NONO could reduce protein levels of phosphorylated Akt and Erk1/2. Our findings suggest that NONO plays a potent role in multiple biological aspects of ESCC through activation of the Akt and Erk1/2 signaling pathways. Taken together, our findings suggest that NONO might play an important role in promoting tumorigenesis of ESCC. It may provide a promising approach to prevent the progress of ESCC.

C1 [Cheng, Rui; Zhu, Shengtao; Guo, Shuilong; Min, Li; Xing, Jie; Guo, Qingdong; Li, Peng; Zhang, Shutian] Capital Med Univ, Beijing Friendship Hosp, Dept Gastroenterol, 95 Yong An Rd, Beijing 100050, Peoples R China.

[Cheng, Rui; Zhu, Shengtao; Guo, Shuilong; Min, Li; Xing, Jie; Guo, Qingdong; Li, Peng; Zhang, Shutian] Natl Clin Res Ctr Digest Dis, Beijing 100050, Peoples R China.

[Cheng, Rui; Zhu, Shengtao; Guo, Shuilong; Min, Li; Xing, Jie; Guo, Qingdong; Li, Peng; Zhang, Shutian] Beijing Digest Dis Ctr, Beijing 100050, Peoples R China.

[Cheng, Rui; Zhu, Shengtao; Guo, Shuilong; Min, Li; Xing, Jie; Guo, Qingdong; Li, Peng; Zhang, Shutian] Beijing Key Lab Precancerous Lesion Digest Dis, Beijing 100050, Peoples R China.

RP Zhang, ST (corresponding author), Capital Med Univ, Beijing Friendship Hosp, Dept Gastroenterol, 95 Yong An Rd, Beijing 100050, Peoples R China.

EM zhangshutian@ccmu.edu.cn

RI Min, Li/AAG-2088-2021; Min, Li/T-8107-2018

OI Min, Li/0000-0001-9595-5536; Min, Li/0000-0001-9595-5536

FU Beijing Municipal Administration of Hospitals Clinical Medicine

Development of Special Funding Support [ZY201308]; Beijing Natural

Science Foundation [7152043]

FX This study was supported by Beijing Municipal Administration of

Hospitals Clinical Medicine Development of Special Funding Support (no.

ZY201308), and Beijing Natural Science Foundation (no. 7152043).

CR Barboro P, 2008, CELL ONCOL, V30, P13

Basu A, 1997, MOL CELL BIOL, V17, P677, DOI 10.1128/MCB.17.2.677

Bond CS, 2009, J CELL BIOL, V186, P637, DOI 10.1083/jcb.200906113

Chan-Hui PY, 1998, BIOCHEM J, V336, P599, DOI 10.1042/bj3360599

Clark J, 1997, ONCOGENE, V15, P2233, DOI 10.1038/sj.onc.1201394

DETRE S, 1995, J CLIN PATHOL, V48, P876, DOI 10.1136/jcp.48.9.876

DONG BH, 1993, NUCLEIC ACIDS RES, V21, P4085, DOI 10.1093/nar/21.17.4085

Edge SB, 2009, AM JOINT COMMITTEE C

Grille SJ, 2003, CANCER RES, V63, P2172

Hanahan D, 2011, CELL, V144, P646, DOI 10.1016/j.cell.2011.02.013

Holmes RS, 2007, SEMIN RADIAT ONCOL, V17, P2, DOI 10.1016/j.semradonc.2006.09.003

Hu N, 2010, BMC GENOMICS, V11, DOI 10.1186/1471-2164-11-576

Ishiguro H, 2003, INT J CANCER, V105, P26, DOI 10.1002/ijc.11021

Ito T, 2008, BIOCHEM J, V411, P201, DOI 10.1042/BJ20071075

Jemal A, 2010, CA-CANCER J CLIN, V60, P277, DOI 10.3322/caac.20073

Kim YM, 2007, J PROTEOME RES, V6, P3278, DOI 10.1021/pr070168k

Kimchi ET, 2005, CANCER RES, V65, P3146, DOI 10.1158/0008-5472.CAN-04-2490

Li B, 2009, INT J CANCER, V125, P2576, DOI 10.1002/ijc.24675

Li SY, 2009, NUCLEIC ACIDS RES, V37, P6746, DOI 10.1093/nar/gkp741

Liu FX, 2013, MOL CELL BIOCHEM, V381, P115, DOI 10.1007/s11010-013-1693-8

Liu PY, 2014, JNCI-J NATL CANCER I, V106, DOI 10.1093/jnci/dju113

Meng XY, 2012, J DIGEST DIS, V13, P208, DOI 10.1111/j.1751-2980.2012.00578.x

NISHIHIRA T, 1993, J CANCER RES CLIN, V119, P441, DOI 10.1007/BF01215923

PATTON JG, 1993, GENE DEV, V7, P393, DOI 10.1101/gad.7.3.393

Schiffnery S, 2011, CARCINOGENESIS, V32, P1176, DOI 10.1093/carcin/bgr103

Shav-Tal Y, 2002, FEBS LETT, V531, P109, DOI 10.1016/S0014-5793(02)03447-6

Sobin LH, 2010, CANCER-AM CANCER SOC, V116, P5336, DOI 10.1002/cncr.25537

Su H, 2011, CLIN CANCER RES, V17, P2955, DOI 10.1158/1078-0432.CCR-10-2724

Sun XJ, 2013, PLOS ONE, V8, DOI 10.1371/journal.pone.0077353

Vara JAF, 2004, CANCER TREAT REV, V30, P193, DOI 10.1016/j.ctrv.2003.07.007

Wada T, 2004, ONCOGENE, V23, P2838, DOI 10.1038/sj.onc.1207556

Wang X, 2013, ASIAN PAC J CANCER P, V14, P2429, DOI 10.7314/APJCP.2013.14.4.2429

Zhang NN, 2015, PLOS ONE, V10, DOI 10.1371/journal.pone.0118845

Zhu Z, 2016, ONCOGENE, V35, P1399, DOI 10.1038/onc.2015.197

NR 34

TC 8

Z9 8

U1 0

U2 6

PU SPANDIDOS PUBL LTD

PI ATHENS

PA POB 18179, ATHENS, 116 10, GREECE

SN 1021-335X

EI 1791-2431

J9 ONCOL REP

JI Oncol. Rep.

PD JUN

PY 2018

VL 39

IS 6

BP 2575

EP 2583

DI 10.3892/or.2018.6334

PG 9

WC Oncology

WE Science Citation Index Expanded (SCI-EXPANDED)

SC Oncology

GA GF8SA

UT WOS:000432241900011

PM 29620226

OA Green Submitted, Green Published, hybrid

DA 2022-05-19

ER

PT J

AU Situ, D

Wei, WD

Lin, P

Long, H

Zhang, LJ

Fu, JH

Rong, TH

Ma, GW

AF Situ, Dongrong

Wei, Weidong

Lin, Peng

Long, Hao

Zhang, Lanjun

Fu, Jianhua

Rong, Tiehua

Ma, Guowei

TI Do Tumor Grade and Location Affect Survival in Esophageal Squamous Cell

Carcinoma? Survival Analysis of 302 Cases of pT3N0M0 Esophageal Squamous

Cell Carcinoma

SO ANNALS OF SURGICAL ONCOLOGY

LA English

DT Article

ID PROGNOSTIC-FACTORS; RISK-FACTORS; LYMPH-NODES; CANCER; SMOKING;

CLASSIFICATION; RESECTION

AB The 7th edition of the American Joint Committee on Cancer (AJCC) cancer staging manual incorporates tumor grade and location for staging pT2-3N0M0 esophageal squamous cell carcinoma. Patients with pT3N0M0, classified as stage IIa according to the 6th edition of the AJCC cancer staging manual, can now be classified as stage Ib, IIa, or IIb. We aimed to discuss whether these changes affect survival and determine other potential prognostic factors.

We retrospectively analyzed 302 patients with postoperative pathologic stage T3N0M0 who underwent esophagectomy between 1990 and 2005 at Sun Yat-sen University Cancer Center. We performed univariate and multivariate analyses to identify prognostic factors for survival and used the Kaplan-Meier method to compare survival difference in each prognostic factor, including tumor grade and location.

The 5-year overall survival rate was 46 %, with a median survival of 1,244.5 days. Gender, age, alcohol consumption, forced expiratory volume in 1 s (FEV1), and number of removed lymph nodes were independent prognostic factors in both univariate and multivariate analyses. Smoking was also a prognostic factor in survival analysis by the Kaplan-Meier method. However, histologic tumor grade and location had no significant influence on patient survival.

Age, gender, alcohol consumption, FEV1, number of removed lymph nodes, and cigarette smoking are independent prognostic factors in patients with pT3N0M0 esophageal squamous cell carcinoma. However, tumor grade and location may not be as strong predictors in these patients as indicated in the 7th edition of the AJCC cancer staging manual.

C1 [Situ, Dongrong; Wei, Weidong; Lin, Peng; Long, Hao; Zhang, Lanjun; Fu, Jianhua; Rong, Tiehua; Ma, Guowei] Sun Yat Sen Univ, Dept Thorac Surg, Ctr Canc, State Key Lab Oncol S China, Guangzhou 510275, Guangdong, Peoples R China.

RP Situ, D (corresponding author), Sun Yat Sen Univ, Dept Thorac Surg, Ctr Canc, State Key Lab Oncol S China, Guangzhou 510275, Guangdong, Peoples R China.

EM gzlmst@yeah.net

FU Fundamental Research Funds for the Central Universities [09YKPY51];

Science and Technology Planning Project of Guangdong Province, China

[2010B31500010]; National High Technology Research and Development

Program of China (863 Program) [2009AA02Z421]

FX This work was supported by the Fundamental Research Funds for the

Central Universities (09YKPY51); the Science and Technology Planning

Project of Guangdong Province, China (2010B31500010); and the National

High Technology Research and Development Program of China (863 Program;

2009AA02Z421). The authors would like to thank Enago (www.enago.cn) for

the English language review.

CR Altorki NK, 2008, ANN SURG, V248, P221, DOI 10.1097/SLA.0b013e31817bbe59

Bogoevski D, 2008, ANN SURG, V247, P633, DOI 10.1097/SLA.0b013e3181656d07

Dickson GH, 2001, EUR J SURG ONCOL, V27, P641

Doki Y, 2005, WORLD J SURG, V29, P700, DOI 10.1007/s00268-005-7596-4

Edge S., 2010, AM JOINT COMMITTEE C, VSeventh

Eloubeidi MA, 2002, CANCER-AM CANCER SOC, V95, P1434, DOI 10.1002/cncr.10868

Elsayed H, 2010, INTERACT CARDIOV TH, V11, P65, DOI 10.1510/icvts.2009.223826

Enzinger PC, 2003, NEW ENGL J MED, V349, P2241, DOI 10.1056/NEJMra035010

GAO YT, 1994, INT J CANCER, V58, P192, DOI 10.1002/ijc.2910580208

Greene FL, 2002, AJCC CANC STAGING MA

Hsu PK, 2010, ANN THORAC SURG, V89, P1024, DOI 10.1016/j.athoracsur.2010.01.017

Jemal A, 2009, CA-CANCER J CLIN, V59, P225, DOI 10.3322/caac.20006

Khan NA, 2011, J CANCER RES THER, V7, P15, DOI 10.4103/0973-1482.80431

Kountourakis P, 2011, CANCER-AM CANCER SOC, V117, P925, DOI 10.1002/cncr.25651

Lagarde SM, 2008, ANN THORAC SURG, V85, P1938, DOI 10.1016/j.athoracsur.2008.03.014

Lee CH, 2005, INT J CANCER, V113, P475, DOI 10.1002/ijc.20619

Lin CS, 2009, ANN THORAC SURG, V87, P1056, DOI 10.1016/j.athoracsur.2008.11.051

Rice TW, 2009, DIS ESOPHAGUS, V22, P1, DOI 10.1111/j.1442-2050.2008.00901.x

RODER JD, 1994, BRIT J SURG, V81, P410, DOI 10.1002/bjs.1800810330

Shitara K, 2010, CANCER SCI, V101, P1001, DOI 10.1111/j.1349-7006.2009.01466.x

Sundelof M, 2008, EUR J CANCER, V44, P1566, DOI 10.1016/j.ejca.2008.04.002

Wijnhoven BPL, 2007, ANN SURG, V245, P717, DOI 10.1097/01.sla.0000251703.35919.02

WONG J, 1987, AM J SURG, V153, P18, DOI 10.1016/0002-9610(87)90196-6

NR 23

TC 16

Z9 21

U1 0

U2 7

PU SPRINGER

PI NEW YORK

PA 233 SPRING ST, NEW YORK, NY 10013 USA

SN 1068-9265

J9 ANN SURG ONCOL

JI Ann. Surg. Oncol.

PD FEB

PY 2013

VL 20

IS 2

BP 580

EP 585

DI 10.1245/s10434-012-2656-0

PG 6

WC Oncology; Surgery

WE Science Citation Index Expanded (SCI-EXPANDED)

SC Oncology; Surgery

GA 081BS

UT WOS:000314291100031

PM 23015029

DA 2022-05-19

ER

PT J

AU Cavallin, F

Alfieri, R

Scarpa, M

Cagol, M

Ruol, A

Fassan, M

Rugge, M

Ancona, E

Castoro, C

AF Cavallin, Francesco

Alfieri, Rita

Scarpa, Marco

Cagol, Matteo

Ruol, Alberto

Fassan, Matteo

Rugge, Massimo

Ancona, Ermanno

Castoro, Carlo

TI Nodal skip metastasis in thoracic esophageal squamous cell carcinoma: a

cohort study

SO BMC SURGERY

LA English

DT Article

DE Esophageal cancer; Nodal skip metastasis; Metastasis; Lymph node

involvement; Esophagectomy

ID RISK-FACTORS; LYMPH-NODES; CANCER; PREVALENCE; SURVIVAL; PATTERN; SPREAD

AB Background: Nodal skip metastasis is a prognostic factor in some sites of malignancies, but its role in esophageal cancer is still unclear. The present study aimed to investigate occurrence and effect of nodal skip metastases in thoracic esophageal squamous cell carcinoma.

Methods: All 578 patients undergoing esophagectomy for thoracic esophageal squamous cell carcinoma at the Center for Esophageal Diseases located in Padova between January 1992 and December 2010 were retrospectively evaluated. Selection criteria were R0 resection, pathological M0 stage and pathological lymph node involvement. Patients receiving neoadjuvant therapy were excluded.

Results: The selection identified 88 patients with lymph node involvement confirmed by pathological evaluation. Sixteen patients (18.2%) had nodal skip metastasis. Adjusting for the number of lymph node metastases, patient with nodal skip metastasis had similar 5-year overall survival (14% vs. 13%, p = 0.93) and 5-year disease free survival (14% vs. 9%, p = 0.48) compared to patients with both peritumoral and distant lymph node metastases. The risk difference of nodal skip metastasis was: -24.1% (95% C.I. -43.1% to -5.2%) in patients with more than one lymph node metastasis compared to those with one lymph node metastasis; -2.3% (95% C.I. -29.8% to 25.2%) in middle thoracic esophagus and -23.0% (95% C.I. -47.8% to 1.8%) in lower thoracic esophagus compared to upper thoracic esophagus; 18.1% (95% C.I. 3.2% to 33.0%) in clinical N0 stage vs. clinical N+ stage.

Conclusions: Nodal skip metastasis is a common pattern of metastatic lymph involvement in thoracic esophageal squamous cell carcinoma. However, neither overall survival nor disease free survival are associated with nodal skip metastasis occurrence.

C1 [Cavallin, Francesco; Alfieri, Rita; Scarpa, Marco; Cagol, Matteo; Ancona, Ermanno; Castoro, Carlo] IRCCS, Reg Ctr Esophageal Dis, Esophageal & Digest Tract Surg Unit, Veneto Inst Oncol IOV, Padua, Italy.

[Ruol, Alberto] Azienda Osped Padova, Surg Clin 3, Padua, Italy.

[Fassan, Matteo; Rugge, Massimo] Univ Padua, Surg Pathol & Cytopathol Unit, Dept Med DIMED, Padua, Italy.

RP Cavallin, F (corresponding author), IRCCS, Reg Ctr Esophageal Dis, Esophageal & Digest Tract Surg Unit, Veneto Inst Oncol IOV, Padua, Italy.

EM cescocava@libero.it

RI Scarpa, Marco/K-9970-2016; Castoro, Carlo/AAO-6927-2020; Alfieri,

Rita/F-1676-2018; Castoro, Carlo/L-1046-2016; Fassan, Matteo/F-5152-2012

OI Scarpa, Marco/0000-0002-0217-6523; Alfieri, Rita/0000-0002-4993-2197;

Castoro, Carlo/0000-0001-9601-9336; Fassan, Matteo/0000-0001-6515-5482

CR Akutsu Y, 2012, J SURG ONCOL, V105, P756, DOI 10.1002/jso.23007

Castoro C, 2011, ANN SURG ONCOL, V18, P3743, DOI 10.1245/s10434-011-1753-9

Chen JQ, 2009, EUR J CARDIO-THORAC, V36, P480, DOI 10.1016/j.ejcts.2009.03.056

Arnal MJD, 2015, WORLD J GASTROENTERO, V21, P7933, DOI 10.3748/wjg.v21.i26.7933

Dunn GP, 2002, NAT IMMUNOL, V3, P991, DOI 10.1038/ni1102-991

Edge SB, 2009, AJCC CANC STAGING MA, V7th

Filip B, 2014, SURG ENDOSC, V28, P1238, DOI 10.1007/s00464-013-3314-8

Hosch SB, 2001, J CLIN ONCOL, V19, P1970, DOI 10.1200/JCO.2001.19.7.1970

Kayani B, 2011, EJSO-EUR J SURG ONC, V37, P747, DOI 10.1016/j.ejso.2011.06.018

Li B, 2013, J THORAC CARDIOV SUR, V146, P1198, DOI 10.1016/j.jtcvs.2013.07.006

Li HC, 2007, EUR SURG RES, V39, P1, DOI 10.1159/000096925

Machens A, 2004, ARCH SURG-CHICAGO, V139, P43, DOI 10.1001/archsurg.139.1.43

Mariette C, 2008, ANN SURG, V247, P365, DOI 10.1097/SLA.0b013e31815aaadf

Prenzel KL, 2003, J SURG ONCOL, V82, P256, DOI 10.1002/jso.10219

Prenzel KL, 2010, ANN THORAC SURG, V90, P1662, DOI 10.1016/j.athoracsur.2010.07.008

Skandalakis JE, 2000, SURG CLIN N AM, V80, P85, DOI 10.1016/S0039-6109(05)70399-6

Tachibana M, 2008, EUR J CARDIO-THORAC, V34, P427, DOI 10.1016/j.ejcts.2008.04.022

Torre LA, 2015, CA-CANCER J CLIN, V65, P87, DOI 10.3322/caac.21262

Tran GD, 2005, INT J CANCER, V113, P456, DOI 10.1002/ijc.20616

Wu J, 2012, WORLD J SURG ONCOL, V10, DOI 10.1186/1477-7819-10-210

Xu QR, 2011, WORLD J SURG, V35, P1303, DOI 10.1007/s00268-011-1015-9

Xu XL, 2015, PLOS ONE, V10, DOI 10.1371/journal.pone.0133076

Zheng YZ, 2015, WORLD J GASTROENTERO, V21, P8644, DOI 10.3748/wjg.v21.i28.8644

Zhu ZF, 2013, ANN SURG ONCOL, V20, P3052, DOI 10.1245/s10434-013-2987-5

NR 24

TC 8

Z9 10

U1 0

U2 0

PU BMC

PI LONDON

PA CAMPUS, 4 CRINAN ST, LONDON N1 9XW, ENGLAND

SN 1471-2482

J9 BMC SURG

JI BMC Surg.

PD MAY 2

PY 2017

VL 17

AR 49

DI 10.1186/s12893-017-0247-5

PG 7

WC Surgery

WE Science Citation Index Expanded (SCI-EXPANDED)

SC Surgery

GA EZ9GW

UT WOS:000405038200001

PM 28464907

OA Green Published, gold

DA 2022-05-19

ER

PT J

AU Jiang, WP

Wang, Z

Jia, Y

AF Jiang, Wenpeng

Wang, Zhou

Jia, Yang

TI CEP55 overexpression predicts poor prognosis in patients with locally

advanced esophageal squamous cell carcinoma

SO ONCOLOGY LETTERS

LA English

DT Article

DE CEP55 overexpression; locally advanced esophageal squamous cell

carcinoma; poor prognosis

ID AMERICAN JOINT COMMITTEE; LYMPH-NODE METASTASIS; RADICAL ESOPHAGECTOMY;

MIDBODY; RECURRENCE; PATTERN; PROTEIN; CANCER; CYTOKINESIS; EXPRESSION

AB Development of esophageal squamous cell carcinoma (ESCC) involves alterations in multiple genes with corresponding proteins. Recent studies have demonstrated that centrosomal protein 55 (CEP55) shares certain features with oncogenes, and CEP55 overexpression is associated with the development and progression of malignant tumors. The present study aimed to analyze, for the first time, whether CEP55 expression is related to clinicopothalogic features in the esophageal squamous cell carcinoma (ESCC), as well as patient survival. A total of 110 patients with mid-thoracic ES CC who suffered from Ivor-Lewis were enrolled. The CEP55 expression profile of these patients in tumour tissues and corresponding healthy esophageal mucosa (CHEM) was detected by immunohistochemistry and semi-quantitative reverse transcription-polymerase chain reaction analyses. Correlations between CEP55 expression and clinicopathological factors were analyzed using x-2 test. The log-rank test was employed to calculate survival rate. A Cox regression multivariate analysis was performed to determine independent prognostic factors. The results demonstrated that CEP55 expression in ESCC was significantly higher than that of CHEM (P<0.001). Overexpression of CEP55 was significantly associated with differentiation degree (P=0.022), T stage (P=0.019), lymph node metastasis (P=0.033), clinicopathological staging (P=0.002) and tumor recurrence (P=0.021) in locally advanced ESCC patients. In addition, CEP55 overexpression was significantly associated with reduced overall survival of patients after surgery (P=0.012). The 5-year survival rate of patients without CEP55 overexpression was significantly higher than that of patients with CEP55 overexpression (P=0.012). Therefore, these findings suggest that CEP55 overexpression correlates with poor prognosis in locally advanced ESCC patients.

C1 [Jiang, Wenpeng; Wang, Zhou; Jia, Yang] Shandong Univ, Shandong Prov Hosp, Dept Thorac Surg, 324 Jingwu Rd, Jinan 250021, Shandong, Peoples R China.

RP Wang, Z (corresponding author), Shandong Univ, Shandong Prov Hosp, Dept Thorac Surg, 324 Jingwu Rd, Jinan 250021, Shandong, Peoples R China.

EM wangzhou620226@163.com

CR Baba M, 1998, Dis Esophagus, V11, P28, DOI 10.1093/dote/11.1.28

Carlton JG, 2007, SCIENCE, V316, P1908, DOI 10.1126/science.1143422

Chang YC, 2010, J CELL BIOCHEM, V109, P1274, DOI 10.1002/jcb.22517

Chen CH, 2007, ONCOGENE, V26, P4272, DOI 10.1038/sj.onc.1210207

Chen CH, 2009, ONCOGENE, V28, P2723, DOI 10.1038/onc.2009.128

Chen CH, 2009, PLOS ONE, V4, DOI 10.1371/journal.pone.0005052

Chen G, 2009, WORLD J SURG, V33, P278, DOI 10.1007/s00268-008-9849-5

Chen HX, 2013, ANN SURG ONCOL, V20, P2721, DOI 10.1245/s10434-013-2928-3

D'Annoville T, 2013, EUR J CARDIO-THORAC, V44, pE207, DOI 10.1093/ejcts/ezt332

Dresner SM, 2000, BRIT J SURG, V87, P1426, DOI 10.1046/j.1365-2168.2000.01541.x

Fabbro M, 2005, DEV CELL, V9, P477, DOI 10.1016/j.devcel.2005.09.003

Fujiwara T, 2005, NATURE, V437, P1043, DOI 10.1038/nature04217

Glotzer M, 2005, SCIENCE, V307, P1735, DOI 10.1126/science.1096896

Gromley A, 2005, CELL, V123, P75, DOI 10.1016/j.cell.2005.07.027

Gu Y, 2006, CANCER-AM CANCER SOC, V106, P1017, DOI 10.1002/cncr.21693

Hou X, 2014, ANN SURG ONCOL, V21, P337, DOI 10.1245/s10434-013-3265-2

Inoda S, 2009, J IMMUNOTHER, V32, P474, DOI 10.1097/CJI.0b013e3181a1d109

Jemal A, 2011, CA-CANCER J CLIN, V61, P69, DOI [10.3322/caac.20107, 10.3322/caac.20115]

Li B, 2012, J THORAC CARDIOV SUR, V144, P778, DOI 10.1016/j.jtcvs.2012.07.002

Matsubara T, 1996, J AM COLL SURGEONS, V182, P340

Morita E, 2007, EMBO J, V26, P4215, DOI 10.1038/sj.emboj.7601850

Nakagawa S, 2004, J AM COLL SURGEONS, V198, P205, DOI 10.1016/j.jamcollsurg.2003.10.005

Natsugoe S, 2001, ANN SURG, V234, P613, DOI 10.1097/00000658-200111000-00005

Paweletz N, 2001, NAT REV MOL CELL BIO, V2, P72, DOI 10.1038/35048077

Sakai M, 2006, ONCOGENE, V25, P480, DOI 10.1038/sj.onc.1209051

Schmittgen TD, 2008, NAT PROTOC, V3, P1101, DOI 10.1038/nprot.2008.73

Song YM, 2012, ANN SURG ONCOL, V19, P3606, DOI 10.1245/s10434-012-2419-y

Talsma K, 2012, ANN SURG ONCOL, V19, P2142, DOI 10.1245/s10434-012-2218-5

Tanabe G, 1986, Nihon Geka Gakkai Zasshi, V87, P315

Tao JQ, 2014, TUMOR BIOL, V35, P4389, DOI 10.1007/s13277-013-1578-1

Valverde CM, 2006, CRIT REV ONCOL HEMAT, V59, P128, DOI 10.1016/j.critrevonc.2006.02.001

Yu Y, 2010, WORLD J SURG, V34, P272, DOI 10.1007/s00268-009-0341-7

Zhao WM, 2006, MOL BIOL CELL, V17, P3881, DOI 10.1091/mbc.E06-01-0015

NR 33

TC 20

Z9 20

U1 1

U2 3

PU SPANDIDOS PUBL LTD

PI ATHENS

PA POB 18179, ATHENS, 116 10, GREECE

SN 1792-1074

EI 1792-1082

J9 ONCOL LETT

JI Oncol. Lett.

PD JAN

PY 2017

VL 13

IS 1

BP 236

EP 242

DI 10.3892/ol.2016.5414

PG 7

WC Oncology

WE Science Citation Index Expanded (SCI-EXPANDED)

SC Oncology

GA EJ2CV

UT WOS:000393018400034

PM 28123547

OA Green Submitted, Green Published, gold

DA 2022-05-19

ER

PT J

AU Situ, DR

Wang, JY

Lin, P

Long, H

Zhang, LJ

Rong, TH

Ma, GW

AF Situ, Dongrong

Wang, Junye

Lin, Peng

Long, Hao

Zhang, Lanjun

Rong, Tiehua

Ma, Guowei

TI Do tumor location and grade affect survival in pT2N0M0 esophageal

squamous cell carcinoma?

SO JOURNAL OF THORACIC AND CARDIOVASCULAR SURGERY

LA English

DT Article

ID PROGNOSTIC-FACTORS; RISK-FACTORS; CANCER; SMOKING; CLASSIFICATION;

RESECTION

AB Background: The TNM staging system for esophageal cancer in the seventh edition of the AJCC Cancer Staging Manual incorporates tumor grade and location for staging pT2-3N0M0 esophageal squamous cell carcinoma. Patients with pT2N0M0, classified as stage IIA according to the sixth edition of the AJCC Cancer Staging Manual, can now be classified as stage IB, IIA, or IIB. We discuss whether these changes lead to a better prediction of the prognosis of these patients and aimed to find out other factors to forecast patient prognosis.

Methods: We retrospectively analyzed 317 patients with postoperative pathologic stage T2N0M0 who underwent esophagectomy between 1990 and 2005 at Sun Yat-sen University Cancer Center. We performed univariate and multivariate analyses to identify prognostic factors for survival and used the Kaplan-Meier method to demonstrate the prognostic efficacy of each prognostic factor, including tumor grade and location.

Results: The 5-year overall survival was 57%, with a median survival of 84.5 months (6.94 years). Univariate analysis indicated that age, alcohol consumption, and tumor grade were associated with survival. Multivariate Cox proportional hazard regression analysis revealed that alcohol consumption and tumor grade were independent prognostic factors. Survival analysis using the Kaplan-Meier method demonstrated age, cigarette smoking, alcohol consumption, tumor grade, and location as prognostic factors.

Conclusions: For pT2N0M0 esophageal squamous cell carcinoma, the seventh edition of the AJCC Cancer Staging Manual does not provide a more distinguishable prediction of prognosis compared with the sixth edition. Tumor grade is an independent prognostic factor in patients with pT2N0M0 esophageal squamous cell carcinoma, whereas tumor location is not. Furthermore, alcohol consumption is an independent prognostic factor that may imply a worse prognosis.

C1 [Situ, Dongrong; Wang, Junye; Lin, Peng; Long, Hao; Zhang, Lanjun; Rong, Tiehua; Ma, Guowei] Sun Yat Sen Univ, Ctr Canc, State Key Lab Oncol South China, Guangzhou, Peoples R China.

RP Ma, GW (corresponding author), Sun Yat Sen Univ, Ctr Canc, Dept Thorac Surg, State Key Lab Oncol South China, 651 Dongfengdong Rd, Guangzhou, Guangdong, Peoples R China.

EM gzlmst@yeah.net

FU Fundamental Research Funds for the Central Universities [09YKPY51];

Science and Technology Planning Project of Guangdong Province, China

[2010B31500010]; National High Technology Research and Development

Program of China (863 Program) [2009AA02Z421]

FX This work was supported by the Fundamental Research Funds for the

Central Universities (No. 09YKPY51); the Science and Technology Planning

Project of Guangdong Province, China (No. 2010B31500010); and the

National High Technology Research and Development Program of China (863

Program; No. 2009AA02Z421).

CR Bogoevski D, 2008, ANN SURG, V247, P633, DOI 10.1097/SLA.0b013e3181656d07

Dickson GH, 2001, EUR J SURG ONCOL, V27, P641

Doki Y, 2005, WORLD J SURG, V29, P700, DOI 10.1007/s00268-005-7596-4

Edge S., 2010, AM JOINT COMMITTEE C, VSeventh

Eloubeidi MA, 2002, CANCER-AM CANCER SOC, V95, P1434, DOI 10.1002/cncr.10868

Elsayed H, 2010, INTERACT CARDIOV TH, V11, P65, DOI 10.1510/icvts.2009.223826

Enzinger PC, 2003, NEW ENGL J MED, V349, P2241, DOI 10.1056/NEJMra035010

GAO YT, 1994, INT J CANCER, V58, P192, DOI 10.1002/ijc.2910580208

Greene FL, 2002, AJCC CANC STAGING MA

Hsu PK, 2010, ANN THORAC SURG, V89, P1024, DOI 10.1016/j.athoracsur.2010.01.017

Khan NA, 2011, J CANCER RES THER, V7, P15, DOI 10.4103/0973-1482.80431

Killinger WA, 1996, J THORAC CARDIOV SUR, V111, P935, DOI 10.1016/S0022-5223(96)70367-7

Kountourakis P, 2011, CANCER-AM CANCER SOC, V117, P925, DOI 10.1002/cncr.25651

Lee CH, 2005, INT J CANCER, V113, P475, DOI 10.1002/ijc.20619

Li HH, 2009, J THORAC CARDIOV SUR, V137, P55, DOI 10.1016/j.jtcvs.2008.05.024

Lin CS, 2009, ANN THORAC SURG, V87, P1056, DOI 10.1016/j.athoracsur.2008.11.051

Rice TW, 2009, DIS ESOPHAGUS, V22, P1, DOI 10.1111/j.1442-2050.2008.00901.x

Rice TW, 2007, J THORAC CARDIOV SUR, V133, P317, DOI 10.1016/j.jtcvs.2006.09.023

RODER JD, 1994, BRIT J SURG, V81, P410, DOI 10.1002/bjs.1800810330

Shitara K, 2010, CANCER SCI, V101, P1001, DOI 10.1111/j.1349-7006.2009.01466.x

Sundelof M, 2008, EUR J CANCER, V44, P1566, DOI 10.1016/j.ejca.2008.04.002

Tachibana M, 1999, ARCH SURG-CHICAGO, V134, P50, DOI 10.1001/archsurg.134.1.50

WONG J, 1987, AM J SURG, V153, P18, DOI 10.1016/0002-9610(87)90196-6

NR 23

TC 17

Z9 19

U1 4

U2 9

PU MOSBY-ELSEVIER

PI NEW YORK

PA 360 PARK AVENUE SOUTH, NEW YORK, NY 10010-1710 USA

SN 0022-5223

J9 J THORAC CARDIOV SUR

JI J. Thorac. Cardiovasc. Surg.

PD JUL

PY 2013

VL 146

IS 1

BP 45

EP 51

DI 10.1016/j.jtcvs.2013.01.034

PG 7

WC Cardiac & Cardiovascular Systems; Respiratory System; Surgery

WE Science Citation Index Expanded (SCI-EXPANDED)

SC Cardiovascular System & Cardiology; Respiratory System; Surgery

GA 166UH

UT WOS:000320583300014

PM 23490249

OA Bronze

DA 2022-05-19

ER

PT J

AU Zhu, YL

Li, Q

Gao, JM

Li, JS

He, ZY

Xu, JJ

Lu, XJ

Liu, YM

AF Zhu, Yu-liang

Li, Qun

Gao, Jian-ming

Li, Ji-shi

He, Zhen-yu

Xu, Jun-jie

Lu, Xiao-jun

Liu, Yu-meng

TI A Retrospective Evaluation of Radiotherapy for the Treatment of Local

Esophageal Squamous Cell Carcinoma Recurrence After Initial Complete

Surgical Resection

SO JOURNAL OF INVESTIGATIVE MEDICINE

LA English

DT Article

DE esophageal cancer; recurrence; recurrent esophageal carcinoma; radiation

therapy; squamous cell carcinoma

ID PROGNOSTIC-FACTORS; RADIATION-THERAPY; CANCER; SURGERY;

CHEMORADIOTHERAPY; PATTERN

AB Introduction: Our purpose was to assess the efficacy of radiation therapy in the treatment of locoregional recurrence of esophageal squamous cell carcinoma recurrence after curative resection.

Methods: Patients with local esophageal squamous cell carcinoma recurrence who were treated at the Cancer Prevention and Treatment Center of Sun Yat-sen University from January 1990 to December 2002 and whose initial treatment was complete surgical resection (N = 152) were included. Of the 152 patients, 60 patients received treatment with radiation therapy, and the others did not receive radiation. None of the included patients received chemotherapy or radiotherapy before surgery. The Kaplan-Meier method was used to calculate the cumulative survival rate.

Results: The median survival time was 16.0 +/- 2.7 months for those who received radiotherapy versus 6.5 +/- 1.6 months for those who did not (P < 0.001). The median survival time was greater (22.3 +/- 4.0 months) for patients who receive a dosage greater than 50 Gy compared with those patients who received a dosage of 50 Gy or less (7.7 +/- 0.3 months; P = 0.033). The overall 3- and 5-year survival rates were 16.4% and 10.4%, respectively. The 1-, 2-, and 3- year recurrence-free survival rates were 56.1 +/- 4.3%, 32.9 +/- 4.1%, and 22.0 +/- 3.7%, respectively.

Conclusion: Radiotherapy may help improve the survival rate in patients with local esophageal squamous cell carcinoma recurrence after complete surgical resection who have no history of radiotherapy.

C1 [Zhu, Yu-liang; Li, Qun; Gao, Jian-ming; He, Zhen-yu; Xu, Jun-jie] Sun Yat Sen Univ, Ctr Canc, State Key Lab Oncol S China, Guangzhou 510060, Guangdong, Peoples R China.

[Zhu, Yu-liang; Lu, Xiao-jun; Liu, Yu-meng] Sun Yat Sen Univ, Zhongshan Hosp, Dept Radiotherapy, Zhongshan, Guangdong, Peoples R China.

[Li, Qun; Gao, Jian-ming; He, Zhen-yu; Xu, Jun-jie] Sun Yat Sen Univ, Ctr Canc, Dept Radiotherapy, Guangzhou 510060, Guangdong, Peoples R China.

[Li, Ji-shi] Panyu Peoples Hosp, Dept Clin Oncol, Guangzhou, Guangdong, Peoples R China.

RP Li, Q (corresponding author), Sun Yat Sen Univ, Ctr Canc, State Key Lab Oncol S China, Guangzhou 510060, Guangdong, Peoples R China.

EM Liqun0799@126.com

CR Ando N, 2000, ANN SURG, V232, P225, DOI 10.1097/00000658-200008000-00013

Baxi SH, 2008, J MED IMAG RADIAT ON, V52, P583, DOI 10.1111/j.1440-1673.2008.02023.x

Bhansali MS, 1997, WORLD J SURG, V21, P275, DOI 10.1007/s002689900228

Collard JM, 1995, HEPATO-GASTROENTEROL, V42, P619

Gao Xian-Shu, 2010, Chin J Cancer, V29, P860

Gockel I, 2011, EXPERT REV GASTROENT, V5, P371, DOI [10.1586/EGH.11.33, 10.1586/egh.11.33]

Hongo M, 2009, J GASTROEN HEPATOL, V24, P729, DOI 10.1111/j.1440-1746.2009.05824.x

Hsu PK, 2011, J GASTROINTEST SURG, V15, P558, DOI 10.1007/s11605-011-1458-1

JAMES RD, 1983, BRIT J SURG, V70, P469, DOI 10.1002/bjs.1800700807

KURTZ JM, 1989, CANCER, V63, P1912, DOI 10.1002/1097-0142(19890515)63:10<1912::AID-CNCR2820631007>3.0.CO;2-Y

Mariette C, 2003, CANCER-AM CANCER SOC, V97, P1616, DOI 10.1002/cncr.11228

Natsugoe S, 2006, EJSO, V32, P544, DOI 10.1016/j.ejso.2006.02.014

NCCN GuidelinesTM, 2011, NCCN GUID VERS 2 201

Nemoto K, 2001, RADIOTHER ONCOL, V61, P165, DOI 10.1016/S0167-8140(01)00392-9

Shioyama Y, 2007, JPN J CLIN ONCOL, V37, P918, DOI 10.1093/jjco/hym138

Stahl M, 2009, ANN ONCOL, V20, P32, DOI 10.1093/annonc/mdp121

TAM PC, 1987, ANN SURG, V205, P189, DOI 10.1097/00000658-198702000-00014

Tang Z., 2005, MODERN ONCOLOGY

van Hagen P, 2012, NEW ENGL J MED, V366, P2074, DOI 10.1056/NEJMoa1112088

Yang TS, 1996, AM J CLIN ONCOL-CANC, V19, P337, DOI 10.1097/00000421-199608000-00003

Zhang He-lin, 2009, Zhonghua Zhongliu Zazhi, V31, P541, DOI 10.3760/cma.j.issn.0253-3766.2009.07.015

NR 21

TC 6

Z9 8

U1 0

U2 2

PU BMJ PUBLISHING GROUP

PI LONDON

PA BRITISH MED ASSOC HOUSE, TAVISTOCK SQUARE, LONDON WC1H 9JR, ENGLAND

SN 1081-5589

EI 1708-8267

J9 J INVEST MED

JI J. Invest. Med.

PD JAN

PY 2013

VL 61

IS 1

BP 34

EP 39

PG 6

WC Medicine, General & Internal; Medicine, Research & Experimental

WE Science Citation Index Expanded (SCI-EXPANDED)

SC General & Internal Medicine; Research & Experimental Medicine

GA 058TT

UT WOS:000312657900005

PM 23160184

DA 2022-05-19

ER

PT J

AU Cao, W

Yu, GZ

Lu, Q

Zhang, JL

AF Cao, Wei

Yu, Guozheng

Lu, Qiang

Zhang, Juliang

TI Low expression of N-myc downstream-regulated gene 2 in oesophageal

squamous cell carcinoma correlates with a poor prognosis

SO BMC CANCER

LA English

DT Article

DE NDRG2; Oesophageal squamous cell carcinoma (ESCC); Prognosis

ID NDRG2 EXPRESSION; CANCER; DIFFERENTIATION; SUPPRESSOR; MUTATION; FAMILY;

MEMBER; LIVER; CD24

AB Background: It is currently unclear whether a correlation exists between N-myc downstream-regulated gene 2 (NDRG2) expression and oesophageal squamous cell carcinoma (ESCC). The aim of this study was to examine the underlying clinical significance of NDRG2 expression in ESCC patients and to investigate the effects of NDRG2 up-regulation on ESCC cell growth in vitro and in vivo.

Methods: Immunohistochemistry was used to determine the level of NDRG2 expressions in ESCC tissue, which was then compared to specific clinicopathological features in the patient and tissue specimens. Factors associated with patient survival were analysed. Moreover, the effects of up-regulating NDRG2 expression on the growth of an ESCC cell line were examined by MTT, colony formation, DNA replication activity and nude mouse model assays.

Results: Notably low expression of NDRG2 in ESCC patients was inversely associated with clinical stage, NM classification, histological differentiation and patients' vital status (all P < 0.05). ESCC patients expressing high levels of NDRG2 exhibited a substantially higher 5-year overall survival rate than NDRG2-negative patients. Furthermore, NDRG2 over-expression reduced the proliferation, colony formation and DNA replication activity in ESCC cells, as well as inhibiting the growth of ESCC cells in vivo.

Conclusion: The present experiments demonstrated that NDRG2 may be a diagnostic and prognostic marker in patients with ESCC, and up-regulation of NDRG2 might act as a promising therapeutic strategy for aggressive ESCC.

C1 [Cao, Wei] Fourth Mil Med Univ, Tangdu Hosp, Dept Intervent Radiol, Xian 710038, Shaanxi, Peoples R China.

[Yu, Guozheng] Shaanxi Canc Hosp, Dept Intervent Radiol, Xian 710061, Shaanxi, Peoples R China.

[Lu, Qiang] Fourth Mil Med Univ, Tangdu Hosp, Dept Thorac Surg, Xian 710038, Shaanxi, Peoples R China.

[Zhang, Juliang] Fourth Mil Med Univ, Xijing Hosp, Dept Vasc & Endocrine Surg, Xian 710032, Shaanxi, Peoples R China.

RP Zhang, JL (corresponding author), Fourth Mil Med Univ, Xijing Hosp, Dept Vasc & Endocrine Surg, Xian 710032, Shaanxi, Peoples R China.

EM zhangjuliang75@gmail.com

FU National Natural Science Foundation of China [81170400]

FX This work was supported by grants from the National Natural Science

Foundation of China (No.81170400).

CR Boulkroun S, 2002, J BIOL CHEM, V277, P31506, DOI 10.1074/jbc.M200272200

Brown LM, 2001, AM J EPIDEMIOL, V153, P114, DOI 10.1093/aje/153.2.114

Choi SC, 2003, FEBS LETT, V553, P413, DOI 10.1016/S0014-5793(03)01030-5

Choi SC, 2007, EXP MOL MED, V39, P705, DOI 10.1038/emm.2007.77

Deng YC, 2003, INT J CANCER, V106, P342, DOI 10.1002/ijc.11228

Enzinger PC, 2003, NEW ENGL J MED, V349, P2241, DOI 10.1056/NEJMra035010

Hu XL, 2004, WORLD J GASTROENTERO, V10, P3518

Kamangar F, 2006, J CLIN ONCOL, V24, P2137, DOI 10.1200/JCO.2005.05.2308

Kim A, 2009, CARCINOGENESIS, V30, P927, DOI 10.1093/carcin/bgp072

Kim YJ, 2009, INT J CANCER, V124, P7, DOI 10.1002/ijc.23945

Kim YJ, 2009, CARCINOGENESIS, V30, P598, DOI 10.1093/carcin/bgp047

Lee DC, 2008, CANCER RES, V68, P4210, DOI 10.1158/0008-5472.CAN-07-5040

Liu N, 2007, BIOCHEM BIOPH RES CO, V358, P164, DOI 10.1016/j.bbrc.2007.04.089

Lorentzen A, 2011, BMC CANCER, V11, DOI 10.1186/1471-2407-11-14

Lusis EA, 2005, CANCER RES, V65, P7121, DOI 10.1158/0008-5472.CAN-05-0043

Ma J, 2012, CANCER-AM CANCER SOC, V118, P4105, DOI 10.1002/cncr.27393

Ma J, 2012, PLOS ONE, V7, DOI 10.1371/journal.pone.0032368

Ma J, 2012, MED ONCOL, V29, P119, DOI 10.1007/s12032-010-9774-y

Nichols NR, 2003, ANN NY ACAD SCI, V1007, P349, DOI 10.1196/annals.1286.034

Pickens A, 2003, ANN THORAC SURG, V76, pS1367, DOI 10.1016/S0003-4975(03)01202-5

Piquemal D, 1999, BBA-MOL CELL RES, V1450, P364, DOI 10.1016/S0167-4889(99)00056-7

Qu XH, 2002, MOL CELL BIOCHEM, V229, P35, DOI 10.1023/A:1017934810825

Shon SK, 2009, BIOCHEM BIOPH RES CO, V385, P198, DOI 10.1016/j.bbrc.2009.05.038

Stoner GD, 2001, CARCINOGENESIS, V22, P1737, DOI 10.1093/carcin/22.11.1737

Tong L, 2012, DIS ESOPHAGUS, V25, P72, DOI 10.1111/j.1442-2050.2011.01211.x

vanBelzen N, 1997, LAB INVEST, V77, P85

Yao LB, 2008, ACTA BIOCH BIOPH SIN, V40, P625, DOI 10.1111/j.1745-7270.2008.00434.x

Zhang J, 2006, J BIOL CHEM, V281, P39159, DOI 10.1074/jbc.M605820200

Zheng J, 2011, BMC CANCER, V11, DOI 10.1186/1471-2407-11-251

Zheng J, 2010, ASIAN PAC J CANCER P, V11, P1817

Zhou RH, 2001, GENOMICS, V73, P86, DOI 10.1006/geno.2000.6496

NR 31

TC 12

Z9 15

U1 1

U2 6

PU BMC

PI LONDON

PA CAMPUS, 4 CRINAN ST, LONDON N1 9XW, ENGLAND

SN 1471-2407

J9 BMC CANCER

JI BMC Cancer

PD JUN 24

PY 2013

VL 13

AR 305

DI 10.1186/1471-2407-13-305

PG 9

WC Oncology

WE Science Citation Index Expanded (SCI-EXPANDED)

SC Oncology

GA 173ED

UT WOS:000321060000001

PM 23800335

OA Green Published, gold

DA 2022-05-19

ER

PT J

AU Tang, SH

Qin, JP

Liu, WH

Zheng, XS

Wu, XL

Xu, H

Qiao, LJ

Fan, QS

Zeng, WZ

Jiang, MD

AF Tang, Shanhong

Qin, Jianping

Liu, Weihui

Zheng, Xiushan

Wu, Xiaoling

Xu, Hui

Qiao, Lijuan

Fan, Quanshui

Zeng, Weizheng

Jiang, Mingde

TI Overexpression of p28GANK accelerates the metastasis of oesophageal

squamous cell carcinoma

SO MOLECULAR MEDICINE REPORTS

LA English

DT Article

DE oesophageal squamous cell carcinoma; p28GANK; invasiveness; metastasis

ID COLORECTAL-CANCER; GANKYRIN PROMOTES; EXPRESSION; PROLIFERATION;

DEGRADATION; P53

AB The present study aimed to evaluate the effects of p28GANK expression on the metastasis of oesophageal squamous cell carcinoma (ESCC) tissues and to investigate its roles in the metastasis of highly invasive and non-invasive ESCC cell lines. Quantitative polymerase chain reaction (qPCR) and immunohistochemical analyses were performed to assess p28GANK mRNA and protein expression in ESCC tissues and to analyse its significance in ESCC metastasis. qPCR and western blot analyses were used to detect p28GANK mRNA and protein expression in highly invasive and non-invasive cell lines. Subsequently, lentivirus-mediated p28GANK short interfering RNA (siRNA) was transfected into highly invasive ESCC cells, and Transwell assays were performed to analyse the effects of p28GANK knockdown on their migration and invasion. The mean expression levels of p28GANK mRNA in the ESCC tissues of patients with metastasis were significantly higher than those in the ESCC specimens from patients without metastasis. p28GANK expression in ESCC tissues was correlated with T-stage, lymph node metastasis and lymphatic invasion. The mRNA and protein expression levels of p28GANK were significantly higher in highly invasive cell lines compared with those of matched, non-invasive cell lines. Lentivirus-mediated siRNA knockdown of p28GANK markedly decreased p28GANK expression in EC109-P and EC9706-P cells and supressed the metastasis of ESCC cells in vitro. In conclusion, p28GANK expression was increased in metastatic ESCC tissues and cells, and p28GANK knockdown decreased the metastatic ability of ESCC cells. These results suggested that p28GANK may be a potential therapeutic marker for ESCC metastasis.

C1 [Tang, Shanhong; Qin, Jianping; Wu, Xiaoling; Xu, Hui; Qiao, Lijuan; Zeng, Weizheng; Jiang, Mingde] Chengdu Mil Command, Dept Digest, Gen Hosp, Chengdu 610083, Sichuan, Peoples R China.

[Tang, Shanhong; Fan, Quanshui] Chengdu Mil Command, Prevent & Control Ctr Dis, Chengdu 610083, Sichuan, Peoples R China.

[Liu, Weihui] Chengdu Mil Command, Dept Gen Surg, Gen Hosp, Chengdu 610083, Sichuan, Peoples R China.

[Zheng, Xiushan] Chengdu Mil Command, Dept Thorac Surg, Gen Hosp, Chengdu 610083, Sichuan, Peoples R China.

RP Zeng, WZ (corresponding author), Chengdu Mil Command, Dept Digest, Gen Hosp, 72 Rongdu Rd, Chengdu 610083, Sichuan, Peoples R China.

EM zengweizheng@163.com; 15928956390@163.com

FU National Natural Science Foundations of China [81401993]

FX The present study was supported by a grant from the National Natural

Science Foundations of China (grant no. 81401993, to Tang S).

CR Bai ZF, 2013, CANCER RES, V73, P4548, DOI 10.1158/0008-5472.CAN-12-4586

Bi Q, 2012, PLOS ONE, V7, DOI 10.1371/journal.pone.0040169

Chu YW, 1997, AM J RESP CELL MOL, V17, P353, DOI 10.1165/ajrcmb.17.3.2837

Fu J, 2011, HEPATOLOGY, V53, P181, DOI 10.1002/hep.24015

Gao LC, 2014, MOL MED REP, V9, P1032, DOI 10.3892/mmr.2013.1860

Gong TQ, 2012, CANCER BIOL THER, V13, P606, DOI 10.4161/cbt.19851

Higashitsuji H, 2005, CANCER CELL, V8, P75, DOI 10.1016/j.ccr.2005.06.006

Li JN, 2011, ANTICANCER RES, V31, P2683

Lozano G, 2005, CANCER CELL, V8, P3, DOI 10.1016/j.ccr.2005.06.014

Meng Y, 2010, CANCER LETT, V297, P9, DOI 10.1016/j.canlet.2010.04.019

Mine H, 2013, DIGEST DIS SCI, V58, P2337, DOI 10.1007/s10620-013-2627-8

Mohebbi M, 2008, BMC CANCER, V8, DOI 10.1186/1471-2407-8-137

Ortiz CM, 2008, INT J CANCER, V122, P325, DOI 10.1002/ijc.23106

Qian YW, 2012, GASTROENTEROLOGY, V142, P1547, DOI 10.1053/j.gastro.2012.02.042

Tang SH, 2013, PLOS ONE, V8, DOI 10.1371/journal.pone.0052184

Tang SH, 2010, CANCER BIOL THER, V9, P88, DOI 10.4161/cbt.9.2.10283

Tie J, 2010, PLOS GENET, V6, DOI 10.1371/journal.pgen.1000879

Wang Guowen, 2010, Mol Biol (Mosk), V44, P1010

Xu GH, 2013, DIGEST DIS SCI, V58, P1871, DOI 10.1007/s10620-013-2611-3

Yang Y, 2012, WORLD J SURG ONCOL, V10, DOI 10.1186/1477-7819-10-169

Yu CP, 2009, CARCINOGENESIS, V30, P894, DOI 10.1093/carcin/bgp064

Zhen C, 2013, ONCOGENE, V32, P3452, DOI 10.1038/onc.2012.356

NR 22

TC 2

Z9 2

U1 1

U2 5

PU SPANDIDOS PUBL LTD

PI ATHENS

PA POB 18179, ATHENS, 116 10, GREECE

SN 1791-2997

EI 1791-3004

J9 MOL MED REP

JI Mol. Med. Rep.

PD JUN

PY 2015

VL 11

IS 6

BP 4291

EP 4296

DI 10.3892/mmr.2015.3286

PG 6

WC Oncology; Medicine, Research & Experimental

WE Science Citation Index Expanded (SCI-EXPANDED)

SC Oncology; Research & Experimental Medicine

GA CJ4ZL

UT WOS:000355497100039

PM 25634618

OA Bronze

DA 2022-05-19

ER

PT J

AU Hu, YH

Xie, CY

Yang, H

Ho, JWK

Wen, J

Han, LJ

Chiu, KWH

Fu, JH

Vardhanabhuti, V

AF Hu, Yihuai

Xie, Chenyi

Yang, Hong

Ho, Joshua W. K.

Wen, Jing

Han, Lujun

Chiu, Keith W. H.

Fu, Jianhua

Vardhanabhuti, Varut

TI Assessment of Intratumoral and Peritumoral Computed Tomography Radiomics

for Predicting Pathological Complete Response to Neoadjuvant

Chemoradiation in Patients With Esophageal Squamous Cell Carcinoma

SO JAMA NETWORK OPEN

LA English

DT Article

ID POSITRON-EMISSION-TOMOGRAPHY; LYMPH-NODE METASTASIS; PHASE-III TRIAL;

PREOPERATIVE CHEMORADIOTHERAPY; CANCER; SURGERY; PET/CT; RADIOTHERAPY;

SIGNATURES; ACCURACY

AB This diagnostic study assesses the use of intratumoral and peritumoral computed tomography (CT) radiomics for predicting pathological complete response to neoadjuvant chemoradiation in patients with esophageal squamous cell carcinoma (ESCC).

Question Are peritumoral radiomics features extracted from pretreatment computed tomography images predictive of pathological complete response following neoadjuvant chemoradiation in patients with esophageal squamous cell carcinoma? Findings In this diagnostic study of 231 patients, the developed model integrating intratumoral and peritumoral radiomics features achieved improvement of predictive performance (area under the receiver operating characteristic curve, 0.852) compared with the conventionally constructed model merely using intratumoral radiomics features (area under the receiver operating characteristic curve, 0.730). Meaning Peritumoral radiomics may provide additional predictive value for treatment response estimation in esophageal squamous cell carcinoma and thus benefit individualized therapeutic strategies.

Importance For patients with locally advanced esophageal squamous cell carcinoma, neoadjuvant chemoradiation has been shown to improve long-term outcomes, but the treatment response varies among patients. Accurate pretreatment prediction of response remains an urgent need. Objective To determine whether peritumoral radiomics features derived from baseline computed tomography images could provide valuable information about neoadjuvant chemoradiation response and enhance the ability of intratumoral radiomics to estimate pathological complete response. Design, Setting, and Participants A total of 231 patients with esophageal squamous cell carcinoma, who underwent baseline contrast-enhanced computed tomography and received neoadjuvant chemoradiation followed by surgery at 2 institutions in China, were consecutively included. This diagnostic study used single-institution data between April 2007 and December 2018 to extract radiomics features from intratumoral and peritumoral regions and established intratumoral, peritumoral, and combined radiomics models using different classifiers. External validation was conducted using independent data collected from another hospital during the same period. Radiogenomics analysis using gene expression profile was done in a subgroup of the training set for pathophysiological explanation. Data were analyzed from June to December 2019. Exposures Computed tomography-based radiomics. Main Outcomes and Measures The discriminative performances of radiomics models were measured by area under the receiver operating characteristic curve. Results Among the 231 patients included (192 men [83.1%]; mean [SD] age, 59.8 [8.7] years), the optimal intratumoral and peritumoral radiomics models yielded similar areas under the receiver operating characteristic curve of 0.730 (95% CI, 0.609-0.850) and 0.734 (0.613-0.854), respectively. The combined model was composed of 7 intratumoral and 6 peritumoral features and achieved better discriminative performance, with an area under the receiver operating characteristic curve of 0.852 (95% CI, 0.753-0.951), accuracy of 84.3%, sensitivity of 90.3%, and specificity of 79.5% in the test set. Gene sets associated with the combined model mainly involved lymphocyte-mediated immunity. The association of peritumoral area with response identification might be partially attributed to type I interferon-related biological process. Conclusions and Relevance A combination of peritumoral radiomics features appears to improve the predictive performance of intratumoral radiomics to estimate pathological complete response after neoadjuvant chemoradiation in patients with esophageal squamous cell carcinoma. This study underlines the significant application of peritumoral radiomics to assess treatment response in clinical practice.

C1 [Hu, Yihuai; Yang, Hong; Fu, Jianhua] Sun Yat Sen Univ, Canc Ctr, Dept Thorac Surg, 651 Dongfeng Rd E, Guangzhou 510060, Peoples R China.

[Hu, Yihuai; Yang, Hong; Wen, Jing; Han, Lujun; Fu, Jianhua] Collaborat Innovat Ctr Canc Med, State Key Lab Oncol South China, Guangzhou, Peoples R China.

[Hu, Yihuai; Yang, Hong; Wen, Jing; Fu, Jianhua] Guangdong Esophageal Canc Inst, Guangzhou, Peoples R China.

[Xie, Chenyi; Chiu, Keith W. H.; Vardhanabhuti, Varut] Univ Hong Kong, Li Ka Shing Fac Med, Dept Diagnost Radiol, Hong Kong, Hong Kong, Peoples R China.

[Ho, Joshua W. K.] Univ Hong Kong, Li Ka Shing Fac Med, Sch Biomed Sci, Hong Kong, Hong Kong, Peoples R China.

[Han, Lujun] Sun Yat Sen Univ, Canc Ctr, Dept Med Imaging, Guangzhou, Peoples R China.

RP Vardhanabhuti, V (corresponding author), Univ Hong Kong, Queen Mary Hosp, Li Ka Shing Fac Med, Dept Diagnost Radiol, 102 Pokfulam Rd, Hong Kong, Hong Kong, Peoples R China.; Fu, JH (corresponding author), Sun Yat Sen Univ, Canc Ctr, Dept Thorac Surg, 651 Dongfeng Rd E, Guangzhou 510060, Peoples R China.

EM fu_jh@outlook.com; varv@hku.hk

RI Ho, Joshua W. K./AAK-7759-2020; Chiu, Keith Wan Hang/Z-3985-2019

OI Ho, Joshua W. K./0000-0003-2331-7011; Fu, Jianhua/0000-0002-8451-3705;

Chiu, Keith Wan Hang/0000-0002-7930-1193

FU Health AMP; Medical Collaborative Innovation Project of Guangzhou City,

China [201803040018]; National Natural Science Foundation of China

[81972614, 81871975]; Fundamental Research Funds for the Central

Universities [19ykyjs79]; Hui Pun Hing Memorial Postgraduate Fellowship

FX This work was supported by the Health & Medical Collaborative Innovation

Project of Guangzhou City, China (grant 201803040018), the National

Natural Science Foundation of China (grants 81972614 and 81871975), and

the Fundamental Research Funds for the Central Universities (grant

19ykyjs79). Dr Xie is supported by the Hui Pun Hing Memorial

Postgraduate Fellowship.

CR Beig N, 2019, RADIOLOGY, V290, P783, DOI 10.1148/radiol.2018180910

Berger AC, 2005, J CLIN ONCOL, V23, P4330, DOI 10.1200/JCO.2005.05.017

Beukinga RJ, 2018, RADIOLOGY, V287, P983, DOI 10.1148/radiol.2018172229

Beukinga RJ, 2017, J NUCL MED, V58, P723, DOI 10.2967/jnumed.116.180299

Blom RLGM, 2013, EUR J NUCL MED MOL I, V40, P1500, DOI 10.1007/s00259-013-2468-x

Braman N, 2019, JAMA NETW OPEN, V2, DOI 10.1001/jamanetworkopen.2019.2561

BRAMAN NM, 2017, BREAST CANCER RES, V0019

Bray F, 2018, CA-CANCER J CLIN, V68, P394, DOI 10.3322/caac.21492

Cerfolio RJ, 2005, J THORAC CARDIOV SUR, V129, P1232, DOI 10.1016/j.jtcvs.2004.12.042

Chen YH, 2019, ANN NUCL MED, V33, P657, DOI 10.1007/s12149-019-01380-7

Chirieac LR, 2005, CANCER-AM CANCER SOC, V103, P1347, DOI 10.1002/cncr.20916

DELONG ER, 1988, BIOMETRICS, V44, P837, DOI 10.2307/2531595

Gao Y, 2009, BMC CANCER, V9, DOI 10.1186/1471-2407-9-269

Heneghan HM, 2016, ANN SURG, V264, P831, DOI 10.1097/SLA.0000000000001902

Hung FH, 2019, SCI REP-UK, V9, DOI 10.1038/s41598-019-46707-6

Jiang Y, 2020, ANN ONCOL, V31, P760, DOI 10.1016/j.annonc.2020.03.295

Khorrami M, 2019, REMOTE SENS-BASEL, V11, DOI 10.3390/rs11040377

Kucera Mike, 2016, F1000Res, V5, P1717

Kukar M, 2015, JAMA SURG, V150, P555, DOI 10.1001/jamasurg.2014.3867

Lambin P, 2017, NAT REV CLIN ONCOL, V14, P749, DOI 10.1038/nrclinonc.2017.141

Lazar C, 2013, BRIEF BIOINFORM, V14, P469, DOI 10.1093/bib/bbs037

Li SH, 2012, EUR J CARDIO-THORAC, V42, P958, DOI 10.1093/ejcts/ezs136

Liberzon A, 2015, CELL SYST, V1, P417, DOI 10.1016/j.cels.2015.12.004

Mariette C, 2014, J CLIN ONCOL, V32, P2416, DOI 10.1200/JCO.2013.53.6532

Meistey N, 2007, EUR J CANCER, V43, P1833, DOI 10.1016/j.ejca.2007.05.028

Merico D, 2010, PLOS ONE, V5, DOI 10.1371/journal.pone.0013984

Post AEM, 2018, CLIN CANCER RES, V24, P3397, DOI 10.1158/1078-0432.CCR-17-2551

Rohatgi PR, 2005, CANCER-AM CANCER SOC, V104, P1349, DOI 10.1002/cncr.21346

Rustgi AK, 2014, NEW ENGL J MED, V371, P2499, DOI 10.1056/NEJMra1314530

Shalapour S, 2015, NATURE, V521, P94, DOI 10.1038/nature14395

Shannon P, 2003, GENOME RES, V13, P2498, DOI 10.1101/gr.1239303

Shapiro J, 2015, LANCET ONCOL, V16, P1090, DOI 10.1016/S1470-2045(15)00040-6

Sim CK, 2019, J IMMUNOL RES, V2019, DOI 10.1155/2019/7596786

Stassi G, 2003, CANCER RES, V63, P6784

Subramanian A, 2005, P NATL ACAD SCI USA, V102, P15545, DOI 10.1073/pnas.0506580102

Sun CX, 2019, EBIOMEDICINE, V46, P160, DOI 10.1016/j.ebiom.2019.07.049

Sun QC, 2020, FRONT ONCOL, V10, DOI 10.3389/fonc.2020.00053

Tan S, 2013, INT J RADIAT ONCOL, V85, P1375, DOI 10.1016/j.ijrobp.2012.10.017

Tandberg DJ, 2018, INT J RADIAT ONCOL, V102, P1002, DOI 10.1016/j.ijrobp.2018.07.187

Tepper J, 2008, J CLIN ONCOL, V26, P1086, DOI 10.1200/JCO.2007.12.9593

van Griethuysen JJM, 2017, CANCER RES, V77, pE104, DOI 10.1158/0008-5472.CAN-17-0339

van Hagen P, 2012, NEW ENGL J MED, V366, P2074, DOI 10.1056/NEJMoa1112088

van Heijl M, 2011, EJSO-EUR J SURG ONC, V37, P1064, DOI 10.1016/j.ejso.2011.09.004

Vickers AJ, 2008, BMC MED INFORM DECIS, V8, DOI 10.1186/1472-6947-8-53

Wang SF, 2018, BREAST CANCER RES TR, V168, P703, DOI 10.1007/s10549-017-4638-1

Wang WM, 2016, CELL, V165, P1092, DOI 10.1016/j.cell.2016.04.009

Wu J, 2017, RADIOLOGY, V285, P401, DOI 10.1148/radiol.2017162823

Wu QX, 2019, RADIOTHER ONCOL, V138, P141, DOI 10.1016/j.radonc.2019.04.035

Yang H, 2018, J CLIN ONCOL, V36, P2796, DOI 10.1200/JCO.2018.79.1483

Yang ZN, 2019, J RADIAT RES, V60, P538, DOI 10.1093/jrr/rrz027

Yuan H, 2016, NUCL MED COMMUN, V37, P947, DOI 10.1097/MNM.0000000000000527

ZHAO BS, 2016, SCI REP UK, V0006

NR 52

TC 18

Z9 18

U1 4

U2 11

PU AMER MEDICAL ASSOC

PI CHICAGO

PA 330 N WABASH AVE, STE 39300, CHICAGO, IL 60611-5885 USA

SN 2574-3805

J9 JAMA NETW OPEN

JI JAMA Netw. Open

PD SEP 10

PY 2020

VL 3

IS 9

AR e2015927

DI 10.1001/jamanetworkopen.2020.15927

PG 14

WC Medicine, General & Internal

WE Science Citation Index Expanded (SCI-EXPANDED)

SC General & Internal Medicine

GA NR9EO

UT WOS:000571866100003

PM 32910196

OA Green Published, gold

DA 2022-05-19

ER

PT J

AU Yang, HX

Feng, W

Wei, JC

Zeng, TS

Li, ZD

Zhang, LJ

Lin, P

Luo, RZ

He, JH

Fu, JH

AF Yang, H. X.

Feng, W.

Wei, J. C.

Zeng, T. S.

Li, Z. D.

Zhang, L. J.

Lin, P.

Luo, R. Z.

He, J. H.

Fu, J. H.

TI Support vector machine-based nomogram predicts postoperative distant

metastasis for patients with oesophageal squamous cell carcinoma

SO BRITISH JOURNAL OF CANCER

LA English

DT Article

DE nomogram; oesophageal squamous cell carcinoma; surgery; metastasis

ID LYMPH-NODE METASTASIS; NEOADJUVANT CHEMORADIOTHERAPY; CANCER;

EXPRESSION; SURVIVAL; ADENOCARCINOMA; PROFILES; SIGNATURE

AB Background: We aim to develop effective models for predicting postoperative distant metastasis for oesophageal squamous cell carcinoma (OSCC) for the purpose of guiding tailored therapy.

Methods: We used data from two centres to establish training (n = 319) and validation (n = 164) cohorts. All patients underwent curative surgical treatment. The clinicopathological features and 23 immunomarkers detected by immunohistochemistry were involved for variable selection. We constructed eight support vector machine (SVM)-based nomograms (SVM1-SVM4 and SVM1'-SVM4'). The nomogram constructed with the training cohort was tested further with the validation cohort.

Results: The outcome of the SVM1 model in predicting postoperative distant metastasis was as follows: sensitivity, 44.7%; specificity, 90.9%; positive predictive value, 81.0%; negative predictive value, 65.6%; and overall accuracy, 69.5%. The corresponding outcome of the SVM2 model was as follows: 44.7%, 92.1%, 82.9%, 65.9%, and 70.1%, respectively. The corresponding outcome of the SVM3 model was as follows: 55.3%, 93.2%, 87.5%, 70.7%, and 75.6%, respectively. The SVM4 model was the most effective nomogram in prediction, and the corresponding outcome was as follows: 56.6%, 97.7%, 95.6%, 72.3%, and 78.7%, respectively. Similar results were observed in SVM1', SVM2', SVM3', and SVM4', respectively.

Conclusion: The SVM-based models integrating clinicopathological features and molecular markers as variables are helpful in selecting the patients of OSCC with high risk of postoperative distant metastasis.

C1 [Yang, H. X.; Zhang, L. J.; Lin, P.; Fu, J. H.] Sun Yat Sen Univ, Ctr Canc, Dept Thorac Surg, Guangzhou 510060, Guangdong, Peoples R China.

[Yang, H. X.; Zhang, L. J.; Lin, P.; Luo, R. Z.; He, J. H.; Fu, J. H.] State Key Lab Oncol South China, Guangzhou 510060, Guangdong, Peoples R China.

[Feng, W.] Sun Yat Sen Univ, Dept Sci Comp & Comp Applicat, Sch Math & Computat Sci, Guangzhou 510275, Guangdong, Peoples R China.

[Wei, J. C.] Linzhou Oesophageal Canc Hosp, Dept Thorac Surg, Linzhou City 456592, Henan Province, Peoples R China.

[Zeng, T. S.] S China Normal Univ, Sch Math Sci, Dept Informat & Computat Sci, Guangzhou 510631, Guangdong, Peoples R China.

[Li, Z. D.] SUNY Buffalo, Dept Comp Sci & Engn, Buffalo, NY 14260 USA.

[Luo, R. Z.; He, J. H.] Sun Yat Sen Univ, Ctr Canc, Dept Pathol, Guangzhou 510060, Guangdong, Peoples R China.

RP Fu, JH (corresponding author), Sun Yat Sen Univ, Ctr Canc, Dept Thorac Surg, Guangzhou 510060, Guangdong, Peoples R China.

EM j_hfu@yahoo.com.cn

FU Young Scientists Fund of the National Natural Science Foundation of

China [81000935]; 46th China Postdoctoral Science Foundation

[20090460815]; Young Teachers' Training Program of Sun Yat-sen

University [11ykpy59]; Guangdong Provincial Science and Technology

Planning Foundation [2011B031800179]; Young Teachers' Key Program of Sun

Yat-sen University; Guangzhou Science and Technology Planning Foundation

[12A562061978]

FX We thank Ms MQ Lin for patient follow-up, and Dr XY Jiao and Dr JL Li

from the Linzhou Oesophageal Cancer Hospital for helping us in the

collection of paraffin-embedded tissues. This study was partly supported

by the Young Scientists Fund of the National Natural Science Foundation

of China (81000935); the 46th China Postdoctoral Science Foundation

(20090460815); the Young Teachers' Training Program of Sun Yat-sen

University (11ykpy59); the Guangdong Provincial Science and Technology

Planning Foundation (2011B031800179); the Young Teachers' Key Program of

Sun Yat-sen University; and the Guangzhou Science and Technology

Planning Foundation (12A562061978).

CR [Anonymous], 2012, NCCN CLIN PRACT GUID

Chen HY, 2007, NEW ENGL J MED, V356, P11, DOI 10.1056/NEJMoa060096

Chiang AC, 2007, NEW ENGL J MED, V359, P2814

Gebski V, 2007, LANCET ONCOL, V8, P226, DOI 10.1016/S1470-2045(07)70039-6

Gerlinger M, 2012, NEW ENGL J MED, V366, P883, DOI 10.1056/NEJMoa1113205

Guo Y, 2008, CANCER RES, V68, P26, DOI 10.1158/0008-5472.CAN-06-4418

Gupta GP, 2007, NATURE, V446, P765, DOI 10.1038/nature05760

Hayes DN, 2006, J CLIN ONCOL, V24, P5079, DOI 10.1200/JCO.2005.05.1748

Huang E, 2003, LANCET, V361, P1590, DOI 10.1016/S0140-6736(03)13308-9

Jin HL, 2009, WORLD J GASTROENTERO, V15, P5983, DOI 10.3748/wjg.15.5983

Kan T, 2004, ANN SURG ONCOL, V11, P1070, DOI 10.1245/ASO.2004.03.007

Lagarde SM, 2007, BRIT J SURG, V94, P1361, DOI 10.1002/bjs.5832

Lagarde SM, 2008, ANN SURG, V248, P1006, DOI 10.1097/SLA.0b013e318190a0a2

Law S, 2002, J GASTROEN HEPATOL, V17, P374, DOI 10.1046/j.1440-1746.2002.02728.x

Liu R, 2007, NEW ENGL J MED, V356, P217, DOI 10.1056/NEJMoa063994

Mathe EA, 2009, CLIN CANCER RES, V15, P6192, DOI 10.1158/1078-0432.CCR-09-1467

Mofidi R, 2006, EJSO, V32, P533, DOI 10.1016/j.ejso.2006.02.020

Nguyen DX, 2007, NAT REV GENET, V8, P341, DOI 10.1038/nrg2101

Potti A, 2006, NEW ENGL J MED, V355, P570, DOI 10.1056/NEJMoa060467

Rice TW, 2009, DIS ESOPHAGUS, V22, P1, DOI 10.1111/j.1442-2050.2008.00901.x

Rice TW, 2009, AJCC CANC STAGING MA, P103

Sato F, 2005, CANCER-AM CANCER SOC, V103, P1596, DOI 10.1002/cncr.20938

Takeno S, 2007, ANN SURG ONCOL, V14, P1431, DOI 10.1245/s10434-006-9286-3

Tamoto E, 2004, CLIN CANCER RES, V10, P3629, DOI 10.1158/1078-0432.CCR-04-0048

Vapnik V, 1998, NATURE STAT LEARNING, P138

Wang YX, 2005, LANCET, V365, P671, DOI 10.1016/S0140-6736(05)17947-1

Wijnhoven BPL, 2007, ANN SURG, V245, P717, DOI 10.1097/01.sla.0000251703.35919.02

Yanaihara N, 2006, CANCER CELL, V9, P189, DOI 10.1016/j.ccr.2006.01.025

Zhu ZH, 2009, J CLIN ONCOL, V27, P1091, DOI 10.1200/JCO.2008.16.6991

NR 29

TC 16

Z9 17

U1 1

U2 11

PU NATURE PUBLISHING GROUP

PI LONDON

PA MACMILLAN BUILDING, 4 CRINAN ST, LONDON N1 9XW, ENGLAND

SN 0007-0920

EI 1532-1827

J9 BRIT J CANCER

JI Br. J. Cancer

PD SEP 3

PY 2013

VL 109

IS 5

BP 1109

EP 1116

DI 10.1038/bjc.2013.379

PG 8

WC Oncology

WE Science Citation Index Expanded (SCI-EXPANDED)

SC Oncology

GA 214WP

UT WOS:000324168500005

OA Green Published, hybrid

DA 2022-05-19

ER

PT J

AU Wang, Z

Yang, H

Zhang, RS

Luo, B

Xu, BC

Zhu, ZH

Lin, P

AF Wang, Zhen

Yang, Han

Zhang, Rusi

Luo, Bin

Xu, Bingchen

Zhu, Zhihua

Lin, Peng

TI MEOX2 serves as a novel biomarker associated with macrophage

infiltration in oesophageal squamous cell carcinoma and other digestive

system carcinomas

SO AUTOIMMUNITY

LA English

DT Article

DE MEOX2; macrophage; oesophageal squamous cell carcinoma; digestive system

carcinomas; immune infiltration

ID TUMOR-ASSOCIATED MACROPHAGES; CANCER; EXPRESSION; GENE; PROGRESSION;

PROGNOSIS; RESOURCE; SURVIVAL; TARGETS

AB Background Oesophageal squamous cell carcinoma (ESCC) is a malignant tumour of the digestive system that is associated with high morbidity and mortality rates worldwide. With the increased use of immunotherapy in cancer treatment, there is an urgent need to elucidate the immune-related mechanisms in ESCC and other digestive system carcinomas. Methods In our study, single-sample gene set enrichment analysis (ssGSEA) was first performed to analyse the expression profile downloaded from the NCBI Gene Expression Omnibus (GEO) database. Then, via a series of bioinformatic analyses, including the Mann-Whitney test, weighted gene co-expression network analysis (WGCNA), functional enrichment analysis and differentially expressed genes (DEGs) analysis, we identified target immunocytes and related genes. Finally, we validated the results with the TIMER database. Results Our analyses showed that the numbers of infiltrating macrophages were obviously higher in advanced stages in ESCC compared with other types of immunocytes. MEOX2 was identified as a biomarker correlated with macrophage infiltration in ESCC and other types of digestive system carcinomas. And MEOX2 expression was strongly associated with the mRNA expression of colony-stimulating factor 1 (CSF-1) and CSF-1 receptor (CSF-1R) in these kinds of carcinomas. Conclusion We speculated that MEOX2 could facilitate macrophage infiltration via CSF-1/CSF-1R signalling in ESCC and other kinds of digestive system carcinomas, and MEOX2 might serve as a novel target in prospective tumour immunotherapies.

C1 [Wang, Zhen; Yang, Han; Zhang, Rusi; Luo, Bin; Xu, Bingchen; Zhu, Zhihua; Lin, Peng] Sun Yat Sen Univ, State Key Lab Oncol South China, Collaborat Innovat Ctr Canc Med, Dept Thorac Oncol,Canc Ctr, 651 Dongfengdong, Guangzhou 510060, Guangdong, Peoples R China.

RP Zhu, ZH; Lin, P (corresponding author), Sun Yat Sen Univ, State Key Lab Oncol South China, Collaborat Innovat Ctr Canc Med, Dept Thorac Oncol,Canc Ctr, 651 Dongfengdong, Guangzhou 510060, Guangdong, Peoples R China.

EM zhuzhh@sysucc.org.cn; linpeng@sysucc.org.cn

CR Ammannagari N, 2018, J GASTROINTEST ONCOL, V9, P196, DOI 10.21037/jgo.2017.06.12

Armas-Lopez L, 2017, ONCOTARGET, V8, P67056, DOI 10.18632/oncotarget.17715

Brown GR, 2015, NUCLEIC ACIDS RES, V43, pD36, DOI 10.1093/nar/gku1055

Cannarile MA, 2017, J IMMUNOTHER CANCER, V5, DOI 10.1186/s40425-017-0257-y

Charoentong P, 2017, CELL REP, V18, P248, DOI 10.1016/j.celrep.2016.12.019

Dhupar R, 2017, ANN THORAC SURG, V103, P1340, DOI 10.1016/j.athoracsur.2016.12.011

Ferlay J, 2019, INT J CANCER, V144, P1941, DOI 10.1002/ijc.31937

Hanzelmann S, 2013, BMC BIOINFORMATICS, V14, DOI 10.1186/1471-2105-14-7

Holderried TAW, 2019, CLIN EPIGENETICS, V11, DOI 10.1186/s13148-019-0752-8

Huang TX, 2019, CANCER COMMUN, V39, DOI 10.1186/s40880-019-0427-z

Nguyen KQ, 2013, AUTOIMMUNITY, V46, P294, DOI 10.3109/08916934.2013.794515

Komohara Y, 2016, ADV DRUG DELIVER REV, V99, P180, DOI 10.1016/j.addr.2015.11.009

Langfelder P, 2008, BMC BIOINFORMATICS, V9, DOI 10.1186/1471-2105-9-559

Li J, 2019, FRONT GENET, V10, DOI 10.3389/fgene.2019.01119

Li TW, 2020, NUCLEIC ACIDS RES, V48, pW509, DOI 10.1093/nar/gkaa407

Lin EY, 2006, CANCER RES, V66, P11238, DOI 10.1158/0008-5472.CAN-06-1278

Lin EY, 2001, J EXP MED, V193, P727, DOI 10.1084/jem.193.6.727

Mankoo BS, 1999, NATURE, V400, P69, DOI 10.1038/21892

Mantovani A, 2017, NAT REV CLIN ONCOL, V14, P399, DOI 10.1038/nrclinonc.2016.217

Mascher M, 2019, NAT GENET, V51, P1076, DOI 10.1038/s41588-019-0443-6

Nishimura J, 2019, ESOPHAGUS-TOKYO, V16, P333, DOI 10.1007/s10388-019-00669-w

Ostuni R, 2015, TRENDS IMMUNOL, V36, P229, DOI 10.1016/j.it.2015.02.004

Patel S, 2005, CANCER RES, V65, P1414, DOI 10.1158/0008-5472.CAN-04-3431

Pollard JW, 2004, NAT REV CANCER, V4, P71, DOI 10.1038/nrc1256

Ries CH, 2014, CANCER CELL, V25, P846, DOI 10.1016/j.ccr.2014.05.016

Ritchie ME, 2015, NUCLEIC ACIDS RES, V43, DOI 10.1093/nar/gkv007

Rustgi AK, 2014, NEW ENGL J MED, V371, P2499, DOI 10.1056/NEJMra1314530

Schumacher K, 2001, CANCER RES, V61, P3932

Shigeoka M, 2013, CANCER SCI, V104, P1112, DOI 10.1111/cas.12188

Tanaka Y, 2015, PLOS ONE, V10, DOI 10.1371/journal.pone.0143804

Tang HX, 2018, AUTOIMMUNITY, V51, P191, DOI 10.1080/08916934.2018.1477133

Tap WD, 2015, NEW ENGL J MED, V373, P428, DOI 10.1056/NEJMoa1411366

Valcourt U, 2007, MOL ONCOL, V1, P55, DOI 10.1016/j.molonc.2007.02.002

Vlaicu P, 2013, BMC CANCER, V13, DOI 10.1186/1471-2407-13-197

Watanabe M, 2020, SURG TODAY, V50, P12, DOI 10.1007/s00595-019-01878-7

Wu A, 2010, NEURO-ONCOLOGY, V12, P1113, DOI 10.1093/neuonc/noq082

Wyckoff JB, 2007, CANCER RES, V67, P2649, DOI 10.1158/0008-5472.CAN-06-1823

Yagi T, 2019, EUR J CANCER, V111, P38, DOI 10.1016/j.ejca.2019.01.018

Yamamoto K, 2020, CANCER SCI, V111, P1103, DOI 10.1111/cas.14328

Yoshihara K, 2013, NAT COMMUN, V4, DOI 10.1038/ncomms3612

Zhou YY, 2019, NAT COMMUN, V10, DOI 10.1038/s41467-019-09234-6

Zhu XD, 2008, J CLIN ONCOL, V26, P2707, DOI 10.1200/JCO.2007.15.6521

NR 42

TC 2

Z9 2

U1 2

U2 3

PU TAYLOR & FRANCIS LTD

PI ABINGDON

PA 2-4 PARK SQUARE, MILTON PARK, ABINGDON OR14 4RN, OXON, ENGLAND

SN 0891-6934

EI 1607-842X

J9 AUTOIMMUNITY

JI Autoimmunity

PD AUG 18

PY 2021

VL 54

IS 6

BP 373

EP 383

DI 10.1080/08916934.2021.1919880

EA JUN 2021

PG 11

WC Immunology

WE Science Citation Index Expanded (SCI-EXPANDED)

SC Immunology

GA UI7KV

UT WOS:000664936200001

PM 34160343

OA hybrid

DA 2022-05-19

ER

PT J

AU Rao, WQ

Lin, Z

Liu, S

Zhang, ZH

Xie, QW

Chen, HL

Lin, X

Chen, YM

Yang, HM

Yu, KL

Hu, ZJ

AF Rao, Wenqing

Lin, Zheng

Liu, Shuang

Zhang, Zhihui

Xie, Qianwen

Chen, Huilin

Lin, Xi

Chen, Yuanmei

Yang, Huimin

Yu, Kaili

Hu, Zhijian

TI Association between alcohol consumption and oesophageal microbiota in

oesophageal squamous cell carcinoma

SO BMC MICROBIOLOGY

LA English

DT Article

DE Oesophageal squamous cell carcinoma (ESCC); Alcohol; Microbiota

AB Background Microbiota has been reported to play a role in cancer patients. Nevertheless, little is known about the association between alcohol consumption and resultant changes in the diversity and composition of oesophageal microbiota in oesophageal squamous cell carcinoma (ESCC). Methods We performed a hospital-based retrospective study of 120 patients with pathologically diagnosed primary ESCC. The relevant information for all study participants were collected through a detailed questionnaire. The differences in adjacent tissues between non-drinkers and drinkers were explored using 16S rRNA gene sequencing. Raw sequencing data were imported into QIIME 2 to analyse the diversity and abundance of microbiota. Linear discriminant analysis effect size (LEfSe) and unconditional logistic regression were performed to determine the bacterial taxa that were associated with drinking. Results The Shannon diversity index and Bray-Curtis distance of oesophageal microbiota were significantly different among drinkers(P < 0.05). The alcohol-related bacteria were primarily from the orders Clostridiales, Gemellales and Pasteurellales, family Clostridiaceae, Lanchnospiraceae, Helicobacteraceae, Alcaligenaceae, Bacteroidaceae, Pasteurellaceae and Gemellaceae; genus Clostridium, Helicobacter, Catonella, Bacteroides, Bacillus, Moraxella, and Bulleidia; and species B. moorei and longum (genus Bifidobacterium). In addition, the diversity and abundance of these microbiota were observed to be affected by the age, residential districts of the patients, and sampling seasons. Moreover, the higher the frequency and years of alcohol consumption, the lower was the relative abundance of genus Catonella that was observed. Conclusion Alcohol consumption is associated with alterations in both the diversity and composition the of the oesophageal microbiota in ESCC patients.

C1 [Rao, Wenqing; Lin, Zheng; Liu, Shuang; Zhang, Zhihui; Xie, Qianwen; Yang, Huimin; Yu, Kaili; Hu, Zhijian] Fujian Med Univ, Dept Epidemiol & Hlth Stat, Fujian Prov Key Lab Environm Factors & Canc, Sch Publ Hlth, Fuzhou 350122, Peoples R China.

[Chen, Huilin] Anxi Cty Hosp, Dept Radiat Oncol, Quanzhou 352400, Peoples R China.

[Lin, Xi] Fujian Med Univ, Dept Stat Off, Zhangzhou Affiliated Hosp, Zhangzhou 363000, Peoples R China.

[Chen, Yuanmei] Fujian Med Univ, Dept Thorac Surg, Fujian Prov Canc Hosp, Fuzhou 350014, Peoples R China.

[Hu, Zhijian] Fujian Med Univ, Key Lab, Minist Educ Gastrointestinal Canc, Fuzhou 350122, Peoples R China.

RP Hu, ZJ (corresponding author), Fujian Med Univ, Dept Epidemiol & Hlth Stat, Fujian Prov Key Lab Environm Factors & Canc, Sch Publ Hlth, Fuzhou 350122, Peoples R China.; Hu, ZJ (corresponding author), Fujian Med Univ, Key Lab, Minist Educ Gastrointestinal Canc, Fuzhou 350122, Peoples R China.

EM huzhijian@fjmu.edu.cn

FU National Key R&D Program of China [2017YFC0907100]; Medical Innovation

project of Fujian Province; Central government-led local science and

technology development special project [2020L3009]

FX Funding was obtained from the National Key R&D Program of China

(No.2017YFC0907100), Medical Innovation project of Fujian Province

(No.2018-CX-38), Central government-led local science and technology

development special project (2020L3009).

CR [Anonymous], 2017, LANCET, V390, P2215, DOI [10.1016/S0140-6736(17)32868-4, 10.1200/JCO.2017.76.1155]

Bajaj JS, 2019, NAT REV GASTRO HEPAT, V16, P235, DOI 10.1038/s41575-018-0099-1

Bolyen E, 2019, NAT BIOTECHNOL, V37, P852, DOI 10.1038/s41587-019-0209-9

Bray F, 2018, CA-CANCER J CLIN, V68, P394, DOI 10.3322/caac.21492

Bull-Otterson L, 2013, PLOS ONE, V8, DOI 10.1371/journal.pone.0053028

Capurso G, 2017, BEST PRACT RES CL GA, V31, P579, DOI 10.1016/j.bpg.2017.10.006

Chandel Siddhartha, 2017, Natl J Maxillofac Surg, V8, P106, DOI 10.4103/njms.NJMS_36_17

Chen WQ, 2016, CA-CANCER J CLIN, V66, P115, DOI 10.3322/caac.21338

Chen YF, 2020, AGING-US, V12, P4778, DOI 10.18632/aging.102872

Davenport ER, 2014, PLOS ONE, V9, DOI 10.1371/journal.pone.0090731

Duan YF, 2019, FISH SHELLFISH IMMUN, V88, P142, DOI 10.1016/j.fsi.2019.02.047

Elamin EE, 2013, NUTR REV, V71, P483, DOI 10.1111/nure.12027

Fan XZ, 2018, MICROBIOME, V6, DOI 10.1186/s40168-018-0448-x

Gopalakrishnan V, 2018, CANCER CELL, V33, P570, DOI 10.1016/j.ccell.2018.03.015

He Y, 2018, NAT MED, V24, P1532, DOI 10.1038/s41591-018-0164-x

Queipo-Ortuno MI, 2012, AM J CLIN NUTR, V95, P1323, DOI 10.3945/ajcn.111.027847

Ji BT, 2002, EUR J CANCER PREV, V11, P237, DOI 10.1097/00008469-200206000-00007

Kazuoka T, 2007, EXTREMOPHILES, V11, P257, DOI 10.1007/s00792-006-0034-1

Kumagai S, 2015, EUR J CLIN MICROBIOL, V34, P1839, DOI 10.1007/s10096-015-2421-y

Lagergren J, 2017, LANCET, V390, P2383, DOI 10.1016/S0140-6736(17)31462-9

Li XY, 2019, EUR J EPIDEMIOL, V34, P753, DOI 10.1007/s10654-019-00530-5

Liu HL, 2018, NUTRIENTS, V10, DOI 10.3390/nu10091298

Liu S, 2019, BMC COMPLEM ALTERN M, V19, DOI 10.1186/s12906-019-2770-7

[刘双 Liu Shuang], 2019, [中国公共卫生, China Journal of Public Health], V35, P731

Liu YF, 2018, J MED MICROBIOL, V67, P1058, DOI 10.1099/jmm.0.000754

Llopis M, 2016, GUT, V65, P830, DOI 10.1136/gutjnl-2015-310585

Matthews D, 2018, INT J GEN MED, V11, P451, DOI 10.2147/IJGM.S184406

Meroni M, 2019, INT J MOL SCI, V20, DOI 10.3390/ijms20184568

Millwood IY, 2013, INT J EPIDEMIOL, V42, P816, DOI 10.1093/ije/dyt078

Moy KA, 2010, CANCER EPIDEM BIOMAR, V19, P2287, DOI 10.1158/1055-9965.EPI-10-0362

Mutlu E, 2009, ALCOHOL CLIN EXP RES, V33, P1836, DOI 10.1111/j.1530-0277.2009.01022.x

Mutlu EA, 2012, AM J PHYSIOL-GASTR L, V302, pG966, DOI 10.1152/ajpgi.00380.2011

Nardone G, 2017, LANCET GASTROENTEROL, V2, P298, DOI 10.1016/S2468-1253(16)30108-X

Nasrollahzadeh D, 2015, SCI REP-UK, V5, DOI 10.1038/srep08820

Oakley BB, 2018, POULTRY SCI, V97, P3635, DOI 10.3382/ps/pey214

Pelucchi C, 2012, CLIN MICROBIOL INFEC, V18, P1, DOI 10.1111/j.1469-0691.2012.03766.x

Rampelli S, 2020, MSYSTEMS, V5, DOI 10.1128/mSystems.00124-20

Rezasoltani S, 2019, IRAN J MICROBIOL, V11, P1, DOI 10.18502/ijm.v11i1.696

Rinninella E, 2019, NUTRIENTS, V11, DOI 10.3390/nu11102393

Samiei H, 2019, IMMUNOL RES, V67, P212, DOI 10.1007/s12026-019-09079-7

Segata N, 2011, GENOME BIOL, V12, DOI 10.1186/gb-2011-12-6-r60

Sender R, 2016, PLOS BIOL, V14, DOI 10.1371/journal.pbio.1002533

Shao DT, 2019, CANCER-AM CANCER SOC, V125, P3993, DOI 10.1002/cncr.32403

Sun BH, 2018, AM J PRIMATOL, V80, DOI 10.1002/ajp.22880

Szabo G, 1999, ALCOHOL ALCOHOLISM, V34, P830, DOI 10.1093/alcalc/34.6.830

Szabo G, 1996, ALCOHOL CLIN EXP RES, V20, P900, DOI 10.1111/j.1530-0277.1996.tb05269.x

Tang C, 2018, FOOD FUNCT, V9, P937, DOI [10.1039/c7fo01302g, 10.1039/C7FO01302G]

Thevaranjan N, 2017, CELL HOST MICROBE, V21, P455, DOI 10.1016/j.chom.2017.03.002

Tsigos I, 1998, EUR J BIOCHEM, V254, P356, DOI 10.1046/j.1432-1327.1998.2540356.x

Tsuruya A, 2016, SCI REP-UK, V6, DOI 10.1038/srep27923

Wang HJ, 2010, WORLD J GASTROENTERO, V16, P1304, DOI 10.3748/wjg.v16.i11.1304

Xia CF, 2019, TOB CONTROL, V28, P669, DOI 10.1136/tobaccocontrol-2018-054583

NR 52

TC 0

Z9 0

U1 1

U2 1

PU BMC

PI LONDON

PA CAMPUS, 4 CRINAN ST, LONDON N1 9XW, ENGLAND

SN 1471-2180

J9 BMC MICROBIOL

JI BMC Microbiol.

PD MAR 6

PY 2021

VL 21

IS 1

AR 73

DI 10.1186/s12866-021-02137-x

PG 12

WC Microbiology

WE Science Citation Index Expanded (SCI-EXPANDED)

SC Microbiology

GA QS5OC

UT WOS:000625948500001

PM 33673801

OA Green Published, gold

DA 2022-05-19

ER

PT J

AU Liu, YF

Lin, Z

Lin, YY

Chen, YM

Peng, XE

He, F

Liu, S

Yan, SY

Huang, LP

Lu, WT

Xiang, ZS

Hu, ZJ

AF Liu, Yanfang

Lin, Zheng

Lin, Yingying

Chen, Yuanmei

Peng, Xian-e

He, Fei

Liu, Shuang

Yan, Siyou

Huang, Liping

Lu, Wanting

Xiang, Zhisheng

Hu, Zhijian

TI Streptococcus and Prevotella are associated with the prognosis of

oesophageal squamous cell carcinoma

SO JOURNAL OF MEDICAL MICROBIOLOGY

LA English

DT Article

DE oesophageal squamous cell carcinoma; 16S rRNA; Streptococcus and

Prevotella; prognosis

ID RIBOSOMAL-RNA SEQUENCES; CANCER STATISTICS; MICROBIOTA; INFLAMMATION;

INFECTION; BACTERIA; PERFORMANCE; HEALTH

AB Purpose. Oesophageal squamous cell carcinoma (ESCC) is the dominant type of oesophageal cancer among the East Asian population. The role of ESCC tissue bacteria in neoplastic progression has not been fully elucidated. Our goal was to uncover different bacterial communities in pathological staging grouping of ESCC and to identify microorganisms that could predict the likelihood of prognosis.

Methodology. Tissue samples were obtained from 45 patients and assessed using 16S ribosomal RNA gene sequencing. Significant bacteria were selected to perform survival analysis and evaluate prognostic biomarker.

Results/Key findings. We observed variations in the abundance of oesophageal flora among different pathological characteristics of ESCC. Phylum Bacteroidetes, Firmicutes and Spirochaetes showed significantly higher relative abundances among N+ (positive lymph node) patients when compared to N- (negative lymph node) controls, whereas Proteobacteria showed lower abundances in N+ patients. Both genera Prevotella and Treponema were more abundant in the N+ group. In regard to T stage, the abundance of only Streptococcus in T3-4 was significantly higher than that in T1-2, while the other genera showed no significance. On multivariable analysis adjusted for the effects of standard clinicopathological features, combined Streptococcus and Prevotella abundance retained its association with unfavourable survival (hazard ratio, 6.094; 95% confidence interval, 1.072-34.646; P=0.042), suggesting that this may be an independent prognostic indicator for ESCC.

Conclusion. Combined Streptococcus and Prevotella abundance is regarded as an independent species prognostic biomarker in ESCC patients.

C1 [Liu, Yanfang; Lin, Zheng; Lin, Yingying; Peng, Xian-e; He, Fei; Liu, Shuang; Yan, Siyou; Huang, Liping; Lu, Wanting; Xiang, Zhisheng; Hu, Zhijian] Fujian Med Univ, Sch Publ Hlth, Dept Epidemiol & Hlth Stat, Fujian Prov Key Lab Environm Factors & Canc, Fuzhou 350108, Fujian, Peoples R China.

[Lin, Zheng; Peng, Xian-e; He, Fei; Hu, Zhijian] Fujian Med Univ, Key Lab, Minist Educ Gastrointestinal Canc, 1 Xueyuan Rd, Fuzhou 350108, Fujian, Peoples R China.

[Chen, Yuanmei] Fujian Med Univ, Dept Thorac Surg, Fujian Prov Canc Hosp, Fuzhou 350014, Fujian, Peoples R China.

RP Hu, ZJ (corresponding author), Fujian Med Univ, Sch Publ Hlth, Dept Epidemiol & Hlth Stat, Fujian Prov Key Lab Environm Factors & Canc, Fuzhou 350108, Fujian, Peoples R China.; Hu, ZJ (corresponding author), Fujian Med Univ, Key Lab, Minist Educ Gastrointestinal Canc, 1 Xueyuan Rd, Fuzhou 350108, Fujian, Peoples R China.

EM huzhijian@fjmu.edu.cn

OI Lin, Zheng/0000-0001-5242-716X

FU National Key R&D Program of China [2017YFC0907100]; Nature Science

Foundation of Fujian Province [2015J01305]; Professor Academic

Development Foundation of Fujian Medical University [JS14005]; Science

and Technology Project of Anxi Tieguanyin Group Co., Ltd. Fujian

[2013B013]

FX This research was supported by the following grants: National Key R&D

Program of China (No. 2017YFC0907100), Nature Science Foundation of

Fujian Province (No. 2015J01305), Professor Academic Development

Foundation of Fujian Medical University (No. JS14005) and Science and

Technology Project of Anxi Tieguanyin Group Co., Ltd. Fujian (No.

2013B013).

CR Anahtar MN, 2015, IMMUNITY, V42, P965, DOI 10.1016/j.immuni.2015.04.019

[Anonymous], 1994, IARC MONOGR EVAL CAR, V61, P177

Burke CM, 2016, PEERJ, V4, DOI 10.7717/peerj.2492

Chen WQ, 2016, CA-CANCER J CLIN, V66, P115, DOI 10.3322/caac.21338

Cover TL, 2013, GUT MICROBES, V4, P482, DOI 10.4161/gmic.26262

Cywes C, 2001, NATURE, V414, P648, DOI 10.1038/414648a

Deans DAC, 2006, BRIT J CANCER, V95, P1568, DOI 10.1038/sj.bjc.6603446

Di Pilato V, 2016, ANN NY ACAD SCI, V1381, P21, DOI 10.1111/nyas.13127

Dillon SM, 2014, MUCOSAL IMMUNOL, V7, P983, DOI 10.1038/mi.2013.116

Downes J, 2005, INT J SYST EVOL MICR, V55, P1551, DOI 10.1099/ijs.0.63634-0

Edgar RC, 2010, BIOINFORMATICS, V26, P2460, DOI 10.1093/bioinformatics/btq461

Fernandez-Canigia L, 2015, ANAEROBE, V33, P8, DOI 10.1016/j.anaerobe.2015.01.007

Gao XY, 2014, PLOS ONE, V9, DOI 10.1371/journal.pone.0101229

Goh CE, 2016, ARTHRITIS RHEUMATOL, V68, P2384, DOI 10.1002/art.39724

Gosmann C, 2017, IMMUNITY, V46, P29, DOI 10.1016/j.immuni.2016.12.013

Grivennikov SI, 2013, SEMIN IMMUNOPATHOL, V35, P229, DOI 10.1007/s00281-012-0352-6

Guerrero-Preston R, 2016, ONCOTARGET, V7, P51320, DOI 10.18632/oncotarget.9710

Gursoy M, 2017, ANAEROBE, V47, P89, DOI 10.1016/j.anaerobe.2017.04.008

Huttenhower C, 2012, NATURE, V486, P207, DOI 10.1038/nature11234

Johansson I, 2016, J DENT RES, V95, P80, DOI 10.1177/0022034515609554

Joseph R, 2013, RHEUMATOL INT, V33, P103, DOI 10.1007/s00296-011-2284-1

KAWAMURA Y, 1995, INT J SYST BACTERIOL, V45, P406, DOI 10.1099/00207713-45-2-406

Larsen JM, 2017, IMMUNOLOGY, V151, P363, DOI 10.1111/imm.12760

Lau SKP, 2012, J CLIN MICROBIOL, V50, P2239, DOI 10.1128/JCM.00128-12

Li Y, 2015, INT J CLIN EXP MED, V8, P5310

Louis P, 2014, NAT REV MICROBIOL, V12, P661, DOI 10.1038/nrmicro3344

Lozupone C, 2005, APPL ENVIRON MICROB, V71, P8228, DOI 10.1128/AEM.71.12.8228-8235.2005

Mager DL, 2005, J TRANSL MED, V3, DOI 10.1186/1479-5876-3-27

Moffatt CE, 2011, MOL ORAL MICROBIOL, V26, P365, DOI 10.1111/j.2041-1014.2011.00624.x

Morita E, 2003, CANCER SCI, V94, P492, DOI 10.1111/j.1349-7006.2003.tb01471.x

Nardone G, 2015, UNITED EUR GASTROENT, V3, P255, DOI 10.1177/2050640614566846

Nasrollahzadeh D, 2015, SCI REP-UK, V5, DOI 10.1038/srep08820

Nistal E, 2015, FRONT ONCOL, V5, DOI 10.3389/fonc.2015.00220

Parks DH, 2014, BIOINFORMATICS, V30, P3123, DOI 10.1093/bioinformatics/btu494

Parsonnet J, 1995, ENVIRON HEALTH PERSP, V103, P263, DOI 10.2307/3432323

Pinato DJ, 2014, BRIT J CANCER, V110, P1930, DOI 10.1038/bjc.2014.145

Reimers LL, 2016, J INFECT DIS, V214, P1361, DOI 10.1093/infdis/jiw374

Sasaki H, 1998, CANCER RES, V58, P2991

Scher JU, 2014, CURR OPIN RHEUMATOL, V26, P424, DOI 10.1097/BOR.0000000000000076

Schwarz-Linek U, 2003, NATURE, V423, P177, DOI 10.1038/nature01589

Si J, 2017, CELL HOST MICROBE, V21, P97, DOI 10.1016/j.chom.2016.11.010

Squier C A, 2001, J Natl Cancer Inst Monogr, P7

Thomas AM, 2016, FRONT CELL INFECT MI, V6, DOI 10.3389/fcimb.2016.00179

Torre LA, 2015, CA-CANCER J CLIN, V65, P87, DOI 10.3322/caac.21262

Vazquez-Castellanos JF, 2015, MUCOSAL IMMUNOL, V8, P760, DOI 10.1038/mi.2014.107

Verstraelen H, 2016, PEERJ, V4, DOI 10.7717/peerj.1602

Wang K, 2016, SCI REP-UK, V6, DOI 10.1038/srep22943

Wang Q, 2007, APPL ENVIRON MICROB, V73, P5261, DOI 10.1128/AEM.00062-07

Winther M, 2015, ACTA ONCOL, V54, P1582, DOI 10.3109/0284186X.2015.1064161

Yang LY, 2009, GASTROENTEROLOGY, V137, P588, DOI 10.1053/j.gastro.2009.04.046

Ye Y, 2013, CLIN MICROBIOL INFEC, V19, pE559, DOI 10.1111/1469-0691.12287

NR 51

TC 22

Z9 26

U1 1

U2 10

PU MICROBIOLOGY SOC

PI LONDON

PA CHARLES DARWIN HOUSE, 12 ROGER ST, LONDON WC1N 2JU, ERKS, ENGLAND

SN 0022-2615

EI 1473-5644

J9 J MED MICROBIOL

JI J. Med. Microbiol.

PD AUG

PY 2018

VL 67

IS 8

BP 1058

EP 1068

DI 10.1099/jmm.0.000754

PG 11

WC Microbiology

WE Science Citation Index Expanded (SCI-EXPANDED)

SC Microbiology

GA GO9XV

UT WOS:000440462400008

PM 29923815

OA Bronze

DA 2022-05-19

ER

PT J

AU Lagergren, J

Mattson, F

AF Lagergren, Jesper

Mattson, Fredrik

TI Diverging Trends in Recent Population-Based Survival Rates in

Oesophageal and Gastric Cancer

SO PLOS ONE

LA English

DT Article

ID CARDIA CANCER; ADENOCARCINOMAS; SWEDEN; SEX

AB Background: Survival trends in oesophageal and gastric cancer need to be updated. A nationwide Swedish population-based study in 1961-2009 was based on registry data.

Methodology/Principal Findings: Relative survival rate, i.e. the ratio of the observed to the expected survival, adjusted for age, sex, and calendar period, and presented with 95% confidence intervals (CI), was the main outcome measure. The expected survival was calculated using the corresponding Swedish general population with no exclusions. The relative survival rates in oesophageal and gastric cardia adenocarcinoma have improved since the 1990s (p for trend <0.001), but not in oesophageal squamous cell carcinoma or gastric non-cardia adenocarcinoma. The relative 5-year survival rates during the two recent periods 1990-1999 and 2000-2008 were 12.5% (95% CI 10.1%-14.9%) and 10.3% (95% CI 8.5-12.0%) for oesophageal squamous cell carcinoma, 12.5% (95% CI 10.1%-14.9%) and 14.6% (95% CI 12.6-16.6%) for oesophageal adenocarcinoma, 11.1% (95% CI 9.6%-12.6%) and 14.3% (95% CI 12.3-16.3%) for gastric cardia adenocarcinoma, and 20.2% (95% CI 19.2%-21.1%) and 19.0% (95% CI 17.7-20.2%) for gastric non-cardia adenocarcinoma. The 3-year survival in tumour stage III in 2004-2008 was about 25% for all four tumour types.

Conclusions/Significance: The survival in oesophageal and cardia adenocarcinoma is increasing, but the lack of such increase in oesophageal squamous cell carcinoma and gastric non-cardia adenocarcinoma is a concern.

C1 [Lagergren, Jesper; Mattson, Fredrik] Karolinska Inst, Dept Mol Med & Surg, Stockholm, Sweden.

[Lagergren, Jesper] Kings Coll London, Div Canc Studies, London, England.

RP Lagergren, J (corresponding author), Karolinska Inst, Dept Mol Med & Surg, Stockholm, Sweden.

EM jesper.lagergren@ki.se

OI Lagergren, Jesper/0000-0002-5143-5448

FU Swedish Research Council [80749601]

FX Funding was provided by the Swedish Research Council (ID 80749601),

(http://www.vr.se/inenglish.4.12fff4451215cbd83e4800015152.html). The

funders had no role in study design, data collection and analysis,

decision to publish, or preparation of the manuscript.

CR Barlow L, 2009, ACTA ONCOL, V48, P27, DOI 10.1080/02841860802247664

Brown LM, 2008, J NATL CANCER I, V100, P1184, DOI 10.1093/jnci/djn211

Cook MB, 2009, BRIT J CANCER, V101, P855, DOI 10.1038/sj.bjc.6605246

Crane SJ, 2008, MAYO CLIN PROC, V83, P1087, DOI 10.4065/83.10.1087

CUTLER S J, 1958, J Chronic Dis, V8, P699, DOI 10.1016/0021-9681(58)90126-7

Davies AR, 2006, DIS ESOPHAGUS, V19, P496, DOI 10.1111/j.1442-2050.2006.00629.x

Ekstrom AM, 1999, JNCI-J NATL CANCER I, V91, P786, DOI 10.1093/jnci/91.9.786

Greene FL, 2002, J SURG ONCOL, V80, P119, DOI 10.1002/jso.10114

Lagergren J, 2011, INT J CANCER, V129, P513, DOI 10.1002/ijc.25701

Lindblad M, 2006, ANN SURG, V243, P479, DOI 10.1097/01.sla.0000205825.34452.43

Ludvigsson JF, 2009, EUR J EPIDEMIOL, V24, P659, DOI 10.1007/s10654-009-9350-y

Sant M, 2009, EUR J CANCER, V45, P931, DOI 10.1016/j.ejca.2008.11.018

Stitzenberg KB, 2010, ANN SURG ONCOL, V17, P2824, DOI 10.1245/s10434-010-1159-0

Sundelof M, 2002, INT J CANCER, V99, P751, DOI 10.1002/ijc.10420

Sundelof M, 2008, EUR J CANCER, V44, P1566, DOI 10.1016/j.ejca.2008.04.002

Whitson BA, 2010, J THORAC CARDIOV SUR, V139, P43, DOI 10.1016/j.jtcvs.2009.04.011

Wouters MWJM, 2009, ANN SURG ONCOL, V16, P1789, DOI 10.1245/s10434-009-0458-9

NR 17

TC 36

Z9 36

U1 1

U2 5

PU PUBLIC LIBRARY SCIENCE

PI SAN FRANCISCO

PA 1160 BATTERY STREET, STE 100, SAN FRANCISCO, CA 94111 USA

SN 1932-6203

J9 PLOS ONE

JI PLoS One

PD JUL 18

PY 2012

VL 7

IS 7

AR e41352

DI 10.1371/journal.pone.0041352

PG 6

WC Multidisciplinary Sciences

WE Science Citation Index Expanded (SCI-EXPANDED)

SC Science & Technology - Other Topics

GA 975YY

UT WOS:000306548900112

PM 22815994

OA Green Published, gold, Green Submitted

DA 2022-05-19

ER

PT J

AU Liu, X

Wang, X

Lin, S

Yuan, J

Yu, ITS

AF Liu, X.

Wang, X.

Lin, S.

Yuan, J.

Yu, I. T-S

TI Dietary patterns and oesophageal squamous cell carcinoma: a systematic

review and meta-analysis

SO BRITISH JOURNAL OF CANCER

LA English

DT Article

DE dietary pattern; oesophageal squamous cell carcinoma; systematic review;

meta-analysis

ID VITAMIN MINERAL SUPPLEMENTATION; AERODIGESTIVE TRACT CANCERS; BODY-MASS

INDEX; ALCOHOL-DRINKING; COLORECTAL-CANCER; NUTRIENT PATTERNS; RISK;

LINXIAN; FRUIT; VEGETABLES

AB Background/Objective: Dietary patterns, which represent a complex integration of food and nutrients, have been used to explore the association between dietary factors and the risk of oesophageal cancer. However, the association remains unclear. This systematic review was performed to evaluate the relationship between dietary patterns and oesophageal squamous cell carcinoma (ESCC) by pooling available data from existing studies.

Methods: Pertinent articles published up to the end of 2013 were systematically searched and retrieved. The most common dietary patterns with high loadings of foods/nutrients were selected. Adjusted odds ratios (ORs) were derived by comparing the highest with the lowest categories of dietary pattern scores and by using a random-effect model. Heterogeneity was tested using I-2 statistic.

Results: From nine available case-control studies, in which smoking and other confounding factors were considered, three most common dietary patterns were selected: western pattern, healthy pattern, and drinker/alcohol pattern. Healthy pattern was significantly associated with a decreased risk of ESCC (OR = 0.36, 95% confidence interval (CI): 0.23, 0.49); drinker/alcohol pattern was related to a significantly increased risk (OR = 2.34, 95% CI: 1.22, 3.45), while no significant association with western pattern was observed (OR = 1.29, 95% CI: 0.83, 1.75).

Conclusions: Based on available studies, though limited in number, this meta-analysis suggests that some dietary patterns may be associated with the risk of ESCC.

C1 [Liu, X.; Wang, X.; Lin, S.; Yuan, J.; Yu, I. T-S] Chinese Univ Hong Kong, Prince Wales Hosp, JC Sch Publ Hlth & Primary Care, Div Occupat & Environm Hlth,F Sch Publ Hlth 4, Shatin, Hong Kong, Peoples R China.

RP Wang, X (corresponding author), Chinese Univ Hong Kong, Prince Wales Hosp, JC Sch Publ Hlth & Primary Care, Div Occupat & Environm Hlth,F Sch Publ Hlth 4, Shatin, Hong Kong, Peoples R China.

EM xrwang@cuhk.edu.hk

RI Yu, Ignatius Tak Sun/A-9936-2008

FU World Cancer Research Fund, International [2010/240]

FX This study was supported by World Cancer Research Fund, International

(Grant No. 2010/240).

CR Bagnardi V, 2013, ANN ONCOL, V24, P301, DOI 10.1093/annonc/mds337

Bahmanyar S, 2006, NUTR CANCER, V54, P171, DOI 10.1207/s15327914nc5402_3

Bertuccio P, 2013, ANN ONCOL, V24, P1450, DOI 10.1093/annonc/mdt108

Bosetti C, 2003, CANCER EPIDEM BIOMAR, V12, P1091

Bravi F, 2012, ANN ONCOL, V23, P3024, DOI 10.1093/annonc/mds197

Bravi F, 2012, ANN ONCOL, V23, P765, DOI 10.1093/annonc/mdr295

Brennan SF, 2010, AM J CLIN NUTR, V91, P1294, DOI 10.3945/ajcn.2009.28796

Choi Y, 2013, WORLD J GASTROENTERO, V19, P1020, DOI 10.3748/wjg.v19.i7.1020

De Stefani E, 2008, ANTICANCER RES, V28, P2499

De Stefani E, 2008, NUTR CANCER, V60, P188, DOI 10.1080/01635580701630487

De Stefani E, 2012, NUTR CANCER, V64, P294, DOI 10.1080/01635581.2012.648299

De Stefani E, 2009, INT J CANCER, V124, P1391, DOI 10.1002/ijc.24035

Dong LM, 2008, JAMA-J AM MED ASSOC, V299, P2423, DOI 10.1001/jama.299.20.2423

Engel LS, 2003, J NATL CANCER I, V95, P1404, DOI 10.1093/jnci/djg047

Erickson KL, 2002, AM J CLIN NUTR, V75, P5, DOI 10.1093/ajcn/75.1.5

Freedman ND, 2007, INT J CANCER, V121, P2753, DOI 10.1002/ijc.22993

Hajizadeh B, 2012, DIS ESOPHAGUS, V25, P442, DOI 10.1111/j.1442-2050.2011.01272.x

Hajizadeh B, 2010, PUBLIC HEALTH NUTR, V13, P1107, DOI 10.1017/S1368980010000145

Higgins J, 2012, COCHRANE DB SYST REV, DOI 10.1002/14651858.ED000049

Hu FB, 2002, CURR OPIN LIPIDOL, V13, P3, DOI 10.1097/00041433-200202000-00002

Ibiebele TI, 2012, BRIT J NUTR, V107, P1207, DOI 10.1017/S0007114511004247

Islami F, 2011, INT J CANCER, V129, P2473, DOI 10.1002/ijc.25885

Jacques PF, 2001, AM J CLIN NUTR, V73, P1, DOI 10.1093/ajcn/73.1.1

Jemal A, 2011, CA-CANCER J CLIN, V61, P69, DOI [10.3322/caac.20107, 10.3322/caac.20115]

Jessri M, 2011, ANN NUTR METAB, V59, P166, DOI 10.1159/000334334

LI JY, 1993, J NATL CANCER I, V85, P1492, DOI 10.1093/jnci/85.18.1492

Liu J, 2013, INT J CANCER, V133, P473, DOI 10.1002/ijc.28024

Magalhaes B, 2012, EUR J CANCER PREV, V21, P15, DOI 10.1097/CEJ.0b013e3283472241

Martinez ME, 1998, AM J EPIDEMIOL, V148, P17, DOI 10.1093/oxfordjournals.aje.a009552

Mayne ST, 2001, CANCER EPIDEM BIOMAR, V10, P1055

MORABIA A, 1990, AM J CLIN NUTR, V52, P933, DOI 10.1093/ajcn/52.5.933

Oze I, 2011, JPN J CLIN ONCOL, V41, P677, DOI 10.1093/jjco/hyr026

Palladino-Davis AG, 2015, DIS ESOPHAGUS, V28, P59, DOI 10.1111/dote.12097

Petitti D., 2000, METAANALYSIS DECISIO

Qiao YL, 2009, JNCI-J NATL CANCER I, V101, P507, DOI 10.1093/jnci/djp037

RAO M, 1994, CANCER EPIDEM BIOMAR, V3, P277

Reedy J, 2010, AM J EPIDEMIOL, V171, P479, DOI 10.1093/aje/kwp393

Renehan AG, 2008, LANCET, V371, P569, DOI 10.1016/S0140-6736(08)60269-X

Reszka E, 2006, BRIT J NUTR, V96, P609, DOI 10.1079/BJN20061878

Sakata K, 2005, J EPIDEMIOL, V15, pS212, DOI 10.2188/jea.15.S212

Salehi M, 2013, NUTR REV, V71, P257, DOI 10.1111/nure.12028

Seitz HK, 2007, NAT REV CANCER, V7, P599, DOI 10.1038/nrc2191

Silvera SAN, 2011, ANN EPIDEMIOL, V21, P543, DOI 10.1016/j.annepidem.2010.11.019

Smith M, 2008, INT J CANCER, V122, P1604, DOI 10.1002/ijc.23198

STEINMETZ KA, 1991, CANCER CAUSE CONTROL, V2, P427, DOI 10.1007/BF00054304

Sterne JAC, 2001, J CLIN EPIDEMIOL, V54, P1046, DOI 10.1016/S0895-4356(01)00377-8

Stroup DF, 2000, JAMA-J AM MED ASSOC, V283, P2008, DOI 10.1001/jama.283.15.2008

Terry P, 2000, INT J CANCER, V87, P750, DOI 10.1002/1097-0215(20000901)87:5&lt;750::AID-IJC19&gt;3.0.CO;2-6

Toh Y, 2010, INT J CLIN ONCOL, V15, P135, DOI 10.1007/s10147-010-0057-6

Wells GA, 2019, NEWCASTLE OTTAWA SCA

World Cancer Research Fund; American Institute for Cancer Research, 2007, FOOD NUTR PHYS ACT P

Wright ME, 2007, CANCER, V109, P891, DOI 10.1002/cncr.22482

Wu J, 2013, NUTRITION, V29, P1310, DOI 10.1016/j.nut.2013.04.010

Wu M, 2011, INT J CANCER, V128, P2147, DOI 10.1002/ijc.25532

Yusof AS, 2012, ASIAN PAC J CANCER P, V13, P4713, DOI 10.7314/APJCP.2012.13.9.4713

NR 55

TC 34

Z9 34

U1 0

U2 9

PU SPRINGERNATURE

PI LONDON

PA CAMPUS, 4 CRINAN ST, LONDON, N1 9XW, ENGLAND

SN 0007-0920

EI 1532-1827

J9 BRIT J CANCER

JI Br. J. Cancer

PD MAY 27

PY 2014

VL 110

IS 11

BP 2785

EP 2795

DI 10.1038/bjc.2014.172

PG 11

WC Oncology

WE Science Citation Index Expanded (SCI-EXPANDED)

SC Oncology

GA AI8HB

UT WOS:000337150600018

PM 24714753

OA hybrid, Green Published

DA 2022-05-19

ER

PT J

AU Ni, WJ

Yu, SF

Xiao, ZF

Zhou, ZM

Chen, DF

Feng, QF

Liang, J

Lv, JM

Gao, SG

Mao, YS

Xue, Q

Sun, KL

Liu, XY

Fang, DK

Li, J

Wang, DL

Zhao, J

Gao, YS

AF Ni, Wenjie

Yu, Shufei

Xiao, Zefen

Zhou, Zongmei

Chen, Dongfu

Feng, Qinfu

Liang, Jun

Lv, Jima

Gao, Shugeng

Mao, Yousheng

Xue, Qi

Sun, Kelin

Liu, Xiangyang

Fang, Dekang

Li, Jian

Wang, Dali

Zhao, Jun

Gao, Yushun

TI Postoperative Adjuvant Therapy Versus Surgery Alone for Stage IIB-III

Esophageal Squamous Cell Carcinoma: A Phase III Randomized Controlled

Trial

SO ONCOLOGIST

LA English

DT Article; Early Access

DE Esophageal neoplasm; Surgery; Radiation therapy; Chemotherapy;

Efficiency

ID INTENSITY-MODULATED RADIOTHERAPY; RADIATION-THERAPY; CURATIVE RESECTION;

PREOPERATIVE CHEMORADIOTHERAPY; IMPROVED SURVIVAL; CANCER; RECURRENCE;

CHEMORADIATION; CHEMOTHERAPY; NUMBER

AB Background Retrospective studies have shown that adjuvant treatment improves survival of patients with stage IIB-III esophageal squamous cell carcinoma, but there is no evidence from prospective trials so far. Materials and Methods Patients with pathological stage IIB-III esophageal squamous cell carcinoma were randomly assigned to receive surgery alone (SA), postoperative radiotherapy (PORT), or postoperative concurrent chemoradiotherapy (POCRT). PORT patients received 54 Gy in 27 fractions; the POCRT group received 50.4 Gy in 28 fractions, plus concurrent chemotherapy with paclitaxel (135-150 mg/m(2)) and cisplatin or nedaplatin (50-75 mg/m(2)) every 28 days. The primary endpoint was disease-free survival (DFS), and the secondary endpoint was overall survival (OS). Results A total of 172 patients were enrolled (SA, n = 54; PORT, n = 54; POCRT, n = 64). The 3-year DFS was significantly better in PORT/POCRT patients than in SA patients (53.8% vs. 36.7%; p = .020); the 3-year OS was also better in PORT/POCRT patients (63.9% vs. 48.0%; p = .025). The 3-year DFS for SA, PORT, and POCRT patients were 36.7%, 50.0%, 57.3%, respectively (p = .048). The 3-year OS for SA, PORT, and POCRT patients were 48.0%, 60.8%, 66.5%, respectively (p = .048). Conclusion PORT/POCRT (especially POCRT) may significantly improve DFS and OS in stage IIB-III esophageal squamous cell carcinoma. Implications for Practice The results of this phase III study indicated that postoperative radiotherapy/postoperative concurrent chemoradiotherapy (PORT/POCRT) could significantly improve disease-free survival and overall survival in stage IIB-III esophageal squamous cell carcinoma compared with surgery alone with acceptable toxicities. In-field and out-of-field recurrences were comparable between the POCRT and PORT groups, which demonstrates the rationality and safety of the radiation field used in this study. The postoperative regimens in this trial might be accepted as standard treatment options for pathological stage IIB-III esophageal cancer. Larger sample size prospective randomized trials to identify the value are warranted.

C1 [Ni, Wenjie; Xiao, Zefen; Zhou, Zongmei; Chen, Dongfu; Feng, Qinfu; Liang, Jun; Lv, Jima] Chinese Acad Med Sci & Peking Union Med Coll, Dept Radiat Oncol, Natl Clin Res Ctr Cancer, Natl Canc Ctr Canc Hosp, 17 South Panjiayuan Lane, Beijing 100021, Peoples R China.

[Gao, Shugeng; Mao, Yousheng; Xue, Qi; Sun, Kelin; Liu, Xiangyang; Fang, Dekang; Li, Jian; Wang, Dali; Zhao, Jun; Gao, Yushun] Chinese Acad Med Sci & Peking Union Med Coll, Dept Thorac Surg, Natl Clin Res Ctr Cancer, Natl Canc Ctr Canc Hosp, Beijing, Peoples R China.

[Ni, Wenjie] Capital Med Univ, Beijing Shijitan Hosp, Dept Radiat Oncol, Beijing, Peoples R China.

[Yu, Shufei] Capital Med Univ, Beijing Chaoyang Hosp, Dept Radiotherapy, Beijing, Peoples R China.

RP Xiao, ZF (corresponding author), Chinese Acad Med Sci & Peking Union Med Coll, Dept Radiat Oncol, Natl Clin Res Ctr Cancer, Natl Canc Ctr Canc Hosp, 17 South Panjiayuan Lane, Beijing 100021, Peoples R China.

EM xiaozefen@sina.com

FU Capital Fund for Health Improvement and Research [2016-2-4021]; Youth

Fund of Beijing Shijitan Hospital [2020-q12]

FX We thank all the patients who participated in this trial, all

participating investigators who devote their time and passion in the

implementation of this study. This study was approved by the Ethics

Committee of Cancer Institute and Hospital, Chinese Academy of Medical

Sciences, Beijing (Approval number: 14-090/880). All patients provided

written informed consent. The study was conducted in accordance with the

Declaration of Helsinki. This work was supported by the Capital Fund for

Health Improvement and Research (grant number 2016-2-4021) and Youth

Fund of Beijing Shijitan Hospital (grant number 2020-q12). The

manuscript has been peer reviewed by the funding body. The funding

source has no role in study design, data collection, analysis,

interpretation, the writing of the manuscript, or the decision to submit

the current study.

CR Bray F, 2018, CA-CANCER J CLIN, V68, P394, DOI 10.3322/caac.21492

Chen JQ, 2013, INT J RADIAT ONCOL, V86, P671, DOI 10.1016/j.ijrobp.2013.03.026

Chen JQ, 2012, INT J RADIAT ONCOL, V82, P475, DOI 10.1016/j.ijrobp.2010.08.037

Chen JQ, 2010, ANN THORAC SURG, V90, P435, DOI 10.1016/j.athoracsur.2010.04.002

Chen WQ, 2016, CA-CANCER J CLIN, V66, P115, DOI 10.3322/caac.21338

Chen Y, 2019, J CLIN ONCOL, V37, P1695, DOI 10.1200/JCO.18.02122

Cooper JS, 1999, JAMA-J AM MED ASSOC, V281, P1623, DOI 10.1001/jama.281.17.1623

Ferlay J, 2019, INT J CANCER, V144, P1941, DOI 10.1002/ijc.31937

FOK M, 1993, SURGERY, V113, P138

HERSKOVIC A, 1992, NEW ENGL J MED, V326, P1593, DOI 10.1056/NEJM199206113262403

Hsu PK, 2014, ANN THORAC SURG, V97, P1734, DOI 10.1016/j.athoracsur.2013.12.041

Hsu PK, 2011, J GASTROINTEST SURG, V15, P558, DOI 10.1007/s11605-011-1458-1

Hwang JY, 2016, ANN SURG, V264, P100, DOI 10.1097/SLA.0000000000001410

Kelly RJ, 2021, NEW ENGL J MED, V384, P1191, DOI 10.1056/NEJMoa2032125

KUNATH U, 1984, DEUT MED WOCHENSCHR, V109, P450, DOI 10.1055/s-2008-1069212

Lu JC, 2013, CHINESE J CANCER RES, V25, P549, DOI 10.3978/j.issn.1000-9604.2013.10.05

Miyata H, 2011, ANN SURG ONCOL, V18, P3353, DOI 10.1245/s10434-011-1747-7

Ni W J, 2019, Zhonghua Zhong Liu Za Zhi, V41, P415, DOI 10.3760/cma.j.issn.0253-3766.2019.06.004

Ni WJ, 2020, BMC CANCER, V20, DOI 10.1186/s12885-020-6592-2

Oppedijk V, 2014, J CLIN ONCOL, V32, P385, DOI 10.1200/JCO.2013.51.2186

Schreiber D, 2010, J THORAC ONCOL, V5, P244, DOI 10.1097/JTO.0b013e3181c5e34f

Shridhar R, 2013, J GASTROINTEST SURG, V17, P1339, DOI 10.1007/s11605-013-2192-7

Song T, 2020, CANCER MANAG RES, V12, P1631, DOI 10.2147/CMAR.S232930

TENIERE P, 1991, SURG GYNECOL OBSTET, V173, P123

van Hagen P, 2012, NEW ENGL J MED, V366, P2074, DOI 10.1056/NEJMoa1112088

Wang ZW, 2014, NEOPLASMA, V61, P732, DOI 10.4149/neo_2014_089

Wong AT, 2017, ANN SURG, V265, P1146, DOI 10.1097/SLA.0000000000001825

Worni M, 2012, J AM COLL SURGEONS, V215, P643, DOI 10.1016/j.jamcollsurg.2012.07.006

Xiao ZF, 2005, INT J RADIAT ONCOL, V62, P82, DOI 10.1016/j.ijrobp.2004.08.046

Xiao ZF, 2003, ANN THORAC SURG, V75, P331, DOI 10.1016/S0003-4975(02)04401-6

Xu YP, 2013, RADIAT ONCOL, V8, DOI 10.1186/1748-717X-8-116

Yeh HL, 2012, FORMOS J SURG, V45, P172, DOI 10.1016/j.fjs.2012.10.001

Yu SF, 2019, RADIOTHER ONCOL, V140, P159, DOI 10.1016/j.radonc.2019.06.020

Zhang WC, 2015, THORAC CANCER, V6, P597, DOI 10.1111/1759-7714.12228

Zhang WC, 2015, ONCOL RES TREAT, V38, P97, DOI 10.1159/000375391

ZIEREN HU, 1995, WORLD J SURG, V19, P444, DOI 10.1007/BF00299187

Zou BW, 2020, THORAC CANCER, V11, P631, DOI 10.1111/1759-7714.13307

NR 37

TC 0

Z9 0

U1 0

U2 0

PU WILEY

PI HOBOKEN

PA 111 RIVER ST, HOBOKEN 07030-5774, NJ USA

SN 1083-7159

EI 1549-490X

J9 ONCOLOGIST

JI Oncologist

DI 10.1002/onco.13914

EA AUG 2021

PG 10

WC Oncology

WE Science Citation Index Expanded (SCI-EXPANDED)

SC Oncology

GA UB9NM

UT WOS:000686164200001

PM 34309117

OA Green Published, Bronze

DA 2022-05-19

ER

PT J

AU Yuan, XL

Lv, JH

Dong, H

Wang, JH

AF Yuan, Xiaoli

Lv, Jiahua

Dong, Hang

Wang, Jiuhui

TI Does cervical lymph node recurrence after oesophagectomy or definitive

chemoradiotherapy for thoracic oesophageal squamous cell carcinoma

benefit from salvage treatment?

SO INTERACTIVE CARDIOVASCULAR AND THORACIC SURGERY

LA English

DT Article

DE Oesophageal neoplasm; Cervical lymph node; Lymphatic metastasis;

Recurrence

ID CURATIVE RESECTION; CANCER; LYMPHADENECTOMY

AB A best evidence topic in thoracic surgery was written according to a structured protocol. The question addressed was 'Does cervical lymph node recurrence after oesophagectomy or definitive chemoradiotherapy for thoracic oesophageal squamous cell carcinoma benefit from salvage treatment?' Altogether, 659 articles were found using the reported search, of which 8 represented the best evidence to answer the clinical question. The authors, journal, date, country of publication, patient group studied, study type, relevant outcomes and results of these articles were tabulated. All were retrospective or institutional reports. Seven of the 8 articles suggested that cervical lymphadenectomy (LD) or multimodal treatment with LD and radiotherapy (RT)/chemoradiotherapy (CRT) was associated with good outcomes in patients with cervical lymph node (LN) recurrence after curative oesophagectomy for thoracic oesophageal squamous cell carcinoma (ESCC). Salvage cervical LD was the main treatment and could achieve locoregional disease control and prolonged survival. It was safe with no postoperative mortality, although minor LD complications such as recurrent laryngeal nerve palsy were observed. Most reports highlighted that a solitary cervical LN relapse after curative oesophagectomy could be considered a good prognostic indicator for salvage cervical LD or multimodal treatment. Patients with microscopic residual tumour after salvage cervical LD or suspected extensive metastasis might benefit from a combination of LD and CRT. One article described salvage RT alone, again with a positive effect on survival. Conversely, data suggested that salvage LD in patients after definitive CRT for thoracic ESCC was not likely to control locoregional disease.

C1 [Yuan, Xiaoli; Dong, Hang; Wang, Jiuhui] Sichuan Canc Hosp & Inst, Comprehens Wards, 55 Sect 4,South Peoples Rd, Chengdu 610041, Peoples R China.

[Lv, Jiahua] Sichuan Canc Hosp & Inst, Dept Radiat Oncol, Chengdu, Peoples R China.

RP Wang, JH (corresponding author), Sichuan Canc Hosp & Inst, Comprehens Wards, 55 Sect 4,South Peoples Rd, Chengdu 610041, Peoples R China.

EM wjh8239@126.com

CR Dunning Joel, 2003, Interact Cardiovasc Thorac Surg, V2, P405, DOI 10.1016/S1569-9293(03)00191-9

Kato Takuya, 2014, Gan To Kagaku Ryoho, V41, P2393

Komatsu S, 2005, HEPATO-GASTROENTEROL, V52, P796

Ma X, 2015, ANN SURG ONCOL, V22, P624, DOI 10.1245/s10434-014-4008-8

Motoyama S, 2006, WORLD J SURG, V30, P191, DOI 10.1007/s00268-005-0092-z

Takemura Masashi, 2014, Gan To Kagaku Ryoho, V41, P2010

Watanabe M, 2012, DIS ESOPHAGUS, V25, P62, DOI 10.1111/j.1442-2050.2011.01215.x

Watanabe M, 2014, GEN THORAC CARDIOVAS, V62, P685, DOI 10.1007/s11748-014-0444-4

Yano M, 2006, DIS ESOPHAGUS, V19, P73, DOI 10.1111/j.1442-2050.2006.00543.x

NR 9

TC 4

Z9 4

U1 0

U2 2

PU OXFORD UNIV PRESS

PI OXFORD

PA GREAT CLARENDON ST, OXFORD OX2 6DP, ENGLAND

SN 1569-9293

EI 1569-9285

J9 INTERACT CARDIOV TH

JI Interact Cardiovasc. Thorac. Surg.

PD MAY

PY 2017

VL 24

IS 5

BP 792

EP 795

DI 10.1093/icvts/ivw441

PG 4

WC Cardiac & Cardiovascular Systems; Respiratory System; Surgery

WE Science Citation Index Expanded (SCI-EXPANDED)

SC Cardiovascular System & Cardiology; Respiratory System; Surgery

GA EY5UB

UT WOS:000404044600026

PM 28453803

OA Bronze

DA 2022-05-19

ER

PT J

AU Hirashima, K

Baba, Y

Watanabe, M

Karashima, RI

Sato, N

Imamura, Y

Nagai, Y

Hayashi, N

Iyama, KI

Baba, H

AF Hirashima, K.

Baba, Y.

Watanabe, M.

Karashima, R-I

Sato, N.

Imamura, Y.

Nagai, Y.

Hayashi, N.

Iyama, K-I

Baba, H.

TI Aberrant activation of the mTOR pathway and anti-tumour effect of

everolimus on oesophageal squamous cell carcinoma

SO BRITISH JOURNAL OF CANCER

LA English

DT Article

DE everolimus; RAD001; mTOR; oesophageal squamous cell carcinoma;

proliferation

ID RAPAMYCIN INHIBITOR EVEROLIMUS; MAMMALIAN TARGET; PHASE-I; RAD001

EVEROLIMUS; SIGNALING PATHWAY; INDUCED APOPTOSIS; BREAST-CANCER;

EXPRESSION; RATIONALE; RESISTANT

AB BACKGROUND: The mammalian target of rapamycin (mTOR) protein is important for cellular growth and homeostasis. The presence and prognostic significance of inappropriate mTOR activation have been reported for several cancers. Mammalian target of rapamycin inhibitors, such as everolimus (RAD001), are in development and show promise as anti-cancer drugs; however, the therapeutic effect of everolimus on oesophageal squamous cell carcinoma (OSCC) remains unknown.

METHODS: Phosphorylation of mTOR (p-mTOR) was evaluated in 167 resected OSCC tumours and 5 OSCC cell lines. The effects of everolimus on the OSCC cell lines TE4 and TE11 in vitro and alone or in combination with cisplatin on tumour growth in vivo were evaluated.

RESULTS: Mammalian target of rapamycin phosphorylation was detected in 116 tumours (69.5%) and all the 5 OSCC cell lines. Everolimus suppressed p-mTOR downstream pathways, inhibited proliferation and invasion, and induced apoptosis in both TE4 and TE11 cells. In a mouse xenograft model established with TE4 and TE11 cells, everolimus alone or in combination with cisplatin inhibited tumour growth.

CONCLUSION: The mTOR pathway was aberrantly activated in most OSCC tumours. Everolimus had a therapeutic effect both as a single agent and in combination with cisplatin. Everolimus could be a useful anti-cancer drug for patients with OSCC. British Journal of Cancer (2012) 106, 876-882. doi:10.1038/bjc.2012.36 www.bjcancer.com Published online 14 February 2012 (C) 2012 Cancer Research UK

C1 [Hirashima, K.; Baba, Y.; Watanabe, M.; Karashima, R-I; Sato, N.; Imamura, Y.; Nagai, Y.; Hayashi, N.; Baba, H.] Kumamoto Univ, Grad Sch Med Sci, Dept Surg Gastroenterol, Kumamoto 8608556, Japan.

[Hirashima, K.; Baba, Y.; Iyama, K-I] Kumamoto Univ Hosp, Dept Surg Pathol, Kumamoto 8608556, Japan.

RP Baba, H (corresponding author), Kumamoto Univ, Grad Sch Med Sci, Dept Surg Gastroenterol, 1-1-1 Honjo, Kumamoto 8608556, Japan.

EM hdobaba@kumamoto-u.ac.jp

FU Novartis Pharma AG (Basel, Switzerland) [RAD001]

FX This work was made possible by the provision of everolimus (RAD001) by

Novartis Pharma AG (Basel, Switzerland). We thank Novartis Pharma AG for

their kindness. We thank Dr Katsunori Imai and Keisuke Miyake for in

vitro technical assistance, and Dr Satoshi Ida and Dr Hasta Holrad for

in vivo technical assistance. The content is solely the responsibility

of the authors and does not necessarily represent the official views of

the NCI or NIH. The funders had no role in the study design, data

collection and analysis, decision to publish, or preparation of the

manuscript.

CR Abraham RT, 2007, CLIN CANCER RES, V13, P3109, DOI 10.1158/1078-0432.CCR-06-2798

Antonarakis ES, 2010, CANCER LETT, V291, P1, DOI 10.1016/j.canlet.2009.08.012

Awada A, 2008, EUR J CANCER, V44, P84, DOI 10.1016/j.ejca.2007.10.003

Beuvink I, 2005, CELL, V120, P747, DOI 10.1016/j.cell.2004.12.040

Bianco R, 2008, BRIT J CANCER, V98, P923, DOI 10.1038/sj.bjc.6604269

Bianco RB, 2006, EUR J CANCER, V42, P290, DOI 10.1016/j.ejca.2005.07.034

Bjornsti MA, 2004, NAT REV CANCER, V4, P335, DOI 10.1038/nrc1362

Boone J, 2008, J CLIN PATHOL, V61, P909, DOI 10.1136/jcp.2008.055772

Boulay A, 2004, CANCER RES, V64, P252, DOI 10.1158/0008-5472.CAN-3554-2

Carmeliet P, 2000, NATURE, V407, P249, DOI 10.1038/35025220

Chan S, 2004, BRIT J CANCER, V91, P1420, DOI 10.1038/sj.bjc.6602162

Coppin C, 2010, BIOL-TARGETS THER, V4, P91

Dancey JE, 2006, CANCER BIOL THER, V5, P1065, DOI 10.4161/cbt.5.9.3175

Del Bufalo D, 2004, NEOPLASIA, V6, P513, DOI 10.1593/neo.04133

Easton JB, 2006, ONCOGENE, V25, P6436, DOI 10.1038/sj.onc.1209886

Enzinger PC, 2003, NEW ENGL J MED, V349, P2241, DOI 10.1056/NEJMra035010

Fouladi M, 2007, J CLIN ONCOL, V25, P4806, DOI 10.1200/JCO.2007.11.4017

Gridelli C, 2007, CLIN LUNG CANCER, V8, P568, DOI 10.3816/CLC.2007.n.045

Herberger B, 2007, CLIN CANCER RES, V13, P4795, DOI 10.1158/1078-0432.CCR-07-0738

Hidalgo M, 2000, ONCOGENE, V19, P6680, DOI 10.1038/sj.onc.1204091

Hirashima K, 2010, ANN SURG ONCOL, V17, P2486, DOI 10.1245/s10434-010-1040-1

Hou GQ, 2007, CANCER LETT, V253, P236, DOI 10.1016/j.canlet.2007.01.026

Hou GQ, 2010, CANCER LETT, V290, P248, DOI 10.1016/j.canlet.2009.09.015

Hudes GR, 2009, CANCER, V115, P2313, DOI 10.1002/cncr.24239

Johnson BE, 2007, CLIN CANCER RES, V13, p4628S, DOI 10.1158/1078-0432.CCR-07-0717

Johnston SRD, 2006, CLIN CANCER RES, V12, p1061S, DOI 10.1158/1078-0432.CCR-05-2125

Kapoor A, 2009, CURR ONCOL, V16, pS35

Lane HA, 2009, CLIN CANCER RES, V15, P1612, DOI 10.1158/1078-0432.CCR-08-2057

Ma BBY, 2010, INVEST NEW DRUG, V28, P413, DOI 10.1007/s10637-009-9269-x

Mabuchi S, 2007, CLIN CANCER RES, V13, P4261, DOI 10.1158/1078-0432.CCR-06-2770

Manegold PC, 2008, CLIN CANCER RES, V14, P892, DOI 10.1158/1078-0432.CCR-07-0955

Menon S, 2008, Oncogene, V27 Suppl 2, pS43, DOI 10.1038/onc.2009.352

Milella M, 2004, CLIN CANCER RES, V10, P7747, DOI 10.1158/1078-0432.CCR-04-0908

Mita MM, 2003, CANCER BIOL THER, V2, pS169

O'Donnell A, 2008, J CLIN ONCOL, V26, P1588, DOI 10.1200/JCO.2007.14.0988

Panwalkar A, 2004, CANCER-AM CANCER SOC, V100, P657, DOI 10.1002/cncr.20026

Scott KL, 2009, NATURE, V459, P1085, DOI 10.1038/nature08109

Sparks CA, 2010, ONCOGENE, V29, P3733, DOI 10.1038/onc.2010.139

Tabernero J, 2008, J CLIN ONCOL, V26, P1603, DOI 10.1200/JCO.2007.14.5482

Tanaka Chiaki, 2008, J Clin Oncol, V26, P1596, DOI 10.1200/JCO.2007.14.1127

Wolpin BM, 2009, J CLIN ONCOL, V27, P193, DOI 10.1200/JCO.2008.18.9514

Workman P, 2010, BRIT J CANCER, V102, P1555, DOI 10.1038/sj.bjc.6605642

Wouters BG, 2008, NAT REV CANCER, V8, P851, DOI 10.1038/nrc2501

Yao JC, 2008, J CLIN ONCOL, V26, P4311, DOI 10.1200/JCO.2008.16.7858

Yee KWL, 2006, CLIN CANCER RES, V12, P5165, DOI 10.1158/1078-0432.CCR-06-0764

Yoshioka A, 2008, ONCOL REP, V19, P1099

NR 46

TC 41

Z9 44

U1 1

U2 2

PU NATURE PUBLISHING GROUP

PI LONDON

PA MACMILLAN BUILDING, 4 CRINAN ST, LONDON N1 9XW, ENGLAND

SN 0007-0920

EI 1532-1827

J9 BRIT J CANCER

JI Br. J. Cancer

PD FEB 28

PY 2012

VL 106

IS 5

BP 876

EP 882

DI 10.1038/bjc.2012.36

PG 7

WC Oncology

WE Science Citation Index Expanded (SCI-EXPANDED)

SC Oncology

GA 900NO

UT WOS:000300894300011

PM 22333597

OA hybrid, Green Published

DA 2022-05-19

ER

PT J

AU Chen, J

Yin, WM

Yao, H

Gu, WD

AF Chen, Jie

Yin, Wenming

Yao, Hui

Gu, Wendong

TI Salvage treatment for lymph node recurrence after radical resection of

esophageal squamous cell carcinoma

SO RADIATION ONCOLOGY

LA English

DT Article

DE Esophageal squamous cell carcinoma; Radical surgery; Lymph node

recurrence; Radiotherapy; Chemoradiotherapy

AB Background Patients with regional lymph node recurrence after radical resection of esophageal cancer have poor therapeutic outcomes. Currently, there is no standard treatment for regional lymph node recurrence, and its prognostic risk factors are not well-understood. This study retrospectively analyzed 83 patients with postoperative regional lymph node recurrence after radical resection of esophageal squamous cell carcinoma. The aim was to evaluate the clinical efficacy and prognostic factors of salvage radiotherapy with or without chemotherapy in these patients. Methods The survival and prognostic factors of 83 patients with esophageal squamous cell carcinoma with regional lymph node recurrence after radical surgery were retrospectively analyzed. All patients underwent radiotherapy, of which 74 patients received volumetric modulated arc therapy (VMAT), 9 patients received three-dimensional conformal radiation therapy (3DCRT), administered using a conventional segmentation protocol with a dose distribution range of 50.4-66.2Gy (median dose of 60Gy). In total, 41 patients received radiotherapy alone, 42 received radiotherapy combined with chemotherapy, and the concurrent chemotherapy regimen was mainly composed of either platinum or fluorouracil monotherapy, except for 4 patients who were given 5-fluorouracil plus platinum (FP) or paclitaxel plus platinum (TP). Results The median follow-up time was 24 (range, 9-75) months. The overall survival (OS) rates at 1 year, 2 years, 3 years, and 5 years were 83.0, 57.1, 40.1, and 35.1%, respectively. The median overall survival (OS) time was 18 (range, 5-75) months. The 3-year survival rate was 47.5% in patients with radiation alone and 41.9% in patients receiving concurrent chemoradiotherapy(p = 0.570), while the response rate (CR + PR) in those two groups was 73.2 and 91.4%, respectively. By multivariate analysis of OS, age (worse in younger patients, p = 0.034) was found to be significantly associated with disease prognosis. The commonly toxicities were esophagitis, neutropenia and anemia. 18% patients experienced grade 3 toxicity and no treatment-related death occurred. Conclusions These results of this retrospective analysis suggest that radiotherapy with or without chemotherapy is an effective and feasible salvage treatment for lymph node recurrence after radical resection of esophageal squamous cell carcinoma.

C1 [Chen, Jie; Yin, Wenming; Yao, Hui; Gu, Wendong] Soochow Univ, Affiliated Hosp 3, Dept Radiat Oncol, 185 Juqian St, Changzhou 213003, Jiangsu, Peoples R China.

RP Gu, WD (corresponding author), Soochow Univ, Affiliated Hosp 3, Dept Radiat Oncol, 185 Juqian St, Changzhou 213003, Jiangsu, Peoples R China.

EM guwendong1415@czfph.com

FU CSCO-Hengrui Cancer Research Fund [Y-HR2018-063]

FX This work was supported by CSCO-Hengrui Cancer Research Fund

(Y-HR2018-063).

CR Bao Y, 2013, RADIAT ONCOL, V8, DOI 10.1186/1748-717X-8-241

Baxi SH, 2008, J MED IMAG RADIAT ON, V52, P583, DOI 10.1111/j.1440-1673.2008.02023.x

CARLISLE JG, 1993, RADIOLOGY, V189, P271, DOI 10.1148/radiology.189.1.8372204

Dresner SM, 2000, BRIT J SURG, V87, P1426, DOI 10.1046/j.1365-2168.2000.01541.x

Fakhrian K, 2012, STRAHLENTHER ONKOL, V188, P136, DOI 10.1007/s00066-011-0023-x

Haefner MF, 2017, RADIAT ONCOL, V12, DOI 10.1186/s13014-017-0863-3

Hellwig D, 2007, J NUCL MED, V48, P1761, DOI 10.2967/jnumed.107.044362

Hsu PK, 2011, J GASTROINTEST SURG, V15, P558, DOI 10.1007/s11605-011-1458-1

Jeene PM, 2017, DIS ESOPHAGUS, V30, DOI 10.1111/dote.12539

Jingu K, 2006, BMC CANCER, V6, DOI 10.1186/1471-2407-6-50

Jingu K, 2012, BMC CANCER, V12, DOI 10.1186/1471-2407-12-542

Jingu K, 2012, INT J RADIAT ONCOL, V83, P172, DOI 10.1016/j.ijrobp.2011.06.1978

Kawamoto T, 2018, JPN J CLIN ONCOL, V48, P259, DOI 10.1093/jjco/hyx171

Kosuga T, 2011, WORLD J SURG, V35, P798, DOI 10.1007/s00268-011-0978-x

Ma CL, 2018, J CANCER RES THER, V14, P896, DOI 10.4103/jcrt.JCRT_1049_17

Ma DY, 2014, RADIAT ONCOL, V9, DOI 10.1186/1748-717X-9-28

Ma X, 2015, ANN SURG ONCOL, V22, P624, DOI 10.1245/s10434-014-4008-8

Mariette C, 2003, CANCER-AM CANCER SOC, V97, P1616, DOI 10.1002/cncr.11228

Motoyama S, 2006, WORLD J SURG, V30, P191, DOI 10.1007/s00268-005-0092-z

Nakagawa S, 2004, J AM COLL SURGEONS, V198, P205, DOI 10.1016/j.jamcollsurg.2003.10.005

Nemoto K, 2001, RADIOTHER ONCOL, V61, P165, DOI 10.1016/S0167-8140(01)00392-9

PALIVOS T, 1993, ECON LETT, V41, P107, DOI 10.1016/0165-1765(93)90120-2

RAOUL JL, 1995, GUT, V37, P174, DOI 10.1136/gut.37.2.174

Shen HC, 2012, GENE, V509, P173, DOI 10.1016/j.gene.2012.07.060

Shioyama Y, 2007, JPN J CLIN ONCOL, V37, P918, DOI 10.1093/jjco/hym138

TAKASHIMA S, 1991, AM J ROENTGENOL, V156, P297, DOI 10.2214/ajr.156.2.1898802

Watanabe M, 2014, GEN THORAC CARDIOVAS, V62, P685, DOI 10.1007/s11748-014-0444-4

Yamashita H, 2017, RADIAT ONCOL, V12, DOI 10.1186/s13014-017-0780-5

Yang H, 2017, DIS ESOPHAGUS, V30, DOI 10.1111/dote.12497

Zhang J, 2012, RADIAT ONCOL, V7, DOI 10.1186/1748-717X-7-93

Zhang WW, 2015, SCI REP-UK, V5, DOI 10.1038/srep08071

NR 31

TC 8

Z9 11

U1 0

U2 0

PU BMC

PI LONDON

PA CAMPUS, 4 CRINAN ST, LONDON N1 9XW, ENGLAND

EI 1748-717X

J9 RADIAT ONCOL

JI Radiat. Oncol.

PD SEP 18

PY 2019

VL 14

IS 1

AR 169

DI 10.1186/s13014-019-1377-y

PG 8

WC Oncology; Radiology, Nuclear Medicine & Medical Imaging

WE Science Citation Index Expanded (SCI-EXPANDED)

SC Oncology; Radiology, Nuclear Medicine & Medical Imaging

GA VK0ZB

UT WOS:000658393300001

PM 31533757

OA Green Published, gold

DA 2022-05-19

ER

PT J

AU Xia, MH

Xu, LF

Leng, YH

Gao, F

Xia, H

Zhang, DD

Ding, XW

AF Xia, Manhui

Xu, Lifang

Leng, Yunhua

Gao, Feng

Xia, Hong

Zhang, Diandian

Ding, Xuewu

TI Downregulation of MLL3 in esophageal squamous cell carcinoma is required

for the growth and metastasis of cancer cells

SO TUMOR BIOLOGY

LA English

DT Article

DE ESCC; MLL3; Cancer metastasis; Cell proliferation

ID MUTATIONS; RECEPTORS; RISK

AB The mixed lineage leukemia 3 (MLL3), a member of the mixed lineage leukemia (MLL) family, has been reported to be mutated in multiple cancer types. However, its function in esophageal squamous cell carcinoma (ESCC) remains poorly understood. Here, we found that the expression of MLL3 was downregulated in ESCC tissues. Moreover, over-expression of MLL3 in ESCC cells inhibited cell proliferation and migration, while the knockdown expression of MLL3 promoted the tumorigenicity of ESCC cells. Mechanistically, MLL3 regulated the expression of multiple growth-related and migration-related genes. Taken together, our study suggested that downregulation of MLL3 was very important in the progression of ESCC.

C1 [Xia, Manhui; Leng, Yunhua; Xia, Hong; Zhang, Diandian; Ding, Xuewu] Jingjiang Peoples Hosp Jiangsu Prov, Dept Thorac Surg, Jingjiang City 214500, Jiangsu, Peoples R China.

[Xu, Lifang; Leng, Yunhua; Gao, Feng] Jingjiang Peoples Hosp Jiangsu Prov, Jingjiang City 214500, Jiangsu, Peoples R China.

RP Leng, YH (corresponding author), Jingjiang Peoples Hosp Jiangsu Prov, 28 East Zhongzhou Rd, Jingjiang City 214500, Jiangsu, Peoples R China.

EM lengyunhua0709@163.com; gaofeng0705@163.com

CR Adham SA, 2012, J HISTOCHEM CYTOCHEM, V62, P619

Ansari KI, 2012, J MOL ENDOCRINOL, V48, P61, DOI 10.1530/JME-11-0078

Ashktorab H, 2010, PLOS ONE, V5, DOI 10.1371/journal.pone.0008879

Balakrishnan A, 2007, CANCER RES, V67, P3545, DOI 10.1158/0008-5472.CAN-07-0065

Cui Y, 2013, WORLD J SURG ONCOL, V11, DOI 10.1186/1477-7819-11-266

Forghanifard MM, 2012, ANN SURG ONCOL, V19, P743, DOI 10.1245/s10434-011-2074-8

Gonzaga IM, 2012, BMC CANCER, V12, DOI 10.1186/1471-2407-12-569

Hara F, 2005, CANCER LETT, V226, P37, DOI 10.1016/j.canlet.2004.12.025

Jin H, 2012, BIOMED RES, V31, P105

Kondo Y, 2012, CANCER SCI, V105, P363

Lee J, 2009, P NATL ACAD SCI USA, V106, P8513, DOI 10.1073/pnas.0902873106

Li B, 2013, ASIAN PAC J CANCER P, V14, P4239, DOI 10.7314/APJCP.2013.14.7.4239

Li WD, 2013, BLOOD, V121, P1478, DOI 10.1182/blood-2012-12-470559

Litt M, 2009, BIOSCIENCE REP, V29, P131, DOI 10.1042/BSR20080176

Morgan MA, 2013, MOL CELL BIOL, V33, P1698, DOI 10.1128/MCB.00203-13

Morgan S, 2009, EXP CELL RES, V315, P572, DOI 10.1016/j.yexcr.2008.08.009

Natsugoe S, 2007, ONCOL REP, V17, P517

Siegel R, 2013, CA-CANCER J CLIN, V63, P11, DOI 10.3322/caac.21166

Song Y, 2013, NATURE, V509, P91

Stewart S, 2009, P NATL ACAD SCI USA, V106, P19889, DOI 10.1073/pnas.0904132106

Suzuki Hiromu, 2013, Frontiers in Genetics, V4, P258, DOI 10.3389/fgene.2013.00258

Vos M, 2003, CANCER GENET CYTOGEN, V140, P23, DOI 10.1016/S0165-4608(02)00638-6

Wang C, 2013, INT J ONCOL, V43, P1607, DOI 10.3892/ijo.2013.2083

Wang N, 2009, MOL BIOL REP, V36, P357, DOI 10.1007/s11033-007-9187-y

Watanabe Y, 2012, PLOS ONE, V6

Wu XH, 2012, J TRANSL MED, V10, DOI 10.1186/1479-5876-10-180

Yamamoto H, 2014, WORLD J GASTROENTERO, V20, P3927, DOI 10.3748/wjg.v20.i14.3927

Zang ZJ, 2012, NAT GENET, V44, P570, DOI 10.1038/ng.2246

NR 28

TC 12

Z9 13

U1 0

U2 2

PU SAGE PUBLICATIONS LTD

PI LONDON

PA 1 OLIVERS YARD, 55 CITY ROAD, LONDON EC1Y 1SP, ENGLAND

SN 1010-4283

EI 1423-0380

J9 TUMOR BIOL

JI Tumor Biol.

PD FEB

PY 2015

VL 36

IS 2

BP 605

EP 613

DI 10.1007/s13277-014-2616-3

PG 9

WC Oncology

WE Science Citation Index Expanded (SCI-EXPANDED)

SC Oncology

GA CC6MD

UT WOS:000350478200018

PM 25273170

DA 2022-05-19

ER

PT J

AU Lee, SJ

Kim, S

Kim, M

Lee, J

Park, YH

Im, YH

Park, SH

AF Lee, Su Jin

Kim, Sungmin

Kim, Moonjin

Lee, Jeeyun

Park, Yeon Hee

Im, Young-Hyuck

Park, Se Hoon

TI Capecitabine in combination with either cisplatin or weekly paclitaxel

as a first-line treatment for metastatic esophageal squamous cell

carcinoma: a randomized phase II study

SO BMC CANCER

LA English

DT Article

DE Esophageal squamous cell carcinoma; First-line palliative chemotherapy;

Capecitabine; Cisplatin; Paclitaxel

ID FLUOROURACIL PLUS LEUCOVORIN; COLORECTAL-CANCER; ORAL CAPECITABINE;

CHEMOTHERAPY; OXALIPLATIN; 5-FLUOROURACIL; THERAPY; TRIAL; XP

AB Background: The aim of this study was to assess the efficacy and safety of a combination regimen of capecitabine plus cisplatin (CC) or capecitabine plus paclitaxel (CP) as a first-line treatment in patients with metastatic esophageal squamous cell carcinoma.

Methods: Patients with recurrent or metastatic esophageal squamous cell carcinoma were enrolled in this open-label, phase II, randomized trial. Patients were assigned to either the CC arm (days [D] 1-14 capecitabine 1000 mg/m(2) twice daily + D1 cisplatin 75 mg/m(2), every 3 weeks) or the CP arm (D1-14 capecitabine 1000 mg/m(2) twice daily + D1, 8 paclitaxel 80 mg/m(2), every 3 weeks). The primary endpoint of the study was response rate and secondary endpoints were progression-free survival (PFS), overall survival (OS), toxicity and quality of life.

Results: A total of 94 patients were entered into this study between October 2008 and October 2012, 46 patients in the CC arm and 48 in the CP arm. Patients in both arms received a median of six cycles of treatment (range, 1-14) and the response rates were 57 and 58 % in the cisplatin and paclitaxel arm, respectively. With a median follow-up of 23 months, the median PFS was 5.1 months (95 % CI 4.0-6.2 months) in the cisplatin arm and 6.7 months (95 % CI 4.9-8.5 months) in the paclitaxel arm, whereas the median OS was 10.5 months (95 % CI 9.2-11.9 months) in the cisplatin arm and 13.2 months (95 % CI 9.4-17.0 months) in the paclitaxel arm. Patients in the cisplatin arm were more likely to experience neutropenia and thrombocytopenia, whereas patients in the paclitaxel arm had a higher frequency of neuropathy and alopecia. Quality of life was similar between treatment arms.

Conclusions: Both CC and CP regimens were effective and well tolerated as a first-line treatment in patients with metastatic esophageal squamous cell carcinoma.

C1 [Lee, Su Jin; Kim, Sungmin; Kim, Moonjin; Lee, Jeeyun; Park, Yeon Hee; Im, Young-Hyuck; Park, Se Hoon] Sungkyunkwan Univ, Sch Med, Samsung Med Ctr, Dept Med,Div Hematol Oncol, Seoul, South Korea.

RP Park, SH (corresponding author), Sungkyunkwan Univ, Sch Med, Samsung Med Ctr, Dept Med,Div Hematol Oncol, Seoul, South Korea.

EM hematoma@skku.edu

RI Lee, Jee Yun/C-9646-2011

OI Lee, Su Jin/0000-0003-3405-9935

CR Bleiberg H, 1997, EUR J CANCER, V33, P1216, DOI 10.1016/S0959-8049(97)00088-9

Blum JL, 2006, J CLIN ONCOL, V24, P4384, DOI 10.1200/JCO.2005.05.1383

Cunningham D, 2008, NEW ENGL J MED, V358, P36, DOI 10.1056/NEJMoa073149

Dimopoulou I, 2006, ANN ONCOL, V17, P372, DOI 10.1093/annonc/mdj057

Enzinger PC, 1999, SEMIN ONCOL, V26, P12

Hoff PM, 2001, J CLIN ONCOL, V19, P2282, DOI 10.1200/JCO.2001.19.8.2282

Jung KW, 2013, CANCER RES TREAT, V45, P15, DOI 10.4143/crt.2013.45.1.15

Kamangar F, 2006, J CLIN ONCOL, V24, P2137, DOI 10.1200/JCO.2005.05.2308

Kang YK, 2009, ANN ONCOL, V20, P666, DOI 10.1093/annonc/mdn717

Lee J, 2008, CANCER CHEMOTH PHARM, V62, P77, DOI 10.1007/s00280-007-0577-6

Lee SS, 2007, JPN J CLIN ONCOL, V37, P829, DOI 10.1093/jjco/hym117

Levard H, 1998, EUR J SURG, V164, P849

Lorenzen S, 2005, BRIT J CANCER, V92, P2129, DOI 10.1038/sj.bjc.6602645

Polee MB, 2002, BRIT J CANCER, V86, P669, DOI 10.1038/sj.bjc.6600166

Qin TJ, 2009, WORLD J GASTROENTERO, V15, P871, DOI 10.3748/wjg.15.871

Sawada N, 1998, CLIN CANCER RES, V4, P1013

SIMON R, 1989, CONTROL CLIN TRIALS, V10, P1, DOI 10.1016/0197-2456(89)90015-9

Uhlmann C, 2004, ONCOLOGY-BASEL, V67, P117, DOI 10.1159/000080997

Van Cutsem E, 2001, J CLIN ONCOL, V19, P4097, DOI 10.1200/JCO.2001.19.21.4097

van Meerten E, 2007, BRIT J CANCER, V96, P1348, DOI 10.1038/sj.bjc.6603750

Yun T, 2011, BMC CANCER, V11, DOI 10.1186/1471-2407-11-385

Zhang XD, 2008, AM J CLIN ONCOL-CANC, V31, P29, DOI 10.1097/COC.0b013e3181131ca9

NR 22

TC 10

Z9 12

U1 2

U2 4

PU BMC

PI LONDON

PA CAMPUS, 4 CRINAN ST, LONDON N1 9XW, ENGLAND

SN 1471-2407

J9 BMC CANCER

JI BMC Cancer

PD OCT 14

PY 2015

VL 15

AR 693

DI 10.1186/s12885-015-1716-9

PG 7

WC Oncology

WE Science Citation Index Expanded (SCI-EXPANDED)

SC Oncology

GA CT3KL

UT WOS:000362705500006

PM 26468007

OA Green Published, gold

DA 2022-05-19

ER

PT J

AU Wu, N

Du, ZG

Zhu, YJ

Song, Y

Pang, LW

Chen, ZM

AF Wu, Ning

Du, Zunguo

Zhu, Yongjun

Song, Yang

Pang, Liewen

Chen, Zhiming

TI The Expression and Prognostic Impact of the PI3K/AKT/mTOR Signaling

Pathway in Advanced Esophageal Squamous Cell Carcinoma

SO TECHNOLOGY IN CANCER RESEARCH & TREATMENT

LA English

DT Article

DE esophageal squamous cell carcinoma; PI3K/AKT/mTOR signaling pathway;

PTEN; Prognosis

ID GASTRIC-CANCER; MTOR; ACTIVATION; THERAPY

AB The abnormal phosphatase and tensin homolog expression and activated phosphoinositide-3 kinase/Protein kinase B (AKT)/mammalian target of rapamycin signaling pathway are involved in the progression of esophageal squamous cell carcinoma. By assessing the expression pattern of key components in the phosphoinositide-3 kinase/AKT/mammalian target of rapamycin signaling pathway by immunohistochemistry in tumor and nontumor esophageal mucosa from patients with esophageal squamous cell carcinomas, we aimed to carefully explore the relationship between the various protein expressions and clinicopathological factors, as well as patient outcome. A total of 145 tumor and 145 nontumor samples from patients with esophageal squamous cell carcinoma, collected from HuaShan Hospital (Shanghai, China) were evaluated. Clinical characteristics, the targeted protein expressions (including phosphatase and tensin homolog, phosphoinositide-3 kinase, AKT, p-AKT, mammalian target of rapamycin, p-mTOR, p70S6 kinase 1, p-P70S6K1, elongation initiation factor 4E binding protein-1, and p-4E-BP1, and survival rate were analyzed. Among them, phosphoinositide-3 kinase, AKT, p-AKT, mammalian target of rapamycin, p-mTOR, elongation initiation factor 4E binding protein-1, p70S6 kinase 1, and p-P70S6K1 proteins were significantly upregulated in tumor tissue. Conversely, phosphatase and tensin homolog was largely downregulated in tumor tissue, notably in pT3-T4 tumors. Low expression of phosphatase and tensin homolog whereas high expression of mammalian target of rapamycin signaling components in tumors was closely related to the presence of lymph node metastases and advanced TNM stage (all P < .05). Moreover phosphatase and tensin homolog, mammalian target of rapamycin, and p70S6 kinase 1 were correlated with overall survival as well as p-mTOR was correlated with progression-free survival (all P < .05). Overexpression of mammalian target of rapamycin was proved to be an independent adverse prognostic factor for overall survival in esophageal squamous cell carcinomas. Our results suggest that the phosphoinositide-3 kinase/AKT/mammalian target of rapamycin signaling pathway is activated in esophageal squamous cell carcinoma, with the low expression of phosphatase and tensin homolog and the high expression of the mammalian target of rapamycin component proteins (both total and phosphorylated) in tumor tissue. Our result might offer a new strategy for specific targeted therapy and prognostic assessment in esophageal cancer.

C1 [Wu, Ning; Zhu, Yongjun; Song, Yang; Pang, Liewen; Chen, Zhiming] Fudan Univ, HuaShan Hosp, Dept Cardiothorac Surg, 12 Middle Wulumuqi Rd, Shanghai 200040, Peoples R China.

[Du, Zunguo] Fudan Univ, HuaShan Hosp, Dept Pathol, Shanghai, Peoples R China.

RP Wu, N; Chen, ZM (corresponding author), Fudan Univ, HuaShan Hosp, Dept Cardiothorac Surg, 12 Middle Wulumuqi Rd, Shanghai 200040, Peoples R China.

EM wuning@fudan.edu.cn; czm_md@163.com

FU Shanghai Municipal Health & Family Planning Commission [20174Y0238];

National Natural Science Foundation of China [81602079]

FX The author(s) disclosed receipt of the following financial support for

the research, authorship, and/or publication of this article: This work

was supported by grant from the Shanghai Municipal Health & Family

Planning Commission (no 20174Y0238) and the National Natural Science

Foundation of China (no 81602079).

CR Chen W, 2011, THORAC CANC, V5, P343

Hildebrandt MAT, 2009, J CLIN ONCOL, V27, P857, DOI 10.1200/JCO.2008.17.6297

Hirashima K, 2012, BRIT J CANCER, V106, P876, DOI 10.1038/bjc.2012.36

Hou GQ, 2014, J IMMUNOL RES, V2014, DOI 10.1155/2014/845763

Kamangar F, 2006, J CLIN ONCOL, V24, P2137, DOI 10.1200/JCO.2005.05.2308

Li B, 2017, ONCOTARGET, V8, P38755, DOI 10.18632/oncotarget.16333

Lin DC, 2014, NAT GENET, V46, P467, DOI 10.1038/ng.2935

Lin JW, 2017, GENET TEST MOL BIOMA, V21, P236, DOI 10.1089/gtmb.2016.0316

LU J, 2015, BIOMED RES INT, V2015, DOI DOI 10.1155/2015/417210

Ma XJM, 2009, NAT REV MOL CELL BIO, V10, P307, DOI 10.1038/nrm2672

Malinowsky K, 2014, BRIT J CANCER, V110, P2081, DOI 10.1038/bjc.2014.100

Ocana A, 2014, PLOS ONE, V9, DOI 10.1371/journal.pone.0095219

Paquette M, 2018, CANCERS, V10, DOI 10.3390/cancers10010018

Strimpakos AS, 2009, CANCER TREAT REV, V35, P148, DOI 10.1016/j.ctrv.2008.09.006

Sukhbaatar N, 2016, TRENDS IMMUNOL, V37, P778, DOI 10.1016/j.it.2016.08.009

Tachibana M, 2002, CANCER-AM CANCER SOC, V94, P1955, DOI 10.1002/cncr.0678

Torre LA, 2015, CA-CANCER J CLIN, V65, P87, DOI 10.3322/caac.21262

Vivanco I, 2002, NAT REV CANCER, V2, P489, DOI 10.1038/nrc839

Waniczek D, 2013, POL J PATHOL, V64, P15, DOI 10.5114/PJP.2013.34598

Wu N, 2013, WIEN KLIN WOCHENSCHR, V125, P26, DOI 10.1007/s00508-012-0310-2

Wu N, 2012, CHINESE MED J-PEKING, V125, P4445, DOI 10.3760/cma.j.issn.0366-6999.2012.24.022

Ying JE, 2015, ONCOTARGETS THER, V8, P2427, DOI 10.2147/OTT.S88592

Yoon MS, 2017, NUTRIENTS, V9, DOI 10.3390/nu9111176

Zhang HF, 2016, STEM CELLS, V34, P2040, DOI 10.1002/stem.2395

Zhang X, 2015, ONCOLOGY-BASEL, V88, P76, DOI 10.1159/000366426

Zhu JH, 2015, SCI REP-UK, V5, DOI 10.1038/srep08250

NR 26

TC 23

Z9 26

U1 1

U2 1

PU SAGE PUBLICATIONS INC

PI THOUSAND OAKS

PA 2455 TELLER RD, THOUSAND OAKS, CA 91320 USA

SN 1533-0346

EI 1533-0338

J9 TECHNOL CANCER RES T

JI Technol. Cancer Res. Treat.

PD JAN

PY 2018

VL 17

DI 10.1177/1533033818758772

PG 9

WC Oncology

WE Science Citation Index Expanded (SCI-EXPANDED)

SC Oncology

GA GB8DN

UT WOS:000429305700002

PM 29463194

OA Green Published, gold

DA 2022-05-19

ER

PT J

AU Xu, J

Kang, DY

Zeng, YP

Zhuo, SM

Zhu, XQ

Jiang, LW

Chen, JX

Lin, JB

AF Xu, Jian

Kang, Deyong

Zeng, Yaping

Zhuo, Shuangmu

Zhu, Xiaoqin

Jiang, Liwei

Chen, Jianxin

Lin, Jiangbo

TI Multiphoton microscopy for label-free identification of intramural

metastasis in human esophageal squamous cell carcinoma

SO BIOMEDICAL OPTICS EXPRESS

LA English

DT Article

ID PROGNOSTIC-SIGNIFICANCE; 2-PHOTON; CANCER; QUANTIFICATION; ORGANIZATION;

MUCOSA; VIVO

AB For complete removal of cancerous tissue in esophageal squamous cell carcinoma (ESCC), intramural metastasis (IM) should be identified preoperatively or intraoperatively. Here, multiphoton microscopy (MPM) was introduced for label-free identification of IM in the esophageal wall, by a combination of two-photon excited fluorescence (TPEF), second harmonic generation (SHG) imaging, and spectral analysis. Three-dimensional (3D) imaging of the IM region was also performed. Quantitative parameters, including 3D fiber orientation, were measured by 3D-weighted orientation vector summation. Overall, MPM showed the potential to identify IM. With the development of the advanced MPM endoscope, clinical identification of IM by MPM will be possible. (C) 2017 Optical Society of America

C1 [Xu, Jian; Zeng, Yaping; Zhuo, Shuangmu; Zhu, Xiaoqin; Jiang, Liwei; Chen, Jianxin] Fujian Normal Univ, Fujian Prov Key Lab Photon Technol, Minist Educ, Key Lab OptoElect Sci & Technol Med, Fuzhou 350007, Peoples R China.

[Kang, Deyong] Fujian Med Univ, Affiliated Union Hosp, Dept Pathol, Fuzhou 350001, Peoples R China.

[Lin, Jiangbo] Fujian Med Univ, Affiliated Union Hosp, Dept Thorac Surg, Fuzhou 350001, Peoples R China.

RP Chen, JX (corresponding author), Fujian Normal Univ, Fujian Prov Key Lab Photon Technol, Minist Educ, Key Lab OptoElect Sci & Technol Med, Fuzhou 350007, Peoples R China.

EM chenjianxin@fjnu.edu.cn; 135279779@qq.com

FU National Natural Science Foundation of China [81671730, 81271620];

National Key Basic Research Program of China [2015CB352006]; Joint Funds

of Fujian Provincial Health and Education Research [WKJ2016-2-28];

Program for Changjiang Scholars and Innovative Research Team in

University [IRT_15R10]

FX National Natural Science Foundation of China (Grant No. 81671730,

81271620), National Key Basic Research Program of China (2015CB352006),

Joint Funds of Fujian Provincial Health and Education Research

(WKJ2016-2-28), Program for Changjiang Scholars and Innovative Research

Team in University (IRT_15R10).

CR Bahlmann K, 2007, OPT EXPRESS, V15, P10991, DOI 10.1364/OE.15.010991

Bayan C, 2009, J APPL PHYS, V105, DOI 10.1063/1.3116626

Beaurepaire E, 2001, OPT COMMUN, V188, P25, DOI 10.1016/S0030-4018(00)01156-1

Birk JW, 2014, DIGEST DIS SCI, V59, P1529, DOI 10.1007/s10620-014-3121-7

Chen G, 2013, SCANNING, V35, P362, DOI 10.1002/sca.21076

CHEONG WF, 1990, IEEE J QUANTUM ELECT, V26, P2166, DOI 10.1109/3.64354

Cicchi R, 2005, OPT EXPRESS, V13, P2337, DOI 10.1364/OPEX.13.002337

Cicchi R, 2013, BIOMED OPT EXPRESS, V4, P1204, DOI 10.1364/BOE.4.001204

Fakhrian K, 2014, STRAHLENTHER ONKOL, V190, P1133, DOI 10.1007/s00066-014-0711-4

Goetz M, 2007, ENDOSCOPY, V39, P350, DOI 10.1055/s-2007-966262

Hashimoto T, 2013, GASTRIC CANCER, V16, P537, DOI 10.1007/s10120-012-0228-4

Ide H., 1980, JPN J GASTROENTEROL, V13, P781

KATO H, 1992, INT J CANCER, V50, P49, DOI 10.1002/ijc.2910500111

KUWANO H, 1994, J SURG ONCOL, V57, P17, DOI 10.1002/jso.2930570106

Lin DC, 2014, NAT GENET, V46, P467, DOI 10.1038/ng.2935

Liu ZY, 2017, BIOMATERIALS, V116, P34, DOI 10.1016/j.biomaterials.2016.11.041

Liu ZY, 2015, BIOMED OPT EXPRESS, V6, P2294, DOI 10.1364/BOE.6.002294

Makale M, 2009, J BIOMED OPT, V14, DOI 10.1117/1.3103783

McConnell G, 2006, J BIOMED OPT, V11, DOI 10.1117/1.2360593

Murari K, 2011, OPT LETT, V36, P1299, DOI 10.1364/OL.36.001299

Nielsen T, 2001, J MICROSC-OXFORD, V201, P368, DOI 10.1046/j.1365-2818.2001.00852.x

Perry SW, 2012, ANN BIOMED ENG, V40, P277, DOI 10.1007/s10439-012-0512-9

Robinson PS, 2009, TISSUE ENG PT A, V15, P2763, DOI 10.1089/ten.tea.2008.0426

Rogart JN, 2008, CLIN GASTROENTEROL H, V6, P95, DOI 10.1016/j.cgh.2007.10.008

Schie IW, 2015, ANALYST, V140, P3897, DOI 10.1039/c5an00178a

Sivaguru M, 2010, OPT EXPRESS, V18, P24983, DOI 10.1364/OE.18.024983

Sun TL, 2010, J BIOMED OPT, V15, DOI 10.1117/1.3427146

TAKUBO K, 1990, CANCER, V65, P1816, DOI 10.1002/1097-0142(19900415)65:8<1816::AID-CNCR2820650825>3.0.CO;2-L

Thomopoulos S, 2006, J BIOMECH, V39, P1842, DOI 10.1016/j.jbiomech.2005.05.021

Tilbury K, 2015, PERSPECT MED CHEM, V7, P21, DOI 10.4137/PMC.S13214

TSUTSUI S, 1995, ANN SURG, V222, P193, DOI 10.1097/00000658-199508000-00012

Xu J, 2017, LASER PHYS LETT, V14, DOI 10.1088/1612-202X/aa519f

Xu J, 2013, SCANNING, V35, P387, DOI 10.1002/sca.21079

Yuasa N, 2004, LANGENBECK ARCH SURG, V389, P122, DOI 10.1007/s00423-003-0453-8

Zipfel WR, 2003, P NATL ACAD SCI USA, V100, P7075, DOI 10.1073/pnas.0832308100

NR 35

TC 17

Z9 18

U1 1

U2 19

PU OPTICAL SOC AMER

PI WASHINGTON

PA 2010 MASSACHUSETTS AVE NW, WASHINGTON, DC 20036 USA

SN 2156-7085

J9 BIOMED OPT EXPRESS

JI Biomed. Opt. Express

PD JUL 1

PY 2017

VL 8

IS 7

BP 3360

EP 3368

DI 10.1364/BOE.8.003360

PG 9

WC Biochemical Research Methods; Optics; Radiology, Nuclear Medicine &

Medical Imaging

WE Science Citation Index Expanded (SCI-EXPANDED)

SC Biochemistry & Molecular Biology; Optics; Radiology, Nuclear Medicine &

Medical Imaging

GA EZ5FJ

UT WOS:000404738300015

PM 28717572

OA gold, Green Published

DA 2022-05-19

ER

PT J

AU Qian, WX

Li, GM

AF Qian, Wenxiu

Li, Guomin

TI Extracellular Vesicles from Esophageal Squamous Cell Carcinoma Promote

Angiogenesis and Tumor Growth

SO JOURNAL OF BIOMATERIALS AND TISSUE ENGINEERING

LA English

DT Article

DE ESCC; Angiogenesis; Extracellular Vesicles

ID ENDOTHELIAL-CELLS; EXOSOMES

AB Angiogenesis is a prerequisite for tumor development and metastasis. Emerging evidence shows that tumor-derived extracellular vesicles (EVs) are an important component of tumor microenvironment, which participate in the communication between normal cells and tumor cells. In this study, we aimed to investigate the role of EVs derived from esophageal squamous cell carcinoma (ESCC) on tumor angiogenesis. We found that ESCC cell-derived EVs promoted the proliferation, migration, and tubule formation of human umbilical vein endothelial cells in vitro, and enhanced angiogenesis and tumor growth in vivo. Our results suggest that ESCC cell-derived EVs could promote angiogenesis and tumor growth, which also indicated the application of EVs as a valuable therapeutic strategy of ESCC.

C1 [Qian, Wenxiu] Jiangsu Univ, Jintan Hosp, Dept Pediat, Changzhou 213200, Jiangsu, Peoples R China.

[Li, Guomin] Jiangsu Univ, Jintan Hosp, Dept Crit Care Med, Changzhou 213200, Jiangsu, Peoples R China.

RP Li, GM (corresponding author), Jiangsu Univ, Jintan Hosp, Dept Crit Care Med, Changzhou 213200, Jiangsu, Peoples R China.

CR Ferlay J, 2015, INT J CANCER, V136, pE359, DOI 10.1002/ijc.29210

Folkman J, 2002, SEMIN ONCOL, V29, P15, DOI 10.1053/sonc.2002.37263

Kalluri R, 2016, J CLIN INVEST, V126, P1208, DOI 10.1172/JCI81135

Kara-Terki L, 2020, INT J MOL SCI, V21, DOI 10.3390/ijms21176024

Kim DH, 2020, CANCER LETT, V475, P2, DOI 10.1016/j.canlet.2020.01.023

Lagergren J, 2017, LANCET, V390, P2383, DOI 10.1016/S0140-6736(17)31462-9

Latifkar A, 2019, J CELL SCI, V132, DOI 10.1242/jcs.222406

Ma YB, 2019, J CELL MOL MED, V23, P2822, DOI 10.1111/jcmm.14190

Maacha S, 2019, MOL CANCER, V18, DOI 10.1186/s12943-019-0965-7

Mao Y, 2019, J EXP CLIN CANC RES, V38, DOI 10.1186/s13046-019-1384-8

Margolis L, 2019, PLOS BIOL, V17, DOI 10.1371/journal.pbio.3000363

Momen-Heravi F, 2017, METHODS MOL BIOL, V1660, P25, DOI 10.1007/978-1-4939-7253-1_3

Pontecorvi G, 2020, CYTOKINE GROWTH F R, V51, P75, DOI 10.1016/j.cytogfr.2019.12.010

Rashed MH, 2017, INT J MOL SCI, V18, DOI 10.3390/ijms18030538

Sato S, 2019, JCI INSIGHT, V4, DOI 10.1172/jci.insight.132447

Siegel RL, 2019, CA-CANCER J CLIN, V69, P7, DOI 10.3322/caac.21551

Sobierajska K, 2020, ADV EXP MED BIOL, V1234, P71, DOI 10.1007/978-3-030-37184-5_6

Song W, 2018, BIOMED PHARMACOTHER, V102, P1203, DOI 10.1016/j.biopha.2018.03.148

Tkach M, 2016, CELL, V164, P1226, DOI 10.1016/j.cell.2016.01.043

Viallard C, 2017, ANGIOGENESIS, V20, P409, DOI 10.1007/s10456-017-9562-9

Vu LT, 2020, CELL PROLIFERAT, V53, DOI 10.1111/cpr.12877

Wang ZF, 2019, J EXP CLIN CANC RES, V38, DOI 10.1186/s13046-019-1181-4

Yang HO, 2018, MOL THER, V26, P2466, DOI 10.1016/j.ymthe.2018.07.023

Zhou XH, 2019, INT J ONCOL, V54, P1843, DOI 10.3892/ijo.2019.4747

Zhu JM, 2019, THERANOSTICS, V9, P6901, DOI 10.7150/thno.37357

NR 25

TC 0

Z9 0

U1 2

U2 2

PU AMER SCIENTIFIC PUBLISHERS

PI VALENCIA

PA 26650 THE OLD RD, STE 208, VALENCIA, CA 91381-0751 USA

SN 2157-9083

EI 2157-9091

J9 J BIOMATER TISS ENG

JI J. Biomater. Tissue Eng.

PD FEB

PY 2022

VL 12

IS 2

BP 293

EP 298

DI 10.1166/jbt.2022.2896

PG 6

WC Cell & Tissue Engineering

WE Science Citation Index Expanded (SCI-EXPANDED)

SC Cell Biology

GA XA6LT

UT WOS:000720756400001

DA 2022-05-19

ER

PT J

AU Zhang, ZH

Xu, MQ

Guo, MF

Liu, XG

AF Zhang, Zhenghua

Xu, Meiqing

Guo, Mingfa

Liu, Xuegang

TI Long-term outcomes of minimally invasive Ivor Lewis esophagostomy for

esophageal squamous cell carcinoma: Compared with open approach

SO INTERNATIONAL JOURNAL OF SURGERY

LA English

DT Article

DE Esophageal squamous cell carcinoma; Thoracoscopy; Laparoscopy; Ivor

Lewis esophagostomy

ID ESOPHAGOGASTRIC ANASTOMOSIS; CANCER; COMPLICATIONS; SINGLE

AB Objective: To investigate the safety and long-term efficacy of combined thoraco-laparoscopic minimally invasive Ivor Lewis esophagostomy(MI-ILE) in the treatment of esophageal squamous cell carcinoma.

Methods: The clinical data of patients with esophageal squamous cell carcinoma who underwent Ivor Lewis esophagostomy of esophageal cancer from October 2011 to June 2013 were retrospectively analyzed. Of which 90 patients received MI-ILE, 95 patients underwent open Ivor Lewis esophagostomy (O-ILE). The clinicopathological features, intraoperative records and incidences of postoperative complications of the two groups were compared with t-test and x(2) test. The primary end point of the study was 3-year disease-free survival (DFS) and 3-year overall survival (OS) was a secondary end point.

Results: There were no statistically significant differences in gender, age, preoperative comorbidities, American Society of Anesthesiologists score and position of the tumor between the two groups. There was also no significant difference in clinicopathological characteristics, operation time, length of tumor resection margin and number of resected lymph nodes between the two groups (P > 0.05). In MI-ILE group, the blood loss was lower than in the O-ILE group [(159.1 + 97.4) ml vs. (191.7 + 141.9) ml, t = 1.811, P = 1.811] and the postoperative hospital stay was shorter [(11.5 + 4.5) d vs. (13.9 + 6.2) d, t = 2.944, P = 0.004]. There was no significant difference in the incidences of perioperative mortality and major morbidities (P > 0.05). Minor complications including incision infection rate (1.1% vs 8.4%, x(2) = 3.873, P = 0.049) and pulmonary infection incidence (3.3% vs 11.57%, x(2) = 4.492, P = 0.034) is lower in MIILE group. There was no significant difference in 3-year disease-free survival (DFS) and 3-year overall survival (OS) between the two groups.

Conclusion: MI-ILE is a technically safe and feasible approach for esophageal squamous cell carcinoma treatment. The oncologic outcomes of MI-ILE are comparable to that of O-ILE 3 years after resection. (C) 2017 IJS Publishing Group Ltd. Published by Elsevier Ltd. All rights reserved.

C1 [Zhang, Zhenghua] Shandong Univ, 17923 Jingshi Rd, Jinan 250000, Shandong, Peoples R China.

[Xu, Meiqing; Guo, Mingfa] Anhui Prov Hosp, Dept Thorac Surg, 17 Lujiang Rd, Hefei 230000, Anhui, Peoples R China.

[Liu, Xuegang] Bengbu Med Coll, Dept Thorac Surg, Affiliated Hosp 1, 287 Changhuai Rd, Bengbu 233000, Anhui, Peoples R China.

RP Liu, XG (corresponding author), Bengbu Med Coll, Dept Thorac Surg, Affiliated Hosp 1, 287 Changhuai Rd, Bengbu 233000, Anhui, Peoples R China.

EM liuxuegang_byyfy@yeah.net

CR Berger AC, 2011, J AM COLL SURGEONS, V212, P560, DOI 10.1016/j.jamcollsurg.2010.12.042

Chen BF, 2013, PLOS ONE, V8, DOI 10.1371/journal.pone.0082428

Dantoc M, 2012, ARCH SURG-CHICAGO, V147, P768, DOI 10.1001/archsurg.2012.1326

Dantoc MM, 2012, J GASTROINTEST SURG, V16, P486, DOI 10.1007/s11605-011-1792-3

Dindo D, 2004, ANN SURG, V240, P205, DOI 10.1097/01.sla.0000133083.54934.ae

Dong YN, 2015, ANN THORAC SURG, V99, P1459, DOI 10.1016/j.athoracsur.2014.12.047

Guven H, 2014, SURG LAPARO ENDO PER, V24, pE151, DOI 10.1097/SLE.0000000000000014

Hochwald SN, 2012, J GASTROINTEST SURG, V16, P1775, DOI 10.1007/s11605-012-1895-5

Kauppi J, 2015, SURG ENDOSC, V29, P2614, DOI 10.1007/s00464-014-3978-8

Luketich JD, 2000, ANN THORAC SURG, V70, P906, DOI 10.1016/S0003-4975(00)01711-2

Maas KW, 2015, WORLD J SURG, V39, P1986, DOI 10.1007/s00268-015-3100-y

Mao T, 2015, THORAC CANCER, V6, P303, DOI 10.1111/1759-7714.12184

Noble F, 2013, DIS ESOPHAGUS, V26, P263, DOI 10.1111/j.1442-2050.2012.01356.x

Orringer MB, 2000, ANN THORAC SURG, V70, P911

Sihag S, 2012, EUR J CARDIO-THORAC, V42, P430, DOI 10.1093/ejcts/ezs031

Singh RK, 2011, ARCH SURG-CHICAGO, V146, P711, DOI 10.1001/archsurg.2011.146

Tapias LF, 2012, DIS ESOPHAGUS, V25, P449, DOI 10.1111/j.1442-2050.2011.01269.x

Wang HB, 2016, WORLD J SURG, V40, P1655, DOI 10.1007/s00268-016-3482-5

Wang WL, 2015, INT J CLIN EXP MED, V8, P15983

Xiao P, 2015, ANN THORAC SURG, V100, P2372, DOI 10.1016/j.athoracsur.2015.04.140

Xie MR, 2014, ANN THORAC SURG, V97, P1721, DOI 10.1016/j.athoracsur.2014.01.054

Zeng FC, 2014, J THORAC DIS, V6, P152, DOI 10.3978/j.issn.2072-1439.2014.02.04

Zhou C, 2015, WORLD J SURG ONCOL, V13, DOI 10.1186/s12957-015-0661-z

Zhu YW, 2015, J BUON, V20, P1585

NR 24

TC 5

Z9 5

U1 1

U2 2

PU ELSEVIER SCIENCE BV

PI AMSTERDAM

PA PO BOX 211, 1000 AE AMSTERDAM, NETHERLANDS

SN 1743-9191

EI 1743-9159

J9 INT J SURG

JI Int. J. Surg.

PD SEP

PY 2017

VL 45

BP 98

EP 104

DI 10.1016/j.ijsu.2017.07.104

PG 7

WC Surgery

WE Science Citation Index Expanded (SCI-EXPANDED)

SC Surgery

GA FH1KW

UT WOS:000410899900016

PM 28778695

OA Bronze

DA 2022-05-19

ER

PT J

AU Zhu, HP

Lu, XL

Jiang, J

Lu, JF

Sun, XC

Zuo, Y

AF Zhu, Huiping

Lu, Xiaoling

Jiang, Jian

Lu, Jingfeng

Sun, Xinchen

Zuo, Yun

TI Radiotherapy Combined With Concurrent Nedaplatin-Based Chemotherapy for

Stage II-III Esophageal Squamous Cell Carcinoma

SO DOSE-RESPONSE

LA English

DT Article

DE Esophageal squamous cell carcinoma; concurrent chemoradiotherapy; dose

fractionation; radiation; nedaplatin

ID RADIATION-THERAPY; PLUS CISPLATIN; CANCER; CHEMORADIOTHERAPY; TRIAL;

IRRADIATION; RECURRENCE; PATTERNS; OUTCOMES; S-1

AB Objective This study was conducted to explore the appropriate radical radiation dose in concurrent chemoradiotherapy (CCRT) for patients with inoperable stage II-III esophageal squamous cell carcinoma (ESCC). Methods This retrospective study included patients with esophageal cancer (EC) from the database of patients treated at the Affiliated Zhangjiagang Hospital of Soochow University (1/2015-12/2019). Overall survival (OS), progression-free survival (PFS), objective remission rate (ORR), first failure pattern, and toxicities were collected. Results 112 patients treated with intensity-modulated radiation therapy (IMRT) combined with concurrent chemotherapy of nedaplatin-based regimens were included. Fifty-eight (51.8%) and 54 (48.2%) patients received 60 (HD) and 50.4 (LD) Gy of radiotherapy, respectively. The HD group showed superior OS and a trend for longer PFS compared with the LD group (median OS: 25.5 vs 17.5 months, P = .021; median PFS: 14.0 vs 10.5 months, P = .076). There were more patients with a complete remission (CR) in the HD group than in the LD group (P=.016). The treatment-related toxicities were generally acceptable, but HD radiotherapy would increase the incidence of grade >= 3 late radiotoxicity (22.4% vs 5.6%, P = .011). Conclusion In nedaplatin-based CCRT for stage II-III ESCC, the radiotherapy dose of 60 Gy achieved a better prognosis. Strengths and limitations of this study A comparative study of 50.4 Gy and 60 Gy was conducted to evaluate whether 50.4 Gy can be used as a radical radiotherapy dose for inoperable stage II-III esophageal squamous cell carcinoma from a real-world perspective. The highly consistent selection criteria in our study make analysis results highly reliable and scientific. The existing research results support that nedaplatin can be used in concurrent chemoradiotherapy for esophageal squamous cell carcinoma, and this study focuses on the discovery of a better nedaplatin-based combination regimen. The findings of this study are limited to a single-center study with a non-large sample size. Inevitably, recall bias may exist in this retrospective study. Surgery was not involved in the follow-up treatment after concurrent chemoradiotherapy, which may worsen the prognosis of some patients.

C1 [Zhu, Huiping; Lu, Xiaoling; Jiang, Jian; Lu, Jingfeng; Zuo, Yun] Soochow Univ, Affiliated Zhangjiagang Hosp, Dept Oncol, 68 Jiyang West Rd, Suzhou 215600, Jiangsu, Peoples R China.

[Sun, Xinchen] Nanjing Med Univ, Affiliated Hosp 1, Dept Radiat Oncol, 300 Guangzhou Rd, Nanjing 210029, Jiangsu, Peoples R China.

RP Zuo, Y (corresponding author), Soochow Univ, Affiliated Zhangjiagang Hosp, Dept Oncol, 68 Jiyang West Rd, Suzhou 215600, Jiangsu, Peoples R China.; Sun, XC (corresponding author), Nanjing Med Univ, Affiliated Hosp 1, Dept Radiat Oncol, 300 Guangzhou Rd, Nanjing 210029, Jiangsu, Peoples R China.

EM xinchen_sun@163.com; zuoyun_zjgyy@163.com

FU Zhangjiagang City Science and Technology Support Program (Social

Development) [ZKS1816]

FX This study was supported by grants from the Zhangjiagang City Science

and Technology Support Program (Social Development) (#ZKS1816).

CR Ajani JA, 2013, EUR J CANCER, V49, P3616, DOI 10.1016/j.ejca.2013.07.003

Ajani JA., 2021, ESOPHAGEAL ESOPHAGOG

Bodoky Gyorgy, 2015, J Gastrointest Cancer, V46, P109, DOI 10.1007/s12029-014-9680-1

Chang CL, 2017, RADIOTHER ONCOL, V125, P73, DOI 10.1016/j.radonc.2017.08.025

Chang H, 2014, CANCER CHEMOTH PHARM, V73, P665, DOI 10.1007/s00280-013-2371-y

Chen WQ, 2012, CHINESE J CANCER RES, V24, P1, DOI 10.1007/s11670-012-0001-6

Chen Y, 2017, ONCOTARGET, V8, P89095, DOI 10.18632/oncotarget.18760

Chiu PWY, 2005, J GASTROINTEST SURG, V9, P794, DOI 10.1016/j.gassur.2005.05.005

Cohen DJ, 2011, EXPERT OPIN PHARMACO, V12, P225, DOI 10.1517/14656566.2010.517748

Cooper JS, 1999, JAMA-J AM MED ASSOC, V281, P1623, DOI 10.1001/jama.281.17.1623

Cosset J M, 1998, Cancer Radiother, V2, P523, DOI 10.1016/S1278-3218(98)80067-X

Di Pardo BJ, 2016, WORLD J SURG, V40, P395, DOI 10.1007/s00268-015-3356-2

Hartmann JT, 2003, EXPERT OPIN PHARMACO, V4, P889, DOI 10.1517/14656566.4.6.889

HERSKOVIC A, 1992, NEW ENGL J MED, V326, P1593, DOI 10.1056/NEJM199206113262403

Hsu CH, 1999, ANTICANCER RES, V19, P4463

Hsu FM, 2011, INT J RADIAT ONCOL, V81, pE593, DOI 10.1016/j.ijrobp.2011.04.032

Kim HW, 2014, DIGESTION, V90, P254, DOI 10.1159/000368983

Koizumi W, 2008, LANCET ONCOL, V9, P215, DOI 10.1016/S1470-2045(08)70035-4

Liu Q, 2020, RADIAT ONCOL, V15, DOI 10.1186/s13014-020-01518-2

Lordick F, 2016, ANN ONCOL, V27, pv50, DOI 10.1093/annonc/mdw329

Luo HS, 2019, RADIAT ONCOL, V14, DOI 10.1186/s13014-019-1386-x

Minsky BD, 2002, J CLIN ONCOL, V20, P1167, DOI 10.1200/JCO.20.5.1167

Miyata H, 2011, ANN SURG ONCOL, V18, P3353, DOI 10.1245/s10434-011-1747-7

Ohnuma H, 2018, CANCER SCI, V109, P3554, DOI 10.1111/cas.13772

Onozawa M, 2009, RADIOTHER ONCOL, V92, P266, DOI 10.1016/j.radonc.2008.09.025

Oppedijk V, 2014, J CLIN ONCOL, V32, P385, DOI 10.1200/JCO.2013.51.2186

PETERS LJ, 1990, RADIOTHER ONCOL, V17, P177, DOI 10.1016/0167-8140(90)90202-8

Qiu XN, 2020, MEDICINE, V99, DOI 10.1097/MD.0000000000018732

Rice TW, 2010, ANN SURG ONCOL, V17, P1721, DOI 10.1245/s10434-010-1024-1

Song T, 2015, EXPERT REV ANTICANC, V15, P1157, DOI 10.1586/14737140.2015.1074041

Suh YG, 2014, JPN J CLIN ONCOL, V44, P534, DOI 10.1093/jjco/hyu047

Sun X, 2020, FRONT ONCOL, V10, DOI 10.3389/fonc.2020.01222

Sung H, 2021, CA-CANCER J CLIN, V71, P209, DOI 10.3322/caac.21660

Suntharalingam M, 2003, INT J RADIAT ONCOL, V56, P981, DOI 10.1016/S0360-3016(03)00256-6

Takeuchi M, 2019, WORLD J SURG, V43, P2006, DOI 10.1007/s00268-019-05000-3

Torre LA, 2016, CANCER EPIDEM BIOMAR, V25, P16, DOI 10.1158/1055-9965.EPI-15-0578

Tu LL, 2013, RADIAT ONCOL, V8, DOI 10.1186/1748-717X-8-75

West H, 2015, JAMA ONCOL, V1, P998, DOI 10.1001/jamaoncol.2015.3113

Xiao LL, 2020, J CANCER, V11, P4605, DOI 10.7150/jca.44447

Xu Rui-Hua, 2006, Ai Zheng, V25, P1565

Xu YJ, 2018, J CLIN ONCOL, V36, DOI 10.1200/JCO.2018.36.15_suppl.4013

Yamashita H, 2011, RADIOTHER ONCOL, V98, P255, DOI 10.1016/j.radonc.2010.10.021

Yoon DH, 2015, INT J RADIAT ONCOL, V91, P489, DOI 10.1016/j.ijrobp.2014.11.019

Zhang F, 2017, DIS ESOPHAGUS, V30, DOI 10.1111/dote.12490

Zhang Z, 2005, INT J RADIAT ONCOL, V61, P656, DOI 10.1016/j.ijrobp.2004.06.022

Zhao T, 2012, MED ONCOL, V29, P3017, DOI 10.1007/s12032-012-0228-6

Zhu HT, 2017, WORLD J GASTROENTERO, V23, P540, DOI 10.3748/wjg.v23.i3.540

Zhu HP, 2019, ONCOL RES TREAT, V42, P470, DOI 10.1159/000501594

Zhu HP, 2019, ONCOL LETT, V17, P594, DOI 10.3892/ol.2018.9564

NR 49

TC 0

Z9 0

U1 0

U2 0

PU SAGE PUBLICATIONS INC

PI THOUSAND OAKS

PA 2455 TELLER RD, THOUSAND OAKS, CA 91320 USA

SN 1559-3258

J9 DOSE-RESPONSE

JI Dose-Response

PD JAN

PY 2022

VL 20

IS 1

AR 15593258221076720

DI 10.1177/15593258221076720

PG 12

WC Pharmacology & Pharmacy; Radiology, Nuclear Medicine & Medical Imaging;

Toxicology

WE Science Citation Index Expanded (SCI-EXPANDED)

SC Pharmacology & Pharmacy; Radiology, Nuclear Medicine & Medical Imaging;

Toxicology

GA ZP1HV

UT WOS:000766176600001

PM 35273471

OA gold, Green Published

DA 2022-05-19

ER

PT J

AU Fendereski, M

Zia, MF

Shafiee, M

Safari, F

Saneie, MH

Tavassoli, M

AF Fendereski, Mona

Zia, Mohammad Farid

Shafiee, Mohammad

Safari, Forousan

Saneie, Mohammad Hosien

Tavassoli, Manoochehr

TI MicroRNA-196a as a Potential Diagnostic Biomarker for Esophageal

Squamous Cell Carcinoma

SO CANCER INVESTIGATION

LA English

DT Article

DE ESCC; miR-196a; biomarker; saliva

ID DOWN-REGULATION; EXPRESSION; ADENOCARCINOMA; ASSOCIATION; PROGNOSIS;

MECHANISM

AB We observed significant up-regulation of miR-196a in esophageal squamous cell carcinoma (ESCC) as compared with their adjacent normal tissue (p = .002). Receiver operating characteristics curve analysis confirmed the suitability of miR-196a as a potential tumor marker for diagnosis of ESCC. Furthermore, analysis of miR-196a levels in saliva samples determined an average of 27-fold up-regulations in ESCC patients compared with healthy group. Our results suggest that salivary miR-196a may be a suitable noninvasive biomarker for diagnosis of ESCC. In addition, molecular pathway enrichment analysis of microRNA (miR)-196a determined focal adhesion, spliceosome and p53 signaling pathways as the most relevant pathways with miR-196a targetome.

C1 [Fendereski, Mona; Safari, Forousan; Tavassoli, Manoochehr] Univ Isfahan, Fac Sci, Dept Biol, Esfahan, Iran.

[Zia, Mohammad Farid] Isfahan Univ Med Sci, Fac Pharmaceut Sci, Dept Pharmaceut Biotechnol, Gorgan, Iran.

[Shafiee, Mohammad] Golestan Univ Med Sci, Sch Adv Med Technol, Dept Med Genet, Gorgan, Iran.

[Saneie, Mohammad Hosien] Isfahan Univ Med Sci, Dept Pathol, Esfahan, Iran.

RP Tavassoli, M (corresponding author), Univ Isfahan, Fac Sci, Dept Biol, Esfahan, Iran.

EM manoochehr@biol.ui.ac.ir

RI Shafiee, Mohammad/L-5721-2017; MohammadShafiee, MohammadReza/J-2988-2013

OI Shafiee, Mohammad/0000-0002-8931-1301; Zia, Mohammad

Farid/0000-0002-2513-9565

FU University of Isfahan

FX This work was supported by the University of Isfahan.

CR Bartel DP, 2004, CELL, V116, P281, DOI 10.1016/S0092-8674(04)00045-5

Bloomston M, 2007, JAMA-J AM MED ASSOC, V297, P1901, DOI 10.1001/jama.297.17.1901

Braig S, 2010, CELL MOL LIFE SCI, V67, P3535, DOI 10.1007/s00018-010-0394-7

Chen CY, 2011, J CELL MOL MED, V15, P14, DOI 10.1111/j.1582-4934.2010.01219.x

Croce CM, 2009, NAT REV GENET, V10, P704, DOI 10.1038/nrg2634

De Martino I, 2009, ONCOGENE, V28, P1432, DOI 10.1038/onc.2008.495

Di Leva G, 2013, CURR OPIN GENET DEV, V23, P3, DOI 10.1016/j.gde.2013.01.004

Fan Y, 2015, INT J CLIN EXP PATHO, V8, P4132

Fukuhara Tatsuma, 2010, J Clin Gastroenterol, V44, pe27, DOI 10.1097/MCG.0b013e3181b31325

Hafner M, 2010, CELL, V141, P129, DOI 10.1016/j.cell.2010.03.009

He FC, 2015, WORLD J GASTROENTERO, V21, P4660, DOI 10.3748/wjg.v21.i15.4660

Helwak A, 2013, CELL, V153, P654, DOI 10.1016/j.cell.2013.03.043

Hsu SD, 2011, NUCLEIC ACIDS RES, V39, pD163, DOI 10.1093/nar/gkq1107

Huang DW, 2009, NAT PROTOC, V4, P44, DOI 10.1038/nprot.2008.211

Hui ABY, 2009, LAB INVEST, V89, P597, DOI 10.1038/labinvest.2009.12

Jemal A, 2011, CA-CANCER J CLIN, V61, P69, DOI [10.3322/caac.20107, 10.3322/caac.20115]

Kimura S, 2010, ONCOL REP, V23, P1625, DOI 10.3892/or_00000804

Kunej T, 2012, CANCER J, V18, P223, DOI 10.1097/PPO.0b013e318258b771

Li BX, 2015, INT J CLIN EXP MED, V8, P7241

Lin DC, 2014, NAT GENET, V46, P467, DOI 10.1038/ng.2935

Luthra R, 2008, ONCOGENE, V27, P6667, DOI 10.1038/onc.2008.256

Maru DM, 2009, AM J PATHOL, V174, P1940, DOI 10.2353/ajpath.2009.080718

Mathe EA, 2009, CLIN CANCER RES, V15, P6192, DOI 10.1158/1078-0432.CCR-09-1467

Mu YP, 2014, ASIAN PAC J CANCER P, V15, P8893, DOI 10.7314/APJCP.2014.15.20.8893

Qi B, 2015, WORLD J GASTROENTERO, V21, P6884, DOI 10.3748/wjg.v21.i22.6884

Roth C, 2010, BREAST CANCER RES, V12, DOI 10.1186/bcr2766

Wang GQ, 2005, GUT, V54, P187, DOI 10.1136/gut.2004.046631

Wu BL, 2011, WORLD J GASTROENTERO, V17, P79, DOI 10.3748/wjg.v17.i1.79

Xiong HZ, 2014, PLOS ONE, V9, DOI 10.1371/journal.pone.0110163

Yekta S, 2004, SCIENCE, V304, P594, DOI 10.1126/science.1097434

Zaravinos A, 2015, J ONCOL, V2015, DOI 10.1155/2015/865816

Zhao YQ, 2013, INT J CANCER, V132, P2901, DOI 10.1002/ijc.27954

Zhu Y, 2014, CELL PHYSIOL BIOCHEM, V33, P1340, DOI 10.1159/000358701

NR 33

TC 17

Z9 17

U1 1

U2 8

PU TAYLOR & FRANCIS INC

PI PHILADELPHIA

PA 530 WALNUT STREET, STE 850, PHILADELPHIA, PA 19106 USA

SN 0735-7907

EI 1532-4192

J9 CANCER INVEST

JI Cancer Invest.

PY 2017

VL 35

IS 2

BP 78

EP 84

DI 10.1080/07357907.2016.1254228

PG 7

WC Oncology

WE Science Citation Index Expanded (SCI-EXPANDED)

SC Oncology

GA EM3BO

UT WOS:000395189700002

PM 28095062

DA 2022-05-19

ER

PT J

AU Wong, IYH

Lam, KO

Chan, W

Wong, CD

So, TH

Chan, KK

Choi, CW

Law, TT

Chiu, K

Chan, FSY

Kwong, DLW

Law, S

AF Wong, Ian Yu Hong

Lam, Ka On

Chan, Wendy

Wong, Claudia

So, Tsz Him

Chan, Kwan Kit

Choi, Cheuk Wai

Law, Tsz Ting

Chiu, Keith

Chan, Fion Siu Yin

Kwong, Dora Lai Wan

Law, Simon

TI Real-world Scenario: CROSS Regimen as Preoperative Therapy for

Oesophageal Squamous Cell Carcinoma

SO JOURNAL OF GASTROINTESTINAL SURGERY

LA English

DT Article

DE Oesophageal cancer; Oesophageal squamous cell carcinoma;

Chemoradiotherapy; Neoadjuvant therapy; Oesophagectomy

ID NEOADJUVANT CHEMORADIOTHERAPY; TREATMENT RESPONSE; CANCER;

CHEMORADIATION; REGRESSION; LONG; CISPLATIN; DOCETAXEL; IMPACT

AB Background Preoperative chemoradiation (CROSS regimen) has been widely adopted worldwide. The survival advantage imparted is especially impressive for oesophageal squamous cell carcinoma (OSCC). This study aimed at investigating the efficacy of the CROSS regimen in real-world scenario.

Methods This is a retrospective study of all patients with OSCC intended for preoperative treatment using the CROSS regimen during 2012-2017. Patients were divided into two groups: those within the selection criteria in the CROSS trial and those beyond criteria, namely age > 75 years old, tumour length > 8 cm or clinical M1 stage of lymph node involvement (AJCC 6th edition). Clinical outcome and survival data were compared.

Results Eighty-eight patients were included. There were 46 patients in the "CROSS eligible" group and 42 in the "CROSS ineligible" group. By intention-to-treat, the estimated median survival was 24.2 months vs. 12.7 months, respectively (p = 0.047). The results were much inferior compared to that published in the original CROSS trial. Univariable and multivariable analyses showed tumour length and resectability as independent variables affecting survival.

Discussion In a real-world scenario, the clinical outcome remains suboptimal and the excellent results in the trial setting were not reproducible in this Asian cohort. Patient selection is one key element accountable for the difference. The efficacy of the CROSS regimen may not be adequate for patients with more advanced disease. The optimal multimodal therapy for this group of patients remains undefined.

C1 [Wong, Ian Yu Hong; Wong, Claudia; Chan, Kwan Kit; Law, Tsz Ting; Chan, Fion Siu Yin; Law, Simon] Univ Hong Kong, Dept Surg, Hong Kong, Peoples R China.

[Lam, Ka On; Chan, Wendy; So, Tsz Him; Choi, Cheuk Wai; Kwong, Dora Lai Wan] Univ Hong Kong, Dept Clin Oncol, Hong Kong, Peoples R China.

[Chiu, Keith] Univ Hong Kong, Dept Diagnost Radiol, Hong Kong, Peoples R China.

RP Law, S (corresponding author), Univ Hong Kong, Dept Surg, Hong Kong, Peoples R China.

EM slaw@hku.hk

CR AKIYAMA H, 1994, ANN SURG, V220, P364, DOI 10.1097/00000658-199409000-00012

Al-Batran SE, 2019, LANCET, V393, P1948, DOI 10.1016/S0140-6736(18)32557-1

Al-Batran SE, 2016, LANCET ONCOL, V17, P1697, DOI 10.1016/S1470-2045(16)30531-9

Ando N, 2012, ANN SURG ONCOL, V19, P68, DOI 10.1245/s10434-011-2049-9

Bray F, 2018, CA-CANCER J CLIN, V68, P394, DOI 10.3322/caac.21492

Gusella M, 2018, PHARMACOGENOMICS J, V18, P14, DOI 10.1038/tpj.2017.25

Haisley KR, 2017, DIS ESOPHAGUS, V30, DOI 10.1093/dote/dox015

Jarvinen T, 2018, WORLD J SURG ONCOL, V16, DOI 10.1186/s12957-018-1327-4

Law S, 1997, J THORAC CARDIOV SUR, V114, P210, DOI 10.1016/S0022-5223(97)70147-8

Law S, 2003, ANN SURG, V238, P339, DOI 10.1097/01.sla.0000086545.45918.ee

Law S, 2012, MASTERY SURG, P886

Law S, 2006, J GASTROINTEST SURG, V10, P1301, DOI 10.1016/j.gassur.2006.06.009

Law TT, 2016, DIS ESOPHAGUS S1, V29, p128A, DOI [10.1111/dote.12528, DOI 10.1111/DOTE.12528]

MANDARD AM, 1994, CANCER, V73, P2680, DOI 10.1002/1097-0142(19940601)73:11<2680::AID-CNCR2820731105>3.0.CO;2-C

Murimwa GZ, 2017, J GASTROINTEST ONCOL, V8, P808, DOI 10.21037/jgo.2017.06.11

Nakamura K, 2013, JPN J CLIN ONCOL, V43, P752, DOI 10.1093/jjco/hyt061

Sasaki K, 2019, CANCER CHEMOTH PHARM, V83, P581, DOI 10.1007/s00280-018-03764-4

Shapiro J, 2015, LANCET ONCOL, V16, P1090, DOI 10.1016/S1470-2045(15)00040-6

Tong DKH, 2010, ANN SURG ONCOL, V17, P2184, DOI 10.1245/s10434-010-0995-2

van Hagen P, 2012, NEW ENGL J MED, V366, P2074, DOI 10.1056/NEJMoa1112088

Wang QL, 2018, CLIN EPIDEMIOL, V10, P717, DOI 10.2147/CLEP.S166078

Wong C, 2017, J THORAC DIS, V9, pS773, DOI 10.21037/jtd.2017.04.29

Wong I, 2018, ESOPHAGEAL CANC DIAG, P99

Wong I, 2018, BEST PRACT RES CL GA, V36-37, P85, DOI 10.1016/j.bpg.2018.11.012

Wong I, 2016, TRANSL CANCER RES, V5, pS415, DOI 10.21037/tcr.2016.08.32

Yang H, 2018, J CLIN ONCOL, V36, P2796, DOI 10.1200/JCO.2018.79.1483

Yuan H, 2016, NUCL MED COMMUN, V37, P947, DOI 10.1097/MNM.0000000000000527

NR 27

TC 6

Z9 6

U1 0

U2 2

PU SPRINGER

PI NEW YORK

PA ONE NEW YORK PLAZA, SUITE 4600, NEW YORK, NY, UNITED STATES

SN 1091-255X

EI 1873-4626

J9 J GASTROINTEST SURG

JI J. Gastrointest. Surg.

PD SEP

PY 2020

VL 24

IS 9

BP 1937

EP 1947

DI 10.1007/s11605-020-04704-5

EA JUL 2020

PG 11

WC Gastroenterology & Hepatology; Surgery

WE Science Citation Index Expanded (SCI-EXPANDED)

SC Gastroenterology & Hepatology; Surgery

GA NR1UF

UT WOS:000548835800010

PM 32671794

DA 2022-05-19

ER

PT J

AU Mynhardt, C

Damelin, LH

Jivan, R

Peres, J

Prince, S

Veale, RB

Mavri-Damelin, D

AF Mynhardt, Charissa

Damelin, Leonard H.

Jivan, Rupal

Peres, Jade

Prince, Sharon

Veale, Robin B.

Mavri-Damelin, Demetra

TI Metformin-induced alterations in nucleotide metabolism cause

5-fluorouracil resistance but gemcitabine susceptibility in oesophageal

squamous cell carcinoma

SO JOURNAL OF CELLULAR BIOCHEMISTRY

LA English

DT Article

DE gemcitabine; metformin; oesophageal squamous cell carcinoma; thymidylate

synthase; 5-fluorouracil

ID PANCREATIC-CANCER CELLS; THYMIDYLATE SYNTHASE; GENOMIC IMBALANCES;

INDUCED APOPTOSIS; DNA-DAMAGE; KINASE; LINES; 2,2-DIFLUORODEOXYCYTIDINE;

COMBINATION; MECHANISMS

AB 5-Fluorouracil (5-FU) is a chemotherapeutic agent used to treat a variety of gastric cancers including oesophageal squamous cell carcinoma (OSCC), for which the 5-year mortality rate exceeds 85%. Our study investigated the effects of metformin, an antidiabetic drug with established anti-cancer activity, in combination with 5-FU as a novel chemotherapy strategy, using the OSCC cell lines, WHCO1 and WHCO5. Our results indicate that metformin treatment induces significant resistance to 5-FU in WHCO1 and WHCO5 cells, by more than five- and sixfolds, respectively, as assessed by MTT assay. We show that this is due to global alterations in nucleotide metabolism, including elevated expression of thymidylate synthase and thymidine kinase 1 (established 5-FU resistance mechanisms), which likely result in an increase in intracellular dTTP pools and a dilution of 5-FU anabolites. Metformin treatment also increases deoxycytidine kinase (dCK) expression and, as the chemotherapeutic agent gemcitabine relies on dCK for its efficient activity, we speculated that metformin would enhance the sensitivity of OSCC cells to gemcitabine. Indeed we show that metformin pre-treatment greatly increases gemcitabine toxicity and DNA fragmentation in comparison to gemcitabine alone. Taken together, our findings show that metformin alters nucleotide metabolism in OSCC cells and while responsible for inducing resistance to 5-FU, it conversely increases sensitivity to gemcitabine, thereby highlighting metformin and gemcitabine as a potentially novel combination therapy for OSCC.

C1 [Mynhardt, Charissa; Jivan, Rupal; Veale, Robin B.; Mavri-Damelin, Demetra] Univ Witwatersrand, Sch Mol & Cell Biol, Johannesburg, South Africa.

[Damelin, Leonard H.] Univ Witwatersrand, Fac Hlth Sci, Sch Pathol, Johannesburg, South Africa.

[Damelin, Leonard H.] Natl Inst Communicable Dis, Cell Biol Grp, Ctr HIV & STIs, Johannesburg, South Africa.

[Peres, Jade; Prince, Sharon] Univ Cape Town, Fac Hlth Sci, Dept Human Biol, Div Cell Biol, Cape Town, South Africa.

RP Mavri-Damelin, D (corresponding author), Univ Witwatersrand, Sch Mol & Cell Biol, Private Bag X3, ZA-2050 Johannesburg, South Africa.

EM demetra.mavri-damelin@wits.ac.za

FU National Research Foundation of South Africa [90710]; Cancer Association

of South Africa [DMD]

FX National Research Foundation of South Africa, Grant number: 90710;

Cancer Association of South Africa, Grant number: DMD

CR Bellini MF, 2010, GENET MOL BIOL, V33, P205, DOI 10.1590/S1415-47572010005000028

Berg RW, 2002, CURR DRUG TARGETS, V3, P297, DOI 10.2174/1389450023347605

BOUFFARD DY, 1993, BIOCHEM PHARMACOL, V45, P1857, DOI 10.1016/0006-2952(93)90444-2

Boyer CR, 2002, ANTI-CANCER DRUG, V13, P29, DOI 10.1097/00001813-200201000-00003

BRADFORD MM, 1976, ANAL BIOCHEM, V72, P248, DOI 10.1016/0003-2697(76)90527-3

Brown J, 2011, WORLD J GASTROENTERO, V17, P2909, DOI 10.3748/wjg.v17.i24.2909

Buolamwini JK, 1997, CURR MED CHEM, V4, P35

Cavalcante LD, 2014, EUR J PHARMACOL, V741, P8, DOI [10.1010/j.ejphar.2014.07.041, 10.1016/j.ejphar.2014.07.041]

Chen YL, 2010, J BIOL CHEM, V285, P27327, DOI 10.1074/jbc.M110.137042

Chou TC, 2010, CANCER RES, V70, P440, DOI 10.1158/0008-5472.CAN-09-1947

Damelin LH, 2014, BMC CANCER, V14, DOI 10.1186/1471-2407-14-314

DeCensi A, 2010, CANCER PREV RES, V3, P1451, DOI 10.1158/1940-6207.CAPR-10-0157

Evans JMM, 2005, BMJ-BRIT MED J, V330, P1303, DOI [10.1136/bmj.38393.572188.EB, DOI 10.1136/BMJ.38393.572188.EB]

Feng Y, 2014, CELL DEATH DIS, V5, DOI 10.1038/cddis.2014.59

Feoktistova Maria, 2016, Cold Spring Harb Protoc, V2016, DOI 10.1101/pdb.prot087379

Ferlay J, 2010, INT J CANCER, V127, P2893, DOI 10.1002/ijc.25516

Fitzmaurice C, 2015, JAMA ONCOL, V1, P505, DOI 10.1001/jamaoncol.2015.0735

Grem JL, 2000, INVEST NEW DRUG, V18, P299, DOI 10.1023/A:1006416410198

Habiro A, 2004, BIOCHEM BIOPH RES CO, V316, P71, DOI 10.1016/j.bbrc.2004.02.017

Hao JJ, 2012, BMC CANCER, V12, DOI 10.1186/1471-2407-12-367

Harada K, 2016, INT J ONCOL, V49, P276, DOI 10.3892/ijo.2016.3523

HEINEMANN V, 1992, CANCER RES, V52, P533

HEINEMANN V, 1988, CANCER RES, V48, P4024

HUANG P, 1991, CANCER RES, V51, P6110

Iliopoulos D, 2011, CANCER RES, V71, P3196, DOI 10.1158/0008-5472.CAN-10-3471

Ilson David H, 2008, Gastrointest Cancer Res, V2, P85

Jivan R, 2015, J CELL BIOCHEM, V116, P2334, DOI 10.1002/jcb.25184

Kato K, 2011, INT J RADIAT ONCOL, V81, P684, DOI 10.1016/j.ijrobp.2010.06.033

Klein TJ, 2010, SEMIN RADIAT ONCOL, V20, P282, DOI 10.1016/j.semradonc.2010.05.006

Libby G, 2009, DIABETES CARE, V32, P1620, DOI 10.2337/dc08-2175

Longley DB, 2003, NAT REV CANCER, V3, P330, DOI 10.1038/nrc1074

Mini E, 2006, ANN ONCOL, V17, pV7, DOI 10.1093/annonc/mdj941

Nakakuki K, 2002, CARCINOGENESIS, V23, P19, DOI 10.1093/carcin/23.1.19

Nakashima M, 2011, CANCER LETT, V313, P218, DOI 10.1016/j.canlet.2011.09.008

Parker WB., 2007, CANC THER, V5B, P331

Pennathur A, 2013, LANCET, V381, P400, DOI 10.1016/S0140-6736(12)60643-6

Peters GJ, 2002, BBA-MOL BASIS DIS, V1587, P194, DOI 10.1016/S0925-4439(02)00082-0

Peters GJ., 2003, FLUOROPYRIMIDINES CA, P1

PRESSACCO J, 1995, CANCER RES, V55, P1505

Quinn BJ, 2013, TRENDS ENDOCRIN MET, V24, P469, DOI 10.1016/j.tem.2013.05.004

Rustgi AK, 2014, NEW ENGL J MED, V371, P2499, DOI 10.1056/NEJMra1314530

Vazquez-Martin A, 2011, CELL CYCLE, V10, P1499, DOI 10.4161/cc.10.9.15423

VEALE RB, 1989, S AFR J SCI, V85, P375

Yao SYM, 2011, J BIOL CHEM, V286, P32552, DOI 10.1074/jbc.M111.236117

Zhang N, 2008, MOLECULES, V13, P1551, DOI 10.3390/molecules13081551

NR 45

TC 9

Z9 10

U1 0

U2 14

PU WILEY

PI HOBOKEN

PA 111 RIVER ST, HOBOKEN 07030-5774, NJ USA

SN 0730-2312

EI 1097-4644

J9 J CELL BIOCHEM

JI J. Cell. Biochem.

PD JAN

PY 2018

VL 119

IS 1

BP 1193

EP 1203

DI 10.1002/jcb.26291

PG 11

WC Biochemistry & Molecular Biology; Cell Biology

WE Science Citation Index Expanded (SCI-EXPANDED)

SC Biochemistry & Molecular Biology; Cell Biology

GA FN5CO

UT WOS:000416024300115

PM 28722177

DA 2022-05-19

ER

PT J

AU Ming, XY

Fu, L

Zhang, LY

Qin, YR

Cao, TT

Chan, KW

Ma, S

Xie, D

Guan, XY

AF Ming, Xiao-Yan

Fu, Li

Zhang, Li-Yi

Qin, Yan-Ru

Cao, Ting-Ting

Chan, Kwok Wah

Ma, Stephanie

Xie, Dan

Guan, Xin-Yuan

TI Integrin alpha 7 is a functional cancer stem cell surface marker in

oesophageal squamous cell carcinoma

SO NATURE COMMUNICATIONS

LA English

DT Article

ID IDENTIFICATION; METASTASIS; PLASTICITY; BIOLOGY

AB Non-CG methylation has been associated with stemness regulation in embryonic stem cells. By comparing differentially expressed genes affected by non-CG methylation between tumour and corresponding non-tumour tissues in oesophageal squamous cell carcinoma (OSCC), we find that Integrin alpha 7 (ITGA7) is characterized as a potential cancer stem cell (CSC) marker. Clinical data show that a high frequency of ITGA7(+) cells in OSCC tissues is significantly associated with poor differentiation, lymph node metastasis and worse prognosis. Functional studies demonstrate that both sorted ITGA7(+) cells and ITGA7 overexpressing cells display enhanced stemness features, including elevated expression of stemness-associated genes and epithelial-mesenchymal transition features, as well as increased abilities to self-renew, differentiate and resist chemotherapy. Mechanistic studies find that ITGA7 regulates CSC properties through the activation of the FAK-mediated signalling pathways. As knockdown of ITGA7 can effectively reduce the stemness of OSCC cells, ITGA7 could be a potential therapeutic target in OSCC treatment.

C1 [Ming, Xiao-Yan; Zhang, Li-Yi; Cao, Ting-Ting; Guan, Xin-Yuan] Univ Hong Kong, Li Ka Shing Fac Med, Dept Clin Oncol, Hong Kong 852, Hong Kong, Peoples R China.

[Ming, Xiao-Yan; Zhang, Li-Yi; Chan, Kwok Wah; Ma, Stephanie; Guan, Xin-Yuan] Univ Hong Kong, Li Ka Shing Fac Med, Ctr Canc Res, Hong Kong 852, Hong Kong, Peoples R China.

[Fu, Li; Cao, Ting-Ting] Shenzhen Univ, Sch Med, Shenzhen Key Lab Translat Med Tumor & Canc Res Ct, Dept Pharmacol, Shenzhen 518000, Peoples R China.

[Qin, Yan-Ru] Zhengzhou Univ, Affiliated Hosp 1, Dept Clin Oncol, Zhengzhou 450052, Peoples R China.

[Chan, Kwok Wah] Univ Hong Kong, Li Ka Shing Fac Med, Dept Pathol, Hong Kong 852, Hong Kong, Peoples R China.

[Ma, Stephanie] Univ Hong Kong, Li Ka Shing Fac Med, Sch Biomed Sci, Hong Kong 852, Hong Kong, Peoples R China.

[Guan, Xin-Yuan] Sun Yat Sen Univ, Ctr Canc, State Key Lab Oncol Southern China, Guangzhou 510060, Guangdong, Peoples R China.

RP Guan, XY (corresponding author), Univ Hong Kong, Li Ka Shing Fac Med, Dept Clin Oncol, Hong Kong 852, Hong Kong, Peoples R China.; Guan, XY (corresponding author), Univ Hong Kong, Li Ka Shing Fac Med, Ctr Canc Res, Hong Kong 852, Hong Kong, Peoples R China.; Guan, XY (corresponding author), Sun Yat Sen Univ, Ctr Canc, State Key Lab Oncol Southern China, Guangzhou 510060, Guangdong, Peoples R China.

EM xyguan@hku.hk

RI Fu, Li/F-7182-2010; /A-3639-2009; Ma, Stephanie Kwai Yee/C-4766-2009

OI Fu, Li/0000-0003-2643-6278; /0000-0003-1874-9805; Ma, Stephanie Kwai

Yee/0000-0002-2029-7943

FU Hong Kong Research Grant Council (RGC) [C7038-14G, C7027-14G,

HKU/7668/11M, 767313, CUHK/766613]; NSFC/RGC Joint Research Scheme

[N_HKU712/12]; Hong Kong Health and Medical Research Found [02133366];

National Basic Research Program of China [2012CB967001]; China National

Key Sci-Tech Special Project of Infectious Diseases

[2013ZX10002-011-005]; National Natural Science Foundation of China

[81272416, 81372583]; Science and Technology Foundation of Shenzhen

[CXZZ20150430092951135, KQTD20140630100658078]

FX We thank the Faculty Core Facility of the LKS Faculty of Medicine at The

University of Hong Kong for help with the flow cytometry cell sorting,

as well as Dr. Jianhua Luo (Department of Pathology, University of

Pittsburgh School of Medicine) for providing the ITGA7-expressing

plasmid. This work was supported by grants from the Hong Kong Research

Grant Council (RGC) grants including Collaborative Research Funds

(C7038-14G and C7027-14G), General Research Funds (HKU/7668/11M, 767313

and CUHK/766613), NSFC/RGC Joint Research Scheme (N_HKU712/12), Hong

Kong Health and Medical Research Found (02133366), grants from the

National Basic Research Program of China (2012CB967001), China National

Key Sci-Tech Special Project of Infectious Diseases

(2013ZX10002-011-005), National Natural Science Foundation of China

(81272416 and 81372583) and the Science and Technology Foundation of

Shenzhen (CXZZ20150430092951135 and KQTD20140630100658078). Professor

X.Y. Guan is Sophie YM Chan Professor in Cancer Research.

CR Adorno-Cruz V, 2015, CANCER RES, V75, P924, DOI 10.1158/0008-5472.CAN-14-3225

Al-Hajj M, 2003, P NATL ACAD SCI USA, V100, P3983, DOI 10.1073/pnas.0530291100

Brabletz T, 2012, CANCER CELL, V22, P699, DOI 10.1016/j.ccr.2012.11.009

Brizzi MF, 2012, CURR OPIN CELL BIOL, V24, P645, DOI 10.1016/j.ceb.2012.07.001

Desgrosellier JS, 2010, NAT REV CANCER, V10, P9, DOI 10.1038/nrc2748

ElShamy WM, 2013, CANCER LETT, V341, P2, DOI 10.1016/j.canlet.2013.06.020

Ferlay J, 2010, INT J CANCER, V127, P2893, DOI 10.1002/ijc.25516

Ginestier C, 2007, CELL STEM CELL, V1, P555, DOI 10.1016/j.stem.2007.08.014

Golubovskaya VM, 2013, ANTI-CANCER AGENT ME, V13, P576

Hanahan D, 2011, CELL, V144, P646, DOI 10.1016/j.cell.2011.02.013

Hu YF, 2009, J IMMUNOL METHODS, V347, P70, DOI 10.1016/j.jim.2009.06.008

Lathia JD, 2010, CELL STEM CELL, V6, P421, DOI 10.1016/j.stem.2010.02.018

Li J Y, 1982, Natl Cancer Inst Monogr, V62, P113

Lister R, 2009, NATURE, V462, P315, DOI 10.1038/nature08514

Liu SL, 2006, CANCER RES, V66, P6063, DOI 10.1158/0008-5472.CAN-06-0054

Lo PK, 2012, ONCOGENE, V31, P2614, DOI [10.1038/onc.2011.439, 10.1038/onc.2012.147]

Ma S, 2008, ONCOGENE, V27, P1749, DOI 10.1038/sj.onc.1210811

Ma S, 2007, GASTROENTEROLOGY, V132, P2542, DOI 10.1053/j.gastro.2007.04.025

Mani SA, 2008, CELL, V133, P704, DOI 10.1016/j.cell.2008.03.027

Okumura T, 2006, CLIN CANCER RES, V12, P5096, DOI 10.1158/1078-0432.CCR-05-2852

Pardal R, 2003, NAT REV CANCER, V3, P895, DOI 10.1038/nrc1232

Pinto CA, 2013, CANCER LETT, V341, P56, DOI 10.1016/j.canlet.2013.06.003

Qian CN, 2006, CANCER RES, V66, P10365, DOI 10.1158/0008-5472.CAN-06-2977

Ramsahoye B., 2000, P NATL ACAD SCI USA, V97, P6

Reya T, 2001, NATURE, V414, P105, DOI 10.1038/35102167

Schatton T, 2008, NATURE, V451, P345, DOI 10.1038/nature06489

Tang KH, 2013, CANCER RES, V73, P2322, DOI 10.1158/0008-5472.CAN-12-2991

Tong CM, 2011, J GASTROEN HEPATOL, V26, P1229, DOI 10.1111/j.1440-1746.2011.06762.x

Tong M, 2012, CANCER RES, V72, P6024, DOI 10.1158/0008-5472.CAN-12-1269

Veevers-Lowe J, 2011, J CELL SCI, V124, P1288, DOI 10.1242/jcs.076935

Yoshioka T, 2013, J CLIN INVEST, V123, P682, DOI 10.1172/JCI60720

Zhao JS, 2011, PLOS ONE, V6, DOI 10.1371/journal.pone.0021419

NR 32

TC 48

Z9 50

U1 0

U2 10

PU NATURE PUBLISHING GROUP

PI LONDON

PA MACMILLAN BUILDING, 4 CRINAN ST, LONDON N1 9XW, ENGLAND

SN 2041-1723

J9 NAT COMMUN

JI Nat. Commun.

PD DEC 7

PY 2016

VL 7

AR 13568

DI 10.1038/ncomms13568

PG 14

WC Multidisciplinary Sciences

WE Science Citation Index Expanded (SCI-EXPANDED)

SC Science & Technology - Other Topics

GA EE5OG

UT WOS:000389656900001

PM 27924820

OA gold, Green Published

DA 2022-05-19

ER

PT J

AU Xu, B

Chen, H

Xu, ZP

Yao, XJ

Sun, XC

Cheng, HY

AF Xu, Bing

Chen, Hui

Xu, Zhipeng

Yao, Xijuan

Sun, Xinchen

Cheng, Hongyan

TI CDCA2 promotes tumorigenesis and induces radioresistance in oesophageal

squamous cell carcinoma cells

SO MOLECULAR MEDICINE REPORTS

LA English

DT Article

DE tumorigenesis; radiosensitivity; The Cancer Genome Atlas; DNA repair;

gene set enrichment analysis; cell cycle phase

ID CANCER-CELLS; DNA-REPAIR; CYCLE; SENSITIVITY; EXPRESSION; BIOMARKERS;

CHROMATIN; GENES

AB Cell division cycle-associated 2 (CDCA2) overexpression has been demonstrated to serve a significant role in tumorigenesis in certain types of cancer. Nevertheless, its role in tumour proliferation and radioresistance in oesophageal squamous cell carcinoma (ESCC) remains to be elucidated. Thus, the present study aimed to elucidate these roles. Data were downloaded from The Cancer Genome Atlas (TCGA) to compare the gene expression profiles. The expression of CDCA2 was higher in ESCC tissues compared with normal tissues. Gene set enrichment analysis was performed based on the ESCC cohorts in TCGA database. This demonstrated that higher expression of CDCA2 was significantly associated with the expression of related components of the cell cycle phase transition and G(2)/M phase transition pathways. Collectively, the results revealed that CDCA2 could serve as an underlying target to regulate tumour growth and radioresistance among patients with ESCC.

C1 [Xu, Bing; Chen, Hui; Yao, Xijuan; Sun, Xinchen] Nanjing Med Univ, Affiliated Hosp 1, Dept Radiat Oncol, 300 Guangzhou Rd, Nanjing 210029, Jiangsu, Peoples R China.

[Xu, Zhipeng] Southeast Univ, Affiliated Zhongda Hosp, Dept Urol, Nanjing 210009, Jiangsu, Peoples R China.

[Cheng, Hongyan] Nanjing Med Univ, Affiliated Hosp 1, Dept Synthet Internal Med, 300 Guangzhou Rd, Nanjing 210029, Jiangsu, Peoples R China.

RP Sun, XC (corresponding author), Nanjing Med Univ, Affiliated Hosp 1, Dept Radiat Oncol, 300 Guangzhou Rd, Nanjing 210029, Jiangsu, Peoples R China.; Cheng, HY (corresponding author), Nanjing Med Univ, Affiliated Hosp 1, Dept Synthet Internal Med, 300 Guangzhou Rd, Nanjing 210029, Jiangsu, Peoples R China.

EM sunxinchen@njmu.edu.cn; chenghongyan68@126.com

OI Yao, Xijuan/0000-0003-1732-096X

FU National Natural Science Foundation of China [81672983 (BA16)]

FX The present study was supported by the National Natural Science

Foundation of China [grant no. 81672983 (BA16)].

CR Bouzinab K, 2020, ACS APPL MATER INTER, V12, P12609, DOI 10.1021/acsami.0c01514

Bray F, 2018, CA-CANCER J CLIN, V68, P394, DOI 10.3322/caac.21492

Buckley AM, 2020, NAT REV GASTRO HEPAT, V17, P298, DOI 10.1038/s41575-019-0247-2

Chang L, 2014, CELL DEATH DIS, V5, DOI 10.1038/cddis.2014.415

Dai T, 2015, BEST PRACT RES CL GA, V29, P193, DOI 10.1016/j.bpg.2014.11.006

Dillon MT, 2014, CLIN ONCOL-UK, V26, P257, DOI 10.1016/j.clon.2014.01.009

Feng YF, 2019, BMC CANCER, V19, DOI 10.1186/s12885-019-5793-z

Ferlay J, 2019, INT J CANCER, V144, P1941, DOI 10.1002/ijc.31937

He GF, 2018, CANCER SCI, V109, P3774, DOI 10.1111/cas.13810

Krasnoselsky AL, 2005, ONCOGENE, V24, P1533, DOI 10.1038/sj.onc.1208341

Le Bras GF, 2016, PHARMACOL RES, V113, P236, DOI 10.1016/j.phrs.2016.08.021

Liang H, 2017, CANCER BIOL MED, V14, P33, DOI 10.20892/j.issn.2095-3941.2016.0093

Liu C, 2020, J CELL MOL MED, V24, P2444, DOI 10.1111/jcmm.14929

Livak KJ, 2001, METHODS, V25, P402, DOI 10.1006/meth.2001.1262

Lynam-Lennon N, 2010, RADIAT RES, V174, P703, DOI 10.1667/RR2295.1

MAITY A, 1994, RADIOTHER ONCOL, V31, P1, DOI 10.1016/0167-8140(94)90408-1

Ohashi S, 2015, GASTROENTEROLOGY, V149, P1700, DOI 10.1053/j.gastro.2015.08.054

Pawlik TM, 2004, INT J RADIAT ONCOL, V59, P928, DOI 10.1016/j.ijrobp.2004.03.005

Peng AM, 2010, CURR BIOL, V20, P387, DOI 10.1016/j.cub.2010.01.020

Petersen EV, 2020, FRONT ONCOL, V10, DOI 10.3389/fonc.2020.575569

Prevost M, 2013, MOL CELL PROTEOMICS, V12, P1468, DOI 10.1074/mcp.M112.023291

Sadoughi F, 2021, DNA REPAIR, V101, DOI 10.1016/j.dnarep.2021.103074

Sak A, 2010, SEMIN RADIAT ONCOL, V20, P223, DOI 10.1016/j.semradonc.2010.05.004

Shi R, 2017, ONCOTARGET, V8, P19768, DOI 10.18632/oncotarget.15519

Siegel RL, 2019, CA-CANCER J CLIN, V69, P7, DOI 10.3322/caac.21551

SINCLAIR WK, 1966, RADIAT RES, V29, P450, DOI 10.2307/3572025

TERASIMA T, 1963, BIOPHYS J, V3, P11, DOI 10.1016/S0006-3495(63)86801-0

Uchida F, 2013, PLOS ONE, V8, DOI 10.1371/journal.pone.0056381

Vagnarelli P, 2014, ADV EXP MED BIOL, V773, P401, DOI 10.1007/978-1-4899-8032-8_18

van Rossum PSN, 2018, NAT REV GASTRO HEPAT, V15, P235, DOI 10.1038/nrgastro.2017.162

Xiao B, 2018, BMC CANCER, V18, DOI 10.1186/s12885-018-4314-9

Zhao J, 2012, ASIAN PAC J CANCER P, V13, P4613, DOI 10.7314/APJCP.2012.13.9.4613

Zhao YY, 2018, CELL DEATH DIS, V9, DOI 10.1038/s41419-018-0466-4

Zheng N, 2021, PHARMACOL RES, V166, DOI 10.1016/j.phrs.2021.105507

NR 34

TC 0

Z9 0

U1 2

U2 2

PU SPANDIDOS PUBL LTD

PI ATHENS

PA POB 18179, ATHENS, 116 10, GREECE

SN 1791-2997

EI 1791-3004

J9 MOL MED REP

JI Mol. Med. Rep.

PD JUL

PY 2021

VL 24

IS 1

AR 530

DI 10.3892/mmr.2021.12169

PG 10

WC Oncology; Medicine, Research & Experimental

WE Science Citation Index Expanded (SCI-EXPANDED)

SC Oncology; Research & Experimental Medicine

GA SM2OB

UT WOS:000657448200001

PM 34036376

OA hybrid, Green Published

DA 2022-05-19

ER

PT J

AU Du, ZP

Wu, BL

Xie, YM

Zhang, YL

Liao, LD

Zhou, F

Xie, JJ

Zeng, FM

Xu, XE

Fang, WK

Li, EM

Xu, LY

AF Du, Ze-Peng

Wu, Bing-Li

Xie, Yang-Min

Zhang, Ying-Li

Liao, Lian-Di

Zhou, Fei

Xie, Jian-Jun

Zeng, Fa-Min

Xu, Xiu-E

Fang, Wang-Kai

Li, En-Min

Xu, Li-Yan

TI Lipocalin 2 promotes the migration and invasion of esophageal squamous

cell carcinoma cells through a novel positive feedback loop

SO BIOCHIMICA ET BIOPHYSICA ACTA-MOLECULAR CELL RESEARCH

LA English

DT Article

DE Esophageal squamous cell carcinoma; Lipocalin 2; Positive feedback;

Migration and invasion of cancer cells

ID GELATINASE-ASSOCIATED LIPOCALIN; NEUTROPHIL-GELATINASE; GROWTH-FACTOR;

CANCER; NGAL; RECEPTOR; PROTEIN; PATHWAY; EZRIN/RADIXIN/MOESIN;

DEPHOSPHORYLATION

AB Lipocalin 2 (LCN2) is a poor prognostic factor in esophageal squamous cell carcinoma (ESCC), however its functional roles and molecular mechanisms of action remain to be clarified. Here, we described the functions and signaling pathways for LCN2 in ESCC. Overexpression of LCN2 in ESCC cells accelerated cell migration and invasion in vitro, and promoted lung metastasis in vivo. Blocking LCN2 expression inhibited its pro-oncogenic effect. Either overexpression of LCN2 or treatment with recombinant human LCN2 protein enhanced the activation of MEK/ERIC pathway, which in turn increases endogenous LCN2 to increase MMP-9 activity. The decreased p-cofilin and increased p-ERM induced by pERK1/2 cause the cytoskeleton F-actin rearrangement and alter the behavior of ESCC cells mediated by LCN2. As a consequence, activation of MMP-9 and the rearrangement of F-actin throw light on the mechanisms for LCN2 in ESCC. These results imply that LCN2 promotes the migration and invasion of ESCC cells through a novel positive feedback loop. (C) 2015 Elsevier B.V. All rights reserved.

C1 [Du, Ze-Peng; Wu, Bing-Li; Xie, Yang-Min; Zhang, Ying-Li; Liao, Lian-Di; Zhou, Fei; Xie, Jian-Jun; Zeng, Fa-Min; Xu, Xiu-E; Fang, Wang-Kai; Li, En-Min; Xu, Li-Yan] Shantou Univ, Coll Med, Key Lab Mol Biol High Canc Incidence Coastal Chao, Shantou 515041, Peoples R China.

[Du, Ze-Peng; Liao, Lian-Di; Xu, Xiu-E; Xu, Li-Yan] Shantou Univ, Coll Med, Inst Oncol Pathol, Shantou 515041, Peoples R China.

[Wu, Bing-Li; Zhang, Ying-Li; Xie, Jian-Jun; Zeng, Fa-Min; Fang, Wang-Kai; Li, En-Min] Shantou Univ, Coll Med, Dept Biochem & Mol Biol, Shantou 515041, Peoples R China.

[Xie, Yang-Min; Zhou, Fei] Shantou Univ, Coll Med, Dept Expt Anim Ctr, Shantou 515041, Peoples R China.

[Du, Ze-Peng] Sun Yat Sen Univ, Affiliated Shantou Hosp, Shantou Cent Hosp, Dept Pathol, Shantou 515041, Guangdong, Peoples R China.

RP Li, EM (corresponding author), Shantou Univ, Coll Med, Key Lab Mol Biol High Canc Incidence Coastal Chao, Shantou 515041, Peoples R China.

EM nmli@stu.edu.cn; lyxu@stu.edu.cn

RI Fang, Wangkai/AAU-8664-2020

OI Fang, Wang-Kai/0000-0002-9417-2422

FU National Natural Science Foundation of China [81172264, 81472613];

National High Technology Research and Development Program of China

[2012AA02A503, 2012AA02A209]; Natural Science Foundation of

China-Guangdong Joint Fund [U1301227]; Science and Technology Program of

Guangdong [2011B060300025]

FX This work was supported by grants from the National Natural Science

Foundation of China (No.81172264 and No.81472613), the National High

Technology Research and Development Program of China (No.2012AA02A503

and No.2012AA02A209), and the Natural Science Foundation of

China-Guangdong Joint Fund (No.U1301227), the Science and Technology

Program of Guangdong (2011B060300025). The authors have declared that no

competing interests exist.

CR Darmellah A, 2012, J BIOL CHEM, V287, P34583, DOI 10.1074/jbc.M112.400010

Deng YZ, 2007, J BIOL CHEM, V282, P36571, DOI 10.1074/jbc.M704141200

Di Carlo A, 2013, ONCOL LETT, V5, P1677, DOI 10.3892/ol.2013.1252

Du ZP, 2011, BIOCHEM CELL BIOL, V89, P314, DOI 10.1139/O11-002

Du ZP, 2011, J CLIN PATHOL, V64, P69, DOI 10.1136/jcp.2010.083907

Fang WK, 2007, BIOCHEM J, V403, P297, DOI 10.1042/BJ20060836

Flower DR, 1996, BIOCHEM J, V318, P1

Flower DR, 2000, BBA-PROTEIN STRUCT M, V1482, P46, DOI 10.1016/S0167-4838(00)00147-3

Hanai J, 2005, J BIOL CHEM, V280, P13641, DOI 10.1074/jbc.M413047200

Hu LM, 2009, LAB INVEST, V89, P531, DOI 10.1038/labinvest.2009.17

Hvidberg V, 2005, FEBS LETT, V579, P773, DOI 10.1016/j.febslet.2004.12.031

KJELDSEN L, 1993, J BIOL CHEM, V268, P10425

Klein RM, 2008, MOL BIOL CELL, V19, P498, DOI 10.1091/mbc.E07-09-0895

Lannetti A, 2008, P NATL ACAD SCI USA, V105, P14058, DOI 10.1073/pnas.0710846105

Lee EK, 2011, INT J ONCOL, V38, P325, DOI 10.3892/ijo.2010.854

Leng XH, 2009, CANCER RES, V69, P8579, DOI 10.1158/0008-5472.CAN-09-1934

Li YY, 2014, BIOCHEM BIOPH RES CO, V452, P490, DOI 10.1016/j.bbrc.2014.08.110

Lim R, 2007, INT J CANCER, V120, P2426, DOI 10.1002/ijc.22352

Liu MF, 2011, J NEURO-ONCOL, V104, P119, DOI 10.1007/s11060-010-0486-0

Marti J, 2013, WORLD J SURG, V37, P1103, DOI 10.1007/s00268-013-1930-z

Mizuno K, 2013, CELL SIGNAL, V25, P457, DOI 10.1016/j.cellsig.2012.11.001

Mohammed MA, 2013, BMC UROL, V13, DOI 10.1186/1471-2490-13-25

Paragas N, 2012, BBA-MOL CELL RES, V1823, P1451, DOI 10.1016/j.bbamcr.2012.06.014

Parisiadou L, 2009, J NEUROSCI, V29, P13971, DOI 10.1523/JNEUROSCI.3799-09.2009

Pawlak G, 2002, MOL BIOL CELL, V13, P336, DOI 10.1091/mbc.01-06-0302

Pullikuth AK, 2007, CELL SIGNAL, V19, P1621, DOI 10.1016/j.cellsig.2007.04.012

Shen Zhongying, 2002, Zhonghua Zhongliu Zazhi, V24, P107

Tong ZM, 2008, CANCER RES, V68, P6100, DOI 10.1158/0008-5472.CAN-08-0540

Wabnitz GH, 2006, J IMMUNOL, V176, P1668, DOI 10.4049/jimmunol.176.3.1668

Wenners AS, 2012, PLOS ONE, V7, DOI 10.1371/journal.pone.0045826

Xie JJ, 2010, AM J PATHOL, V176, P939, DOI 10.2353/ajpath.2010.090118

Xu LY, 2001, PROG BIOCHEM BIOPHYS, V28, P839

Yan L, 2001, J BIOL CHEM, V276, P37258, DOI 10.1074/jbc.M106089200

Yang J, 2002, MOL CELL, V10, P1045, DOI 10.1016/S1097-2765(02)00710-4

Yang J, 2009, P NATL ACAD SCI USA, V106, P3913, DOI 10.1073/pnas.0810617106

Zhang HH, 2007, J CLIN PATHOL, V60, P555, DOI 10.1136/jcp.2006.039297

NR 36

TC 20

Z9 21

U1 1

U2 5

PU ELSEVIER SCIENCE BV

PI AMSTERDAM

PA PO BOX 211, 1000 AE AMSTERDAM, NETHERLANDS

SN 0167-4889

EI 0006-3002

J9 BBA-MOL CELL RES

JI Biochim. Biophys. Acta-Mol. Cell Res.

PD OCT

PY 2015

VL 1853

IS 10

BP 2240

EP 2250

DI 10.1016/j.bbamcr.2015.07.007

PN A

PG 11

WC Biochemistry & Molecular Biology; Cell Biology

WE Science Citation Index Expanded (SCI-EXPANDED)

SC Biochemistry & Molecular Biology; Cell Biology

GA CS0TM

UT WOS:000361775100006

PM 26190820

OA Bronze

DA 2022-05-19

ER

PT J

AU Xue, WH

Fan, ZR

Li, LF

Lu, JL

Ma, BJ

Kan, QC

Zhao, J

AF Xue, Wen-Hua

Fan, Zhi-Rui

Li, Li-Feng

Lu, Jing-Li

Ma, Bing-Jun

Kan, Quan-Cheng

Zhao, Jie

TI Construction of an oesophageal cancer-specific ceRNA network based on

miRNA, lncRNA, and mRNA expression data

SO WORLD JOURNAL OF GASTROENTEROLOGY

LA English

DT Article

DE Competing endogenous RNA; MicroRNA; Long non-coding RNA; mRNA;

Oesophageal squamous cell carcinoma

ID SQUAMOUS-CELL CARCINOMA; GENE FUSION EVENTS; KYOTO ENCYCLOPEDIA; PATHWAY

ANALYSIS; STRING DATABASE; GENOMES; TARGET; KEGG; IDENTIFICATION;

PROGRESSION

AB AIM

To explore the expression profiles of microRNAs (miRNAs), long non-coding RNAs (lncRNAs), and mRNAs in oesophageal squamous cell carcinoma (ESCC) in order to construct an oesophageal cancer-specific competing endogenous RNA (ceRNA) network.

METHODS

In this work, the expression data of miRNAs, lncRNAs, and mRNAs in ESCC were obtained. An oesophageal cancer-specific ceRNA network was then constructed and investigated.

RESULTS

CeRNAs have the ability to reduce the targeting activity of miRNAs, leading to the de-repression of specific mRNAs with common miRNA response elements. CeRNA interactions have a critical effect in gene regulation and cancer development.

CONCLUSION

This study suggests a novel perspective on potential oesophageal cancer mechanisms as well as novel pathways for modulating ceRNA networks for treating cancers.

C1 [Xue, Wen-Hua; Lu, Jing-Li; Ma, Bing-Jun; Kan, Quan-Cheng; Zhao, Jie] Zhengzhou Univ, Affiliated Hosp 1, Dept Pharm, Zhengzhou 450052, Henan, Peoples R China.

[Fan, Zhi-Rui; Li, Li-Feng] Zhengzhou Univ, Affiliated Hosp 1, Canc Ctr, Zhengzhou 450052, Henan, Peoples R China.

RP Zhao, J (corresponding author), Zhengzhou Univ, Affiliated Hosp 1, Dept Pharm, Engn Lab Digital Telemed Serv, 1 Jianshe Rd, Zhengzhou 450052, Henan, Peoples R China.

EM jiezhaoz2016@163.com

OI Lu, Jingli/0000-0001-7411-1430

FU National Natural Science Foundation of China [31670895, 71673254]

FX Supported by the National Natural Science Foundation of China, No.

31670895 and No. 71673254.

CR Abbaszadegan MR, 2005, J GASTROINTEST CANC, V36, P47, DOI 10.1385/IJGC:36:1:047

Altermann E, 2005, BMC GENOMICS, V6, DOI 10.1186/1471-2164-6-60

Arun G, 2016, GENE DEV, V30, P34, DOI 10.1101/gad.270959.115

Assenov Y, 2008, BIOINFORMATICS, V24, P282, DOI 10.1093/bioinformatics/btm554

Chen J, 2015, DIS ESOPHAGUS, V28, P84, DOI 10.1111/dote.12088

Chiu HS, 2015, GENOME RES, V25, P257, DOI 10.1101/gr.178194.114

Coleman HG, 2013, NUTR REV, V71, P474, DOI 10.1111/nure.12032

Conte F, 2017, PLOS ONE, V12, DOI 10.1371/journal.pone.0171661

Denzler R, 2014, MOL CELL, V54, P766, DOI 10.1016/j.molcel.2014.03.045

Du JL, 2014, MOL BIOSYST, V10, P2441, DOI 10.1039/c4mb00287c

Enright AJ, 1999, NATURE, V402, P86, DOI 10.1038/47056

Gaidatzis D, 2007, BMC BIOINFORMATICS, V8, DOI 10.1186/1471-2105-8-69

Grahne G, 2003, INFORM SYST, V28, P311, DOI 10.1016/S0306-4379(02)00075-3

Heberle H, 2015, BMC BIOINFORMATICS, V16, DOI 10.1186/s12859-015-0611-3

Hu N, 2015, BMC GENOMICS, V16, DOI 10.1186/s12864-015-1919-0

Huang DW, 2009, NAT PROTOC, V4, P44, DOI 10.1038/nprot.2008.211

Inoue K, 1997, CANCER, V79, P206, DOI 10.1002/(SICI)1097-0142(19970115)79:2<206::AID-CNCR2>3.0.CO;2-I

Jang HJ, 2017, GUT, V66, P215, DOI 10.1136/gutjnl-2015-311238

Jin JG, 2017, TUMOR BIOL, V39, DOI 10.1177/1010428317716250

Kanehisa M, 2000, NUCLEIC ACIDS RES, V28, P27, DOI 10.1093/nar/28.1.27

Kano M, 2010, INT J CANCER, V127, P2804, DOI 10.1002/ijc.25284

Karreth FA, 2011, CELL, V147, P382, DOI 10.1016/j.cell.2011.09.032

Li XM, 2016, ONCOTARGET, V7, P23197, DOI 10.18632/oncotarget.8099

Liang CW, 2010, PLOS ONE, V5, DOI 10.1371/journal.pone.0010698

Lin DC, 2009, DIS ESOPHAGUS, V22, P9, DOI 10.1111/j.1442-2050.2008.00845.x

Liu Q, 2017, ONCOTARGET, V8, P43768, DOI 10.18632/oncotarget.10844

Lotia S, 2013, BIOINFORMATICS, V29, P1350, DOI 10.1093/bioinformatics/btt138

Luan T, 2017, ONCOTARGET, V8, P76153, DOI 10.18632/oncotarget.19190

Makiuchi T, 2007, GENE, V394, P78, DOI 10.1016/j.gene.2007.02.009

Martin B, 2012, PLOS ONE, V7, DOI 10.1371/journal.pone.0036911

Martirosyan A, 2017, SCI REP-UK, V7, DOI 10.1038/srep43673

Mercier G, 2004, NUCLEIC ACIDS RES, V32, DOI 10.1093/nar/gnh002

Mutowo P, 2016, J BIOMED SEMANT, V7, DOI 10.1186/s13326-016-0102-0

Noorbakhsh J, 2013, PLOS ONE, V8, DOI 10.1371/journal.pone.0072676

Song YM, 2014, NATURE, V509, P91, DOI 10.1038/nature13176

Starkov AA, 2004, J NEUROSCI, V24, P7779, DOI 10.1523/JNEUROSCI.1899-04.2004

Stratton MR, 2009, NATURE, V458, P719, DOI 10.1038/nature07943

Suhre K, 2004, NUCLEIC ACIDS RES, V32, pD273, DOI 10.1093/nar/gkh053

Szklarczyk D, 2011, NUCLEIC ACIDS RES, V39, pD561, DOI 10.1093/nar/gkq973

Ventura A, 2008, CELL, V132, P875, DOI 10.1016/j.cell.2008.02.019

Wang Q, 2013, BMC CANCER, V13, DOI 10.1186/1471-2407-13-137

Weinstein JN, 2013, NAT GENET, V45, P1113, DOI 10.1038/ng.2764

Wen J, 2014, ANN ONCOL, V25, P1769, DOI 10.1093/annonc/mdu201

Xu J, 2015, NUCLEIC ACIDS RES, V43, P8169, DOI 10.1093/nar/gkv853

Zhang W, 2014, ZHONGGUO YIYAO DAOBA, V11, P4

Zhang YP, 2016, ONCOTARGET, V7, P64148, DOI 10.18632/oncotarget.11637

Zhou M, 2016, ONCOTARGET, V7, P12598, DOI 10.18632/oncotarget.7181

Zhu SM, 2007, J BIOL CHEM, V282, P14328, DOI 10.1074/jbc.M611393200

NR 48

TC 22

Z9 22

U1 1

U2 10

PU BAISHIDENG PUBLISHING GROUP INC

PI PLEASANTON

PA 8226 REGENCY DR, PLEASANTON, CA 94588 USA

SN 1007-9327

EI 2219-2840

J9 WORLD J GASTROENTERO

JI World J. Gastroenterol.

PD JAN 7

PY 2018

VL 24

IS 1

BP 23

EP 34

DI 10.3748/wjg.v24.i1.23

PG 12

WC Gastroenterology & Hepatology

WE Science Citation Index Expanded (SCI-EXPANDED)

SC Gastroenterology & Hepatology

GA FS7BS

UT WOS:000419953400004

PM 29358879

OA Green Published, Green Submitted, hybrid

DA 2022-05-19

ER

PT J

AU Sun, BY

Wei, QQ

Liu, CX

Zhang, L

Luo, G

Li, T

Lu, MH

AF Sun, B-Y

Wei, Q-Q

Liu, C-X

Zhang, L.

Luo, G.

Li, T.

Lu, M-H

TI ECT2 promotes proliferation and metastasis of esophageal squamous cell

carcinoma via the RhoA- ERK signaling pathway

SO EUROPEAN REVIEW FOR MEDICAL AND PHARMACOLOGICAL SCIENCES

LA English

DT Article

DE Esophageal squamous cell carcinoma (ESCC); Epithelial cell

transformation sequence 2 (ECT2); RhoA-ERK signaling pathway;

Proliferation; Migration

ID EXCHANGE FACTOR; PROTEIN; EXPRESSION; GTPASES; P53

AB OBJECTIVE: In this study, the effect of epithelial cell transformation sequence 2 (ECT2) on the proliferation, invasion and migration of esophageal squamous cell carcinoma (ESCC) was investigated by interfering the expression of ECT2.

PATIENTS AND METHODS: Interfering with the expression level of ECT2 in human squamous cell carcinomas KYSE140 and EC9706 cell lines, the changes of KYSE140 and EC9706 cell proliferation, invasion, and migration were measured using the CCK-8 method, transwell test, and scratch test, respectively. The effects of ECT2 on the Ras homolog gene family, member A-extracellular regulated protein kinases (RhoA-ERK) signaling pathway were also observed.

RESULTS: Compared with the control group, the proliferation, migration, and invasion ability of EC9706 and KYSE140 cells after ECT2 knockout were significantly reduced (p <0.05). The knockdown of ECT2 expression in ESCC cell lines suppressed the activation of RhoA-ERK signaling pathway and protein expression of VEGF and MMP9.

CONCLUSIONS: ECT2 could regulated the expression of VEGF and MMP9 to inhibit cells proliferation, invasion, migration and tumor development through RhoA-ERK signaling pathway. Therefore, ECT2 could be an available marker, and provide a new theoretical basis for the treatment of ESCC.

C1 [Sun, B-Y; Wei, Q-Q; Liu, C-X; Zhang, L.; Luo, G.; Lu, M-H] Southwest Med Univ, Affiliated Hosp, Dept Gastroenterol, Luzhou City, Sichuan, Peoples R China.

[Li, T.] Sichuan Univ, West China Hosp, Dept Anesthesiol, Chengdu, Sichuan, Peoples R China.

[Lu, M-H] Southwest Med Univ, Affiliated Hosp, Nucl Med & Mol Imaging Key Lab Sichuan Prov, Luzhou City, Sichuan, Peoples R China.

RP Lu, MH (corresponding author), Southwest Med Univ, Affiliated Hosp, Dept Gastroenterol, Luzhou City, Sichuan, Peoples R China.; Lu, MH (corresponding author), Southwest Med Univ, Affiliated Hosp, Nucl Med & Mol Imaging Key Lab Sichuan Prov, Luzhou City, Sichuan, Peoples R China.

EM lvmuhan@swmu.edu.cn

FU Sichuan Provincial Science and Technology Department [2016JY0101,

2017JQ0052]; Luzhou City-Luzhou Medical College Funding

[2015LZCYD-S01(9/15), 2016LZX-NYD-T06]; Southwest Medical University

Funding [2017-R-63]; Affiliated Hospital of Southwest Medical University

[15039]

FX This research was supported by the Sichuan Provincial Science and

Technology Department (No. 2016JY0101 and No. 2017JQ0052), Luzhou

City-Luzhou Medical College Funding (No. 2015LZCYD-S01(9/15) and No.

2016LZX-NYD-T06), Southwest Medical University Funding (No. 2017-R-63).

The Affiliated Hospital of Southwest Medical University (15039).

CR Chen JX, 2015, J HEPATOL, V62, P1287, DOI 10.1016/j.jhep.2015.01.014

Chen WQ, 2016, CA-CANCER J CLIN, V66, P115, DOI 10.3322/caac.21338

Chen XR, 2018, J EXP CLIN CANC RES, V37, DOI 10.1186/s13046-018-0787-2

Chen Y, 2017, MED SCI MONITOR, V23, P3154, DOI 10.12659/MSM.905388

Chen ZG, 2017, MED SCI MONITOR, V23, DOI 10.12659/MSM.905951

Cook DR, 2014, ONCOGENE, V33, P4021, DOI 10.1038/onc.2013.362

Eguchi T, 2007, ONCOGENE, V26, P509, DOI 10.1038/sj.onc.1209810

Jin Y, 2014, INT J CLIN EXP PATHO, V7, P8724

Justilien V, 2009, ONCOGENE, V28, P3597, DOI 10.1038/onc.2009.217

Li M, 2014, CELL CYCLE, V13, P2944, DOI 10.4161/15384101.2014.947197

Li YM, 2017, EXP THER MED, V14, P4862, DOI 10.3892/etm.2017.5132

MIKI T, 1993, NATURE, V362, P462, DOI 10.1038/362462a0

Pfaffl MW, 2001, NUCLEIC ACIDS RES, V29, DOI 10.1093/nar/29.9.e45

Saito S, 2004, J BIOL CHEM, V279, P7169, DOI 10.1074/jbc.M306725200

Scoumanne A, 2006, CANCER RES, V66, P6271, DOI 10.1158/0008-5472.CAN-06-0121

Short MW, 2017, AM FAM PHYSICIAN, V95, P22

Solski PA, 2004, J BIOL CHEM, V279, P25226, DOI 10.1074/jbc.M313792200

Tatsumoto T, 1999, J CELL BIOL, V147, P921, DOI 10.1083/jcb.147.5.921

Tian L, 2017, FRONT IMMUNOL, V8, DOI 10.3389/fimmu.2017.01214

Vanni C, 2004, ONCOGENE, V23, P4098, DOI 10.1038/sj.onc.1207509

Wang HK, 2018, J CLIN PATHOL, V71, P442, DOI 10.1136/jclinpath-2017-204569

Wang Y, 2013, MOL CANCER RES, V11, P1624, DOI 10.1158/1541-7786.MCR-13-0371-T

Xu YB, 2013, EUR REV MED PHARMACO, V17, P2486

Yu YJ, 2019, J CELL BIOCHEM, V120, P967, DOI 10.1002/jcb.27459

Yue CL, 2017, ONCOTARGET, V8, P47167, DOI 10.18632/oncotarget.17006

Zhang L, 2016, EXPERT REV GASTROENT, V10, P595, DOI 10.1586/17474124.2016.1140036

Zhi TL, 2019, NEURO-ONCOLOGY, V21, P462, DOI 10.1093/neuonc/noy207

Zhou SJ, 2017, PLOS ONE, V12, DOI 10.1371/journal.pone.0187356

NR 28

TC 2

Z9 2

U1 0

U2 0

PU VERDUCI PUBLISHER

PI ROME

PA VIA GREGORIO VII, ROME, 186-00165, ITALY

SN 1128-3602

J9 EUR REV MED PHARMACO

JI Eur. Rev. Med. Pharmacol. Sci.

PY 2020

VL 24

IS 15

BP 7991

EP 8000

DI 10.26355/eurrev_202008_22482

PG 10

WC Pharmacology & Pharmacy

WE Science Citation Index Expanded (SCI-EXPANDED)

SC Pharmacology & Pharmacy

GA MX0BN

UT WOS:000557394400010

PM 32767325

DA 2022-05-19

ER

PT J

AU Zhang, YW

Yuan, X

Yue, N

Wang, LD

Liu, JQ

Dai, NT

Yang, HJ

Fan, RT

Zhou, FY

AF Zhang, Yaowen

Yuan, Xiang

Yue, Ning

Wang, Lidong

Liu, Junqi

Dai, Ningtao

Yang, Haijun

Fan, Ruitai

Zhou, Fuyou

TI hsa_circRNA6448-14 promotes carcinogenesis in esophageal squamous cell

carcinoma

SO AGING-US

LA English

DT Article

DE hsa_circRNA6448-14; esophageal squamous cell carcinoma; microarray;

proliferation; microRNA sponge

ID CIRCULAR RNA; PROLIFERATION; LANDSCAPE; INVASION; CANCER

AB Circular RNAs (circRNAs) play important roles in cancer progression. hsa_circRNA6448-14 originates from exon 5 to exon 11 of the TGFBI gene. We investigated the roles of hsa_circRNA6448-14 in esophageal squamous cell carcinoma (ESCC) with microarrays and quantitative real-time polymerase chain reaction (qRT-PCR), Kaplan Meier analysis, loss-of-function and gain-of-function assays, and pull-down assays for miRNA binding. The hsa_circRNA6448-14-miRNA-mRNA network was drawn using Circos. hsa_circRNA6448-14 was significantly up regulated in ESCC tissues and cell lines. As a diagnostic biomarker, hsa_circRNA6448-14 had an area under the curve (AUC), sensitivity, and specificity of 0.906, 82.9%, and 85.5%, respectively. hsa_circRNA6448-14 upregulation was correlated with poor differentiation, advanced pTNM stage, poor disease-free survival (DFS), and poor overall survival (OS). Elevated hsa_circRNA6448-14 promoted cell proliferation, migration, invasion, and inhibited apoptosis in vitro. hsa_circRNA6448-14 functioned as a miRNA sponge to competitively bind miR455-3p, and hsa_circRNA6448-14 expression negatively correlated with that of miR-455-3p. hsa_circRNA644814 promoted carcinogenesis in ESCC, suggesting that hsa_circRNA6448-14 could serve as a diagnostic and prognostic biomarker for ESCC.

C1 [Zhang, Yaowen; Dai, Ningtao; Yang, Haijun; Zhou, Fuyou] Anyang Canc Hosp, Dept Radiat Oncol, Anyang 455000, Peoples R China.

[Zhang, Yaowen; Wang, Lidong; Liu, Junqi; Fan, Ruitai] Zhengzhou Univ, Dept Radiat Oncol, Henan Key Lab Canc Res, Affiliated Hosp 1, Zhengzhou 450000, Peoples R China.

[Yuan, Xiang] Henan Univ Sci & Technol, Affiliated Hosp 1, Canc Inst, Henan Key Lab Canc Epigenet, Luoyang 471000, Peoples R China.

[Yuan, Xiang] Henan Univ Sci & Technol, Coll Clin Med, Luoyang 471000, Peoples R China.

[Yue, Ning] Rutgers Robert Wood Johnson Med Sch, Rutgers Canc Inst New Jersey, Dept Radiat Oncol, New Brunswick, NJ 08903 USA.

RP Zhou, FY (corresponding author), Anyang Canc Hosp, Dept Radiat Oncol, Anyang 455000, Peoples R China.; Fan, RT (corresponding author), Zhengzhou Univ, Dept Radiat Oncol, Henan Key Lab Canc Res, Affiliated Hosp 1, Zhengzhou 450000, Peoples R China.

EM fccfanrt@zzu.edu.cn; ayzhoufuyou@163.com

OI Liu, Junqi/0000-0002-6015-1033

FU National Natural Science Foundation of China [U1504814]; Major projects

of Science and Technology Department in Henan Province [16110311200,

161100311300]; Anyang Science Foundation of Henan Province

FX This study was supported by National Natural Science Foundation of China

(U1504814), Major projects of Science and Technology Department in Henan

Province (16110311200, 161100311300) and Anyang Science Foundation of

Henan Province (2016).

CR Bray F, 2018, CA-CANCER J CLIN, V68, P394, DOI 10.3322/caac.21492

Chen JX, 2019, MOL CANCER, V18, DOI 10.1186/s12943-019-1076-1

Cheng L, 2016, ONCOTARGET, V7, P61199, DOI 10.18632/oncotarget.11284

Cheng ZA, 2019, NAT COMMUN, V10, DOI 10.1038/s41467-019-11162-4

COCQUERELLE C, 1993, FASEB J, V7, P155, DOI 10.1096/fasebj.7.1.7678559

Dube U, 2019, NAT NEUROSCI, V22, P1903, DOI 10.1038/s41593-019-0501-5

Fan LY, 2019, MOL CANCER, V18, DOI 10.1186/s12943-018-0936-4

GU X, 2019, CELL, V176, DOI DOI 10.1016/J.CELL.2019.01.025

Guo JU, 2014, GENOME BIOL, V15, DOI 10.1186/s13059-014-0409-z

Han B, 2018, PHARMACOL THERAPEUT, V187, P31, DOI 10.1016/j.pharmthera.2018.01.010

Hanniford D, 2020, CANCER CELL, V37, P55, DOI 10.1016/j.ccell.2019.12.007

Hansen TB, 2013, NATURE, V495, P384, DOI 10.1038/nature11993

HSU MT, 1979, NATURE, V280, P339, DOI 10.1038/280339a0

Hu ZQ, 2020, HEPATOLOGY, V72, P906, DOI 10.1002/hep.31068

Huang SL, 2019, CIRCULATION, V139, P2857, DOI 10.1161/CIRCULATIONAHA.118.038361

Kristensen LS, 2019, NAT REV GENET, V20, P675, DOI 10.1038/s41576-019-0158-7

Lagergren J, 2017, LANCET, V390, P2383, DOI 10.1016/S0140-6736(17)31462-9

Lan YL, 2018, CELL DEATH DIS, V9, DOI 10.1038/s41419-018-0262-1

Li RC, 2018, CELL DEATH DIS, V9, DOI 10.1038/s41419-018-0852-y

Li ZY, 2015, NAT STRUCT MOL BIOL, V22, P256, DOI 10.1038/nsmb.2959

Liu CX, 2019, CELL, V177, P865, DOI 10.1016/j.cell.2019.03.046

Liu TY, 2020, CELL DEATH DIS, V11, DOI 10.1038/s41419-020-2226-5

Mehta SL, 2020, PROG NEUROBIOL, V186, DOI 10.1016/j.pneurobio.2020.101746

NIGRO JM, 1991, CELL, V64, P607, DOI 10.1016/0092-8674(91)90244-S

Otsubo T, 2017, ONCOTARGET, V8, P84434, DOI 10.18632/oncotarget.21375

Pan ZH, 2019, AGING-US, V11, P11937, DOI 10.18632/aging.102519

Qin L, 2016, ONCOL REP, V36, P3325, DOI 10.3892/or.2016.5139

Rice TW, 2017, J THORAC ONCOL, V12, P36, DOI 10.1016/j.jtho.2016.10.016

SANGER HL, 1976, P NATL ACAD SCI USA, V73, P3852, DOI 10.1073/pnas.73.11.3852

Siegel RL, 2019, CA-CANCER J CLIN, V69, P7, DOI 10.3322/caac.21551

Suo C, 2019, J THORAC ONCOL, V14, P712, DOI 10.1016/j.jtho.2018.12.023

Thrumurthy SG, 2019, BMJ-BRIT MED J, V366, DOI 10.1136/bmj.l4373

Verduci L, 2019, MOL ONCOL, V13, P669, DOI 10.1002/1878-0261.12468

Vo JN, 2019, CELL, V176, P869, DOI 10.1016/j.cell.2018.12.021

Wang CD, 2019, MOL CANCER, V18, DOI 10.1186/s12943-019-1061-8

Wang S, 2019, MOL CANCER, V18, DOI 10.1186/s12943-019-1081-4

Wu HY, 2019, CLIN EPIGENETICS, V11, DOI 10.1186/s13148-019-0610-8

Yang H, 2017, EUR REV MED PHARMACO, V21, P3200

Zeng Y, 2019, J BIOL CHEM, V294, P15808, DOI 10.1074/jbc.RA119.010800

Zhan T, 2018, MOL THER-NUCL ACIDS, V10, P215, DOI 10.1016/j.omtn.2017.12.002

Zhang XO, 2016, GENOME RES, V26, P1277, DOI 10.1101/gr.202895.115

Zhang YW, 2019, BIOCHEM BIOPH RES CO, V519, P497, DOI 10.1016/j.bbrc.2019.09.015

Zhao X, 2019, INT J MOL SCI, V20, DOI 10.3390/ijms20163926

NR 43

TC 4

Z9 4

U1 0

U2 4

PU IMPACT JOURNALS LLC

PI ORCHARD PARK

PA 6666 E QUAKER ST, STE 1, ORCHARD PARK, NY 14127 USA

SN 1945-4589

J9 AGING-US

JI Aging-US

PD AUG 15

PY 2020

VL 12

IS 15

BP 15581

EP 15602

PG 22

WC Cell Biology; Geriatrics & Gerontology

WE Science Citation Index Expanded (SCI-EXPANDED)

SC Cell Biology; Geriatrics & Gerontology

GA NM1ZH

UT WOS:000567901800009

PM 32805720

OA gold, Green Published

DA 2022-05-19

ER

PT J

AU Wang, TT

Wang, R

Liu, LK

AF Wang, Tingting

Wang, Rong

Liu, Lianke

TI Long-term survival of an unresectable upper thoracic esophageal squamous

cell carcinoma with severe dysphagia following nasogastric tube feeding

and camrelizumab-containing therapy: a case report

SO TRANSLATIONAL CANCER RESEARCH

LA English

DT Article

DE Camrelizumab; enteral nutrition; esophageal squamous cell carcinoma

(ESCC); case report

ID INCREASE

AB For upper thoracic esophageal cancer, surgery is difficult, and the clinical effects of chemoradiotherapy, radiotherapy, and chemotherapy are limited. Camrelizumab is a PD-1 (programmed cell death-1) antibody developed by China. There are few studies on camrelizumab in esophageal cancer. Here, a 66-year-old man was admitted to the hospital with severe dysphagia. He was diagnosed as upper thoracic esophageal squamous cell carcinoma (ESCC). The patient received nasogastric tube placement, chemotherapy with docetaxel and nedaplatin, monotherapy with camrelizumab, combination therapy with camrelizumab and docetaxel, maintenance therapy with docetaxel, successively. One year later, complete response was observed and the nasogastric tube was removed. The progression-free survival (PFS) exceeded 28 months. The patient is still being followed up. In this paper, temporary nasogastric tube feeding not only provided nutrients safely, but also won the time for immunotherapy to work. Camrelizumab-containing therapy achieved complete response with long-term survival in unresectable upper thoracic ESCC patients. Comprehensive therapy regimens involved with nasogastric tube feeding and camrelizumab-containing therapy are effective and safe in unresectable upper thoracic ESCC patients with severe dysphagia.

C1 [Wang, Tingting; Wang, Rong; Liu, Lianke] Nanjing Med Univ, Affiliated Hosp 1, Dept Oncol, 300 Guangzhou Rd, Nanjing 210029, Peoples R China.

RP Liu, LK (corresponding author), Nanjing Med Univ, Affiliated Hosp 1, Dept Oncol, 300 Guangzhou Rd, Nanjing 210029, Peoples R China.

EM liulianke@jsph.org.cn

CR Bray F, 2018, CA-CANCER J CLIN, V68, P394, DOI 10.3322/caac.21492

Fang WF, 2018, LANCET ONCOL, V19, P1338, DOI 10.1016/S1470-2045(18)30495-9

Finlay WJJ, 2019, MABS-AUSTIN, V11, P26, DOI 10.1080/19420862.2018.1550321

Gao F, 2020, CLIN RADIOL, V75, DOI 10.1016/j.crad.2019.10.008

Grooteman KV, 2017, GASTROINTEST ENDOSC, V85, P332, DOI 10.1016/j.gie.2016.07.062

Huang J, 2018, CLIN CANCER RES, V24, P1296, DOI 10.1158/1078-0432.CCR-17-2439

Lin YS, 2013, J EPIDEMIOL, V23, P233, DOI 10.2188/jea.JE20120162

Siddique MZ, 2019, J GASTROINTEST ONCOL, V10, P492, DOI 10.21037/jgo.2019.03.08

Tu LL, 2013, RADIAT ONCOL, V8, DOI 10.1186/1748-717X-8-75

Zhang JQ, 2019, CANCER MANAG RES, V11, P8285, DOI 10.2147/CMAR.S203575

NR 10

TC 1

Z9 1

U1 1

U2 3

PU AME PUBL CO

PI SHATIN

PA FLAT-RM C 16F, KINGS WING PLAZA 1, NO 3 KWAN ST, SHATIN, HONG KONG

00000, PEOPLES R CHINA

SN 2218-676X

EI 2219-6803

J9 TRANSL CANCER RES

JI Transl. Cancer Res.

PD APR

PY 2020

VL 9

IS 4

BP 3003

EP 3006

DI 10.21037/tcr.2020.03.13

PG 4

WC Oncology

WE Science Citation Index Expanded (SCI-EXPANDED)

SC Oncology

GA LK5NZ

UT WOS:000530914200086

PM 35117657

OA gold, Green Published

DA 2022-05-19

ER

PT J

AU Hu, X

Wang, M

Cao, L

Cong, L

Gao, YJ

Lu, JW

Feng, JF

Shen, B

Liu, DL

AF Hu, Xiao

Wang, Min

Cao, Lei

Cong, Li

Gao, Yujie

Lu, Jianwei

Feng, Jifeng

Shen, Bo

Liu, Delin

TI miR-4319 Suppresses the Growth of Esophageal Squamous Cell Carcinoma Via

Targeting NLRC5

SO CURRENT MOLECULAR PHARMACOLOGY

LA English

DT Article

DE miR-4319; NLRC5; esophageal squamous cell carcinoma; cell growth; cell

cycle; cell culture

AB Background: The functions of microRNAs (miRNAs) in cancer progression have been recognized in recent years. However, the role of miR-4319 in esophageal squamous cell carcinoma (ESCC) remains unclear. Objective: We aimed to investigate the biological roles of miR-4319 in ESCC progression and the associated mechanisms.

Methods: Real-time PCR was performed to examine the levels of miR-4319 in ESCC cell lines. The effects of miR-4319 and NOD-like receptor (NLR) family, caspase activation and recruitment domain (CARD) domain containing 5 (NLRC5) on cell proliferation and cell cycle progression were evaluated using MIT assay, colony formation and flow cytometry assays. Bioinformatics techniques and luciferase reporter assay were applied to validate NLRC5 as a miR-4319 target.

Results: The miR-4319 expression was lower in ESCC cells than in the normal cell line. The expression of miR-4319 repressed cell growth and induced cell cycle arrest. NLRC5 was validated as a direct downstream target of miR-4319. Overexpression of NLRC5 potentiated the effects of miR-4319 on cell growth and cell cycle distribution.

Conclusion: Our results demonstrated that miR-4319 might function as a tumor suppressor by targeting NLRC5 in ESCC.

C1 [Hu, Xiao; Cao, Lei; Cong, Li; Gao, Yujie] Suqian First Hosp, Dept Oncol, Suqian 223800, Peoples R China.

[Wang, Min] Nanjing Med Univ, Pain Clin, Jiangsu Canc Hosp, Nanjing 210009, Peoples R China.

[Wang, Min; Lu, Jianwei; Feng, Jifeng; Shen, Bo; Liu, Delin] Nanjing Med Univ, Jiangsu Inst Canc Res, Nanjing 210009, Peoples R China.

[Wang, Min; Lu, Jianwei; Feng, Jifeng; Shen, Bo; Liu, Delin] Nanjing Med Univ, Affiliated Canc Hosp, Nanjing 210009, Peoples R China.

[Lu, Jianwei; Feng, Jifeng; Shen, Bo; Liu, Delin] Nanjing Med Univ, Jiangsu Canc Hosp, Dept Oncol, Nanjing 210009, Peoples R China.

RP Liu, DL (corresponding author), Nanjing Med Univ, Jiangsu Inst Canc Res, Nanjing 210009, Peoples R China.; Liu, DL (corresponding author), Nanjing Med Univ, Affiliated Canc Hosp, Nanjing 210009, Peoples R China.; Liu, DL (corresponding author), Nanjing Med Univ, Jiangsu Canc Hosp, Dept Oncol, Nanjing 210009, Peoples R China.

EM liudelin_nj@126.com

CR Bartel DP, 2004, CELL, V116, P281, DOI 10.1016/S0092-8674(04)00045-5

Bian SQ, 2020, J CELL BIOCHEM, V121, P174, DOI 10.1002/jcb.29026

Bray F, 2018, CA-CANCER J CLIN, V68, P394, DOI 10.3322/caac.21492

Huang LC, 2019, UNITED EUR GASTROENT, V7, P517, DOI 10.1177/2050640619837440

Kaikkonen MU, 2018, TRENDS BIOCHEM SCI, V43, P654, DOI 10.1016/j.tibs.2018.06.002

Kim T, 2011, J SURG ONCOL, V103, P179, DOI 10.1002/jso.21784

Li YM, 2018, OPEN MED-WARSAW, V13, P443, DOI 10.1515/med-2018-0066

Lin X, 2018, CLIN TRANSL ONCOL, V20, P1400, DOI 10.1007/s12094-018-1871-y

Qiao GE, 2019, INT J MOL MED, V43, P1817, DOI 10.3892/ijmm.2019.4107

Rustgi AK, 2014, NEW ENGL J MED, V371, P2499, DOI 10.1056/NEJMra1314530

Tang DY, 2019, J CELL BIOCHEM, V120, P9522, DOI 10.1002/jcb.28228

Wang Q, 2019, CANCER LETT, V444, P9, DOI 10.1016/j.canlet.2018.11.024

Yang Y, 2019, BIOMED PHARMACOTHER, V115, DOI 10.1016/j.biopha.2019.108956

Yoshihama S, 2017, TRENDS CANCER, V3, P28, DOI 10.1016/j.trecan.2016.12.003

Zhao X, 2019, J CELL PHYSIOL, V234, P19886, DOI 10.1002/jcp.28587

Zheng ST, 2019, CELL BIOL INT, V43, P634, DOI 10.1002/cbin.11136

NR 16

TC 6

Z9 6

U1 0

U2 4

PU BENTHAM SCIENCE PUBL LTD

PI SHARJAH

PA EXECUTIVE STE Y-2, PO BOX 7917, SAIF ZONE, 1200 BR SHARJAH, U ARAB

EMIRATES

SN 1874-4672

EI 1874-4702

J9 CURR MOL PHARMACOL

JI Curr. Molec. Pharmacol.

PY 2020

VL 13

IS 2

BP 144

EP 149

DI 10.2174/1874467212666191119094636

PG 6

WC Biochemistry & Molecular Biology; Pharmacology & Pharmacy

WE Science Citation Index Expanded (SCI-EXPANDED)

SC Biochemistry & Molecular Biology; Pharmacology & Pharmacy

GA LJ2BU

UT WOS:000529976200006

PM 31746301

DA 2022-05-19

ER

PT J

AU Bai, G

Yang, M

Zheng, C

Zhang, L

Eli, M

AF Bai, Ge

Yang, Mei

Zheng, Chao

Zhang, Li

Eli, Mayinur

TI Suppressor PAQR3 associated with the clinical significance and prognosis

in esophageal squamous cell carcinoma

SO ONCOLOGY LETTERS

LA English

DT Article

DE suppressor; extracellular signal-regulated kinase; prognosis; esophageal

squamous cell carcinoma; progestin and adipoQ receptor family member 3

ID GASTRIC-CANCER CELLS; TUMOR-SUPPRESSOR; RKTG; TUMORIGENESIS;

DEGRADATION; METASTASIS; EXPRESSION; PLAYS; GENE; RAF

AB Progestin and adipoQ receptor family member 3 (PAQR3) is a novel tumor suppressor; however, its function in esophageal cancer is not well understood. The present study explored the association between PAQR3, and the survival and clinical phenotype in patients with esophageal squamous cell carcinoma (ESCC). The expression of PAQR3 in 80 cases of ESCC and its corresponding adjacent tissues was detected by reverse transcription-quantitative polymerase chain reaction. The results demonstrated that PAQR3 expression in cancer tissues was significantly lower compared with that in adjacent tissues. Clinicopathological analysis indicated that PAQR3 expression was significantly correlated with ethnicity (P=0.032), tumor length (P=0.019), lymph node metastasis (P=0.011) and local recurrence (P=0.009). Notably, the Kaplan-Meier survival curve demonstrated that a decrease in PAQR3 expression was associated with poor prognosis in patients with ESCC. Multivariate analysis indicated that PAQR3 expression was an independent prognostic indicator for patients with ESCC. PAQR3 may serve an important role in the progress of ESCC and become a potential candidate for ESCC targeted therapy.

C1 [Bai, Ge; Yang, Mei; Zheng, Chao; Eli, Mayinur] Xinjiang Med Univ, Affiliated Hosp 1, Ctr Canc, 137 Liyushan South Rd, Urumqi 830011, Xinjiang Uyghur, Peoples R China.

[Zhang, Li] Xinjiang Med Univ, Affiliated Hosp 1, VIP Med, 137 Liyushan South Rd, Urumqi 830011, Xinjiang Uyghur, Peoples R China.

RP Eli, M (corresponding author), Xinjiang Med Univ, Affiliated Hosp 1, Ctr Canc, 137 Liyushan South Rd, Urumqi 830011, Xinjiang Uyghur, Peoples R China.; Zhang, L (corresponding author), Xinjiang Med Univ, Affiliated Hosp 1, VIP Med, 137 Liyushan South Rd, Urumqi 830011, Xinjiang Uyghur, Peoples R China.

EM 263266418@qq.com; mayinur224@126.com

OI STEPHEN ELI, MCCOY/0000-0002-2304-2364

FU State Key Lab Incubation Base of Xinjiang Major Diseases Research

[SKLIB-XJMDR-2016-5]

FX The present study was supported by the State Key Lab Incubation Base of

Xinjiang Major Diseases Research (grant no. SKLIB-XJMDR-2016-5).

CR Bai G, 2017, BIOMED PHARMACOTHER, V94, P813, DOI 10.1016/j.biopha.2017.07.154

Chen X, 2016, ONCOTARGET, V7, P45948, DOI 10.18632/oncotarget.10008

Fan FJ, 2008, CARCINOGENESIS, V29, P1157, DOI 10.1093/carcin/bgn119

Feng L, 2007, P NATL ACAD SCI USA, V104, P14348, DOI 10.1073/pnas.0701298104

Ferlay J, 2010, INT J CANCER, V127, P2893, DOI 10.1002/ijc.25516

Guo WW, 2016, CARCINOGENESIS, V37, P397, DOI 10.1093/carcin/bgw013

Huang J, 2013, J 3 MILITARY MED U, V8, P1658

Kamangar F, 2006, J CLIN ONCOL, V24, P2137, DOI 10.1200/JCO.2005.05.2308

Li ZH, 2015, ONCOTARGET, V6, P12357, DOI 10.18632/oncotarget.3657

Ling ZQ, 2014, ANN ONCOL, V25, P1363, DOI 10.1093/annonc/mdu168

Livak KJ, 2001, METHODS, V25, P402, DOI 10.1006/meth.2001.1262

Qiao SS, 2015, BIOCHEM J, V469, P469, DOI 10.1042/BJ20150253

Samatar AA, 2014, NAT REV DRUG DISCOV, V13, P928, DOI 10.1038/nrd4281

Tasioudi KE, 2012, PATHOL RES PRACT, V208, P398, DOI 10.1016/j.prp.2012.05.009

Torre LA, 2015, CA-CANCER J CLIN, V65, P87, DOI 10.3322/caac.21262

Wang L, 2017, CELL SIGNAL, V33, P98, DOI 10.1016/j.cellsig.2017.02.017

Wang LD, 2013, ENDOCRINOLOGY, V154, P4525, DOI 10.1210/en.2013-1633

Wang X, 2012, CARCINOGENESIS, V33, P2228, DOI 10.1093/carcin/bgs245

Wu HG, 2014, ONCOL REP, V32, P2687, DOI 10.3892/or.2014.3532

Wu Q, 2016, TUMOR BIOL, V37, P561, DOI 10.1007/s13277-015-3770-y

Xie XD, 2008, CARCINOGENESIS, V29, P1632, DOI 10.1093/carcin/bgn139

Ya MZ, 1988, XINJIANG YIXUEYUAN X, V20, P139

Yu X, 2015, AM J CANCER RES, V5, P2562

NR 23

TC 7

Z9 7

U1 0

U2 0

PU SPANDIDOS PUBL LTD

PI ATHENS

PA POB 18179, ATHENS, 116 10, GREECE

SN 1792-1074

EI 1792-1082

J9 ONCOL LETT

JI Oncol. Lett.

PD APR

PY 2018

VL 15

IS 4

BP 5703

EP 5711

DI 10.3892/ol.2018.8004

PG 9

WC Oncology

WE Science Citation Index Expanded (SCI-EXPANDED)

SC Oncology

GA GD9HA

UT WOS:000430822500219

PM 29552204

OA Green Published, Green Submitted, gold

DA 2022-05-19

ER

PT J

AU Huang, XP

Li, X

Situ, MY

Huang, LY

Wang, JY

He, TC

Yan, QH

Xie, XY

Zhang, YJ

Gao, YH

Li, YH

Rong, TH

Wang, MR

Cai, QQ

Fu, JH

AF Huang, Xiao-Ping

Li, Xuan

Situ, Min-Yi

Huang, Li-Yun

Wang, Jun-Ye

He, Tian-Cheng

Yan, Qi-Hang

Xie, Xiu-Ying

Zhang, Yu-Jing

Gao, Yuan-Hong

Li, Yu-Hong

Rong, Tie-Hua

Wang, Ming-Rong

Cai, Qing-Qing

Fu, Jian-Hua

TI Entinostat reverses cisplatin resistance in esophageal squamous cell

carcinoma via down-regulation of multidrug resistance gene 1

SO CANCER LETTERS

LA English

DT Article

DE Esophageal squamous cell carcinoma; Cisplatin; Entinostat; MDR1

ID HISTONE DEACETYLASE INHIBITORS; BREAST-CANCER; MYELOMA CELLS;

CHEMOTHERAPY; PATHWAY; EFFICACY; MODELS; AGENTS; MS-275; MDR1

AB Cisplatin resistance frequently occurs in esophageal squamous cell carcinoma (ESCC). The underlying mechanism for cisplatin resistance in ESCC remains largely obscure. Here we report that entinostat reversed cisplatin resistance in ESCC both in vitro and in vivo by induction of apoptosis and inhibition of cell proliferation, accompanied by a decrease of multidrug resistance gene 1 (MDR1), P-Src, Mcl-1, Cyclin D1 and an increase of cleaved PARP. MDR1 expression was associated with worsen survival of ESCC patients with cisplatin-based chemotherapy. Dasatinib potentiated entinostat to overcome cisplatin resistance. By inhibiting Src, dasatinib reduced the expression of MDR1 and Mcl-1. Furthermore, Obatoclax, an inhibitor of Mcl-1, obviously decreased the expression of MDR1, suggesting that entinostat might surmount cisplatin resistance in ESCC via a Src-Mcl-1-MDR1 pathway. Interestingly, cisplatin also enhanced the effect of entinostat both in vitro and in vivo. Our data disclose a molecular basis that entinostat reverses cisplatin resistance, and provide a promising strategy with combinatorial drugs to treat cisplatin resistant ESCC patients. (C) 2017 Elsevier B.V. All rights reserved.

C1 [Huang, Xiao-Ping; Li, Xuan; Situ, Min-Yi; Huang, Li-Yun; Wang, Jun-Ye; He, Tian-Cheng; Yan, Qi-Hang; Xie, Xiu-Ying; Zhang, Yu-Jing; Gao, Yuan-Hong; Li, Yu-Hong; Rong, Tie-Hua; Cai, Qing-Qing; Fu, Jian-Hua] Sun Yat Sen Univ, Canc Ctr, State Key Lab Oncol South China, Bldg 2,Room 802,Dongfeng East Rd 651, Guangzhou 510060, Guangdong, Peoples R China.

[Huang, Xiao-Ping; Li, Xuan; He, Tian-Cheng; Yan, Qi-Hang; Xie, Xiu-Ying; Rong, Tie-Hua; Cai, Qing-Qing; Fu, Jian-Hua] Esophageal Canc Inst Guangdong Prov, Guangzhou, Guangdong, Peoples R China.

[Wang, Ming-Rong] Peking Union Med Coll, Canc Hosp, Natl Canc Ctr, State Key Lab Mol Oncol, Beijing, Peoples R China.

[Wang, Ming-Rong] Chinese Acad Med Sci, Beijing, Peoples R China.

RP Huang, XP; Cai, QQ; Fu, JH (corresponding author), Sun Yat Sen Univ, Canc Ctr, State Key Lab Oncol South China, Bldg 2,Room 802,Dongfeng East Rd 651, Guangzhou 510060, Guangdong, Peoples R China.; Wang, MR (corresponding author), Peking Union Med Coll, Canc Hosp, Natl Canc Ctr, State Key Lab Mol Oncol, Beijing, Peoples R China.; Wang, MR (corresponding author), Chinese Acad Med Sci, Beijing, Peoples R China.

EM huangxp@sysucc.org.cn; wangmr2015@126.com; caiqq@sysucc.org.cn;

fujh@sysucc.org.cn

FU Sun Yat-sen University [84000-18821105]; National Natural Science

Foundation of China [30572103, 81330052, 81272635, 81372883]

FX This study was supported by Hundreds of people plan to introduce talent

start fund from Sun Yat-sen University (to Xiao-Ping Huang,

84000-18821105), and National Natural Science Foundation of China

(30572103, 81330052, 81272635, 81372883). We thank professors G.

Srivastava and G.S. Tsao from University of Hong Kong for their

providing us with the cell line HEKSC1.

CR Ahrens TD, 2015, EPIGENETICS-US, V10, P431, DOI 10.1080/15592294.2015.1039216

Friboulet L, 2013, NEW ENGL J MED, V368, P1101, DOI 10.1056/NEJMoa1214271

Guan HT, 2016, TUMOR BIOL, V37, P12153, DOI 10.1007/s13277-016-5092-0

Huang XP, 2011, CANCER LETT, V307, P72, DOI 10.1016/j.canlet.2011.03.019

Huang XP, 2009, CANCER RES, V69, P8403, DOI 10.1158/0008-5472.CAN-09-2146

Lee CK, 2010, CANCER LETT, V296, P233, DOI 10.1016/j.canlet.2010.04.014

Lee JH, 2011, P NATL ACAD SCI USA, V108, P19629, DOI 10.1073/pnas.1117544108

Ma J, 2016, GENE, V587, P147, DOI 10.1016/j.gene.2016.04.054

Merino VF, 2016, CANCER RES, V76, P2013, DOI 10.1158/0008-5472.CAN-15-1619

Mo DL, 2016, CANCER RES, V76, P3057, DOI 10.1158/0008-5472.CAN-15-2361

Mustafi SB, 2016, BBA-GENE REGUL MECH, V1859, P983, DOI [10.1016/j.bbagrm.2016.06.062, 10.1016/j.bbagrm.2016.06.002]

Nidhyanandan S, 2015, EUR J PHARMACOL, V764, P508, DOI 10.1016/j.ejphar.2015.07.048

Purrucker JC, 2010, ONCOL REP, V23, P263, DOI 10.3892/or_00000632

Robert C, 2016, LEUKEMIA RES, V45, P14, DOI 10.1016/j.leukres.2016.03.007

Sakamoto T, 2013, BIOCHEM BIOPH RES CO, V433, P456, DOI 10.1016/j.bbrc.2013.03.009

Strauss J, 2015, LANCET ONCOL, V16, P1013, DOI 10.1016/S1470-2045(15)00231-4

Theodoris CV, 2017, J CLIN INVEST, V127, P1683, DOI 10.1172/JCI90338

Tomita Y, 2016, ONCOIMMUNOLOGY, V5, DOI 10.1080/2162402X.2016.1219008

Tsubaki M, 2014, LEUKEMIA RES, V38, P121, DOI 10.1016/j.leukres.2013.10.017

Vendetti FP, 2015, ONCOTARGET, V6, P56, DOI 10.18632/oncotarget.2695

Yardley DA, 2013, J CLIN ONCOL, V31, P2128, DOI 10.1200/JCO.2012.43.7251

NR 21

TC 14

Z9 17

U1 4

U2 26

PU ELSEVIER IRELAND LTD

PI CLARE

PA ELSEVIER HOUSE, BROOKVALE PLAZA, EAST PARK SHANNON, CO, CLARE, 00000,

IRELAND

SN 0304-3835

EI 1872-7980

J9 CANCER LETT

JI Cancer Lett.

PD FEB 1

PY 2018

VL 414

BP 294

EP 300

DI 10.1016/j.canlet.2017.10.023

PG 7

WC Oncology

WE Science Citation Index Expanded (SCI-EXPANDED)

SC Oncology

GA FS5CK

UT WOS:000419810900030

PM 29107111

DA 2022-05-19

ER

PT J

AU Ma, J

Zhang, J

Ma, YG

Zheng, J

Cheng, YX

Xue, Y

Liu, WC

AF Ma, Ji

Zhang, Jian

Ma, Yuguang

Zheng, Jin

Cheng, Yuanxiong

Xue, Yan

Liu, Wenchao

TI Adenovirus-mediated RhoA shRNA suppresses growth of esophageal squamous

cell carcinoma cells in vitro and in vivo

SO MEDICAL ONCOLOGY

LA English

DT Article

DE RhoA; Esophageal squamous cell carcinoma (ESCC); Adenovirus-mediated

small hairpin RNA (Ad-shRNA)

ID CANCER; PROLIFERATION; MECHANISM; RAC1; RNA

AB Over-expression of RhoA in esophageal squamous cell carcinoma (ESCC) indicates a poor prognosis and is correlated with the tumor-node-metastasis (TNM) clinical classification. However, until now RhoA function in the ESCC progression remains to be established. We employed adenovirus-mediated small hairpin RNA (shRNA) against human RhoA (Ad-sh-RhoA) to efficiently silence target gene expression in RhoA-expressing Eca-109 ESCC cells at both protein and mRNA levels. Consequently, Ad-sh-RhoA reduced the proliferation and migration of Eca-109 cells assayed by MTT assay and cell wound healing, respectively. Moreover, Ad-sh-RhoA increased cell apoptosis and inhibited the cell cycle G1-S-phase progression of Eca-109 cells assessed by flow cytometry. Finally, in a nude mouse model, intratumoral injections of adenovirus-delivered RhoA shRNA every 3 days for 20 days significantly inhibited the growth and angiogenesis of xenografted Eca-109 tumors. In summary, these data indicate that RhoA may be a key molecule in ESCC cells, and thus, specific inhibition of the Rho signaling pathway with adenovirus-delivered shRNA represents a promising approach for the treatment of aggressive ESCC.

C1 [Ma, Ji; Ma, Yuguang; Zheng, Jin; Xue, Yan; Liu, Wenchao] Fourth Mil Med Univ, Xijing Hosp, Dept Oncol, Xian 710032, Peoples R China.

[Zhang, Jian] Fourth Mil Med Univ, Dept Biochem & Mol Biol, Xian 710032, Peoples R China.

[Cheng, Yuanxiong] So Med Univ, Nanfang Hosp, Dept Resp, Guangzhou 510515, Guangdong, Peoples R China.

RP Xue, Y (corresponding author), Fourth Mil Med Univ, Xijing Hosp, Dept Oncol, Xian 710032, Peoples R China.

EM xjcancer@fmmu.edu.cn; xjcancer@fmmu.edu.cn

FU National Natural Science Foundation of China [30770823, 30572100]

FX This work was supported by grants from the National Natural Science

Foundation of China (nos. 30770823 and 30572100).

CR Brown LM, 2001, AM J EPIDEMIOL, V153, P114, DOI 10.1093/aje/153.2.114

del Pulgar TG, 2005, BIOESSAYS, V27, P602, DOI 10.1002/bies.20238

Denoyelle C, 2003, CELL SIGNAL, V15, P327, DOI 10.1016/S0898-6568(02)00124-9

Enzinger PC, 2003, NEW ENGL J MED, V349, P2241, DOI 10.1056/NEJMra035010

Fan YM, 2008, NEUROSCI LETT, V440, P170, DOI 10.1016/j.neulet.2008.05.045

Faried A, 2006, EUR J CANCER, V42, P1455, DOI 10.1016/j.ejca.2006.02.012

Faried A, 2005, EJSO, V31, P410, DOI 10.1016/j.ejso.2004.12.014

Faried A, 2007, ANN SURG ONCOL, V14, P3593, DOI 10.1245/s10434-007-9562-x

Feig LA, 1999, NAT CELL BIOL, V1, pE25, DOI 10.1038/10018

Frame MC, 2002, CURR OPIN GENET DEV, V12, P36, DOI 10.1016/S0959-437X(01)00261-1

Hannon GJ, 2002, NATURE, V418, P244, DOI 10.1038/418244a

Jemal A, 2008, CA-CANCER J CLIN, V58, P71, DOI 10.3322/CA.2007.0010

Kamangar F, 2006, J CLIN ONCOL, V24, P2137, DOI 10.1200/JCO.2005.05.2308

Li H, 2001, HUM GENE THER, V12, P515, DOI 10.1089/104303401300042429

Liberto M, 2002, ONCOGENE, V21, P1590, DOI 10.1038/sj.onc.1205242

Mammoto A, 2004, J BIOL CHEM, V279, P26323, DOI 10.1074/jbc.M402725200

Marler KJM, 2005, MOL CELL BIOL, V25, P5226, DOI 10.1128/MCB.25.12.5226-5241.2005

Pickens A, 2003, ANN THORAC SURG, V76, pS1367, DOI 10.1016/S0003-4975(03)01202-5

Pille JY, 2005, MOL THER, V11, P267, DOI 10.1016/j.ymthe.2004.08.029

Price LS, 2001, SEMIN CANCER BIOL, V11, P167, DOI 10.1006/scbi.2000.0367

Sebok A, 1999, J NEUROCHEM, V73, P949, DOI 10.1046/j.1471-4159.1999.0730949.x

Soga N, 2001, CELL COMMUN ADHES, V8, P1, DOI 10.3109/15419060109080703

Stoner GD, 2001, CARCINOGENESIS, V22, P1737, DOI 10.1093/carcin/22.11.1737

Takata K, 2008, MOL CANCER THER, V7, P1551, DOI 10.1158/1535-7163.MCT-07-0428

Woodward HN, 2009, AM J PHYSIOL-LUNG C, V297, pL954, DOI 10.1152/ajplung.00038.2009

Xue Y, 2004, BIOCHEM BIOPH RES CO, V320, P1309, DOI 10.1016/j.bbrc.2004.06.088

Xue Y, 2006, INT J CANCER, V118, P2965, DOI 10.1002/ijc.21763

Zhang SY, 2009, MOL CANCER RES, V7, P570, DOI 10.1158/1541-7786.MCR-08-0248

NR 28

TC 6

Z9 9

U1 2

U2 14

PU HUMANA PRESS INC

PI TOTOWA

PA 999 RIVERVIEW DRIVE SUITE 208, TOTOWA, NJ 07512 USA

SN 1357-0560

EI 1559-131X

J9 MED ONCOL

JI Med. Oncol.

PD MAR

PY 2012

VL 29

IS 1

BP 119

EP 126

DI 10.1007/s12032-010-9774-y

PG 8

WC Oncology

WE Science Citation Index Expanded (SCI-EXPANDED)

SC Oncology

GA 892WV

UT WOS:000300317200018

PM 21161732

DA 2022-05-19

ER

PT J

AU Meng, J

Zhang, C

Zhao, TQ

Shi, GW

Zhao, JJ

Lin, ZX

AF Meng, Jing

Zhang, Chao

Zhao, Tongquan

Shi, Guangwen

Zhao, Jingjing

Lin, Zhaoxia

TI MicroRNA-210 targets FBXO31 to inhibit tumor progression and regulates

the Wnt/beta-catenin signaling pathway and EMT in esophageal squamous

cell carcinoma

SO THORACIC CANCER

LA English

DT Article

DE Epithelial-mesenchymal transition; Esophageal squamous cell carcinoma;

F-Box only protein 31; miR-210; Wnt/beta-catenin

AB Evidence from previous studies showed that the dysregulation of microRNA (miR) is frequently associated with tumor progression. The aberrant miR-210 expression has been identified in a variety of tumors. However, its biological roles in esophageal squamous cell carcinoma (ESCC) still need further elucidation. Thus, in the current study we explore the roles of miR-210 in ESCC progression. The findings of our study reveal that miR-210 is down-regulated in ESCC, which indicates poor prognosis and aggressive tumor progression. Moreover, miR-210 restoration was found to enhance ESCC viability, invasion, and migration abilities. F-Box only protein 31 (FBXO31) was confirmed to be one of the targets of miR-210 in ESCC cells. Results also revealed that miR-210 played crucial roles in regulating ESCC cell epithelial-mesenchymal transition (EMT) and Wnt/beta-catenin signaling. In conclusion, data show that miR-210 serves as an anti-ESCC miR via down-regulation of FBXO31 and regulation of EMT and Wnt signaling, suggesting that the miR-210/FBXO31 axis may function as promising therapeutic targets and effective prognostic markers for ESCC patients.

C1 [Meng, Jing; Zhang, Chao] Rizhao Hosp TCM, Dept Gastroenterol, Rizhao, Peoples R China.

[Zhao, Tongquan] Peoples Hosp Rizhao, Dept Gen Surg, Rizhao, Peoples R China.

[Shi, Guangwen] Zhangqiu Dist Peoples Hosp, Hlth Management Ctr, Jinan, Peoples R China.

[Zhao, Jingjing] Zhangqiu Dist Peoples Hosp, Dept Surg, Jinan, Peoples R China.

[Lin, Zhaoxia] Shandong Univ, Jinan Cent Hosp, Cheeloo Coll Med, Dept Clin Lab, 105 Jiefang Rd, Jinan 250013, Shandong, Peoples R China.

RP Lin, ZX (corresponding author), Shandong Univ, Jinan Cent Hosp, Cheeloo Coll Med, Dept Clin Lab, 105 Jiefang Rd, Jinan 250013, Shandong, Peoples R China.

EM zdp787878@163.com

CR Acloque H, 2009, J CLIN INVEST, V119, P1438, DOI 10.1172/JCI38019

Balachandran AA, 2020, CANCERS, V12, DOI 10.3390/cancers12092534

Bavelloni A, 2017, ANTICANCER RES, V37, P6511, DOI 10.21873/anticanres.12107

Breunig C, 2017, CELL DEATH DIS, V8, DOI 10.1038/cddis.2017.364

Feng S, 2019, ONCOTARGETS THER, V12, P479, DOI 10.2147/OTT.S184564

Gao GD, 2018, EUR REV MED PHARMACO, V22, P422, DOI 10.26355/eurrev_201801_14191

Ge CL, 2015, ONCOTARGET, V6, P10964, DOI 10.18632/oncotarget.3696

Ghobadi N, 2019, J CELL PHYSIOL, V234, P5070, DOI 10.1002/jcp.27310

Hagio K, 2019, ANTICANCER RES, V39, P557, DOI 10.21873/anticanres.13148

Huang HL, 2015, AM J CANCER RES, V5, P1814

Huang HL, 2010, ONCOL REP, V24, P715, DOI 10.3892/or_00000912

Jiang B, 2018, EUR REV MED PHARMACO, V22, P3789, DOI 10.26355/eurrev_201806_15261

Kumar R, 2005, CANCER RES, V65, P11304, DOI 10.1158/0008-5472.CAN-05-0936

Larue L, 2005, ONCOGENE, V24, P7443, DOI 10.1038/sj.onc.1209091

Liu S, 2020, CANCER SCI, V111, P3881, DOI 10.1111/cas.14550

Luo JQ, 2018, ONCOL LETT, V15, P5803, DOI 10.3892/ol.2018.8025

Maghsudlu M, 2020, J GASTROINTEST CANC, V51, P227, DOI 10.1007/s12029-019-00232-x

Mariette C, 2007, LANCET ONCOL, V8, P545, DOI 10.1016/S1470-2045(07)70172-9

Napier KJ, 2014, WORLD J GASTRO ONCOL, V6, P112, DOI 10.4251/wjgo.v6.i5.112

Santra MK, 2009, NATURE, V459, P722, DOI 10.1038/nature08011

Shang C, 2014, MED SCI MONITOR, V20, P2571, DOI 10.12659/MSM.892994

Shiloh Y, 2009, SCI SIGNAL, V2, DOI 10.1126/scisignal.296pe73

Shukla Girish C, 2011, Mol Cell Pharmacol, V3, P83

Siegel RL, 2019, CA-CANCER J CLIN, V69, P7, DOI 10.3322/caac.21551

Song YM, 2014, NATURE, V509, P91, DOI 10.1038/nature13176

Stemmler MP, 2019, NAT CELL BIOL, V21, P102, DOI 10.1038/s41556-018-0196-y

Taciak B, 2018, J PHYSIOL PHARMACOL, V69, DOI 10.26402/jpp.2018.2.07

Toh Y, 2013, GEN THORAC CARDIOVAS, V61, P262, DOI 10.1007/s11748-013-0235-3

Tsuchiya S, 2012, YAKUGAKU ZASSHI, V132, P1069, DOI 10.1248/yakushi.132.1069

Uhlenhopp DJ, 2020, CLIN J GASTROENTEROL, V13, P1010, DOI 10.1007/s12328-020-01237-x

Xie SP, 2019, J CELL PHYSIOL, V234, P14050, DOI 10.1002/jcp.28093

Xie Y, 2019, ARTIF CELL NANOMED B, V47, P1722, DOI 10.1080/21691401.2019.1606006

Yang H, 2017, EUR REV MED PHARMACO, V21, P3200

Yang S, 2021, J SURG RES, V257, P267, DOI 10.1016/j.jss.2020.07.074

Yuan RB, 2018, EUR REV MED PHARMACO, V22, P7265, DOI 10.26355/eurrev_201811_16261

Zhang L, 2020, BIOSCIENCE REP, V40, DOI 10.1042/BSR20201901

Zhang L, 2020, ACTA BIOCH BIOPH SIN, V52, P842, DOI 10.1093/abbs/gmaa059

Zhang N, 2020, BIOENGINEERED, V11, P972, DOI 10.1080/21655979.2020.1814658

Zheng SY, 2017, CANCER BIOMARK, V20, P49, DOI 10.3233/CBM-170079

Zheng YJ, 2020, ARTIF CELL NANOMED B, V48, P977, DOI 10.1080/21691401.2020.1773479

Zou SY, 2018, MOL CANCER RES, V16, P286, DOI 10.1158/1541-7786.MCR-17-0432

NR 41

TC 1

Z9 1

U1 1

U2 2

PU WILEY

PI HOBOKEN

PA 111 RIVER ST, HOBOKEN 07030-5774, NJ USA

SN 1759-7706

EI 1759-7714

J9 THORAC CANCER

JI Thorac. Cancer

PD MAR

PY 2021

VL 12

IS 6

BP 932

EP 940

DI 10.1111/1759-7714.13860

EA FEB 2021

PG 9

WC Oncology; Respiratory System

WE Science Citation Index Expanded (SCI-EXPANDED)

SC Oncology; Respiratory System

GA QV0SY

UT WOS:000614330600001

PM 33538099

OA Green Published, gold

DA 2022-05-19

ER

PT J

AU Li, HL

Chu, J

Jia, JL

Sheng, JX

Zhao, X

Xing, YR

He, FC

AF Li, Hongle

Chu, Jie

Jia, Jinlin

Sheng, Jinxiu

Zhao, Xue

Xing, Yurong

He, Fucheng

TI LncRNA LOXL1-AS1 promotes esophageal squamous cell carcinoma progression

by targeting DESC1

SO JOURNAL OF CANCER

LA English

DT Article

DE Esophageal squamous cell carcinoma; long non-coding RNA; LOXL1-AS1;

DESC1

ID LONG NONCODING RNAS; HEPATOCELLULAR-CARCINOMA; TUMOR-SUPPRESSOR;

BREAST-CANCER; METASTASIS; EXPRESSION; INVASION; ACTS; PROLIFERATION

AB Recently, ample evidence indicated that numerous aberrantly expressed long non-coding RNAs (lncRNAs) participated in the development of multiple malignancies. However, the expression and function of lncRNA LOXL1-AS1 in mediating esophageal squamous cell carcinoma (ESCC) carcinogenesis remains largely elusive. Here we validated that LOXL1-AS1 was significantly upregulated in ESCC tissues compared with the corresponding adjacent non-neoplastic tissues, and LOXL1-AS1 expression was positively correlated with ESCC patients' lymph node metastasis. Besides, LOXL1-AS1 knockdown impaired ESCC cells proliferation, migration and invasion capabilities in vitro. Furthermore, inhibiting LOXL1-AS1 in ESCC cells increased the percentage of cells at the G1 phase, accompanied by reducing in S phase in contrast to scramble control, and silencing of LOXL1-AS1 evoked ESCC cell apoptosis. From high throughput RNA sequencing (RNA-seq) analysis, we identified that differentially expressed in squamous cell carcinoma 1 (DESC1) was a critical downstream target of LOXL1-AS1. Taken together, we demonstrated the function and mechanism of LOXL1-AS1 in contributing ESCC progression for the first time, and indicated LOXL1-AS1 may be a novel therapeutic biomarker of ESCC.

C1 [Li, Hongle] Zhengzhou Univ, Dept Mol Pathol, Affiliated Canc Hosp, Zhengzhou 450008, Henan, Peoples R China.

[Chu, Jie; Jia, Jinlin; Sheng, Jinxiu; He, Fucheng] Zhengzhou Univ, Dept Med Lab, Affiliated Hosp 1, Zhengzhou 450008, Henan, Peoples R China.

[Zhao, Xue] Zhengzhou Univ, Inst Med & Pharmaceut Sci, Zhengzhou 450052, Henan, Peoples R China.

[Xing, Yurong] Zhengzhou Univ, Dept Phys Examinat, Affiliated Hosp 1, Zhengzhou 450052, Henan, Peoples R China.

RP Li, HL (corresponding author), Zhengzhou Univ, Dept Mol Pathol, Affiliated Canc Hosp, Zhengzhou 450008, Henan, Peoples R China.; He, FC (corresponding author), Zhengzhou Univ, Dept Med Lab, Affiliated Hosp 1, Zhengzhou 450008, Henan, Peoples R China.

EM llhl73@163.com; hefucheng@zzu.edu.cn

FU Medical Science and Technology Joint Construction Project of Henan

province of China [201901102]; Medical Scientific Research Foundation of

Health Department of Henan Province of China [201403077]; Science and

Technology Project of Henan province of China [192102310133]

FX The study was supported by grants from Medical Science and Technology

Joint Construction Project of Henan province of China (Grant No.

201901102), Medical Scientific Research Foundation of Health Department

of Henan Province of China (Grant No. 201403077), the Science and

Technology Project of Henan province of China (Grant No. 202102310112)

and the Science and Technology Project of Henan province of China (Grant

No. 192102310133).

CR Bai TL, 2019, IUBMB LIFE, V71, P1537, DOI 10.1002/iub.2075

Chang ZW, 2018, J EXP CLIN CANC RES, V37, DOI 10.1186/s13046-018-0724-4

Chen JZ, 2018, AGING-US, V10, P3371, DOI 10.18632/aging.101645

Chen XG, 2019, BIOMED PHARMACOTHER, V120, DOI 10.1016/j.biopha.2019.109438

Chu J, 2020, BIOMED PHARMACOTHER, V128, DOI 10.1016/j.biopha.2020.110313

Chu J, 2019, BIOMED PHARMACOTHER, V116, DOI 10.1016/j.biopha.2019.109029

Codipilly DC, 2018, GASTROINTEST ENDOSC, V88, P413, DOI 10.1016/j.gie.2018.04.2352

Cotton RGH, 2014, ANN NY ACAD SCI, V1325, P138, DOI 10.1111/nyas.12522

Dai FQ, 2017, GENOMICS, V109, P241, DOI 10.1016/j.ygeno.2017.04.005

Dong HT, 2020, MOL THER-NUCL ACIDS, V19, P696, DOI 10.1016/j.omtn.2019.12.016

Duan Y, 2020, J CANCER, V11, P3492, DOI 10.7150/jca.42070

Gao R, 2018, ANAL CELL PATHOL, V2018, DOI 10.1155/2018/9275685

Guo YJ, 2019, BIOMED PHARMACOTHER, V118, DOI 10.1016/j.biopha.2019.109272

Han DF, 2019, J CANCER, V10, P6738, DOI 10.7150/jca.35770

Kyrieleis OJP, 2007, FEBS J, V274, P2148, DOI 10.1111/j.1742-4658.2007.05756.x

Li GH, 2019, EUR REV MED PHARMACO, V23, P10400, DOI 10.26355/eurrev_201912_19679

Li W, 2020, CANCER MED-US, V9, P689, DOI 10.1002/cam4.2641

Liang Y, 2018, CELL DEATH DIFFER, V25, P1980, DOI 10.1038/s41418-018-0084-9

Liu SH, 2019, J CANCER, V10, P6649, DOI 10.7150/jca.29213

Mercer TR, 2009, NAT REV GENET, V10, P155, DOI 10.1038/nrg2521

Pennathur A, 2013, LANCET, V381, P400, DOI 10.1016/S0140-6736(12)60643-6

Sun KY, 2018, LIFE SCI, V202, P124, DOI 10.1016/j.lfs.2018.03.050

Sun Q, 2019, CELL PROLIFERAT, V52, DOI 10.1111/cpr.12687

Wang HJ, 2018, BIOCHEM BIOPH RES CO, V500, P518, DOI 10.1016/j.bbrc.2018.04.133

Wang R, 2018, MOL CANCER, V17, P1, DOI DOI 10.1186/s12943-017-0753-1

Wang ZQ, 2019, J CANCER, V10, P6414, DOI 10.7150/jca.32251

Yang LJ, 2019, CANCER MANAG RES, V11, P4425, DOI 10.2147/CMAR.S196004

Yang LJ, 2018, BIOMED PHARMACOTHER, V106, P1570, DOI 10.1016/j.biopha.2018.07.129

Yang XL, 2020, BIOMED PHARMACOTHER, V125, DOI 10.1016/j.biopha.2020.109839

You ST, 2019, BIOMATER SCI SER, V3, P1

Zhang BQ, 2019, BIOCHEM BIOPH RES CO, V513, P827, DOI 10.1016/j.bbrc.2019.04.089

Zhang EB, 2018, GENOME BIOL, V19, DOI 10.1186/s13059-018-1523-0

Zhang GJ, 2019, BIOMED PHARMACOTHER, V118, DOI 10.1016/j.biopha.2019.109309

Zhao WY, 2019, J CANCER, V10, P6693, DOI 10.7150/jca.30877

NR 34

TC 3

Z9 3

U1 1

U2 1

PU IVYSPRING INT PUBL

PI LAKE HAVEN

PA PO BOX 4546, LAKE HAVEN, NSW 2263, AUSTRALIA

SN 1837-9664

J9 J CANCER

JI J. Cancer

PY 2021

VL 12

IS 2

BP 530

EP 538

DI 10.7150/jca.51136

PG 9

WC Oncology

WE Science Citation Index Expanded (SCI-EXPANDED)

SC Oncology

GA OV1BD

UT WOS:000591953400023

PM 33391449

OA Green Published, gold

DA 2022-05-19

ER

PT J

AU Matsui, T

Chiyo, T

Kobara, H

Fujihara, S

Fujita, K

Namima, D

Nakahara, M

Kobayashi, N

Nishiyama, N

Yachida, T

Morishita, A

Iwama, H

Masaki, T

AF Matsui, Takanori

Chiyo, Taiga

Kobara, Hideki

Fujihara, Shintaro

Fujita, Koji

Namima, Daisuke

Nakahara, Mai

Kobayashi, Nobuya

Nishiyama, Noriko

Yachida, Tatsuo

Morishita, Asahiro

Iwama, Hisakazu

Masaki, Tsutomu

TI Telmisartan Inhibits Cell Proliferation and Tumor Growth of Esophageal

Squamous Cell Carcinoma by Inducing S-Phase Arrest In Vitro and In Vivo

SO INTERNATIONAL JOURNAL OF MOLECULAR SCIENCES

LA English

DT Article

DE esophageal squamous cell carcinoma; telmisartan; cyclin; angiotensin II

type 1 receptor blocker; cell cycle arrest

ID ANGIOTENSIN-II; CANCER-CELLS; HEPATOCELLULAR-CARCINOMA;

PROGNOSTIC-SIGNIFICANCE; RECEPTOR; CHOLANGIOCARCINOMA; ADENOCARCINOMA;

MICRORNA-765; PROGRESSION; ACTIVATION

AB Esophageal squamous cell carcinoma (ESCC) is the most common primary esophageal malignancy. Telmisartan, an angiotensin II type 1 (AT1) receptor blocker (ARB) and a widely used antihypertensive, has been shown to inhibit proliferation of various cancer types. This study evaluated the effects of telmisartan on human ESCC cell proliferation in vitro and in vivo and sought to identify the microRNAs (miRNAs) involved in these antitumor effects. We examined the effects of telmisartan on three human ESCC cell lines (KYSE150, KYSE180, and KYSE850). Telmisartan inhibited proliferation of these three cell lines by inducing S-phase arrest, which was accompanied by decreased expression of cyclin A2, cyclin-dependent kinase 2, and other cell cycle-related proteins. Additionally, telmisartan reduced levels of phosphorylated ErbB3 and thrombospondin-1 in KYSE180 cells. Furthermore, expression of miRNAs was remarkably altered by telmisartan in vitro. Telmisartan also inhibited tumor growth in vivo in a xenograft mouse model. In conclusion, telmisartan inhibited cell proliferation and tumor growth in ESCC cells by inducing cell-cycle arrest.

C1 [Matsui, Takanori; Chiyo, Taiga; Kobara, Hideki; Fujihara, Shintaro; Fujita, Koji; Namima, Daisuke; Nakahara, Mai; Kobayashi, Nobuya; Nishiyama, Noriko; Yachida, Tatsuo; Morishita, Asahiro; Masaki, Tsutomu] Kagawa Univ, Dept Gastroenterol & Neurol, Miki, Kagawa 7610793, Japan.

[Iwama, Hisakazu] Kagawa Univ, Life Sci Res Ctr, Miki, Kagawa 7610793, Japan.

RP Masaki, T (corresponding author), Kagawa Univ, Dept Gastroenterol & Neurol, Miki, Kagawa 7610793, Japan.

EM tmasaki@med.kagawa-u.ac.jp

RI Fujihara, Shintaro/AAW-6092-2021

OI Fujihara, Shintaro/0000-0002-1250-6012

CR Beji A, 2012, CLIN CANCER RES, V18, P956, DOI 10.1158/1078-0432.CCR-11-1186

Bray F, 2018, CA-CANCER J CLIN, V68, P394, DOI 10.3322/caac.21492

Chen YH, 2015, J RENIN-ANGIO-ALDO S, V16, P1185, DOI 10.1177/1470320314535275

CORDONCARDO C, 1995, AM J PATHOL, V147, P545

D'Incalci M, 2003, EUR J CANCER, V39, P1920, DOI 10.1016/S0959-8049(03)00490-8

Du N, 2012, ONCOL REP, V27, P1893, DOI 10.3892/or.2012.1720

Fujihara S, 2017, ONCOTARGET, V8, P8536, DOI 10.18632/oncotarget.14345

Funao K, 2008, ONCOL REP, V20, P295, DOI 10.3892/or_00000006

Furihata M, 1996, CLIN CANCER RES, V2, P1781

Hong XC, 2015, MOL MED REP, V12, P6445, DOI 10.3892/mmr.2015.4326

Huang JX, 2012, ONCOL LETT, V4, P607, DOI 10.3892/ol.2012.814

HUNTER T, 1994, CELL, V79, P573, DOI 10.1016/0092-8674(94)90543-6

Kinoshita J, 2009, INT J ONCOL, V34, P1573, DOI 10.3892/ijo_00000287

Koyama N, 2014, PLOS ONE, V9, DOI 10.1371/journal.pone.0093050

Kozako T, 2016, FEBS OPEN BIO, V6, P442, DOI 10.1002/2211-5463.12055

Lee H, 2014, ONCOTARGET, V5, P5138, DOI 10.18632/oncotarget.2094

Lee LD, 2014, ONCOL LETT, V8, P2681, DOI 10.3892/ol.2014.2592

Li J, 2014, MOLECULES, V19, P2862, DOI 10.3390/molecules19032862

Liang W, 2017, J CANCER, V8, P1542, DOI 10.7150/jca.18680

Masaki T, 2003, HEPATOLOGY, V37, P534, DOI 10.1053/jhep.2003.50112

Matsuyama M, 2010, EXP THER MED, V1, P301, DOI 10.3892/etm_00000046

Morishita A, 2015, HEPATOL RES, V45, P128, DOI 10.1111/hepr.12386

Okamoto K, 2010, INT J ONCOL, V37, P1251, DOI 10.3892/ijo_00000776

Oura K, 2017, ONCOL REP, V38, P2825, DOI 10.3892/or.2017.5977

PAGANO M, 1991, Progress in Growth Factor Research, V3, P267, DOI 10.1016/0955-2235(91)90004-N

Pennathur A, 2013, LANCET, V381, P400, DOI 10.1016/S0140-6736(12)60643-6

Pu ZC, 2016, MOL MED REP, V13, P555, DOI 10.3892/mmr.2015.4512

Samukawa E, 2017, INT J ONCOL, V51, P1674, DOI 10.3892/ijo.2017.4177

Short MW, 2017, AM FAM PHYSICIAN, V95, P22

Sithanandam G, 2008, CANCER GENE THER, V15, P413, DOI 10.1038/cgt.2008.15

Smyth EC, 2017, NAT REV DIS PRIMERS, V3, DOI 10.1038/nrdp.2017.48

Tzeng HT, 2017, CLIN CANCER RES, V23, P2335, DOI 10.1158/1078-0432.CCR-16-1520

World Cancer Research Fund International, OES CANC REP 2016

Zhang SL, 2018, ONCOL LETT, V15, P5859, DOI 10.3892/ol.2018.8002

Zhou ZQ, 2009, BMC CANCER, V9, DOI 10.1186/1471-2407-9-291

NR 35

TC 21

Z9 22

U1 0

U2 1

PU MDPI

PI BASEL

PA ST ALBAN-ANLAGE 66, CH-4052 BASEL, SWITZERLAND

SN 1422-0067

J9 INT J MOL SCI

JI Int. J. Mol. Sci.

PD JUL 1

PY 2019

VL 20

IS 13

AR 3197

DI 10.3390/ijms20133197

PG 14

WC Biochemistry & Molecular Biology; Chemistry, Multidisciplinary

WE Science Citation Index Expanded (SCI-EXPANDED)

SC Biochemistry & Molecular Biology; Chemistry

GA IL1EG

UT WOS:000477041100085

PM 31261874

OA Green Published, Green Submitted, gold

DA 2022-05-19

ER

PT J

AU Xu, CH

Guo, YM

Liu, HY

Chen, GB

Yan, YJ

Liu, T

AF Xu, Caihui

Guo, Yinmou

Liu, Haiyan

Chen, Gongbin

Yan, Yanju

Liu, Teng

TI TUG1 confers cisplatin resistance in esophageal squamous cell carcinoma

by epigenetically suppressing PDCD4 expression via EZH2

SO CELL AND BIOSCIENCE

LA English

DT Article

DE Esophageal squamous cell carcinoma; Cisplatin; Taurine upregulated gene

1; Enhancer of zeste homolog 2; PDCD4

ID LONG NONCODING RNA; COLORECTAL-CANCER; PROLIFERATION; MIGRATION;

THERAPY; GROWTH

AB Increasing evidence has suggested the involvement of long non-coding RNA taurine upregulated gene 1 (TUG1) in chemoresistance of cancer treatment. However, its function and molecular mechanisms in esophageal squamous cell carcinoma (ESCC) chemoresistance are still not well elucidated. In the present study, we investigate the functional role of TUG1 in cisplatin (DDP) resistance of ESCC and discover the underlying molecular mechanism.

Our study revealed that TUG1 was up-regulated in DDP-resistant ESCC tissues and cells. High TUG1 expression was correlated with poor prognosis of ESCC patients. TUG1 knockdown improved the sensitivity of ECA109/DDP and EC9706/DDP cells to DDP. Moreover, TUG1 could epigenetically suppress PDCD4 expression via recruiting enhancer of zeste homolog 2. PDCD4 overexpression could mimic the functional role of down-regulated TUG1 in DDP resistance. PDCD4 knockdown counteracted the inductive effect of TUG1 inhibition on DDP sensitivity of ECA109/DDP and EC9706/DDP cells. Furthermore, TUG1 knockdown facilitated DDP sensitivity of DDP-resistant ESCC cells in vivo.

TUG1 knockdown overcame DDP resistance of ESCC by epigenetically silencing PDCD4, providing a novel therapeutic target for ESCC.

C1 [Xu, Caihui; Guo, Yinmou; Liu, Haiyan; Chen, Gongbin; Yan, Yanju] Shangqiu First Peoples Hosp, Dept Oncol, 292 Kaixuan South Rd, Shangqiu 476100, Peoples R China.

[Liu, Teng] Xinxiang Med Coll, Dept Oncol, 601 Jinsui Ave, Xinxiang 453003, Peoples R China.

RP Guo, YM (corresponding author), Shangqiu First Peoples Hosp, Dept Oncol, 292 Kaixuan South Rd, Shangqiu 476100, Peoples R China.

EM guoym18917@163.com

CR Cao R, 2004, CURR OPIN GENET DEV, V14, P155, DOI 10.1016/j.gde.2004.02.001

Cao R, 2002, SCIENCE, V298, P1039, DOI 10.1126/science.1076997

Chen YA, 2016, ONCOL LETT, V12, P5170, DOI 10.3892/ol.2016.5323

He LR, 2010, INT J CANCER, V127, P138, DOI 10.1002/ijc.25031

Hummel R, 2011, J GASTROINTEST SURG, V15, P429, DOI 10.1007/s11605-011-1418-9

Jemal A, 2011, CA-CANCER J CLIN, V61, P69, DOI [10.3322/caac.20107, 10.3322/caac.20115]

Lei KF, 2007, BMC CANCER, V7, DOI 10.1186/1471-2407-7-172

Li CF, 2017, BIOCHEM BIOPH RES CO, V491, P552, DOI 10.1016/j.bbrc.2017.03.042

Lin PC, 2016, BMC CANCER, V16, DOI 10.1186/s12885-016-2569-6

Liu CY, 2013, J CHIN MED ASSOC, V76, P570, DOI 10.1016/j.jcma.2013.07.001

Liu H, 2017, ONCOTARGET, V8, P101899, DOI 10.18632/oncotarget.22058

Lv J, 2018, INT J BIOCHEM CELL B, V101, P19, DOI 10.1016/j.biocel.2018.05.010

Meng H, 2015, AM J CANCER RES, V5, P1062

Niu YC, 2017, MOL CANCER, V16, DOI 10.1186/s12943-016-0575-6

Pasquali S, 2017, ANN SURG, V265, P481, DOI 10.1097/SLA.0000000000001905

Pennathur A, 2013, LANCET, V381, P400, DOI 10.1016/S0140-6736(12)60643-6

Prensner JR, 2011, CANCER DISCOV, V1, P391, DOI 10.1158/2159-8290.CD-11-0209

Qi P, 2013, MODERN PATHOL, V26, P155, DOI 10.1038/modpathol.2012.160

Qiu MT, 2013, TUMOR BIOL, V34, P613, DOI 10.1007/s13277-013-0658-6

Qu L, 2016, CANCER CELL, V29, P653, DOI 10.1016/j.ccell.2016.03.004

Santhanam AN, 2010, ONCOGENE, V29, P3921, DOI 10.1038/onc.2010.158

Shi GH, 2010, ACTA PHARMACOL SIN, V31, P867, DOI 10.1038/aps.2010.48

Sun CC, 2016, MOL THER-NUCL ACIDS, V5, DOI 10.1038/mtna.2016.94

Tang TL, 2018, BIOMED PHARMACOTHER, V107, P338, DOI 10.1016/j.biopha.2018.07.076

Wang HH, 2017, ONCOTARGET, V8, P64638, DOI 10.18632/oncotarget.18344

Wang HJ, 2018, BIOMED PHARMACOTHER, V106, P1607, DOI 10.1016/j.biopha.2018.07.131

Wang W, 2013, PLOS ONE, V8, DOI 10.1371/journal.pone.0076538

Xu YT, 2015, TUMOR BIOL, V36, P1643, DOI 10.1007/s13277-014-2763-6

Yen YC, 2017, CANCER-AM CANCER SOC, V123, P2043, DOI 10.1002/cncr.30565

Yin YK, 2018, CELL BIOSCI, V8, DOI 10.1186/s13578-018-0240-4

Young TL, 2005, CURR BIOL, V15, P501, DOI 10.1016/j.cub.2005.02.027

Zhang E, 2016, CELL DEATH DIS, V7, DOI 10.1038/cddis.2015.356

Zhang EB, 2014, CELL DEATH DIS, V5, DOI 10.1038/cddis.2014.201

Zhang YF, 2016, J MED BIOCHEM, V35, P137, DOI 10.1515/jomb-2015-0016

Zhen Y, 2017, ONCOGENE, V36, P275, DOI 10.1038/onc.2016.201

NR 35

TC 27

Z9 28

U1 1

U2 10

PU BMC

PI LONDON

PA CAMPUS, 4 CRINAN ST, LONDON N1 9XW, ENGLAND

EI 2045-3701

J9 CELL BIOSCI

JI Cell Biosci.

PD NOV 28

PY 2018

VL 8

AR 61

DI 10.1186/s13578-018-0260-0

PG 11

WC Biochemistry & Molecular Biology

WE Science Citation Index Expanded (SCI-EXPANDED)

SC Biochemistry & Molecular Biology

GA HD5ST

UT WOS:000452591500001

PM 30519392

OA Green Published, gold

DA 2022-05-19

ER

PT J

AU Li, K

Wang, J

Ma, ZB

Guo, GH

AF Li, Kun

Wang, Jian

Ma, Zhen-Bin

Guo, Guang-Hong

TI Association between clusterin polymorphisms and esophageal squamous cell

carcinoma risk in Han Chinese population

SO INTERNATIONAL JOURNAL OF CLINICAL AND EXPERIMENTAL MEDICINE

LA English

DT Article

DE Allele; CLU; esophageal squamous cell carcinoma; polymorphism

ID CLUSTERIN/APOLIPOPROTEIN-J; ALZHEIMERS-DISEASE; CANCER STATISTICS;

PROSTATE-CANCER; SECRETED FORM; EXPRESSION; GENE; TUMORIGENESIS;

PREDICTOR; SURVIVAL

AB Genetic susceptibility plays an essential role in an individual's risk of esophageal squamous cell carcinoma (ESCC). The aim of this study is to investigate the associations between clusterin (CLU) gene polymorphisms and ESCC risk. We undertook a case-control study to analyze three CLU polymorphisms (gene rs9331888 C>G, rs17466684 A>G and rs1532278 T>C) in an Han Chinese population, by extraction of genomic DNA from the peripheral blood of 642 patients with ESCC and 658 control participants, and performed CLU genotyping using DNA sequencing. The obtained results indicated that overall, no statistically significant association was observed in rs17466684 and rs1532278. However, gene rs9331888 C>G genotype was at increased risk of ESCCs (P=0.037; odds ratio (OR)=1.089, 95% CI: 1.006-1.175). Moreover, rs9331888 G/G genotype ESCCs were more significantly common in patients with tumor size of >5 cm than T allele ESCC and in cases of poor differentiation and lower advanced pathological stage. In conclusion, polymorphism in rs9331888 C>G was observed to be associated with susceptibility of ESCC. Nevertheless, further investigation with a larger sample size is needed to support our results.

C1 [Li, Kun; Wang, Jian; Ma, Zhen-Bin] Binzhou Med Coll, Affiliated Hosp, Dept Gastroenterol, Binzhou 256600, Peoples R China.

[Guo, Guang-Hong] Binzhou Med Coll, Affiliated Hosp, Dept Gynaecol, Binzhou 256600, Peoples R China.

RP Ma, ZB (corresponding author), Binzhou Med Coll, Affiliated Hosp, Dept Gastroenterol, 661 Yellow River Second St, Binzhou 256600, Peoples R China.

EM mazhenbin@yeah.net

CR DANIK M, 1993, J COMP NEUROL, V334, P209, DOI 10.1002/cne.903340205

DESILVA HV, 1990, BIOCHEMISTRY-US, V29, P5380, DOI 10.1021/bi00474a025

Edge S., 2010, AM JOINT COMMITTEE C, VSeventh

Guerreiro RJ, 2010, PLOS ONE, V5, DOI 10.1371/journal.pone.0009510

He LR, 2009, CANCER SCI, V100, P2354, DOI 10.1111/j.1349-7006.2009.01349.x

Jones SE, 2002, INT J BIOCHEM CELL B, V34, P427, DOI 10.1016/S1357-2725(01)00155-8

July LV, 2004, MOL CANCER THER, V3, P223

Kuot A, 2012, EUR J HUM GENET, V20, P632, DOI 10.1038/ejhg.2011.248

Kurahashi T, 2005, BJU INT, V96, P895, DOI 10.1111/j.1464-410X.2005.05733.x

Lambert JC, 2009, NAT GENET, V41, P1094, DOI 10.1038/ng.439

Lau SH, 2006, ONCOGENE, V25, P1242, DOI 10.1038/sj.onc.1209141

Lieberman L, 2013, AM J PUBLIC HEALTH, V103, P1942, DOI 10.2105/AJPH.2013.301284

Ma ZB, 2014, INT J MOL SCI, V15, P12688, DOI 10.3390/ijms150712688

Miyake H, 2005, ONCOL REP, V14, P1371

Miyake H, 2001, CLIN CANCER RES, V7, P4245

Moretti RM, 2007, CANCER RES, V67, P10325, DOI 10.1158/0008-5472.CAN-07-0516

Nazir M, 2011, ASIAN PAC J CANCER P, V12, P2767

ODA T, 1994, BIOCHEM BIOPH RES CO, V204, P1131, DOI 10.1006/bbrc.1994.2580

Pucci S, 2004, ONCOGENE, V23, P2298, DOI 10.1038/sj.onc.1207404

Redondo M, 2000, AM J PATHOL, V157, P393, DOI 10.1016/S0002-9440(10)64552-X

ROSENBERG ME, 1995, INT J BIOCHEM CELL B, V27, P633, DOI 10.1016/1357-2725(95)00027-M

Shannan B, 2006, CELL DEATH DIFFER, V13, P12, DOI 10.1038/sj.cdd.4401779

Shi H, 2012, BREAST CANCER RES TR, V131, P1039, DOI 10.1007/s10549-011-1826-2

Siegel R, 2013, CA-CANCER J CLIN, V63, P11, DOI 10.3322/caac.21166

Szymanski M, 2011, TRANSL PSYCHIAT, V1, DOI 10.1038/tp.2011.17

Thrift AP, 2012, INT J CANCER, V131, pE759, DOI 10.1002/ijc.27420

Torre LA, 2015, CA-CANCER J CLIN, V65, P87, DOI 10.3322/caac.21262

Trougakos IP, 2002, INT J BIOCHEM CELL B, V34, P1430, DOI 10.1016/S1357-2725(02)00041-9

Trougakos IP, 2004, CANCER RES, V64, P1834, DOI 10.1158/0008-5472.CAN-03-2664

Wang YF, 2013, EUR J MED RES, V18, DOI 10.1186/2047-783X-18-11

Watari H, 2008, GYNECOL ONCOL, V108, P527, DOI 10.1016/j.ygyno.2007.11.026

Wilson MR, 2000, TRENDS BIOCHEM SCI, V25, P95, DOI 10.1016/S0968-0004(99)01534-0

WONG P, 1993, J BIOL CHEM, V268, P5021

Xie D, 2005, CANCER-AM CANCER SOC, V103, P277, DOI 10.1002/cncr.20765

Xie D, 2005, WORLD J GASTROENTERO, V11, P3285, DOI 10.3748/wjg.v11.i21.3285

Yan H, 2002, SCIENCE, V297, P1143, DOI 10.1126/science.1072545

Zellweger T, 2002, CLIN CANCER RES, V8, P3276

Zhang XJ, 2013, CARCINOGENESIS, V34, P2309, DOI 10.1093/carcin/bgt213

NR 38

TC 1

Z9 1

U1 0

U2 0

PU E-CENTURY PUBLISHING CORP

PI MADISON

PA 40 WHITE OAKS LN, MADISON, WI 53711 USA

SN 1940-5901

J9 INT J CLIN EXP MED

JI Int. J. Clin. Exp. Med.

PY 2015

VL 8

IS 8

BP 14004

EP 14010

PG 7

WC Medicine, Research & Experimental

WE Science Citation Index Expanded (SCI-EXPANDED)

SC Research & Experimental Medicine

GA CW8TJ

UT WOS:000365271900261

PM 26550359

DA 2022-05-19

ER

PT J

AU Shin, WS

Park, MK

Kim, JH

Oh, SW

Jang, JY

Lee, H

Lee, ST

AF Shin, Won-Sik

Park, Mi-Kyung

Kim, Jae Hoon

Oh, Si Won

Jang, Ji-Yun

Lee, Ho

Lee, Seung-Taek

TI PTK7, a Catalytically Inactive Receptor Tyrosine Kinase, Increases

Oncogenic Phenotypes in Xenograft Tumors of Esophageal Squamous Cell

Carcinoma KYSE-30 Cells

SO INTERNATIONAL JOURNAL OF MOLECULAR SCIENCES

LA English

DT Article

DE esophageal squamous cell carcinoma; KYSE-30 cells; oncogene; PTK7;

receptor protein tyrosine kinase

ID PROTEIN; MIGRATION; EXPRESSION; PHOSPHORYLATION; PROLIFERATION;

LANDSCAPE; INVASION; POLARITY; PATHWAY; LINES

AB Protein tyrosine kinase 7 (PTK7), a catalytically defective receptor protein tyrosine kinase, is upregulated in tumor tissues and cell lines of esophageal squamous cell carcinoma (ESCC). We showed that PTK7 plays an oncogenic role in various ESCC cell lines. However, its role as an oncogene has not been demonstrated in vivo. Here, we examined the influence of PTK7 on the tumorigenic potential of ESCC KYSE-30 cells, which are known to establish xenograft tumors. Overexpression of PTK7 enhanced the proliferation, adhesion, wound healing, and migration of KYSE-30 cells, and these effects were reversed by the knockdown of PTK7. PTK7 overexpression and knockdown, respectively, increased and decreased the tyrosine phosphorylation of cellular proteins and the phosphorylation of ERK, AKT, and FAK, which are important for cell proliferation, survival, adhesion, and migration. Additionally, PTK7 overexpression and silencing, respectively, increased and decreased the weight, volume, and number of Ki-67-positive proliferating cells in xenograft tumors of KYSE-30 cells. Therefore, we propose that PTK7 plays an important role in the tumorigenesis of ESCC cells in vivo and is a potential therapeutic target for ESCC.

C1 [Shin, Won-Sik; Kim, Jae Hoon; Oh, Si Won; Lee, Seung-Taek] Yonsei Univ, Coll Life Sci & Biotechnol, Dept Biochem, Seoul 03722, South Korea.

[Park, Mi-Kyung; Jang, Ji-Yun; Lee, Ho] Natl Canc Ctr, Grad Sch Canc Sci & Policy, Dept Canc Biomed Sci, Goyang 10408, South Korea.

[Jang, Ji-Yun] Dongguk Univ, Coll Pharm, Seoul 04620, South Korea.

RP Lee, ST (corresponding author), Yonsei Univ, Coll Life Sci & Biotechnol, Dept Biochem, Seoul 03722, South Korea.; Lee, H (corresponding author), Natl Canc Ctr, Grad Sch Canc Sci & Policy, Dept Canc Biomed Sci, Goyang 10408, South Korea.

EM amgod0306@hanmail.net; mkpark@ncc.re.kr; ebahspa@yonsei.ac.kr;

dhtldnjs1993@naver.com; yun9230@ncc.re.kr; ho25lee@ncc.re.kr;

stlee@yonsei.ac.kr

OI Lee, Ho/0000-0001-5573-742X; Shin, Won-Sik/0000-0001-9432-1320; Lee,

Seung-Taek/0000-0001-7300-9784

FU National Cancer Center of Korea [NCC-2010271, 2110150]; National

Research Foundation of Korea [2019M3A9A8065054]; Korea Drug Development

Fund - Ministry of Science and ICT, [HN21C1214000021]; Korea Drug

Development Fund - Ministry of Trade, Industry, and Energy

[HN21C1214000021]; Korea Drug Development Fund - Ministry of Health and

Welfare of Korea [HN21C1214000021]

FX This work was supported by grants from the National Cancer Center of

Korea (NCC-2010271 and 2110150 to H.L.), the National Research

Foundation of Korea (2019M3A9A8065054 to S.-T.L.), and the Korea Drug

Development Fund funded by the Ministry of Science and ICT, Ministry of

Trade, Industry, and Energy, and Ministry of Health and Welfare of Korea

(HN21C1214000021 to S.-T.L.).

CR Abedi-Ardekani B, 2012, BMC CANCER, V12, DOI 10.1186/1471-2407-12-602

Barnas C, 1997, INT J CANCER, V71, P79, DOI 10.1002/(SICI)1097-0215(19970328)71:1<79::AID-IJC14>3.0.CO;2-4

Bray F, 2018, CA-CANCER J CLIN, V68, P394, DOI 10.3322/caac.21492

Chang D, 2007, DIS ESOPHAGUS, V20, P491, DOI 10.1111/j.1442-2050.2007.00695.x

Cui NP, 2021, FRONT ONCOL, V11, DOI 10.3389/fonc.2021.699889

Duan F, 2020, EUR REV MED PHARMACO, V24, P6809, DOI 10.26355/eurrev_202006_21670

Dutton SJ, 2014, LANCET ONCOL, V15, P894, DOI 10.1016/S1470-2045(14)70024-5

Gao YB, 2014, NAT GENET, V46, P1097, DOI 10.1038/ng.3076

Guo YJ, 2020, EXP THER MED, V19, P1997, DOI 10.3892/etm.2020.8454

Hanawa M, 2006, INT J CANCER, V118, P1173, DOI 10.1002/ijc.21454

Islami F, 2011, INT J CANCER, V129, P2473, DOI 10.1002/ijc.25885

Jin XY, 2021, EXP THER MED, V22, DOI 10.3892/etm.2021.10806

Kamangar F, 2009, GASTROENTEROL CLIN N, V38, P27, DOI 10.1016/j.gtc.2009.01.004

Kanteti R, 2016, ONCOTARGET, V7, P31586, DOI 10.18632/oncotarget.8040

Kashyap MK, 2018, MOL CANCER, V17, DOI 10.1186/s12943-018-0790-4

Kim JH, 2014, ONCOL REP, V31, P2708, DOI 10.3892/or.2014.3164

Kodama T, 2020, LAB INVEST, V100, P1140, DOI 10.1038/s41374-020-0441-4

Lagergren J, 1999, NEW ENGL J MED, V340, P825, DOI 10.1056/NEJM199903183401101

Lee NP, 2018, J BIOMED SCI, V25, DOI 10.1186/s12929-018-0468-7

Lee YH, 2019, INT J MOL SCI, V20, DOI 10.3390/ijms20112734

Lhoumeau AC, 2015, PLOS ONE, V10, DOI 10.1371/journal.pone.0123768

Li RL, 2020, AM J TRANSL RES, V12, P1553

Lin DC, 2014, NAT GENET, V46, P467, DOI 10.1038/ng.2935

Liu K, 2017, WORLD J SURG ONCOL, V15, DOI 10.1186/s12957-017-1172-x

Liu M, 2016, CANCER LETT, V377, P97, DOI 10.1016/j.canlet.2016.04.034

Lu XW, 2004, NATURE, V430, P93, DOI 10.1038/nature02677

Luo H, 2018, ONCOL REP, V39, P2270, DOI 10.3892/or.2018.6304

Luo LL, 2015, CHIN J CANCER, V34, DOI 10.1186/s40880-015-0046-2

Martinez S, 2015, J BIOL CHEM, V290, P30562, DOI 10.1074/jbc.M115.697615

Meng L, 2010, PLOS ONE, V5, DOI 10.1371/journal.pone.0014018

MOSSIE K, 1995, ONCOGENE, V11, P2179

Na HW, 2012, J BIOL CHEM, V287, P25001, DOI 10.1074/jbc.M112.348904

NISHIHIRA T, 1993, J CANCER RES CLIN, V119, P441, DOI 10.1007/BF01215923

Park SK, 1996, J BIOCHEM, V119, P235

Peradziryi H, 2011, EMBO J, V30, P3729, DOI 10.1038/emboj.2011.236

Prebet T, 2010, BLOOD, V116, P2315, DOI 10.1182/blood-2010-01-262352

Puppo F, 2011, EMBO REP, V12, P43, DOI 10.1038/embor.2010.185

Sawada G, 2016, GASTROENTEROLOGY, V150, P1171, DOI 10.1053/j.gastro.2016.01.035

Shigaki H, 2013, CLIN CANCER RES, V19, P2451, DOI 10.1158/1078-0432.CCR-12-3559

SHIMADA Y, 1992, CANCER, V69, P277, DOI 10.1002/1097-0142(19920115)69:2<277::AID-CNCR2820690202>3.0.CO;2-C

Shin WS, 2008, BIOCHEM BIOPH RES CO, V371, P793, DOI 10.1016/j.bbrc.2008.04.168

Shin WS, 2020, CANCER SCI, V111, P3292, DOI 10.1111/cas.14568

Shin WS, 2019, FASEB J, V33, P12960, DOI 10.1096/fj.201900932R

Shin WS, 2018, SCI REP-UK, V8, DOI 10.1038/s41598-018-26957-6

Shin WS, 2017, J CELL BIOCHEM, V118, P2887, DOI 10.1002/jcb.25939

Shin WS, 2016, ONCOTARGET, V7, P73242, DOI 10.18632/oncotarget.12303

Shin WS, 2013, CANCER SCI, V104, P1120, DOI 10.1111/cas.12194

Song S, 2014, THORAC CANCER, V5, P417, DOI 10.1111/1759-7714.12112

Tanaka H, 1996, INT J CANCER, V65, P372, DOI 10.1002/(SICI)1097-0215(19960126)65:3<372::AID-IJC16>3.3.CO;2-3

Testa Ugo, 2017, Medicines (Basel), V4, DOI 10.3390/medicines4030067

Wang HY, 2014, INT J CLIN EXP PATHO, V7, P7881

Wang X, 2016, ONCOTARGET, V7, P24744, DOI 10.18632/oncotarget.8271

Yang J, 2019, MOL CANCER, V18, DOI 10.1186/s12943-019-0954-x

Yang Y, 2020, MOL THER-NUCL ACIDS, V22, P846, DOI 10.1016/j.omtn.2020.09.012

Yoshioka M, 2017, J EXP CLIN CANC RES, V36, DOI 10.1186/s13046-017-0572-7

Zhang F, 2020, J CANCER, V11, P414, DOI 10.7150/jca.31427

Zhang LY, 2021, MOL CARCINOGEN, V60, P113, DOI 10.1002/mc.23273

Zhao L, 2012, J TRANSL MED, V10, DOI 10.1186/1479-5876-10-249

Zhu HT, 2018, J THORAC DIS, V10, P5328, DOI 10.21037/jtd.2018.09.18

Zou RC, 2019, MED SCI MONITOR, V25, P8618, DOI 10.12659/MSM.917142

NR 60

TC 0

Z9 0

U1 0

U2 0

PU MDPI

PI BASEL

PA ST ALBAN-ANLAGE 66, CH-4052 BASEL, SWITZERLAND

EI 1422-0067

J9 INT J MOL SCI

JI Int. J. Mol. Sci.

PD FEB

PY 2022

VL 23

IS 4

AR 2391

DI 10.3390/ijms23042391

PG 14

WC Biochemistry & Molecular Biology; Chemistry, Multidisciplinary

WE Science Citation Index Expanded (SCI-EXPANDED)

SC Biochemistry & Molecular Biology; Chemistry

GA ZK5NG

UT WOS:000763034200001

PM 35216506

OA gold, Green Submitted, Green Published

DA 2022-05-19

ER

PT J

AU Zhang, Z

He, QS

Fu, S

Zheng, Z

AF Zhang, Zhe

He, Qinsi

Fu, Si

Zheng, Zhi

TI Estrogen Receptors in Regulating Cell Proliferation of Esophageal

Squamous Cell Carcinoma: Involvement of Intracellular Ca2+ Signaling

SO PATHOLOGY & ONCOLOGY RESEARCH

LA English

DT Article

DE Estrogen receptor; Esophageal squamous cell carcinoma; Gender

difference; Cell proliferation; Ca2+ signaling

ID BREAST-CANCER; CALCIUM-CHANNELS; ADENOCARCINOMA; EXPRESSION; ALPHA;

TARGET; TRPV6; BETA

AB Esophageal cancer is a deadly disease in the esophagus with a poor prognosis. Over 90 % of esophageal cancer is esophageal squamous cell carcinoma (ESCC) and its pathogenic mechanisms remain unclear. Epidemiology study found a strong gender difference with a sex ratio of 8-9:1 in favor of males, but the molecular mechanisms for so striking gender difference are poorly understood so far. In the present study, we demonstrated the expression of estrogen receptors in human ESCC cells. 17 beta-E2 but not 17 alpha-E2 was found to dose-dependently suppress the cell proliferation of human ESCC cells, which was attenuated by estrogen receptor antagonist ICI1 82,780. Furthermore, 17 beta -E2 but not 17 alpha-E2 10 nM markedly induced both intracellular Ca2+ release and extracellular Ca2+ entry into ESCC cells, which was again attenuated by estrogen receptor antagonist ICI182,780. Taken together, our data clearly demonstrate that estrogen exerts anti-proliferative action on human ESCC cells likely through estrogen receptor-Ca2+ signaling pathway, which may provide a reasonable explanation on the striking male predominance of ESCC.

C1 [Zhang, Zhe; Fu, Si] China Japan Friendship Hosp, Dept Chinese Med Gastrointestinal, 2 Yinghua Dongjie, Beijing 100029, Peoples R China.

[He, Qinsi; Zheng, Zhi] Nanchang Univ, Medicl Coll, Nanchang 330029, Jiangxi, Peoples R China.

[He, Qinsi; Zheng, Zhi] Jiangxi Canc Hosp, Jiangxi Prov Key Lab Oncol Translat Med, 519 Beijing East Rd, Nanchang 330029, Jiangxi, Peoples R China.

RP Zheng, Z (corresponding author), Nanchang Univ, Medicl Coll, Nanchang 330029, Jiangxi, Peoples R China.; Zheng, Z (corresponding author), Jiangxi Canc Hosp, Jiangxi Prov Key Lab Oncol Translat Med, 519 Beijing East Rd, Nanchang 330029, Jiangxi, Peoples R China.

EM zheng_sheva@hotmail.com

FU National Natural Science Foundation of China [81260592]

FX This work was supported by National Natural Science Foundation of China

(No. 81260592). The funding institutes had no role in study design, data

collection and analysis, decision to publish, or preparation of the

manuscript.

CR Blot WJ, 1999, SEMIN ONCOL, V26, P2

Bodelon C, 2011, CANCER PREV RES, V4, P840, DOI 10.1158/1940-6207.CAPR-10-0389

Boros S, 2009, PFLUG ARCH EUR J PHY, V458, P99, DOI 10.1007/s00424-008-0602-6

Freedman ND, 2010, CANCER-AM CANCER SOC, V116, P1572, DOI 10.1002/cncr.24880

Guo ZY, 2002, J BIOL CHEM, V277, P7044, DOI 10.1074/jbc.M109808200

HAZELTON B, 1979, J CELL BIOL, V83, P487, DOI 10.1083/jcb.83.2.487

Hogan AM, 2009, INT J COLORECTAL DIS, V24, P1367, DOI 10.1007/s00384-009-0785-0

Jiang HP, 2008, CHINESE MED J-PEKING, V121, P1025, DOI 10.1097/00029330-200806010-00012

Kahl CR, 2003, ENDOCR REV, V24, P719, DOI 10.1210/er.2003-0008

Kazerounian S, 2005, J PHARMACOL EXP THER, V314, P1013, DOI 10.1124/jpet.105.089052

Levin ER, 1999, TRENDS ENDOCRIN MET, V10, P374, DOI 10.1016/S1043-2760(99)00192-7

Levin ER, 2002, STEROIDS, V67, P471, DOI 10.1016/S0039-128X(01)00179-9

Liao WX, 2005, BIOL REPROD, V72, P530, DOI 10.1095/biolreprod.104.035949

Litle VR, 2011, SEMIN THORAC CARDIOV, V23, P131, DOI 10.1053/j.semtcvs.2011.08.008

LL Y, 2009, FERTIL STERIL, V91, P611

Monteith GR, 2007, NAT REV CANCER, V7, P519, DOI 10.1038/nrc2171

Monteith GR, 2012, J BIOL CHEM, V287, P31666, DOI 10.1074/jbc.R112.343061

Notarnicola M, 2008, SCAND J GASTROENTERO, V43, P66, DOI 10.1080/00365520701559011

PANDIELLA A, 1989, J BIOL CHEM, V264, P12914

Peters AA, 2012, MOL CANCER THER, V11, P2158, DOI 10.1158/1535-7163.MCT-11-0965

Pigozzi D, 2006, CELL CALCIUM, V39, P401, DOI 10.1016/j.ceca.2006.01.003

Prossnitz ER, 2011, NAT REV ENDOCRINOL, V7, P715, DOI 10.1038/nrendo.2011.122

Renoir JM, 2013, BIOCHEM PHARMACOL, V85, P449, DOI 10.1016/j.bcp.2012.10.018

Sarkar SN, 2008, P NATL ACAD SCI USA, V105, P15148, DOI 10.1073/pnas.0802379105

Sergeev IN, 2012, ANTICANCER RES, V32, P299

Wang JY, 2000, AM J PHYSIOL-CELL PH, V278, pC303, DOI 10.1152/ajpcell.2000.278.2.C303

Wang QM, 2011, MED ONCOL, V28, P188, DOI 10.1007/s12032-010-9457-8

Wang ZM, 2006, BMC CANCER, V6, DOI 10.1186/1471-2407-6-287

Yang HQ, 2012, WORLD J GASTROENTERO, V18, P393, DOI 10.3748/wjg.v18.i5.393

Zuguchi M, 2012, CANCER SCI, V103, P1348, DOI 10.1111/j.1349-7006.2012.02288.x

NR 30

TC 5

Z9 5

U1 1

U2 2

PU SPRINGER

PI DORDRECHT

PA VAN GODEWIJCKSTRAAT 30, 3311 GZ DORDRECHT, NETHERLANDS

SN 1219-4956

EI 1532-2807

J9 PATHOL ONCOL RES

JI Pathol. Oncol. Res.

PD APR

PY 2017

VL 23

IS 2

BP 329

EP 334

DI 10.1007/s12253-016-0105-2

PG 6

WC Oncology; Pathology

WE Science Citation Index Expanded (SCI-EXPANDED)

SC Oncology; Pathology

GA ER7NQ

UT WOS:000399000200013

PM 27595756

DA 2022-05-19

ER

PT J

AU Cai, QY

Liang, GY

Zheng, YF

Tan, QY

Wang, RW

Li, K

AF Cai, Qing Yong

Liang, Gui You

Zheng, Yi Feng

Tan, Qun You

Wang, Ru Wen

Li, Kun

TI Sox3 silencing inhibits metastasis and growth of esophageal squamous

cell carcinoma cell via down-regulating GSK-3 beta

SO INTERNATIONAL JOURNAL OF CLINICAL AND EXPERIMENTAL PATHOLOGY

LA English

DT Article

DE Esophageal squamous cell carcinoma; migration; invasion; Sox3; GSK-3

beta

ID IN-VITRO; EXPRESSION; MIGRATION; PROMOTES; CANCER

AB The early stages of localized esophageal squamous cell carcinoma (ESCC) can be effectively treated by surgical resection of the early esophageal cancer, but there is still no effective treatment once it has been metastasis to distant tissues. Although a number of both metastasis promoting and metastasis suppressor genes have been reported, the underlying molecular mechanisms responsible for ESCC metastatic cascade are still not fully understood. Therefore, defining the potential molecules that govern ESCC metastasis may aid the development of new therapeutic strategies for combating ESCC. In the present study, we found Sox3 is involved in the metastasis of ESCC cells and demonstrated that Sox3 (Sex determining region Y-box 3) disruption impaired ESCC cells migration and invasion. The requirement of Sox3 in the metastasis of ESCC cells was further confirmed by gene silencing in vitro. Moreover, down-regulation of Sox3 expression strikingly inhibited ESCC cellular growth both in vitro and vivo. Finally, we found that Sox3 promotes ESCC migration and invasion through the GSK-3 beta signaling pathway. To conclude, our findings suggest a novel mechanism underlying the metastasis of ESCC cells which might serve as a new intervention target for the treatment of ESCC.

C1 [Cai, Qing Yong; Zheng, Yi Feng; Tan, Qun You; Wang, Ru Wen; Li, Kun] Third Mil Med Univ, Inst Surg Res, Daping Hosp, Dept Thorac Surg, Chongqing, Peoples R China.

[Cai, Qing Yong; Liang, Gui You] Zunyi Med Coll, Affiliated Hosp, Dept Thorac & Cardiovasc Surg, Zunyi, Guizhou, Peoples R China.

RP Wang, RW; Li, K (corresponding author), Third Mil Med Univ, Inst Surg Res, Daping Hosp, Dept Thorac Surg,Otsubo Yangtze River Branch 10, Chongqing 400042, Peoples R China.

EM wangthebest2015@sina.com; likunbiomedi-cine@sina.com

FU National Natural Sciences Foundation of China [[2013] 81301730]

FX This project was supported by a grant from the National Natural Sciences

Foundation of China (No. [2013] 81301730).

CR Balakrishnan M, 2013, PLOS ONE, V8, DOI 10.1371/journal.pone.0074163

Bi HX, 2015, CHINESE MED J-PEKING, V128, P373, DOI 10.4103/0366-6999.150109

Chang L, 2013, MOL BIOL REP, V40, P669, DOI 10.1007/s11033-012-2106-x

Chen MQ, 2014, J CHIN MED ASSOC, V77, P562, DOI 10.1016/j.jcma.2014.05.014

Ito G, 2015, BIOCHEM BIOPH RES CO, V456, P792, DOI 10.1016/j.bbrc.2014.12.037

Kimchi ET, 2005, CANCER RES, V65, P3146, DOI 10.1158/0008-5472.CAN-04-2490

Lai Dengpan, 2014, Nan Fang Yi Ke Da Xue Xue Bao, V34, P1738

Li K, 2013, ANN SURG ONCOL, V20, pS459, DOI 10.1245/s10434-012-2792-6

Li Q, 2015, LIFE SCI, V127, P106, DOI 10.1016/j.lfs.2015.01.028

Luo KJ, 2012, ANN THORAC SURG, V93, P1682, DOI 10.1016/j.athoracsur.2012.01.102

Min AJ, 2015, J BIOCHEM MOL TOXIC, V29, P70, DOI 10.1002/jbt.21669

Oh SJ, 2014, TOXICOL LETT, V225, P422, DOI 10.1016/j.toxlet.2014.01.015

Ortega CE, 2014, PLOS ONE, V9, DOI 10.1371/journal.pone.0115609

Pan YQ, 2015, INT J CLIN EXP PATHO, V8, P384

STAMBOLIC V, 1994, BIOCHEM J, V303, P701, DOI 10.1042/bj3030701

Sun LL, 2015, INT J CANCER, V136, pE569, DOI 10.1002/ijc.29211

Takeshita N, 2013, BRIT J CANCER, V108, P644, DOI 10.1038/bjc.2013.8

Wu CY, 2014, PLOS ONE, V9, DOI 10.1371/journal.pone.0092292

Xia Y, 2000, CANCER RES, V60, P6303

Yamada S, 2012, J GASTROEN HEPATOL, V27, P1816, DOI 10.1111/j.1440-1746.2012.07275.x

Zeng J, 2014, PLOS ONE, V9, DOI 10.1371/journal.pone.0091231

Zeng R, 2013, CHINESE J CANCER RES, V25, P637, DOI 10.3978/j.issn.1000-9604.2013.11.03

NR 22

TC 4

Z9 4

U1 1

U2 6

PU E-CENTURY PUBLISHING CORP

PI MADISON

PA 40 WHITE OAKS LN, MADISON, WI 53711 USA

SN 1936-2625

J9 INT J CLIN EXP PATHO

JI Int. J. Clin. Exp. Pathol.

PY 2016

VL 9

IS 3

BP 2939

EP 2949

PG 11

WC Oncology; Pathology

WE Science Citation Index Expanded (SCI-EXPANDED)

SC Oncology; Pathology

GA DO2FA

UT WOS:000377594100019

DA 2022-05-19

ER

PT J

AU Wang, S

Wang, Z

Yang, Y

Shi, M

Sun, ZG

AF Wang, Shuai

Wang, Zhou

Yang, Yu

Shi, Mo

Sun, Zhenguo

TI Overexpression of Ku80 correlates with aggressive clinicopathological

features and adverse prognosis in esophageal squamous cell carcinoma

SO ONCOLOGY LETTERS

LA English

DT Article

DE esophageal squamous cell carcinoma; Ku80; prognosis; pathology; clinical

relevance

ID DEPENDENT PROTEIN-KINASE; NF-KAPPA-B; CANCER INCIDENCE; EXPRESSION;

BINDING; PROLIFERATION; SURVIVAL; ER

AB Ku80, a subunit of the heterodymeric Ku protein, is clearly implicated in nonhomologous end joining DNA repair, chemoresistance and radioresistance in malignant tumors. In the present study, the clinicopathological significance of Ku80 in esophageal squamous cell carcinoma (ESCC) was investigated. The expression levels of Ku80 were determined by reverse transcription-quantitative polymerase chain reaction and immunohistochemistry in ESCC specimens and normal esophageal mucosa. The mRNA and protein levels of Ku80 were significantly higher in ESCC tissues than in normal esophageal mucosa, and were significantly associated with tumor differentiation, local invasion, lymph node metastasis and tumor-node-metastasis (TNM) stage. However, overexpression of Ku80 mRNA and protein levels were not significantly correlated with age, gender, tumor site or tumor size. Cox proportional hazards regression model demonstrated that tumor local invasion, lymph node metastasis, TNM stage and Ku80 mRNA and protein levels were independent risk factors indicating the overall survival of patients with ESCC. The present study demonstrated that aberrant Ku80 overexpression is observed in ESCC. In addition, high expression levels of Ku80 are associated with adverse clinicopathological features and unfavorable prognosis in ESCC patients.

C1 [Wang, Shuai; Wang, Zhou; Shi, Mo; Sun, Zhenguo] Shandong Univ, Prov Hosp, Dept Thorac Surg, Jinan 250021, Shandong, Peoples R China.

[Yang, Yu] Shandong Univ, Prov Hosp, Dept Pathol, Jinan 250021, Shandong, Peoples R China.

RP Wang, Z (corresponding author), Shandong Univ, Prov Hosp, Dept Thorac Surg, 324 Jingwu Rd, Jinan 250021, Shandong, Peoples R China.

EM wz620226@hotmail.com

RI guo, sun/C-4291-2014

OI Wang, Shuai/0000-0001-8503-9024

CR Aaltonen L., 2000, WHO CLASSIFICATION T, P237

Alshareeda AT, 2013, BREAST CANCER RES TR, V139, P301, DOI 10.1007/s10549-013-2542-x

D'Annoville T, 2013, EUR J CARDIO-THORAC, V44, pE207, DOI 10.1093/ejcts/ezt332

Ferlay J, 2010, INT J CANCER, V127, P2893, DOI 10.1002/ijc.25516

Fink LS, 2010, CELL CYCLE, V9, P3798, DOI 10.4161/cc.9.18.13129

FINNIE NJ, 1995, P NATL ACAD SCI USA, V92, P320, DOI 10.1073/pnas.92.1.320

Grabsch H, 2006, CLIN CANCER RES, V12, P1494, DOI 10.1158/1078-0432.CCR-05-2105

Groselj B, 2013, RADIOTHER ONCOL, V108, P429, DOI 10.1016/j.radonc.2013.06.021

Gullo C, 2006, BBA-REV CANCER, V1765, P223, DOI 10.1016/j.bbcan.2006.01.001

Hosoi Y, 2004, INT J ONCOL, V25, P461

Kamangar F, 2009, GASTROENTEROL CLIN N, V38, P27, DOI 10.1016/j.gtc.2009.01.004

Kamangar F, 2006, J CLIN ONCOL, V24, P2137, DOI 10.1200/JCO.2005.05.2308

Koike M, 2001, J BIOL CHEM, V276, P11167, DOI 10.1074/jbc.M010902200

Kruer TL, 2013, CLIN BIOCHEM, V46, P1739, DOI 10.1016/j.clinbiochem.2013.07.005

Leifler KS, 2010, INT J ONCOL, V37, P1547, DOI 10.3892/ijo_00000808

Lieber MR, 2010, SUBCELL BIOCHEM, V50, P279, DOI 10.1007/978-90-481-3471-7_14

Lim JW, 2002, J BIOL CHEM, V277, P46093, DOI 10.1074/jbc.M206603200

LIU ES, 1991, NUCLEIC ACIDS RES, V19, P5425, DOI 10.1093/nar/19.19.5425

Ma QS, 2012, J EXP CLIN CANC RES, V31, DOI 10.1186/1756-9966-31-99

Rasool S, 2013, ONKOLOGIE, V36, P213, DOI 10.1159/000349960

Shibata-Kobayashi S, 2013, ONCOL LETT, V5, P903, DOI 10.3892/ol.2013.1130

Shrivastav M, 2009, DNA REPAIR, V8, P920, DOI 10.1016/j.dnarep.2009.05.006

Sobin LH., 2009, TNM CLASSIFICATION M, V7

Song YM, 2012, ANN SURG ONCOL, V19, P3606, DOI 10.1245/s10434-012-2419-y

Tonotsuka N, 2006, INT J MOL MED, V18, P441

Trivers KF, 2008, INT J CANCER, V123, P1422, DOI 10.1002/ijc.23691

Wang S, 2014, MOL MED REP, V9, P1259, DOI 10.3892/mmr.2014.1945

Yang QS, 2008, J RADIAT RES, V49, P399, DOI 10.1269/jrr.07096

NR 28

TC 6

Z9 7

U1 0

U2 2

PU SPANDIDOS PUBL LTD

PI ATHENS

PA POB 18179, ATHENS, 116 10, GREECE

SN 1792-1074

EI 1792-1082

J9 ONCOL LETT

JI Oncol. Lett.

PD NOV

PY 2015

VL 10

IS 5

BP 2705

EP 2712

DI 10.3892/ol.2015.3635

PG 8

WC Oncology

WE Science Citation Index Expanded (SCI-EXPANDED)

SC Oncology

GA CV5QB

UT WOS:000364324800005

PM 26722230

OA gold, Green Submitted, Green Published

DA 2022-05-19

ER

PT J

AU Duan, LL

Ma, JJ

Yang, WL

Cao, L

Wang, XQ

Niu, LR

Li, YD

Zhou, W

Zhang, YJ

Liu, JQ

Zhang, HW

Zhao, QC

Hong, L

Fan, DM

AF Duan, Lili

Ma, Jiaojiao

Yang, Wanli

Cao, Lu

Wang, Xiaoqian

Niu, Liaoran

Li, Yiding

Zhou, Wei

Zhang, Yujie

Liu, Jinqiang

Zhang, Hongwei

Zhao, Qingchuan

Hong, Liu

Fan, Daiming

TI EI24 Inhibits Cell Proliferation and Drug Resistance of Esophageal

Squamous Cell Carcinoma

SO FRONTIERS IN ONCOLOGY

LA English

DT Article

DE EI24; esophageal squamous cell carcinoma; proliferation; drug

resistance; prognosis

ID MOLECULAR-MECHANISMS; CONFERS RESISTANCE; CANCER; AUTOPHAGY; REGION;

CYCLE

AB Drug resistance, whether intrinsic or acquired, often leads to treatment failure in esophageal squamous cell carcinoma (ESCC). Clarifying the mechanism of drug resistance in ESCC has great significance for reversing drug resistance, as well as improving the prognosis of patients. Previously, we demonstrated that etoposide-induced 2.4-kb mRNA (EI24) is the target of miR-483-3p, which promoted the growth, migration, and drug resistance in ESCC, suggesting that EI24 participates in repressing the tumorigenesis and progression of ESCC. Here, we observed that EI24 was remarkably decreased in ESCC tissues. Moreover, its expression was directly linked to the prognosis of patients. We then confirmed that the forced overexpression of EI24 repressed cell growth and sensitized ESCC cells to chemotherapeutic agents, whereas EI24 silencing had the opposite effect. Furthermore, gene microarray and ingenuity pathway analysis (IPA) were performed to establish the potential mechanisms and indicated that EI24 exerts a tumor-suppressive role via suppressing the acute phase response signaling pathway or IL-1 signaling pathway in ESCC. Collectively, our data reveal that EI24 overexpression attenuates malignant phenotypes of ESCC and that it is a novel possible ESCC therapeutic target.

C1 [Duan, Lili; Yang, Wanli; Wang, Xiaoqian; Niu, Liaoran; Li, Yiding; Zhou, Wei; Zhang, Yujie; Liu, Jinqiang; Zhao, Qingchuan; Hong, Liu; Fan, Daiming] Air Force Mil Med Univ, Natl Clin Res Ctr Digest Dis, State Key Lab Canc Biol, Xian, Peoples R China.

[Duan, Lili; Yang, Wanli; Wang, Xiaoqian; Niu, Liaoran; Li, Yiding; Zhou, Wei; Zhang, Yujie; Liu, Jinqiang; Zhao, Qingchuan; Hong, Liu; Fan, Daiming] Air Force Mil Med Univ, Xijing Hosp Digest Dis, Xian, Peoples R China.

[Ma, Jiaojiao; Cao, Lu] Xi An Jiao Tong Univ, Affiliated Hosp 2, Dept Endocrinol, Xian, Peoples R China.

[Cao, Lu] Air Force Mil Med Univ, Dept Biomed Engn, Xian, Peoples R China.

[Zhang, Hongwei] Wuxi Mingci Cardiovasc Hosp, Dept Digest Dis, Wuxi, Jiangsu, Peoples R China.

RP Hong, L (corresponding author), Air Force Mil Med Univ, Natl Clin Res Ctr Digest Dis, State Key Lab Canc Biol, Xian, Peoples R China.; Hong, L (corresponding author), Air Force Mil Med Univ, Xijing Hosp Digest Dis, Xian, Peoples R China.

EM hongliufmmu@163.com

FU Scientific Foundation of Shaanxi Province [2019ZDLSF01-02-01]; State Key

Laboratory of Cancer Biology [CBSKL2014Z13]; National Clinical Research

Center for Digestive Diseases [2015BAI13B07]

FX This study was supported in part by grant from the Scientific Foundation

of Shaanxi Province (2019ZDLSF01-02-01), grant from the State Key

Laboratory of Cancer Biology (CBSKL2014Z13), and the National Clinical

Research Center for Digestive Diseases (2015BAI13B07). It was not

supported by any private or public company or organization.

CR Arnold M, 2020, GASTROENTEROLOGY, V159, P335, DOI 10.1053/j.gastro.2020.02.068

Arnold M, 2015, GUT, V64, P381, DOI 10.1136/gutjnl-2014-308124

Bray F, 2018, CA-CANCER J CLIN, V68, P394, DOI 10.3322/caac.21492

Cassetta L, 2018, NAT REV DRUG DISCOV, V17, P887, DOI 10.1038/nrd.2018.169

Chen NZ, 2019, PLOS ONE, V14, DOI 10.1371/journal.pone.0222035

Choi JM, 2015, LUNG CANCER, V90, P175, DOI 10.1016/j.lungcan.2015.08.019

Choi JM, 2013, ONCOTARGET, V4, P2383, DOI 10.18632/oncotarget.1434

Crago AM, 2012, CLIN CANCER RES, V18, P1334, DOI 10.1158/1078-0432.CCR-11-2820

Grivennikov SI, 2010, CELL, V140, P883, DOI 10.1016/j.cell.2010.01.025

Gu Z, 2000, CYTOGENET CELL GENET, V89, P230, DOI 10.1159/000015620

Gu ZM, 2000, MOL CELL BIOL, V20, P233, DOI 10.1128/MCB.20.1.233-241.2000

Holohan C, 2013, NAT REV CANCER, V13, P714, DOI 10.1038/nrc3599

Kaliszczak M, 2013, BRIT J CANCER, V109, P2356, DOI 10.1038/bjc.2013.584

Kartal-Yandim M, 2016, CRIT REV BIOTECHNOL, V36, P716, DOI 10.3109/07388551.2015.1015957

Kathawala RJ, 2015, DRUG RESIST UPDATE, V18, P1, DOI 10.1016/j.drup.2014.11.002

Kramer A, 2014, BIOINFORMATICS, V30, P523, DOI 10.1093/bioinformatics/btt703

KUSHNER I, 1988, METHOD ENZYMOL, V163, P373

Kuwano H, 1998, INT J CANCER, V78, P149, DOI 10.1002/(SICI)1097-0215(19981005)78:2<149::AID-IJC4>3.0.CO;2-Z

Li ZS, 2017, ONCOTARGET, V8, P19455, DOI 10.18632/oncotarget.14307

Lin CY, 2018, MOL ONCOL, V12, P756, DOI 10.1002/1878-0261.12196

Ma JJ, 2016, CELL BIOL INT, V40, P448, DOI 10.1002/cbin.10585

Massague J, 2004, NATURE, V432, P298, DOI 10.1038/nature03094

Mazumder D, 2011, INT J CANCER, V129, P1859, DOI 10.1002/ijc.25849

Merkow RP, 2014, JNCI-J NATL CANCER I, V106, DOI 10.1093/jnci/dju133

Mork CN, 2007, FEBS LETT, V581, P5440, DOI 10.1016/j.febslet.2007.10.046

Ozbabacan SEA, 2014, PLOS COMPUT BIOL, V10, DOI 10.1371/journal.pcbi.1003470

Reimann E, 2014, HUM GENOMICS, V8, DOI 10.1186/s40246-014-0020-0

Rustgi AK, 2014, NEW ENGL J MED, V371, P2499, DOI 10.1056/NEJMra1314530

Shi RZ, 2013, J CELL BIOCHEM, V114, P1890, DOI 10.1002/jcb.24533

Sinha S, 2011, MOL ONCOL, V5, P454, DOI 10.1016/j.molonc.2011.06.005

Sjoquist KM, 2011, LANCET ONCOL, V12, P681, DOI 10.1016/S1470-2045(11)70142-5

Vermeulen K, 2003, CELL PROLIFERAT, V36, P165, DOI 10.1046/j.1365-2184.2003.00267.x

Wang M, 2020, MOL CANCER RES, V18, P943, DOI 10.1158/1541-7786.MCR-19-1033

Xue JF, 2017, BIOMED PHARMACOTHER, V89, P1252, DOI 10.1016/j.biopha.2017.01.130

Yang WL, 2017, EXPERT REV GASTROENT, V11, P1151, DOI 10.1080/17474124.2017.1372189

Zang Y, 2018, GASTROENT RES PRACT, V2018, DOI [10.1155/2018/2626545, 10.1080/00207543.2018.1471238]

Zhao YG, 2012, J BIOL CHEM, V287, P42053, DOI 10.1074/jbc.M112.415968

Zheng GXY, 2011, PLOS GENET, V7, DOI 10.1371/journal.pgen.1002054

Zhou W, 2011, BRIT J CANCER, V104, P128, DOI 10.1038/sj.bjc.6606013

NR 39

TC 4

Z9 4

U1 2

U2 4

PU FRONTIERS MEDIA SA

PI LAUSANNE

PA AVENUE DU TRIBUNAL FEDERAL 34, LAUSANNE, CH-1015, SWITZERLAND

SN 2234-943X

J9 FRONT ONCOL

JI Front. Oncol.

PD AUG 21

PY 2020

VL 10

AR 1570

DI 10.3389/fonc.2020.01570

PG 18

WC Oncology

WE Science Citation Index Expanded (SCI-EXPANDED)

SC Oncology

GA NO5VH

UT WOS:000569558100001

PM 32974192

OA gold, Green Published

DA 2022-05-19

ER

PT J

AU Liang, BY

Wu, Y

Han, X

Zheng, XF

Zhan, QM

Tong, T

AF Liang, Baoyu

Wu, Yan

Han, Xu

Zheng, Xiaofei

Zhan, Qimin

Tong, Tong

TI MiR-205 inhibits the invasion and migration of esophageal squamous cell

carcinoma by modulating SMAD1 expression

SO CHINESE SCIENCE BULLETIN

LA English

DT Article

DE Esophageal squamous cell carcinoma; Invasion and migration; MiR-205;

SMAD1

ID CANCER-CELLS; PROSTATE-CANCER; ACTIVATION; MICRORNAS; PROLIFERATION;

HISTOLOGY

AB Esophageal squamous cell carcinoma (ESCC) is one of the most lethal cancers worldwide. In this study, we aimed to investigate the underlying mechanisms of metastasis inhibition by miR-205 in ESCC. In microRNA (miRNA) array and quantitative RT-PCR analyses, we found that the expression level of miR-205 was significantly lower in patients with lymph node metastasis compared with that in patients without lymph node metastasis. After transfection of miR-205 mimics or inhibitors into ESCC cell lines, a significant negative correlation was observed between the expression level of miR-205 and Smad1. In luciferase reporter assays, we revealed that miR-205 inhibited the expression of SMAD1 by targeting the 3' untranslated region (3'-UTR) of SMAD1 mRNA in ESCC cells. Furthermore, our results showed that miR-205 suppressed the invasion and migration of ESCC cells, whereas Smad1 increased their invasion and migration. Taken together, our study demonstrates that miR-205 functions as a suppressor of tumor metastasis by regulating SMAD1 expression through targeting the 3'-UTR of SMAD1 mRNA in ESCC. Therefore, miR-205 may be a potential therapeutic target for miRNA-based therapy of ESCC.

C1 [Liang, Baoyu; Wu, Yan; Han, Xu; Zhan, Qimin; Tong, Tong] Chinese Acad Med Sci, State Key Lab Mol Oncol, Canc Inst & Canc Hosp, Beijing 100021, Peoples R China.

[Liang, Baoyu; Wu, Yan; Han, Xu; Zhan, Qimin; Tong, Tong] Peking Union Med Coll, Beijing 100021, Peoples R China.

[Zheng, Xiaofei] Mil Med Sci, Beijing Inst Radiat Med, Beijing 100850, Peoples R China.

RP Zhan, QM (corresponding author), Chinese Acad Med Sci, State Key Lab Mol Oncol, Canc Inst & Canc Hosp, Beijing 100021, Peoples R China.

EM Zhanqimin@gmail.com; Tongt5@yahoo.com

FU National Basic Research Program [2009CB521801, 2010CB912801]

FX This work was supported by the National Basic Research Program

(2009CB521801 and 2010CB912801).

CR Bagnyukova T. V., 2006, Experimental Oncology, V28, P263

Bartel DP, 2004, CELL, V116, P281, DOI 10.1016/S0092-8674(04)00045-5

Bharathy S, 2008, CANCER RES, V68, P1656, DOI 10.1158/0008-5472.CAN-07-5089

Bhattacharya R, 2010, J ONCOL, V2010, DOI 10.1155/2010/856105

Fujishiro M, 2008, DIS ESOPHAGUS, V21, P181, DOI 10.1111/j.1442-2050.2007.00707.x

Hulf T, 2013, ONCOGENE, V32, P2891, DOI 10.1038/onc.2012.300

Jung S, 2010, STEM CELLS DEV, V19, P831, DOI 10.1089/scd.2009.0093

Karaayvaz M, 2012, PLOS ONE, V7, DOI 10.1371/journal.pone.0035158

Kimura S, 2010, ONCOL REP, V23, P1625, DOI 10.3892/or_00000804

LAW SYK, 1992, SURG GYNECOL OBSTET, V175, P107

Li B, 2007, CARCINOGENESIS, V28, P2313, DOI 10.1093/carcin/bgm152

LIU FS, 1993, INT J CANCER, V55, P577, DOI 10.1002/ijc.2910550410

Liu JJ, 2013, CHINESE J CANCER RES, V25, P46, DOI 10.3978/j.issn.1000-9604.2012.11.04

Matsushima K, 2011, J TRANSL MED, V9, DOI 10.1186/1479-5876-9-30

Okano Jun-ichi, 2003, Methods Mol Biol, V222, P131

Redova M, 2013, TUMOR BIOL, V34, P481, DOI 10.1007/s13277-012-0573-2

Shola DTN, 2012, ONCOGENE, V31, P2480, DOI 10.1038/onc.2011.422

NR 17

TC 6

Z9 6

U1 0

U2 15

PU SCIENCE PRESS

PI BEIJING

PA 16 DONGHUANGCHENGGEN NORTH ST, BEIJING 100717, PEOPLES R CHINA

SN 1001-6538

EI 1861-9541

J9 CHINESE SCI BULL

JI Chin. Sci. Bull.

PD JUL

PY 2014

VL 59

IS 19

BP 2232

EP 2239

DI 10.1007/s11434-014-0272-z

PG 8

WC Multidisciplinary Sciences

WE Science Citation Index Expanded (SCI-EXPANDED)

SC Science & Technology - Other Topics

GA AI8EF

UT WOS:000337139000003

DA 2022-05-19

ER

PT J

AU Zhang, JG

Yang, XJ

Wang, YC

Shi, H

Guan, CQ

Yao, L

Huang, XT

Ding, ZM

Huang, YJ

Wang, HJ

Cheng, C

AF Zhang, Jianguo

Yang, Xiaojing

Wang, Yuchan

Shi, Hui

Guan, Chengqi

Yao, Li

Huang, Xianting

Ding, Zongmei

Huang, Yuejiao

Wang, Huijie

Cheng, Chun

TI Low Expression of CyclinH and Cyclin-Dependent Kinase 7 Can Decrease the

Proliferation of Human Esophageal Squamous Cell Carcinoma

SO DIGESTIVE DISEASES AND SCIENCES

LA English

DT Article

DE Esophageal squamous cell carcinoma; CyclinH; CDK7; Tumor immunology;

Proliferation

ID SURVIVAL; CANCER; TUMOR; OVEREXPRESSION; RNA

AB Increased expression of cyclinH (CCNH) and cyclin-dependent kinase 7 (CDK7) has a relationship with poor prognosis in most human cancers.

Investigate the expression of CCNH and CDK7 in human esophageal squamous cell carcinoma (ESCC) and the effect of chemotherapy on their expression.

Western blotting and immunohistochemistry were used to measure the expression of CCNH and CDK7 proteins in ESCC and adjacent normal tissue in 98 patients. We use Cell Counting Kit-8 and cell flow to analyze the effects of cisplatin and interference of CCNH and CDK7 in cell cycle process.

Immunohistochemical analysis showed that CCNH and CDK7 expression were significantly associated with unfavorable clinicopathologic variables. CCNH and CDK7 protein levels were elevated in ESCC tissues in comparison with adjacent normal tissues. Survival analysis revealed that CCNH and CDK7 overexpression were significantly associated with overall survival (P < 0.001). Cisplatin or interference of CCNH or CDK7 led cells to grow slowly. Overexpression of CCNH and CDK7 in TE1 cells can lead to resistance to cisplatin.

We can conclude that CCNH and CDK7 may play an important role in the tumorigenesis and development of ESCC. CCNH and CDK7 expression affected the chemotherapy of tumor.

C1 [Zhang, Jianguo; Yang, Xiaojing; Wang, Yuchan; Shi, Hui; Guan, Chengqi; Yao, Li; Huang, Xianting; Ding, Zongmei; Huang, Yuejiao; Cheng, Chun] Nantong Univ, Dept Immunol, Coll Med, Nantong 226001, Jiangsu, Peoples R China.

[Wang, Huijie] Fudan Univ, Shanghai Canc Ctr, Dept Med Oncol, Shanghai 200032, Peoples R China.

RP Cheng, C (corresponding author), Nantong Univ, Dept Immunol, Coll Med, 19 Qi Xiu Rd, Nantong 226001, Jiangsu, Peoples R China.

EM wanghj98@hotmail.com; cheng_chun@yahoo.com.cn

FU National Basic Research Program of China (973 Program) [2011CB910604,

2012CB822104]; National Natural Science Foundation of China [81172879,

81201858]; Natural Scientific Foundation of Jiangsu Province

[BK2012231]; Priority Academic Program Development of Jiangsu Higher

Education Institutions (PAPD)

FX This work was supported by the National Basic Research Program of China

(973 Program, No. 2011CB910604, and No. 2012CB822104); the National

Natural Science Foundation of China (No. 81172879, No. 81201858);

Natural Scientific Foundation of Jiangsu Province Grant (No. BK2012231);

A Project Funded by the Priority Academic Program Development of Jiangsu

Higher Education Institutions (PAPD).

CR Ali S, 2007, GENE, V401, P38, DOI 10.1016/j.gene.2007.06.017

Bahmanyar S, 2007, GUT, V56, P464, DOI 10.1136/gut.2006.109082

Bartkova J, 1996, INT J CANCER, V66, P732, DOI 10.1002/(SICI)1097-0215(19960611)66:6<732::AID-IJC4>3.0.CO;2-0

Bauer S, 2007, ONCOGENE, V26, P7560, DOI 10.1038/sj.onc.1210558

Eloubeidi MA, 2002, CANCER-AM CANCER SOC, V95, P1434, DOI 10.1002/cncr.10868

Enzinger PC, 2003, NEW ENGL J MED, V349, P2241, DOI 10.1056/NEJMra035010

Fei M, 2009, MED ONCOL, V26, P178, DOI 10.1007/s12032-008-9105-8

FISHER RP, 1994, CELL, V78, P713, DOI 10.1016/0092-8674(94)90535-5

Glade M J, 1999, Nutrition, V15, P523

Gopisetty G, 2006, MOL IMMUNOL, V43, P1729, DOI 10.1016/j.molimm.2005.11.010

Guerenstein PG, 2004, NATURWISSENSCHAFTEN, V91, P329, DOI 10.1007/s00114-004-0532-x

Johnell O, 2004, OSTEOPOROSIS INT, V15, P38, DOI 10.1007/s00198-003-1490-4

Lepage C, 2008, AM J GASTROENTEROL, V103, P2694, DOI 10.1111/j.1572-0241.2008.02191.x

Liu QY, 2007, CELL RES, V17, P163, DOI 10.1038/sj.cr.7310144

Liu YH, 2010, J NEUROTRAUM, V27, P361, DOI 10.1089/neu.2008.0581

Lolli G, 2005, CELL CYCLE, V4, P572, DOI 10.4161/cc.4.4.1607

Luo KJ, 2011, J SURG ONCOL, V104, P236, DOI 10.1002/jso.21921

Medvec B R, 1988, Semin Oncol Nurs, V4, P246, DOI 10.1016/0749-2081(88)90076-9

Mohebbi M, 2008, BMC CANCER, V8, DOI 10.1186/1471-2407-8-137

Mouriaux F, 1998, INVEST OPHTH VIS SCI, V39, P876

Murakami K, 2011, ESOPHAGUS-TOKYO, V8, P209, DOI 10.1007/s10388-011-0284-6

Patel SA, 2010, J BIOL CHEM, V285, P15587, DOI 10.1074/jbc.M109.081687

Poele RHT, 1999, ONCOGENE, V18, P5765, DOI 10.1038/sj.onc.1202961

Stein HJ, 2005, LANGENBECK ARCH SURG, V390, P280, DOI 10.1007/s00423-004-0504-9

Takeno S, 2002, CANCER, V94, P2874, DOI 10.1002/cncr.10542

Teng Y, 2006, CANCER RES, V66, P6972, DOI 10.1158/0008-5472.CAN-06-0507

Tornillo L, 2006, J CLIN PATHOL, V59, P557, DOI 10.1136/jcp.2005.031112

Wang BX, 2012, J EXP CLIN CANC RES, V31, DOI 10.1186/1756-9966-31-11

Yamada H, 1995, MOL CELL BIOCHEM, V152, P149, DOI 10.1007/BF01076077

Yang Chun-Xia, 2005, Asian Pac J Cancer Prev, V6, P256

Yu CP, 2009, CARCINOGENESIS, V30, P894, DOI 10.1093/carcin/bgp064

NR 31

TC 15

Z9 19

U1 0

U2 7

PU SPRINGER

PI DORDRECHT

PA VAN GODEWIJCKSTRAAT 30, 3311 GZ DORDRECHT, NETHERLANDS

SN 0163-2116

EI 1573-2568

J9 DIGEST DIS SCI

JI Dig. Dis. Sci.

PD JUL

PY 2013

VL 58

IS 7

BP 2028

EP 2037

DI 10.1007/s10620-013-2597-x

PG 10

WC Gastroenterology & Hepatology

WE Science Citation Index Expanded (SCI-EXPANDED)

SC Gastroenterology & Hepatology

GA 173IA

UT WOS:000321071300032

PM 23456497

DA 2022-05-19

ER

PT J

AU Deng, Y

Li, YJ

Wu, TT

Chen, XY

Li, X

Cai, KC

Wu, X

AF Deng, Yu

Li, Yujiang

Wu, Tiantong

Chen, Xuyuan

Li, Xiang

Cai, Kaican

Wu, Xu

TI RAD6 Positively Affects Tumorigenesis of Esophageal Squamous Cell

Carcinoma by Regulating Histone Ubiquitination of CCNB1

SO BIOLOGICAL PROCEDURES ONLINE

LA English

DT Article

DE Esophageal squamous cell carcinoma; RAD6A; RAD6B; CCNB1; Ubiquitin

ID DNA-REPAIR; THERAPEUTIC TARGET; CANCER; ADENOCARCINOMA; IDENTIFICATION;

EPIDEMIOLOGY; EXPRESSION; UBE2B; CYCLE

AB Objective Esophageal carcinoma (ESCA) is deadly cancer worldwide with unknown etiology. This study aimed to investigate the impact and mechanism of RAD6 on the development of Esophageal squamous cell carcinoma (ESCC). Expressions of RAD6A and RAD6B in ESCA were investigated from TCGA dataset and their expressions in tissue sample of ESCA patients and cells were determined. Functional experiments were conducted to explore the impact of RAD6A and RAD6B on malignant characteristics of several kinds of ESCC cells. Animal experiment was established and injected with RAD6A and RAD6B shRNA to evaluate the effect on tumor growth. RAD6A and RAD6B were up-regulated in ESCC cells and tissues. Overexpressed RAD6A and RAD6B similarly increased ESCC cell proliferation, invasion and migration and silencing of RAD6 exerted opposite effects. Knockdown of RAD6A suppressed tumor growth and decreased the level of H2B, as data demonstrated positive correlation between RAD6A and CCNB1 in ESCC tissues. Collectively, this study elucidates that RAD6 is up-regulated in ESCC and promotes the progression of ESCC through up-regulation of CCNB1 to enhance H2B ubiquitination. These evidence provide a novel insight into the pathogenesis of ESCC and might contribute to the development of targeted therapy.

C1 [Deng, Yu; Li, Yujiang; Chen, Xuyuan; Cai, Kaican; Wu, Xu] Southern Med Univ, Dept Thorac Surg, Nanfang Hosp, Guangzhou, Peoples R China.

[Li, Yujiang] Southern Med Univ, Dept Thorac & Cardiovasc Surg, Affiliated Dongguan Peoples Hosp, Dongguan, Peoples R China.

[Wu, Tiantong] Southern Med Univ, Dept Gen Surg, Nanfang Hosp, Guangzhou, Peoples R China.

[Li, Xiang] Southern Med Univ, Dept Emergency Med, Nanfang Hosp, Guangzhou, Peoples R China.

RP Cai, KC; Wu, X (corresponding author), Southern Med Univ, Dept Thorac Surg, Nanfang Hosp, Guangzhou, Peoples R China.

EM doc_cai@163.com; wuxu20210727@163.com

FU National Nature Science Foundation of China [81671905]

FX This study was supported by National Nature Science Foundation of China

(No.81671905).

CR Abnet CC, 2018, GASTROENTEROLOGY, V154, P360, DOI 10.1053/j.gastro.2017.08.023

An JY, 2010, P NATL ACAD SCI USA, V107, P1912, DOI 10.1073/pnas.0910267107

Cai FF, 2014, PLOS ONE, V9, DOI 10.1371/journal.pone.0113727

Cao CX, 2015, JNCI-J NATL CANCER I, V107, DOI 10.1093/jnci/dju358

Chen FY, 2018, PATHOL RES PRACT, V214, P2081, DOI 10.1016/j.prp.2018.10.009

Cockram PE, 2021, CELL DEATH DIFFER, V28, P591, DOI 10.1038/s41418-020-00708-5

Coleman HG, 2018, GASTROENTEROLOGY, V154, P390, DOI 10.1053/j.gastro.2017.07.046

Dang FB, 2021, CELL DEATH DIFFER, V28, P427, DOI 10.1038/s41418-020-00648-0

di Pietro M, 2018, GASTROENTEROLOGY, V154, P421, DOI 10.1053/j.gastro.2017.07.041

Do HA, 2021, AGEING RES REV, V69, DOI 10.1016/j.arr.2021.101367

Feng H, 2020, ONCOGENE, V39, P5495, DOI 10.1038/s41388-020-1380-7

Gajan A, 2019, CELLS-BASEL, V8, DOI 10.3390/cells8111375

Gerard Brigitte, 2011, J Mol Signal, V6, P6, DOI 10.1186/1750-2187-6-6

Guo YL, 2018, CELL CYCLE, V17, P162, DOI 10.1080/15384101.2017.1361066

Gwon D, 2020, INT J MOL SCI, V21, DOI 10.3390/ijms21072352

Ho J, 2019, SEMIN CANCER BIOL, V55, P70, DOI 10.1016/j.semcancer.2018.04.004

Huang WL, 2020, ANTICANCER RES, V40, P6305, DOI 10.21873/anticanres.14651

Kashyap MK, 2018, MOL CANCER, V17, DOI 10.1186/s12943-018-0790-4

Kim J, 2009, CELL, V137, P459, DOI 10.1016/j.cell.2009.02.027

Kim YW, 2013, NEURO-ONCOLOGY, V15, P829, DOI 10.1093/neuonc/not024

KOKEN MHM, 1991, P NATL ACAD SCI USA, V88, P8865, DOI 10.1073/pnas.88.20.8865

LAWRENCE C, 1994, BIOESSAYS, V16, P253, DOI 10.1002/bies.950160408

Liu XS, 2021, FRONT ONCOL, V11, DOI 10.3389/fonc.2021.665388

Liu XS, 2020, J CANCER, V11, P4851, DOI 10.7150/jca.44754

Magistroni V, 2019, HAEMATOLOGICA, V104, P1789, DOI 10.3324/haematol.2017.179937

Miyazaki T, 2007, CELL CYCLE, V6, P1419

Pucci B, 2000, NEOPLASIA, V2, P291, DOI 10.1038/sj.neo.7900101

Rey R, 2019, MOL NEUROBIOL, V56, P4697, DOI 10.1007/s12035-018-1406-0

Rosner K, 2014, J SKIN CANCER, V2014, DOI 10.1155/2014/439205

Sarma A, 2021, AM J PATHOL, V191, P368, DOI 10.1016/j.ajpath.2020.10.015

Shchebet A, 2012, CELL CYCLE, V11, P2122, DOI 10.4161/cc.20548

Shekhar MPV, 2008, CANCER RES, V68, P1741, DOI 10.1158/0008-5472.CAN-07-2111

Shen JD, 2018, ONCOL LETT, V15, P7362, DOI 10.3892/ol.2018.8189

Siegel RL, 2019, CA-CANCER J CLIN, V69, P7, DOI 10.3322/caac.21551

Somasagara RR, 2017, ONCOGENE, V36, P6680, DOI 10.1038/onc.2017.279

Wang D, 2017, SEMIN CANCER BIOL, V44, P60, DOI 10.1016/j.semcancer.2017.03.010

Wang ZQ, 2014, DEV CELL, V29, P217, DOI 10.1016/j.devcel.2014.03.012

Xu WX, 2019, SIGNAL TRANSDUCT TAR, V4, DOI 10.1038/s41392-019-0081-6

Yang YM, 2020, SIGNAL TRANSDUCT TAR, V5, DOI 10.1038/s41392-020-00323-3

Yatsenko AN, 2013, MOL HUM REPROD, V19, P388, DOI 10.1093/molehr/gat008

Zhao YH, 2016, GENES-BASEL, V7, DOI 10.3390/genes7120116

NR 41

TC 0

Z9 0

U1 1

U2 1

PU BMC

PI LONDON

PA CAMPUS, 4 CRINAN ST, LONDON N1 9XW, ENGLAND

EI 1480-9222

J9 BIOL PROCED ONLINE

JI Biol. Proced. Online

PD MAR 23

PY 2022

VL 24

IS 1

AR 4

DI 10.1186/s12575-022-00165-z

PG 12

WC Biochemical Research Methods

WE Science Citation Index Expanded (SCI-EXPANDED)

SC Biochemistry & Molecular Biology

GA ZY2JG

UT WOS:000772414800001

PM 35321657

OA gold, Green Published

DA 2022-05-19

ER

PT J

AU Yao, WJ

Chen, Q

Li, SS

Jia, XB

Xu, L

Wei, L

AF Yao, Wenjian

Chen, Qing

Li, Saisai

Jia, Xiangbo

Xu, Lei

Wei, Li

TI RELT promotes the growth of esophageal squamous cell carcinoma by

activating the NF-kappa B pathway

SO CELL CYCLE

LA English

DT Article

DE RELT; esophageal squamous cell carcinoma; NF-kappa B pathway

ID FACTOR-RECEPTOR SUPERFAMILY; TUMOR-NECROSIS-FACTOR; TNF RECEPTOR;

CANCER; INFLAMMATION

AB The members of the tumor necrosis factor receptor (TNFR) family have been demonstrated to play critical roles in various cancers. However, little is known about the function of the Receptor Expressed in Lymphoid Tissues (RELT) in cancers, which is a member of the TNFR family, especially in the esophageal squamous cell carcinoma (ESCC). In this study, we found that RELT expression was increased in ESCC tissues and was consequently associated with poor overall survival of ESCC patients. Moreover, RELT overexpression was found to promote cell growth, cell cycle progression, and suppressed cell apoptosis in vitro; it also decreased the expression of p27 and caspase 3, and increased the expression of survivin. In addition, RELT contributed to the tumorigenesis of ESCC in vivo. Furthermore, we suggest that RELT may function in the pathogenesis of ESCC by activating the nuclear factor kappa B (NF-kappa B) pathway. An inhibitor of NF-kappa B reversed the RELT-induced malignancy in the ESCC cells. Altogether, our findings identified that RELT served as an oncogene in ESCC through the NF-kappa B pathway, suggesting that RELT may be developed as a novel biomarker for the diagnosis and treatment of the ESCC.

C1 [Yao, Wenjian; Li, Saisai; Jia, Xiangbo; Xu, Lei; Wei, Li] Henan Univ, Sch Clin Med, Henan Prov Peoples Hosp, Peoples Hosp,Zhengzhou Univ,Dept Thorac Surg, 7 Weiwu Rd, Zhengzhou 450003, Henan, Peoples R China.

[Chen, Qing] Jingjiang Peoples Hosp, Dept Oncol, Jingjiang, Jiangsu, Peoples R China.

RP Wei, L (corresponding author), Henan Univ, Sch Clin Med, Henan Prov Peoples Hosp, Peoples Hosp,Zhengzhou Univ,Dept Thorac Surg, 7 Weiwu Rd, Zhengzhou 450003, Henan, Peoples R China.

EM wlixxt@163.com

OI Yao, Wenjian/0000-0003-4672-113X

FU Henan Provincial People's Hospital & Medical Service Capacity

Improvement Project in Henan Provincial Medical Institutions [23456]

FX This study was supported by "23456 Talent Project" of Henan Provincial

People's Hospital & Medical Service Capacity Improvement Project in

Henan Provincial Medical Institutions.

CR Aggarwal BB, 2003, NAT REV IMMUNOL, V3, P745, DOI 10.1038/nri1184

Aggarwal BB, 2012, BLOOD, V119, P651, DOI 10.1182/blood-2011-04-325225

Ben-Neriah Y, 2011, NAT IMMUNOL, V12, P715, DOI 10.1038/ni.2060

Bodmer JL, 2002, TRENDS BIOCHEM SCI, V27, P19, DOI 10.1016/S0968-0004(01)01995-8

Croft M, 2013, NAT REV DRUG DISCOV, V12, P147, DOI 10.1038/nrd3930

Cusick JK, 2006, BIOCHEM BIOPH RES CO, V340, P535, DOI 10.1016/j.bbrc.2005.12.033

DiDonato JA, 2012, IMMUNOL REV, V246, P379, DOI 10.1111/j.1600-065X.2012.01099.x

Ferlay J, 2015, INT J CANCER, V136, pE359, DOI 10.1002/ijc.29210

Hehlgans T, 2005, IMMUNOLOGY, V115, P1, DOI 10.1111/j.1365-2567.2005.02143.x

Johansson J, 2015, FRONT ONCOL, V5, DOI 10.3389/fonc.2015.00003

Li QT, 2005, TRENDS IMMUNOL, V26, P318, DOI 10.1016/j.it.2005.04.003

Liu ZF, 2018, HUM PATHOL, V80, P179, DOI 10.1016/j.humpath.2018.03.027

Locksley RM, 2001, CELL, V104, P487, DOI 10.1016/S0092-8674(01)00237-9

Naugler WE, 2008, CURR OPIN GENET DEV, V18, P19, DOI 10.1016/j.gde.2008.01.020

Ohashi S, 2015, GASTROENTEROLOGY, V149, P1700, DOI 10.1053/j.gastro.2015.08.054

Pennathur A, 2013, LANCET, V381, P400, DOI 10.1016/S0140-6736(12)60643-6

Perkins ND, 2012, NAT REV CANCER, V12, P121, DOI 10.1038/nrc3204

Polek TC, 2006, BIOCHEM BIOPH RES CO, V343, P125, DOI 10.1016/j.bbrc.2006.02.125

Sica GL, 2001, BLOOD, V97, P2702, DOI 10.1182/blood.V97.9.2702

Sun YY, 2017, SCI REP-UK, V7, DOI 10.1038/s41598-017-18056-9

Wu ZZ, 2014, METHODS MOL BIOL, V1155, P1, DOI 10.1007/978-1-4939-0669-7_1

Zhong L, 2008, BREAST CANCER RES, V10, DOI 10.1186/bcr2091

NR 22

TC 0

Z9 0

U1 1

U2 3

PU TAYLOR & FRANCIS INC

PI PHILADELPHIA

PA 530 WALNUT STREET, STE 850, PHILADELPHIA, PA 19106 USA

SN 1538-4101

EI 1551-4005

J9 CELL CYCLE

JI Cell Cycle

PD JUL 3

PY 2021

VL 20

IS 13

BP 1231

EP 1241

DI 10.1080/15384101.2021.1924451

EA JUN 2021

PG 11

WC Cell Biology

WE Science Citation Index Expanded (SCI-EXPANDED)

SC Cell Biology

GA TS3JC

UT WOS:000661006300001

PM 34121605

DA 2022-05-19

ER

PT J

AU Liu, J

Mayekar, MK

Wu, W

Yan, M

Guan, HY

Wang, JW

Zaman, A

Cui, YB

Bivona, TG

Choudhry, H

Xing, QH

Cao, W

AF Liu, Jia

Mayekar, Manasi K.

Wu, Wei

Yan, Ming

Guan, Hongya

Wang, Jinwu

Zaman, Aubhishek

Cui, Yuanbo

Bivona, Trever G.

Choudhry, Hani

Xing, Qinghe

Cao, Wei

TI Long non-coding RNA ESCCAL-1 promotes esophageal squamous cell carcinoma

by down regulating the negative regulator of APOBEC3G

SO CANCER LETTERS

LA English

DT Article

DE LncRNA; Esophageal squamous cell carcinoma; miR-590-3p; PI3K-Akt pathway

ID SIGNALING PATHWAY; POOR-PROGNOSIS; CANCER; APOPTOSIS; MIGRATION;

INVASION; GENE; PROLIFERATION; CONTRIBUTES; SUPPRESSES

AB The expression of lncRNA ESCCAL-1 is upregulated in esophageal squamous cell carcinoma (ESCC). However, the molecular pathways regulated by ESCCAL-1 in esophageal cancer remain obscure. We found that high expression of the lncRNA ESCCAL-1 in human ESCC tumors correlated with worse clinicopathologic features. Furthermore, depletion of ESCCAL-1 in ESCC models inhibited the cellular processes associated with malignancy, including proliferation, migration and invasion, resistance to apoptosis, and impaired tumor growth in mice. Using a combinatorial approach, we discovered that ESCCAL-1 regulates malignant phenotypes in ESCC by acting as a molecular sponge for miR-590-3p. This interaction prevents miR-590-3p from suppressing APOBEC3G expression. Increased APOBEC3G was also a biomarker of worse clinicopathologic features in human ESCC tumors. Depletion of ESSCAL-1 or APOBEC3G, or overexpression of miR-590-3p resulted in increased apoptosis due to downregulation of the PI3K/Akt signaling. This study demonstrates that the lncRNA ESCCAL-1 promotes malignant features of ESCC by relieving the inhibitory effect of miR-590-3p on APOBEC3G expression and identifies potential biomarkers or therapeutic targets to improve ESCC treatment outcomes.

C1 [Liu, Jia; Guan, Hongya; Cui, Yuanbo; Cao, Wei] Zhengzhou Univ, Translat Med Ctr, Zhengzhou Cent Hosp, 16 Tongbai North Rd, Zhengzhou 450007, Peoples R China.

[Mayekar, Manasi K.; Wu, Wei; Zaman, Aubhishek; Bivona, Trever G.] Univ Calif San Francisco, Dept Med, San Francisco, CA 94143 USA.

[Yan, Ming] Zhengzhou Univ, Basic Med Coll, Zhengzhou, Peoples R China.

[Wang, Jinwu] Linzhou Canc Hosp, Dept Pathol, Linzhou, Peoples R China.

[Choudhry, Hani] King Abdulaziz Univ, Fac Sci, King Fahd Med Res Ctr, Dept Biochem,Canc & Mutagenesis Unit, Jeddah, Saudi Arabia.

[Xing, Qinghe] Fudan Univ, Inst Biomed Sci, Shanghai, Peoples R China.

[Xing, Qinghe] Fudan Univ, Childrens Hosp, Shanghai, Peoples R China.

RP Cao, W (corresponding author), Zhengzhou Univ, Translat Med Ctr, Zhengzhou Cent Hosp, 16 Tongbai North Rd, Zhengzhou 450007, Peoples R China.; Xing, QH (corresponding author), Fudan Univ, Inst Biomed Sci, Shanghai, Peoples R China.; Xing, QH (corresponding author), Fudan Univ, Childrens Hosp, Shanghai, Peoples R China.

EM liujia880318@163.com; qhxing@sjtu.edu.cn; canwei7@zzu.edu.cn

RI wu, wei/J-2797-2019

OI wu, wei/0000-0002-6556-067X; CAO, WEI/0000-0002-2851-4359

FU National Natural Science Foundation of China [81171992, 31570899];

Natural Science Foundation of Henan [182102310328, 162300410279,

192102310096]; Education Department of Henan Province [18B310022,

19A320037]; Medical Science Research Project of Henan Province

[SB201903032, 2018020765]

FX This work was supported by the National Natural Science Foundation of

China (Grants 81171992, 31570899); the Natural Science Foundation of

Henan (Grants, 182102310328, 162300410279, 192102310096); the Education

Department of Henan Province (Grants 18B310022, 19A320037); the Medical

Science Research Project of Henan Province (SB201903032, 2018020765).

CR Buisson R, 2019, SCIENCE, V364, P1251, DOI 10.1126/science.aaw2872

Burns MB, 2013, NATURE, V494, P366, DOI 10.1038/nature11881

Cao W, 2020, NAT COMMUN, V11, DOI 10.1038/s41467-020-17227-z

Cao W, 2018, HISTOL HISTOPATHOL, V33, P117, DOI 10.14670/HH-11-912

Cao W, 2013, INT J GENOMICS, V2013, DOI 10.1155/2013/480534

Cao XG, 2015, CANCER BIOMARK, V15, P717, DOI 10.3233/CBM-150513

Chen JA, 2019, MOL CANCER, V18, DOI 10.1186/s12943-019-0947-9

Cui YB, 2018, ONCOTARGET, V9, P783, DOI 10.18632/oncotarget.23153

Dail M, 2014, NATURE, V513, P512, DOI 10.1038/nature13495

Ding QQ, 2011, J CLIN INVEST, V121, P4526, DOI 10.1172/JCI45008

Dunham I, 2012, NATURE, V489, P57, DOI 10.1038/nature11247

Feng MG, 2018, BIOMED PHARMACOTHER, V101, P1003, DOI 10.1016/j.biopha.2018.02.043

Feng ZY, 2017, EUR REV MED PHARMACO, V21, P4844

Gao GD, 2018, EUR REV MED PHARMACO, V22, P422, DOI 10.26355/eurrev_201801_14191

Gao TY, 2015, MOL CARCINOGEN, V54, P359, DOI 10.1002/mc.22106

Garg A, 2015, BLOOD CELL MOL DIS, V55, P248, DOI 10.1016/j.bcmd.2015.07.009

Ge XS, 2013, CANCER SCI, V104, P1675, DOI 10.1111/cas.12296

Ge X, 2017, TUMOR BIOL, V39, DOI 10.1177/1010428317695947

Hao YB, 2015, BMC CANCER, V15, DOI 10.1186/s12885-015-1179-z

Lagergren J, 2017, LANCET, V390, P2383, DOI 10.1016/S0140-6736(17)31462-9

Li XW, 2019, ONCOTARGETS THER, V12, P423, DOI 10.2147/OTT.S181914

Li YJ, 2019, MOL ONCOL, V13, P2375, DOI 10.1002/1878-0261.12565

Liang H, 2017, CANCER BIOL MED, V14, P33, DOI 10.20892/j.issn.2095-3941.2016.0093

Liu J, 2017, ONCOL LETT, V14, P5711, DOI 10.3892/ol.2017.6953

McLoughlin NM, 2018, CELL CHEM BIOL, V25, P19, DOI 10.1016/j.chembiol.2017.10.009

Mercer TR, 2009, NAT REV GENET, V10, P155, DOI 10.1038/nrg2521

Nishitsuji H, 2016, P NATL ACAD SCI USA, V113, P10388, DOI 10.1073/pnas.1525022113

Okada A, 2016, FRONT MICROBIOL, V7, DOI 10.3389/fmicb.2016.02027

Pan ZW, 2016, CANCER MED-US, V5, P2442, DOI 10.1002/cam4.770

Pang HY, 2015, BIOCHEM BIOPH RES CO, V468, P739, DOI 10.1016/j.bbrc.2015.11.025

Parkin DM, 2005, CA-CANCER J CLIN, V55, P74, DOI 10.3322/canjclin.55.2.74

Pennathur A, 2013, LANCET, V381, P400, DOI 10.1016/S0140-6736(12)60643-6

Sidi S, 2008, CELL, V133, P864, DOI 10.1016/j.cell.2008.03.037

Stein LD, 2004, NATURE, V431, P915, DOI 10.1038/431915a

Testa Ugo, 2017, Medicines (Basel), V4, DOI 10.3390/medicines4030067

Wensveen FM, 2011, APOPTOSIS, V16, P708, DOI 10.1007/s10495-011-0599-8

Wu DD, 2018, BMC CANCER, V18, DOI 10.1186/s12885-018-4391-9

Wu J, 2015, SCI REP-UK, V5, DOI 10.1038/srep12230

Wu YY, 2017, MOL CANCER, V16, DOI 10.1186/s12943-017-0715-7

Yuan JY, 2016, CELL, V167, P1693, DOI 10.1016/j.cell.2016.11.047

Zhang YL, 2019, AM J TRANSL RES, V11, P1084

Zheng ZQ, 2019, CANCER RES, V79, P4612, DOI 10.1158/0008-5472.CAN-19-0799

NR 42

TC 7

Z9 9

U1 4

U2 17

PU ELSEVIER IRELAND LTD

PI CLARE

PA ELSEVIER HOUSE, BROOKVALE PLAZA, EAST PARK SHANNON, CO, CLARE, 00000,

IRELAND

SN 0304-3835

EI 1872-7980

J9 CANCER LETT

JI Cancer Lett.

PD NOV 28

PY 2020

VL 493

BP 217

EP 227

DI 10.1016/j.canlet.2020.09.001

PG 11

WC Oncology

WE Science Citation Index Expanded (SCI-EXPANDED)

SC Oncology

GA OF9MO

UT WOS:000581522200026

PM 32905814

DA 2022-05-19

ER

PT J

AU Jin, F

Wang, HB

Li, D

Fang, CC

Li, WY

Shi, QT

Diao, YL

Ding, ZY

Dai, XJ

Tao, L

Sunagawa, M

Wu, F

Qian, YY

Liu, YQ

AF Jin, Feng

Wang, Haibo

Li, Dan

Fang, Chuanchi

Li, Wenyuan

Shi, Qingtong

Diao, Yali

Ding, Zhiyan

Dai, Xiaojun

Tao, Li

Sunagawa, Masataka

Wu, Feng

Qian, Yayun

Liu, Yanqing

TI DJ-1 promotes cell proliferation and tumor metastasis in esophageal

squamous cell carcinoma via the Wnt/beta-catenin signaling pathway

SO INTERNATIONAL JOURNAL OF ONCOLOGY

LA English

DT Article

DE DJ-1; esophageal squamous cell carcinoma; cell proliferation; invasion;

Wnt; beta-catenin signaling pathway

ID EPITHELIAL-MESENCHYMAL TRANSITION; HEPATOCELLULAR-CARCINOMA; CANCER

PROGRESSION; STATISTICS; REGULATOR; DISEASE; PROTEIN; LRP6; EMT

AB DJ-1, an oncogene, has been reported to be an independent prognostic indicator of poor survival in patients with esophageal squamous cell carcinoma (ESCC). The aim of the present study was to investigate the role of DJ-1 in tumor cell proliferation and invasion in ESCC and its underlying mechanisms. It was observed that the expression level of DJ-1 was upregulated and positively associated with EMT biomarkers in 84 human ESCC tissue specimens. Overexpression and knockdown experiments demonstrated that DJ-1 was involved in proliferation, migration, invasion and EMT in ECA-109 cells in vitro and extensive peritoneal seeding in a peritoneal dissemination mice model. Furthermore, the present data revealed that DJ-1 could activate the Wnt/beta-catenin signaling pathway, which mediates the EMT and metastasis in ESCC. In conclusions, DJ-1 promoted proliferation, invasion, metastasis and the EMT in ESCC via activation of the Wnt/beta-catenin signal pathway. The present results suggested DJ-1 could represent a promising therapeutic target for the prevention and treatment of ESCC-related metastasis.

C1 [Jin, Feng; Wu, Feng] Yangzhou Univ, Affiliated Hosp, Dept Resp Med, Yangzhou 225001, Jiangsu, Peoples R China.

[Wang, Haibo; Li, Dan; Fang, Chuanchi; Li, Wenyuan; Dai, Xiaojun; Tao, Li; Qian, Yayun; Liu, Yanqing] Yangzhou Univ, Inst Combining Chinese Tradit & Western Med, Med Coll, 136 Jiangyang Middle Rd, Yangzhou 225009, Jiangsu, Peoples R China.

[Wang, Haibo; Li, Dan; Fang, Chuanchi; Li, Wenyuan; Dai, Xiaojun; Tao, Li; Qian, Yayun; Liu, Yanqing] Yangzhou Univ, State Adm Tradit Chinese Med, Key Lab Tox Pathogens Based Therapeut Approaches, Yangzhou 225009, Jiangsu, Peoples R China.

[Shi, Qingtong; Diao, Yali] Yangzhou Univ, Affiliated Hosp, Dept Thorac Surg, Yangzhou 225001, Jiangsu, Peoples R China.

[Ding, Zhiyan] Yangzhou Univ, Affiliated Hosp, Dept Pathol, Yangzhou 225001, Jiangsu, Peoples R China.

[Dai, Xiaojun] Yangzhou Tradit Chinese Med Hosp, Yangzhou 225009, Jiangsu, Peoples R China.

[Sunagawa, Masataka] Showa Univ, Sch Med, Dept Physiol, Tokyo 1428555, Japan.

RP Liu, YQ (corresponding author), Yangzhou Univ, Inst Combining Chinese Tradit & Western Med, Med Coll, 136 Jiangyang Middle Rd, Yangzhou 225009, Jiangsu, Peoples R China.

EM yzumpi@163.com

RI Sunagawa, Masataka/ABE-4996-2021

FU National Natural Science Foundation of China [81573656, 81773944];

Natural Science Foundation of Jiangsu Province of China [BK20171290];

Natural Science Foundation of Jiangsu Province for Youths [BK20170516]

FX This work was financially supported by the National Natural Science

Foundation of China (grant nos. 81573656 and 81773944), the Natural

Science Foundation of Jiangsu Province of China (grant no. BK20171290)

and the Natural Science Foundation of Jiangsu Province for Youths (grant

no. BK20170516).

CR Aleyasin H, 2010, P NATL ACAD SCI USA, V107, P3186, DOI 10.1073/pnas.0914876107

Benati M, 2018, J CLIN LAB ANAL, V32, DOI 10.1002/jcla.22223

Bonapace L, 2014, NATURE, V515, DOI 10.1038/nature13862

Chan JYH, 2015, PHARMACOL THERAPEUT, V156, P69, DOI 10.1016/j.pharmthera.2015.09.005

Chen J, 2017, ONCOTARGET, V8, P2501, DOI 10.18632/oncotarget.12388

Chen WQ, 2016, CA-CANCER J CLIN, V66, P115, DOI 10.3322/caac.21338

Chiurillo M.A., 2015, WORLD J EXP MED, V5, P84

Clements CM, 2006, P NATL ACAD SCI USA, V103, P15091, DOI 10.1073/pnas.0607260103

Clevers H, 2006, CELL, V127, P469, DOI 10.1016/j.cell.2006.10.018

Da CL, 2016, ONCOL REP, V35, P2767, DOI 10.3892/or.2016.4661

Du CL, 2014, J TRANSL MED, V12, DOI 10.1186/s12967-014-0259-1

Ferlay J, 2015, INT J CANCER, V136, pE359, DOI 10.1002/ijc.29210

Fife CM, 2014, BRIT J PHARMACOL, V171, P5507, DOI 10.1111/bph.12704

Geng XF, 2016, MOL MED REP, V14, P3007, DOI 10.3892/mmr.2016.5632

Gu Y, 2016, J PATHOL, V239, P60, DOI 10.1002/path.4697

Guo WX, 2017, ONCOL LETT, V14, P6585, DOI 10.3892/ol.2017.7100

Hurst DR, 2011, INT REV CEL MOL BIO, V286, P107, DOI 10.1016/B978-0-12-385859-7.00003-3

Joyce JA, 2009, NAT REV CANCER, V9, P239, DOI 10.1038/nrc2618

Kawate T, 2015, CANCER SCI, V106, P938, DOI 10.1111/cas.12673

Kim RH, 2005, CANCER CELL, V7, P263, DOI 10.1016/j.ccr.2005.02.010

Lin DC, 2018, GASTROENTEROLOGY, V154, P374, DOI 10.1053/j.gastro.2017.06.066

MacDonald BT, 2009, DEV CELL, V17, P9, DOI 10.1016/j.devcel.2009.06.016

McCubrey James A, 2015, Adv Biol Regul, V57, P75, DOI 10.1016/j.jbior.2014.09.016

Nusse R, 2017, CELL, V169, P985, DOI 10.1016/j.cell.2017.05.016

Song YM, 2014, NATURE, V509, P91, DOI 10.1038/nature13176

Steeg PS, 2016, NAT REV CANCER, V16, P201, DOI 10.1038/nrc.2016.25

Tang X, 2016, DIS ESOPHAGUS, V29, P207, DOI 10.1111/dote.12289

To SKY, 2017, ONCOGENE, V36, P5927, DOI 10.1038/onc.2017.185

Torre LA, 2015, CA-CANCER J CLIN, V65, P87, DOI 10.3322/caac.21262

Tsai JH, 2013, GENE DEV, V27, P2192, DOI 10.1101/gad.225334.113

Tung EKK, 2012, PLOS ONE, V7, DOI 10.1371/journal.pone.0036565

Turajlic S, 2016, SCIENCE, V352, P169, DOI 10.1126/science.aaf2784

Valastyan S, 2011, CELL, V147, P275, DOI 10.1016/j.cell.2011.09.024

White BD, 2012, GASTROENTEROLOGY, V142, P219, DOI 10.1053/j.gastro.2011.12.001

Wu XF, 2016, INT J ONCOL, V48, P1333, DOI 10.3892/ijo.2016.3360

Xiao JF, 2018, BMC CANCER, V18, DOI 10.1186/s12885-018-4850-3

Xu K, 2017, TUMOR BIOL, V39, DOI 10.1177/1010428317705504

Yamamoto H, 2006, DEV CELL, V11, P213, DOI 10.1016/j.devcel.2006.07.003

Yuen HF, 2008, CANCER EPIDEM BIOMAR, V17, P3593, DOI 10.1158/1055-9965.EPI-08-0214

Zhou J, 2018, CELL DEATH DIS, V9, DOI 10.1038/s41419-018-0883-4

Zhu Yaodong, 2014, BMC Complement Altern Med, V14, P433, DOI 10.1186/1472-6882-14-433

NR 41

TC 3

Z9 3

U1 1

U2 6

PU SPANDIDOS PUBL LTD

PI ATHENS

PA POB 18179, ATHENS, 116 10, GREECE

SN 1019-6439

EI 1791-2423

J9 INT J ONCOL

JI Int. J. Oncol.

PD MAY

PY 2020

VL 56

IS 5

BP 1115

EP 1128

DI 10.3892/ijo.2020.5005

PG 14

WC Oncology

WE Science Citation Index Expanded (SCI-EXPANDED)

SC Oncology

GA LH5KL

UT WOS:000528823800007

PM 32319588

OA Green Published, hybrid

DA 2022-05-19

ER

PT J

AU Peng, LN

Cheng, SJ

Lin, Y

Cui, QH

Luo, YY

Chu, JH

Shao, MM

Fan, WY

Chen, YM

Lin, A

Xi, YY

Sun, YX

Zhang, L

Zhang, C

Tan, W

Gao, G

Wu, C

Lin, DX

AF Peng, Linna

Cheng, Sijin

Lin, Yuan

Cui, Qionghua

Luo, Yingying

Chu, Jiahui

Shao, Mingming

Fan, Wenyi

Chen, Yamei

Lin, Ai

Xi, Yiyi

Sun, Yanxia

Zhang, Lei

Zhang, Chao

Tan, Wen

Gao, Ge

Wu, Chen

Lin, Dongxin

TI CCGD-ESCC: A Comprehensive Database for Genetic Variants Associated with

Esophageal Squamous Cell Carcinoma in Chinese Population

SO GENOMICS PROTEOMICS & BIOINFORMATICS

LA English

DT Article; Data Paper

DE Esophageal cancer; Germline variants; Somatic mutations; Association

database; Chinese population

ID GENOME-WIDE ASSOCIATION; SUSCEPTIBILITY LOCI; CANCER STATISTICS;

LANDSCAPE; SURVIVAL; PLCE1

AB Esophageal squamous-cell carcinoma (ESCC) is one of the most lethal malignancies in the world and occurs at particularly higher frequency in China. While several genome-wide association studies (GWAS) of germline variants and whole-genome or whole-exome sequencing studies of somatic mutations in ESCC have been published, there is no comprehensive database publically available for this cancer. Here, we developed the Chinese Cancer Genomic Database-Esophageal Squamous Cell Carcinoma (CCGD-ESCC) database, which contains the associations of 69,593 single nucleotide polymorphisms (SNPs) with ESCC risk in 2022 cases and 2039 controls, survival time of 1006 ESCC patients (survival GWAS) and gene expression (expression quantitative trait loci, eQTL) in 94 ESCC patients. Moreover, this database also provides the associations between 8833 somatic mutations and survival time in 675 ESCC patients. Our user-friendly database is a resource useful for biologists and oncologists not only in identifying the associations of genetic variants or somatic mutations with the development and progression of ESCC but also in studying the underlying mechanisms for tumorigenesis of the cancer. CCGD-ESCC is freely accessible at http://db.cbi.pku.edu.cn/ccgd/ESCCdb.

C1 [Peng, Linna; Cui, Qionghua; Luo, Yingying; Chu, Jiahui; Shao, Mingming; Fan, Wenyi; Chen, Yamei; Lin, Ai; Xi, Yiyi; Sun, Yanxia; Tan, Wen; Wu, Chen; Lin, Dongxin] Chinese Acad Med Sci, Dept Etiol & Carcinogenesis, Natl Canc Ctr, Canc Hosp, Beijing 100021, Peoples R China.

[Peng, Linna; Cui, Qionghua; Luo, Yingying; Chu, Jiahui; Shao, Mingming; Fan, Wenyi; Chen, Yamei; Lin, Ai; Xi, Yiyi; Sun, Yanxia; Tan, Wen; Wu, Chen; Lin, Dongxin] Peking Union Med Coll, Beijing 100021, Peoples R China.

[Cheng, Sijin; Lin, Yuan; Gao, Ge] Peking Univ, Sch Life Sci, Ctr Bioinformat, State Key Lab Prot & Plant Gene Res, Beijing 100871, Peoples R China.

[Cheng, Sijin; Lin, Yuan; Gao, Ge] Peking Univ, Beijing Adv Innovat Ctr Genom ICG, Beijing 100871, Peoples R China.

[Zhang, Lei] Natl Engn Res Ctr Beijing Biochip Technol, Beijing 102206, Peoples R China.

[Zhang, Chao] Peking Univ, Sch Life Sci, Beijing 100871, Peoples R China.

RP Wu, C (corresponding author), Chinese Acad Med Sci, Dept Etiol & Carcinogenesis, Natl Canc Ctr, Canc Hosp, Beijing 100021, Peoples R China.; Wu, C (corresponding author), Peking Union Med Coll, Beijing 100021, Peoples R China.; Gao, G (corresponding author), Peking Univ, Sch Life Sci, Ctr Bioinformat, State Key Lab Prot & Plant Gene Res, Beijing 100871, Peoples R China.; Gao, G (corresponding author), Peking Univ, Beijing Adv Innovat Ctr Genom ICG, Beijing 100871, Peoples R China.

EM gaog@mail.cbi.pku.edu.cn; chenwu@cicams.ac.cn

RI Cui, Qionghua/AAV-3079-2020; Gao, Ge/F-3615-2012

OI Wu, Chen/0000-0003-4954-1011; Peng, Linna/0000-0002-4094-8002; Fan,

Wenyi/0000-0002-7875-7404; Zhang, Chao/0000-0003-4167-4872; Cheng,

Si-Jin/0000-0002-2565-7127; Lin, Dongxin/0000-0002-8723-8868

FU CAMS Innovation Fund for Medical Sciences [2016-I2M-4-002]; National Key

RD Program [2016YFC1302701, 2016YFC0901603]

FX We would like to thank Yun Li and Mengjie Chen at University of North

Carolina and bioinformatic core facility at Peking University for their

help in imputation analysis and helpful suggestions of this study. Part

of the analysis is performed on the Computing Platform of the Center for

Life Sciences of Peking University. This work was supported by CAMS

Innovation Fund for Medical Sciences (Grant No. 2016-I2M-4-002 awarded

to CW) and the National Key R&D Program (Grant No. 2016YFC1302701 to CW

and Grant No. 2016YFC0901603 to GG).

CR Abnet CC, 2012, HUM MOL GENET, V21, P2132, DOI 10.1093/hmg/dds029

Abnet CC, 2010, NAT GENET, V42, P764, DOI 10.1038/ng.649

Albert FW, 2015, NAT REV GENET, V16, P197, DOI 10.1038/nrg3891

Altshuler DM, 2015, NATURE, V526, P68, DOI 10.1038/nature15393

Ardlie KG, 2015, SCIENCE, V348, P648, DOI 10.1126/science.1262110

Chang J, 2017, NAT COMMUN, V8, DOI 10.1038/ncomms15290

Chen WQ, 2016, CA-CANCER J CLIN, V66, P115, DOI 10.3322/caac.21338

Cheng XX, 2017, GASTROENTEROLOGY, V152, P1985, DOI 10.1053/j.gastro.2017.02.006

Gao YB, 2014, NAT GENET, V46, P1097, DOI 10.1038/ng.3076

Garrison E., 2012, HAPLOTYPE BASED VARI, DOI DOI 10.48550/ARXIV.1207.3907

Gibson G, 2015, GENOME MED, V7, DOI 10.1186/s13073-015-0186-7

Kim J, 2017, NATURE, V541, P169, DOI 10.1038/nature20805

Liu JG, 2012, EUR J CARDIO-THORAC, V41, pE7, DOI 10.1093/ejcts/ezr240

Montgomery SB, 2011, NAT REV GENET, V12, P277, DOI 10.1038/nrg2969

Nica AC, 2013, PHILOS T R SOC B, V368, DOI 10.1098/rstb.2012.0362

Qin HD, 2016, AM J HUM GENET, V98, P709, DOI 10.1016/j.ajhg.2016.02.021

Sawada G, 2016, GASTROENTEROLOGY, V150, P1171, DOI 10.1053/j.gastro.2016.01.035

Shabalin AA, 2012, BIOINFORMATICS, V28, P1353, DOI 10.1093/bioinformatics/bts163

Song YM, 2014, NATURE, V509, P91, DOI 10.1038/nature13176

Torre LA, 2015, CA-CANCER J CLIN, V65, P87, DOI 10.3322/caac.21262

Wang K, 2010, NUCLEIC ACIDS RES, V38, DOI 10.1093/nar/gkq603

Wang LD, 2010, NAT GENET, V42, P759, DOI 10.1038/ng.648

Wu C, 2014, NAT GENET, V46, P1001, DOI 10.1038/ng.3064

Wu C, 2013, NAT GENET, V45, P632, DOI 10.1038/ng.2638

Wu C, 2012, NAT GENET, V44, P1090, DOI 10.1038/ng.2411

Wu C, 2011, NAT GENET, V43, P679, DOI 10.1038/ng.849

Xing DY, 2003, ONCOL REP, V10, P1615

Zhang L, 2015, AM J HUM GENET, V96, P597, DOI 10.1016/j.ajhg.2015.02.017

NR 28

TC 10

Z9 10

U1 1

U2 8

PU ELSEVIER SCIENCE BV

PI AMSTERDAM

PA PO BOX 211, 1000 AE AMSTERDAM, NETHERLANDS

SN 1672-0229

EI 2210-3244

J9 GENOM PROTEOM BIOINF

JI Genom. Proteomics Bioinformatics

PD AUG

PY 2018

VL 16

IS 4

SI SI

BP 262

EP 268

DI 10.1016/j.gpb.2018.03.005

PG 7

WC Genetics & Heredity

WE Science Citation Index Expanded (SCI-EXPANDED)

SC Genetics & Heredity

GA GY1ZE

UT WOS:000448335600006

PM 30208340

OA Green Published, gold

DA 2022-05-19

ER

PT J

AU Song, G

Jing, W

Xue, S

Guo, HB

Yu, JM

AF Song, Ge

Jing, Wang

Xue, Song

Guo, Hongbo

Yu, Jinming

TI The prognostic value of nodal skip metastasis in resectable thoracic

esophageal squamous cell carcinoma

SO ONCOTARGETS AND THERAPY

LA English

DT Article

DE esophageal squamous cell carcinoma; surgery; nodal skip metastasis;

incidence; risk factors; prognosis

ID LYMPH-NODES; STAGING SYSTEM; CANCER; SURVIVAL; SURGERY; NUMBER;

CLASSIFICATION; SPREAD

AB Purpose: This study aimed to investigate the incidence of nodal skip metastasis (NSM) to identify the risk factors that influence NSM and to assess the prognostic value of NSM in patients with thoracic esophageal squamous cell carcinoma (ESCC).

Patients and methods: Between January 2009 and December 2013, 285 patients with ESCC with positive lymph nodes who underwent complete resection were enrolled.

Results: For the entire group, NSM occurred in 32.3% (92/285) of patients. The median survival time and 5-year survival rate in the NSM group were 28 months and 12.0%, respectively, compared with 36.3 months and 25.0%, respectively, in the non-NSM group (P=0.008). Both N stage (P=0.001) and T stage (P=0.014) were associated with the incidence of NSM. NSM (P=0.008), T stage (P=0.000), and N stage (P=0.000) were independent prognostic factors for survival. In the NSM group, T stage (P=0.014) and N stage (P=0.000) were independent prognostic factors for survival.

Conclusion: It was concluded that NSM is common in ESCC and is associated with poor survival.

C1 [Song, Ge; Xue, Song; Yu, Jinming] Univ Jinan, Shandong Acad Med Sci, Sch Med & Life Sci, Dept Radiat Oncol, Jinan, Peoples R China.

[Song, Ge; Xue, Song; Yu, Jinming] Shandong Univ, Shandong Canc Hosp, Dept Radiat Oncol, Jiyan Rd 440, Jinan 250117, Shandong, Peoples R China.

[Jing, Wang] Zhengzhou Univ, Affiliated Hosp 1, Dept Radiat Oncol, Zhengzhou, Peoples R China.

[Guo, Hongbo] Shandong Univ, Shandong Canc Hosp, Dept Thorac Surg, Jinan, Peoples R China.

RP Yu, JM (corresponding author), Shandong Univ, Shandong Canc Hosp, Dept Radiat Oncol, Jiyan Rd 440, Jinan 250117, Shandong, Peoples R China.

EM sdyujinming@163.com

FU Shandong Provincial Natural Science Foundation [ZR2015HZ004]; Natural

Science Foundation of Shandong Province [ZR2011HL029]

FX This study was supported by the grant from Shandong Provincial Natural

Science Foundation (ZR2015HZ004) to Jinming Yu and grant number

ZR2011HL029 from the Natural Science Foundation of Shandong Province to

Hongbo Guo.

CR Altorki NK, 2008, ANN SURG, V248, P221, DOI 10.1097/SLA.0b013e31817bbe59

An Feng-Shan, 2003, Ai Zheng, V22, P974

Brennan MF, 2001, ANN SURG, V234, P368

Chen JQ, 2010, ANN THORAC SURG, V90, P435, DOI 10.1016/j.athoracsur.2010.04.002

Chen JQ, 2009, EUR J CARDIO-THORAC, V36, P480, DOI 10.1016/j.ejcts.2009.03.056

Edge SB, 2010, ANN SURG ONCOL, V17, P1471, DOI 10.1245/s10434-010-0985-4

Ferlay J, 2010, INT J CANCER, V127, P2893, DOI 10.1002/ijc.25516

Groth SS, 2010, J THORAC CARDIOV SUR, V139, P612, DOI 10.1016/j.jtcvs.2009.07.017

Hofstetter W, 2007, ANN THORAC SURG, V84, P365, DOI 10.1016/j.athoracsur.2007.01.067

Hosch SB, 2001, J CLIN ONCOL, V19, P1970, DOI 10.1200/JCO.2001.19.7.1970

Jiang YQ, 2010, ANN THORAC SURG, V90, P908, DOI 10.1016/j.athoracsur.2010.05.060

Kunisaki C, 2005, J AM COLL SURGEONS, V201, P884, DOI 10.1016/j.jamcollsurg.2005.07.002

Li HH, 2009, J THORAC CARDIOV SUR, V137, P55, DOI 10.1016/j.jtcvs.2008.05.024

Liu J, 2016, EJSO-EUR J SURG ONC, V42, P1202, DOI 10.1016/j.ejso.2016.05.025

Morita M, 2008, SURGERY, V143, P499, DOI 10.1016/j.surg.2007.12.007

Natsugoe S, 2005, J SURG ONCOL, V89, P6, DOI 10.1002/jso.20172

Pardo BJD, 2015, WORLD J SURG, V87, P395

Patti MG, 1997, SURG CLIN N AM, V77, P959, DOI 10.1016/S0039-6109(05)70600-9

Prenzel KL, 2004, CANCER-AM CANCER SOC, V100, P1909, DOI 10.1002/cncr.20165

Prenzel KL, 2010, ANN THORAC SURG, V90, P1662, DOI 10.1016/j.athoracsur.2010.07.008

Rice TW, 2003, J THORAC CARDIOV SUR, V125, P1103, DOI 10.1067/mtc.2003.170

Rizk N, 2006, J THORAC CARDIOV SUR, V132, P1374, DOI 10.1016/j.jtcvs.2006.07.039

Scheunemann P, 1999, NEW ENGL J MED, V340, P1687, DOI 10.1056/NEJM199905273402116

Shiozawa M, 2007, HEPATO-GASTROENTEROL, V54, P81

Skandalakis JE, 2000, SURG CLIN N AM, V80, P85, DOI 10.1016/S0039-6109(05)70399-6

Stein HJ, 2005, ANN SURG, V242, P566, DOI 10.1097/01.sla.0000184211.75970.85

Tachibana M, 2001, DIGEST LIVER DIS, V33, P534, DOI 10.1016/S1590-8658(01)80103-1

Tachibana M, 2008, EUR J CARDIO-THORAC, V34, P427, DOI 10.1016/j.ejcts.2008.04.022

Torre LA, 2015, CA-CANCER J CLIN, V65, P87, DOI 10.3322/caac.21262

Wilson M, 2008, J SURG RES, V146, P11, DOI 10.1016/j.jss.2007.07.028

Wu J, 2012, WORLD J SURG ONCOL, V10, DOI 10.1186/1477-7819-10-210

Xu QR, 2011, WORLD J SURG, V35, P1303, DOI 10.1007/s00268-011-1015-9

Yong ELC, 2009, ANZ J SURG, V79, P724, DOI 10.1111/j.1445-2197.2009.05058.x

Zhang HL, 2010, DIS ESOPHAGUS, V23, P53, DOI 10.1111/j.1442-2050.2009.00971.x

Zhu ZF, 2013, ANN SURG ONCOL, V20, P3052, DOI 10.1245/s10434-013-2987-5

NR 35

TC 2

Z9 3

U1 0

U2 1

PU DOVE MEDICAL PRESS LTD

PI ALBANY

PA PO BOX 300-008, ALBANY, AUCKLAND 0752, NEW ZEALAND

SN 1178-6930

J9 ONCOTARGETS THER

JI OncoTargets Ther.

PY 2017

VL 10

BP 2729

EP 2736

DI 10.2147/OTT.S132062

PG 8

WC Biotechnology & Applied Microbiology; Oncology

WE Science Citation Index Expanded (SCI-EXPANDED)

SC Biotechnology & Applied Microbiology; Oncology

GA EW1RY

UT WOS:000402271500002

PM 28579811

OA Green Submitted, Green Published, gold

DA 2022-05-19

ER

PT J

AU Li, F

Zhu, DY

Yang, Y

Wu, K

Zhao, S

AF Li, Feng

Zhu, Dengyan

Yang, Yang

Wu, Kai

Zhao, Song

TI Overexpression of calcyphosine is associated with poor prognosis in

esophageal squamous cell carcinoma

SO ONCOLOGY LETTERS

LA English

DT Article

DE calcyphosine; calcium-binding protein; esophageal squamous cell

carcinoma; prognosis

ID CYCLIC-AMP; PLASMA-MEMBRANE; BINDING PROTEIN; CANCER; EXPRESSION;

PROLIFERATION; GENE; IDENTIFICATION; BIOLOGY; MARKER

AB Calcyphosine (CAPS), a calcium-binding protein, has been identified as a potential diagnostic and prognostic biomarker in several human carcinomas. However, little is known about CAPS in esophageal squamous cell carcinoma (ESCC). The present study aimed to investigate the expression levels of CAPS in ESCC tissues and evaluate its clinicopathological significance. Reverse transcription-quantitative polymerase chain reaction and immunohistochemical staining were conducted to detect the expression of CAPS in ESCC tissues and adjacent non-cancerous tissues. ESCC samples exhibited higher levels of CAPS mRNA than paired non-cancerous samples (P=0.0015), and the mRNA level of CAPS was positively associated with histological grade (P=0.0013) and tumor invasion depth (P=0.0206). In addition, Kaplan-Meier survival analysis revealed that patients with high CAPS expression experienced significantly shorter 5-year overall survival times than those with low CAPS expression (P=0.0112). Multivariate analysis demonstrated that CAPS protein expression was an independent prognostic biomarker for patients with ESCC. In conclusion, the findings of the present study demonstrated that CAPS may represent a novel diagnostic indicator and an independent prognostic biomarker in ESCC.

C1 [Li, Feng; Zhu, Dengyan; Yang, Yang; Wu, Kai; Zhao, Song] Zhengzhou Univ, Affiliated Hosp 1, Dept Thorac Surg, 1 Jianshe Rd, Zhengzhou 450052, Henan, Peoples R China.

RP Zhao, S (corresponding author), Zhengzhou Univ, Affiliated Hosp 1, Dept Thorac Surg, 1 Jianshe Rd, Zhengzhou 450052, Henan, Peoples R China.

EM zhaosong@zzu.edu.cn

FU Science and Technology Innovation Team Support Plan in Universities of

Henan Province [13IRTSTHN011]

FX The present study was supported by the Science and Technology Innovation

Team Support Plan in Universities of Henan Province (grant no.

13IRTSTHN011). The authors would like to thank Dr Xinfeng Chen (the

First Affiliated Hospital of Zhengzhou University, Zhengzhou, China) for

providing assistance with the experiments and Mr Jing He (Huashan

Hospital Affiliated to Fudan University, Shanghai, China) for partaking

in valuable discussions.

CR Chen TC, 1998, LAB INVEST, V78, P165

Clement S, 1997, BIOCHEM BIOPH RES CO, V232, P407, DOI 10.1006/bbrc.1997.6297

de Bont JM, 2007, J NEUROPATH EXP NEUR, V66, P505, DOI 10.1097/01.jnen.0000240475.35414.c3

Deliot N, 2015, BBA-BIOMEMBRANES, V1848, P2512, DOI 10.1016/j.bbamem.2015.06.009

Di Virgilio F, 2012, CANCER RES, V72, P5441, DOI 10.1158/0008-5472.CAN-12-1600

Dong H, 2008, J MOL BIOL, V383, P455, DOI 10.1016/j.jmb.2008.08.048

DUMONT JE, 1989, TRENDS BIOCHEM SCI, V14, P67, DOI 10.1016/0968-0004(89)90046-7

ElHousni H, 1997, BBA-GENE STRUCT EXPR, V1352, P249, DOI 10.1016/S0167-4781(97)00073-0

Fujita Y, 2008, CANCER LETT, V263, P280, DOI 10.1016/j.canlet.2008.01.013

Johansson HJ, 2015, CLIN PROTEOM, V12, DOI 10.1186/s12014-015-9080-y

Kamangar F, 2006, J CLIN ONCOL, V24, P2137, DOI 10.1200/JCO.2005.05.2308

LAMY F, 1986, EUR J BIOCHEM, V155, P265, DOI 10.1111/j.1432-1033.1986.tb09485.x

LECOCQ R, 1979, EUR J BIOCHEM, V102, P147, DOI 10.1111/j.1432-1033.1979.tb06274.x

LECOCQ R, 1990, ELECTROPHORESIS, V11, P200, DOI 10.1002/elps.1150110303

LEFORT A, 1989, EMBO J, V8, P111, DOI 10.1002/j.1460-2075.1989.tb03354.x

Li ZY, 2008, INT J CANCER, V123, P2377, DOI 10.1002/ijc.23808

Li ZY, 2010, INT J GYNECOL CANCER, V20, P9, DOI 10.1111/IGC.0b013e3181a9026d

Livak KJ, 2001, METHODS, V25, P402, DOI 10.1006/meth.2001.1262

MacManus J P, 1975, Adv Cyclic Nucleotide Res, V5, P719

Mehrani H, 2009, PROTEOM CLIN APPL, V3, P1191, DOI 10.1002/prca.200900001

NEMOTO Y, 1993, J CELL BIOL, V123, P963, DOI 10.1083/jcb.123.4.963

Partheen K, 2006, EUR J CANCER, V42, P2846, DOI 10.1016/j.ejca.2006.06.026

Pastor MD, 2013, J PROTEOMICS, V89, P227, DOI 10.1016/j.jprot.2013.04.037

Raphael M, 2014, P NATL ACAD SCI USA, V111, pE3870, DOI 10.1073/pnas.1413409111

Rice TW, 2010, ANN SURG ONCOL, V17, P1721, DOI 10.1245/s10434-010-1024-1

Shao WW, 2016, ONCOTARGETS THER, V9, P477, DOI 10.2147/OTT.S92226

SHEPPARD JR, 1984, NATURE, V308, P544, DOI 10.1038/308544a0

Shimada H, 2000, CANCER-AM CANCER SOC, V89, P1677, DOI 10.1002/1097-0142(20001015)89:8<1677::AID-CNCR5>3.0.CO;2-9

Skubitz APN, 2006, TRANSL RES, V148, P223, DOI 10.1016/j.trsl.2006.06.001

Tentzeris V, 2011, INTERACT CARDIOV TH, V12, P32, DOI 10.1510/icvts.2010.247213

Xu YP, 2012, WORLD J SURG ONCOL, V10, DOI 10.1186/1477-7819-10-173

Yarden Y, 2012, NAT REV CANCER, V12, P553, DOI 10.1038/nrc3309

Yuasa HJ, 2002, GENE, V298, P21, DOI 10.1016/S0378-1119(02)00920-4

NR 33

TC 7

Z9 7

U1 0

U2 1

PU SPANDIDOS PUBL LTD

PI ATHENS

PA POB 18179, ATHENS, 116 10, GREECE

SN 1792-1074

EI 1792-1082

J9 ONCOL LETT

JI Oncol. Lett.

PD NOV

PY 2017

VL 14

IS 5

BP 6231

EP 6237

DI 10.3892/ol.2017.6973

PG 7

WC Oncology

WE Science Citation Index Expanded (SCI-EXPANDED)

SC Oncology

GA FM5NQ

UT WOS:000415083000164

PM 29113272

OA Green Published, gold

DA 2022-05-19

ER

PT J

AU Cui, YT

Ding, M

Dong, SW

Wang, YG

Zhang, P

AF Cui, Yuantao

Ding, Min

Dong, Shangwen

Wang, Yuanguo

Zhang, Peng

TI The unusual yin-yang fashion of RIZ1/RIZ2 contributes to the progression

of esophageal squamous cell carcinoma

SO OPEN LIFE SCIENCES

LA English

DT Article

DE RIZ1; RIZ2; esophageal squamous cell carcinoma; yin-yang fashion

ID ZINC-FINGER PROTEIN; PR-DOMAIN; RETINOBLASTOMA PROTEIN; DNA METHYLATION;

BREAST-CANCER; RIZ1 GENE; EXPRESSION; HYPERMETHYLATION; CARCINOGENESIS;

PRODUCT

AB Retinoblastoma protein-interacting zinc finger gene RIZ encodes two different protein products, RIZ1 and RIZ2. Observations suggest that RIZ1 is a tumor suppressor, while RIZ2 acts as a negative regulator of RIZ1 and may play a positive role in oncogenesis. The imbalance amount of RIZ1 and RIZ2 may be involved in cancer development. In this study we detected the expression levels of RIZ1 and RIZ2 mRNA in human esophageal squamous cell carcinoma (ESCC) tissue specimens, reexpressed RIZ1 in the human ESCC cell line EC109 in which RIZ1 mRNA level was not detected, examined the changes of RIZ1 and RIZ2 mRNA expression, investigated the changes of proliferation, and apoptosis of the cells. We found that RIZ1 mRNA expression is commonly decreased or at undetectable level in human esophageal squamous cancer tissue specimens compared to the normal tissue specimens, while RIZ2 is usually expressed. With the forced expression of RIZ1, RIZ2 mRNA expression did not change, The ESCC cell proliferation was inhibited and apoptosis was induced. This unusual yin-yang fashion of RIZ1/RIZ2 may contribute to the progression of ESCC.

C1 [Cui, Yuantao; Dong, Shangwen; Wang, Yuanguo; Zhang, Peng] Tianjin Med Univ, Gen Hosp, Dept Cardiothorac Surg, Tianjin 300070, Peoples R China.

[Ding, Min] Tianjin Med Univ, Key Lab Hormones & Dev, Tianjin Key Lab Metab Dis, Minist Hlth,Tianjin Metab Dis Hosp, Tianjin 300070, Peoples R China.

[Ding, Min] Tianjin Med Univ, Tianjin Inst Endocrinol, Tianjin 300070, Peoples R China.

RP Zhang, P (corresponding author), Tianjin Med Univ, Gen Hosp, Dept Cardiothorac Surg, Tianjin 300070, Peoples R China.

EM zhp66036@163.com

RI Dong, Shangwen/G-6083-2014

OI Dong, Shangwen/0000-0003-4272-9572

FU National Natural Science Foundation of China [81201945]; Science

Foundation of Tianjin Medical University [2014KYM02]

FX This study was supported by the National Natural Science Foundation of

China (grant no. 81201945), the Science Foundation of Tianjin Medical

University (grant no. 2014KYM02).

CR Abbondanza C, 2012, J CELL PHYSIOL, V227, P964, DOI 10.1002/jcp.22803

An JY, 2005, WORLD J GASTROENTERO, V11, P2055, DOI 10.3748/wjg.v11.i14.2055

BUYSE IM, 1995, P NATL ACAD SCI USA, V92, P4467, DOI 10.1073/pnas.92.10.4467

Buyse IM, 1996, GENOMICS, V34, P119, DOI 10.1006/geno.1996.0249

Dave BJ, 1999, CLIN CANCER RES, V5, P1401

Deng QD, 2004, ONCOGENE, V23, P4903, DOI 10.1038/sj.onc.1207615

Dong SW, 2012, CLIN LAB, V58, P41

Dong SW, 2012, WORLD J GASTROENTERO, V18, P576, DOI 10.3748/wjg.v18.i6.576

Du Y, 2001, CANCER RES, V61, P8094

Enzinger PC, 2003, NEW ENGL J MED, V349, P2241, DOI 10.1056/NEJMra035010

Guo XQ, 2007, J EXP CLIN CANC RES, V26, P587

Hasegawa Y, 2007, CANCER SCI, V98, P32, DOI 10.1111/j.1349-7006.2006.00338.x

He LS, 1998, CANCER RES, V58, P4238

Huang S, 1998, J BIOL CHEM, V273, P15933, DOI 10.1074/jbc.273.26.15933

Jemal A, 2011, CA-CANCER J CLIN, V61, P69, DOI [10.3322/caac.20107, 10.3322/caac.20115]

Jiang GL, 1999, INT J CANCER, V83, P541, DOI 10.1002/(SICI)1097-0215(19991112)83:4<541::AID-IJC17>3.0.CO;2-F

Jiang GL, 2000, HISTOL HISTOPATHOL, V15, P109, DOI 10.14670/HH-15.109

Lal G, 2006, CANCER, V107, P2752, DOI 10.1002/cncr.22325

Liu LM, 1997, J BIOL CHEM, V272, P2984, DOI 10.1074/jbc.272.5.2984

Long HK, 2013, BIOCHEM SOC T, V41, P727, DOI 10.1042/BST20130028

Ooi SKT, 2008, CELL, V133, P1145, DOI 10.1016/j.cell.2008.06.009

Zeng HM, 2016, THORAC CANCER, V7, P232, DOI 10.1111/1759-7714.12322

NR 22

TC 2

Z9 2

U1 0

U2 1

PU DE GRUYTER POLAND SP Z O O

PI WARSAW

PA BOGUMILA ZUGA 32A STR, 01-811 WARSAW, MAZOVIA, POLAND

SN 2391-5412

J9 OPEN LIFE SCI

JI Open Life Sci.

PD JAN

PY 2016

VL 11

IS 1

BP 136

EP 141

DI 10.1515/biol-2016-0019

PG 6

WC Biology

WE Science Citation Index Expanded (SCI-EXPANDED)

SC Life Sciences & Biomedicine - Other Topics

GA DZ3RJ

UT WOS:000385766400010

OA gold

DA 2022-05-19

ER

PT J

AU Chen, ZX

Li, GS

Yang, LH

Liu, HC

Qin, GM

Shen, L

He, WY

Gan, TQ

Li, JJ

AF Chen, Zu-Xuan

Li, Guo-Sheng

Yang, Li-Hua

Liu, He-Chuan

Qin, Guang-Mei

Shen, Lang

He, Wei-Ying

Gan, Ting-Qing

Li, Jian-Jun

TI Upregulation of BIRC5 plays essential role in esophageal squamous cell

carcinoma

SO MATHEMATICAL BIOSCIENCES AND ENGINEERING

LA English

DT Article

DE esophageal squamous cell carcinoma; baculoviral inhibitor of apoptosis

repeat containing 5; microarray; RNA sequencing; prognosis

ID CANCER; EXPRESSION; SURVIVIN; OVEREXPRESSION; AMPLIFICATION;

EPIDEMIOLOGY; PROGNOSIS; PACKAGE; CDC20

AB Background: Esophageal squamous cell carcinoma (ESCC) is one of the most common cancers in the world, the detection and prognosis of which are still unsatisfactory. Thus, it is essential to explore the factors that may identify ESCC and evaluate the prognosis of ESCC patients. Results: Both protein and mRNA expression levels of BIRC5 are upregulated in ESCC group rather than non-ESCC group (standardized mean difference 0.05). BIRC5 expression makes it feasible to distinguish ESCC from non-ESCC (area under the curve 0.9), and its high expression is related to poor prognosis of ESCC patients (restrictive survival time difference = -0.036, p < 0.05). BIRC5 may play an important role in ESCC by influencing the cell cycle pathway, and CDK1, MAD2L and CDC20 may be the hub genes of this pathway. The transcription factors-MAZ and TFPD1 -are likely to regulate the transcription of BIRC5, which may be one of the factors for the high expression of BIRC5 in ESCC. Conclusions: The current study shows that upregulation of BIRC5 may have essential clinical value in ESCC, and contributes to the understanding of the pathogenesis of ESCC.

C1 [Chen, Zu-Xuan; Li, Guo-Sheng; Liu, He-Chuan; Qin, Guang-Mei; Shen, Lang; He, Wei-Ying; Gan, Ting-Qing] Guangxi Med Univ, Dept Med Oncol, Affiliated Hosp 2, 166 DaxueXi Rd, Nanning 530021, Guangxi Zhuang, Peoples R China.

[Yang, Li-Hua] Guangxi Med Univ, Dept Med Oncol, Affiliated Hosp 1, 6 Shuangyong Rd, Nanning 530021, Guangxi Zhuang, Peoples R China.

[Li, Jian-Jun] Guangxi Med Univ, Dept Gen Surg, Affiliated Hosp 2, 166 DaxueXi Rd, Nanning 530021, Guangxi Zhuang, Peoples R China.

RP Gan, TQ (corresponding author), Guangxi Med Univ, Dept Med Oncol, Affiliated Hosp 2, 166 DaxueXi Rd, Nanning 530021, Guangxi Zhuang, Peoples R China.; Li, JJ (corresponding author), Guangxi Med Univ, Dept Gen Surg, Affiliated Hosp 2, 166 DaxueXi Rd, Nanning 530021, Guangxi Zhuang, Peoples R China.

EM gantingqing_gxmu@163.com; lijianjunmail@163.com

FU College Student Innovation and Entrepreneurship Training Program Project

of the First Clinical Medical University of Guangxi Medical University

[2020YFYA05, 2020YFYA06]; College Student Innovation and

Entrepreneurship Training Program Project [202010598052]; Guangxi

Medical University Future Academic Stars Project [WLXSZX20081]

FX This work was supported by the College Student Innovation and

Entrepreneurship Training Program Project of the First Clinical Medical

University of Guangxi Medical University (2020YFYA05, 2020YFYA06) , the

College Student Innovation and Entrepreneurship Training Program Project

(202010598052) and the Guangxi Medical University Future Academic Stars

Project (WLXSZX20081). The results shown in the study are in part based

upon data generated by the TCGA Research Network: www.cancer.gov/tcga.

CR Abnet CC, 2018, GASTROENTEROLOGY, V154, P360, DOI 10.1053/j.gastro.2017.08.023

Arnold M, 2015, GUT, V64, P381, DOI 10.1136/gutjnl-2014-308124

Balduzzi S, 2019, EVID-BASED MENT HEAL, V22, P153, DOI 10.1136/ebmental-2019-300117

Beardmore VA, 2004, J CELL SCI, V117, P4033, DOI 10.1242/jcs.01242

Bi YH, 2020, CELL DEATH DIS, V11, DOI 10.1038/s41419-020-2492-2

Cao W, 2020, NAT COMMUN, V11, DOI 10.1038/s41467-020-17227-z

Castillo SD, 2010, J PATHOL, V222, P89, DOI 10.1002/path.2732

CHEN SW, 2020, FRONTIERS GENETICS, V0011

di Pietro M, 2018, GASTROENTEROLOGY, V154, P421, DOI 10.1053/j.gastro.2017.07.041

Arnal MJD, 2015, WORLD J GASTROENTERO, V21, P7933, DOI 10.3748/wjg.v21.i26.7933

Enzinger PC, 2003, NEW ENGL J MED, V349, P2241, DOI 10.1056/NEJMra035010

Ferlay J, 2015, INT J CANCER, V136, pE359, DOI 10.1002/ijc.29210

Ghaffari Kimia, 2016, Iranian Biomedical Journal, V20, P241, DOI 10.7508/ibj.2016.04.009

Grant CE, 2011, BIOINFORMATICS, V27, P1017, DOI 10.1093/bioinformatics/btr064

Hesari A, 2019, INT J CANCER, V144, P1215, DOI 10.1002/ijc.31947

Hu H, 2019, NUCLEIC ACIDS RES, V47, pD33, DOI 10.1093/nar/gky822

Jamali L, 2018, J CELL PHYSIOL, V233, P8538, DOI 10.1002/jcp.26850

Kamangar F, 2006, J CLIN ONCOL, V24, P2137, DOI 10.1200/JCO.2005.05.2308

Karra H, 2014, BRIT J CANCER, V110, P2905, DOI 10.1038/bjc.2014.252

Leek JT, 2012, BIOINFORMATICS, V28, P882, DOI 10.1093/bioinformatics/bts034

Li CG, 2012, PLOS ONE, V7, DOI 10.1371/journal.pone.0044764

Malhotra U, 2013, PLOS ONE, V8, DOI 10.1371/journal.pone.0078343

Recalde M, 2021, BMC MED, V19, DOI 10.1186/s12916-020-01877-3

Reichenbach ZW, 2019, ADV CANCER RES, V144, P95, DOI 10.1016/bs.acr.2019.05.004

Ritchie ME, 2015, NUCLEIC ACIDS RES, V43, DOI 10.1093/nar/gkv007

Robinson MD, 2010, BIOINFORMATICS, V26, P139, DOI 10.1093/bioinformatics/btp616

Shang XB, 2018, ONCOL LETT, V16, P3373, DOI 10.3892/ol.2018.8986

Sisinni L, 2017, J PATHOL, V243, P123, DOI 10.1002/path.4936

Stormo Gary D, 2013, Quant Biol, V1, P115

Then EO, 2020, WORLD J ONCOL, V11, P55, DOI 10.14740/wjon1254

Tian S., 2020, ANTICANCER AGENTS ME

Wang LX, 2015, PHARMACOL THERAPEUT, V151, P141, DOI 10.1016/j.pharmthera.2015.04.002

Wang Zhi, 2018, Nan Fang Yi Ke Da Xue Xue Bao, V38, P1151, DOI 10.3969/j.issn.1673-4254.2018.10.01

Warnecke-Eberz U, 2005, ONCOL REP, V13, P1241

Wasserman WW, 2004, NAT REV GENET, V5, P276, DOI 10.1038/nrg1315

Watanabe M, 2021, ESOPHAGUS-TOKYO, V18, P1, DOI 10.1007/s10388-020-00785-y

Wheatley SP, 2019, J CELL SCI, V132, DOI 10.1242/jcs.223826

Wu WJ, 2013, J TRANSL MED, V11, DOI 10.1186/1479-5876-11-142

Xia HF, 2015, CLIN CHIM ACTA, V446, P82, DOI 10.1016/j.cca.2015.04.009

Xie ZC, 2019, AM J TRANSL RES, V11, P4010

Yang Q, 2019, J EXP CLIN CANC RES, V38, DOI 10.1186/s13046-019-1374-x

Yasui K, 2002, HEPATOLOGY, V35, P1476, DOI 10.1053/jhep.2002.33683

Zhao LH, 2016, BIOMETRICS, V72, P215, DOI 10.1111/biom.12384

Zheng RB, 2019, NUCLEIC ACIDS RES, V47, pD729, DOI 10.1093/nar/gky1094

Zhou YX, 2019, FUTURE ONCOL, V15, P4181, DOI 10.2217/fon-2018-0884

NR 45

TC 0

Z9 0

U1 1

U2 3

PU AMER INST MATHEMATICAL SCIENCES-AIMS

PI SPRINGFIELD

PA PO BOX 2604, SPRINGFIELD, MO 65801-2604 USA

SN 1547-1063

EI 1551-0018

J9 MATH BIOSCI ENG

JI Math. Biosci. Eng.

PY 2021

VL 18

IS 5

BP 6941

EP 6960

DI 10.3934/mbe.2021345

PG 20

WC Mathematical & Computational Biology

WE Science Citation Index Expanded (SCI-EXPANDED)

SC Mathematical & Computational Biology

GA UF1NG

UT WOS:000688347400013

PM 34517565

OA gold

DA 2022-05-19

ER

PT J

AU Lu, T

Chen, D

Wang, YY

Sun, X

Li, SC

Miao, SC

Wo, Y

Dong, YT

Leng, XL

Du, WX

Jiao, WJ

AF Lu, Tong

Chen, Di

Wang, Yuanyong

Sun, Xiao

Li, Shicheng

Miao, Shuncheng

Wo, Yang

Dong, Yanting

Leng, Xiaoliang

Du, Wenxing

Jiao, Wenjie

TI Identification of DNA methylation-driven genes in esophageal squamous

cell carcinoma: a study based on The Cancer Genome Atlas

SO CANCER CELL INTERNATIONAL

LA English

DT Article

DE Esophageal squamous cell carcinoma; Methylation; Biomarker; TCGA

ID ABERRANT METHYLATION; PROMOTER; EPIGENOMICS; TOBACCO

AB BackgroundAberrant DNA methylations are significantly associated with esophageal squamous cell carcinoma (ESCC). In this study, we aimed to investigate the DNA methylation-driven genes in ESCC by integrative bioinformatics analysis.MethodsData of DNA methylation and transcriptome profiling were downloaded from TCGA database. DNA methylation-driven genes were obtained by methylmix R package. David database and ConsensusPathDB were used to perform gene ontology (GO) analysis and pathway analysis, respectively. Survival R package was used to analyze overall survival analysis of methylation-driven genes.ResultsTotally 26 DNA methylation-driven genes were identified by the methylmix, which were enriched in molecular function of DNA binding and transcription factor activity. Then, ABCD1, SLC5A10, SPIN3, ZNF69, and ZNF608 were recognized as significant independent prognostic biomarkers from 26 methylation-driven genes. Additionally, a further integrative survival analysis, which combined methylation and gene expression data, was identified that ABCD1, CCDC8, FBXO17 were significantly associated with patients' survival. Also, multiple aberrant methylation sites were found to be correlated with gene expression.ConclusionIn summary, we studied the DNA methylation-driven genes in ESCC by bioinformatics analysis, offering better understand of molecular mechanisms of ESCC and providing potential biomarkers precision treatment and prognosis detection.

C1 [Lu, Tong; Wang, Yuanyong; Sun, Xiao; Li, Shicheng; Miao, Shuncheng; Wo, Yang; Dong, Yanting; Leng, Xiaoliang; Du, Wenxing; Jiao, Wenjie] Qingdao Univ, Dept Thorac Surg, Affiliated Hosp, 16 Jiangsu Rd, Qingdao 266003, Peoples R China.

[Chen, Di] Qingdao Univ, Dept Gastroenterol, Affiliated Hosp, 16 Jiangsu Rd, Qingdao 266003, Peoples R China.

RP Jiao, WJ (corresponding author), Qingdao Univ, Dept Thorac Surg, Affiliated Hosp, 16 Jiangsu Rd, Qingdao 266003, Peoples R China.

EM jiaowj@qduhospital.cn

RI Wang, Yuanyong/AAE-3803-2022

OI Wang, Yuanyong/0000-0002-7753-8736

FU Shandong Provincial Natural Science Foundation, China (CN) [zr2016hm58]

FX This work was supported by Shandong Provincial Natural Science

Foundation, China (CN) (No. zr2016hm58).

CR Bell A, 2011, CANCER-AM CANCER SOC, V117, P2898, DOI 10.1002/cncr.25818

Chen ZY, 2014, SCI REP-UK, V4, DOI 10.1038/srep07035

Cheng YD, 2010, CANCER RES, V70, P6516, DOI 10.1158/0008-5472.CAN-09-4566

Dinardo CD, 2017, AM J HEMATOL, V92, pE14, DOI 10.1002/ajh.24597

Enzinger PC, 2003, NEW ENGL J MED, V349, P2241, DOI 10.1056/NEJMra035010

Fan GR, 2018, CANCER CELL INT, V18, DOI 10.1186/s12935-018-0629-5

Ganji SM, 2010, DIS ESOPHAGUS, V23, P597, DOI 10.1111/j.1442-2050.2010.01059.x

Gao CD, 2019, J CELL PHYSIOL, V234, P6519, DOI 10.1002/jcp.27389

Gevaert O, 2015, BIOINFORMATICS, V31, P1839, DOI 10.1093/bioinformatics/btv020

Gevaert O, 2015, GENOME BIOL, V16, DOI 10.1186/s13059-014-0579-8

Gloss BS, 2014, CANCER LETT, V342, P257, DOI 10.1016/j.canlet.2011.12.036

Green JA, 2000, DIGEST DIS SCI, V45, P2367, DOI 10.1023/A:1005638924929

Harris MA, 2006, NUCLEIC ACIDS RES, V34, pD322, DOI 10.1093/nar/gkj021

Huang FL, 2018, ASIAN J SURG, V41, P210, DOI 10.1016/j.asjsur.2016.10.005

Jones PA, 2007, CELL, V128, P683, DOI 10.1016/j.cell.2007.01.029

Kamburov A, 2013, NUCLEIC ACIDS RES, V41, pD793, DOI 10.1093/nar/gks1055

Katarzyna T, 2015, CONT ONCOLOGY, V19, P68, DOI DOI 10.5114/W0.2014.47136

Kim MC, 2016, J CANCER, V7, P1668, DOI 10.7150/jca.15423

Kuwano H, 2015, ESOPHAGUS-TOKYO, V12, P1, DOI 10.1007/s10388-014-0465-1

Lee CH, 2007, EUR J CANCER, V43, P1188, DOI 10.1016/j.ejca.2007.01.039

Lee EJ, 2006, EUR J CANCER, V42, P972, DOI 10.1016/j.ejca.2006.01.021

Morris MR, 2011, ONCOGENE, V30, P1390, DOI 10.1038/onc.2010.525

Ohashi S, 2015, GASTROENTEROLOGY, V149, P1700, DOI 10.1053/j.gastro.2015.08.054

Pangeni RP, 2015, CLIN EPIGENETICS, V7, DOI 10.1186/s13148-015-0089-x

Roy R, 2019, INT J CANCER, V144, P1160, DOI 10.1002/ijc.31755

Sandoval J, 2012, CURR OPIN GENET DEV, V22, P50, DOI 10.1016/j.gde.2012.02.008

Shi B, 2018, AM J REPROD IMMUNOL, V79, DOI 10.1111/aji.12779

Short MW, 2017, AM FAM PHYSICIAN, V95, P22

Testa Ugo, 2017, Medicines (Basel), V4, DOI 10.3390/medicines4030067

VAUGHAN TL, 1995, CANCER EPIDEM BIOMAR, V4, P85

Veugelers PJ, 2006, DIS ESOPHAGUS, V19, P321, DOI 10.1111/j.1442-2050.2006.00602.x

Vogelsang M, 2014, J CANCER RES CLIN, V140, P1825, DOI 10.1007/s00432-014-1736-x

Vuillemenot BR, 2006, MOL CANCER RES, V4, P267, DOI 10.1158/1541-7786.MCR-05-0218

Wu L, 2014, PLOS ONE, V9, DOI 10.1371/journal.pone.0095509

Zhao JJ, 2014, TUMOR BIOL, V35, P10085, DOI 10.1007/s13277-014-2276-3

NR 35

TC 31

Z9 31

U1 0

U2 8

PU BMC

PI LONDON

PA CAMPUS, 4 CRINAN ST, LONDON N1 9XW, ENGLAND

SN 1475-2867

J9 CANCER CELL INT

JI Cancer Cell Int.

PD MAR 6

PY 2019

VL 19

AR 52

DI 10.1186/s12935-019-0770-9

PG 13

WC Oncology

WE Science Citation Index Expanded (SCI-EXPANDED)

SC Oncology

GA HO3EX

UT WOS:000460803400001

PM 30886542

OA Green Published, gold

DA 2022-05-19

ER

PT J

AU Moody, S

Senkin, S

Islam, SMA

Wang, JW

Nasrollahzadeh, D

Penha, RCC

Fitzgerald, S

Bergstrom, EN

Atkins, J

He, YD

Khandekar, A

Smith-Byrne, K

Carreira, C

Gaborieau, V

Latimer, C

Thomas, E

Abnizova, I

Bucciarelli, PE

Jones, D

Teague, JW

Abedi-Ardekani, B

Serra, S

Scoazec, JY

Saffar, H

Azmoudeh-Ardalan, F

Sotoudeh, M

Nikmanesh, A

Poustchi, H

Niavarani, A

Gharavi, S

Eden, M

Richman, P

Campos, LS

Fitzgerald, RC

Ribeiro, LF

Soares-Lima, SC

Dzamalala, C

Mmbaga, BT

Shibata, T

Menya, D

Goldstein, AM

Hu, N

Malekzadeh, R

Fazel, A

McCormack, V

McKay, J

Perdomo, S

Scelo, G

Chanudet, E

Humphreys, L

Alexandrov, LB

Brennan, P

Stratton, MR

AF Moody, Sarah

Senkin, Sergey

Islam, S. M. Ashiqul

Wang, Jingwei

Nasrollahzadeh, Dariush

Penha, Ricardo Cortez Cardoso

Fitzgerald, Stephen

Bergstrom, Erik N.

Atkins, Joshua

He, Yudou

Khandekar, Azhar

Smith-Byrne, Karl

Carreira, Christine

Gaborieau, Valerie

Latimer, Calli

Thomas, Emily

Abnizova, Irina

Bucciarelli, Pauline E.

Jones, David

Teague, Jon W.

Abedi-Ardekani, Behnoush

Serra, Stefano

Scoazec, Jean-Yves

Saffar, Hiva

Azmoudeh-Ardalan, Farid

Sotoudeh, Masoud

Nikmanesh, Arash

Poustchi, Hossein

Niavarani, Ahmadreza

Gharavi, Samad

Eden, Michael

Richman, Paul

Campos, Lia S.

Fitzgerald, Rebecca C.

Ribeiro, Luis Felipe

Soares-Lima, Sheila Coelho

Dzamalala, Charles

Mmbaga, Blandina Theophil

Shibata, Tatsuhiro

Menya, Diana

Goldstein, Alisa M.

Hu, Nan

Malekzadeh, Reza

Fazel, Abdolreza

McCormack, Valerie

McKay, James

Perdomo, Sandra

Scelo, Ghislaine

Chanudet, Estelle

Humphreys, Laura

Alexandrov, Ludmil B.

Brennan, Paul

Stratton, Michael R.

TI Mutational signatures in esophageal squamous cell carcinoma from eight

countries with varying incidence

SO NATURE GENETICS

LA English

DT Article

ID RISK; CANCER; ASSOCIATION; LANDSCAPE; TOBACCO; CONSUMPTION

AB The incidence of esophageal squamous cell carcinoma varies significantly across different geographical regions. Mutational signature analysis of tumors sampled from high- and low-incidence areas suggests that these variations may not be explained by mutagenic exposures.

Esophageal squamous cell carcinoma (ESCC) shows remarkable variation in incidence that is not fully explained by known lifestyle and environmental risk factors. It has been speculated that an unknown exogenous exposure(s) could be responsible. Here we combine the fields of mutational signature analysis with cancer epidemiology to study 552 ESCC genomes from eight countries with varying incidence rates. Mutational profiles were similar across all countries studied. Associations between specific mutational signatures and ESCC risk factors were identified for tobacco, alcohol, opium and germline variants, with modest impacts on mutation burden. We find no evidence of a mutational signature indicative of an exogenous exposure capable of explaining differences in ESCC incidence. Apolipoprotein B mRNA-editing enzyme, catalytic polypeptide-like (APOBEC)-associated mutational signatures single-base substitution (SBS)2 and SBS13 were present in 88% and 91% of cases, respectively, and accounted for 25% of the mutation burden on average, indicating that APOBEC activation is a crucial step in ESCC tumor development.

C1 [Moody, Sarah; Wang, Jingwei; Fitzgerald, Stephen; Latimer, Calli; Thomas, Emily; Abnizova, Irina; Bucciarelli, Pauline E.; Jones, David; Teague, Jon W.; Humphreys, Laura; Stratton, Michael R.] Wellcome Genome Campus, Canc Ageing & Somat Mutat, Wellcome Sanger Inst, Cambridge, England.

[Senkin, Sergey; Nasrollahzadeh, Dariush; Penha, Ricardo Cortez Cardoso; Atkins, Joshua; Smith-Byrne, Karl; Gaborieau, Valerie; Abedi-Ardekani, Behnoush; McKay, James; Perdomo, Sandra; Scelo, Ghislaine; Chanudet, Estelle; Brennan, Paul] Int Agcy Res Canc IARC WHO, Genom Epidemiol Branch, Lyon, France.

[Islam, S. M. Ashiqul; Bergstrom, Erik N.; He, Yudou; Khandekar, Azhar; Alexandrov, Ludmil B.] UC San Diego Hlth, Moores Canc Ctr, La Jolla, CA USA.

[Islam, S. M. Ashiqul; Bergstrom, Erik N.; He, Yudou; Khandekar, Azhar; Alexandrov, Ludmil B.] Univ Calif, Dept Cellular & Mol Med, La Jolla, CA USA.

[Islam, S. M. Ashiqul; Bergstrom, Erik N.; He, Yudou; Khandekar, Azhar; Alexandrov, Ludmil B.] Univ Calif, Dept Bioengn, La Jolla, CA USA.

[Nasrollahzadeh, Dariush; Sotoudeh, Masoud; Nikmanesh, Arash; Poustchi, Hossein; Niavarani, Ahmadreza; Gharavi, Samad; Malekzadeh, Reza] Univ Tehran Med Sci, Shariati Hosp, Digest Oncol Res Ctr, Digest Dis Res Inst, Tehran, Iran.

[Carreira, Christine] Int Agcy Res Canc IARC WHO, Evidence Synth & Classificat Branch, Lyon, France.

[Serra, Stefano] Univ Hlth Network, Toronto, ON, Canada.

[Scoazec, Jean-Yves] Gustave Roussy, Dept Lab Med & Pathol, Paris, France.

[Saffar, Hiva] Univ Tehran Med Sci, Shariati Hosp, Dept Pathol, Tehran, Iran.

[Azmoudeh-Ardalan, Farid] Univ Tehran Med Sci, Liver Transplantat Res Ctr, Imam Khomeini Hosp Complex, Tehran, Iran.

[Eden, Michael] Cambridge Biomed Campus, Cambridge Univ Hosp NHS Fdn Trust, Cambridge, England.

[Richman, Paul] Hemel Hempstead Gen Hosp, Histopathol Dept, Hemel Hempstead, England.

[Campos, Lia S.] West Suffolk NHS Fdn Trust, Bury St Edmunds, Suffolk, England.

[Fitzgerald, Rebecca C.] Univ Cambridge, MRC Canc Unit, Cambridge, England.

[Ribeiro, Luis Felipe; Soares-Lima, Sheila Coelho] Brazilian Natl Canc Inst, Rio De Janeiro, Brazil.

[Dzamalala, Charles] Univ Malawi, Zomba, Malawi.

[Mmbaga, Blandina Theophil] Kilimanjaro Christian Med Ctr, Kilimanjaro Clin Res Inst, Moshi, Tanzania.

[Mmbaga, Blandina Theophil] Kilimanjaro Christian Med Univ Coll, Moshi, Tanzania.

[Shibata, Tatsuhiro] Natl Canc Ctr, Div Canc Genom, Res Inst, Tokyo, Japan.

[Menya, Diana] Moi Univ, Mombasa, Kenya.

[Goldstein, Alisa M.; Hu, Nan] NCI, Div Canc Epidemiol & Genet, NIH, Rockville, MD USA.

[Fazel, Abdolreza] Golestan Univ Med Sci, Golestan Res Ctr Gastroenterol & Hepatol, Gorgan, Golestan, Iran.

[McCormack, Valerie] Int Agcy Res Canc IARC WHO, Environm & Lifestyle Epidemiol Branch, Lyon, France.

[Scelo, Ghislaine] Univ Turin, Dept Med Sci, Canc Epidemiol Unit, Turin, Italy.

RP Stratton, MR (corresponding author), Wellcome Genome Campus, Canc Ageing & Somat Mutat, Wellcome Sanger Inst, Cambridge, England.

EM mrs@sanger.ac.uk

RI Abedi-Ardekani, Behnoush/O-7829-2016; Niavarani, Ahmadreza/B-9946-2017

OI Abedi-Ardekani, Behnoush/0000-0002-0980-0587; Perdomo,

Sandra/0000-0001-7740-8141; Wang, Jingwei/0000-0002-0201-2369;

Niavarani, Ahmadreza/0000-0001-7773-6778; Senkin,

Sergey/0000-0001-5848-005X; Thomas, Emily/0000-0002-4563-8695;

Smith-Byrne, Karl/0000-0002-1932-7463

FU Cancer Grand Challenges Mutographs team award - Cancer Research UK

[C98/A24032]; Wellcome Trust [206194]; Alfred P. Sloan Research

Fellowship; Packard Fellowship for Science and Engineering; Wereld

Kanker Onderzoek Fonds (World Cancer Research Fund) [2018/1795]; IARC

Branch of Environment and Lifestyle Epidemiology; Medical Research

Council [RG84369]; NIHR Cambridge Biomedical Research Centre

[BRC-1215-20014]; Cancer Research UK [RG81771/84119]; Practical Research

Project for Innovative Cancer Control from the Japan Agency for Medical

Research and Development [JP20ck0106547h0001]; Intramural Research

Program of the Division of Cancer Epidemiology and Genetics, National

Cancer Institute, National Institutes of Health; IARC; [R21CA191965]

FX This article is dedicated to the memory of Eleanor Wilde. We thank L.

O'Neill, J. Hewinson, K. Roberts, N. Smerdon, J. Mack, E. Gray and the

staff of DNA Pipelines at the Wellcome Sanger Institute for their

contribution. We are grateful for the support provided by the IARC

ESCCAPE team (M. Odour, N. Kigen, F. Some (Eldoret), G. Mushi (Moshi),

M. Suwedi, T. Soloman, R. Malamba (Blantyre) and D. Middleton), S.

Magat, P. Boutarin, D. Middleton and A. Hajimohammadsadegh, as well as

IARC General Services, including the Laboratory Services and Biobank

team led by Z. Kozlakidis, the Section of Support to Research overseen

by T. Landesz and the Evidence Synthesis and Classification Section led

by I. Cree, under IARC regular budget funding. We thank C. Giffen, P.

Taylor and A. Hutchinson for help with data, sample preparation and

processing; and I. Soerjomataram for assistance with Fig. 1a. We also

thank P. Campbell, I. Martincorena, T. Butler, L. Moore, D.

Leongamornlet, P. Robinson, T. Coorens, A. Steif, S. Cheema, B. Otlu, C.

Lallemand, H. Renard, T. Cholin, P. Chopard, M. Vallee, M. Milojevic, M.

Blanks and M. McCord for useful discussions. We also thank all patients

involved in this study. This work was supported by a Cancer Grand

Challenges Mutographs team award funded by Cancer Research UK

(C98/A24032) awarded to M.R.S., P.B. and L.B.A. Work at the Wellcome

Sanger Institute was also supported by the Wellcome Trust (grant number

206194), and work at the IARC/WHO was supported by regular budget

funding. L.B.A. is an Abeloff V Scholar and is supported by an Alfred P.

Sloan Research Fellowship. Research at UC San Diego was also supported

by a Packard Fellowship for Science and Engineering to L.B.A. The work

was also partly funded by R21CA191965 (D.M. and V.M.), Wereld Kanker

Onderzoek Fonds (World Cancer Research Fund) (2018/1795) and the IARC

Branch of Environment and Lifestyle Epidemiology (V.M.). The work of

R.C.C.P. reported in this paper was undertaken during the tenure of an

IARC Postdoctoral Fellowship at the International Agency for Research on

Cancer. The laboratory of R.C.F. is funded by a Core Programme Grant

from the Medical Research Council (RG84369); this research was supported

by the NIHR Cambridge Biomedical Research Centre (BRC-1215-20014); and

OCCAMS2 was funded by a Programme Grant from Cancer Research UK

(RG81771/84119). The work was also partly funded by the Practical

Research Project for Innovative Cancer Control from the Japan Agency for

Medical Research and Development (JP20ck0106547h0001 to T.S.). A.M.G.

and N.H. were supported by the Intramural Research Program of the

Division of Cancer Epidemiology and Genetics, National Cancer Institute,

National Institutes of Health. For the purpose of open access, the

authors have applied a CC BY public copyright license to any author

accepted manuscript version arising from this submission. Disclaimer:

when authors are identified as personnel of the International Agency for

Research on Cancer or the World Health Organization, the authors alone

are responsible for the views expressed in this article, and they do not

necessarily represent the decisions, policy or views of the

International Agency for Research on Cancer or the World Health

Organization.

CR Abnet CC, 2018, GASTROENTEROLOGY, V154, P360, DOI 10.1053/j.gastro.2017.08.023

Akbari MR, 2008, ONCOGENE, V27, P1290, DOI 10.1038/sj.onc.1210739

Alexandrov LB, 2020, NATURE, V578, P94, DOI 10.1038/s41586-020-1943-3

Alexandrov LB, 2016, SCIENCE, V354, P618, DOI 10.1126/science.aag0299

Alexandrov LB, 2015, NAT GENET, V47, P1402, DOI 10.1038/ng.3441

Arnold M, 2015, GUT, V64, P381, DOI 10.1136/gutjnl-2014-308124

Auton A, 2015, NATURE, V526, P68, DOI 10.1038/nature15393

Balmain A, 2020, NAT GENET, V52, P1139, DOI 10.1038/s41588-020-00727-5

Bergmann Ewa A, 2016, Bioinformatics, V32, P3196, DOI 10.1093/bioinformatics/btw389

Bergstrom EN, 2020, BMC BIOINFORMATICS, V21, DOI 10.1186/s12859-020-03772-3

Bergstrom EN, 2019, BMC GENOMICS, V20, DOI 10.1186/s12864-019-6041-2

Bray F, 2018, CA-CANCER J CLIN, V68, P394, DOI 10.3322/caac.21492

Buisson R, 2019, SCIENCE, V364, P1251, DOI 10.1126/science.aaw2872

Campbell PJ, 2020, NATURE, V578, P82, DOI 10.1038/s41586-020-1969-6

Chang CC, 2015, GIGASCIENCE, V4, DOI 10.1186/s13742-015-0047-8

Chang J, 2017, NAT COMMUN, V8, DOI 10.1038/ncomms15290

Cui R, 2009, GASTROENTEROLOGY, V137, P1768, DOI 10.1053/j.gastro.2009.07.070

Degasperi A, 2020, NAT CANCER, V1, P249, DOI 10.1038/s43018-020-0027-5

Deng JY, 2017, NAT COMMUN, V8, DOI 10.1038/s41467-017-01730-x

Gao YB, 2014, NAT GENET, V46, P1097, DOI 10.1038/ng.3076

Gao Y, 2011, CANCER EPIDEMIOL, V35, pE91, DOI 10.1016/j.canep.2011.06.006

Islam S. M. A, UNCOVERING NOVEL MUT, DOI [10.1101/2020.12.13.422570(2020, DOI 10.1101/2020.12.13.422570(2020]

Jones David, 2016, Curr Protoc Bioinformatics, V56, DOI 10.1002/cpbi.20

Kidd JM, 2007, PLOS GENET, V3, DOI 10.1371/journal.pgen.0030063

Kim J, 2017, NATURE, V541, P169, DOI 10.1038/nature20805

Kim S, 2018, NAT METHODS, V15, P591, DOI 10.1038/s41592-018-0051-x

Ko JMY, 2020, INT J CANCER, V146, P1042, DOI 10.1002/ijc.32619

Li XC, 2018, ANN ONCOL, V29, P938, DOI 10.1093/annonc/mdy011

Liu X, 2017, GASTROENTEROLOGY, V153, P166, DOI 10.1053/j.gastro.2017.03.033

Macgregor S, 2009, HUM MOL GENET, V18, P580, DOI 10.1093/hmg/ddn372

Mangalaparthi KK, 2020, FRONT ONCOL, V10, DOI 10.3389/fonc.2020.01457

Martincorena I, 2018, SCIENCE, V362, P911, DOI 10.1126/science.aau3879

Martincorena I, 2017, CELL, V171, P1029, DOI 10.1016/j.cell.2017.09.042

Mas-Ponte D, 2020, NAT GENET, V52, P958, DOI 10.1038/s41588-020-0674-6

McCormack VA, 2017, INT J CANCER, V140, P259, DOI 10.1002/ijc.30292

Mello FW, 2019, CLIN ORAL INVEST, V23, P2849, DOI 10.1007/s00784-019-02958-1

Middlebrooks CD, 2016, NAT GENET, V48, P1330, DOI 10.1038/ng.3670

Murphy G, 2017, ANN ONCOL, V28, P2086, DOI 10.1093/annonc/mdx279

Nasrollahzadeh D, 2008, BRIT J CANCER, V98, P1857, DOI 10.1038/sj.bjc.6604369

Nik-Zainal S, 2016, NATURE, V534, P47, DOI 10.1038/nature17676

Nik-Zainal S, 2014, NAT GENET, V46, P487, DOI 10.1038/ng.2955

Nik-Zainal S, 2012, CELL, V149, P979, DOI 10.1016/j.cell.2012.04.024

Raine Keiran M, 2015, Curr Protoc Bioinformatics, V52, DOI 10.1002/0471250953.bi1507s52

Riva L, 2020, NAT GENET, V52, P1189, DOI 10.1038/s41588-020-0692-4

Sawada G, 2016, GASTROENTEROLOGY, V150, P1171, DOI 10.1053/j.gastro.2016.01.035

Scelo G, 2014, NAT COMMUN, V5, DOI 10.1038/ncomms6135

Senkin S, MSA REPRODUCIBLE MUT, DOI [10.1101/2020.12.14.422764(2020, DOI 10.1101/2020.12.14.422764(2020]

Sheikh M, 2019, GASTROENTEROLOGY, V156, P1416, DOI 10.1053/j.gastro.2018.12.024

Song YM, 2014, NATURE, V509, P91, DOI 10.1038/nature13176

Warnakulasuriya S, 2020, LANCET ONCOL, V21, P1407, DOI 10.1016/S1470-2045(20)30611-2

Wu C, 2011, NAT GENET, V43, P679, DOI 10.1038/ng.849

Yokoyama A, 2019, NATURE, V565, P312, DOI 10.1038/s41586-018-0811-x

Zhang L, 2015, AM J HUM GENET, V96, P597, DOI 10.1016/j.ajhg.2015.02.017

NR 53

TC 8

Z9 8

U1 10

U2 14

PU NATURE PORTFOLIO

PI BERLIN

PA HEIDELBERGER PLATZ 3, BERLIN, 14197, GERMANY

SN 1061-4036

EI 1546-1718

J9 NAT GENET

JI Nature Genet.

PD NOV

PY 2021

VL 53

IS 11

BP 1553

EP +

DI 10.1038/s41588-021-00928-6

EA OCT 2021

PG 27

WC Genetics & Heredity

WE Science Citation Index Expanded (SCI-EXPANDED)

SC Genetics & Heredity

GA XU6GD

UT WOS:000708396000001

PM 34663923

OA Green Submitted

DA 2022-05-19

ER

PT J

AU Wang, S

Wang, Z

Liu, XY

Yang, Y

Shi, M

Sun, ZG

AF Wang, Shuai

Wang, Zhou

Liu, Xiangyan

Yang, Yu

Shi, Mo

Sun, Zhenguo

TI Prognostic significance of Ku80 in pT2N0M0 esophageal squamous cell

carcinoma after Ivor-Lewis esophagectomy

SO TUMOR BIOLOGY

LA English

DT Article

DE Prognosis; Ku80; Esophageal squamous cell carcinoma; Esophagectomy

ID AMERICAN JOINT COMMITTEE; CANCER STAGING SYSTEM; DISEASE-FREE SURVIVAL;

BREAST-CANCER; REPAIR PROTEIN; ADJUVANT RADIOTHERAPY; COLORECTAL-CANCER;

EXPRESSION; PROLIFERATION; AUTOANTIGEN

AB Recent studies have shown that Ku80, a DNA repair protein, was involved in progression of malignant tumors. This study aimed to clarify the clinicopathological significance and prognostic value of Ku80 in pT2N0M0 esophageal squamous cell carcinoma (ESCC). We enrolled 217 patients with pT2N0M0 midthoracic ESCC who had undergone Ivor-Lewis esophagectomy. The expression profile of Ku80 was examined by immunohistochemistry. The results were correlated with the clinicopathological variables, overall survival (OS) and disease-free survival (DFS), in pT2N0M0 ESCC patients. The expression of Ku80 were higher in ESCC tissues than the corresponding health esophageal mucosa (P < 0.001). Clinically, the Ku80 expression levels were significantly related to tumor size (P = 0.018), differentiation degree (P = 0.010), and tumor-node-metastasis (TNM) stage (P = 0.001). Subsequent multivariate analysis demonstrated that tumor size, differentiation degree, TNM stage, and Ku80 expression were independent prognostic factors for the OS and the DFS of pT2N0M0 ESCC patients. Our data indicated that Ku80 expression level associates with key clinicopathological features and is an independent predictor of the OS and the DFS in pT2N0M0 ESCC patients.

C1 [Wang, Shuai; Wang, Zhou; Liu, Xiangyan; Shi, Mo; Sun, Zhenguo] Shandong Univ, Prov Hosp, Dept Thorac Surg, Jinan 250021, Shandong, Peoples R China.

[Yang, Yu] Shandong Univ, Prov Hosp, Dept Pathol, Jinan 250021, Shandong, Peoples R China.

RP Wang, Z (corresponding author), Shandong Univ, Prov Hosp, Dept Thorac Surg, 324 Jingwu Rd, Jinan 250021, Shandong, Peoples R China.

EM wz620226@hotmail.com

RI guo, sun/C-4291-2014

OI Wang, Shuai/0000-0001-8503-9024

CR Aleskandarany M, 2015, BREAST CANCER RES TR, V150, P81, DOI 10.1007/s10549-015-3306-6

Alshareeda AT, 2013, BREAST CANCER RES TR, V139, P301, DOI 10.1007/s10549-013-2542-x

Chen G, 2009, ANN THORAC SURG, V87, P1697, DOI 10.1016/j.athoracsur.2009.03.060

Chen G, 2009, EUR J CARDIO-THORAC, V35, P1, DOI 10.1016/j.ejcts.2008.09.002

D'Annoville T, 2013, EUR J CARDIO-THORAC, V44, pE207, DOI 10.1093/ejcts/ezt332

Ferlay J, 2010, INT J CANCER, V127, P2893, DOI 10.1002/ijc.25516

Gao YJ, 2000, NATURE, V404, P897, DOI 10.1038/35009138

Grabsch H, 2006, CLIN CANCER RES, V12, P1494, DOI 10.1158/1078-0432.CCR-05-2105

Green AR, 2015, BREAST CANCER RES TR, V149, P353, DOI 10.1007/s10549-014-3230-1

Groselj B, 2013, RADIOTHER ONCOL, V108, P429, DOI 10.1016/j.radonc.2013.06.021

Hosoi Y, 2004, INT J ONCOL, V25, P461

Hou X, 2014, ANN SURG ONCOL, V21, P337, DOI 10.1245/s10434-013-3265-2

Kamangar F, 2009, GASTROENTEROL CLIN N, V38, P27, DOI 10.1016/j.gtc.2009.01.004

Kamangar F, 2006, J CLIN ONCOL, V24, P2137, DOI 10.1200/JCO.2005.05.2308

Koike M, 2001, J BIOL CHEM, V276, P11167, DOI 10.1074/jbc.M010902200

Komuro Y, 2002, CANCER-AM CANCER SOC, V95, P1199, DOI 10.1002/cncr.10807

Kruer TL, 2013, CLIN BIOCHEM, V46, P1739, DOI 10.1016/j.clinbiochem.2013.07.005

Leifler KS, 2010, INT J ONCOL, V37, P1547, DOI 10.3892/ijo_00000808

Lim JW, 2002, J BIOL CHEM, V277, P46093, DOI 10.1074/jbc.M206603200

Lu YF, 2014, ONCOTARGETS THER, V7, P1955, DOI 10.2147/OTT.S67814

Ma QS, 2012, J EXP CLIN CANC RES, V31, DOI 10.1186/1756-9966-31-99

Mariette C, 2007, LANCET ONCOL, V8, P545, DOI 10.1016/S1470-2045(07)70172-9

Monferran S, 2004, EMBO J, V23, P3758, DOI 10.1038/sj.emboj.7600403

Muller C, 2005, CELL CYCLE, V4, P438, DOI 10.4161/cc.4.3.1565

Sobin LH., 2009, TNM CLASSIFICATION M, V7

Song YM, 2012, ANN SURG ONCOL, V19, P3606, DOI 10.1245/s10434-012-2419-y

Teoh G, 1998, J CLIN INVEST, V101, P1379

Tong WM, 2002, CANCER RES, V62, P6990

Tuteja R, 2000, CRIT REV BIOCHEM MOL, V35, P1, DOI 10.1080/10409230091169177

Wang Zhou, 2004, Zhonghua Wai Ke Za Zhi, V42, P68

Yang QS, 2008, J RADIAT RES, V49, P399, DOI 10.1269/jrr.07096

NR 31

TC 4

Z9 4

U1 0

U2 3

PU SAGE PUBLICATIONS LTD

PI LONDON

PA 1 OLIVERS YARD, 55 CITY ROAD, LONDON EC1Y 1SP, ENGLAND

SN 1010-4283

EI 1423-0380

J9 TUMOR BIOL

JI Tumor Biol.

PD AUG

PY 2015

VL 36

IS 8

BP 6181

EP 6189

DI 10.1007/s13277-015-3302-9

PG 9

WC Oncology

WE Science Citation Index Expanded (SCI-EXPANDED)

SC Oncology

GA CP9DL

UT WOS:000360193800054

PM 25758053

DA 2022-05-19

ER

PT J

AU Gao, DN

Zhang, ZC

Yang, QW

Li, BS

AF Gao, Dongni

Zhang, Zicheng

Yang, Qiwei

Li, Baosheng

TI Multiregion sequencing and subclonal analysis reveal intratumoral

heterogeneity in esophageal squamous cell carcinoma

SO JOURNAL OF CANCER RESEARCH AND THERAPEUTICS

LA English

DT Article

DE Esophageal squamous cell carcinoma; intratumoral heterogeneity; tumor

evolution

ID EVOLUTION

AB Purpose: The aim of this study was to investigate intratumoral genomic heterogeneity and subclonal structure of esophageal squamous cell carcinoma (ESCC). Materials and Methods: Multiregion whole-exome sequencing was performed on 24 surgically acquired tumor samples from five untreated ESCC patients collected in 2019 to determine the heterogeneity of mutational landscape within tumors. Phylogenetic analysis and mutation process analysis were used to explore the distribution and dynamic changes of mutation spectrum, and subclone analysis was used to explore the subclonal composition and spatial structure of ESCC. Results: An average of 60.2% of mutations were found heterogenous. TP53 and NOTCH1 mutations were confirmed to be early events, and mutations unique in different tumor regions showed a pattern of branching evolution. A large proportion of mutations were associated with abnormal activity of the apolipoprotein B mRNA editing enzyme, catalytic polypeptide-like (APOBEC) family, and significant differences in mutation types between trunk and branch variants were found. Subclonal structure exhibited spatial correspondence and spatial limitations, and different genomic features were characterized between close and distant clones. Conclusions: There is significant intratumoral genomic heterogeneity in the five ESCCs, and their subclonal structure is related to spatial locations.

C1 [Gao, Dongni; Yang, Qiwei] Shandong Univ, Dept Clin Med, Cheeloo Coll Med, Jinan, Shandong, Peoples R China.

[Gao, Dongni; Zhang, Zicheng; Yang, Qiwei; Li, Baosheng] Shandong First Med Univ & Shandong Acad Med Sci, Shandong Canc Hosp & Inst, Dept Radiat Oncol, Jinan, Shandong, Peoples R China.

[Zhang, Zicheng] Guangzhou Univ Chinese Med, Clin Med Coll 4, Dept Radiat Oncol, Shenzhen Tradit Chinese Med Hosp, Shenzhen, Guangdong, Peoples R China.

RP Li, BS (corresponding author), 440 Jiyan Rd, Jinan 250117, Shandong, Peoples R China.

EM bsli@sdfmu.edu.cn

FU Taishan Scholar Construction Project [ts20120505]; National Natural

Science Foundation of China [81874224]; Academic promotion program of

Shandong First Medical University [2019LJ004]

FX 1. Taishan Scholar Construction Project, Grant/Award Number: ts20120505;

2. National Natural Science Foundation of China, Grant/Award Number:

81874224; 3. Academic promotion program of Shandong First Medical

University, Grant/Award Number: 2019LJ004.

CR Carter SL, 2012, NAT BIOTECHNOL, V30, P413, DOI 10.1038/nbt.2203

Chen XX, 2017, NAT COMMUN, V8, DOI 10.1038/s41467-017-00650-0

Cheng CX, 2016, AM J HUM GENET, V98, P256, DOI 10.1016/j.ajhg.2015.12.013

Davis A, 2017, BBA-REV CANCER, V1867, P151, DOI 10.1016/j.bbcan.2017.01.003

Gao YB, 2014, NAT GENET, V46, P1097, DOI 10.1038/ng.3076

Hao JJ, 2016, NAT GENET, V48, P1500, DOI 10.1038/ng.3683

Li H, 2009, BIOINFORMATICS, V25, P1754, DOI 10.1093/bioinformatics/btp324

McDonald KA, 2019, ANN SURG ONCOL, V26, P2191, DOI 10.1245/s10434-019-07338-3

Miura S, 2020, SCI REP-UK, V10, DOI 10.1038/s41598-020-59006-2

Miura S, 2018, BIOINFORMATICS, V34, P4017, DOI 10.1093/bioinformatics/bty469

Mroz EA, 2020, CANCER-AM CANCER SOC, V126, P1895, DOI 10.1002/cncr.32742

Pfeifer GP, 2003, MUTAT RES-FUND MOL M, V526, P39, DOI 10.1016/S0027-5107(03)00013-7

Schliep KP, 2011, BIOINFORMATICS, V27, P592, DOI 10.1093/bioinformatics/btq706

Swanton C, 2015, CANCER DISCOV, V5, P704, DOI 10.1158/2159-8290.CD-15-0344

Wang S, 2021, PLOS GENET, V17, P1

Yates LR, 2015, NAT MED, V21, P751, DOI 10.1038/nm.3886

Zeng HM, 2016, THORAC CANCER, V7, P232, DOI 10.1111/1759-7714.12322

Zeng HM, 2015, INT J CANCER, V136, P1921, DOI 10.1002/ijc.29227

NR 18

TC 0

Z9 0

U1 0

U2 1

PU WOLTERS KLUWER MEDKNOW PUBLICATIONS

PI MUMBAI

PA WOLTERS KLUWER INDIA PVT LTD , A-202, 2ND FLR, QUBE, C T S NO 1498A-2

VILLAGE MAROL, ANDHERI EAST, MUMBAI, 400059, INDIA

SN 0973-1482

EI 1998-4138

J9 J CANCER RES THER

JI J. Canc. Res. Ther.

PD JUL

PY 2021

VL 17

IS 3

BP 756

EP +

DI 10.4103/jcrt.jcrt_270_21

PG 11

WC Oncology

WE Science Citation Index Expanded (SCI-EXPANDED)

SC Oncology

GA TL8TR

UT WOS:000675128300022

PM 34269310

OA gold

DA 2022-05-19

ER

PT J

AU Zhang, L

Qin, B

Zou, BC

Wang, SH

Quan, XJ

Wang, JH

Zhao, HL

AF Zhang, Li

Qin, Bin

Zou, Baicang

Wang, Shenhao

Quan, Xiaojing

Wang, Jinhai

Zhao, Hongli

TI Knockdown of TRIM15 inhibits the proliferation, migration and invasion

of esophageal squamous cell carcinoma cells through inactivation of the

Wnt/beta-catenin signaling pathway

SO JOURNAL OF BIOENERGETICS AND BIOMEMBRANES

LA English

DT Article

DE Esophageal squamous cell carcinoma (ESCC); TRIM15;

Epithelial-mesenchymal transition (EMT); Metastasis; Wnt/beta-catenin

pathway

AB TRIM15 is a member of tripartite motif-containing protein (TRIM) protein family, which plays important roles in several cancers. The aim of the present study was to evaluate the role of TRIM15 in esophageal squamous cell carcinoma (ESCC). Our results showed that TRIM15 was upregulated in human ESCC tissues and cell lines. In vitro studies showed that knockdown of TRIM15 significantly inhibited the proliferation, migration, and invasion of ESCC cells. Knockdown of TRIM15 caused a significant increase in E-cadherin expression, as well as decreases in expression of N-cadherin and Vimentin proteins. Moreover, in vivo assay proved that tumor growth was suppressed by knockdown of TRIM15. Furthermore, the protein expression levels of beta-catenin, C-myc, and CyclinD1 were markedly decreased in sh-TRIM15-infected ESCC cells. Additionally, treatment with LiCl reversed the inhibitory effects of TRIM15 knockdown on ESCC cells. In conclusion, these findings indicated that knockdown of TRIM15 blocked the growth and metastasis of ESCC in part through inhibiting the Wnt/beta-catenin signaling pathway. Thus, TRIM15 might serve as a promising therapeutic target for ESCC.

C1 [Zhang, Li; Qin, Bin; Zou, Baicang; Wang, Shenhao; Quan, Xiaojing; Wang, Jinhai; Zhao, Hongli] Xi An Jiao Tong Univ, Affiliated Hosp 2, Dept Gastroenterol, 157 Xiwu Rd, Xian 710004, Shaanxi, Peoples R China.

RP Zou, BC (corresponding author), Xi An Jiao Tong Univ, Affiliated Hosp 2, Dept Gastroenterol, 157 Xiwu Rd, Xian 710004, Shaanxi, Peoples R China.

EM baicang_zou@126.com

FU standardized endoscopic diagnosis and treatment of early upper

gastrointestinal cancer and related proteins [2018ZDXM-SF-055]

FX This study was supported by a standardized endoscopic diagnosis and

treatment of early upper gastrointestinal cancer and related proteins

(No.2018ZDXM-SF-055).

CR Abnet CC, 2018, GASTROENTEROLOGY, V154, P360, DOI 10.1053/j.gastro.2017.08.023

Chen WL, 2018, MED SCI MONITOR, V24, P8033, DOI 10.12659/MSM.911142

Chi J, 2016, AGING-US, V8, P2204, DOI 10.18632/aging.101037

Deng FY, 2015, INT J CLIN EXP PATHO, V8, P3045

Esposito D, 2017, BIOCHEM SOC T, V45, P183, DOI 10.1042/BST20160325

Han XY, 2019, J CANCER, V10, P843, DOI 10.7150/jca.27856

Hatakeyama S, 2017, TRENDS BIOCHEM SCI, V42, P297, DOI 10.1016/j.tibs.2017.01.002

Hirano H, 2019, JPN J CLIN ONCOL, V49, P412, DOI 10.1093/jjco/hyz034

Huang FL, 2018, ASIAN J SURG, V41, P210, DOI 10.1016/j.asjsur.2016.10.005

Kimura T, 2015, J CELL BIOL, V210, P973, DOI 10.1083/jcb.201503023

Krishnamurthy N, 2018, CANCER TREAT REV, V62, P50, DOI 10.1016/j.ctrv.2017.11.002

Kwon YT, 2017, TRENDS BIOCHEM SCI, V42, P873, DOI 10.1016/j.tibs.2017.09.002

Lee HJ, 2018, J CANCER PREV, V23, P162, DOI 10.15430/JCP.2018.23.4.162

Lee OH, 2015, BBA-MOL CELL RES, V1853, P409, DOI 10.1016/j.bbamcr.2014.11.007

Li F, 2015, ONCOTARGET, V6, P6001, DOI 10.18632/oncotarget.3469

Li JS, 2010, CANCER BIOL THER, V10, P617, DOI 10.4161/cbt.10.6.12609

Lin J, 2017, J THORAC CARDIOV SUR, V154, P1444, DOI 10.1016/j.jtcvs.2017.07.048

Liu B, 2019, PATHOL ONCOL RES, V25, P1645, DOI 10.1007/s12253-018-0558-6

Ma L, 2019, CANCER CELL INT, V19, DOI 10.1186/s12935-019-0998-4

Ma SH, 2018, ANN NY ACAD SCI, V1434, P164, DOI 10.1111/nyas.13681

Marin I, 2012, PLOS ONE, V7, DOI 10.1371/journal.pone.0050030

Ozato K, 2008, NAT REV IMMUNOL, V8, P849, DOI 10.1038/nri2413

Varshavsky A, 2017, ANNU REV BIOCHEM, V86, P123, DOI 10.1146/annurev-biochem-061516-044859

Venuto S, 2019, CELLS-BASEL, V8, DOI 10.3390/cells8050510

Vilchez V, 2016, WORLD J GASTROENTERO, V22, P823, DOI 10.3748/wjg.v22.i2.823

Watanabe M, 2017, J BIOCHEM, V161, P135, DOI 10.1093/jb/mvw087

Yao H, 2011, EXPERT OPIN THER TAR, V15, P873, DOI 10.1517/14728222.2011.577418

NR 27

TC 2

Z9 3

U1 2

U2 3

PU SPRINGER/PLENUM PUBLISHERS

PI NEW YORK

PA 233 SPRING ST, NEW YORK, NY 10013 USA

SN 0145-479X

EI 1573-6881

J9 J BIOENERG BIOMEMBR

JI J. Bioenerg. Biomembr.

PD APR

PY 2021

VL 53

IS 2

BP 213

EP 222

DI 10.1007/s10863-021-09872-w

EA JAN 2021

PG 10

WC Biophysics; Cell Biology

WE Science Citation Index Expanded (SCI-EXPANDED)

SC Biophysics; Cell Biology

GA QZ8WS

UT WOS:000613056200001

PM 33515345

DA 2022-05-19

ER

PT J

AU Li, ZZ

Li, X

He, XY

Jia, XC

Zhang, XF

Lu, BB

Zhao, JM

Lu, J

Chen, LX

Dong, ZM

Liu, KD

Dong, ZG

AF Li, Zhenzhen

Li, Xin

He, Xinyu

Jia, Xuechao

Zhang, Xiaofan

Lu, Bingbing

Zhao, Jimin

Lu, Jing

Chen, Lexia

Dong, Ziming

Liu, Kangdong

Dong, Zigang

TI Proteomics Reveal the Inhibitory Mechanism of Levodopa Against

Esophageal Squamous Cell Carcinoma

SO FRONTIERS IN PHARMACOLOGY

LA English

DT Article

DE esophageal squamous cell carcinoma; levodopa; proteomic; mitochondria;

succinate dehydrogenase subunit D

ID FATTY LIVER-DISEASE; PARKINSONS-DISEASE; CANCER; SDHD

AB High recurrence rates and poor survival of patients with esophageal squamous cell carcinoma (ESCC) after treatment make ongoing research on chemoprevention drugs for ESCC particularly important. In this study, we screened a large number of FDA-approved drugs and found levodopa, a drug used to treat Parkinson's disease, had an inhibitory effect on the growth of ESCC cells. To elucidate the molecular mechanisms involved, we applied quantitative proteomics to investigate the anti-tumor activity of levodopa on ESCC. The results suggest that levodopa could down-regulate oxidative phosphorylation, non-alcoholic fatty liver disease, and Parkinson's disease pathways. Major mitochondrial respiratory compounds were involved in the pathways, including succinate dehydrogenase subunit D, NADH-ubiquinone oxidoreductase Fe-S protein 4, and mitochondrial cytochrome c oxidase subunit 3. Down-regulation of these proteins was associated with mitochondrial dysfunction. Western blotting and immunofluorescence results confirmed the proteomics findings. Cell viability assays indicated mitochondrial activity was suppressed after levodopa treatment. Reduced mitochondrial membrane potential was detected using JC-1 staining and TMRE assays. Transmission electron microscopy revealed changes in the morphology of mitochondria. Taken together, these results indicate that levodopa inhibited the growth of ESCC through restraining mitochondria function.

C1 [Li, Zhenzhen; Li, Xin; He, Xinyu; Jia, Xuechao; Zhang, Xiaofan; Lu, Bingbing; Zhao, Jimin; Lu, Jing; Chen, Lexia; Dong, Ziming; Liu, Kangdong; Dong, Zigang] Zhengzhou Univ, Coll Med, Dept Pathophysiol, Sch Basic Med Sci,AMS, Zhengzhou, Peoples R China.

[Li, Zhenzhen; He, Xinyu; Jia, Xuechao; Zhang, Xiaofan; Lu, Bingbing; Chen, Lexia; Dong, Ziming; Liu, Kangdong; Dong, Zigang] China US Henan Hormel Canc Inst, Zhengzhou, Peoples R China.

[Zhao, Jimin; Lu, Jing; Dong, Ziming; Liu, Kangdong] Henan Prov Cooperat Innovat Ctr Canc Chemoprevent, Zhengzhou, Peoples R China.

[Zhao, Jimin; Lu, Jing; Dong, Ziming; Liu, Kangdong; Dong, Zigang] State Key Lab Esophageal Canc Prevent & Treatment, Zhengzhou, Peoples R China.

[Liu, Kangdong; Dong, Zigang] Canc Chemoprevent Int Collaborat Lab, Zhengzhou, Peoples R China.

RP Liu, KD; Dong, ZG (corresponding author), Zhengzhou Univ, Coll Med, Dept Pathophysiol, Sch Basic Med Sci,AMS, Zhengzhou, Peoples R China.; Liu, KD; Dong, ZG (corresponding author), China US Henan Hormel Canc Inst, Zhengzhou, Peoples R China.; Liu, KD (corresponding author), Henan Prov Cooperat Innovat Ctr Canc Chemoprevent, Zhengzhou, Peoples R China.; Liu, KD; Dong, ZG (corresponding author), State Key Lab Esophageal Canc Prevent & Treatment, Zhengzhou, Peoples R China.; Liu, KD; Dong, ZG (corresponding author), Canc Chemoprevent Int Collaborat Lab, Zhengzhou, Peoples R China.

EM kdliu@zzu.edu.cn; dongzg@zzu.edu.cn

OI He, Xinyu/0000-0002-7656-2590

FU National Natural Science Foundations of China [81872335, 81572812];

Natural Science Foundations of Henan [161100510300]; National Science &

Technology Major Project "Key New Drug Creation and Manufacturing

Program," China [2018ZX09711002]

FX This work was supported by grants from the National Natural Science

Foundations of China (grant numbers 81872335 and 81572812), the Natural

Science Foundations of Henan (grant number 161100510300), and National

Science & Technology Major Project "Key New Drug Creation and

Manufacturing Program," China (grant number 2018ZX09711002).

CR Abbott A, 2010, NATURE, V466, pS6, DOI 10.1038/466S6a

Bray F, 2018, CA-CANCER J CLIN, V68, P394, DOI 10.3322/caac.21492

Budde SMS, 2002, MITOCHONDRION, V2, P109, DOI 10.1016/S1567-7249(02)00035-1

Chen K, 2017, MOL CANCER, V16, DOI 10.1186/s12943-017-0701-0

Dias MH, 2016, DRUG DISCOV TODAY, V21, P264, DOI 10.1016/j.drudis.2015.10.004

He W, 2017, NPJ PRECIS ONCOL, V1, DOI 10.1038/s41698-017-0014-1

Hoekstra AS, 2016, HUM MOL GENET, V25, P3715, DOI 10.1093/hmg/ddw218

Huang Z, 2017, PROTEOMICS, V17, DOI 10.1002/pmic.201600240

JONES MK, 1978, BRIT J CLIN PHARMACO, V5, P425, DOI 10.1111/j.1365-2125.1978.tb01649.x

Kang KS, 2013, BRAIN RES, V1497, P1, DOI 10.1016/j.brainres.2012.11.043

Kisluk J, 2014, J PHARMACEUT BIOMED, V101, P40, DOI 10.1016/j.jpba.2014.07.038

Kolialexi A, 2009, EXPERT REV PROTEOMIC, V6, P111, DOI 10.1586/EPR.09.6

Kuhn K, 2015, PLANT PHYSIOL, V168, P1537, DOI 10.1104/pp.15.00589

Kugelberg J, 2014, WORLD J SURG, V38, P724, DOI 10.1007/s00268-013-2373-2

Lao YZ, 2014, J ETHNOPHARMACOL, V155, P1, DOI 10.1016/j.jep.2014.05.022

LeWitt PA, 2015, MOVEMENT DISORD, V30, P64, DOI 10.1002/mds.26082

Li HT, 2015, EBIOMEDICINE, V2, P447, DOI 10.1016/j.ebiom.2015.03.019

Lin DC, 2018, GASTROENTEROLOGY, V154, P374, DOI 10.1053/j.gastro.2017.06.066

Liu FF, 2019, MOL CARCINOGEN, V58, P533, DOI 10.1002/mc.22948

Liu QL, 2018, CLIN CANCER RES, V24, P5622, DOI 10.1158/1078-0432.CCR-18-0420

Mohs Richard C, 2017, Alzheimers Dement (N Y), V3, P651, DOI 10.1016/j.trci.2017.10.005

Papa S, 2012, ADV EXP MED BIOL, V942, P3, DOI 10.1007/978-94-007-2869-1_1

Poewe W, 2010, CLIN INTERV AGING, V5, P229, DOI 10.2147/CIA.86456

Song MQ, 2018, MOL CANCER THER, V17, P1540, DOI 10.1158/1535-7163.MCT-17-0823

Stickel F, 2010, GUT, V59, P1303, DOI 10.1136/gut.2009.199661

Tian Y, 2013, SEMIN CANCER BIOL, V23, P471, DOI 10.1016/j.semcancer.2013.08.010

Valsecchi F, 2013, MITOCHONDRION, V13, P436, DOI 10.1016/j.mito.2012.12.001

van der Bliek AM, 2017, GENETICS, V207, P843, DOI 10.1534/genetics.117.300262

Wang M, 2017, PEERJ, V5, DOI 10.7717/peerj.3339

Xie XM, 2019, CANCER LETT, V442, P126, DOI 10.1016/j.canlet.2018.10.016

Xu S, 2018, MOL CANCER THER, V17, P1291, DOI 10.1158/1535-7163.MCT-17-0927

Younossi ZM, 2016, HEPATOLOGY, V64, P73, DOI 10.1002/hep.28431

NR 32

TC 2

Z9 2

U1 1

U2 6

PU FRONTIERS MEDIA SA

PI LAUSANNE

PA AVENUE DU TRIBUNAL FEDERAL 34, LAUSANNE, CH-1015, SWITZERLAND

SN 1663-9812

J9 FRONT PHARMACOL

JI Front. Pharmacol.

PD SEP 25

PY 2020

VL 11

AR 568459

DI 10.3389/fphar.2020.568459

PG 11

WC Pharmacology & Pharmacy

WE Science Citation Index Expanded (SCI-EXPANDED)

SC Pharmacology & Pharmacy

GA NY2FS

UT WOS:000576212600001

PM 33101026

OA gold, Green Published

DA 2022-05-19

ER

PT J

AU Pan, BQ

Xie, ZH

Hao, JJ

Zhang, Y

Xu, X

Cai, Y

Wang, MR

AF Pan, Bei-Qing

Xie, Zhi-Hui

Hao, Jia-Jie

Zhang, Yu

Xu, Xin

Cai, Yan

Wang, Ming-Rong

TI PTP1B up-regulates EGFR expression by dephosphorylating MYH9 at Y1408 to

promote cell migration and invasion in esophageal squamous cell

carcinoma

SO BIOCHEMICAL AND BIOPHYSICAL RESEARCH COMMUNICATIONS

LA English

DT Article

DE Esophageal squamous cell carcinoma; PTP1B; EGFR; MYH9; Migration;

Invasion

ID TYROSINE-PHOSPHATASE 1B; GASTRIC-CANCER CELLS; INSULIN SENSITIVITY;

CONTRIBUTES; DEFICIENCY; RESISTANCE; MOTILITY; GENE

AB Esophageal squamous cell carcinoma (ESCC) is one of the most common cancers worldwide. Protein tyrosine phosphatase 1B (PTP1B) is a member of protein tyrosine phosphatases (PTPs) family. In our previous work, PTP1B was found to be overexpressed in ESCC tissues and made contributions to the the cell migration and invasion as well as lung metastasis of ESCC. In this study, we explored the underlying molecular mechanisms. PTP1B enhanced cell migration and invasion by promoting epidermal growth factor receptor (EGFR) expression in ESCC, which was relied on phosphatase activity of PTP1B. Using GST-pulldown combined with LC/MS/MS, we found that nonmuscle myosin IIA (MYH9) was a novel substrate of PTP1B in ESCC cells. PTP1B dephosphorylated MYH9 at Y1408, by which PTP1B up-regulated EGFR expression and enhanced cell migration and invasion in ESCC. In conclusion, our study first reported that PTP1B was the positive regulator of EGFR by dephosphorylating MYH9 at Y1408 to promote cell migration and invasion, which revealed the regulatory mechanism of PTP1B-MYH9-EGFR axis in ESCC. (C) 2019 Elsevier Inc. All rights reserved.

C1 [Pan, Bei-Qing; Xie, Zhi-Hui; Hao, Jia-Jie; Zhang, Yu; Xu, Xin; Cai, Yan; Wang, Ming-Rong] Chinese Acad Med Sci, State Key Lab Mol Oncol, Ctr Canc Precis Med, Natl Canc Ctr,Natl Clin Res Ctr Canc,Canc Hosp,Pe, 17 Panjiayuan Nanli, Beijing 100021, Peoples R China.

RP Wang, MR (corresponding author), Chinese Acad Med Sci, State Key Lab Mol Oncol, Ctr Canc Precis Med, Natl Canc Ctr,Natl Clin Res Ctr Canc,Canc Hosp,Pe, 17 Panjiayuan Nanli, Beijing 100021, Peoples R China.

EM wangmr2015@126.com

FU National Natural Science Foundation of China [81572841]; National Key

R&D Program of China [2016YFC1303201]; CAMS Innovation Fund for Medical

Sciences (CIFMS) [2016-I2M-1-001]

FX This work was supported by National Natural Science Foundation of China

to Jiajie Hao (No. 81572841), National Key R&D Program of China to Xin

Xu (No. 2016YFC1303201), CAMS Innovation Fund for Medical Sciences

(CIFMS) to Mingrong Wang (No. 2016-I2M-1-001).

CR Althaus K, 2009, SEMIN THROMB HEMOST, V35, P189, DOI 10.1055/s-0029-1220327

Bourdeau A, 2005, CURR OPIN CELL BIOL, V17, P203, DOI 10.1016/j.ceb.2005.02.001

Chen WQ, 2016, CA-CANCER J CLIN, V66, P115, DOI 10.3322/caac.21338

Del Re M, 2019, INT J MOL SCI, V20, DOI 10.3390/ijms20163951

Du XL, 2009, ONCOGENE, V28, P3714, DOI 10.1038/onc.2009.237

Duma N, 2019, MAYO CLIN PROC, V94, P1623, DOI 10.1016/j.mayocp.2019.01.013

Elchebly M, 1999, SCIENCE, V283, P1544, DOI 10.1126/science.283.5407.1544

GOLDSTEIN BJ, 1993, RECEPTOR, V3, P1

Gonzalez-Rodriguez A, 2007, ENDOCRINOLOGY, V148, P594, DOI 10.1210/en.2006-0644

Hsu PC, 2019, INT J MOL SCI, V20, DOI 10.3390/ijms20153821

Huang Y, 2009, J CELL SCI, V122, P1637, DOI 10.1242/jcs.046219

Julien SG, 2007, NAT GENET, V39, P338, DOI 10.1038/ng1963

Kenner KA, 1996, J BIOL CHEM, V271, P19810, DOI 10.1074/jbc.271.33.19810

Klaman LD, 2000, MOL CELL BIOL, V20, P5479, DOI 10.1128/MCB.20.15.5479-5489.2000

Kostrzewa T, 2019, ANTICANCER RES, V39, P3379, DOI 10.21873/anticanres.13481

Kumar A, 2020, CURR MOL PHARMACOL, V13, P17, DOI 10.2174/1874467212666190724150723

Le Sommer S, 2018, CANCER RES, V78, P75, DOI 10.1158/0008-5472.CAN-17-0946

Li FP, 2018, J CANCER, V9, P3839, DOI 10.7150/jca.25469

Liang SL, 2011, PLOS ONE, V6, DOI 10.1371/journal.pone.0018409

Liao SC, 2017, J ZHEJIANG UNIV-SC B, V18, P334, DOI 10.1631/jzus.B1600184

Lin HC, 2013, J PROTEOME RES, V12, P3423, DOI 10.1021/pr400273z

Liu DN, 2012, INT J MOL SCI, V13, P15291, DOI 10.3390/ijms131115291

Nakamura Y, 2008, CIRC RES, V102, P1182, DOI 10.1161/CIRCRESAHA.107.167080

Pecci A, 2018, GENE, V664, P152, DOI 10.1016/j.gene.2018.04.048

Przychodzen P, 2019, TOXICOL IN VITRO, V61, DOI 10.1016/j.tiv.2019.104624

Franco MMR, 2016, BREAST CANCER-BASIC, V10, P177, DOI 10.4137/BCBCR.S40934TYPE

Wang JG, 2010, CELL BIOL INT, V34, P747, DOI 10.1042/CBI20090447

Wang WY, 2018, BIOCHEM BIOPH RES CO, V503, P903, DOI 10.1016/j.bbrc.2018.06.094

Wang XM, 2013, MOL CANCER RES, V11, P986, DOI 10.1158/1541-7786.MCR-12-0704

Xia ZK, 2012, DIS ESOPHAGUS, V25, P427, DOI 10.1111/j.1442-2050.2011.01261.x

Yang M, 2014, MOL CELL, V55, P782, DOI 10.1016/j.molcel.2014.07.018

Yu MC, 2019, FEBS J, V286, P1136, DOI 10.1111/febs.14724

Yu MX, 2017, ONCOTARGET, V8, P156, DOI 10.18632/oncotarget.10613

Zhu S, 2007, CANCER RES, V67, P10129, DOI 10.1158/0008-5472.CAN-06-4338

NR 34

TC 4

Z9 4

U1 6

U2 12

PU ACADEMIC PRESS INC ELSEVIER SCIENCE

PI SAN DIEGO

PA 525 B ST, STE 1900, SAN DIEGO, CA 92101-4495 USA

SN 0006-291X

EI 1090-2104

J9 BIOCHEM BIOPH RES CO

JI Biochem. Biophys. Res. Commun.

PD JAN 29

PY 2020

VL 522

IS 1

BP 53

EP 60

DI 10.1016/j.bbrc.2019.10.168

PG 8

WC Biochemistry & Molecular Biology; Biophysics

WE Science Citation Index Expanded (SCI-EXPANDED)

SC Biochemistry & Molecular Biology; Biophysics

GA KC9KH

UT WOS:000507489400009

PM 31735331

DA 2022-05-19

ER

PT J

AU Zhong, L

Zhou, S

Tong, RS

Shi, JY

Bai, L

Zhu, YX

Duan, XM

Liu, WZ

Bao, JK

Su, LY

Peng, Q

AF Zhong, Lei

Zhou, Shu

Tong, Rongsheng

Shi, Jianyou

Bai, Lan

Zhu, Yuxuan

Duan, Xingmei

Liu, Wenzhao

Bao, Jinku

Su, Lingyu

Peng, Qian

TI Preclinical assessment of histone deacetylase inhibitor quisinostat as a

therapeutic agent against esophageal squamous cell carcinoma

SO INVESTIGATIONAL NEW DRUGS

LA English

DT Article

DE Esophageal squamous cell carcinoma; Quisinostat; Histone deacetylase;

Cancer therapy

AB Esophageal squamous cell carcinoma (ESCC) is one of the most serious life-threatening malignancies. Although chemotherapeutic targets and agents for ESCC have made much progress recently, the efficacy is still unsatisfactory. Therefore, there is still an unmet medical need for patients with ESCC. Here, we report the expression status of HDAC1 in human ESCC and matched paracancerous tissues, and the results indicated that HDAC1 was generally upregulated in ESCC specimens. Furthermore, we comprehensively assessed the anti-ESCC activity of a highly active HDAC1 inhibitor quisinostat. Quisinostat could effectively suppress cellular viability and proliferation of ESCC cells, as well as induce cell cycle arrest and apoptosis even at low treatment concentrations. The effectiveness was also observed in KYSE150 xenograft model when quisinostat was administered at tolerated doses (3mg/kg and 10mg/kg). Meanwhile, quisinostat also had the ability to suppress the migration and invasion (pivotal steps of tumor metastasis) of ESCC cells. Western blot analysis indicated that quisinostat exerted its anti-ESCC effects mainly through blockade of Akt/mTOR and MAPK/ERK signaling cascades. Overall, HDAC1 may serve as a potential therapeutic target for ESCC, and quisinostat deserves to be further assessed as a promising drug candidate for the treatment of ESCC.

C1 [Zhong, Lei; Tong, Rongsheng; Shi, Jianyou; Bai, Lan; Zhu, Yuxuan; Duan, Xingmei] Sichuan Acad Med Sci, Personalized Drug Therapy Key Lab Sichuan Prov, Chengdu, Sichuan, Peoples R China.

[Zhong, Lei; Tong, Rongsheng; Shi, Jianyou; Bai, Lan; Zhu, Yuxuan; Duan, Xingmei; Peng, Qian] Sichuan Prov Peoples Hosp, Chengdu, Sichuan, Peoples R China.

[Zhou, Shu; Bao, Jinku] Sichuan Univ, Sch Life Sci, Chengdu, Sichuan, Peoples R China.

[Zhou, Shu; Bao, Jinku] Sichuan Univ, Minist Educ, Key Lab Bioresources & Ecoenvironm, Chengdu, Sichuan, Peoples R China.

[Liu, Wenzhao; Su, Lingyu] Chongqing Med Univ, Coll Stomatol, Chongqing, Peoples R China.

[Liu, Wenzhao; Su, Lingyu] Chongqing Key Lab Oral Dis & Biomed Sci, Chongqing, Peoples R China.

[Liu, Wenzhao; Su, Lingyu] Chongqing Municipal Key Lab Oral Biomed Engn High, Chongqing, Peoples R China.

[Peng, Qian] Sichuan Acad Med Sci, Canc Ctr, Chengdu, Sichuan, Peoples R China.

RP Peng, Q (corresponding author), Sichuan Prov Peoples Hosp, Chengdu, Sichuan, Peoples R China.; Su, LY (corresponding author), Chongqing Med Univ, Coll Stomatol, Chongqing, Peoples R China.; Su, LY (corresponding author), Chongqing Key Lab Oral Dis & Biomed Sci, Chongqing, Peoples R China.; Su, LY (corresponding author), Chongqing Municipal Key Lab Oral Biomed Engn High, Chongqing, Peoples R China.; Peng, Q (corresponding author), Sichuan Acad Med Sci, Canc Ctr, Chengdu, Sichuan, Peoples R China.

EM sly4089@163.com; pengqian0522@163.com

RI ; liu, wenzhao/W-6265-2018

OI Peng, Qian/0000-0001-5537-0656; liu, wenzhao/0000-0001-7506-0663

FU National Natural Science Foundation of China [81702286, 81300882];

Fundamental Research Funds of Science & Technology Department of Sichuan

Province [2017YSKY0001]; Special Foundation for Young Scientists of

Sichuan Academy of Medical Sciences & Sichuan Provincial People's

Hospital [2016QN11]

FX This work was supported by the National Natural Science Foundation of

China (81702286 and 81300882), the Fundamental Research Funds of Science

& Technology Department of Sichuan Province (2017YSKY0001), and the

Special Foundation for Young Scientists of Sichuan Academy of Medical

Sciences & Sichuan Provincial People's Hospital (2016QN11).

CR Arnold M, 2015, GUT, V64, P381, DOI 10.1136/gutjnl-2014-308124

Arts J, 2009, CLIN CANCER RES, V15, P6841, DOI 10.1158/1078-0432.CCR-09-0547

Barret M, 2018, ANN GASTROENTEROL, V31, P256, DOI 10.20524/aog.2018.0252

Cao LL, 2017, ONCOTARGET, V8, P39241, DOI 10.18632/oncotarget.16843

Colarossi L, 2014, PROTEOM CLIN APPL, V8, P924, DOI 10.1002/prca.201400058

Conte M, 2018, INT J BIOCHEM CELL B, V98, P65, DOI 10.1016/j.biocel.2018.03.004

Eckschlager T, 2017, INT J MOL SCI, V18, DOI 10.3390/ijms18071414

Goey AKL, 2016, PHARMACOGENOMICS, V17, P1807, DOI 10.2217/pgs-2016-0113

Hsu PK, 2018, ANN TRANSL MED, V6, DOI 10.21037/atm.2018.01.37

Liu FT, 2017, ONCOTARGET, V8, P93029, DOI 10.18632/oncotarget.21746

Muller BM, 2013, BMC CANCER, V13, DOI 10.1186/1471-2407-13-215

New M, 2012, MOL ONCOL, V6, P637, DOI 10.1016/j.molonc.2012.09.003

Qin HT, 2017, EXPERT OPIN THER PAT, V27, DOI 10.1080/13543776.2017.1276565

Shaikh T, 2017, SURG ONCOL CLIN N AM, V26, P405, DOI 10.1016/j.soc.2017.01.009

Song M, 2016, J CANCER, V7, P1694, DOI 10.7150/jca.15086

Torre LA, 2015, CA-CANCER J CLIN, V65, P87, DOI 10.3322/caac.21262

Vicente-Duenas C, 2018, TRENDS CANCER, V4, P408, DOI 10.1016/j.trecan.2018.04.007

Wahaib K, 2016, AM J HEALTH-SYST PH, V73, P441, DOI 10.2146/ajhp150487

Witt O, 2000, BLOOD, V95, P2391, DOI 10.1182/blood.V95.7.2391.007k21_2391_2396

Yoon Somy, 2016, Chonnam Med J, V52, P1, DOI 10.4068/cmj.2016.52.1.1

Zhang P, 2015, PLOS ONE, V10, DOI 10.1371/journal.pone.0136193

Zhong L, 2014, INT J CANCER, V135, P2972, DOI 10.1002/ijc.28944

Zibelman M, 2015, INVEST NEW DRUG, V33, P1040, DOI 10.1007/s10637-015-0261-3

NR 23

TC 8

Z9 8

U1 2

U2 15

PU SPRINGER

PI DORDRECHT

PA VAN GODEWIJCKSTRAAT 30, 3311 GZ DORDRECHT, NETHERLANDS

SN 0167-6997

EI 1573-0646

J9 INVEST NEW DRUG

JI Invest. New Drugs

PD AUG

PY 2019

VL 37

IS 4

BP 616

EP 624

DI 10.1007/s10637-018-0651-4

PG 9

WC Oncology; Pharmacology & Pharmacy

WE Science Citation Index Expanded (SCI-EXPANDED)

SC Oncology; Pharmacology & Pharmacy

GA IJ0ZC

UT WOS:000475627500003

PM 30168013

DA 2022-05-19

ER

PT J

AU Wu, J

Gao, FX

Xu, T

Deng, X

Wang, C

Yang, XY

Hu, Z

Long, Y

He, XM

Liang, GN

Ren, DL

Dai, TY

AF Wu, Jian

Gao, Fengxia

Xu, Tao

Deng, Xin

Wang, Chao

Yang, Xiaoyan

Hu, Zhi

Long, Yang

He, Xuemei

Liang, Guannan

Ren, Delian

Dai, Tianyang

TI miR-503 suppresses the proliferation and metastasis of esophageal

squamous cell carcinoma by triggering autophagy via PKA/mTOR signaling

SO INTERNATIONAL JOURNAL OF ONCOLOGY

LA English

DT Article

DE microRNA-503; proliferation and metastasis; autophagy; protein kinase A;

mammalian target of rapamycin signaling; esophageal squamous cell

carcinoma

ID HUMAN HEPATOCELLULAR-CARCINOMA; LUNG-CANCER CELLS; BETA-CATENIN;

APOPTOSIS; RESISTANCE; EXPRESSION; PATHWAY; CISPLATIN; INVASION;

TUMORIGENESIS

AB MicroRNA (miR)-503 is involved in the regulation of the malignant phenotype in multiple tumor types, and has been proven to be a novel diagnostic and therapeutic target; however, its function and mechanisms of action have not yet been fully elucidated in esophageal squamous cell carcinoma (ESCC). In the current study, we detected miR-503 expression by RT-qPCR and found that miR-503 expression was increased in ESCC, but negatively correlated with lymph node metastasis, TNM stage and tumor differentiation. Functionally, we confirmed that miR-503 inhibited the proliferation and metastasis of ESCC cells by triggering cellular autophagy. Mechanistically, we confirmed that miR-503 exerted its biological effects by targeting protein kinase CAMP-activated catalytic subunit alpha (PRKACA) in ESCC by dual luciferase reporter assay. Moreover, miR-503 was found to trigger autophagy in ESCC cells through the protein kinase A (PKA)/mammalian target of rapamycin (mTOR) pathway. Taken together, our results demonstrate that miR-503 suppresses the proliferation and metastasis of ESCC via the activation of autophagy, mediated by the PKA/mTOR signaling pathway.

C1 [Wu, Jian; Xu, Tao; Wang, Chao; Yang, Xiaoyan; Hu, Zhi; Dai, Tianyang] Southwest Med Univ, Affiliated Hosp, Dept Thorac Surg, 25 Taiping St, Luzhou 646000, Sichuan, Peoples R China.

[Gao, Fengxia; Ren, Delian] Southwest Med Univ, Dept Immunol, Coll Basic Med, 25 Taiping St, Luzhou 646000, Sichuan, Peoples R China.

[Deng, Xin] Southwest Med Univ, Drug Discovery Res Ctr, Luzhou 646000, Sichuan, Peoples R China.

[Deng, Xin] Southwest Med Univ, Sch Pharm, Dept Pharmacol, Lab Cardiovasc Pharmacol, Luzhou 646000, Sichuan, Peoples R China.

[Long, Yang; He, Xuemei; Liang, Guannan] Southwest Med Univ, Ctr Med Expt, Affiliated Hosp, Luzhou 646000, Sichuan, Peoples R China.

RP Dai, TY (corresponding author), Southwest Med Univ, Affiliated Hosp, Dept Thorac Surg, 25 Taiping St, Luzhou 646000, Sichuan, Peoples R China.; Ren, DL (corresponding author), Southwest Med Univ, Dept Immunol, Coll Basic Med, 25 Taiping St, Luzhou 646000, Sichuan, Peoples R China.

EM rendelian0605@sina.com; daitianyang0502@sina.com

FU Technology Department, Sichuan Province [2014TSX-0102, 2015SX-00010];

Youth Foundation of the Affiliated Hospital of South West Medical

University [16025]; Sichuan Science and Technology Plan projects

[2016RZ0076]; Health and Family Planning Commission of Sichuan Province

Research Program [16ZD033]

FX This study was supported by two Joint Fund of Technology Department,

Sichuan Province (nos. 2014TSX-0102 and 2015SX-00010), and by the Youth

Foundation of the Affiliated Hospital of South West Medical University

(16025), and by Sichuan Science and Technology Plan projects (no.

2016RZ0076) and Health and Family Planning Commission of Sichuan

Province Research Program (no. 16ZD033).

CR Angelousi A, 2017, HORM METAB RES, V49, P786, DOI 10.1055/s-0043-116385

Beristain AG, 2015, ONCOGENE, V34, P1160, DOI 10.1038/onc.2014.41

Berthon AS, 2015, FRONT CELL DEV BIOL, V3, DOI 10.3389/fcell.2015.00026

Catalano M, 2015, MOL ONCOL, V9, P1612, DOI 10.1016/j.molonc.2015.04.016

Chan CM, 2018, ONCOL LETT, V15, P3273, DOI 10.3892/ol.2017.7698

Chen HI, 2016, PATHOL ONCOL RES, V22, P699, DOI 10.1007/s12253-016-0051-z

Chen LM, 2017, ONCOTARGET, V8, P63911, DOI 10.18632/oncotarget.19201

Chen XX, 2017, NAT COMMUN, V8, DOI 10.1038/s41467-017-00650-0

Chong Y, 2014, PLOS ONE, V9, DOI 10.1371/journal.pone.0114585

Cilek EE, 2017, PLOS ONE, V12, DOI 10.1371/journal.pone.0185558

Corbetta S, 2010, ENDOCR-RELAT CANCER, V17, P135, DOI 10.1677/ERC-09-0134

de Joussineau C, 2014, HUM MOL GENET, V23, P5418, DOI 10.1093/hmg/ddu265

Deng XS, 2017, J SURG RES, V218, P285, DOI 10.1016/j.jss.2017.05.088

Endo S, 2018, BIOCHEM BIOPH RES CO, V497, P437, DOI 10.1016/j.bbrc.2018.02.105

Furukawa Y, 2017, CANCER GENE THER, V24, P393, DOI 10.1038/cgt.2017.33

Grassi G, 2015, CELL DEATH DIS, V6, DOI 10.1038/cddis.2015.249

Gu Y, 2017, MOL MED REP, V16, P8130, DOI 10.3892/mmr.2017.7646

He C, 2017, CANCER SCI, V108, P2503, DOI 10.1111/cas.13399

Hou SS, 2018, CELL DEATH DIS, V9, DOI 10.1038/s41419-018-0319-1

Hou ZC, 2017, ONCOTARGET, V8, P60539, DOI 10.18632/oncotarget.16966

Hu JZ, 2017, CELL PHYSIOL BIOCHEM, V43, P367, DOI 10.1159/000480416

Hu XT, 2017, CELL DEATH DIS, V8, DOI 10.1038/cddis.2017.447

Jin CC, 2018, MINERVA CHIR, V73, P113, DOI 10.23736/S0026-4733.17.07472-7

Kastenhuber ER, 2017, P NATL ACAD SCI USA, V114, P13076, DOI 10.1073/pnas.1716483114

Lan SH, 2014, AUTOPHAGY, V10, P1687, DOI 10.4161/auto.29959

Livak KJ, 2001, METHODS, V25, P402, DOI 10.1006/meth.2001.1262

Lu YC, 2012, CANCER PREV RES, V5, P665, DOI 10.1158/1940-6207.CAPR-11-0358

Ma T, 2018, BIOMED PHARMACOTHER, V98, P680, DOI 10.1016/j.biopha.2017.12.095

Ma Z, 2017, DIS ESOPHAGUS, V30, DOI 10.1093/dote/dox059

Mei LL, 2017, INT J MOL SCI, V18, DOI 10.3390/ijms18091833

Mei LL, 2017, PLOS ONE, V12, DOI 10.1371/journal.pone.0185636

Moody SE, 2015, ONCOGENE, V34, P2061, DOI 10.1038/onc.2014.153

Peng Y, 2014, ONCOL LETT, V7, P1233, DOI 10.3892/ol.2014.1868

Qi Y, 2017, ONCOL LETT, V14, P3415, DOI 10.3892/ol.2017.6614

Qiu TZ, 2013, INT J MOL MED, V32, P593, DOI 10.3892/ijmm.2013.1439

Samaras P, 2017, CANCER CHEMOTH PHARM, V80, P1005, DOI 10.1007/s00280-017-3446-y

Shang MH, 2018, ONCOTARGETS THER, V11, P639, DOI 10.2147/OTT.S157638

Shivapurkar N, 2017, CLIN TRANSL MED, V6, DOI 10.1186/s40169-017-0169-y

Sun Y, 2017, CANCER BIOMARK, V20, P597, DOI 10.3233/CBM-170585

Tombol Z, 2009, ENDOCR-RELAT CANCER, V16, P895, DOI 10.1677/ERC-09-0096

Wang TS, 2014, CHINESE MED J-PEKING, V127, P2357, DOI 10.3760/cma.j.issn.0366-6999.20140318

Wu DY, 2017, ONCOTARGETS THER, V10, P129, DOI 10.2147/OTT.S114303

Xu K, 2017, ONCOTARGET, V8, P21719, DOI 10.18632/oncotarget.15559

Xu YY, 2013, FEBS J, V280, P3768, DOI 10.1111/febs.12365

Yang XY, 2017, ONCOL REP, V37, P563, DOI 10.3892/or.2016.5220

Yang Y, 2014, INT J CANCER, V135, P1531, DOI 10.1002/ijc.28799

Yu HY, 2017, MOL CANCER THER, V16, P2711, DOI 10.1158/1535-7163.MCT-17-0498

Zhang B, 2017, SCI REP-UK, V7, DOI 10.1038/s41598-017-03680-2

Zhao JJ, 2009, CHILD NERV SYST, V25, P13, DOI 10.1007/s00381-008-0701-x

Zhou HH, 2017, MED SCI MONITOR, V23, P4817, DOI 10.12659/MSM.906583

Zhu J, 2017, AM J TRANSL RES, V9, P4046

NR 51

TC 16

Z9 17

U1 0

U2 18

PU SPANDIDOS PUBL LTD

PI ATHENS

PA POB 18179, ATHENS, 116 10, GREECE

SN 1019-6439

EI 1791-2423

J9 INT J ONCOL

JI Int. J. Oncol.

PD MAY

PY 2018

VL 52

IS 5

BP 1427

EP 1442

DI 10.3892/ijo.2018.4320

PG 16

WC Oncology

WE Science Citation Index Expanded (SCI-EXPANDED)

SC Oncology

GA GB5HV

UT WOS:000429096000006

PM 29568867

OA Green Published, hybrid, Green Submitted

DA 2022-05-19

ER

PT J

AU Peng, KZ

Ke, Y

Zhao, Q

Tian, F

Liu, HM

Hou, GQ

Lu, ZM

AF Peng, Ke-Zheng

Ke, Yu

Zhao, Qi

Tian, Fei

Liu, Hong-Min

Hou, Guiqin

Lu, Zhaoming

TI OP16, a novel ent-kaurene diterpenoid, potentiates the antitumor effect

of rapamycin by inhibiting rapamycin-induced feedback activation of Akt

signaling in esophageal squamous cell carcinoma

SO BIOCHEMICAL PHARMACOLOGY

LA English

DT Article

DE Esophageal squamous cell carcinoma; Rapamycin; Oridonin; Akt; mTOR

ID MTOR INHIBITORS; CANCER STATISTICS; GROWTH; PATHWAY; RICTOR;

PHOSPHORYLATION; COMBINATION; ORIDONIN; THERAPY; DISEASE

AB Hyperactivation of mTOR signaling pathway has been viewed as a significant molecular pathogenesis of cancer. However, inhibition of mTOR by rapamycin and its analogs could induce numerous negative feedback loops to attenuate their therapeutic efficacy. As a traditional Chinese herbal medicine, Rabdosia rubescens has been used to treat esophageal squamous cell carcinoma (ESCC) for hundreds of years, and its major effective component is oridonin. Here we reported that OP16, a novel analog of oridonin, showed potent inhibition of cell proliferation and Akt phosphorylation in ESCC cells. The combination of OP16 and rapamycin possesses synergistic anti-proliferative and pro-apoptotic effects both in ESCC cells and ESCC xenografts, and no obvious adverse effect was observed in vivo. Mechanistic analysis revealed that OP16 could inhibit rapamycin-induced Akt activation through the p70S6K-mediated negative feedback loops, and the combination of OP16 and rapamycin was more effective in activating caspase-dependent apoptotic signaling cascade. This study supports the combined use of OP16 with rapamycin as a feasible and effective therapeutic approach for future treatment of ESCC. (C) 2017 Elsevier Inc. All rights reserved.

C1 [Peng, Ke-Zheng; Ke, Yu; Zhao, Qi; Tian, Fei; Liu, Hong-Min; Hou, Guiqin; Lu, Zhaoming] Zhengzhou Univ, Sch Pharmaceut Sci, 100 Kexue Ave, Zhengzhou 450001, Henan, Peoples R China.

[Peng, Ke-Zheng; Ke, Yu; Liu, Hong-Min; Hou, Guiqin] Zhengzhou Univ, Inst Drug Discovery & Dev, Zhengzhou, Henan, Peoples R China.

[Peng, Ke-Zheng; Ke, Yu; Liu, Hong-Min; Hou, Guiqin] Zhengzhou Univ, Minist Educ, Key Lab Technol Drug Preparat, Zhengzhou, Henan, Peoples R China.

RP Hou, GQ; Lu, ZM (corresponding author), Zhengzhou Univ, Sch Pharmaceut Sci, 100 Kexue Ave, Zhengzhou 450001, Henan, Peoples R China.

EM hougq@zzu.edu.cn; lzm310@zzu.edu.cn

OI Peng, Ke-Zheng/0000-0002-4004-8928

FU National Science Foundation of China [30901778]; National Science and

Technology Major Projects for "Major New Drugs Innovation and

Development" of China [20092X09102-154]; Open Foundation Project of

Pharmacy in Zhejiang Province [YKFJ2-010]; Basic & Frontier Research

Foundation of Henan Science & Technology Agency [162300410122]

FX This study is supported by the National Science Foundation of China (No.

30901778), the National Science and Technology Major Projects for "Major

New Drugs Innovation and Development" of China (No. 20092X09102-154),

the Open Foundation Project of Pharmacy in Zhejiang Province (No.

YKFJ2-010) and the Basic & Frontier Research Foundation of Henan Science

& Technology Agency (No. 162300410122).

CR Albert V, 2015, CURR OPIN CELL BIOL, V33, P55, DOI 10.1016/j.ceb.2014.12.001

Amornphimoltham P, 2005, CANCER RES, V65, P9953, DOI 10.1158/0008-5472.CAN-05-0921

Breuleux M, 2009, MOL CANCER THER, V8, P742, DOI 10.1158/1535-7163.MCT-08-0668

Chen WQ, 2016, CA-CANCER J CLIN, V66, P115, DOI 10.3322/caac.21338

Chiarini F, 2015, TRENDS PHARMACOL SCI, V36, P124, DOI 10.1016/j.tips.2014.11.004

Chou TC, 2006, PHARMACOL REV, V58, P621, DOI 10.1124/pr.58.3.10

Dienstmann R, 2014, MOL CANCER THER, V13, P1021, DOI 10.1158/1535-7163.MCT-13-0639

Ding Y, 2016, EUR J MED CHEM, V122, P102, DOI 10.1016/j.ejmech.2016.06.015

Efeyan A, 2010, CURR OPIN CELL BIOL, V22, P169, DOI 10.1016/j.ceb.2009.10.007

Engelman JA, 2008, NAT MED, V14, P1351, DOI 10.1038/nm.1890

Guba M, 2002, NAT MED, V8, P128, DOI 10.1038/nm0202-128

Hou GQ, 2007, CANCER LETT, V253, P236, DOI 10.1016/j.canlet.2007.01.026

Hou GQ, 2014, J IMMUNOL RES, V2014, DOI 10.1155/2014/845763

Hou GQ, 2010, CANCER LETT, V290, P248, DOI 10.1016/j.canlet.2009.09.015

Hudes G, 2007, NEW ENGL J MED, V356, P2271, DOI 10.1056/NEJMoa066838

Julien LA, 2010, MOL CELL BIOL, V30, P908, DOI 10.1128/MCB.00601-09

Kim J, 2017, NATURE, V541, P169, DOI 10.1038/nature20805

Laplante M, 2012, CELL, V149, P274, DOI 10.1016/j.cell.2012.03.017

Li CY, 2011, INT J BIOCHEM CELL B, V43, P701, DOI 10.1016/j.biocel.2011.01.020

Li ZJ, 2012, CLIN CANCER RES, V18, P3603, DOI 10.1158/1078-0432.CCR-11-3321

Liu H, Patent, Patent No. 8084430

Liu H., Patent No. [ZL200610017358.0, 2006100173580]

Liu Qingsong, 2009, Drug Discov Today Ther Strateg, V6, P47

Lu Z., 2016, DIS ESOPHAGUS

Motzer RJ, 2008, LANCET, V372, P449, DOI 10.1016/S0140-6736(08)61039-9

Mundi PS, 2016, BRIT J CLIN PHARMACO, V82, P943, DOI 10.1111/bcp.13021

Nguyen SA, 2012, CURR TREAT OPTION ON, V13, P71, DOI 10.1007/s11864-011-0180-2

Peng KZ, 2016, J ETHNOPHARMACOL, V192, P161, DOI 10.1016/j.jep.2016.07.012

Peterson TR, 2009, CELL, V137, P873, DOI 10.1016/j.cell.2009.03.046

Rodrik-Outmezguine VS, 2011, CANCER DISCOV, V1, P248, DOI 10.1158/2159-8290.CD-11-0085

Santulli G, 2013, PHARMACOGENOMICS, V14, P1517, DOI 10.2217/pgs.13.143

Sarbassov DD, 2005, SCIENCE, V307, P1098, DOI 10.1126/science.1106148

Sengupta S, 2010, MOL CELL, V40, P310, DOI 10.1016/j.molcel.2010.09.026

Shimobayashi M, 2014, NAT REV MOL CELL BIO, V15, P155, DOI 10.1038/nrm3757

Siegel RL, 2016, CA-CANCER J CLIN, V66, P7, DOI 10.3322/caac.21332

Siewert JR, 2007, SEMIN RADIAT ONCOL, V17, P38, DOI 10.1016/j.semradonc.2006.09.007

Tan W, 2011, CHIN MED-UK, V6, DOI 10.1186/1749-8546-6-27

Wan X, 2007, ONCOGENE, V26, P1932, DOI 10.1038/sj.onc.1209990

Wander SA, 2011, J CLIN INVEST, V121, P1231, DOI 10.1172/JCI44145

Wang C, 2015, PLOS ONE, V10, DOI 10.1371/journal.pone.0130284

Xu ST, 2016, J MED CHEM, V59, P5022, DOI 10.1021/acs.jmedchem.6b00408

Yori JL, 2014, CANCER RES, V74, P4762, DOI 10.1158/0008-5472.CAN-13-3627

Zaytseva YY, 2012, CANCER LETT, V319, P1, DOI 10.1016/j.canlet.2012.01.005

Zoncu R, 2011, NAT REV MOL CELL BIO, V12, P21, DOI 10.1038/nrm3025

NR 44

TC 16

Z9 17

U1 3

U2 29

PU PERGAMON-ELSEVIER SCIENCE LTD

PI OXFORD

PA THE BOULEVARD, LANGFORD LANE, KIDLINGTON, OXFORD OX5 1GB, ENGLAND

SN 0006-2952

EI 1873-2968

J9 BIOCHEM PHARMACOL

JI Biochem. Pharmacol.

PD SEP 15

PY 2017

VL 140

BP 16

EP 27

DI 10.1016/j.bcp.2017.05.013

PG 12

WC Pharmacology & Pharmacy

WE Science Citation Index Expanded (SCI-EXPANDED)

SC Pharmacology & Pharmacy

GA FE2UR

UT WOS:000408072900003

PM 28539264

DA 2022-05-19

ER

PT J

AU Han, GH

Tian, Y

Duan, BS

Sheng, HH

Gao, HJ

Huang, JX

AF Han, Gaohua

Tian, Ye

Duan, Bensong

Sheng, Haihui

Gao, Hengjun

Huang, Junxing

TI Association of nuclear annexin A1 with prognosis of patients with

esophageal squamous cell carcinoma

SO INTERNATIONAL JOURNAL OF CLINICAL AND EXPERIMENTAL PATHOLOGY

LA English

DT Article

DE Annexin A1; three prime repair exonuclease 1; apurinic/apyrimidinic

endonuclease-1; esophageal squamous cell carcinoma; prognosis

ID DECREASED EXPRESSION; DOWN-REGULATION; CANCER; PROGRESSION;

GASTROESOPHAGEAL; OVEREXPRESSION; METASTASIS; SURVIVAL; TREX1

AB Although recent progress has been made in the diagnosis and treatment of cancer, the prognosis of esophageal squamous cell carcinoma (ESCC) remains poor. The identification of biomarkers for ESCC prognosis is important for treatment decisions. The aim of this study was to evaluate the relationship between the expressions of Annexin A1 (ANXA1), three prime repair exonuclease 1 (TREX1) and apurinic/apyrimidinic endonuclease-1 (APE1) and clinical outcome of patients with ESCC. The expressions of ANXA1, TREX1 and APE1 in 93 pairs of ESCC and paracancerous tissues were tested using immunohistochemistry. ANX1, TREX1 and APE1 were dysregulated in ESCC. Nuclear expressions of ANXA1 and APE1 were significantly associated with pathologic type (P = 0.004 and 0.040, respectively). Patients with low expression of nuclear ANXA1 had a better prognosis than those with high expression of nuclear ANXA1 (HR = 0. 448, 95% CI 0.236-0.849, P = 0.014), especially for those with histologic grade 1 and 2 (HR = 0.303, 95% CI: 0.155-0.593, P < 0.001). In conclusion, nuclear ANXA1 may be potentially used as a prognostic biomarker for ESCC.

C1 [Han, Gaohua; Tian, Ye] Suzhou Univ, Affiliated Hosp 2, Dept Oncol, Suzhou 215006, Jiangsu, Peoples R China.

[Han, Gaohua; Huang, Junxing] Taizhou Peoples Hosp, Dept Oncol, Taizhou 225300, Jiangsu, Peoples R China.

[Duan, Bensong; Gao, Hengjun] Tongji Univ, Dept Gastroenterol, Inst Digest Dis, Tongji Hosp, Shanghai 200092, Peoples R China.

[Duan, Bensong; Sheng, Haihui; Gao, Hengjun] Natl Engn Ctr Biochip Shanghai, Shanghai, Peoples R China.

RP Tian, Y (corresponding author), Suzhou Univ, Affiliated Hosp 2, Dept Oncol, 181 Sanxiang Rd, Suzhou 215006, Jiangsu, Peoples R China.

EM dryetian@hotmail.com; hjxtz@sina.cn

FU Jiangsu Province Ministry of Health, China [H201260]; 12th Five-Year

Plan Key Project of Science and Technology, China [2013ZX10002007];

Shanghai Committee of Science and Technology, China [13440701500];

Jiangsu Province Science and Technology Support Program, China

[BE2012729]

FX This work was supported by the Jiangsu Province Ministry of Health,

China (grant No. H201260), the 12th Five-Year Plan Key Project of

Science and Technology, China (grant No. 2013ZX10002007), the Shanghai

Committee of Science and Technology, China (grant No. 13440701500), and

the Jiangsu Province Science and Technology Support Program, China

(grant No. BE2012729).

CR Al-Attar A, 2010, BRIT J CANCER, V102, P704, DOI 10.1038/sj.bjc.6605541

Bai XF, 2004, WORLD J GASTROENTERO, V10, P1466

Calmon MF, 2013, PLOS ONE, V8, DOI 10.1371/journal.pone.0053260

Cheng TY, 2012, CANCER-AM CANCER SOC, V118, P5757, DOI 10.1002/cncr.27565

Dai N, 2013, PLOS ONE, V8, DOI 10.1371/journal.pone.0058001

Dong XQ, 2011, ONCOLOGIST, V16, P61, DOI 10.1634/theoncologist.2010-0127

Fareed KR, 2010, BRIT J CANCER, V102, P1600, DOI 10.1038/sj.bjc.6605686

Guo CM, 2013, FUTURE ONCOL, V9, P1773, DOI [10.2217/FON.13.114, 10.2217/fon.13.114]

He J, 2012, 2012 CHINESE CANC RE, P27

Hu N, 2004, CLIN CANCER RES, V10, P6013, DOI 10.1158/1078-0432.CCR-04-0317

Huang J, 2000, CARCINOGENESIS, V21, P2019, DOI 10.1093/carcin/21.11.2019

Jemal A, 2011, CA-CANCER J CLIN, V61, P69, DOI [10.3322/caac.20107, 10.3322/caac.20115]

Jorge YC, 2013, MEDIAT INFLAMM, V2013, DOI 10.1155/2013/152860

Kang H, 2012, BIOCHEM BIOPH RES CO, V423, P188, DOI 10.1016/j.bbrc.2012.05.114

Li CF, 2010, PATHOLOGY, V42, P43, DOI 10.3109/00313020903434405

Lin CY, 2008, J SURG ONCOL, V97, P544, DOI 10.1002/jso.20992

Morita M, 2004, MOL CELL BIOL, V24, P6719, DOI 10.1128/MCB.24.15.6719-6727.2004

Mu DW, 2013, PLOS ONE, V8, DOI 10.1371/journal.pone.0074922

Patton KT, 2005, HISTOPATHOLOGY, V47, P597, DOI 10.1111/j.1365-2559.2005.02300.x

Petrella A, 2006, CANCER BIOL THER, V5, P643, DOI 10.4161/cbt.5.6.2700

Shen DJ, 2006, HUM PATHOL, V37, P1583, DOI 10.1016/j.humpath.2006.06.001

Shimizu T, 2012, PLOS ONE, V7, DOI 10.1371/journal.pone.0045510

Tomicic MT, 2013, BBA-MOL CELL RES, V1833, P1832, DOI 10.1016/j.bbamcr.2013.03.029

Wang CJ, 2009, DNA REPAIR, V8, P1179, DOI 10.1016/j.dnarep.2009.06.006

Wang D, 2010, EUR SURG RES, V45, P151, DOI 10.1159/000320237

Wang KL, 2006, CLIN CANCER RES, V12, P4598, DOI 10.1158/1078-0432.CCR-06-0483

Wang LD, 2008, J INT MED RES, V36, P665, DOI 10.1177/147323000803600407

Wang LP, 2010, NEOPLASMA, V57, P253, DOI 10.4149/neo_2010_03_253

Xia L, 2013, BIOMED RES INT, V2013, DOI 10.1155/2013/957913

Yan W, 2011, AM J CANCER RES, V1, P275

Yom CK, 2011, J BREAST CANCER, V14, P262, DOI 10.4048/jbc.2011.14.4.262

Yu GZ, 2008, CLIN EXP METASTAS, V25, P695, DOI 10.1007/s10585-008-9178-y

Zhang XL, 2013, PATHOL RES PRACT, V209, P510, DOI 10.1016/j.prp.2013.06.005

Zhu DW, 2013, BMC CANCER, V13, DOI 10.1186/1471-2407-13-301

NR 34

TC 24

Z9 24

U1 1

U2 9

PU E-CENTURY PUBLISHING CORP

PI MADISON

PA 40 WHITE OAKS LN, MADISON, WI 53711 USA

SN 1936-2625

J9 INT J CLIN EXP PATHO

JI Int. J. Clin. Exp. Pathol.

PY 2014

VL 7

IS 2

BP 751

EP 759

PG 9

WC Oncology; Pathology

WE Science Citation Index Expanded (SCI-EXPANDED)

SC Oncology; Pathology

GA AG2DS

UT WOS:000335226800032

PM 24551299

DA 2022-05-19

ER

PT J

AU Gao, JJ

Tian, LS

Sun, YL

Li, W

Zhao, LN

Sun, Y

Jing, ZP

Zhou, LP

Liu, F

Zhao, XH

AF Gao, Jiajia

Tian, Lusong

Sun, Yulin

Li, Wei

Zhao, Lina

Sun, Yan

Jing, Zongpan

Zhou, Lanping

Liu, Fang

Zhao, Xiaohang

TI PUR alpha mediates epithelial-mesenchymal transition to promote

esophageal squamous cell carcinoma progression by regulating Snail2

SO CANCER LETTERS

LA English

DT Article

DE Esophageal squamous cell carcinoma; PUR alpha; Epithelial-mesenchymal

transition; Snail2; Migration and invasion

ID POSTNATAL BRAIN-DEVELOPMENT; EXPRESSION; IDENTIFICATION;

DIFFERENTIATION; INDUCTION; DNA; OVEREXPRESSION; METASTASIS; MIGRATION;

FAMILY

AB Esophageal squamous cell carcinoma (ESCC) is one of the most common lethal cancers in the world. Dysregulation of purine-rich element binding protein alpha (PUR alpha), which contributes to the initiation of PUR. syndrome, is reportedly involved in the progression of multiple cancers, but its function and underlying mechanisms in ESCC progression remain unclear. Here, we first demonstrated that PUR alpha promoted cell growth, migration and invasion in ESCC both in vitro and in vivo. An immunohistochemistry assay was then performed on 225 ESCC tissues, showing that high PUR alpha expression was positively associated with lymph node metastasis and the AJCC stage, and the ESCC patients with positive PURa expression had worse survival. In addition, RNA sequencing implied that PUR alpha induced epithelial-mesenchymal transition (EMT) in ESCC, which was further confirmed by qPCR, Western blotting and immunofluorescence analyses. Mechanistically, PUR alpha enhanced the transcription of Snail2 by binding to its promoter region. Knockdown of Snail2 reversed PUR alpha-induced EMT and inhibited the migration and invasion of ESCC cells. In conclusion, this study indicated that PUR alpha promotes Snail2 transcriptional activity to induce EMT during ESCC progression.

C1 [Gao, Jiajia; Tian, Lusong; Sun, Yulin; Li, Wei; Zhao, Lina; Sun, Yan; Jing, Zongpan; Zhou, Lanping; Liu, Fang; Zhao, Xiaohang] Chinese Acad Med Sci & Peking Union Med Coll, State Key Lab Mol Oncol, Natl Canc Ctr, Natl Clin Res Ctr Canc,Canc Hosp, Beijing, Peoples R China.

RP Zhao, XH (corresponding author), Chinese Acad Med Sci & Peking Union Med Coll, State Key Lab Mol Oncol, Natl Canc Ctr, Natl Clin Res Ctr Canc,Canc Hosp, Beijing, Peoples R China.

EM zhaoxh@cicams.ac.cn

FU National Key RD Program [2018YFC1313101, 2017ZX10203205-003-001,

2016YFC0901403]; National Natural Science Foundation [81872033,

81572365]; CAMS Innovation Fund for Medical Sciences of China

[2019-I2M-1-003, 2016-I2M-1-001, 2017-I2M-3-005]

FX This work was supported by grants from the National Key R&D Program

(nos. 2018YFC1313101, 2017ZX10203205-003-001 and 2016YFC0901403 to X

Zhao), the National Natural Science Foundation (nos. 81872033 and

81572365 to X Zhao) and the CAMS Innovation Fund for Medical Sciences

(nos. 2019-I2M-1-003, 2016-I2M-1-001 and 2017-I2M-3-005 to X Zhao and Y

Sun) of China.

CR Barr SM, 2001, J CELL BIOCHEM, V81, P621, DOI 10.1002/jcb.1099

Bray F, 2018, CA-CANCER J CLIN, V68, P394, DOI 10.3322/caac.21492

Bruchova H, 2002, LEUKEMIA LYMPHOMA, V43, P1289, DOI 10.1080/10428190290026358

Caramel J, 2013, CANCER CELL, V24, P466, DOI 10.1016/j.ccr.2013.08.018

Da Silva N, 2002, BLOOD, V100, P3536, DOI 10.1182/blood.V100.10.3536

Daniel DC, 2018, GENE, V643, P133, DOI 10.1016/j.gene.2017.12.004

Darbinian N, 2001, J NEUROIMMUNOL, V121, P3, DOI 10.1016/S0165-5728(01)00372-1

Darbinian N, 2001, J CELL PHYSIOL, V189, P334, DOI 10.1002/jcp.10029

De Craene B, 2013, NAT REV CANCER, V13, P97, DOI 10.1038/nrc3447

Esteves P, 2020, ONCOGENE, V39, P617, DOI 10.1038/s41388-019-1009-x

Hao XP, 2001, CANCER RES, V61, P8085

Hirano H, 2019, JPN J CLIN ONCOL, V49, P412, DOI 10.1093/jjco/hyz034

Hokkanen S, 2012, HUM MOL GENET, V21, P473, DOI 10.1093/hmg/ddr476

Hu Y, 2019, CELL ADHES MIGR, V13, P285, DOI 10.1080/19336918.2019.1638689

Hunt D, 2014, J MED GENET, V51, P806, DOI 10.1136/jmedgenet-2014-102798

Inoue T, 2008, PROSTATE, V68, P1048, DOI 10.1002/pros.20764

Inoue T, 2009, PROSTATE, V69, P861, DOI 10.1002/pros.20936

Ji JF, 2010, J HEPATOL, V52, P690, DOI 10.1016/j.jhep.2009.12.025

Kelm RJ, 2018, J CELL BIOCHEM, V119, P2073, DOI 10.1002/jcb.26369

Khalili K, 2003, MOL CELL BIOL, V23, P6857, DOI 10.1128/MCB.23.19.6857-6875.2003

Kong TQ, 2007, J IMMUNOL, V179, P1934, DOI 10.4049/jimmunol.179.3.1934

Lasham A, 2000, GENE, V252, P1, DOI 10.1016/S0378-1119(00)00220-1

Lehoux S, 2010, GLYCOCONJUGATE J, V27, P99, DOI 10.1007/s10719-009-9260-y

Lezon-Geyda K, 2001, LEUKEMIA, V15, P954, DOI 10.1038/sj.leu.2402108

Li W, 2016, ONCOTARGET, V7, P21542, DOI 10.18632/oncotarget.7775

Lin DC, 2018, GASTROENTEROLOGY, V154, P374, DOI 10.1053/j.gastro.2017.06.066

Lin ZW, 2014, INT J ONCOL, V45, P57, DOI 10.3892/ijo.2014.2436

Liu XM, 2010, PROSTATE, V70, P179, DOI 10.1002/pros.21051

MA ZW, 1995, CYTOGENET CELL GENET, V71, P64, DOI 10.1159/000134065

Martincorena I, 2018, SCIENCE, V362, P911, DOI 10.1126/science.aau3879

Maseleno A, 2013, INT CONF COMP SCI, P8, DOI 10.1109/CSIT.2013.6588750

Natsuizaka M, 2017, NAT COMMUN, V8, DOI 10.1038/s41467-017-01500-9

Peinado H, 2007, NAT REV CANCER, V7, P415, DOI 10.1038/nrc2131

Polyak K, 2009, NAT REV CANCER, V9, P265, DOI 10.1038/nrc2620

Rustgi AK, 2014, NEW ENGL J MED, V371, P2499, DOI 10.1056/NEJMra1314530

Sharma P, 2016, INDIAN J MED RES, V143, P91, DOI 10.4103/0971-5916.191796

Shelley CS, 2002, J IMMUNOL, V168, P3887, DOI 10.4049/jimmunol.168.8.3887

Shelley CS, 2001, BRIT J HAEMATOL, V115, P159, DOI 10.1046/j.1365-2141.2001.03066.x

Siegel RL, 2019, CA-CANCER J CLIN, V69, P7, DOI 10.3322/caac.21551

Song YM, 2014, NATURE, V509, P91, DOI 10.1038/nature13176

Stacey DW, 1999, ONCOGENE, V18, P4254, DOI 10.1038/sj.onc.1202795

Tanaka A.J., 2015, COLD SPRING HARB MOL, V1

Thiery JP, 2009, CELL, V139, P871, DOI 10.1016/j.cell.2009.11.007

Trau SP, 2020, PEDIATR NEUROL, V104, P62, DOI 10.1016/j.pediatrneurol.2019.09.008

Uchikado Y, 2005, CLIN CANCER RES, V11, P1174

Ulger C, 2003, CANCER GENET CYTOGEN, V147, P28, DOI 10.1016/S0165-4608(03)00155-9

Usami Y, 2008, J PATHOL, V215, P330, DOI 10.1002/path.2365

Wang G, 2017, THERANOSTICS, V7, P3478, DOI 10.7150/thno.20717

Wang LG, 2008, CANCER RES, V68, P2678, DOI 10.1158/0008-5472.CAN-07-6017

Wang M, 2019, PATHOL ONCOL RES, V25, P937, DOI 10.1007/s12253-018-0395-7

Yokoyama A, 2019, NATURE, V565, P312, DOI 10.1038/s41586-018-0811-x

Zhao N, 2015, ONCOTARGET, V6, P18445, DOI 10.18632/oncotarget.4315

Zirkel A, 2013, NUCLEIC ACIDS RES, V41, P6618, DOI 10.1093/nar/gkt410

NR 53

TC 2

Z9 2

U1 1

U2 3

PU ELSEVIER IRELAND LTD

PI CLARE

PA ELSEVIER HOUSE, BROOKVALE PLAZA, EAST PARK SHANNON, CO, CLARE, 00000,

IRELAND

SN 0304-3835

EI 1872-7980

J9 CANCER LETT

JI Cancer Lett.

PD FEB 1

PY 2021

VL 498

BP 98

EP 110

DI 10.1016/j.canlet.2020.10.030

PG 13

WC Oncology

WE Science Citation Index Expanded (SCI-EXPANDED)

SC Oncology

GA PH8TG

UT WOS:000600677200008

PM 33144099

DA 2022-05-19

ER

PT J

AU Xie, XM

Zu, XY

Liu, FF

Wang, T

Wang, XY

Chen, HY

Liu, KD

Wang, PL

Liu, FF

Zheng, Y

Bode, AM

Dong, ZG

Kim, DJ

AF Xie, Xiaomeng

Zu, Xueyin

Liu, Feifei

Wang, Ting

Wang, Xiangyu

Chen, Hanyong

Liu, Kangdong

Wang, Penglei

Liu, Fangfang

Zheng, Yan

Bode, Ann M.

Dong, Zigang

Kim, Dong Joon

TI Purpurogallin is a novel mitogen-activated protein kinase kinase 1/2

inhibitor that suppresses esophageal squamous cell carcinoma growth in

vitro and in vivo

SO MOLECULAR CARCINOGENESIS

LA English

DT Article

DE esophageal squamous cell carcinoma; mitogen-activated protein kinase

kinase; patient-derived xenograft; purpurogallin

ID CANCER; PATHWAY; MEK; TRANSFORMATION; PROLIFERATION; ERK; DEATH

AB Purpurogallin is a natural compound that is extracted from nutgalls and oak bark and it possesses antioxidant, anticancer, and anti-inflammatory properties. However, the anticancer capacity of purpurogallin and its molecular target have not been investigated in esophageal squamous cell carcinoma (ESCC). Herein, we report that purpurogallin suppresses ESCC cell growth by directly targeting the mitogen-activated protein kinase kinase 1/2 (MEK1/2) signaling pathway. We found that purpurogallin inhibits anchorage-dependent and -independent ESCC growth. The results of in vitro kinase assays and cell-based assays indicated that purpurogallin also strongly attenuates the extracellular signal-regulated kinase 1/2 (ERK1/2) signaling pathway and also directly binds to and inhibits MEK1 and MEK2 activity. Furthermore, purpurogallin contributed to S and G2 phase cell cycle arrest by reducing cyclin A2 and cyclin B1 expression and also induced apoptosis by activating poly (ADP ribose) polymerase (PARP). Notably, purpurogallin suppressed patient-derived ESCC tumor growth in an in vivo mouse model. These findings indicated that purpurogallin is a novel MEK1/2 inhibitor that could be useful for treating ESCC.

C1 [Xie, Xiaomeng; Zu, Xueyin; Liu, Feifei; Wang, Ting; Wang, Xiangyu; Liu, Kangdong; Wang, Penglei; Liu, Fangfang; Dong, Zigang; Kim, Dong Joon] China US Henan Hormel Canc Inst, Dept Mol & Cellular Biol, Zhengzhou, Henan, Peoples R China.

[Zu, Xueyin; Wang, Ting; Liu, Kangdong; Wang, Penglei; Liu, Fangfang; Dong, Zigang; Kim, Dong Joon] Zhengzhou Univ, Sch Basic Med Sci, Dept Pathophysiol, Zhengzhou, Henan, Peoples R China.

[Chen, Hanyong; Bode, Ann M.; Dong, Zigang] Univ Minnesota, Hormel Inst, 801 16th Ave NE, Austin, MN 55912 USA.

[Liu, Kangdong; Zheng, Yan] Zhengzhou Univ, Affiliated Canc Hosp, Zhengzhou, Henan, Peoples R China.

[Liu, Kangdong; Dong, Zigang] Collaborat Innovat Ctr Henan Prov Canc Chemopreve, Zhengzhou, Henan, Peoples R China.

[Dong, Zigang] Int Joint Res Ctr Canc Chemoprevent, Zhengzhou, Henan, Peoples R China.

RP Dong, ZG (corresponding author), Univ Minnesota, Hormel Inst, 801 16th Ave NE, Austin, MN 55912 USA.; Kim, DJ (corresponding author), China US Henan Hormel Canc Inst, 127 Dongming Rd, Zhengzhou 450008, Henan, Peoples R China.

EM zgdong@hi.umn.edu; djkim@hci-cn.org

RI Kim, Dong Joon/AAL-8967-2020

FU Key program of Henan Province, China [NO/161100510300]; Henan Joint

Fund, China [No/U1804196]; National Natural Science Foundation China

(NSFC) [NO/81572812]

FX Key program of Henan Province, China, Grant/Award Number: Grant

NO/161100510300; Henan Joint Fund, China, Grant/Award Number: Grant

No/U1804196; National Natural Science Foundation China (NSFC),

Grant/Award Number: Grant NO/81572812

CR Abou-Karam M, 1999, PHYTOTHER RES, V13, P337, DOI 10.1002/(SICI)1099-1573(199906)13:4&lt;337::AID-PTR451&gt;3.0.CO;2-J

Belanger LF, 2003, MOL CELL BIOL, V23, P4778, DOI 10.1128/MCB.23.14.4778-4787.2003

Birner P, 2012, CLIN EXP METASTAS, V29, P551, DOI 10.1007/s10585-012-9470-8

Cagnol S, 2010, FEBS J, V277, P2, DOI 10.1111/j.1742-4658.2009.07366.x

Cao YY, 2018, CELL DEATH DIS, V9, DOI 10.1038/s41419-017-0068-6

Cargnello M, 2011, MICROBIOL MOL BIOL R, V75, P50, DOI 10.1128/MMBR.00031-10

Chen BQ, 2018, CANCER BIOL THER, V19, P76, DOI 10.1080/15384047.2017.1394541

Chiu CY, 2012, MOL CANCER RES, V10, P415, DOI 10.1158/1541-7786.MCR-11-0293

COWLEY S, 1994, CELL, V77, P841, DOI 10.1016/0092-8674(94)90133-3

Ding M, 2001, J BIOL CHEM, V276, P9108, DOI 10.1074/jbc.M007666200

Ferlay J, 2015, INT J CANCER, V136, pE359, DOI 10.1002/ijc.29210

Fung KP, 1996, CHEMOTHERAPY, V42, P199, DOI 10.1159/000239442

Giroux S, 1999, CURR BIOL, V9, P369, DOI 10.1016/S0960-9822(99)80164-X

Gysin S, 2005, CANCER RES, V65, P4870, DOI 10.1158/0008-5472.CAN-04-2848

Handra-Luca A, 2003, AM J PATHOL, V163, P957, DOI 10.1016/S0002-9440(10)63455-4

Hayne C, 2000, J BIOL CHEM, V275, P31876, DOI 10.1074/jbc.M002766200

Hidalgo M, 2014, CANCER DISCOV, V4, P998, DOI 10.1158/2159-8290.CD-14-0001

Jacques PF, 2015, BRIT J NUTR, V114, P1496, DOI 10.1017/S0007114515003141

Kitada S, 2003, J MED CHEM, V46, P4259, DOI 10.1021/jm030190z

Knekt P, 2002, AM J CLIN NUTR, V76, P560, DOI 10.1093/ajcn/76.3.560

Ku SK, 2014, BMB REP, V47, P376, DOI 10.5483/BMBRep.2014.47.7.195

Lee KW, 2007, CARCINOGENESIS, V28, P1918, DOI 10.1093/carcin/bgm110

Li ZG, 2014, MOL CELL BIOL, V34, P3642, DOI 10.1128/MCB.00814-14

Liao CZ, 2010, ACS MED CHEM LETT, V1, P110, DOI 10.1021/ml100020e

Liu FF, 2019, MOL CARCINOGEN, V58, P533, DOI 10.1002/mc.22948

Liu X, 2004, ONCOGENE, V23, P763, DOI 10.1038/sj.onc.1207188

Lugowska I, 2015, ONCOTARGETS THER, V8, P2251, DOI 10.2147/OTT.S72951

Luo H, 2018, ONCOL REP, V39, P2270, DOI 10.3892/or.2018.6304

Maehara O, 2017, CARCINOGENESIS, V38, P1073, DOI 10.1093/carcin/bgx095

MANSOUR SJ, 1994, SCIENCE, V265, P966, DOI 10.1126/science.8052857

Marampon F, 2009, MOL CANCER THER, V8, P543, DOI 10.1158/1535-7163.MCT-08-0570

Neuzillet C, 2014, PHARMACOL THERAPEUT, V141, P160, DOI 10.1016/j.pharmthera.2013.10.001

Normanno N, 2006, GENE, V366, P2, DOI 10.1016/j.gene.2005.10.018

Park HY, 2013, INT J MOL MED, V32, P1171, DOI 10.3892/ijmm.2013.1478

Rescan C, 2005, LAB INVEST, V85, P65, DOI 10.1038/labinvest.3700204

Roberts PJ, 2007, ONCOGENE, V26, P3291, DOI 10.1038/sj.onc.1210422

Rustgi AK, 2014, NEW ENGL J MED, V371, P2499, DOI 10.1056/NEJMra1314530

SHIMADA Y, 1992, CANCER, V69, P277, DOI 10.1002/1097-0142(19920115)69:2<277::AID-CNCR2820690202>3.0.CO;2-C

Sia D, 2015, PHARMACOGENOMICS, V16, P1671, DOI 10.2217/pgs.15.102

Simard FA, 2015, J LEUKOCYTE BIOL, V98, P565, DOI 10.1189/jlb.2MA1214-599R

Wang H, 2017, ONCOTARGET, V8, P45249, DOI 10.18632/oncotarget.16902

Wong CH, 2015, AM J CANCER RES, V5, P3588

Yewale C, 2013, BIOMATERIALS, V34, P8690, DOI 10.1016/j.biomaterials.2013.07.100

Yoshioka M, 2017, J EXP CLIN CANC RES, V36, DOI 10.1186/s13046-017-0572-7

ZHENG CF, 1993, J BIOL CHEM, V268, P11435

Zheng ST, 2011, MOL BIOL REP, V38, P865, DOI 10.1007/s11033-010-0178-z

Zhou J, 2017, NAT COMMUN, V8, DOI 10.1038/ncomms13897

NR 47

TC 10

Z9 12

U1 2

U2 19

PU WILEY

PI HOBOKEN

PA 111 RIVER ST, HOBOKEN 07030-5774, NJ USA

SN 0899-1987

EI 1098-2744

J9 MOL CARCINOGEN

JI Mol. Carcinog.

PD JUL

PY 2019

VL 58

IS 7

BP 1248

EP 1259

DI 10.1002/mc.23007

PG 12

WC Biochemistry & Molecular Biology; Oncology

WE Science Citation Index Expanded (SCI-EXPANDED)

SC Biochemistry & Molecular Biology; Oncology

GA IC2KR

UT WOS:000470789200014

PM 31100197

DA 2022-05-19

ER

PT J

AU Liu, YL

Chen, XJ

Wang, YH

Wang, F

Gong, YY

Zhang, JG

AF Liu, Yongli

Chen, Xiaojun

Wang, Yuhu

Wang, Fang

Gong, Yuyan

Zhang, Jingui

TI Clinical features and prognostic factors for surgical treatment of

esophageal squamous cell carcinoma in elderly patients

SO JOURNAL OF BUON

LA English

DT Article

DE elderly patients; esophageal squamous cell carcinoma; esophagectomy;

prognostic factors; surgery; survival

ID ASSISTED THORACOSCOPIC SURGERY; LAPAROSCOPIC TOTAL GASTRECTOMY;

LONG-TERM OUTCOMES; LUNG-CANCER; SUBLOBAR RESECTION; LOBECTOMY; SURVIVAL

AB Purpose: This research was designed to analyze the clinical features and prognostic factors for surgical treatment of esophageal squamous cell carcinoma (ESCC) in elderly patients aged 70 years and over.

Methods: The clinical data and follow-up data of 68 ESCC patients aged 70 years and over were collected. The characteristics of surgical treatment, perioperative complications, overall survival (OS), and the factors affecting survival were analyzed.

Results: The incidence rate of postoperative complications was 36% and the mortality rate was 0% during and 90 days after surgery. The 5-year OS was 45.0% and the 5-year disease-free survival (DFS) was 38.0%. Univariate analysis showed that gender, Charlson Comorbidity Index (CCI), pathological type, tumor differentiation, depth of invasion, postoperative complications, and lymph node metastasis were the factors associated with OS. Multivariate analysis showed that pathologic type, depth of invasion, and lymph node metastasis were the independent predictors of OS. The ideal long-term survival in elderly patients with ESCC was achieved with radical resection.

Conclusion: The pathological type and pathological stage were the important independent risk factors of prognosis.

C1 [Liu, Yongli; Chen, Xiaojun; Wang, Yuhu; Wang, Fang; Gong, Yuyan; Zhang, Jingui] Cent Hosp Binzhou, Binzhou 251700, Shandong, Peoples R China.

RP Zhang, JG (corresponding author), Cent Hosp Binzhou, Binzhou 251700, Shandong, Peoples R China.

EM ie27ju@163.com

CR Bonavina L, 2003, DIS ESOPHAGUS, V16, P90, DOI 10.1046/j.1442-2050.2003.00300.x

Cijs TM, 2010, ANN THORAC SURG, V90, P900, DOI 10.1016/j.athoracsur.2010.05.039

Dong GC, 2016, ONCOTARGET, V7, P68170, DOI 10.18632/oncotarget.11906

El-Sherif A, 2006, ANN THORAC SURG, V82, P408, DOI 10.1016/j.athoracsur.2006.02.029

Fang WT, 2001, ANN THORAC SURG, V72, P867, DOI 10.1016/S0003-4975(01)02896-X

Ginsberg RJ, 1995, ANN THORAC SURG, V60, P22, DOI [10.1016/0003-4975, DOI 10.1016/0003-4975(95)00537-U]

Ginsberg Robert J., 1995, Annals of Thoracic Surgery, V60, P615, DOI 10.1016/0003-4975(95)00537-U

Internullo E, 2008, EUR J CARDIO-THORAC, V33, P1096, DOI 10.1016/j.ejcts.2008.03.004

Ji WH, 2016, MEDICINE, V95, DOI 10.1097/MD.0000000000004204

Jing W, 2016, MEDICINE, V95, DOI 10.1097/MD.0000000000005630

Kinugasa S, 2001, ANN THORAC SURG, V71, P414, DOI 10.1016/S0003-4975(00)02333-X

Kurokawa Y, 2017, SURG ONCOL CLIN N AM, V26, P213, DOI 10.1016/j.soc.2016.10.011

Li CC, 2016, ONCOTARGET, V7, P71548, DOI 10.18632/oncotarget.12250

Li Y, 2013, WORLD J SURG, V37, P1661, DOI 10.1007/s00268-013-2032-7

Lin Z, 2016, ONCOTARGET, V7, P76261, DOI 10.18632/oncotarget.12842

Liu CY, 2014, WORLD J SURG, V38, P2875, DOI 10.1007/s00268-014-2699-4

Lv YP, 2017, EUR REV MED PHARMACO, V21, P518

Ma JY, 2006, WORLD J GASTROENTERO, V12, P1296, DOI 10.3748/wjg.v12.i8.1296

Mariette C, 2012, J VISC SURG, V149, pE163, DOI 10.1016/j.jviscsurg.2012.03.007

Markar SR, 2013, ANN SURG ONCOL, V20, P1020, DOI 10.1245/s10434-012-2703-x

Masasyesva BG, 2005, INT J CANCER, V113, P1022, DOI 10.1002/ijc.20683

Moskovitz AH, 2006, ANN THORAC SURG, V82, P2031, DOI 10.1016/j.athoracsur.2006.06.053

Oh D, 2016, MEDICINE, V95, DOI 10.1097/MD.0000000000004591

Park HS, 2012, ANN THORAC SURG, V93, P372, DOI 10.1016/j.athoracsur.2011.06.054

Peel J, 2014, ANN THORAC SURG, V98, P706, DOI 10.1016/j.athoracsur.2013.10.058

Peyre CG, 2013, SURG ONCOL CLIN N AM, V22, P15, DOI 10.1016/j.soc.2012.08.010

Pultrum BB, 2010, ANN SURG ONCOL, V17, P1572, DOI 10.1245/s10434-010-0966-7

Shu B, 2016, J BUON, V21, P101

Sun HH, 2016, SEMIN RESP CRIT CARE, V37, P708, DOI 10.1055/s-0036-1592173

Tapias LF, 2013, ANN THORAC SURG, V95, P1741, DOI 10.1016/j.athoracsur.2013.01.084

Thomas P, 1996, EUR J CARDIO-THORAC, V10, P941, DOI 10.1016/S1010-7940(96)80394-0

Tian R, 2016, ONCOTARGET, V7, P67485, DOI 10.18632/oncotarget.11268

Wang Y, 2016, J BUON, V21, P427

Wu HW, 2016, J BUON, V21, P603

Yang HX, 2010, BRIT J SURG, V97, P862, DOI 10.1002/bjs.7005

Yin Z, 2013, ANN SURG ONCOL, V20, P1203, DOI 10.1245/s10434-012-2705-8

Zhang BH, 2016, MEDICINE, V95, DOI 10.1097/MD.0000000000004768

Zhang HD, 2016, ONCOTARGET, V7, P66623, DOI 10.18632/oncotarget.11286

Zhang XM, 2016, J BUON, V21, P118

Zou BW, 2016, MEDICINE, V95, DOI 10.1097/MD.0000000000003507

NR 40

TC 1

Z9 1

U1 0

U2 1

PU IMPRIMATUR PUBLICATIONS

PI ATHENS

PA 30 DEM POLIORKETES ST, ATHENS, 136 76, GREECE

SN 1107-0625

EI 2241-6293

J9 J BUON

JI J. BUON

PD MAY-JUN

PY 2019

VL 24

IS 3

BP 1240

EP 1244

PG 5

WC Oncology

WE Science Citation Index Expanded (SCI-EXPANDED)

SC Oncology

GA ID6CK

UT WOS:000471764000050

PM 31424685

DA 2022-05-19

ER

PT J

AU Yu, WW

Yang, X

Chu, L

Zhao, K

Chen, HQ

Xiang, JQ

Zhang, YW

Li, HC

Zhao, WX

Sun, MH

Wei, Q

Fu, XL

Xie, CY

Zhu, ZF

AF Yu, Weiwei

Yang, Xi

Chu, Li

Zhao, Kuaile

Chen, Haiquan

Xiang, Jiaqing

Zhang, Yawei

Li, Hecheng

Zhao, Weixin

Sun, Menghong

Wei, Qiao

Fu, Xiaolong

Xie, Congying

Zhu, Zhengfei

TI Prognostic value of EGFR family expression in lymph node-negative

esophageal squamous cell carcinoma patients

SO PATHOLOGY RESEARCH AND PRACTICE

LA English

DT Article

DE Esophageal cancer; Esophageal squamous cell carcinoma; Epidermal growth

factor receptor; Prognostic factor; Lymph node-negative

ID EPIDERMAL-GROWTH-FACTOR; FACTOR RECEPTOR FAMILY; CANCER; ERBB4; THERAPY;

OVEREXPRESSION; HER2; PROLIFERATION; AMPLIFICATION; METASTASIS

AB The human epidermal growth factor receptor (EGFR) family has been widely studied in cancer, however, the prognostic role of EGFR family expression in lymph node-negative esophageal squamous cell carcinoma (ESCC) patients have not been invalidated. This study was designed to determine the prognostic value of EGFR family expression in a population of lymph node-negative ESCC patients treated with curative resection. EGFR family protein expression was examined by immunohistochemical analysis of tissue microarrays of 94 patients with lymph node-negative ESCC after radical esophagectomy with three-field lymphadenectomy. Survival differences were compared using Kaplan-Meier analysis. Cox regression analyses were performed to determine the prognostic factors for overall survival and disease-free survival (DFS). ErbB4 expression was found to be an independent prognostic factor for DFS in patients without lymph node metastasis; increased ErbB4 expression was associated with decreased DFS. Additionally, patients with high ErbB4 expression tended to have worse overall survival. EGFR, ErbB2 and ErbB3 expression were not significantly associated with survival in lymph node-negative ESCC patients. Increased ErbB4 immunohistochemical expression was associated with poor prognosis in lymph node-negative ESCC patients.

C1 [Yu, Weiwei; Yang, Xi; Chu, Li; Zhao, Kuaile; Zhao, Weixin; Fu, Xiaolong; Zhu, Zhengfei] Fudan Univ, Shanghai Canc Ctr, Dept Radiat Oncol, Shanghai, Peoples R China.

[Yu, Weiwei] Shanghai Jiao Tong Univ, Affiliated Peoples Hosp 6, Dept Radiat Oncol, Shanghai, Peoples R China.

[Chen, Haiquan; Xiang, Jiaqing; Zhang, Yawei; Li, Hecheng] Fudan Univ, Shanghai Canc Ctr, Dept Thorac Surg, Shanghai, Peoples R China.

[Sun, Menghong; Wei, Qiao] Fudan Univ, Shanghai Canc Ctr, Dept Pathol, Shanghai, Peoples R China.

[Yang, Xi; Chu, Li; Zhao, Kuaile; Chen, Haiquan; Xiang, Jiaqing; Zhang, Yawei; Li, Hecheng; Zhao, Weixin; Sun, Menghong; Wei, Qiao; Zhu, Zhengfei] Fudan Univ, Shanghai Med Coll, Dept Oncol, Shanghai 200032, Peoples R China.

[Fu, Xiaolong] Shanghai Jiao Tong Univ, Shanghai Chest Hosp, Dept Radiat Oncol, Shanghai, Peoples R China.

[Xie, Congying] Wenzhou Med Univ, Affiliated Hosp 1, Radiotherapy & Chemotherapy Dept, Wenzhou, Peoples R China.

RP Zhu, ZF (corresponding author), Fudan Univ, Shanghai Canc Ctr, Dept Radiat Oncol, Shanghai, Peoples R China.

EM fuscczzf@163.com

OI wei, qiao/0000-0001-9476-6071

FU Shanghai Committee of Science and Technology; Natural Science Foundation

of Shanghai, China [15ZR1432100]; Scientific Research Foundation for the

Returned Overseas Chinese Scholars, State Education Ministry; Foundation

of Shanghai Municipal Commission of Health and Family Planning

[201540211]

FX This work was sponsored by the Shanghai Committee of Science and

Technology, Natural Science Foundation of Shanghai, China (No.

15ZR1432100), and Scientific Research Foundation for the Returned

Overseas Chinese Scholars, State Education Ministry, and Foundation of

Shanghai Municipal Commission of Health and Family Planning (No.

201540211). We thank James P. Mahaffey, PhD, from Liwen Bianji, Edanz

Editing China (www.liwenbianji.cn/ac), for editing the English text of a

draft of this manuscript.

CR Chen JQ, 2014, BMC SURG, V14, DOI 10.1186/1471-2482-14-110

Chen Y, 2016, ONCOL LETT, V11, P745, DOI 10.3892/ol.2015.3975

Citri A, 2006, NAT REV MOL CELL BIO, V7, P505, DOI 10.1038/nrm1962

Delektorskaya VV, 2009, B EXP BIOL MED+, V148, P241, DOI 10.1007/s10517-009-0659-z

Elenius K, 1997, EMBO J, V16, P1268, DOI 10.1093/emboj/16.6.1268

Friess H, 1999, WORLD J SURG, V23, P1010, DOI 10.1007/s002689900616

Jiang DX, 2015, BMC CANCER, V15, DOI 10.1186/s12885-015-1393-8

Kato H, 2013, INT J ONCOL, V42, P1151, DOI 10.3892/ijo.2013.1830

Lee CM, 2005, GYNECOL ONCOL, V99, P415, DOI 10.1016/j.ygyno.2005.05.045

Lin G, 2015, ONCOL LETT, V10, P901, DOI 10.3892/ol.2015.3277

Ma G, 2015, EJSO-EUR J SURG ONC, V41, P541, DOI 10.1016/j.ejso.2015.01.013

Mendoza-Naranjo A, 2013, EMBO MOL MED, V5, P1087, DOI 10.1002/emmm.201202343

Ohashi Y, 2016, OR SURG OR MED OR PA, V122, P313, DOI 10.1016/j.oooo.2016.04.017

Riese DJ, 1998, J BIOL CHEM, V273, P11288, DOI 10.1074/jbc.273.18.11288

Riese DJ, 1996, ONCOGENE, V12, P345

Siegel R, 2013, CA-CANCER J CLIN, V63, P11, DOI 10.3322/caac.21166

Skliris GP, 2008, HISTOPATHOLOGY, V52, P355, DOI 10.1111/j.1365-2559.2007.02955.x

Starr A, 2006, INT J CANCER, V119, P269, DOI 10.1002/ijc.21818

Tebbutt N, 2013, NAT REV CANCER, V13, P663, DOI 10.1038/nrc3559

Wang HX, 2016, TUMOR BIOL, V37, P9771, DOI 10.1007/s13277-015-4692-4

Wang J, 2016, ONCOTARGET, V7, P76693, DOI 10.18632/oncotarget.12485

Wei QC, 2007, INT J ONCOL, V31, P493

Williams CS, 2015, CARCINOGENESIS, V36, P710, DOI 10.1093/carcin/bgv049

Xu S, 2008, J SURG ONCOL, V97, P44, DOI 10.1002/jso.20892

Yarden Y, 2001, NAT REV MOL CELL BIO, V2, P127, DOI 10.1038/35052073

Yu T, 2015, ONCOGENE, V34, P413, DOI 10.1038/onc.2013.574

Yu WW, 2011, HEPATO-GASTROENTEROL, V58, P426

Zhan N, 2012, MED ONCOL, V29, P933, DOI 10.1007/s12032-011-9850-y

Zhang GY, 2010, J CANCER RES CLIN, V136, P587, DOI 10.1007/s00432-009-0693-2

ZHANG MX, 2014, J EXP CLIN CANC RES, V33

Zhu ZF, 2015, J EXP CLIN CANC RES, V34, DOI 10.1186/s13046-015-0212-z

Zhu ZF, 2014, ANN SURG ONCOL, V21, P2857, DOI 10.1245/s10434-014-3665-y

Zhu ZF, 2013, ANN SURG ONCOL, V20, P3052, DOI 10.1245/s10434-013-2987-5

NR 33

TC 4

Z9 5

U1 0

U2 5

PU ELSEVIER GMBH, URBAN & FISCHER VERLAG

PI JENA

PA OFFICE JENA, P O BOX 100537, 07705 JENA, GERMANY

SN 0344-0338

J9 PATHOL RES PRACT

JI Pathol. Res. Pract.

PD JUL

PY 2018

VL 214

IS 7

BP 1017

EP 1023

DI 10.1016/j.prp.2018.04.017

PG 7

WC Pathology

WE Science Citation Index Expanded (SCI-EXPANDED)

SC Pathology

GA GN6YY

UT WOS:000439254000014

PM 29729836

DA 2022-05-19

ER

PT J

AU Ma, ZB

Guo, GH

Niu, Q

Shi, N

AF Ma, Zhen-Bin

Guo, Guang-Hong

Niu, Qiong

Shi, Ning

TI Role of EZH2 Polymorphisms in Esophageal Squamous Cell Carcinoma Risk in

Han Chinese Population

SO INTERNATIONAL JOURNAL OF MOLECULAR SCIENCES

LA English

DT Article

DE allele; EZH2; esophageal squamous cell carcinoma; polymorphism

ID GROUP PROTEIN EZH2; COLORECTAL-CANCER; ZESTE HOMOLOG-2; HIGH EXPRESSION;

POOR-PROGNOSIS; GENE; PROLIFERATION; CHEMORADIOTHERAPY; PROSTATE;

ENHANCER

AB Gene single nucleotide polymorphisms play a critical role in the development of esophageal squamous cell carcinoma (ESCC). The aim of this study is to investigate the associations between EZH2 gene polymorphisms and ESCC risk. We undertook a case-control study to analyze three EZH2 polymorphisms (148505302C > T, 2110 + 6A > C and 626 - 394T > C) in an Han Chinese population, by extraction of genomic DNA from the peripheral blood of 476 patients with ESCC and 492 control participants, and performed EZH2 genotyping using DNA sequencing. The obtained results indicated that overall, no statistically significant association was observed in 148505302C > T and 2110 + 6A > C. However, 626 - 394T > C genotype was at increased risk of ESCCs (p = 0.006; odds ratio (OR) = 1.131, CI 95%: 1.034-1.236). Moreover, 626 - 394C/C genotype ESCCs were more significantly common in patients with tumor size of > 5 cm than T allele ESCC and in cases of poor differentiation and lower advanced pathological stage. In conclusion, polymorphism in 626 - 394T > C was observed to be associated with susceptibility of ESCC. Nevertheless, further investigation with a larger sample size is needed to support our results.

C1 [Ma, Zhen-Bin; Niu, Qiong; Shi, Ning] Binzhou Med Coll, Affiliated Hosp, Dept Gastroenterol, Binzhou 256600, Shandong, Peoples R China.

[Guo, Guang-Hong] Binzhou Med Coll, Affiliated Hosp, Dept Gynaecol, Binzhou 256600, Shandong, Peoples R China.

RP Guo, GH (corresponding author), Binzhou Med Coll, Affiliated Hosp, Dept Gynaecol, 661 Yellow River Second St, Binzhou 256600, Shandong, Peoples R China.

EM mazhenbin@yeah.net; gghbyfy@yeah.net; niuqiong@126.com;

shiningbyfy@yeah.net

CR Bachmann IM, 2006, J CLIN ONCOL, V24, P268, DOI 10.1200/JCO.2005.01.5180

Cardoso C, 2000, EUR J HUM GENET, V8, P174, DOI 10.1038/sj.ejhg.5200439

Collett K, 2006, CLIN CANCER RES, V12, P1168, DOI 10.1158/1078-0432.CCR-05-1533

Crea F, 2012, ANN ONCOL, V23, P1207, DOI 10.1093/annonc/mdr387

Di Fiore F, 2006, WORLD J GASTROENTERO, V12, P4185

Edge S., 2010, AJCC CANC STAGING HD

Fornaro L, 2012, ANN ONCOL, V23, P1370, DOI 10.1093/annonc/mds031

He LR, 2010, INT J CANCER, V127, P138, DOI 10.1002/ijc.25031

HU JF, 1994, INT J CANCER, V57, P38, DOI 10.1002/ijc.2910570108

Jacobs JJL, 1999, NATURE, V397, P164, DOI 10.1038/16476

Jemal A, 2011, CA-CANCER J CLIN, V61, P69, DOI [10.3322/caac.20107, 10.3322/caac.20115]

Kidani K, 2009, ORAL ONCOL, V45, P39, DOI 10.1016/j.oraloncology.2008.03.016

Kleer CG, 2003, P NATL ACAD SCI USA, V100, P11606, DOI 10.1073/pnas.1933744100

Lipkin SM, 2010, CANCER PREV RES, V3, P597, DOI 10.1158/1940-6207.CAPR-10-0007

Lund AH, 2004, CURR OPIN CELL BIOL, V16, P239, DOI 10.1016/j.ceb.2004.03.010

Mathews LA, 2009, DIFFERENTIATION, V78, P1, DOI 10.1016/j.diff.2009.04.002

Matsukawa Y, 2006, CANCER SCI, V97, P484, DOI 10.1111/j.1349-7006.2006.00203.x

Mimori K, 2005, EJSO-EUR J SURG ONC, V31, P376, DOI 10.1016/j.ejso.2004.11.001

Nazir M, 2011, ASIAN PAC J CANCER P, V12, P2767

Rohatgi PR, 2005, CANCER-AM CANCER SOC, V104, P1349, DOI 10.1002/cncr.21346

Sasaki M, 2008, J PATHOL, V215, P175, DOI 10.1002/path.2345

Shen Z, 2010, J CELL MOL MED, V14, P226, DOI 10.1111/j.1582-4934.2008.00370.x

Simon JA, 2008, MUTAT RES-FUND MOL M, V647, P21, DOI 10.1016/j.mrfmmm.2008.07.010

Suntharalingam M, 2007, SEMIN RADIAT ONCOL, V17, P22, DOI 10.1016/j.semradonc.2006.09.008

Tonini T, 2008, J CELL PHYSIOL, V214, P295, DOI 10.1002/jcp.21241

Valk-Lingbeek ME, 2004, CELL, V118, P409, DOI 10.1016/j.cell.2004.08.005

van Kemenade FJ, 2001, BLOOD, V97, P3896, DOI 10.1182/blood.V97.12.3896

Varambally S, 2002, NATURE, V419, P624, DOI 10.1038/nature01075

Wang J, 2014, MED ONCOL, V31, DOI 10.1007/s12032-014-0874-y

Yoon KA, 2010, J THORAC ONCOL, V5, P10, DOI 10.1097/JTO.0b013e3181c422d9

Zhang W, 2011, ANN ONCOL, V22, P104, DOI 10.1093/annonc/mdq315

Zhou LQ, 2013, PLOS ONE, V8, DOI 10.1371/journal.pone.0064331

Zhu M.L., 2012, PLOS ONE, V7

NR 33

TC 8

Z9 8

U1 0

U2 6

PU MDPI

PI BASEL

PA ST ALBAN-ANLAGE 66, CH-4052 BASEL, SWITZERLAND

EI 1422-0067

J9 INT J MOL SCI

JI Int. J. Mol. Sci.

PD JUL

PY 2014

VL 15

IS 7

BP 12688

EP 12697

DI 10.3390/ijms150712688

PG 10

WC Biochemistry & Molecular Biology; Chemistry, Multidisciplinary

WE Science Citation Index Expanded (SCI-EXPANDED)

SC Biochemistry & Molecular Biology; Chemistry

GA AM7ID

UT WOS:000340038500092

PM 25036033

OA Green Published, gold, Green Submitted

DA 2022-05-19

ER

PT J

AU Chen, DY

Jiang, RY

Mao, CM

Shi, L

Wang, SJ

Yu, LC

Hu, Q

Dai, DF

Xu, HX

AF Chen, Deyu

Jiang, Riyue

Mao, Chaoming

Shi, Liang

Wang, Shengjun

Yu, Lichao

Hu, Qin

Dai, Dongfang

Xu, Huaxi

TI Chemokine/chemokine receptor interactions contribute to the accumulation

of Th17 cells in patients with esophageal squamous cell carcinoma

SO HUMAN IMMUNOLOGY

LA English

DT Article

DE Esophageal squamous cell carcinoma; Th17 cells; Chemokine receptor;

Chemokine

ID CD4(+) T-CELLS; GROWTH-FACTOR-BETA; PROINFLAMMATORY CYTOKINES;

INFILTRATING LYMPHOCYTES; CHEMOKINE RECEPTORS; CUTTING EDGE; GENERATION;

DIFFERENTIATION; T(H)17; INFLAMMATION

AB Chemokine/chemokine receptor interactions play a critical role in lymphocyte infiltration of tumors. Recent studies suggest that Th17 cells accumulate within many types of tumors, although the mechanisms that control this are unclear. We studied the distribution and phenotypic features of Th17 cells chemokine receptors, as well as the mRNA levels of CCL2, CCL17, CCL20, and CCL22 in tumors of patients with esophageal squamous cell carcinoma. We found that Th17 cells accumulated in tumors, and high expressions of CCR4, CCR6 were detected in Th17 cells. Levels of the chemokines CCL17, CCL20, and CCL22 in tumors were significantly higher than in tumor-free tissues, and were positively correlated with the distribution of Th17 cells in tumors. Furthermore, an in vitro migration assay showed that CCL17, CCL20 and CCL22 had chemotactic effects on tumor-derived Th17 cells. In conclusion, the CCR4-CCL17/22 and CCR6-CCL20 axis might play an important role in Th17 cell infiltration of tumors. (c) 2012 American Society for Histocompatibility and Immunogenetics. Published by Elsevier Inc. All rights reserved.

C1 [Chen, Deyu; Jiang, Riyue; Hu, Qin; Dai, Dongfang] Jiangsu Univ, Affiliated Hosp, Inst Oncol, Zhenjiang 212001, Peoples R China.

[Mao, Chaoming; Shi, Liang] Jiangsu Univ, Dept Nucl Med, Sch Med Sci & Lab Med, Zhenjiang 212001, Peoples R China.

[Wang, Shengjun; Xu, Huaxi] Jiangsu Univ, Dept Immunol & Lab Immunol, Sch Med Sci & Lab Med, Zhenjiang 212001, Peoples R China.

[Yu, Lichao] Jiangsu Univ, Affiliated Hosp, Dept Thorac Surg, Zhenjiang 212001, Peoples R China.

RP Chen, DY (corresponding author), Jiangsu Univ, Affiliated Hosp, Inst Oncol, Zhenjiang 212001, Peoples R China.

EM cdeyu@hotmail.com; jq1001@ujs.edu.cn

OI Wang, Shengjun/0000-0001-6584-1183

FU Health Department Foundation of Jiangsu Province [H200944]; National

Natural Science Foundation of China [30972748, 30871193, 81072453];

Science and Technology Commission of Zhenjiang Municipality [SH2011019]

FX This work was supported by the Health Department Foundation of Jiangsu

Province (H200944), the National Natural Science Foundation of China

(30972748, 30871193 and 81072453), and the Science and Technology

Commission of Zhenjiang Municipality (SH2011019).

CR Acosta-Rodriguez EV, 2007, NAT IMMUNOL, V8, P639, DOI 10.1038/ni1467

Bettelli E, 2006, NATURE, V441, P235, DOI 10.1038/nature04753

Bonecchi R, 1998, J EXP MED, V187, P129, DOI 10.1084/jem.187.1.129

Brucklacher-Waldert V, 2009, BRAIN, V132, P3329, DOI 10.1093/brain/awp289

Cavassani KA, 2006, J IMMUNOL, V177, P5811, DOI 10.4049/jimmunol.177.9.5811

Gao P, 2003, J LEUKOCYTE BIOL, V73, P273, DOI 10.1189/jlb.0602269

Gu DS, 2010, HUM IMMUNOL, V71, P586, DOI 10.1016/j.humimm.2010.02.010

Hoglund RA, 2011, HUM IMMUNOL, V72, P124, DOI 10.1016/j.humimm.2010.10.016

Iellem A, 2001, J EXP MED, V194, P847, DOI 10.1084/jem.194.6.847

Kennedy R, 2008, IMMUNOL REV, V222, P129, DOI 10.1111/j.1600-065X.2008.00616.x

Kryczek I, 2009, BLOOD, V114, P1141, DOI 10.1182/blood-2009-03-208249

Leipe J, 2010, ARTHRITIS RHEUM-US, V62, P2876, DOI 10.1002/art.27622

Lim HW, 2008, J IMMUNOL, V180, P122, DOI 10.4049/jimmunol.180.1.122

Liu JK, 2011, BIOCHEM BIOPH RES CO, V407, P348, DOI 10.1016/j.bbrc.2011.03.021

Loetscher P, 1998, NATURE, V391, P344, DOI 10.1038/34814

Lv L, 2011, PLOS ONE, V6, DOI 10.1371/journal.pone.0018219

Manel N, 2008, NAT IMMUNOL, V9, P641, DOI 10.1038/ni.1610

Maniati E, 2010, ONCOGENE, V29, P5653, DOI 10.1038/onc.2010.367

Martin-Orozco N, 2009, CURR OPIN INVEST DR, V10, P543

Maruyama T, 2010, CANCER SCI, V101, P1947, DOI 10.1111/j.1349-7006.2010.01624.x

Miyahara Y, 2008, P NATL ACAD SCI USA, V105, P15505, DOI 10.1073/pnas.0710686105

Murugaiyan G, 2009, J IMMUNOL, V183, P4169, DOI 10.4049/jimmunol.0901017

Pelletier M, 2010, BLOOD, V115, P335, DOI 10.1182/blood-2009-04-216085

Sallusto F, 1998, J EXP MED, V187, P875, DOI 10.1084/jem.187.6.875

Sato W, 2007, J IMMUNOL, V178, P7525, DOI 10.4049/jimmunol.178.12.7525

Sfanos KS, 2008, CLIN CANCER RES, V14, P3254, DOI 10.1158/1078-0432.CCR-07-5164

Singh SP, 2008, J IMMUNOL, V180, P214, DOI 10.4049/jimmunol.180.1.214

Strober W, 2010, CURR OPIN GASTROEN, V26, P310, DOI 10.1097/MOG.0b013e328339d099

Su XM, 2010, J IMMUNOL, V184, P1630, DOI 10.4049/jimmunol.0902813

Volpe E, 2008, NAT IMMUNOL, V9, P650, DOI 10.1038/ni.1613

Wang DZ, 2009, CURR OPIN PHARMACOL, V9, P688, DOI 10.1016/j.coph.2009.08.003

Webb A, 2008, J LEUKOCYTE BIOL, V84, P1202, DOI 10.1189/jlb.0408234

Yang L, 2008, NATURE, V454, P350, DOI 10.1038/nature07021

Ye ZJ, 2010, J IMMUNOL, V185, P6348, DOI 10.4049/jimmunol.1001728

Zhang B, 2008, BIOCHEM BIOPH RES CO, V374, P533, DOI 10.1016/j.bbrc.2008.07.060

Zhang JY, 2010, HEPATOLOGY, V51, P81, DOI 10.1002/hep.23273

Zhang JP, 2009, J HEPATOL, V50, P980, DOI 10.1016/j.jhep.2008.12.033

Zingoni A, 1998, J IMMUNOL, V161, P547

NR 38

TC 30

Z9 30

U1 0

U2 8

PU ELSEVIER SCIENCE INC

PI NEW YORK

PA STE 800, 230 PARK AVE, NEW YORK, NY 10169 USA

SN 0198-8859

EI 1879-1166

J9 HUM IMMUNOL

JI Hum. Immunol.

PD NOV

PY 2012

VL 73

IS 11

BP 1068

EP 1072

DI 10.1016/j.humimm.2012.07.333

PG 5

WC Immunology

WE Science Citation Index Expanded (SCI-EXPANDED)

SC Immunology

GA 032KO

UT WOS:000310717500002

PM 22863447

DA 2022-05-19

ER

PT J

AU Megumi, K

Ishigami, S

Uchikado, Y

Kita, Y

Okumura, H

Matsumoto, M

Uenosono, Y

Arigami, T

Kijima, Y

Kitazono, M

Shinchi, H

Ueno, S

Natsugoe, S

AF Megumi, Koichi

Ishigami, Sumiya

Uchikado, Yasuto

Kita, Yoshiaki

Okumura, Hiroshi

Matsumoto, Masataka

Uenosono, Yoshikazu

Arigami, Takaaki

Kijima, Yuko

Kitazono, Masaki

Shinchi, Hiroyuki

Ueno, Shinichi

Natsugoe, Shoji

TI Clinicopathological Significance of BMP7 Expression in Esophageal

Squamous Cell Carcinoma

SO ANNALS OF SURGICAL ONCOLOGY

LA English

DT Article

ID BONE MORPHOGENETIC PROTEIN-7; EPITHELIAL-MESENCHYMAL TRANSITIONS; BETA

SUPERFAMILY; HUMAN PROSTATE; IN-VIVO; CANCER; RELEVANCE; GROWTH;

PROLIFERATION; HOMEOSTASIS

AB Bone morphogenetic proteins (BMPs) are secreted signaling molecules belonging to the transforming growth factor-beta (TGF-beta) superfamily of growth factors. Recent studies have shown that the influence of the expression of BMP7 was altered in several tumors. The purpose of the current study was to examine the expression of BMP7 in esophageal squamous cell carcinoma and to clarify the clinical impact of BMP7 expression in esophageal squamous cell carcinoma (ESCC).

A total of 180 patients with ESCC who underwent surgical resection from 1991 to 2004 were eligible for this study. The expression of BMP7 in esophageal tumor tissues was examined immunohistochemically.

BMP7 expression was found in the cytoplasm of cancer cells. BMP7 positivity was observed in 61.7% of tumors. The BMP7-positive group had deeper progression, more advanced stages, and greater venous invasion than those without BMP7 expression ( < 0.001, < 0.005, and < 0.0005, respectively). In addition, expression of BMP7 correlated with poorer prognosis ( < 0.0005). Multivariate analysis showed that BMP7 expression status was an independent prognostic factor ( < 0.05).

Patients with expression of BMP7 in ESCC had high malignant potential. BMP7 could be a useful prognostic marker for patients with ESCC.

C1 [Megumi, Koichi; Ishigami, Sumiya; Uchikado, Yasuto; Kita, Yoshiaki; Okumura, Hiroshi; Matsumoto, Masataka; Uenosono, Yoshikazu; Arigami, Takaaki; Kijima, Yuko; Kitazono, Masaki; Shinchi, Hiroyuki; Ueno, Shinichi; Natsugoe, Shoji] Kagoshima Univ, Dept Surg Oncol, Grad Sch Med, Kagoshima 890, Japan.

RP Megumi, K (corresponding author), Kagoshima Univ, Dept Surg Oncol, Grad Sch Med, Kagoshima 890, Japan.

EM aircarter@goo.jp

CR Alarmo EL, 2008, ANN ONCOL, V19, P308, DOI 10.1093/annonc/mdm453

Alarmo EL, 2006, GENE CHROMOSOME CANC, V45, P411, DOI 10.1002/gcc.20307

Aoki M, 2011, BRIT J CANCER, V104, P714, DOI 10.1038/sj.bjc.6606075

Attisano L, 2002, SCIENCE, V296, P1646, DOI 10.1126/science.1071809

Beck SE, 2007, CELL SIGNAL, V19, P1465, DOI 10.1016/j.cellsig.2007.01.017

Buijs JT, 2007, AM J PATHOL, V171, P1047, DOI 10.2353/ajpath.2007.070168

Chan KW, 2006, J CLIN PATHOL, V59, P101, DOI 10.1136/jcp.2005.027508

DUDLEY AT, 1995, GENE DEV, V9, P2795, DOI 10.1101/gad.9.22.2795

Kazuo M, 2008, ANN SURG ONCOL, V15, P1530, DOI 10.1245/s10434-007-9746-4

Kita Y, 2006, BRIT J CANCER, V95, P634, DOI 10.1038/sj.bjc.6603296

Kwak C, 2007, J UROLOGY, V178, P1062, DOI 10.1016/j.juro.2007.05.005

Liu WL, 2010, BMC CANCER, V10, DOI 10.1186/1471-2407-10-467

LUO G, 1995, GENE DEV, V9, P2808, DOI 10.1101/gad.9.22.2808

Massague J, 2005, GENE DEV, V19, P2783, DOI 10.1101/gad.1350705

Masuda H, 2004, PROSTATE, V59, P101, DOI 10.1002/pros.20030

Milan M, 2007, NAT CELL BIOL, V9, P17, DOI 10.1038/ncb0107-17

Miyazaki H, 2004, ONCOGENE, V23, P9326, DOI 10.1038/sj.onc.1208127

NAITOH H, 1995, AM J PATHOL, V146, P1161

Natsugoe S, 2002, CLIN CANCER RES, V8, P1838

Patel SR, 2005, TRENDS MOL MED, V11, P512, DOI 10.1016/j.molmed.2005.09.007

Peinado H, 2003, J BIOL CHEM, V278, P21113, DOI 10.1074/jbc.M211304200

Piek E, 1999, J CELL SCI, V112, P4557

Rothhammer Tania, 2007, Cancer Biomarkers, V3, P111

Simic P, 2005, CYTOKINE GROWTH F R, V16, P299, DOI 10.1016/j.cytogfr.2005.02.010

Sobin LH, 1997, CANCER, V80, P1803, DOI 10.1002/(SICI)1097-0142(19971101)80:9<1803::AID-CNCR16>3.0.CO;2-9

Sulzbacher I, 2002, J CLIN PATHOL, V55, P381, DOI 10.1136/jcp.55.5.381

Suzuki S, 2011, ANN SURG ONCOL, V18, P888, DOI 10.1245/s10434-010-1387-3

Takeno S, 2002, CANCER, V94, P2874, DOI 10.1002/cncr.10542

Thiery JP, 2002, NAT REV CANCER, V2, P442, DOI 10.1038/nrc822

Vukicevic S, 1996, P NATL ACAD SCI USA, V93, P9021, DOI 10.1073/pnas.93.17.9021

Wang SN, 2003, AM J PHYSIOL-RENAL, V284, pF1006, DOI 10.1152/ajprenal.00382.2002

Wang SN, 2001, J AM SOC NEPHROL, V12, P2392, DOI 10.1681/ASN.V12112392

Zeisberg EM, 2007, NAT MED, V13, P952, DOI 10.1038/nm1613

Zeisberg M, 2003, NAT MED, V9, P964, DOI 10.1038/nm888

NR 34

TC 23

Z9 24

U1 1

U2 2

PU SPRINGER

PI NEW YORK

PA 233 SPRING ST, NEW YORK, NY 10013 USA

SN 1068-9265

EI 1534-4681

J9 ANN SURG ONCOL

JI Ann. Surg. Oncol.

PD JUN

PY 2012

VL 19

IS 6

BP 2066

EP 2071

DI 10.1245/s10434-011-2024-5

PG 6

WC Oncology; Surgery

WE Science Citation Index Expanded (SCI-EXPANDED)

SC Oncology; Surgery

GA 944NF

UT WOS:000304209800047

PM 21913019

OA Green Published, hybrid

DA 2022-05-19

ER

PT J

AU Xie, YH

Li, LY

He, JZ

Xu, XE

Liao, LD

Zhang, Q

Xie, JJ

Xu, LY

Li, EM

AF Xie, Ying-Hua

Li, Li-Yan

He, Jian-Zhong

Xu, Xiu-E

Liao, Lian-Di

Zhang, Qiang

Xie, Jian-Jun

Xu, Li-Yan

Li, En-Min

TI Heat shock protein family B member 1 facilitates ezrin activation to

control cell migration in esophageal squamous cell carcinoma

SO INTERNATIONAL JOURNAL OF BIOCHEMISTRY & CELL BIOLOGY

LA English

DT Article

DE Ezrin; HSPB1; Activation; Migration; Esophageal squamous cell carcinoma

(ESCC)

ID REGULATES INVASION; TERMINAL REGION; BINDING; PHOSPHORYLATION; DOMAIN;

METASTASIS; MOESIN; IDENTIFICATION; INVASIVENESS; EXPRESSION

AB Ezrin plays an important role in the development and progression of human esophageal squamous cell carcinoma (ESCC), providing a link between the cortical actin cytoskeleton and the plasma membrane to govern membrane structure and protrusions. However, the mechanism by which ezrin is activated still remains unknown in ESCC. Here, we identify a novel interaction between ezrin and heat shock protein family B (small) member 1 (HSPB1) in ESCC cells by mass spectroscopy and co-immunoprecipitation. HSPB1 only interacts with inactive ezrin and binds to the alpha-helical coiled coil region of ezrin. Knockdown of HSPB1 resulted to the decline of phosphorylation at ezrin Thr567, markedly suppressing the ability of ezrin to bind to the actin cytoskeleton and migration of ESCC cells. Furthermore, neither the constitutively active phosphomimetic ezrin T567D, nor inactivated ezrin T567A could restore cell migration following HSPB1 knockdown. Low HSPB1 expression was associated with favorable overall survival of ESCC patients. Taken together, HSPB1, as an important partner, participates in the activation of ezrin and merits further evaluation as a novel therapeutic target against human ESCC.

C1 [Xie, Ying-Hua; Li, Li-Yan; He, Jian-Zhong; Xu, Xiu-E; Liao, Lian-Di; Zhang, Qiang; Xie, Jian-Jun; Xu, Li-Yan; Li, En-Min] Shantou Univ, Med Coll, Key Lab Mol Biol High Canc Incidence Coastal Chao, Shantou 515041, Guangdong, Peoples R China.

[Xie, Ying-Hua; Li, Li-Yan; He, Jian-Zhong; Zhang, Qiang; Xie, Jian-Jun; Li, En-Min] Shantou Univ, Med Coll, Dept Biochem & Mol Biol, Shantou 515091, Guangdong, Peoples R China.

[Xu, Xiu-E; Liao, Lian-Di; Xu, Li-Yan] Shantou Univ, Med Coll, Inst Oncol Pathol, Shantou 515041, Guangdong, Peoples R China.

RP Xu, LY (corresponding author), Shantou Univ, Med Coll, Key Lab Mol Biol High Canc Incidence Coastal Chao, Shantou 515041, Guangdong, Peoples R China.; Li, EM (corresponding author), Shantou Univ, Med Coll, Dept Biochem & Mol Biol, Shantou 515091, Guangdong, Peoples R China.

EM lyxu@stu.edu.cn; nmli@stu.edu.cn

OI He, Jian-Zhong/0000-0003-1256-472X

FU National Natural Science Foundation of China [81172264, 81472613,

81872372]; Natural Science Foundation of China-Guangdong Joint Fund

[U0932001]

FX We thank Dr. Stanley Li Lin from the Department of Cell Biology and

Genetics of Shantou University Medical College for assistance in

revising the manuscript. This work was supported by grants from the

Natural Science Foundation of China-Guangdong Joint Fund (No.U0932001)

and the National Natural Science Foundation of China (No.81172264,

81472613 and No. 81872372).

CR Adada M, 2014, BBA-MOL CELL BIOL L, V1841, P727, DOI 10.1016/j.bbalip.2013.07.002

Ben-Aissa K, 2012, J BIOL CHEM, V287, P16311, DOI 10.1074/jbc.M111.304881

Blin G, 2008, BIOPHYS J, V94, P1021, DOI 10.1529/biophysj.107.110213

Bruey JM, 2000, ONCOGENE, V19, P4855, DOI 10.1038/sj.onc.1203850

Camp RL, 2004, CLIN CANCER RES, V10, P7252, DOI 10.1158/1078-0432.CCR-04-0713

Doshi BM, 2010, ANN NY ACAD SCI, V1197, P76, DOI 10.1111/j.1749-6632.2010.05191.x

Fanelli MA, 2008, CELL STRESS CHAPERON, V13, P207, DOI 10.1007/s12192-007-0007-z

Fievet BT, 2004, J CELL BIOL, V164, P653, DOI 10.1083/jcb.200307032

Garrido C, 1998, CANCER RES, V58, P5495

GARY R, 1995, MOL BIOL CELL, V6, P1061, DOI 10.1091/mbc.6.8.1061

Gibert B, 2012, BRIT J CANCER, V107, P63, DOI 10.1038/bjc.2012.188

GOULD KL, 1989, EMBO J, V8, P4133, DOI 10.1002/j.1460-2075.1989.tb08598.x

Hao JJ, 2009, J CELL BIOL, V184, P451, DOI 10.1083/jcb.200807047

He JZ, 2017, HUM PATHOL, V66, P115, DOI 10.1016/j.humpath.2017.06.003

Katsogiannou M, 2014, FRONT GENET, V5, DOI 10.3389/fgene.2014.00346

Li LY, 2017, INT J BIOCHEM CELL B, V88, P162, DOI 10.1016/j.biocel.2017.05.018

Liu W, 2018, BR J CANC

Lv GQ, 2014, BIOCHEM CELL BIOL, V92, P379, DOI 10.1139/bcb-2014-0046

Mak H, 2012, BMC CANCER, V12, DOI 10.1186/1471-2407-12-82

Mischak H, 2010, SCI TRANSL MED, V2, DOI 10.1126/scitranslmed.3001249

Miyake H, 2006, ANTICANCER RES, V26, P1583

Mori T, 2008, J BIOL CHEM, V283, P29602, DOI 10.1074/jbc.M803606200

NIGGLI V, 1995, FEBS LETT, V376, P172, DOI 10.1016/0014-5793(95)01270-1

Pearson MA, 2000, CELL, V101, P259, DOI 10.1016/S0092-8674(00)80836-3

Reczek D, 1998, J BIOL CHEM, V273, P18452, DOI 10.1074/jbc.273.29.18452

Sauvanet C, 2015, ANNU REV CELL DEV BI, V31, P593, DOI 10.1146/annurev-cellbio-100814-125234

Xie JJ, 2011, J SURG ONCOL, V104, P538, DOI 10.1002/jso.21909

Xie JJ, 2010, AM J PATHOL, V176, P939, DOI 10.2353/ajpath.2010.090118

Xie JJ, 2009, INT J CANCER, V124, P2549, DOI 10.1002/ijc.24216

Yonemura S, 2002, J CELL SCI, V115, P2569

Zeng FM, 2017, AMINO ACIDS, V49, P943, DOI 10.1007/s00726-017-2398-1

Zhang XD, 2018, NUCLEIC ACIDS RES, V46, P1793, DOI 10.1093/nar/gkx1259

NR 32

TC 3

Z9 3

U1 1

U2 5

PU PERGAMON-ELSEVIER SCIENCE LTD

PI OXFORD

PA THE BOULEVARD, LANGFORD LANE, KIDLINGTON, OXFORD OX5 1GB, ENGLAND

SN 1357-2725

EI 1878-5875

J9 INT J BIOCHEM CELL B

JI Int. J. Biochem. Cell Biol.

PD JUL

PY 2019

VL 112

BP 79

EP 87

DI 10.1016/j.biocel.2019.05.005

PG 9

WC Biochemistry & Molecular Biology; Cell Biology

WE Science Citation Index Expanded (SCI-EXPANDED)

SC Biochemistry & Molecular Biology; Cell Biology

GA IF1CJ

UT WOS:000472814600010

PM 31082616

DA 2022-05-19

ER

PT J

AU Razavi, M

Ghorbian, S

AF Razavi, Maryam

Ghorbian, Saeid

TI UP-REGULATION OF LONG NON-CODING RNA-PCAT-1 PROMOTES INVASION AND

METASTASIS IN ESOPHAGEAL SQUAMOUS CELL CARCINOMA

SO EXCLI JOURNAL

LA English

DT Article

DE LncRNA-PCAT-1; esophageal squamous cell carcinoma; ESCC; lncRNA

ID RNA PCAT-1; CANCER; EXPRESSION; GROWTH; GENE

AB Long non-coding RNA prostate cancer associated transcript-1 ncRNA (lncRNA-PCAT-1) plays an important role in the progression of prostate cancer. The present investigation was aimed to evaluate the potential roles of the lncRNA-PCAT-1 gene expression changes in esophageal squamous cell carcinoma (ESCC) between Iranian population. In the case-control investigation, we have analyzed a total of 150 fresh tissue samples, compromising of 75 cancerous tissues and 75 adjacent normal tissues from patients with ESCC. We used quantitative Real-time polymerase chain reaction (qRT-PCR) to evaluate the lncRNA-PCAT-1 gene expression levels in ESCC patients and correlation between the lncRNA-PCAT-1 expression changes and clinical characteristics. Our findings showed that the lncRNA-PCAT-1 gene was up-regulated in cancerous tissues compared with the adjacent non-cancerous tissues (p=0.0016). In addition, the results revealed a significant correlation between up-regulating of lncRNAPCAT-1 and hot liquid drinking (p =0.017). These findings offer the potential roles of lncRNA-PCAT-1 in the pathogenesis of ESCC and may consider as a candidate prognostic biomarker for ESCC in an Iranian population.

C1 [Razavi, Maryam; Ghorbian, Saeid] Islamic Azad Univ, Dept Mol Genet, Ahar Branch, Ahar, Iran.

RP Ghorbian, S (corresponding author), Islamic Azad Univ, Dept Mol Genet, Ahar Branch, Ahar, Iran.

EM ghorbian20@yahoo.com

RI ghorbian, saeid/AAN-6554-2021

OI ghorbian, saeid/0000-0003-0780-3159; razavi, maryam/0000-0001-6887-7616

CR Aalijahan H, 2019, EXP MOL PATHOL, V106, P7, DOI 10.1016/j.yexmp.2018.11.010

Bracken AP, 2009, NAT REV CANCER, V9, P773, DOI 10.1038/nrc2736

Faghihi MA, 2009, NAT REV MOL CELL BIO, V10, P637, DOI 10.1038/nrm2738

Jendrzejewski J, 2012, P NATL ACAD SCI USA, V109, P8646, DOI 10.1073/pnas.1205654109

Ling H, 2013, GENOME RES, V23, P1446, DOI 10.1101/gr.152942.112

Liu L, 2015, TUMOR BIOL, V36, P7685, DOI 10.1007/s13277-015-3490-3

Liu Y, 2012, PLOS ONE, V7, DOI 10.1371/journal.pone.0035145

Lu KH, 2013, BMC CANCER, V13, DOI 10.1186/1471-2407-13-461

Ponting CP, 2009, CELL, V136, P629, DOI 10.1016/j.cell.2009.02.006

Prensner JR, 2013, NAT GENET, V45, P1392, DOI 10.1038/ng.2771

Ren YL, 2017, J BIOL CHEM, V292, P17939, DOI 10.1074/jbc.M116.773978

Sadeghpour S, 2019, MOL BIOL REP, V46, P991, DOI 10.1007/s11033-018-4556-2

Schmitt AM, 2016, CANCER CELL, V29, P452, DOI 10.1016/j.ccell.2016.03.010

Shams F, 2019, J CELL BIOCHEM, V120, P11471, DOI 10.1002/jcb.28425

Shi WH, 2015, TUMOR BIOL, V36, P2501, DOI 10.1007/s13277-014-2863-3

St Laurent G, 2012, BMC GENOMICS, V13, DOI 10.1186/1471-2164-13-504

Struhl K, 2007, NAT STRUCT MOL BIOL, V14, P103, DOI 10.1038/nsmb0207-103

Tseng YY, 2014, NATURE, V512, P82, DOI 10.1038/nature13311

Volders PJ, 2013, NUCLEIC ACIDS RES, V41, pD246, DOI 10.1093/nar/gks915

Wang KC, 2011, MOL CELL, V43, P904, DOI 10.1016/j.molcel.2011.08.018

Wapinski O, 2011, TRENDS CELL BIOL, V21, P354, DOI 10.1016/j.tcb.2011.04.001

Whitehead J, 2009, BBA-GEN SUBJECTS, V1790, P936, DOI 10.1016/j.bbagen.2008.10.007

Yan TH, 2015, INT J CLIN EXP PATHO, V8, P4126

Zhang DY, 2017, BIOMED PHARMACOTHER, V95, P1187, DOI 10.1016/j.biopha.2017.09.045

Zhao B, 2015, INT J CLIN EXP MED, V8, P18482

Zhen Q, 2018, CELL BIOCHEM FUNCT, V36, P27, DOI 10.1002/cbf.3314

NR 26

TC 9

Z9 9

U1 0

U2 1

PU EXCLI JOURNAL MANAGING OFFICE

PI DORTMUND

PA LEIBNIZ RESEARCH CENTRE WORKING ENVIRONMENT & HUMAN FACTORS EXCLI

JOURNAL, ARDEYSTR 67, DORTMUND, D-44139, GERMANY

SN 1611-2156

J9 EXCLI J

JI EXCLI J.

PY 2019

VL 18

BP 422

EP 428

DI 10.17179/excli2018-1847

PG 7

WC Biology

WE Science Citation Index Expanded (SCI-EXPANDED)

SC Life Sciences & Biomedicine - Other Topics

GA IF0WI

UT WOS:000472798600001

PM 31338011

DA 2022-05-19

ER

PT J

AU Ma, W

Zhang, CQ

Li, HL

Gu, J

Miao, GY

Cai, HY

Wang, JK

Zhang, LJ

Song, YM

Tian, YH

Song, YH

AF Ma, W.

Zhang, C-Q.

Li, H-L.

Gu, J.

Miao, G-Y.

Cai, H-Y.

Wang, J-K.

Zhang, L-J.

Song, Y-M

Tian, Y-H.

Song, Y-H.

TI LncRNA FER1L4 suppressed cancer cell growth and invasion in esophageal

squamous cell carcinoma

SO EUROPEAN REVIEW FOR MEDICAL AND PHARMACOLOGICAL SCIENCES

LA English

DT Article

DE IncRNA FER1L4; Esophageal squamous cell carcinoma; Proliferation;

Invasion

ID LONG NONCODING RNA; EXPRESSION; PROLIFERATION; MIR-106A-5P

AB OBJECTIVE: To investigate the regulatory effect of long non-coding ribonucleic acid (IncRNA) FER1L4 on biological behaviors of esophageal squamous cell carcinoma (ESCC) cells, such as proliferation and invasion.

PATIENTS AND METHODS: The expressions of FER1L4 were detected in 42 pairs of ESCC tissues and corresponding para-carcinoma tissues and 5 kinds of ESCC cell lines via quantitative reverse transcription-polymerase chain reaction (qRT-PCR). Polyethyleneimine (PEI) and liposomes were used for FER1L4 expression or interference elimination assays, respectively. The proliferation and invasion of ESCC cells were detected via MTT (3-(4,5-dimethylthiazol-2-yl)-2,5-diphenyl tetrazolium bromide), apoptosis assay, cell cycle assay, and transwell chamber.

RESULTS: Results of qRT-PCR showed that, compared with that in normal tissues, FER1L4 was lowly expressed in ESCC tissues. Overexpression of FER1L4 could inhibit cell proliferation and invasion, promote apoptosis and increase the cell cycle distribution in G0/G1 phase. Knockout of FER1L4 could promote the proliferation and invasion of ESCC cells, inhibit apoptosis and decrease the cell cycle distribution in G0/G1 phase.

CONCLUSIONS: FER1L4 is involved in the occurrence and development of ESCC and plays a key role as a tumor suppressor gene in ESCC.

C1 [Ma, W.; Miao, G-Y.; Cai, H-Y.; Wang, J-K.; Zhang, L-J.] Gansu Prov Hosp, Dept Radiotherapy, Lanzhou, Gansu, Peoples R China.

[Zhang, C-Q.] Gansu Prov Hosp, Dept Radiol, Lanzhou, Gansu, Peoples R China.

[Li, H-L.; Song, Y-M; Tian, Y-H.; Song, Y-H.] Gansu Univ Chinese Med, Sch Clin Med, Basic Teaching & Res Off Clin Examinat, Lanzhou, Gansu, Peoples R China.

[Gu, J.] Gansu Univ Chinese Med, Sch Clin Med, Dept Physiol, Lanzhou, Gansu, Peoples R China.

RP Zhang, CQ (corresponding author), Gansu Prov Hosp, Dept Radiol, Lanzhou, Gansu, Peoples R China.

EM 13893212070@126.com

OI ma, wen/0000-0001-6873-5984

FU Foundation of the Fundamental Scientific Research Funds for Colleges and

Universities of Gansu Province [[2014]63-15]; NSFC (Natural Science

Foundation of China) Regional Science Funds Project [81560093]; Gansu

Provincial Science and Technology Department [17JR5RA037]

FX This study was supported by the Foundation of the Fundamental Scientific

Research Funds for Colleges and Universities of Gansu Province (No.

[2014]63-15); NSFC (Natural Science Foundation of China) Regional

Science Funds Project (No. 81560093) and Gansu Provincial Science and

Technology Department (No.17JR5RA037).

CR Abnet CC, 2018, GASTROENTEROLOGY, V154, P360, DOI 10.1053/j.gastro.2017.08.023

Bancewicz J, 2002, LANCET, V359, P1727

Enzinger PC, 2003, NEW ENGL J MED, V349, P2241, DOI 10.1056/NEJMra035010

Gupta RA, 2010, NATURE, V464, P1071, DOI 10.1038/nature08975

Jemal A, 2011, CA-CANCER J CLIN, V61, P69, DOI [10.3322/caac.20107, 10.3322/caac.20115]

Khaitan D, 2011, CANCER RES, V71, P3852, DOI 10.1158/0008-5472.CAN-10-4460

Khalil AM, 2009, P NATL ACAD SCI USA, V106, P11667, DOI 10.1073/pnas.0904715106

Lai XJ, 2018, EUR REV MED PHARMACO, V22, P322, DOI 10.26355/eurrev_201801_14175

Liu Z, 2014, TUMOR BIOL, V35, P9613, DOI 10.1007/s13277-014-2259-4

Mercer TR, 2009, NAT REV GENET, V10, P155, DOI 10.1038/nrg2521

Ponting CP, 2009, CELL, V136, P629, DOI 10.1016/j.cell.2009.02.006

Qiao Q, 2016, BIOCHEM BIOPH RES CO, V478, P507, DOI 10.1016/j.bbrc.2016.06.160

Tsai MC, 2010, SCIENCE, V329, P689, DOI 10.1126/science.1192002

Wang JY, 2010, NUCLEIC ACIDS RES, V38, P5366, DOI 10.1093/nar/gkq285

Wapinski O, 2011, TRENDS CELL BIOL, V21, P354, DOI 10.1016/j.tcb.2011.04.001

Wu J, 2017, CANCER BIOMARK, V20, P55, DOI 10.3233/CBM-170090

Yue B, 2015, CANCER SCI, V106, P1323, DOI 10.1111/cas.12759

NR 17

TC 30

Z9 31

U1 1

U2 4

PU VERDUCI PUBLISHER

PI ROME

PA VIA GREGORIO VII, ROME, 186-00165, ITALY

SN 1128-3602

J9 EUR REV MED PHARMACO

JI Eur. Rev. Med. Pharmacol. Sci.

PD MAY

PY 2018

VL 22

IS 9

BP 2638

EP 2645

PG 8

WC Pharmacology & Pharmacy

WE Science Citation Index Expanded (SCI-EXPANDED)

SC Pharmacology & Pharmacy

GA GG0NS

UT WOS:000432376300017

PM 29771417

DA 2022-05-19

ER

PT J

AU Hong, YC

Wang, Z

Peng, B

Xia, LG

Lin, LW

Xu, ZL

AF Hong, Ying-Cai

Wang, Zheng

Peng, Bin

Xia, Li-Gang

Lin, Lie-Wen

Xu, Zheng-Lei

TI BAG2 overexpression correlates with growth and poor prognosis of

esophageal squamous cell carcinoma

SO OPEN LIFE SCIENCES

LA English

DT Article

DE Esophageal squamous cell carcinoma; BAG2; Proliferation; Migration;

Invasion; Overall survival

ID CANCER STATISTICS; COCHAPERONE BAG2; METASTASIS; INHIBITOR; TAU

AB Previous studies have suggested that Bcl2-associated athanogene 2 (BAG2) serves as a crucial regulator for tumorigenesis in multiple tumors. However, little is known about the effect of BAG2 on esophageal squamous cell carcinoma (ESCC). This study focused on investigating whether BAG2 functions as a cancer-promoting gene in ESCC. In this work, gene expression data and clinical information from the NCBI Gene Expression Omnibus (GEO), Oncomine and The Cancer Genome Atlas (TCGA) were collected and analyzed. Expression of BAG2 in ESCC was determined using quantitative reverse transcription polymerase chain reaction (qRT-PCR). BAG2 was knocked down using small interference RNA (si-RNA) approach. Cell proliferation, migration and invasion were assessed by Cell Counting Kit-8 (CCK-8) and transwell assays. Molecular mechanism was detected by western blotting assay. The expression of BAG2 both in ESCC tissues and cells was upregulated and overexpression was associated with worsened prognosis. BAG2 silencing inhibited ESCC cell proliferation, migration and invasion, which was regulated by the phosphatidylinositol-3-kinase (PI3K)/protein kinase B (AKT) signaling pathway. These results reveal contributions of BAG2 as a predictor and potential therapeutic target in ESCC.

C1 [Xu, Zheng-Lei] Jinan Univ, Clin Med Coll 2, Shenzhen Peoples Hosp, Dept Gastroenterol, Shenzhen 518020, Guangdong, Peoples R China.

[Hong, Ying-Cai; Wang, Zheng; Peng, Bin] Jinan Univ, Clin Med Coll 2, Shenzhen Peoples Hosp, Dept Thorac Surg, Shenzhen 518020, Guangdong, Peoples R China.

[Xia, Li-Gang; Lin, Lie-Wen] Jinan Univ, Clin Med Coll 2, Shenzhen Peoples Hosp, Dept Gastrointestinal Surg, Shenzhen 518020, Guangdong, Peoples R China.

RP Xu, ZL (corresponding author), Jinan Univ, Clin Med Coll 2, Shenzhen Peoples Hosp, Dept Gastroenterol, Shenzhen 518020, Guangdong, Peoples R China.

EM sumsintry0755@163.com

RI Xu, Zhenglei/AAP-4563-2020

CR Andersen MH, 2005, LEUKEMIA, V19, P484, DOI 10.1038/sj.leu.2403621

Arndt V, 2005, MOL BIOL CELL, V16, P5891, DOI 10.1091/mbc.E05-07-0660

Asperger A, 2011, CANCER INVEST, V29, P272, DOI 10.3109/07357907.2010.550666

Bao Y, 2018, STROMAL EXPRESSION J

Carrettiero DC, 2009, J NEUROSCI, V29, P2151, DOI 10.1523/JNEUROSCI.4660-08.2009

Castro C, 2014, ANN ONCOL, V25, P283, DOI 10.1093/annonc/mdt486

Che XQ, 2013, BIOCHEM BIOPH RES CO, V441, P488, DOI 10.1016/j.bbrc.2013.10.086

Chen J, 2015, ARCH BIOCHEM BIOPHYS, V575, P38, DOI 10.1016/j.abb.2014.11.008

Chen WQ, 2016, CA-CANCER J CLIN, V66, P115, DOI 10.3322/caac.21338

Fang SS, 2013, ACTA CRYSTALLOGR D, V69, P934, DOI 10.1107/S0907444913003624

Huang Y, 2013, ANTI-CANCER DRUG, V24, P889, DOI 10.1097/CAD.0b013e328363c64e

Jian Y, 2016, BIOMED REP, V4, P723, DOI 10.3892/br.2016.650

Jin H, 2014, J PROTEOME RES, V13, P4091, DOI 10.1021/pr500483z

Klopfleisch R, 2013, J COMP PATHOL, V148, P197, DOI 10.1016/j.jcpa.2012.06.004

Li SD, 2010, PLOS ONE, V5, DOI 10.1371/journal.pone.0010486

Liu M, 2016, CANCER LETT, V377, P97, DOI 10.1016/j.canlet.2016.04.034

Patil SP, 2013, NEUROSCI LETT, V554, P121, DOI 10.1016/j.neulet.2013.09.008

Piltti J, 2018, J CELL BIOCHEM, V119, P2320, DOI 10.1002/jcb.26394

Qu DB, 2015, J BIOL CHEM, V290, P30441, DOI 10.1074/jbc.M115.677815

Ray RB, 2015, PLOS ONE, V10

Torre LA, 2015, CA-CANCER J CLIN, V65, P87, DOI 10.3322/caac.21262

Wu N, 2018, EXPRESSION PROGNOSTI, V17

Xi RX, 2016, ONCOTARGET, V7, P57050, DOI 10.18632/oncotarget.10959

Yang KM, 2017, CELL REP, V21, P2952, DOI 10.1016/j.celrep.2017.11.026

Yue XT, 2015, ELIFE, V4, DOI 10.7554/eLife.08401

Zhu DY, 2018, ONCOL LETT, V15, P4775, DOI 10.3892/ol.2018.7914

NR 26

TC 2

Z9 2

U1 0

U2 1

PU DE GRUYTER POLAND SP ZOO

PI WARSAW

PA BOGUMILA ZUGA 32A STR., 01-811 WARSAW, POLAND

SN 2391-5412

J9 OPEN LIFE SCI

JI Open Life Sci.

PY 2018

VL 13

IS 1

BP 582

EP 588

DI 10.1515/biol-2018-0069

PG 7

WC Biology

WE Science Citation Index Expanded (SCI-EXPANDED)

SC Life Sciences & Biomedicine - Other Topics

GA HK2GV

UT WOS:000457728800005

PM 33817129

OA Green Published, gold

DA 2022-05-19

ER

PT J

AU Chen, GY

Xu, R

Yue, B

Mei, X

Li, P

Zhou, XG

Huang, SF

Gong, LP

Zhang, ST

AF Chen, Guangyong

Xu, Rui

Yue, Bing

Mei, Xue

Li, Peng

Zhou, Xiaoge

Huang, Shoufang

Gong, Liping

Zhang, Shutian

TI The expression of podoplanin protein is a diagnostic marker to

distinguish the early Infiltration of esophageal squamous cell carcinoma

SO ONCOTARGET

LA English

DT Article

DE podoplanin; esophageal squamous cell carcinoma

ID TUMOR INVASION; ORAL-CANCER; RISK

AB The esophageal squamous cell carcinoma (ESCC) is usually develped from lowgrade intraepithelial neoplasia (LGIEN) and high-grade intraepithelial neoplasia (HGIEN) to infiltrative squamous cell carcinoma. Till now, it remains hard to screen for infiltration at earlier stages, especially the differentiation between HGEIN and early infiltrative carcinoma. The purpose of this study is to determine a role of podoplanin in differentiating between HGEIN and early infiltrative squamous cell carcinoma. Totally 133 patients pathologically diagnosed with early ESCC and/or precancerous lesions were enrolled. The EnVision two-step IHC staining technique was applied using the monoclonal mouse anti-human Podoplanin antibody (clone number: D2-40). The expressions of PDPN protein on the basal layer of squamous epithelium lesions could be divided into three different patterns: complete type, incomplete (non-continuous) type, or missing type. A diagnosis of HGEIN can be made if the basal layer showed non-continuous or complete expression of PDPN and a diagnosis of early infiltration can be made if the expression of PDPN is completely missing. Our study confirmed that PDPN was a potential biomarker to identify the presence of early infiltrative squamous cell carcinoma.

C1 [Chen, Guangyong; Xu, Rui; Yue, Bing; Mei, Xue; Zhou, Xiaoge; Huang, Shoufang] Capital Med Univ, Beijing Friendship Hosp, Natl Clin Res Ctr Digest Dis, Dept Pathol, Beijing 10050, Peoples R China.

[Li, Peng; Zhang, Shutian] Capital Med Univ, Beijing Friendship Hosp, Natl Clin Res Ctr Digest Dis, Dept Gastroenterol & Hepatol, Beijing 10050, Peoples R China.

[Gong, Liping] Capital Med Univ, Basic Med Coll, Dept Pathol, Beijing 100069, Peoples R China.

RP Zhang, ST (corresponding author), Capital Med Univ, Beijing Friendship Hosp, Natl Clin Res Ctr Digest Dis, Dept Gastroenterol & Hepatol, Beijing 10050, Peoples R China.

EM zhangst@ccmu.Edu.cn

CR Bosman F.T., 2010, WHO CLASSIFICATION T, V4th Edn

Breiteneder-Geleff S, 1999, AM J PATHOL, V154, P385, DOI 10.1016/S0002-9440(10)65285-6

Chuang WY, 2009, HISTOL HISTOPATHOL, V24, P1021, DOI 10.14670/HH-24.1021

Dumoff KL, 2006, MODERN PATHOL, V19, P708, DOI 10.1038/modpathol.3800580

Imamura M, 2000, SUPERFICIAL ESOPHAGE

Kawaguchi H, 2008, J CLIN ONCOL, V26, P354, DOI 10.1200/JCO.2007.13.4072

Martin-Villar E, 2005, INT J CANCER, V113, P899, DOI 10.1002/ijc.20656

Patil Ashok, 2015, SCI CAIRO, V2015, P354

Schacht V, 2005, AM J PATHOL, V166, P913, DOI 10.1016/S0002-9440(10)62311-5

Schlemper RJ, 2000, CANCER, V88, P996, DOI 10.1002/(SICI)1097-0142(20000301)88:5<996::AID-CNCR8>3.0.CO;2-Q

Takubo Kaiyo, 2008, PATHOLOGY ESOPHAGUS

Wang GQ, 2005, GUT, V54, P187, DOI 10.1136/gut.2004.046631

Wicki A, 2006, CANCER CELL, V9, P261, DOI 10.1016/j.ccr.2006.03.010

Wicki A, 2007, BRIT J CANCER, V96, P1, DOI 10.1038/sj.bjc.6603518

Yuan P, 2006, CANCER, V107, P563, DOI 10.1002/cncr.22061

NR 15

TC 6

Z9 9

U1 0

U2 2

PU IMPACT JOURNALS LLC

PI ORCHARD PARK

PA 6666 E QUAKER ST, STE 1, ORCHARD PARK, NY 14127 USA

EI 1949-2553

J9 ONCOTARGET

JI Oncotarget

PD MAR 21

PY 2017

VL 8

IS 12

BP 19013

EP 19020

PG 8

WC Oncology; Cell Biology

WE Science Citation Index Expanded (SCI-EXPANDED)

SC Oncology; Cell Biology

GA EO7OE

UT WOS:000396879200035

PM 28086225

OA Green Published, Green Submitted, gold

DA 2022-05-19

ER

PT J

AU Lin, ZW

Gu, J

Liu, RH

Liu, XM

Xu, FK

Zhao, GY

Lu, CL

Ge, D

AF Lin, Zong-wu

Gu, Jie

Liu, Rong-hua

Liu, Xiao-ming

Xu, Feng-kai

Zhao, Guang-yin

Lu, Chun-lai

Ge, Di

TI Genome-wide screening and co-expression network analysis identify

recurrence-specific biomarkers of esophageal squamous cell carcinoma

SO TUMOR BIOLOGY

LA English

DT Article

DE Esophageal squamous cell carcinoma; RNA sequencing; Recurrence;

Metastasis

ID GTPASE-ACTIVATING PROTEIN; RNA-SEQ; BREAST-CANCER; EXPRESSION;

METASTASIS; PROGNOSIS; OVEREXPRESSION; PROGRESSION; PROFILES; LUNG

AB Tumor recurrence and metastasis after surgery are the leading causes of death in patients with esophageal squamous cell carcinoma (ESCC). Next-generation sequencing techniques have improved our understanding of the genetic alterations underlying tumor initiation and progression. To explore recurrence-specific transcriptional profiles, functional properties, and gene co-expression networks in ESCC, samples from recurrence (n = 4) and nonrecurrence (n = 4) groups were analyzed by RNA sequencing. Patients included in the nonrecurrence group had five or more years of survival without any evidence of recurrence or metastasis, while those included in the recurrence group exhibited early recurrence and metastasis and died within 2 years. We identified 533 significantly differentially expressed protein-coding and noncoding genes. Functional enrichment analysis indicated that ESCC recurrence was related to dysregulated cell-cell adherence, microenvironment homeostasis, information processing, and the immune response. Co-expression networks demonstrated differences in the patterns of gene expression and co-expression between the recurrence and nonrecurrence groups. This study provided important insights into ESCC progression and the differentially expressed genes that may represent potential targets for ESCC diagnosis and therapy.

C1 [Lin, Zong-wu; Gu, Jie; Xu, Feng-kai; Zhao, Guang-yin; Lu, Chun-lai; Ge, Di] Fudan Univ, Affiliated Zhongshan Hosp, Dept Thorac Surg, Shanghai 200032, Peoples R China.

[Liu, Rong-hua; Liu, Xiao-ming] Fudan Univ, Shanghai Med Coll, Dept Immunol, Key Lab Mol Med,Minist Educ, Shanghai 200032, Peoples R China.

RP Ge, D (corresponding author), Fudan Univ, Affiliated Zhongshan Hosp, Dept Thorac Surg, Shanghai 200032, Peoples R China.

EM gedi6902@hotmail.com

FU National Natural Science Foundation of China [81302099]

FX This study was supported by The National Natural Science Foundation of

China (no. 81302099).

CR Ashburner M, 2000, NAT GENET, V25, P25, DOI 10.1038/75556

Barabasi AL, 2004, NAT REV GENET, V5, P101, DOI 10.1038/nrg1272

Blanc L, 2012, P NATL ACAD SCI USA, V109, P12099, DOI 10.1073/pnas.1204948109

Buhmeida A, 2014, TUMOR BIOL, V35, P2817, DOI 10.1007/s13277-013-1374-y

Carlson MRJ, 2006, BMC GENOMICS, V7, DOI 10.1186/1471-2164-7-40

Chun HK, 2010, BMB REP, V43, P349, DOI 10.5483/BMBRep.2010.43.5.349

Davies AR, 2014, J SURG ONCOL, V109, P459, DOI 10.1002/jso.23511

Feng S, 2013, CANCER RES, V73, P2551, DOI 10.1158/0008-5472.CAN-12-4108

Fu X, 2009, BMC GENOMICS, V10, DOI 10.1186/1471-2164-10-161

Gu J, 2013, LUNG CANCER, V81, P259, DOI 10.1016/j.lungcan.2013.04.020

Guerrero-Preston R, 2014, ONCOL REP, V32, P505, DOI 10.3892/or.2014.3262

Guijarro MV, 2007, CARCINOGENESIS, V28, P1646, DOI 10.1093/carcin/bgm083

Han QQ, 2014, TUMOR BIOL, V35, P715, DOI 10.1007/s13277-013-1098-z

He DN, 2012, J MOL CELL BIOL, V4, P140, DOI 10.1093/jmcb/mjs011

Horvath S, 2006, P NATL ACAD SCI USA, V103, P17402, DOI 10.1073/pnas.0608396103

Ivliev AE, 2010, CANCER RES, V70, P10060, DOI 10.1158/0008-5472.CAN-10-2465

Kan T, 2001, BIOCHEM BIOPH RES CO, V286, P792, DOI 10.1006/bbrc.2001.5400

Kanehisa M, 2004, NUCLEIC ACIDS RES, V32, pD277, DOI 10.1093/nar/gkh063

Koinuma J, 2012, CANCER SCI, V103, P577, DOI 10.1111/j.1349-7006.2011.02167.x

Lin YS, 2013, J EPIDEMIOL, V23, P233, DOI 10.2188/jea.JE20120162

Low-Marchelli JM, 2013, CANCER RES, V73, P662, DOI 10.1158/0008-5472.CAN-12-0653

Lu CL, 2014, DIS ESOPHAGUS, V27, P294, DOI 10.1111/dote.12100

Mardis ER, 2008, TRENDS GENET, V24, P133, DOI 10.1016/j.tig.2007.12.007

Marioni JC, 2008, GENOME RES, V18, P1509, DOI 10.1101/gr.079558.108

Mortazavi A, 2008, NAT METHODS, V5, P621, DOI 10.1038/nmeth.1226

Murat A, 2008, J CLIN ONCOL, V26, P3015, DOI 10.1200/JCO.2007.15.7164

Nakamura E, 2011, CANCER BIOL THER, V12, P181, DOI 10.4161/cbt.12.3.15964

Nguyen DX, 2009, NAT REV CANCER, V9, P274, DOI 10.1038/nrc2622

Oldham MC, 2008, NAT NEUROSCI, V11, P1271, DOI 10.1038/nn.2207

Pennathur A, 2013, LANCET, V381, P400, DOI 10.1016/S0140-6736(12)60643-6

Prieto C, 2008, PLOS ONE, V3, DOI 10.1371/journal.pone.0003911

Pujana MA, 2007, NAT GENET, V39, P1338, DOI 10.1038/ng.2007.2

Ryu B, 2007, PLOS ONE, V2, DOI 10.1371/journal.pone.0000594

STAHLEBACKDHAL M, 1995, AM J PATHOL, V146, P1073

Talsma K, 2012, ANN SURG ONCOL, V19, P2142, DOI 10.1245/s10434-012-2218-5

Tao YS, 2012, TOHOKU J EXP MED, V226, P301, DOI 10.1620/tjem.226.301

Wang K, 2010, NUCLEIC ACIDS RES, V38, DOI 10.1093/nar/gkq622

Wang Z, 2009, NAT REV GENET, V10, P57, DOI 10.1038/nrg2484

Xu HD, 2014, EXP MOL MED, V46, DOI 10.1038/emm.2014.23

Xu X, 2013, BMC BIOINFORMATICS, V14, DOI 10.1186/1471-2105-14-S9-S1

Yang Q, 2013, MED ONCOL, V30, DOI 10.1007/s12032-013-0689-2

Zhang B, 2005, STAT APPL GENET MOL, V4, DOI 10.2202/1544-6115.1128

Zhu CJ, 2005, MOL BIOL CELL, V16, P3187, DOI 10.1091/mbc.E05-02-0167

Zhu ZJ, 2011, ANN THORAC SURG, V91, P1502, DOI 10.1016/j.athoracsur.2011.01.007

NR 44

TC 9

Z9 9

U1 1

U2 7

PU SAGE PUBLICATIONS LTD

PI LONDON

PA 1 OLIVERS YARD, 55 CITY ROAD, LONDON EC1Y 1SP, ENGLAND

SN 1010-4283

EI 1423-0380

J9 TUMOR BIOL

JI Tumor Biol.

PD NOV

PY 2014

VL 35

IS 11

BP 10959

EP 10968

DI 10.1007/s13277-014-2388-9

PG 10

WC Oncology

WE Science Citation Index Expanded (SCI-EXPANDED)

SC Oncology

GA AU1YJ

UT WOS:000345413100042

PM 25087095

DA 2022-05-19

ER

PT J

AU Takeshita, N

Mori, M

Kano, M

Hoshino, I

Akutsu, Y

Hanari, N

Yoneyama, Y

Ikeda, N

Isozaki, Y

Maruyama, T

Akanuma, N

Miyazawa, Y

Matsubara, H

AF Takeshita, Nobuyoshi

Mori, Mikito

Kano, Masayuki

Hoshino, Isamu

Akutsu, Yasunori

Hanari, Naoyuki

Yoneyama, Yasuo

Ikeda, Norimasa

Isozaki, Yuka

Maruyama, Tetsurou

Akanuma, Naoki

Miyazawa, Yukimasa

Matsubara, Hisahiro

TI miR-203 inhibits the migration and invasion of esophageal squamous cell

carcinoma by regulating LASP1

SO INTERNATIONAL JOURNAL OF ONCOLOGY

LA English

DT Article

DE microRNA; miR-203; esophageal squamous cell carcinoma; LASP1

ID MICRORNA EXPRESSION PROFILES; LYMPH-NODE DISSECTION; REPRESSING

DELTA-NP63; PANCREATIC-CANCER; GENE-EXPRESSION; PROLIFERATION; SURVIVAL;

METASTASIS; SIGNATURE; ONCOGENE

AB The expression of microRNA-203 (miR-203) in esophageal squamous cell carcinoma (ESCC) tissues is remarkably lower than that in non-ESCC tissues. We investigated how miR-203 could influence the development of ESCC cells. Our analyses revealed that miR-203 inhibited the migration and invasion of ESCC cells. Genome-wide gene expression data and target site inhibition assays showed that miR-203 appears to directly regulate LIM and SH3 protein 1 (LASP1). The knockdown of LASP1 resulted in inhibition of the migration and invasion of ESCC cells. Our results suggest that miR-203 and its target LASP1, may be associated with the progression of ESCC. In clinical ESCC specimens, the expression levels of miR-203, which were lower compared to those in normal tissues, were inversely correlated with the mRNA expression levels of LASP1. Moreover, we found that there was a significant correlation between the expression levels of miR-203 and the relapse-free survival. The identification of a cancer network regulated by miR-203 could provide new insights into the potential mechanisms of the progression of ESCC.

C1 [Mori, Mikito] Chiba Univ, Dept Frontier Surg, Grad Sch Med, Chuo Ku, Chiba 2608670, Japan.

RP Mori, M (corresponding author), Chiba Univ, Dept Frontier Surg, Grad Sch Med, Chuo Ku, Inohana 1-8-1, Chiba 2608670, Japan.

EM mikimori@faculty.chiba-u.jp

CR Altorki N, 2002, ANN SURG, V236, P177, DOI 10.1097/00000658-200208000-00005

Ambros V, 2004, NATURE, V431, P350, DOI 10.1038/nature02871

Castilla MA, 2011, J PATHOL, V223, P72, DOI 10.1002/path.2802

Bancewicz J, 2002, LANCET, V359, P1727

Bartel DP, 2004, CELL, V116, P281, DOI 10.1016/S0092-8674(04)00045-5

Blamey RW, 2010, EUR J CANCER, V46, P56, DOI 10.1016/j.ejca.2009.09.009

Bo JJ, 2011, FEBS J, V278, P786, DOI 10.1111/j.1742-4658.2010.07997.x

Bueno MJ, 2008, CANCER CELL, V13, P496, DOI 10.1016/j.ccr.2008.04.018

Calin GA, 2005, NEW ENGL J MED, V352, P1667, DOI 10.1056/NEJMoa042280

Chen HY, 2011, MED ONCOL

Chen JQ, 2012, INT J RADIAT ONCOL, V82, P475, DOI 10.1016/j.ijrobp.2010.08.037

Chew CS, 2002, J CELL SCI, V115, P4787, DOI 10.1242/jcs.00174

Chiyomaru T, 2012, UROL ONCOL-SEMIN ORI, V30, P434, DOI 10.1016/j.urolonc.2010.05.008

Choi SY, 2011, CLIN EXP MED, V11, P219, DOI 10.1007/s10238-010-0130-5

Feber A, 2008, J THORAC CARDIOV SUR, V135, P255, DOI 10.1016/j.jtcvs.2007.08.055

Furuta M, 2010, CARCINOGENESIS, V31, P766, DOI 10.1093/carcin/bgp250

Guo Y, 2008, CANCER RES, V68, P26, DOI 10.1158/0008-5472.CAN-06-4418

He L, 2005, NATURE, V435, P828, DOI 10.1038/nature03552

He L, 2004, NAT REV GENET, V5, P522, DOI 10.1038/nrg1379

Hiyoshi Y, 2009, CLIN CANCER RES, V15, P1915, DOI 10.1158/1078-0432.CCR-08-2545

Iorio MV, 2005, CANCER RES, V65, P7065, DOI 10.1158/0008-5472.CAN-05-1783

Jiang YQ, 2010, ANN THORAC SURG, V90, P908, DOI 10.1016/j.athoracsur.2010.05.060

Kano M, 2010, INT J CANCER, V127, P2804, DOI 10.1002/ijc.25284

Lena AM, 2008, CELL DEATH DIFFER, V15, P1187, DOI 10.1038/cdd.2008.69

Lin YH, 2004, J CELL BIOL, V165, P421, DOI 10.1083/jcb.200311045

Lu J, 2005, NATURE, V435, P834, DOI 10.1038/nature03702

Morris EJA, 2011, GUT, V60, P1087, DOI 10.1136/gut.2010.229575

NISHIHIRA T, 1979, GANN, V70, P575

Sadanandam A, 2010, INT J CANCER, V127, P1373, DOI 10.1002/ijc.25166

Saini S, 2011, CLIN CANCER RES, V17, P5287, DOI 10.1158/1078-0432.CCR-10-2619

Schickel R, 2008, ONCOGENE, V27, P5959, DOI 10.1038/onc.2008.274

Song K, 2011, BIOCHEM J, V436, P271, DOI 10.1042/BJ20110297

Takahashi K, 1990, JPN J ORAL MAXILLOFA, V36, P67

TOMASETTO C, 1995, GENOMICS, V28, P367, DOI 10.1006/geno.1995.1163

Viticchie G, 2011, CELL CYCLE, V10, P1121, DOI 10.4161/cc.10.7.15180

Volinia S, 2006, P NATL ACAD SCI USA, V103, P2257, DOI 10.1073/pnas.0510565103

Yanaihara N, 2006, CANCER CELL, V9, P189, DOI 10.1016/j.ccr.2006.01.025

Yi R, 2008, NATURE, V452, P225, DOI 10.1038/nature06642

Yuan Y, 2011, BMC CANCER, V11, DOI 10.1186/1471-2407-11-57

Zhao LA, 2010, GUT, V59, P1226, DOI 10.1136/gut.2009.202739

NR 40

TC 62

Z9 64

U1 0

U2 10

PU SPANDIDOS PUBL LTD

PI ATHENS

PA POB 18179, ATHENS, 116 10, GREECE

SN 1019-6439

EI 1791-2423

J9 INT J ONCOL

JI Int. J. Oncol.

PD NOV

PY 2012

VL 41

IS 5

BP 1653

EP 1661

DI 10.3892/ijo.2012.1614

PG 9

WC Oncology

WE Science Citation Index Expanded (SCI-EXPANDED)

SC Oncology

GA 024MU

UT WOS:000310114100013

PM 22940702

OA Green Submitted, hybrid

DA 2022-05-19

ER

PT J

AU Terasaki, K

Gen, Y

Iwai, N

Soda, T

Kitaichi, T

Dohi, O

Taketani, H

Seko, Y

Umemura, A

Nishikawa, T

Yamaguchi, K

Moriguchi, M

Konishi, H

Naito, Y

Itoh, Y

Yasui, K

AF Terasaki, Kei

Gen, Yasuyuki

Iwai, Naoto

Soda, Tomohiro

Kitaichi, Tomoko

Dohi, Osamu

Taketani, Hiroyoshi

Seko, Yuya

Umemura, Atsushi

Nishikawa, Taichiro

Yamaguchi, Kanji

Moriguchi, Michihisa

Konishi, Hideyuki

Naito, Yuji

Itoh, Yoshito

Yasui, Kohichiroh

TI SOX2 enhances cell survival and induces resistance to apoptosis under

serum starvation conditions through the AKT/GSK-3 beta signaling pathway

in esophageal squamous cell carcinoma

SO ONCOLOGY LETTERS

LA English

DT Article

DE SOX2; AKT; survival; serum starvation; esophageal squamous cell

carcinoma

AB The human SOX2 gene was recently identified as a novel major oncogene, recurrently amplified and overexpressed in esophageal squamous cell carcinoma (ESCC). However, the role and molecular mechanism of SOX2 in the carcinogenesis of ESCC remain to be elucidated. The present study investigated the effect of SOX2 on ESCC cell survival and resistance to apoptosis under serum starvation conditions. An adenoviral vector-mediated expression system and RNA interference were used to study the effect of SOX2. The present results revealed that SOX2 promoted ESCC cell survival and enhanced resistance to apoptosis under serum starvation conditions, but not in culture conditions with serum. Mechanistically, SOX2 increased the expression levels of phosphorylated AKT and glycogen synthase kinase-3 beta (GSK-3 beta), a downstream factor of AKT, under serum starvation conditions, leading to the promotion of ESCC cell survival. Additionally, SOX2 activated AKT through the PTEN/PI3K/phosphoinositide-dependent protein kinase 1 and mammalian target of rapamycin complex 2 signaling pathways. Therefore, SOX2 may facilitate the survival of ESCC cells under poor nutrient conditions by activating the AKT/GSK-3 beta signaling pathway.

C1 [Terasaki, Kei; Gen, Yasuyuki; Iwai, Naoto; Soda, Tomohiro; Kitaichi, Tomoko; Dohi, Osamu; Taketani, Hiroyoshi; Seko, Yuya; Umemura, Atsushi; Nishikawa, Taichiro; Yamaguchi, Kanji; Moriguchi, Michihisa; Konishi, Hideyuki; Naito, Yuji; Itoh, Yoshito] Kyoto Prefectural Univ Med, Dept Mol Gastroenterol & Hepatol, Kamigyo Ku, Kyoto 6028566, Japan.

[Yasui, Kohichiroh] Bukkyo Univ, Sch Hlth Sci, Nakagyo Ku, 7 Higashitoganoocho, Kyoto 6048418, Japan.

RP Yasui, K (corresponding author), Bukkyo Univ, Sch Hlth Sci, Nakagyo Ku, 7 Higashitoganoocho, Kyoto 6048418, Japan.

EM k-yasui@bukkyo-u.ac.jp

OI Umemura, Atsushi/0000-0002-7433-8501

CR Bass AJ, 2009, NAT GENET, V41, P1238, DOI 10.1038/ng.465

Chresta CM, 2010, CANCER RES, V70, P288, DOI 10.1158/0008-5472.CAN-09-1751

Engelman JA, 2006, NAT REV GENET, V7, P606, DOI 10.1038/nrg1879

Ferlay J, 2019, INT J CANCER, V144, P1941, DOI 10.1002/ijc.31937

Gen Y, 2013, CANCER SCI, V104, P810, DOI 10.1111/cas.12155

Gen Y, 2010, CANCER GENET CYTOGEN, V202, P82, DOI 10.1016/j.cancergencyto.2010.01.023

Hussenet T, 2010, PLOS ONE, V5, DOI 10.1371/journal.pone.0008960

Jeong HC, 2017, STEM CELLS, V35, P2037, DOI 10.1002/stem.2642

Maier S, 2011, HUM PATHOL, V42, P1078, DOI 10.1016/j.humpath.2010.11.010

Masui S, 2007, NAT CELL BIOL, V9, P625, DOI 10.1038/ncb1589

Maurer U, 2014, J CELL SCI, V127, P1369, DOI 10.1242/jcs.138057

Pevny LH, 1997, CURR OPIN GENET DEV, V7, P338, DOI 10.1016/S0959-437X(97)80147-5

Que J, 2007, DEVELOPMENT, V134, P2521, DOI 10.1242/dev.003855

Rustgi AK, 2014, NEW ENGL J MED, V371, P2499, DOI 10.1056/NEJMra1314530

Schaefer T, 2015, ONCOTARGET, V6, P43540, DOI 10.18632/oncotarget.6183

Singh S, 2012, MOL CANCER, V11, DOI 10.1186/1476-4598-11-73

Vivanco I, 2002, NAT REV CANCER, V2, P489, DOI 10.1038/nrc839

Walz A, 2017, CLIN CANCER RES, V23, P1891, DOI 10.1158/1078-0432.CCR-15-2240

Yang G, 2015, CELL REP, V12, P937, DOI 10.1016/j.celrep.2015.07.016

Zen K, 2009, GENE CHROMOSOME CANC, V48, P109, DOI 10.1002/gcc.20624

NR 20

TC 0

Z9 0

U1 1

U2 2

PU SPANDIDOS PUBL LTD

PI ATHENS

PA POB 18179, ATHENS, 116 10, GREECE

SN 1792-1074

EI 1792-1082

J9 ONCOL LETT

JI Oncol. Lett.

PD APR

PY 2021

VL 21

IS 4

AR 269

DI 10.3892/ol.2021.12530

PG 7

WC Oncology

WE Science Citation Index Expanded (SCI-EXPANDED)

SC Oncology

GA QI6TL

UT WOS:000619110400001

PM 33717266

OA Green Published, gold

DA 2022-05-19

ER

PT J

AU Park, SR

Yoon, DH

Kim, JH

Kim, YH

Kim, HR

Lee, HJ

Jung, HY

Lee, GH

Song, HJ

Kim, DH

Choi, KD

Lee, JH

Ahn, JY

Ryu, JS

Cho, KJ

Kim, SB

AF Park, Sook Ryun

Yoon, Dok Hyun

Kim, Jong Hoon

Kim, Yong-Hee

Kim, Hyeong Ryul

Lee, Hyun Joo

Jung, Hwoon-Yong

Lee, Gin-Hyug

Song, Ho June

Kim, Do Hoon

Choi, Kee Don

Lee, Jeong Hoon

Ahn, Ji Yong

Ryu, Jin-Sook

Cho, Kyung-Ja

Kim, Sung-Bae

TI A Randomized Phase III Trial on the Role of Esophagectomy in Complete

Responders to Preoperative Chemoradiotherapy for Esophageal Squamous

Cell Carcinoma (ESOPRESSO)

SO ANTICANCER RESEARCH

LA English

DT Article

DE Esophageal squamous cell carcinoma; chemoradiotherapy; induction

chemotherapy; clinical complete response; surgery; observation

ID SURGERY; CANCER; CHEMORADIATION; THERAPY; CHEMOTHERAPY

AB Background/Aim: We investigated the role of esophagectomy after clinical complete response (cCR) to chemoradiotherapy for esophageal squamous cell carcinoma (ESCC). Patients and Methods: Patients with resectable cT3-T4a/anyN/M0 or anyT/N+/M0 thoracic ESCC received two cycles of induction chemotherapy and then chemoradiotherapy (50.4 Gy/28 fractions). Patients with cCR were randomized to surgery or observation. Results: Among 86 patients, 38 (44.2%) achieved cCR after chemoradiotherapy; 37 were randomized to surgery (n=19) or observation (n=18). Although there were trends of better disease-free survival (DFS) toward the surgery arm in the intent-to-treat analysis (2-year DFS, 66.7% vs. 42.7%; p=0.262) or as-treated analysis (66.7% vs. 50.2%; p=0.273), overall survival was not different between the two arms in the intent-to-treat (HR=1.48; p=0.560) or as-treated analysis (HR=1.09; p=0.903). Among the 11 patients having recurrence during observation, 8 underwent surgery (n=7) or endoscopic dissection (n=1). Conclusion: Close observation with salvage surgery might be a reasonable option in resectable ESCC patients achieving cCR after chemoradiation.

C1 [Park, Sook Ryun; Yoon, Dok Hyun; Kim, Sung-Bae] Univ Ulsan, Coll Med, Asan Med Ctr, Dept Oncol, 88 Olymp Ro,43 Gil, Seoul 05505, South Korea.

[Kim, Jong Hoon] Univ Ulsan, Coll Med, Asan Med Ctr, Dept Radiat Oncol, Seoul, South Korea.

[Kim, Yong-Hee; Kim, Hyeong Ryul] Univ Ulsan, Coll Med, Asan Med Ctr, Dept Thorac Surg, Seoul, South Korea.

[Lee, Hyun Joo] Univ Ulsan, Coll Med, Asan Med Ctr, Dept Radiol, Seoul, South Korea.

[Jung, Hwoon-Yong; Lee, Gin-Hyug; Song, Ho June; Kim, Do Hoon; Choi, Kee Don; Lee, Jeong Hoon; Ahn, Ji Yong] Univ Ulsan, Coll Med, Asan Med Ctr, Dept Gastroenterol, Seoul, South Korea.

[Ryu, Jin-Sook] Univ Ulsan, Coll Med, Asan Med Ctr, Dept Nucl Med, Seoul, South Korea.

[Cho, Kyung-Ja] Univ Ulsan, Coll Med, Asan Med Ctr, Dept Pathol, Seoul, South Korea.

RP Kim, SB (corresponding author), Univ Ulsan, Coll Med, Asan Med Ctr, Dept Oncol, 88 Olymp Ro,43 Gil, Seoul 05505, South Korea.

EM sbkim3@amc.seoul.kr

RI Ahn, Ji Yong/AGO-1695-2022

CR Adenis A, 2013, BMC CANCER, V13, DOI 10.1186/1471-2407-13-413

Best LMJ, 2016, COCHRANE DB SYST REV, DOI 10.1002/14651858.CD011498.pub2

Bosset JF, 1997, NEW ENGL J MED, V337, P161, DOI 10.1056/NEJM199707173370304

Bouche O, 2007, J CLIN ONCOL, V25, DOI 10.1200/JCO.2005.04.7118

Castoro C, 2013, J GASTROINTEST SURG, V17, P1375, DOI 10.1007/s11605-013-2269-3

Eisenhauer EA, 2009, EUR J CANCER, V45, P228, DOI 10.1016/j.ejca.2008.10.026

Eyck B, 2020, ANN SURG, V271, P245, DOI 10.1097/SLA.0000000000003397

Ishihara R, 2010, INT J RADIAT ONCOL, V76, P123, DOI 10.1016/j.ijrobp.2009.01.038

Kelsen DP, 1998, NEW ENGL J MED, V339, P1979, DOI 10.1056/NEJM199812313392704

Noordman BJ, 2018, LANCET ONCOL, V19, P965, DOI 10.1016/S1470-2045(18)30201-8

Noordman BJ, 2018, BMC CANCER, V18, DOI 10.1186/s12885-018-4034-1

Piessen G, 2013, ANN SURG, V258, P793, DOI 10.1097/SLA.0000000000000228

Sjoquist KM, 2011, LANCET ONCOL, V12, P681, DOI 10.1016/S1470-2045(11)70142-5

Stahl M, 2005, J CLIN ONCOL, V23, P2310, DOI 10.1200/JCO.2005.00.034

Tepper J, 2008, J CLIN ONCOL, V26, P1086, DOI 10.1200/JCO.2007.12.9593

Torre LA, 2015, CA-CANCER J CLIN, V65, P87, DOI 10.3322/caac.21262

van den Ende T, 2019, J CLIN ONCOL, V37, DOI 10.1200/JCO.2019.37.15_suppl.4045

van der Sluis PC, 2019, ANN SURG, V269, P621, DOI 10.1097/SLA.0000000000003031

van Hagen P, 2012, NEW ENGL J MED, V366, P2074, DOI 10.1056/NEJMoa1112088

Van Meerten E, 2009, J SURG ONCOL, V100, P32, DOI 10.1002/jso.21295

Walsh TN, 1996, NEW ENGL J MED, V335, P462, DOI 10.1056/NEJM199608153350702

WANG HY, 2019, J CLIN ONCOL, V35, DOI DOI 10.1200/JCO.2019.37.15_SUPPL.4027

Wang J, 2018, THORAC CANCER, V9, P1638, DOI 10.1111/1759-7714.12874

Young H, 1999, EUR J CANCER, V35, P1773, DOI 10.1016/S0959-8049(99)00229-4

NR 24

TC 6

Z9 7

U1 0

U2 0

PU INT INST ANTICANCER RESEARCH

PI ATHENS

PA EDITORIAL OFFICE 1ST KM KAPANDRITIOU-KALAMOU RD KAPANDRITI, PO BOX 22,

ATHENS 19014, GREECE

SN 0250-7005

EI 1791-7530

J9 ANTICANCER RES

JI Anticancer Res.

PD SEP

PY 2019

VL 39

IS 9

BP 5123

EP 5133

DI 10.21873/anticanres.13707

PG 11

WC Oncology

WE Science Citation Index Expanded (SCI-EXPANDED)

SC Oncology

GA IY5TX

UT WOS:000486457600070

PM 31519624

OA Bronze

DA 2022-05-19

ER

PT J

AU Chai, AWY

Cheung, AKL

Dai, W

Ko, JMY

Ip, JCY

Chan, KW

Kwong, DLW

Ng, WT

Lee, AWM

Ngan, RKC

Yau, CC

Tung, SY

Lee, VHF

Lam, AKY

Pillai, S

Law, S

Lung, ML

AF Chai, Annie Wai Yeeng

Cheung, Arthur Kwok Leung

Dai, Wei

Ko, Josephine Mun Yee

Ip, Joseph Chok Yan

Chan, Kwok Wah

Kwong, Dora Lai-Wan

Ng, Wai Tong

Lee, Anne Wing Mui

Ngan, Roger Kai Cheong

Yau, Chun Chung

Tung, Stewart Yuk

Lee, Victor Ho Fun

Lam, Alfred King-Yin

Pillai, Suja

Law, Simon

Lung, Maria Li

TI Metastasis-suppressing NID2, an epigenetically-silenced gene, in the

pathogenesis of nasopharyngeal carcinoma and esophageal squamous cell

carcinoma

SO ONCOTARGET

LA English

DT Article

DE Nidogen-2 (NID2); promoter hypermethylation; metastasis; nasopharyngeal

carcinoma (NPC); esophageal squamous cell carcinoma (ESCC)

ID TUMOR-SUPPRESSOR; PROMOTER HYPERMETHYLATION; MONOCHROMOSOME TRANSFER;

EXTRACELLULAR-MATRIX; FUNCTIONAL EVIDENCE; METHYLATED GENES; DNA

METHYLATION; EGF RECEPTOR; CANCER; GROWTH

AB Nidogen-2 (NID2) is a key component of the basement membrane that stabilizes the extracellular matrix (ECM) network. The aim of the study is to analyze the functional roles of NID2 in the pathogenesis of nasopharyngeal carcinoma (NPC) and esophageal squamous cell carcinoma (ESCC). We performed genome-wide methylation profiling of NPC and ESCC and validated our findings using the methylation-sensitive high-resolution melting (MS-HRM) assay. Results showed that promoter methylation of NID2 was significantly higher in NPC and ESCC samples than in their adjacent non-cancer counterparts. Consistently, down-regulation of NID2 was observed in the clinical samples and cell lines of both NPC and ESCC. Re-expression of NID2 suppresses clonogenic survival and migration abilities of transduced NPC and ESCC cells. We showed that NID2 significantly inhibits liver metastasis. Mechanistic studies of signaling pathways also confirm that NID2 suppresses the EGFR/Akt and integrin/ FAK/PLC. metastasis-related pathways. This study provides novel insights into the crucial tumor metastasis suppression roles of NID2 in cancers.

C1 [Chai, Annie Wai Yeeng; Cheung, Arthur Kwok Leung; Dai, Wei; Ko, Josephine Mun Yee; Ip, Joseph Chok Yan; Kwong, Dora Lai-Wan; Lee, Anne Wing Mui; Lee, Victor Ho Fun; Lung, Maria Li] Univ Hong Kong, Dept Clin Oncol, Hong Kong, Hong Kong, Peoples R China.

[Chan, Kwok Wah; Law, Simon; Lung, Maria Li] Univ Hong Kong, Ctr Canc Res, Hong Kong, Hong Kong, Peoples R China.

[Chan, Kwok Wah] Univ Hong Kong, Dept Pathol, Hong Kong, Hong Kong, Peoples R China.

[Kwong, Dora Lai-Wan; Ng, Wai Tong; Lee, Anne Wing Mui; Ngan, Roger Kai Cheong; Yau, Chun Chung; Tung, Stewart Yuk; Lee, Victor Ho Fun; Lung, Maria Li] Univ Hong Kong, Ctr Nasopharyngeal Carcinoma Res, Hong Kong, Hong Kong, Peoples R China.

[Ng, Wai Tong] Pamela Youde Nethersole Eastern Hosp, Dept Clin Oncol, Hong Kong, Hong Kong, Peoples R China.

[Ngan, Roger Kai Cheong] Queen Elizabeth Hosp, Dept Clin Oncol, Hong Kong, Hong Kong, Peoples R China.

[Yau, Chun Chung] Princess Margaret Hosp, Dept Oncol, Hong Kong, Hong Kong, Peoples R China.

[Tung, Stewart Yuk] Tuen Mun Hosp, Dept Clin Oncol, Hong Kong, Hong Kong, Peoples R China.

[Lam, Alfred King-Yin; Pillai, Suja] Griffith Univ, Dept Canc Mol Pathol, Griffith Med Sch, Gold Coast, Australia.

[Lam, Alfred King-Yin; Pillai, Suja] Griffith Univ, Menzies Hlth Inst Queensland, Gold Coast, Australia.

[Law, Simon] Univ Hong Kong, Dept Surg, Hong Kong, Hong Kong, Peoples R China.

RP Lung, ML (corresponding author), Univ Hong Kong, Dept Clin Oncol, Hong Kong, Hong Kong, Peoples R China.; Lung, ML (corresponding author), Univ Hong Kong, Ctr Canc Res, Hong Kong, Hong Kong, Peoples R China.; Lung, ML (corresponding author), Univ Hong Kong, Ctr Nasopharyngeal Carcinoma Res, Hong Kong, Hong Kong, Peoples R China.

EM mlilung@hku.hk

RI Dai, Wei/T-8889-2019; Ko, Josephine/U-2972-2019; Lam,

Alfred/C-1652-2008; Pillai, Suja/T-3276-2017; Yeeng, Annie Chai

Wai/X-4608-2019; Ng, Wai Tong/H-9525-2019; Pillai, Suja/AAG-4056-2019;

Law, Simon/C-4324-2009; Lung, Maria Li/C-4495-2009

OI Dai, Wei/0000-0002-5558-0685; Ko, Josephine/0000-0002-7997-331X; Lam,

Alfred/0000-0003-2771-564X; Pillai, Suja/0000-0002-6572-1840; Yeeng,

Annie Chai Wai/0000-0002-8015-6050; Ng, Wai Tong/0000-0001-6696-3000;

Pillai, Suja/0000-0002-6572-1840; Law, Simon/0000-0002-6518-5806; Lee,

Victor/0000-0002-6283-978X; Lung, Maria Li/0000-0003-2559-3626

FU Research Grants Council of the Hong Kong Special Administrative Region,

People's Republic of China [17115214]

FX This work was supported by the Research Grants Council of the Hong Kong

Special Administrative Region, People's Republic of China: General

Research Fund grant number 17115214 to MLL.

CR Abern MR, 2014, UROL ONCOL, V32, pe21

Aoudjit Fawzi, 2012, Chemother Res Pract, V2012, P283181, DOI 10.1155/2012/283181

Baylin SB, 1998, ADV CANCER RES, V72, P141

Bill HM, 2004, MOL CELL BIOL, V24, P8586, DOI 10.1128/MCB.24.19.8586-8599.2004

Cabodi S, 2004, BIOCHEM SOC T, V32, P438, DOI 10.1042/BST0320438

Chan KC, 2011, INT J CANCER, V129, P1826, DOI 10.1002/ijc.25855

Chan SHK, 2011, INT J CANCER, V129, P565, DOI 10.1002/ijc.25698

Chen H, 2012, CANCER LETT, V325, P89, DOI 10.1016/j.canlet.2012.06.005

Cheng Y, 1998, P NATL ACAD SCI USA, V95, P3042, DOI 10.1073/pnas.95.6.3042

Cheung AKL, 2008, CANCER RES, V68, P8137, DOI 10.1158/0008-5472.CAN-08-0904

Cheung AKL, 2015, ONCOTARGET, V6, P13434

Cheung AKL, 2009, P NATL ACAD SCI USA, V106, P14478, DOI 10.1073/pnas.0900198106

Cheung ST, 1999, INT J CANCER, V83, P121, DOI 10.1002/(SICI)1097-0215(19990924)83:1<121::AID-IJC21>3.0.CO;2-F

Dai W, 2015, CANC MED

Dai W, 2016, CHIN CLIN ONCOL, V5, DOI 10.21037/cco.2016.03.06

Esteller M, 1999, CANCER RES, V59, P67

Franken NAP, 2006, NAT PROTOC, V1, P2315, DOI 10.1038/nprot.2006.339

Geng JF, 2012, ONCOL LETT, V3, P901, DOI 10.3892/ol.2012.587

Guerrero-Preston R, 2011, CANCER PREV RES, V4, P1061, DOI 10.1158/1940-6207.CAPR-11-0006

Gustafson KS, 2009, CANCER CYTOPATHOL, V117, P351

Hanawa M, 2006, INT J CANCER, V118, P1173, DOI 10.1002/ijc.21454

HERMAN JG, 1995, CANCER RES, V55, P4525

Hood JD, 2002, NAT REV CANCER, V2, P91, DOI 10.1038/nrc727

HUANG DP, 1980, INT J CANCER, V26, P127, DOI 10.1002/ijc.2910260202

Ilic D, 1998, J CELL BIOL, V143, P547

Jones PA, 2002, NAT REV GENET, V3, P415, DOI 10.1038/nrg816

Jones PA, 2007, CELL, V128, P683, DOI 10.1016/j.cell.2007.01.029

Kan R, 2015, PLOS ONE, V10, DOI 10.1371/journal.pone.0127239

Karnes RJ, 2012, MAYO CLIN PROC, V87, P835, DOI 10.1016/j.mayocp.2012.04.013

Kim SH, 2011, J ENDOCRINOL, V209, P139, DOI 10.1530/JOE-10-0377

Ko JMY, 2005, GENE CHROMOSOME CANC, V43, P284, DOI 10.1002/gcc.20190

Ko JMY, 2008, MOL CANCER RES, V6, P592, DOI 10.1158/1541-7786.MCR-07-0154

Kohfeldt E, 1998, J MOL BIOL, V282, P99, DOI 10.1006/jmbi.1998.2004

Law EWL, 2012, ONCOGENE, V31, P728, DOI 10.1038/onc.2011.272

Li HM, 2006, INT J CANCER, V119, P1567, DOI 10.1002/ijc.22032

Li LC, 2002, BIOINFORMATICS, V18, P1427, DOI 10.1093/bioinformatics/18.11.1427

Lin DC, 2014, NAT GENET, V46, P467, DOI 10.1038/ng.2935

Lo KW, 2000, CANCER RES, V60, P3348

Lo PHY, 2007, ONCOGENE, V26, P148, DOI 10.1038/sj.onc.1209767

Lo PHY, 2007, CANCER LETT, V257, P199, DOI 10.1016/j.canlet.2007.07.018

Lo PHY, 2010, CANCER RES, V70, P5567, DOI 10.1158/0008-5472.CAN-09-4510

Lu PF, 2012, J CELL BIOL, V196, P395, DOI 10.1083/jcb.201102147

Lukashev ME, 1998, TRENDS CELL BIOL, V8, P437, DOI 10.1016/S0962-8924(98)01362-2

Lung HL, 2005, ONCOGENE, V24, P6525, DOI 10.1038/sj.onc.1208812

Lung HL, 2008, INT J CANCER, V123, P401, DOI 10.1002/ijc.23528

Lung HL, 2012, SEMIN CANCER BIOL, V22, P87, DOI 10.1016/j.semcancer.2011.11.002

Lung HL, 2010, INT J CANCER, V127, P304, DOI 10.1002/ijc.25047

Maldonado L, 2014, ONCOTARGET, V5, P5218, DOI 10.18632/oncotarget.2129

Miosge N, 2001, HISTOCHEM J, V33, P523, DOI 10.1023/A:1014995523521

Mitra SK, 2006, CURR OPIN CELL BIOL, V18, P516, DOI 10.1016/j.ceb.2006.08.011

Mokkapati S, 2012, J HISTOCHEM CYTOCHEM, V60, P280, DOI 10.1369/0022155412436586

Mouw JK, 2014, NAT MED, V20, P360, DOI 10.1038/nm.3497

Phoon YP, 2015, INT J CANC

Reinert T, 2011, CLIN CANCER RES, V17, P5582, DOI 10.1158/1078-0432.CCR-10-2659

Renard Isabelle, 2010, Eur Urol, V58, P96, DOI 10.1016/j.eururo.2009.07.041

Schneider CA, 2012, NAT METHODS, V9, P671, DOI 10.1038/nmeth.2089

Seewaldt V, 2014, NAT MED, V20, P332, DOI 10.1038/nm.3523

Tse EYT, 2012, J PATHOL, V226, P645, DOI 10.1002/path.3957

Ulazzi L, 2007, MOL CANCER, V6, DOI 10.1186/1476-4598-6-17

Valastyan S, 2011, CELL, V147, P275, DOI 10.1016/j.cell.2011.09.024

Wojdacz Tomasz K, 2008, BMC Res Notes, V1, P54, DOI 10.1186/1756-0500-1-54

Wong VCL, 2008, INT J CANCER, V123, P2816, DOI 10.1002/ijc.23882

Wong VCL, 2012, INT J CANCER, V130, P83, DOI 10.1002/ijc.25970

Yang XS, 2015, INT J CANCER, V136, pE127, DOI 10.1002/ijc.29192

Yegin Z, 2013, DNA CELL BIOL, V32, P386, DOI 10.1089/dna.2013.2030

Yip WK, 2008, ONCOL REP, V19, P319

Yu VZ, 2015, GASTROENTEROLOGY, V149, P1825, DOI 10.1053/j.gastro.2015.08.025

Yurchenco PD, 2011, CSH PERSPECT BIOL, V3, DOI 10.1101/cshperspect.a004911

NR 68

TC 25

Z9 25

U1 0

U2 8

PU IMPACT JOURNALS LLC

PI ORCHARD PARK

PA 6666 E QUAKER ST, STE 1, ORCHARD PARK, NY 14127 USA

EI 1949-2553

J9 ONCOTARGET

JI Oncotarget

PD NOV 29

PY 2016

VL 7

IS 48

BP 78859

EP 78871

DI 10.18632/oncotarget.12889

PG 13

WC Oncology; Cell Biology

WE Science Citation Index Expanded (SCI-EXPANDED)

SC Oncology; Cell Biology

GA EE5HH

UT WOS:000389636000055

PM 27793011

OA Green Published, gold

DA 2022-05-19

ER

PT J

AU Xu, YT

Wang, J

Qiu, MT

Xu, L

Li, M

Jiang, F

Yin, R

Xu, L

AF Xu, Youtao

Wang, Jie

Qiu, Mantang

Xu, Lei

Li, Ming

Jiang, Feng

Yin, Rong

Xu, Lin

TI Upregulation of the long noncoding RNA TUG1 promotes proliferation and

migration of esophageal squamous cell carcinoma

SO TUMOR BIOLOGY

LA English

DT Article

DE TUG1; Long noncoding RNA; Esophageal squamous cell carcinoma; Expression

ID LUNG-CANCER; ADENOCARCINOMA CELLS; GENE-EXPRESSION; HOTAIR;

DIFFERENTIATION; METASTASIS; COMPLEXES; PROGNOSIS; INVASION; DISEASE

AB Esophageal squamous cell carcinoma (ESCC) is one of the prevalent and deadly cancers worldwide, especially in Eastern Asia. The prognosis of ESCC remains poor; thus, it is still necessary to further dissect the underlying mechanisms and explore therapeutic targets of ESCC. Recent studies show that long noncoding RNAs (lncRNAs) have critical roles in diverse biological processes, including tumorigenesis. Some lncRNAs, such as HOTAIR and POU3F3, were reported to play important roles in ESCC. Here, we characterized the expression profile of taurine-upregulated gene 1 (TUG1), a lncRNA recruiting and binding to polycomb repressive complex 2 (PRC2), in ESCC. In a cohort of 62 patients, TUG1 was significantly overexpressed in ESCC tissues compared with paired adjacent normal tissues, and high expression level of TUG1 was associated with family history and upper segment of esophageal cancer (p < 0.05). Further, in vitro silencing TUG1 via siRNA inhibited the proliferation and migration of ESCC cells and blocked the progression of cell cycle. Therefore, our study indicates that TUG1 promotes proliferation and migration of ESCC cells and is a potential oncogene of ESCC.

C1 [Xu, Youtao; Qiu, Mantang; Li, Ming; Jiang, Feng; Yin, Rong; Xu, Lin] Nanjing Med Univ, Dept Thorac Surg, Affiliated Canc Hosp, Jiangsu Key Lab Mol & Translat Canc Res,Canc Inst, Nanjing 210009, Peoples R China.

[Xu, Youtao] Nanjing Med Univ, Clin Coll 1, Nanjing 210000, Jiangsu, Peoples R China.

[Wang, Jie] Nanjing Med Univ, Canc Inst Jiangsu Prov, Affiliated Canc Hosp, Dept Sci Res, Nanjing 210009, Peoples R China.

[Qiu, Mantang] Nanjing Med Univ, Clin Coll 4, Nanjing 210000, Jiangsu, Peoples R China.

[Xu, Lei] NanjingMed Univ, Dept Thorac Surg, Affiliated Nanjing Hosp, Nanjing 211100, Jiangsu, Peoples R China.

RP Yin, R (corresponding author), Nanjing Med Univ, Dept Thorac Surg, Affiliated Canc Hosp, Jiangsu Key Lab Mol & Translat Canc Res,Canc Inst, Baiziting 42, Nanjing 210009, Peoples R China.

EM yinhero001@126.com; xulin83cn@gmail.com

RI Qiu, Mantang/L-8937-2019

FU Natural Science Foundation of China [81372321, 81201830, 81472200];

Natural Science Foundation for High Education of Jiangsu Province

[13KJB320010]; Foundation of Jiangsu Cancer Hospital [ZQ201103]; Jiangsu

Provincial Special Program of Medical Science [BL2012030]

FX This study is funded by the Natural Science Foundation of China

(81372321 to L. Xu; 81201830 and 81472200 to R. Yin), Natural Science

Foundation for High Education of Jiangsu Province (13KJB320010 to R.

Yin), Foundation of Jiangsu Cancer Hospital (ZQ201103 to J. Wang), and

Jiangsu Provincial Special Program of Medical Science (BL2012030 to L.

Xu).

CR EL Andaloussi S, 2013, NAT REV DRUG DISCOV, V12, P348, DOI 10.1038/nrd3978

Enzinger PC, 2003, NEW ENGL J MED, V349, P2241, DOI 10.1056/NEJMra035010

Esteller M, 2011, NAT REV GENET, V12, P861, DOI 10.1038/nrg3074

Gao Y, 2009, BMC CANCER, V9, DOI 10.1186/1471-2407-9-269

Geng YJ, 2011, J INT MED RES, V39, P2119, DOI 10.1177/147323001103900608

Gezer U, 2014, CELL BIOL INT

GUO WD, 1994, INT J EPIDEMIOL, V23, P444, DOI 10.1093/ije/23.3.444

Gupta RA, 2010, NATURE, V464, P1071, DOI 10.1038/nature08975

Guttman M, 2011, NATURE, V477, P295, DOI 10.1038/nature10398

Han YH, 2013, J SURG ONCOL, V107, P555, DOI 10.1002/jso.23264

Hao JJ, 2013, GENE, V513, P196, DOI 10.1016/j.gene.2012.09.091

ISONO K, 1991, ONCOLOGY, V48, P411

Khalil AM, 2009, P NATL ACAD SCI USA, V106, P11667, DOI 10.1073/pnas.0904715106

Kim K, 2013, ONCOGENE, V32, P1616, DOI 10.1038/onc.2012.193

Li J, 2013, INT J MOL SCI, V14, P18790, DOI 10.3390/ijms140918790

Li W, 2014, GASTROENTEROLOGY, V146, P1714, DOI 10.1053/j.gastro.2014.03.002

Li X, 2013, BRIT J CANCER, V109, P2266, DOI 10.1038/bjc.2013.548

Ma LN, 2013, RNA BIOL, V10, P925, DOI 10.4161/rna.24604

MAHBOUBI E, 1973, BRIT J CANCER, V28, P192, DOI 10.1038/bjc.1973.138

Matsushima K, 2011, J TRANSL MED, V9, DOI 10.1186/1479-5876-9-30

McCann J, 1999, J NATL CANCER I, V91, P497, DOI 10.1093/jnci/91.6.497

Nakagawa T, 2013, BIOCHEM BIOPH RES CO, V436, P319, DOI 10.1016/j.bbrc.2013.05.101

Parkin DM, 2005, CA-CANCER J CLIN, V55, P74, DOI 10.3322/canjclin.55.2.74

Pohl H, 2005, J NATL CANCER I, V97, P142, DOI 10.1093/jnci/dji024

Qi P, 2013, MODERN PATHOL, V26, P155, DOI 10.1038/modpathol.2012.160

Qiu MT, 2013, TUMOR BIOL, V34, P613, DOI 10.1007/s13277-013-0658-6

Qiu MT, 2014, TUMOR BIOL, V35, P5375, DOI 10.1007/s13277-014-1700-z

Ren SC, 2013, EUR J CANCER, V49, P2949, DOI 10.1016/j.ejca.2013.04.026

Schmidt LH, 2011, J THORAC ONCOL, V6, P1984, DOI 10.1097/JTO.0b013e3182307eac

Tano K, 2010, FEBS LETT, V584, P4575, DOI 10.1016/j.febslet.2010.10.008

Tsai MC, 2010, SCIENCE, V329, P689, DOI 10.1126/science.1192002

VanGuilder HD, 2008, BIOTECHNIQUES, V44, P619, DOI 10.2144/000112776

VIGNESWARAN WT, 1994, J THORAC CARDIOV SUR, V107, P901

Wang CM, 2014, DIGEST DIS SCI, V59, P591, DOI 10.1007/s10620-013-2956-7

Wang KC, 2011, MOL CELL, V43, P904, DOI 10.1016/j.molcel.2011.08.018

Wilusz JE, 2009, GENE DEV, V23, P1494, DOI 10.1101/gad.1800909

Yamaguchi H, 2014, CANCER RES TREAT, V46, P209, DOI 10.4143/crt.2014.46.3.209

Yang X, 2014, GUT, V63, P881, DOI 10.1136/gutjnl-2013-305266

Young TL, 2005, CURR BIOL, V15, P501, DOI 10.1016/j.cub.2005.02.027

Zhang EB, 2014, CELL DEATH DIS, V5, DOI 10.1038/cddis.2014.201

Zhang Q, 2013, ASIAN PAC J CANCER P, V14, P2311, DOI 10.7314/APJCP.2013.14.4.2311

NR 41

TC 126

Z9 141

U1 1

U2 39

PU SAGE PUBLICATIONS LTD

PI LONDON

PA 1 OLIVERS YARD, 55 CITY ROAD, LONDON EC1Y 1SP, ENGLAND

SN 1010-4283

EI 1423-0380

J9 TUMOR BIOL

JI Tumor Biol.

PD MAR

PY 2015

VL 36

IS 3

BP 1643

EP 1651

DI 10.1007/s13277-014-2763-6

PG 9

WC Oncology

WE Science Citation Index Expanded (SCI-EXPANDED)

SC Oncology

GA CE5OC

UT WOS:000351884000037

PM 25366138

DA 2022-05-19

ER

PT J

AU Shi, H

Ren, HR

Yang, XJ

Zhu, HZ

Yao, L

Hang, QL

Mao, H

Huang, YJ

Zhang, JG

Wang, YC

AF Shi, Hui

Ren, Hanru

Yang, Xiaojing

Zhu, Hongzhen

Yao, Li

Hang, Qinglei

Mao, Hui

Huang, Yuejiao

Zhang, Jianguo

Wang, Yuchan

TI Overexpression of activator of G-protein signaling 3 decreases the

proliferation of esophageal squamous cell carcinoma

SO PATHOLOGY RESEARCH AND PRACTICE

LA English

DT Article

DE Human esophageal squamous cell carcinoma; AGS3; Proliferation; Prognosis

ID CYCLE PROGRESSION; REGULATOR AGS3; BETA-GAMMA; CANCER; EXPRESSION;

SEEKING; MACROAUTOPHAGY; MIGRATION; MOTIF

AB Background: Activator of G-protein Signaling 3 (AGS3, also known as GPSM1), is related to cell cycle progression. We investigated the expression of AGS3 in human esophageal squamous cell carcinoma (ESCC) and the therapeutic effect of chemotherapy drugs.

Methods: Immunohistochemistry and Western blot analysis were performed for AGS3 in 85ESCC samples. The data were correlated with clinicopathological features. The univariate and multivariate survival analyses were also performed to determine its prognostic significance. The effect of overexpression of AGS3 on proliferation of esophageal carcinoma TE1 cells was analyzed by serum starvation.

Results: AGS3 was down regulated in ESCC as compared with the adjacent normal tissue. Low expression of AGS3 was associated with tumor grade (P=0.002), and AGS3 was negatively correlated with proliferation marker Ki-67 (P < 0.01). Univariate analysis showed that AGS3 expression did have a remarkable prediction for poor prognosis (P = 0.004), while in vitro, the expression of AGS3 was down regulated with release from serum starvation of TE1 cells.

Conclusions: This study shows that AGS3 is an important regulator of ESCC proliferation. (C) 2015 Elsevier GmbH. All rights reserved.

C1 [Shi, Hui] Nantong Univ, Affiliated Hosp, Dept Thorac Surg, Nantong 226001, Jiangsu, Peoples R China.

[Ren, Hanru] Tongji Univ, Sch Med, Shanghai East Hosp, Dept Thorac Surg, Shanghai 200120, Peoples R China.

[Zhang, Jianguo] Nantong Univ, Affiliated Hosp, Dept Pathol, Nantong 226001, Jiangsu, Peoples R China.

[Yang, Xiaojing; Zhu, Hongzhen; Hang, Qinglei; Mao, Hui; Huang, Yuejiao; Wang, Yuchan] Nantong Univ, Jiangsu Prov Key Lab Inflammat & Mol Drug Target, Nantong 226001, Jiangsu, Peoples R China.

[Wang, Yuchan] Nantong Univ, Dept Pathogen & Immunol, Coll Med, Nantong 226001, Jiangsu, Peoples R China.

[Yang, Xiaojing] Shanghai Jiao Tong Univ, Dept Oncol, Affiliated Peoples Hosp 6, Shanghai 200233, Peoples R China.

[Yao, Li] Jiangnan Univ, Dept Immunol, Coll Med, Wuxi 214122, Jiangsu, Peoples R China.

RP Zhang, JG (corresponding author), Nantong Univ, Affiliated Hosp, Dept Pathol, 20Xi Si Rd, Nantong 226001, Jiangsu, Peoples R China.

EM zangjg125@163.com; ychan.wang@163.com

FU National Natural Science Foundation of China [81201858]; Natural

Scientific Foundation of Jiangsu Province [BK2012231]; Priority Academic

Program Development of Jiangsu Higher Education Institutions (PAPD);

Social Scientific and Technological Innovation of Nantong City

[HS2012060]

FX Supported by grants from the National Natural Science Foundation of

China (No. 81201858); Natural Scientific Foundation of Jiangsu Province

Grant (No. BK2012231); A Project Funded by the Priority Academic Program

Development of Jiangsu Higher Education Institutions (PAPD); Social

Scientific and Technological Innovation of Nantong City (HS2012060).

CR BAUM HP, 1993, ARCH DERMATOL RES, V285, P291, DOI 10.1007/BF00371599

Bernard ML, 2001, J BIOL CHEM, V276, P1585, DOI 10.1074/jbc.M005291200

Blumer JB, 2003, J BIOL CHEM, V278, P23217, DOI 10.1074/jbc.C200686200

Blumer JB, 2008, ENDOCRINOLOGY, V149, P3842, DOI 10.1210/en.2008-0050

Bowers MS, 2008, P NATL ACAD SCI USA, V105, P12533, DOI 10.1073/pnas.0706999105

Bowers MS, 2004, NEURON, V42, P269, DOI 10.1016/S0896-6273(04)00159-X

Cismowski MJ, 1999, NAT BIOTECHNOL, V17, P878, DOI 10.1038/12867

Enzinger PC, 2003, NEW ENGL J MED, V349, P2241, DOI 10.1056/NEJMra035010

Fan PD, 2009, MOL PHARMACOL, V76, P526, DOI 10.1124/mol.109.057802

Groves B, 2007, P NATL ACAD SCI USA, V104, P18103, DOI 10.1073/pnas.0709282104

Groves B, 2010, PLOS ONE, V5, DOI 10.1371/journal.pone.0008877

Gu Y, 2012, ONCOGENE, V31, P469, DOI 10.1038/onc.2011.247

Jemal A, 2010, CA-CANCER J CLIN, V60, P277, DOI 10.3322/caac.20073

Kaushik R, 2003, MOL BIOL CELL, V14, P3144, DOI 10.1091/mbc.E03-04-0212

Li M, 2013, MED ONCOL, V30, DOI 10.1007/s12032-012-0396-4

Maddams J, 2011, INT J CANCER, V129, P2885, DOI 10.1002/ijc.26240

Pattingre S, 2003, J BIOL CHEM, V278, P20995, DOI 10.1074/jbc.M300917200

Peterson YK, 2002, J BIOL CHEM, V277, P6767, DOI 10.1074/jbc.C100699200

Roth JA, 1997, J NATL CANCER I, V89, P21, DOI 10.1093/jnci/89.1.21

Sanada K, 2005, CELL, V122, P119, DOI 10.1016/j.cell.2005.05.009

Shimizu T, 2012, PLOS ONE, V7, DOI 10.1371/journal.pone.0045510

Takesono A, 1999, J BIOL CHEM, V274, P33202, DOI 10.1074/jbc.274.47.33202

WASEEM NH, 1990, J CELL SCI, V96, P121

Yao L, 2005, P NATL ACAD SCI USA, V102, P8746, DOI 10.1073/pnas.0503419102

NR 24

TC 3

Z9 4

U1 0

U2 2

PU ELSEVIER GMBH, URBAN & FISCHER VERLAG

PI JENA

PA OFFICE JENA, P O BOX 100537, 07705 JENA, GERMANY

SN 0344-0338

J9 PATHOL RES PRACT

JI Pathol. Res. Pract.

PY 2015

VL 211

IS 6

BP 449

EP 455

DI 10.1016/j.prp.2014.12.016

PG 7

WC Pathology

WE Science Citation Index Expanded (SCI-EXPANDED)

SC Pathology

GA CJ6XV

UT WOS:000355639400005

PM 25812748

DA 2022-05-19

ER

PT J

AU Ren, P

Zhang, JG

Xiu, L

Yu, ZT

AF Ren, P.

Zhang, J-G.

Xiu, L.

Yu, Z-T.

TI Clinical significance of phospholipase A2 group IIA (PLA2G2A) expression

in primary resected esophageal squamous cell carcinoma

SO EUROPEAN REVIEW FOR MEDICAL AND PHARMACOLOGICAL SCIENCES

LA English

DT Article

DE Esophageal squamous cell carcinoma; Phospholipase A2 group IIA;

Prognosis

ID SECRETORY PHOSPHOLIPASE-A2; GENETIC POLYMORPHISMS; PROLONGED SURVIVAL;

CANCER; A(2); ADENOCARCINOMA; BIOMARKER; CLASSIFICATION; A2-IIA

AB AIM: The aim of this study was to clarify the clinico-pathological outcome and prognostic significance of phospholipase A2 group IIA (PLA2G2A) in esophageal squamous cell carcinoma (ESCC).

PATIENTS AND METHODS: Immunohistochemical staining for PLA2G2A was performed on surgical specimens obtained from 132 patients with ESCC, and 43 from matched adjacent non-malignant sites. Differences in PLA2G2A expression and clinical characteristics were compared by chi(2) test. Correlations between prognostic outcomes and with PLA2G2A expression were investigated using Kaplan-Meier analysis and the Cox proportional hazards model.

RESULTS: Immunoreactivity of PLA2G2A was observed in 32% (42 of 132) of ESCC tissues compared with negative staining in matched adjacent non-malignant sites. In addition, PLA2G2A expression inversely correlated with pathological classification (p < 0.05 for T, N, and M classifications) and clinical staging (p = 0.03). Furthermore, patients with positive PLA2G2A had prolonged overall survival (p < 0.01).

CONCLUSIONS: Reduced PLA2G2A expression may be a risk factor for advanced clinico-pathological classification and poor patient survival. These findings suggest that PLA2G2A may serve as a useful marker for the prognostic evaluation of ESCC patients.

C1 [Yu, Z-T.] Tianjin Med Univ, Canc Inst & Hosp, Dept Esophageal Canc, Tianjin, Peoples R China.

Key Lab Canc Prevent & Therapy, Tianjin, Peoples R China.

RP Yu, ZT (corresponding author), Tianjin Med Univ, Canc Inst & Hosp, Dept Esophageal Canc, Tianjin, Peoples R China.

EM renpengtj@126.com

CR Avoranta T, 2010, VIRCHOWS ARCH, V457, P659, DOI 10.1007/s00428-010-0992-7

Birts CN, 2010, TRENDS BIOCHEM SCI, V35, P28, DOI 10.1016/j.tibs.2009.08.003

Buhmeida A, 2009, ANN ONCOL, V20, P1230, DOI 10.1093/annonc/mdn783

Dong ZY, 2010, CARCINOGENESIS, V31, P1948, DOI 10.1093/carcin/bgq188

Fijneman RJA, 2008, FRONT BIOSCI-LANDMRK, V13, P4144, DOI 10.2741/2998

Graff JR, 2001, CLIN CANCER RES, V7, P3857

Hiyama T, 2007, INT J CANCER, V121, P1643, DOI 10.1002/ijc.23044

Jiang JZ, 2002, AM J PATHOL, V160, P667, DOI 10.1016/S0002-9440(10)64886-9

Kamangar F, 2006, J CLIN ONCOL, V24, P2137, DOI 10.1200/JCO.2005.05.2308

Kashiwagi M, 1999, GUT, V45, P605, DOI 10.1136/gut.45.4.605

Kupert E, 2011, BMC CANCER, V11, DOI 10.1186/1471-2407-11-513

Leung SY, 2002, P NATL ACAD SCI USA, V99, P16203, DOI 10.1073/pnas.212646299

Mauchley D, 2010, J THORAC CARDIOV SUR, V139, P591, DOI 10.1016/j.jtcvs.2009.10.061

Mounier CM, 2008, BRIT J CANCER, V98, P587, DOI 10.1038/sj.bjc.6604184

Reilly MP, 1998, J NUTR, V128, p434S, DOI 10.1093/jn/128.2.434S

Shappell SB, 2003, CANCER RES, V63, P2256

Six DA, 2000, BBA-MOL CELL BIOL L, V1488, P1, DOI 10.1016/S1388-1981(00)00105-0

Sobin LH, 1997, CANCER, V80, P1803, DOI 10.1002/(SICI)1097-0142(19971101)80:9<1803::AID-CNCR16>3.0.CO;2-9

Xing DY, 2003, ONCOL REP, V10, P1615

Xing XF, 2011, HISTOPATHOLOGY, V59, P198, DOI 10.1111/j.1365-2559.2011.03913.x

NR 20

TC 14

Z9 15

U1 0

U2 1

PU VERDUCI PUBLISHER

PI ROME

PA VIA GREGORIO VII, ROME, 186-00165, ITALY

SN 1128-3602

J9 EUR REV MED PHARMACO

JI Eur. Rev. Med. Pharmacol. Sci.

PD MAR

PY 2013

VL 17

IS 6

BP 752

EP 757

PG 6

WC Pharmacology & Pharmacy

WE Science Citation Index Expanded (SCI-EXPANDED)

SC Pharmacology & Pharmacy

GA 228SE

UT WOS:000325207900006

PM 23609358

DA 2022-05-19

ER

PT J

AU Xu, XF

Guan, XY

Tao, HS

Yang, K

Bai, Y

AF Xu, Xiaofeng

Guan, Xingying

Tao, Huansheng

Yang, Kang

Bai, Yun

TI An Association Study on Genetic Polymorphisms of Rab37 Gene with the

Risk of Esophageal Squamous Cell Carcinoma in a Chinese Han Population

SO INTERNATIONAL JOURNAL OF MEDICAL SCIENCES

LA English

DT Article

DE Rab37; Esophageal squamous cell carcinoma; Polymorphisms; Case-control

study

ID SECRETORY GRANULES; CANCER; SUSCEPTIBILITY; EXPRESSION; C20ORF54; PLCE1;

LINES; LOCI

AB Background: Rab37 encodes a Rab GTPase which regulates the vesicular transport of exocytosis. But the different findings in two types of cancers made its roles in oncology more confused. In this study, a clinical research on genetic polymorphisms was performed to evaluate the association between Rab37 and esophageal squamous cell carcinoma (ESCC).

Methods: The mRNA expression was tested by reverse transcription-polymerase chain reaction (RT-PCR) in four ESCC cell lines. A case-control study including 212 ESCC patients and 213 cancer-free controls was genotyped by PCR-restriction fragment length polymorphism. The Hardy-Weinberg equilibrium test, association analysis and haplotype analysis were performed with SPSS and SHEsis software respectively.

Results: Rab37 mRNA could be specifically detected in two ESCC cell lines, EC109 and EC9706, but not in KYS150 and KYS450. The allele, genotype and haplotype frequencies of rs9904078G>A, rs2034310T>C and rs5018106T>C, located in Rab37, did not significantly differ between the patients and the controls. No association between the polymorphisms and the TNM stages of patients was found.

Conclusions: Rab37 mRNA was specifically expressed in some ESCC cell lines but its genetic polymorphisms were not associated with ESCC.

C1 [Xu, Xiaofeng; Guan, Xingying; Tao, Huansheng; Bai, Yun] Third Mil Med Univ, Dept Med Genet, Chongqing 400038, Peoples R China.

[Yang, Kang] Third Mil Med Univ, Southwest Hosp, Dept Thorac & Cardiac Surg, Chongqing 400038, Peoples R China.

RP Bai, Y (corresponding author), Third Mil Med Univ, Dept Med Genet, Chongqing 400038, Peoples R China.

EM kang-yang@tmmu.edu.cn; baiyungene@gmail.com

FU National Natural Science Foundation of China [31100131]; Innovational

Foundation for Youth of Third Military Medical University [2010XQN07]

FX This work was partially supported by grants 31100131 from the National

Natural Science Foundation of China and 2010XQN07 from the Innovational

Foundation for Youth of Third Military Medical University.

CR Abnet CC, 2010, NAT GENET, V42, P764, DOI 10.1038/ng.649

Barrett JC, 2005, BIOINFORMATICS, V21, P263, DOI 10.1093/bioinformatics/bth457

Brunner Y, 2007, MOL CELL PROTEOMICS, V6, P1007, DOI 10.1074/mcp.M600443-MCP200

Bryant DM, 2004, TRENDS CELL BIOL, V14, P427, DOI 10.1016/j.tcb.2004.07.007

DIAMOND L, 1980, CARCINOGENESIS, V1, P871, DOI 10.1093/carcin/1.10.871

Dobashi S, 2009, ONCOL REP, V21, P305, DOI 10.3892/or_00000222

Doki Y, 1997, CARCINOGENESIS, V18, P1139, DOI 10.1093/carcin/18.6.1139

Enzinger PC, 2003, NEW ENGL J MED, V349, P2241, DOI 10.1056/NEJMra035010

Fukuda M, 2003, J BIOL CHEM, V278, P15373, DOI 10.1074/jbc.M212341200

Gu HY, 2012, MOL BIOL REP, V39, P9105, DOI 10.1007/s11033-012-1782-x

Guo H, 2010, FAM CANCER, V9, P599, DOI 10.1007/s10689-010-9370-5

Hoffman AE, 2009, CANCER RES, V69, P5970, DOI 10.1158/0008-5472.CAN-09-0236

Ji AF, 2011, ASIAN PAC J CANCER P, V12, P3207

Kamangar F, 2006, J CLIN ONCOL, V24, P2137, DOI 10.1200/JCO.2005.05.2308

Masuda ES, 2000, FEBS LETT, V470, P61, DOI 10.1016/S0014-5793(00)01288-6

Mori R, 2011, EUR J IMMUNOL, V41, P3230, DOI 10.1002/eji.201141640

NAKABAYASHI H, 1984, GANN, V75, P151

Pellinen T, 2006, J CELL SCI, V119, P3723, DOI 10.1242/jcs.03216

Pickens A, 2003, ANN THORAC SURG, V76, pS1367, DOI 10.1016/S0003-4975(03)01202-5

Shi YY, 2005, CELL RES, V15, P97, DOI 10.1038/sj.cr.7290272

SHIMADA Y, 1992, CANCER, V69, P277, DOI 10.1002/1097-0142(19920115)69:2<277::AID-CNCR2820690202>3.0.CO;2-C

Tsuboi T, 2006, J CELL SCI, V119, P2196, DOI 10.1242/jcs.02962

Wang HT, 2003, WORLD J GASTROENTERO, V9, P392, DOI 10.3748/wjg.v9.i3.392

Wang K, 2010, BIOMARKERS, V15, P614, DOI 10.3109/1354750X.2010.505299

Wang LD, 2010, NAT GENET, V42, P759, DOI 10.1038/ng.648

Wu CY, 2009, LUNG CANCER, V63, P360, DOI 10.1016/j.lungcan.2008.06.014

Xiong G, 2010, TUMOR BIOL, V31, P443, DOI 10.1007/s13277-010-0053-5

Yang XY, 2009, J CANCER RES CLIN, V135, P1341, DOI 10.1007/s00432-009-0575-7

YU MC, 1988, CANCER RES, V48, P3843

NR 29

TC 5

Z9 5

U1 1

U2 2

PU IVYSPRING INT PUBL

PI LAKE HAVEN

PA PO BOX 4546, LAKE HAVEN, NSW 2263, AUSTRALIA

SN 1449-1907

J9 INT J MED SCI

JI Int. J. Med. Sci.

PY 2013

VL 10

IS 3

BP 235

EP 242

DI 10.7150/ijms.5524

PG 8

WC Medicine, General & Internal

WE Science Citation Index Expanded (SCI-EXPANDED)

SC General & Internal Medicine

GA 098QP

UT WOS:000315564100004

PM 23372429

OA gold, Green Submitted, Green Published

DA 2022-05-19

ER

PT J

AU Cao, W

Lee, HY

Wu, W

Zaman, A

McCorkle, S

Yan, M

Chen, JT

Xing, QH

Sinnott-Armstrong, N

Xu, HG

Sailani, MR

Tang, WX

Cui, YB

Liu, J

Guan, HY

Lv, PJ

Sun, XY

Sun, L

Han, PL

Lou, YA

Chang, J

Wang, JW

Gao, YC

Guo, JC

Schenk, G

Shain, AH

Biddle, FG

Collisson, E

Snyder, M

Bivona, TG

AF Cao, Wei

Lee, Hayan

Wu, Wei

Zaman, Aubhishek

McCorkle, Sean

Yan, Ming

Chen, Justin

Xing, Qinghe

Sinnott-Armstrong, Nasa

Xu, Hongen

Sailani, M. Reza

Tang, Wenxue

Cui, Yuanbo

Liu, Jia

Guan, Hongyan

Lv, Pengju

Sun, Xiaoyan

Sun, Lei

Han, Pengli

Lou, Yanan

Chang, Jing

Wang, Jinwu

Gao, Yuchi

Guo, Jiancheng

Schenk, Gundolf

Shain, Alan Hunter

Biddle, Fred G.

Collisson, Eric

Snyder, Michael

Bivona, Trever G.

TI Multi-faceted epigenetic dysregulation of gene expression promotes

esophageal squamous cell carcinoma

SO NATURE COMMUNICATIONS

LA English

DT Article

ID LONG NONCODING RNA; DNA METHYLATION; GENOMIC CHARACTERIZATION; CANCER;

ACCURATE; ENCYCLOPEDIA; MUTATIONS; LANDSCAPE; ALIGNMENT; INVASION

AB Epigenetic landscapes can shape physiologic and disease phenotypes. We used integrative, high resolution multi-omics methods to delineate the methylome landscape and characterize the oncogenic drivers of esophageal squamous cell carcinoma (ESCC). We found 98% of CpGs are hypomethylated across the ESCC genome. Hypo-methylated regions are enriched in areas with heterochromatin binding markers (H3K9me3, H3K27me3), while hyper-methylated regions are enriched in polycomb repressive complex (EZH2/SUZ12) recognizing regions. Altered methylation in promoters, enhancers, and gene bodies, as well as in polycomb repressive complex occupancy and CTCF binding sites are associated with cancer-specific gene dysregulation. Epigenetic-mediated activation of non-canonical WNT/beta-catenin/MMP signaling and a YY1/lncRNA ESCCAL-1/ribosomal protein network are uncovered and validated as potential novel ESCC driver alterations. This study advances our understanding of how epigenetic landscapes shape cancer pathogenesis and provides a resource for biomarker and target discovery. The epigenetic landscape of esophageal squamous cell carcinoma (ESCC) at genome-wide high resolution is incompletely studied. Here, the authors performed an integrated multi-omics analysis of ESCC and non-tumor tissues to define the genome-wide methylome landscape and epigenetic alterations to uncover oncogenic drivers of ESCC.

C1 [Cao, Wei; Cui, Yuanbo; Liu, Jia; Guan, Hongyan; Lv, Pengju; Sun, Xiaoyan; Sun, Lei; Han, Pengli; Lou, Yanan] Zhengzhou Univ, Zhengzhou Cent Hosp Affiliated, Translat Med Ctr, Zhengzhou, Peoples R China.

[Lee, Hayan; Chen, Justin; Sinnott-Armstrong, Nasa; Sailani, M. Reza; Snyder, Michael] Stanford Univ, Sch Med, Dept Genet, Stanford, CA 94305 USA.

[Wu, Wei; Zaman, Aubhishek; Collisson, Eric; Bivona, Trever G.] Univ Calif San Francisco, Dept Med, San Francisco, CA 94143 USA.

[Wu, Wei; Zaman, Aubhishek; Collisson, Eric; Bivona, Trever G.] Univ Calif San Francisco, Helen Diller Family Comprehens Canc Ctr, San Francisco, CA 94143 USA.

[McCorkle, Sean] Brookhaven Natl Lab, Computat Sci Initiat, Upton, NY 11973 USA.

[Yan, Ming] Zhengzhou Univ, Basic Med Coll, Zhengzhou, Peoples R China.

[Xing, Qinghe] Fudan Univ, Inst Biomed Sci, Shanghai, Peoples R China.

[Xing, Qinghe] Fudan Univ, Childrens Hosp, Shanghai, Peoples R China.

[Xu, Hongen; Tang, Wenxue; Guo, Jiancheng] Zhengzhou Univ, Acad Med Sci, Precis Med Ctr, Zhengzhou, Peoples R China.

[Chang, Jing] Jiangsu Mai Jian Biotechnol Dev Co, Wuxi, Jiangsu, Peoples R China.

[Wang, Jinwu] Linzhou Canc Hosp, Dept Pathol, Linzhou, Peoples R China.

[Gao, Yuchi] Annoroad Gene Co, Beijing, Peoples R China.

[Schenk, Gundolf] Univ Calif San Francisco, Bakar Computat Hlth Sci Inst, San Francisco, CA 94143 USA.

[Shain, Alan Hunter] Univ Calif San Francisco, Dept Dermatol, San Francisco, CA USA.

[Biddle, Fred G.] Univ Calgary, Dept Biol Sci, Calgary, AB, Canada.

RP Cao, W (corresponding author), Zhengzhou Univ, Zhengzhou Cent Hosp Affiliated, Translat Med Ctr, Zhengzhou, Peoples R China.; Snyder, M (corresponding author), Stanford Univ, Sch Med, Dept Genet, Stanford, CA 94305 USA.; Wu, W; Bivona, TG (corresponding author), Univ Calif San Francisco, Dept Med, San Francisco, CA 94143 USA.; Wu, W; Bivona, TG (corresponding author), Univ Calif San Francisco, Helen Diller Family Comprehens Canc Ctr, San Francisco, CA 94143 USA.

EM caowei7@zzu.edu.cn; wei.wu@ucsf.edu; mpsnyder@stanford.edu;

trever.bivona@ucsf.edu

RI wu, wei/J-2797-2019; Xu, HongEn/D-2418-2012

OI wu, wei/0000-0002-6556-067X; /0000-0002-4197-849X; Xu,

HongEn/0000-0001-7895-0899; Lee, Hayan/0000-0003-0571-3192; Xing,

Qinghe/0000-0003-1780-4761; tang, wenxue/0000-0001-8087-0900; McCorkle,

Sean/0000-0003-1126-4071; CAO, WEI/0000-0002-2851-4359

FU National Natural Science Foundation of China [81171992, 31570899];

Natural Science Foundation of Henan [182102310328, 162300410279,

182300410374, 192102310096]; Education Department of Henan Province

[18B310022, 19A320037]; NIH [S10OD020141]; NCI [CA178015, CA222862,

CA227807, CA239604, CA230263, CA210974]; NIH/NCI [R01CA169338,

U01CA217882, U54CA224081, R01CA204302, R01CA211052]; Pew-Stewart

Foundations

FX This study was supported by the National Natural Science Foundation of

China (Grants 81171992, 31570899), the Natural Science Foundation of

Henan (Grants 182102310328, 162300410279, 182300410374, 192102310096),

the Education Department of Henan Province(18B310022,19A320037). This

work used the Genome Sequencing Service Center by Stanford Center for

Genomics and Personalized Medicine Sequencing Center, supported by the

grant award NIH S10OD020141. E.C. acknowledges funding support from NCI

Grants R01 [CA178015, CA222862, CA227807, CA239604, CA230263] and U24

[CA210974]. T.G.B. acknowledges funding support from NIH/NCI

U01CA217882, NIH/NCI U54CA224081, NIH/NCI R01CA204302, NIH/NCI

R01CA211052, NIH/NCI R01CA169338, and the Pew-Stewart Foundations.

CR Angers S, 2009, NAT REV MOL CELL BIO, V10, P468, DOI 10.1038/nrm2717

Arnold M, 2015, GUT, V64, P381, DOI 10.1136/gutjnl-2014-308124

Bolger AM, 2014, BIOINFORMATICS, V30, P2114, DOI 10.1093/bioinformatics/btu170

Breitwieser FP, 2011, J PROTEOME RES, V10, P2758, DOI 10.1021/pr1012784

Cao W, 2015, ONCOGENESIS, V4, DOI 10.1038/oncsis.2015.34

Cao W, 2013, INT J GENOMICS, V2013, DOI 10.1155/2013/480534

Castro MAA, 2012, GENOME BIOL, V13, DOI 10.1186/gb-2012-13-4-r29

Chang J, 2017, NAT COMMUN, V8, DOI 10.1038/ncomms15290

Chase A, 2011, CLIN CANCER RES, V17, P2613, DOI 10.1158/1078-0432.CCR-10-2156

Chen HT, 2018, NAT GENET, V50, P1296, DOI 10.1038/s41588-018-0175-z

Chen XY, 2016, BIOINFORMATICS, V32, P1220, DOI 10.1093/bioinformatics/btv710

Chen Z, 2013, MOL CELL PROTEOMICS, V12, P2266, DOI 10.1074/mcp.M112.025817

Cibulskis K, 2013, NAT BIOTECHNOL, V31, P213, DOI 10.1038/nbt.2514

Corces MR, 2018, SCIENCE, V362, P420, DOI 10.1126/science.aav1898

Dunham I, 2012, NATURE, V489, P57, DOI 10.1038/nature11247

Feinberg AP, 2018, NEW ENGL J MED, V378, P1323, DOI [10.1056/NEJMra1402513, 10.1056/nejmra1402513]

Feinberg AP, 2016, NAT REV GENET, V17, P284, DOI 10.1038/nrg.2016.13

FEINBERG AP, 1983, NATURE, V301, P89, DOI 10.1038/301089a0

FROMMER M, 1992, P NATL ACAD SCI USA, V89, P1827, DOI 10.1073/pnas.89.5.1827

GAMASOSA MA, 1983, NUCLEIC ACIDS RES, V11, P6883, DOI 10.1093/nar/11.19.6883

Gao L, 2019, INT J ONCOL, V54, P1651, DOI 10.3892/ijo.2019.4758

Gao YB, 2014, NAT GENET, V46, P1097, DOI 10.1038/ng.3076

Gel B, 2016, BIOINFORMATICS, V32, P289, DOI 10.1093/bioinformatics/btv562

Gordon S, 2006, ONCOGENE, V25, P1125, DOI 10.1038/sj.onc.1209080

Hansen KD, 2014, GENOME RES, V24, P177, DOI 10.1101/gr.157743.113

Hansen KD, 2011, NAT GENET, V43, P768, DOI 10.1038/ng.865

Hu XW, 2019, J CELL PHYSIOL, V234, P10800, DOI 10.1002/jcp.27903

Hu YQ, 2011, CLIN EXP METASTAS, V28, P541, DOI 10.1007/s10585-011-9390-z

Kanehisa M, 2000, NUCLEIC ACIDS RES, V28, P27, DOI 10.1093/nar/28.1.27

Kim J, 2017, NATURE, V541, P169, DOI 10.1038/nature20805

Kishino T, 2016, MOL CARCINOGEN, V55, P2077, DOI 10.1002/mc.22452

Klaus A, 2008, NAT REV CANCER, V8, P387, DOI 10.1038/nrc2389

Kumagai N, 2013, MOL CLIN ONCOL, V1, P811, DOI 10.3892/mco.2013.142

Lachmann A, 2010, BIOINFORMATICS, V26, P2438, DOI 10.1093/bioinformatics/btq466

Lai ZW, 2016, NUCLEIC ACIDS RES, V44, DOI 10.1093/nar/gkw227

Landan G, 2012, NAT GENET, V44, P1207, DOI 10.1038/ng.2442

Landau DA, 2014, CANCER CELL, V26, P813, DOI 10.1016/j.ccell.2014.10.012

Layer RM, 2014, GENOME BIOL, V15, DOI 10.1186/gb-2014-15-6-r84

Li H, 2010, BIOINFORMATICS, V26, P589, DOI 10.1093/bioinformatics/btp698

Liang Y, 2018, CELL DEATH DIFFER, V25, P1980, DOI 10.1038/s41418-018-0084-9

Lin DC, 2014, NAT GENET, V46, P467, DOI 10.1038/ng.2935

Liu XS, 2016, CELL, V167, P233, DOI 10.1016/j.cell.2016.08.056

Ma P, 2017, BIOMED PHARMACOTHER, V87, P20, DOI 10.1016/j.biopha.2016.12.079

Mohiyuddin M, 2015, BIOINFORMATICS, V31, P2741, DOI 10.1093/bioinformatics/btv204

Murugaesu N, 2015, CANCER DISCOV, V5, P821, DOI 10.1158/2159-8290.CD-15-0412

Orgaz JL, 2014, NAT COMMUN, V5, DOI 10.1038/ncomms5255

Pan ZW, 2016, CANCER MED-US, V5, P2442, DOI 10.1002/cam4.770

Parkin DM, 2005, CA-CANCER J CLIN, V55, P74, DOI 10.3322/canjclin.55.2.74

Qin HD, 2016, AM J HUM GENET, V98, P709, DOI 10.1016/j.ajhg.2016.02.021

Saunders CT, 2012, BIOINFORMATICS, V28, P1811, DOI 10.1093/bioinformatics/bts271

Sheffield NC, 2017, NAT MED, V23, P386, DOI 10.1038/nm.4273

Slenter DN, 2018, NUCLEIC ACIDS RES, V46, pD661, DOI 10.1093/nar/gkx1064

Song YM, 2014, NATURE, V509, P91, DOI 10.1038/nature13176

Talevich E, 2018, CNVKIT RNA COPY NUMB, DOI [10.1101/408534v1, DOI 10.1101/408534V1]

Talevich E, 2016, PLOS COMPUT BIOL, V12, DOI 10.1371/journal.pcbi.1004873

Tarasov A, 2015, BIOINFORMATICS, V31, P2032, DOI 10.1093/bioinformatics/btv098

Timp W, 2013, NAT REV CANCER, V13, P497, DOI 10.1038/nrc3486

Tukey J., 1997, EXPLORATORY DATA ANA

Tungekar A, 2018, SCI REP-UK, V8, DOI 10.1038/s41598-018-30579-3

van Riggelen J, 2010, NAT REV CANCER, V10, P301, DOI 10.1038/nrc2819

Vidal E, 2017, ONCOGENE, V36, P5648, DOI 10.1038/onc.2017.176

Vojta A, 2016, NUCLEIC ACIDS RES, V44, P5615, DOI 10.1093/nar/gkw159

Wang JR, 2017, FRONT CELL NEUROSCI, V11, DOI 10.3389/fncel.2017.00329

Wen B, 2014, PROTEOMICS, V14, P2280, DOI 10.1002/pmic.201300361

Wu CS, 2016, J HEMATOL ONCOL, V9, DOI 10.1186/s13045-016-0349-z

Wu WQ, 2013, SCI REP-UK, V3, DOI 10.1038/srep01352

Wu YY, 2017, MOL CANCER, V16, DOI 10.1186/s12943-017-0715-7

Xia Y, 2017, BIOCHEM BIOPH RES CO, V489, P386, DOI 10.1016/j.bbrc.2017.05.136

Yang L, 2017, BMC GENOMICS, V18

Yang Y, 2019, CELL DEATH DIS, V10

Zhang L, 2015, AM J HUM GENET, V96, P597, DOI 10.1016/j.ajhg.2015.02.017

Zhang Y, 2008, GENOME BIOL, V9

Ziller MJ, 2013, NATURE, V500, P477, DOI 10.1038/nature12433

NR 73

TC 11

Z9 11

U1 4

U2 10

PU NATURE RESEARCH

PI BERLIN

PA HEIDELBERGER PLATZ 3, BERLIN, 14197, GERMANY

SN 2041-1723

J9 NAT COMMUN

JI Nat. Commun.

PD JUL 22

PY 2020

VL 11

IS 1

AR 3675

DI 10.1038/s41467-020-17227-z

PG 19

WC Multidisciplinary Sciences

WE Science Citation Index Expanded (SCI-EXPANDED)

SC Science & Technology - Other Topics

GA MU9HF

UT WOS:000555975700003

PM 32699215

OA Green Submitted, Green Published, gold

DA 2022-05-19

ER

PT J

AU Huang, B

Zhong, N

Xia, LL

Yu, GP

Cao, HB

AF Huang, Bin

Zhong, Ning

Xia, Lili

Yu, Guiping

Cao, Hongbao

TI Sparse Representation-Based Patient-Specific Diagnosis and Treatment for

Esophageal Squamous Cell Carcinoma

SO BULLETIN OF MATHEMATICAL BIOLOGY

LA English

DT Article

DE Esophageal squamous cell carcinoma; Sparse representation; Gene

selection

ID EXPRESSION; CANCER; INHIBITION; PROGNOSIS; RECEPTOR; LESIONS

AB Precision medicine and personalized treatment have attracted attention in recent years. However, most genetic medicines mainly target one genetic site, while complex diseases like esophageal squamous cell carcinoma (ESCC) usually present heterogeneity that involves variations of many genetic markers. Here, we seek an approach to leverage genetic data and ESCC knowledge data to forward personalized diagnosis and treatment for ESCC. First, 851 ESCC-related gene markers and their druggability were studied through a comprehensive literature analysis. Then, a sparse representation-based variable selection (SRVS) was employed for patient-specific genetic marker selection using gene expression datasets. Results showed that the SRVS method could identify a unique gene vector for each patient group, leading to significantly higher classification accuracies compared to randomly selected genes (100, 97.17, 100, 100%; permutation p values: 0.0032, 0.0008, 0.0004, and 0.0008). The SRVS also outperformed an ANOVA-based gene selection method in terms of the classification ratio. The patient-specific gene markers are targets of ESCC effective drugs, providing specific guidance for medicine selection. Our results suggest the effectiveness of integrating previous database utilizing SRVS in assisting personalized medicine selection and treatment for ESCC.

C1 [Huang, Bin; Yu, Guiping] Southeast Univ, Dept Cardiothorac Surg, Affiliated Jiangyin Hosp, Coll Med, 163 Shoushan Rd, Jiangyin 214400, Jiangsu, Peoples R China.

[Zhong, Ning] First Peoples Hosp Kunshan, Dept Cardiothorac Surg, Kunshan 215300, Jiangsu, Peoples R China.

[Xia, Lili] Peoples Hosp Tongling, Dept Ultrasound, Tongling 215300, Anhui, Peoples R China.

[Cao, Hongbao] Elsevier Inc, Dept Genom Res, R&D Solut, Rockville, MD 20852 USA.

[Cao, Hongbao] NIH, Unit Stat Genom, Bldg 10, Bethesda, MD 20892 USA.

RP Yu, GP (corresponding author), Southeast Univ, Dept Cardiothorac Surg, Affiliated Jiangyin Hosp, Coll Med, 163 Shoushan Rd, Jiangyin 214400, Jiangsu, Peoples R China.; Cao, HB (corresponding author), Elsevier Inc, Dept Genom Res, R&D Solut, Rockville, MD 20852 USA.; Cao, HB (corresponding author), NIH, Unit Stat Genom, Bldg 10, Bethesda, MD 20892 USA.

EM g.yu@gousinfo.com; h.cao@elsevier.com

FU Jiangsu Provincial Planning Commission; Wuxi City Health Planning

Commission [MS201625]; Fifth Provincial '333 Project' Research Project

FX We would like to thank Rachel Amey from University of Delaware for her

contribution to the English writing of the manuscript. This study is

partly supported by non-small cell lung cancer research funding, Jiangsu

Provincial Planning Commission; by clinical diagnosis and treatment of

small lung lesions and normative research, Wuxi City Health Planning

Commission, MS201625; and by 2017 Fifth Provincial '333 Project'

Research Project.

CR Abu-Farsakh S, 2017, BMC GASTROENTEROL, V17, DOI 10.1186/s12876-017-0590-0

Ando T, 2008, DIS ESOPHAGUS, V21, P15, DOI 10.1111/j.1442-2050.2007.00700.x

Cao HB, 2014, NEUROIMAGE, V102, P220, DOI 10.1016/j.neuroimage.2014.01.021

Chen X, 2019, BRIEF BIOINFORM, V20, P515, DOI 10.1093/bib/bbx130

Chen X, 2017, BRIEF BIOINFORM, V18, P558, DOI 10.1093/bib/bbw060

Chen X, 2018, CELL DEATH DIS, V9, DOI 10.1038/s41419-017-0003-x

Chen X, 2017, PLOS COMPUT BIOL, V13, DOI 10.1371/journal.pcbi.1005912

Chen X, 2013, BIOINFORMATICS, V29, P2617, DOI 10.1093/bioinformatics/btt426

Chen YB, 2017, INT J ONCOL, V50, P622, DOI 10.3892/ijo.2016.3823

Davis G, 1997, CONSTR APPROX, V13, P57, DOI 10.1007/s003659900033

Donoho DL, 2008, IEEE T INFORM THEORY, V54, P4789, DOI 10.1109/TIT.2008.929958

Fu L, 2007, CANCER-AM CANCER SOC, V110, P2672, DOI 10.1002/cncr.23110

Gan Si-Yuan, 2010, Chin J Cancer, V29, P300

Gao Y, 2011, CANCER EPIDEMIOL, V35, pE91, DOI 10.1016/j.canep.2011.06.006

Hara H, 2013, CANCER SCI, V104, P1455, DOI 10.1111/cas.12274

Hiratsuka J, 1988, Gan No Rinsho, V34, P807

Kaneko K, 2013, ONCOLOGY-BASEL, V84, P57, DOI 10.1159/000337981

Lam KY, 1997, INT J CANCER, V74, P212, DOI 10.1002/(SICI)1097-0215(19970422)74:2<212::AID-IJC13>3.0.CO;2-F

LAW SYK, 1992, SURG GYNECOL OBSTET, V175, P107

Lawrence MS, 2013, NATURE, V499, P214, DOI 10.1038/nature12213

Li JQ, 2017, ONCOTARGET, V8, P21187, DOI 10.18632/oncotarget.15061

Lorenzi PL, 2014, AUTOPHAGY, V10, P1316, DOI 10.4161/auto.28773

Lu YF, 2014, CSH PERSPECT MED, V4, DOI 10.1101/cshperspect.a008581

Nakakubo Y, 2002, CANCER LETT, V177, P101, DOI 10.1016/S0304-3835(01)00773-X

Narita M, 2015, INT J ONCOL, V46, P1699, DOI 10.3892/ijo.2015.2846

Ohashi S, 2015, GASTROENTEROLOGY, V149, P1700, DOI 10.1053/j.gastro.2015.08.054

Rosenfeld MR, 1997, J NATL CANCER I, V89, P381, DOI 10.1093/jnci/89.5.381

Roth MJ, 1997, CANCER-AM CANCER SOC, V80, P2047, DOI 10.1002/(SICI)1097-0142(19971201)80:11<2047::AID-CNCR3>3.0.CO;2-U

Shi HY, 2011, CANCER LETT, V309, P19, DOI 10.1016/j.canlet.2011.05.010

Shi ZX, 2015, TUMOR BIOL, V36, P8519, DOI 10.1007/s13277-015-3608-7

Stilgenbauer S, 2014, BLOOD, V123, P3247, DOI 10.1182/blood-2014-01-546150

Sun YL, 2014, CARCINOGENESIS, V35, P1941, DOI 10.1093/carcin/bgu043

Takikita M, 2009, BMC CANCER, V9, DOI 10.1186/1471-2407-9-310

Wang QF, 2013, WORLD J SURG ONCOL, V11, DOI 10.1186/1477-7819-11-278

Wang XX, 2017, ONCOTARGET, V8, P19661, DOI 10.18632/oncotarget.14914

Yang ZT, 2016, PLOS ONE, V11, DOI 10.1371/journal.pone.0145807

You ZH, 2017, PLOS COMPUT BIOL, V13, DOI 10.1371/journal.pcbi.1005455

Zhang WC, 2014, ANN THORAC SURG, V98, P513, DOI 10.1016/j.athoracsur.2014.03.015

NR 38

TC 0

Z9 0

U1 0

U2 2

PU SPRINGER

PI NEW YORK

PA 233 SPRING ST, NEW YORK, NY 10013 USA

SN 0092-8240

EI 1522-9602

J9 B MATH BIOL

JI Bull. Math. Biol.

PD AUG

PY 2018

VL 80

IS 8

BP 2124

EP 2136

DI 10.1007/s11538-018-0449-8

PG 13

WC Biology; Mathematical & Computational Biology

WE Science Citation Index Expanded (SCI-EXPANDED)

SC Life Sciences & Biomedicine - Other Topics; Mathematical & Computational

Biology

GA GM6PL

UT WOS:000438293400005

PM 29869044

DA 2022-05-19

ER

PT J

AU Zhang, Z

Wen, PH

Li, FF

Yao, CS

Wang, TF

Liang, B

Yang, QL

Ma, L

He, LM

AF Zhang, Zhen

Wen, Penghai

Li, Fangfang

Yao, Chuanshan

Wang, Tongfu

Liang, Bing

Yang, Qingle

Ma, Lei

He, Limin

TI HIPK2 inhibits cell metastasis and improves chemosensitivity in

esophageal squamous cell carcinoma

SO EXPERIMENTAL AND THERAPEUTIC MEDICINE

LA English

DT Article

DE esophageal squamous cell carcinoma; homeodomain-interacting protein

kinase-2; metastasis; chemoresistance

ID PROTEIN-KINASE 2; CANCER-CELLS; DRUG-RESISTANCE; PHOSPHORYLATION;

APOPTOSIS; CSCS; EMT

AB Esophageal squamous cell carcinoma (ESCC) is one of the most aggressive and lethal malignancies worldwide. At present, the underlying mechanisms of ESCC development and progression are poorly understood. Previous studies have demonstrated that homeodomain-interacting protein kinase-2 (HIPK2) serves an important role in cancer biology, particularly in proliferation and metastasis. However, the role of HIPK2 in ESCC cells remains unclear. In the current study, the expression of HIPK2 in ESCC specimens, adjacent non-cancerous tissues and cell lines was assessed using reverse transcription-quantitative polymerase chain reaction (RT-qPCR). The effects of HIPK2 on cell metastasis, epithelial-mesenchymal transition (EMT) and proliferation were studied using a Transwell assay, RT-qPCR and a Cell Counting Kit-8 assay, respectively. The results indicated that HIPK2 expression was downregulated in ESCC specimens and cell lines, and HIPK2 expression was associated with tumor invasion and lymph node metastasis. Functional studies demonstrated that HIPK2 overexpression inhibited cell metastasis and EMT. Furthermore, HIPK2 overexpression suppressed cell viability during cisplatin treatment. These results suggest that HIPK2 serves an important role in regulating metastasis and the chemosensitivity of ESCC cells, implicating the potential application of HIPK2 in ESCC therapy.

C1 [Zhang, Zhen; Li, Fangfang; Yao, Chuanshan; Wang, Tongfu; Liang, Bing; Yang, Qingle; Ma, Lei; He, Limin] First Peoples Hosp Nanyang, Dept Clin Oncol, 12 Renmin Rd, Nanyang 473000, Henan, Peoples R China.

[Wen, Penghai] Henan Univ, Affiliated Nanshi Hosp, Dept Clin Oncol, Nanyang 473000, Henan, Peoples R China.

RP Ma, L; He, LM (corresponding author), First Peoples Hosp Nanyang, Dept Clin Oncol, 12 Renmin Rd, Nanyang 473000, Henan, Peoples R China.

EM malei1225ch@163.com; helimin01ch@163.com

CR Akita H, 2006, J SURG ONCOL, V93, P401, DOI 10.1002/jso.20501

Ann EJ, 2016, CANCER RES, V76, P4728, DOI 10.1158/0008-5472.CAN-15-3310

Bon G, 2009, CANCER RES, V69, P5978, DOI 10.1158/0008-5472.CAN-09-0244

Brown LM, 2001, AM J EPIDEMIOL, V153, P114, DOI 10.1093/aje/153.2.114

Cheng Y, 2012, MOL MED REP, V5, P1251, DOI 10.3892/mmr.2012.810

Chung S, 2013, ONCOGENE, V32, P422, DOI 10.1038/onc.2012.65

D'Orazi G, 2006, CLIN CANCER RES, V12, P735, DOI 10.1158/1078-0432.CCR-05-1557

Di Stefano V, 2004, ONCOGENE, V23, P5185, DOI 10.1038/sj.onc.1207656

Han T, 2014, ONCOL REP, V32, P1133, DOI 10.3892/or.2014.3314

Hongo M, 2009, J GASTROEN HEPATOL, V24, P729, DOI 10.1111/j.1440-1746.2009.05824.x

Hosch SB, 2001, J CLIN ONCOL, V19, P1970, DOI 10.1200/JCO.2001.19.7.1970

Hsu WH, 2009, J GASTROINTEST SURG, V13, P1913, DOI 10.1007/s11605-009-0982-8

Jin YY, 2016, MOL CELL BIOCHEM, V422, P161, DOI 10.1007/s11010-016-2816-9

Jin YM, 2012, NAT MED, V18, P580, DOI 10.1038/nm.2685

Lagergren J, 2017, LANCET	0622

Lazzari C, 2011, ONCOGENE, V30, P4802, DOI 10.1038/onc.2011.182

Lin JW, 2014, PLOS ONE, V9, DOI 10.1371/journal.pone.0094040

Liu AB, 2017, MOL CANCER, V16, DOI 10.1186/s12943-017-0669-9

Livak KJ, 2001, METHODS, V25, P402, DOI 10.1006/meth.2001.1262

Mitra A, 2015, ONCOTARGET, V6, P10697, DOI 10.18632/oncotarget.4037

Morita M, 2010, INT J CLIN ONCOL, V15, P126, DOI 10.1007/s10147-010-0056-7

Nardinocchi L, 2009, PLOS ONE, V4, DOI 10.1371/journal.pone.0006819

Nodale C, 2012, CANCER BIOL THER, V13, DOI 10.4161/cbt.13.4.18694

Pennathur A, 2013, LANCET, V381, P400, DOI 10.1016/S0140-6736(12)60643-6

Pistritto G, 2007, CELL DEATH DIFFER, V14, P1837, DOI 10.1038/sj.cdd.4402186

Puca R, 2010, ONCOGENE, V29, P4378, DOI 10.1038/onc.2010.183

Puca R, 2008, GYNECOL ONCOL, V109, P403, DOI 10.1016/j.ygyno.2008.02.018

Qian X, 2014, ONCOGENE, V33, P3411, DOI 10.1038/onc.2013.310

Rice TW, 2010, ANN SURG ONCOL, V17, P1721, DOI 10.1245/s10434-010-1024-1

Shibue T, 2017, NAT REV CLIN ONCOL, V14, P611, DOI 10.1038/nrclinonc.2017.44

Tan MY, 2014, J CELL BIOCHEM, V115, P1762, DOI 10.1002/jcb.24842

Voulgari A, 2009, BBA-REV CANCER, V1796, P75, DOI 10.1016/j.bbcan.2009.03.002

Wang LD, 2010, NAT GENET, V42, P759, DOI 10.1038/ng.648

Yoshida T, 2009, ANTICANCER RES, V29, P1163

Zeisberg M, 2009, J CLIN INVEST, V119, P1429, DOI 10.1172/JCI36183

NR 35

TC 9

Z9 11

U1 0

U2 4

PU SPANDIDOS PUBL LTD

PI ATHENS

PA POB 18179, ATHENS, 116 10, GREECE

SN 1792-0981

EI 1792-1015

J9 EXP THER MED

JI Exp. Ther. Med.

PD JAN

PY 2018

VL 15

IS 1

BP 1113

EP 1118

DI 10.3892/etm.2017.5468

PG 6

WC Medicine, Research & Experimental

WE Science Citation Index Expanded (SCI-EXPANDED)

SC Research & Experimental Medicine

GA FV0WL

UT WOS:000424281600161

PM 29434701

OA gold, Green Published

DA 2022-05-19

ER

PT J

AU Ma, LF

Xiang, JQ

Zhang, YW

Hu, H

Shao, RR

Lin, D

AF Ma, Longfei

Xiang, Jiaqing

Zhang, Yawei

Hu, Hong

Shao, Rongrong

Lin, Dong

TI Characteristics and clinical significance of recurrent laryngeal nerve

lymph node metastasis in esophageal squamous cell carcinoma

SO JOURNAL OF BUON

LA English

DT Article

DE esophageal cancer; lymph node; metastasis; recurrent laryngeal nerve;

survival

ID DISSECTION; SURVIVAL

AB Purpose: The recurrent laryngeal nerve lymph nodes (RLN LNs) are among the most common metastatic sites in esophageal cancer, and the dissection of these lymph nodes (LNs) is considered beneficial. The purpose of this study was to evaluate the characteristics of RLN LN metastases from esophageal squamous cell carcinoma, and the effects of these metastases on the prognosis of patients. In addition, we aimed to determine the reasonable range of dissection of regional LNs.

Methods: The clinical data from 348 patients who underwent resection for esophageal carcinoma were retrospectively analyzed.

Results: Recurrent laryngeal nerve palsy occurred in 37.6% of the patients. In a subgroup of patients with lower esophageal tumors, cervical LN metastases were significantly more common in patients with positive rather than negative RLN LNs. The primary tumor site, tumor differentiation, and tumor invasion depth were factors that significantly influenced RLN LN metastasis. Multivariate analysis revealed that RLN LN metastasis was a significant factor associated with overall survival (OS) and disease-free survival (DFS) (p<0.001).

Conclusion: Metastasis to RLN LNs is a reliable indicator of cervical LN metastasis in middle/lower thoracic esophageal cancer. RLN LN metastasis may act as a prognostic indicator for patients with esophageal squamous cell carcinoma.

C1 [Ma, Longfei; Xiang, Jiaqing; Zhang, Yawei; Hu, Hong; Shao, Rongrong; Lin, Dong] Fudan Univ, Dept Thorac Surg, Shanghai Canc Ctr, 270 Dongan Rd, Shanghai 200032, Peoples R China.

RP Xiang, JQ (corresponding author), Fudan Univ, Dept Thorac Surg, Shanghai Canc Ctr, 270 Dongan Rd, Shanghai 200032, Peoples R China.

EM m263td@163.com

CR Altorki NK, 2008, ANN SURG, V248, P221, DOI 10.1097/SLA.0b013e31817bbe59

Chen WQ, 2015, CHIN J CANCER, V34, DOI 10.1186/s40880-015-0042-6

Cho JW, 2014, CLIN ENDOSC, V47, P523, DOI 10.5946/ce.2014.47.6.523

Hu Y, 2010, ANN SURG ONCOL, V17, P784, DOI 10.1245/s10434-009-0818-5

Jang HJ, 2011, ANN THORAC SURG, V92, P1091, DOI 10.1016/j.athoracsur.2011.03.093

Jemal A, 2011, CA-CANCER J CLIN, V61, P69, DOI [10.3322/caac.20107, 10.3322/caac.20115]

Jiao Wenpeng, 2015, Zhonghua Zhong Liu Za Zhi, V37, P899

Kosugi S, 2013, ANN SURG ONCOL, V20, P4016, DOI 10.1245/s10434-013-3141-0

Li HC, 2012, J SURG ONCOL, V105, P548, DOI 10.1002/jso.22148

Mizutani M, 2006, SURG RADIOL ANAT, V28, P333, DOI 10.1007/s00276-006-0115-y

Murakami Hirotomo, 2015, Gan To Kagaku Ryoho, V42, P1626

Nakashima Shingo, 2014, Gan To Kagaku Ryoho, V41, P2000

Ohashi S, 2015, GASTROENTEROLOGY, V149, P1700, DOI 10.1053/j.gastro.2015.08.054

Sato F, 2002, ANN SURG ONCOL, V9, P65, DOI 10.1245/aso.2002.9.1.65

Shiozaki H, 2001, DIS ESOPHAGUS, V14, P191, DOI 10.1046/j.1442-2050.2001.00206.x

Siegel RL, 2015, CA-CANCER J CLIN, V65, P5, DOI [10.3322/caac.21254, 10.1136/bmj.g1502, 10.1001/jamaoto.2014.2530, 10.3322/caac.21208]

Straatman J, 2016, DIS ESOPHAGUS, V29, P760, DOI 10.1111/dote.12405

Tabira Y, 2000, J AM COLL SURGEONS, V191, P232, DOI 10.1016/S1072-7515(00)00348-3

Taniyama Y, 2015, INTERACT CARDIOV TH, V20, P41, DOI 10.1093/icvts/ivu336

Ueda Y, 2006, ONCOL REP, V16, P1061

van der Schaaf M, 2015, JNCI-J NATL CANCER I, V107, DOI 10.1093/jnci/djv043

Wu J, 2014, BMC SURG, V14, DOI 10.1186/1471-2482-14-43

Ye K, 2014, GENET MOL RES, V13, P6411, DOI 10.4238/2014.August.25.4

NR 23

TC 11

Z9 12

U1 0

U2 1

PU IMPRIMATUR PUBLICATIONS

PI ATHENS

PA 30 DEM POLIORKETES ST, ATHENS, 136 76, GREECE

SN 1107-0625

EI 2241-6293

J9 J BUON

JI J. BUON

PD NOV-DEC

PY 2017

VL 22

IS 6

BP 1533

EP 1539

PG 7

WC Oncology

WE Science Citation Index Expanded (SCI-EXPANDED)

SC Oncology

GA FO8RE

UT WOS:000417154000025

PM 29332349

DA 2022-05-19

ER

PT J

AU Yi, YF

Lu, XJ

Chen, JM

Jiao, CJ

Zhong, J

Song, ZM

Yu, XP

Lin, BL

AF Yi, Yunfeng

Lu, Xiujuan

Chen, Jianming

Jiao, Changjie

Zhong, Jing

Song, Zhiming

Yu, Xiaoping

Lin, Baoli

TI Downregulated miR-486-5p acts as a tumor suppressor in esophageal

squamous cell carcinoma

SO EXPERIMENTAL AND THERAPEUTIC MEDICINE

LA English

DT Article

DE esophageal squamous cell carcinoma; miR-486-5p; downregulated; tumor

suppressor

ID MICRORNA EXPRESSION; CANCER; INVASION; PROLIFERATION; GROWTH;

IDENTIFICATION; PROGRESSION; MIGRATION; DIAGNOSIS; PROFILES

AB microRNAs (miRNAs/miRs) are crucial regulators of gene expression at the post-translational level through promoting mRNA degradation or the repression of translation of target genes. miRs have been confirmed to serve a dominant role in tumor biology. miR-486-5p has been ascertained to be involved in non-small-cell lung cancer, breast cancer and hepatocellular carcinoma; however, the expression and function of miR-486-5p in esophageal squamous cell carcinoma (ESCC) has yet to be elucidated. The present study aimed to analyze the expression levels of miR-486-5p in ESCC tissues and paired normal adjacent tissues, and determine the effects of miR-486-5p on esophageal cancer cells using MTT, wound scratch and apoptosis assays. The current results showed that miR-486-5p was significantly downregulated in ESCC specimens. Ectopic expression of miR-486-5p by synthetic mimics reduced cell proliferation and migration and induced increased cell apoptosis. The results indicated miR-486-5p may function as a tumor suppressor in ESCC. The present study demonstrated that miR-486-5p was downregulated in ESCC and served a anti-oncogene role in ESCC via affecting cellular migration.

C1 [Yi, Yunfeng; Lu, Xiujuan; Chen, Jianming; Jiao, Changjie; Zhong, Jing; Song, Zhiming; Yu, Xiaoping; Lin, Baoli] Xiamen Univ, Dongnan Affiliated Hosp, Dept Thorac Surg, 269 Zhanghuazhong Rd, Zhangzhou 363000, Fujian, Peoples R China.

RP Chen, JM (corresponding author), Xiamen Univ, Dongnan Affiliated Hosp, Dept Thorac Surg, 269 Zhanghuazhong Rd, Zhangzhou 363000, Fujian, Peoples R China.

EM chenjianming321@126.com

CR Akao Y, 2011, CANCER LETT, V300, P197, DOI 10.1016/j.canlet.2010.10.006

Bansal A, 2011, AM J GASTROENTEROL, V106, P1055, DOI 10.1038/ajg.2011.37

Bentwich I, 2005, NAT GENET, V37, P766, DOI 10.1038/ng1590

Bhattacharyya S, 2011, J BIOL CHEM, V286, P11604, DOI 10.1074/jbc.M110.198390

Bohanes P, 2012, J CLIN ONCOL, V30, P2265, DOI 10.1200/JCO.2011.38.8751

Enzinger PC, 2003, NEW ENGL J MED, V349, P2241, DOI 10.1056/NEJMra035010

Feber A, 2008, J THORAC CARDIOV SUR, V135, P255, DOI 10.1016/j.jtcvs.2007.08.055

Guo SQ, 2012, P NATL ACAD SCI USA, V109, P16636, DOI 10.1073/pnas.1213196109

Ha Tai-You, 2011, Immune Netw, V11, P135, DOI [10.4110/in.2011.11.1.11, 10.4110/in.2011.11.3.135]

Hiyoshi Y, 2009, CLIN CANCER RES, V15, P1915, DOI 10.1158/1078-0432.CCR-08-2545

Hong L, 2010, ANN SURG, V251, P1056, DOI 10.1097/SLA.0b013e3181dd4ea9

Huang XP, 2015, FEBS J, V282, P579, DOI 10.1111/febs.13167

Ito T, 2011, INT J CANCER, V129, P2134, DOI 10.1002/ijc.25874

Jiang L, 2010, BIOCHEM J, V432, P199, DOI 10.1042/BJ20100859

Jing Q, 2005, CELL, V120, P623, DOI 10.1016/j.cell.2004.12.038

Kano M, 2010, INT J CANCER, V127, P2804, DOI 10.1002/ijc.25284

Kollarova Helena, 2007, Biomedical Papers (Olomouc), V151, P17

Liu JG, 2012, EUR J CARDIO-THORAC, V41, pE7, DOI 10.1093/ejcts/ezr240

Liu Xiqiang, 2009, Cancer Genomics & Proteomics, V6, P131

Livak KJ, 2001, METHODS, V25, P402, DOI 10.1006/meth.2001.1262

Lucotti S, 2013, MOL CANCER, V12, DOI 10.1186/1476-4598-12-52

Ma WJ, 2011, MOL BIOL REP, V38, P3253, DOI 10.1007/s11033-010-0480-9

Mathe EA, 2009, CLIN CANCER RES, V15, P6192, DOI 10.1158/1078-0432.CCR-09-1467

Matsushima K, 2010, DIGESTION, V82, P138, DOI 10.1159/000310918

Ogawa R, 2009, MED MOL MORPHOL, V42, P102, DOI 10.1007/s00795-009-0443-1

Ragusa M, 2010, MOL CANCER THER, V9, P3396, DOI 10.1158/1535-7163.MCT-10-0137

Sarver AL, 2009, BMC CANCER, V9, DOI 10.1186/1471-2407-9-401

Shen J, 2011, BMC CANCER, V11, DOI 10.1186/1471-2407-11-374

Shibuya H, 2010, ONCOLOGY-BASEL, V79, P313, DOI 10.1159/000323283

Soeda S, 2013, INT J MOL MED, V31, P533, DOI 10.3892/ijmm.2013.1251

Steyerberg EW, 2007, J CLIN ONCOL, V25, P2389, DOI 10.1200/JCO.2006.09.7931

Tan XG, 2011, CLIN CANCER RES, V17, P6802, DOI 10.1158/1078-0432.CCR-11-0419

Tian YY, 2010, J BIOL CHEM, V285, P7986, DOI 10.1074/jbc.M109.062877

Tili E, 2011, P NATL ACAD SCI USA, V108, P4908, DOI 10.1073/pnas.1101795108

Tsuchiya S, 2011, J BIOL CHEM, V286, P420, DOI 10.1074/jbc.M110.170852

Wang J, 2014, ONCOGENE, V33, P1181, DOI 10.1038/onc.2013.42

Xiong Y, 2011, PLOS ONE, V6, DOI 10.1371/journal.pone.0024717

Zhai QN, 2012, BIOCHEM BIOPH RES CO, V419, P621, DOI 10.1016/j.bbrc.2012.02.060

Zhang GQ, 2014, TUMOR BIOL, V35, P11137, DOI 10.1007/s13277-014-2412-0

Zhao P, 2010, JPN J CLIN ONCOL, V40, P281, DOI 10.1093/jjco/hyp187

NR 40

TC 33

Z9 33

U1 0

U2 2

PU SPANDIDOS PUBL LTD

PI ATHENS

PA POB 18179, ATHENS, 116 10, GREECE

SN 1792-0981

EI 1792-1015

J9 EXP THER MED

JI Exp. Ther. Med.

PD NOV

PY 2016

VL 12

IS 5

BP 3411

EP 3416

DI 10.3892/etm.2016.3783

PG 6

WC Medicine, Research & Experimental

WE Science Citation Index Expanded (SCI-EXPANDED)

SC Research & Experimental Medicine

GA ED4ME

UT WOS:000388822000097

PM 27882172

OA Green Published, Green Submitted, gold

DA 2022-05-19

ER

PT J

AU Su, YY

Yin, LH

Liu, R

Sheng, JY

Yang, M

Wang, Y

Pan, EC

Guo, W

Pu, YP

Zhang, J

Liang, GY

AF Su, Yaoyao

Yin, Lihong

Liu, Ran

Sheng, JingYi

Yang, Miao

Wang, Yi

Pan, Enchun

Guo, Wei

Pu, Yuepu

Zhang, Juan

Liang, Geyu

TI Promoter methylation status of MGMT, hMSH2, and hMLH1 and its

relationship to corresponding protein expression and TP53 mutations in

human esophageal squamous cell carcinoma

SO MEDICAL ONCOLOGY

LA English

DT Article

DE Promoter methylation; TP53 mutation; Human esophageal squamous cell

carcinoma

ID CPG ISLAND HYPERMETHYLATION; TUMOR-SUPPRESSOR GENES; MISMATCH REPAIR;

COLORECTAL-CANCER; DNA-REPAIR; INDUCED APOPTOSIS; METHYLTRANSFERASE;

O-6-METHYLGUANINE; TUMORIGENESIS; INACTIVATION

AB To determine the relevance of O-6-methylguanine-DNA methyltransferase (MGMT), human mutS homolog 2 (hMSH2), and human mutL homolog 1 (hMLH1) in TP53 mutations in esophageal squamous cell carcinoma, we employed methylation-sensitive high-resolution melting technology and methylation-specific polymerase chain reaction (PCR) to analyze promoter hypermethylation of MGMT, hMSH2, and hMLH1, respectively, in 51 paired tumors and their adjacent normal tissues. The protein expression of the three proteins was also evaluated by Western blot analysis, and the PCR products of TP53, from exon 5 to exon 8, were directly sequenced to measure the mutation spectrum. Esophageal tumor tissues embraced statistically higher MGMT and hMSH2 promoter methylation level than normal tissue. The promoter methylation status of MGMT and hMSH2 corresponds positively with the protein expression level of MGMT and hMSH2. However, such relevance was not found for hMLH1. Furthermore, TP53 mutation status was well associated with MGMT and hMSH2 promoter methylation status, indicating that silencing of the two genes could lead to TP53 mutation in ESCC.

C1 [Su, Yaoyao; Yin, Lihong; Liu, Ran; Sheng, JingYi; Yang, Miao; Pu, Yuepu; Zhang, Juan; Liang, Geyu] Southeast Univ, Sch Publ Hlth, Minist Educ, Key Lab Environm Med Engn, Nanjing 210009, Jiangsu, Peoples R China.

[Wang, Yi; Pan, Enchun] Dis Control & Prevent Ctr Huaian, Huaian, Peoples R China.

[Guo, Wei] First Peoples Hosp Huaian, Huaian, Peoples R China.

RP Yin, LH (corresponding author), Southeast Univ, Sch Publ Hlth, Minist Educ, Key Lab Environm Med Engn, Nanjing 210009, Jiangsu, Peoples R China.

EM suyao@mergene.com; geyuliang_tb@163.com

FU National Natural Science Foundation of China [81072259, 30671732,

30800891]; Research Fund for the Doctoral Program of Higher Education of

China [200802861045]; Natural Science Foundation of JiangSu province of

China [BK2010407]; Research and Teaching Fund for the Excellent Youth

Scholars of Southeast University

FX This study was funded by the National Natural Science Foundation of

China (Nos. 81072259, 30671732, 30800891), Research Fund for the

Doctoral Program of Higher Education of China (No. 200802861045),

Natural Science Foundation of JiangSu province of China (No. BK2010407),

and Research and Teaching Fund for the Excellent Youth Scholars of

Southeast University (2009).

CR BLIN N, 1976, NUCLEIC ACIDS RES, V3, P2303, DOI 10.1093/nar/3.9.2303

Brock MV, 2003, CLIN CANCER RES, V9, P2912

de la Chapelle A, 2004, NAT REV CANCER, V4, P769, DOI 10.1038/nrc1453

Duckett DR, 1996, P NATL ACAD SCI USA, V93, P6443, DOI 10.1073/pnas.93.13.6443

Egger G, 2004, NATURE, V429, P457, DOI 10.1038/nature02625

Esteller M, 2002, ONCOGENE, V21, P5427, DOI 10.1038/sj.onc.1205600

Esteller M, 1999, CANCER RES, V59, P793

FEARON ER, 1990, CELL, V61, P759, DOI 10.1016/0092-8674(90)90186-I

Feinberg AP, 2006, NAT REV GENET, V7, P21, DOI 10.1038/nrg1748

Feinberg AP, 2004, NAT REV CANCER, V4, P143, DOI 10.1038/nrc1279

Fox EJ, 2006, J MOL DIAGN, V8, P68, DOI 10.2353/jmoldx.2006.050084

GALLOWAY SM, 1995, MUTAT RES LETT, V346, P231, DOI 10.1016/0165-7992(95)90040-3

Herman JG, 1998, P NATL ACAD SCI USA, V95, P6870, DOI 10.1073/pnas.95.12.6870

Herman JG, 1999, SEMIN CANCER BIOL, V9, P359, DOI 10.1006/scbi.1999.0138

Herman JG, 1996, P NATL ACAD SCI USA, V93, P9821, DOI 10.1073/pnas.93.18.9821

Kaina B, 2007, DNA REPAIR, V6, P1079, DOI 10.1016/j.dnarep.2007.03.008

KARRAN P, 1994, BIOESSAYS, V16, P833, DOI 10.1002/bies.950161110

Kawate H, 1998, P NATL ACAD SCI USA, V95, P5116, DOI 10.1073/pnas.95.9.5116

Kuester D, 2009, CANCER LETT, V275, P117, DOI 10.1016/j.canlet.2008.10.009

Leung SY, 1999, CANCER RES, V59, P159

Liu R., 2007, THESIS SE U

Lu SH, 1991, IARC SCI PUBL, V84, P538

Margison GP, 2002, MUTAGENESIS, V17, P483, DOI 10.1093/mutage/17.6.483

Martin P, 2006, MODERN PATHOL, V19, P914, DOI 10.1038/modpathol.3800590

Mukai T, 2002, ONCOGENE, V21, P9033, DOI 10.1038/sj.onc.1206095

Murata H, 2005, CANCER LETT, V223, P143, DOI 10.1016/j.canlet.2004.09.039

Nakagawachi T, 2003, ONCOGENE, V22, P8835, DOI 10.1038/sj.onc.1207183

Naumann SC, 2009, BRIT J CANCER, V100, P322, DOI 10.1038/sj.bjc.6604856

Nie Y, 2002, CARCINOGENESIS, V23, P1713, DOI 10.1093/carcin/23.10.1713

Ochs K, 2000, CANCER RES, V60, P5815

Poynter JN, 2008, CANCER EPIDEM BIOMAR, V17, P3208, DOI 10.1158/1055-9965.EPI-08-0512

ROWLEY JD, 1990, CANCER, V65, P2178, DOI 10.1002/1097-0142(19900515)65:10<2178::AID-CNCR2820651004>3.0.CO;2-#

Takagi Y, 2003, DNA REPAIR, V2, P1135, DOI 10.1016/S1568-7864(03)00134-4

TOORCHEN D, 1983, CARCINOGENESIS, V4, P1591, DOI 10.1093/carcin/4.12.1591

Tzao C, 2005, J THORAC CARDIOV SUR, V130, P1371, DOI 10.1016/j.jtcvs.2005.06.004

Wojdacz TK, 2007, NUCLEIC ACIDS RES, V35, P1

Wojdacz TK, 2008, NAT PROTOC, V3, P1903, DOI 10.1038/nprot.2008.191

WU Y, 1993, INT J CANCER, V54, P713, DOI 10.1002/ijc.2910540502

NR 38

TC 19

Z9 19

U1 1

U2 18

PU HUMANA PRESS INC

PI TOTOWA

PA 999 RIVERVIEW DRIVE SUITE 208, TOTOWA, NJ 07512 USA

SN 1357-0560

EI 1559-131X

J9 MED ONCOL

JI Med. Oncol.

PD FEB

PY 2014

VL 31

IS 2

AR 784

DI 10.1007/s12032-013-0784-4

PG 10

WC Oncology

WE Science Citation Index Expanded (SCI-EXPANDED)

SC Oncology

GA AB7NS

UT WOS:000331977900001

PM 24366688

DA 2022-05-19

ER

PT J

AU Yang, HP

Liu, JF

Rao, J

Zhang, XM

Qian, HL

Niu, XQ

Zhao, ZL

AF Yang, H. -P.

Liu, J. -F.

Rao, J.

Zhang, X. -M.

Qian, H. -L.

Niu, X. -Q.

Zhao, Z. -L.

TI Insulin-like growth factor binding protein-3 (IGFBP-3) genetic variant

and the risk of esophageal squamous cell carcinoma in a Chinese

population

SO GENETICS AND MOLECULAR RESEARCH

LA English

DT Article

DE Esophageal squamous cell carcinoma; IGFBP-3; Genetic polymorphism;

Molecular epidemiology

ID FACTOR PATHWAY GENES; CANCER-RISK; CIRCULATING LEVELS; IGF-I;

PROSTATE-CANCER; TEA DRINKING; ALCOHOL; TOBACCO; BREAST; POLYMORPHISMS

AB Insulin-like growth factor binding protein-3 (IGFBP-3) exerts anti-proliferative or pro-apoptotic effects through IGF-dependent as well as IGF-independent mechanisms in vitro. The purpose of this study was to examine the association between genetic variants in IGFBP-3 (rs2270628) and the risk of esophageal squamous cell carcinoma (ESCC) in a Chinese Han population. Five hundred ESCC cases and 500 cancer-free controls of the Chinese Han population were involved in this study. The IGFBP-3 single-nucleotide polymorphism (SNP) rs2270628 was genotyped and the estimated adjusted odds ratios (ORs) and 95% confidence intervals (CIs) for its association with the risk of ESCC were determined using unconditional logistic regression analysis. Compared with the rs2270628 CC genotype, TT genotype was associated with a significantly increased ESCC risk with OR (95% CI) of 2.07 (1.05-4.09), but CT genotype was not (OR = 1.25, 95% CI = 0.94-1.66). IGFBP-3 SNP rs2270628 may contribute to the risk of ESCC in the Chinese Han population.

C1 [Yang, H. -P.; Liu, J. -F.] Hebei Med Univ, Dept Thorac Surg, Hosp 4, Shijiazhuang, Hebei Province, Peoples R China.

[Rao, J.; Zhang, X. -M.] Hebei United Univ, Inst Mol Genet, Tangshang, Hebei Province, Peoples R China.

[Qian, H. -L.] Chinese Acad Med Sci, State Key Lab Mol Oncol, Canc Hosp Inst, Beijing 100730, Peoples R China.

[Niu, X. -Q.] Cixian Peoples Hosp, Dept Thorac Surg, Handan, Hebei Province, Peoples R China.

[Zhao, Z. -L.] Dalian Univ, Dept Cardiothorac, Zhongshan Hosp, Dalian 116012, Liaoning Provin, Peoples R China.

RP Liu, JF (corresponding author), Hebei Med Univ, Dept Thorac Surg, Hosp 4, Shijiazhuang, Hebei Province, Peoples R China.

EM junfengliucn@163.com

CR Baxter RC, 2000, AM J PHYSIOL-ENDOC M, V278, pE967, DOI 10.1152/ajpendo.2000.278.6.E967

Baxter RC, 2001, J CLIN PATHOL-MOL PA, V54, P145, DOI 10.1136/mp.54.3.145

Castellsague X, 2004, INT J CANCER, V108, P741, DOI 10.1002/ijc.11627

Chen WQ, 2013, J THORAC DIS, V5, P19, DOI 10.3978/j.issn.2072-1439.2013.01.04

Firth SM, 2002, ENDOCR REV, V23, P824, DOI 10.1210/er.2001-0033

Freedman ND, 2007, AM J EPIDEMIOL, V165, P1424, DOI 10.1093/aje/kwm051

Gallagher EJ, 2010, TRENDS ENDOCRIN MET, V21, P610, DOI 10.1016/j.tem.2010.06.007

Gu FY, 2010, CANCER EPIDEM BIOMAR, V19, P2877, DOI 10.1158/1055-9965.EPI-10-0507

Harrela M, 1996, J CLIN INVEST, V98, P2612, DOI 10.1172/JCI119081

Hiyama T, 2007, INT J CANCER, V121, P1643, DOI 10.1002/ijc.23044

Holmes MD, 2002, CANCER EPIDEM BIOMAR, V11, P862

Holmes RS, 2007, SEMIN RADIAT ONCOL, V17, P2, DOI 10.1016/j.semradonc.2006.09.003

Ishiguro S, 2009, CANCER LETT, V275, P240, DOI 10.1016/j.canlet.2008.10.020

Islami Farhad, 2009, BMJ, V338, pb929, DOI 10.1136/bmj.b929

Islami F, 2009, INT J CANCER, V125, P491, DOI 10.1002/ijc.24445

Jemal A, 2011, CA-CANCER J CLIN, V61, P69, DOI [10.3322/caac.20107, 10.3322/caac.20115]

Khandwala HM, 2000, ENDOCR REV, V21, P215, DOI 10.1210/er.21.3.215

Le Marchand L, 2005, CANCER EPIDEM BIOMAR, V14, P1319, DOI 10.1158/1055-9965.EPI-04-0847

Malaguarnera R, 2012, J ONCOL, V2012, DOI 10.1155/2012/635614

McElholm AR, 2010, GASTROENTEROLOGY, V139, P204, DOI 10.1053/j.gastro.2010.04.014

McGrath M, 2011, GYNECOL ONCOL, V120, P174, DOI 10.1016/j.ygyno.2010.10.012

Pearce CL, 2011, HUM MOL GENET, V20, P2263, DOI 10.1093/hmg/ddr087

Renehan AG, 2004, LANCET, V363, P1346, DOI 10.1016/S0140-6736(04)16044-3

Schumacher FR, 2010, HUM MOL GENET, V19, P3089, DOI 10.1093/hmg/ddq210

Sharp L, 2001, BRIT J CANCER, V85, P1667, DOI 10.1054/bjoc.2001.2147

Su XF, 2010, INT J CANCER, V126, P180, DOI 10.1002/ijc.24674

Tamimi RM, 2007, BREAST CANCER RES, V9, DOI 10.1186/bcr1655

Terry KL, 2009, CARCINOGENESIS, V30, P2042, DOI 10.1093/carcin/bgp257

Tsilidis KK, 2013, INT J CANCER, V133, P495, DOI 10.1002/ijc.28042

Wu IC, 2006, EUR J CLIN INVEST, V36, P236, DOI 10.1111/j.1365-2362.2006.01621.x

Wu M, 2009, INT J CANCER, V124, P1907, DOI 10.1002/ijc.24142

Xing DY, 2003, ONCOL REP, V10, P1615

Yu H, 2000, JNCI-J NATL CANCER I, V92, P1472, DOI 10.1093/jnci/92.18.1472

Zhao L, 2012, MED ONCOL, V29, P2669, DOI 10.1007/s12032-011-0133-4

Znaor A, 2003, INT J CANCER, V105, P681, DOI 10.1002/ijc.11114

NR 35

TC 8

Z9 8

U1 0

U2 4

PU FUNPEC-EDITORA

PI RIBEIRAO PRETO

PA RUA FLORIANO PEIXOTO 2444, ALTO DA BOA VISTA, RIBEIRAO PRETO, SP 00000,

BRAZIL

EI 1676-5680

J9 GENET MOL RES

JI Genet. Mol. Res.

PY 2014

VL 13

IS 2

BP 4146

EP 4153

DI 10.4238/2014.May.30.10

PG 8

WC Biochemistry & Molecular Biology; Genetics & Heredity

WE Science Citation Index Expanded (SCI-EXPANDED)

SC Biochemistry & Molecular Biology; Genetics & Heredity

GA AI3AC

UT WOS:000336729500189

PM 24938707

OA Bronze

DA 2022-05-19

ER

PT J

AU Zhang, YY

Shi, XD

Xie, XM

Laster, KV

Pang, MJ

Liu, KD

Hwang, J

Kim, DJ

AF Zhang, Yuanyuan

Shi, Xiaodan

Xie, Xiaomeng

Laster, Kyle Vaughn

Pang, Mengjun

Liu, Kangdong

Hwang, Joonsung

Kim, Dong Joon

TI Harmaline isolated from Peganum harmala suppresses growth of esophageal

squamous cell carcinoma through targeting mTOR

SO PHYTOTHERAPY RESEARCH

LA English

DT Article

DE cell proliferation; esophageal squamous cell carcinoma; Harmaline; mTOR;

patient-derived xenograft

ID RAPAMYCIN; ACTIVATION; PATHWAY; CANCER; EXPRESSION; EVEROLIMUS; KINASE

AB Harmaline is a naturally occurring beta-carboline alkaloid that is isolated from Peganum harmala. It has shown efficacy in treating Parkinson's disease and has been reported to exhibit antimicrobial and anticancer properties. However, the molecular mechanism of harmaline in the context of esophageal squamous cell carcinoma (ESCC) has not been characterized. Here, we report that harmaline attenuates ESCC growth by directly targeting the mammalian target of rapamycin (mTOR). Harmaline strongly reduced cell proliferation and anchorage-independent cell growth. Additionally, harmaline treatment induced G2/M phase cell-cycle arrest through upregulation of p27. The results of in vitro and cell-based assays showed that harmaline directly inhibited the activity of mTOR kinase and the phosphorylation of its downstream pathway components. Depletion of mTOR using an shRNA-mediated strategy in ESCC cell lines indicated that reduced mTOR protein expression levels are correlated with decreased cell proliferation. Additionally, we observed that the inhibitory effect of harmaline was dependent upon mTOR expression. Notably, oral administration of harmaline suppressed ESCC patient-derived tumor growth in vivo. Taken together, harmaline is a potential mTOR inhibitor that might be used for therapeutically treating ESCC.

C1 [Zhang, Yuanyuan; Xie, Xiaomeng; Pang, Mengjun; Liu, Kangdong; Kim, Dong Joon] Zhengzhou Univ, Sch Basic Med Sci, Pathophysiol Dept, Zhengzhou, Peoples R China.

[Zhang, Yuanyuan; Shi, Xiaodan; Xie, Xiaomeng; Laster, Kyle Vaughn; Pang, Mengjun; Liu, Kangdong; Kim, Dong Joon] China US Henan Hormel Canc Inst, 127 Dongming Rd, Zhengzhou 450008, Henan, Peoples R China.

[Liu, Kangdong] Zhengzhou Univ, Affiliated Canc Hosp, Zhengzhou, Peoples R China.

[Liu, Kangdong; Kim, Dong Joon] Collaborat Innovat Ctr Henan Prov Canc Chemopreve, Zhengzhou, Peoples R China.

[Liu, Kangdong] Int Joint Res Ctr Canc Chemoprevent, Zhengzhou, Peoples R China.

[Hwang, Joonsung] Korea Res Inst Biosci & Biotechnol KRIBB, Anticanc Agent Res Ctr, Cheongju 28116, Chungcheongbuk, South Korea.

RP Kim, DJ (corresponding author), China US Henan Hormel Canc Inst, 127 Dongming Rd, Zhengzhou 450008, Henan, Peoples R China.; Hwang, J (corresponding author), Korea Res Inst Biosci & Biotechnol KRIBB, Anticanc Agent Res Ctr, Cheongju 28116, Chungcheongbuk, South Korea.

EM hwangj1@kribb.re.kr; djkim@hci-cn.org

OI Kim, Dong Joon/0000-0001-6910-9213

FU National Natural Science Foundation China [81872335]; General project,

National Natural Science Foundation China (NSFC) [82103193]; Henan Joint

Fund, China [U1804196]; Youth Science Foundation of Natural Science

Foundation of Henan Province, China [212300410315]

FX General project, National Natural Science Foundation China, Grant/Award

Number: 81872335; General project, National Natural Science Foundation

China (NSFC), Grant/Award Number: 82103193; Henan Joint Fund, China,

Grant/Award Number: U1804196; Youth Science Foundation of Natural

Science Foundation of Henan Province, China, Grant/Award Number:

212300410315

CR Alijanpour S, 2020, PSYCHOPHARMACOLOGY, V237, P557, DOI 10.1007/s00213-019-05389-8

Bang YJ, 2010, LANCET, V376, P1302

Burnett PE, 1998, P NATL ACAD SCI USA, V95, P1432, DOI 10.1073/pnas.95.4.1432

Caron E, 2010, MOL SYST BIOL, V6, DOI 10.1038/msb.2010.108

Coqueret O, 2003, TRENDS CELL BIOL, V13, P65, DOI 10.1016/S0962-8924(02)00043-0

Ferlay J, 2015, INT J CANCER, V136, pE359, DOI 10.1002/ijc.29210

Frost P, 2004, BLOOD, V104, P4181, DOI 10.1182/blood-2004-03-1153

Fuchs CS, 2014, LANCET, V383, P31, DOI 10.1016/S0140-6736(13)61719-5

Heinrich M, 2020, J ETHNOPHARMACOL, V246, DOI 10.1016/j.jep.2019.112230

HEITMAN J, 1991, SCIENCE, V253, P905, DOI 10.1126/science.1715094

Hirashima K, 2012, BRIT J CANCER, V106, P876, DOI 10.1038/bjc.2012.36

Iranshahy M, 2019, AVICENNA J PHYTOMEDI, V9, P530, DOI 10.22038/AJP.2019.13382

Izzo AA, 2020, BRIT J PHARMACOL, V177, P2169, DOI 10.1111/bph.15054

Johnson SM, 2010, J AM COLL SURGEONS, V210, P767, DOI 10.1016/j.jamcollsurg.2009.12.008

Kashyap MK, 2018, MOL CANCER, V17, DOI 10.1186/s12943-018-0790-4

Kim LC, 2017, ONCOGENE, V36, P2191, DOI 10.1038/onc.2016.363

Kim SH, 2013, HUM PATHOL, V44, P226, DOI 10.1016/j.humpath.2012.05.011

Li SH, 2015, SURGERY, V157, P570, DOI 10.1016/j.surg.2014.10.014

Liu FF, 2019, CANCER PREV RES, V12, P849, DOI 10.1158/1940-6207.CAPR-19-0244

LU J, 2015, BIOMED RES INT, V2015, DOI DOI 10.1155/2015/417210

Mebius RE, 2005, NAT REV IMMUNOL, V5, P606, DOI 10.1038/nri1669

Moshiri M, 2013, AVICENNA J PHYTOMEDI, V3, P288

Murugan AK, 2019, SEMIN CANCER BIOL, V59, P92, DOI 10.1016/j.semcancer.2019.07.003

Naing A, 2012, BRIT J CANCER, V107, P1093, DOI 10.1038/bjc.2012.368

Nair Anroop B, 2016, J Basic Clin Pharm, V7, P27, DOI 10.4103/0976-0105.177703

Nasehi M, 2016, NEUROSCIENCE, V312, P1, DOI 10.1016/j.neuroscience.2015.11.004

O'Reilly KE, 2006, CANCER RES, V66, P1500, DOI 10.1158/0008-5472.CAN-05-2925

Ohtsu A, 2013, J CLIN ONCOL, V31, P3935, DOI 10.1200/JCO.2012.48.3552

Okada S, 2019, CELLS-BASEL, V8, DOI 10.3390/cells8080889

Pineiro-Carrero VM, 2004, PEDIATRICS, V113, P1097

Powles T, 2016, EUR UROL, V69, P450, DOI 10.1016/j.eururo.2015.08.035

Radi ZA, 2019, INT J TOXICOL, V38, P215, DOI 10.1177/1091581819831701

Rustgi AK, 2014, NEW ENGL J MED, V371, P2499, DOI 10.1056/NEJMra1314530

Sarbassov DD, 2004, CURR BIOL, V14, P1296, DOI 10.1016/j.cub.2004.06.054

Saxton RA, 2017, CELL, V168, P960, DOI [10.1016/j.cell.2017.02.004, 10.1016/j.cell.2017.03.035]

Siegel R, 2013, CA-CANCER J CLIN, V63, P11, DOI 10.3322/caac.21166

Tabrizizadeh M, 2018, PHYTOTHER RES, V32, P672, DOI 10.1002/ptr.6015

Wang CN, 2017, CHIN J CANCER, V36, DOI 10.1186/s40880-017-0232-5

Wang HT, 2019, INT J BIOL SCI, V15, P929, DOI 10.7150/ijbs.32489

Wang YH, 2015, SCI REP-UK, V5, DOI 10.1038/srep18613

Xu B, 2018, ONCOL LETT, V15, P1931, DOI 10.3892/ol.2017.7495

Yoon H.H., 2018, JAMA ONCOL, V4, P1

Zhao L, 2008, ONCOGENE, V27, P5486, DOI 10.1038/onc.2008.244

Zu XY, 2020, J EXP CLIN CANC RES, V39, DOI 10.1186/s13046-020-01608-9

Zu XY, 2019, PHYTOTHER RES, V33, P2337, DOI 10.1002/ptr.6415

NR 45

TC 0

Z9 0

U1 4

U2 8

PU WILEY

PI HOBOKEN

PA 111 RIVER ST, HOBOKEN 07030-5774, NJ USA

SN 0951-418X

EI 1099-1573

J9 PHYTOTHER RES

JI Phytother. Res.

PD NOV

PY 2021

VL 35

IS 11

BP 6377

EP 6388

DI 10.1002/ptr.7289

EA SEP 2021

PG 12

WC Chemistry, Medicinal; Pharmacology & Pharmacy

WE Science Citation Index Expanded (SCI-EXPANDED)

SC Pharmacology & Pharmacy

GA XB8LL

UT WOS:000697272400001

PM 34545650

DA 2022-05-19

ER

PT J

AU Wei, L

Wang, B

Hu, LW

Xu, Y

Li, ZD

Shen, Y

Huang, HR

AF Wei, Lei

Wang, Bo

Hu, Liwen

Xu, Yang

Li, Zhongdong

Shen, Yi

Huang, Hairong

TI MEX3A is upregulated in esophageal squamous cell carcinoma (ESCC) and

promotes development and progression of ESCC through targeting CDK6

SO AGING-US

LA English

DT Article

DE esophageal squamous cell carcinoma; MEX3A; CDK6; cell proliferation;

cell apoptosis

ID CANCER; THERAPY; AMPLIFICATION; MULTICENTER; PROTEINS; PHASE-3; MEX-3

AB Esophageal squamous cell carcinoma (ESCC) is one of the most commonly diagnosed malignant tumors worldwide and identified as a serious threat to human health. The role of MEX3A in ESCC remains unclear. In this study, we found that MEX3A was upregulated in tumor tissues of ESCC and positively associated with more advanced tumor stage, higher risk of lymphatic metastasis and poor prognosis. The downregulation of MEX3A in ESCC cell lines could induce inhibition of cell proliferation, colony formation, cell migration, and the promotion of cell apoptosis, while MEX3A overexpression exhibited opposite effects. In vivo experiments also verified the inhibition of ESCC induced by MEX3A knockdown. Moreover, we identified CDK6 as a potential target of MEX3A, which was also upregulated in ESCC. Further studies demonstrated that knockdown of CDK6 showed similar effects on the development of ESCC with MEX3A. More importantly, it was illustrated that CDK6 knockdown could alleviate the promotion effects of MEX3A overexpression on ESCC. In conclusion, MEX3A was identified as a tumor promotor in the development and progression of ESCC by targeting CDK6, which may be considered as a novel prognostic indicator and therapeutic target in treatment of ESCC.

C1 [Wei, Lei; Hu, Liwen; Xu, Yang; Li, Zhongdong; Shen, Yi; Huang, Hairong] Jinling Hosp, Dept Cardiothorac Surg, Nanjing 210002, Peoples R China.

[Wang, Bo] Nanjing Chest Hosp, Dept Thorac Surg, Nanjing 210029, Peoples R China.

RP Shen, Y; Huang, HR (corresponding author), Jinling Hosp, Dept Cardiothorac Surg, Nanjing 210002, Peoples R China.

EM dryishen@nju.edu.cn; huang_hai_rong_01@163.com

CR [Anonymous], 2017, LANCET, V389, P2350, DOI 10.1016/S0140-6736(17)31662-8

Baba Y, 2017, DIS ESOPHAGUS, V30, DOI 10.1111/dote.12482

Baba Y, 2014, CLIN CANCER RES, V20, P1114, DOI 10.1158/1078-0432.CCR-13-1645

Bray F, 2018, CA-CANCER J CLIN, V68, P394, DOI 10.3322/caac.21492

Buchet-Poyau K, 2007, NUCLEIC ACIDS RES, V35, P1289, DOI 10.1093/nar/gkm016

Chen XH, 2019, ONCOL REP, V41, P1627, DOI 10.3892/or.2018.6944

Arnal MJD, 2015, WORLD J GASTROENTERO, V21, P7933, DOI 10.3748/wjg.v21.i26.7933

Draper BW, 1996, CELL, V87, P205, DOI 10.1016/S0092-8674(00)81339-2

Dutton SJ, 2014, LANCET ONCOL, V15, P894, DOI 10.1016/S1470-2045(14)70024-5

Geng F, 2017, CANCER BIOTHER RADIO, V32, P149, DOI 10.1089/cbr.2017.2210

Han XH, 2017, MED SCI MONITOR, V23, DOI 10.12659/MSM.902645

Huang J, 2016, J THORAC ONCOL, V11, P910, DOI 10.1016/j.jtho.2016.02.020

Janni W, 2016, ONCOL RES TREAT, V39, P100, DOI 10.1159/000444685

Jiang H, 2012, MOL MED REP, V6, P575, DOI 10.3892/mmr.2012.943

Joazeiro CAP, 2000, CELL, V102, P549, DOI 10.1016/S0092-8674(00)00077-5

Kang XZ, 2015, WORLD J GASTROENTERO, V21, P7648, DOI 10.3748/wjg.v21.i25.7648

Kashyap MK, 2018, MOL CANCER, V17, DOI 10.1186/s12943-018-0790-4

Kojima T, 2017, CURR ONCOL REP, V19, DOI 10.1007/s11912-017-0590-9

Krepischi ACV, 2016, MOL CYTOGENET, V9, DOI 10.1186/s13039-016-0227-y

Lee YT, 2018, EUR J PHARMACOL, V834, P188, DOI 10.1016/j.ejphar.2018.07.034

Li JQ, 2017, ONCOTARGETS THER, V10, P3965, DOI 10.2147/OTT.S132756

Liu L, 2019, J CELL BIOCHEM, V120, P18736, DOI 10.1002/jcb.29186

Lyons TG, 2017, CHIN CLIN ONCOL, V6, DOI 10.21037/cco.2017.07.02

Marks EI, 2016, CURR CANCER DRUG TAR, V16, P53, DOI 10.2174/1568009615666150916092903

Miyamoto Y, 2017, INT J MOL SCI, V18, DOI 10.3390/ijms18040752

Ohashi S, 2015, GASTROENTEROLOGY, V149, P1700, DOI 10.1053/j.gastro.2015.08.054

Pennathur A, 2013, LANCET, V381, P400, DOI 10.1016/S0140-6736(12)60643-6

Pereira B, 2013, NUCLEIC ACIDS RES, V41, P3986, DOI 10.1093/nar/gkt087

Sharma P, 2017, ONCOL REP, V37, P3116, DOI 10.3892/or.2017.5546

Siegel RL, 2016, CA-CANCER J CLIN, V66, P7, DOI 10.3322/caac.21332

Smyth EC, 2017, NAT REV DIS PRIMERS, V3, DOI 10.1038/nrdp.2017.48

Suntharalingam M, 2017, JAMA ONCOL, V3, P1520, DOI 10.1001/jamaoncol.2017.1598

Tao H, 2018, J CELL BIOCHEM, V119, P6623, DOI 10.1002/jcb.26841

Valverde R, 2008, FEBS J, V275, P2712, DOI 10.1111/j.1742-4658.2008.06411.x

Vellayappan BA, 2017, FUTUR SCI OA, V3, DOI 10.4155/fsoa-2017-0050

Wang DG, 2018, INT J MOL MED, V41, P2473, DOI 10.3892/ijmm.2018.3460

Won E, 2014, CURR TREAT OPTION ON, V15, P395, DOI 10.1007/s11864-014-0292-6

Xue MH, 2019, CANCER CELL INT, V19, DOI 10.1186/s12935-019-0737-x

Yuan HY, 2018, EXP CELL RES, V367, P47, DOI 10.1016/j.yexcr.2018.03.017

Zeng R, 2018, INT J ONCOL, V52, P1443, DOI 10.3892/ijo.2018.4316

Zhang L, 2016, EXPERT REV GASTROENT, V10, P595, DOI 10.1586/17474124.2016.1140036

Zhao CH, 2016, ONCOTARGET, V7, P57310, DOI 10.18632/oncotarget.9809

NR 42

TC 5

Z9 5

U1 1

U2 4

PU IMPACT JOURNALS LLC

PI ORCHARD PARK

PA 6666 E QUAKER ST, STE 1, ORCHARD PARK, NY 14127 USA

SN 1945-4589

J9 AGING-US

JI Aging-US

PD NOV 14

PY 2020

VL 12

IS 21

BP 21091

EP 21113

PG 23

WC Cell Biology; Geriatrics & Gerontology

WE Science Citation Index Expanded (SCI-EXPANDED)

SC Cell Biology; Geriatrics & Gerontology

GA OS1NT

UT WOS:000589932800002

PM 33188661

OA gold, Green Published

DA 2022-05-19

ER

PT J

AU Zhu, XJ

Wang, JL

Li, LH

Deng, L

Wang, JT

Liu, L

Zeng, R

Wang, Q

Zheng, YF

AF Zhu, Xiongjie

Wang, Jiale

Li, Lihua

Deng, Lian

Wang, Jinting

Liu, Lu

Zeng, Rong

Wang, Qien

Zheng, Yanfang

TI GPX3 suppresses tumor migration and invasion via the FAK/AKT pathway in

esophageal squamous cell carcinoma

SO AMERICAN JOURNAL OF TRANSLATIONAL RESEARCH

LA English

DT Article

DE Esophageal squamous cell carcinoma; glutathione peroxidase 3; FAK/AKT

pathway; methylation; migration; invasion

ID GLUTATHIONE-PEROXIDASE 3; HEPATOCELLULAR-CARCINOMA;

CLINICAL-SIGNIFICANCE; DNA-DAMAGE; CANCER; EXPRESSION; TUMORIGENESIS;

METHYLATION; GROWTH; MATRIX-METALLOPROTEINASE-9

AB Although an increasing number of findings have proven that glutathione peroxidase 3 (GPX3) is methylated and down-regulated in various cancers, the underlying mechanism of its occurrence in esophageal squamous cell carcinoma (ESCC) remains unknown. In the present study, we found that the methylation rate in advanced cancers was significantly higher than that in early stage cancers by a methylation-specific polymerase chain reaction. Furthermore, the proliferation and migration capacities of KYSE-510 cells were inhibited after up-regulating GPX3 expression by GPX3 lentivirus transfection. As expected, the proliferation and migration capacities of KYSE-150 cells were promoted after down-regulating GPX3 expression with siRNA interfering. Moreover, we found that GPX3 might have deactivated the FAK/AKT signaling pathway to lower the expression of MMP-9 to suppress the migration and invasive capacities of KYSE-150 and KYSE-510 cells. Our findings suggested that GPX3 played a pivotal role in the suppression of carcinogenesis and progression in ESCC, and GPX3 has the potential as a novel biomarker in the diagnosis of ESCC.

C1 [Zhu, Xiongjie; Wang, Jiale; Li, Lihua; Deng, Lian; Wang, Jinting; Liu, Lu; Zeng, Rong; Zheng, Yanfang] Southern Med Univ, Dept Oncol, Zhujiang Hosp, Guangzhou 510282, Guangdong, Peoples R China.

[Wang, Qien] Ohio State Univ, Wexner Med Ctr, Div Radiobiol, Dept Radiol, Columbus, OH 43210 USA.

RP Zhu, XJ (corresponding author), Southern Med Univ, Dept Oncol, Zhujiang Hosp, Guangzhou 510282, Guangdong, Peoples R China.

EM 18665000236@163.com

OI Zheng, Yanfang/0000-0002-5591-6425

FU Natural Science Foundation of Guangdong Province [2015-A030313273];

Science and Technology Development Project of Southern Medical

University [C1033032]

FX This work is supported by Natural Science Foundation of Guangdong

Province (No. 2015-A030313273), and Science and Technology Development

Project of Southern Medical University (No. C1033032).

CR Agnani D, 2011, J OVARIAN RES, V4, DOI 10.1186/1757-2215-4-18

Barrett CW, 2013, CANCER RES, V73, P1245, DOI 10.1158/0008-5472.CAN-12-3150

Beraud C, 2015, INT J CANCER, V137, P1549, DOI 10.1002/ijc.29522

Cao SL, 2015, CLIN RES HEPATOL GAS, V39, P198, DOI 10.1016/j.clinre.2014.09.003

Chang SN, 2016, PROSTATE, V76, P1387, DOI 10.1002/pros.23223

Chen BS, 2011, CANCER LETT, V309, P37, DOI 10.1016/j.canlet.2011.05.013

Cooke MS, 2003, FASEB J, V17, P1195, DOI 10.1096/fj.02-0752rev

Cox G, 2000, CLIN CANCER RES, V6, P2349

Debelec-Butuner B, 2015, J INFLAMM-LOND, V12, DOI 10.1186/s12950-015-0057-4

Do Jee C, 2009, EUR J CANCER, V45, P1282, DOI 10.1016/j.ejca.2008.12.027

Dreher D, 1996, EUR J CANCER, V32A, P30, DOI 10.1016/0959-8049(95)00531-5

Du M, 2017, ANTICANCER DRUGS

Fan X, 2018, CLIN RADIOL, V73, P283, DOI 10.1016/j.crad.2017.10.013

He YL, 2011, DIGEST DIS SCI, V56, P681, DOI 10.1007/s10620-010-1369-0

Kanno SI, 2017, INT J MOL MED, V40, P748, DOI 10.3892/ijmm.2017.3049

Lee OJ, 2005, NEOPLASIA, V7, P854, DOI 10.1593/neo.05328

Li XF, 2014, PLOS ONE, V9, DOI 10.1371/journal.pone.0103162

Mohamed MM, 2014, OXID MED CELL LONGEV, V2014, DOI 10.1155/2014/787195

Peng DF, 2012, PLOS ONE, V7, DOI 10.1371/journal.pone.0046214

Qi X, 2016, THERANOSTICS, V6, P1934, DOI 10.7150/thno.16023

Qi X, 2014, ONCOTARGET, V5, P11103, DOI 10.18632/oncotarget.2549

Shang N, 2015, HEPATOLOGY, V61, P214, DOI 10.1002/hep.27402

Shin SY, 2017, ONCOTARGET, V8, P94591, DOI 10.18632/oncotarget.21813

Takebe G, 2002, J BIOL CHEM, V277, P41254, DOI 10.1074/jbc.M202773200

Yu YP, 2007, CANCER RES, V67, P8043, DOI 10.1158/0008-5472.CAN-07-0648

Zhang XL, 2014, ONCOL REP, V31, P2587, DOI 10.3892/or.2014.3152

Zhou JD, 2017, CANCER MED-US, V6, P267, DOI 10.1002/cam4.984

NR 27

TC 16

Z9 16

U1 0

U2 4

PU E-CENTURY PUBLISHING CORP

PI MADISON

PA 40 WHITE OAKS LN, MADISON, WI 53711 USA

SN 1943-8141

J9 AM J TRANSL RES

JI Am. J. Transl. Res.

PY 2018

VL 10

IS 6

BP 1908

EP 1920

PG 13

WC Oncology; Medicine, Research & Experimental

WE Science Citation Index Expanded (SCI-EXPANDED)

SC Oncology; Research & Experimental Medicine

GA GL8HJ

UT WOS:000437458800030

PM 30018730

DA 2022-05-19

ER

PT J

AU Li, ZW

Jin, H

Mao, GZ

Wu, LG

Guo, QW

AF Li, Zhiwei

Jin, Hui

Mao, Guozhang

Wu, Liuguang

Guo, Qingwei

TI Msi2 plays a carcinogenic role in esophageal squamous cell carcinoma via

regulation of the Wnt/beta-catenin and Hedgehog signaling pathways

SO EXPERIMENTAL CELL RESEARCH

LA English

DT Article

DE Esophageal squamous cell carcinoma; Msi2; Proliferation;

Epithelial-mesenchymal transition; Migration

ID RNA-BINDING PROTEIN; CHRONIC MYELOID-LEUKEMIA; HEPATOCELLULAR-CARCINOMA;

POOR-PROGNOSIS; STEM-CELLS; CANCER; ACTIVATION; MUSASHI2; PROGRESSION;

METASTASIS

AB Msi2 has been widely reported to be upregulated and strongly associated with fast progress and poor prognosis in many cancers. However, the expression and role of Msi2 in esophageal squamous cell carcinoma (ESCC) remain unknown. In this study, we found that Msi2 was upregulated in ESCC clinical samples, and was significantly associated with tumor size, differentiation status, and lymph node metastasis in ESCC patients. Multivariate Cox regression analysis showed that Msi2 was an independent predictor for disease-free survival (DFS) and overall survival (OS). Moreover, knockdown of Msi2 impaired ESCC cell proliferation, epithelial-mesenchymal transition (EMT) and migration, while overexpression of Msi2 promoted ESCC cell proliferation, EMT and migration in vitro. Animal experiments also confirmed that Msi2 promoted ESCC cell proliferation in vivo. Mechanistically, Msi2 promoted ESCC cell proliferation, EMT and migration via regulation of the Wnt/beta-catenin and Hedgehog (Hh) signaling pathways. Taken together, our study suggested that Msi2 could serve as a candidate for diagnosis and prognosis and as a potential therapeutic target in ESCC.

C1 [Li, Zhiwei; Jin, Hui; Mao, Guozhang; Wu, Liuguang; Guo, Qingwei] Zhoukou Cent Hosp, Dept Thorac Surg, 26 Renmin East Rd, Zhoukou 466000, Henan, Peoples R China.

RP Guo, QW (corresponding author), Zhoukou Cent Hosp, Dept Thorac Surg, 26 Renmin East Rd, Zhoukou 466000, Henan, Peoples R China.

EM Qingweiguo@126.com

CR Abdalla M., 2014, MSI2 DIRECTLY LINKED

Anastas JN, 2013, NAT REV CANCER, V13, P11, DOI 10.1038/nrc3419

Arnold M, 2014, GUT

Barbouti A, 2003, CANCER RES, V63, P1202

Byers RJ, 2011, BLOOD, V118, P2857, DOI 10.1182/blood-2011-04-346767

Clevers H, 2006, CELL, V127, P469, DOI 10.1016/j.cell.2006.10.018

Clevers H, 2012, CELL, V149, P1192, DOI 10.1016/j.cell.2012.05.012

Deng FY, 2015, INT J CLIN EXP PATHO, V8, P3045

Evangelista M, 2006, CLIN CANCER RES, V12, P5924, DOI 10.1158/1078-0432.CCR-06-1736

Ge CL, 2015, ONCOTARGET, V6, P10964, DOI 10.18632/oncotarget.3696

Good P, 1998, GENOMICS, V52, P382, DOI 10.1006/geno.1998.5456

Grunert S, 2003, NAT REV MOL CELL BIO, V4, P657, DOI 10.1038/nrm1175

He L, 2014, J CELL MOL MED, V18, P49, DOI 10.1111/jcmm.12158

Hope KJ, 2010, CELL STEM CELL, V7, P101, DOI 10.1016/j.stem.2010.06.007

Hu FQ, 2013, TUMOR BIOL, V34, P3893, DOI 10.1007/s13277-013-0977-7

Ingham PW, 2001, GENE DEV, V15, P3059, DOI 10.1101/gad.938601

Jiang J, 2008, DEV CELL, V15, P801, DOI 10.1016/j.devcel.2008.11.010

Kaeda J, 2015, LEUKEMIA LYMPHOMA, V56, P2105, DOI 10.3109/10428194.2014.981175

Kapinas K, 2010, J BIOL CHEM, V285, P25221, DOI 10.1074/jbc.M110.116137

Kudinov AE, 2016, P NATL ACAD SCI USA, V113, P6955, DOI 10.1073/pnas.1513616113

Li F, 2015, ONCOTARGET, V6, P6001, DOI 10.18632/oncotarget.3469

Ma XL, 2005, CARCINOGENESIS, V26, P1698, DOI 10.1093/carcin/bgi130

Mori Y., 2005, ACTIVATION HH SIGNAL

NAKAMURA M, 1994, NEURON, V13, P67, DOI 10.1016/0896-6273(94)90460-X

Nishimoto Y, 2010, CELL RES, V20, P1083, DOI 10.1038/cr.2010.122

Pennathur A, 2013, LANCET, V381, P400, DOI 10.1016/S0140-6736(12)60643-6

Reya T, 2005, NATURE, V434, P843, DOI 10.1038/nature03319

Sakakibara S, 2001, J NEUROSCI, V21, P8091, DOI 10.1523/JNEUROSCI.21-20-08091.2001

Schlabach MR, 2008, SCIENCE, V319, P620, DOI 10.1126/science.1149200

Wang MH, 2015, AM J CANCER RES, V5, P1089

Yang CL, 2016, LAB INVEST, V96, P950, DOI 10.1038/labinvest.2016.71

Yang L, 2010, ONCOGENE, V29, P469, DOI 10.1038/onc.2009.392

Yang Ling, 2012, Int J Biochem Mol Biol, V3, P46

Zhang HJ, 2014, EXP CELL RES, V320, P119, DOI 10.1016/j.yexcr.2013.09.009

Zhang YW, 2013, WORLD J GASTROENTERO, V19, P5598, DOI 10.3748/wjg.v19.i34.5598

Zong Z, 2016, CANCER MED-US, V5, P623, DOI 10.1002/cam4.624

NR 36

TC 19

Z9 20

U1 2

U2 6

PU ELSEVIER INC

PI SAN DIEGO

PA 525 B STREET, STE 1900, SAN DIEGO, CA 92101-4495 USA

SN 0014-4827

EI 1090-2422

J9 EXP CELL RES

JI Exp. Cell Res.

PD DEC 1

PY 2017

VL 361

IS 1

BP 170

EP 177

DI 10.1016/j.yexcr.2017.10.016

PG 8

WC Oncology; Cell Biology

WE Science Citation Index Expanded (SCI-EXPANDED)

SC Oncology; Cell Biology

GA FP5MG

UT WOS:000417663000020

PM 29054489

DA 2022-05-19

ER

PT J

AU Zhu, XZ

Zou, ST

Zhou, JD

Zhu, HS

Zhang, SY

Shang, ZF

Ding, WQ

Wu, JC

Chen, YH

AF Zhu, Xiaozhong

Zou, Shitao

Zhou, Jundong

Zhu, Hongsheng

Zhang, Shuyu

Shang, Zengfu

Ding, Wei-Qun

Wu, Jinchang

Chen, Yihong

TI REV3L, the catalytic subunit of DNA polymerase zeta, is involved in the

progression and chemoresistance of esophageal squamous cell carcinoma

SO ONCOLOGY REPORTS

LA English

DT Article

DE REV3L; esophageal squamous cell carcinoma; progression; chemoresistance;

5-fluorouracil

ID CANCER-CELLS; CYCLIN D1; POL-ZETA; EXPRESSION; 5-FLUOROURACIL;

MUTAGENESIS; RESISTANCE; PROGNOSIS; DISCOVERY; SURVIVIN

AB Protein reversionless 3-like (REV3L), the catalytic subunit of DNA polymerase (pol) zeta, is well known to participate in error-prone translesion synthesis (TLS) with less stringent and lower processivity. Recent evidence has demonstrated that REV3L is involved in carcinogenesis and tumor progression. However, the function of REV3L remains unclear in esophageal squamous cell carcinoma (ESCC). In the present study, we examined REV3L expression in ESCC tissues and its association with clinicopathological parameters. REV3L was found to be significantly upregulated and correlated with lymph node metastasis and clinical stage in the ESCC tissues. To further investigate the potential role of REV3L in esophageal cancer, stable ESCC cell lines with suppression of REV3L expression were established. Downregulation of REV3L expression led to a decrease in cell proliferation and invasive capacity partly through suppression of cyclin D1 and survivin expression, and an increase in cellular sensitivity to 5-fluorouracil (5-FU) by induction of G1 phase arrest and apoptosis. Therefore, REV3L plays an important role in ESCC progression and chemoresistance, and is a potential diagnostic marker and therapeutic target for ESCC.

C1 [Zhu, Xiaozhong; Zhou, Jundong; Wu, Jinchang] Nanjing Med Univ, Affiliated Suzhou Hosp, Dept Radiooncol, Suzhou 215001, Jiangsu, Peoples R China.

[Zou, Shitao; Zhou, Jundong] Nanjing Med Univ, Affiliated Suzhou Hosp, Suzhou Canc Ctr, Core Lab, Suzhou 215001, Jiangsu, Peoples R China.

[Zhu, Hongsheng] Nanjing Med Univ, Affiliated Suzhou Hosp, Dept Clin Lab, Suzhou 215001, Jiangsu, Peoples R China.

[Zhang, Shuyu; Shang, Zengfu] Soochow Univ, Coll Med, Sch Radiat Med & Protect, Suzhou 215123, Jiangsu, Peoples R China.

[Ding, Wei-Qun] Univ Oklahoma, Hlth Sci Ctr, Dept Pathol, Oklahoma City, OK 73104 USA.

[Chen, Yihong] Wannan Med Coll, Affiliated Hosp 1, Dept Radiooncol, Wuhu 241001, Anhui, Peoples R China.

RP Wu, JC (corresponding author), Nanjing Med Univ, Affiliated Suzhou Hosp, Dept Radiooncol, 16 West Baita Rd, Suzhou 215001, Jiangsu, Peoples R China.

EM wjinchang@sina.com; chenyihongf01@163.com

OI Shang, Zeng-Fu/0000-0002-2396-1416

FU National Natural Science Foundation of China [81372433, 81472919];

Natural Science Foundation of Jiangsu Province of China [BK20131149];

Suzhou Administration of Science and Technology [SYS201360, SYS201254,

SYS201571]

FX The present study was supported by the National Natural Science

Foundation of China (81372433 and 81472919), the Natural Science

Foundation of Jiangsu Province of China (BK20131149), and the Suzhou

Administration of Science and Technology (SYS201360, SYS201254 and

SYS201571).

CR Altieri DC, 2008, NAT REV CANCER, V8, P61, DOI 10.1038/nrc2293

Bijnsdorp IV, 2010, INT J CANCER, V126, P2457, DOI 10.1002/ijc.24943

Brondello JM, 2008, ONCOGENE, V27, P6093, DOI 10.1038/onc.2008.212

Diehl JA, 2002, CANCER BIOL THER, V1, P226, DOI 10.4161/cbt.72

Enzinger PC, 2003, NEW ENGL J MED, V349, P2241, DOI 10.1056/NEJMra035010

Friedberg EC, 2015, DNA REPAIR, V33, P35, DOI 10.1016/j.dnarep.2015.06.007

Gan GN, 2008, CELL RES, V18, P174, DOI 10.1038/cr.2007.117

Gibbs PEM, 1998, P NATL ACAD SCI USA, V95, P6876, DOI 10.1073/pnas.95.12.6876

Gueranger Q, 2008, DNA REPAIR, V7, P1551, DOI 10.1016/j.dnarep.2008.05.012

Hicks JK, 2010, MOL CELL BIOL, V30, P1217, DOI 10.1128/MCB.00993-09

Jares P, 2007, NAT REV CANCER, V7, P750, DOI 10.1038/nrc2230

Jemal A, 2009, CA-CANCER J CLIN, V59, P225, DOI 10.3322/caac.20006

Kato J, 2001, INT J CANCER, V95, P92

Kawamura K, 2001, INT J ONCOL, V18, P97

Knobel PA, 2011, NEOPLASIA, V13, P961, DOI 10.1593/neo.11828

PINEDO HM, 1988, J CLIN ONCOL, V6, P1653, DOI 10.1200/JCO.1988.6.10.1653

Sarbia M, 1999, INT J CANCER, V84, P86, DOI 10.1002/(SICI)1097-0215(19990219)84:1<86::AID-IJC16>3.3.CO;2-Z

Talukdar FR, 2013, PLOS ONE, V8, DOI 10.1371/journal.pone.0060996

Thomas GR, 2005, INT J EXP PATHOL, V86, P347, DOI 10.1111/j.0959-9673.2005.00447.x

Varadi V, 2011, BREAST CANCER RES TR, V129, P235, DOI 10.1007/s10549-011-1460-z

Wang HB, 2009, NEURO-ONCOLOGY, V11, P790, DOI 10.1215/15228517-2009-015

Wang L, 2014, CANCER CELL INT, V14, DOI 10.1186/s12935-014-0071-2

Waters LS, 2009, MICROBIOL MOL BIOL R, V73, P134, DOI 10.1128/MMBR.00034-08

Xiao W, 1998, CARCINOGENESIS, V19, P945, DOI 10.1093/carcin/19.5.945

Yang GQ, 2013, MED ONCOL, V30, DOI [10.1007/s12032-013-0697-2, 10.1007/s12032-013-0500-4]

Yang L, 2015, PLOS ONE, V10, DOI 10.1371/journal.pone.0120334

Zhang S, 2013, ONCOGENE, V32, P242, DOI 10.1038/onc.2012.32

Zhou JD, 2012, CANCER SCI, V103, P1574, DOI 10.1111/j.1349-7006.2012.02309.x

NR 28

TC 12

Z9 15

U1 0

U2 7

PU SPANDIDOS PUBL LTD

PI ATHENS

PA POB 18179, ATHENS, 116 10, GREECE

SN 1021-335X

EI 1791-2431

J9 ONCOL REP

JI Oncol. Rep.

PD MAR

PY 2016

VL 35

IS 3

BP 1664

EP 1670

DI 10.3892/or.2016.4549

PG 7

WC Oncology

WE Science Citation Index Expanded (SCI-EXPANDED)

SC Oncology

GA DB0WP

UT WOS:000368228900051

PM 26752104

OA Bronze

DA 2022-05-19

ER

PT J

AU Ji, CH

Liu, H

Xiang, M

Liu, JJ

Yue, FT

Wang, WQ

Chu, X

AF Ji, Chunhua

Liu, Hui

Xiang, Ming

Liu, Junjun

Yue, Fengtao

Wang, Weiqing

Chu, Xiao

TI Deregulation of decorin and FHL1 are associated with esophageal squamous

cell carcinoma progression and poor prognosis

SO INTERNATIONAL JOURNAL OF CLINICAL AND EXPERIMENTAL MEDICINE

LA English

DT Article

DE Decorin; FHL1; prognosis; esophageal squamous cell carcinoma; cancer

biomarker

ID CLINICAL-SIGNIFICANCE; EXPRESSION; GROWTH; CANCER; INHIBITION; MIGRATION

AB To investigate the expression of FHL1 and Decorin in esophageal squamous cell carcinoma (ESCC) and its clinical significance. 82 ESCC tissues were evaluated by immunohistochemistry for the expression of FHL1 and Decorin. The role of the expression of FHL1 and Decorin in ESCC were statistically analyzed. The expression level of FHL1 and Decorin were reduced in malignant tissue samples in comparison to normal matched tissue (P < 0.05). It was also proved that the positive expressions of FHL1 and Decorin were associated with ESCC histological grade, lymph node metastasis, tumor stage and clinical stage (P < 0.05). In addition, the Kaplan-Meier survival curves revealed that the positive expressions of FHL1 and Decorin were associated with favorable prognosis in ESCC patients. Multivariate analysis showed that the positive expressions of FHL1 and Decorin were independent prognostic markers of overall survival of ESCC patients, respectively. The positive expressions of FHL1 and Decorin were associated with ESCC progression and good prognosis. Our results indicate that the positive expressions of FHL1 and Decorin were independent prognostic factors for patients with esophageal cancer, which might be potential valuable biomarkers for ESCC.

C1 [Ji, Chunhua; Liu, Hui; Xiang, Ming; Liu, Junjun; Yue, Fengtao; Wang, Weiqing; Chu, Xiao] Fudan Univ, Peoples Hosp Shanghai 5, Dept Thorac Surg, Heqing Rd 801, Shanghai 200240, Peoples R China.

RP Liu, H (corresponding author), Fudan Univ, Peoples Hosp Shanghai 5, Dept Thorac Surg, Heqing Rd 801, Shanghai 200240, Peoples R China.

EM huiliu2015@sina.com

FU Scientific Research Fund from The Fifth People's Hospital [2013WYYJ03]

FX This study was financially supported by the Scientific Research Fund

from The Fifth People's Hospital affiliated to Fudan University

(Shanghai, China) (2013WYYJ03).

CR Baghy K, 2012, J HISTOCHEM CYTOCHEM, V60, P262, DOI 10.1369/0022155412438104

Bi XL, 2013, CHIN J CANCER, V32, P266, DOI 10.5732/cjc.012.10301

Bi XL, 2012, CARCINOGENESIS, V33, P326, DOI 10.1093/carcin/bgr293

Buraschi S, 2012, PLOS ONE, V7, DOI 10.1371/journal.pone.0045559

Dil N, 2011, HEAD NECK ONCOL, V3, DOI 10.1186/1758-3284-3-44

Ding LH, 2011, J CELL MOL MED, V15, P72, DOI 10.1111/j.1582-4934.2009.00938.x

Horvath Z, 2014, MATRIX BIOL, V35, P194, DOI 10.1016/j.matbio.2013.11.004

Hu YP, 2009, NEOPLASIA, V11, P1042, DOI 10.1593/neo.09760

Jemal A, 2011, CA-CANCER J CLIN, V61, P69, DOI [10.3322/caac.20107, 10.3322/caac.20115]

Lin YS, 2013, J EPIDEMIOL, V23, P233, DOI 10.2188/jea.JE20120162

Niu C, 2012, INT J CANCER, V130, P2549, DOI 10.1002/ijc.26259

Sakashita K, 2008, ANN SURG ONCOL, V15, P2293, DOI 10.1245/s10434-008-9904-3

Santra M, 2000, J BIOL CHEM, V275, P35153, DOI 10.1074/jbc.M006821200

Walker RA, 2006, HISTOPATHOLOGY, V49, P406, DOI 10.1111/j.1365-2559.2006.02514.x

Xu YY, 2015, BMC CANCER, V15, DOI 10.1186/s12885-014-1001-3

Xu YH, 2012, MOL CELL BIOCHEM, V363, P93, DOI 10.1007/s11010-011-1161-2

Yang JC, 2000, CIRCULATION, V102, P3046

Yao WJ, 2014, INT J CLIN EXP PATHO, V7, P7158

Zhang BJ, 2014, INT J CLIN EXP PATHO, V7, P6213

NR 19

TC 12

Z9 12

U1 0

U2 3

PU E-CENTURY PUBLISHING CORP

PI MADISON

PA 40 WHITE OAKS LN, MADISON, WI 53711 USA

SN 1940-5901

J9 INT J CLIN EXP MED

JI Int. J. Clin. Exp. Med.

PY 2015

VL 8

IS 11

BP 20965

EP 20970

PG 6

WC Medicine, Research & Experimental

WE Science Citation Index Expanded (SCI-EXPANDED)

SC Research & Experimental Medicine

GA DF0FA

UT WOS:000371013200137

PM 26885026

DA 2022-05-19

ER

PT J

AU Wang, XS

Luo, KJ

Bella, AE

Bu, SS

Wen, J

Zhang, SS

Hu, Y

AF Wang, Xiu-Shen

Luo, Kong-Jia

Bella, Amos Ela

Bu, Shan-Shan

Wen, Jing

Zhang, Shui-Shen

Hu, Yi

TI Caspase-3 expression in metastatic lymph nodes of esophageal squamous

cell carcinoma is prognostic of survival

SO WORLD JOURNAL OF GASTROENTEROLOGY

LA English

DT Article

DE Esophageal squamous cell carcinoma; Caspase-3 protein; prognosis; Lymph

node

ID GROWTH-FACTOR; CANCER STATISTICS; LUNG-CANCER; E-CADHERIN; APOPTOSIS;

PROTEIN; LESIONS; EGFR; SITES; P53

AB AIM: To assess whether differential expression of caspase-3 in paired metastatic lymph nodes (LNs) is prognostic of survival in patients with resectable esophageal squamous cell carcinoma (ESCC).

METHODS: Capases-3 expression was evaluated immunohistochemically in 122 pairs of primary ESCCs and regional metastatic LNs assembled on tissue microarrays. The impact of caspase-3 expression on survival outcomes was analyzed by the Kaplan-Meier method and Cox proportional hazards regression model.

RESULTS: The level of caspase-3 expression was significantly higher in LN metastases than in primary tumors (P < 0.001). Caspase-3 expression in the primary tumors was associated with longer median survival (23 mo vs 21 mo, P = 0.033), whereas higher expression in paired metastatic LNs was associated with shorter median survival (20 mo vs 22 mo, P = 0.043). Multivariate analysis showed that both were independent prognostic factors.

CONCLUSION: Caspase-3 expression in metastatic LNs may be a potential independent predictor of poorer overall survival in patients with resected ESCC and LN metastasis. Protein expression in metastatic tumors may be a biomarker prognostic of survival. (C) 2014 Baishideng Publishing Group Co., Limited. All rights reserved.

C1 [Wang, Xiu-Shen; Bu, Shan-Shan] Zhengzhou Univ, Canc Hosp, Dept Radiat Oncol, Zhengzhou 450008, Henan Province, Peoples R China.

[Luo, Kong-Jia; Bella, Amos Ela; Wen, Jing; Zhang, Shui-Shen; Hu, Yi] Sun Yat Sen Univ, State Key Lab Oncol South China, Ctr Canc, Guangzhou 510080, Guangdong, Peoples R China.

[Luo, Kong-Jia; Bella, Amos Ela; Wen, Jing; Zhang, Shui-Shen; Hu, Yi] Sun Yat Sen Univ, Dept Thorac Oncol, Ctr Canc, Guangzhou 510080, Guangdong, Peoples R China.

[Luo, Kong-Jia; Bella, Amos Ela; Wen, Jing; Zhang, Shui-Shen; Hu, Yi] Guangdong Esophageal Canc Res Inst, Guangzhou 510080, Guangdong, Peoples R China.

RP Hu, Y (corresponding author), Sun Yat Sen Univ, Dept Thorac Oncol, Ctr Canc, 651 Dong Feng Rd East, Guangzhou 510080, Guangdong, Peoples R China.

EM huyi@sysucc.org.cn

CR Abdul-Ghani M, 2008, CELL STEM CELL, V2, P515, DOI 10.1016/j.stem.2008.05.013

Bellini MF, 2010, ANTICANCER RES, V30, P2845

Cao Fu-min, 2005, Zhonghua Zhongliu Zazhi, V27, P416

Ferlay J, 2010, INT J CANCER, V127, P2893, DOI 10.1002/ijc.25516

FIDLER IJ, 1982, SCIENCE, V217, P998, DOI 10.1126/science.7112116

FRISCH SM, 1994, J CELL BIOL, V124, P619, DOI 10.1083/jcb.124.4.619

Fujita J, 2008, CELL STEM CELL, V2, P595, DOI 10.1016/j.stem.2008.04.001

Glinsky GV, 1996, CANCER LETT, V101, P43, DOI 10.1016/0304-3835(96)04112-2

Gomez-Roca C, 2009, J THORAC ONCOL, V4, P1212, DOI 10.1097/JTO.0b013e3181b44321

Hsia JY, 2003, EUR J SURG ONCOL, V29, P44, DOI 10.1053/ejso.2002.1338

Italiano A, 2006, ANN ONCOL, V17, P981, DOI 10.1093/annonc/mdl038

Italiano A, 2005, ANN ONCOL, V16, P1503, DOI 10.1093/annonc/mdi282

Janzen V, 2008, CELL STEM CELL, V2, P584, DOI 10.1016/j.stem.2008.03.012

Jemal A, 2010, CA-CANCER J CLIN, V60, P277, DOI 10.3322/caac.20073

Jiang H, 2010, DIS ESOPHAGUS, V23, P487, DOI 10.1111/j.1442-2050.2009.01043.x

Kononen J, 1998, NAT MED, V4, P844, DOI 10.1038/nm0798-844

Kurabayashi A, 2001, MODERN PATHOL, V14, P741, DOI 10.1038/modpathol.3880383

Luo KJ, 2011, J SURG ONCOL, V104, P236, DOI 10.1002/jso.21921

NICHOLSON DW, 1995, NATURE, V376, P37, DOI 10.1038/376037a0

Oudejans JJ, 2005, MODERN PATHOL, V18, P877, DOI 10.1038/modpathol.3800398

Parkin DM, 2005, CA-CANCER J CLIN, V55, P74, DOI 10.3322/canjclin.55.2.74

POSTE G, 1980, NATURE, V283, P139, DOI 10.1038/283139a0

Rice TW, 2010, ANN SURG ONCOL, V17, P1721, DOI 10.1245/s10434-010-1024-1

Sato F, 1999, BRIT J CANCER, V80, P1366, DOI 10.1038/sj.bjc.6690530

Takaoka A, 1997, ONCOGENE, V14, P2971, DOI 10.1038/sj.onc.1201147

Takayama N, 2003, ANTICANCER RES, V23, P4435

Tsutsui S, 2002, EUR J SURG ONCOL, V28, P383, DOI 10.1053/ejso.2002.1259

Wang LD, 1999, ANTICANCER RES, V19, P369

WEISS L, 1983, BRIT J CANCER, V47, P81, DOI 10.1038/bjc.1983.10

NR 29

TC 5

Z9 5

U1 0

U2 4

PU BAISHIDENG PUBLISHING GROUP INC

PI PLEASANTON

PA 8226 REGENCY DR, PLEASANTON, CA 94588 USA

SN 1007-9327

EI 2219-2840

J9 WORLD J GASTROENTERO

JI World J. Gastroenterol.

PD APR 21

PY 2014

VL 20

IS 15

BP 4414

EP 4420

DI 10.3748/wjg.v20.i15.4414

PG 7

WC Gastroenterology & Hepatology

WE Science Citation Index Expanded (SCI-EXPANDED)

SC Gastroenterology & Hepatology

GA AF3HQ

UT WOS:000334603000032

PM 24764681

OA Green Published, hybrid

DA 2022-05-19

ER

PT J

AU Xiong, G

Huang, ZY

Cai, KC

Cai, RJ

Zhang, J

Wang, WJ

AF Xiong, Gang

Huang, Zhi-yong

Cai, Kai-chan

Cai, Ruijing

Zhang, Jun

Wang, Wu-jun

TI DIFFERENTIALLY EXPRESSED PROTEINS BETWEEN ESOPHAGEAL SQUAMOUS CELL

CARCINOMA AND ADJACENT NORMAL ESOPHAGEAL TISSUE

SO JOURNAL OF MEDICAL BIOCHEMISTRY

LA English

DT Article

DE esophageal squamous cell carcinoma; proteomics; differentially expressed

proteins; immunohistochemistry

ID PROTEOMIC ANALYSIS; TRIOSEPHOSPHATE ISOMERASE; LUNG ADENOCARCINOMA;

CANCER CELLS; ANNEXIN 1; ANTIGEN; IDENTIFICATION; TUMORIGENESIS;

INHIBITORS; SILVER

AB Proteomics was employed to identify the differentially expressed proteins between esophageal squamous cell carcinoma (ESCC) and adjacent normal esophageal tissues. ESCC tissues and adjacent normal tissues were obtained from 10 patients with ESCC and the proteins were extracted and subjected to two-dimensional gel electrophoresis (2-DE). The differentially expressed proteins were identified after image analysis, and matrix assisted laser desorption ionisation time-of-flight mass spectrometry (MALDI-TOF-MS) was used to confirm these proteins. Immunohistochemistry was then performed to detect the expressions of HSP27 and ANX1 in ESCC tissues and adjacent normal tissues. A total of 6 differentially expressed proteins were identified by peptide mass fingerprinting, among which SCCA1, KRT4 and ANX1 were down-regulated and TIM1, MnSOD and HSP27 up-regulated in the ESCC. Immuno histochemistry showed HSP27 was highly expressed in the ESCC which, however, had a low expression of ANX1. These findings were consistent with those in proteomics. There were differentially expressed proteins between ESCC and adjacent normal tissues. The investigation of differentially expressed proteins between ESCC and normal esophageal tissue may provide evidence for the molecular pathogenesis of ESCC.

C1 [Xiong, Gang; Huang, Zhi-yong; Cai, Kai-chan; Cai, Ruijing; Zhang, Jun; Wang, Wu-jun] So Med Univ, Nanfang Hosp, Dept Thorac Surg, Guangzhou 510515, Guangdong, Peoples R China.

RP Huang, ZY (corresponding author), So Med Univ, Nanfang Hosp, Dept Thorac Surg, Guangzhou 510515, Guangdong, Peoples R China.

EM huanghuang2000@163.com

CR Bai XF, 2004, WORLD J GASTROENTERO, V10, P1466

BANKSSCHLEGEL SP, 1983, EXP CELL RES, V146, P271, DOI 10.1016/0014-4827(83)90129-5

Candiano G, 2004, ELECTROPHORESIS, V25, P1327, DOI 10.1002/elps.200305844

Chen GA, 2002, CLIN CANCER RES, V8, P2298

Chung JY, 2006, CANCER EPIDEM BIOMAR, V15, P1403, DOI 10.1158/1055-9965.EPI-05-0651

Concannon CG, 2003, APOPTOSIS, V8, P61, DOI 10.1023/A:1021601103096

Daly JM, 1996, CANCER, V78, P1820, DOI 10.1002/(SICI)1097-0142(19961015)78:8<1820::AID-CNCR25>3.0.CO;2-Z

Du XL, 2007, J MOL MED, V85, P863, DOI 10.1007/s00109-007-0159-4

Gorg A, 2000, ELECTROPHORESIS, V21, P1037, DOI 10.1002/(SICI)1522-2683(20000401)21:6<1037::AID-ELPS1037>3.0.CO;2-V

Hasegawa N, 2006, UROL INT, V77, P347, DOI 10.1159/000096340

Hirano T, 1997, BRIT J CANCER, V75, P978, DOI 10.1038/bjc.1997.169

Jungblut PR, 1999, ELECTROPHORESIS, V20, P2100

KATO H, 1977, CANCER, V40, P1621, DOI 10.1002/1097-0142(197710)40:4<1621::AID-CNCR2820400435>3.0.CO;2-I

Kojic S, 2010, J MED BIOCHEM, V29, P157, DOI 10.2478/v10011-010-0024-9

Leonardi R, 2002, ONCOL REP, V9, P261

MATSUDA H, 1990, CANCER, V65, P2261, DOI 10.1002/1097-0142(19900515)65:10<2261::AID-CNCR2820651017>3.0.CO;2-8

Montgomerie JZ, 1997, CLIN BIOCHEM, V30, P613, DOI 10.1016/S0009-9120(97)00115-X

Paweletz CP, 2000, CANCER RES, V60, P6293

Qi YJ, 2005, PROTEOMICS, V5, P2960, DOI 10.1002/pmic.200401175

Ramagli LS, 1999, METH MOL B, V112, P99

Shi YY, 2006, BREAST CANCER RES TR, V100, P33, DOI 10.1007/s10549-006-9232-x

Silverman GA, 2001, J BIOL CHEM, V276, P33293, DOI 10.1074/jbc.R100016200

Singh A, 2009, APPL IMMUNOHISTO M M, V17, P419, DOI 10.1097/PAI.0b013e31819d3753

Srinivas PR, 2002, CLIN CHEM, V48, P1160

Suminami Y, 1998, TUMOR BIOL, V19, P488, DOI 10.1159/000030042

SUMINAMI Y, 1991, BIOCHEM BIOPH RES CO, V181, P51, DOI 10.1016/S0006-291X(05)81380-4

Wang X, 2008, J CANCER RES CLIN, V134, P995, DOI 10.1007/s00432-008-0367-5

Xue L, 2006, BMC CANCER, V6, DOI 10.1186/1471-2407-6-296

Yan JX, 2000, ELECTROPHORESIS, V21, P3666, DOI 10.1002/1522-2683(200011)21:17<3666::AID-ELPS3666>3.0.CO;2-6

Yokoe H, 2009, J CANCER RES CLIN, V135, P1625, DOI 10.1007/s00432-009-0610-8

NR 30

TC 1

Z9 2

U1 0

U2 13

PU VERSITA

PI WARSAW

PA SOLIPSKA 14A-1, 02-482 WARSAW, POLAND

SN 1452-8258

J9 J MED BIOCHEM

JI J. Med. Biochem.

PD APR-JUN

PY 2012

VL 31

IS 2

BP 107

EP 114

DI 10.2478/v10011-011-0048-9

PG 8

WC Biochemistry & Molecular Biology

WE Science Citation Index Expanded (SCI-EXPANDED)

SC Biochemistry & Molecular Biology

GA 891NR

UT WOS:000300224400005

OA gold

DA 2022-05-19

ER

PT J

AU Ma, JF

Li, N

Zhao, JM

Lu, J

Ma, YQ

Zhu, QH

Dong, ZM

Liu, KD

Ming, L

AF Ma, Junfen

Li, Nan

Zhao, Jimin

Lu, Jing

Ma, Yanqiu

Zhu, Qinghua

Dong, Ziming

Liu, Kangdong

Ming, Liang

TI Histone deacetylase inhibitor trichostatin A enhances the antitumor

effect of the oncolytic adenovirus H101 on esophageal squamous cell

carcinoma in vitro and in vivo

SO ONCOLOGY LETTERS

LA English

DT Article

DE histone deacetylase inhibitor; trichostatin A; esophageal squamous cell

carcinoma; oncolytic adenovirus

ID CANCER-CELLS; GENE-THERAPY; RECOMBINANT ADENOVIRUS; COLORECTAL-CANCER;

BREAST-CANCER; COMBINATION; VIROTHERAPY; RECEPTOR; CHEMOTHERAPY;

REPLICATION

AB Replication-selective oncolytic virotherapy provides a novel modality to treat cancer by inducing cell death in tumor cells but not in normal cells. However, the utilization of oncolytic viruses as a stand-alone treatment is problematic due to their poor transduction efficiency in vivo. H101 was the first oncolytic adenovirus (Ads) to be approved by the Chinese FDA, and exhibits modest antitumor effects when applied as a single agent. The multiple histone deacetylase inhibitor trichostatin A (TSA) has been demonstrated to potently enhance the spread and replication of oncolytic Ads in several infection-resistant types of cancer. The present study aimed to investigate the antitumor effects of H101 in combination with TSA on esophageal squamous cell carcinoma (ESCC) in vitro and in vivo, and determine the mechanisms underlying these effects. H101 and TSA in combination increased the survival of mice harboring human ESCC cell line-tumor xenografts, as compared with mice treated with these agents individually. Therefore, TSA may enhance the antitumor effects of H101 in ESCC.

C1 [Ma, Junfen; Li, Nan; Zhu, Qinghua; Ming, Liang] Zhengzhou Univ, Affiliated Hosp 1, Dept Clin Lab, 1 Jianshe Rd, Zhengzhou 450052, Henan, Peoples R China.

[Zhao, Jimin; Lu, Jing; Dong, Ziming; Liu, Kangdong] Zhengzhou Univ, Sch Basic Med Sci, Dept Pathophysiol, 100 Kexue Ave, Zhengzhou 450001, Henan, Peoples R China.

[Ma, Yanqiu] Henan Univ, Huaihe Hosp, Dept Obstet & Gynecol, Kaifeng 475001, Henan, Peoples R China.

RP Ming, L (corresponding author), Zhengzhou Univ, Affiliated Hosp 1, Dept Clin Lab, 1 Jianshe Rd, Zhengzhou 450052, Henan, Peoples R China.; Liu, KD (corresponding author), Zhengzhou Univ, Sch Basic Med Sci, Dept Pathophysiol, 100 Kexue Ave, Zhengzhou 450001, Henan, Peoples R China.

EM kdliu@zzu.edu.cn; mingliang@zzu.edu.cn

FU National Natural Science Foundation of China [81372269]; Science

Foundation of Henan Education Department [13A310622]; Key project on

Science and Technology provided by Henan Province, China [152102410067,

162102310142, 201403035]

FX This study was supported by the National Natural Science Foundation of

China (grant no. 81372269), the Science Foundation of Henan Education

Department (grant no. 13A310622) and the Key project on Science and

Technology provided by Henan Province, China (grant nos. 152102410067,

162102310142 and 201403035).

CR Alvarez-Breckenridge C, 2009, CHEM REV, V109, P3125, DOI 10.1021/cr900048k

Auer D, 2009, BREAST CANCER RES TR, V116, P103, DOI 10.1007/s10549-008-0108-0

Barbetti V, 2013, EPIGENETICS-US, V8, P210, DOI 10.4161/epi.23538

Bhattacharyya M, 2011, CANCER GENE THER, V18, P734, DOI 10.1038/cgt.2011.45

Bonavina L, 2003, DIS ESOPHAGUS, V16, P90, DOI 10.1046/j.1442-2050.2003.00300.x

Chen Gang, 2005, Ai Zheng, V24, P1196

Chen X, 2013, CELL DEATH DIS, V4, DOI 10.1038/cddis.2013.416

D'Assoro AB, 2010, INT J ONCOL, V37, P1167, DOI 10.3892/ijo_00000768

Eikenes L, 2004, CANCER RES, V64, P4768, DOI 10.1158/0008-5472.CAN-03-1472

Ellis L, 2009, MOL CANCER THER, V8, P1409, DOI 10.1158/1535-7163.MCT-08-0860

Essmann F, 2012, BRIT J PHARMACOL, V165, P328, DOI 10.1111/j.1476-5381.2011.01570.x

Ferreira TB, 2005, BIOTECHNOL LETT, V27, P1809, DOI 10.1007/s10529-005-3558-z

Fujiwara Y, 2013, MOL CLIN ONCOL, V1, P773, DOI 10.3892/mco.2013.128

Fujiwara Y, 2012, ONCOL REP, V28, P446, DOI 10.3892/or.2012.1847

Garber K, 2006, J NATL CANCER I, V98, P298, DOI 10.1093/jnci/djj111

Henry O, 2004, BIOTECHNOL BIOENG, V86, P765, DOI 10.1002/bit.20074

Huang HL, 2012, ACTA BIOCH BIOPH SIN, V44, P737, DOI 10.1093/abbs/gms060

Huang KY, 2014, ONCOL LETT, V8, P1441, DOI 10.3892/ol.2014.2343

Jardon M, 2003, BIOTECHNOL PROGR, V19, P202, DOI 10.1021/bp025585a

Jemal A, 2010, CANCER EPIDEM BIOMAR, V19, P1893, DOI 10.1158/1055-9965.EPI-10-0437

Kothari V, 2010, INT J CANCER, V126, P733, DOI 10.1002/ijc.24700

Krell J, 2013, EPIGENOMICS-UK, V5, P417, DOI [10.2217/EPI.13.41, 10.2217/epi.13.41]

Kumar S, 2008, CURR OPIN MOL THER, V10, P371

Lee JY, 2013, NUCLEIC ACIDS RES, V41, P5614, DOI 10.1093/nar/gkt222

Li LX, 2013, J TRANSL MED, V11, DOI 10.1186/1479-5876-11-257

Lichtenstein DL, 2004, CANCER GENE THER, V11, P819, DOI 10.1038/sj.cgt.7700765

Lu W, 2004, WORLD J GASTROENTERO, V10, P3634, DOI 10.3748/wjg.v10.i24.3634

Ma JF, 2012, INT J MOL MED, V30, P1403, DOI 10.3892/ijmm.2012.1133

MacTavish H, 2010, PLOS ONE, V5, DOI 10.1371/journal.pone.0014462

Meng FL, 2013, INT J ONCOL, V43, P495, DOI 10.3892/ijo.2013.1960

Minsky BD, 2002, J CLIN ONCOL, V20, P1167, DOI 10.1200/JCO.20.5.1167

Minucci S, 2006, NAT REV CANCER, V6, P38, DOI 10.1038/nrc1779

Nettelbeck DM, 2000, TRENDS GENET, V16, P174, DOI 10.1016/S0168-9525(99)01950-2

Nguyen TLA, 2010, CYTOKINE GROWTH F R, V21, P153, DOI 10.1016/j.cytogfr.2010.03.002

Pandha HS, 2003, PROSTATE CANCER P D, V6, P6, DOI 10.1038/sj.pcan.4500611

Reid TR, 2005, CANCER GENE THER, V12, P673, DOI 10.1038/sj.cgt.7700819

Sauthoff H, 2003, HUM GENE THER, V14, P425, DOI 10.1089/104303403321467199

Song X, 2010, INVEST OPHTH VIS SCI, V51, P2626, DOI 10.1167/iovs.09-3516

Stankov MV, 2014, LEUKEMIA, V28, P577, DOI 10.1038/leu.2013.264

Thorne SH, 2005, SEMIN ONCOL, V32, P537, DOI 10.1053/j.seminoncol.2005.09.007

Toth K, 2010, VIRUSES-BASEL, V2, P1844, DOI 10.3390/v2091844

Tysome JR, 2009, CURR OPIN MOL THER, V11, P664

Walters RW, 2002, CELL, V110, P789, DOI 10.1016/S0092-8674(02)00912-1

Wang YH, 2005, CLIN CANCER RES, V11, P351

Wilson PM, 2013, INVEST NEW DRUG, V31, P845, DOI 10.1007/s10637-012-9914-7

Ye RR, 2013, CHEM-EUR J, V19, P10160, DOI 10.1002/chem.201300814

Yen CC, 2001, CANCER, V92, P2769, DOI 10.1002/1097-0142(20011201)92:11<2769::AID-CNCR10118>3.0.CO;2-M

Zhu HW, 2013, MOL CLIN ONCOL, V1, P1009, DOI 10.3892/mco.2013.161

NR 48

TC 9

Z9 9

U1 2

U2 5

PU SPANDIDOS PUBL LTD

PI ATHENS

PA POB 18179, ATHENS, 116 10, GREECE

SN 1792-1074

EI 1792-1082

J9 ONCOL LETT

JI Oncol. Lett.

PD JUN

PY 2017

VL 13

IS 6

BP 4868

EP 4874

DI 10.3892/ol.2017.6069

PG 7

WC Oncology

WE Science Citation Index Expanded (SCI-EXPANDED)

SC Oncology

GA EY3XP

UT WOS:000403909200126

PM 28599488

OA Green Published, gold

DA 2022-05-19

ER

PT J

AU Li, QH

Mao, LH

Wang, RL

Zhu, LQ

Xue, LX

AF Li, Qinghua

Mao, Lihong

Wang, Ruili

Zhu, Liqiang

Xue, Lexun

TI Overexpression of S-adenosylhomocysteine hydrolase (SAHH) in esophageal

squamous cell carcinoma (ESCC) cell lines: effects on apoptosis,

migration and adhesion of cells

SO MOLECULAR BIOLOGY REPORTS

LA English

DT Article

DE Apoptosis; Esophageal squamous cell carcinoma; Receptor of activated

kinase C1; S-adenosylhomocysteine hydrolase

ID L-HOMOCYSTEINE HYDROLASE; DOWN-REGULATION; PROLIFERATION; INVASION;

RECEPTOR; GROWTH

AB S-adenosylhomocysteine hydrolase (SAHH) is the sole enzyme that catalyses the hydrolysis of S-adenosylhomocysteine (SAH) in methylation reaction. Previous studies have shown that its inhibition or deficiency leads to several human disorders such as severe coagulopathy, hepatopathy and myopathy. However, the effects of SAHH on esophageal squamous cell carcinoma (ESCC) cells have not been explored so far. To determine whether SAHH is involved in carcinogenesis of the esophagus, we investigated the expression of SAHH in ESCC and normal esophageal epithelial cells and found that SAHH was downregulated in ESCC cells compared with normal esophageal epithelial cells (P < 0.05). The overexpressed SAHH in ESCC cells promoted cell apoptosis, inhibited cell migration and adhesion, but did not affect the cell proliferation and cell cycle. Furthermore, an interaction of SAHH with receptor of activated C kinase 1 (RACK1) protein was detected by coimmunoprecipitation and an increased RACK1, which is caused by overexpression of SAHH, was verified by Western blotting. The findings mentioned above demonstrate that SAHH can promote apoptosis, inhibit migration and adhesion of ESCC cells suggesting that it may be involved in carcinogenesis of the esophagus.

C1 [Li, Qinghua; Xue, Lexun] Zhengzhou Univ, Affiliated Hosp 1, Cell Biol Lab, Zhengzhou 450052, Henan, Peoples R China.

[Mao, Lihong; Wang, Ruili; Xue, Lexun] Zhengzhou Univ, Dept Biol, Zhengzhou 450001, Henan, Peoples R China.

[Zhu, Liqiang] Zhengzhou Univ, Affiliated Hosp 2, Clin Lab, Zhengzhou 450052, Henan, Peoples R China.

RP Xue, LX (corresponding author), Zhengzhou Univ, Affiliated Hosp 1, Cell Biol Lab, 40 Daxue Rd, Zhengzhou 450052, Henan, Peoples R China.

EM xuelx@zzu.edu.cn

FU International Science and Technology Cooperation Program of the Ministry

of Science and Technology of P.R. China [2007DFA01240]; National Natural

Science Foundation of China [30700014]

FX This study was supported by the grants from International Science and

Technology Cooperation Program of the Ministry of Science and Technology

of P.R. China (No. 2007DFA01240), and the National Natural Science

Foundation of China (No. 30700014)

CR Barry CP, 2000, CANCER RES, V60, P1887

Borley AC, 2008, BREAST CANCER RES, V10, DOI 10.1186/bcr2206

Coskun U, 2006, INT IMMUNOPHARMACOL, V6, P672, DOI 10.1016/j.intimp.2005.10.009

Deng YZ, 2012, GASTROENTEROLOGY, V142, P812, DOI 10.1053/j.gastro.2011.12.046

Harrington EO, 2000, AM J PHYSIOL, V279, P733

Hayden A, 2011, BREAST CANCER RES TR, V127, P109, DOI 10.1007/s10549-010-0982-0

Hermes M, 2008, CELL PHYSIOL BIOCHEM, V22, P223, DOI 10.1159/000149800

Hermes M, 2007, EXP CELL RES, V313, P264, DOI 10.1016/j.yexcr.2006.10.003

Honzik T, 2012, MOL GENET METAB, V107, P611, DOI 10.1016/j.ymgme.2012.08.014

Hou GQ, 2010, CANCER LETT, V290, P248, DOI 10.1016/j.canlet.2009.09.015

Leal JF, 2008, CARCINOGENESIS, V29, P2089, DOI 10.1093/carcin/bgn198

Li QH, 2013, CURR MICROBIOL, V164, P1028

Lleonart ME, 2006, ONCOL REP, V16, P603

Nakanishi M, 2007, YAKUGAKU ZASSHI, V127, P977, DOI 10.1248/yakushi.127.977

PALMER JL, 1979, J BIOL CHEM, V254, P1217

Sedding DG, 2009, CIRC RES, V104, P1192, DOI 10.1161/CIRCRESAHA.109.194357

Shi S, 2011, J BIOCH, V287, P7845

Tanaka N, 2004, J MOL BIOL, V343, P1007, DOI 10.1016/j.jmb.2004.08.104

Wang F, 2011, CANCER SCI, V102, P2007, DOI 10.1111/j.1349-7006.2011.02065.x

Wang LL, 2010, CANCER INVEST, V28, P993, DOI 10.3109/07357907.2010.483503

Wang Zhong-yue, 2011, Zhonghua Yi Xue Za Zhi, V91, P1397

Zaina S, 2005, J NUTR, V135, P5, DOI 10.1093/jn/135.1.5

NR 22

TC 10

Z9 11

U1 1

U2 14

PU SPRINGER

PI DORDRECHT

PA VAN GODEWIJCKSTRAAT 30, 3311 GZ DORDRECHT, NETHERLANDS

SN 0301-4851

EI 1573-4978

J9 MOL BIOL REP

JI Mol. Biol. Rep.

PD APR

PY 2014

VL 41

IS 4

BP 2409

EP 2417

DI 10.1007/s11033-014-3095-8

PG 9

WC Biochemistry & Molecular Biology

WE Science Citation Index Expanded (SCI-EXPANDED)

SC Biochemistry & Molecular Biology

GA AE1DN

UT WOS:000333707100057

PM 24430301

DA 2022-05-19

ER

PT J

AU Xia, TL

Yan, SM

Yuan, L

Zeng, MS

AF Xia, Tian-Liang

Yan, Shu-Mei

Yuan, Li

Zeng, Mu-Sheng

TI Upregulation of METTL3 Expression Predicts Poor Prognosis in Patients

with Esophageal Squamous Cell Carcinoma

SO CANCER MANAGEMENT AND RESEARCH

LA English

DT Article

DE esophageal squamous cell carcinoma; METTL3; prognosis; survival

ID MESSENGER-RNA METHYLATION; NUCLEAR-RNA; N6-METHYLADENOSINE; TRANSLATION;

SUBUNIT; YTHDC2

AB Background: The N-6-methyladenosine (m6A) RNA modification of mRNA mediates various cellular functions and cancer progression. However, the roles of m(6)A RNA modification in the regulation of esophageal squamous cell carcinoma (ESCC), the dominant subtype of esophageal cancer in Asia, were unclear.

Materials and Methods: Here, we analyzed the mRNA expression level of methyltransferase like 3 (METTL3) in the public available datasets of ESCC tissues and matched adjacent normal tissues. We also performed immunohistochemistry (IHC) assays to detect the protein expression of METTL3 in human ESCC tissue specimen. In our study, we also analyzed the association between METTL3 expression and prognosis using Cox proportional hazard regression in 207 ESCC patients.

Results: The results of public available datasets and IHC assays showed that METTL3 was upregulated in tumor compared with adjacent nonmalignant esophageal mucosal tissues. The IHC results indicated that higher expression level of METTL3 was associated with worse survival. We also found that METTL3 expression level was an independent predictor for disease-free survival and overall survival of ESCC patients.

Conclusion: Our results revealed that the METTL3 expression level could be used as an independent prognostic biomarker for ESCC prognosis.

C1 [Xia, Tian-Liang; Yan, Shu-Mei; Yuan, Li; Zeng, Mu-Sheng] State Key Lab Oncol South China, Guangzhou, Peoples R China.

[Xia, Tian-Liang; Yan, Shu-Mei; Yuan, Li; Zeng, Mu-Sheng] Collaborat Innovat Ctr Canc Med, Guangzhou, Peoples R China.

[Xia, Tian-Liang; Yan, Shu-Mei; Yuan, Li; Zeng, Mu-Sheng] Sun Yat sen Univ, Canc Ctr, Guangzhou, Peoples R China.

RP Zeng, MS (corresponding author), State Key Lab Oncol South China, Guangzhou, Peoples R China.

EM zengmsh@sysucc.org

FU National Natural Science Foundation of China [81830090, 81802775];

Natural Science Foundation of Guang Dong Province [20181025201938243]

FX This study was supported by the National Natural Science Foundation of

China (81830090, 81802775), Natural Science Foundation of Guang Dong

Province (No. 20181025201938243).

CR Abbas G, 2017, ANN CARDIOTHORAC SUR, V6, P131, DOI 10.21037/acs.2017.03.03

Bokar JA, 1997, RNA, V3, P1233

Cai X, 2017, CANC LETT, V415, P11, DOI DOI 10.1016/J.CANLET.2017.11.018

Chen MN, 2018, HEPATOLOGY, V67, P2254, DOI 10.1002/hep.29683

DESROSIERS R, 1974, P NATL ACAD SCI USA, V71, P3971, DOI 10.1073/pnas.71.10.3971

Dominissini D, 2012, NATURE, V485, P201, DOI 10.1038/nature11112

Edupuganti RR, 2017, NAT STRUCT MOL BIOL, V24, P870, DOI 10.1038/nsmb.3462

Hsu PJ, 2017, CELL RES, V27, P1115, DOI 10.1038/cr.2017.99

Jemal A, 2010, CA-CANCER J CLIN, V60, P277, DOI 10.3322/caac.20073

Jia GF, 2011, NAT CHEM BIOL, V7, P885, DOI [10.1038/NCHEMBIO.687, 10.1038/nchembio.687]

Li A, 2017, CELL RES, V27, P444, DOI 10.1038/cr.2017.10

Liu JZ, 2014, NAT CHEM BIOL, V10, P93, DOI 10.1038/nchembio.1432

Meyer KD, 2012, CELL, V149, P1635, DOI 10.1016/j.cell.2012.05.003

Ping XL, 2014, CELL RES, V24, P177, DOI 10.1038/cr.2014.3

Schwartz S, 2014, CELL REP, V8, P284, DOI 10.1016/j.celrep.2014.05.048

Shi HL, 2017, CELL RES, V27, P315, DOI 10.1038/cr.2017.15

Visvanathan A, 2018, ONCOGENE, V37, P522, DOI 10.1038/onc.2017.351

Vu LP, 2017, NAT MED, V23, P1369, DOI 10.1038/nm.4416

Wang X, 2015, CELL, V161, P1388, DOI 10.1016/j.cell.2015.05.014

Wang X, 2014, NATURE, V505, P117, DOI 10.1038/nature12730

WEI CM, 1976, BIOCHEMISTRY-US, V15, P397, DOI 10.1021/bi00647a024

Wojtas MN, 2017, MOL CELL, V68, P374, DOI 10.1016/j.molcel.2017.09.021

Xiao W, 2016, MOL CELL, V61, P507, DOI 10.1016/j.molcel.2016.01.012

Zhao J, 2012, ASIAN PAC J CANCER P, V13, P4613, DOI 10.7314/APJCP.2012.13.9.4613

Zheng GQ, 2013, MOL CELL, V49, P18, DOI 10.1016/j.molcel.2012.10.015

NR 25

TC 10

Z9 10

U1 2

U2 4

PU DOVE MEDICAL PRESS LTD

PI ALBANY

PA PO BOX 300-008, ALBANY, AUCKLAND 0752, NEW ZEALAND

SN 1179-1322

J9 CANCER MANAG RES

JI Cancer Manag. Res.

PY 2020

VL 12

BP 5729

EP 5737

DI 10.2147/CMAR.S245019

PG 9

WC Oncology

WE Science Citation Index Expanded (SCI-EXPANDED)

SC Oncology

GA MM0MN

UT WOS:000549854500001

PM 32765076

OA gold, Green Published

DA 2022-05-19

ER

PT J

AU Zhong, CX

Fan, LM

Li, ZG

Yao, F

Zhao, H

AF Zhong, Chenxi

Fan, Limin

Li, Zhigang

Yao, Feng

Zhao, Heng

TI SREBP2 is upregulated in esophageal squamous cell carcinoma and

co-operates with c-Myc to regulate HMGCR expression

SO MOLECULAR MEDICINE REPORTS

LA English

DT Article

DE sterol regulatory element-binding protein 2; esophageal squamous cell

carcinoma; c-Myc; statin

ID MEVALONATE PATHWAY; HALLMARKS; P53

AB Dysregulations of the mevalonate pathway (MVA) have been previously identified. Our previous study demonstrated that 3-hydroxy-3-methylglutaryl-coenzyme A reductase (HMGCR), the rate-limiting enzyme of the MVA pathway, was upregulated in esophageal squamous cell carcinoma (ESCC) and statin-inhibited ESCC tumorigenesis. However, the underlying mechanism of HMGCR regulation in ESCC remains unknown. In the present study, western blotting and immunohistochemistry analysis demonstrated that sterol regulatory element-binding protein 2 (SREBP2), the master regulator for HMGCR, was upregulated in ESCC clinical samples. Overexpression of SREBP2 expression in ESCC cell lines promoted the growth, migration and colony formation of cancer cells in the MTT, Boyden chamber and soft agar assays, respectively, which was inhibited by lovastatin. Downregulation of SREBP2 expression in ESCC cell lines inhibited the viability, and migration and colony formation abilities of cancer cells. Assessment of the molecular mechanism demonstrated that SREBP2 interacted with c-Myc and cooperated with c-Myc to activate HMGCR expression. Collectively, the present study identified SREBP2 as an oncogene associated with the tumorigenesis of ESCC and further demonstrated the therapeutic effects of statins in ESCC.

C1 [Zhong, Chenxi; Fan, Limin; Li, Zhigang; Yao, Feng; Zhao, Heng] Shanghai Jiao Tong Univ, Shanghai Chest Hosp, Dept Thorac Surg, 241 West Huaihai Rd, Shanghai 200030, Peoples R China.

RP Yao, F; Zhao, H (corresponding author), Shanghai Jiao Tong Univ, Shanghai Chest Hosp, Dept Thorac Surg, 241 West Huaihai Rd, Shanghai 200030, Peoples R China.

EM yaofeng20120913@aliyun.com; shchestsurgery@163.com

OI Yao, Feng/0000-0002-9635-9581

FU National Science Foundation of China [81401933]; Shanghai Science and

Technology Committee [13ZR1461300]

FX The present study was supported by the National Science Foundation of

China (grant no. 81401933) and Shanghai Science and Technology Committee

(grant no. 13ZR1461300; Shanghai, China).

CR Andela VB, 2003, CLIN ORTHOP RELAT R, pS59, DOI 10.1097/01.blo.0000093846.72468.66

Bathaie SZ, 2017, CURR MOL PHARMACOL, V10, P77, DOI 10.2174/1874467209666160112123205

Brown J, 2011, WORLD J GASTROENTERO, V17, P2909, DOI 10.3748/wjg.v17.i24.2909

Caruso MG, 2005, ANTICANCER RES, V25, P3393

Freed-Pastor WA, 2012, CELL, V148, P244, DOI 10.1016/j.cell.2011.12.017

GOLDSTEIN JL, 1990, NATURE, V343, P425, DOI 10.1038/343425a0

Gomes TS, 2013, PATHOL RES PRACT, V209, P6, DOI 10.1016/j.prp.2012.08.011

Gordan JD, 2007, CANCER CELL, V12, P108, DOI 10.1016/j.ccr.2007.07.006

Ishizuka T, 2002, BIOCHEM BIOPH RES CO, V296, P152, DOI 10.1016/S0006-291X(02)00836-7

Kajinami K, 2004, ATHEROSCLEROSIS, V177, P219, DOI 10.1016/j.atherosclerosis.2004.09.004

LANDAU BR, 1985, METHOD ENZYMOL, V110, P100

Livak KJ, 2001, METHODS, V25, P402, DOI 10.1006/meth.2001.1262

Ma K, 2014, PLOS ONE, V9, DOI 10.1371/journal.pone.0084221

Madison BB, 2016, J LIPID RES, V57, P333, DOI 10.1194/jlr.C066712

Mandard AM, 2000, MUTAT RES-REV MUTAT, V462, P335, DOI 10.1016/S1383-5742(00)00019-3

Miller DM, 2012, CLIN CANCER RES, V18, P5546, DOI 10.1158/1078-0432.CCR-12-0977

Mukherjee M, 2014, INT J BIOCHEM CELL B, V55, P196, DOI 10.1016/j.biocel.2014.08.019

Munkley J, 2016, ONCOTARGET, V7, P35478, DOI 10.18632/oncotarget.8155

Schwartz L, 2017, ANTI-CANCER AGENT ME, V17, P164, DOI 10.2174/1871520616666161031143301

Shi JX, 2013, TUMOR BIOL, V34, P429, DOI 10.1007/s13277-012-0567-0

Shukla SK, 2015, ONCOTARGET, V6, P19118, DOI 10.18632/oncotarget.4078

Siegel RL, 2017, CA-CANCER J CLIN, V67, P7, DOI 10.3322/caac.21387

Swanson KM, 2006, CURR CANCER DRUG TAR, V6, P15, DOI 10.2174/156800906775471743

Wang TT, 2016, ONCOTARGET, V7, P2532, DOI 10.18632/oncotarget.6304

Wang W, 2011, MED ONCOL, V28, P163, DOI 10.1007/s12032-010-9436-0

Weinberger C, 1996, TRENDS ENDOCRIN MET, V7, P1, DOI 10.1016/1043-2760(95)00180-8

Xie JJ, 2010, AM J PATHOL, V176, P939, DOI 10.2353/ajpath.2010.090118

Zhong CX, 2014, TUMOR BIOL, V35, P4123, DOI 10.1007/s13277-013-1539-8

NR 28

TC 7

Z9 9

U1 1

U2 4

PU SPANDIDOS PUBL LTD

PI ATHENS

PA POB 18179, ATHENS, 116 10, GREECE

SN 1791-2997

EI 1791-3004

J9 MOL MED REP

JI Mol. Med. Rep.

PD OCT

PY 2019

VL 20

IS 4

BP 3003

EP 3010

DI 10.3892/mmr.2019.10577

PG 8

WC Oncology; Medicine, Research & Experimental

WE Science Citation Index Expanded (SCI-EXPANDED)

SC Oncology; Research & Experimental Medicine

GA JC3IA

UT WOS:000489171200003

PM 31432128

OA Green Published, hybrid, Green Submitted

DA 2022-05-19

ER

PT J

AU Zu, XY

Xie, XM

Zhang, YY

Liu, KD

Bode, AM

Dong, ZG

Kim, DJ

AF Zu, Xueyin

Xie, Xiaomeng

Zhang, Yuanyuan

Liu, Kangdong

Bode, Ann M.

Dong, Zigang

Kim, Dong Joon

TI Lapachol is a novel ribosomal protein S6 kinase 2 inhibitor that

suppresses growth and induces intrinsic apoptosis in esophageal squamous

cell carcinoma cells

SO PHYTOTHERAPY RESEARCH

LA English

DT Article

DE apoptosis; esophageal squamous cell carcinoma; lapachol; RSK2

ID CYCLIN D1 EXPRESSION; RSK FAMILY; PHOSPHORYLATION; MULTICENTER;

ISOFORMS; DOMAIN; EGFR

AB Lapachol is a 1,4-naphthoquinone that is isolated from the Bignoniaceae family. It has been reported to exert anti-inflammatory, antibacterial, and anticancer activities. However, the anticancer activity of lapachol and its molecular mechanisms against esophageal squamous cell carcinoma (ESCC) cells have not been fully investigated. Herein, we report that lapachol is a novel ribosomal protein S6 kinase 2 (RSK2) inhibitor that suppresses growth and induces intrinsic apoptosis in ESCC cells. We found that lapachol strongly attenuates downstream signaling molecules of RSK2 in ESCC cells and also directly inhibits RSK2 activity in vitro. The RSK protein is highly activated in ESCC cells and knockdown of RSK2 significantly suppresses anchorage-dependent and anchorage-independent growth of ESCC cells. Additionally, lapachol inhibits anchorage-dependent and anchorage-independent growth of ESCC cells, and the inhibition of cell growth by lapachol is dependent on the expression of RSK2. We also found that lapachol induces mitochondria-mediated cellular apoptosis by activating caspases-3, -7, and PARP, inducing the expression of cytochrome c and BAX by inhibiting downstream molecules of RSK2. Overall, lapachol is a potent RSK2 inhibitor that might be used for chemotherapy against ESCC.

C1 [Zu, Xueyin; Xie, Xiaomeng; Zhang, Yuanyuan; Liu, Kangdong; Dong, Zigang; Kim, Dong Joon] China US Henan Hormel Canc Inst, Dept Mol & Cellular Biol, 127 Dongming Rd, Zhengzhou 450008, Henan, Peoples R China.

[Zu, Xueyin; Xie, Xiaomeng; Zhang, Yuanyuan; Liu, Kangdong; Dong, Zigang; Kim, Dong Joon] Zhengzhou Univ, Sch Basic Med Sci, Pathophysiol Dept, Zhengzhou, Henan, Peoples R China.

[Liu, Kangdong] Zhengzhou Univ, Affiliated Canc Hosp, Zhengzhou, Henan, Peoples R China.

[Liu, Kangdong; Dong, Zigang; Kim, Dong Joon] Collaborat Innovat Ctr Henan Prov Canc Chemopreve, Zhengzhou, Henan, Peoples R China.

[Bode, Ann M.; Dong, Zigang] Univ Minnesota, Hormel Inst, 801 16th Ave NE, Austin, MN 55912 USA.

[Dong, Zigang] Int Joint Res Ctr Canc Chemoprevent, Zhengzhou, Henan, Peoples R China.

RP Kim, DJ (corresponding author), China US Henan Hormel Canc Inst, Dept Mol & Cellular Biol, 127 Dongming Rd, Zhengzhou 450008, Henan, Peoples R China.

EM djkim@hci-cn.org

RI Kim, Dong Joon/AAL-8967-2020

OI Kim, Dong Joon/0000-0001-6910-9213

FU National Natural Science Foundation China [81572812] Funding Source:

Medline; Henan Joint Fund [U1804196] Funding Source: Medline; Key

program of Henan Province, China [161100510300] Funding Source: Medline

CR Anjum R, 2008, NAT REV MOL CELL BIO, V9, P747, DOI 10.1038/nrm2509

Babu MS, 2018, PLOS ONE, V13, DOI 10.1371/journal.pone.0191419

Casalvieri KA, 2017, TRENDS CANCER, V3, P302, DOI 10.1016/j.trecan.2017.03.004

Chen BQ, 2018, CANCER BIOL THER, V19, P76, DOI 10.1080/15384047.2017.1394541

Cho YY, 2017, ARCH PHARM RES, V40, P291, DOI 10.1007/s12272-016-0880-z

Crosby T, 2013, LANCET ONCOL, V14, P627, DOI 10.1016/S1470-2045(13)70136-0

Cui XB, 2014, INT J CLIN EXP PATHO, V7, P2382

DAKSIS JI, 1994, ONCOGENE, V9, P3635

De Cesare D, 1998, P NATL ACAD SCI USA, V95, P12202, DOI 10.1073/pnas.95.21.12202

Dutton SJ, 2014, LANCET ONCOL, V15, P894, DOI 10.1016/S1470-2045(14)70024-5

Enzinger PC, 2003, NEW ENGL J MED, V349, P2241, DOI 10.1056/NEJMra035010

Ferlay J, 2015, INT J CANCER, V136, pE359, DOI 10.1002/ijc.29210

Hu XY, 2019, MOL ONCOL, V13, P246, DOI 10.1002/1878-0261.12400

Hussain H, 2017, EXPERT OPIN THER PAT, V27, P1111, DOI 10.1080/13543776.2017.1339792

Kang XZ, 2015, WORLD J GASTROENTERO, V21, P7648, DOI 10.3748/wjg.v21.i25.7648

Lara R, 2013, CANCER RES, V73, P5301, DOI 10.1158/0008-5472.CAN-12-4448

Lavoie JN, 1996, J BIOL CHEM, V271, P20608, DOI 10.1074/jbc.271.34.20608

LEE T, 2018, CELLS-BASEL, V7, DOI DOI 10.3390/CELLS7110187

Li CQ, 2017, ONCOGENESIS, V6, DOI 10.1038/oncsis.2017.1

Liu F, 1999, MOL CELL BIOL, V19, P5113

Maehara O, 2017, CARCINOGENESIS, V38, P1073, DOI 10.1093/carcin/bgx095

Malakhova M, 2008, NAT STRUCT MOL BIOL, V15, P112, DOI 10.1038/nsmb1347

Malakhova M, 2009, PLOS ONE, V4, DOI 10.1371/journal.pone.0008044

Miller KD, 2016, CA-CANCER J CLIN, V66, P271, DOI 10.3322/caac.21349

Moritz A, 2010, SCI SIGNAL, V3, DOI 10.1126/scisignal.2000998

Roberts PJ, 2007, ONCOGENE, V26, P3291, DOI 10.1038/sj.onc.1210422

Romeo Y, 2012, BIOCHEM J, V441, P553, DOI 10.1042/BJ20110289

Sassone-Corsi P, 1999, SCIENCE, V285, P886, DOI 10.1126/science.285.5429.886

SHIMADA Y, 1992, CANCER, V69, P277, DOI 10.1002/1097-0142(19920115)69:2<277::AID-CNCR2820690202>3.0.CO;2-C

Shimura Y, 2012, MOL CANCER THER, V11, P2600, DOI 10.1158/1535-7163.MCT-12-0605

Sulzmaier FJ, 2013, CANCER RES, V73, P6099, DOI 10.1158/0008-5472.CAN-13-1087

Tungekar A, 2018, SCI REP-UK, V8, DOI 10.1038/s41598-018-30579-3

Wang CN, 2017, CHIN J CANCER, V36, DOI 10.1186/s40880-017-0232-5

Wang H, 2017, ONCOTARGET, V8, P45249, DOI 10.18632/oncotarget.16902

Wang L, 2019, PHYTOTHER RES, V33, P640, DOI 10.1002/ptr.6253

Wong CH, 2015, AM J CANCER RES, V5, P3588

Xu HL, 2016, J EXP CLIN CANC RES, V35, DOI 10.1186/s13046-016-0455-3

Yoshioka M, 2017, J EXP CLIN CANC RES, V36, DOI 10.1186/s13046-017-0572-7

Zhang JT, 2011, J PROTEOME RES, V10, P2863, DOI 10.1021/pr200141c

Zhou J, 2017, NAT COMMUN, V8, DOI 10.1038/ncomms13897

NR 40

TC 4

Z9 4

U1 1

U2 11

PU WILEY

PI HOBOKEN

PA 111 RIVER ST, HOBOKEN 07030-5774, NJ USA

SN 0951-418X

EI 1099-1573

J9 PHYTOTHER RES

JI Phytother. Res.

PD SEP

PY 2019

VL 33

IS 9

BP 2337

EP 2346

DI 10.1002/ptr.6415

PG 10

WC Chemistry, Medicinal; Pharmacology & Pharmacy

WE Science Citation Index Expanded (SCI-EXPANDED)

SC Pharmacology & Pharmacy

GA IV9GY

UT WOS:000484573200016

PM 31225674

DA 2022-05-19

ER

PT J

AU Zhu, XZ

Zhang, YT

Li, QH

Yang, L

Zhang, NN

Ma, SS

Zhang, K

Song, JS

Guan, FX

AF Zhu, Xiangzhan

Zhang, Yanting

Li, Qinghua

Yang, Lu

Zhang, Nannan

Ma, Shanshan

Zhang, Kun

Song, Jishi

Guan, Fangxia

TI beta-Carotene Induces Apoptosis in Human Esophageal Squamous Cell

Carcinoma Cell Lines via the Cav-1/AKT/NF-B Signaling Pathway

SO JOURNAL OF BIOCHEMICAL AND MOLECULAR TOXICOLOGY

LA English

DT Article

DE beta-Carotene; Caveolin-1; Esophageal Squamous Cell Carcinoma;

Proliferation; Apoptosis; AKT; NF-B

ID PROSTATE-CANCER CELLS; LYMPH-NODE METASTASIS; NF-KAPPA-B; TUMOR

DIFFERENTIATION; CYCLE ARREST; CAVEOLIN-1; OVEREXPRESSION; SUPPRESSION;

PROLIFERATION; PROGRESSION

AB beta-carotene, a type of terpenoid, has many metabolic and physiological functions. In particular, -carotene has an antitumor effect. However, the efficacy of -carotene against esophageal squamous cell carcinoma (ESCC) remains unclear. In our study, -carotene inhibited the growth of ESCC cells and downregulated expression of the Caveolin-1 (Cav-1) protein. Cav-1 protein was expressed only in ESCC cells, not in Het-1A cells. Moreover, -carotene triggered apoptosis, induced cell cycle G0/G1 phase arrest, and inhibited cell migration. To explore the mechanism involved in these processes, we further examined the effect of -carotene on the Cav-1-mediated AKT/NF-B pathway. The results showed that the level of AKT and NF-B phosphorylation was dramatically inhibited, which led to an increase in the Bax/Bcl-2 ratio. Correspondingly, the activity of Caspase-3 was also enhanced. These data suggest that -carotene has an antiproliferative role in ESCC cells and may be a promising chemotherapeutic agent for use against ESCC cells.

C1 [Zhu, Xiangzhan; Zhang, Yanting; Li, Qinghua; Yang, Lu; Zhang, Nannan; Ma, Shanshan; Zhang, Kun; Song, Jishi; Guan, Fangxia] Zhengzhou Univ, Sch Life Sci, Zhengzhou 450001, Henan, Peoples R China.

[Zhang, Nannan; Guan, Fangxia] Zhengzhou Univ, Affiliated Hosp 1, Zhengzhou 450052, Henan, Peoples R China.

RP Guan, FX (corresponding author), Zhengzhou Univ, Sch Life Sci, Zhengzhou 450001, Henan, Peoples R China.; Guan, FX (corresponding author), Zhengzhou Univ, Affiliated Hosp 1, Zhengzhou 450052, Henan, Peoples R China.

EM guanfangxia@126.com

FU Innovative Research Team [in Science and Technology] at the University

of Henan Province [15IRTSTHN022]; Plan for Scientific Innovation Talent

of Henan Province [154200510008]; Joint Talent Cultivation Funds of

National Natural Science Foundation of China [U1504310]; Joint Talent

Cultivation Funds of National Natural Science Foundation of Henan

province [U1504310]; Key Research Project of Higher Education of Henan

Province [15A310028, 15A180022]

FX Contract Grant Sponsor: Innovative Research Team [in Science and

Technology] at the University of Henan Province.; Contract Grant Number:

15IRTSTHN022.; Contract Grant Sponsor: Plan for Scientific Innovation

Talent of Henan Province.; Contract Grant Numbers: 154200510008.;

Contract Grant Sponsor: The Joint Talent Cultivation Funds of National

Natural Science Foundation of China and Henan province.; Contract Grant

Number: U1504310.; Contract Grant Sponsor: Key Research Project of

Higher Education of Henan Province.; Contract Grant Numbers: 15A310028

and 15A180022.

CR Berx G, 2001, BREAST CANCER RES, V3, P289, DOI 10.1186/bcr309

Chai JY, 2012, WORLD J GASTROENTERO, V18, P6521, DOI 10.3748/wjg.v18.i45.6521

Chanvorachote P, 2015, MOL CELL BIOCHEM, V399, P291, DOI 10.1007/s11010-014-2255-4

Chanvorachote P, 2014, CANCER CELL INT, V14, DOI 10.1186/1475-2867-14-52

Chatterjee M, 2015, SCI REP-UK, V5, DOI 10.1038/srep10867

Chen HY, 2015, NUTR CANCER, V67, P676, DOI 10.1080/01635581.2015.1019633

Delektorskaya V, 2011, VIRCHOWS ARCH, V459, pS163

Donnellan R, 1998, J CLIN PATHOL-MOL PA, V51, P1

Gloria NF, 2014, ANTICANCER RES, V34, P1377

Grandage VL, 2005, LEUKEMIA, V19, P586, DOI 10.1038/sj.leu.2403653

Haller D, 2002, J BIOL CHEM, V277, P38168, DOI 10.1074/jbc.M205737200

Hu YC, 2001, CLIN CANCER RES, V7, P3519

Huang YL, 2015, J AGR FOOD CHEM, V63, P4561, DOI 10.1021/jf5059304

Kato K, 2002, CANCER, V94, P929, DOI 10.1002/cncr.10329

Li LK, 2003, MOL CELL BIOL, V23, P9389, DOI 10.1128/MCB.23.24.9389-9404.2003

Liang W, 2014, UROL ONCOL-SEMIN ORI, V32, P855, DOI 10.1016/j.urolonc.2014.01.005

Lobos-Gonzalez L, 2013, PIGM CELL MELANOMA R, V26, DOI 10.1111/pcmr.12085

Lu MS, 2015, EUR J NUTR, V54, P619, DOI 10.1007/s00394-014-0743-7

Musgrove EA, 2011, NAT REV CANCER, V11, P558, DOI 10.1038/nrc3090

Palozza P, 2008, CARCINOGENESIS, V29, P2153, DOI 10.1093/carcin/bgn018

Pennathur A, 2013, LANCET, V381, P400, DOI 10.1016/S0140-6736(12)60643-6

Podar K, 2003, J BIOL CHEM, V278, P5794, DOI 10.1074/jbc.M208636200

Shrimali D, 2013, CANCER LETT, V341, P139, DOI 10.1016/j.canlet.2013.08.023

Song MY, 2015, GASTROENTEROLOGY, V148, P1244, DOI 10.1053/j.gastro.2014.12.035

Tahir SA, 2013, CANCER RES, V73, P1900, DOI 10.1158/0008-5472.CAN-12-3040

Tian XG, 2015, ONCOL REP, V33, P3061, DOI 10.3892/or.2015.3942

Wang HY, 2014, ONCOL REP, V32, P1465, DOI 10.3892/or.2014.3352

Wang SK, 2012, ASIAN PAC J CANCER P, V13, P6327, DOI 10.7314/APJCP.2012.13.12.6327

Zhao P, 2015, J BIOCHEM MOL TOXIC, V29, P199, DOI 10.1002/jbt.21685

NR 29

TC 18

Z9 18

U1 0

U2 13

PU WILEY

PI HOBOKEN

PA 111 RIVER ST, HOBOKEN 07030-5774, NJ USA

SN 1095-6670

EI 1099-0461

J9 J BIOCHEM MOL TOXIC

JI J. Biochem. Mol. Toxicol.

PD MAR

PY 2016

VL 30

IS 3

BP 148

EP 157

DI 10.1002/jbt.21773

PG 10

WC Biochemistry & Molecular Biology; Toxicology

WE Science Citation Index Expanded (SCI-EXPANDED)

SC Biochemistry & Molecular Biology; Toxicology

GA DH7HK

UT WOS:000372963300005

PM 26733226

DA 2022-05-19

ER

PT J

AU Hu, W

Liang, Y

Zhang, S

Hu, Y

Liu, J

AF Hu, W.

Liang, Y.

Zhang, S.

Hu, Y.

Liu, J.

TI Impact of tumor location on prognosis in patients with thoracic

esophageal squamous cell carcinoma

SO EUROPEAN SURGERY-ACTA CHIRURGICA AUSTRIACA

LA English

DT Article

DE Esophageal squamous cell carcinoma; Staging; Prognosis; Tumor location

ID AMERICAN JOINT COMMITTEE; CANCER; UNION

AB The impact of tumor location on prognosis is still controversial in patients with esophageal squamous cell carcinoma (ESCC). The purpose of this study was to investigate the impact of tumor location on prognosis in patients with thoracic ESCC and evaluate the scientific value of adding tumor location as a staging factor in the seventh edition of American Joint Cancer Committee staging system of ESCC.

We retrospectively analyzed the clinical data of 1,512 consecutive patients with thoracic ESCC who underwent esophagectomy in The First Affiliated Hospital of Zhengzhou University and The Cancer Center of Sun Yat-sen University from December 1996 to December 2008.

Prognosis could be evaluated satisfactorily for the entire cohort of patients regardless of employing the sixth or the seventh edition staging system. In the entire cohort of patients, no significant difference in survival was found concerning different tumor locations (p > 0.05). In the patients staged T2-3N0M0, there was no significant difference in survival between upper/middle thoracic and lower thoracic groups (p > 0.05).

Adding tumor location as a staging factor in the new American Joint Cancer Committee staging system of ESCC may be not suitable for patients with ESCC in China.

C1 [Hu, W.; Liu, J.] Zhengzhou Univ, Affiliated Hosp 1, Dept Thorac Surg, Zhengzhou 450052, Peoples R China.

[Liang, Y.] Sun Yat Sen Univ, Dept Pediat, Affiliated Hosp 3, Guangzhou 510275, Guangdong, Peoples R China.

[Zhang, S.; Hu, Y.] Sun Yat Sen Univ, Dept Thorac Oncol, Ctr Canc, Guangzhou 510275, Guangdong, Peoples R China.

RP Liu, J (corresponding author), Zhengzhou Univ, Affiliated Hosp 1, Dept Thorac Surg, 1 Jianshe Rd, Zhengzhou 450052, Peoples R China.

EM liujingeng888@163.com

CR Gertler R, 2011, ANN SURG, V253, P689, DOI 10.1097/SLA.0b013e31821111b5

Hsu PK, 2010, ANN THORAC SURG, V89, P1024, DOI 10.1016/j.athoracsur.2010.01.017

Jiang YQ, 2010, ANN THORAC SURG, V90, P908, DOI 10.1016/j.athoracsur.2010.05.060

Li HH, 2009, J THORAC CARDIOV SUR, V137, P55, DOI 10.1016/j.jtcvs.2008.05.024

Rice TW, 2010, CANCER-AM CANCER SOC, V116, P3763, DOI 10.1002/cncr.25146

Ruol A, 2009, ARCH SURG-CHICAGO, V144, P247, DOI 10.1001/archsurg.2008.574

Xie YT, 2004, ZHONGHUA WEI CHANG W, V7, P453

Zhang He-lin, 2009, Zhonghua Zhongliu Zazhi, V31, P541, DOI 10.3760/cma.j.issn.0253-3766.2009.07.015

NR 8

TC 1

Z9 1

U1 0

U2 4

PU SPRINGER WIEN

PI WIEN

PA SACHSENPLATZ 4-6, PO BOX 89, A-1201 WIEN, AUSTRIA

SN 1682-8631

EI 1682-4016

J9 EUR SURG

JI Eur. Surg.

PD AUG

PY 2013

VL 45

IS 4

BP 200

EP 204

DI 10.1007/s10353-013-0223-y

PG 5

WC Surgery

WE Science Citation Index Expanded (SCI-EXPANDED)

SC Surgery

GA 197TI

UT WOS:000322873700004

DA 2022-05-19

ER

PT J

AU Jiang, H

Wang, XH

Yu, XM

Zheng, ZG

AF Jiang, Hong

Wang, Xiao-Hong

Yu, Xin-Min

Zheng, Zhi-Guo

TI Detection and Prognostic Analysis of Serum Protein Expression in

Esophageal Squamous Cell Cancer

SO ASIAN PACIFIC JOURNAL OF CANCER PREVENTION

LA English

DT Article

DE Esophageal cancer; prognosis; serum protein fingerprint

ID SELDI-TOF-MS; FLIGHT MASS-SPECTROMETRY; PROSTATE-CANCER; POTENTIAL

BIOMARKERS; SURFACE; CARCINOMA; ADENOCARCINOMA; IDENTIFICATION;

DIAGNOSIS

AB Objective: To assess differences in serum proteins in esophageal squamous cell carcinoma patients. Methods: 144 esophageal squamous cell carcinoma patients and 50 healthy volunteers were included in this study, with surface-enhanced laser desorption-ionization time-of-flight mass spectrometry and weak cation exchange magnetic beads. Follow-up allowed the relations between serum proteins and prognosis to be analyzed. Results: A total of 93 protein peaks were detected (molecular weight range: 1500-30000), 10 demonstrating statistically significant differences. There were no differences in protein peaks between 92 patients with a survival more than 2 years and 52 patients with survival less than 2 years. There were two significantly different protein peaks between 45 stage II patients with a survival more than 2 years and 14 stage II patients with survival less than 2 years. There was one significantly different protein peak between 22 stage III patients with a survival more than 2 years and 29 stage III patients with survival less than 2 years. Conclusion: Differences of serum proteins in esophageal squamous cell carcinoma are related to prognosis of patients. The protein fingerprint can be helpful for clinical diagnosis and treatment.

C1 [Wang, Xiao-Hong; Yu, Xin-Min] Zhejiang Canc Hosp, Dept Chemotherapy, Hangzhou, Zhejiang, Peoples R China.

[Zheng, Zhi-Guo] Zhejiang Canc Inst, Hangzhou, Zhejiang, Peoples R China.

[Jiang, Hong] Zhejiang Canc Hosp, Key Lab Thorac Tumor Diag Esophagus Lung & Treatm, Dept Thorac Surg, Hangzhou, Zhejiang, Peoples R China.

RP Wang, XH (corresponding author), Zhejiang Canc Hosp, Dept Chemotherapy, Hangzhou, Zhejiang, Peoples R China.

EM jhmecn@163.com

CR Breton J, 2008, J PROTEOME RES, V7, P1953, DOI 10.1021/pr7007835

Cadron I, 2009, ANTICANCER RES, V29, P1039

Calvano CD, 2010, J PHARMACEUT BIOMED, V51, P907, DOI 10.1016/j.jpba.2009.10.014

Grizzle WE, 2004, UROL ONCOL-SEMIN ORI, V22, P337, DOI 10.1016/j.urolonc.2004.04.008

Guo RY, 2011, J CANCER RES CLIN, V137, P513, DOI 10.1007/s00432-010-0899-3

Hammoud ZT, 2007, ANN THORAC SURG, V84, P384, DOI 10.1016/j.athoracsur.2007.03.088

Kohli Manish, 2006, Cancer Biomarkers, V2, P249

Langbein S, 2006, TECHNOL CANCER RES T, V5, P67, DOI 10.1177/153303460600500109

Liu LH, 2010, CLIN CHEM LAB MED, V48, P855, DOI 10.1515/CCLM.2010.138

Malik G, 2007, PROSTATE, V67, P1565, DOI 10.1002/pros.20646

McLerran D, 2008, CLIN CHEM, V54, P53, DOI 10.1373/clinchem.2007.091496

Panicker G, 2009, J PROTEOMICS, V71, P637, DOI 10.1016/j.jprot.2008.11.004

Parkin DM, 2005, CA-CANCER J CLIN, V55, P74, DOI 10.3322/canjclin.55.2.74

Piyathilake CJ, 2006, CANCER INFORM, V2, P345

Ren H, 2006, WORLD J GASTROENTERO, V12, P2789, DOI 10.3748/wjg.v12.i17.2789

Ricolleau G, 2006, PROTEOMICS, V6, P1963, DOI 10.1002/pmic.200500283

Xia Ting, 2008, Ai Zheng, V27, P279

Xu SY, 2009, BIOMARKERS, V14, P340, DOI 10.1080/13547500902903055

Xu Wen-hong, 2006, J Zhejiang Univ Sci B, V7, P235, DOI 10.1631/jzus.2006.B0235

Yu JS, 2005, BIOINFORMATICS, V21, P2200, DOI 10.1093/bioinformatics/bti370

Zhang Chang-ming, 2011, Zhonghua Yi Xue Za Zhi, V91, P171

Zhang H, 2006, GYNECOL ONCOL, V102, P61, DOI 10.1016/j.ygyno.2005.11.029

NR 22

TC 2

Z9 2

U1 0

U2 1

PU ASIAN PACIFIC ORGANIZATION CANCER PREVENTION

PI GYEONGGI-DO

PA APJCP HEAD OFFICE, KOREAN NATL CANCER CENTER, 323 ILAN -RO,

ILSANDONG-GU, GOYANG-SI, GYEONGGI-DO, 410-769, SOUTH KOREA

SN 1513-7368

J9 ASIAN PAC J CANCER P

JI Asian Pac. J. Cancer Prev.

PY 2012

VL 13

IS 4

BP 1579

EP 1582

DI 10.7314/APJCP.2012.13.4.1579

PG 4

WC Oncology

WE Science Citation Index Expanded (SCI-EXPANDED)

SC Oncology

GA 981DA

UT WOS:000306945700084

PM 22799370

OA gold, Green Submitted

DA 2022-05-19

ER

PT J

AU Wang, XJ

Zhou, LF

Zhang, HY

Ou, H

Long, WX

Liu, XB

AF Wang, Xiaojun

Zhou, Liangfen

Zhang, Huiyun

Ou, Hui

Long, Wenxing

Liu, Xiaobao

TI Upregulation of cervical carcinoma expressed PCNA regulatory long

non-coding RNA promotes esophageal squamous cell carcinoma progression

SO ONCOLOGY LETTERS

LA English

DT Article

DE esophageal squamous cell carcinoma; long non-coding RNA; cervical

carcinoma expressed PCNA regulatory long non-coding RNA; prognosis

ID CANCER STATISTICS; POOR-PROGNOSIS; LNCRNA CCHE1; CARCINOGENESIS;

PROLIFERATION; METASTASIS; APOPTOSIS

AB Cervical carcinoma expressed PCNA regulatory long non-coding (lnc)RNA (CCEPR) has recently been reported to play oncogenic roles in some common types of human cancer. However, the clinical significance of CCEPR mRNA expression levels in esophageal squamous cell carcinoma (ESCC) and the exact function of CCEPR in regulating the malignant phenotypes of ESCC cells have not been previously investigated. In the present study, CCEPR mRNA expression level was upregulated in ESCC tissues and cell lines, and overexpression of CCEPR was associated with advanced TNM stage, lymph node metastasis, and poor prognosis in ESCC. In vitro experiments showed that silencing CCEPR mRNA expression levels significantly suppressed the proliferation, migration, and invasion of ESCC cells, while inducing ESCC cell apoptosis. Furthermore, inhibition of CCEPR decreased the protein expression levels of matrix metalloproteinase (MMP)2 and MMP9 and inhibited epithelial-mesenchymal transition in ESCC cells. In conclusion, the results showed that CCEPR plays an oncogenic role in ESCC and suggests that CCEPR could be used as a potential therapeutic target for ESCC treatment.

C1 [Wang, Xiaojun; Zhang, Huiyun; Ou, Hui] First Peoples Hosp Chenzhou City, Dept Oncol, 8 Qingnian Rd, Chenzhou 423000, Hunan, Peoples R China.

[Zhou, Liangfen] First Peoples Hosp Chenzhou City, Dept Neonatol, Chenzhou 423000, Hunan, Peoples R China.

[Long, Wenxing] Xiangnan Coll, Affiliated Hosp, Dept Invas Technol, Chenzhou 423000, Hunan, Peoples R China.

[Liu, Xiaobao] Second Peoples Hosp Chenzhou City, Dept Oncol, Chenzhou 423000, Hunan, Peoples R China.

RP Wang, XJ (corresponding author), First Peoples Hosp Chenzhou City, Dept Oncol, 8 Qingnian Rd, Chenzhou 423000, Hunan, Peoples R China.

EM 563060314@qq.com

CR Al-Rugeebah A, 2019, PATHOL ONCOL RES, V25, P859, DOI 10.1007/s12253-019-00614-3

Bo H, 2019, ONCOL REP, V41, P2431, DOI 10.3892/or.2019.7027

Chen WL, 2017, MOL NEUROBIOL, V54, P7551, DOI 10.1007/s12035-017-0581-8

Chen Y, 2017, EUR REV MED PHARMACO, V21, P479

Cui ZH, 2019, J GENE MED, V21, DOI 10.1002/jgm.3083

Dahal U, 2019, MELANOMA RES, V29, P382, DOI 10.1097/CMR.0000000000000580

Dong ZM, 2019, CLIN EXP METASTAS, V36, P225, DOI 10.1007/s10585-019-09964-3

Gaballah HH, 2019, MOL BIOL REP, V46, P657, DOI 10.1007/s11033-018-4521-0

He YL, 2019, INT J BIOL MACROMOL, V125, P116, DOI 10.1016/j.ijbiomac.2018.11.273

Hua WF, 2018, GYNECOL ONCOL, V151, P356, DOI 10.1016/j.ygyno.2018.09.015

Hua Y, 2017, ONCOL REP, V38, P3030, DOI 10.3892/or.2017.5995

Huang ZL, 2017, ONCOL REP, V37, P3093, DOI 10.3892/or.2017.5568

Liang R, 2018, BIOMED PHARMACOTHER, V107, P1434, DOI 10.1016/j.biopha.2018.08.099

Liao Y, 2018, EUR REV MED PHARMACO, V22, P1686, DOI 10.26355/eurrev_201803_14581

Livak KJ, 2001, METHODS, V25, P402, DOI 10.1006/meth.2001.1262

Lv DS, 2016, TUMOR BIOL, V37, P16259, DOI 10.1007/s13277-016-5426-y

Parkin DM, 2005, CA-CANCER J CLIN, V55, P74, DOI 10.3322/canjclin.55.2.74

Peng W, 2016, BIOMED PHARMACOTHER, V83, P450, DOI 10.1016/j.biopha.2016.06.056

Siegel RL, 2019, CA-CANCER J CLIN, V69, P7, DOI 10.3322/caac.21551

Siegel RL, 2015, CA-CANCER J CLIN, V65, P5, DOI [10.3322/caac.21254, 10.1136/bmj.g1502, 10.1001/jamaoto.2014.2530, 10.3322/caac.21208]

Singh M, 2018, PHARMACOL THERAPEUT, V182, P80, DOI 10.1016/j.pharmthera.2017.08.009

Xia XL, 2018, ONCOL LETT, V15, P4105, DOI 10.3892/ol.2018.7815

Xu G, 2018, EUR REV MED PHARMACO, V22, P2631, DOI 10.26355/eurrev_201805_14957

Xu Y, 2017, DIS ESOPHAGUS, V30, DOI 10.1111/dote.12436

Yang W, 2018, CANCER BIOMARK, V23, P589, DOI 10.3233/CBM-181863

Yu XF, 2017, BMC CANCER, V17, DOI 10.1186/s12885-017-3442-y

Zhan YH, 2017, ONCOTARGET, V8, P44326, DOI 10.18632/oncotarget.17872

Zhang C, 2019, J CELL BIOCH

Zhang J, 2014, PLOS ONE, V9, DOI 10.1371/journal.pone.0109318

Zhang SX, 2019, BIOCHEM BIOPH RES CO, V513, P73, DOI 10.1016/j.bbrc.2019.03.012

Zhang YL, 2019, AM J TRANSL RES, V11, P1084

Zhang ZW, 2018, GENE, V650, P60, DOI 10.1016/j.gene.2018.01.096

Zhao M, 2018, NEUROSCIENCE, V390, P231, DOI 10.1016/j.neuroscience.2018.08.029

Zhu YX, 2019, J CELL MOL MED, V23, P93, DOI 10.1111/jcmm.13887

NR 34

TC 1

Z9 1

U1 1

U2 2

PU SPANDIDOS PUBL LTD

PI ATHENS

PA POB 18179, ATHENS, 116 10, GREECE

SN 1792-1074

EI 1792-1082

J9 ONCOL LETT

JI Oncol. Lett.

PD NOV

PY 2020

VL 20

IS 5

AR 142

DI 10.3892/ol.2020.12006

PG 9

WC Oncology

WE Science Citation Index Expanded (SCI-EXPANDED)

SC Oncology

GA OS4ZJ

UT WOS:000590173300023

PM 32934710

OA Green Published, gold

DA 2022-05-19

ER

PT J

AU Yang, N

Tian, JB

Wang, XY

Mei, SF

Zou, DY

Peng, XT

Zhu, Y

Yang, Y

Gong, YJ

Ke, JT

Zhong, R

Chang, J

Miao, XP

AF Yang, Nan

Tian, Jianbo

Wang, Xiaoyang

Mei, Shufang

Zou, Danyi

Peng, Xiating

Zhu, Ying

Yang, Yang

Gong, Yajie

Ke, Juntao

Zhong, Rong

Chang, Jiang

Miao, Xiaoping

TI A functional variant in TNXB promoter associates with the risk of

esophageal squamous-cell carcinoma

SO MOLECULAR CARCINOGENESIS

LA English

DT Article

DE esophageal squamous-cell carcinoma (ESCC); rs411337; single-nucleotide

polymorphism (SNP); TNXB

ID GENOME-WIDE ASSOCIATION; EHLERS-DANLOS-SYNDROME; CANCER-RISK;

SUSCEPTIBILITY LOCI; GENE-EXPRESSION; TENASCIN-X; IDENTIFICATION;

EPIDEMIOLOGY; BREAST

AB Our previous study identified a tag single-nucleotide polymorphism (SNP) rs204900 in TNXB associated with risk of esophageal squamous-cell carcinoma (ESCC) in the Chinese population. However, the functional role of TNXB and causal variants had not been interrogated in that study. In the present study, we explored the effects of TNXB expression in the development of ESCC and searched for functional variants in this gene. We found TNXB was downregulated in ESCC tumors. Using small interfering RNAs and CRISPR-Cas9 methods, we identified that both knockdown and knockout of TNXB significantly promoted ESCC cell growth in vitro, suggesting a tumor suppressor role of this gene in ESCC. Through further fine-mapping analysis, we identified that a noncoding variant in the promoter of TNXB, rs411337, predisposed to ESCC risk (odds ratio = 1.36, 95% confidence interval: 1.22-1.51, P = 9.10 x 10(-9)). These findings revealed the functional mechanism of TNXB in the development of ESCC and may contribute to the prevention and treatment of this disease in the future.

C1 [Yang, Nan; Tian, Jianbo; Wang, Xiaoyang; Mei, Shufang; Zou, Danyi; Peng, Xiating; Zhu, Ying; Yang, Yang; Gong, Yajie; Ke, Juntao; Zhong, Rong; Chang, Jiang; Miao, Xiaoping] Huazhong Univ Sci & Technol, Tongji Med Coll, Dept Epidemiol & Biostat, Wuhan, Peoples R China.

RP Chang, J; Miao, XP (corresponding author), Huazhong Univ Sci & Technol, Tongji Med Coll, Sch Publ Hlth, Key Lab Environm & Hlth,Minist Educ, Wuhan 430000, Peoples R China.

EM changjiang815@hust.edu.cn; miaoxp@hust.edu.cn

RI Chang, Jiang/AAP-6156-2020; miao, xiaoping/C-4336-2011

OI miao, xiaoping/0000-0002-6818-9722

FU Young Elite Scientist Sponsorship Program by CAST [2018QNRC001];

National Key Research and Development Plan Program [2016YFC1302702,

2016YFC1302703]; National Natural Science Foundation of China [81872696]

FX Young Elite Scientist Sponsorship Program by CAST, Grant/Award Number:

2018QNRC001; National Key Research and Development Plan Program,

Grant/Award Numbers: 2016YFC1302702, 2016YFC1302703; National Natural

Science Foundation of China, Grant/Award Number: 81872696 to J.C.

CR Abnet CC, 2018, GASTROENTEROLOGY, V154, P360, DOI 10.1053/j.gastro.2017.08.023

Ardlie KG, 2015, SCIENCE, V348, P648, DOI 10.1126/science.1262110

Blot W J, 1985, Natl Cancer Inst Monogr, V69, P29

Chang J, 2018, NAT GENET, V50, P338, DOI 10.1038/s41588-018-0045-8

Chang J, 2017, NAT COMMUN, V8, DOI 10.1038/ncomms15290

Chen WQ, 2016, CA-CANCER J CLIN, V66, P115, DOI 10.3322/caac.21338

Chen WY, 2016, HUM MUTAT, V37, P893, DOI 10.1002/humu.23028

Cheung WY, 2009, GASTROENTEROL CLIN N, V38, P75, DOI 10.1016/j.gtc.2009.01.009

Chiquet-Ehrismann R, 2011, CSH PERSPECT BIOL, V3, DOI 10.1101/cshperspect.a004960

Cowper-Sal-lari R, 2012, NAT GENET, V44, P1191, DOI 10.1038/ng.2416

Cui R, 2009, GASTROENTEROLOGY, V137, P1768, DOI 10.1053/j.gastro.2009.07.070

Engel LS, 2003, J NATL CANCER I, V95, P1404, DOI 10.1093/jnci/djg047

Guo HY, 2016, NAT GENET, V48, P1142, DOI 10.1038/ng.3637

Hamidi A, 2017, SCI SIGNAL, V10, DOI 10.1126/scisignal.aal4186

Hongo M, 2009, J GASTROEN HEPATOL, V24, P729, DOI 10.1111/j.1440-1746.2009.05824.x

Hsu MK, 2015, ONCOTARGET, V6, P28755, DOI 10.18632/oncotarget.4810

Hu N, 2010, BMC GENOMICS, V11, DOI 10.1186/1471-2164-11-576

Hu XF, 2009, OMICS, V13, P291, DOI 10.1089/omi.2009.0016

Huang DW, 2009, NUCLEIC ACIDS RES, V37, P1, DOI 10.1093/nar/gkn923

Ishiguro S, 2009, CANCER LETT, V275, P240, DOI 10.1016/j.canlet.2008.10.020

Kim YS, 2010, BMC CANCER, V10, DOI 10.1186/1471-2407-10-576

Levy P, 2007, CLIN CANCER RES, V13, P398, DOI 10.1158/1078-0432.CCR-06-0182

Livak KJ, 2001, METHODS, V25, P402, DOI 10.1006/meth.2001.1262

Mao JR, 2002, NAT GENET, V30, P421, DOI 10.1038/ng850

Maurano MT, 2012, SCIENCE, V337, P1190, DOI 10.1126/science.1222794

Men CD, 2017, J GYNECOL ONCOL, V28, DOI 10.3802/jgo.2017.28.e83

Samur MK, 2014, PLOS ONE, V9, DOI 10.1371/journal.pone.0106397

Sanchez-Antolin G, 2015, PLOS ONE, V10, DOI 10.1371/journal.pone.0137128

Su H, 2011, CLIN CANCER RES, V17, P2955, DOI 10.1158/1078-0432.CCR-10-2724

Ward LD, 2016, NUCLEIC ACIDS RES, V44, pD877, DOI 10.1093/nar/gkv1340

Wu C, 2014, NAT GENET, V46, P1001, DOI 10.1038/ng.3064

Wu C, 2012, NAT GENET, V44, P1090, DOI 10.1038/ng.2411

Wu C, 2011, NAT GENET, V43, P679, DOI 10.1038/ng.849

Yao LJ, 2014, NAT COMMUN, V5, DOI 10.1038/ncomms6114

Yuan Y, 2009, AM J SURG PATHOL, V33, P1673, DOI 10.1097/PAS.0b013e3181b6bde3

Zendehdel K, 2008, INT J CANCER, V122, P1095, DOI 10.1002/ijc.23076

Zhang XY, 2012, GENOME RES, V22, P1437, DOI 10.1101/gr.135665.111

Zhu J, 2008, NAT GENET, V40, P854, DOI 10.1038/ng.167

NR 38

TC 3

Z9 3

U1 1

U2 4

PU WILEY

PI HOBOKEN

PA 111 RIVER ST, HOBOKEN 07030-5774, NJ USA

SN 0899-1987

EI 1098-2744

J9 MOL CARCINOGEN

JI Mol. Carcinog.

PD APR

PY 2020

VL 59

IS 4

BP 439

EP 446

DI 10.1002/mc.23166

EA FEB 2020

PG 8

WC Biochemistry & Molecular Biology; Oncology

WE Science Citation Index Expanded (SCI-EXPANDED)

SC Biochemistry & Molecular Biology; Oncology

GA KR0WA

UT WOS:000513174100001

PM 32056283

DA 2022-05-19

ER

PT J

AU Wu, XL

Dinglin, XX

Wang, X

Luo, W

Shen, Q

Li, Y

Gu, L

Zhou, QH

Zhu, HT

Li, YJ

Tan, CD

Yang, XZ

Zhang, ZF

AF Wu, Xiaoliang

Dinglin, Xiaoxiao

Wang, Xing

Luo, Wen

Shen, Qi

Li, Yong

Gu, Ling

Zhou, Qianghua

Zhu, Haotu

Li, Yanjie

Tan, Chaodi

Yang, Xianzi

Zhang, Zhenfeng

TI Long noncoding RNA XIST promotes malignancies of esophageal squamous

cell carcinoma via regulation of miR-101/EZH2

SO ONCOTARGET

LA English

DT Article

DE long non-coding RNA; XIST; EZH2; esophageal squamous cell carcinoma; EMT

ID X-CHROMOSOME INACTIVATION; CANCER STATISTICS; LUNG-CANCER; EZH2;

PROLIFERATION; EXPRESSION; MIGRATION; PRC2

AB The long non-coding RNA XIST is a long non-coding RNA that associates with polycomb repressive complex 2 to regulate X-chromosome inactivation in female mammals. The biological roles as well as the underlying mechanisms of XIST in esophageal squamous cell carcinoma remained yet to be solved. Our data indicated that XIST was significantly upregulated in esophageal squamous cancerous tissues and cancer cell lines, as compared with that in the corresponding non-cancerous tissues and immortalized normal squamous epithelial cells. High XIST expression predicted poor prognosis of esophageal squamous cancer patients. Lentivirus mediated knockdown of XIST inhibited proliferation, migration and invasion of esophageal squamous cancer cells in vitro and suppressed tumor growth in vivo. Knockdown of XIST resulted in elevated expression of miR-101 and decreased expression of EZH2. Further analysis showed that XIST functioned as the competitive endogenous RNA of miR-101 to regulate EZH2 expression. Moreover, enforced expression of EZH2 significantly attenuated the anti-proliferation activity upon XIST knockdown. Conclusively, XIST plays an important role in malignant progression of ESCC via modulation of miR-101/EZH2 axis.

C1 [Wu, Xiaoliang; Luo, Wen; Shen, Qi; Li, Yong] Guizhou Prov Peoples Hosp, Dept Oncol, Guiyang, Guizhou, Peoples R China.

[Wu, Xiaoliang; Wang, Xing; Gu, Ling; Zhou, Qianghua; Zhu, Haotu; Zhang, Zhenfeng] Sun Yat Sen Univ, Canc Ctr, State Key Lab Oncol South China, Guangzhou, Guangdong, Peoples R China.

[Wu, Xiaoliang; Wang, Xing; Gu, Ling; Zhou, Qianghua; Zhu, Haotu; Zhang, Zhenfeng] Sun Yat Sen Univ, Canc Ctr, Collaborat Innovat Ctr Canc Med, Guangzhou, Guangdong, Peoples R China.

[Dinglin, Xiaoxiao] Sun Yat Sen Univ, Sun Yat Sen Mem Hosp, Dept Med Oncol, Guangzhou, Guangdong, Peoples R China.

[Gu, Ling] Puer Univ, Puer, Peoples R China.

[Li, Yanjie] Sun Yat Sen Univ, Affiliated Hosp 3, Guangzhou, Guangdong, Peoples R China.

[Tan, Chaodi] Sun Yat Sen Univ, Guangzhou, Guangdong, Peoples R China.

[Yang, Xianzi] Guangzhou Med Univ, Affiliated Canc Hosp & Inst, Guangzhou, Guangdong, Peoples R China.

[Zhang, Zhenfeng] Guangzhou Med Univ, Affiliated Hosp 2, Dept Radiol, Guangzhou, Guangdong, Peoples R China.

RP Zhang, ZF (corresponding author), Sun Yat Sen Univ, Canc Ctr, State Key Lab Oncol South China, Guangzhou, Guangdong, Peoples R China.; Zhang, ZF (corresponding author), Sun Yat Sen Univ, Canc Ctr, Collaborat Innovat Ctr Canc Med, Guangzhou, Guangdong, Peoples R China.; Yang, XZ (corresponding author), Guangzhou Med Univ, Affiliated Canc Hosp & Inst, Guangzhou, Guangdong, Peoples R China.; Zhang, ZF (corresponding author), Guangzhou Med Univ, Affiliated Hosp 2, Dept Radiol, Guangzhou, Guangdong, Peoples R China.

EM yangxz@mail2.sysu.edu.cn; zhangzhf@gzhmu.edu.cn

RI Wu, Xiaoliang/S-4946-2019

FU National Natural Science Foundation of China [81672276, 81372274,

81461168028]; Natural Science Foundation of Guangdong Province, China

[2014A030313033, 2014A050503037]; International Program for Ph.D.

Candidates, Sun Yat-sen University; Guizhou Provincial People's Hospital

Youth Funding [GZSQN [2016]20]

FX This work was supported by grants from National Natural Science

Foundation of China (No. 81672276, 81372274, and 81461168028) and

Natural Science Foundation of Guangdong Province, China (No.

2014A030313033 and 2014A050503037). Xiaoliang Wu was supported by

International Program for Ph.D. Candidates, Sun Yat-sen University and

Guizhou Provincial People's Hospital Youth Funding (No. GZSQN [2016]20).

CR Nieto MA, 2016, CELL, V166, P21, DOI 10.1016/j.cell.2016.06.028

Batista PJ, 2013, CELL, V152, P1298, DOI 10.1016/j.cell.2013.02.012

Briggs SF, 2014, CURR OPIN GENET DEV, V28, P78, DOI 10.1016/j.gde.2014.09.010

Casale V, 1999, J EXP CLIN CANC RES, V18, P63

Cerase A, 2015, GENOME BIOL, V16, DOI 10.1186/s13059-015-0733-y

Chen DL, 2016, J EXP CLIN CANC RES, V35, DOI 10.1186/s13046-016-0420-1

Chen WQ, 2016, CA-CANCER J CLIN, V66, P115, DOI 10.3322/caac.21338

Clemson CM, 1996, J CELL BIOL, V132, P259, DOI 10.1083/jcb.132.3.259

D'Angelo V, 2015, J EXP CLIN CANC RES, V34, DOI 10.1186/s13046-015-0191-0

Debeb BG, 2014, J EXP CLIN CANC RES, V33, DOI 10.1186/s13046-014-0058-9

Demeester Steven R, 2009, Gastrointest Cancer Res, V3, pS2

Fang J, 2016, BIOCHEM BIOPH RES CO, V478, P811, DOI 10.1016/j.bbrc.2016.08.030

Hanahan D, 2011, CELL, V144, P646, DOI 10.1016/j.cell.2011.02.013

Huang SD, 2012, MOL CANCER, V11, DOI 10.1186/1476-4598-11-51

Huang YS, 2016, ONCOTARGET, V7, P43256, DOI 10.18632/oncotarget.9673

Jeon Y, 2011, CELL, V146, P119, DOI 10.1016/j.cell.2011.06.026

Kottakis F, 2011, MOL CELL, V43, P285, DOI 10.1016/j.molcel.2011.06.020

Lee JT, 2011, NAT REV MOL CELL BIO, V12, P815, DOI 10.1038/nrm3231

Lin C, 2014, INT J CLIN EXP PATHO, V7, P6543

Markar SR, 2015, LANCET ONCOL, V16, pE23, DOI 10.1016/S1470-2045(14)70419-X

McHugh CA, 2015, NATURE, V521, P232, DOI 10.1038/nature14443

Payer B, 2008, ANNU REV GENET, V42, P733, DOI 10.1146/annurev.genet.42.110807.091711

Rustgi AK, 2014, NEW ENGL J MED, V371, P2499, DOI 10.1056/NEJMra1314530

Sarma K, 2014, CELL, V159, P869, DOI 10.1016/j.cell.2014.10.019

Schmitt AM, 2016, CANCER CELL, V29, P452, DOI 10.1016/j.ccell.2016.03.010

Song HZ, 2012, J EXP CLIN CANC RES, V31, DOI 10.1186/1756-9966-31-35

Sun M, 2015, ENDOCR REV, V36, P25, DOI 10.1210/er.2014-1034

Torre LA, 2015, CA-CANCER J CLIN, V65, P87, DOI 10.3322/caac.21262

Wang BX, 2012, J EXP CLIN CANC RES, V31, DOI 10.1186/1756-9966-31-11

Wassef M, 2017, J MOL BIOL, V429, P1978, DOI 10.1016/j.jmb.2016.10.012

Wu JJ, 2015, J EXP CLIN CANC RES, V34, DOI 10.1186/s13046-015-0215-9

Wu XL, 2017, ONCOL RES TREAT, V40, P370, DOI 10.1159/000464465

Xu LB, 2014, J HEPATOL, V60, P590, DOI 10.1016/j.jhep.2013.10.028

Yamada A, 2011, INT J ONCOL, V38, P345, DOI 10.3892/ijo.2010.868

Yao YL, 2015, CANCER LETT, V359, P75, DOI 10.1016/j.canlet.2014.12.051

Yildirim E, 2013, CELL, V152, P727, DOI 10.1016/j.cell.2013.01.034

Zhao J, 2008, SCIENCE, V322, P750, DOI 10.1126/science.1163045

NR 37

TC 58

Z9 61

U1 2

U2 13

PU IMPACT JOURNALS LLC

PI ORCHARD PARK

PA 6666 E QUAKER ST, STE 1, ORCHARD PARK, NY 14127 USA

EI 1949-2553

J9 ONCOTARGET

JI Oncotarget

PD SEP 29

PY 2017

VL 8

IS 44

BP 76015

EP 76028

DI 10.18632/oncotarget.18638

PG 14

WC Oncology; Cell Biology

WE Science Citation Index Expanded (SCI-EXPANDED)

SC Oncology; Cell Biology

GA FI5ZG

UT WOS:000412066700021

PM 29100288

OA Green Published, gold

DA 2022-05-19

ER

PT J

AU Shen, TY

Mei, LL

Qiu, YT

Shi, ZZ

AF Shen, Tian-Yun

Mei, Li-Li

Qiu, Yun-Tan

Shi, Zhi-Zhou

TI Identification of candidate target genes of genomic aberrations in

esophageal squamous cell carcinoma

SO ONCOLOGY LETTERS

LA English

DT Article

DE esophageal squamous cell carcinoma; cyclin-dependent kinase inhibitor 2A

and 2B; fascin actin-bundling protein 1; homer scaffolding protein 3

ID CANCER; EXPRESSION; MIR-133A; SURVIVAL; FSCN1; RESTORATION; IMPACT;

GROWTH

AB The aim of the present study was to identify the candidate target genes of genomic aberrations in esophageal squamous cell carcinoma (ESCC). Array comparative genomic hybridization (CGH) and quantitative polymerase chain reaction were applied to analyze the copy number changes and expression level of candidate genes, respectively. Integrative analysis revealed that homozygous deletions of cyclin-dependent kinase inhibitor (CDKN) 2A and CDKN2B and gains of fascin actin-bundling protein 1 (FSCN1) and homer scaffolding protein 3 (HOMER3) occurred frequently in ESCC. The results demonstrated that the homozygous deletion of CDKN2A or CDKN2B was significantly associated with lymph node metastasis. Notably, the expression of CDKN2A and CDKN2B was lower in dysplasia than in normal esophageal epithelium. We also observed that the copy number increase of FSCN1 was significantly associated with pT, pN and pStage, and that the gain of HOMER3 was significantly linked with pN and pStage. We further revealed that FSCN1 and HOMER3 were overexpressed in ESCC, and that their overexpression was correlated with copy number increase. In conclusion, CDKN2A, CDKN2B, FSCN1 and HOMER3 are candidate cancer-associated genes and may play a tumorigenic role in ESCC.

C1 [Shen, Tian-Yun; Mei, Li-Li; Qiu, Yun-Tan; Shi, Zhi-Zhou] Kunming Univ Sci & Technol, Fac Med, 727 South Jingming Rd, Kunming 650500, Yunnan, Peoples R China.

RP Shi, ZZ (corresponding author), Kunming Univ Sci & Technol, Fac Med, 727 South Jingming Rd, Kunming 650500, Yunnan, Peoples R China.

EM zhizhoushi@126.com

OI Shen, Tianyun/0000-0003-4300-5349; Qiu, Yuntan/0000-0001-5653-7956

FU National Natural Science Foundation of China [81460425]; Yunnan

Provincial Research Foundation for Basic Research, China [2013FD012];

Foundation for the Talents of Kunming University of Science and

Technology [KKSY201226099]; Yunnan Provincial Engineering Center of

Translational Cancer Medicine [2011DH011]

FX This study was funded by the National Natural Science Foundation of

China (grant no. 81460425), the Yunnan Provincial Research Foundation

for Basic Research, China (grant no. 2013FD012), the Foundation for the

Talents of Kunming University of Science and Technology (grant no.

KKSY201226099) and Yunnan Provincial Engineering Center of Translational

Cancer Medicine (grant no. 2011DH011).

CR Akanuma N, 2014, BRIT J CANCER, V110, P189, DOI 10.1038/bjc.2013.676

Aravidis C, 2012, ANTICANCER RES, V32, P5309

Bortoloso E, 2006, AM J PHYSIOL-CELL PH, V290, pC711, DOI 10.1152/ajpcell.00217.2005

Chen MB, 2014, CELL SIGNAL, V26, P102, DOI 10.1016/j.cellsig.2013.07.017

Cheung CCM, 2014, MOL CANCER, V13, DOI 10.1186/1476-4598-13-184

Ferlay J, 2015, INT J CANCER, V136, pE359, DOI 10.1002/ijc.29210

Fuse M, 2011, INT J ONCOL, V38, P1093, DOI 10.3892/ijo.2011.919

Hanker LC, 2013, ANTICANCER RES, V33, P371

Hirasaki S, 2007, ONCOLOGIST, V12, P406, DOI 10.1634/theoncologist.12-4-406

Ishiguro K, 2004, BLOOD, V103, P2248, DOI 10.1182/blood-2003-08-2671

Kano M, 2010, INT J CANCER, V127, P2804, DOI 10.1002/ijc.25284

Kohonen-Corish MRJ, 2014, INT J CANCER, V134, P2820, DOI 10.1002/ijc.28619

Lee HS, 2014, ONCOTARGET, V5, P3472, DOI 10.18632/oncotarget.1927

Li Z, 2013, ASIAN PAC J CANCER P, V14, P2525, DOI 10.7314/APJCP.2013.14.4.2525

Liu R, 2012, PLOS ONE, V7, DOI 10.1371/journal.pone.0033987

Negrini S, 2010, NAT REV MOL CELL BIO, V11, P220, DOI 10.1038/nrm2858

Parkin DM, 2005, CA-CANCER J CLIN, V55, P74, DOI 10.3322/canjclin.55.2.74

Qin Y, 2013, ONCOL RES, V21, P353, DOI 10.3727/096504014X14024160459122

Riehmer V, 2014, GENE CHROMOSOME CANC, V53, P589, DOI 10.1002/gcc.22169

Shi ZZ, 2013, CLIN CANCER RES, V19, P5867, DOI 10.1158/1078-0432.CCR-12-3753

Shi ZZ, 2011, GENE CHROMOSOME CANC, V50, P518, DOI 10.1002/gcc.20875

Stirewalt DL, 2008, GENE CHROMOSOME CANC, V47, P8, DOI 10.1002/gcc.20500

Tam KW, 2013, J THORAC ONCOL, V8, P1378, DOI 10.1097/JTO.0b013e3182a46c0c

Togashi Y, 2014, ONCOTARGET, V5, P2962, DOI 10.18632/oncotarget.1561

Valk PJM, 2004, NEW ENGL J MED, V350, P1617, DOI 10.1056/NEJMoa040465

Wu ZS, 2012, BMC CANCER, V12, DOI 10.1186/1471-2407-12-51

Xiao B, 1998, NEURON, V21, P707, DOI 10.1016/S0896-6273(00)80588-7

Xing X, 2013, BRIT J CANCER, V108, P2542, DOI 10.1038/bjc.2013.251

Yen CC, 2001, CANCER, V92, P2769, DOI 10.1002/1097-0142(20011201)92:11<2769::AID-CNCR10118>3.0.CO;2-M

NR 29

TC 8

Z9 9

U1 1

U2 6

PU SPANDIDOS PUBL LTD

PI ATHENS

PA POB 18179, ATHENS, 116 10, GREECE

SN 1792-1074

EI 1792-1082

J9 ONCOL LETT

JI Oncol. Lett.

PD OCT

PY 2016

VL 12

IS 4

BP 2956

EP 2961

DI 10.3892/ol.2016.4947

PG 6

WC Oncology

WE Science Citation Index Expanded (SCI-EXPANDED)

SC Oncology

GA DZ1DN

UT WOS:000385579200111

PM 27698883

OA Green Published, gold

DA 2022-05-19

ER

PT J

AU Jin, L

Yi, J

Gao, YP

Han, SQ

He, ZY

Chen, LB

Song, HZ

AF Jin, Li

Yi, Jun

Gao, Yanping

Han, Siqi

He, Zhenyue

Chen, Longbang

Song, Haizhu

TI MiR-630 inhibits invasion and metastasis in esophageal squamous cell

carcinoma

SO ACTA BIOCHIMICA ET BIOPHYSICA SINICA

LA English

DT Article

DE miR-630; esophageal squamous cell carcinoma; suppressor;

epithelial-mesenchymal transition; invasion and metastasis

ID TO-MESENCHYMAL TRANSITION; BREAST-CANCER; LUNG-CANCER; PROLIFERATION;

SUPPRESSES; PROMOTES; PROGRESSION; EXPRESSION; REGULATOR; SLUG

AB Esophageal squamous cell carcinoma (ESCC) is among the most aggressive malignancies and has a high incidence in China. MicroRNAs (miRNAs) are small endogenous RNAs that regulate multiple tumorigenic processes, including proliferation, invasion, metastasis and prognosis. Using miRNA expression profiling analysis, we found that miR-630 was markedly down-regulated in three ESCC tissue samples compared with that in paired normal esophageal tissues. Differential miR-630 expression was subsequently confirmed using quantitative real-time PCR. To determine whether miR-630 down-regulation could be considered as a diagnostic indicator and adverse prognostic factor, we investigated the association between miR-630 and clinicopathological characteristics in patients with ESCC. It was found that decreased miR-630 expression was associated with poor overall survival in these patients. In addition, we also explored the biological function of miR-630 by targeting Slug and investigated the correlation between miR-630 expression and epithelial-mesenchymal transition (EMT) progression in vivo and in vitro. Ectopic miR-630 expression could inhibit proliferation, invasion and metastasis, whereas miR-630 knockdown induced proliferation, invasion, metastasis and EMT traits. Overall, our study supports a role for miR-630 as a critical novel modulator in ESCC.

C1 [Jin, Li; Gao, Yanping; Han, Siqi; He, Zhenyue; Chen, Longbang; Song, Haizhu] Jinling Hosp, Dept Med Oncol, Nanjing 210002, Jiangsu, Peoples R China.

[Yi, Jun] Jinling Hosp, Dept Cardiothorac Surg, Nanjing 210002, Jiangsu, Peoples R China.

RP Chen, LB; Song, HZ (corresponding author), Jinling Hosp, Dept Med Oncol, Nanjing 210002, Jiangsu, Peoples R China.

EM chenlongbang@yeah.net; songhaizhu@163.com

FU National Natural Science Foundation of China [81301914]; Six Big Talent

of Jiangsu Province

FX This work was supported by the grants from the National Natural Science

Foundation of China (No. 81301914) and Six Big Talent of Jiangsu

Province.

CR Allaya N, 2015, TUMOR BIOL, V36, P3807, DOI 10.1007/s13277-014-3022-6

Ansari MH, 2015, TUMOUR BIOL

Bakhshandeh B, 2012, J GENET, V91, P171, DOI 10.1007/s12041-012-0168-0

Belair Cedric, 2011, Silence, V2, P7, DOI 10.1186/1758-907X-2-7

Cao JX, 2014, CELL DEATH DIS, V5, DOI 10.1038/cddis.2014.386

Cao JX, 2014, BIOCHEM BIOPH RES CO, V450, P470, DOI 10.1016/j.bbrc.2014.05.138

Chen DQ, 2014, EUR J CANCER, V50, P3050, DOI 10.1016/j.ejca.2014.09.008

Chen WX, 2016, ONCOTARGET, V7, P22674, DOI 10.18632/oncotarget.8047

Chen ZL, 2011, J BIOL CHEM, V286, P10725, DOI 10.1074/jbc.M110.165654

Corcoran C, 2014, MOL CANCER, V13, DOI 10.1186/1476-4598-13-71

Dhasarathy A, 2011, PLOS ONE, V6, DOI 10.1371/journal.pone.0026514

Fang L, 2012, CELL CYCLE, V11, P4352, DOI 10.4161/cc.22670

Farhana L, 2013, PLOS ONE, V8, DOI 10.1371/journal.pone.0061015

Feng SJ, 2014, TUMOR BIOL, V35, P12339, DOI 10.1007/s13277-014-2546-0

Galluzzi L, 2010, CANCER RES, V70, P1793, DOI 10.1158/0008-5472.CAN-09-3112

Gasparini P, 2014, P NATL ACAD SCI USA, V111, P4536, DOI 10.1073/pnas.1402604111

Gebeshuber CA, 2009, EMBO REP, V10, P400, DOI 10.1038/embor.2009.9

Gregory PA, 2008, NAT CELL BIOL, V10, P593, DOI 10.1038/ncb1722

Hajra KM, 2002, CANCER RES, V62, P1613

Harada K, 2015, ANTICANCER RES, V35, P4617

Higashi T, 2015, BRIT J CANCER, V113, P252, DOI 10.1038/bjc.2015.170

Ke XS, 2003, CURR OPIN CHEM BIOL, V7, P516, DOI 10.1016/S1367-5931(03)00075-9

Keditsu KK, 2013, INDIA J SURG ONCOL, V4, P96, DOI 10.1007/s13193-013-0216-0

Kuo TC, 2013, J CLIN INVEST, V123, P1082, DOI 10.1172/JCI64044

Lee GD, 2015, ANN SURG, V262, P965, DOI 10.1097/SLA.0000000000001039

Li SJ, 2015, AM J TRANSL RES, V7, P1390

Liu FX, 2013, MOL CELL BIOCHEM, V381, P115, DOI 10.1007/s11010-013-1693-8

Loeffler I, 2015, CELLS-BASEL, V4, P631, DOI 10.3390/cells4040631

Melhado Rachel E, 2010, Cancers (Basel), V2, P1379, DOI 10.3390/cancers2031379

Mitamura T, 2014, MOL CANCER, V13, DOI 10.1186/1476-4598-13-97

Nandy SB, 2015, ONCOTARGET, V6, P17366, DOI 10.18632/oncotarget.3953

Pang LJ, 2016, SCI REP-UK, V6, DOI 10.1038/srep22179

Patnaik SK, 2012, PLOS ONE, V7, DOI 10.1371/journal.pone.0046045

Patnaik SK, 2010, CANCER RES, V70, P36, DOI 10.1158/0008-5472.CAN-09-3153

Ragusa M, 2012, J MOL MED, V90, P1421, DOI 10.1007/s00109-012-0918-8

Rustgi AK, 2014, NEW ENGL J MED, V371, P2499, DOI 10.1056/NEJMra1314530

Sakurai MA, 2014, PLOS ONE, V9, DOI 10.1371/journal.pone.0100124

Song YF, 2015, AM J TRANSL RES, V7, P1271

Taccioli C, 2015, JNCI-J NATL CANCER I, V107, DOI 10.1093/jnci/djv220

Tan GS, 2015, ONCOTARGET, V6, P8676, DOI 10.18632/oncotarget.3254

Zhang MJ, 2015, BIOCHEM BIOPH RES CO, V460, P806, DOI 10.1016/j.bbrc.2015.03.110

Zhang YQ, 2015, PLOS ONE, V10, DOI 10.1371/journal.pone.0120884

Zheng XF, 2015, NATURE, V527, P525, DOI 10.1038/nature16064

Zhou CX, 2016, ONCOTARGET, V7, P1288, DOI 10.18632/oncotarget.6339

NR 44

TC 29

Z9 31

U1 0

U2 7

PU OXFORD UNIV PRESS

PI OXFORD

PA GREAT CLARENDON ST, OXFORD OX2 6DP, ENGLAND

SN 1672-9145

EI 1745-7270

J9 ACTA BIOCH BIOPH SIN

JI Acta Biochim. Biophys. Sin.

PD SEP

PY 2016

VL 48

IS 9

BP 810

EP 819

DI 10.1093/abbs/gmw073

PG 10

WC Biochemistry & Molecular Biology; Biophysics

WE Science Citation Index Expanded (SCI-EXPANDED)

SC Biochemistry & Molecular Biology; Biophysics

GA DX5SK

UT WOS:000384441800005

PM 27563011

OA Bronze

DA 2022-05-19

ER

PT J

AU Li, J

Sang, MX

Zheng, Y

Meng, LJ

Gu, LN

Li, ZY

Liu, F

Wu, YY

Li, WJ

Shan, BE

AF Li, Juan

Sang, Meixiang

Zheng, Yang

Meng, Lingjiao

Gu, Lina

Li, Ziyi

Liu, Fei

Wu, Yunyan

Li, Weijing

Shan, Baoen

TI HNRNPUL1 inhibits cisplatin sensitivity of esophageal squamous cell

carcinoma through regulating the formation of circMAN1A2

SO EXPERIMENTAL CELL RESEARCH

LA English

DT Article

DE Esophageal squamous cell carcinoma; Cisplatin sensitivity; HNRNPUL1;

RNA-binding protein

ID NONCODING RNAS; DNA-REPAIR; CANCER; MECHANISMS; RESISTANCE; PROTEIN

AB Cisplatin (CDDP) is widely used for chemotherapy of esophageal squamous cell carcinoma (ESCC) but the drug resistance limits its therapeutic benefit. Heterogeneous nuclear ribonucleoprotein U-like 1 (HNRNPUL1) belongs to the family of RNA-binding proteins (RBPs) and is involved in DNA damage repair. To investigate whether and how HNRNPUL1 affects CDDP resistance of ESCC, we evaluated the expression of HNRNPUL1 and found that it was associated with recurrence in ESCC patients receiving postoperative platinum-based chemotherapy and was an independent prognostic factor for disease-free survival (DFS). Besides, we showed that the reduced expression of HNRNPUL1 enhanced the CDDP sensitivity of ESCC cells. Furthermore, RNA immunoprecipitation coupled with high-throughput sequencing (RIP-seq) were performed and a range of HNRNPUL1-binding RNAs influenced by CDDP treatment were identified followed by bioinformatics analysis. In terms of mechanism, we found that HNRNPUL1 inhibited CDDP sensitivity of ESCC cells by regulating the CDDP sensitivity-inhibited circular RNA (circRNA) MAN1A2 formation. Taken together, our results first demonstrated the role of HNRNPUL1 in CDDP resistance of ESCC and suggested that HNRNPUL1 may be a potential target of ESCC chemotherapy.

C1 [Li, Juan; Sang, Meixiang; Zheng, Yang; Meng, Lingjiao; Gu, Lina; Li, Ziyi; Liu, Fei; Wu, Yunyan; Shan, Baoen] Hebei Med Univ, Res Ctr, Affiliated Hosp 4, Shijiazhuang 050017, Hebei, Peoples R China.

[Li, Juan; Sang, Meixiang; Zheng, Yang; Meng, Lingjiao; Gu, Lina; Li, Ziyi; Liu, Fei; Wu, Yunyan; Shan, Baoen] Hebei Med Univ, Tumor Res Inst, Affiliated Hosp 4, Shijiazhuang 050017, Hebei, Peoples R China.

[Li, Weijing] Hebei Med Univ, Dept Anesthesiol, Affiliated Hosp 4, Shijiazhuang 050017, Hebei, Peoples R China.

RP Shan, BE (corresponding author), Hebei Med Univ, Res Ctr, Affiliated Hosp 4, Shijiazhuang 050017, Hebei, Peoples R China.; Shan, BE (corresponding author), Hebei Med Univ, Tumor Res Inst, Affiliated Hosp 4, Shijiazhuang 050017, Hebei, Peoples R China.

EM baoenshan@hotmail.com

FU Medical Science Research Key Project of Hebei Provincial Health

Department [:20180532]

FX This study was supported by the Medical Science Research Key Project of

Hebei Provincial Health Department (NO:20180532).

CR Alfano L, 2019, NUCLEIC ACIDS RES, V47, P4068, DOI 10.1093/nar/gkz076

Bonanno L, 2014, ANTICANCER RES, V34, P493

Cerasuolo A, 2020, FRONT CELL DEV BIOL, V8, DOI 10.3389/fcell.2020.00474

Chen Q, 2020, MOL THER, V28, P2503, DOI 10.1016/j.ymthe.2020.07.001

Conn SJ, 2015, CELL, V160, P1125, DOI 10.1016/j.cell.2015.02.014

Dasari S, 2014, EUR J PHARMACOL, V740, P364, DOI 10.1016/j.ejphar.2014.07.025

Fei T, 2017, P NATL ACAD SCI USA, V114, pE5207, DOI 10.1073/pnas.1617467114

Fredericks AM, 2015, BIOMOLECULES, V5, P893, DOI 10.3390/biom5020893

Gabler S, 1998, J VIROL, V72, P7960, DOI 10.1128/JVI.72.10.7960-7971.1998

Geuens T, 2016, HUM GENET, V135, P851, DOI 10.1007/s00439-016-1683-5

Guo YX, 2019, ONCOL REP, V42, P2716, DOI 10.3892/or.2019.7347

Gurunathan G, 2015, SCI REP-UK, V5, DOI 10.1038/srep10475

Han SP, 2010, BIOCHEM J, V430, P379, DOI 10.1042/BJ20100396

Hong ZH, 2013, PLOS ONE, V8, DOI 10.1371/journal.pone.0060208

Huang XX, 2019, MOL CANCER, V18, DOI 10.1186/s12943-019-0969-3

Jang R, 2015, J NATL COMPR CANC NE, V13, P229, DOI 10.6004/jnccn.2015.0029

Jeyaraman S, 2020, FRONT GENET, V10, DOI 10.3389/fgene.2019.01369

Jiang HJ, 2017, CELL DEATH DIS, V8, DOI 10.1038/cddis.2017.258

Koberle B, 2010, BBA-REV CANCER, V1806, P172, DOI 10.1016/j.bbcan.2010.07.004

Kramer MC, 2015, GENE DEV, V29, P2168, DOI 10.1101/gad.270421.115

Kuo IY, 2019, J BIOMED SCI, V26, DOI 10.1186/s12929-019-0510-4

Lee SJ, 2018, MOL CELL PROTEOMICS, V17, P2091, DOI 10.1074/mcp.RA118.000603

Lo Piccolo L, 2014, CHROMOSOMA, V123, P515, DOI 10.1007/s00412-014-0470-0

McHugh PJ, 2001, LANCET ONCOL, V2, P483, DOI 10.1016/S1470-2045(01)00454-5

Olaussen KA, 2006, NEW ENGL J MED, V355, P983, DOI 10.1056/NEJMoa060570

Polo SE, 2012, MOL CELL, V45, P505, DOI 10.1016/j.molcel.2011.12.035

Qin H, 2020, J HEMATOL ONCOL, V13, DOI 10.1186/s13045-020-00927-w

Sarmento-Ribeiro AB, 2019, DRUG RESIST UPDATE, V47, DOI 10.1016/j.drup.2019.100646

Siddik ZH, 2003, ONCOGENE, V22, P7265, DOI 10.1038/sj.onc.1206933

Slack FJ, 2019, CELL, V179, P1033, DOI 10.1016/j.cell.2019.10.017

Smyth EC, 2017, NAT REV DIS PRIMERS, V3, DOI 10.1038/nrdp.2017.48

Sun XH, 2017, BIOCHEM J, V474, P2925, DOI 10.1042/BCJ20170280

Takeiwa T, 2020, INT J MOL SCI, V21, DOI 10.3390/ijms21051551

Torre LA, 2015, CA-CANCER J CLIN, V65, P87, DOI 10.3322/caac.21262

Wang WJ, 2017, CLIN CANCER RES, V23, P503, DOI 10.1158/1078-0432.CCR-15-1169

Wang WT, 2019, J HEMATOL ONCOL, V12, DOI 10.1186/s13045-019-0748-z

Zang JK, 2020, J NEUROSCI RES, V98, P87, DOI 10.1002/jnr.24356

Zeng HM, 2016, THORAC CANCER, V7, P232, DOI 10.1111/1759-7714.12322

Zhang K, 2020, J CANCER, V11, P301, DOI 10.7150/jca.33983

Zhou T, 2020, THORAC CANCER, V11, P3071, DOI 10.1111/1759-7714.13142

Zou FW, 2020, INT J ONCOL, V57, P151, DOI 10.3892/ijo.2020.5050

NR 41

TC 0

Z9 0

U1 6

U2 6

PU ELSEVIER INC

PI SAN DIEGO

PA 525 B STREET, STE 1900, SAN DIEGO, CA 92101-4495 USA

SN 0014-4827

EI 1090-2422

J9 EXP CELL RES

JI Exp. Cell Res.

PD DEC 1

PY 2021

VL 409

IS 1

AR 112891

DI 10.1016/j.yexcr.2021.112891

EA NOV 2021

PG 12

WC Oncology; Cell Biology

WE Science Citation Index Expanded (SCI-EXPANDED)

SC Oncology; Cell Biology

GA XG3JY

UT WOS:000724653600001

PM 34688610

DA 2022-05-19

ER

PT J

AU Xia, L

Liang, W

Que, D

Xie, QC

AF Xia, Lei

Liang, Wei

Que, Dan

Xie, Qichao

TI Correlation Analysis of circRNA Circ_0071662 in Diagnosis and Prognosis

of Esophageal Squamous Cell Carcinoma

SO INTERNATIONAL JOURNAL OF GENERAL MEDICINE

LA English

DT Article

DE esophageal squamous cell carcinoma; circ_0071662; diagnosis; prognosis

ID CANCER; EPIDEMIOLOGY; SURVIVAL

AB Background: The role of circRNA circ_0071662 has been studied in bladder cancer. The present study aimed to analyze its involvement in esophageal squamous cell carcinoma (ESCC).

Methods: Patients with ESCC (n = 66), esophageal ulcer (EU, n = 66), or gastroesophageal reflux disease (GERD, n = 66) and healthy controls (n = 66) were enrolled in this study. Plasma samples were collected from all patients and controls. ESCC and paired non-tumor tissue samples were collected from ESCC patients. Circ_0071662 levels in these samples were determined by RT-qPCRs. Diagnostic and prognostic values of circ_0071662 for ESCC were analyzed with ROC curve and survival curve analyses.

Results: Circ_0071662 level was decreased in ESCC, but not in GERN and EU compared to the controls and in ESCC tissues compared to the non-tumor tissues. Plasma circ_0071662 was closely correlated with patients' tumor size but not with other clinical features. Decreased plasma circ_0071662 levels separated ESCC patients from GERN patients, EU patients, and healthy controls. Low plasma circ_0071662 levels were closely correlated with worse survival outcomes of ESCC patients.

Conclusion: Circ_0071662 is lowly expressed in ESCC and may serve as a potential diagnostic and prognostic biomarker for ESCC.

C1 [Xia, Lei] Chongqing Med Univ, Affiliated Hosp 2, Canc Ctr, Chongqing 400000, Peoples R China.

[Liang, Wei; Que, Dan; Xie, Qichao] Chongqing Med Univ, Affiliated Hosp 3, Dept Oncol, Chongqing 400000, Peoples R China.

RP Xie, QC (corresponding author), Chongqing Med Univ, Affiliated Hosp 3, Dept Oncol, Chongqing 400000, Peoples R China.

EM qichaoxieyubei@163.com

FU Chongqing Science and Health Combined Medical Research Project

[2019MSXM024]

FX The study was supported by the Chongqing Science and Health Combined

Medical Research Project (No. 2019MSXM024).

CR Abnet CC, 2018, GASTROENTEROLOGY, V154, P360, DOI 10.1053/j.gastro.2017.08.023

Abulizi Rena, 2019, Oncol Res, DOI 10.3727/096504019X15740729375088

Chadwick G, 2014, ENDOSCOPY, V46, P553, DOI 10.1055/s-0034-1365646

Codipilly DC, 2018, GASTROINTEST ENDOSC, V88, P413, DOI 10.1016/j.gie.2018.04.2352

Guo CC, 2021, CANCER MED-US, V10, P7895, DOI 10.1002/cam4.3703

Hao J, 2009, BMC GASTROENTEROL, V9, DOI 10.1186/1471-230X-9-59

Horie Y, 2019, GASTROINTEST ENDOSC, V89, P25, DOI 10.1016/j.gie.2018.07.037

Huang XM, 2018, CANCER LETT, V413, P94, DOI 10.1016/j.canlet.2017.10.046

Murphy G, 2017, ANN ONCOL, V28, P2086, DOI 10.1093/annonc/mdx279

Niu C, 2019, DIS MARKERS, V2019, DOI 10.1155/2019/9673129

Sasaki Y, 2017, J GEN FAM MED, V18, P171, DOI 10.1002/jgf2.55

Shaheen O, 2017, GASTROENT RES PRACT, V2017, DOI 10.1155/2017/1657310

Then EO, 2020, WORLD J ONCOL, V11, P55, DOI 10.14740/wjon1254

Visaggi P, 2021, CANCERS, V13, DOI 10.3390/cancers13133162

Wang Q, 2019, ONCOL LETT, V18, P1199, DOI 10.3892/ol.2019.10440

Wu SG, 2017, CANCER MANAG RES, V9, P781, DOI 10.2147/CMAR.S150350

Zhang XQ, 2020, CANCER CELL INT, V20, DOI 10.1186/s12935-020-01451-0

NR 17

TC 0

Z9 0

U1 1

U2 1

PU DOVE MEDICAL PRESS LTD

PI ALBANY

PA PO BOX 300-008, ALBANY, AUCKLAND 0752, NEW ZEALAND

EI 1178-7074

J9 INT J GEN MED

JI Int. J. Gen. Med.

PY 2021

VL 14

BP 10423

EP 10428

DI 10.2147/IJGM.S343889

PG 6

WC Medicine, General & Internal

WE Science Citation Index Expanded (SCI-EXPANDED)

SC General & Internal Medicine

GA YB5KZ

UT WOS:000739052400004

PM 35002298

OA gold, Green Published

DA 2022-05-19

ER

PT J

AU Chen, CY

Li, CC

Chien, CR

AF Chen, Chih-Yi

Li, Chia-Chin

Chien, Chun-Ru

TI Does higher radiation dose lead to better outcome for non-operated

localized esophageal squamous cell carcinoma patients who received

concurrent chemoradiotherapy? A population based propensity-score

matched analysis

SO RADIOTHERAPY AND ONCOLOGY

LA English

DT Article

DE Esophageal squamous cell carcinoma; Concurrent chemoradiotherapy;

Radiotherapy dose

ID LUNG-CANCER; STAGE; RADIOTHERAPY; CHEMOTHERAPY; THERAPY; SURGERY; COST;

CHEMORADIATION; SURVIVAL; TRIAL

AB Background: The optimal radiotherapy dose for non-operated localized esophageal squamous cell carcinoma (NOL-ESCC) patients undergoing concurrent chemoradiotherapy (CCRT) is hotly debated.

Methods: We identified eligible patients diagnosed within 2008-2013 from Taiwan Cancer Registry and constructed a propensity score matched cohort (1:1 for high dose (>= 60 Gy) vs standard dose (50-50.4 Gy)) to balance observable potential confounders. We compared the hazard ratio (HR) of death between standard and high radiotherapy dose groups during the entire follow-up period. We performed sensitivity analysis (SA) to evaluate the robustness of our finding regarding potential unobserved confounders & index date definition.

Results: Our study population constituted 648 patients with well balance in observed co-variables. The HR of death when high dose was compared to standard dose was 0.75 (95% confidence interval 0.64-0.88). Our result was sensitive to potential unobserved confounders but robust to alternative index date definition in SA.

Conclusions: We found that higher than standard radiotherapy dose may lead to better survival for NOL-ESCC patients undergoing CCRT. (C) 2016 Elsevier Ireland Ltd. All rights reserved.

C1 [Chen, Chih-Yi] Chung Shan Med Univ, Chung Shan Med Univ Hosp, Dept Surg, Div Thorac Surg, Taichung, Taiwan.

[Li, Chia-Chin] China Med Univ Hosp, Ctr Canc, Taichung, Taiwan.

[Chien, Chun-Ru] China Med Univ Hosp, Ctr Canc, Dept Radiat Oncol, Taichung, Taiwan.

[Chien, Chun-Ru] China Med Univ, Coll Med, Sch Med, 91 Hsueh Shih Rd, Taichung 40402, Taiwan.

RP Chien, CR (corresponding author), China Med Univ, Coll Med, Sch Med, 91 Hsueh Shih Rd, Taichung 40402, Taiwan.

EM d16181@gmail.com

FU Health Promotion Administration, Ministry of Health and Welfare, Taiwan

(R.O.C.)

FX This work is supported by Health Promotion Administration, Ministry of

Health and Welfare, Taiwan (R.O.C.). Sources of funding are from the

tobacco control and health care funds. We thank the comments from the

editor and the two anonymous reviewers.

CR Austin PC, 2013, STAT MED

Bedenne L, 2007, J CLIN ONCOL, V25, P1160, DOI 10.1200/JCO.2005.04.7118

Berger B, 2009, RADIOTHER ONCOL, V92, P276, DOI 10.1016/j.radonc.2009.02.019

Chiang CJ, 2015, JPN J CLIN ONCOL, V45, P291, DOI 10.1093/jjco/hyu211

Chien CR, 2014, THORAC CANCER, V5, P530, DOI 10.1111/1759-7714.12125

Chien CR, 2009, JNCI-J NATL CANCER I, V101, P1428, DOI 10.1093/jnci/djp304

Chiu PWY, 2005, J GASTROINTEST SURG, V9, P794, DOI 10.1016/j.gassur.2005.05.005

Fang HY, 2014, J THORAC DIS, V6, P1690, DOI 10.3978/j.issn.2072-1439.2014.10.27

Garrido MM, 2014, HEALTH SERV RES, V49, P1701, DOI 10.1111/1475-6773.12182

Hsia TC, 2015, J THORAC DIS, V7, P1643, DOI 10.3978/j.issn.2072-1439.2015.09.36

Lang K, 2014, BMC HEALTH SERV RES, V14, DOI 10.1186/1472-6963-14-298

Lin CY, 2015, THORAC CANC

Minsky BD, 2002, J CLIN ONCOL, V20, P1167, DOI 10.1200/JCO.20.5.1167

National Comprehensive Cancer Network (NCCN), 2015, GUID ES ES JUNCT CAN

Pennathur A, 2013, LANCET, V381, P400, DOI 10.1016/S0140-6736(12)60643-6

Roder D, 2010, ANZ J SURG, V80, P776, DOI 10.1111/j.1445-2197.2010.05341.x

Semrau R, 2012, DIS ESOPHAGUS, V25, P545, DOI 10.1111/j.1442-2050.2011.01286.x

Stahl M, 2005, J CLIN ONCOL, V23, P2310, DOI 10.1200/JCO.2005.00.034

Stahl M, 2013, ANN ONCOL, V24, P51, DOI 10.1093/annonc/mdt342

Suh YG, 2014, JPN J CLIN ONCOL, V44, P534, DOI 10.1093/jjco/hyu047

Tai P, 2014, WORLD J GASTRO ONCOL, V6, P263, DOI 10.4251/wjgo.v6.i8.263

Wolf MC, 2012, RADIAT ONCOL, V7, DOI 10.1186/1748-717X-7-60

Yamoah K, 2015, INT J RADIAT ONCOL, V93, P737, DOI 10.1016/j.ijrobp.2015.07.2284

Zhang Z, 2005, INT J RADIAT ONCOL, V61, P656, DOI 10.1016/j.ijrobp.2004.06.022

NR 24

TC 38

Z9 45

U1 0

U2 4

PU ELSEVIER IRELAND LTD

PI CLARE

PA ELSEVIER HOUSE, BROOKVALE PLAZA, EAST PARK SHANNON, CO, CLARE, 00000,

IRELAND

SN 0167-8140

J9 RADIOTHER ONCOL

JI Radiother. Oncol.

PD JUL

PY 2016

VL 120

IS 1

BP 136

EP 139

DI 10.1016/j.radonc.2016.04.042

PG 4

WC Oncology; Radiology, Nuclear Medicine & Medical Imaging

WE Science Citation Index Expanded (SCI-EXPANDED)

SC Oncology; Radiology, Nuclear Medicine & Medical Imaging

GA DU5SN

UT WOS:000382271700021

PM 27207358

DA 2022-05-19

ER

PT J

AU Liu, JY

Li, F

Ping, Y

Wang, LP

Chen, XF

Wang, D

Cao, L

Zhao, S

Li, B

Kalinski, P

Thorne, SH

Zhang, B

Zhang, Y

AF Liu, Jinyan

Li, Feng

Ping, Yu

Wang, Liping

Chen, Xinfeng

Wang, Dan

Cao, Ling

Zhao, Song

Li, Bing

Kalinski, Pawel

Thorne, Stephen H.

Zhang, Bin

Zhang, Yi

TI Local production of the chemokines CCL5 and CXCL10 attracts CD8(+) T

lymphocytes into esophageal squamous cell carcinoma

SO ONCOTARGET

LA English

DT Article

DE esophageal squamous cell carcinoma; T lymphocyte; chemotaxis; CCL5;

CXCL10

ID TUMOR-INFILTRATING LYMPHOCYTES; EXPRESSION; CANCER; RECEPTORS;

RECRUITMENT; TISSUE; PROGNOSIS; MIGRATION; SURVIVAL; DELIVERY

AB Esophageal squamous cell carcinoma (ESCC) is a very common malignant tumor with poor prognosis in China. Chemokines secreted by tumors are pivotal for the accumulation of CD8(+) T lymphocytes within malignant lesions in several types of cancers, but the exact mechanism underlying CD8(+) T lymphocyte homing is still unknown in ESCC. In this study, we revealed that, compared with marginal tissues, the expression of both chemokine (C-C motif) ligand 5 (CCL5) and (C-X-C motif) ligand 10 (CXCL10) was upregulated in ESCC tissues. CCL5 expression was positively associated with the overall survival of patients. Meanwhile, RT-PCR data showed that the expression of CCL5 and CXCL10 was positively correlated with the local expressions of the CD8(+) T lymphocyte markers (CD8 and Granzyme B) in tumor tissues. Correspondingly, CD8(+) T lymphocytes were more frequently CCR5- and CXCR3-positive in tumor than in peripheral blood. Transwell analysis showed both CCL5 and CXCL10 were important for the chemotactic movement of CD8(+) T lymphocytes. Our data indicate that CCL5 and CXCL10 serve as the key chemokines to recruit CD8(+) T lymphocytes into ESCC tissue and may play a role in patient survival.

C1 [Liu, Jinyan; Li, Feng; Ping, Yu; Chen, Xinfeng; Wang, Dan; Cao, Ling; Zhang, Yi] Zhengzhou Univ, Affiliated Hosp 1, Biotherapy Ctr, Zhengzhou 450052, Henan, Peoples R China.

[Wang, Liping; Zhang, Yi] Zhengzhou Univ, Affiliated Hosp 1, Dept Oncol, Zhengzhou 450052, Henan, Peoples R China.

[Zhao, Song; Li, Bing] Zhengzhou Univ, Affiliated Hosp 1, Dept Thorac Surg, Zhengzhou 450052, Henan, Peoples R China.

[Zhang, Yi] Engn Key Lab Cell Therapy Henan Prov, Zhengzhou, Henan, Peoples R China.

[Kalinski, Pawel; Thorne, Stephen H.] Univ Pittsburgh, Dept Surg, Inst Canc, Pittsburgh, PA USA.

[Zhang, Bin] Northwestern Univ, Dept Med, Robert H Lurie Comprehens Canc Ctr, Div Hematol Oncol,Feinberg Sch Med, Chicago, IL 60611 USA.

RP Zhang, Y (corresponding author), Zhengzhou Univ, Affiliated Hosp 1, Biotherapy Ctr, Zhengzhou 450052, Henan, Peoples R China.

EM yizhang@zzu.edu.cn

OI Zhang, Yi/0000-0001-9861-4681

FU China-US (NFSC-NIH) Program for Biomedical Collaborative Research

[812111102]; National Natural Science Foundation of China [81171986];

Ministry of Public Health [20110110001]; Basic and Advanced Technology

Research Foundation from Science and Technology Department of Henan

Province [112300410153, 122300410155]; NATIONAL CANCER INSTITUTE

[P30CA060553] Funding Source: NIH RePORTER

FX This study was supported by grants from the China-US (NFSC-NIH) Program

for Biomedical Collaborative Research (Grant no.812111102), the National

Natural Science Foundation of China (Grant no.81171986), the Ministry of

Public Health (Grant no.20110110001), and the Basic and Advanced

Technology Research Foundation from Science and Technology Department of

Henan Province (Grant nos. 112300410153 and 122300410155).

CR Asai H, 2013, PLOS ONE, V8, DOI 10.1371/journal.pone.0056820

Balkwill F, 2004, NAT REV CANCER, V4, P540, DOI 10.1038/nrc1388

Berghuis D, 2011, J PATHOL, V223, P347, DOI 10.1002/path.2819

Charo IF, 2006, NEW ENGL J MED, V354, P610, DOI 10.1056/NEJMra052723

Chen DY, 2012, HUM IMMUNOL, V73, P1068, DOI 10.1016/j.humimm.2012.07.333

Chen KJ, 2011, PLOS ONE, V6, DOI 10.1371/journal.pone.0024671

Chew V, 2012, GUT, V61, P427, DOI 10.1136/gutjnl-2011-300509

Djenidi F, 2015, J IMMUNOL, V194, P3475, DOI 10.4049/jimmunol.1402711

Franciszkiewicz K, 2012, CANCER RES, V72, P6325, DOI 10.1158/0008-5472.CAN-12-2027

Gu-Trantien C, 2013, J CLIN INVEST, V123, P2873, DOI 10.1172/JCI67428

Hang D, 2012, DIS ESOPHAGUS, V25, P638, DOI 10.1111/j.1442-2050.2011.01298.x

Hannelien V, 2012, BBA-REV CANCER, V1825, P117, DOI 10.1016/j.bbcan.2011.10.008

Harlin H, 2009, CANCER RES, V69, P3077, DOI 10.1158/0008-5472.CAN-08-2281

Hombach AA, 2013, MOL THER, V21, P2268, DOI 10.1038/mt.2013.192

Hu CY, 2013, INT J ONCOL, V43, P1487, DOI 10.3892/ijo.2013.2078

Kim HJ, 2011, PLOS ONE, V6, DOI 10.1371/journal.pone.0026264

Lanca T, 2013, J IMMUNOL, V190, P6673, DOI 10.4049/jimmunol.1300434

Liu JL, 2011, PLOS ONE, V6, DOI 10.1371/journal.pone.0019495

Mariette C, 2007, LANCET ONCOL, V8, P545, DOI 10.1016/S1470-2045(07)70172-9

Masopust D, 2013, NAT REV IMMUNOL, V13, P309, DOI 10.1038/nri3442

Miyake M, 2013, BMC CANCER, V13, DOI 10.1186/1471-2407-13-322

Mlecnik B, 2010, GASTROENTEROLOGY, V138, P1429, DOI 10.1053/j.gastro.2009.10.057

Mulligan AM, 2013, CLIN CANCER RES, V19, P336, DOI 10.1158/1078-0432.CCR-11-3314

Musha H, 2005, INT J CANCER, V116, P949, DOI 10.1002/ijc.21135

Muthuswamy R, 2012, CANCER RES, V72, P3735, DOI 10.1158/0008-5472.CAN-11-4136

Oldham KA, 2012, EUR UROL, V61, P385, DOI 10.1016/j.eururo.2011.10.035

Panse J, 2008, BRIT J CANCER, V99, P930, DOI 10.1038/sj.bjc.6604621

Parsonage G, 2012, AM J PATHOL, V180, P1215, DOI 10.1016/j.ajpath.2011.11.032

Pivarcsi A, 2007, P NATL ACAD SCI USA, V104, P19055, DOI 10.1073/pnas.0705673104

Rooney MS, 2015, CELL, V160, P48, DOI 10.1016/j.cell.2014.12.033

Salerno EP, 2014, INT J CANCER, V134, P563, DOI 10.1002/ijc.28391

Sato E, 2005, P NATL ACAD SCI USA, V102, P18538, DOI 10.1073/pnas.0509182102

Sherwood AM, 2013, CANCER IMMUNOL IMMUN, V62, P1453, DOI 10.1007/s00262-013-1446-2

Sugiyama D, 2013, P NATL ACAD SCI USA, V110, P17945, DOI 10.1073/pnas.1316796110

Tachezy M, 2013, J TRANSL MED, V11, DOI 10.1186/1479-5876-11-238

Thanarajasingam U, 2007, CANCER RES, V67, P300, DOI 10.1158/0008-5472.CAN-06-1017

Urbanska K, 2012, CANCER RES, V72, P1844, DOI 10.1158/0008-5472.CAN-11-3890

Wehler TC, 2011, ANTICANCER RES, V31, P2797

Winkler AE, 2011, CANCER RES, V71, P5707, DOI 10.1158/0008-5472.CAN-11-0907

Zeng J, 2013, J TRANSL MED, V11, DOI 10.1186/1479-5876-11-6

Zhang TQ, 2006, CANCER BIOL THER, V5, P1304, DOI 10.4161/cbt.5.10.3153

Zhao JS, 2011, PLOS ONE, V6, DOI 10.1371/journal.pone.0021419

Zlotnik A, 2012, IMMUNITY, V36, P705, DOI 10.1016/j.immuni.2012.05.008

Zou Y, 2014, BRIT J CANCER, V110, P1992, DOI 10.1038/bjc.2014.140

Zumwalt TJ, 2015, ONCOTARGET, V6, P2981, DOI 10.18632/oncotarget.3205

NR 45

TC 59

Z9 61

U1 3

U2 10

PU IMPACT JOURNALS LLC

PI ORCHARD PARK

PA 6666 E QUAKER ST, STE 1, ORCHARD PARK, NY 14127 USA

EI 1949-2553

J9 ONCOTARGET

JI Oncotarget

PD SEP 22

PY 2015

VL 6

IS 28

BP 24978

EP 24989

DI 10.18632/oncotarget.4617

PG 12

WC Oncology; Cell Biology

WE Science Citation Index Expanded (SCI-EXPANDED)

SC Oncology; Cell Biology

GA CT9TS

UT WOS:000363160100037

PM 26317795

OA Green Published, gold, Green Submitted

DA 2022-05-19

ER

PT J

AU Yang, M

Yang, CS

Pei, YH

AF Yang, Mei

Yang, Chunsong

Pei, Yuhua

TI Effects of downregulation of SIRT3 expression on proliferation and

apoptosis in esophageal squamous cell carcinoma EC9706 cells and its

molecular mechanisms

SO BIO-MEDICAL MATERIALS AND ENGINEERING

LA English

DT Article

DE Esophageal squamous cell carcinoma; SIRT3; small interfering RNA;

proliferation; apoptosis

ID SIRTUIN-3 SIRT3

AB To investigate the effect of downregulation of SIRT3 expression on cell proliferation and invasion in esophageal squamous cell carcinoma (ESCC) EC9706 cells, and to explore its possible molecular mechanisms, we transfected siRNA targeting SIRT3 into EC9706 cells, and then divided cells into three groups: untreated, control siRNA and SIRT3 siRNA groups. The expression levels of SIRT3 protein were detected in different groups by western blotting. The effect of SIRT3 siRNA on cell proliferation was investigated using the CCK-8 kit. The changes of cell apoptosis were examined by flow cytometry. Finally, the expression levels of cell proliferation and apoptosis related proteins such as p21, Bcl-2 and Bax were determined by western blotting. SIRT3 siRNA effectively down-regulated the expression of SIRT3 protein in EC9706 cells, and the reduced expression of SIRT3 significantly inhibited cell proliferation and induced cell apoptosis. Most notably, the SIRT3 depletion markedly increased the expressions of p21 and Bax proteins but reduced Bcl-2 protein expression. The proliferation inhibition and apoptosis of EC9706 cells mediated by SIRT3 downregulation may be closely associated with the expression levels of p21, Bcl-2 and Bax proteins.

C1 [Yang, Mei; Yang, Chunsong; Pei, Yuhua] Wuhan City Vocat Coll, Wuhan 430064, Peoples R China.

RP Pei, YH (corresponding author), Wuhan City Vocat Coll, 10 West Yezhi Lake Rd, Wuhan 430064, Peoples R China.

EM pei0623@163.com

CR Ahn BH, 2008, P NATL ACAD SCI USA, V105, P14447, DOI 10.1073/pnas.0803790105

Alhazzazi Turki Y, 2013, Genes Cancer, V4, P164, DOI 10.1177/1947601913486351

Alhazzazi TY, 2011, CANCER-AM CANCER SOC, V117, P1670, DOI 10.1002/cncr.25676

Ashraf N, 2006, BRIT J CANCER, V95, P1056, DOI 10.1038/sj.bjc.6603384

Buler M, 2012, PLOS ONE, V7, DOI 10.1371/journal.pone.0049863

Chen Y, 2014, CELL DEATH DIS, V5, DOI 10.1038/cddis.2014.14

Guarente L, 2013, METHODS MOL BIOL, V1077, P3, DOI 10.1007/978-1-62703-637-5_1

Hallows William C, 2008, Biochem J, V411, pe11, DOI 10.1042/BJ20080336

Hallows WC, 2006, P NATL ACAD SCI USA, V103, P10230, DOI 10.1073/pnas.0604392103

Hanahan D, 2011, CELL, V144, P646, DOI 10.1016/j.cell.2011.02.013

Huang KH, 2014, WORLD J SURG, V38, P910, DOI 10.1007/s00268-013-2359-0

Kamarajan P, 2012, CANCER-AM CANCER SOC, V118, P5800, DOI 10.1002/cncr.27655

Li HQ, 2013, INT J ONCOL, V43, P1420, DOI 10.3892/ijo.2013.2103

Marfe G, 2009, J CELL BIOCHEM, V106, P643, DOI 10.1002/jcb.22044

Sundaresan NR, 2009, J CLIN INVEST, V119, P2758, DOI 10.1172/JCI39162

Weir Heather J M, 2013, Genes Cancer, V4, P118, DOI 10.1177/1947601913476949

Xiao K, 2013, ONCOL REP, V30, P1323, DOI 10.3892/or.2013.2604

Zhang YY, 2012, BIOCHEM BIOPH RES CO, V423, P26, DOI 10.1016/j.bbrc.2012.05.053

Zhao Y, 2013, ANN DIAGN PATHOL, V17, P483, DOI 10.1016/j.anndiagpath.2013.06.001

NR 19

TC 12

Z9 13

U1 0

U2 3

PU IOS PRESS

PI AMSTERDAM

PA NIEUWE HEMWEG 6B, 1013 BG AMSTERDAM, NETHERLANDS

SN 0959-2989

EI 1878-3619

J9 BIO-MED MATER ENG

JI Bio-Med. Mater. Eng.

PY 2014

VL 24

IS 6

BP 3883

EP 3890

DI 10.3233/BME-141219

PG 8

WC Engineering, Biomedical; Materials Science, Biomaterials

WE Science Citation Index Expanded (SCI-EXPANDED)

SC Engineering; Materials Science

GA AQ7NW

UT WOS:000343005700222

PM 25227106

OA Bronze

DA 2022-05-19

ER

PT J

AU Chang, L

Guo, FJ

Lv, YL

Wang, YD

Huo, BJ

Wang, L

Liu, W

AF Chang, Liang

Guo, Fengjie

Lv, Yalei

Wang, Yudong

Huo, Bingjie

Wang, Long

Liu, Wei

TI The inhibitory effects of Endostar combined with chemotherapy on human

esophageal squamous cell carcinoma xenograft in mice

SO MOLECULAR BIOLOGY REPORTS

LA English

DT Article

DE Endostar; Chemotherapy; Esophageal carcinoma; Ki-67; Xenograft

ID RECOMBINANT HUMAN ENDOSTATIN; ADENOCARCINOMA; THERAPY; LUNG;

RADIOTHERAPY; COMBINATION; RADIORESPONSE; EFFICACY; CANCER; MODEL

AB The purpose of this study was to investigate the efficacy of Endostar combined with chemotherapy on human esophageal squamous cell carcinoma Eca-109 in mice. The tumor xenograft models were established and randomly assigned to 4 groups: control group, Endostar group (1.5 mg/kg day, once daily for 3 weeks), chemotherapy group (Paclitaxel 10 mg/kg day, Cisplatin 5 mg/kg day, for 1, 7, 14, 21 days), and chemotherapy + Endostar group (combination). The length and width of tumor were measured and the tumor volumes (cm(3)) were calculated every other day. Three weeks later, the mice were executed, and the tumor tissues were collected to weigh and analyse the histopathology and proliferation for tumor xenograft. The results demonstrated that the tumor volumes and weight in chemotherapy + Endostar group were significantly lower than that in other three groups. Meanwhile, the cell proliferation of tumor xenograft in combined treatment group was significantly lower than that in other three groups. It was concluded that Endostar combined with chemotherapy could obviously enhance the inhibitory effect on esophageal squamous cell carcinoma Eca-109 in mice.

C1 [Chang, Liang; Lv, Yalei; Wang, Yudong; Huo, Bingjie; Wang, Long; Liu, Wei] Hebei Med Univ, Dept Med Oncol, Hosp 4, Shijiazhuang, Hebei, Peoples R China.

[Guo, Fengjie] Tianjin Med Univ, Gen Hosp, Tianjin Lung Canc Inst, Tianjin Key Lab Lung Canc Metastasis & Tumor Micr, Tianjin, Peoples R China.

RP Liu, W (corresponding author), Hebei Med Univ, Dept Med Oncol, Hosp 4, 12 Jiankang Rd, Shijiazhuang, Hebei, Peoples R China.

EM hebeiliuwei@hotmail.com

RI Wang, Yudong/AAE-7557-2020; HUO, bingjie/AGJ-1037-2022

FU International Medical Foundation of China [CIMF-F-H001-049]

FX This work was supported by the International Medical Foundation of China

(CIMF-F-H001-049).

CR Abrams JA, 2011, CANCER EPIDEM BIOMAR, V20, P183, DOI 10.1158/1055-9965.EPI-10-0802

Deng G, 2011, CARDIOVASC INTER RAD, V34, P824, DOI 10.1007/s00270-011-0179-x

Fu XH, 2011, CANCER LETT, V312, P109, DOI 10.1016/j.canlet.2011.08.008

Ge W, 2011, ASIAN PAC J CANCER P, V12, P2705

Hu SY, 2010, INT J NANOMED, V5, P1039, DOI 10.2147/IJN.S14753

Jia YT, 2012, PATHOL ONCOL RES, V18, P315, DOI 10.1007/s12253-011-9447-y

Kisker O, 2001, CANCER RES, V61, P7669

Li Y, 2010, ASIAN PAC J CANCER P, V11, P1119

Ling Y, 2007, BIOCHEM BIOPH RES CO, V361, P79, DOI 10.1016/j.bbrc.2007.06.155

Peng F, 2012, PLOS ONE, V7, DOI 10.1371/journal.pone.0034646

Peng XC, 2012, CANCER CHEMOTH PHARM, V69, P239, DOI 10.1007/s00280-011-1695-8

Sheyhidin I, 2011, WORLD J GASTROENTERO, V17, P3745, DOI 10.3748/wjg.v17.i32.3745

Sun Y, 2005, J CLIN ONCOL, V23, p654S

Wen QL, 2009, CANCER SCI, V100, P1510, DOI 10.1111/j.1349-7006.2009.01193.x

Wu DS, 2008, RADIAT ENVIRON BIOPH, V47, P285, DOI 10.1007/s00411-007-0144-x

Wu HT, 2010, J HUAZHONG U SCI-MED, V30, P108, DOI 10.1007/s11596-010-0120-6

Yang HQ, 2012, WORLD J GASTROENTERO, V18, P393, DOI 10.3748/wjg.v18.i5.393

Yoon SS, 1999, CANCER RES, V59, P6251

You ZY, 2010, CANCER CELL INT, V10, DOI 10.1186/1475-2867-10-17

Zhan Q, 2011, PHOTODIAGN PHOTODYN, V8, P314, DOI 10.1016/j.pdpdt.2011.04.002

Zhang L, 2012, MOL BIOL REP, V39, P89, DOI 10.1007/s11033-011-0713-6

Zhou JF, 2011, CHINESE MED J-PEKING, V124, P4299, DOI 10.3760/cma.j.issn.0366-6999.2011.24.031

Zhou N, 2011, J HUAZHONG U SCI-MED, V31, P62, DOI 10.1007/s11596-011-0151-7

NR 23

TC 8

Z9 9

U1 0

U2 8

PU SPRINGER

PI DORDRECHT

PA VAN GODEWIJCKSTRAAT 30, 3311 GZ DORDRECHT, NETHERLANDS

SN 0301-4851

EI 1573-4978

J9 MOL BIOL REP

JI Mol. Biol. Rep.

PD JAN

PY 2013

VL 40

IS 1

BP 669

EP 673

DI 10.1007/s11033-012-2106-x

PG 5

WC Biochemistry & Molecular Biology

WE Science Citation Index Expanded (SCI-EXPANDED)

SC Biochemistry & Molecular Biology

GA 055RV

UT WOS:000312435500079

PM 23054020

DA 2022-05-19

ER

PT J

AU Xu, CS

Zheng, JY

Zhang, HL

Zhao, HD

Zhang, J

Wu, GQ

Wu, L

Wang, Q

Wang, WZ

Zhang, J

AF Xu, Chun-Sheng

Zheng, Jian-Yong

Zhang, Hai-Long

Zhao, Hua-Dong

Zhang, Jing

Wu, Guo-Qiang

Wu, Lin

Wang, Qing

Wang, Wei-Zhong

Zhang, Jian

TI MSP58 Knockdown Inhibits the Proliferation of Esophageal Squamous Cell

Carcinoma in Vitro and in Vivo

SO ASIAN PACIFIC JOURNAL OF CANCER PREVENTION

LA English

DT Article

DE MSP58; esophageal squamous cell carcinoma; small hairpin RNA (shRNA)

ID 58-KDA MICROSPHERULE PROTEIN; NEOADJUVANT CHEMOTHERAPY; CANCER;

P27(KIP1); INTERACTS; GROWTH

AB Esophageal carcinoma (EC) is one of the most aggressive cancers with a poor prognosis. Understanding the molecular mechanisms underlying esophageal cancer progression is a high priority for improved EC diagnosis and prognosis. Recently, MSP58 was shown to behave as an oncogene in colorectal carcinomas and gliomas. However, little is known about its function in esophageal carcinomas. We therefore examined the effects of MSP58 knockdown on the growth of esophageal squamous cell carcinoma (ESCC) cells in vitro and in vivo in order to gain a better understanding of its potential as a tumor therapeutic target. We employed lentiviral-mediated small hairpin RNA (shRNA) to knock down the expression of MSP58 in the ESCC cell lines Eca-109 and EC9706 and demonstrated inhibition of ESCC cell proliferation and colony formation in vitro. Furthermore, flow cytometry and western blot analyses revealed that MSP58 depletion induced cell cycle arrest by regulating the expression of P21, CDK4 and cyclin D1. Notably, the downregulation of MSP58 significantly inhibited the growth of ESCC xenografts in nude mice. Our results suggest that MSP58 may play an important role in ESCC progression.

C1 [Xu, Chun-Sheng; Zheng, Jian-Yong; Wang, Wei-Zhong] Fourth Mil Med Univ, Xijing Hosp, Dept Gastrointestinal Surg, State Key Lab Canc Biol, Xian 710032, Peoples R China.

[Zhang, Hai-Long] Fourth Mil Med Univ, Xijing Hosp, Dept Dermatol, Xian 710032, Peoples R China.

[Zhang, Jing; Wu, Lin; Zhang, Jian] Fourth Mil Med Univ, Dept Biochem & Mol Biol, State Key Lab Canc Biol, Xian 710032, Peoples R China.

[Zhao, Hua-Dong; Wang, Qing] Fourth Mil Med Univ, Tangdu Hosp, Dept Gen Surg, Xian 710032, Peoples R China.

RP Wang, WZ (corresponding author), Fourth Mil Med Univ, Xijing Hosp, Dept Gastrointestinal Surg, State Key Lab Canc Biol, Xian 710032, Peoples R China.

EM weichang@fmmu.edu.cn; biozhangj@yahoo.com.cn

FU National Natural Science Foundation of China [30830054, 81172292,

81001123, 81001161, 81072050]; National Program on Key Basic Research

Project [2009CB521704]

FX This work was supported by grants from the National Natural Science

Foundation of China (No. 30830054, 81172292, 81001123, 81001161, and

81072050) and National Program on Key Basic Research Project

(2009CB521704).

CR Allum WH, 2009, J CLIN ONCOL, V27, P5062, DOI 10.1200/JCO.2009.22.2083

Anayama T, 2001, INT J ONCOL, V18, P151

Bader AG, 2001, ONCOGENE, V20, P7524, DOI 10.1038/sj.onc.1204938

Bos TJ, 2009, CURR GENE THER, V9, P192, DOI 10.2174/156652309788488578

Boudreau RL, 2012, METHOD ENZYMOL, V507, P275, DOI 10.1016/B978-0-12-386509-0.00014-4

Chumakov SP, 2010, PLASMID, V63, P143, DOI 10.1016/j.plasmid.2009.12.003

Davidovic L, 2006, HUM MOL GENET, V15, P1525, DOI 10.1093/hmg/ddl074

Ekholm SV, 2000, CURR OPIN CELL BIOL, V12, P676, DOI 10.1016/S0955-0674(00)00151-4

Enzinger PC, 2003, NEW ENGL J MED, V349, P2241, DOI 10.1056/NEJMra035010

Ferrandiz N, 2012, PLOS ONE, V7, DOI 10.1371/journal.pone.0037759

Hanahan D, 2011, CELL, V144, P646, DOI 10.1016/j.cell.2011.02.013

HARTWELL LH, 1994, SCIENCE, V266, P1821, DOI 10.1126/science.7997877

Howell AS, 2012, GENETICS, V190, P51, DOI 10.1534/genetics.111.128314

Ivanova AV, 2005, CELL MOL LIFE SCI, V62, P471, DOI 10.1007/s00018-004-4423-2

Jemal A, 2011, CA-CANCER J CLIN, V61, P69, DOI [10.3322/caac.20107, 10.3322/caac.20115]

Lin DY, 2002, J BIOL CHEM, V277, P25446, DOI 10.1074/jbc.M200633200

Lin W, 2009, J CELL MOL MED, V13, P4608, DOI 10.1111/j.1582-4934.2008.00499.x

Matrai J, 2010, MOL THER, V18, P477, DOI 10.1038/mt.2009.319

MATSUOKA S, 1995, GENE DEV, V9, P650, DOI 10.1101/gad.9.6.650

Okumura K, 2005, P NATL ACAD SCI USA, V102, P2703, DOI 10.1073/pnas.0409370102

Pai SI, 2006, GENE THER, V13, P464, DOI 10.1038/sj.gt.3302694

Pickens A, 2003, ANN THORAC SURG, V76, pS1367, DOI 10.1016/S0003-4975(03)01202-5

POLYAK K, 1994, CELL, V78, P59, DOI 10.1016/0092-8674(94)90572-X

Ren Y, 1998, EUR J BIOCHEM, V253, P734, DOI 10.1046/j.1432-1327.1998.2530734.x

Shi H, 2009, CANCER SCI, V100, P1585, DOI 10.1111/j.1349-7006.2009.01223.x

Siegel R, 2012, CA-CANCER J CLIN, V62, P10, DOI 10.3322/caac.20138

Sjoquist KM, 2011, LANCET ONCOL, V12, P681, DOI 10.1016/S1470-2045(11)70142-5

Stoner GD, 2001, CARCINOGENESIS, V13, p[3237, 1737]

Sumimoto Hidetoshi, 2007, Future Oncol, V3, P655, DOI 10.2217/14796694.3.6.655

Swanton C, 2004, LANCET ONCOL, V5, P27, DOI 10.1016/S1470-2045(03)01321-4

XIONG Y, 1993, NATURE, V366, P701, DOI 10.1038/366701a0

Xu XH, 2012, ASIAN PAC J CANCER P, V13, P103, DOI 10.7314/APJCP.2012.13.1.103

Yu X, 2010, ASIAN PAC J CANCER P, V11, P1827

Zhang J, 2007, BIOCHEM BIOPH RES CO, V352, P6, DOI 10.1016/j.bbrc.2006.10.141

NR 34

TC 8

Z9 9

U1 4

U2 12

PU ASIAN PACIFIC ORGANIZATION CANCER PREVENTION

PI GYEONGGI-DO

PA APJCP HEAD OFFICE, KOREAN NATL CANCER CENTER, 323 ILAN -RO,

ILSANDONG-GU, GOYANG-SI, GYEONGGI-DO, 410-769, SOUTH KOREA

SN 1513-7368

J9 ASIAN PAC J CANCER P

JI Asian Pac. J. Cancer Prev.

PY 2012

VL 13

IS 7

BP 3233

EP 3238

DI 10.7314/APJCP.2012.13.7.3233

PG 6

WC Oncology

WE Science Citation Index Expanded (SCI-EXPANDED)

SC Oncology

GA 015WB

UT WOS:000309476000038

PM 22994740

OA gold

DA 2022-05-19

ER

PT J

AU Liu, Z

Yu, SB

Ye, ST

Shen, ZM

Gao, L

Han, ZY

Zhang, PP

Luo, F

Chen, S

Kang, MQ

AF Liu, Zhun

Yu, Shaobin

Ye, Shuting

Shen, Zhimin

Gao, Lei

Han, Ziyang

Zhang, Peipei

Luo, Fei

Chen, Sui

Kang, Mingqiang

TI Keratin 17 activates AKT signalling and induces epithelial-mesenchymal

transition in oesophageal squamous cell carcinoma

SO JOURNAL OF PROTEOMICS

LA English

DT Article

DE Keratin 17; Oesophageal squamous cell carcinoma; The serine/threonine

protein kinase AKT; Epithelial-mesenchymal transition

ID PROMOTES TUMOR-GROWTH; PI3K/AKT PATHWAY; POOR-PROGNOSIS; CANCER;

EXPRESSION; DIFFERENTIATION; GENES; KRT17

AB Oesophageal squamous cell carcinoma (ESCC) is an aggressive malignancy and a leading cause of cancer-related death worldwide. Lack of effective early diagnosis strategies and ensuing complications from tumour metastasis account for the majority of ESCC death. Thus, identification of key molecular targets involved in ESCC carcinogenesis and progression is crucial for ESCC prognosis. In this study, four pairs of ESCC tissues were used for mRNA sequencing to determine differentially expressed genes (DEGs). 347 genes were found to be upregulated whereas 255 genes downregulated. By screening DEGs plus bioinformatics analyses such as KEGG, PPI and IPA, we found that there were independent interactions between KRT family members. KRT17 upregulation was confirmed in ESCC and its relationship with clinicopathological features were analysed. KRT17 was significantly associated with ESCC histological grade, lymph node and distant metastasis, TNM stage and five-year survival rate. Upregulation of KRT17 promoted ESCC cell growth, migration, and lung metastasis. Mechanistically, we found that KRT17-promoted ESCC cell growth and migration was accompanied by activation of AKT signalling and induction of EMT. These findings suggested that KRT17 is significantly related to malignant progression and poor prognosis of ESCC patients, and it may serve as a new biological target for ESCC therapy.

Significance: Oesophageal cancer is one of the leading causes of cancer mortality worldwide and oesophageal squamous cell carcinoma (ESCC) is the major histological type of oesophageal cancer in Eastern Asia. However, the molecular basis for the development and progression of ESCC remains largely unknown. In this study, RNA sequencing was used to establish the whole-transcriptome profile in ESCC tissues versus the adjacent non-cancer tissues and the results were bioinformatically analysed to predict the roles of the identified differentially expressed genes. We found that upregulation of KRT17 was significantly associated with advanced clinical stage, lymph node and distant metastasis, TNM stage and poor clinical outcome. Keratin 17 (KRT17) upregulation in ESCC cells not only promoted cell proliferation but also increased invasion and metastasis accompanied with AKT activation and epithelial-mesenchymal transition (EMT). These data suggested that KRT17 played an important role in ESCC development and progression and may serve as a prognostic biomarker and therapeutic target in ESCC.

C1 [Liu, Zhun; Yu, Shaobin; Shen, Zhimin; Gao, Lei; Han, Ziyang; Zhang, Peipei; Luo, Fei; Chen, Sui; Kang, Mingqiang] Fujian Med Univ, Union Hosp, Dept Thorac Surg, Fuzhou 350001, Fujian, Peoples R China.

[Ye, Shuting; Kang, Mingqiang] Fujian Med Univ, Minist Educ, Key Lab Gastrointestinal Canc, Fuzhou 350122, Fujian, Peoples R China.

[Kang, Mingqiang] Fujian Med Univ, Fujian Key Lab Tumor Microbiol, Fuzhou 350122, Fujian, Peoples R China.

RP Chen, S; Kang, MQ (corresponding author), Fujian Med Univ, Union Hosp, 29 Xinquan Rd, Fuzhou 350001, Fujian, Peoples R China.

EM xhyyxwkcs@163.com; mingqiang_kang@126.com

OI Kang, Mingqiang/0000-0002-0715-6556

FU Fujian Young Teacher Fund [JAT16020]; Sailing Fund of Fujian Medical

University [2016QH036]; Joint Funds for the Innovation of Science and

Technology, Fujian province [2017Y9039, 2017Y9013]; Program for

Innovative Research Team in Science and technology in Fujian Province

University [2017XQ2027]; Startup Fund for Scientific Research, Fujian

Medical University [2017XQ2027]; National Natural Science Foundation of

China [81773129]

FX This study was supported by Fujian Young Teacher Fund (JAT16020),

Sailing Fund of Fujian Medical University (Grant number: 2016QH036),

Joint Funds for the Innovation of Science and Technology, Fujian

province (Grant number: 2017Y9039 and 2017Y9013), Program for Innovative

Research Team in Science and technology in Fujian Province University

and Startup Fund for Scientific Research, Fujian Medical University

(Grant number: 2017XQ2027), the National Natural Science Foundation of

China (Grant number: 81773129).

CR [Anonymous], 2013, LANCET, V381, P347

Bambang IF, 2013, ONCOGENE, V32, P1240, DOI 10.1038/onc.2012.149

Bournet B, 2012, PANCREATOLOGY, V12, P27, DOI 10.1016/j.pan.2011.12.003

Bray F, 2018, CA-CANCER J CLIN, V68, P394, DOI 10.3322/caac.21492

Cheung M, 2013, CURR CANCER DRUG TAR, V13, P234, DOI 10.2174/1568009611313030002

Chiang CH, 2016, J PROTEOME RES, V15, P2981, DOI 10.1021/acs.jproteome.6b00138

Chivu-Economescu M, 2017, GASTRIC CANCER, V20, P948, DOI 10.1007/s10120-017-0712-y

Dong PX, 2014, J TRANSL MED, V12, DOI 10.1186/s12967-014-0231-0

Du Q, 2013, AM J CANCER RES, V3, P402

Duff D, 2017, CELL SIGNAL, V35, P250, DOI 10.1016/j.cellsig.2017.03.005

Grille SJ, 2003, CANCER RES, V63, P2172

Hanahan D, 2011, CELL, V144, P646, DOI 10.1016/j.cell.2011.02.013

Hennessy BT, 2005, NAT REV DRUG DISCOV, V4, P988, DOI 10.1038/nrd1902

Herskovic A, 2012, ANN ONCOL, V23, P1095, DOI 10.1093/annonc/mdr433

Hobbs RP, 2016, ONCOGENE, V35, P5653, DOI 10.1038/onc.2016.102

Hong CC, 2014, CANCER RES, V74, P6280, DOI 10.1158/0008-5472.CAN-14-0524

Hongo M, 2009, J GASTROEN HEPATOL, V24, P729, DOI 10.1111/j.1440-1746.2009.05824.x

Hu H, 2018, J CANCER, V9, P346, DOI 10.7150/jca.19838

Jacob JT, 2018, CSH PERSPECT BIOL, V10, DOI 10.1101/cshperspect.a018275

Khanom R, 2016, PLOS ONE, V11, DOI 10.1371/journal.pone.0161163

Kim S, 2006, NATURE, V441, P362, DOI 10.1038/nature04659

Kurokawa I, 2011, EXP DERMATOL, V20, P217, DOI 10.1111/j.1600-0625.2009.01006.x

Liang Y, 2018, CELL DEATH DIFFER, V25, P1980, DOI 10.1038/s41418-018-0084-9

Luo AP, 2004, ONCOGENE, V23, P1291, DOI 10.1038/sj.onc.1207218

Ma G, 2015, EJSO-EUR J SURG ONC, V41, P541, DOI 10.1016/j.ejso.2015.01.013

Mak MP, 2016, CLIN CANCER RES, V22, P609, DOI 10.1158/1078-0432.CCR-15-0876

Mayer IA, 2016, ANNU REV MED, V67, P11, DOI 10.1146/annurev-med-062913-051343

McGowan KM, 1998, J CELL BIOL, V143, P469, DOI 10.1083/jcb.143.2.469

Quan YJ, 2015, J CANCER, V6, P342, DOI 10.7150/jca.10969

Sankar S, 2013, MOL CELL BIOL, V33, P4448, DOI 10.1128/MCB.00241-13

Syed N, 2015, PROTEOMICS, V15, P374, DOI 10.1002/pmic.201400379

Thiery JP, 2009, CELL, V139, P871, DOI 10.1016/j.cell.2009.11.007

Toivola DM, 2015, CURR OPIN CELL BIOL, V32, P73, DOI 10.1016/j.ceb.2014.12.008

van de Rijn M, 2002, AM J PATHOL, V161, P1991, DOI 10.1016/S0002-9440(10)64476-8

von Mering C, 2005, NUCLEIC ACIDS RES, V33, pD433, DOI 10.1093/nar/gki005

Yang LT, 2019, J PATHOL, V247, P158, DOI 10.1002/path.5178

Zhang JT, 2011, J PROTEOME RES, V10, P2863, DOI 10.1021/pr200141c

Zhang YQ, 2017, CANCER CELL, V31, P820, DOI 10.1016/j.ccell.2017.04.013

NR 38

TC 13

Z9 14

U1 1

U2 13

PU ELSEVIER

PI AMSTERDAM

PA RADARWEG 29, 1043 NX AMSTERDAM, NETHERLANDS

SN 1874-3919

EI 1876-7737

J9 J PROTEOMICS

JI J. Proteomics

PD JAN 16

PY 2020

VL 211

AR 103557

DI 10.1016/j.jprot.2019.103557

PG 12

WC Biochemical Research Methods

WE Science Citation Index Expanded (SCI-EXPANDED)

SC Biochemistry & Molecular Biology

GA JS5VX

UT WOS:000500375300010

PM 31669361

DA 2022-05-19

ER

PT J

AU Shou, YW

Wang, XQ

Liang, YH

Liu, XN

Chen, KS

AF Shou, Yuwei

Wang, Xiaoqian

Liang, Yinghao

Liu, Xiaonan

Chen, Kuisheng

TI Exosomes-derived miR-154-5p attenuates esophageal squamous cell

carcinoma progression and angiogenesis by targeting kinesin family

member 14

SO BIOENGINEERED

LA English

DT Article

DE Exosomes; miR-154-5p; kinesin family member 14 (KIF14); esophageal

squamous cell carcinoma (ESCC); angiogenesis

ID PROLIFERATION; KIF14; INVASION; MIGRATION; PROTEINS

AB Exosomes participate in the progression and angiogenesis of esophageal squamous cell carcinoma (ESCC). This study aimed to explore the effect and mechanism of exosomes-derived miR-154-5p on the progression and angiogenesis of ESCC. The exosomes with the diameter of 40-270 nm were successfully isolated from ESCC cells by ultracentrifugation. They were then assessed by transmission electron microscope (TEM), nanoparticle tracking analysis (NTA), and Western blotting. Kinesin family member 14 (KIF14) was upregulated, while miR-154-5p was downregulated in ESCC as examined by Quantitative Real-time PCR (qRT-PCR). Exosomes-derived miR-154-5p from ESCC cells was found to attenuate the cellular migration, invasion, and angiogenesis of ESCC using Cell Counting Kit-8 (CCK-8), wound healing assay, transwell migration assay, and tumor formation assays. Moreover, KIF14 was proven to be a direct downstream target gene of miR-154-5p in ESCC cells using luciferase assay. In conclusion, our study identified that exosomes-derived miR-154-5p attenuates ESCC progression and angiogenesis by targeting KIF14 in vitro, which might provide a novel approach for the diagnosis and treatment of ESCC.

C1 [Shou, Yuwei; Liu, Xiaonan] Zhengzhou Univ, Acad Med Sci, Zhengzhou, Henan, Peoples R China.

[Shou, Yuwei; Chen, Kuisheng] Zhengzhou Univ, Dept Pathol, Affiliated Hosp 1, 1 Jianshedong Rd, Zhengzhou 450000, Henan, Peoples R China.

[Wang, Xiaoqian; Liang, Yinghao] Zhengzhou Univ, Henan Key Lab Tumor Pathol, Zhengzhou, Henan, Peoples R China.

RP Chen, KS (corresponding author), Zhengzhou Univ, Dept Pathol, Affiliated Hosp 1, 1 Jianshedong Rd, Zhengzhou 450000, Henan, Peoples R China.

EM KuishengChen2@163.com

FU National Natural Science Foundation of China [81872455, U170410141]

FX This work was supported by the National Natural Science Foundation of

China (grant numbers 81872455 and U170410141).

CR Abnet CC, 2018, GASTROENTEROLOGY, V154, P360, DOI 10.1053/j.gastro.2017.08.023

Bartel DP, 2009, CELL, V136, P215, DOI 10.1016/j.cell.2009.01.002

Beckler MD, 2013, MOL CELL PROTEOMICS, V12, P343, DOI 10.1074/mcp.M112.022806

Bray F, 2018, CA-CANCER J CLIN, V68, P394, DOI 10.3322/caac.21492

Carleton M, 2006, MOL CELL BIOL, V26, P3853, DOI 10.1128/MCB.26.10.3853-3863.2006

Chen J, 2020, ONCOTARGETS THER, V13, P2235, DOI 10.2147/OTT.S242939

Chi JR, 2019, MOL THER-NUCL ACIDS, V17, P138, DOI 10.1016/j.omtn.2019.05.013

Corson TW, 2005, ONCOGENE, V24, P4741, DOI 10.1038/sj.onc.1208641

Ding NX, 2018, INT J MOL MED, V42, P381, DOI 10.3892/ijmm.2018.3639

Gao DC, 2020, EUR REV MED PHARMACO, V24, P6097, DOI 10.26355/eurrev_202006_21505

Hirokawa N, 2009, NAT REV MOL CELL BIO, V10, P682, DOI 10.1038/nrm2774

Huang W, 2015, CELL PHYSIOL BIOCHEM, V37, P1659, DOI 10.1159/000438532

Li XY, 2015, MOL MED REP, V12, P3951, DOI 10.3892/mmr.2015.3820

Liu YJ, 2016, ANN NY ACAD SCI, V1381, P66, DOI 10.1111/nyas.13144

Livak KJ, 2001, METHODS, V25, P402, DOI 10.1006/meth.2001.1262

Mohammadi S, 2020, IUBMB LIFE, V72, P724, DOI 10.1002/iub.2182

Nakagawa T, 1997, P NATL ACAD SCI USA, V94, P9654, DOI 10.1073/pnas.94.18.9654

Pavlyukov MS, 2018, CANCER CELL, V34, P119, DOI 10.1016/j.ccell.2018.05.012

Qiu ML, 2020, EUR REV MED PHARMACO, V24, P5412, DOI 10.26355/eurrev_202005_21325

Siegel RL, 2019, CA-CANCER J CLIN, V69, P7, DOI 10.3322/caac.21551

Sun XZ, 2021, THORAC CANCER, V12, P512, DOI 10.1111/1759-7714.13787

Theriault BL, 2014, PLOS ONE, V9, DOI 10.1371/journal.pone.0091540

Tian B, 2020, ONCOTARGETS THER, V13, P7447, DOI 10.2147/OTT.S256291

Wang ZZ, 2018, INT J ONCOL, V53, P1939, DOI 10.3892/ijo.2018.4546

Xu M, 2018, MOL CANCER, V17, DOI 10.1186/s12943-018-0894-x

Yang B, 2020, INT J MED SCI, V17, P2013, DOI 10.7150/ijms.46234

Yang Y, 2018, CELL RES, V28, P862, DOI 10.1038/s41422-018-0060-4

Yin Z, 2020, DRUG DES DEV THER, V14, P4561, DOI 10.2147/DDDT.S269915

Zeng QX, 2020, BIOSCIENCE REP, V40, DOI 10.1042/BSR20201858

Zhang CY, 2020, CANCER MED-US, V9, P8600, DOI 10.1002/cam4.3463

Zhang N, 2020, BIOENGINEERED, V11, P972, DOI 10.1080/21655979.2020.1814658

Zhang Y, 2019, CELL BIOSCI, V9, DOI 10.1186/s13578-019-0282-2

Zhao L, 2012, MED ONCOL, V29, P2669, DOI 10.1007/s12032-011-0133-4

Zhao Q, 2021, BIOENGINEERED, V12, P7656, DOI 10.1080/21655979.2021.1982275

Zhuang HR, 2020, CANCER MANAG RES, V12, P10651, DOI 10.2147/CMAR.S259077

NR 35

TC 0

Z9 0

U1 1

U2 1

PU TAYLOR & FRANCIS INC

PI PHILADELPHIA

PA 530 WALNUT STREET, STE 850, PHILADELPHIA, PA 19106 USA

SN 2165-5979

EI 2165-5987

J9 BIOENGINEERED

JI Bioengineered

PD FEB 1

PY 2022

VL 13

IS 2

BP 4610

EP 4620

DI 10.1080/21655979.2022.2037322

PG 11

WC Biotechnology & Applied Microbiology

WE Science Citation Index Expanded (SCI-EXPANDED)

SC Biotechnology & Applied Microbiology

GA YY5TI

UT WOS:000754851100001

PM 35156510

OA gold, Green Published

DA 2022-05-19

ER

PT J

AU Zhu, ZF

Yu, WW

Fu, XL

Sun, MH

Wei, Q

Li, DL

Chen, HQ

Xiang, JQ

Li, HC

Zhang, YW

Zhao, WX

Zhao, KL

AF Zhu, Zhengfei

Yu, Weiwei

Fu, Xiaolong

Sun, Menghong

Wei, Qiao

Li, Dali

Chen, Haiquan

Xiang, Jiaqing

Li, Hecheng

Zhang, Yawei

Zhao, Weixin

Zhao, Kuaile

TI Phosphorylated AKT1 is associated with poor prognosis in esophageal

squamous cell carcinoma

SO JOURNAL OF EXPERIMENTAL & CLINICAL CANCER RESEARCH

LA English

DT Article

DE Esophageal carcinoma; Esophageal squamous cell carcinoma; Epidermal

growth factor receptor; p-AKT1; Prognostic factor; Prognosis;

Immunohistochemistry

ID GROWTH-FACTOR RECEPTOR; ACTIVATED AKT; PI3K PATHWAY; EXPRESSION;

PREDICTOR; EGFR; SURVIVAL; MUTATION; PROTEIN; CHEMORADIOTHERAPY

AB Background: The epidermal growth factor receptor (EGFR) signaling pathway is important in regulating biological behaviors in many malignancies. We explored whether expression and activation of EGFR and several components on its downstream pathways have prognostic significance in patients with esophageal squamous cell carcinoma (ESCC).

Methods: Expression of EGFR, phosphorylated (p)-EGFR, AKT1, p-AKT1, AKT2, p-AKT2, ERK1, ERK2, p-ERK1/2, STAT3, and p-STAT3 was assessed by immunohistochemical analysis of tissue microarrays for 275 ESCC patients who had undergone complete three-field lymphadenectomy. Spearman rank correlation tests were used to determine the relationships among protein expression, and Cox regression analyses were performed to determine the prognostic factors on overall survival (OS).

Results: p-EGFR expression was correlated statistically with all of the other phosphorylated markers. Gender, N stage, and p-AKT1 expression were found to be independent prognostic factors for OS. Increased expression of p-AKT1 was associated with decreased patient survival. EGFR and p-EGFR expression was not significantly associated with patient survival.

Conclusion: Activation of AKT1 was associated with poor prognosis in ESCC.

C1 [Zhu, Zhengfei; Fu, Xiaolong; Zhao, Weixin; Zhao, Kuaile] Fudan Univ, Dept Radiat Oncol, Shanghai Canc Ctr, Shanghai 200032, Peoples R China.

[Yu, Weiwei] Jiao Tong Univ, Dept Radiat Oncol, Hosp 6, Shanghai 200030, Peoples R China.

[Sun, Menghong; Wei, Qiao; Li, Dali] Fudan Univ, Dept Pathol, Shanghai Canc Ctr, Shanghai 200032, Peoples R China.

[Chen, Haiquan; Xiang, Jiaqing; Li, Hecheng; Zhang, Yawei] Fudan Univ, Dept Thorac Surg, Shanghai Canc Ctr, Shanghai 200032, Peoples R China.

[Zhu, Zhengfei; Fu, Xiaolong; Sun, Menghong; Wei, Qiao; Li, Dali; Chen, Haiquan; Xiang, Jiaqing; Li, Hecheng; Zhang, Yawei; Zhao, Weixin; Zhao, Kuaile] Fudan Univ, Dept Oncol, Shanghai Med Coll, Shanghai 200032, Peoples R China.

RP Fu, XL (corresponding author), Fudan Univ, Dept Radiat Oncol, Shanghai Canc Ctr, 270 Dong An Rd, Shanghai 200032, Peoples R China.

EM xlfu1964@hotmail.com; kuaile_z@sina.com

RI Li, Dali/N-6000-2014; Li, Dali/G-4192-2016; Zhang, Yong/H-1774-2012

OI Zhang, Yong/0000-0002-5831-637X; wei, qiao/0000-0001-9476-6071

FU Scientific Research Foundation for the Returned Overseas Chinese

Scholars, State Education Ministry; Foundation of Shanghai Municipal

Commission of Health and Family Planning; Shanghai Committee of Science

and Technology, Natural Science Foundation of Shanghai, China

[11ZR1407300]

FX We thank the Department of Scientific Publication at The University of

Texas MD Anderson Cancer Center for editorial review of this paper. This

work was sponsored by the Scientific Research Foundation for the

Returned Overseas Chinese Scholars, State Education Ministry, and

Foundation of Shanghai Municipal Commission of Health and Family

Planning, and partially supported by Shanghai Committee of Science and

Technology, Natural Science Foundation of Shanghai, China (NO.

11ZR1407300).

CR Altomare DA, 2005, ONCOGENE, V24, P7455, DOI 10.1038/sj.onc.1209085

Baba Y, 2011, CANCER-AM CANCER SOC, V117, P1399, DOI 10.1002/cncr.25630

Bleeker FE, 2008, ONCOGENE, V27, P5648, DOI 10.1038/onc.2008.170

Carpten JD, 2007, NATURE, V448, P439, DOI 10.1038/nature05933

Courtney KD, 2010, J CLIN ONCOL, V28, P1075, DOI 10.1200/JCO.2009.25.3641

Dai DL, 2005, J CLIN ONCOL, V23, P1473, DOI 10.1200/JCO.2005.07.168

Davies MA, 2008, BRIT J CANCER, V99, P1265, DOI 10.1038/sj.bjc.6604637

Dillon RL, 2009, CANCER RES, V69, P5057, DOI 10.1158/0008-5472.CAN-08-4287

Ferlay J, 2010, INT J CANCER, V127, P2893, DOI 10.1002/ijc.25516

Gibault L, 2005, BRIT J CANCER, V93, P107, DOI 10.1038/sj.bjc.6602625

Gotoh M, 2007, JPN J CLIN ONCOL, V37, P652, DOI 10.1093/jjco/hym089

Hanawa M, 2006, INT J CANCER, V118, P1173, DOI 10.1002/ijc.21454

Herbst RS, 2004, INT J RADIAT ONCOL, V59, P21, DOI 10.1016/j.ijrobp.2003.11.041

Hirsch FR, 2008, CANCER-AM CANCER SOC, V112, P1114, DOI 10.1002/cncr.23282

Hoshino M, 2007, PATHOBIOLOGY, V74, P15, DOI 10.1159/000101047

Javle MM, 2006, J GASTROEN HEPATOL, V21, P1744, DOI 10.1111/j.1440-1746.2006.04373.x

Jemal A, 2011, CA-CANCER J CLIN, V61, P69, DOI [10.3322/caac.20107, 10.3322/caac.20115]

Kii T, 2007, JPN J CLIN ONCOL, V37, P583, DOI 10.1093/jjco/hym077

Kreisberg JI, 2004, CANCER RES, V64, P5232, DOI 10.1158/0008-5472.CAN-04-0272

Malanga D, 2008, CELL CYCLE, V7, P665, DOI 10.4161/cc.7.5.5485

Manning BD, 2007, CELL, V129, P1261, DOI 10.1016/j.cell.2007.06.009

Nakajima M, 2013, EXPERT OPIN PHARMACO, V14, P1345, DOI 10.1517/14656566.2013.801454

Schlieman MG, 2003, BRIT J CANCER, V89, P2110, DOI 10.1038/sj.bjc.6601396

Seshacharyulu P, 2012, EXPERT OPIN THER TAR, V16, P15, DOI 10.1517/14728222.2011.648617

Shah A, 2005, CLIN CANCER RES, V11, P2930, DOI 10.1158/1078-0432.CCR-04-1385

Skliris GP, 2008, HISTOPATHOLOGY, V52, P355, DOI 10.1111/j.1365-2559.2007.02955.x

Sunpaweravong P, 2005, J CANCER RES CLIN, V131, P111, DOI 10.1007/s00432-004-0610-7

Veeriah Selvaraju, 2012, J Chem Biol, V5, P115, DOI 10.1007/s12154-012-0076-z

Wang KL, 2007, CANCER-AM CANCER SOC, V109, P658, DOI 10.1002/cncr.22445

Wei QC, 2007, INT J ONCOL, V31, P493

Wilkinson NW, 2004, J GASTROINTEST SURG, V8, P448, DOI 10.1016/j.gassur.2004.01.006

Xiao XY, 2013, J TRANSL MED, V11, DOI 10.1186/1479-5876-11-151

Yamamoto Y, 2012, ANN SURG ONCOL, V19, P757, DOI 10.1245/s10434-011-2071-y

Yamazaki M, 2012, ONCOL REP, V28, P49, DOI 10.3892/or.2012.1803

Yoshioka A, 2008, ONCOL REP, V19, P1099

Yuan TL, 2008, ONCOGENE, V27, P5497, DOI 10.1038/onc.2008.245

Zhu ZF, 2014, ANN SURG ONCOL, V21, P2857, DOI 10.1245/s10434-014-3665-y

Zhu ZF, 2013, ANN SURG ONCOL, V20, P3052, DOI 10.1245/s10434-013-2987-5

NR 38

TC 14

Z9 14

U1 1

U2 7

PU BMC

PI LONDON

PA CAMPUS, 4 CRINAN ST, LONDON N1 9XW, ENGLAND

EI 1756-9966

J9 J EXP CLIN CANC RES

JI J. Exp. Clin. Cancer Res.

PD SEP 4

PY 2015

VL 34

AR 95

DI 10.1186/s13046-015-0212-z

PG 8

WC Oncology

WE Science Citation Index Expanded (SCI-EXPANDED)

SC Oncology

GA CQ3SS

UT WOS:000360523400005

PM 26338103

OA Green Published, gold

DA 2022-05-19

ER

PT J

AU Wang, NN

Tan, BX

Cao, FL

Song, QX

Wang, JB

Jia, YB

Cheng, YF

AF Wang, Nana

Tan, Bingxu

Cao, Fangli

Song, Qingxu

Wang, Jianbo

Jia, Yibin

Cheng, Yufeng

TI Prognostic influence of smoking on esophageal squamous cell carcinoma

SO INTERNATIONAL JOURNAL OF CLINICAL AND EXPERIMENTAL MEDICINE

LA English

DT Article

DE Smoking; esophageal squamous cell carcinoma; prognosis

ID GASTRIC CARDIA; CANCER; TOBACCO; SURVIVAL; TRENDS; IMPACT; RISK

AB Background: Smoking is well-known as a risk factor for esophageal squamous cell carcinoma. However, little is known about the effect of this factor on survival. Methods: Esophageal cancer patients who underwent esophagectomy without any preoperative therapy were retrospectively reviewed. Patients' postoperative overall and disease-free survivals were compared between 2 groups (non-heavy smokers and heavy smokers). Results: A total of 109 cases were evaluated in this study. The 5-year OS rate of the non-heavy smokers was 53.2% and 32.3% for the heavy group. The 5-year DFS rate of the non-heavy smokers was 51.1% and 27.4% for the heavy group. Kaplan-Meier survival analysis revealed that heavy smokers had significantly poorer OS (P=0.018) and DFS (P=0.009) than non-heavy smokers. In multivariate analysis, smoking was found to be an independent prognostic factor for OS (P=0.003; HR: 2.186; 95% CI: 1.309-3.650) and DFS (P=0.001; HR: 2.471; 95% CI: 1.467-4.163). Conclusion: Smoking was associated with survival among patients with ESCC, and it was recognized as an independent factor in both OS and DFS.

C1 [Wang, Nana; Tan, Bingxu; Cao, Fangli; Song, Qingxu; Wang, Jianbo; Jia, Yibin; Cheng, Yufeng] Shandong Univ, Qilu Hosp, Dept Radiat Oncol, 107 West Wenhua Rd, Jinan 250012, Peoples R China.

RP Cheng, YF (corresponding author), Shandong Univ, Qilu Hosp, Dept Radiat Oncol, 107 West Wenhua Rd, Jinan 250012, Peoples R China.

EM qlcyf@sdu.edu.cn

OI cheng, yufeng/0000-0002-4143-5399

FU Academics Independent Innovation Project of Jinan City [201401252]

FX This study was supported by the Academics Independent Innovation Project

of Jinan City (201401252).

CR Castro C, 2014, ANN ONCOL, V25, P283, DOI 10.1093/annonc/mdt486

Coleman HG, 2012, GASTROENTEROLOGY, V142, P233, DOI 10.1053/j.gastro.2011.10.034

Cook MB, 2009, BRIT J CANCER, V101, P855, DOI 10.1038/sj.bjc.6605246

Ishiguro S, 2009, CANCER LETT, V275, P240, DOI 10.1016/j.canlet.2008.10.020

Lagergren J, 2000, INT J CANCER, V85, P340

Lee CH, 2012, INT J CANCER, V131, pE733, DOI 10.1002/ijc.27401

Lin YB, 2012, CANCER SCI, V103, P1985, DOI 10.1111/cas.12000

Lu CL, 2010, CANCER CAUSE CONTROL, V21, P269, DOI 10.1007/s10552-009-9458-0

Nozoe T, 2002, CANCER LETT, V184, P49, DOI 10.1016/S0304-3835(02)00188-X

Otterstatter MC, 2012, CAN J GASTROENTEROL, V26, P723, DOI 10.1155/2012/649108

Ramus JR, 2012, EUR J CANCER PREV, V21, P507, DOI 10.1097/CEJ.0b013e328350b06f

Shitara K, 2010, CANCER SCI, V101, P1001, DOI 10.1111/j.1349-7006.2009.01466.x

Sundelof M, 2008, EUR J CANCER, V44, P1566, DOI 10.1016/j.ejca.2008.04.002

Thrift AP, 2012, INT J CANCER, V131, pE759, DOI 10.1002/ijc.27420

Torre LA, 2015, CA-CANCER J CLIN, V65, P87, DOI 10.3322/caac.21262

Tramacere I, 2011, EPIDEMIOLOGY, V22, P344, DOI 10.1097/EDE.0b013e31821092cd

Wu IC, 2013, PLOS ONE, V8, DOI 10.1371/journal.pone.0055834

Yoshino I, 2007, SURG TODAY, V37, P725, DOI 10.1007/s00595-007-3516-6

NR 18

TC 6

Z9 6

U1 1

U2 2

PU E-CENTURY PUBLISHING CORP

PI MADISON

PA 40 WHITE OAKS LN, MADISON, WI 53711 USA

SN 1940-5901

J9 INT J CLIN EXP MED

JI Int. J. Clin. Exp. Med.

PY 2015

VL 8

IS 10

BP 18867

EP 18872

PG 6

WC Medicine, Research & Experimental

WE Science Citation Index Expanded (SCI-EXPANDED)

SC Research & Experimental Medicine

GA DA3BB

UT WOS:000367669800217

PM 26770509

DA 2022-05-19

ER

PT J

AU Chen, DY

Zhang, ZY

Mao, CM

Zhou, YP

Yu, LC

Yin, Y

Wu, S

Mou, X

Zhu, Y

AF Chen, Deyu

Zhang, Zhaoyue

Mao, Chaoming

Zhou, Yuepeng

Yu, Lichao

Yin, Yue

Wu, Shi

Mou, Xiao

Zhu, Yan

TI ANRIL inhibits p15(INK4b) through the TGF beta 1 signaling pathway in

human esophageal squamous cell carcinoma

SO CELLULAR IMMUNOLOGY

LA English

DT Article

DE Long non-coding RNA; Antisense non-coding RNA in the INK4 locus;

Transforming growth factor beta 1; p15(INk4b); Esophageal squamous cell

carcinoma

ID GENOME-WIDE ASSOCIATION; LONG NONCODING RNAS; CANCER; LOCUS;

SUSCEPTIBILITY; SURVIVAL; POLYCOMB

AB The INK4b-ARF-INK4a gene cluster encodes three tumor suppressors: p15(INK4b), pl4(ARF), and p 6(INK4a). Antisense non-coding RNA in the INK4 locus (ANRIL) is transcribed in the opposite direction from this gene cluster. Recent studies suggest that ANRIL represses the expression of p15(INK4b), p14(ARF), and p16(INK4a); however, the underlying mechanism is unclear. In this study, the expressions of ANRIL in human esophageal squamous cell carcinoma (ESCC) tissues and matched adjacent non-tumor tissues were examined by quantitative real-time polymerase chain reaction. Compared with matched adjacent non-tumor tissues, the expression levels of ANRIL in ESCC tissues were significantly increased. Furthermore, inhibition of ANRIL was found to increase the expression of pl 15(INK4b) and transforming growth factor beta 1 (TGF beta 1) and depletion of ANRIL in ESCC cell lines may inhibit cellular proliferation. Thus, our findings suggest a significant role of ANRIL in the occurrence and development of ESCC through TGF beta 1 signaling pathways. (C) 2014 Elsevier Inc. All rights reserved.

C1 [Chen, Deyu; Mao, Chaoming; Yin, Yue; Wu, Shi; Mou, Xiao] Jiangsu Univ, Affiliated Hosp, Inst Oncol, Zhenjiang 212001, Peoples R China.

[Zhang, Zhaoyue; Zhou, Yuepeng; Zhu, Yan] Jiangsu Univ, Sch Clin Med, Zhenjiang 212001, Peoples R China.

[Yu, Lichao] Jiangsu Univ, Affiliated Hosp, Dept Thorac Surg, Zhenjiang 212001, Peoples R China.

RP Mao, CM (corresponding author), Jiangsu Univ, Affiliated Hosp, Inst Oncol, Jiefang Rd 438, Zhenjiang 212001, Peoples R China.

EM jq1001@ujs.edu.cn

FU Natural Science Foundation of Jiangsu Province [BK20131245, BK20131248];

Science and Technology Commission of Zhenjiang Municipality [SH2012023,

SH2013031]

FX This study was supported by grants from the Natural Science Foundation

of Jiangsu Province (BK20131245, BK20131248), and the Science and

Technology Commission of Zhenjiang Municipality (SH2012023, SH2013031).

CR Aguilo F, 2011, CANCER RES, V71, P5365, DOI 10.1158/0008-5472.CAN-10-4379

Broadbent HM, 2008, HUM MOL GENET, V17, P806, DOI 10.1093/hmg/ddm352

Cugino D, 2012, NUTR METAB CARDIOVAS, V22, P619, DOI 10.1016/j.numecd.2010.11.010

Emanuele E, 2011, NEUROBIOL AGING, V32, P1231, DOI 10.1016/j.neurobiolaging.2009.07.003

Gutschner T, 2012, RNA BIOL, V9, P703, DOI 10.4161/rna.20481

Kotake Y, 2011, ONCOGENE, V30, P1956, DOI 10.1038/onc.2010.568

Mercer TR, 2009, NAT REV GENET, V10, P155, DOI 10.1038/nrg2521

MOSMANN T, 1983, J IMMUNOL METHODS, V65, P55, DOI 10.1016/0022-1759(83)90303-4

NIGG EA, 1995, BIOESSAYS, V17, P471, DOI 10.1002/bies.950170603

Pardali E, 2010, TRENDS CELL BIOL, V20, P556, DOI 10.1016/j.tcb.2010.06.006

Pasmant E, 2007, CANCER RES, V67, P3963, DOI 10.1158/0008-5472.CAN-06-2004

Rajaraman P, 2012, HUM GENET, V131, P1877, DOI 10.1007/s00439-012-1212-0

Sherborne AL, 2010, NAT GENET, V42, P492, DOI 10.1038/ng.585

Tano Keiko, 2012, Frontiers in Genetics, V3, P219, DOI 10.3389/fgene.2012.00219

Turnbull C, 2010, NAT GENET, V42, P504, DOI 10.1038/ng.586

Uno S, 2010, NAT GENET, V42, P707, DOI 10.1038/ng.612

Wilusz JE, 2009, GENE DEV, V23, P1494, DOI 10.1101/gad.1800909

Yap KL, 2010, MOL CELL, V38, P662, DOI 10.1016/j.molcel.2010.03.021

Zhang YH, 2010, CANCER LETT, V291, P76, DOI 10.1016/j.canlet.2009.10.003

Zheng CG, 2009, CANCER RES, V69, P2324, DOI 10.1158/0008-5472.CAN-08-4113

Zhuang JH, 2012, PLOS ONE, V7, DOI 10.1371/journal.pone.0047193

NR 21

TC 90

Z9 99

U1 0

U2 23

PU ACADEMIC PRESS INC ELSEVIER SCIENCE

PI SAN DIEGO

PA 525 B ST, STE 1900, SAN DIEGO, CA 92101-4495 USA

SN 0008-8749

EI 1090-2163

J9 CELL IMMUNOL

JI Cell. Immunol.

PD MAY-JUN

PY 2014

VL 289

IS 1-2

BP 91

EP 96

DI 10.1016/j.cellimm.2014.03.015

PG 6

WC Cell Biology; Immunology

WE Science Citation Index Expanded (SCI-EXPANDED)

SC Cell Biology; Immunology

GA AI7ZG

UT WOS:000337120900014

PM 24747824

DA 2022-05-19

ER

PT J

AU Wang, M

An, SH

Wang, DY

Ji, HZ

Geng, M

Guo, XJ

Wang, ZJ

AF Wang, Ming

An, Shuhong

Wang, Diyi

Ji, Haizhen

Geng, Min

Guo, Xingjing

Wang, Zhaojin

TI Quantitative Proteomics Identify the Possible Tumor Suppressive Role of

Protease-Activated Receptor-4 in Esophageal Squamous Cell Carcinoma

Cells

SO PATHOLOGY & ONCOLOGY RESEARCH

LA English

DT Article

DE Proteomic; Protease-activated receptors-4; Apoptosis; Tumor suppressor;

Esophageal squamous cell carcinoma

ID DECREASED EXPRESSION; IDENTIFICATION; PROLIFERATION; GENE

AB Exposure to carcinogens of tobacco smoke may result in methylation of protease-activated receptors-4 (PAR4) gene and further induces the loss of PAR4 expression, which is considered to be involved in carcinogenesis of esophageal squamous cell carcinoma (ESCC). Here we employed a TMT-based quantitative proteomic approach to identify PAR4-regulated changes of proteomic profiles in ESCC cells and to identify potentially therapeutic value. A total of 33 proteins were found significantly changed with 15 up-regulated and 18 down-regulated in PAR4-activating peptide (PAR4-AP) treated ESCC cells compared with controls. Bioinformatics analysis showed that key higher expressed proteins included those associated with apoptosis and tumor suppressor (e.g. CASP9), and lower expressed proteins included those associated with anti-apoptosis, autophagy and promoting cell proliferation (e.g. CHMP1B, PURA, PARG and HIST1H2AH). Western blot verified changes in five representative proteins including CASP9, CHMP1B, PURA, PARG and HIST1H2AH. Immunohistochemistry analysis showed that CHMP1B, PURA, PARG and HIST1H2AH expression in ESCC tissues were significantly higher than those in adjacent nontumorous tissues. Our findings will be helpful in further investigations into the functions and molecular mechanisms of PAR4 in ESCC.

C1 [Wang, Ming; An, Shuhong; Geng, Min; Wang, Zhaojin] Taishan Med Univ, Dept Human Anat, 2 Ying Sheng Dong Lu, Tai An 271000, Shandong, Peoples R China.

[Wang, Diyi] Taishan Med Univ, Affiliated Hosp, Dept Pathol, Tai An 271000, Shandong, Peoples R China.

[Ji, Haizhen; Guo, Xingjing] Taishan Med Univ, Dept Physiol, Tai An, Shandong, Peoples R China.

RP Wang, ZJ (corresponding author), Taishan Med Univ, Dept Human Anat, 2 Ying Sheng Dong Lu, Tai An 271000, Shandong, Peoples R China.

EM zjwang@tsmc.edu.cn

FU National Natural Science Foundation of China [81371234]; Natural Science

Foundation of Shandong Province, China [2016ZRB14346]

FX The study was supported by National Natural Science Foundation of China

(81371234) and Natural Science Foundation of Shandong Province, China

(2016ZRB14346). The authors would like to thank Dr. Zhen Ye for skillful

assistance in Gene Ontology (GO) functional and Kyoto Encyclopedia of

Genes and Genomes (KEGG) pathway enrichment analysis.

CR Bhatnagar S, 2014, NATURE, V516, P116, DOI 10.1038/nature13955

Cox J, 2008, NAT BIOTECHNOL, V26, P1367, DOI 10.1038/nbt.1511

Elste AP, 2010, J MOL HISTOL, V41, P89, DOI 10.1007/s10735-010-9274-6

Fasanelli F, 2015, NAT COMMUN, V6, DOI 10.1038/ncomms10192

Hu BY, 2015, TUMOR BIOL, V36, P2569, DOI 10.1007/s13277-014-2873-1

Kim B, 2015, EXPERT OPIN THER TAR, V19, P113, DOI 10.1517/14728222.2014.961425

Kolpakov MA, 2016, J MOL CELL CARDIOL, V90, P21, DOI 10.1016/j.yjmcc.2015.11.030

Lee S, 2014, NEOPLASMA, V61, P546, DOI 10.4149/neo_2014_067

Lin ZW, 2014, INT J ONCOL, V45, P57, DOI 10.3892/ijo.2014.2436

Liu H, 2002, NUCLEIC ACIDS RES, V30, P2417, DOI 10.1093/nar/30.11.2417

Loeser H, 2017, MOL CLIN ONCOL, V7, P225, DOI 10.3892/mco.2017.1286

Michael JV, 2017, BLOOD, V130, P567, DOI 10.1182/blood-2016-11-751099

Ohashi S, 2015, GASTROENTEROLOGY, V149, P1700, DOI 10.1053/j.gastro.2015.08.054

Ramachandran R, 2008, BRIT J PHARMACOL, V153, pS263, DOI 10.1038/sj.bjp.0707507

Shahmoradgoli M, 2011, INT J CANCER, V128, P2865, DOI 10.1002/ijc.25632

Spitzer C, 2015, PLANT CELL, V27, P391, DOI 10.1105/tpc.114.135939

Steevens J, 2010, GUT, V59, P39, DOI 10.1136/gut.2009.191080

Tanaka F, 2010, GUT, V59, P1457, DOI 10.1136/gut.2009.205724

Vergnolle N, 2000, ALIMENT PHARM THER, V14, P257

Wang HB, 2004, NATURE, V431, P873, DOI 10.1038/nature02985

Zhang Y, 2011, INT J BIOCHEM CELL B, V43, P1277, DOI 10.1016/j.biocel.2011.05.008

Zhou CH, 2012, BREAST CANCER RES, V14, DOI 10.1186/bcr3144

Zhou JF, 2016, AUTOPHAGY, V12, P515, DOI 10.1080/15548627.2015.1136770

NR 23

TC 11

Z9 11

U1 0

U2 5

PU SPRINGER

PI DORDRECHT

PA VAN GODEWIJCKSTRAAT 30, 3311 GZ DORDRECHT, NETHERLANDS

SN 1219-4956

EI 1532-2807

J9 PATHOL ONCOL RES

JI Pathol. Oncol. Res.

PD JUL

PY 2019

VL 25

IS 3

BP 937

EP 943

DI 10.1007/s12253-018-0395-7

PG 7

WC Oncology; Pathology

WE Science Citation Index Expanded (SCI-EXPANDED)

SC Oncology; Pathology

GA II9YX

UT WOS:000475558500012

PM 29502225

DA 2022-05-19

ER

PT J

AU Dong, GC

Chen, Q

Jiang, F

Yu, DC

Mao, QX

Xia, WJ

Shi, R

Wang, J

Xu, L

AF Dong, Gaochao

Chen, Qiang

Jiang, Feng

Yu, Decai

Mao, Qixing

Xia, Wenjie

Shi, Run

Wang, Jie

Xu, Lin

TI Diisopropylamine dichloroacetate enhances radiosensitization in

esophageal squamous cell carcinoma by increasing mitochondria-derived

reactive oxygen species levels

SO ONCOTARGET

LA English

DT Article

DE diisopropylamine dichloroacetate; esophageal squamous cell carcinoma;

ROS; radiosensitization

ID LACTATE-DEHYDROGENASE 5; PROSTATE-CANCER; IN-VITRO; RADIOTHERAPY;

METABOLISM; HYPOXIA; OPPORTUNITIES; SENSITIZES

AB Radiotherapy is generally applied in the treatment of esophageal squamous cell carcinoma (ESCC). However, the radioresistance of ESCC still remains an obstacle for the curative effect of this treatment. We hypothesized that diisopropylamine dichloroacetate (DADA), an inhibitor of pyruvate dehydrogenase kinase (PDK), might enhance radiosensitizationin resistant ESCC. The clonogenic survival assay revealed that DADA sensitized ESCC cells to radiotherapy in vitro; furthermore, the combination of DADA and radiotherapy increased the expression of gamma-H2AX, which is a hallmark of DNA double-strand breaks. Arrest at G2/M phase as well as the induction of apoptosis of ESCC cells were also observed in the cells treated with the combination of DADA and radiotherapy. Notably, xenograft tumor growth was significantly suppressed in vivo by combined radiotherapy and DADA administration. It has been proven that glycolysis is highly correlated with radioresistance, which could be reversed by the shift from glycolysis to mitochondrial oxidation. In our present study, we found that DADA could modulate oxidative phosphorylation as well as increase the intracellular levels of reactive oxygen species (ROS). Collectively, we concluded that DADA-induced intracellular ROS accumulation was identified as the key factor of radiotherapy sensitization of ESCC.

C1 [Dong, Gaochao; Jiang, Feng; Mao, Qixing; Xia, Wenjie; Shi, Run; Wang, Jie; Xu, Lin] Nanjing Med Univ, Affiliated Canc Hosp, Canc Inst Jiangsu Prov, Jiangsu Key Lab Mol & Translat Canc Res,Dept Thor, Nanjing, Jiangsu, Peoples R China.

[Chen, Qiang; Mao, Qixing; Xia, Wenjie; Shi, Run; Xu, Lin] Nanjing Med Univ, Dept Clin Coll 4, Nanjing, Jiangsu, Peoples R China.

[Chen, Qiang] Xuzhou Ctr Hosp, Dept Thorac Surg, Xuzhou, Jiangsu, Peoples R China.

[Yu, Decai] Nanjing Univ, Affiliated Drum Tower Hosp, Coll Med, Dept Hepatobiliary Surg, Nanjing, Jiangsu, Peoples R China.

RP Xu, L (corresponding author), Nanjing Med Univ, Affiliated Canc Hosp, Canc Inst Jiangsu Prov, Jiangsu Key Lab Mol & Translat Canc Res,Dept Thor, Nanjing, Jiangsu, Peoples R China.; Xu, L (corresponding author), Nanjing Med Univ, Dept Clin Coll 4, Nanjing, Jiangsu, Peoples R China.

EM xulin83cn@gmail.com

FU Natural Science Foundation of China [81472702, 81501977]; Foundation of

Jiangsu Cancer Hospital [ZQ201509]; Innovation Capability Development

Project of Jiangsu Province [BM2015004]

FX This research was supported by the Natural Science Foundation of China

(Nos. 81472702, 81501977), Foundation of Jiangsu Cancer Hospital (No.

ZQ201509) and the Innovation Capability Development Project of Jiangsu

Province (No. BM2015004).

CR Augoff K, 2015, CANCER LETT, V358, P1, DOI 10.1016/j.canlet.2014.12.035

Bol V, 2015, ACTA ONCOL, V54, P266, DOI 10.3109/0284186X.2014.932006

Bonnet S, 2007, CANCER CELL, V11, P37, DOI 10.1016/j.ccr.2006.10.020

Cao WG, 2008, PROSTATE, V68, P1223, DOI 10.1002/pros.20788

D'Journo XB, 2014, J THORAC DIS, V6, pS253, DOI 10.3978/j.issn.2072-1439.2014.04.16

Fu HL, 2013, CELL BIOCHEM BIOPHYS, V67, P657, DOI 10.1007/s12013-013-9554-3

Hanahan D, 2011, CELL, V144, P646, DOI 10.1016/j.cell.2011.02.013

HERBERT V, 1979, AM J CLIN NUTR, V32, P1534, DOI 10.1093/ajcn/32.7.1534

Jang M, 2013, EXP MOL MED, V45, DOI 10.1038/emm.2013.85

Jemal A, 2011, CA-CANCER J CLIN, V61, P69, DOI [10.3322/caac.20107, 10.3322/caac.20115]

Kamangar F, 2006, J CLIN ONCOL, V24, P2137, DOI 10.1200/JCO.2005.05.2308

Koukourakis MI, 2014, BRIT J CANCER, V110, P2217, DOI 10.1038/bjc.2014.158

Li W, 2014, MED ONCOLOGY, V31

Meijer TWH, 2012, CLIN CANCER RES, V18, P5585, DOI 10.1158/1078-0432.CCR-12-0858

Meng MB, 2015, CANCER LETT, V356, P985, DOI 10.1016/j.canlet.2014.11.016

Moeller BJ, 2007, CANCER METAST REV, V26, P241, DOI 10.1007/s10555-007-9056-0

Nishida N, 2014, J RADIAT RES, V55, P455, DOI 10.1093/jrr/rrt142

Pennathur A, 2013, LANCET, V381, P400, DOI 10.1016/S0140-6736(12)60643-6

Ren Y, 2013, MOL CELL PROTEOMICS, V12, P485, DOI 10.1074/mcp.M112.018325

Sattler UGA, 2009, INT J RADIAT BIOL, V85, P963, DOI 10.3109/09553000903258889

Schaue D, 2015, NAT REV CLIN ONCOL, V12, P527, DOI 10.1038/nrclinonc.2015.120

Schweigert M, 2013, NAT REV GASTRO HEPAT, V10, P230, DOI 10.1038/nrgastro.2012.236

Shen H, 2015, MOL CANC THERAPEUTIC

Shen H, 2015, MOL CANCER THER, V14, P1794, DOI 10.1158/1535-7163.MCT-15-0247

Shen YA, 2013, EVID-BASED COMPL ALT, V2013, DOI 10.1155/2013/590393

Szumiel I, 2015, INT J RADIAT BIOL, V91, P1, DOI 10.3109/09553002.2014.934929

Wang J, 2008, CANCER BIOL THER, V7, P1875, DOI 10.4161/cbt.7.12.7067

WARBURG O, 1956, SCIENCE, V123, P309, DOI 10.1126/science.123.3191.309

WARD J F, 1987, British Journal of Cancer, V55, P105

Xiao L, 2014, ONCOGENE, V33, P4568, DOI 10.1038/onc.2014.32

Yahya N, 2014, RADIAT ONCOL, V9, DOI 10.1186/s13014-014-0282-7

Yamane K, 2014, PLOS ONE, V9, DOI 10.1371/journal.pone.0098032

Yang YZ, 2013, J CELL BIOCHEM, V114, P967, DOI 10.1002/jcb.24438

NR 33

TC 12

Z9 13

U1 0

U2 7

PU IMPACT JOURNALS LLC

PI ORCHARD PARK

PA 6666 E QUAKER ST, STE 1, ORCHARD PARK, NY 14127 USA

EI 1949-2553

J9 ONCOTARGET

JI Oncotarget

PD OCT 18

PY 2016

VL 7

IS 42

BP 68170

EP 68178

DI 10.18632/oncotarget.11906

PG 9

WC Oncology; Cell Biology

WE Science Citation Index Expanded (SCI-EXPANDED)

SC Oncology; Cell Biology

GA EB5UT

UT WOS:000387446800038

PM 27626688

OA Green Published, Green Submitted, gold

DA 2022-05-19

ER

PT J

AU Xue, ZF

Wu, XM

Chen, X

Luo, Q

AF Xue, Zengfu

Wu, Xiumin

Chen, Xiong

Luo, Qi

TI MT3-MMP down-regulation promotes tumorigenesis and correlates to poor

prognosis in esophageal squamous cell carcinoma

SO CANCER MEDICINE

LA English

DT Article

DE Cell cycle; esophageal squamous cell carcinoma; immunohistochemistry;

MT3-MMP; prognosis

ID MATRIX-METALLOPROTEINASE; GASTRIC-CANCER; EXPRESSION; ACTIVATION;

KINASE; METASTASIS; TRANSITION; INVASION; MT1-MMP

AB The membrane-type matrix metalloproteinases (MT-MMPs) play an important role in degrading the extracellular matrix (ECM) and facilitating protease-dependent tumor progression and invasion. Here, we report that unlike MT1-MMP, MT3-MMP was down-regulated in esophageal squamous cell carcinoma (ESCC) as detected by real-time PCR (qPCR), Western blot analysis, and immunohistochemistry (IHC). Down-regulation of MT3-MMP was observed at protein level in 66.3% of ESCC specimens (by IHC, n=86) for routine pathologic diagnosis, as well as at mRNA level in 63.3% of surgically resected ESCC tumors paired with surrounding nontumor tissues (by qPCR, n=30). Notably, MT3-MMP down-regulation significantly correlated with lymph node metastasis and poor overall survival of patients with ESCC (median 5-year survival=50.69 vs. 30.77months for patients with MT3-MMP-negative and -positive ESCC, respectively). Mechanistically, MT3-MMP negatively regulated proliferation, colony formation, and migration of ESCC cells, in association with cell cycle arrest at G1, due to up-regulation of p21(Cip1) and p27(Kip1). Together, as a tumor suppressor in ESCC, MT3-MMP down-regulation represents an unfavorable factor for prognosis of patients with ESCC.

C1 [Xue, Zengfu; Luo, Qi] Xiamen Univ, Affiliated Hosp 1, Xiamen Canc Hosp, Xiamen, Fujian, Peoples R China.

[Wu, Xiumin] Xiamen Univ, Affiliated Hosp 1, Dept Pharm, Xiamen, Fujian, Peoples R China.

[Chen, Xiong] Xiamen Univ, Affiliated Dongfang Hosp, Dept Med Oncol, Fuzhou, Fujian, Peoples R China.

RP Xue, ZF; Luo, Q (corresponding author), Xiamen Univ, Affiliated Hosp 1, 10 Shanggu Rd, Xiamen 361003, Fujian, Peoples R China.

EM zengfuxue@aliyun.com; zfxue@xmu.edu.cn

FU National Natural Science Foundation of China [81201674]; Nature Science

Foundation of Fujian Province [2016J01638, 2012D051]

FX This study was supported by the National Natural Science Foundation of

China (Grant No. 81201674), Nature Science Foundation of Fujian Province

(Grant No. 2016J01638, 2012D051).

CR Balbin M, 2003, NAT GENET, V35, P252, DOI 10.1038/ng1249

Chen LJ, 2010, ANTICANCER RES, V30, P4363

Cheung LWT, 2006, CANCER RES, V66, P10902, DOI 10.1158/0008-5472.CAN-06-2217

Decock J, 2011, J CELL MOL MED, V15, P1254, DOI 10.1111/j.1582-4934.2011.01302.x

Enzinger PC, 2003, NEW ENGL J MED, V349, P2241, DOI 10.1056/NEJMra035010

Etoh T, 2000, GUT, V47, P50, DOI 10.1136/gut.47.1.50

Gong TQ, 2012, CANCER BIOL THER, V13, P606, DOI 10.4161/cbt.19851

Hadler-Olsen E, 2011, FEBS J, V278, P28, DOI 10.1111/j.1742-4658.2010.07920.x

Hotary KB, 2003, CELL, V114, P33, DOI 10.1016/S0092-8674(03)00513-0

Jha KN, 2010, J BIOL CHEM, V285, P35180, DOI 10.1074/jbc.M110.134767

Kang TB, 2000, FASEB J, V14, P2559, DOI 10.1096/fj.00-0269com

Ling QZ, 2014, J CELL PHYSIOL, V229, P108, DOI 10.1002/jcp.24425

Lowy AM, 2006, CANCER RES, V66, P4734, DOI 10.1158/0008-5472.CAN-05-4268

Nie F, 2010, INT J ONCOL, V37, P1261, DOI 10.3892/ijo_00000777

Ning XX, 2012, PLOS ONE, V7, DOI 10.1371/journal.pone.0030185

Pahwa S, 2014, CANCERS, V6, P416, DOI 10.3390/cancers6010416

Parkin DM, 2005, CA-CANCER J CLIN, V55, P74, DOI 10.3322/canjclin.55.2.74

Rines AK, 2012, FASEB J, V26, P4685, DOI 10.1096/fj.12-212282

Shofuda K, 2001, FASEB J, V15, P2010, DOI 10.1096/fj.00-0871fje

Tatti O, 2015, CANCER RES, V75, P2083, DOI 10.1158/0008-5472.CAN-14-1923

Tatti O, 2011, PLOS ONE, V6, DOI 10.1371/journal.pone.0028325

Wolf K, 2007, NAT CELL BIOL, V9, P893, DOI 10.1038/ncb1616

Xue YJ, 2012, J TRANSL MED, V10, DOI 10.1186/1479-5876-10-200

Xue ZF, 2015, J CELL BIOCHEM, V116, P618, DOI 10.1002/jcb.25013

Zhao HR, 2004, J BIOL CHEM, V279, P8592, DOI 10.1074/jbc.M308708200

Zitka O, 2010, CURR MED CHEM, V17, P3751, DOI 10.2174/092986710793213724

NR 26

TC 9

Z9 9

U1 0

U2 2

PU WILEY-BLACKWELL

PI HOBOKEN

PA 111 RIVER ST, HOBOKEN 07030-5774, NJ USA

SN 2045-7634

J9 CANCER MED-US

JI Cancer Med.

PD SEP

PY 2016

VL 5

IS 9

BP 2459

EP 2468

DI 10.1002/cam4.790

PG 10

WC Oncology

WE Science Citation Index Expanded (SCI-EXPANDED)

SC Oncology

GA DY8CG

UT WOS:000385355500032

PM 27292876

OA Green Published, gold

DA 2022-05-19

ER

PT J

AU Takabatake, K

Konishi, H

Arita, T

Kataoka, S

Shibamoto, J

Furuke, H

Takaki, W

Shoda, K

Shimizu, H

Yamamoto, Y

Komatsu, S

Shiozaki, A

Fujiwara, H

Okamoto, K

Otsuji, E

AF Takabatake, Kazuya

Konishi, Hirotaka

Arita, Tomohiro

Kataoka, Satoshi

Shibamoto, Jun

Furuke, Hirotaka

Takaki, Wataru

Shoda, Katsutoshi

Shimizu, Hiroki

Yamamoto, Yusuke

Komatsu, Shuhei

Shiozaki, Atsushi

Fujiwara, Hitoshi

Okamoto, Kazuma

Otsuji, Eigo

TI Anterior gradient 2 regulates cancer progression in TP53-wild-type

esophageal squamous cell carcinoma

SO ONCOLOGY REPORTS

LA English

DT Article

DE anterior gradient 2; esophageal squamous cell carcinoma; TP53 mutation;

phosphorylation of p53; prognostic factor

ID ESTROGEN-RECEPTOR; AGR2 EXPRESSION; P53 PROTEIN; GENE; METASTASIS;

GROWTH; HEAD; OVEREXPRESSION; ADENOCARCINOMA; PROLIFERATION

AB Anterior gradient 2 (AGR2) reportedly promotes tumor growth and has an unfavorable impact on survival in several cancers. However, no comprehensive functional analysis of AGR2 in esophageal squamous cell carcinoma (ESCC) has been performed. In the present study, the function and clinical significance of AGR2 were examined using ESCC cell lines and clinical samples. AGR2 was upregulated in EC tissue and ESCC cell lines. The downregulation of AGR2 suppressed cell proliferation and increased the proportion of G2/M-phase cells and phosphorylation of p53 in TP53-wild-type ESCC and osteosarcoma cells. However, these changes were not observed in TP53-mutant ESCC cells. In addition, immunohistochemistry results demonstrated that high AGR2 and low p53 expression levels in ESCC tissues were correlated with a worse prognosis. These results suggested that although AGR2 enhanced cell proliferation by inhibiting p53 phosphorylation in TP53-wild-type ESCC, the same mechanism did not regulate cell functions in TP53-mutant ESCC. Thus, AGR2 served an important role in ESCC progression and might be a useful prognostic marker in patients with TP53-wild-type ESCC.

C1 [Takabatake, Kazuya; Konishi, Hirotaka; Arita, Tomohiro; Kataoka, Satoshi; Shibamoto, Jun; Furuke, Hirotaka; Takaki, Wataru; Shimizu, Hiroki; Yamamoto, Yusuke; Komatsu, Shuhei; Shiozaki, Atsushi; Fujiwara, Hitoshi; Okamoto, Kazuma; Otsuji, Eigo] Kyoto Prefectural Univ Med, Div Digest Surg, Dept Surg, Kamigyo Ku, 465 Kajii Cho, Kyoto 6028566, Japan.

[Shoda, Katsutoshi] Univ Yamanashi, Dept Surg 1, Fac Med, Yamanashi 4093898, Japan.

RP Konishi, H (corresponding author), Kyoto Prefectural Univ Med, Div Digest Surg, Dept Surg, Kamigyo Ku, 465 Kajii Cho, Kyoto 6028566, Japan.

EM h-koni7@koto.kpu-m.ac.jp

RI Arita, Tomohiro/AFV-4071-2022

OI Arita, Tomohiro/0000-0001-7127-6504

CR Alavi M, 2015, BMC CANCER, V15, DOI 10.1186/s12885-015-1658-2

Albrechtsen N, 1999, ONCOGENE, V18, P7706, DOI 10.1038/sj.onc.1202952

Armes JE, 2013, PATHOLOGY, V45, P49, DOI 10.1097/PAT.0b013e32835bd561

Arumugam T, 2015, MOL CANCER THER, V14, P941, DOI 10.1158/1535-7163.MCT-14-0470

Barnas C, 1997, INT J CANCER, V71, P79, DOI 10.1002/(SICI)1097-0215(19970328)71:1<79::AID-IJC14>3.0.CO;2-4

BENNETT WP, 1991, ONCOGENE, V6, P1779

Brierley JD., 2016, TNM CLASSIFICATION M, V8th ed.

Brychtova V, 2011, CANCER LETT, V304, P1, DOI 10.1016/j.canlet.2010.12.023

Cohen DJ, 2015, J CLIN ONCOL, V33, P1754, DOI 10.1200/JCO.2014.59.7765

DiMaio MA, 2012, HUM PATHOL, V43, P1799, DOI 10.1016/j.humpath.2012.03.019

Ferlay J, 2015, INT J CANCER, V136, pE359, DOI 10.1002/ijc.29210

FINLAY CA, 1988, MOL CELL BIOL, V8, P531, DOI 10.1128/MCB.8.2.531

Gray TA, 2014, MOL BIOSYST, V10, P1409, DOI 10.1039/c4mb00113c

Hrstka R, 2016, MOL ONCOL, V10, P652, DOI 10.1016/j.molonc.2015.12.003

Hrstka R, 2013, CANCER LETT, V333, P187, DOI 10.1016/j.canlet.2013.01.034

Hu ZY, 2012, CARCINOGENESIS, V33, P1178, DOI 10.1093/carcin/bgs141

Janssens V, 2000, J BIOL CHEM, V275, P20488, DOI 10.1074/jbc.M909370199

Joruiz SM, 2016, CSH PERSPECT MED, V6, DOI 10.1101/cshperspect.a026039

LANE DP, 1992, NATURE, V358, P15, DOI 10.1038/358015a0

Li LY, 2005, WORLD J GASTROENTERO, V11, P2998, DOI 10.3748/wjg.v11.i19.2998

Liu QG, 2018, PANCREATOLOGY, V18, P678, DOI 10.1016/j.pan.2018.07.003

Liu RH, 2019, CANCER MED-US, V8, P6095, DOI 10.1002/cam4.2507

Livak KJ, 2001, METHODS, V25, P402, DOI 10.1006/meth.2001.1262

Lowe AW, 2007, PLOS ONE, V2, DOI 10.1371/journal.pone.0000323

Ma SR, 2017, AM J TRANSL RES, V9, P507

Ma SR, 2015, ONCOTARGET, V6, P8807, DOI 10.18632/oncotarget.3556

Ondrouskova E, 2017, EXP MOL PATHOL, V102, P280, DOI 10.1016/j.yexmp.2017.02.016

Park SW, 2009, P NATL ACAD SCI USA, V106, P6950, DOI 10.1073/pnas.0808722106

Pizzi M, 2012, APPL IMMUNOHISTO M M, V20, P31, DOI 10.1097/PAI.0b013e3182233f9f

Pohler E, 2004, MOL CELL PROTEOMICS, V3, P534, DOI 10.1074/mcp.M300089-MCP200

Rustgi AK, 2014, NEW ENGL J MED, V371, P2499, DOI 10.1056/NEJMra1314530

Saeki H, 2011, CLIN CANCER RES, V17, P1731, DOI 10.1158/1078-0432.CCR-10-1996

Sicari D, 2021, EMBO REP, V22, DOI 10.15252/embr.202051412

Siliciano JD, 1997, GENE DEV, V11, P3471, DOI 10.1101/gad.11.24.3471

Soussi T, 2005, BIOCHEM BIOPH RES CO, V331, P834, DOI 10.1016/j.bbrc.2005.03.190

Sun BL, 2018, INT J CLIN EXP PATHO, V11, P822

Sweeny L, 2012, EXP CELL RES, V318, P1788, DOI 10.1016/j.yexcr.2012.04.022

Tang ZF, 2017, NUCLEIC ACIDS RES, V45, pW98, DOI 10.1093/nar/gkx247

Thompson DA, 1998, BIOCHEM BIOPH RES CO, V251, P111, DOI 10.1006/bbrc.1998.9440

Tian SB, 2017, SCI REP-UK, V7, DOI 10.1038/s41598-017-15757-z

Tsuji T, 2015, CANCER RES, V75, P356, DOI 10.1158/0008-5472.CAN-14-1693

WAGATA T, 1993, CANCER RES, V53, P846

Wang Z, 2008, CANCER RES, V68, P492, DOI 10.1158/0008-5472.CAN-07-2930

Zhang Y, 2007, PROSTATE CANCER P D, V10, P293, DOI 10.1038/sj.pcan.4500960

NR 44

TC 0

Z9 0

U1 1

U2 2

PU SPANDIDOS PUBL LTD

PI ATHENS

PA POB 18179, ATHENS, 116 10, GREECE

SN 1021-335X

EI 1791-2431

J9 ONCOL REP

JI Oncol. Rep.

PD DEC

PY 2021

VL 46

IS 6

AR 260

DI 10.3892/or.2021.8211

PG 11

WC Oncology

WE Science Citation Index Expanded (SCI-EXPANDED)

SC Oncology

GA WQ8SZ

UT WOS:000714081800001

PM 34713298

DA 2022-05-19

ER

PT J

AU Su, M

Tang, JM

Zhang, BH

Yang, DS

Wu, ZN

Wu, J

Zhou, Y

Liao, QJ

Wang, H

Wang, WX

Xiao, YH

AF Su, Min

Tang, Jinming

Zhang, Baihua

Yang, Desong

Wu, Zhining

Wu, Jie

Zhou, Yong

Liao, Qianjin

Wang, Hui

Wang, Wenxiang

Xiao, Yuhang

TI LncRNA GACAT3 promotes esophageal squamous cell carcinoma progression

through regulation of miR-149/FOXM1

SO CANCER CELL INTERNATIONAL

LA English

DT Article

DE Esophageal squamous cell carcinoma; Long noncoding RNA; GACAT3; miR-149;

FOXM1

ID LONG NONCODING RNAS; CANCER; FOXM1; EXPRESSION; MIGRATION; INVASION

AB Background The long noncoding RNA gastric cancer associated transcript 3 (GACAT3) has been demonstrated to be implicated in the carcinogenesis and progression of many malignancies. However, GACAT3's levels and role in esophageal squamous cell carcinoma (ESCC) has not been elucidated. Methods GACAT3 amounts were investigated in ESCC tissues and cell lines by qPCR. Its biological functions were examined by CCK-8 assay, colony formation assay, flow cytometry, wound healing assay, transwell assay, and xenograft model establishment. The relationship between GACAT3 and miR-149 was assessed by dual-luciferase reporter assay. Results GACAT3 amounts were elevated in ESCC tissue and cell specimens. Functional studies showed that GACAT3 silencing reduced the proliferation, migration and invasion of cultured ESCC cells, and decreased tumor growth in mice. Furthermore, GACAT could directly interact with miR-149. In addition, colony formation and invasion assays verified that GACAT3 promotes ESCC tumor progression through miR-149. Moreover, GACAT3 acted as a competing endogenous RNA (ceRNA) to modulate FOXM1 expression. Conclusions These findings indicate that GACAT3 functions as an oncogene by acting as a ceRNA for miR-149 to modulate FOXM1 expression in ESCC, suggesting that GACAT3 might constitute a therapeutic target in ESCC.

C1 [Su, Min; Tang, Jinming; Zhang, Baihua; Yang, Desong; Wu, Zhining; Wu, Jie; Zhou, Yong; Wang, Wenxiang; Xiao, Yuhang] Cent South Univ, Hunan Canc Hosp, Hunan Clin Med Res Ctr Accurate Diag & Treatment, Changsha 410013, Hunan, Peoples R China.

[Su, Min; Tang, Jinming; Zhang, Baihua; Yang, Desong; Wu, Zhining; Wu, Jie; Zhou, Yong; Liao, Qianjin; Wang, Hui; Wang, Wenxiang; Xiao, Yuhang] Cent South Univ, Affiliated Canc Hosp, Xiangya Sch Med, Changsha 410013, Hunan, Peoples R China.

[Su, Min; Tang, Jinming; Zhang, Baihua; Yang, Desong; Wu, Zhining; Wu, Jie; Zhou, Yong; Wang, Wenxiang] Cent South Univ, Hunan Canc Hosp, Thorac Surg Dept 2, Changsha 410013, Hunan, Peoples R China.

[Su, Min; Liao, Qianjin] Cent South Univ, Hunan Canc Hosp, Hunan Key Lab Canc Metab, Changsha 410013, Hunan, Peoples R China.

[Su, Min; Wang, Hui] Cent South Univ, Hunan Canc Hosp, Hunan Key Lab Translat Radiat Oncol, Changsha 410013, Hunan, Peoples R China.

[Xiao, Yuhang] Cent South Univ, Xiangya Hosp, Xiangya Sch Med, Dept Pharm, Changsha 410001, Hunan, Peoples R China.

RP Wang, WX; Xiao, YH (corresponding author), Cent South Univ, Hunan Canc Hosp, Hunan Clin Med Res Ctr Accurate Diag & Treatment, Changsha 410013, Hunan, Peoples R China.; Wang, WX; Xiao, YH (corresponding author), Cent South Univ, Affiliated Canc Hosp, Xiangya Sch Med, Changsha 410013, Hunan, Peoples R China.; Wang, WX (corresponding author), Cent South Univ, Hunan Canc Hosp, Thorac Surg Dept 2, Changsha 410013, Hunan, Peoples R China.; Xiao, YH (corresponding author), Cent South Univ, Xiangya Hosp, Xiangya Sch Med, Dept Pharm, Changsha 410001, Hunan, Peoples R China.

EM wangwenxiang@hnca.org.cn; xiaoyuhang1986@163.com

FU Natural Science Foundation of Hunan Province [2019JJ50968, 2020JJ5337];

Hunan Cancer Hospital Climb Plan [ZX2020005]

FX This study was supported by Natural Science Foundation of Hunan Province

(Grant No. 2019JJ50968, 2020JJ5337); Hunan Cancer Hospital Climb Plan

(ZX2020005).

CR Batista PJ, 2013, CELL, V152, P1298, DOI 10.1016/j.cell.2013.02.012

Bray F, 2018, CA-CANCER J CLIN, V68, P394, DOI 10.3322/caac.21492

Chan JJ, 2018, INT J MOL SCI, V19, DOI 10.3390/ijms19051310

Chen X, 2013, MOL CELL BIOL, V33, P227, DOI 10.1128/MCB.00881-12

Dey P, 2020, CANCERS, V12, DOI 10.3390/cancers12092677

Fan CM, 2017, MOL CANCER, V16, DOI 10.1186/s12943-017-0699-3

Hammond SM, 2015, ADV DRUG DELIVER REV, V87, P3, DOI 10.1016/j.addr.2015.05.001

Hangauer MJ, 2013, PLOS GENET, V9, DOI 10.1371/journal.pgen.1003569

He YJ, 2018, J CANCER, V9, P375, DOI 10.7150/jca.21044

Iyer MK, 2015, NAT GENET, V47, P199, DOI 10.1038/ng.3192

Ke Y, 2013, BIOCHEM RES INT, V2013, DOI 10.1155/2013/506731

Laoukili J, 2005, NAT CELL BIOL, V7, P126, DOI 10.1038/ncb1217

Li DZ, 2019, ONCOTARGETS THER, V12, P4065, DOI 10.2147/OTT.S201310

Liao ZX, 2007, J THORAC ONCOL, V2, P553, DOI 10.1097/01.JTO.0000275339.62831.5e

Ma JL, 2019, SCI REP-UK, V9, DOI 10.1038/s41598-019-45493-5

Ma LN, 2013, RNA BIOL, V10, P925, DOI 10.4161/rna.24604

Mao FX, 2019, CANCER CELL INT, V19, DOI 10.1186/s12935-019-1082-9

Ohashi S, 2015, GASTROENTEROLOGY, V149, P1700, DOI 10.1053/j.gastro.2015.08.054

Pan BL, 2019, J CELL PHYSIOL, V234, P8825, DOI 10.1002/jcp.27542

Pennathur A, 2013, LANCET, V381, P400, DOI 10.1016/S0140-6736(12)60643-6

Song HJ, 2013, J TRANSL MED, V11, DOI 10.1186/1479-5876-11-225

Song L, 2018, ONCOL LETT, V15, P5910, DOI 10.3892/ol.2018.8035

Su M, 2018, J HEMATOL ONCOL, V11, DOI 10.1186/s13045-018-0663-8

Xiao ZH, 2018, THORAC CANCER, V9, P997, DOI 10.1111/1759-7714.12776

Xu CJ, 2014, TUMOR BIOL, V35, P9701, DOI 10.1007/s13277-014-2274-5

Xu ZQ, 2020, IUBMB LIFE, V72, P426, DOI 10.1002/iub.2202

Zhang ZF, 2021, FRONT MOL BIOSCI, V7, DOI 10.3389/fmolb.2020.627774

Zhi YR, 2018, CELL PROLIFERAT, V51, DOI 10.1111/cpr.12465

Zhong H, 2018, CANCER BIOMARK, V22, P787, DOI 10.3233/CBM-181354

Zhou WW, 2018, ONCOTARGETS THER, V11, P1543, DOI 10.2147/OTT.S144103

NR 30

TC 0

Z9 0

U1 0

U2 2

PU BMC

PI LONDON

PA CAMPUS, 4 CRINAN ST, LONDON N1 9XW, ENGLAND

EI 1475-2867

J9 CANCER CELL INT

JI Cancer Cell Int.

PD SEP 8

PY 2021

VL 21

IS 1

AR 478

DI 10.1186/s12935-021-02192-4

PG 9

WC Oncology

WE Science Citation Index Expanded (SCI-EXPANDED)

SC Oncology

GA UN2II

UT WOS:000693843100003

PM 34496842

OA Green Published, gold, Green Submitted

DA 2022-05-19

ER

PT J

AU Wang, J

Yu, PY

Yu, JP

Luo, JD

Sun, ZQ

Sun, F

Kong, Z

Wang, JL

AF Wang, Jian

Yu, Peng-Yi

Yu, Jing-Ping

Luo, Ju-Dong

Sun, Zhi-Qiang

Sun, Fei

Kong, Ze

Wang, Jian-Lin

TI KIF22 promotes progress of esophageal squamous cell carcinoma cells and

is negatively regulated by miR-122

SO AMERICAN JOURNAL OF TRANSLATIONAL RESEARCH

LA English

DT Article

DE Esophageal squamous cell carcinoma; KIF22; miR-122; proliferation;

invasion

ID DNA-BINDING PROTEIN; KINESIN SUPERFAMILY; TUMOR-GROWTH; CANCER;

PROLIFERATION; SUPPRESSES; KID; EXPRESSION

AB Esophageal squamous cell carcinoma (ESCC) increases at fast rate of all cancer types in China, which urges the investigations of its potential mechanism. In this research, a highly expressed kinesin superfamily protein 22 (KIF22) was founded both in ESCC tissues and cancer cell lines. The following experiments pointed out that down-regulation of KIF22 remarkably restrained the malignant progression of ESCC cells. Besides, KIF22 knockdown promoted ESCC cells apoptosis and arrested cells in G0/G1 phase, while KIF22 also regulated the expression of cell cycle-and EMT-related proteins. Previous research revealed that the aberrant expressions of microRNAs (miRNAs) are related to tumors development. Based on the predict result, KIF22 was considered as the target of miR-122, which was demonstrated by luciferase reporter assay. miR-122 inhibitor could significantly reverse the function of KIF22 knockdown, including cell proliferation, migration and invasion. Furthermore, down expressed miR-122 altered the function of KIF22 knockdown on cell cycle-and EMT-related proteins. In a word, this work illustrated the regulatory function of KIF22/miR-122 axis in ESSC and provided potential targets for potential targets for ESSC treatment.

C1 [Yu, Jing-Ping; Luo, Ju-Dong; Sun, Zhi-Qiang; Sun, Fei; Kong, Ze; Wang, Jian-Lin] Nanjing Med Univ, Affiliated Changzhou Peoples Hosp 2, Dept Radiotherapy, 29 Xinglong Lane, Changzhou 213003, Jiangsu, Peoples R China.

[Wang, Jian] Jiangyin Peoples Hosp, Dept Radiotherapy, Jiangyin 214400, Jiangsu, Peoples R China.

[Yu, Peng-Yi] Soochow Univ, Affiliated Hosp 3, Dept Cardiothorac Surg, Changzhou 213003, Jiangsu, Peoples R China.

RP Wang, JL (corresponding author), Nanjing Med Univ, Affiliated Changzhou Peoples Hosp 2, Dept Radiotherapy, 29 Xinglong Lane, Changzhou 213003, Jiangsu, Peoples R China.

EM wangjianlin@njmu.edu.cn

FU National Natural Science Foundation of China [11705095, 82073339]; Young

Talent Development Plan of Changzhou Health Commission [CZQM2020074];

Changzhou Sci Tech Program [CJ20200053]; 333 High-level personnel

training Project [BRA2019025]; Key Research & Development Program of

Jiangsu Province [BE2020637]; Wuxi double hundred young and middle-aged

medical and health top-notch talent project [202014]

FX We thanked the support from National Natural Science Foundation of China

(11705095, 82073339); Young Talent Development Plan of Changzhou Health

Commission (CZQM2020074); Changzhou Sci & Tech Program (CJ20200053); 333

High-level personnel training Project (BRA2019025); The Key Research &

Development Program of Jiangsu Province (BE2020637); Wuxi double hundred

young and middle-aged medical and health top-notch talent project (No.

202014).

CR Bandiera S, 2015, J HEPATOL, V62, P448, DOI 10.1016/j.jhep.2014.10.004

Basu S, 2014, NUCLEIC ACIDS RES, V42, P7170, DOI 10.1093/nar/gku346

Brabletz T, 2018, NAT REV CANCER, V18, P128, DOI 10.1038/nrc.2017.118

Conteduca V, 2012, INT J ONCOL, V41, P414, DOI 10.3892/ijo.2012.1481

Ginzinger DG, 2002, EXP HEMATOL, V30, P503, DOI 10.1016/S0301-472X(02)00806-8

Hirokawa N, 2008, PHYSIOL REV, V88, P1089, DOI 10.1152/physrev.00023.2007

Hirokawa N, 2009, NAT REV MOL CELL BIO, V10, P682, DOI 10.1038/nrm2774

Koshy M, 2004, ONCOLOGIST, V9, P147, DOI 10.1634/theoncologist.9-2-147

Li B, 2020, CANCER BIOTHER RADIO, V35, P50, DOI 10.1089/cbr.2019.3045

Miki H, 2005, TRENDS CELL BIOL, V15, P467, DOI 10.1016/j.tcb.2005.07.006

Ohsugi M, 2003, EMBO J, V22, P2091, DOI 10.1093/emboj/cdg208

Park SM, 2016, EXP NEUROBIOL, V25, P33, DOI 10.5607/en.2016.25.1.33

Pike R, 2018, SCI SIGNAL, V11, DOI 10.1126/scisignal.aaq1060

Qin HF, 2015, ONCOTARGETS THER, V8, DOI 10.2147/OTT.S91696

Qiu YZ, 2016, BIOCHEM BIOPH RES CO, V474, P560, DOI 10.1016/j.bbrc.2016.04.112

Reddy KB, 2015, CANCER CELL INT, V15, DOI 10.1186/s12935-015-0185-1

Shukla Girish C, 2011, Mol Cell Pharmacol, V3, P83

Tokai N, 1996, EMBO J, V15, P457, DOI 10.1002/j.1460-2075.1996.tb00378.x

Venkatadri R, 2016, CELL DEATH DIS, V7, DOI 10.1038/cddis.2016.6

Wang BY, 2012, PLOS ONE, V7, DOI 10.1371/journal.pone.0047053

Wang CZ, 2017, BIOTECHNOL LETT, V39, P485, DOI 10.1007/s10529-016-2273-2

Wang MC, 2019, MOL MED REP, V19, P1942, DOI 10.3892/mmr.2018.9786

Yu Y, 2014, CARCINOGENESIS, V35, P1416, DOI 10.1093/carcin/bgu065

Yu Y, 2010, CANCER-AM CANCER SOC, V116, P5150, DOI 10.1002/cncr.25461

Yu ZY, 2020, CHINESE MED J-PEKING, V133, P919, DOI 10.1097/CM9.0000000000000742

Zhang F, 2014, CANCER LETT, V342, P121, DOI 10.1016/j.canlet.2013.08.037

Zhang HS, 2016, ARCH BIOCHEM BIOPHYS, V598, P50, DOI 10.1016/j.abb.2016.03.031

Zhang JB, 2019, REPROD BIOL ENDOCRIN, V17, DOI 10.1186/s12958-018-0450-y

Zhang K, 2018, ONCOTARGET, V9, P1028, DOI 10.18632/oncotarget.23178

Zhang YW, 2013, WORLD J GASTROENTERO, V19, P5598, DOI 10.3748/wjg.v19.i34.5598

Zhang Z, 2018, MED SCI MONITOR, V24, P8190, DOI 10.12659/MSM.912643

NR 31

TC 0

Z9 0

U1 1

U2 1

PU E-CENTURY PUBLISHING CORP

PI MADISON

PA 40 WHITE OAKS LN, MADISON, WI 53711 USA

SN 1943-8141

J9 AM J TRANSL RES

JI Am. J. Transl. Res.

PY 2021

VL 13

IS 5

BP 4152

EP 4166

PG 15

WC Oncology; Medicine, Research & Experimental

WE Science Citation Index Expanded (SCI-EXPANDED)

SC Oncology; Research & Experimental Medicine

GA SM3BI

UT WOS:000657484100001

PM 34150005

DA 2022-05-19

ER

PT J

AU Li, CY

Lee, SC

Lai, WL

Chang, KF

Huang, XF

Hung, PY

Lee, CP

Hsieh, MC

Tsai, NM

AF Li, Chia-Yu

Lee, Shan-Chih

Lai, Wen-Lin

Chang, Kai-Fu

Huang, Xiao-Fan

Hung, Peng-Yun

Lee, Chi-Pin

Hsieh, Ming-Chang

Tsai, Nu-Man

TI Cell cycle arrest and apoptosis induction by Juniperus communis extract

in esophageal squamous cell carcinoma through activation of p53-induced

apoptosis pathway

SO FOOD SCIENCE & NUTRITION

LA English

DT Article

DE apoptosis; cell cycle; esophageal squamous cell carcinoma; Juniperus

communis; synergistic effect

ID NATURAL-PRODUCTS; ANTIOXIDANT ACTIVITIES; CHEMICAL-COMPOSITION; CANCER

STATISTICS; LEAVES EXTRACTS; ESSENTIAL OIL; MECHANISMS; ADENOCARCINOMA;

THIOACETAMIDE; CHEMOTHERAPY

AB Esophageal squamous cell carcinoma (ESCC) is one of the most common cancers. It has a high mortality rate and requires novel effective drugs and therapeutic approaches. Juniperus communis (JCo), used to flavor gin and food, has been documented to have anti-tumor activity. The aim of this study was to investigate the antitumor activity of JCo extract against ESCC and its possible mechanisms. JCo extract suppressed cell growth in ESCC and showed higher selection for ESCC cells than normal cells compared to the clinical drug 5-fluorouracil (5-FU). JCo extract induced cell cycle arrest at the G(0)/G(1) phase by regulating the expression of p53/p21 and CDKs/cyclins, triggering cell apoptosis by activating both the extrinsic (Fas/FasL/Caspase 8) and intrinsic (Bcl-2/Bax/Caspase 9) apoptosis pathways. Moreover, a combination treatment of JCo and 5-FU synergistically inhibited proliferation of ESCC cells. These results suggest that JCo extract is a potential natural therapeutic agent for esophageal cancer, as it could induce cell cycle arrest and apoptosis in ESCC cells.

C1 [Li, Chia-Yu] Nanhua Univ, Dept Life & Death Studies, Chiayi, Taiwan.

[Lee, Shan-Chih] Chung Shan Med Univ, Dept Med Imaging & Radiol Sci, Taichung, Taiwan.

[Lee, Shan-Chih] Chung Shan Med Univ Hosp, Dept Med Imaging, Taichung, Taiwan.

[Lai, Wen-Lin; Chang, Kai-Fu; Huang, Xiao-Fan; Hung, Peng-Yun; Hsieh, Ming-Chang; Tsai, Nu-Man] Chung Shan Med Univ, Dept Med Lab & Biotechnol, 110 Sec 1,Jianguo N Rd, Taichung 40201, Taiwan.

[Lai, Wen-Lin; Hsieh, Ming-Chang; Tsai, Nu-Man] Chung Shan Med Univ Hosp, Clin Lab, Taichung, Taiwan.

[Chang, Kai-Fu; Huang, Xiao-Fan] Chung Shan Med Univ, Inst Med, Taichung, Taiwan.

[Lee, Chi-Pin] Distmanson Med Fdn Chia Yi Christian Hosp, Dept Internal Med, Div Cardiol, Chiayi, Taiwan.

RP Tsai, NM (corresponding author), Chung Shan Med Univ, Dept Med Lab & Biotechnol, 110 Sec 1,Jianguo N Rd, Taichung 40201, Taiwan.

EM numan@csmu.edu.tw

OI Li, Chia-Yu/0000-0001-7769-1290; Tsai, Nu-Man/0000-0002-2629-1833

FU Chung Shan Medical University Hospital Foundation, Taiwan, Republic of

China [CSH-2014-A-023, CSH-2014-C-031]

FX The present study was supported by grants from the Chung Shan Medical

University Hospital Foundation, Taiwan, Republic of China (No.

CSH-2014-A-023 and CSH-2014-C-031).

CR Al-Attar AM, 2017, SAUDI J BIOL SCI, V24, P15, DOI 10.1016/j.sjbs.2015.08.013

Al-Attar AM, 2016, SAUDI J BIOL SCI, V23, P363, DOI 10.1016/j.sjbs.2015.08.011

Bais Souravh, 2014, Int Sch Res Notices, V2014, P634723, DOI 10.1155/2014/634723

Bayazit V, 2004, SAUDI MED J, V25, P156

Cabral C, 2012, PHYTOTHER RES, V26, P1352, DOI 10.1002/ptr.3730

Carpenter CD, 2012, J ETHNOPHARMACOL, V143, P695, DOI 10.1016/j.jep.2012.07.035

Chen Q, 2018, SIGNAL TRANSDUCT TAR, V3, DOI 10.1038/s41392-018-0018-5

Chen WQ, 2016, CA-CANCER J CLIN, V66, P115, DOI 10.3322/caac.21338

Coqueret O, 2003, TRENDS CELL BIOL, V13, P65, DOI 10.1016/S0962-8924(02)00043-0

Ferte C, 2011, J CHEMOTHERAPY, V23, P358, DOI 10.1179/joc.2011.23.6.358

Flatt PM, 2000, DRUG METAB REV, V32, P283, DOI 10.1081/DMR-100102335

Fulda S, 2010, PLANTA MED, V76, P1075, DOI 10.1055/s-0030-1249961

Gao HW, 2019, AM J CHINESE MED, V47, P1171, DOI [10.1142/S0192415X19500605, 10.1142/s0192415x19500605]

Hajdari A, 2015, CHEM BIODIVERS, V12, P1706, DOI 10.1002/cbdv.201400439

Khare P. K., 2007, INDIAN MED PLANTS IL

Lantto TA, 2016, INT J MOL SCI, V17, DOI 10.3390/ijms17071113

Leporatti ML, 2003, J ETHNOPHARMACOL, V87, P123, DOI 10.1016/S0378-8741(03)00047-3

Liang H, 2017, CANCER BIOL MED, V14, P33, DOI 10.20892/j.issn.2095-3941.2016.0093

Lin XJ, 2015, INT J MOL SCI, V16, P10888, DOI 10.3390/ijms160510888

Longley DB, 2003, NAT REV CANCER, V3, P330, DOI 10.1038/nrc1074

Mann J, 2002, NAT REV CANCER, V2, P143, DOI 10.1038/nrc723

Mileo AM, 2016, OXID MED CELL LONGEV, V2016, DOI 10.1155/2016/6475624

Moein MR, 2010, PHARMACOGN RES, V2, P128, DOI 10.4103/0974-8490.65505

MORGAN DO, 1995, NATURE, V374, P131, DOI 10.1038/374131a0

Nakanishi T, 2004, PHYTOCHEMISTRY, V65, P207, DOI 10.1016/j.phytochem.2003.10.025

Orhan N, 2011, FOOD CHEM TOXICOL, V49, P2305, DOI 10.1016/j.fct.2011.06.031

Padumadasa C, 2016, BMC COMPLEM ALTERN M, V16, DOI 10.1186/s12906-016-1335-2

Pan L, 2010, PHYTOCHEM LETT, V3, P1, DOI 10.1016/j.phytol.2009.11.005

Pennathur A, 2013, LANCET, V381, P400, DOI 10.1016/S0140-6736(12)60643-6

Pfeffer CM, 2018, INT J MOL SCI, V19, DOI 10.3390/ijms19020448

Pollio A, 2016, MOLECULES, V21, DOI 10.3390/molecules21040395

Raasmaja A, 2019, INT J MOL SCI, V20, DOI 10.3390/ijms20092054

Rong Q, 2016, BMC COMPLEM ALTERN M, V16, DOI 10.1186/s12906-016-1310-y

Siegel RL, 2019, CA-CANCER J CLIN, V69, P7, DOI 10.3322/caac.21551

Soobrattee MA, 2005, MUTAT RES-FUND MOL M, V579, P200, DOI 10.1016/j.mrfmmm.2005.03.023

Soukand R, 2015, J ETHNOPHARMACOL, V170, P284, DOI 10.1016/j.jep.2015.05.018

Taya Y, 1997, TRENDS BIOCHEM SCI, V22, P14, DOI 10.1016/S0968-0004(96)10070-0

Wainberg ZA, 2011, BRIT J CANCER, V105, P760, DOI 10.1038/bjc.2011.280

Wang Y, 2018, CURR DRUG METAB, V19, P739, DOI 10.2174/1389200219666180511154722

Wu N, 2013, WIEN KLIN WOCHENSCHR, V125, P26, DOI 10.1007/s00508-012-0310-2

Wu N, 2012, CHINESE MED J-PEKING, V125, P4445, DOI 10.3760/cma.j.issn.0366-6999.2012.24.022

Yaman T, 2021, NUTR CANCER, V73, P133, DOI 10.1080/01635581.2019.1673450

NR 42

TC 4

Z9 4

U1 2

U2 7

PU WILEY

PI HOBOKEN

PA 111 RIVER ST, HOBOKEN 07030-5774, NJ USA

SN 2048-7177

J9 FOOD SCI NUTR

JI Food Sci. Nutr.

PD FEB

PY 2021

VL 9

IS 2

BP 1088

EP 1098

DI 10.1002/fsn3.2084

EA DEC 2020

PG 11

WC Food Science & Technology

WE Science Citation Index Expanded (SCI-EXPANDED)

SC Food Science & Technology

GA QD2RF

UT WOS:000603409500001

PM 33598192

OA Green Published, gold

DA 2022-05-19

ER

PT J

AU Zeng, R

Liu, Y

Jiang, ZJ

Huang, JP

Wang, Y

Li, XF

Xiong, WB

Wu, XC

Zhang, JR

Wang, QE

Zheng, YF

AF Zeng, Rong

Liu, Yi

Jiang, Zhao-Jing

Huang, Jun-Peng

Wang, Yu

Li, Xu-Feng

Xiong, Wei-Bin

Wu, Xiao-Cong

Zhang, Ji-Ren

Wang, Qi-En

Zheng, Yan-Fang

TI EPB41L3 is a potential tumor suppressor gene and prognostic indicator in

esophageal squamous cell carcinoma

SO INTERNATIONAL JOURNAL OF ONCOLOGY

LA English

DT Article

DE erythrocyte membrane protein band 4; 1 like 3; esophageal squamous cell

carcinoma; methylation; prognostic indicator; tumor suppressor

ID PREDICTS POOR-PROGNOSIS; LUNG-CANCER; PROMOTER HYPERMETHYLATION; DNA

METHYLATION; GROWTH; EXPRESSION; APOPTOSIS; PROGRESSION; ADENOCARCINOMA;

INHIBITION

AB Although there have been reports about the role of erythrocyte membrane protein band 4.1 like 3 (EPB41L3) in several types of cancer, primarily in non-small-cell lung carcinoma, the molecular function and modulatory mechanisms of EPB41L3 remain unclear. In specific, the functional and clinical significance of EPB41L3 in esophageal squamous cell carcinoma (ESCC) has not been explored to date. In the present study, reduced EPB41L3 expression was demonstrated in ESCC cell lines and tissues, which was due to its high methylation rate. Ectopic expression of EPB41L3 in ESCC cells inhibited cell proliferation in vivo and in vitro. In addition, EPB41L3 overexpression induced apoptosis and G2/M cell cycle arrest by activating Caspase-3/8/9 and Cyclin-dependent kinase 1/Cyclin B1 signaling, respectively. Notably, patients with higher EPB41L3 expression had markedly higher overall survival rates compared with patients with lower EPB41L3 expression. In summary, the present results suggest that EPB41L3 may be a tumor suppressor gene in ESCC development, representing a potential therapeutic target and a prognostic indicator for ESCC.

C1 [Zeng, Rong; Jiang, Zhao-Jing; Li, Xu-Feng; Xiong, Wei-Bin; Wu, Xiao-Cong; Zhang, Ji-Ren; Zheng, Yan-Fang] Southern Med Univ, Zhujiang Hosp, Ctr Oncol, 253 Ind Ave, Guangzhou 510280, Guangdong, Peoples R China.

[Liu, Yi] Guangzhou Med Univ, Dept Neurosurg, Affiliated Hosp 2, Guangzhou 510280, Guangdong, Peoples R China.

[Huang, Jun-Peng] Fujian Prov Hosp, Dept Med Oncol, Fuzhou 350001, Fujian, Peoples R China.

[Wang, Yu] Southern Med Univ, Zhujiang Hosp, Dept Pathol, Guangzhou 510280, Guangdong, Peoples R China.

[Wang, Qi-En] Ohio State Univ, Div Radiobiol, Dept Radiol, Wexner Med Ctr, Columbus, OH 43210 USA.

RP Zheng, YF (corresponding author), Southern Med Univ, Zhujiang Hosp, Ctr Oncol, 253 Ind Ave, Guangzhou 510280, Guangdong, Peoples R China.

EM 18665000236@163.com

OI Zheng, Yanfang/0000-0002-5591-6425

FU Natural Science Foundation of Guangdong Province, China

[2015A030313273]; NATIONAL CANCER INSTITUTE [R01CA211175, P30CA016058]

Funding Source: NIH RePORTER

FX This study was supported by the Natural Science Foundation of Guangdong

Province, China (grant no. 2015A030313273).

CR Al-Kaabi A, 2014, DIS MARKERS, V2014, DOI 10.1155/2014/260549

Baylin SB, 2005, COLD SH Q B, V70, P427, DOI 10.1101/sqb.2005.70.010

Bock C, 2016, NAT BIOTECHNOL, V34, P726, DOI 10.1038/nbt.3605

Castedo M, 2004, ONCOGENE, V23, P2825, DOI 10.1038/sj.onc.1207528

Deshpande A, 2005, ONCOGENE, V24, P2909, DOI 10.1038/sj.onc.1208618

Fareed KR, 2009, GUT, V58, P127, DOI 10.1136/gut.2008.155861

Ferlay J, 2015, INT J CANCER, V136, pE359, DOI 10.1002/ijc.29210

Gerber MA, 2006, CANCER RES, V66, P5295, DOI 10.1158/0008-5472.CAN-05-1628

Guo HW, 2015, THORAC CANCER, V6, P665, DOI 10.1111/1759-7714.12294

Guo MZ, 2006, CLIN CANCER RES, V12, P4515, DOI 10.1158/1078-0432.CCR-05-2858

Herman JG, 2003, NEW ENGL J MED, V349, P2042, DOI 10.1056/NEJMra023075

Homig-Holzel C, 2012, DIAGN MOL PATHOL, V21, P189, DOI 10.1097/PDM.0b013e3182595516

Jemal A, 2005, CA-CANCER J CLIN, V55, P10, DOI 10.3322/canjclin.55.1.10

Ji L, 1999, CANCER RES, V59, P3333

Jiang W, 2006, MOL CANCER, V5, DOI 10.1186/1476-4598-5-4

Kaz AM, 2014, CANCER LETT, V342, P193, DOI 10.1016/j.canlet.2012.02.036

Kikuchi S, 2005, CLIN CANCER RES, V11, P2954, DOI 10.1158/1078-0432.CCR-04-2206

Kong KL, 2010, CANCER RES, V70, P8832, DOI 10.1158/0008-5472.CAN-10-0779

Kuns R, 2005, ONCOGENE, V24, P6502, DOI 10.1038/sj.onc.1208813

Lee EJ, 2006, EUR J CANCER, V42, P972, DOI 10.1016/j.ejca.2006.01.021

Li XF, 2014, PLOS ONE, V9, DOI 10.1371/journal.pone.0103162

Liu Y, 2014, J NEUROINFLAMM, V11, DOI 10.1186/1742-2094-11-135

Livak KJ, 2001, METHODS, V25, P402, DOI 10.1006/meth.2001.1262

Lobrich M, 2007, NAT REV CANCER, V7, P861, DOI 10.1038/nrc2248

Ma XT, 2013, EPIGENOMICS-UK, V5, P301, DOI [10.2217/epi.13.26, 10.2217/EPI.13.26]

Mao XL, 2004, ONCOGENE, V23, P5632, DOI 10.1038/sj.onc.1207756

Nagata M, 2012, INT J CANCER, V130, P1329, DOI 10.1002/ijc.26160

Offner FA, 2000, PATHOLOGE, V21, P349, DOI 10.1007/s002920000414

Pennathur A, 2013, LANCET, V381, P400, DOI 10.1016/S0140-6736(12)60643-6

Reed K, 2010, ANAL BIOCHEM, V397, P96, DOI 10.1016/j.ab.2009.10.021

Rice TW, 2010, CANCER-AM CANCER SOC, V116, P3763, DOI 10.1002/cncr.25146

Roz L, 2002, P NATL ACAD SCI USA, V99, P3615, DOI 10.1073/pnas.062030799

Saeki T, 2002, ONCOGENE, V21, P4558, DOI 10.1038/sj.onc.1205553

Salam I, 2009, MOL CELL BIOCHEM, V332, P51, DOI 10.1007/s11010-009-0173-7

Taghavi N, 2010, BMC CANCER, V10, DOI 10.1186/1471-2407-10-138

Takahashi Y, 2012, BREAST CANCER-TOKYO, V19, P242, DOI 10.1007/s12282-011-0272-7

Wang Y, 2009, CANCER RES, V69, P5194, DOI 10.1158/0008-5472.CAN-08-3694

Wang Z, 2014, CELL MOL LIFE SCI, V71, P4815, DOI 10.1007/s00018-014-1707-z

Wong SY, 2007, P NATL ACAD SCI USA, V104, P12784, DOI 10.1073/pnas.0705499104

Xiang QF, 2015, J EXP CLIN CANC RES, V34, DOI 10.1186/s13046-015-0238-2

Yamada D, 2006, INT J CANCER, V118, P916, DOI 10.1002/ijc.21450

Yang JY, 2015, SCI REP-UK, V5, DOI 10.1038/srep10575

Zare M, 2009, BMC CANCER, V9, DOI 10.1186/1471-2407-9-24

Zeng R, 2016, CELL BIOCHEM FUNCT, V34, P133, DOI 10.1002/cbf.3170

Zhang YJ, 2012, TUMOR BIOL, V33, P1915, DOI 10.1007/s13277-012-0452-x

Zhu YH, 2013, CANCER RES, V73, P2298, DOI 10.1158/0008-5472.CAN-12-2663

NR 46

TC 14

Z9 14

U1 1

U2 8

PU SPANDIDOS PUBL LTD

PI ATHENS

PA POB 18179, ATHENS, 116 10, GREECE

SN 1019-6439

EI 1791-2423

J9 INT J ONCOL

JI Int. J. Oncol.

PD MAY

PY 2018

VL 52

IS 5

BP 1443

EP 1454

DI 10.3892/ijo.2018.4316

PG 12

WC Oncology

WE Science Citation Index Expanded (SCI-EXPANDED)

SC Oncology

GA GB5HV

UT WOS:000429096000007

PM 29568917

OA Green Published, Green Submitted, hybrid

DA 2022-05-19

ER

PT J

AU Piao, JM

Shin, MH

Kim, HN

Song, HR

Kweon, SS

Choi, JS

Shim, HJ

Hwang, JE

Bae, WK

Kim, SH

Choi, YD

Cui, LH

AF Piao, J. -M.

Shin, M. -H.

Kim, H. N.

Song, H. -R.

Kweon, S. -S.

Choi, J. -S.

Shim, H. -J.

Hwang, J. -E.

Bae, W. -K.

Kim, S. -H.

Choi, Y. -D.

Cui, L. -H.

TI Replication of results of genome-wide association studies on esophageal

squamous cell carcinoma susceptibility loci in a Korean population

SO DISEASES OF THE ESOPHAGUS

LA English

DT Article

DE C20orf54; esophageal squamous cell carcinoma; PLCE1; South Korea

ID CHINESE POPULATION; CANCER; PLCE1; VARIANTS; RISK; POLYMORPHISM;

SURVIVAL; 10Q23

AB Two recent genome-wide association studies have identified that the rs2274223 single-nucleotide polymorphism inphospholipase C epsilon 1 and the single-nucleotide polymorphism rs13042395 in C20orf54 are involved in esophageal squamous cell carcinoma (ESCC) in Chinese populations. We hypothesized that genetic polymorphisms of phospholipase C epsilon 1 and C20orf54 are also associated with ESCC in a Korean population. The rs2274223 and rs13042395 genotyping was performed using high-resolution melting analysis. The rs2274223 GG genotype was significantly associated with an increased risk of ESCC (odds ratio [OR] = 1.86, 95% confidence interval [CI] = 1.08-3.25) compared with the rs2274223 AA genotype. The rs13042395 G allele showed a significantly decreased risk of ESCC in the younger age group (OR = 0.71, 95% CI = 0.52-0.97) and no significant association in the older group (OR = 1.19, 95% CI = 0.87-1.62). We observed that the rs2274223 polymorphism was associated with an increased risk of ESCC in this Korean case-control study and that age may modify the association between the rs13042395 polymorphism and the risk of ESCC.

C1 [Piao, J. -M.; Cui, L. -H.] Qingdao Univ, Coll Med, Dept Publ Hlth, Qingdao 266071, Peoples R China.

[Shin, M. -H.; Song, H. -R.; Kweon, S. -S.; Choi, J. -S.] Chonnam Natl Univ, Sch Med, Dept Prevent Med, Kwangju, South Korea.

[Shim, H. -J.; Hwang, J. -E.; Bae, W. -K.] Chonnam Natl Univ, Sch Med, Dept Hematol Oncol, Kwangju, South Korea.

[Kim, S. -H.] Chonnam Natl Univ, Sch Med, Dept Lab Med, Kwangju, South Korea.

[Choi, Y. -D.] Chonnam Natl Univ, Sch Med, Dept Pathol, Kwangju, South Korea.

[Kim, H. N.] Chonnam Natl Univ, Hwasun Hosp, Genome Res Ctr Hematopoiet Dis, Hwasun, South Korea.

[Kweon, S. -S.] Chonnam Natl Univ, Hwasun Hosp, Jeonnam Reg Canc Ctr, Hwasun, South Korea.

RP Shin, MH (corresponding author), Chonnam Natl Univ, Sch Med, Dept Prevent Med, 5,Hak1Dong, Gwangju City 501746, South Korea.

EM mhshinx@paran.com

RI Kweon, Sun-Seog/AAZ-4732-2021

OI Kweon, Sun-Seog/0000-0003-2378-8550

FU National R&D Program for Cancer Control, Ministry for Health and

Welfare, Republic of Korea [0720570]

FX Support for this study was provided by the National R&D Program for

Cancer Control, Ministry for Health and Welfare, Republic of Korea

(0720570).

CR Abnet CC, 2010, NAT GENET, V42, P764, DOI 10.1038/ng.649

Bai YF, 2004, CANCER RES, V64, P8808, DOI 10.1158/0008-5472.CAN-04-3143

Brown KM, 2008, NAT GENET, V40, P838, DOI 10.1038/ng.163

Ferlay J, 2010, INT J CANCER, V127, P2893, DOI 10.1002/ijc.25516

Hu HC, 2012, ANN SURG ONCOL, V19, P2403, DOI 10.1245/s10434-011-2160-y

Jung KW, 2010, J KOREAN MED SCI, V25, P1113, DOI 10.3346/jkms.2010.25.8.1113

Kim HN, 2008, BRIT J HAEMATOL, V140, P287, DOI 10.1111/j.1365-2141.2007.06893.x

Lange EM, 2012, PROSTATE, V72, P147, DOI 10.1002/pros.21414

Li MZ, 2009, CARCINOGENESIS, V30, P1424, DOI 10.1093/carcin/bgp125

Luo DW, 2011, J GASTROENTEROL, V46, P1260, DOI 10.1007/s00535-011-0445-3

Ma HX, 2011, BMC CANCER, V11, DOI 10.1186/1471-2407-11-258

Okobia MN, 2011, PROSTATE, V71, P1054, DOI 10.1002/pros.21320

Shao Y, 2008, DIS ESOPHAGUS, V21, P139, DOI 10.1111/j.1442-2050.2007.00746.x

Sobin LH., 2009, TNM CLASSIFICATION M, V7

Sorensen M, 2004, INT J CANCER, V110, P219, DOI 10.1002/ijc.20075

Wang LD, 2010, NAT GENET, V42, P759, DOI 10.1038/ng.648

Wu C, 2011, NAT GENET, V43, P679, DOI 10.1038/ng.849

Zhang HZ, 2011, CARCINOGENESIS, V32, P848, DOI 10.1093/carcin/bgr051

NR 18

TC 7

Z9 8

U1 0

U2 5

PU WILEY-BLACKWELL

PI HOBOKEN

PA 111 RIVER ST, HOBOKEN 07030-5774, NJ USA

SN 1120-8694

EI 1442-2050

J9 DIS ESOPHAGUS

JI Dis. Esophagus

PD NOV-DEC

PY 2014

VL 27

IS 8

BP 798

EP 801

DI 10.1111/dote.12155

PG 4

WC Gastroenterology & Hepatology

WE Science Citation Index Expanded (SCI-EXPANDED)

SC Gastroenterology & Hepatology

GA AS4DU

UT WOS:000344224300016

PM 24152165

DA 2022-05-19

ER

PT J

AU Shibata, T

Matsuo, Y

Shamoto, T

Hirokawa, T

Tsuboi, K

Takahashi, H

Ishiguro, H

Kimura, M

Takeyama, H

Inagaki, H

AF Shibata, Takahiro

Matsuo, Yoichi

Shamoto, Tomoya

Hirokawa, Takahisa

Tsuboi, Ken

Takahashi, Hiroki

Ishiguro, Hideyuki

Kimura, Masahiro

Takeyama, Hiromitsu

Inagaki, Hiroshi

TI Girdin, a regulator of cell motility, is a potential prognostic marker

for esophageal squamous cell carcinoma

SO ONCOLOGY REPORTS

LA English

DT Article

DE Girdin; esophageal squamous cell carcinoma; cell motility; prognosis

ID TUMOR PROGRESSION; CANCER; EXPRESSION; SURVIVAL; REORGANIZATION;

ORGANIZATION; MIGRATION; PROTEIN

AB Girdin, an actin-binding Akt substrate, regulates actin reconstruction and Akt-dependent cell motility in fibroblasts and in a human breast cancer cell line. We examined whether Girdin is also involved in the motility of esophageal squamous cell carcinoma (ESCC) cells. Immunofluorescent staining and migration assays were performed, using KYSE cell lines, to examine whether Girdin is involved in the motility of ESCC cells. Upon EGF stimulation, Girdin colocalized with filamentous actin (F-actin) in the lamellipodia as determined by immunofluorescent staining. In migration assays, cell motility was significantly reduced in KYSE cell lines transfected with Girdin siRNA compared with the negative control. In addition, we examined the relationship between Girdin expression and clinical data, using specimens resected from ESCC patients. In immunohistochemical (IHC) analyses using specimens resected from ESCC patients, overall survival was significantly longer in cases showing lower Girdin expression compared to cases with higher Girdin expression. Collectively, Girdin appears to be involved in the motility of ESCC cells. The levels of Girdin expression correlated inversely with the survival of ESCC patients. Therefore, in ESCC, Girdin may be a prognostic marker and may serve as a therapeutic target as well.

C1 [Shibata, Takahiro; Matsuo, Yoichi; Shamoto, Tomoya; Hirokawa, Takahisa; Tsuboi, Ken; Takahashi, Hiroki; Ishiguro, Hideyuki; Kimura, Masahiro; Takeyama, Hiromitsu] Nagoya City Univ, Dept Surg Gastroenterol, Nagoya, Aichi 4678601, Japan.

[Inagaki, Hiroshi] Nagoya City Univ, Dept Anat Pathol & Mol Diagnost, Nagoya, Aichi 4678601, Japan.

RP Matsuo, Y (corresponding author), Nagoya City Univ, Dept Surg Gastroenterol, Mizuho Ku, Kawasumi 1,Mizuho Cho, Nagoya, Aichi 4678601, Japan.

EM matsuo@med.nagoya-cu.ac.jp

CR Altomare DA, 2012, CURR MED CHEM, V19, P3748

Ando N, 2000, ANN SURG, V232, P225, DOI 10.1097/00000658-200008000-00013

Boyce GA, 2003, TEXT GASTROENTEROLOG, P1180

Enomoto A, 2005, DEV CELL, V9, P389, DOI 10.1016/j.devcel.2005.08.001

Garcia-Marcos M, 2011, FASEB J, V25, P590, DOI 10.1096/fj.10-167304

Harris Albert K., 1999, P315

Hofstetter W, 2002, ANN SURG, V236, P376, DOI 10.1097/00000658-200209000-00014

Ide H, 2002, JAPANESE SOC ESOPHAG

Jiang P, 2008, CANCER RES, V68, P1310, DOI 10.1158/0008-5472.CAN-07-5111

Konishi S, 2002, CANCER-AM CANCER SOC, V95, P2473, DOI 10.1002/cncr.10987

Kuwano Hiroyuki, 2003, Ann Thorac Cardiovasc Surg, V9, P6

Lin YC, 2004, WORLD J GASTROENTERO, V10, P3235, DOI 10.3748/wjg.v10.i22.3235

Machesky LM, 1997, J CELL BIOL, V138, P913, DOI 10.1083/jcb.138.4.913

Ohta Y, 1999, P NATL ACAD SCI USA, V96, P2122, DOI 10.1073/pnas.96.5.2122

Olson MF, 2009, CLIN EXP METASTAS, V26, P273, DOI 10.1007/s10585-008-9174-2

Ozawa S, 2010, ESOPHAGUS-TOKYO, V7, P7, DOI 10.1007/s10388-010-0228-6

Qian Y, 2004, AM J PHYSIOL-CELL PH, V286, pC153, DOI 10.1152/ajpcell.00142.2003

Ren Y, 2005, CLIN CANCER RES, V11, P6190, DOI 10.1158/1078-0432.CCR-04-2553

RIVAS RJ, 1995, J NEUROSCI, V15, P981

Roy S, 2010, J BIOMECH ENG-T ASME, V132, DOI 10.1115/1.4001074

Shimada Y, 1997, ANN SURG, V226, P162, DOI 10.1097/00000658-199708000-00007

Takashima N, 2006, ONCOL REP, V15, P667

Terashita Y, 2004, ONCOL REP, V12, P827

Trivers KF, 2008, INT J CANCER, V123, P1422, DOI 10.1002/ijc.23691

Welch MD, 1999, TRENDS CELL BIOL, V9, P423, DOI 10.1016/S0962-8924(99)01651-7

Yamazaki D, 2005, CANCER SCI, V96, P379, DOI 10.1111/j.1349-7006.2005.00062.x

NR 26

TC 17

Z9 18

U1 0

U2 4

PU SPANDIDOS PUBL LTD

PI ATHENS

PA POB 18179, ATHENS, 116 10, GREECE

SN 1021-335X

J9 ONCOL REP

JI Oncol. Rep.

PD JUN

PY 2013

VL 29

IS 6

BP 2127

EP 2132

DI 10.3892/or.2013.2406

PG 6

WC Oncology

WE Science Citation Index Expanded (SCI-EXPANDED)

SC Oncology

GA 148GO

UT WOS:000319232800006

PM 23588413

OA Bronze

DA 2022-05-19

ER

PT J

AU Li, Y

Zhu, XZ

Yang, ML

Wang, YY

Li, JH

Fang, JR

Guo, WN

Ma, SS

Guan, FX

AF Li, Ya

Zhu, Xiangzhan

Yang, Minglei

Wang, Yingying

Li, Jianhui

Fang, Jiarui

Guo, Wenna

Ma, Shanshan

Guan, Fangxia

TI YAP/TEAD4-induced KIF4A contributes to the progression and worse

prognosis of esophageal squamous cell carcinoma

SO MOLECULAR CARCINOGENESIS

LA English

DT Article

DE esophageal squamous cell carcinoma; KIF4A; proliferation; TEAD4; YAP

ID POOR-PROGNOSIS; HEPATOCELLULAR-CARCINOMA; SIGNALING PATHWAY; HIPPO

PATHWAY; PROLIFERATION; BIOMARKER; ACTIVATION; RESISTANCE; THERAPY

AB Aberrant expression of kinesin family member 4A (KIF4A), which is associated with tumor progression, has been reported in several types of cancer. However, its expression and the underlying molecular mechanisms regulating the transcription of KIF4A in esophageal squamous cell carcinoma (ESCC) remain largely unclear. Here, we found that high KIF4A expression was positively correlated with tumor stage and poor prognosis in ESCC patients. KIF4A silencing significantly inhibited the growth and migration of ESCC cells, arrested cell cycle, and induced apoptosis. Interestingly, KIF4A expression was positively related to the expression of YAP in human ESCC tissues. YAP knockdown or disrupting YAP/TEAD4 interaction by verteporfin repressed KIF4A expression. Also, KIF4A knockdown significantly inhibited the cell growth induced by YAP overexpression. Mechanistically, YAP activated KIF4A transcriptional expression by TEAD4-mediated direct binding to KIF4A promoter. Finally, KIF4A knockdown and verteporfin treatment synergistically inhibited tumor growth in xenograft models. Together, these results indicated that KIF4A, a novel target gene of YAP/TEAD4, may be a progression and prognostic biomarker of ESCC. Targeting drugs for KIF4A combined with YAP inhibitor may be a novel therapeutic strategy for ESCC.

C1 [Li, Ya; Wang, Yingying; Fang, Jiarui; Guo, Wenna; Ma, Shanshan; Guan, Fangxia] Zhengzhou Univ, Sch Life Sci, 100 Sci Ave, Zhengzhou 450001, Henan, Peoples R China.

[Zhu, Xiangzhan] East China Normal Univ, Inst Biomed Sci, Sch Life Sci, Shanghai Key Lab Regulatory Biol, Shanghai, Peoples R China.

[Yang, Minglei] Second Mil Med Univ, Changzheng Hosp, Dept Orthoped Oncol, Shanghai, Peoples R China.

[Li, Jianhui] Henan Univ Sci & Technol, Xuchang Cent Hosp, Dept Pathol, Xuchang, Peoples R China.

RP Ma, SS; Guan, FX (corresponding author), Zhengzhou Univ, Sch Life Sci, 100 Sci Ave, Zhengzhou 450001, Henan, Peoples R China.

EM mashanshan84@163.com; guanfangxia@126.com

FU Key Special Project of Zhengzhou University Disciplinary Construction

[XKZDJC202001]; Discipline Innovation and Wisdom Introduction Plan of

Higher Education in Henan Province [CXJD2021002]; Central Plains

Thousand People Plan of Henan Province [204200510013]

FX Key Special Project of Zhengzhou University Disciplinary Construction,

Grant/Award Number: XKZDJC202001; Discipline Innovation and Wisdom

Introduction Plan of Higher Education in Henan Province, Grant/Award

Number: CXJD2021002; Central Plains Thousand People Plan of Henan

Province, Grant/Award Number: 204200510013

CR Abnet CC, 2018, GASTROENTEROLOGY, V154, P360, DOI 10.1053/j.gastro.2017.08.023

Bolgioni AF, 2016, CHROMOSOME RES, V24, P93, DOI 10.1007/s10577-015-9502-8

Cao Q, 2020, CLIN CANCER RES, V26, P1516, DOI 10.1158/1078-0432.CCR-19-0396

Chen JH, 2020, CLIN INVEST MED, V43, pE49, DOI 10.25011/cim.v43i3.34393

Chen M, 2020, ONCOTARGETS THER, V13, P9865, DOI 10.2147/OTT.S266649

Codipilly DC, 2018, GASTROINTEST ENDOSC, V88, P413, DOI 10.1016/j.gie.2018.04.2352

Cross RA, 2014, NAT REV MOL CELL BIO, V15, P257, DOI 10.1038/nrm3768

Arnal MJD, 2015, WORLD J GASTROENTERO, V21, P7933, DOI 10.3748/wjg.v21.i26.7933

Feng W, 2015, BIOMED PHARMACOTHER, V74, P257, DOI 10.1016/j.biopha.2015.08.017

Gao YB, 2014, NAT GENET, V46, P1097, DOI 10.1038/ng.3076

Harvey KF, 2013, NAT REV CANCER, V13, P246, DOI 10.1038/nrc3458

He LL, 2019, CANCER RES, V79, P4399, DOI 10.1158/0008-5472.CAN-19-0012

Heng BC, 2021, CELL MOL LIFE SCI, V78, P497, DOI 10.1007/s00018-020-03579-8

Hou GJ, 2017, SCI REP-UK, V7, DOI 10.1038/s41598-017-04176-9

Hou PF, 2018, CELL DEATH DIS, V9, DOI 10.1038/s41419-018-0550-9

Hu GH, 2019, J EXP CLIN CANC RES, V38, DOI 10.1186/s13046-019-1202-3

Huang YL, 2018, CELL DEATH DIS, V9, DOI 10.1038/s41419-017-0114-4

Jiang KX, 2020, BIOMED RES INT, V2020, DOI 10.1155/2020/6387519

Li XS, 2020, AM J TRANSL RES, V12, P1614

Liu K, 2020, CANCER CELL INT, V20, DOI 10.1186/s12935-020-01304-w

Liu Y, 2016, ONCOGENE, V35, P2789, DOI 10.1038/onc.2015.342

Ma YL, 2015, INT J CANCER, V137, P2275, DOI 10.1002/ijc.29073

Maehama T, 2021, CANCER SCI, V112, P51, DOI 10.1111/cas.14725

Malumbres M, 2014, GENOME BIOL, V15, DOI 10.1186/gb4184

Meng ZP, 2016, GENE DEV, V30, P1, DOI 10.1101/gad.274027.115

Moya IM, 2019, NAT REV MOL CELL BIO, V20, P211, DOI 10.1038/s41580-018-0086-y

Ohashi S, 2015, GASTROENTEROLOGY, V149, P1700, DOI 10.1053/j.gastro.2015.08.054

Pan LN, 2017, J ZHEJIANG UNIV-SC B, V18, P1046, DOI 10.1631/jzus.B1700129

Plouffe SW, 2015, TRENDS MOL MED, V21, P212, DOI 10.1016/j.molmed.2015.01.003

Qin HD, 2016, AM J HUM GENET, V98, P709, DOI 10.1016/j.ajhg.2016.02.021

Qu Y, 2019, EXP THER MED, V18, P2993, DOI 10.3892/etm.2019.7896

Rath O, 2012, NAT REV CANCER, V12, P527, DOI 10.1038/nrc3310

Sheng L, 2018, GENE, V678, P90, DOI 10.1016/j.gene.2018.08.005

Sun XZ, 2021, THORAC CANCER, V12, P512, DOI 10.1111/1759-7714.13787

Taniwaki M, 2007, CLIN CANCER RES, V13, P6624, DOI 10.1158/1078-0432.CCR-07-1328

Wang LH, 2019, ONCOGENE, V38, P2042, DOI 10.1038/s41388-018-0476-9

Wang XH, 2018, ONCOL LETT, V15, P8983, DOI 10.3892/ol.2018.8464

Watanabe M, 2020, SURG TODAY, V50, P12, DOI 10.1007/s00595-019-01878-7

Wu ZQ, 2020, CHEMOTHERAPY, V64, P187, DOI 10.1159/000505014

Xie BW, 2019, CANCER LETT, V443, P56, DOI 10.1016/j.canlet.2018.11.019

Xue D, 2018, ONCOTARGETS THER, V11, P4755, DOI 10.2147/OTT.S164730

Zeng HM, 2016, THORAC CANCER, V7, P232, DOI 10.1111/1759-7714.12322

Zheng YG, 2019, DEV CELL, V50, P264, DOI 10.1016/j.devcel.2019.06.003

Zhou XF, 2020, THERANOSTICS, V10, P9443, DOI 10.7150/thno.46078

Zhu XZ, 2021, ANTIOXID REDOX SIGN, V35, P75, DOI 10.1089/ars.2019.7911

NR 45

TC 0

Z9 0

U1 2

U2 8

PU WILEY

PI HOBOKEN

PA 111 RIVER ST, HOBOKEN 07030-5774, NJ USA

SN 0899-1987

EI 1098-2744

J9 MOL CARCINOGEN

JI Mol. Carcinog.

PD JUL

PY 2021

VL 60

IS 7

BP 440

EP 454

DI 10.1002/mc.23303

EA MAY 2021

PG 15

WC Biochemistry & Molecular Biology; Oncology

WE Science Citation Index Expanded (SCI-EXPANDED)

SC Biochemistry & Molecular Biology; Oncology

GA SO8ML

UT WOS:000651533400001

PM 34003522

DA 2022-05-19

ER

PT J

AU Jiang, L

Wang, WC

Li, GL

Sun, CL

Ren, ZQ

Sheng, HH

Gao, HJ

Wang, CF

Yu, H

AF Jiang, Lin

Wang, Wenchao

Li, Guoli

Sun, Canlin

Ren, Zhenqin

Sheng, Haihui

Gao, Hengjun

Wang, Chaofu

Yu, Hong

TI High TUG1 expression is associated with chemotherapy resistance and poor

prognosis in esophageal squamous cell carcinoma

SO CANCER CHEMOTHERAPY AND PHARMACOLOGY

LA English

DT Article

DE Long noncoding RNA; TUG1; Esophageal squamous cell carcinoma;

Chemotherapy; Drug resistance

ID LONG NONCODING RNA; UP-REGULATION; COLORECTAL-CANCER; LUNG-CANCER;

PROLIFERATION; MIGRATION; METASTASIS; APOPTOSIS; HOTAIR; LNCRNA

AB Long noncoding RNAs (lncRNAs) play critical roles in diverse biological processes such as tumorigenesis and metastasis. Taurine upregulated gene 1 (TUG1) is a cancer-related lncRNA that is associated with chromatin-modifying complexes and plays an important role in gene regulation. In this study, we determined the expression patterns of TUG1 in esophageal squamous cell carcinoma (ESCC) and evaluated its clinical significance.

The expression level of TUG1 was examined in 218 pairs of ESCC and adjacent non-cancerous tissues by using quantitative real-time polymerase chain reaction. The relationship between TUG1 expression and clinical features and prognosis was statistically analyzed.

The expression level of TUG1 was significantly upregulated in ESCC tissues compared with paired adjacent normal tissues. High TUG1 expression was significantly correlated with chemotherapy resistance. Survival analysis showed that patients with high TUG1 expression had poor prognosis, especially for cases with well and moderate differentiation, ulcerative type, smaller size, and chemotherapy-sensitive tumors.

Our findings suggest that elevated TUG1 expression is related to chemotherapy resistance and may help predict a poor prognostic outcome of ESCC. TUG1 may provide a potential therapeutic target for ESCC.

C1 [Jiang, Lin; Sun, Canlin; Ren, Zhenqin] Taizhou Peoples Hosp, Dept Anesthesiol, Taizhou, Jiangsu, Peoples R China.

[Wang, Wenchao; Li, Guoli] Yangzhou Univ, Sch Med, Dept Pathol, Yangzhou, Jiangsu, Peoples R China.

[Sheng, Haihui; Gao, Hengjun] Natl Engn Ctr Biochip Shanghai, Shanghai Engn Ctr Mol Med, Shanghai, Peoples R China.

[Wang, Chaofu] Shanghai Jiao Tong Univ, Sch Med, Ruijing Hosp, Dept Pathol, Shanghai 00032, Peoples R China.

[Yu, Hong] Taizhou Peoples Hosp, Dept Pathol, Taizhou 225300, Jiangsu, Peoples R China.

RP Wang, CF (corresponding author), Shanghai Jiao Tong Univ, Sch Med, Ruijing Hosp, Dept Pathol, Shanghai 00032, Peoples R China.; Yu, H (corresponding author), Taizhou Peoples Hosp, Dept Pathol, Taizhou 225300, Jiangsu, Peoples R China.

EM wangchaofu@126.com; yuhongmiaomiao@163.com

FU Taizhou Society Development Project, Jiangsu, China [TS201532]; 333

Project of Scientific Research Project Foundation, Jiangsu, China

[BRA2015224]; 12th five-year plan Key Project of Science and Technology,

China [2013ZX10002007]

FX This study was supported by Taizhou Society Development Project,

Jiangsu, China (Grant No. TS201532); the 333 Project of Scientific

Research Project Foundation, Jiangsu, China (Grant No. BRA2015224); and

the 12th five-year plan Key Project of Science and Technology, China

(Grant No. 2013ZX10002007).

CR Bhan A, 2014, CHEMMEDCHEM, V9, P1932, DOI 10.1002/cmdc.201300534

Chen WQ, 2016, CA-CANCER J CLIN, V66, P115, DOI 10.3322/caac.21338

Eisenhauer EA, 2009, EUR J CANCER, V45, P228, DOI 10.1016/j.ejca.2008.10.026

Gupta RA, 2010, NATURE, V464, P1071, DOI 10.1038/nature08975

Guttman M, 2009, NATURE, V458, P223, DOI 10.1038/nature07672

Han YH, 2013, J SURG ONCOL, V107, P555, DOI 10.1002/jso.23264

Huang MD, 2015, MOL CANCER, V14, DOI 10.1186/s12943-015-0431-0

Huarte M, 2010, CELL, V142, P409, DOI 10.1016/j.cell.2010.06.040

Koti M, 2013, BMC CANCER, V13, DOI 10.1186/1471-2407-13-549

Lalevee S, 2015, EPIGENOMICS-UK, V7, P877, DOI 10.2217/epi.15.55

Li J, 2016, EXP BIOL MED, V241, P644, DOI 10.1177/1535370215622708

Li X, 2013, BRIT J CANCER, V109, P2266, DOI 10.1038/bjc.2013.548

Liao WT, 2011, CLIN CANCER RES, V17, P3569, DOI 10.1158/1078-0432.CCR-10-2533

Ma B, 2016, TUMOR BIOL, V37, P4445, DOI 10.1007/s13277-015-4301-6

Matsushima K, 2011, J TRANSL MED, V9, DOI 10.1186/1479-5876-9-30

Qi P, 2013, MODERN PATHOL, V26, P155, DOI 10.1038/modpathol.2012.160

Raveh E, 2015, MOL CANCER, V14, DOI 10.1186/s12943-015-0458-2

Schmidt LH, 2011, J THORAC ONCOL, V6, P1984, DOI 10.1097/JTO.0b013e3182307eac

Siegel RL, 2015, CA-CANCER J CLIN, V65, P5, DOI [10.3322/caac.21254, 10.1136/bmj.g1502, 10.1001/jamaoto.2014.2530, 10.3322/caac.21208]

Sun JF, 2016, J TRANSL MED, V14, DOI 10.1186/s12967-016-0786-z

Tan JM, 2015, FEBS LETT, V589, P3175, DOI 10.1016/j.febslet.2015.08.020

Wang CM, 2014, DIGEST DIS SCI, V59, P591, DOI 10.1007/s10620-013-2956-7

Wilusz JE, 2009, GENE DEV, V23, P1494, DOI 10.1101/gad.1800909

Xu TP, 2015, ONCOGENE, V34, P5648, DOI 10.1038/onc.2015.18

Xu YT, 2015, TUMOR BIOL, V36, P1643, DOI 10.1007/s13277-014-2763-6

Yang GD, 2014, BBA-GENE REGUL MECH, V1839, P1097, DOI 10.1016/j.bbagrm.2014.08.012

Yang X, 2014, GUT, V63, P881, DOI 10.1136/gutjnl-2013-305266

Young TL, 2005, CURR BIOL, V15, P501, DOI 10.1016/j.cub.2005.02.027

Zhang EB, 2014, CELL DEATH DIS, V5, DOI 10.1038/cddis.2014.201

Zhang L, 2016, CANCER CHEMOTH PHARM, V77, P629, DOI 10.1007/s00280-016-2963-4

Zhang XL, 2015, J SURG ONCOL, V111, P834, DOI 10.1002/jso.23888

NR 31

TC 60

Z9 62

U1 1

U2 17

PU SPRINGER

PI NEW YORK

PA ONE NEW YORK PLAZA, SUITE 4600, NEW YORK, NY, UNITED STATES

SN 0344-5704

EI 1432-0843

J9 CANCER CHEMOTH PHARM

JI Cancer Chemother. Pharmacol.

PD AUG

PY 2016

VL 78

IS 2

BP 333

EP 339

DI 10.1007/s00280-016-3066-y

PG 7

WC Oncology; Pharmacology & Pharmacy

WE Science Citation Index Expanded (SCI-EXPANDED)

SC Oncology; Pharmacology & Pharmacy

GA DV1TG

UT WOS:000382703400014

PM 27329359

DA 2022-05-19

ER

PT J

AU Ma, GL

Zhang, FM

Dong, XG

Wang, XL

Ren, YG

AF Ma, Guoliang

Zhang, Fengmei

Dong, Xueguang

Wang, Xiaoli

Ren, Yuguo

TI Low expression of microRNA-202 is associated with the metastasis of

esophageal squamous cell carcinoma

SO EXPERIMENTAL AND THERAPEUTIC MEDICINE

LA English

DT Article

DE esophageal squamous cell carcinoma; microRNA-202; invasion; metastasis

ID MESENCHYMAL TRANSITION; GENE-EXPRESSION; CANCER; PROLIFERATION;

APOPTOSIS; MIRNA; RNAS

AB The present study aimed to determine the expression levels and biological functions of microRNA-202 (miR-202) in patients with esophageal squamous cell carcinoma (ESCC). A total of 60 patients with ESCC and 30 healthy individuals were enrolled and reverse transcription-quantitative polymerase chain reaction was performed to measure the expression levels of miR-202. In order to investigate the effects of miR-202 expression levels on the proliferative, migratory and invasive abilities of ESCC cells, methylthiazolyl-tetrazolium bromide proliferation, in vitro scratch and Transwell (R) chamber assays were performed. Expression levels of miR-202 were significantly decreased in the peripheral blood of patients with ESCC, which is associated with the degree of cell differentiation and lymph node metastasis (P<0.05). Following miR-202 transfection, cell proliferation was significantly inhibited (P<0.05). Cell migration and invasion was also significantly inhibited by miR-202 transfection (P<0.05). The results of the present study demonstrated that the expression of miR-202 inhibited the proliferation, migration and invasion of ESCC cells. Furthermore, low expression levels of miR-202 were detected in the peripheral blood of patients with ESCC, which is associated with the development, invasion and metastasis of ESCC.

C1 [Ma, Guoliang; Dong, Xueguang; Wang, Xiaoli; Ren, Yuguo] Laiwu Peoples Hosp, Dept Clin Lab, 1 Xuehu St, Laiwu 271199, Shandong, Peoples R China.

[Zhang, Fengmei] Laiwu Peoples Hosp, Dept Endocrinol, Laiwu 271199, Shandong, Peoples R China.

RP Ren, YG (corresponding author), Laiwu Peoples Hosp, Dept Clin Lab, 1 Xuehu St, Laiwu 271199, Shandong, Peoples R China.

EM ryg000@163.com

CR Akagi I, 2011, DIS ESOPHAGUS, V24, P523, DOI 10.1111/j.1442-2050.2011.01177.x

Arigami T, 2015, MEDICINE, V94, DOI 10.1097/MD.0000000000001702

Gadducci A, 2014, GYNECOL ENDOCRINOL, V30, P266, DOI 10.3109/09513590.2013.871525

Guo XF, 2014, J CARDIOTHORAC SURG, V9, DOI 10.1186/s13019-014-0150-4

Hu N, 2010, BMC GENOMICS, V11, DOI 10.1186/1471-2164-11-576

Jing W, 2015, PLOS ONE, V10, DOI 10.1371/journal.pone.0143007

Liu XL, 2015, CELL IMMUNOL, V295, P77, DOI 10.1016/j.cellimm.2015.03.011

Livak KJ, 2001, METHODS, V25, P402, DOI 10.1006/meth.2001.1262

Lu Y, 2015, J CANCER, V6, P1260, DOI 10.7150/jca.12659

Meng XR, 2014, BRAZ J MED BIOL RES, V47, P811, DOI 10.1590/1414-431X20143906

Niwa Y, 2014, J SURG ONCOL, V110, P764, DOI 10.1002/jso.23694

Okumura T, 2014, INT J ONCOL, V44, P1923, DOI 10.3892/ijo.2014.2365

Rawlings-Goss RA, 2014, BMC MED GENOMICS, V7, DOI 10.1186/1755-8794-7-53

Sakai NS, 2013, SEMIN CANCER BIOL, V23, P512, DOI 10.1016/j.semcancer.2013.08.005

Song B, 2015, ONCOTARGET, DOI 10.18632/oncotarget.6120

Sun ZW, 2014, MOL CELL BIOCHEM, V397, P277, DOI 10.1007/s11010-014-2195-z

Taccioli C, 2015, JNCI-J NATL CANCER I, V107, DOI 10.1093/jnci/djv220

Tang Shanhong, 2014, Xi Bao Yu Fen Zi Mian Yi Xue Za Zhi, V30, P411

Wen J, 2015, ANN SURG

Xing SZ, 2015, SUPPORT CARE CANCER, V23, P753, DOI 10.1007/s00520-014-2419-5

Yang M, 2014, MOL CELLS, V37, P873, DOI 10.14348/molcells.2014.0147

Yin XR, 2014, ONCOL REP, V32, P1748, DOI 10.3892/or.2014.3361

Yu Jia-jia, 2013, Zhonghua Zhong Liu Za Zhi, V35, P886

Yu XY, 2014, STEM CELLS DEV, V23, P576, DOI 10.1089/scd.2013.0308

Zhang HY, 2014, ONCOL REP, V32, P2134, DOI 10.3892/or.2014.3453

Zhang Y, 2014, FEBS LETT, V588, P1913, DOI 10.1016/j.febslet.2014.03.030

Zhong CX, 2014, TUMOR BIOL, V35, P4123, DOI 10.1007/s13277-013-1539-8

Zhong YL, 2015, BIOMED RES INT, V2015, DOI 10.1155/2015/960108

NR 28

TC 8

Z9 8

U1 0

U2 6

PU SPANDIDOS PUBL LTD

PI ATHENS

PA POB 18179, ATHENS, 116 10, GREECE

SN 1792-0981

EI 1792-1015

J9 EXP THER MED

JI Exp. Ther. Med.

PD MAR

PY 2016

VL 11

IS 3

BP 951

EP 956

DI 10.3892/etm.2016.3014

PG 6

WC Medicine, Research & Experimental

WE Science Citation Index Expanded (SCI-EXPANDED)

SC Research & Experimental Medicine

GA DC9PV

UT WOS:000369554400038

PM 26998018

OA Green Published, gold

DA 2022-05-19

ER

PT J

AU Yu, Q

Li, BX

Li, P

Shi, ZL

Vaughn, A

Zhu, LC

Fu, S

AF Yu, Qi

Li, Bingxin

Li, Ping

Shi, Zeliang

Vaughn, Amanda

Zhu, Liucun

Fu, Shen

TI Plasma microRNAs to predict the response of radiotherapy in esophageal

squamous cell carcinoma patients

SO AMERICAN JOURNAL OF TRANSLATIONAL RESEARCH

LA English

DT Article

DE Esophageal squamous cell carcinoma; microRNAs; plasma; radiotherapy

ID CIRCULATING MICRORNAS; RADIATION SENSITIVITY; GENE-EXPRESSION;

PROSTATE-CANCER; SERUM; RADIOSENSITIVITY; OVEREXPRESSION; MODULATION;

PROFILES; SURVIVAL

AB Although microRNAs (miRNAs) play an important role in esophageal squamous cell carcinoma (ESCC), their roles in radiotherapy response remain unexplored. Our study aims to investigate whether plasma miRNAs can be used as predictors of radiotherapy outcome in ESCC patients. We selected nine miRNAs, which were reported to be associated with carcinogenesis or radiobiology of ESCC, as the targets of our study. Plasma miRNA expression was investigated with 24 subjects (pre-, at the first week and post-radiotherapy). Tumor radiographic response and 3-year overall survival were used to evaluate the response. The results showed that the level of miR-16 in the patients with good outcome was significantly higher than that in the patients with poor outcome (P < 0.05, AUC: 0.762) at the post-radiotherapy. We also found that the variation tendency of miRNA expression were related to its radiotherapy response. Moreover, miR-16 levels increased by more than 2-fold following treatment, which were shown to be associated with longer OS (log-rank P = 0.009). In conclusion, miR-16 has considerable clinical value in predicting radiotherapy outcomes for ESCC patients.

C1 [Yu, Qi] Fudan Univ, Dept Radiat Oncol, Shanghai Canc Ctr, Shanghai 200433, Peoples R China.

[Yu, Qi; Fu, Shen] Fudan Univ, Shanghai Med Coll, Dept Oncol, Shanghai 200433, Peoples R China.

[Li, Bingxin] Wenzhou Med Univ, Affiliated Hosp 5, Cent Hosp Lishui, Dept Radiat Oncol, Lishui, Peoples R China.

[Li, Bingxin; Li, Ping; Shi, Zeliang; Fu, Shen] Shanghai Jiao Tong Univ, Dept Radiat Oncol, Affiliated Peoples Hosp 6, Shanghai 200030, Peoples R China.

[Vaughn, Amanda] Univ Texas, Inst Cellular & Mol Biol, Dept Mol Biosci, Austin, TX USA.

[Zhu, Liucun] Shanghai Univ, Sch Life Sci, Shanghai, Peoples R China.

[Fu, Shen] Fudan Univ, Dept Radiat Oncol, Shanghai Proton & Heavy Ion Ctr, Shanghai Canc Ctr, Shanghai 201321, Peoples R China.

RP Fu, S (corresponding author), Fudan Univ, Dept Radiat Oncol, Shanghai Proton & Heavy Ion Ctr, Shanghai Canc Ctr, 4365 Kangxin Rd, Shanghai 201321, Peoples R China.

EM shen_fu@hotmail.com

OI zhu, liucun/0000-0002-8334-0216

FU Shanghai Jiao Tong University [YG2012ZD02, 2JC1407400]; Science and

Technology Commission of Shanghai, China; National Natural Science

Foundation of China [81272506, 61227017]

FX We would like to thank Prof. Ying Wu, Department of Pathology, Shanghai

Medical College, Fudan University, Shanghai, China, for experimental

guidance. This article is funded by the research grant (YG2012ZD02,

2JC1407400) from Shanghai Jiao Tong University and the Science and

Technology Commission of Shanghai, China and the grant (81272506,

61227017) from National Natural Science Foundation of China.

CR Aqeilan RI, 2010, CELL DEATH DIFFER, V17, P215, DOI 10.1038/cdd.2009.69

Bolton WD, 2009, J THORAC CARDIOV SUR, V138, P831, DOI 10.1016/j.jtcvs.2009.02.003

Bonci D, 2008, NAT MED, V14, P1271, DOI 10.1038/nm.1880

Cai EH, 2012, ASIAN PAC J CANCER P, V13, P1563, DOI 10.7314/APJCP.2012.13.4.1563

Cellini F, 2014, MOLECULES, V19, P5379, DOI 10.3390/molecules19045379

Chen X, 2008, CELL RES, V18, P997, DOI 10.1038/cr.2008.282

Crosby ME, 2009, CANCER RES, V69, P1221, DOI 10.1158/0008-5472.CAN-08-2516

Edge SB, 2010, ANN SURG ONCOL, V17, P1471, DOI 10.1245/s10434-010-0985-4

Enzinger PC, 2003, NEW ENGL J MED, V349, P2241, DOI 10.1056/NEJMra035010

Fassan M, 2012, DIS ESOPHAGUS, V25, P263, DOI 10.1111/j.1442-2050.2011.01236.x

Feber A, 2008, J THORAC CARDIOV SUR, V135, P255, DOI 10.1016/j.jtcvs.2007.08.055

Glazer PM, 2011, RADIAT RES, V176, P412, DOI 10.1667/RRXX33.1

Guo Y, 2008, CANCER RES, V68, P26, DOI 10.1158/0008-5472.CAN-06-4418

Halimi M, 2012, INT J MOL CELL MED, V1, P178

Hamilton S R, 2000, WHO CLASSIFICATION T, V9-11

HANLEY JA, 1983, RADIOLOGY, V148, P839, DOI 10.1148/radiology.148.3.6878708

Hu J, 2014, INT J CANCER, V135, P1187, DOI 10.1002/ijc.28757

Huang S, 2013, DIS ESOPHAGUS, V26, P823, DOI 10.1111/j.1442-2050.2012.01389.x

Jemal A, 2011, CA-CANCER J CLIN, V61, P69, DOI [10.3322/caac.20107, 10.3322/caac.20115]

Josson S, 2008, PROSTATE, V68, P1599, DOI 10.1002/pros.20827

Kamangar F, 2006, INT J CANCER, V119, P579, DOI 10.1002/ijc.21871

Kelsen DP, 2000, CANCER J, V6, pS177

Komatsu S, 2011, BRIT J CANCER, V105, P104, DOI 10.1038/bjc.2011.198

Kong KL, 2012, GUT, V61, P33, DOI 10.1136/gutjnl-2011-300178

Kurashige J, 2012, BRIT J CANCER, V106, P182, DOI 10.1038/bjc.2011.509

Lan FM, 2015, INT J RADIAT ONCOL, V91, P73, DOI 10.1016/j.ijrobp.2014.09.021

Leidner RS, 2012, GENE CHROMOSOME CANC, V51, P473, DOI 10.1002/gcc.21934

Li SJ, 2011, J BIOMED SCI, V18, DOI 10.1186/1423-0127-18-24

Linsley PS, 2007, MOL CELL BIOL, V27, P2240, DOI 10.1128/MCB.02005-06

Liu Q, 2008, NUCLEIC ACIDS RES, V36, P5391, DOI 10.1093/nar/gkn522

Ma WJ, 2011, MOL BIOL REP, V38, P3253, DOI 10.1007/s11033-010-0480-9

Mahaney BL, 2009, BIOCHEM J, V417, P639, DOI 10.1042/BJ20080413

Mitchell PS, 2008, P NATL ACAD SCI USA, V105, P10513, DOI 10.1073/pnas.0804549105

Reynolds JV, 2007, ANN SURG, V245, P707, DOI 10.1097/01.sla.0000254367.15810.38

Sobin LH, 1997, CANCER, V80, P1803, DOI 10.1002/(SICI)1097-0142(19971101)80:9<1803::AID-CNCR16>3.0.CO;2-9

Song ZG, 2013, MED ONCOL, V30, DOI 10.1007/s12032-013-0756-8

Tsang TY, 2011, APOPTOSIS, V16, P524, DOI 10.1007/s10495-011-0581-5

Wang SY, 2015, INT J CANCER, V136, P152, DOI 10.1002/ijc.28136

Wang XC, 2013, J RADIAT RES, V54, P401, DOI 10.1093/jrr/rrs113

Wang XC, 2011, LUNG CANCER, V72, P92, DOI 10.1016/j.lungcan.2010.07.014

Wu KL, 2009, RADIOTHER ONCOL, V93, P454, DOI 10.1016/j.radonc.2009.10.014

Wu SB, 2014, INT J RADIAT BIOL, V90, P407, DOI 10.3109/09553002.2014.894649

Wu Y, 2012, J RADIAT RES, V53, P516, DOI 10.1093/jrr/rrs002

Yin E, 2003, INT J RADIAT BIOL, V79, P759, DOI 10.1080/09553000310001610961

Zamore PD, 2005, SCIENCE, V309, P1519, DOI 10.1126/science.1111444

Zhang CZ, 2010, BMC CANCER, V10, DOI 10.1186/1471-2407-10-367

Zhang CN, 2010, CLIN CHEM, V56, P1871, DOI 10.1373/clinchem.2010.147553

Zhang CZ, 2011, INT J RADIAT ONCOL, V80, P240, DOI 10.1016/j.ijrobp.2010.12.049

Zhu Y, 2014, CELL PHYSIOL BIOCHEM, V33, P1340, DOI 10.1159/000358701

ZWEIG MH, 1993, CLIN CHEM, V39, P561

NR 50

TC 14

Z9 17

U1 0

U2 2

PU E-CENTURY PUBLISHING CORP

PI MADISON

PA 40 WHITE OAKS LN, MADISON, WI 53711 USA

SN 1943-8141

J9 AM J TRANSL RES

JI Am. J. Transl. Res.

PY 2015

VL 7

IS 10

BP 2060

EP 2071

PG 12

WC Oncology; Medicine, Research & Experimental

WE Science Citation Index Expanded (SCI-EXPANDED)

SC Oncology; Research & Experimental Medicine

GA DA3BF

UT WOS:000367670200034

PM 26692950

DA 2022-05-19

ER

PT J

AU Li, Y

Guo, H

Dong, DF

Wu, HL

Li, EX

AF Li, Yi

Guo, Hui

Dong, Danfeng

Wu, Huili

Li, Enxiao

TI Expression and prognostic relevance of Cyclophilin A and matrix

metalloproteinase 9 in esophageal squamous cell carcinoma

SO DIAGNOSTIC PATHOLOGY

LA English

DT Article

DE Esophageal squamous cell carcinoma; Cyclophilin A; Matrix

metalloproteinase 9

ID COMPARATIVE PROTEOMIC ANALYSIS; PROTEIN EXPRESSION; COLORECTAL-CANCER;

POOR-PROGNOSIS; LUNG-CANCER; IDENTIFICATION; METASTASIS; BIOMARKERS;

GROWTH; CD147

AB Aims: To guide clinicians in selecting treatment options for esophageal squamous cell carcinoma (ESCC) patients, reliable markers predictive of clinical outcome are desirable. This study analyzed the correlation of cyclophilin A (CypA) and matrix metalloproteinase 9 (MMP9) in ESCC and their relationships to clinicopathological features and survival.

Methods: We immunohistochemically investigated 70 specimens of ESCC tissues using CypA and MMP9 antibodies. Then, the correlations between CypA and MMP9 expression and clinicopathological features and its prognostic relevance were determined.

Results: Significant correlations were only found in high level of CypA and MMP9 expression with tumor differentiation and lymph node status. Significant positive correlations were found between the expression status of CypA and that of MMP9. Overexpression of CypA and metastasis were significantly associated with shorter progression free survival times in univariate analysis. Multivariate analysis confirmed that CypA expression was an independent prognostic factor.

Conclusions: CypA might be correlated with the differentiation, and its elevated expression may be an adverse prognostic indicator for the patients of ESCC. CypA/MMP9 signal pathway may be attributed to the malignant transformation of ESCC, and attention should be paid to a possible target for therapy.

C1 [Li, Yi; Guo, Hui; Dong, Danfeng; Wu, Huili; Li, Enxiao] Xi An Jiao Tong Univ, Affiliated Hosp 1, Sch Med, Dept Oncol, Xian 710061, Shaanxi Provinc, Peoples R China.

RP Li, Y (corresponding author), Xi An Jiao Tong Univ, Affiliated Hosp 1, Sch Med, Dept Oncol, Yanta West Rd 277, Xian 710061, Shaanxi Provinc, Peoples R China.

EM liyi_0712@163.com

CR Al-Ghoul M, 2008, J PROTEOME RES, V7, P4107, DOI 10.1021/pr800174k

Calhoun CC, 2009, MOL CELL BIOCHEM, V320, P35, DOI 10.1007/s11010-008-9896-0

Cao W, 2013, BMC CANCER, V13, DOI 10.1186/1471-2407-13-305

Corton JC, 1998, CANCER LETT, V134, P61, DOI 10.1016/S0304-3835(98)00241-9

FRANKE EK, 1994, NATURE, V372, P359, DOI 10.1038/372359a0

Gwinn WM, 2006, J IMMUNOL, V177, P4870, DOI 10.4049/jimmunol.177.7.4870

Hathout Y, 2002, J PROTEOME RES, V1, P435, DOI 10.1021/pr020006i

Howard BA, 2004, LUNG CANCER, V46, P313, DOI 10.1016/j.lungcan.2004.05.013

Huang CF, 2011, ORAL DIS, V17, P328, DOI 10.1111/j.1601-0825.2010.01730.x

Huang CF, 2012, HISTOPATHOLOGY, V60, P793, DOI 10.1111/j.1365-2559.2011.04130.x

Jin ZG, 2000, CIRC RES, V87, P789, DOI 10.1161/01.RES.87.9.789

Kessenbrock K, 2010, CELL, V141, P52, DOI 10.1016/j.cell.2010.03.015

Lee J, 2010, J INT MED RES, V38, P1561, DOI 10.1177/147323001003800501

Li M, 2006, CANCER-AM CANCER SOC, V106, P2284, DOI 10.1002/cncr.21862

Li M, 2013, NANOSCALE RES LETT, V8, P1, DOI [10.1186/1556-276X-8-226, 10.1186/1556-276X-8-182]

Li ZY, 2008, MOL CELL PROTEOMICS, V7, P1810, DOI 10.1074/mcp.M700544-MCP200

Lockwood CJ, 2008, BIOL REPROD, V78, P1064, DOI 10.1095/biolreprod.107.063743

Lou JR, 2006, CANCER EPIDEM BIOMAR, V15, P1598, DOI 10.1158/1055-9965.EPI-06-0216

Moss LAS, 2012, AM J PATHOL, V181, P1895, DOI 10.1016/j.ajpath.2012.08.044

Obchoei S, 2009, MED SCI MONITOR, V15, pRA221

Parkin DM, 2001, EUR J CANCER, V37, pS4, DOI 10.1016/S0959-8049(01)00267-2

Peng J, 2013, UNIVERSITAS-TAIWAN, V40, P177

Peng Y, 2012, MOL BIOSYST, V8, P3077, DOI 10.1039/c2mb25286d

Qi YJ, 2008, J CELL BIOCHEM, V104, P1625, DOI 10.1002/jcb.21727

Qian Z, 2012, BMC CANCER, V12, DOI 10.1186/1471-2407-12-442

Samantaray S, 2004, J CANCER RES CLIN, V130, P37, DOI 10.1007/s00432-003-0500-4

SHERRY B, 1992, P NATL ACAD SCI USA, V89, P3511, DOI 10.1073/pnas.89.8.3511

Umar SB, 2008, NAT CLIN PRACT GASTR, V5, P517, DOI 10.1038/ncpgasthep1223

Yang J, 2011, CANCER BIOL THER, V11, P535, DOI 10.4161/cbt.11.5.14678

Zhang H, 2013, WORLD J SURG ONCOL, V11, DOI 10.1186/1477-7819-11-141

Zhang MJ, 2011, MOL CELL BIOCHEM, V357, P387, DOI 10.1007/s11010-011-0909-z

NR 31

TC 13

Z9 14

U1 0

U2 12

PU BIOMED CENTRAL LTD

PI LONDON

PA 236 GRAYS INN RD, FLOOR 6, LONDON WC1X 8HL, ENGLAND

SN 1746-1596

J9 DIAGN PATHOL

JI Diagn. Pathol.

PD DEC 18

PY 2013

VL 8

AR 207

DI 10.1186/1746-1596-8-207

PG 6

WC Pathology

WE Science Citation Index Expanded (SCI-EXPANDED)

SC Pathology

GA 290AO

UT WOS:000329724400001

PM 24351116

OA Green Published, gold

DA 2022-05-19

ER

PT J

AU Yang, CY

Guo, ZC

Zhao, ZT

Wei, Y

Wang, XX

Song, YM

AF Yang, Chengyuan

Guo, Zichan

Zhao, Zitong

Wei, Yuan

Wang, Xiaoxia

Song, Yongmei

TI miR-4306 Suppresses Proliferation of Esophageal Squamous Cell Carcinoma

Cell by Targeting SIX3

SO CELL BIOCHEMISTRY AND BIOPHYSICS

LA English

DT Article

DE miR-4306; Esophageal squamous cell carcinoma; SIX3; proliferation;

Invasion

ID THERAPEUTIC TARGET; METASTASIS

AB Esophageal squamous cell carcinoma (ESCC) is one of the most aggressive cancers and the primary cause of cancer-related mortality in China. micoRNA plays a vital role during tumor initiation and malignant progression. miR-4306 has been reported to negatively regulate aggressive cell phenotypes in triple-negative breast cancer (TNBC). Nevertheless, the function of miR-4306 in ESCC was still not clear. In this study, we detected miR-4306 expression by quantitative real-time reverse transcription-PCR (qRT-PCR) and found that miR-4306 expression was downregulated in human ESCC tissue samples and cell lines. Moreover, miR-4306 overexpression could restrain ESCC cell proliferation, migratory and invasive ability and epithelial-mesenchymal transition (EMT), promote cell apoptosis after treatment with or without cisplatin. In contrast, inhibiting the expression of miR-4306 showed the opposing results. Furthermore, we explored the molecular mechanism of effects of miR-4306 and found that miR-4306 inhibited the expression of SIX3 by interaction with SIX3 3 ' UTR in ESCC cells, and SIX3 overexpression significantly reversed the effect of miR-4306-mediated ESCC cells proliferation. The current study provided evidence of miR-4306 as a tumor suppression gene in ESCC.

C1 [Yang, Chengyuan; Guo, Zichan; Wei, Yuan; Wang, Xiaoxia] Shanxi Med Univ, Dept Biochem & Mol Biol, Taiyuan 030001, Shanxi, Peoples R China.

[Zhao, Zitong; Song, Yongmei] Chinese Acad Med Sci & Peking Union Med Coll, Natl Canc Ctr, State Key Lab Mol Oncol, Natl Clin Res Ctr Canc,Canc Hosp, Beijing 100021, Peoples R China.

RP Wang, XX (corresponding author), Shanxi Med Univ, Dept Biochem & Mol Biol, Taiyuan 030001, Shanxi, Peoples R China.; Song, YM (corresponding author), Chinese Acad Med Sci & Peking Union Med Coll, Natl Canc Ctr, State Key Lab Mol Oncol, Natl Clin Res Ctr Canc,Canc Hosp, Beijing 100021, Peoples R China.

EM wxiaoxia99007@126.com; songym@cicams.ac.cn

OI song, yongmei/0000-0002-7789-0158

FU National Natural Science Foundation of China [81872398]; Natural Science

Foundation of Shanxi Province, China [201901D111214]; Key Laboratory of

Cellular Physiology (Shanxi Medical University), Ministry of Education,

China [KLMC/SXMU-201906]

FX This research was supported by the National Natural Science Foundation

of China [grant no. 81872398], the Natural Science Foundation of Shanxi

Province, China [grant no. 201901D111214] and the Open Fund from Key

Laboratory of Cellular Physiology (Shanxi Medical University), Ministry

of Education, China [grant no. KLMC/SXMU-201906].

CR Abnet CC, 2018, GASTROENTEROLOGY, V154, P360, DOI 10.1053/j.gastro.2017.08.023

Appolloni I, 2008, CEREB CORTEX, V18, P553, DOI 10.1093/cercor/bhm092

Brabletz S, 2010, EMBO REP, V11, P670, DOI 10.1038/embor.2010.117

Bray F, 2018, CA-CANCER J CLIN, V68, P394, DOI 10.3322/caac.21492

Cantini L, 2019, NUCLEIC ACIDS RES, V47, P2205, DOI 10.1093/nar/gkz016

Chen YS, 2020, THERANOSTICS, V10, P4983, DOI 10.7150/thno.43046

Chen ZH, 2020, FASEB J, V34, P4072, DOI 10.1096/fj.201901764R

Du J, 2019, ANTI-CANCER DRUG, V30, P596, DOI 10.1097/CAD.0000000000000751

Hong BS, 2019, CANCER RES, V79, P1520, DOI 10.1158/0008-5472.CAN-18-0891

Hou J, 2011, CANCER CELL, V19, P232, DOI 10.1016/j.ccr.2011.01.001

Lai EC, 2002, NAT GENET, V30, P363, DOI 10.1038/ng865

Liu LY, 2015, J GENET GENOMICS, V42, P601, DOI 10.1016/j.jgg.2015.09.010

Liu Q, 2016, J HEMATOL ONCOL, V9, DOI 10.1186/s13045-016-0339-1

Ma L, 2007, NATURE, V449, P682, DOI 10.1038/nature06174

Mittal V, 2018, ANNU REV PATHOL-MECH, V13, P395, DOI 10.1146/annurev-pathol-020117-043854

Qi YJ, 2012, J PROTEOMICS, V75, P3129, DOI 10.1016/j.jprot.2012.04.025

Reichenbach ZW, 2019, ADV CANCER RES, V144, P95, DOI 10.1016/bs.acr.2019.05.004

Rooj AK, 2017, CELL REP, V19, P2026, DOI 10.1016/j.celrep.2017.05.040

Srivastava AK, 2019, CANCER RES, V79, P2314, DOI 10.1158/0008-5472.CAN-18-3668

Tanabe S, 2020, FRONT PHARMACOL, V11, DOI 10.3389/fphar.2020.00904

Turco C, 2020, FRONT CELL DEV BIOL, V8, DOI 10.3389/fcell.2020.00427

Umbreit C, 2014, ONCOL REP, V32, P668, DOI 10.3892/or.2014.3225

Van Roosbroeck K, 2017, CLIN CANCER RES, V23, P2891, DOI 10.1158/1078-0432.CCR-16-1025

Wang L, 2019, THORAC CANCER, V10, P60, DOI 10.1111/1759-7714.12902

Yang D, 2013, CANCER CELL, V23, P186, DOI 10.1016/j.ccr.2012.12.020

Yin HD, 2020, FRONT GENET, V11, DOI 10.3389/fgene.2020.00512

Zeng RJ, 2019, MOL ONCOL, V13, P2010, DOI 10.1002/1878-0261.12548

Zhang BX, 2017, J NEURO-ONCOL, V133, P509, DOI 10.1007/s11060-017-2476-y

Zhao ZT, 2019, THERANOSTICS, V9, P1401, DOI 10.7150/thno.30701

Zheng Y, 2018, THERANOSTICS, V8, P972, DOI 10.7150/thno.22328

NR 30

TC 0

Z9 0

U1 2

U2 2

PU HUMANA PRESS INC

PI TOTOWA

PA 999 RIVERVIEW DRIVE SUITE 208, TOTOWA, NJ 07512 USA

SN 1085-9195

EI 1559-0283

J9 CELL BIOCHEM BIOPHYS

JI Cell Biochem. Biophys.

PD DEC

PY 2021

VL 79

IS 4

BP 769

EP 779

DI 10.1007/s12013-021-00994-x

EA MAY 2021

PG 11

WC Biochemistry & Molecular Biology; Biophysics; Cell Biology

WE Science Citation Index Expanded (SCI-EXPANDED)

SC Biochemistry & Molecular Biology; Biophysics; Cell Biology

GA WP4XH

UT WOS:000652926300001

PM 34021861

DA 2022-05-19

ER

PT J

AU Dutta, M

Nakagawa, H

Kato, H

Maejima, K

Sasagawa, S

Nakano, K

Sasaki-Oku, A

Fujimoto, A

Mateos, RN

Patil, A

Tanaka, H

Miyano, S

Yasuda, T

Nakai, K

Fujita, M

AF Dutta, Munmee

Nakagawa, Hidewaki

Kato, Hiroaki

Maejima, Kazuhiro

Sasagawa, Shota

Nakano, Kaoru

Sasaki-Oku, Aya

Fujimoto, Akihiro

Mateos, Raul Nicolas

Patil, Ashwini

Tanaka, Hiroko

Miyano, Satoru

Yasuda, Takushi

Nakai, Kenta

Fujita, Masashi

TI Whole genome sequencing analysis identifies recurrent structural

alterations in esophageal squamous cell carcinoma

SO PEERJ

LA English

DT Article

DE Esophageal squamous cell carcinoma; Whole genome sequencing; Coding

mutation; Mutational signature; Structural variation; Copy number

alteration; Druggable gene; LRP1B; FGFR1; FAT1

ID MUTATIONAL SIGNATURES; SOMATIC MUTATION; WIDE ASSOCIATION;

BREAST-CANCER; EXPRESSION; PATTERNS; GENES; CHROMOTHRIPSIS;

TUMORIGENESIS; LANDSCAPE

AB Esophageal squamous cell carcinoma (ESCC) is the predominant type of esophageal cancer in the Asian region, including Japan. A previous study reported mutational landscape of Japanese ESCCs by using exome sequencing. However, somatic structural alterations were yet to be explored. To provide a comprehensive mutational landscape, we performed whole genome sequencing (WGS) analysis of biopsy specimens from 20 ESCC patients in a Japanese population. WGS analysis identified non-silent coding mutations of TP53, ZNF750 and FAT1 in ESCC. We detected six mutational signatures in ESCC, one of which showed significant association with smoking status. Recurrent structural variations, many of which were chromosomal deletions, affected genes such as LRP1B, TTC28, CSMD1, PDE4D, SDKI and WWOX in 25%-30% of tumors. Somatic copy number amplifications at 11q13.3 (CCNDI), 3q26.33 (TP63/SOX2), and 8p11.23 (FGFRI) and deletions at 9p21.3 (CDKN2A) were identified. Overall, these multi-dimensional view of genomic alterations improve the understanding of the ESCC development at molecular level and provides future prognosis and therapeutic implications for ESCC in Japan.

C1 [Dutta, Munmee; Mateos, Raul Nicolas; Nakai, Kenta] Univ Tokyo, Grad Sch Frontier Sci, Dept Computat Biol & Med Sci, Chiba, Japan.

[Dutta, Munmee; Mateos, Raul Nicolas; Patil, Ashwini; Tanaka, Hiroko; Miyano, Satoru; Nakai, Kenta] Univ Tokyo, Human Genome Ctr, Inst Med Sci, Tokyo, Japan.

[Nakagawa, Hidewaki; Maejima, Kazuhiro; Sasagawa, Shota; Nakano, Kaoru; Sasaki-Oku, Aya; Fujita, Masashi] RIKEN Ctr Integrat Med Sci, Lab Canc Genom, Yokohama, Kanagawa, Japan.

[Kato, Hiroaki; Yasuda, Takushi] Kindai Univ, Fac Med, Dept Surg, Osaka, Japan.

[Fujimoto, Akihiro] Kyoto Univ, Dept Drug Discovery Med, Grad Sch Med, Kyoto, Japan.

[Miyano, Satoru] Univ Tokyo, Hlth Intelligence Ctr, Inst Med Sci, Tokyo, Japan.

RP Nakagawa, H; Fujita, M (corresponding author), RIKEN Ctr Integrat Med Sci, Lab Canc Genom, Yokohama, Kanagawa, Japan.

EM hidewaki@riken.jp; m-fujita@riken.jp

RI Nakai, Kenta/B-7293-2009; Dutta, Munmee/AAB-7976-2022

OI Nakai, Kenta/0000-0002-8721-8883; Patil, Ashwini/0000-0002-3353-1970;

Dutta, Munmee/0000-0003-1644-0396

FU Japan Agency for Medical Research and Development (AMED) Project for

Cancer Research and Therapeutic Evolution (P-CREATE); JSPS KAKENHI

[19K09206]; Japanese Government Scholarship (Monbukagakusho, MEXT)

FX This work was supported by the Japan Agency for Medical Research and

Development (AMED) Project for Cancer Research and Therapeutic Evolution

(P-CREATE) (to Hidewaki Nakagawa) and JSPS KAKENHI Grant Number 19K09206

(to Masashi Fujita). Munmee Dutta was supported by the Japanese

Government Scholarship (Monbukagakusho, MEXT) for graduate studies.

There was no additional external funding received for this study. The

funders had no role in study design, data collection and analysis,

decision to publish, or preparation of the manuscript.

CR Alexandrov LB, 2016, SCIENCE, V354, P618, DOI 10.1126/science.aag0299

Alexandrov LB, 2013, NATURE, V500, P415, DOI 10.1038/nature12477

Baba Y, 2014, WORLD J GASTRO ONCOL, V6, P121, DOI 10.4251/wjgo.v6.i5.121

Campbell PJ, 2010, NATURE, V467, P1109, DOI 10.1038/nature09460

Candi E, 2014, HUM MUTAT, V35, P702, DOI 10.1002/humu.22523

Chang J, 2017, NAT COMMUN, V8, DOI 10.1038/ncomms15290

Chang JJ, 2014, PLOS ONE, V9, DOI 10.1371/journal.pone.0105524

Chattopadhyay I, 2010, MUTAT RES-GEN TOX EN, V696, P130, DOI 10.1016/j.mrgentox.2010.01.001

Cheng CX, 2016, AM J HUM GENET, V98, P256, DOI 10.1016/j.ajhg.2015.12.013

Choi M, 2017, ANN ONCOL, V28, P83, DOI 10.1093/annonc/mdw437

Cortes-Ciriano I, 2020, NAT GENET, V52, P331, DOI 10.1038/s41588-019-0576-7

Cotto Kelsy C, 2018, Nucleic Acids Res, V46, pD1068, DOI 10.1093/nar/gkx1143

D'Antonio M, 2016, CELL REP, V16, P672, DOI 10.1016/j.celrep.2016.06.026

De Mello Ramon Andrade, 2018, Am Soc Clin Oncol Educ Book, V38, P249, DOI 10.1200/EDBK_198805

Deng NT, 2012, GUT, V61, P673, DOI 10.1136/gutjnl-2011-301839

Du PN, 2017, SCI REP-UK, V7, DOI 10.1038/s41598-017-14909-5

Fang JA, 2017, BMC CANCER, V17, DOI 10.1186/s12885-017-3317-2

Gentilini D, 2017, ONCOTARGET, V8, P41890, DOI 10.18632/oncotarget.17462

Griffith M, 2013, NAT METHODS, V10, P1209, DOI [10.1038/NMETH.2689, 10.1038/nmeth.2689]

Harris RS, 2002, MOL CELL, V10, P1247, DOI 10.1016/S1097-2765(02)00742-6

Hazawa M, 2017, ONCOGENE, V36, P2243, DOI 10.1038/onc.2016.377

Hermetz KE, 2014, PLOS GENET, V10, DOI 10.1371/journal.pgen.1004139

Hussain T, 2019, GENE CHROMOSOME CANC, V58, P324, DOI 10.1002/gcc.22693

Juhlin CC, 2015, J CLIN ENDOCR METAB, V100, pE493, DOI 10.1210/jc.2014-3282

Jung AR, 2018, INT J MOL SCI, V19, DOI 10.3390/ijms19123996

Kim J, 2017, NATURE, V541, P169, DOI 10.1038/nature20805

Koboldt DC, 2012, GENOME RES, V22, P568, DOI 10.1101/gr.129684.111

Korbel JO, 2013, CELL, V152, P1226, DOI 10.1016/j.cell.2013.02.023

Lawrence MS, 2015, NATURE, V517, P576, DOI 10.1038/nature14129

Li CG, 2015, SCI REP-UK, V5, DOI 10.1038/srep14237

Li XC, 2018, ANN ONCOL, V29, P938, DOI 10.1093/annonc/mdy011

Lin DC, 2014, NAT GENET, V46, P467, DOI 10.1038/ng.2935

Lin DC, 2013, P NATL ACAD SCI USA, V110, P6109, DOI 10.1073/pnas.1218206110

Lin SC, 2018, CARCINOGENESIS, V39, P1320, DOI 10.1093/carcin/bgy107

Lin YS, 2013, J EPIDEMIOL, V23, P233, DOI 10.2188/jea.JE20120162

Martincorena I, 2017, CELL, V171, P1029, DOI 10.1016/j.cell.2017.09.042

Mayakonda A, 2018, GENOME RES, V28, P1747, DOI 10.1101/gr.239244.118

Mermel CH, 2011, GENOME BIOL, V12, DOI 10.1186/gb-2011-12-4-r41

Morris LGT, 2013, NAT GENET, V45, P253, DOI 10.1038/ng.2538

Nik-Zainal S, 2012, CELL, V149, P979, DOI 10.1016/j.cell.2012.04.024

Ohnami S, 2017, MOL CELL BIOCHEM, V431, P75, DOI 10.1007/s11010-017-2977-1

Padhi SS, 2017, ORAL ONCOL, V73, P27, DOI 10.1016/j.oraloncology.2017.07.030

Qin HD, 2016, AM J HUM GENET, V98, P709, DOI 10.1016/j.ajhg.2016.02.021

Rode A, 2016, INT J CANCER, V138, P2322, DOI 10.1002/ijc.29888

Sawada G, 2016, GASTROENTEROLOGY, V150, P1171, DOI 10.1053/j.gastro.2016.01.035

Secrier M, 2016, NAT GENET, V48, P1131, DOI 10.1038/ng.3659

Song YM, 2014, NATURE, V509, P91, DOI 10.1038/nature13176

Szczepanski MJ, 2013, ORAL ONCOL, V49, P144, DOI 10.1016/j.oraloncology.2012.08.005

Tabesh GA, 2017, CANCER INVEST, V35, P36, DOI 10.1080/07357907.2016.1247455

von Loga K, 2015, PLOS ONE, V10, DOI 10.1371/journal.pone.0141867

Wang Jingming, 2019, Oncotarget, V10, P3835, DOI 10.18632/oncotarget.26982

Wang K, 2010, NUCLEIC ACIDS RES, V38, DOI 10.1093/nar/gkq603

Weber M, 2014, J CRANIO MAXILL SURG, V42, P1087, DOI 10.1016/j.jcms.2014.01.035

Wen H., 2016, SCI REP-UK, V6, P1, DOI [10.1038/srep25408, DOI 10.1038/SREP25408), 10.1038/s41598-016-0001-8, DOI 10.1038/S41598-016-0001-8]

Wu C, 2011, NAT GENET, V43, P679, DOI 10.1038/ng.849

Yang B, 2017, J HUM GENET, V62, P783, DOI 10.1038/jhg.2017.40

Ying JM, 2012, PLOS ONE, V7, DOI 10.1371/journal.pone.0039797

Yokota T, 2018, BMC CANCER, V18, DOI 10.1186/s12885-018-4733-7

Yoshida K, 2018, RECENT ADV ESOPHAGEA, P253, DOI [10.1002/ags3.12174, DOI 10.1002/AGS3.12174]

Zhang L, 2015, AM J HUM GENET, V96, P597, DOI 10.1016/j.ajhg.2015.02.017

Zhang XZ, 2019, PEERJ, V7, DOI 10.7717/peerj.6375

Zhen DB, 2015, GENET MED, V17, P569, DOI 10.1038/gim.2014.153

NR 62

TC 5

Z9 5

U1 1

U2 3

PU PEERJ INC

PI LONDON

PA 341-345 OLD ST, THIRD FLR, LONDON, EC1V 9LL, ENGLAND

SN 2167-8359

J9 PEERJ

JI PeerJ

PD JUN 26

PY 2020

VL 8

AR e9294

DI 10.7717/peerj.9294

PG 24

WC Multidisciplinary Sciences

WE Science Citation Index Expanded (SCI-EXPANDED)

SC Science & Technology - Other Topics

GA MD0AP

UT WOS:000543639200002

PM 32617189

OA gold, Green Published

DA 2022-05-19

ER

PT J

AU Qian, ZY

Wei, B

Zhou, Y

Wang, QZ

Wang, JR

Sun, Y

Gao, Y

Chen, XF

AF Qian, Zhaoye

Wei, Bin

Zhou, Yu

Wang, Qiuzi

Wang, Jiru

Sun, Yuan

Gao, Yong

Chen, Xiaofei

TI PRAF2 overexpression predicts poor prognosis and promotes tumorigenesis

in esophageal squamous cell carcinoma

SO BMC CANCER

LA English

DT Article

DE PRAF2; Prognosis; Proliferation; Invasion; Apoptosis; Esophageal

squamous cell carcinoma

ID DOMAIN FAMILY; EXPRESSION; CANCER; JWA; SURVIVAL; MIGRATION; THERAPY;

BINDING; EXPORT; CCR5

AB BackgroundPrenylated Rab acceptor 1 domain family, member 2 (PRAF2) is involved in the occurrence and progression of several malignant tumors. However, its potential role in esophageal squamous cell carcinoma (ESCC) is still unknown.MethodsPRAF2 mRNA expression was determined in 77 frozen ESCC samples by quantitative reverse transcription-polymerase chain reaction (qPCR) and its association with clinical features and overall survival were evaluated. The roles of PRAF2 in ESCC cells were investigated by proliferation, cell cycle, invasion and apoptosis assays in vitro.ResultsThe PRAF2 mRNA expression was significantly increased in ESCC tissues compared with matched surrounding non-tumor tissues. Survival analysis showed that high PRAF2 mRNA expression was associated with worse overall survival in ESCC patients. Multivariate analysis revealed that PRAF2 (hazard ratio 2.05, 95% CI 1.10-3.85, P=0.025) emerged as the independent predictor for poor overall survival in ESCC. The in vitro experiments revealed that knockdown of PRAF2 expression blocked cell proliferation, cell cycle progression and cell invasion and induced cell apoptosis in ESCC cells.ConclusionTaken together, our data demonstrate that PRAF2 could be used as a potential prognostic biomarker and represent a potential therapeutic target for ESCC.

C1 [Qian, Zhaoye; Wei, Bin; Zhou, Yu; Wang, Qiuzi; Wang, Jiru; Sun, Yuan; Gao, Yong; Chen, Xiaofei] Nanjing Med Univ, Affiliated Huaian Peoples Hosp 1, Dept Med Oncol, Huaian 223300, Peoples R China.

RP Gao, Y; Chen, XF (corresponding author), Nanjing Med Univ, Affiliated Huaian Peoples Hosp 1, Dept Med Oncol, Huaian 223300, Peoples R China.

EM qzy09010625@sina.cn; hayy_gy@163.com; hayycxf@163.com

OI Zhou, Yu/0000-0003-0457-221X

FU National Natural Science Foundation of China [81572421]; Jiangsu

Provincial Commission of Health and Family Planning Program [H201555];

Huai'an Governmental Science Developing program [HACZ2014002]

FX The present study was supported by the National Natural Science

Foundation of China (grant no. 81572421), the Jiangsu Provincial

Commission of Health and Family Planning Program (grant no. H201555) and

Huai'an Governmental Science Developing program (grant no. HACZ2014002).

The funding body had no role in the design of the study and collection,

analysis, and interpretation of data and in writing the manuscript.

CR Bai J, 2010, ONCOGENE, V29, P1227, DOI 10.1038/onc.2009.408

Borsics T, 2010, CANCER SCI, V101, P1624, DOI 10.1111/j.1349-7006.2010.01570.x

Chandrashekar DS, 2017, NEOPLASIA, V19, P649, DOI 10.1016/j.neo.2017.05.002

Chen HR, 2007, CELL SIGNAL, V19, P1315, DOI 10.1016/j.cellsig.2007.01.007

Cifuentes-Diaz C, 2016, BRAIN STRUCT FUNCT, V221, P4169, DOI 10.1007/s00429-015-1159-8

Doly S, 2016, MOL PSYCHIATR, V21, P480, DOI 10.1038/mp.2015.72

Doly S, 2015, TRENDS PHARMACOL SCI, V36, P636, DOI 10.1016/j.tips.2015.06.007

Ferlay J, 2015, INT J CANCER, V136, pE359, DOI 10.1002/ijc.29210

Fo CS, 2006, GENE, V371, P154, DOI 10.1016/j.gene.2005.12.009

Geerts D, 2007, CLIN CANCER RES, V13, P6312, DOI 10.1158/1078-0432.CCR-07-0829

Kim JT, 2006, BIOCHEM BIOPH RES CO, V349, P200, DOI 10.1016/j.bbrc.2006.08.026

Kouno J, 2004, J NEURO-ONCOL, V70, P301, DOI 10.1007/s11060-004-9165-3

Li LY, 2001, J BIOL CHEM, V276, P27354, DOI 10.1074/jbc.M103821200

Liu YJ, 2016, ANN NY ACAD SCI, V1381, P66, DOI 10.1111/nyas.13144

Pennathur A, 2013, LANCET, V381, P400, DOI 10.1016/S0140-6736(12)60643-6

Pennathur A, 2009, ANN THORAC SURG, V87, P1048, DOI 10.1016/j.athoracsur.2008.12.060

Pusung M, 2016, ADV EXP MED BIOL, V908, P237, DOI 10.1007/978-3-319-41388-4_12

Roos WP, 2016, NAT REV CANCER, V16, P20, DOI 10.1038/nrc.2015.2

Ruggiero AM, 2008, J BIOL CHEM, V283, P6175, DOI 10.1074/jbc.M701008200

Schweneker M, 2005, FEBS LETT, V579, P1751, DOI 10.1016/j.febslet.2005.02.037

Torre LA, 2015, CA-CANCER J CLIN, V65, P87, DOI 10.3322/caac.21262

Vento MT, 2010, PLOS ONE, V5, DOI 10.1371/journal.pone.0015636

Wang CH, 2018, CANCER MANAG RES, V10, P2241, DOI 10.2147/CMAR.S166789

Wei B, 2015, BMC CANCER, V15, DOI 10.1186/s12885-015-1364-0

Wu XF, 2014, MOL CARCINOGEN, V53, P325, DOI 10.1002/mc.21981

Yco LP, 2013, INT J ONCOL, V42, P1408, DOI 10.3892/ijo.2013.1836

Zhang HZ, 2012, CHIN J CANCER, V31, P281, DOI 10.5732/cjc.011.10390

NR 27

TC 6

Z9 6

U1 3

U2 3

PU BMC

PI LONDON

PA CAMPUS, 4 CRINAN ST, LONDON N1 9XW, ENGLAND

SN 1471-2407

J9 BMC CANCER

JI BMC Cancer

PD JUN 14

PY 2019

VL 19

AR 585

DI 10.1186/s12885-019-5818-7

PG 8

WC Oncology

WE Science Citation Index Expanded (SCI-EXPANDED)

SC Oncology

GA ID3XK

UT WOS:000471610200005

PM 31200670

OA Green Published, gold

DA 2022-05-19

ER

PT J

AU Zhang, J

Kong, RR

Sun, LZ

AF Zhang, Jin

Kong, Ranran

Sun, Liangzhang

TI Silencing of Rab3D suppresses the proliferation and invasion of

esophageal squamous cell carcinoma cells

SO BIOMEDICINE & PHARMACOTHERAPY

LA English

DT Article

DE Esophageal squamous cell carcinoma (ESCC); Rab3D; Proliferation;

Invasion

ID CANCER; METASTASIS; ACTIVATION; GTPASES; FAMILY

AB Rab3D is a member of the ras-related GTP-binding protein Rab family and was found up-regulated in several types of cancer. However, little is known about the role of Rab3D in carcinogenesis and progression of esophageal squamous cell carcinoma (ESCC). Thus, in this study, we investigated the expression patterns and functional roles of Rab3D in human ESCC. We demonstrated that Rab3D was highly expressed in human ESCC cell lines. In addition, knockdown of Rab3D significantly inhibited the proliferation of ESCC cells and reduced the tumorigenesis in vivo. Moreover, knockdown of Rab3D significantly suppressed ESCC cell migration/invasion and accordingly alerted EMT related markers, which including up-regulated E-cadherin and down-regulated N-cadherin in ESCC cells. Finally, knockdown of Rab3D inhibited the levels of p-PI3K and p-Akt in ECA-109 cells. In conclusion, our data demonstrated that Rab3D functions as an oncogene in ESCC and knockdown of Rab3D suppressed ESCC cell proliferation and invasion, potentially through the PI3K/Akt signaling pathway. Overall, these findings suggest that targeting the Rab3D may be a potential therapeutic target for treatment of ESCC. (C) 2017 Elsevier Masson SAS. All rights reserved.

C1 [Zhang, Jin; Kong, Ranran; Sun, Liangzhang] Xi An Jiao Tong Univ, Affiliated Hosp 2, Dept Thorac Surg, Xian 710004, Shaanxi, Peoples R China.

RP Zhang, J (corresponding author), Xi An Jiao Tong Univ, Affiliated Hosp 2, Dept Thorac Surg, Xian 710004, Shaanxi, Peoples R China.

EM jinzhangxa@126.com

RI kong, Ranran/AAT-7513-2021; Kong, Ranran/AAA-2052-2020

OI kong, ranran/0000-0002-3658-8745

CR Chen J, 2015, DIS ESOPHAGUS, V28, P84, DOI 10.1111/dote.12088

Chia WJ, 2009, BBA-REV CANCER, V1795, P110, DOI 10.1016/j.bbcan.2008.10.001

D'Journo X. B., 2014, J THORAC DIS, V6, P1

da Silva SD, 2015, CLIN EXP METASTAS, V32, P55, DOI 10.1007/s10585-014-9690-1

De Luca A, 2012, EXPERT OPIN THER TAR, V16, pS17, DOI 10.1517/14728222.2011.639361

Kim T, 2011, J SURG ONCOL, V103, P179, DOI 10.1002/jso.21784

Kranzfelder M, 2009, ADV MED SCI-POLAND, V54, P158, DOI 10.2478/v10039-009-0044-1

Li B, 2009, INT J CANCER, V125, P2576, DOI 10.1002/ijc.24675

Li J, 2011, BMC CANCER, V11, P1

Liu M, 2016, CANCER LETT, V377, P97, DOI 10.1016/j.canlet.2016.04.034

Liu T, 2016, ONCOTARGET, V7, P66851, DOI 10.18632/oncotarget.11694

Luo Y, 2016, INT J BIOCHEM CELL B, V75, P53, DOI 10.1016/j.biocel.2016.03.017

Martini M, 2014, ANN MED, V46, P372, DOI 10.3109/07853890.2014.912836

Micalizzi DS, 2009, FUTURE ONCOL, V5, P1129, DOI [10.2217/fon.09.94, 10.2217/FON.09.94]

Nie ZC, 2015, INT J CLIN EXP PATHO, V8, P4745

NOVICK P, 1993, CELL, V75, P597, DOI 10.1016/0092-8674(93)90478-9

Pennathur A, 2013, LANCET, V381, P400, DOI 10.1016/S0140-6736(12)60643-6

Stenmark H, 2001, GENOME BIOL, V2

Torre L., 2011, CA-CANCER J CLIN, V61

Valentijn JA, 1996, EUR J CELL BIOL, V70, P33

Vara JAF, 2004, CANCER TREAT REV, V30, P193, DOI 10.1016/j.ctrv.2003.07.007

Wang YY, 2015, DRUG DES DEV THER, V9, P993, DOI 10.2147/DDDT.S73493

Yang J, 2015, ONCOTARGET, V6, P11125, DOI 10.18632/oncotarget.3575

Yoshioka A, 2008, ONCOL REP, V19, P1099

Zhao H, 2012, TUMOR BIOL, V33, P1363, DOI 10.1007/s13277-012-0384-5

NR 25

TC 18

Z9 19

U1 0

U2 5

PU ELSEVIER FRANCE-EDITIONS SCIENTIFIQUES MEDICALES ELSEVIER

PI ISSY-LES-MOULINEAUX

PA 65 RUE CAMILLE DESMOULINS, CS50083, 92442 ISSY-LES-MOULINEAUX, FRANCE

SN 0753-3322

EI 1950-6007

J9 BIOMED PHARMACOTHER

JI Biomed. Pharmacother.

PD JUL

PY 2017

VL 91

BP 402

EP 407

DI 10.1016/j.biopha.2017.04.010

PG 6

WC Medicine, Research & Experimental; Pharmacology & Pharmacy

WE Science Citation Index Expanded (SCI-EXPANDED)

SC Research & Experimental Medicine; Pharmacology & Pharmacy

GA FB8PB

UT WOS:000406399900047

PM 28472755

DA 2022-05-19

ER

PT J

AU Jiang, YA

Wu, Q

Yang, XW

Zhao, JM

Jin, YX

Li, K

Ma, YH

Chen, XH

Tian, F

Zhao, S

Xu, JL

Lu, J

Yin, XS

Liu, KD

Dong, ZM

AF Jiang, Yanan

Wu, Qiong

Yang, Xiawen

Zhao, Jimin

Jin, Yuxi

Li, Ke

Ma, Yihui

Chen, Xinhuan

Tian, Fang

Zhao, Song

Xu, Jinglong

Lu, Jing

Yin, Xueshan

Liu, Kangdong

Dong, Ziming

TI A method for establishing a patient-derived xenograft model to explore

new therapeutic strategies for esophageal squamous cell carcinoma

SO ONCOLOGY REPORTS

LA English

DT Article

DE esophageal squamous cell carcinoma; patient-derived; xenograft;

signaling pathway

ID TUMOR XENOGRAFTS; BREAST-CANCER; PATHWAY; GROWTH; INHIBITOR; STAT3;

INTERFERON; ACTIVATION; SENSITIZES; CISPLATIN

AB Esophageal squamous cell carcinoma (ESCC) is the predominant histological type of esophageal carcinoma in China. The overall 5-year survival rate of ESCC patients is in the low range of 15-25%. One important reason for the poor prognosis is that the underlying molecular mechanisms are unclear. Furthermore, the development of effective therapeutic strategies to improve patient outcome is needed. Animal models can be beneficial to analyze the molecular mechanisms as well as specific clinical therapeutic strategies for esophageal cancer. In recent years, patient-derived xenografts (PDXs) have been widely used in numerous types of cancers to investigate the basic mechanisms and to conduct preclinical research. Accumulating evidence indicates that the PDX model is an important tool for basic and clinical research. Herein, we successfully established 14 ESCC PDXs. These PDX models preserved the patient pathological characteristics and effectively reflected the patient biological heterogeneity. Cancers exhibit diverse growth rates and tumor texture, even more, they have different signaling pathways. The PDX model is a superior strategy for understanding the underlying molecular mechanisms of ESCC and for screening new therapeutic strategies for ESCC patients.

C1 [Jiang, Yanan; Wu, Qiong; Zhao, Jimin; Jin, Yuxi; Li, Ke; Chen, Xinhuan; Tian, Fang; Xu, Jinglong; Lu, Jing; Yin, Xueshan; Liu, Kangdong; Dong, Ziming] Zhengzhou Univ, Sch Basic Med Sci, Dept Pathophysiol, Zhengzhou 450001, Henan, Peoples R China.

[Jiang, Yanan; Zhao, Jimin; Chen, Xinhuan; Tian, Fang; Lu, Jing; Liu, Kangdong; Dong, Ziming] Zhengzhou Univ, Henan Prov Cooperat Innovat Ctr Canc Chemoprevent, Zhengzhou 450001, Henan, Peoples R China.

[Yang, Xiawen] Zhengzhou Univ, Sch Life Sci, Zhengzhou 450001, Henan, Peoples R China.

[Ma, Yihui; Zhao, Song] Zhengzhou Univ, Affiliated Hosp 1, Zhengzhou 450052, Henan, Peoples R China.

[Wu, Qiong; Liu, Kangdong] China US Henan Hormel Canc Inst, Zhengzhou 450008, Henan, Peoples R China.

RP Dong, ZM (corresponding author), Zhengzhou Univ, Sch Basic Med Sci, Dept Pathophysiol, 100 Kexue Ave, Zhengzhou 450001, Henan, Peoples R China.

EM kdliu@zzu.edu.cn; dongzm@zzu.edu.cn

FU National Sciences Foundation of China [81372269, U1304813, 81472324];

Science Foundation of the Henan Province of China [13A310553,

15B310013]; Young Teacher Special Fund of Zhengzhou University

[1421328055]; Young Teacher Training of Zhengzhou University

FX The present study was supported by the National Sciences Foundation of

China (nos. 81372269, U1304813 and 81472324), the Science Foundation of

the Henan Province of China (nos. 13A310553 and 15B310013), the Young

Teacher Special Fund of Zhengzhou University (no. 1421328055) and the

Young Teacher Training of Zhengzhou University. We would like to express

our thanks to Dr Fred Bogott for critically reading the manuscript and

offering good suggestions.

CR Adachi Y, 2014, TUMOR BIOL, V35, P973, DOI 10.1007/s13277-013-1131-2

Baselga J, 2002, J CLIN ONCOL, V20, P4292, DOI 10.1200/JCO.2002.03.100

Belkhiri A, 2015, ONCOTARGET, V6, P1348, DOI 10.18632/oncotarget.2752

Bhalla S, 2011, BLOOD, V118, P1052, DOI 10.1182/blood-2011-03-340109

BLOBEL GA, 1985, AM J PATHOL, V121, P235

Ciccarelli A, 2014, NEUROPHARMACOLOGY, V80, P34, DOI 10.1016/j.neuropharm.2014.01.039

Dong SW, 2014, ONCOL REP, V32, P1064, DOI 10.3892/or.2014.3307

Geuna E, 2015, EXPERT OPIN INV DRUG, V24, P421, DOI 10.1517/13543784.2015.1008132

Harb WA, 2015, CANCER MANAG RES, V7, P37, DOI 10.2147/CMAR.S72592

Honjo S, 2014, INT J ONCOL, V45, P567, DOI 10.3892/ijo.2014.2450

Hou GQ, 2014, J IMMUNOL RES, V2014, DOI 10.1155/2014/845763

Huang Y, 2013, ANTI-CANCER DRUG, V24, P889, DOI 10.1097/CAD.0b013e328363c64e

Inoue A, 2003, LANCET, V361, P137, DOI 10.1016/S0140-6736(03)12190-3

Li B, 2014, ONCOTARGET, V5, P11576, DOI 10.18632/oncotarget.2596

Lin DC, 2014, NAT GENET, V46, P467, DOI 10.1038/ng.2935

Marangoni E, 2007, CLIN CANCER RES, V13, P3989, DOI 10.1158/1078-0432.CCR-07-0078

Moro M, 2012, J BIOMED BIOTECHNOL, DOI 10.1155/2012/568567

Morton CL, 2007, NAT PROTOC, V2, P247, DOI 10.1038/nprot.2007.25

Napier KJ, 2014, WORLD J GASTRO ONCOL, V6, P112, DOI 10.4251/wjgo.v6.i5.112

Qing YL, 2004, J BIOL CHEM, V279, P41679, DOI 10.1074/jbc.M406413200

Regis G, 2008, SEMIN CELL DEV BIOL, V19, P351, DOI 10.1016/j.semcdb.2008.06.004

Sakamoto C, 2002, J GASTROENTEROL, V37, P575, DOI 10.1007/s005350200090

Siolas D, 2013, CANCER RES, V73, P5315, DOI 10.1158/0008-5472.CAN-13-1069

Song YM, 2014, NATURE, V509, P91, DOI 10.1038/nature13176

Thepot A, 2010, INT J CANCER, V127, P2051, DOI 10.1002/ijc.25221

Tian H, 2015, ONCOL REP, V33, P981, DOI 10.3892/or.2014.3657

Uemura Norihisa, 2014, World J Gastrointest Pathophysiol, V5, P322, DOI 10.4291/wjgp.v5.i3.322

Wang ZP, 2013, CARCINOGENESIS, V34, P678, DOI 10.1093/carcin/bgs361

Ye S, 2014, INT J ONCOL, V44, P2153, DOI 10.3892/ijo.2014.2374

Zhang J, 2005, J PATHOL, V207, P402, DOI 10.1002/path.1846

Zhao P, 2010, JPN J CLIN ONCOL, V40, P281, DOI 10.1093/jjco/hyp187

NR 31

TC 13

Z9 14

U1 3

U2 17

PU SPANDIDOS PUBL LTD

PI ATHENS

PA POB 18179, ATHENS, 116 10, GREECE

SN 1021-335X

EI 1791-2431

J9 ONCOL REP

JI Oncol. Rep.

PD FEB

PY 2016

VL 35

IS 2

BP 785

EP 792

DI 10.3892/or.2015.4459

PG 8

WC Oncology

WE Science Citation Index Expanded (SCI-EXPANDED)

SC Oncology

GA DA8IZ

UT WOS:000368049600021

PM 26718633

OA Bronze

DA 2022-05-19

ER

PT J

AU Zhang, SX

Yang, S

Xu, CQ

Hou, RP

Zhang, CZ

Xu, CP

AF Zhang, Shu-Xiang

Yang, Shan

Xu, Chang-Qing

Hou, Rui-Ping

Zhang, Chuan-Zhen

Xu, Cui-Ping

TI Equivocal Association of RAD51 Polymorphisms with Risk of Esophageal

Squamous Cell Carcinoma in a Chinese Population

SO ASIAN PACIFIC JOURNAL OF CANCER PREVENTION

LA English

DT Article

DE RAD51; esophageal squamous cell carcinoma; genetic polymorphisms

ID DOUBLE-STRAND BREAKS; GASTRIC-CANCER; REPAIR; GENE; INSTABILITY; NECK;

HEAD; P53

AB Aim: To study the contribution of genetic variation in RAD51 to risk of esophageal squamous cell carcinoma (ESCC). Methods: Three single nucleotide polymorphisms (SNPs) in RAD51 (rs1801320, rs4144242 and rs4417527) were genotyped in 316 ESCC patients and 316 healthy controls in Anyang area of China using PCR-RFLP (polymerase chain reaction-restriction fragment length polymorphism). Demographic variables between cases and controls were statistically compared by T test and Chi-square test. Hardy-Weinberg equilibrium was evaluated by the Chi-square test. Odds ratios (ORs) and 95% confidence intervals (CIs) were calculated to measure any association with ESCC. Haplotype frequencies were estimated by Phase 2.1. Result: The genotype frequencies of rs1801320, rs4144242 and rs4417527 in patients with ESCC demonstrated no significant differences from those in control group (P>0.05). When the haplotypes of these three SNPs were constructed and their relationships with ESCC risk investigated, however, CGG was observed to increase the risk (P=0.020, OR=2. 289). Conclusions: There was no association between the three SNPs of RAD51 and ESCC susceptibility in our Chinese population. However, the CGG haplotype might be a risk factor.

C1 [Zhang, Shu-Xiang] Shandong Prov Qianfoshan Hosp, Nursing Dept, Jinan, Peoples R China.

[Yang, Shan] Shandong Prov Qianfoshan Hosp, Dept Ultrasound, Jinan, Peoples R China.

[Xu, Chang-Qing; Hou, Rui-Ping; Zhang, Chuan-Zhen] Shandong Prov Qianfoshan Hosp, Dept Gastroenterol, Jinan, Peoples R China.

[Xu, Cui-Ping] Shandong Prov Qianfoshan Hosp, Dept Human Resources, Jinan, Peoples R China.

RP Xu, CP (corresponding author), Shandong Prov Qianfoshan Hosp, Dept Human Resources, Jinan, Peoples R China.

EM qyxhn@hotmail.com

CR Cheng XL, 2011, HEPATO-GASTROENTEROL, V58, P432

Humar B, 2002, ONCOGENE, V21, P8192, DOI 10.1038/sj.onc.1205921

Khanna KK, 2001, NAT GENET, V27, P247, DOI 10.1038/85798

Kollarova Helena, 2007, Biomedical Papers (Olomouc), V151, P17

Krupa R, 2011, MOL BIOL REP, V38, P2849, DOI 10.1007/s11033-010-0430-6

Lim DS, 1996, MOL CELL BIOL, V16, P7133

Lu JC, 2007, CARCINOGENESIS, V28, P988, DOI 10.1093/carcin/bgl225

Nogueira A, 2012, GENE, V504, P279, DOI 10.1016/j.gene.2012.05.037

O'Driscoll M, 2006, NAT REV GENET, V7, P45, DOI 10.1038/nrg1746

Paques F, 1999, MICROBIOL MOL BIOL R, V63, P349

Parkin DM, 2005, CA-CANCER J CLIN, V55, P74, DOI 10.3322/canjclin.55.2.74

Pasaje CFA, 2011, J MED VIROL, V83, P1892, DOI 10.1002/jmv.22122

Poplawski T, 2006, MUTAT RES-FUND MOL M, V601, P83, DOI 10.1016/j.mrfmmm.2006.06.002

Roberts-Thomson IC, 2005, J GASTROEN HEPATOL, V20, P486, DOI 10.1111/j.1440-1746.2005.03821.x

Romanowicz-Makowska H, 2011, TOHOKU J EXP MED, V224, P201, DOI 10.1620/tjem.224.201

Schild D, 2010, NUCLEIC ACIDS RES, V38, P1061, DOI 10.1093/nar/gkp1063

Shrivastav M, 2008, CELL RES, V18, P134, DOI 10.1038/cr.2007.111

Sliwinski T, 2010, EXP MOL PATHOL, V89, P358, DOI 10.1016/j.yexmp.2010.08.005

Sonoda E, 1998, EMBO J, V17, P598, DOI 10.1093/emboj/17.2.598

Thacker J, 2005, CANCER LETT, V219, P125, DOI 10.1016/j.canlet.2004.08.018

Tsuzuki T, 1996, P NATL ACAD SCI USA, V93, P6236, DOI 10.1073/pnas.93.13.6236

West SC, 2003, NAT REV MOL CELL BIO, V4, P435, DOI 10.1038/nrm1127

NR 22

TC 5

Z9 6

U1 0

U2 0

PU ASIAN PACIFIC ORGANIZATION CANCER PREVENTION

PI GYEONGGI-DO

PA APJCP HEAD OFFICE, KOREAN NATL CANCER CENTER, 323 ILAN -RO,

ILSANDONG-GU, GOYANG-SI, GYEONGGI-DO, 410-769, SOUTH KOREA

SN 1513-7368

J9 ASIAN PAC J CANCER P

JI Asian Pac. J. Cancer Prev.

PY 2014

VL 15

IS 2

BP 763

EP 767

DI 10.7314/APJCP.2014.15.2.763

PG 5

WC Oncology

WE Science Citation Index Expanded (SCI-EXPANDED)

SC Oncology

GA AC4UV

UT WOS:000332517500039

PM 24568492

OA gold

DA 2022-05-19

ER

PT J

AU Fu, HL

Wu, DP

Wang, XF

Wang, JG

Jiao, F

Song, LL

Xie, H

Wen, XY

Shan, HS

Du, YX

Zhao, YP

AF Fu, Hai Long

Wu, De Ping

Wang, Xiu Fang

Wang, Jian Guo

Jiao, Feng

Song, Lei Lei

Xie, Hui

Wen, Xu Yang

Shan, Hu Sheng

Du, Yun Xiang

Zhao, Ya Ping

TI Altered miRNA Expression is Associated with Differentiation, Invasion,

and Metastasis of Esophageal Squamous Cell Carcinoma (ESCC) in Patients

from Huaian, China

SO CELL BIOCHEMISTRY AND BIOPHYSICS

LA English

DT Article

DE Esophageal squamous cell carcinoma; MicroRNA; miRNA-100;

Differentiation; Invasion; Lymph node metastasis; Bioinformatics

analyses

ID FOCAL ADHESION KINASE; MICRORNA EXPRESSION; CANCER; MIR-21; MIR-100;

PROLIFERATION; MIGRATION; SURVIVAL; PROTEIN; APOPTOSIS

AB Esophageal squamous cell carcinoma (ESCC) is the leading malignancy in Huaian, China. Recently, emerging studies have suggested that an aberrant microRNA (miRNA) expression signature exists in ESCC. However, there is discordant information available on specific miRNA expression in patients from different regions. In this study, we identified 12 miRNAs that are differentially expressed in patients with ESCC from Huaian, China. Among these miRNAs that displayed unique miRNA expression signatures, miR-1, miR-29c, miR-100, miR-133a, miR-133b, miR-143, miR-145, and miR-195 were downregulated, and miR-7, miR-21, miR-223, and miR-1246 were upregulated in cancerous tissue compared with the adjacent normal tissue. Bioinformatics analyses identified the major biological processes and signaling pathways that are targeted by these differentially expressed miRNAs. Accordingly, miR-29c, miR-100, miR-133a, and miR-133b were found to be involved in invasion and metastasis of ESCC, and miR-7 and miR-21 were found to be related to the differentiation of ESCC. Thus, our data present new evidence for the important roles of miRNAs in ESCC.

C1 [Fu, Hai Long; Wu, De Ping; Wang, Xiu Fang; Song, Lei Lei; Xie, Hui; Zhao, Ya Ping] 82nd Hosp Peoples Liberat Army, Dept Lab Med, Huaian 223001, Peoples R China.

[Fu, Hai Long; Wang, Xiu Fang; Zhao, Ya Ping] BengBu Med Coll, Dept Postgrad, Bengbu 233030, Peoples R China.

[Wang, Jian Guo; Wen, Xu Yang; Shan, Hu Sheng; Du, Yun Xiang] 82nd Hosp Peoples Liberat Army, Dept Med Oncol, Huaian 223001, Peoples R China.

[Jiao, Feng] 82nd Hosp Peoples Liberat Army, Dept Gen Surg, Huaian 223001, Peoples R China.

RP Zhao, YP (corresponding author), 82nd Hosp Peoples Liberat Army, Dept Lab Med, Huaian 223001, Peoples R China.

EM 549744462@qq.com; zyp82yy@yahoo.com.cn

FU Natural Science Foundation of Jiangsu Province China [BK2012666]

FX The Natural Science Foundation of Jiangsu Province China (BK2012666).

CR Akcakaya P, 2011, INT J ONCOL, V39, P311, DOI 10.3892/ijo.2011.1043

Ambros V, 2004, NATURE, V431, P350, DOI 10.1038/nature02871

Ashburner M, 2000, NAT GENET, V25, P25, DOI 10.1038/75556

Bartel DP, 2004, CELL, V116, P281, DOI 10.1016/S0092-8674(04)00045-5

Bolstad BM, 2003, BIOINFORMATICS, V19, P185, DOI 10.1093/bioinformatics/19.2.185

Calin GA, 2004, P NATL ACAD SCI USA, V101, P2999, DOI 10.1073/pnas.0307323101

Calin GA, 2006, NAT REV CANCER, V6, P857, DOI 10.1038/nrc1997

Chabalier J, 2007, BMC BIOINFORMATICS, V8, DOI 10.1186/1471-2105-8-235

Chou YT, 2010, CANCER RES, V70, P8822, DOI 10.1158/0008-5472.CAN-10-0638

Christodoulou F, 2010, NATURE, V463, P1084, DOI 10.1038/nature08744

Dennis G, 2003, GENOME BIOL, V4, DOI 10.1186/gb-2003-4-9-r60

Feber A, 2008, J THORAC CARDIOV SUR, V135, P255, DOI 10.1016/j.jtcvs.2007.08.055

Ferlay J, 2010, INT J CANCER, V127, P2893, DOI 10.1002/ijc.25516

Gao XL, 2004, BIOPOLYMERS, V73, P579, DOI 10.1002/bip.20005

Guo Y, 2008, CANCER RES, V68, P26, DOI 10.1158/0008-5472.CAN-06-4418

He B, 2012, MOL MED REP, V6, P459, DOI 10.3892/mmr.2012.975

Henson BJ, 2009, GENE CHROMOSOME CANC, V48, P569, DOI 10.1002/gcc.20666

Hiyoshi Y, 2009, CLIN CANCER RES, V15, P1915, DOI 10.1158/1078-0432.CCR-08-2545

Jemal A, 2011, CA-CANCER J CLIN, V61, P69, DOI [10.3322/caac.20107, 10.3322/caac.20115]

Jia XQ, 2012, CELL BIOCHEM BIOPHYS, V62, P237, DOI 10.1007/s12013-011-9263-8

Jung HM, 2012, J BIOL CHEM, V287, P29261, DOI 10.1074/jbc.M112.366518

Kong YW, 2012, LANCET ONCOL, V13, pE249, DOI 10.1016/S1470-2045(12)70073-6

Leite KRM, 2011, UROL ONCOL-SEMIN ORI, V29, P265, DOI 10.1016/j.urolonc.2009.02.002

Lewis BP, 2005, CELL, V120, P15, DOI 10.1016/j.cell.2004.12.035

Li BH, 2011, EUR J CANCER, V47, P2166, DOI 10.1016/j.ejca.2011.04.037

Li HX, 2011, CANCER BIOL THER, V11, P950, DOI 10.4161/cbt.11.11.15531

Li K, 2002, INT J CANCER, V102, P271, DOI [10.1002/ijc.10706, 10.1002/jhc.10706]

Li Q. W., 2012, CHINESE CLIN ONCOLOG, V17, P142

Lioni M, 2008, MOL CANCER THER, V7, P2866, DOI 10.1158/1535-7163.MCT-08-0391

Liu M, 2011, FEBS LETT, V585, P2998, DOI 10.1016/j.febslet.2011.08.014

Mathe EA, 2009, CLIN CANCER RES, V15, P6192, DOI 10.1158/1078-0432.CCR-09-1467

Matsushima K, 2011, J TRANSL MED, V9, DOI 10.1186/1479-5876-9-30

Naganuma S, 2012, AM J CANCER RES, V2, P459

Nagaraja AK, 2010, MOL ENDOCRINOL, V24, P447, DOI 10.1210/me.2009-0295

Ogawa R, 2009, MED MOL MORPHOL, V42, P102, DOI 10.1007/s00795-009-0443-1

Pass HI, 2010, CANCER RES, V70, P1916, DOI 10.1158/0008-5472.CAN-09-3993

Prochnik SE, 2007, DEV GENES EVOL, V217, P73, DOI 10.1007/s00427-006-0116-1

Schwock J, 2010, EXPERT OPIN THER TAR, V14, P77, DOI 10.1517/14728220903460340

Selcuklu SD, 2009, BIOCHEM SOC T, V37, P918, DOI 10.1042/BST0370918

Shi W, 2010, INT J CANCER, V126, P2036, DOI 10.1002/ijc.24880

Smith CM, 2010, WORLD J GASTROENTERO, V16, P531, DOI 10.3748/wjg.v16.i5.531

Sun G, 2007, FOOD ADDIT CONTAM A, V24, P181, DOI 10.1080/02652030601013471

Wang LD, 2010, NAT GENET, V42, P759, DOI 10.1038/ng.648

Wu BL, 2011, WORLD J GASTROENTERO, V17, P79, DOI 10.3748/wjg.v17.i1.79

Wu ZS, 2012, BMC CANCER, V12, DOI 10.1186/1471-2407-12-51

Zhang ZY, 2008, LAB INVEST, V88, P1358, DOI 10.1038/labinvest.2008.94

Zhao X, 2011, ADV DRUG DELIVER REV, V63, P610, DOI 10.1016/j.addr.2010.11.001

Zhu Qi, 2007, V382, P287, DOI 10.1007/978-1-59745-304-2_19

Zhu Q, 2012, ONCOL REP, V27, P1660, DOI 10.3892/or.2012.1682

NR 49

TC 66

Z9 69

U1 1

U2 13

PU HUMANA PRESS INC

PI TOTOWA

PA 999 RIVERVIEW DRIVE SUITE 208, TOTOWA, NJ 07512 USA

SN 1085-9195

EI 1559-0283

J9 CELL BIOCHEM BIOPHYS

JI Cell Biochem. Biophys.

PD NOV

PY 2013

VL 67

IS 2

SI SI

BP 657

EP 668

DI 10.1007/s12013-013-9554-3

PG 12

WC Biochemistry & Molecular Biology; Biophysics; Cell Biology

WE Science Citation Index Expanded (SCI-EXPANDED)

SC Biochemistry & Molecular Biology; Biophysics; Cell Biology

GA 242YP

UT WOS:000326282900043

PM 23516093

DA 2022-05-19

ER

PT J

AU Wang, ZW

Ran, L

Chen, CX

Shi, RR

Dong, Y

Li, YB

Zhou, XM

Qi, YM

Zhu, PP

Gao, YF

Wu, YH

AF Wang, Zhiwei

Ran, Ling

Chen, Chunxia

Shi, Ranran

Dong, Yu

Li, Yubing

Zhou, Xiuman

Qi, Yuanming

Zhu, Pingping

Gao, Yanfeng

Wu, Yahong

TI Identification of HLA-A2-Restricted Mutant Epitopes from Neoantigens of

Esophageal Squamous Cell Carcinoma

SO VACCINES

LA English

DT Article

DE esophageal squamous cell carcinoma; cytotoxic T lymphocytes; mutant

peptide; neoantigen; immunotherapy

ID T-CELLS; CANCER; BLOCKADE; IMMUNOTHERAPY; MELANOMA; VACCINES; IMMUNITY;

PEPTIDE; BURDEN

AB Esophageal squamous cell carcinoma (ESCC), one of the deadliest gastrointestinal cancers, has had limited effective therapeutic strategies up to now. Accumulating evidence suggests that effective immunotherapy in cancer patients has been associated with T cells responsive to mutant peptides derived from neoantigens. Here, we selected 35 human leukocyte antigen-A2 (HLA-A2)-restricted mutant (MUT) peptides stemmed from neoantigens of ESCC. Among them, seven mutant peptides had potent binding affinity to HLA-A*0201 molecules and could form a stable peptide/HLA-A*0201 complex. Three mutant peptides (TP53-R267P, NFE2L2-D13N, and PCLO-E4090Q) of those were immunogenic and could induce the cytotoxic T lymphocytes (CTLs) recognizing mutant peptides presented on transfected cells in an HLA-A2-restricted and MUT peptide-specific manner. In addition, the CTL response in immunized HLA-A2.1/K-b transgenic (Tg) mice was enhanced by coupling MUT peptides to peptide WH, a peptide delivery carrier targeting Clec9a(+) DCs. Then, the possible binding model conversions between the WT and MUT candidate peptides were analyzed by docking with the pockets of HLA-A*0201 molecule. We therefore propose a novel strategy and epitopes for immunotherapy of ESCC based on neoantigens.</p>

C1 [Wang, Zhiwei; Ran, Ling; Chen, Chunxia; Shi, Ranran; Dong, Yu; Li, Yubing; Zhou, Xiuman; Qi, Yuanming; Zhu, Pingping; Wu, Yahong] Zhengzhou Univ, Sch Life Sci, Zhengzhou 450001, Peoples R China.

[Qi, Yuanming; Wu, Yahong] Zhengzhou Univ, Int Joint Lab Prot & Peptide Drugs Henan Prov, Zhengzhou 450001, Peoples R China.

[Zhu, Pingping] Zhengzhou Univ, State Key Lab Esophageal Canc Prevent Treatment, Zhengzhou 450052, Peoples R China.

[Gao, Yanfeng] Sch Pharmaceut Sci Shenzhen, Sun Yat Sen Univ, Shenzhen 510080, Peoples R China.

RP Wu, YH (corresponding author), Zhengzhou Univ, Sch Life Sci, Zhengzhou 450001, Peoples R China.; Wu, YH (corresponding author), Zhengzhou Univ, Int Joint Lab Prot & Peptide Drugs Henan Prov, Zhengzhou 450001, Peoples R China.; Gao, YF (corresponding author), Sch Pharmaceut Sci Shenzhen, Sun Yat Sen Univ, Shenzhen 510080, Peoples R China.

EM 202011161010131@gs.zzu.edu.cn; 18039215962@163.com;

chunxiachen@gs.zzu.edu.cn; srr@gs.zzu.edu.cn; 16637144372@163.com;

liyubing7788@gs.zzu.edu.cn; zhouxm36@mail.sysu.edu.cn; qym@zzu.edu.cn;

zhup@zzu.edu.cn; gaoyf29@mail.sysu.edu.cn; yahongwu@zzu.edu.cn

OI Zhou, Xiuman/0000-0002-5938-450X; yahong, Wu/0000-0001-6381-611X

FU National Natural Science Foundation of China [81822043, 81601448,

U20A20369]

FX This work was supported by the National Natural Science Foundation of

China (81822043, 81601448, U20A20369).

CR Bray F, 2018, CA-CANCER J CLIN, V68, P394, DOI 10.3322/caac.21492

Cafri G, 2020, J CLIN INVEST, V130, P5976, DOI 10.1172/JCI134915

Chen WQ, 2019, LANCET GLOB HEALTH, V7, pE257, DOI 10.1016/S2214-109X(18)30488-1

Chen Y, 2017, CLIN CANCER RES, V23, P6178, DOI 10.1158/1078-0432.CCR-17-1185

Ciemny M, 2018, DRUG DISCOV TODAY, V23, P1530, DOI 10.1016/j.drudis.2018.05.006

Cristescu R, 2018, SCIENCE, V362, P197, DOI 10.1126/science.aar3593

Dotto GP, 2016, CANCER CELL, V29, P622, DOI 10.1016/j.ccell.2016.04.004

Filby A, 2015, METHODS, V82, P29, DOI 10.1016/j.ymeth.2015.02.016

Galluzzi L, 2015, CANCER CELL, V28, P690, DOI 10.1016/j.ccell.2015.10.012

Gao YB, 2014, NAT GENET, V46, P1097, DOI 10.1038/ng.3076

Hoos A, 2016, NAT REV DRUG DISCOV, V15, P235, DOI 10.1038/nrd.2015.35

Ito H, 2015, IMMUNOLOGY, V144, P621, DOI 10.1111/imm.12413

Keskin DB, 2019, NATURE, V565, P234, DOI 10.1038/s41586-018-0792-9

Kuai R, 2017, NAT MATER, V16, P489, DOI [10.1038/NMAT4822, 10.1038/nmat4822]

Lamiable A, 2016, NUCLEIC ACIDS RES, V44, pW449, DOI 10.1093/nar/gkw329

Le DT, 2015, NEW ENGL J MED, V372, P2509, DOI 10.1056/NEJMoa1500596

Lin DC, 2014, NAT GENET, V46, P467, DOI 10.1038/ng.2935

Llosa NJ, 2015, CANCER DISCOV, V5, P43, DOI 10.1158/2159-8290.CD-14-0863

Lu YC, 2014, CLIN CANCER RES, V20, P3401, DOI 10.1158/1078-0432.CCR-14-0433

Mennonna D, 2017, GUT, V66, P454, DOI 10.1136/gutjnl-2015-309453

Nakagawa Y, 2011, BIOMED RES-TOKYO, V32, P159, DOI 10.2220/biomedres.32.159

Pennathur A, 2013, LANCET, V381, P400, DOI 10.1016/S0140-6736(12)60643-6

Plebanski M, 1999, NAT MED, V5, P565, DOI 10.1038/8444

Prickett TD, 2016, CANCER IMMUNOL RES, V4, P669, DOI 10.1158/2326-6066.CIR-15-0215

Restifo NP, 2016, NAT REV CANCER, V16, P121, DOI 10.1038/nrc.2016.2

Rustgi AK, 2014, NEW ENGL J MED, V371, P2499, DOI 10.1056/NEJMra1314530

Sahin U, 2018, SCIENCE, V359, P1355, DOI 10.1126/science.aar7112

Sahin U, 2017, NATURE, V547, P222, DOI 10.1038/nature23003

Sancho D, 2008, J CLIN INVEST, V118, P2098, DOI 10.1172/JCI34584

Schumacher TN, 2015, SCIENCE, V348, P69, DOI 10.1126/science.aaa4971

Shi RR, 2022, SCI CHINA LIFE SCI, V65, P572, DOI 10.1007/s11427-021-1944-5

Siegel RL, 2018, CA-CANCER J CLIN, V68, P7, DOI 10.3322/caac.21442

Topalian SL, 2016, NAT REV CANCER, V16, P275, DOI 10.1038/nrc.2016.36

Tran E, 2015, SCIENCE, V350, P1387, DOI 10.1126/science.aad1253

Tran E, 2014, SCIENCE, V344, P641, DOI 10.1126/science.1251102

van Rossum PSN, 2018, NAT REV GASTRO HEPAT, V15, P235, DOI 10.1038/nrgastro.2017.162

Wu YH, 2018, J IMMUNOL RES, V2018, DOI 10.1155/2018/2942679

Xie XL, 2018, CELL DEATH DIS, V9, DOI 10.1038/s41419-018-0420-5

Xu ZH, 2013, J CONTROL RELEASE, V172, P259, DOI 10.1016/j.jconrel.2013.08.021

Yan ZY, 2016, ONCOTARGET, V7, P40437, DOI 10.18632/oncotarget.9624

Yarchoan M, 2017, NEW ENGL J MED, V377, P2500, DOI 10.1056/NEJMc1713444

Zeng BJ, 2018, J CLIN INVEST, V128, P1971, DOI 10.1172/JCI96791

Zhang L, 2015, AM J HUM GENET, V96, P597, DOI 10.1016/j.ajhg.2015.02.017

Zhu B, 2003, CLIN CANCER RES, V9, P1850

NR 44

TC 0

Z9 0

U1 3

U2 4

PU MDPI

PI BASEL

PA ST ALBAN-ANLAGE 66, CH-4052 BASEL, SWITZERLAND

EI 2076-393X

J9 VACCINES-BASEL

JI Vaccines

PD OCT

PY 2021

VL 9

IS 10

AR 1118

DI 10.3390/vaccines9101118

PG 18

WC Immunology; Medicine, Research & Experimental

WE Science Citation Index Expanded (SCI-EXPANDED)

SC Immunology; Research & Experimental Medicine

GA WN9AU

UT WOS:000712059000001

PM 34696226

OA gold, Green Published

DA 2022-05-19

ER

PT J

AU Xu, YY

Wang, WW

Huang, J

Zhu, WG

AF Xu, Ying-Ying

Wang, Wan-Wei

Huang, Jing

Zhu, Wei-Guo

TI Ellagic acid induces esophageal squamous cell carcinoma cell apoptosis

by modulating SHP-1/STAT3 signaling

SO KAOHSIUNG JOURNAL OF MEDICAL SCIENCES

LA English

DT Article

DE ellagic acid; esophageal squamous cell carcinoma; RNF6; SHP-1; STAT3

ID CANCER; PATHWAY; TARGET; SHP-1

AB Ellagic acid (EA) has been reported to have antiproliferative and antioxidant properties, but its function in esophageal squamous cell carcinoma (ESCC) has not been investigated yet. In the current study, EA was found have a significant anti-tumor activity in ESCC. In specific, EA inhibited ESCC cell survival in both of a concentration- and time-dependent manner. And our results showed that EA promoted ESCC cell apoptosis, including inducing the cleavages of PARP, and inhibiting the expression of anti-apoptotic proteins. In mechanistic, EA markedly suppressed STAT3-driven luciferase activity, and inhibited both of the endogenous and cytokines-induced STAT3 activation in ESCC cells. Further investigations indicated that EA could significantly upregulate SHP-1 expression, a negative modulator of STAT3 signaling. In contrast, knockdown of SHP-1 could attenuate the effects of EA on inhibiting ESCC cell survival. Moreover, we found that EA could inhibit RNF6 expression, an E3 of SHP-1, and overexpressing RNF6 could also significantly attenuate the effects of EA on inhibiting ESCC cell survival, which further revealed that EA could inhibit STAT3 signaling by modulating RNF6/SHP-1 axis. Our present study indicated that EA could be as a novel STAT3 inhibitor for the treatment of ESCC.

C1 [Xu, Ying-Ying; Wang, Wan-Wei; Huang, Jing; Zhu, Wei-Guo] Nanjing Med Univ, Dept Radiat Oncol, Affiliated Huaian 1 Peoples Hosp, 1 Huanghe West Rd, Huaian 223300, Jiangsu, Peoples R China.

RP Zhu, WG (corresponding author), Nanjing Med Univ, Dept Radiat Oncol, Affiliated Huaian 1 Peoples Hosp, 1 Huanghe West Rd, Huaian 223300, Jiangsu, Peoples R China.

EM zhuweiguo_1270@126.com

FU Science and technology development fund of Nanjing Medical University

[NMUB2019350]

FX Science and technology development fund of Nanjing Medical University,

Grant/Award Number: NMUB2019350

CR Abnet CC, 2018, GASTROENTEROLOGY, V154, P360, DOI 10.1053/j.gastro.2017.08.023

Ceci C, 2018, NUTRIENTS, V10, DOI 10.3390/nu10111756

Duan J, 2019, J CELL MOL MED, V23, P143, DOI 10.1111/jcmm.13899

Eskandari E, 2016, J CANCER RES THER, V12, P1266, DOI 10.4103/0973-1482.165873

Eskra JN, 2019, SCI REP-UK, V9, DOI 10.1038/s41598-019-39589-1

Fan LC, 2014, ONCOTARGET, V5, P6243, DOI 10.18632/oncotarget.2191

Hu MH, 2017, ONCOTARGET, V8, P65077, DOI 10.18632/oncotarget.17779

Huang ZM, 2018, ONCOTARGETS THER, V11, P6579, DOI 10.2147/OTT.S174846

Jaman MS, 2018, BREAST CANCER-TOKYO, V25, P517, DOI 10.1007/s12282-018-0866-4

Lei YY, 2018, INT J CLIN EXP PATHO, V11, P3247

Li LW, 2018, EXP THER MED, V16, P29, DOI 10.3892/etm.2018.6182

Li W, 2020, ANAT REC, DOI 10.1002/ar.24363

Liang Q, 2018, CLIN CANCER RES, V24, P1473, DOI 10.1158/1078-0432.CCR-17-2133

Luo HS, 2019, RADIAT ONCOL, V14, DOI 10.1186/s13014-019-1386-x

Rahimi VB, 2020, PHYTOTHER RES, V34, P685, DOI 10.1002/ptr.6565

Tai WP, 2017, MEDICINE, V96, DOI [10.1097/MD.0000000000009325, 10.1097/md.0000000000009325]

Tao JL, 2019, ONCOTARGETS THER, V12, P471, DOI 10.2147/OTT.S193206

Wang TY, 2015, TUMOR BIOL, V36, P9733, DOI 10.1007/s13277-015-3743-1

Xu W, 2018, ONCOL LETT, V16, P898, DOI 10.3892/ol.2018.8712

Xu X, 2016, J BIOL CHEM, V291, P9617, DOI 10.1074/jbc.M115.701979

NR 20

TC 2

Z9 2

U1 1

U2 6

PU WILEY

PI HOBOKEN

PA 111 RIVER ST, HOBOKEN 07030-5774, NJ USA

SN 1607-551X

EI 2410-8650

J9 KAOHSIUNG J MED SCI

JI Kaohsiung J. Med. Sci.

PD SEP

PY 2020

VL 36

IS 9

BP 699

EP 704

DI 10.1002/kjm2.12224

EA MAY 2020

PG 6

WC Medicine, Research & Experimental

WE Science Citation Index Expanded (SCI-EXPANDED)

SC Research & Experimental Medicine

GA NQ9NZ

UT WOS:000530740800001

PM 32374927

OA gold

DA 2022-05-19

ER

PT J

AU Li, XH

Chen, D

Li, MF

Gao, X

Shi, GN

Zhao, H

AF Li, Xiaohui

Chen, Dong

Li, Mengfei

Gao, Xiang

Shi, Gongning

Zhao, Hui

TI Plantamajoside inhibits lipopolysaccharide-induced

epithelial-mesenchymal transition through suppressing the NF-kappa

B/IL-6 signaling in esophageal squamous cell carcinoma cells

SO BIOMEDICINE & PHARMACOTHERAPY

LA English

DT Article

DE Plantamajoside; Epithelial-mesenchymal transition; NF-kappa B; IL-6;

Esophageal squamous cell carcinoma

ID NF-KAPPA-B; CANCER PROGRESSION; TARGETED THERAPY; ACTIVATION;

EXPRESSION; PATHWAYS; RADIOSENSITIVITY; ANGIOGENESIS; MIGRATION; MAPK

AB Plantamajoside (PMS) is a major compound of Plantago asiatica and possesses anti-tumor activity. However, the effect of PMS on esophageal squamous cell carcinoma (ESCC) and the underlying mechanism of action are unclear. The present study aimed to evaluate the effect of PMS on lipopolysaccharide (LPS)-induced epithelial-mesenchymal transition (EMT) in ESCC. The results showed that PMS inhibited viability of ESCC cell lines (Eca109 and TE-1) in a concentration-dependent manner. PMS also inhibited LPS-induced EMT in ESCC cells. PMS inhibited LPS-induced activation of the NF-kappa B pathway and IL-6 expression. PMS also suppressed IL-6-induced EMT in ESCC cells. Treatment of BAY11-7082 (an inhibitor of NF-kappa B) or antibody against IL-6 alleviated the effect of LPS-induced EMT in ESCC cells. Besides, inhibition of NF-kappa B decreased IL-6 expression. In conclusion, the results indicated that PMS inhibited LPS-induced EMT through suppressing the NF-kappa B/IL-6 signaling in ESCC cell lines, suggesting that PMS might be a useful agent for the treatment of ESCC.

C1 [Li, Xiaohui; Chen, Dong; Li, Mengfei; Gao, Xiang; Shi, Gongning; Zhao, Hui] Henan Univ, Huaihe Hosp, Dept Cardiothorac Surg, Baobei Rd 8, Kaifeng 475000, Peoples R China.

RP Zhao, H (corresponding author), Henan Univ, Huaihe Hosp, Dept Cardiothorac Surg, Baobei Rd 8, Kaifeng 475000, Peoples R China.

EM kfzhaohui73@163.com

RI Lee, Kwang-Won/F-2432-2013

OI Lee, Kwang-Won/0000-0001-5326-469X

CR Bollschweiler E, 2017, EXPERT OPIN PHARMACO, V18, P1001, DOI 10.1080/14656566.2017.1334764

Brasier AR, 2006, CARDIOVASC TOXICOL, V6, P111, DOI 10.1385/CT:6:2:111

Chaffer CL, 2016, CANCER METAST REV, V35, P645, DOI 10.1007/s10555-016-9648-7

Chao J, 2017, BIOCHEM PHARMACOL, V139, P94, DOI 10.1016/j.bcp.2017.06.123

Chaudhary AK, 2013, MUTAT RES-REV MUTAT, V753, P7, DOI 10.1016/j.mrrev.2013.01.002

Chen GZ, 2017, J THORAC DIS, V9, P849, DOI 10.21037/jtd.2017.03.23

Chen LJ, 2017, CELL PHYSIOL BIOCHEM, V42, P2267, DOI 10.1159/000480000

Chi XP, 2014, ARCH ORAL BIOL, V59, P954, DOI 10.1016/j.archoralbio.2014.05.022

Cildir G, 2016, TRENDS MOL MED, V22, P414, DOI 10.1016/j.molmed.2016.03.002

Doan D D, 1992, J Ethnopharmacol, V36, P225

de Herreros AG, 2012, BBA-REV CANCER, V1825, P223, DOI 10.1016/j.bbcan.2012.01.003

Gaur Puja, 2014, J Carcinog, V13, P11, DOI 10.4103/1477-3163.143720

Gilmore TD, 2006, ONCOGENE, V25, P6680, DOI 10.1038/sj.onc.1209954

Han AR, 2016, PHOTOCHEM PHOTOBIOL, V92, P708, DOI 10.1111/php.12615

Komoda Y, 1989, Tokyo Ika Shika Daigaku Iyo Kizai Kenkyusho Hokoku, V23, P81

Liang SW, 2017, CANCER LETT, V386, P12, DOI 10.1016/j.canlet.2016.11.003

Mei LL, 2017, INT J MOL SCI, V18, DOI 10.3390/ijms18091833

Paule B, 2007, WORLD J UROL, V25, P477, DOI 10.1007/s00345-007-0175-6

Pei SM, 2015, BMC CANCER, V15, DOI 10.1186/s12885-015-1960-z

Perkins ND, 2007, NAT REV MOL CELL BIO, V8, P49, DOI 10.1038/nrm2083

Ribatti D, 2017, EXP CELL RES, V353, P1, DOI 10.1016/j.yexcr.2017.02.041

Schrump DS, 2005, J SURG ONCOL, V92, P257, DOI 10.1002/jso.20367

Shaheen O, 2017, GASTROENT RES PRACT, V2017, DOI 10.1155/2017/1657310

Singh M, 2018, PHARMACOL THERAPEUT, V182, P80, DOI 10.1016/j.pharmthera.2017.08.009

Son WR, 2017, BMC COMPLEM ALTERN M, V17, DOI 10.1186/s12906-017-1570-1

Song J, 2017, MOL CARCINOGEN, V56, P36, DOI 10.1002/mc.22471

Suarez-Carmona M, 2017, MOL ONCOL, V11, P805, DOI 10.1002/1878-0261.12095

Tai GM, 2015, INT J CLIN EXP PATHO, V8, P4823

Tariq A, 2017, PHYTOTHER RES, V31, P202, DOI 10.1002/ptr.5751

Wang GJ, 2016, EUR J PHARMACOL, V791, P322, DOI 10.1016/j.ejphar.2016.08.029

Wu HC, 2016, INT IMMUNOPHARMACOL, V35, P315, DOI 10.1016/j.intimp.2016.04.013

Xiong SJ, 2017, CELL MOL NEUROBIOL, V37, P1095, DOI 10.1007/s10571-016-0443-3

Zhang JW, 2017, CELL BIOSCI, V7, DOI 10.1186/s13578-017-0173-3

Zhang L, 2016, EXPERT REV GASTROENT, V10, P595, DOI 10.1586/17474124.2016.1140036

Zhao M, 2017, TUMOR BIOL, V39, DOI 10.1177/1010428317691682

Zhu Y, 2015, CELL DEATH DIS, V6, DOI 10.1038/cddis.2015.2

NR 36

TC 13

Z9 16

U1 1

U2 12

PU ELSEVIER FRANCE-EDITIONS SCIENTIFIQUES MEDICALES ELSEVIER

PI ISSY-LES-MOULINEAUX

PA 65 RUE CAMILLE DESMOULINS, CS50083, 92442 ISSY-LES-MOULINEAUX, FRANCE

SN 0753-3322

EI 1950-6007

J9 BIOMED PHARMACOTHER

JI Biomed. Pharmacother.

PD JUN

PY 2018

VL 102

BP 1045

EP 1051

DI 10.1016/j.biopha.2018.03.171

PG 7

WC Medicine, Research & Experimental; Pharmacology & Pharmacy

WE Science Citation Index Expanded (SCI-EXPANDED)

SC Research & Experimental Medicine; Pharmacology & Pharmacy

GA GG3HV

UT WOS:000432583800118

PM 29710521

DA 2022-05-19

ER

PT J

AU Zhang, S

Yang, XJ

Shi, H

Li, M

Xue, Q

Ren, HR

Yao, L

Chen, XY

Zhang, JG

Wang, HJ

AF Zhang, Shu

Yang, Xiaojing

Shi, Hui

Li, Mei

Xue, Qun

Ren, Hanru

Yao, Li

Chen, Xueyu

Zhang, Jianguo

Wang, Huijie

TI Overexpression of leucine aminopeptidase 3 contributes to malignant

development of human esophageal squamous cell carcinoma

SO JOURNAL OF MOLECULAR HISTOLOGY

LA English

DT Article

DE Esophageal squamous cell carcinoma; LAP3; Tumor immunology;

Proliferation; Migration

ID ANGIOTENSIN-II; EXPRESSION; PROLIFERATION; ENZYMOLOGY

AB Leucine aminopeptidases (LAPs) were associated with tumor cell proliferation, invasion and/or angiogenesis. We aimed to examine the biological function of LAP3 in esophageal squamous cell carcinoma (ESCC). LAP3 expressions were examined in human ESCC tissue and cell lines ECA109 and TE1 cells. Recombinant pSilencer4.1-LAP3-shRNA was transfected into ECA109 cells to silence LAP3 expression. The effects of LAP3 silencing on ECA109 cell proliferation in vitro were evaluated. Flow cytometry profiling was used to detect the differentiate cell cycle distribution in LAP3-silenced ECA109 cells. Wound-healing assay and transwell assay were used to examine the activities of migration and invasion in LAP3-silenced ECA109 cells. We overexpressed LAP3 in TE1 cells to find out the corresponding results. LAP3 expression level was abundance in ESCC tissue. LAP3 silencing significantly reduced ECA109 cell proliferation and colony formation. The knockdown of LAP3 resulted in cell cycle arrest at G1-phase. Moreover, over expression of LAP3 favors TE1 cell proliferation and invasiveness which also confirms its contribution in malignant development. We came to the conclusion that LAP3 contributed to ESCC progression by overcoming cell cycle arrest. The proliferative and migration effects of LAP3 might contribute to malignant development of human ESCC.

C1 [Zhang, Shu; Xue, Qun; Ren, Hanru; Zhang, Jianguo] Nantong Univ, Affiliated Hosp, Dept Pathol, Nantong 226001, Jiangsu, Peoples R China.

[Yang, Xiaojing; Li, Mei] Nantong Univ, Affiliated Hosp, Dept Med Oncol, Nantong 226001, Jiangsu, Peoples R China.

[Yang, Xiaojing; Yao, Li] Nantong Univ, Coll Med, Dept Immunol, Nantong 226001, Jiangsu, Peoples R China.

[Shi, Hui] Nantong Univ, Affiliated Hosp, Dept Thorac Surg, Nantong 226001, Jiangsu, Peoples R China.

[Chen, Xueyu] Chinese Acad Sci, Shanghai Inst Mat Med, Shanghai 201203, Peoples R China.

[Wang, Huijie] Fudan Univ, Shanghai Med Coll, Dept Oncol, Shanghai 200032, Peoples R China.

RP Zhang, JG (corresponding author), Nantong Univ, Affiliated Hosp, Dept Pathol, Nantong 226001, Jiangsu, Peoples R China.

EM paper8821@163.com; wanghj98@hotmail.com

OI Xue, Qun/0000-0003-1011-1284

CR Carrera MP, 2006, ANTICANCER RES, V26, P1011

Feng YD, 2011, CELL BIOL INT, V35, P491, DOI 10.1042/CBI20090419

Huang HZ, 2009, ANAL BIOCHEM, V391, P11, DOI 10.1016/j.ab.2009.05.004

Keller SR, 2004, BIOL PHARM BULL, V27, P761, DOI 10.1248/bpb.27.761

KELLER SR, 1995, J BIOL CHEM, V270, P23612, DOI 10.1074/jbc.270.40.23612

Lahmann PH, 2012, CANCER-AM CANCER SOC, V118, P1901, DOI 10.1002/cncr.26455

Mizutani S, 2007, EXPERT OPIN THER TAR, V11, P453, DOI 10.1517/14728222.11.4.453

Radojicic J, 2012, INT J BIOL MARKER, V27, P1, DOI 10.5301/JBM.2011.8737

Shibata K, 2005, TUMOR BIOL, V26, P9, DOI 10.1159/000084181

Suganuma T, 2005, CLIN CANCER RES, V11, P2686, DOI 10.1158/1078-0432.CCR-04-1946

Tamura T, 2012, INT J MED SCI, V9, P833, DOI 10.7150/ijms.4914

Tripathi V, 2013, PLOS GENET, V9, DOI 10.1371/journal.pgen.1003368

Tsujimoto M, 2008, HEART FAIL REV, V13, P285, DOI 10.1007/s10741-007-9064-8

van den Heuvel Sander, 2005, WormBook, P1

Wang F, 2009, J CLIN GASTROENTEROL, V43, P520, DOI 10.1097/MCG.0b013e3181919245

WEBER G, 1983, CANCER RES, V43, P3466

WEBER G, 1977, NEW ENGL J MED, V296, P486, DOI 10.1056/NEJM197703032960905

WEBER G, 1977, NEW ENGL J MED, V296, P541, DOI 10.1056/NEJM197703102961005

Yamazaki T, 2004, BLOOD, V104, P2345, DOI 10.1182/blood-2003-12-4260

Yu CP, 2009, CARCINOGENESIS, V30, P894, DOI 10.1093/carcin/bgp064

Zhang JG, 2013, DIGEST DIS SCI, V58, P2028, DOI 10.1007/s10620-013-2597-x

Zhang L, 2011, WORLD J GASTROENTERO, V17, P4572, DOI 10.3748/wjg.v17.i41.4572

NR 22

TC 28

Z9 28

U1 1

U2 24

PU SPRINGER

PI DORDRECHT

PA VAN GODEWIJCKSTRAAT 30, 3311 GZ DORDRECHT, NETHERLANDS

SN 1567-2379

EI 1567-2387

J9 J MOL HISTOL

JI J. Mol. Histol.

PD JUN

PY 2014

VL 45

IS 3

BP 283

EP 292

DI 10.1007/s10735-014-9566-3

PG 10

WC Cell Biology

WE Science Citation Index Expanded (SCI-EXPANDED)

SC Cell Biology

GA AF0XY

UT WOS:000334439500005

PM 24477662

DA 2022-05-19

ER

PT J

AU Fang, WK

Chen, B

Xu, XE

Liao, LD

Wu, ZY

Wu, JY

Shen, J

Xu, LY

Li, EM

AF Fang, Wang-Kai

Chen, Bo

Xu, Xiu-E

Liao, Lian-Di

Wu, Zhi-Yong

Wu, Jian-Yi

Shen, Jian

Xu, Li-Yan

Li, En-Min

TI Altered expression and localization of desmoglein 3 in esophageal

squamous cell carcinoma

SO ACTA HISTOCHEMICA

LA English

DT Article

DE Desmoglein 3; Esophageal squamous cell carcinoma; Prognosis

ID DESMOSOMAL PROTEINS; PEMPHIGUS-VULGARIS; PROGNOSTIC-FACTOR;

BETA-CATENIN; LUNG-CANCER; SKIN; METASTASIS; ADHESION; CADHERIN; HEAD

AB Desmoglein 3 (DSG3), a transmembrane cadherin of the desmosomal cell-cell adhesion structure, plays vital roles in the maintenance of normal epithelial tissue architecture. Reports implicating a role for DSG3 expression in cancer are few and contradictory. In this study, immunohistochemical staining was employed to investigate DSG3 expression and subcellular localization in esophageal squamous cell carcinoma (ESCC), and to correlate changes with clinical characteristics. Results indicate that in normal squamous cell epithelia, strong DSG3 immunoreactivity was observed in the Stratum spinosum, and localization occurred only at the cell membrane. In ESCC, DSG3 immunoreactivity displayed an abnormal cytoplasmic localization that was correlated with cell differentiation (P = 0.018). Most strikingly, in 74.1% of the tumors, DSG3 expression was up-regulated and correlated with regional lymph node metastasis (P = 0.036). Moreover, in patients without lymph node metastasis, cytoplasmic localization of DSG3 correlated with poor prognosis (P = 0.044). These results suggest that DSG3 is involved in the development of ESCC and imply that DSG3 overexpression is likely to be an essential contributor to the aggressive features of esophageal cancer. (C) 2014 Elsevier GmbH. All rights reserved.

C1 [Fang, Wang-Kai; Wu, Jian-Yi; Li, En-Min] Shantou Univ, Coll Med, Dept Biochem & Mol Biol, Shantou 515041, Peoples R China.

[Fang, Wang-Kai; Xu, Li-Yan; Li, En-Min] Shantou Univ, Coll Med, Key Lab Mol Biol High Canc Incidence Coastal Chao, Shantou 515041, Peoples R China.

[Chen, Bo; Xu, Xiu-E; Liao, Lian-Di; Shen, Jian; Xu, Li-Yan] Shantou Univ, Coll Med, Inst Oncol Pathol, Shantou 515041, Peoples R China.

[Wu, Zhi-Yong] Sun Yat Sen Univ, Affiliated Shantou Hosp, Dept Surg Oncol, Shantou, Peoples R China.

RP Li, EM (corresponding author), Shantou Univ, Coll Med, Dept Biochem & Mol Biol, Key Lab Mol Biol High Canc Incidence Coastal Chao, Shantou 515041, Peoples R China.

EM lyxu@stu.edu.cn; nmli@stu.edu.cn

RI Fang, Wangkai/AAU-8664-2020

OI Fang, Wang-Kai/0000-0002-9417-2422

FU National Natural Science Foundation of China [81101613]; Guangdong

Provincial Natural Science Foundation of China [S2011040004363];

Specialized Research Fund for the Doctoral Program of Higher Education

of China [20114402120005]; Natural Science Foundation of China-Guangdong

Joint Fund [U0932001]

FX This work was supported by grants from the National Natural Science

Foundation of China (No. 81101613), the Guangdong Provincial Natural

Science Foundation of China (No. S2011040004363), the Specialized

Research Fund for the Doctoral Program of Higher Education of China (No.

20114402120005), the Natural Science Foundation of China-Guangdong Joint

Fund (No. U0932001).

CR AMAGAI M, 1991, CELL, V67, P869, DOI 10.1016/0092-8674(91)90360-B

Bosch FX, 2005, INT J CANCER, V114, P779, DOI 10.1002/ijc.20782

Brennan D, 2009, CELL ADHES MIGR, V3, P148, DOI 10.4161/cam.3.2.7539

Brown L, 2013, ONCOGENE

Chambers AF, 2002, NAT REV CANCER, V2, P563, DOI 10.1038/nrc865

Chen YJ, 2007, ONCOGENE, V26, P467, DOI 10.1038/sj.onc.1209802

Chen YJ, 2013, PLOS ONE, V8, DOI 10.1371/journal.pone.0064088

Chidgey M, 2007, BRIT J CANCER, V96, P1783, DOI 10.1038/sj.bjc.6603808

Fang WK, 2013, J PATHOL, V231, P257, DOI 10.1002/path.4236

Fang WK, 2010, HUM PATHOL, V41, P1456, DOI 10.1016/j.humpath.2010.04.003

Fukuoka J, 2007, HUM PATHOL, V38, P276, DOI 10.1016/j.humpath.2006.08.006

Garrod D, 2008, BBA-BIOMEMBRANES, V1778, P572, DOI 10.1016/j.bbamem.2007.07.014

Gomez-Morales M, 2013, HISTOPATHOLOGY, V63, P103, DOI 10.1111/his.12126

Ishii K, 2001, J INVEST DERMATOL, V117, P26, DOI 10.1046/j.0022-202x.2001.01400.x

Kamangar F, 2006, J CLIN ONCOL, V24, P2137, DOI 10.1200/JCO.2005.05.2308

Kottke MD, 2006, J CELL SCI, V119, P797, DOI 10.1242/jcs.02888

Kurzen H, 2003, J CUTAN PATHOL, V30, P621, DOI 10.1034/j.1600-0560.2003.00122.x

Kyrodimou M, 2013, CLIN ORAL INVEST

Myklebust MP, 2012, BRIT J CANCER, V106, P756, DOI 10.1038/bjc.2011.548

Nei H, 1996, CANCER, V78, P461, DOI 10.1002/(SICI)1097-0142(19960801)78:3<461::AID-CNCR13>3.0.CO;2-X

Steeg PS, 2006, NAT MED, V12, P895, DOI 10.1038/nm1469

Teh MT, 2011, J ORAL PATHOL MED, V40, P67, DOI 10.1111/j.1600-0714.2010.00951.x

Tsang SM, 2012, EXP CELL RES, V318, P2269, DOI 10.1016/j.yexcr.2012.07.002

Tsang SM, 2010, PLOS ONE, V5, DOI 10.1371/journal.pone.0014211

Wang LH, 2007, VIRCHOWS ARCH, V451, P959, DOI 10.1007/s00428-007-0485-5

Williamson L, 2006, EMBO J, V25, P3298, DOI 10.1038/sj.emboj.7601224

Yashiro M, 2006, EUR J CANCER, V42, P2397, DOI 10.1016/j.ejca.2006.03.024

NR 27

TC 6

Z9 7

U1 1

U2 6

PU ELSEVIER GMBH, URBAN & FISCHER VERLAG

PI JENA

PA OFFICE JENA, P O BOX 100537, 07705 JENA, GERMANY

SN 0065-1281

EI 1618-0372

J9 ACTA HISTOCHEM

JI Acta Histochem.

PY 2014

VL 116

IS 5

BP 803

EP 809

DI 10.1016/j.acthis.2014.01.010

PG 7

WC Cell Biology

WE Science Citation Index Expanded (SCI-EXPANDED)

SC Cell Biology

GA AM6YM

UT WOS:000340011900022

PM 24630396

DA 2022-05-19

ER

PT J

AU Huang, Y

Xi, QS

Chen, Y

Wang, J

Peng, P

Xia, S

Yu, SY

AF Huang, Yu

Xi, Qingsong

Chen, Yu

Wang, Jing

Peng, Ping

Xia, Shu

Yu, Shiying

TI A dual mTORC1 and mTORC2 inhibitor shows antitumor activity in

esophageal squamous cell carcinoma cells and sensitizes them to

cisplatin

SO ANTI-CANCER DRUGS

LA English

DT Article

DE Akt; antitumor; cisplatin; esophageal squamous cell carcinoma; mTORC1; 2

kinase inhibitor

ID MAMMALIAN TARGET; THERAPEUTIC TARGET; RAPAMYCIN; CANCER; GROWTH;

APOPTOSIS; BREAST; RESISTANCE

AB The mammalian target of rapamycin (mTOR) signaling pathway is critical for the growth and proliferation of various malignant tumors, including esophageal squamous cell carcinoma (ESCC). Therefore, targeting of mTOR protein is a promising strategy for therapy in this disease. In the present study, we examined the antitumor effects of a specific mTOR kinase inhibitor, PP242, which blocks both mTOR complex 1 (mTORC1) and mTOR complex 2 (mTORC2) pathways, in two ESCC cell lines: Eca-109 and TE-1. We showed that PP242, but not rapamycin, attenuated the activities of both mTORC1 and mTORC2 signaling in ESCC. PP242 inhibited 4E-binding protein-1 phosphorylation and abrogated mTORC1-dependent PI3K/Akt feedback activation. Significantly, PP242 effectively suppressed ESCC cell proliferation, induced apoptosis, and arrested the cell cycle. Furthermore, PP242 promoted cisplatin-induced apoptosis and enhanced the antitumor efficacy of cisplatin in ESCC cells, which was likely to be associated with inhibition of Akt activity. Our results show that simultaneous targeting of both mTORC1 and mTORC2 pathways leads to effective antitumor actions in ESCC, and strongly suggest that dual mTORC1/2 inhibitors should be developed as potential agents for the treatment of ESCC.

C1 [Huang, Yu; Xi, Qingsong; Chen, Yu; Wang, Jing; Peng, Ping; Xia, Shu; Yu, Shiying] Huazhong Univ Sci & Technol, Tongji Med Coll, Ctr Canc, Tongji Hosp, Wuhan 430030, Peoples R China.

RP Yu, SY (corresponding author), Huazhong Univ Sci & Technol, Tongji Med Coll, Ctr Canc, Tongji Hosp, Wuhan 430030, Peoples R China.

EM xiashu1900@yahoo.com.cn; syyu@tjh.tjmu.edu.cn

CR Abraham RT, 2008, EXPERT OPIN THER TAR, V12, P209, DOI 10.1517/14728222.12.2.209

Benjamin D, 2011, NAT REV DRUG DISCOV, V10, P868, DOI 10.1038/nrd3531

Beuvink I, 2005, CELL, V120, P747, DOI 10.1016/j.cell.2004.12.040

Boone J, 2008, J CLIN PATHOL, V61, P909, DOI 10.1136/jcp.2008.055772

Buck E, 2006, MOL CANCER THER, V5, P2676, DOI 10.1158/1535-7163.MCT-06-0166

Carayol N, 2010, P NATL ACAD SCI USA, V107, P12469, DOI 10.1073/pnas.1005114107

Choo AY, 2008, P NATL ACAD SCI USA, V105, P17414, DOI 10.1073/pnas.0809136105

Clark AS, 2002, MOL CANCER THER, V1, P707

Dowling RJO, 2010, BBA-PROTEINS PROTEOM, V1804, P433, DOI 10.1016/j.bbapap.2009.12.001

Feldman ME, 2009, PLOS BIOL, V7, P371, DOI 10.1371/journal.pbio.1000038

Garcia-Martinez JM, 2009, BIOCHEM J, V421, P29, DOI 10.1042/BJ20090489

Goldoni M, 2007, TOXICOL IN VITRO, V21, P759, DOI 10.1016/j.tiv.2007.03.003

Gravina GL, 2011, ENDOCR-RELAT CANCER, V18, P385, DOI 10.1530/ERC-11-0045

Guertin DA, 2009, SCI SIGNAL, V2, DOI 10.1126/scisignal.267pe24

Gupta M, 2012, BLOOD, V119, P476, DOI 10.1182/blood-2011-04-346601

Harrington LS, 2004, J CELL BIOL, V166, P213, DOI 10.1083/jcb.200403069

Hirashima K, 2010, ANN SURG ONCOL, V17, P2486, DOI 10.1245/s10434-010-1040-1

Hoang B, 2010, BLOOD, V116, P4560, DOI 10.1182/blood-2010-05-285726

Hou GQ, 2007, CANCER LETT, V253, P236, DOI 10.1016/j.canlet.2007.01.026

Hsu PP, 2011, SCIENCE, V332, P1317, DOI 10.1126/science.1199498

Janes MR, 2010, NAT MED, V16, P205, DOI 10.1038/nm.2091

Jemal A, 2008, CA-CANCER J CLIN, V58, P71, DOI 10.3322/CA.2007.0010

Kim SH, 2013, HUM PATHOL, V44, P226, DOI 10.1016/j.humpath.2012.05.011

Lambert R, 2007, ENDOSCOPY, V39, P659, DOI 10.1055/S-2007-966589

Lambert R, 2007, ENDOSCOPY, V39, P550, DOI 10.1055/s-2007-966530

Laplante M, 2012, CELL, V149, P274, DOI 10.1016/j.cell.2012.03.017

Li HY, 2012, BREAST CANCER RES TR, V134, P1057, DOI 10.1007/s10549-012-2036-2

Liu QS, 2012, J BIOL CHEM, V287, P9742, DOI 10.1074/jbc.M111.304485

Ma XJM, 2009, NAT REV MOL CELL BIO, V10, P307, DOI 10.1038/nrm2672

Marshall G, 2011, BIOCHEM SOC T, V39, P456, DOI 10.1042/BST0390456

Sabatini DM, 2006, NAT REV CANCER, V6, P729, DOI 10.1038/nrc1974

Sakaeda T, 2009, ADV DRUG DELIVER REV, V61, P388, DOI 10.1016/j.addr.2008.10.005

Sancho-Martinez SM, 2012, PHARMACOL THERAPEUT, V136, P35, DOI 10.1016/j.pharmthera.2012.07.003

Siddik ZH, 2003, ONCOGENE, V22, P7265, DOI 10.1038/sj.onc.1206933

Thoreen CC, 2009, J BIOL CHEM, V284, P8023, DOI 10.1074/jbc.M900301200

Toshimitsu H, 2004, CANCER LETT, V211, P69, DOI 10.1016/j.canlet.2004.01.038

Vara JAF, 2004, CANCER TREAT REV, V30, P193, DOI 10.1016/j.ctrv.2003.07.007

Wang ZG, 2010, ONCOL REP, V23, P1167, DOI 10.3892/or_00000747

Weiss RH, 2003, CANCER CELL, V4, P425, DOI 10.1016/S1535-6108(03)00308-8

Willems L, 2012, LEUKEMIA, V26, P1195, DOI 10.1038/leu.2011.339

Yeh CJ, 2011, VIRCHOWS ARCH, V458, P171, DOI 10.1007/s00428-010-0994-5

Yu YH, 2011, SCIENCE, V332, P1322, DOI 10.1126/science.1199484

Zeng ZH, 2012, BLOOD, V120, P2679, DOI 10.1182/blood-2011-11-393934

Zoncu R, 2011, NAT REV MOL CELL BIO, V12, P21, DOI 10.1038/nrm3025

NR 44

TC 22

Z9 23

U1 1

U2 14

PU LIPPINCOTT WILLIAMS & WILKINS

PI PHILADELPHIA

PA TWO COMMERCE SQ, 2001 MARKET ST, PHILADELPHIA, PA 19103 USA

SN 0959-4973

EI 1473-5741

J9 ANTI-CANCER DRUG

JI Anti-Cancer Drugs

PD OCT

PY 2013

VL 24

IS 9

BP 889

EP 898

DI 10.1097/CAD.0b013e328363c64e

PG 10

WC Oncology; Pharmacology & Pharmacy

WE Science Citation Index Expanded (SCI-EXPANDED)

SC Oncology; Pharmacology & Pharmacy

GA 211DM

UT WOS:000323885000003

PM 23838676

DA 2022-05-19

ER

PT J

AU Huo, XD

Huo, B

Wang, HX

Zhang, HD

Ma, Z

Yang, MJ

Wang, HT

Yu, ZT

AF Huo, Xiaodong

Huo, Bin

Wang, Huixing

Zhang, Hongdian

Ma, Zhao

Yang, Mingjian

Wang, Haitao

Yu, Zhentao

TI Prognostic significance of the epithelial-mesenchymal transition factor

zinc finger E-box-binding homeobox 2 in esophageal squamous cell

carcinoma

SO ONCOLOGY LETTERS

LA English

DT Article

DE epithelial-mesenchymal transition; zinc finger E-box-binding homeobox 2;

epithelial cadherin; esophageal squamous cell carcinoma

ID PREDICTS POOR-PROGNOSIS; BETA-CATENIN EXPRESSION; LYMPH-NODE METASTASIS;

E-CADHERIN; GASTRIC-CANCER; TUMOR PROGRESSION; BLADDER-CANCER;

LUNG-CANCER; SIP1; ZEB2

AB Zinc finger E-box-binding homeobox 2 (ZEB2) has been reported to mediate epithelial-mesenchymal transition (EMT) and disease progression in several cancer types. However, the expression of ZEB2 in esophageal squamous cell carcinoma (OSCC) and its association with prognosis remains unclear. In the present study, a tissue microarray and immunohistochemistry were used to investigate ZEB2 and epithelial (E-)cadherin expression in OSCC tissues (n=218) and peritumoral esophageal tissues (POT; n=60). There was a significantly increased incidence of positive ZEB2 expression in OSCC tissues compared with the expresion in POTs (P<0.012). By contrast, the incidence of positive E-cadherin expression in OSCC tissues was significantly decreased compared with the expression in POTs (P<0.004). ZEB2 expression in OSCC was associated with a number of clinicopathological factors, and it was also an independent predictive factor for shorter overall survival time (P<0.001). Overall, ZEB2 may promote OSCC metastasis and is a potential prognostic marker for malignancy.

C1 [Huo, Xiaodong; Zhang, Hongdian; Ma, Zhao; Yang, Mingjian; Yu, Zhentao] Tianjin Med Univ, Canc Inst & Hosp, Key Lab Canc Prevent & Therapy Tianjin, Dept Esophageal Canc, 1 Huanhu West Rd, Tianjin 300060, Peoples R China.

[Huo, Xiaodong; Huo, Bin; Wang, Haitao] Tianjin Med Univ, Hosp 2, Dept Oncol, Tianjin 300211, Peoples R China.

[Wang, Huixing] Tianjin Med Univ, Hosp 2, Pain Management Ctr, Tianjin 300211, Peoples R China.

RP Yu, ZT (corresponding author), Tianjin Med Univ, Canc Inst & Hosp, Key Lab Canc Prevent & Therapy Tianjin, Dept Esophageal Canc, 1 Huanhu West Rd, Tianjin 300060, Peoples R China.

EM yuzhtao@hotmail.com

OI Yu, Zhentao/0000-0002-5785-8492

FU National Natural Science Foundation of Tianjin (Tianjin, China)

[15JCYBJC28400]; Science Foundation of Tianjin Medical University

(Tianjin, China) [2014KYQ27]

FX The present study was supported by the National Natural Science

Foundation of Tianjin (Tianjin, China) (grant no. 15JCYBJC28400) and the

Science Foundation of Tianjin Medical University (Tianjin, China) (grant

no. 2014KYQ27).

CR Beck TN, 2014, PHYSIOL GENOMICS, V46, P699, DOI 10.1152/physiolgenomics.00062.2014

Bhardwaj M, 2015, HUM PATHOL, V46, P1437, DOI 10.1016/j.humpath.2015.05.026

Carneiro P, 2013, EXPERT OPIN THER TAR, V17, P1187, DOI 10.1517/14728222.2013.827174

Chu PY, 2013, ORAL ONCOL, V49, P34, DOI 10.1016/j.oraloncology.2012.07.012

Davalos V, 2012, ONCOGENE, V31, P2062, DOI 10.1038/onc.2011.383

Dong H, 2014, CLIN TRANSL ONCOL, V16, P783, DOI 10.1007/s12094-013-1149-3

Du HF, 2014, CELL SIGNAL, V26, P580, DOI 10.1016/j.cellsig.2013.11.015

Fang WK, 2014, MOL MED REP, V10, P2358, DOI 10.3892/mmr.2014.2485

Fang Y, 2013, PLOS ONE, V8, DOI 10.1371/journal.pone.0062558

Forghanifard MM, 2012, ANN SURG ONCOL, V19, P743, DOI 10.1245/s10434-011-2074-8

Galvan JA, 2015, BRIT J CANCER, V112, P1944, DOI 10.1038/bjc.2015.177

Gamba CO, 2014, RES VET SCI, V97, P554, DOI 10.1016/j.rvsc.2014.09.016

Gong TQ, 2012, CANCER BIOL THER, V13, P606, DOI 10.4161/cbt.19851

Hao XP, 2001, CANCER RES, V61, P8085

Hegarty SV, 2015, PROG NEUROBIOL, V132, P81, DOI 10.1016/j.pneurobio.2015.07.001

Jemal A, 2010, CA-CANCER J CLIN, V60, P277, DOI 10.3322/caac.20073

Jin H, 2010, BIOMED RES-TOKYO, V31, P105, DOI 10.2220/biomedres.31.105

Jolly MK, 2015, FRONT ONCOL, V5, DOI 10.3389/fonc.2015.00155

Jouppila-Matto A, 2015, HISTOL HISTOPATHOL, V30, P569, DOI 10.14670/HH-30.569

Koay MHE, 2012, HISTOPATHOLOGY, V61, P1125, DOI 10.1111/j.1365-2559.2012.04326.x

Kong YH, 2015, PLOS ONE, V10, DOI 10.1371/journal.pone.0134045

Lee KW, 2012, ANN SURG ONCOL, V19, P326, DOI 10.1245/s10434-011-1867-0

Liu TA, 2013, PLOS ONE, V8, DOI 10.1371/journal.pone.0057968

Maeda G, 2005, INT J ONCOL, V27, P1535

Mehendiratta Monica, 2014, J Oral Maxillofac Pathol, V18, P217, DOI 10.4103/0973-029X.140753

Miura N, 2009, ANTICANCER RES, V29, P4099

Miyoshi A, 2004, BRIT J CANCER, V90, P1265, DOI 10.1038/sj.bjc.6601685

Mostaan LV, 2011, PATHOL RES PRACT, V207, P448, DOI 10.1016/j.prp.2011.04.001

Muir AB, 2015, EXP CELL RES, V330, P102, DOI 10.1016/j.yexcr.2014.08.026

National Comprehensive cancer Network. (NCCN), 2015, CLIN PRACT GUID ONC

Niwa Y, 2014, J SURG ONCOL, V110, P764, DOI 10.1002/jso.23694

Okugawa Y, 2013, CLIN EXP METASTAS, V30, P417, DOI 10.1007/s10585-012-9547-4

Peinado H, 2007, NAT REV CANCER, V7, P415, DOI 10.1038/nrc2131

Pennathur A, 2013, LANCET, V381, P400, DOI 10.1016/S0140-6736(12)60643-6

Polyak K, 2009, NAT REV CANCER, V9, P265, DOI 10.1038/nrc2620

Postigo AA, 2003, EMBO J, V22, P2453, DOI 10.1093/emboj/cdg226

Prislei S, 2015, ONCOTARGET, V6, P18966, DOI 10.18632/oncotarget.3943

Rice TW, 2009, DIS ESOPHAGUS, V22, P1, DOI 10.1111/j.1442-2050.2008.00901.x

Sayan AE, 2009, P NATL ACAD SCI USA, V106, P14884, DOI 10.1073/pnas.0902042106

Shapiro IM, 2011, PLOS GENET, V7, DOI 10.1371/journal.pgen.1002218

Song XP, 2015, J MOL HISTOL, V46, P347, DOI 10.1007/s10735-015-9627-2

Song YM, 2014, NATURE, V509, P91, DOI 10.1038/nature13176

Sun XR, 2015, TUMOR BIOL, V36, P7521, DOI 10.1007/s13277-015-3470-7

Torlakovic EE, 2015, J CLIN PATHOL, V68, P879, DOI 10.1136/jclinpath-2014-202705

Wang F, 2015, MED ONCOL, V32, DOI 10.1007/s12032-014-0480-z

Wang NN, 2015, CANCER BIOL THER, V16, P528, DOI 10.1080/15384047.2015.1016687

Wong TS, 2014, BIOMED RES INT, V2014, DOI 10.1155/2014/921564

Wu WH, 2013, INT J NEUROSCI, V123, P318, DOI 10.3109/00207454.2012.758123

Xu YP, 2012, WORLD J SURG ONCOL, V10, DOI 10.1186/1477-7819-10-173

Yoshida R, 2015, ANN SURG ONCOL, V22, P2608, DOI 10.1245/s10434-014-4314-1

Zhao P, 2010, JPN J CLIN ONCOL, V40, P281, DOI 10.1093/jjco/hyp187

NR 51

TC 6

Z9 6

U1 0

U2 2

PU SPANDIDOS PUBL LTD

PI ATHENS

PA POB 18179, ATHENS, 116 10, GREECE

SN 1792-1074

EI 1792-1082

J9 ONCOL LETT

JI Oncol. Lett.

PD SEP

PY 2017

VL 14

IS 3

BP 2683

EP 2690

DI 10.3892/ol.2017.6559

PN A

PG 8

WC Oncology

WE Science Citation Index Expanded (SCI-EXPANDED)

SC Oncology

GA FF0NE

UT WOS:000408597600014

PM 28927031

OA gold, Green Published, Green Submitted

DA 2022-05-19

ER

PT J

AU Zhang, HY

Zheng, XZ

Wang, XH

Xuan, XY

Wang, F

Li, SS

AF Zhang, Hong-Yan

Zheng, Xian-Zhao

Wang, Xin-Hua

Xuan, Xiao-Yan

Wang, Feng

Li, Shan-Shan

TI S100A4 mediated cell invasion and metastasis of esophageal squamous cell

carcinoma via the regulation of MMP-2 and E-cadherin activity

SO MOLECULAR BIOLOGY REPORTS

LA English

DT Article

DE S100A4; Invasion; Metastasis; Esophageal squamous cell carcinoma

ID CALCIUM-BINDING PROTEIN; GASTRIC-CANCER; MTS1 GENE; TRANSCRIPTIONAL

REGULATION; MATRIX METALLOPROTEINASES; OSTEOSARCOMA CELLS;

MOLECULAR-CLONING; BLADDER-CANCER; MESSENGER-RNA; EXPRESSION

AB It is well documented that S100A4 is upregulated in a large amount of invasive tumors and plays a pivotal role in tumor invasion and metastasis. However, the precise role and mechanism S100A4 exerts in the invasion and metastasis of esophageal squamous cell carcinoma (ESCC) have not been fully elucidated to date. Our data demonstrated that S100A4 was overexpressed in human ESCC tissues, especially in ESCC with poor differentiation, deep invasion and lymph node metastasis. Subsequently, the knockdown of S100A4 by RNAi in ESCC cell line (EC-1) could reduce cell invasion, metastasis and proliferation ability in vitro. Most importantly, S100A4 regulated MMP-2 positively and E-cadherin negatively in vivo and in vitro to some extent. Our results suggest that S100A4 is an important factor in the invasion, metastasis and proliferation of ESCC and may control invasion and metastasis at least in part through the regulation of MMP-2 and E-cadherin activity. S100A4 may serve as a biomarker for progression of ESCC and a potential molecular target for biotherapy of ESCC.

C1 [Zhang, Hong-Yan; Wang, Feng; Li, Shan-Shan] Zhengzhou Univ, Dept Pathol, Affiliated Hosp 1, Zhengzhou 450052, Henan Province, Peoples R China.

[Zheng, Xian-Zhao] Peoples Hosp Jiaozuo City, Dept Neurol, Jiaozuo 454002, Henan Province, Peoples R China.

[Wang, Xin-Hua] Zhengzhou Univ, Dept Oncol, Affiliated Hosp 1, Zhengzhou 450052, Henan Province, Peoples R China.

[Xuan, Xiao-Yan] Zhengzhou Univ, Dept Microbiol & Immunol, Coll Basic Med, Zhengzhou 450001, Henan Province, Peoples R China.

RP Li, SS (corresponding author), Zhengzhou Univ, Dept Pathol, Affiliated Hosp 1, 40 Daxue Rd, Zhengzhou 450052, Henan Province, Peoples R China.

EM lsspath@yahoo.com.cn

CR Andersen H, 2005, MOL CELL BIOL, V25, P9138, DOI 10.1128/MCB.25.20.9138-9150.2005

BARRACLOUGH R, 1987, J MOL BIOL, V198, P13, DOI 10.1016/0022-2836(87)90453-0

Barraclough R, 1998, BBA-MOL CELL RES, V1448, P190, DOI 10.1016/S0167-4889(98)00143-8

Bjornland K, 1999, CANCER RES, V59, P4702

Chen Xiang-Li, 2006, Ai Zheng, V25, P1134

Cho YG, 2003, APMIS, V111, P539, DOI 10.1034/j.1600-0463.2003.1110502.x

Conacci-Sorrell M, 2002, J CLIN INVEST, V109, P987, DOI 10.1172/JCI200215429

DAVIES BR, 1993, ONCOGENE, V8, P999

Donato R, 2001, INT J BIOCHEM CELL B, V33, P637, DOI 10.1016/S1357-2725(01)00046-2

EBRALIDZE A, 1989, GENE DEV, V3, P1086, DOI 10.1101/gad.3.7.1086

ENGELKAMP D, 1992, BIOCHEMISTRY-US, V31, P10258, DOI 10.1021/bi00157a012

FORD HL, 1995, ONCOGENE, V11, P2067

Garrett SC, 2006, J BIOL CHEM, V281, P677, DOI 10.1074/jbc.R500017200

GOTO K, 1988, J BIOCHEM-TOKYO, V103, P48, DOI 10.1093/oxfordjournals.jbchem.a122237

Grigorian M, 2001, J BIOL CHEM, V276, P22699, DOI 10.1074/jbc.M010231200

Grum-Schwensen B, 2005, CANCER RES, V65, P3772, DOI 10.1158/0008-5472.CAN-04-4510

JACKSONGRUSBY LL, 1987, NUCLEIC ACIDS RES, V15, P6677, DOI 10.1093/nar/15.16.6677

Keirsebilck A, 1998, CANCER RES, V58, P4587

Kim JH, 2009, ONCOL REP, V22, P41, DOI 10.3892/or_00000404

Kimura K, 2000, INT J ONCOL, V16, P1125

Levett D, 2002, AM J PATHOL, V160, P693, DOI 10.1016/S0002-9440(10)64889-4

Li S, 2010, ONCOL REP, V24, P997, DOI 10.3892/or_00000947

Li Y, 2010, MOL BIOL REP, V37, P197, DOI 10.1007/s11033-009-9593-4

LIOTTA LA, 1991, CELL, V64, P327, DOI 10.1016/0092-8674(91)90642-C

MASIAKOWSKI P, 1988, P NATL ACAD SCI USA, V85, P1277, DOI 10.1073/pnas.85.4.1277

Mathisen B, 2003, CLIN EXP METASTAS, V20, P701, DOI 10.1023/B:CLIN.0000006819.21361.03

Matsumoto K, 2007, UROLOGY, V70, P602, DOI 10.1016/j.urology.2007.04.007

Mazzucchelli L, 2002, AM J PATHOL, V160, P7, DOI 10.1016/S0002-9440(10)64342-8

Moriyama-Kita M, 2004, ORAL ONCOL, V40, P496, DOI 10.1016/j.oraloncology.2003.10.003

Nakamura N, 1998, CLIN EXP METASTAS, V16, P471

Ninomiya I, 2001, INT J ONCOL, V18, P715

Oida Y, 2006, ONCOL REP, V16, P457

PARKER C, 1994, DNA CELL BIOL, V13, P343, DOI 10.1089/dna.1994.13.343

Parkin DM, 2005, CA-CANCER J CLIN, V55, P74, DOI 10.3322/canjclin.55.2.74

Saleem M, 2006, P NATL ACAD SCI USA, V103, P14825, DOI 10.1073/pnas.0606747103

Schafer BW, 1996, TRENDS BIOCHEM SCI, V21, P134, DOI 10.1016/0968-0004(96)10020-7

Schmidt-Hansen B, 2004, ONCOGENE, V23, P5487, DOI 10.1038/sj.onc.1207720

Sengupta N, 2007, PHYSIOLOGY, V22, P401, DOI 10.1152/physiol.00027.2007

Sherbet GV, 1998, ANTICANCER RES, V18, P2415

STRUTZ F, 1995, J CELL BIOL, V130, P393, DOI 10.1083/jcb.130.2.393

TAKEICHI M, 1995, CURR OPIN CELL BIOL, V7, P619, DOI 10.1016/0955-0674(95)80102-2

TULCHINSKY E, 1995, BBA-GENE STRUCT EXPR, V1261, P243, DOI 10.1016/0167-4781(95)00013-7

WATANABE Y, 1992, ARCH BIOCHEM BIOPHYS, V292, P563, DOI 10.1016/0003-9861(92)90031-Q

Wu CY, 2007, EUR J CANCER, V43, P799, DOI 10.1016/j.ejca.2006.10.022

Yonemura Y, 2000, CLIN CANCER RES, V6, P4234

Yoon CS, 2008, HEPATO-GASTROENTEROL, V55, P1916

Zhang Hong-yan, 2010, Nan Fang Yi Ke Da Xue Xue Bao, V30, P1541

Zou M, 2005, BRIT J CANCER, V93, P1277, DOI 10.1038/sj.bjc.6602856

NR 48

TC 34

Z9 34

U1 0

U2 4

PU SPRINGER

PI DORDRECHT

PA VAN GODEWIJCKSTRAAT 30, 3311 GZ DORDRECHT, NETHERLANDS

SN 0301-4851

EI 1573-4978

J9 MOL BIOL REP

JI Mol. Biol. Rep.

PD JAN

PY 2012

VL 39

IS 1

BP 199

EP 208

DI 10.1007/s11033-011-0726-1

PG 10

WC Biochemistry & Molecular Biology

WE Science Citation Index Expanded (SCI-EXPANDED)

SC Biochemistry & Molecular Biology

GA 852KC

UT WOS:000297355700025

PM 21603862

DA 2022-05-19

ER

PT J

AU Fan, NB

Yang, H

Zheng, JB

Chen, DN

Wang, WD

Tan, ZH

Huang, YH

Lin, P

AF Fan, Ningbo

Yang, Han

Zheng, Jiabo

Chen, Dongni

Wang, Weidong

Tan, Zihui

Huang, Yuanheng

Lin, Peng

TI Comparison of short- and long-term outcomes between 3-field and modern

2-field lymph node dissections for thoracic oesophageal squamous cell

carcinoma: a propensity score matching analysis

SO INTERACTIVE CARDIOVASCULAR AND THORACIC SURGERY

LA English

DT Article

DE Oesophageal neoplasms; Neck lymph node excision; Thoracic surgery

ID PREDICTS SURVIVAL; CANCER; PATTERN; LYMPHADENECTOMY; METASTASIS;

RESECTION; NUMBER; IMPACT

AB OBJECTIVES: Our goal was to compare short- and long-term outcomes between 3-field lymphadenectomy (3-FL) and modern 2-field lymphadenectomy (2-FL) in patients with thoracic oesophageal squamous cell carcinoma.

METHODS: We reviewed clinical outcomes for 298 patients with thoracic oesophageal squamous cell carcinoma who underwent 3-FL or modern 2-FL from March 2008 to December 2013 at a major cancer hospital in Guangzhou, southern China. Propensity score matching was used to balance baseline differences, and 83 pairs of cases were selected. Postoperative complications, recurrence patterns and survival outcomes were compared between the 2 groups.

RESULTS: Compared with modern 2-FL, 3-FL led to higher overall operative morbidity rates [78.3% vs 61.4%, odds ratio (OR) 2.266, 95% confidence interval (CI) 1.143-4.490; P = 0.019], with higher recurrent nerve palsy rates (47.0% vs 19.3%, OR 3.712, 95% CI 1.852-7.438; P < 0.0001), more respiratory failures (18.1% vs 6.0%, OR 3.441, 95% CI 1.189-9.963; P = 0.023) and longer postoperative hospital stays (23 vs 17 days, P = 0.002). The 5-year overall survival rate (58.5% vs 59.4%; P = 0.960) and the 5-year disease-free survival rate 50.1% vs 54.5%; P = 0.482) were comparable between the 2 groups. Multivariable analysis showed that additional cervical lymph node dissection was not associated with overall survival [hazard ratio (HR) 1.039, 95% CI 0.637-1.696; P = 0.878] and disease-free survival (HR 0.868, 95% CI 0.548-1.376; P = 0.547). The overall recurrence rate and cervical nodal recurrence rate were not significantly different between the 2 groups.

CONCLUSIONS: Additional cervical lymphadenectomy did not lead to added survival benefit when compared with modern 2-FL in patients with thoracic oesophageal squamous cell carcinoma. Recurrence was similar in patients undergoing 3-FL and modern 2-FL. 3-FL resulted in more postoperative complications.

C1 [Fan, Ningbo; Yang, Han; Chen, Dongni; Wang, Weidong; Tan, Zihui; Huang, Yuanheng; Lin, Peng] Sun Yat Sen Univ, Ctr Canc, Collaborat Innovat Ctr Canc Med, Dept Thorac Oncol,State Key Lab Oncol South China, 651 Dongfeng East Rd, Guangzhou 510060, Guangdong, Peoples R China.

[Zheng, Jiabo] Sun Yat Sen Univ, Dept Gastrointestinal Surg, Affiliated Hosp 1, Guangzhou, Guangdong, Peoples R China.

RP Lin, P (corresponding author), Sun Yat Sen Univ, Ctr Canc, Collaborat Innovat Ctr Canc Med, Dept Thorac Oncol,State Key Lab Oncol South China, 651 Dongfeng East Rd, Guangzhou 510060, Guangdong, Peoples R China.

EM linpeng@sysucc.org.cn

OI Fan, Ningbo/0000-0002-1622-6747

FU '5010 plan' from Sun Yat-sen University [2007044]

FX This work was supported by the '5010 plan' from Sun Yat-sen University

[grant 2007044].

CR AKIYAMA H, 1994, ANN SURG, V220, P364, DOI 10.1097/00000658-199409000-00012

Altorki N, 2002, ANN SURG, V236, P177, DOI 10.1097/00000658-200208000-00005

Altorki NK, 2008, ANN SURG, V248, P221, DOI 10.1097/SLA.0b013e31817bbe59

Altorki NK, 1997, J THORAC CARDIOV SUR, V113, P540, DOI 10.1016/S0022-5223(97)70368-4

Baba M, 1998, Dis Esophagus, V11, P28, DOI 10.1093/dote/11.1.28

Bumm R, 1995, DIS ESOPHAGUS, V8, P78

Chen WQ, 2016, CA-CANCER J CLIN, V66, P115, DOI 10.3322/caac.21338

Dresner SM, 2000, BRIT J SURG, V87, P1426, DOI 10.1046/j.1365-2168.2000.01541.x

Ferlay J, 2015, INT J CANCER, V136, pE359, DOI 10.1002/ijc.29210

FUJITA H, 1995, ANN SURG, V222, P654, DOI 10.1097/00000658-199511000-00008

Igaki H, 2004, ANN SURG, V239, P483, DOI 10.1097/01.sla.0000118562.97742.29

ISONO K, 1991, ONCOLOGY, V48, P411

KATO H, 1991, ANN THORAC SURG, V51, P931, DOI 10.1016/0003-4975(91)91008-J

Kosugi S, 2013, ANN SURG ONCOL, V20, P4016, DOI 10.1245/s10434-013-3141-0

Kuge K, 2003, J THORAC CARDIOV SUR, V125, P1343, DOI 10.1016/S0022-5223(03)00036-9

Lin Dong, 2016, Zhonghua Wei Chang Wai Ke Za Zhi, V19, P990

Ma GW, 2014, WORLD J GASTROENTERO, V20, P18022, DOI 10.3748/wjg.v20.i47.18022

Mariette C, 2003, CANCER-AM CANCER SOC, V97, P1616, DOI 10.1002/cncr.11228

Nakamura M, 2007, SURG TODAY, V37, P379, DOI 10.1007/s00595-006-3413-4

National Cancer Center, 2018, CHIN CANC REG ANN RE

Nishihira T, 1998, AM J SURG, V175, P47, DOI 10.1016/S0002-9610(97)00227-4

Peyre CG, 2008, ANN SURG, V248, P549, DOI 10.1097/SLA.0b013e318188c474

Shim YM, 2010, J THORAC ONCOL, V5, P707, DOI 10.1097/JTO.0b013e3181d3ccb2

Tan ZH, 2014, J GASTROINTEST SURG, V18, P187, DOI 10.1007/s11605-013-2411-2

Ye T, 2013, ANN THORAC SURG, V96, P1933, DOI 10.1016/j.athoracsur.2013.06.050

NR 25

TC 2

Z9 2

U1 0

U2 0

PU OXFORD UNIV PRESS

PI OXFORD

PA GREAT CLARENDON ST, OXFORD OX2 6DP, ENGLAND

SN 1569-9293

EI 1569-9285

J9 INTERACT CARDIOV TH

JI Interact Cardiovasc. Thorac. Surg.

PD SEP

PY 2019

VL 29

IS 3

BP 434

EP 441

DI 10.1093/icvts/ivz108

PG 8

WC Cardiac & Cardiovascular Systems; Respiratory System; Surgery

WE Science Citation Index Expanded (SCI-EXPANDED)

SC Cardiovascular System & Cardiology; Respiratory System; Surgery

GA JI2KJ

UT WOS:000493292400016

PM 31135037

OA Bronze

DA 2022-05-19

ER

PT J

AU Zeng, RJ

Zheng, CW

Gu, JE

Zhang, HX

Xie, L

Xu, LY

Li, EM

AF Zeng, Rui-Jie

Zheng, Chun-Wen

Gu, Jing-E

Zhang, Hai-Xia

Xie, Lei

Xu, Li-Yan

Li, En-Min

TI RAC1 inhibition reverses cisplatin resistance in esophageal squamous

cell carcinoma and induces downregulation of glycolytic enzymes

SO MOLECULAR ONCOLOGY

LA English

DT Article

DE chemoresistance; cisplatin; esophageal squamous cell carcinoma;

glycolysis; RAC1

ID LUNG-CANCER; RATIONAL DESIGN; ACTIVATION; MTOR; AKT; PROGRESSION;

EXPRESSION; MIGRATION; INVASION; PHOSPHORYLATION

AB Development of chemoresistance remains a major challenge in treating esophageal squamous cell carcinoma (ESCC) patients despite treatment advances. However, the role of RAC1 in chemoresistance of ESCC and the underlying mechanisms remain largely unknown. In this study, we found that higher levels of RAC1 expression were associated with poorer prognosis in ESCC patients. Enhanced RAC1 expression increased cell proliferation, migration, and chemoresistance in vitro. Combination therapy using RAC1 inhibitor EHop-016 and cisplatin significantly promoted cell viability inhibition, G2/M phase cycle arrest, and apoptosis when compared to each monotherapy. Mechanistically, glycolysis was significantly downregulated in the RAC1 inhibitor monotherapy group and the combination group via inhibiting AKT/FOXO3a signaling when compared to the control group. Moreover, the silencing of RAC1 inhibited AKT/FOXO3a signaling and cell glycolysis while the upregulation of RAC1 produced an opposite effect. In murine xenograft models, the tumor volume and the expression of glycolytic enzymes were significantly reduced in combination therapy when compared to each monotherapy group. Overall, our study demonstrates that targeting RAC1 with an inhibitor overcomes cisplatin resistance in ESCC by suppressing glycolytic enzymes, which provides a promising strategy for treatment of ESCC in clinical practice.

C1 [Zeng, Rui-Jie; Zheng, Chun-Wen; Gu, Jing-E; Zhang, Hai-Xia; Xie, Lei; Li, En-Min] Shantou Univ, Med Coll, Dept Biochem & Mol Biol, Shantou 515041, Peoples R China.

[Zeng, Rui-Jie; Zheng, Chun-Wen; Gu, Jing-E; Zhang, Hai-Xia; Xie, Lei; Xu, Li-Yan; Li, En-Min] Shantou Univ, Med Coll, Key Lab Mol Biol High Canc Incidence Coastal Chao, Shantou, Peoples R China.

[Xu, Li-Yan] Shantou Univ, Med Coll, Inst Oncol Pathol, Shantou 515041, Peoples R China.

RP Li, EM (corresponding author), Shantou Univ, Med Coll, Dept Biochem & Mol Biol, Shantou 515041, Peoples R China.; Xu, LY (corresponding author), Shantou Univ, Med Coll, Inst Oncol Pathol, Shantou 515041, Peoples R China.

EM lyxu@stu.edu.cn; nmli@stu.edu.cn

FU Natural Science Foundation of China-Guangdong Joint Fund [U1601229];

Special Funds for the Cultivation of Guangdong College Students'

Scientific and Technological Innovation ('Climbing Program' Special

Funds) [pdjh2019a0182]; National Undergraduate Training Program for

Innovation and Entrepreneurship [201810560037]

FX This work was supported by the Natural Science Foundation of

China-Guangdong Joint Fund (U1601229), the Special Funds for the

Cultivation of Guangdong College Students' Scientific and Technological

Innovation ('Climbing Program' Special Funds, pdjh2019a0182), and the

National Undergraduate Training Program for Innovation and

Entrepreneurship (201810560037). The authors thank Liu Peng, Feng Pan,

Yang Chen, Xiu-E Xu, and Hui-Xiang Zhang for technical support. The

authors thank Dr Shao-Hong Wang and Dr Zhi-Yong Wu for kindly offering

the patient samples and clinical data.

CR Abnet CC, 2018, GASTROENTEROLOGY, V154, P360, DOI 10.1053/j.gastro.2017.08.023

Alfarouk KO, 2015, CANCER CELL INT, V15, DOI 10.1186/s12935-015-0221-1

Allum WH, 2014, ANN NY ACAD SCI, V1325, P242, DOI 10.1111/nyas.12533

Anders Simon, 2010, Genome Biol, V11, pR106, DOI 10.1186/gb-2010-11-10-r106

Ashburner M, 2000, NAT GENET, V25, P25, DOI 10.1038/75556

Baugher PJ, 2005, BREAST CANCER RES, V7, pR965, DOI 10.1186/bcr1329

Bhattacharya B, 2016, BRIT J PHARMACOL, V173, P970, DOI 10.1111/bph.13422

Bid HK, 2013, MOL CANCER THER, V12, P1925, DOI 10.1158/1535-7163.MCT-13-0164

Cardama GA, 2017, INT J ONCOL, V51, P1025, DOI 10.3892/ijo.2017.4093

Cardama GA, 2014, ANTI-CANCER AGENT ME, V14, P840, DOI 10.2174/18715206113136660334

Cerella C, 2014, BIOCHEM PHARMACOL, V92, P22, DOI 10.1016/j.bcp.2014.07.005

Chandarlapaty S, 2012, CANCER DISCOV, V2, P311, DOI 10.1158/2159-8290.CD-12-0018

Chen QY, 2011, INT J MOL MED, V28, P769, DOI 10.3892/ijmm.2011.775

D'Amico Thomas A, 2007, Gastrointest Cancer Res, V1, P188

Devic S, 2016, J CANCER, V7, P817, DOI 10.7150/jca.14274

Dutting S, 2015, J THROMB HAEMOST, V13, P827, DOI 10.1111/jth.12861

Eljack ND, 2014, METALLOMICS, V6, P2126, DOI 10.1039/c4mt00238e

Engers R, 2001, J BIOL CHEM, V276, P41889, DOI 10.1074/jbc.M105049200

Esen E, 2013, CELL METAB, V17, P745, DOI 10.1016/j.cmet.2013.03.017

Faes S, 2015, INT J MOL SCI, V16, P21138, DOI 10.3390/ijms160921138

Ferri N, 2009, J MED CHEM, V52, P4087, DOI 10.1021/jm8015987

Foucquier J, 2015, PHARMACOL RES PERSPE, V3, DOI 10.1002/prp2.149

Frances D, 2015, ONCOGENE, V34, P5505, DOI 10.1038/onc.2014.471

Gao Y, 2004, P NATL ACAD SCI USA, V101, P7618, DOI 10.1073/pnas.0307512101

Gastonguay AJ, 2012, CANCER BIOL THER, V13, P647, DOI 10.4161/cbt.20082

Godel M, 2011, J CLIN INVEST, V121, P2197, DOI 10.1172/JCI44774

Hresko RC, 2005, J BIOL CHEM, V280, P40406, DOI 10.1074/jbc.M508361200

Huang DW, 2009, NUCLEIC ACIDS RES, V37, P1, DOI 10.1093/nar/gkn923

Humphries-Bickley T, 2017, MOL CANCER THER, V16, P805, DOI 10.1158/1535-7163.MCT-16-0442

Jacinto E, 2006, CELL, V127, P125, DOI 10.1016/j.cell.2006.08.033

Jemal A, 2011, CA-CANCER J CLIN, V61, P69, DOI [10.3322/caac.20107, 10.3322/caac.20115]

Jiang Y, 2018, NAT COMMUN, V9, DOI 10.1038/s41467-018-06081-9

Kamai T, 2010, BMC CANCER, V10, DOI 10.1186/1471-2407-10-164

Khatri S, 2010, J BIOL CHEM, V285, P15960, DOI 10.1074/jbc.M110.121871

Kihara C, 2001, CANCER RES, V61, P6474

Kwon T, 2000, J BIOL CHEM, V275, P423, DOI 10.1074/jbc.275.1.423

Levay M, 2013, J PHARMACOL EXP THER, V347, P69, DOI 10.1124/jpet.113.207266

Li CQ, 2017, ONCOGENESIS, V6, DOI 10.1038/oncsis.2017.1

Li LY, 2015, ONCOTARGET, V6, P15940, DOI 10.18632/oncotarget.3612

Li LY, 2015, J PROTEOMICS, V117, P145, DOI 10.1016/j.jprot.2015.01.016

Li XB, 2015, THORAC CANCER, V6, P17, DOI 10.1111/1759-7714.12148

Liberti MV, 2016, TRENDS BIOCHEM SCI, V41, P211, DOI 10.1016/j.tibs.2015.12.001

Long F, 2014, ONCOL REP, V32, P2031, DOI 10.3892/or.2014.3473

Ma XJM, 2009, NAT REV MOL CELL BIO, V10, P307, DOI 10.1038/nrm2672

Malliri A, 2002, NATURE, V417, P867, DOI 10.1038/nature00848

Montalvo-Ortiz BL, 2012, J BIOL CHEM, V287, P13228, DOI 10.1074/jbc.M111.334524

Murga C, 2002, ONCOGENE, V21, P207, DOI 10.1038/sj.onc.1205036

Myant KB, 2013, CELL STEM CELL, V12, P761, DOI 10.1016/j.stem.2013.04.006

Nitulescu GM, 2016, INT J ONCOL, V48, P869, DOI 10.3892/ijo.2015.3306

PEREZ RP, 1990, PHARMACOL THERAPEUT, V48, P19, DOI 10.1016/0163-7258(90)90015-T

Ponten F, 2011, J INTERN MED, V270, P428, DOI 10.1111/j.1365-2796.2011.02427.x

Riganti C, 2015, FRONT ONCOL, V5, DOI 10.3389/fonc.2015.00105

Robey RB, 2009, SEMIN CANCER BIOL, V19, P25, DOI 10.1016/j.semcancer.2008.11.010

Saci A, 2011, MOL CELL, V42, P50, DOI 10.1016/j.molcel.2011.03.017

Sakai NS, 2013, SEMIN CANCER BIOL, V23, P512, DOI 10.1016/j.semcancer.2013.08.005

Sarbassov DD, 2005, SCIENCE, V307, P1098, DOI 10.1126/science.1106148

Schilsky RL, 2010, NAT REV DRUG DISCOV, V9, P363, DOI 10.1038/nrd3181

Shen DW, 2004, MOL PHARMACOL, V66, P789, DOI 10.1124/mol.66.4.789

Sirover MA, 2012, J CELL BIOCHEM, V113, P2193, DOI 10.1002/jcb.24113

Skvortsov S, 2014, BRIT J CANCER, V110, P2677, DOI 10.1038/bjc.2014.221

Slinker BK, 1998, J MOL CELL CARDIOL, V30, P723, DOI 10.1006/jmcc.1998.0655

Song Q, 2017, J CANCER, V8, P2492, DOI 10.7150/jca.19601

Takashima A, 2008, DIS ESOPHAGUS, V21, P607, DOI 10.1111/j.1442-2050.2008.00821.x

Taniguchi K, 2015, BBA-MOL BASIS DIS, V1852, P1971, DOI 10.1016/j.bbadis.2015.06.022

Tristan C, 2011, CELL SIGNAL, V23, P317, DOI 10.1016/j.cellsig.2010.08.003

Ucker DS, 2012, J BIOL CHEM, V287, P10325, DOI 10.1074/jbc.M111.314971

Um K, 2014, DEV CELL, V29, P701, DOI 10.1016/j.devcel.2014.05.011

Wang CH, 2015, NEOPLASMA, V62, P541, DOI 10.4149/neo_2015_065

Wang J, 2014, ASIAN PAC J CANCER P, V15, P1803, DOI 10.7314/APJCP.2014.15.4.1803

Wu ZY, 2013, J HISTOCHEM CYTOCHEM, V61, P340, DOI 10.1369/0022155413480181

Xie H, 2014, CELL METAB, V19, P795, DOI 10.1016/j.cmet.2014.03.003

Yu L, 2014, CANCER LETT, V355, P34, DOI 10.1016/j.canlet.2014.09.020

Zhang FR, 2008, J HISTOCHEM CYTOCHEM, V56, P193, DOI 10.1369/jhc.7A7353.2007

Zhang HF, 2016, CLIN CANCER RES, V22, P691, DOI 10.1158/1078-0432.CCR-15-1253

Zhu G, 2015, ONCOGENE, V34, P5971, DOI 10.1038/onc.2015.45

Zhu L, 2013, B CANC, V100, pE15

Zins K, 2013, PLOS ONE, V8, DOI 10.1371/journal.pone.0074924

Zou HY, 2016, INT J BIOCHEM CELL B, V75, P85, DOI 10.1016/j.biocel.2016.04.003

NR 78

TC 23

Z9 23

U1 1

U2 8

PU WILEY

PI HOBOKEN

PA 111 RIVER ST, HOBOKEN 07030-5774, NJ USA

SN 1574-7891

EI 1878-0261

J9 MOL ONCOL

JI Mol. Oncol.

PD SEP

PY 2019

VL 13

IS 9

BP 2010

EP 2030

DI 10.1002/1878-0261.12548

EA JUL 2019

PG 21

WC Oncology

WE Science Citation Index Expanded (SCI-EXPANDED)

SC Oncology

GA IU6IK

UT WOS:000478162700001

PM 31314174

OA Green Published, gold

DA 2022-05-19

ER

PT J

AU Geng, YT

Zhu, DX

Wu, C

Wu, J

Wang, Q

Li, RQ

Jiang, JT

Wu, CP

AF Geng, Yiting

Zhu, Danxia

Wu, Chen

Wu, Jun

Wang, Qi

Li, Ruiqi

Jiang, Jingting

Wu, Changping

TI A novel systemic inflammation response index (SIRI) for predicting

postoperative survival of patients with esophageal squamous cell

carcinoma

SO INTERNATIONAL IMMUNOPHARMACOLOGY

LA English

DT Article

DE Systemic inflammation response index; Esophageal squamous cell

carcinoma; Prognosis

ID LYMPHOCYTE RATIO; CANCER; NEUTROPHIL; CLASSIFICATION; VALIDATION; ISSUES

AB Background: Inflammation is closely associated with the initiation and development of tumors. Based on the counts of peripheral neutrophils, monocytes and lymphocytes, we established a new systemic inflammation response index (SIRI) to predict postoperative survival of patients with esophageal squamous cell carcinoma (ESCC).

Methods: A total of 916 ESCC patients treated with radical esophagectomy were enrolled in the present study.

Results: Survival analysis indicated that the median overall survival (OS) in patients with SIRI <= 1.2 was significantly higher than that in patients with SIRI > 1.2. The nomogram including SIRI could more accurately predict OS compared with the TNM staging system. In addition, the changes of SIRI from baseline to 8 weeks after the operation were correlated with patient survival. The patients with an increase in SIRI > 75% had worse OS compared with those with no change, while the patients with a decrease in SIRI > 75% or in the scope of 25% similar to 75% exhibited better OS.

Conclusions: SIRI was an independent prognostic index of ESCC patients after the radical resection. The nomogram integrating the SIRI could help clinicians to screen the high-risk patients and formulate the individualized treatment schemes.

C1 [Geng, Yiting; Zhu, Danxia; Wu, Chen; Wu, Jun; Wang, Qi; Li, Ruiqi; Wu, Changping] Soochow Univ, Dept Oncol, Affiliated Hosp 3, 185 Juqian St, Changzhou 213003, Jiangsu, Peoples R China.

[Jiang, Jingting; Wu, Changping] Soochow Univ, Dept Tumor Biol Treatment, Affiliated Hosp 3, 185 Juqian St, Changzhou 213003, Jiangsu, Peoples R China.

RP Wu, CP (corresponding author), Soochow Univ, Dept Oncol, Affiliated Hosp 3, 185 Juqian St, Changzhou 213003, Jiangsu, Peoples R China.; Jiang, JT; Wu, CP (corresponding author), Soochow Univ, Dept Tumor Biol Treatment, Affiliated Hosp 3, 185 Juqian St, Changzhou 213003, Jiangsu, Peoples R China.

EM jiangjingting@suda.edu.cn; wcpjjt@163.com

CR Balkwill F, 2001, LANCET, V357, P539, DOI 10.1016/S0140-6736(00)04046-0

Bray F, 2018, CA-CANCER J CLIN, V68, P394, DOI 10.3322/caac.21492

Chen WQ, 2016, CA-CANCER J CLIN, V66, P115, DOI 10.3322/caac.21338

Deng QW, 2015, J TRANSL MED, V13, DOI 10.1186/s12967-015-0409-0

Diakos CI, 2014, LANCET ONCOL, V15, pE493, DOI 10.1016/S1470-2045(14)70263-3

Dutta S, 2011, WORLD J SURG, V35, P1861, DOI 10.1007/s00268-011-1130-7

Ferrone C, 2010, J CLIN ONCOL, V28, P4045, DOI 10.1200/JCO.2010.27.9992

Gabrilovich DI, 2012, NAT REV IMMUNOL, V12, P253, DOI 10.1038/nri3175

Geng YT, 2016, SCI REP-UK, V6, DOI 10.1038/srep39482

Hanahan D, 2011, CELL, V144, P646, DOI 10.1016/j.cell.2011.02.013

Harrell FE, 1996, STAT MED, V15, P361, DOI 10.1002/(SICI)1097-0258(19960229)15:4<361::AID-SIM168>3.0.CO;2-4

Ku JH, 2015, BRIT J CANCER, V112, P461, DOI 10.1038/bjc.2014.631

Kusumanto Yoka H., 2003, Angiogenesis, V6, P283, DOI 10.1023/B:AGEN.0000029415.62384.ba

Law S, 2002, J GASTROEN HEPATOL, V17, P374, DOI 10.1046/j.1440-1746.2002.02728.x

Li S, 2017, J CANCER RES CLIN, V143, P2455, DOI 10.1007/s00432-017-2506-3

Liao Rui, 2016, Oncotarget, V7, P30951, DOI 10.18632/oncotarget.9049

Mantovani A, 2008, NATURE, V454, P436, DOI 10.1038/nature07205

Mantovani A, 2006, CANCER METAST REV, V25, P315, DOI 10.1007/s10555-006-9001-7

McMillan DC, 2013, CANCER TREAT REV, V39, P534, DOI 10.1016/j.ctrv.2012.08.003

Muller I, 2009, TRENDS IMMUNOL, V30, P522, DOI 10.1016/j.it.2009.07.007

Paramanathan A, 2014, SURG ONCOL, V23, P31, DOI 10.1016/j.suronc.2013.12.001

Proctor MJ, 2012, BRIT J CANCER, V107, P695, DOI 10.1038/bjc.2012.292

Qi Q, 2016, CANCER-AM CANCER SOC, V122, P2158, DOI 10.1002/cncr.30057

Reeh M, 2012, ANN THORAC SURG, V93, P890, DOI 10.1016/j.athoracsur.2011.11.035

Shamamian P, 2001, J CELL PHYSIOL, V189, P197, DOI 10.1002/jcp.10014

Shao YJ, 2016, J THORAC ONCOL, V11, P1774, DOI 10.1016/j.jtho.2016.06.019

Sun KY, 2014, J CANCER RES CLIN, V140, P1537, DOI 10.1007/s00432-014-1714-3

Szkandera J, 2013, BRIT J CANCER, V109, P2316, DOI 10.1038/bjc.2013.595

Szkandera J, 2014, INT J CANCER, V135, P362, DOI 10.1002/ijc.28677

Wei XL, 2015, BMC CANCER, V15, DOI 10.1186/s12885-015-1379-6

NR 30

TC 43

Z9 46

U1 1

U2 3

PU ELSEVIER SCIENCE BV

PI AMSTERDAM

PA PO BOX 211, 1000 AE AMSTERDAM, NETHERLANDS

SN 1567-5769

EI 1878-1705

J9 INT IMMUNOPHARMACOL

JI Int. Immunopharmacol.

PD DEC

PY 2018

VL 65

BP 503

EP 510

DI 10.1016/j.intimp.2018.10.002

PG 8

WC Immunology; Pharmacology & Pharmacy

WE Science Citation Index Expanded (SCI-EXPANDED)

SC Immunology; Pharmacology & Pharmacy

GA HE0EV

UT WOS:000452940500056

PM 30408627

DA 2022-05-19

ER

PT J

AU Wang, J

Zhu, XN

Zhu, LL

Chen, W

Ma, YH

Gan, T

Yang, JL

AF Wang, Jin

Zhu, Xiao-Nan

Zhu, Lin-Lin

Chen, Wei

Ma, Yi-Han

Gan, Tao

Yang, Jin-Lin

TI Efficacy and safety of endoscopic submucosal tunnel dissection for

superficial esophageal squamous cell carcinoma and precancerous lesions

SO WORLD JOURNAL OF GASTROENTEROLOGY

LA English

DT Article

DE Superficial esophageal squamous cell carcinoma; Endoscopic submucosal

tunnel dissection; Efficiency; Safety; Esophageal stricture

ID NEOPLASTIC LESIONS; MUCOSAL RESECTION; STRICTURE; EMR; THERAPY

AB AIM

To evaluate the clinical outcomes of patients who underwent endoscopic submucosal tunnel dissection (ESTD) for esophageal squamous cell carcinoma (ESCC) and precancerous lesions.

METHODS

ESTD was performed in 289 patients. The clinical outcomes of the patients and pathological features of the lesions were retrospectively reviewed.

RESULTS

A total of 311 lesions were included in the analysis. The en bloc rate, complete resection rate, and curative resection rate were 99.04%, 81.28%, and 78.46%, respectively. The ESTD procedure time was 102.4 +/- 35.1 min, the mean hospitalization time was 10.3 +/- 2.8 d, and the average expenditure was 3766.5 +/- 846.5 dollars. The intraoperative bleeding rate was 6.43%, the postoperative bleeding rate was 1.61%, the perforation rate was 1.93%, and the postoperative infection rate was 9.65%. Esophageal stricture and positive margin were severe adverse events, with an incidence rate of 14.79% and 15.76%, respectively. No tumor recurrence occurred during the follow-up period.

CONCLUSION

ESTD for ESCC and precancerous lesions is feasible and relatively safe, but for large mucosal lesions, the rate of esophageal stricture and positive margin is high.

C1 [Wang, Jin; Zhu, Xiao-Nan; Chen, Wei; Ma, Yi-Han; Gan, Tao; Yang, Jin-Lin] Sichuan Univ, West China Hosp, Dept Gastroenterol & Hepatol, 37th Guoxue Alley, Chengdu 610041, Sichuan, Peoples R China.

[Wang, Jin] Sichuan Univ, State Key Lab Biotherapy, Chengdu 610041, Sichuan, Peoples R China.

[Wang, Jin] Sichuan Univ, Canc Ctr, Chengdu 610041, Sichuan, Peoples R China.

[Zhu, Lin-Lin] Sichuan Univ, West China Hosp, Dept Gen Practice, Chengdu 610041, Sichuan, Peoples R China.

RP Wang, J (corresponding author), Sichuan Univ, West China Hosp, Dept Gastroenterol & Hepatol, 37th Guoxue Alley, Chengdu 610041, Sichuan, Peoples R China.

EM mouse-577@163.com

FU Science and Technology Department of Sichuan Province for Scientific

Research [2015SZ0123]

FX Supported by the Science and Technology Department of Sichuan Province

for Scientific Research, No. 2015SZ0123.

CR Ezoe Y, 2011, J CLIN GASTROENTEROL, V45, P222, DOI 10.1097/MCG.0b013e3181f39f4e

Gan T, 2016, GASTROINTEST ENDOSC, V84, P143, DOI 10.1016/j.gie.2016.01.049

Hashimoto S, 2011, GASTROINTEST ENDOSC, V74, P1389, DOI 10.1016/j.gie.2011.07.070

Honda M, 2011, J GASTROENTEROL, V46, P866, DOI 10.1007/s00535-011-0400-3

Huang R, 2017, GASTROINTEST ENDOSC, V86, P831, DOI 10.1016/j.gie.2017.03.001

Ishihara R, 2008, GASTROINTEST ENDOSC, V68, P1066, DOI 10.1016/j.gie.2008.03.1114

Joo DC, 2014, GUT LIVER, V8, P612, DOI 10.5009/gnl13130

Katada C, 2003, GASTROINTEST ENDOSC, V57, P165, DOI 10.1067/mge.2003.73

Koike Y, 2015, DIGEST ENDOSC, V27, P303, DOI 10.1111/den.12396

Lian JJ, 2012, GASTROINTEST ENDOSC, V76, P763, DOI 10.1016/j.gie.2012.06.014

Lingan Edmund B., 2014, THERAPEUTICS DIGESTI, P1, DOI DOI 10.1007/978-94-007-7344-8

Linghu E, 2013, ENDOSCOPY, V45, P60, DOI 10.1055/s-0032-1325965

LOIZOU LA, 1991, GASTROENTEROLOGY, V100, P1303

Maeda Y, 2012, ENDOSCOPY, V44, P565, DOI 10.1055/s-0031-1291664

Ono S, 2009, ENDOSCOPY, V41, P661, DOI 10.1055/s-0029-1214867

Ono S, 2009, GASTROINTEST ENDOSC, V70, P860, DOI 10.1016/j.gie.2009.04.044

Oyama Tsuneo, 2005, Clin Gastroenterol Hepatol, V3, pS67, DOI 10.1016/S1542-3565(05)00291-0

Pimentel-Nunes P, 2015, ENDOSCOPY, V47, P829, DOI 10.1055/s-0034-1392882

Qumseya BJ, 2013, J DIGEST DIS, V14, P140, DOI 10.1111/1751-2980.12002

Ratone JP, 2017, ANN GASTROENTEROL, V30, P62, DOI 10.20524/aog.2016.0085

Repici A, 2010, GASTROINTEST ENDOSC, V71, P715, DOI 10.1016/j.gie.2009.11.020

Sato H, 2013, GASTROINTEST ENDOSC, V78, P250, DOI 10.1016/j.gie.2013.01.008

Satodate H, 2003, GASTROINTEST ENDOSC, V58, P288, DOI 10.1067/mge.2003.361

Sohara N, 2013, WORLD J GASTROENTERO, V19, P528, DOI 10.3748/wjg.v19.i4.528

Tsao SKK, 2011, ENDOSCOPY, V43, pE119, DOI 10.1055/s-0030-1256148

Wang Jin, 2015, Sichuan Da Xue Xue Bao Yi Xue Ban, V46, P896

Wen J, 2014, SURG ENDOSC, V28, P1653, DOI 10.1007/s00464-013-3366-9

Xie X, 2017, SURG ENDOSC, V31, P462, DOI 10.1007/s00464-016-4939-1

Yamaguchi N, 2011, GASTROINTEST ENDOSC, V73, P1115, DOI 10.1016/j.gie.2011.02.005

Yano T, 2017, GASTROINTEST ENDOSC, V86, P492, DOI 10.1016/j.gie.2017.01.011

Zhai YQ, 2016, WORLD J GASTROENTERO, V22, P435, DOI 10.3748/wjg.v22.i1.435

Zhai Yaqi, 2014, Nan Fang Yi Ke Da Xue Xue Bao, V34, P36

2017, ESOPHAGUS, V14, P1, DOI DOI 10.1007/S10388-016-0551-7]

NR 33

TC 10

Z9 15

U1 0

U2 6

PU BAISHIDENG PUBLISHING GROUP INC

PI PLEASANTON

PA 8226 REGENCY DR, PLEASANTON, CA 94588 USA

SN 1007-9327

EI 2219-2840

J9 WORLD J GASTROENTERO

JI World J. Gastroenterol.

PD JUL 14

PY 2018

VL 24

IS 26

BP 2878

EP 2885

DI 10.3748/wjg.v24.i26.2878

PG 8

WC Gastroenterology & Hepatology

WE Science Citation Index Expanded (SCI-EXPANDED)

SC Gastroenterology & Hepatology

GA GN0QW

UT WOS:000438680600008

PM 30018482

OA Green Published, Green Submitted, hybrid

DA 2022-05-19

ER

PT J

AU Sun, LL

Sun, XX

Xu, XE

Zhu, MX

Wu, ZY

Shen, JH

Wu, JY

Huang, Q

Li, EM

Xu, LY

AF Sun, Ling-Ling

Sun, Xing-Xing

Xu, Xiu-E

Zhu, Meng-Xiao

Wu, Zhi-Yong

Shen, Jin-Hui

Wu, Jian-Yi

Huang, Qiao

Li, En-Min

Xu, Li-Yan

TI Overexpression of Jumonji AT-rich interactive domain 1B and PHD finger

protein 2 is involved in the progression of esophageal squamous cell

carcinoma

SO ACTA HISTOCHEMICA

LA English

DT Article

DE Jumonji AT-rich interactive domain 1B; PHD finger protein 2; Histone

demethylases; Overexpression; Esophageal squamous cell carcinoma

ID BREAST-CANCER; JMJD2 FAMILY; HISTONE; GENE; DEMETHYLATION; GASC1; PLU-1;

PHF2; TRIMETHYLATION; IDENTIFICATION

AB Jumonji AT-rich interactive domain 1B (JARID1B) and PHD finger protein 2 (PHF2), members of the histone demethylases, have been found to be involved in many types of tumors. However, the expression and prognostic significance of JARID1B and PHF2 in esophageal squamous cell carcinoma (ESCC) still remains unclear. In this study, JARID1B and PHF2 expression were detected on tissue microarrays of ESCC samples in 120 cases using immunohistochemical staining. Our results showed that JARID1B and PHF2 were overexpressed in ESCCs. In addition, a significant correlation was observed between JARID1B nuclear expression level and histological grade (P=0.003). Kaplan-Meier survival analysis showed a tendency that high cytoplasmic expression of JARID1B and PHF2 was associated with decreased overall survival of ESCC patients, whereas JARID1B high expression in the nucleus was associated with high overall survival, although there was no statistical significance. Overall, our data suggest that JARID1B and PHF2 are overexpressed in ESCC and that they may play crucial roles in the course of ESCC initiation and/or progression. (C) 2012 Elsevier GmbH. All rights reserved.

C1 [Sun, Ling-Ling; Sun, Xing-Xing; Xu, Xiu-E; Zhu, Meng-Xiao; Xu, Li-Yan] Shantou Univ, Inst Oncol Pathol, Coll Med, Shantou 515041, Peoples R China.

[Wu, Jian-Yi; Huang, Qiao; Li, En-Min] Shantou Univ, Coll Med, Dept Biochem & Mol Biol, Shantou 515041, Peoples R China.

[Wu, Zhi-Yong] Sun Yat Sen Univ, Shantou Hosp, Shantou Cent Hosp, Dept Surg Oncol, Shantou 515041, Peoples R China.

[Shen, Jin-Hui] Sun Yat Sen Univ, Shantou Hosp, Shantou Cent Hosp, Dept Pathol, Shantou 515041, Peoples R China.

RP Xu, LY (corresponding author), Shantou Univ, Inst Oncol Pathol, Coll Med, 22 Xinling Rd, Shantou 515041, Peoples R China.

EM liyanxu1130@yahoo.com.cn

FU National High Technology Research and Development Program of China

[2006AA02A403]; Natural Science Foundation of China-Guangdong Joint Fund

[U0932001]; Natural Science Foundation of China [81172264]

FX This work was supported by grants from the National High Technology

Research and Development Program of China (No. 2006AA02A403), the

Natural Science Foundation of China-Guangdong Joint Fund (No. U0932001)

and the Natural Science Foundation of China (No. 81172264).

CR Baba A, 2011, NAT CELL BIOL, V13, P668, DOI 10.1038/ncb2228

Barrett A, 2002, INT J CANCER, V101, P581, DOI 10.1002/ijc.10644

Christensen J, 2007, CELL, V128, P1063, DOI 10.1016/j.cell.2007.02.003

Cloos PAC, 2006, NATURE, V442, P307, DOI 10.1038/nature04837

Enzinger PC, 2003, NEW ENGL J MED, V349, P2241, DOI 10.1056/NEJMra035010

Esteller M, 2007, NAT REV GENET, V8, P286, DOI 10.1038/nrg2005

Fischle W, 2003, CURR OPIN CELL BIOL, V15, P172, DOI 10.1016/S0955-0674(03)00013-9

GOUDIE DR, 1993, NAT GENET, V3, P165, DOI 10.1038/ng0293-165

Hasenpusch-Theil K, 1999, MAMM GENOME, V10, P294, DOI 10.1007/s003359900989

Hayami S, 2010, MOL CANCER, V9, DOI 10.1186/1476-4598-9-59

Heinlein CA, 2002, ENDOCR REV, V23, P175, DOI 10.1210/er.23.2.175

Hess JL, 2004, TRENDS MOL MED, V10, P500, DOI 10.1016/j.molmed.2004.08.005

Italiano A, 2006, CANCER GENET CYTOGEN, V167, P122, DOI 10.1016/j.cancergencyto.2006.01.004

Jones PA, 2007, CELL, V128, P683, DOI 10.1016/j.cell.2007.01.029

Kamangar F, 2006, J CLIN ONCOL, V24, P2137, DOI 10.1200/JCO.2005.05.2308

Katoh M, 2004, INT J ONCOL, V24, P1623

Klose RJ, 2007, NAT REV MOL CELL BIO, V8, P307, DOI 10.1038/nrm2143

Klose RJ, 2006, NAT REV GENET, V7, P715, DOI 10.1038/nrg1945

Krivtsov AV, 2007, NAT REV CANCER, V7, P823, DOI 10.1038/nrc2253

Krivtsov AV, 2006, NATURE, V442, P818, DOI 10.1038/nature04980

Lim S, 2010, INT J CANCER, V127, P1991, DOI 10.1002/ijc.25538

Liu G, 2009, ONCOGENE, V28, P4491, DOI 10.1038/onc.2009.297

Loh YH, 2007, GENE DEV, V21, P2545, DOI 10.1101/gad.1588207

Lu PJ, 1999, J BIOL CHEM, V274, P15633, DOI 10.1074/jbc.274.22.15633

Margueron R, 2005, CURR OPIN GENET DEV, V15, P163, DOI 10.1016/j.gde.2005.01.005

Nicholson GA, 1996, NAT GENET, V13, P101, DOI 10.1038/ng0596-101

Northcott PA, 2009, NAT GENET, V41, P465, DOI 10.1038/ng.336

Peterson CL, 2004, CURR BIOL, V14, pR546, DOI 10.1016/j.cub.2004.07.007

Pisani P, 1999, INT J CANCER, V83, P18, DOI 10.1002/(SICI)1097-0215(19990924)83:1&lt;18::AID-IJC5&gt;3.0.CO;2-M

Roesch A, 2005, MODERN PATHOL, V18, P1249, DOI 10.1038/modpathol.3800413

Roesch A, 2006, J INVEST DERMATOL, V126, P1850, DOI 10.1038/sj.jid.5700324

Seward DJ, 2007, NAT STRUCT MOL BIOL, V14, P240, DOI 10.1038/nsmb1200

Shi Y, 2007, MOL CELL, V25, P1, DOI 10.1016/j.molcel.2006.12.010

Sinha S, 2008, MOL CANCER, V7, DOI 10.1186/1476-4598-7-84

Tachibana M, 2008, EUR J CARDIO-THORAC, V34, P427, DOI 10.1016/j.ejcts.2008.04.022

Trojer P, 2006, CELL, V125, P213, DOI 10.1016/j.cell.2006.04.003

Tsukada Y, 2006, NATURE, V439, P811, DOI 10.1038/nature04433

Vaquero Alejandro, 2003, Sci Aging Knowledge Environ, V2003, pRE4, DOI 10.1126/sageke.2003.14.re4

Vogelstein B, 2004, NAT MED, V10, P789, DOI 10.1038/nm1087

Wen H, 2010, J BIOL CHEM, V285, P9322, DOI 10.1074/jbc.C109.097667

Whetstine JR, 2006, CELL, V125, P467, DOI 10.1016/j.cell.2006.03.028

Wissmann M, 2007, NAT CELL BIOL, V9, P347, DOI 10.1038/ncb1546

Xiang Y, 2007, P NATL ACAD SCI USA, V104, P19226, DOI 10.1073/pnas.0700735104

Yamane K, 2007, MOL CELL, V25, P801, DOI 10.1016/j.molcel.2007.03.001

Yang ZQ, 2006, CANCER RES, V66, P11632, DOI 10.1158/0008-5472.CAN-06-2946

Yang ZQ, 2000, CANCER RES, V60, P4735

Zhang FR, 2008, J HISTOCHEM CYTOCHEM, V56, P193, DOI 10.1369/jhc.7A7353.2007

Zhou W, 2009, BMB REP, V42, P154, DOI 10.5483/BMBRep.2009.42.3.154

NR 48

TC 19

Z9 20

U1 0

U2 6

PU ELSEVIER GMBH

PI MUNICH

PA HACKERBRUCKE 6, 80335 MUNICH, GERMANY

SN 0065-1281

EI 1618-0372

J9 ACTA HISTOCHEM

JI Acta Histochem.

PY 2013

VL 115

IS 1

BP 56

EP 62

DI 10.1016/j.acthis.2012.04.001

PG 7

WC Cell Biology

WE Science Citation Index Expanded (SCI-EXPANDED)

SC Cell Biology

GA 111HQ

UT WOS:000316512800009

PM 22534467

DA 2022-05-19

ER

PT J

AU Wang, DW

You, D

Pan, YH

Liu, PJ

AF Wang, Dawei

You, Dong

Pan, Yinghua

Liu, Peiji

TI Downregulation of lncRNA-HEIH curbs esophageal squamous cell carcinoma

progression by modulating miR-4458/PBX3

SO THORACIC CANCER

LA English

DT Article

DE Esophageal squamous cell carcinoma; HEIH; miR-4458; PBX3

ID CANCER; INVASION; GROWTH

AB Background Long non-coding RNAs (lncRNAs) have been found to play a specific part in the development of esophageal squamous cell carcinoma (ESCC), except for lncRNA HEIH. Here, we aimed to discover the molecular mechanisms of HEIH in ESCC.

Methods We detected the expression level of HEIH and miR-4458 in ESCC tissues and cells using qRT-PCR assay. A dual luciferase reporter assay was used to check the relationship between HEIH, miR-4458 or PBX3. Counting Clock Kit-8 (CCK-8) assay and transwell assay were used to detect ESCC cell proliferation and invasion capability. Western blot analysis was used to measure the protein expression level of PBX3.

Results HEIH was confirmed to be upregulated in both ESCC tissues and cell lines. Inversely, there was a downregulation of miR-4458 in ESCC tissues and cell lines. Functionally, we noticed that depletion of HEIH restrained ESCC cell viability, and invasion capability. Moreover, PBX silencing was found to restrain ESCC cell progression, while miR-4458 or HEIH vector both could alleviate its suppressive effect.

Conclusions The present study clarified that HEIH regulated ESCC progression by suppressing miR-4458 and upregulating PBX3. Our findings suggested that HEIH could be a possible therapeutic target for ESCC treatment.

C1 [Wang, Dawei; You, Dong; Liu, Peiji] Qingdao Univ, Dept Radiotherapy, Yantai Yuhuangding Hosp, East Yuhuangding Rd, Yantai 26400, Shandong, Peoples R China.

[Pan, Yinghua] Qingdao Univ, Dept Radiol, Yantai Yuhuangding Hosp, Yantai, Peoples R China.

RP Liu, PJ (corresponding author), Qingdao Univ, Dept Radiotherapy, Yantai Yuhuangding Hosp, East Yuhuangding Rd, Yantai 26400, Shandong, Peoples R China.

EM baiyanjizhe2532@163.com

CR Chang ZW, 2018, J EXP CLIN CANC RES, V37, DOI 10.1186/s13046-018-0724-4

Chen Q, 2020, BIOMED RES INT, V2020, DOI 10.1155/2020/8954513

Chu J, 2019, BIOMED PHARMACOTHER, V116, DOI 10.1016/j.biopha.2019.109029

Cui CH, 2018, CANCER RES TREAT, V50, P992, DOI 10.4143/crt.2017.226

Feng Z, 2020, CANCER BIOTHER RADIO, V35, P66, DOI 10.1089/cbr.2019.2926

Guo JL, 2020, PATHOL ONCOL RES, V26, P1757, DOI 10.1007/s12253-019-00718-w

Jia KG, 2019, J CELL BIOCHEM, V120, P3529, DOI 10.1002/jcb.27629

Kamangar F, 2006, J CLIN ONCOL, V24, P2137, DOI 10.1200/JCO.2005.05.2308

Kano M, 2010, INT J CANCER, V127, P2804, DOI 10.1002/ijc.25284

Li BT, 2017, BIOCHEM BIOPH RES CO, V484, P241, DOI 10.1016/j.bbrc.2017.01.084

Li HF, 2017, ONCOTARGETS THER, V10, P5685, DOI 10.2147/OTT.S150139

Li P, 2019, HUM CELL, V32, P522, DOI 10.1007/s13577-019-00273-1

Li X, 2019, INT J MOL MED, V44, P973, DOI 10.3892/ijmm.2019.4258

Liang Y, 2018, CELL DEATH DIFFER, V25, P1980, DOI 10.1038/s41418-018-0084-9

Ma W, 2018, EUR REV MED PHARMACO, V22, P2638, DOI 10.26355/eurrev_201805_14958

Tang D, 2015, CELL PHYSIOL BIOCHEM, V35, P1797, DOI 10.1159/000373991

Wan T, 2020, BIOMED PHARMACOTHER, V125, DOI 10.1016/j.biopha.2020.109916

Wang Li, 2020, Panminerva Med, DOI 10.23736/S0031-0808.19.03801-1

Wu F, 2018, GENET TEST MOL BIOMA, V22, P29, DOI 10.1089/gtmb.2017.0115

Wu JR, 2019, BIOCHEM CELL BIOL, V97, P722, DOI 10.1139/bcb-2019-0008

Yang F, 2011, HEPATOLOGY, V54, P1679, DOI 10.1002/hep.24563

Yang HM, 2020, NUTR CANCER, V72, P1336, DOI 10.1080/01635581.2020.1733623

Yao J, 2018, ONCOL LETT, V16, P3935, DOI 10.3892/ol.2018.9130

Yi J, 2017, ACTA BIOCH BIOPH SIN, V49, P159, DOI 10.1093/abbs/gmw131

Zhao HY, 2017, BIOSCIENCE REP, V37, DOI 10.1042/BSR20170682

Zhao J, 2019, PHARMAZIE, V74, P471, DOI 10.1691/ph.2019.9428

Zhou HH, 2020, ARCH DERMATOL RES, V312, P665, DOI 10.1007/s00403-020-02051-8

NR 27

TC 4

Z9 4

U1 0

U2 0

PU WILEY

PI HOBOKEN

PA 111 RIVER ST, HOBOKEN 07030-5774, NJ USA

SN 1759-7706

EI 1759-7714

J9 THORAC CANCER

JI Thorac. Cancer

PD JUL

PY 2020

VL 11

IS 7

BP 1963

EP 1971

DI 10.1111/1759-7714.13489

EA MAY 2020

PG 9

WC Oncology; Respiratory System

WE Science Citation Index Expanded (SCI-EXPANDED)

SC Oncology; Respiratory System

GA ME1CN

UT WOS:000535066200001

PM 32449803

OA Green Published, gold

DA 2022-05-19

ER

PT J

AU Lin, J

Liu, ZW

Liao, SS

Li, E

Wu, XH

Zeng, WT

AF Lin, Jing

Liu, Zewa

Liao, Shasha

Li, E.

Wu, Xiaohua

Zeng, Wanting

TI Elevation of long non-coding RNA GAS5 and knockdown of microRNA-21

up-regulate RECK expression to enhance esophageal squamous cell

carcinoma cell radio-sensitivity after radiotherapy

SO GENOMICS

LA English

DT Article

DE Long non-coding RNA GAS5; MicroRNA-21; Esophageal squamous cell

carcinoma; Radio-sensitivity; Reversion-inducing cysteine-rich protein

with Kazal motifs

ID INCREASES RADIOSENSITIVITY; MIR-21; OVEREXPRESSION; SUPPRESSOR; GROWTH;

GENES

AB Objective: Lately, lncRNAs have been proposed to function in the radio-sensitivity of tumor cells, yet the role of lncRNA GAS5 in that of esophageal squamous cell carcinoma (ESCC) has scarcely been studied. This study aims to examine GAS5's effects on ESCC cell radio-sensitivity.

Methods: GAS5, miR-21 and RECK expression in radiation-sensitive and radiation-resistant ESCC tissues, and TE-1 and TE-1-R cells was determined. TE-1 and TE-1-R cells were treated with pcDNA-GAS5 or miR-21 inhibitors to figure out their roles in ESCC cell proliferation, radio-sensitivity, and apoptosis via gain- and loss-of-function experiments.

Results: We found underexpressed GAS5 and RECK, and overexpressed miR-21 in ESCC. GAS5 elevation and miR-21 inhibition reduced viability and the colony formation ability, and enhanced the apoptosis of ESCC cells under radiation.

Conclusion: Our study reveals that GAS5 elevation up-regulates RECK expression by down-regulating miR-21 to increase ESCC cell apoptosis after radiation therapy, thus enhancing cell radio-sensitivity.

C1 [Lin, Jing; Liu, Zewa] Shantou Univ, Med Coll, Affiliated Hosp 1, Dept Oncol, 57 Changping Rd, Shantou 515041, Guangdong, Peoples R China.

[Liao, Shasha; Li, E.; Wu, Xiaohua] Shantou Longhu Peoples Hosp, Dept Oncol, Shantou 515041, Guangdong, Peoples R China.

[Zeng, Wanting] UCL, Div Med, London WC1E 6BT, England.

RP Lin, J (corresponding author), Shantou Univ, Med Coll, Affiliated Hosp 1, Dept Oncol, 57 Changping Rd, Shantou 515041, Guangdong, Peoples R China.

EM jinglinab123@163.com

CR Abnet CC, 2018, GASTROENTEROLOGY, V154, P360, DOI 10.1053/j.gastro.2017.08.023

Ayuk SM, 2016, J PHOTOCH PHOTOBIO B, V161, P368, DOI 10.1016/j.jphotobiol.2016.05.027

Cobanoglu U, 2017, EUR REV MED PHARMACO, V21, P5399, DOI 10.26355/eurrev_201712_13926

Codipilly DC, 2018, GASTROINTEST ENDOSC, V88, P413, DOI 10.1016/j.gie.2018.04.2352

Gao JB, 2019, INT J BIOL MACROMOL, V126, P994, DOI 10.1016/j.ijbiomac.2018.12.176

Garchow BG, 2011, EMBO MOL MED, V3, P605, DOI 10.1002/emmm.201100171

Hu LT, 2016, TUMOR BIOL, V37, P2691, DOI 10.1007/s13277-015-4111-x

Huang JB, 2018, CANCER MED-US, V7, P3157, DOI 10.1002/cam4.1524

Huang S, 2013, DIS ESOPHAGUS, V26, P823, DOI 10.1111/j.1442-2050.2012.01389.x

Jiang HJ, 2017, RADIAT ONCOL, V12, DOI 10.1186/s13014-017-0802-3

Kono K, 2018, ESOPHAGUS-TOKYO, V15, P1, DOI 10.1007/s10388-017-0596-2

Li F, 2018, CANCER CELL INT, V18, DOI 10.1186/s12935-018-0502-6

Li SL, 2007, WORLD J GASTROENTERO, V13, P6076, DOI 10.3748/wjg.13.6076

Lopes AB, 2012, WORLD J GASTRO ENDOS, V4, P9, DOI 10.4253/wjge.v4.i1.9

Matrka MC, 2018, PLOS GENET, V14, DOI 10.1371/journal.pgen.1007227

Matsuzaki T, 2018, BIOL OPEN, V7, DOI 10.1242/bio.033985

Noda M, 2003, CANCER METAST REV, V22, P167, DOI 10.1023/A:1023043315031

Nouraee N, 2013, PLOS ONE, V8, DOI 10.1371/journal.pone.0073009

Qian X, 2017, DIS ESOPHAGUS, V30, DOI 10.1093/dote/dox027

Rinn JL, 2012, ANNU REV BIOCHEM, V81, P145, DOI 10.1146/annurev-biochem-051410-092902

Song J, 2014, J ORTHOP RES, V32, P1628, DOI 10.1002/jor.22718

Wang GJ, 2018, MED SCI MONITOR, V24, P7689, DOI 10.12659/MSM.910867

Wang K, 2018, BIOCHEM BIOPH RES CO, V495, P1151, DOI 10.1016/j.bbrc.2017.11.119

Wang N, 2013, DIGEST DIS SCI, V58, P1863, DOI 10.1007/s10620-013-2612-2

Wang XC, 2012, J RADIAT RES, V53, P72, DOI 10.1269/jrr.11090

Wapinski O, 2011, TRENDS CELL BIOL, V21, P354, DOI 10.1016/j.tcb.2011.04.001

Xue YB, 2017, ONCOL RES, V25, P1305, DOI 10.3727/096504017X14850182723737

Yanagawa M, 2012, J NUCL MED, V53, P872, DOI 10.2967/jnumed.111.098699

Zhang HP, 2015, ONCOL LETT, V10, P1495, DOI 10.3892/ol.2015.3456

Zhao WG, 2015, MOL CELL BIOCHEM, V405, P125, DOI 10.1007/s11010-015-2404-4

Zhou S, 2015, AM J CANCER RES, V5, P267

Zhu J, 2017, ONCOL LETT, V13, P1911, DOI 10.3892/ol.2017.5656

NR 32

TC 8

Z9 10

U1 1

U2 11

PU ACADEMIC PRESS INC ELSEVIER SCIENCE

PI SAN DIEGO

PA 525 B ST, STE 1900, SAN DIEGO, CA 92101-4495 USA

SN 0888-7543

EI 1089-8646

J9 GENOMICS

JI Genomics

PD MAY

PY 2020

VL 112

IS 3

BP 2173

EP 2185

DI 10.1016/j.ygeno.2019.12.013

PG 13

WC Biotechnology & Applied Microbiology; Genetics & Heredity

WE Science Citation Index Expanded (SCI-EXPANDED)

SC Biotechnology & Applied Microbiology; Genetics & Heredity

GA KU8XP

UT WOS:000519997800008

PM 31866421

OA Bronze

DA 2022-05-19

ER

PT J

AU Zhang, ZH

Xiong, R

Li, CW

Xu, MQ

Guo, MF

AF Zhang, Zhenghua

Xiong, Ran

Li, Caiwei

Xu, Meiqing

Guo, Mingfa

TI LncRNA TUG1 promotes cisplatin resistance in esophageal squamous cell

carcinoma cells by regulating Nrf2

SO ACTA BIOCHIMICA ET BIOPHYSICA SINICA

LA English

DT Article

DE esophageal squamous cell carcinoma; taurine upregulated gene 1; Nrf2;

TE-1

ID LONG NONCODING RNA; P-GLYCOPROTEIN; UP-REGULATION; CANCER;

OVEREXPRESSION; PROLIFERATION

AB Esophageal squamous cell carcinoma (ESCC) is a common malignancy with poor prognosis. The drug resistance compromises the efficacy of chemotherapy for ESCC. Long non-coding RNA taurine upregulated gene 1 (TUG1) has been identified as a promoter of cancer progression and chemotherapy resistance in many malignancies. However, the exact role of TUG1 in ESCC chemotherapy resistance remains unclear. In this study, we showed that TUG1 expression in TE-1-derived cisplatin (DDP)-resistant (TE-1/DDP) cells was higher than that in TE-1 cells. Furthermore, TUG1 promoted DDP resistance in TE-1 and TE-1/DDP cells by promoting cell proliferation, suppressing cell apoptosis, and elevating protein expression of the classical multi-drug resistance-related P-gp. In contrast, TUG1 knockdown exerted an opposite effect. Mechanistically, RNA pull-down and RNA immunoprecipitation assays confirmed that TUG1 directly bound to nuclear factor (erythroid-derived 2)-like 2 (Nrf2) protein and elevated Nrf2 protein expression. Moreover, Nrf2-neutralizing antibody effectively reversed the TUG1 overexpression-mediated promotion of ESCC cell resistance to DDP. In conclusion, our findings demonstrated that TUG1 promoted ESCC cell resistance to DDP, at least in part, through upregulating Nrf2.

C1 [Zhang, Zhenghua; Xiong, Ran; Li, Caiwei; Xu, Meiqing; Guo, Mingfa] Univ Sci & Technol China, Div Life Sci & Med, Affiliated Hosp 1, Dept Thorac Surg, Hefei 230001, Anhui, Peoples R China.

RP Guo, MF (corresponding author), Univ Sci & Technol China, Div Life Sci & Med, Affiliated Hosp 1, Dept Thorac Surg, Hefei 230001, Anhui, Peoples R China.

EM gmingfa08@163.com

FU First AffiliatedHospital of University of Science and Technology of

China

FX This work was supported by the research fund from the First

AffiliatedHospital of University of Science and Technology of China.

CR Abnet CC, 2018, GASTROENTEROLOGY, V154, P360, DOI 10.1053/j.gastro.2017.08.023

Akdemir B, 2017, MOL CANCER RES, V15, P1570, DOI 10.1158/1541-7786.MCR-17-0232

Chan D, 2018, CELLS-BASEL, V7, DOI 10.3390/cells7100143

Chen WQ, 2016, CA-CANCER J CLIN, V66, P115, DOI 10.3322/caac.21338

Deng H, 2016, TUMOR BIOL, V37, P11623, DOI 10.1007/s13277-016-5125-8

Gong P, 2018, CELL DEATH DIS, V9, DOI 10.1038/s41419-018-0602-1

He C, 2018, CELL PHYSIOL BIOCHEM, V48, P1928, DOI 10.1159/000492517

Huo JF, 2019, J CELL BIOCHEM, V120, P6127, DOI 10.1002/jcb.27900

Jeddi F, 2018, BIOMED PHARMACOTHER, V97, P286, DOI 10.1016/j.biopha.2017.10.129

Jiang HJ, 2017, RADIAT ONCOL, V12, DOI 10.1186/s13014-017-0802-3

Jiang L, 2016, CANCER CHEMOTH PHARM, V78, P333, DOI 10.1007/s00280-016-3066-y

Kawasaki Y, 2014, ANN SURG ONCOL, V21, P2347, DOI 10.1245/s10434-014-3600-2

Li CF, 2017, BIOCHEM BIOPH RES CO, V491, P552, DOI 10.1016/j.bbrc.2017.03.042

Liu JG, 2016, ASIA-PAC J CLIN ONCO, V12, P308, DOI 10.1111/ajco.12503

Ma SH, 2018, ANN NY ACAD SCI, V1434, P164, DOI 10.1111/nyas.13681

Mao HM, 2018, CELL PHYSIOL BIOCHEM, V48, P1468, DOI 10.1159/000492257

Niu YC, 2017, MOL CANCER, V16, DOI 10.1186/s12943-016-0575-6

Shi H, 2018, INT J ONCOL, V53, P167, DOI 10.3892/ijo.2018.4367

Wang Y, 2017, BIOMED PHARMACOTHER, V87, P548, DOI 10.1016/j.biopha.2016.12.040

Wu B, 2019, EXP THER MED, V17, P1240, DOI 10.3892/etm.2018.7044

Wu JF, 2017, ONCOTARGET, V8, P93502, DOI 10.18632/oncotarget.19548

Xie DL, 2017, ONCOTARGET, V8, P88689, DOI 10.18632/oncotarget.20927

Xu YT, 2015, TUMOR BIOL, V36, P1643, DOI 10.1007/s13277-014-2763-6

Yang B, 2018, BIOSCI REP, V38

Yang F, 2018, J PHARMACOL SCI, V137, P116, DOI 10.1016/j.jphs.2018.06.002

Yang X, 2019, PHARM BIOL, V57, P184, DOI 10.1080/13880209.2019.1582679

Young TL, 2005, CURR BIOL, V15, P501, DOI 10.1016/j.cub.2005.02.027

Zeng B, 2017, ONCOTARGET, V8, P113650, DOI 10.18632/oncotarget.21922

Zhang HF, 2019, CLIN CANCER RES, V25, P1989, DOI 10.1158/1078-0432.CCR-18-0773

Zhang JZ, 2018, THORAC CANCER, V9, P726, DOI 10.1111/1759-7714.12640

Zhao Y, 2018, INT J ONCOL, V53, P644, DOI 10.3892/ijo.2018.4409

Zhu JY, 2016, FREE RADICAL BIO MED, V99, P544, DOI 10.1016/j.freeradbiomed.2016.09.010

Zhu J, 2017, ONCOTARGET, V8, P65253, DOI 10.18632/oncotarget.18224

NR 33

TC 31

Z9 33

U1 2

U2 7

PU OXFORD UNIV PRESS

PI OXFORD

PA GREAT CLARENDON ST, OXFORD OX2 6DP, ENGLAND

SN 1672-9145

EI 1745-7270

J9 ACTA BIOCH BIOPH SIN

JI Acta Biochim. Biophys. Sin.

PD AUG

PY 2019

VL 51

IS 8

BP 826

EP 833

DI 10.1093/abbs/gmz069

PG 8

WC Biochemistry & Molecular Biology; Biophysics

WE Science Citation Index Expanded (SCI-EXPANDED)

SC Biochemistry & Molecular Biology; Biophysics

GA JF2VS

UT WOS:000491244700007

PM 31287493

DA 2022-05-19

ER

PT J

AU Zhang, NN

Zhang, YT

Li, QH

Zhang, MZ

AF Zhang, Nannan

Zhang, Yanting

Li, Qinghua

Zhang, Mingzhi

TI Ring box protein-1 mediates the disassembly of cilia in esophageal

squamous cell carcinoma cells

SO INTERNATIONAL JOURNAL OF CLINICAL AND EXPERIMENTAL MEDICINE

LA English

DT Article

DE RBX1; esophageal squamous cell carcinoma; ciliary disassembly

ID AURORA; EXPRESSION; CYCLE

AB Cilia are structurally and evolutionarily conserved organelles in many cells with an origin similar to that of basal bodies. Recent studies suggest that nearly all types of human cells have cilia. Cilia are predominantly lost in cancer cells. Our previous study reported that the ubiquitin-proteosome was probably associated with ciliary disassembly. As part of the ubiquitin-proteasome complex, RBX1 may respond to ciliary disassembly in esophageal squamous cell carcinoma (ESCC) cells. Compared with Het-1A, which represents the non-cancerous esophageal epithelial cells, cilia are lost in ESCC cells. Therefore, we explored the function of RBX1 in the ciliary disassembly of ESCC cells. Our results showed that RBX1 was overexpressed in ESCC cells of Eca109, EC1 and EC9706. Furthermore, the suppression of RBX1 by the shRNA lentivirus in ESCC cells reversed the phenotype of lost cilia. The expressions of the tumor suppressors LKB1 and AMPK were increased, while the oncogene Aurora A was inhibited in shRBX1 ESCC cells. These findings initially demonstrated that RBX1 mediated the disassembly of human cilia and suggested that the abnormal expression of the cilia-related gene RBX1 influenced cilia formation and the initiation of cancer.

C1 [Zhang, Nannan] Zhengzhou Univ, Affiliated Hosp 1, Dept Clin Lab, Zhengzhou 450052, Henan, Peoples R China.

[Zhang, Mingzhi] Zhengzhou Univ, Affiliated Hosp 1, Dept Oncol, 1 East Jianshe Rd, Zhengzhou 450052, Henan, Peoples R China.

[Zhang, Yanting; Li, Qinghua] Zhengzhou Univ, Sch Life Sci, Zhengzhou 450001, Henan, Peoples R China.

RP Zhang, MZ (corresponding author), Zhengzhou Univ, Affiliated Hosp 1, Dept Oncol, 1 East Jianshe Rd, Zhengzhou 450052, Henan, Peoples R China.

EM mingzhi_zhang1@163.com

FU International Science and Technology Cooperation Program of the Ministry

of Science and Technology of PR China [2007DFA01240]

FX This study was supported by grants from the International Science and

Technology Cooperation Program of the Ministry of Science and Technology

of PR China (no. 2007DFA01240).

CR Basten Sander G, 2013, Cilia, V2, P2, DOI 10.1186/2046-2530-2-2

Basten SG, 2013, CILIA, V2, P1

Bhogaraju S, 2013, CILIA, V2, P1

Boehlke C, 2010, NAT CELL BIOL, V12, P1115, DOI 10.1038/ncb2117

Cardenas-Rodriguez M, 2013, HUM MOL GENET, V22, P4031, DOI 10.1093/hmg/ddt253

Cole DG, 2003, TRAFFIC, V4, P435, DOI 10.1034/j.1600-0854.2003.t01-1-00103.x

Deshaies RJ, 2009, ANNU REV BIOCHEM, V78, P399, DOI 10.1146/annurev.biochem.78.101807.093809

Gong FH, 2014, BIOCHEM BIOPH RES CO, V444, P420, DOI 10.1016/j.bbrc.2014.01.081

Gradilone SA, 2013, CANCER RES, V73, P2259, DOI 10.1158/0008-5472.CAN-12-2938

Hassounah NB, 2013, PLOS ONE, V8, DOI 10.1371/journal.pone.0068521

Ji HB, 2007, NATURE, V448, P807, DOI 10.1038/nature06030

Jia LJ, 2011, J BIOL CHEM, V286, P3379, DOI 10.1074/jbc.M110.188425

Jia LJ, 2009, CANCER RES, V69, P4974, DOI 10.1158/0008-5472.CAN-08-4671

Lin Y, 2012, MED ONCOL, V29, P311, DOI 10.1007/s12032-010-9808-5

McMurray RJ, 2013, SCI REP-UK, V3, DOI 10.1038/srep03545

Migita K, 2014, GASTRIC CANCER, V17, P601, DOI 10.1007/s10120-013-0318-y

Moore R, 2004, GENESIS, V38, P1, DOI 10.1002/gene.10243

Moser JJ, 2009, BMC CANCER, V9, DOI 10.1186/1471-2407-9-448

Noureddine MA, 2002, DEV CELL, V2, P757, DOI 10.1016/S1534-5807(02)00164-8

Pan JM, 2004, DEV CELL, V6, P445, DOI 10.1016/S1534-5807(04)00064-4

Phua SC, 2017, CELL, V168, P264, DOI 10.1016/j.cell.2016.12.032

Plotnikova OV, 2008, CANCER RES, V68, P2058, DOI 10.1158/0008-5472.CAN-07-5838

Pugacheva EN, 2007, CELL, V129, P1351, DOI 10.1016/j.cell.2007.04.035

Schneider L, 2005, CURR BIOL, V15, P1861, DOI 10.1016/j.cub.2005.09.012

Shi K, 2013, MOL BIOL REP, V40, P3113, DOI 10.1007/s11033-012-2385-2

Tan M, 2009, P NATL ACAD SCI USA, V106, P6203, DOI 10.1073/pnas.0812425106

Vogel G, 2005, SCIENCE, V310, P216, DOI 10.1126/science.310.5746.216

Wang W, 2013, J SURG ONCOL, V107, P758, DOI 10.1002/jso.23317

Wilson CW, 2010, BMC BIOL, V8, DOI 10.1186/1741-7007-8-102

Xu JY, 2010, J AM SOC NEPHROL, V21, P2041, DOI 10.1681/ASN.2010040345

Yang D, 2013, CELL DEATH DIFFER, V20, P235, DOI 10.1038/cdd.2012.113

Yuan K, 2010, J HISTOCHEM CYTOCHEM, V58, P857, DOI 10.1369/jhc.2010.955856

Zhang NN, 2017, FOLIA MICROBIOL, V62, P57, DOI 10.1007/s12223-016-0472-9

NR 33

TC 0

Z9 0

U1 0

U2 0

PU E-CENTURY PUBLISHING CORP

PI MADISON

PA 40 WHITE OAKS LN, MADISON, WI 53711 USA

SN 1940-5901

J9 INT J CLIN EXP MED

JI Int. J. Clin. Exp. Med.

PY 2019

VL 12

IS 5

BP 5654

EP 5660

PG 7

WC Medicine, Research & Experimental

WE Science Citation Index Expanded (SCI-EXPANDED)

SC Research & Experimental Medicine

GA IA8FG

UT WOS:000469792800126

DA 2022-05-19

ER

PT J

AU Hijiya, N

Shibata, T

Daa, T

Hamanaka, R

Uchida, T

Matsuura, K

Tsukamoto, Y

Nakada, C

Iha, H

Inomata, M

Moriyama, M

AF Hijiya, Naoki

Shibata, Tomotaka

Daa, Tsutomu

Hamanaka, Ryoji

Uchida, Tomohisa

Matsuura, Keiko

Tsukamoto, Yoshiyuki

Nakada, Chisato

Iha, Hidekatsu

Inomata, Masafumi

Moriyama, Masatsugu

TI Overexpression of cannabinoid receptor 1 in esophageal squamous cell

carcinoma is correlated with metastasis to lymph nodes and distant

organs, and poor prognosis

SO PATHOLOGY INTERNATIONAL

LA English

DT Article

DE cannabinoid receptor 1; esophageal squamous cell carcinoma;

immunohistochemistry; lymph node metastasis; predictive marker

ID MEDIATED APOPTOSIS; CANCER; CB1; EXPRESSION; ACTIVATION; SURVIVAL;

PROGRESSION; RECURRENCE; GROWTH

AB In patients with esophageal squamous cell carcinoma (ESCC), the status of metastasis to lymph nodes is strongly associated with prognosis. Consequently, development of a biomarker to detect the presence of metastasis would be clinically valuable. In this study, we found that overexpression of cannabinoid receptor 1 (CB1R) was applicable as a marker for prediction of metastasis in ESCC. CB1R overexpression was detected immunohistochemically in 54 of 88 cases (61.4%). The intensity of CB1R expression was uniform in both intraepithelial and invasive regions in each case, and was significantly correlated with the status of metastasis to lymph nodes (P = 0.046) and distant organs (P = 0.047). Furthermore, multivariate analysis revealed that CB1R overexpression was independently associated with poor prognosis (P = 0.019). Biological analysis of CB1R overexpression using ESCC cell lines revealed that CB1R activation appeared to promote cell proliferation and invasion. On the basis of these findings, we propose that evaluation of CB1R expression status in biopsy specimens of ESCC using immunohistochemistry might be clinically useful for prediction of metastasis to lymph nodes and distant organs.

C1 [Hijiya, Naoki; Uchida, Tomohisa; Tsukamoto, Yoshiyuki; Nakada, Chisato; Moriyama, Masatsugu] Oita Univ, Dept Mol Pathol, Fac Med, 1-1 Idaigaoka,Hasama Machi, Yufu City, Oita 8795593, Japan.

[Shibata, Tomotaka; Inomata, Masafumi] Oita Univ, Dept Gastroenterol & Pediat Surg, Fac Med, 1-1 Idaigaoka,Hasama Machi, Yufu City, Oita, Japan.

[Daa, Tsutomu] Oita Univ, Dept Diagnost Pathol, Fac Med, 1-1 Idaigaoka,Hasama Machi, Yufu City, Oita, Japan.

[Hamanaka, Ryoji] Oita Univ Nursing & Hlth Sci, Dept Hlth Sci, 2944-9 Megusuno, Oita, Oita 29449, Japan.

[Matsuura, Keiko] Oita Univ, Dept Biol, Fac Med, 1-1 Idaigaoka,Hasama Machi, Yufu City, Oita, Japan.

[Iha, Hidekatsu] Oita Univ, Dept Microbiol, Fac Med, 1-1 Idaigaoka,Hasama Machi, Yufu City, Oita, Japan.

RP Hijiya, N (corresponding author), Oita Univ, Dept Mol Pathol, Fac Med, 1-1 Idaigaoka,Hasama Machi, Yufu City, Oita 8795593, Japan.

EM hijiya@oita-u.ac.jp

OI Iha, Hidekatsu/0000-0002-0999-5636

FU JSPS KAKENHI [25460419]

FX We thank Ms Yoko Miyanari (Department of Surgery II, Oita University

Faculty of Medicine) for her technical assistance. We would like to

dedicate this manuscript to the memory of Dr. Tsuyoshi Noguchi, former

associate professor of the Department of Gastroenterological and

Pediatric Surgery, Oita University, who passed away before publication.

This work was supported in part by JSPS KAKENHI Grant Number 25460419.

CR Akutsu Y, 2013, ANN SURG, V257, P1032, DOI 10.1097/SLA.0b013e31827017fc

Akutsu Y, 2011, SURG TODAY, V41, P1190, DOI 10.1007/s00595-011-4542-y

Baba Y, 2014, WORLD J GASTRO ONCOL, V6, P121, DOI 10.4251/wjgo.v6.i5.121

Chakravarti B, 2014, ONCOTARGET, V5, P5852, DOI 10.18632/oncotarget.2233

Chung SC, 2009, EUR J CANCER, V45, P174, DOI 10.1016/j.ejca.2008.10.010

GERARD CM, 1991, BIOCHEM J, V279, P129, DOI 10.1042/bj2790129

Goan YG, 2005, ANN THORAC SURG, V80, P1007, DOI 10.1016/j.athoracsur.2005.03.050

Greenhough A, 2007, INT J CANCER, V121, P2172, DOI 10.1002/ijc.22917

Gustafsson K, 2006, MOL PHARMACOL, V70, P1612, DOI 10.1124/mol.106.025981

Gustafsson SB, 2011, PLOS ONE, V6, DOI 10.1371/journal.pone.0023003

Hall W, 2005, LANCET ONCOL, V6, P35, DOI 10.1016/S1470-2045(05)70024-3

Hijiya N, 2008, HUM PATHOL, V39, P316, DOI 10.1016/j.humpath.2007.10.027

Hijiya N, 2016, CANCER RES, V76, P2612, DOI 10.1158/0008-5472.CAN-15-1846

Jung CK, 2013, ONCOL LETT, V5, P870, DOI 10.3892/ol.2012.1081

Kato H, 2005, J SURG ONCOL, V90, P89, DOI 10.1002/jso.20249

Kodama M, 1998, SURGERY, V123, P432, DOI 10.1016/S0039-6060(98)70165-5

Malfitano AM, 2012, PHARMACOL RES, V65, P365, DOI 10.1016/j.phrs.2011.11.008

Marshall AD, 2011, CANCER RES, V71, P7471, DOI 10.1158/0008-5472.CAN-11-0924

MATSUDA LA, 1990, NATURE, V346, P561, DOI 10.1038/346561a0

Michalski CW, 2008, INT J CANCER, V122, P742, DOI 10.1002/ijc.23114

Mukhopadhyay B, 2015, HEPATOLOGY, V61, P1615, DOI 10.1002/hep.27686

NISHIHIRA T, 1993, J CANCER RES CLIN, V119, P441, DOI 10.1007/BF01215923

NISHIHIRA T, 1979, GANN, V70, P575

Sanchez MG, 2003, CELL SIGNAL, V15, P851, DOI 10.1016/S0898-6568(03)00036-6

Sarfaraz S, 2006, J BIOL CHEM, V281, P39480, DOI 10.1074/jbc.M603495200

Sarnataro D, 2005, FEBS LETT, V579, P6343, DOI 10.1016/j.febslet.2005.10.016

Sarnataro D, 2006, MOL PHARMACOL, V70, P1298, DOI 10.1124/mol.106.025601

Tachimori Y, 2014, ESOPHAGUS-TOKYO, V11, P21, DOI 10.1007/s10388-013-0393-5

Takubo K, 2009, ESOPHAGUS-TOKYO, V6, P1, DOI 10.1007/s10388-009-0169-0

Wang DZ, 2008, CANCER RES, V68, P6468, DOI 10.1158/0008-5472.CAN-08-0896

Xu XL, 2015, PLOS ONE, V10, DOI 10.1371/journal.pone.0133076

Xu XD, 2006, CANCER GENET CYTOGEN, V171, P31, DOI 10.1016/j.cancergencyto.2006.06.014

Yamamoto H, 2001, CLIN CANCER RES, V7, P896

Yamamoto H, 2001, CANCER-AM CANCER SOC, V91, P1324, DOI 10.1002/1097-0142(20010401)91:7<1324::AID-CNCR1135>3.0.CO;2-2

NR 34

TC 12

Z9 14

U1 0

U2 1

PU WILEY

PI HOBOKEN

PA 111 RIVER ST, HOBOKEN 07030-5774, NJ USA

SN 1320-5463

EI 1440-1827

J9 PATHOL INT

JI Pathol. Int.

PD FEB

PY 2017

VL 67

IS 2

BP 83

EP 90

DI 10.1111/pin.12495

PG 8

WC Pathology

WE Science Citation Index Expanded (SCI-EXPANDED)

SC Pathology

GA EK4JD

UT WOS:000393892000003

PM 27976824

DA 2022-05-19

ER

PT J

AU Li, LY

Jiang, H

Xie, YM

Liao, LD

Cao, HH

Xu, XE

Chen, B

Zeng, FM

Zhang, YL

Du, ZP

Chen, H

Huang, W

Jia, W

Zheng, W

Xie, JJ

Li, EM

Xu, LY

AF Li, Li-Yan

Jiang, Hong

Xie, Yang-Min

Liao, Lian-Di

Cao, Hui-Hui

Xu, Xiu-E

Chen, Bo

Zeng, Fa-Min

Zhang, Ying-Li

Du, Ze-Peng

Chen, Hong

Huang, Wei

Jia, Wei

Zheng, Wei

Xie, Jian-Jun

Li, En-Min

Xu, Li-Yan

TI Macrolide analog F806 suppresses esophageal squamous cell carcinoma

(ESCC) by blocking beta 1 integrin activation

SO ONCOTARGET

LA English

DT Article

DE macrolide analog F806; beta 1 integrin; cell adhesion; anoikis;

esophageal squamous cell carcinoma cells

ID BREAST-CANCER CELLS; ANTIANGIOGENIC THERAPY; INTEGRIN ACTIVATION;

INDUCED APOPTOSIS; PLUS IRINOTECAN; RESISTANCE; PROLIFERATION;

BEVACIZUMAB; TRANSDUCTION; STATISTICS

AB The paucity of new drugs for the treatment of esophageal squamous cell carcinoma (ESCC) limits the treatment options. This study characterized the therapeutic efficacy and action mechanism of a novel natural macrolide compound F806 in human ESCC xenograft models and cell lines. F806 inhibited growth of ESCC, most importantly, it displayed fewer undesirable side effects on normal tissues in two human ESCC xenograft models. F806 inhibited proliferation of six ESCC cells lines, with the half maximal inhibitory concentration (IC50) ranging from 9.31 to 16.43 mu M. Furthermore, F806 induced apoptosis of ESCC cells, contributing to its growth-inhibitory effect. Also, F806 inhibited cell adhesion resulting in anoikis. Mechanistic studies revealed that F806 inhibited the activation of beta 1 integrin in part by binding to a novel site Arg610 of beta 1 integrin, suppressed focal adhesion formation, decreased cell adhesion to extracellular matrix and eventually triggered apoptosis. We concluded that F806 would potentially be a well-tolerated anticancer drug by targeting beta 1 integrin, resulting in anoikis in ESCC cells.

C1 [Li, Li-Yan; Liao, Lian-Di; Cao, Hui-Hui; Xu, Xiu-E; Chen, Bo; Zeng, Fa-Min; Zhang, Ying-Li; Du, Ze-Peng; Xie, Jian-Jun; Li, En-Min; Xu, Li-Yan] Shantou Univ, Coll Med, Key Lab Mol Biol High Canc Incidence Coastal Chao, Shantou, Guangdong, Peoples R China.

[Li, Li-Yan; Liao, Lian-Di; Cao, Hui-Hui; Xu, Xiu-E; Chen, Bo; Zhang, Ying-Li; Du, Ze-Peng; Xu, Li-Yan] Shantou Univ, Inst Oncol Pathol, Coll Med, Shantou, Guangdong, Peoples R China.

[Jiang, Hong; Chen, Hong; Huang, Wei; Jia, Wei; Zheng, Wei] Fujian Inst Microbiol, Fujian Key Lab Screening Novel Microbial Prod, Fuzhou, Fujian, Peoples R China.

[Xie, Yang-Min] Shantou Univ, Coll Med, Expt Anim Ctr, Shantou, Guangdong, Peoples R China.

[Zeng, Fa-Min; Xie, Jian-Jun; Li, En-Min] Shantou Univ, Coll Med, Dept Biochem & Mol Biol, Shantou, Guangdong, Peoples R China.

RP Xu, LY (corresponding author), Shantou Univ, Coll Med, Key Lab Mol Biol High Canc Incidence Coastal Chao, Shantou, Guangdong, Peoples R China.

EM nmli@stu.edu.cn; lyxu@stu.edu.cn

FU National 11th Five-Year Plan major scientific and technological issues

of China [2009ZX9302-004]; Natural Science Foundation of China-Guangdong

Joint Fund [U0932001, U1301227]; National Natural Science Foundation of

China [81172264, 81472613, 81472342]

FX This work was supported by grants from the National 11th Five-Year Plan

major scientific and technological issues of China (No. 2009ZX9302-004),

the Natural Science Foundation of China-Guangdong Joint Fund (No.

U0932001 and No. U1301227) and the National Natural Science Foundation

of China (No. 81172264, No. 81472613 and No. 81472342).

CR Adjei AA, 2005, J CLIN ONCOL, V23, P5386, DOI 10.1200/JCO.2005.23.648

Aoudjit F, 2001, ONCOGENE, V20, P4995, DOI 10.1038/sj.onc.1204554

Azam F, 2010, EUR J CANCER, V46, P1323, DOI 10.1016/j.ejca.2010.02.020

Barkan D, 2011, CLIN CANCER RES, V17, P7219, DOI 10.1158/1078-0432.CCR-11-0642

Bergers G, 2008, NAT REV CANCER, V8, P592, DOI 10.1038/nrc2442

Calderwood DA, 2004, J CELL SCI, V117, P657, DOI 10.1242/jcs.01014

Carbonell WS, 2013, CANCER RES, V73, P3145, DOI 10.1158/0008-5472.CAN-13-0011

Chen Hong, 2012, Zhongguo Kangshengsu Zazhi, V37, P45

Cunningham D, 2004, NEW ENGL J MED, V351, P337, DOI 10.1056/NEJMoa033025

Ebos JML, 2009, CLIN CANCER RES, V15, P5020, DOI 10.1158/1078-0432.CCR-09-0095

Ekman S, 2008, EXPERT REV ANTICANC, V8, P1433, DOI 10.1586/14737140.8.9.1433

ELLIOTT BE, 1994, CELL ADHES COMMUN, V1, P319, DOI 10.3109/15419069409097263

Frisch SM, 1997, CURR OPIN CELL BIOL, V9, P701, DOI 10.1016/S0955-0674(97)80124-X

FRISCH SM, 1994, J CELL BIOL, V124, P619, DOI 10.1083/jcb.124.4.619

Frisch SM, 2001, CURR OPIN CELL BIOL, V13, P555, DOI 10.1016/S0955-0674(00)00251-9

Gahmberg CG, 2009, BBA-GEN SUBJECTS, V1790, P431, DOI 10.1016/j.bbagen.2009.03.007

Giancotti FG, 1999, SCIENCE, V285, P1028, DOI 10.1126/science.285.5430.1028

Hanahan D, 2011, CELL, V144, P646, DOI 10.1016/j.cell.2011.02.013

Huang W, 2014, MOL CANCER, V13, DOI 10.1186/1476-4598-13-150

Huang Wei, 2011, Zhongguo Kangshengsu Zazhi, V36, P502

Hurwitz H, 2004, NEW ENGL J MED, V350, P2335, DOI 10.1056/NEJMoa032691

HYNES RO, 1992, CELL, V69, P11, DOI 10.1016/0092-8674(92)90115-S

Hynes RO, 2002, CELL, V110, P673, DOI 10.1016/S0092-8674(02)00971-6

Ip CKM, 2014, ONCOTARGET, V5, P9133

Jahangiri A, 2014, CANCER RES, V74, P3, DOI 10.1158/0008-5472.CAN-13-1742

Jemal A, 2009, CA-CANCER J CLIN, V59, P225, DOI 10.3322/caac.20006

Kanda R, 2013, CANCER RES, V73, P6243, DOI 10.1158/0008-5472.CAN-12-4502

Kienast Y, 2010, EXPERT REV ANTICANC, V10, P1763, DOI 10.1586/ERA.10.165

Luo BH, 2006, CURR OPIN CELL BIOL, V18, P579, DOI 10.1016/j.ceb.2006.08.005

Ma WW, 2009, CA-CANCER J CLIN, V59, P111, DOI 10.3322/caac.20003

Margadant C, 2011, CURR OPIN CELL BIOL, V23, P607, DOI 10.1016/j.ceb.2011.08.005

Moore MJ, 2007, J CLIN ONCOL, V25, P1960, DOI 10.1200/JCO.2006.07.9525

Moser M, 2009, SCIENCE, V324, P895, DOI 10.1126/science.1163865

Oinuma I, 2006, J CELL BIOL, V173, P601, DOI 10.1083/jcb.200508204

Park CC, 2006, CANCER RES, V66, P1526, DOI 10.1158/0008-5472.CAN-05-3071

Parkin DM, 2005, CA-CANCER J CLIN, V55, P74, DOI 10.3322/canjclin.55.2.74

Reardon David A, 2011, Genes Cancer, V2, P1159, DOI 10.1177/1947601912450586

Reddig PJ, 2005, CANCER METAST REV, V24, P425, DOI 10.1007/s10555-005-5134-3

Schwartz MA, 2001, J CELL SCI, V114, P2553

Serini G, 2003, NATURE, V424, P391, DOI 10.1038/nature01784

Shattil SJ, 2010, NAT REV MOL CELL BIO, V11, P288, DOI 10.1038/nrm2871

Vredenburgh JJ, 2007, CLIN CANCER RES, V13, P1253, DOI 10.1158/1078-0432.CCR-06-2309

WESTLEY JW, 1979, J ANTIBIOT, V32, P874, DOI 10.7164/antibiotics.32.874

Wu YJ, 2002, J BIOL CHEM, V277, P12294, DOI 10.1074/jbc.M110748200

Xie JJ, 2010, AM J PATHOL, V176, P939, DOI 10.2353/ajpath.2010.090118

Zhang X, 2014, ONCOTARGET

NR 46

TC 7

Z9 7

U1 1

U2 3

PU IMPACT JOURNALS LLC

PI ORCHARD PARK

PA 6666 E QUAKER ST, STE 1, ORCHARD PARK, NY 14127 USA

EI 1949-2553

J9 ONCOTARGET

JI Oncotarget

PD JUN 30

PY 2015

VL 6

IS 18

BP 15940

EP 15952

DI 10.18632/oncotarget.3612

PG 13

WC Oncology; Cell Biology

WE Science Citation Index Expanded (SCI-EXPANDED)

SC Oncology; Cell Biology

GA CO2UL

UT WOS:000359012000038

PM 25909284

OA Green Published, Green Submitted, gold

DA 2022-05-19

ER

PT J

AU Li, KK

Mao, CY

Ma, Q

Bao, T

Wang, YJ

Guo, W

Zhao, XL

AF Li, Kun-Kun

Mao, Cheng-Yi

Ma, Qiang

Bao, Tao

Wang, Ying-Jian

Guo, Wei

Zhao, Xiao-Long

TI U three protein 14a (UTP14A) promotes tumour proliferation and

metastasis via the PERK/eIF2a/GRP78 signalling pathway in oesophageal

squamous cell carcinoma

SO JOURNAL OF CANCER

LA English

DT Article

DE UTP14A; PERK; ESCC; metastasis

ID HEPATOCELLULAR-CARCINOMA; RESISTANCE; EXPRESSION; PLAYS

AB Metastasis and malignant proliferation are major obstacles to the treatment of oesophageal squamous cell carcinoma (ESCC), and UTP14A is associated with poor prognosis in ESCC. However, its mechanisms have not been fully elucidated. The TCGA and GEO databases were used to identify candidate target genes and possible downstream targets. Then, the effects were determined in vitro and in vivo through knockdown and overexpression techniques, and the mechanism was explored. UTP14A was significantly higher in the tumour tissue of ESCC patients than in normal tissue. Knockdown of UTP14A significantly suppressed the migration and proliferation of ESCC cells. The PERK/eIF2a signalling pathway was positively regulated by UTP14A, and its tumour-promoting effect was further activated by overexpression of UTP14A. In conclusion, UTP14A might promote the proliferation and metastasis of ESCC cells by inducing PERK/eIF2a signalling pathway expression.

C1 [Li, Kun-Kun; Bao, Tao; Wang, Ying-Jian; Guo, Wei] Army Med Univ, Daping Hosp, Dept Thorac Surg, Chongqing 400042, Peoples R China.

[Mao, Cheng-Yi; Ma, Qiang; Zhao, Xiao-Long] Army Med Univ, Daping Hosp, Dept Pathol, Chongqing, Peoples R China.

RP Zhao, XL (corresponding author), Army Med Univ, Daping Hosp, Dept Thorac Surg, Chongqing 400042, Peoples R China.

EM xiaolong.zhao@tmmu.edu.cn

FU National Natural Science Foundation of China [81071976]

FX This study was supported by the National Natural Science Foundation of

China (No. 81071976).

CR Batra R, 2019, SURG CLIN N AM, V99, P529, DOI 10.1016/j.suc.2019.02.006

Celada LJ, 2018, SCI TRANSL MED, V10, DOI 10.1126/scitranslmed.aar8356

Chen J, 2018, CANCER MED-US, V7, P1394, DOI 10.1002/cam4.1326

Duitman J, 2019, J CLIN MED, V8, DOI 10.3390/jcm8101547

Egyud MR, 2019, SURG CLIN N AM, V99, P419, DOI 10.1016/j.suc.2019.02.002

Hetz C, 2013, NAT REV DRUG DISCOV, V12, P703, DOI 10.1038/nrd3976

Hu LL, 2011, J BIOL CHEM, V286, P3119, DOI 10.1074/jbc.M110.157842

Li KK, 2019, THORAC CANCER, V10, P2071, DOI 10.1111/1759-7714.13176

Li LJ, 2018, J CANCER, V9, P4314, DOI 10.7150/jca.27943

Li Q, 2018, CELL DEATH DIS, V9, DOI 10.1038/s41419-018-0452-x

Ma T, 2017, BIOL CHEM, V398, P1247, DOI 10.1515/hsz-2017-0121

Ma YJ, 2004, NAT REV CANCER, V4, P966, DOI 10.1038/nrc1505

Shi WM, 2019, J CANCER, V10, P2035, DOI 10.7150/jca.29421

Zeng HM, 2015, INT J CANCER, V136, P1921, DOI 10.1002/ijc.29227

Zhang JY, 2017, CHINESE MED J-PEKING, V130, P470, DOI 10.4103/0366-6999.199839

Zhang JY, 2019, CANCER LETT, V440, P106, DOI 10.1016/j.canlet.2018.10.010

Zhu JY, 2016, MOL CELL BIOL, V36, P965, DOI 10.1128/MCB.00773-15

NR 17

TC 1

Z9 1

U1 2

U2 4

PU IVYSPRING INT PUBL

PI LAKE HAVEN

PA PO BOX 4546, LAKE HAVEN, NSW 2263, AUSTRALIA

SN 1837-9664

J9 J CANCER

JI J. Cancer

PY 2021

VL 12

IS 1

BP 134

EP 140

DI 10.7150/jca.44649

PG 7

WC Oncology

WE Science Citation Index Expanded (SCI-EXPANDED)

SC Oncology

GA OO6DB

UT WOS:000587466800013

PM 33391409

OA Green Published, gold

DA 2022-05-19

ER

PT J

AU Ni, PZ

He, JZ

Wu, ZY

Ji, X

Chen, LQ

Xu, XE

Liao, LD

Wu, JY

Li, EM

Xu, LY

AF Ni, Peng-Zhi

He, Jian-Zhong

Wu, Zhi-Yong

Ji, Xiang

Chen, Long-Qi

Xu, Xiu-E

Liao, Lian-Di

Wu, Jian-Yi

Li, En-Min

Xu, Li-Yan

TI Overexpression of Stathmin 1 correlates with poor prognosis and promotes

cell migration and proliferation in oesophageal squamous cell carcinoma

SO ONCOLOGY REPORTS

LA English

DT Article

DE STMN1; prognosis; migration; invasion; proliferation; oesophageal

squamous cell carcinoma

ID BREAST-CANCER; LUNG-CANCER; EXPRESSION; RECURRENCE; INVASION; EZRIN

AB Stathmin 1 (STMN1) is a microtubule-regulated protein that plays an important role in tumour cell proliferation and migration. Overexpression of STMN1 is associated with clinicopathological characteristics in many human cancers. The aim of the present study was to investigate STMN1 expression, its correlation with clinicopathological characteristics, and its exact biological function in oesophageal squamous cell carcinoma (ESCC). STMN1 levels were measured in the ESCC tissue specimens of 276 patients by immunohistochemistry (IHC) to assess the prognostic efficacy of STMN1. IHC showed that patients with overexpression of STMN1 had a poorer prognosis compared with those with low expression, both in regards to 5-year overall survival (OS; 21.2 vs. 53.7%, P<0.001) and disease-free survival (DFS; 20.6 vs. 50.9%, P<0.001). STMN1 overexpression was associated with lower cell differentiation in tumour grade (correlation coefficient: 0.127, P=0.037). In multivariate analysis, STMN1 expression was found to be an independent prognostic factor for both OS (P<0.001; 95% CI, 1.555-2.970) and DFS (P=0.001; 95% CI, 1.978-2.444). Compared with the control, STMN1 downregulation significantly decreased cell migration, invasion and proliferation, whereas these were increased by STMN1 upregulation. STMN1 expression was significantly associated with prognosis and tumour differentiation in ESCC, indicating that STMN1 expression is an independent prognostic factor for ESCC and could be a potential biomarker. Regulating the expression of STMN1 could influence tumour cell motility, invasion and proliferation.

C1 [Ni, Peng-Zhi; Ji, Xiang; Chen, Long-Qi] Sichuan Univ, West China Hosp, Dept Thorac Surg, 37 Guoxue Alley, Chengdu 610041, Sichuan, Peoples R China.

[He, Jian-Zhong; Xu, Xiu-E; Liao, Lian-Di; Wu, Jian-Yi; Li, En-Min; Xu, Li-Yan] Shantou Univ, Med Coll, Key Lab Mol Biol High Canc Incidence Coastal Chao, Shantou 515041, Guangdong, Peoples R China.

[He, Jian-Zhong; Xu, Xiu-E; Liao, Lian-Di; Xu, Li-Yan] Shantou Univ, Med Coll, Inst Oncol Pathol, Shantou 515041, Guangdong, Peoples R China.

[Li, En-Min] Shantou Univ, Med Coll, Dept Biochem & Mol Biol, Shantou 515041, Guangdong, Peoples R China.

[Wu, Zhi-Yong; Wu, Jian-Yi] Sun Yat Sen Univ, Affiliated Shantou Hosp, Shantou Cent Hosp, Dept Oncol Surg, Shantou 515041, Guangdong, Peoples R China.

RP Chen, LQ (corresponding author), Sichuan Univ, West China Hosp, Dept Thorac Surg, 37 Guoxue Alley, Chengdu 610041, Sichuan, Peoples R China.; Xu, LY (corresponding author), Shantou Univ, Med Coll, Key Lab Mol Biol High Canc Incidence Coastal Chao, Shantou 515041, Guangdong, Peoples R China.

EM drchenlq@scu.edu.cn; lyxu@stu.edu.cn

OI He, Jian-Zhong/0000-0003-1256-472X

FU National Natural Science Foundation of China [30770982, 81360331,

81472613, 81572341]; Natural Science Foundation of China-Guangdong Joint

Fund [U1301227, U0932001]; Science and Technology Planning Project of

Guang Dong Province [2014A030304060]; Department of Education, Guangdong

Government under the Top-Tier University Development Scheme for Research

and Control of Infectious Diseases

FX The present study was supported by the National Natural Science

Foundation of China (grant nos. 30770982, 81360331, 81472613 and

81572341), the Natural Science Foundation of China-Guangdong Joint Fund

(grant nos. U1301227 and U0932001), and the Science and Technology

Planning Project of Guang Dong Province (grant no. 2014A030304060), and

the Department of Education, Guangdong Government under the Top-Tier

University Development Scheme for Research and Control of Infectious

Diseases.

CR Akhtar J, 2014, J GASTROEN HEPATOL, V29, P944, DOI 10.1111/jgh.12498

Baquero MT, 2012, CANCER-AM CANCER SOC, V118, P4660, DOI 10.1002/cncr.27453

Chen Y, 2017, INT J ONCOL, V50, P31, DOI 10.3892/ijo.2016.3778

Enzinger PC, 2003, NEW ENGL J MED, V349, P2241, DOI 10.1056/NEJMra035010

Flejou Jean-Francois, 2011, Ann Pathol, V31, pS27, DOI 10.1016/j.annpat.2011.08.001

Gan L, 2010, ONCOL REP, V23, P1037, DOI 10.3892/or_00000730

Ghosh R, 2007, PROSTATE, V67, P1038, DOI 10.1002/pros.20601

Hsieh SY, 2010, MOL CARCINOGEN, V49, P476, DOI 10.1002/mc.20627

Ke B, 2013, TUMOR BIOL, V34, P3137, DOI 10.1007/s13277-013-0882-0

Kollarova Helena, 2007, Biomedical Papers (Olomouc), V151, P17

Kuang XY, 2015, ONCOTARGET, V6, P22227, DOI 10.18632/oncotarget.4276

Li J, 2015, PATHOL ONCOL RES, V21, P1013, DOI 10.1007/s12253-015-9930-y

Li XY, 2015, PATHOL RES PRACT, V211, P816, DOI 10.1016/j.prp.2015.07.009

Liu P, 2013, ONCOL REP, V29, P419, DOI 10.3892/or.2012.2157

Lu Y, 2014, CURR MOL MED, V14, P328, DOI 10.2174/1566524014666140228120913

Ma HL, 2016, J CRANIO MAXILL SURG, V44, P1725, DOI 10.1016/j.jcms.2016.07.033

Machado-Neto JA, 2014, BMB REP, V47, P660, DOI 10.5483/BMBRep.2014.47.12.020

Nie W, 2015, LAB INVEST, V95, P56, DOI 10.1038/labinvest.2014.124

Pennathur A, 2013, LANCET, V381, P400, DOI 10.1016/S0140-6736(12)60643-6

Rana S, 2008, EXPERT REV ANTICANC, V8, P1461, DOI 10.1586/14737140.8.9.1461

Rice TW, 2010, ANN SURG ONCOL, V17, P1721, DOI 10.1245/s10434-010-1024-1

Rubin CI, 2004, J CELL BIOCHEM, V93, P242, DOI 10.1002/jcb.20187

Sherbet G. V., 2005, Cancer Genomics & Proteomics, V2, P227

Sun LL, 2013, AM J CANCER RES, V3, P509

Sun RF, 2015, TUMOR BIOL, V36, P7195, DOI 10.1007/s13277-015-3361-y

Tian FJ, 2015, AM J PATHOL, V185, P2709, DOI 10.1016/j.ajpath.2015.06.010

Wang Feng, 2010, Nan Fang Yi Ke Da Xue Xue Bao, V30, P1552

Wei SH, 2008, ACTA HISTOCHEM, V110, P59, DOI 10.1016/j.acthis.2007.06.002

Xie JJ, 2011, J HISTOCHEM CYTOCHEM, V59, P1001, DOI 10.1369/0022155411418661

Xie JJ, 2011, J SURG ONCOL, V104, P538, DOI 10.1002/jso.21909

Xie JJ, 2010, AM J PATHOL, V176, P939, DOI 10.2353/ajpath.2010.090118

Zhang Hui-Zhong, 2004, Ai Zheng, V23, P493

NR 32

TC 16

Z9 17

U1 1

U2 5

PU SPANDIDOS PUBL LTD

PI ATHENS

PA POB 18179, ATHENS, 116 10, GREECE

SN 1021-335X

EI 1791-2431

J9 ONCOL REP

JI Oncol. Rep.

PD DEC

PY 2017

VL 38

IS 6

BP 3608

EP 3618

DI 10.3892/or.2017.6039

PG 11

WC Oncology

WE Science Citation Index Expanded (SCI-EXPANDED)

SC Oncology

GA FP0TR

UT WOS:000417320500037

PM 29039594

OA Bronze

DA 2022-05-19

ER

PT J

AU Fang, WK

Liao, LD

Li, LY

Xie, YM

Xu, XE

Zhao, WJ

Wu, JY

Zhu, MX

Wu, ZY

Du, ZP

Wu, BL

Xie, D

Guo, MZ

Xu, LY

Li, EM

AF Fang, Wang-Kai

Liao, Lian-Di

Li, Li-Yan

Xie, Yang-Min

Xu, Xiu-E

Zhao, Wei-Jiang

Wu, Jian-Yi

Zhu, Meng-Xiao

Wu, Zhi-Yong

Du, Ze-Peng

Wu, Bing-Li

Xie, Dong

Guo, Ming-Zhou

Xu, Li-Yan

Li, En-Min

TI Down-regulated desmocollin-2 promotes cell aggressiveness through

redistributing adherens junctions and activating beta-catenin signalling

in oesophageal squamous cell carcinoma

SO JOURNAL OF PATHOLOGY

LA English

DT Article

DE DSC2; miR-25; oesophageal squamous cell carcinoma; invasion; E-cadherin

ID E-CADHERIN; EXTRACAPSULAR SPREAD; REDUCED EXPRESSION; CANCER;

DESMOSOMES; PREDICTOR; MICRORNAS; PATTERNS; SURVIVAL; INVASION

AB In contrast to the well-recognized loss of adherens junctions in cancer progression, the role of desmosomal components in cancer development has not been well explored. We previously demonstrated that desmocollin-2 (DSC2), a desmosomal cadherin protein, is reduced in oesophageal squamous cell carcinoma (ESCC), and is associated with enhanced tumour metastasis and poor prognosis. Here, we report that restoration of DSC2 in ESCC cells impeded cell migration and invasion both in vitro and in vivo, whereas siRNA-mediated suppression of DSC2 expression increased cell motility. In E-cadherin-expressing ESCC cells, DSC2 restoration strengthened E-cadherin-mediated adherens junctions and promoted the localization of beta-catenin at these junctions, which indirectly inhibited beta-catenin-dependent transcription. These effects of DSC2 were not present in EC109 cells that lacked E-cadherin expression. ESCC patients with tumours that had reduced E-cadherin and negative DSC2 had poorer clinical outcomes than patients with tumours that lacked either E-cadherin or DSC2, implying that the invasive potential of ESCC cells was restricted by both DSC2 and E-cadherin-dependent junctions. Further studies revealed that DSC2 was a downstream target of miR-25. Enhanced miR-25 promoted ESCC cell invasiveness, whereas restoration of DSC2 abolished these effects. Collectively, our work suggests that miR-25-mediated down-regulation of DSC2 promotes ESCC cell aggressiveness through redistributing adherens junctions and activating beta-catenin signalling. Copyright (c) 2013 Pathological Society of Great Britain and Ireland. Published by John Wiley & Sons, Ltd.

C1 [Fang, Wang-Kai; Wu, Jian-Yi; Wu, Zhi-Yong; Wu, Bing-Li; Li, En-Min] Shantou Univ Med Coll, Dept Biochem & Mol Biol, Shantou 515041, Peoples R China.

[Fang, Wang-Kai; Li, Li-Yan; Zhu, Meng-Xiao; Wu, Bing-Li; Xu, Li-Yan; Li, En-Min] Shantou Univ Med Coll, Key Lab Mol Biol High Canc Incidence Coastal Chao, Shantou 515041, Peoples R China.

[Liao, Lian-Di; Li, Li-Yan; Xu, Xiu-E; Zhu, Meng-Xiao; Du, Ze-Peng; Xu, Li-Yan] Shantou Univ Med Coll, Inst Oncol Pathol, Shantou 515041, Peoples R China.

[Xie, Yang-Min] Shantou Univ Med Coll, Dept Expt Anim Ctr, Shantou 515041, Peoples R China.

[Zhao, Wei-Jiang] Shantou Univ Med Coll, Ctr Neurosci, Shantou 515041, Peoples R China.

[Xie, Dong] Chinese Acad Sci, Inst Nutr Sci, Mol Oncol Lab, Shanghai Inst Biol Sci, Shanghai, Peoples R China.

[Guo, Ming-Zhou] Chinese Peoples Liberat Army Gen Hosp, Dept Gastroenterol & Hepatol, Beijing, Peoples R China.

RP Li, EM (corresponding author), Shantou Univ Med Coll, Dept Biochem & Mol Biol, Key Lab Mol Biol High Canc Incidence Coastal Chao, Shantou 515041, Peoples R China.

EM lyxu@stu.edu.cn; nmli@stu.edu.cn

RI Fang, Wangkai/AAU-8664-2020

OI Fang, Wang-Kai/0000-0002-9417-2422; Xie, Dong/0000-0002-1150-8657

FU National Natural Science Foundation of China [81101613]; Guangdong

Provincial Natural Science Foundation of China [S2011040004363];

Specialized Research Fund for the Doctoral Program of Higher Education

of China [20114402120005]; Natural Science Foundation of China-Guangdong

Joint Fund [U0932001]; National Basic Research Program (973 Program)

[2012CB526608, 2010CB912802]; National High Technology Research and

Development Program of China [2012AA02A503, 2012AA02A209]

FX This work was supported by grants from the National Natural Science

Foundation of China (No 81101613), the Guangdong Provincial Natural

Science Foundation of China (No S2011040004363), the Specialized

Research Fund for the Doctoral Program of Higher Education of China (No

20114402120005), the Natural Science Foundation of China-Guangdong Joint

Fund (No U0932001), the National Basic Research Program (973 Program Nos

2012CB526608 and 2010CB912802), and the National High Technology

Research and Development Program of China (Nos 2012AA02A503 and

2012AA02A209).

CR Bosch FX, 2005, INT J CANCER, V114, P779, DOI 10.1002/ijc.20782

Chan JY, 2011, CANCER SCI, V102, P1855, DOI 10.1111/j.1349-7006.2011.02021.x

Cho WCS, 2007, MOL CANCER, V6, DOI 10.1186/1476-4598-6-60

Chun MGH, 2010, PLOS GENET, V6, DOI 10.1371/journal.pgen.1001120

CICAMS, 1976, CHIN MED J, V2, P357

Conacci-Sorrell ME, 2002, GENE DEV, V16, P2058, DOI 10.1101/gad.227502

Cui L, 2008, CLIN CANCER RES, V14, P7674, DOI 10.1158/1078-0432.CCR-08-0420

de Castro J, 2000, VIRCHOWS ARCH, V437, P599, DOI 10.1007/s004280000266

Fang WK, 2010, HUM PATHOL, V41, P1456, DOI 10.1016/j.humpath.2010.04.003

Fodde R, 2007, CURR OPIN CELL BIOL, V19, P150, DOI 10.1016/j.ceb.2007.02.007

Garrod D, 2008, BBA-BIOMEMBRANES, V1778, P572, DOI 10.1016/j.bbamem.2007.07.014

Green KJ, 2007, J INVEST DERMATOL, V127, P2499, DOI 10.1038/sj.jid.5701015

Green KJ, 2010, COLD SPRING HARB PER, V2

Gu ZD, 2005, AM J GASTROENTEROL, V100, P1835, DOI 10.1111/j.1572-0241.2005.50018.x

Hamidov Z, 2011, J CLIN PATHOL, V64, P990, DOI 10.1136/jclinpath-2011-200099

Hardman MJ, 2005, MOL CELL BIOL, V25, P969, DOI 10.1128/MCB.25.3.969-978.2005

Hayashi T, 2011, HISTOPATHOLOGY, V59, P710, DOI 10.1111/j.1365-2559.2011.03988.x

Heuberger J., 2010, COLD SPRING HARBOR P, V2

Huen AC, 2002, J CELL BIOL, V159, P1005, DOI 10.1083/jcb.200206098

Imre K, 2008, EUR ARCH OTO-RHINO-L, V265, P337, DOI 10.1007/s00405-007-0464-0

Jeanes A, 2008, ONCOGENE, V27, P6920, DOI 10.1038/onc.2008.343

Kamangar F, 2006, J CLIN ONCOL, V24, P2137, DOI 10.1200/JCO.2005.05.2308

Khan K, 2006, BRIT J CANCER, V95, P1367, DOI 10.1038/sj.bjc.6603453

Kimura Y, 1999, INT J CANCER, V84, P174, DOI 10.1002/(SICI)1097-0215(19990420)84:2<174::AID-IJC14>3.3.CO;2-5

Kolegraff K, 2011, MOL BIOL CELL, V22, P1121, DOI 10.1091/mbc.E10-10-0845

Korinek V, 1997, SCIENCE, V275, P1784, DOI 10.1126/science.275.5307.1784

Mariette C, 2003, CANCER-AM CANCER SOC, V97, P1616, DOI 10.1002/cncr.11228

Markowitz SD, 2009, NEW ENGL J MED, V361, P2449, DOI 10.1056/NEJMra0804588

Miravet S, 2003, MOL CELL BIOL, V23, P7391, DOI 10.1128/MCB.23.20.7391-7402.2003

Mulholland DJ, 2012, CANCER RES, V72, P1878, DOI 10.1158/0008-5472.CAN-11-3132

Ninomiya I, 2000, INT J CANCER, V85, P757, DOI 10.1002/(SICI)1097-0215(20000315)85:6<757::AID-IJC3>3.3.CO;2-F

NUBER UA, 1995, EUR J CELL BIOL, V66, P69

Oshiro MM, 2005, BREAST CANCER RES, V7, pR669, DOI 10.1186/bcr1273

Poliseno L, 2010, SCI SIGNAL, V3, DOI 10.1126/scisignal.2000594

Shen ZY, 2000, J CANCER RES CLIN, V126, P589, DOI 10.1007/PL00008469

SHIMADA Y, 1992, CANCER, V69, P277, DOI 10.1002/1097-0142(19920115)69:2<277::AID-CNCR2820690202>3.0.CO;2-C

Suh SS, 2012, P NATL ACAD SCI USA, V109, P5316, DOI 10.1073/pnas.1202465109

Takayama N, 2003, ANTICANCER RES, V23, P4435

Takeno S, 2004, AM J CLIN PATHOL, V122, P78, DOI 10.1309/WJL90JPEM17RBUHT

Valastyan S, 2011, J CELL SCI, V124, P999, DOI 10.1242/jcs.081513

van Zijl F, 2011, MUTAT RES-REV MUTAT, V728, P23, DOI 10.1016/j.mrrev.2011.05.002

Vasioukhin V, 2001, NAT CELL BIOL, V3, P1076, DOI 10.1038/ncb1201-1076

Wijnhoven BPL, 2000, BRIT J SURG, V87, P992, DOI 10.1046/j.1365-2168.2000.01513.x

Wu BL, 2011, WORLD J GASTROENTERO, V17, P79, DOI 10.3748/wjg.v17.i1.79

Xie JJ, 2010, AM J PATHOL, V176, P939, DOI 10.2353/ajpath.2010.090118

Xu XH, 2012, BIOCHEM BIOPH RES CO, V421, P640, DOI 10.1016/j.bbrc.2012.03.048

Zhang G, 2005, PATHOL INT, V55, P310, DOI 10.1111/j.1440-1827.2005.01840.x

Zhang JS, 2012, CANCER METAST REV, V31, P653, DOI 10.1007/s10555-012-9368-6

Zoumalan RA, 2010, J LARYNGOL OTOL, V124, P1284, DOI 10.1017/S0022215110001453

NR 49

TC 42

Z9 44

U1 1

U2 13

PU WILEY

PI HOBOKEN

PA 111 RIVER ST, HOBOKEN 07030-5774, NJ USA

SN 0022-3417

EI 1096-9896

J9 J PATHOL

JI J. Pathol.

PD OCT

PY 2013

VL 231

IS 2

BP 257

EP 270

DI 10.1002/path.4236

PG 14

WC Oncology; Pathology

WE Science Citation Index Expanded (SCI-EXPANDED)

SC Oncology; Pathology

GA 213XC

UT WOS:000324092800012

PM 23836524

DA 2022-05-19

ER

PT J

AU Barclay, ME

Lyratzopoulos, G

Walter, FM

Jefferies, S

Peake, MD

Rintoul, RC

AF Barclay, Matthew E.

Lyratzopoulos, Georgios

Walter, Fiona M.

Jefferies, Sarah

Peake, Michael D.

Rintoul, Robert C.

TI Incidence of second and higher order smoking-related primary cancers

following lung cancer: a population-based cohort study

SO THORAX

LA English

DT Article

ID PRIMARY MALIGNANCIES; RISK; SURVIVORS

AB Background Lung cancer 5-year survival has doubled over 15 years. Although the risk of second primary cancer is recognised, quantification over time is lacking. We describe the incidence of second and higher order smoking-related primary cancers in lung cancer survivors, identifying high-incidence groups and how incidence changes over time from first diagnosis.

Methods Data on smoking-related primary cancers (lung, laryngeal, head and neck, oesophageal squamous cell carcinoma and bladder) diagnosed in England between 2000 and 2014 were obtained from Public Health England National Cancer Registration and Analysis Service. We calculated absolute incidence rates and standardised incidence rate ratios, both overall and for various subgroups of second primary cancer for up to 10 years from the initial diagnosis of lung cancer, using Poisson regression.

Results Elevated incidence of smoking-related second primary cancer persists for at least 10 years from first lung cancer diagnosis with those aged 50 and 79 at first diagnosis at particularly high risk. The most frequent type of second malignancy was lung cancer although the highest standardised incidence rate ratios were for oesophageal squamous cell carcinoma (2.4) and laryngeal cancers (2.8) and consistently higher in women than in men. Over the last decade, the incidence of second primary lung cancer has doubled.

Conclusion Lung cancer survivors have increased the incidence of subsequent lung, laryngeal, head and neck and oesophageal squamous cell carcinoma for at least a decade from the first diagnosis. Consideration should be given to increasing routine follow-up from 5 years to 10 years for those at highest risk, alongside surveillance for other smoking-related cancers.

C1 [Barclay, Matthew E.; Lyratzopoulos, Georgios; Walter, Fiona M.] Univ Cambridge, Dept Publ Hlth & Primary Care, Primary Care Unit, Cambridge, Cambs, England.

[Lyratzopoulos, Georgios] UCL, Dept Behav Sci & Hlth, Epidemiol Canc Healthcare & Outcomes, London, England.

[Lyratzopoulos, Georgios; Peake, Michael D.] PHE, NCRAS, London, England.

[Jefferies, Sarah] Cambridge Univ Hosp NHS Fdn Trust, Dept Oncol, Cambridge, England.

[Peake, Michael D.] Univ Hosp Leicester NHS Trust, Dept Resp Med, Leicester, Leics, England.

[Rintoul, Robert C.] Univ Cambridge, Dept Oncol, Cambridge CB2 0RE, England.

[Rintoul, Robert C.] Royal Papworth Hosp NHS Fdn Trust, Dept Thorac Oncol, Cambridge, England.

RP Rintoul, RC (corresponding author), Univ Cambridge, Dept Oncol, Cambridge CB2 0RE, England.

EM robert.rintoul@nhs.net

RI Walter, Fiona/F-3054-2014; Barclay, Matthew Edward/Q-2626-2019; Rintoul,

Robert Campbell/Q-1727-2015

OI Barclay, Matthew Edward/0000-0003-1148-1922; Rintoul, Robert

Campbell/0000-0003-3875-3780; Walter, Fiona/0000-0002-7191-6476;

Lyratzopoulos, Georgios/0000-0002-2873-7421

FU Early Diagnosis programme Cancer Research UK Cambridge Centre; NIHR

Clinician Scientist award; Cancer Research UK [C18081/A18180]; Cambridge

Biomedical Research Centre; Cancer Research UK Cambridge Centre

FX This study was supported the Early Diagnosis programme Cancer Research

UK Cambridge Centre. FMW is supported by an NIHR Clinician Scientist

award. GL is supported by a Cancer Research UK award (Advanced Clinician

Scientist Fellowship C18081/A18180). RCR is part funded by the Cambridge

Biomedical Research Centre and Cancer Research UK Cambridge Centre.

CR Abdel-Rahman O, 2017, CURR MED RES OPIN, V33, P2009, DOI 10.1080/03007995.2017.1333953

AKAIKE H, 1974, IEEE T AUTOMAT CONTR, VAC19, P716, DOI 10.1109/TAC.1974.1100705

Bennette C, 2012, BMC MED RES METHODOL, V12, DOI 10.1186/1471-2288-12-21

Coyte A, 2014, BMC CANCER, V14, DOI 10.1186/1471-2407-14-272

Curado MP, 2005, EUR J CANCER PREV, V14, P307

De Angelis R, 2014, LANCET ONCOL, V15, P23, DOI 10.1016/S1470-2045(13)70546-1

Department for communities and local government, 2015, ENGL IND DEPR 2015

Donin N, 2016, CANCER-AM CANCER SOC, V122, P3075, DOI 10.1002/cncr.30164

Han SS, 2017, J CLIN ONCOL, V35, P2893, DOI 10.1200/JCO.2017.72.4203

Harrell F.E., 2001, REGRESSION MODELING, DOI [10.1007/978-1-4757-3462-1, DOI 10.1007/978-3-319-19425-7]

Jegu J, 2014, BMC CANCER, V14, DOI 10.1186/1471-2407-14-94

Moller H, 2011, BRIT J CANCER, V105, P170, DOI 10.1038/bjc.2011.168

Siegel RL, 2015, JAMA INTERN MED, V175, P1574, DOI 10.1001/jamainternmed.2015.2398

Su VYF, 2017, TARGET ONCOL, V12, P219, DOI 10.1007/s11523-016-0459-0

Surapaneni R, 2012, J THORAC ONCOL, V7, P1252, DOI 10.1097/JTO.0b013e3182582a79

Tabuchi T, 2013, ANN ONCOL, V24, P2699, DOI 10.1093/annonc/mdt279

Tabuchi T, 2012, CANCER SCI, V103, P1111, DOI 10.1111/j.1349-7006.2012.02254.x

Thakur MK, 2018, J THORAC ONCOL, V13, P46, DOI 10.1016/j.jtho.2017.09.1964

Walters S, 2015, BRIT J CANCER, V113, P848, DOI 10.1038/bjc.2015.265

Warren GW, 2014, LANCET ONCOL, V15, pE568, DOI 10.1016/S1470-2045(14)70266-9

Youlden DR, 2011, BMC CANCER, V11, DOI 10.1186/1471-2407-11-83

NR 21

TC 13

Z9 13

U1 0

U2 1

PU BMJ PUBLISHING GROUP

PI LONDON

PA BRITISH MED ASSOC HOUSE, TAVISTOCK SQUARE, LONDON WC1H 9JR, ENGLAND

SN 0040-6376

EI 1468-3296

J9 THORAX

JI Thorax

PD MAY

PY 2019

VL 74

IS 5

BP 466

EP 472

DI 10.1136/thoraxjnl-2018-212456

PG 7

WC Respiratory System

WE Science Citation Index Expanded (SCI-EXPANDED)

SC Respiratory System

GA IC6ZX

UT WOS:000471122800009

PM 30777897

OA Green Published, hybrid

DA 2022-05-19

ER

PT J

AU Chao, YK

Chuang, WY

Yeh, CJ

Chang, HK

Tseng, CK

AF Chao, Yin-Kai

Chuang, Wen-Yu

Yeh, Chi-Ju

Chang, Hsien-Kun

Tseng, Chen-Kan

TI Anatomical distribution of residual cancer in patients with oesophageal

squamous cell carcinoma who achieved clinically complete response after

neoadjuvant chemoradiotherapy

SO EUROPEAN JOURNAL OF CARDIO-THORACIC SURGERY

LA English

DT Article

DE Oesophageal cancer; Residual cancer; Neoadjuvant chemoradiotherapy;

Clinical complete response; Squamous cell carcinoma

ID PATHOLOGICAL RESPONSE; ENDOSCOPIC ULTRASOUND; JUNCTIONAL CANCER;

CHEMORADIATION; THERAPY; BIOPSY; TUMOR; PREDICTION; REGRESSION; CRITERIA

AB OBJECTIVES: Recent advances in neoadjuvant chemoradiotherapy (nCRT) have significantly increased the rates of pathological complete response achieved by patients with oesophageal cancer. Consequently, a watchful waiting strategy based on 'active endoscopic surveillance and surgery as needed' has been proposed for cases without clinical evidence of disease after neoadjuvant chemoradiotherapy. Here, we investigated whether endoscopic surveillance is a reliable tool for the detection of the initially unidentified residual cancer in this patient group.

METHODS: We performed a careful pathological re-review of all cases with oesophageal squamous cell carcinoma, who attained a clinical complete response, despite showing a pathological non-complete response. The detailed anatomical locations of such unidentified malignancies were investigated in each patient to determine the prevalence of cancer involvement for each oesophageal layer.

RESULTS: Among the 73 patients with clinical complete response, 46 (63%) patients were found to have pathological non-complete response. The majority (89.1%; n = 41) of patients had evidence of residual cancer in the oesophagus, whereas only 5 (10.9%) patients had T0N+ disease. However, a high percentage (39.1%; n = 16) of patients had no detectable cancer in the mucosa and 9 of them also had no detectable cancer in sub-mucosal layer, ultimately hampering their detection via endoscopic biopsy.

CONCLUSIONS: Nearly 40% of patients with oesophageal squamous cell carcinoma who attained clinical complete response but showed a pathological non-complete response had residual cancer hidden underneath a cancer-free mucosa layer.

C1 [Chao, Yin-Kai] Chang Gung Univ, Chang Gung Mem Hosp Linkou, Div Thorac Surg, Taoyuan, Taiwan.

[Chuang, Wen-Yu; Yeh, Chi-Ju] Chang Gung Univ, Chang Gung Mem Hosp Linkou, Dept Pathol, Taoyuan, Taiwan.

[Chang, Hsien-Kun] Chang Gung Univ, Chang Gung Mem Hosp Linkou, Div Hematol & Oncol, Taoyuan, Taiwan.

[Tseng, Chen-Kan] Chang Gung Univ, Chang Gung Mem Hosp Linkou, Dept Radiat Oncol, Taoyuan, Taiwan.

RP Chao, YK (corresponding author), Chang Gung Mem Hosp Linkou, Div Thorac Surg, Taoyuan, Taiwan.

EM chaoyk@cgmh.org.tw

RI Chuang, Wen-Yu/I-8013-2019; Chao, YinKai/ABE-7827-2020

OI Chuang, Wen-Yu/0000-0002-3729-073X; Chao, Yin Kai/0000-0001-8649-8783

FU National Science Council, Executive Yuan [NSC 100-2314-B-182 A-018];

Chang Gung Memorial Hospital, Taiwan, Republic of China [NMRPG2A0011]

FX This study was partly supported by grants from the National Science

Council, Executive Yuan [NSC 100-2314-B-182 A-018] and from the Chang

Gung Memorial Hospital, Taiwan, Republic of China [NMRPG2A0011].

CR Akahoshi K, 2007, WORLD J GASTROENTERO, V13, P2077, DOI 10.3748/wjg.v13.i14.2077

Castoro C, 2013, J GASTROINTEST SURG, V17, P1375, DOI 10.1007/s11605-013-2269-3

Chao YK, 2015, MEDICINE, V94, DOI 10.1097/MD.0000000000001407

Eisenhauer EA, 2009, EUR J CANCER, V45, P228, DOI 10.1016/j.ejca.2008.10.026

Hoda KM, 2009, GASTROINTEST ENDOSC, V69, P1218, DOI 10.1016/j.gie.2008.09.045

Komanduri S, 2011, ENDOSCOPY, V43, P849, DOI 10.1055/s-0030-1256650

Krause BJ, 2009, J NUCL MED, V50, p89S, DOI 10.2967/jnumed.108.057232

Kwee RM, 2010, RADIOLOGY, V254, P707, DOI 10.1148/radiol.09091324

Lin D, 2014, SEMIN THORAC CARDIOV, V26, P102, DOI 10.1053/j.semtcvs.2014.07.002

Noordman BJ, 2015, JMIR RES PROTOC, V4, DOI 10.2196/resprot.4320

Piessen G, 2013, ANN SURG, V258, P793, DOI 10.1097/SLA.0000000000000228

Sarkaria IS, 2009, ANN SURG, V249, P764, DOI 10.1097/SLA.0b013e3181a38e9e

Schneider PM, 2008, ANN SURG, V248, P902, DOI 10.1097/SLA.0b013e31818f3afb

Shapiro J, 2015, LANCET ONCOL, V16, P1090, DOI 10.1016/S1470-2045(15)00040-6

Shapiro J, 2013, ANN SURG, V258, P678, DOI 10.1097/SLA.0b013e3182a6191d

Tae HJ, 2014, ENDOSCOPY, V46, P845, DOI 10.1055/s-0034-1377627

Tahara M, 2005, JPN J CLIN ONCOL, V35, P316, DOI 10.1093/jjco/hyi095

van Hagen P, 2012, NEW ENGL J MED, V366, P2074, DOI 10.1056/NEJMoa1112088

van Rossum PSN, 2015, CLIN RADIOL, V70, P81, DOI 10.1016/j.crad.2014.07.017

van Rossum PSN, 2016, GASTROINTEST ENDOSC, V83, P866, DOI 10.1016/j.gie.2015.11.026

van Rossum PSN, 2015, RADIOTHER ONCOL, V115, P163, DOI 10.1016/j.radonc.2015.04.027

Wu TT, 2007, AM J SURG PATHOL, V31, P58, DOI 10.1097/01.pas.0000213312.36306.cc

NR 22

TC 9

Z9 9

U1 0

U2 0

PU OXFORD UNIV PRESS INC

PI CARY

PA JOURNALS DEPT, 2001 EVANS RD, CARY, NC 27513 USA

SN 1010-7940

EI 1873-734X

J9 EUR J CARDIO-THORAC

JI Eur. J. Cardio-Thorac. Surg.

PD JAN

PY 2018

VL 53

IS 1

BP 201

EP 208

DI 10.1093/ejcts/ezx261

PG 8

WC Cardiac & Cardiovascular Systems; Respiratory System; Surgery

WE Science Citation Index Expanded (SCI-EXPANDED)

SC Cardiovascular System & Cardiology; Respiratory System; Surgery

GA FX5QR

UT WOS:000426135200018

PM 28977486

OA Bronze

DA 2022-05-19

ER

PT J

AU Wang, LH

Li, MM

Sha, BB

Hu, XY

Sun, YX

Zhu, MD

Xu, Y

Li, PP

Wang, YT

Guo, YN

Li, JF

Shi, JX

Li, P

Hu, T

Chen, P

AF Wang, Longhao

Li, Miaomiao

Sha, Beibei

Hu, Xuanyu

Sun, Yaxin

Zhu, Mingda

Xu, Yan

Li, Pingping

Wang, Yating

Guo, Yanyan

Li, Jiangfeng

Shi, Jianxiang

Li, Pei

Hu, Tao

Chen, Ping

TI Inhibition of deubiquitination by PR-619 induces apoptosis and autophagy

via ubi-protein aggregation-activated ER stress in oesophageal squamous

cell carcinoma

SO CELL PROLIFERATION

LA English

DT Article

DE apoptosis; autophagy; ER stress; oesophageal squamous cell carcinoma;

PR&#8208; 619

ID SMALL-MOLECULE INHIBITOR; ENDOPLASMIC-RETICULUM STRESS;

COLORECTAL-CANCER; MULTIPLE-MYELOMA; OVARIAN-CANCER; ENZYME USP14;

CAMKK-BETA; EXPRESSION; AMPK; PROLIFERATION

AB Objectives Targeting the deubiquitinases (DUBs) has become a promising avenue for anti-cancer drug development. However, the effect and mechanism of pan-DUB inhibitor, PR-619, on oesophageal squamous cell carcinoma (ESCC) cells remain to be investigated.

Materials and Methods The effect of PR-619 on ESCC cell growth and cell cycle was evaluated by CCK-8 and PI staining. Annexin V-FITC/PI double staining was performed to detect apoptosis. LC3 immunofluorescence and acridine orange staining were applied to examine autophagy. Intercellular Ca2+ concentration was monitored by Fluo-3AM fluorescence. The accumulation of ubi-proteins and the expression of the endoplasmic reticulum (ER) stress-related protein and CaMKK beta-AMPK signalling were determined by immunoblotting.

Results PR-619 could inhibit ESCC cell growth and induce G2/M cell cycle arrest by downregulating cyclin B1 and upregulating p21. Meanwhile, PR-619 led to the accumulation of ubiquitylated proteins, induced ER stress and triggered apoptosis by the ATF4-Noxa axis. Moreover, the ER stress increased cytoplasmic Ca2+ and then stimulated autophagy through Ca2+-CaMKK beta-AMPK signalling pathway. Ubiquitin E1 inhibitor, PYR-41, could reduce the accumulation of ubi-proteins and alleviate ER stress, G2/M cell cycle arrest, apoptosis and autophagy in PR-619-treated ESCC cells. Furthermore, blocking autophagy by chloroquine or bafilomycin A1 enhanced the cell growth inhibition effect and apoptosis induced by PR-619.

Conclusions Our findings reveal an unrecognized mechanism for the cytotoxic effects of general DUBs inhibitor (PR-619) and imply that targeting DUBs may be a potential anti-ESCC strategy.

C1 [Wang, Longhao; Li, Miaomiao; Sha, Beibei; Hu, Xuanyu; Sun, Yaxin; Zhu, Mingda; Xu, Yan; Li, Pingping; Wang, Yating; Guo, Yanyan; Li, Jiangfeng; Li, Pei; Hu, Tao; Chen, Ping] Zhengzhou Univ, Acad Med Sci, Sch Basic Med Sci, Zhengzhou, Peoples R China.

[Shi, Jianxiang] Zhengzhou Univ, Henan Inst Med & Pharmaceut Sci, Precis Med Ctr, Zhengzhou, Peoples R China.

[Shi, Jianxiang] Zhengzhou Univ, BGI Coll, Zhengzhou, Peoples R China.

[Chen, Ping] Zhengzhou Univ, Henan Key Lab Precis Clin Pharm, Affiliated Hosp 1, Zhengzhou, Peoples R China.

RP Hu, T; Chen, P (corresponding author), Zhengzhou Univ, Sch Basic Med Sci, Zhengzhou 450001, Peoples R China.

EM hnhutao@zzu.edu.cn; zzdx_chenping@zzu.edu.cn

FU Program for Innovation Research Team (in Science and Technology) in

University of Henan Province [20IRTSTHN026]; National Natural Science

Foundation Grant of China [81672421]; Program for Science&Technology

Innovation Talents in Universities of Henan Province [18HASTIT046];

Outstanding Young Talent Research Fund of Zhengzhou University

[51999223, 32210449]; Henan Key Laboratory of Precision Clinical

Pharmacy

FX Program for Innovation Research Team (in Science and Technology) in

University of Henan Province, Grant/Award Number: 20IRTSTHN026; National

Natural Science Foundation Grant of China, Grant/Award Number: 81672421;

Program for Science&Technology Innovation Talents in Universities of

Henan Province, Grant/Award Number: 18HASTIT046; Outstanding Young

Talent Research Fund of Zhengzhou University, Grant/Award Number:

51999223 and 32210449; Open Program of Henan Key Laboratory of Precision

Clinical Pharmacy

CR Albert MC, 2014, MOL CELL ONCOL, V1, DOI 10.4161/mco.29906

Altun M, 2011, CHEM BIOL, V18, P1401, DOI 10.1016/j.chembiol.2011.08.018

An T, 2017, BIOCHEM PHARMACOL, V131, P29, DOI 10.1016/j.bcp.2017.02.011

Boutros R, 2007, NAT REV CANCER, V7, P495, DOI 10.1038/nrc2169

Cai JY, 2017, ONCOTARGET, V8, P63232, DOI 10.18632/oncotarget.18774

Chauhan D, 2012, CANCER CELL, V22, P345, DOI 10.1016/j.ccr.2012.08.007

Chen P, 2016, CLIN CANCER RES, V22, P4145, DOI 10.1158/1078-0432.CCR-15-2254

Chen P, 2015, ONCOTARGET, V6, P9002, DOI 10.18632/oncotarget.3282

Cheng CD, 2013, ONCOL REP, V29, P1730, DOI 10.3892/or.2013.2342

Cheng J, 2019, BBA-REV CANCER, V1872, DOI 10.1016/j.bbcan.2019.188312

Coughlin K, 2014, CLIN CANCER RES, V20, P3174, DOI 10.1158/1078-0432.CCR-13-2658

Crowder RN, 2016, J BIOL CHEM, V291, P5960, DOI 10.1074/jbc.M115.713545

D'Arcy P, 2011, NAT MED, V17, P1636, DOI 10.1038/nm.2536

Deshmukh RR, 2015, BREAST CANCER RES TR, V153, P79, DOI 10.1007/s10549-015-3512-2

Ding X, 2010, JNCI-J NATL CANCER I, V102, P1052, DOI 10.1093/jnci/djq217

Farshi P, 2015, EXPERT OPIN THER PAT, V25, P1191, DOI 10.1517/13543776.2015.1056737

Fischle W, 2005, NATURE, V438, P1116, DOI 10.1038/nature04219

He YT, 2019, CHINESE J CANCER RES, V31, P426, DOI 10.21147/j.issn.1000-9604.2019.03.04

Hou YS, 2015, MOL CELL BIOL, V35, P2740, DOI 10.1128/MCB.00285-15

Hoyer-Hansen M, 2007, MOL CELL, V25, P193, DOI 10.1016/j.molcel.2006.12.009

Hu T, 2019, MOL CARCINOGEN, V58, P42, DOI 10.1002/mc.22905

Iurlaro R, 2016, FEBS J, V283, P2640, DOI 10.1111/febs.13598

Jacomin AC, 2018, CELLS-BASEL, V7, DOI 10.3390/cells7080112

Jayasooriya RGPT, 2018, FOOD CHEM TOXICOL, V121, P648, DOI 10.1016/j.fct.2018.09.057

Jiang SN, 2015, CELL SIGNAL, V27, P1186, DOI 10.1016/j.cellsig.2015.02.024

Kuo KL, 2019, CELLS-BASEL, V8, DOI 10.3390/cells8101268

Lee BSL, 2017, FRONT PHYSIOL, V8, DOI 10.3389/fphys.2017.00489

Lee G, 2016, BIOCHEM BIOPH RES CO, V470, P181, DOI 10.1016/j.bbrc.2016.01.021

Lim KH, 2013, CURR PHARM DESIGN, V19, P4039, DOI 10.2174/1381612811319220013

Lin S, 2018, ONCOL LETT, V15, P2583, DOI 10.3892/ol.2017.7616

Lin ZH, 2015, CELL DISCOV, V1, DOI 10.1038/celldisc.2015.28

Ling SB, 2012, CELL MOL BIOL, V58, P1803, DOI 10.1170/213

Liu B, 2019, PATHOL RES PRACT, V215, DOI 10.1016/j.prp.2019.152592

Ma M, 2016, ONCOTARGETS THER, V9, P1559, DOI 10.2147/OTT.S100050

Min H, 2014, CANCER CHEMOTH PHARM, V74, P167, DOI 10.1007/s00280-014-2451-7

Monda JK, 2018, OPEN BIOL, V8, DOI 10.1098/rsob.180095

Nacarelli T, 2019, NAT CELL BIOL, V21, P397, DOI 10.1038/s41556-019-0287-4

Oh YT, 2017, SCI REP-UK, V7, DOI 10.1038/s41598-017-08424-w

Poondla N, 2019, BMB REP, V52, P181, DOI 10.5483/BMBRep.2019.52.3.048

Sarcar B, 2011, MOL CANCER THER, V10, P2405, DOI 10.1158/1535-7163.MCT-11-0469

Seiberlich V, 2013, CELL BIOCHEM BIOPHYS, V67, P149, DOI 10.1007/s12013-013-9622-8

Seiberlich V, 2012, BBA-MOL CELL RES, V1823, P2057, DOI 10.1016/j.bbamcr.2012.04.011

Setz C, 2017, VIRUSES-BASEL, V9, DOI 10.3390/v9080222

Sha B, 2019, APOPTOSIS, V24, P826, DOI 10.1007/s10495-019-01561-9

Shinji S, 2006, ONCOL REP, V15, P539

Siegel RL, 2019, CA-CANCER J CLIN, V69, P7, DOI 10.3322/caac.21551

Soji K, 2018, PLOS ONE, V13, DOI 10.1371/journal.pone.0202409

Thome MP, 2016, J CELL SCI, V129, P4622, DOI 10.1242/jcs.195057

Tian Z, 2014, BLOOD, V123, P706, DOI 10.1182/blood-2013-05-500033

Wang HB, 2019, BIOCHEM BIOPH RES CO, V517, P477, DOI 10.1016/j.bbrc.2019.07.100

Wang MY, 2017, CELL PHYSIOL BIOCHEM, V43, P1755, DOI 10.1159/000484062

Wang YY, 2015, MED ONCOL, V32, DOI 10.1007/s12032-014-0379-8

Wong VKW, 2013, CELL DEATH DIS, V4, DOI 10.1038/cddis.2013.217

Wu N, 2013, INT J MOL SCI, V14, P10749, DOI 10.3390/ijms140610749

Xi HB, 2013, BIOCHEM PHARMACOL, V85, P1463, DOI 10.1016/j.bcp.2013.02.037

Yi YJ, 2015, REPROD FERT DEVELOP, V27, P1154, DOI 10.1071/RD14012

Young MJ, 2019, J BIOMED SCI, V26, DOI 10.1186/s12929-019-0522-0

Yuan T, 2018, FRONT PHARMACOL, V9, DOI 10.3389/fphar.2018.01080

Zhang BH, 2017, THORAC CANCER, V8, P344, DOI 10.1111/1759-7714.12453

Zhang L, 2016, TOHOKU J EXP MED, V239, P165, DOI 10.1620/tjem.239.165

NR 60

TC 7

Z9 7

U1 6

U2 11

PU WILEY

PI HOBOKEN

PA 111 RIVER ST, HOBOKEN 07030-5774, NJ USA

SN 0960-7722

EI 1365-2184

J9 CELL PROLIFERAT

JI Cell Prolif.

PD JAN

PY 2021

VL 54

IS 1

AR e12919

DI 10.1111/cpr.12919

EA OCT 2020

PG 17

WC Cell Biology

WE Science Citation Index Expanded (SCI-EXPANDED)

SC Cell Biology

GA PS9LS

UT WOS:000582979200001

PM 33129231

OA gold, Green Published

DA 2022-05-19

ER

PT J

AU Jiang, Y

Chen, JL

Shao, YF

AF Jiang, Yun

Chen, Jianle

Shao, Yongfeng

TI Inhibiting CtBP2 expression blocks development of esophageal squamous

cell carcinoma through decreasing angiogenesis

SO INTERNATIONAL JOURNAL OF CLINICAL AND EXPERIMENTAL PATHOLOGY

LA English

DT Article

DE CtBP2; ESCC; biological activity

ID TEL; PROTEIN; CANCER; PROLIFERATION; MIGRATION; INVASION; LUNG

AB The aim of this study is to explain the effects and mechanism of CtBP2 in the development of esophageal squamous cell carcinoma. In this study, we first evaluated CtBP2 protein expression of ESCC tumor and adjacent normal tissues by immunohistochemistry (IHC) and Western blot (WB) assay. Meanwhile, the number of vessels of ESCC and adjacent normal tissues were measured by immunofluorescence. In cell experiments, the effects of CtBP2 were evaluated by wound healing assay, flow cytometry detection, and EPC tube formation. The mechanisms of CtBP2 were investigated by immunofluorescence, qRT-PCR, WB, and EdU incorporation assay. In conclusion, CtBP2 inhibits ESCC in vitro and CtBP2 has a key role in the development of ESCC.

C1 [Jiang, Yun; Shao, Yongfeng] Nanjing Med Univ, Jiangsu Prov Peoples Hosp, Affiliated Hosp 1, Dept Cardiovasc Surg, 300 Guangzhou Rd, Nanjing 210029, Jiangsu, Peoples R China.

[Jiang, Yun] Nantong Univ, Affiliated Hosp, Dept Cardiothorac Surg, Nantong, Jiangsu, Peoples R China.

[Chen, Jianle] First Peoples Hosp Suqian, Dept Cardiothorac Surg, 120 Suzhi Rd, Suqian 223800, Peoples R China.

RP Shao, YF (corresponding author), Nanjing Med Univ, Jiangsu Prov Peoples Hosp, Affiliated Hosp 1, Dept Cardiovasc Surg, 300 Guangzhou Rd, Nanjing 210029, Jiangsu, Peoples R China.

EM shaoyongfen1207@163.com

FU Science and Technology Guiding Project of Nantong Municipal Bureau of

Science and Technology [GJZ16072]

FX This work was supported by 2016 Science and Technology Guiding Project

of Nantong Municipal Bureau of Science and Technology (Project number:

GJZ16072).

CR Bass AJ, 2009, NAT GENET, V41, P1238, DOI 10.1038/ng.465

Birts CN, 2011, BIOL CELL, V103, P1, DOI 10.1042/BC20100067

Edel MJ, 1999, ANTICANCER RES, V19, P2945

Gerhardt H, 2003, J CELL BIOL, V161, P1163, DOI 10.1083/jcb.200302047

Gerhardt H., 2008, ORGANOGENESIS, V4, P241, DOI DOI 10.4161/0RG.4.4.7414

GOLUB TR, 1994, CELL, V77, P307, DOI 10.1016/0092-8674(94)90322-0

Guan CQ, 2013, J CELL BIOCHEM, V114, P1343, DOI 10.1002/jcb.24475

Hellstrom M, 2007, NATURE, V445, P776, DOI 10.1038/nature05571

Kitadai Y, 2004, ONCOL REP, V11, P315

Kleespies A, 2005, ONKOLOGIE, V28, P281, DOI 10.1159/000085198

Lee C, 2014, J BIOL CHEM, V289, P9121, DOI 10.1074/jbc.M114.555888

Li Y, 2017, DIS MARKERS, V2017, DOI 10.1155/2017/5314649

Nan J, 2017, NEUROSCI LETT, V647, P104, DOI 10.1016/j.neulet.2017.02.006

Oshima Y, 2010, ANN THORAC CARDIOVAS, V16, P389

Pennathur A, 2013, LANCET, V381, P400, DOI 10.1016/S0140-6736(12)60643-6

Phng LK, 2009, DEV CELL, V16, P196, DOI 10.1016/j.devcel.2009.01.015

Roukens MG, 2010, NAT CELL BIOL, V12, P933, DOI 10.1038/ncb2096

Stankiewicz Trisha R., 2014, BioMolecular Concepts, V5, P489, DOI 10.1515/bmc-2014-0027

Tammela T, 2008, NATURE, V454, P656, DOI 10.1038/nature07083

Wang LC, 1997, EMBO J, V16, P4374, DOI 10.1093/emboj/16.14.4374

Wang XY, 2015, J BIOL CHEM, V290, P3925, DOI 10.1074/jbc.M114.596866

Zhang CW, 2015, ARCH MED SCI, V11, P1318, DOI 10.5114/aoms.2015.56359

Zhang JG, 2015, TUMOR BIOL, V36, P6701, DOI 10.1007/s13277-015-3354-x

Zhang L, 2016, EXPERT REV GASTROENT, V10, P595, DOI 10.1586/17474124.2016.1140036

NR 24

TC 0

Z9 0

U1 1

U2 2

PU E-CENTURY PUBLISHING CORP

PI MADISON

PA 40 WHITE OAKS LN, MADISON, WI 53711 USA

SN 1936-2625

J9 INT J CLIN EXP PATHO

JI Int. J. Clin. Exp. Pathol.

PY 2018

VL 11

IS 6

BP 2990

EP 2999

PG 10

WC Oncology; Pathology

WE Science Citation Index Expanded (SCI-EXPANDED)

SC Oncology; Pathology

GA GN2TA

UT WOS:000438851900006

PM 31938424

DA 2022-05-19

ER

PT J

AU Zhao, Y

Wang, N

Zhang, XS

Liu, HT

Yang, SJ

AF Zhao, Yan

Wang, Ning

Zhang, Xiaosan

Liu, Hongtao

Yang, Shujun

TI LncRNA ZEB1-AS1 down-regulation suppresses the proliferation and

invasion by inhibiting ZEB1 expression in oesophageal squamous cell

carcinoma

SO JOURNAL OF CELLULAR AND MOLECULAR MEDICINE

LA English

DT Article

DE Epithelial-mesenchymal transition; long non-coding RNA; oesophageal

squamous cell carcinoma; ZEB1; ZEB1-AS1

ID NONCODING RNA ZEB1-AS1; NEOADJUVANT CHEMORADIOTHERAPY; MESENCHYMAL

TRANSITION; CANCER STATISTICS; POOR-PROGNOSIS; LONG; SURVIVAL; PROMOTES;

SURGERY; MARKER

AB Multiple studies have unveiled that long non-coding RNAs (lncRNAs) play a pivotal role in tumour progression and metastasis. However, the biological role of lncRNA ZEB1-AS1 in oesophageal squamous cell carcinoma (ESCC) remains under investigation, and thus, the current study was to investigate the functions of ZEB1-AS1 in proliferation and invasion of ESCC. Here, we discovered that ZEB1-AS1 and ZEB1 were markedly up-regulated in ESCC tissues and cells relative to their corresponding normal control. ZEB1-AS1 and ZEB1 overexpressions were both related to TNM staging and lymph node metastasis as well as poor prognosis in ESCC. The hypomethylation of ZEB1-AS1 promoter triggered ZEB1-AS1 overexpression in ESCC tissues and cells. In addition, ZEB1-AS1 knockdown mediated by siRNA markedly suppressed the proliferation and invasion in vitro in EC9706 and TE1 cells, which was similar with ZEB1 siRNA treatment, coupled with EMT alterations including the up-regulation of E-cadherin level as well as the down-regulation of N-cadherin and vimentin levels. Notably, ZEB1-AS1 depletion dramatically down-regulated ZEB1 expression in EC9706 and TE1 cells, and ZEB1 overexpression obviously reversed the inhibitory effects of proliferation and invasion triggered by ZEB1-AS1 siRNA. ZEB1-AS1 shRNA evidently inhibited tumour growth and weight, whereas ZEB1 elevation partly recovered the tumour growth in ESCC EC9706 and TE1 xenografted nude mice. In conclusion, ZEB1-AS1 overexpression is tightly involved in the development and progression of ESCC, and it exerts the antitumour efficacy by regulating ZEB1 level in ESCC.

C1 [Zhao, Yan; Zhang, Xiaosan; Yang, Shujun] Zhengzhou Univ, Dept Med Oncol, Affiliated Canc Hosp, 127 Dongming Rd, Zhengzhou 450008, Henan, Peoples R China.

[Wang, Ning] Zhengzhou Univ, Henan Acad Med & Pharmaceut Sci, Zhengzhou, Henan, Peoples R China.

[Liu, Hongtao] Zhengzhou Univ, Coll Life Sci, Zhengzhou, Henan, Peoples R China.

RP Yang, SJ (corresponding author), Zhengzhou Univ, Dept Med Oncol, Affiliated Canc Hosp, 127 Dongming Rd, Zhengzhou 450008, Henan, Peoples R China.

EM nkyang001@126.com

OI Liu, Hongtao/0000-0003-3162-0029

FU Natural Science Foundation of Henan Province [182300410377]; Key

Scientific Research Projects of Henan Higher Education Institutions

[17A180016]

FX Natural Science Foundation of Henan Province, Grant/Award Number:

182300410377; Key Scientific Research Projects of Henan Higher Education

Institutions, Grant/Award Number: 17A180016

CR Arnold M, 2015, GUT, V64, P381, DOI 10.1136/gutjnl-2014-308124

Berger AC, 2005, J CLIN ONCOL, V23, P4330, DOI 10.1200/JCO.2005.05.017

Botti G, 2017, CURR DRUG TARGETS, V18, P27, DOI 10.2174/1389450117666151209122950

Chai HN, 2019, TECHNOL CANCER RES T, V18, DOI 10.1177/1533033819849069

Cheng RJ, 2018, ONCOTARGETS THER, V11, P7245, DOI 10.2147/OTT.S179937

Conteduca V, 2012, INT J ONCOL, V41, P414, DOI 10.3892/ijo.2012.1481

Djebali S, 2012, NATURE, V489, P101, DOI 10.1038/nature11233

Dong JQ, 2017, CLIN CANCER RES, V23, P3461, DOI 10.1158/1078-0432.CCR-16-2180

Fu LL, 2017, CRIT REV EUKAR GENE, V27, P183, DOI 10.1615/CritRevEukaryotGeneExpr.2017019244

Gao RX, 2019, J EXP CLIN CANC RES, V38, DOI 10.1186/s13046-019-1102-6

Hanahan D, 2011, CELL, V144, P646, DOI 10.1016/j.cell.2011.02.013

Huang XM, 2018, CANCER LETT, V413, P94, DOI 10.1016/j.canlet.2017.10.046

Jemal A, 2010, CA-CANCER J CLIN, V60, P277, DOI 10.3322/caac.20073

Ji B, 2019, CANCER BIOMARK, V24, P249, DOI 10.3233/CBM-181486

Jin JJ, 2019, CLIN SCI, V133, P381, DOI 10.1042/CS20180983

Law S, 2003, ANN SURG, V238, P339, DOI 10.1097/01.sla.0000086545.45918.ee

Li MF, 2019, EXP THER MED, V17, P3539, DOI 10.3892/etm.2019.7358

Li T, 2016, ONCOGENE, V35, P1575, DOI 10.1038/onc.2015.223

Liu BY, 2017, NAT IMMUNOL, V18, P499, DOI 10.1038/ni.3712

Liu HT, 2010, J SURG ONCOL, V102, P683, DOI 10.1002/jso.21627

Liu HT, 2020, PATHOL ONCOL RES, V26, P1029, DOI 10.1007/s12253-019-00655-8

Lv SY, 2018, CELL CYCLE, V17, P1245, DOI 10.1080/15384101.2018.1471317

Ma PJ, 2017, CLIN CHIM ACTA, V475, P172, DOI 10.1016/j.cca.2017.10.020

Meng L, 2018, AM J TRANSL RES, V10, P3395

Mercer TR, 2009, NAT REV GENET, V10, P155, DOI 10.1038/nrg2521

Oppedijk V, 2014, J CLIN ONCOL, V32, P385, DOI 10.1200/JCO.2013.51.2186

Pennathur A, 2013, LANCET, V381, P400, DOI 10.1016/S0140-6736(12)60643-6

Ponting CP, 2009, CELL, V136, P629, DOI 10.1016/j.cell.2009.02.006

Shapiro J, 2015, LANCET ONCOL, V16, P1090, DOI 10.1016/S1470-2045(15)00040-6

Sjoquist KM, 2011, LANCET ONCOL, V12, P681, DOI 10.1016/S1470-2045(11)70142-5

Torre LA, 2015, CA-CANCER J CLIN, V65, P87, DOI 10.3322/caac.21262

Wang GQ, 2004, ANN THORAC SURG, V77, P1740, DOI 10.1016/j.athoracsur.2003.10.098

Wang Q, 2019, EXP THER MED, V17, P857, DOI 10.3892/etm.2018.7005

Wang Y, 2019, BIOMED PHARMACOTHER, V111, P1297, DOI 10.1016/j.biopha.2018.12.134

Wang YQ, 2019, CANCER RES, V79, P3542, DOI 10.1158/0008-5472.CAN-18-2900

Wu Y, 2018, J CANCER, V9, P3690, DOI 10.7150/jca.27263

Xue WH, 2018, WORLD J GASTROENTERO, V24, P23, DOI 10.3748/wjg.v24.i1.23

Ye BQ, 2018, EMBO J, V37, DOI 10.15252/embj.201797174

Ye GM, 2019, EXP THER MED, V17, P3035, DOI 10.3892/etm.2019.7277

Zhang JW, 2018, HEPATOLOGY, V67, P171, DOI 10.1002/hep.29405

Zhou X, 2019, MEDICINE, V98, DOI 10.1097/MD.0000000000015251

NR 41

TC 16

Z9 16

U1 1

U2 1

PU WILEY

PI HOBOKEN

PA 111 RIVER ST, HOBOKEN 07030-5774, NJ USA

SN 1582-1838

EI 1582-4934

J9 J CELL MOL MED

JI J. Cell. Mol. Med.

PD DEC

PY 2019

VL 23

IS 12

BP 8206

EP 8218

DI 10.1111/jcmm.14692

EA OCT 2019

PG 13

WC Cell Biology; Medicine, Research & Experimental

WE Science Citation Index Expanded (SCI-EXPANDED)

SC Cell Biology; Research & Experimental Medicine

GA JL9PD

UT WOS:000494087000001

PM 31638344

OA Green Published, gold

DA 2022-05-19

ER

PT J

AU Li, CQ

Zhao, SG

Zheng, YY

Han, YC

Chen, XY

Cheng, ZH

Wu, YQ

Feng, XJ

Qi, WX

Chen, K

Xiang, J

Li, J

Lerut, T

Li, HC

AF Li, Chengqiang

Zhao, Shengguang

Zheng, Yuyan

Han, Yichao

Chen, Xiaoyan

Cheng, Zenghui

Wu, Yuquan

Feng, Xijia

Qi, Weixiang

Chen, Kai

Xiang, Jie

Li, Jian

Lerut, Toni

Li, Hecheng

TI Preoperative pembrolizumab combined with chemoradiotherapy for

oesophageal squamous cell carcinoma (PALACE-1)

SO EUROPEAN JOURNAL OF CANCER

LA English

DT Article

DE PD-1 inhibitor; Chemoradiotherapy; Oesophageal squamous cell carcinoma;

Pathologic complete response

ID NEOADJUVANT CHEMORADIOTHERAPY; ESOPHAGOGASTRIC JUNCTION; CANCER;

IMMUNOTHERAPY; LANDSCAPE; SURGERY

AB Background: To investigate the safety and activity of preoperative pembrolizumab combined with chemoradiotherapy for resectable oesophageal squamous cell carcinoma (ESCC) (ClinicalTrials.gov number, NCT03792347).

Methods: Twenty resectable ESCC patients, regardless of programmed death ligand-1 status, received preoperative pembrolizumab with concurrent chemoradiotherapy (PPCT). Preoperative therapy includes carboplatin (area under the curve of 2 mg per milliliter per minute, once a week for 5 weeks), paclitaxel (50 mg/m(2), once a week for 5 weeks), radiotherapy (23 fractions of 1.8 Gy, 5 fraction a week) and pembrolizumab (2 mg/kg) on days 1 and 22. Within 4-6 weeks after preoperative therapy, patients underwent surgery. The primary end-point was safety and secondary outcome measures were feasibility, pathologic complete response (pCR) rate and radiographic response. Immune signature of CD8+ T cells was evaluated in surgical specimens using immunohistochemistry and immunofluorescence.

Results: All patients have received PPCT successfully, except one patient who missed the last dose of chemotherapy due to leukopenia. Grade III and higher adverse events (AEs) were observed in 13 patients (13/20, 65%), and one patient had a grade V AE. The most frequent grade III AE was lymphopenia (12/13, 92%). Eighteen patients underwent surgery within 4-9 weeks after PPCT and the pCR rate was 55.6% (10/18). The percentage of transcription factor 1 positive cells was significantly higher in specimens of pCR group than those of non-pCR group (p value = 0.010).

Conclusions: PPCT was safe, did not delay surgery, and induced a pCR in 55.6% of resected tumours. A phase II multicentre study is undergoing for further confirmation of efficacy (NCT04435197). (C) 2020 Elsevier Ltd. All rights reserved.

C1 [Li, Chengqiang; Zheng, Yuyan; Han, Yichao; Wu, Yuquan; Feng, Xijia; Chen, Kai; Xiang, Jie; Li, Hecheng] Shanghai Jiao Tong Univ, Sch Med, Ruijin Hosp, Dept Thorac Surg, 197 Ruijin 2nd Rd, Shanghai 200025, Peoples R China.

[Zhao, Shengguang; Qi, Weixiang] Shanghai Jiao Tong Univ, Sch Med, Ruijin Hosp, Dept Radiotherapy, Shanghai 200025, Peoples R China.

[Chen, Xiaoyan] Shanghai Jiao Tong Univ, Sch Med, Ruijin Hosp, Dept Pathol, Shanghai 200025, Peoples R China.

[Cheng, Zenghui] Shanghai Jiao Tong Univ, Sch Med, Ruijin Hosp, Dept Radiol, Shanghai 200025, Peoples R China.

[Li, Jian] Shanghai Jiao Tong Univ, Sch Med, Ruijin Hosp, Clin Res Ctr, Shanghai 200025, Peoples R China.

[Lerut, Toni] Univ Hosp Leuven, Dept Thorac Surg, Herestr 49, B-3000 Leuven, Belgium.

RP Li, HC (corresponding author), Shanghai Jiao Tong Univ, Sch Med, Ruijin Hosp, Dept Thorac Surg, 197 Ruijin 2nd Rd, Shanghai 200025, Peoples R China.

EM lihecheng2000@hotmail.com

FU National Natural Science Foundation of China [81871882]; Shanghai

Municipal Commission of Health and Family Planning Outstanding Academic

Leaders Training Program [2017BR055]; Science and Technology Commission

of Shanghai Municipality Excellent Aacademic/Technical Leader Program

[20XD1402300]; Shanghai Municipal Education CommissiondGaofeng Clinical

Medicine Grant [20172005]

FX This work was supported by the National Natural Science Foundation of

China (grant number 81871882), Shanghai Municipal Commission of Health

and Family Planning Outstanding Academic Leaders Training Program (grant

number 2017BR055), Science and Technology Commission of Shanghai

Municipality Excellent Aacademic/Technical Leader Program (grant number

20XD1402300) and Shanghai Municipal Education CommissiondGaofeng

Clinical Medicine Grant (grant number 20172005).

CR Abnet CC, 2018, GASTROENTEROLOGY, V154, P360, DOI 10.1053/j.gastro.2017.08.023

Ajani JA, 2019, J NATL COMPR CANC NE, V17, P855, DOI 10.6004/jnccn.2019.0033

Berger AC, 2005, J CLIN ONCOL, V23, P4330, DOI 10.1200/JCO.2005.05.017

Blank CU, 2019, NAT REV IMMUNOL, V19, P665, DOI 10.1038/s41577-019-0221-9

Bray F, 2018, CA-CANCER J CLIN, V68, P394, DOI 10.3322/caac.21492

Doi T, 2018, J CLIN ONCOL, V36, P61, DOI 10.1200/JCO.2017.74.9846

Elliott JA, 2014, BRIT J SURG, V101, P1702, DOI 10.1002/bjs.9670

Gao YB, 2014, NAT GENET, V46, P1097, DOI 10.1038/ng.3076

Gong J, 2018, J IMMUNOTHER CANCER, V6, DOI 10.1186/s40425-018-0361-7

Held W, 2019, SCI TRANSL MED, V11, DOI 10.1126/scitranslmed.aay6863

Jansen CS, 2019, NATURE, V576, P465, DOI 10.1038/s41586-019-1836-5

Kato K, 2019, LANCET ONCOL, V20, P1506, DOI 10.1016/S1470-2045(19)30626-6

Kato K, 2019, FUTURE ONCOL, V15, P1057, DOI 10.2217/fon-2018-0609

Kim J, 2017, NATURE, V541, P169, DOI 10.1038/nature20805

Kojima T, 2019, J CLIN ONCOL, V37, DOI 10.1200/JCO.2019.37.4_suppl.2

Leng XF, 2021, ANN SURG, V274, pE1022, DOI 10.1097/SLA.0000000000003727

Lin DC, 2014, NAT GENET, V46, P467, DOI 10.1038/ng.2935

Mariette C, 2014, J CLIN ONCOL, V32, P2416, DOI 10.1200/JCO.2013.53.6532

McLane LM, 2019, ANNU REV IMMUNOL, V37, P457, DOI 10.1146/annurev-immunol-041015-055318

Nishimaki T, 1999, ANN THORAC SURG, V68, P2059, DOI 10.1016/S0003-4975(99)01171-6

Ohigashi Y, 2005, CLIN CANCER RES, V11, P2947, DOI 10.1158/1078-0432.CCR-04-1469

Rice TW, 2017, J THORAC ONCOL, V12, P36, DOI 10.1016/j.jtho.2016.10.016

Sade-Feldman M, 2018, CELL, V175, P998, DOI 10.1016/j.cell.2018.10.038

Sawada G, 2016, GASTROENTEROLOGY, V150, P1171, DOI 10.1053/j.gastro.2016.01.035

Shah MA, 2019, JAMA ONCOL, V5, P546, DOI 10.1001/jamaoncol.2018.5441

Shapiro J, 2015, LANCET ONCOL, V16, P1090, DOI 10.1016/S1470-2045(15)00040-6

Sharabi AB, 2015, LANCET ONCOL, V16, pE498, DOI 10.1016/S1470-2045(15)00007-8

Song YM, 2014, NATURE, V509, P91, DOI 10.1038/nature13176

Twyman-Saint Victor C, 2015, NATURE, V520, P373, DOI 10.1038/nature14292

van Hagen P, 2012, NEW ENGL J MED, V366, P2074, DOI 10.1056/NEJMoa1112088

Vodnala SK, 2019, NATURE, V576, P385, DOI 10.1038/d41586-019-03670-6

Wan T, 2019, ANN SURG ONCOL, V26, P1412, DOI 10.1245/s10434-018-07147-0

Yagi T, 2019, ANN SURG, V269, P471, DOI 10.1097/SLA.0000000000002616

Yang H, 2018, J CLIN ONCOL, V36, P2796, DOI 10.1200/JCO.2018.79.1483

Yarchoan M, 2017, NAT REV CANCER, V17, DOI 10.1038/nrc.2017.74

Zhang L, 2015, AM J HUM GENET, V96, P597, DOI 10.1016/j.ajhg.2015.02.017

NR 36

TC 16

Z9 22

U1 5

U2 20

PU ELSEVIER SCI LTD

PI OXFORD

PA THE BOULEVARD, LANGFORD LANE, KIDLINGTON, OXFORD OX5 1GB, OXON, ENGLAND

SN 0959-8049

EI 1879-0852

J9 EUR J CANCER

JI Eur. J. Cancer

PD FEB

PY 2021

VL 144

BP 232

EP 241

DI 10.1016/j.ejca.2020.11.039

PG 10

WC Oncology

WE Science Citation Index Expanded (SCI-EXPANDED)

SC Oncology

GA PV8EW

UT WOS:000610215700023

PM 33373868

DA 2022-05-19

ER

PT J

AU Liu, JG

Wei, ZR

Wang, YB

Xia, ZJ

Zhao, GF

AF Liu, Jingeng

Wei, Zhiru

Wang, Yuebin

Xia, Zongjiang

Zhao, Gaofeng

TI Hepatic resection for post-operative solitary liver metastasis from

oesophageal squamous cell carcinoma

SO ANZ JOURNAL OF SURGERY

LA English

DT Article

DE hepatic resection; oesophageal squamous cell carcinoma; prognosis;

solitary hepatic metastasis

ID COLORECTAL METASTASES; SURGICAL RESECTION; SINGLE-CENTER; CANCER;

SURVIVAL; SURGERY; PATTERN; RECURRENCE; CHEMOTHERAPY; PROGNOSIS

AB BackgroundLiver metastasis is common in patients with oesophageal cancer. The effect of operative intervention for post-operative solitary liver metastasis from oesophageal squamous cell carcinoma (ESCC) has not previously been examined. This research was to compare the effect of surgery and non-surgical therapy in patients with post-operative solitary liver metastasis from ESCC.

MethodsWe retrospectively analysed the clinical data of 69 consecutive patients with solitary hepatic metastasis who had undergone oesophagectomy for ESCC and were subsequently referred to the First Affiliated Hospital of Zhengzhou University from January 2005 to December 2013. The survival rates of the surgical and non-surgical groups were compared.

ResultsThere were 26 patients in the surgical group and 43 patients in the non-surgical group. There was no operative death in the surgical group. Post-operative complications were observed in six patients, and all of these patients recovered after additional treatments. Patients in the surgical group had 1- and 2-year cumulative survival rates of 50.8 and 21.2%, respectively, which were significantly higher than the 31.0 and 7.1% survival rates of patients in the non-surgical group (P<0.05). In each group, the patients with a disease-free interval (DFI) lasting >12months had a better survival rate than those with a DFI lasting 12months (all P<0.05).

ConclusionsOperative intervention is a better treatment choice for patients with post-operative solitary liver metastasis from ESCC, especially for patients with a DFI lasting >12months. Patients selected for hepatic resection should be considered on an individual basis through a multidisciplinary team of specialists.

C1 [Liu, Jingeng; Wang, Yuebin; Xia, Zongjiang; Zhao, Gaofeng] Zhengzhou Univ, Dept Thorac Surg, Affiliated Hosp 1, 1 Jianshe Rd East, Zhengzhou 450052, Henan, Peoples R China.

[Wei, Zhiru] Zhengzhou Univ, Dept Plast Surg, Affiliated Hosp 1, Zhengzhou, Henan, Peoples R China.

RP Liu, JG (corresponding author), Zhengzhou Univ, Dept Thorac Surg, Affiliated Hosp 1, 1 Jianshe Rd East, Zhengzhou 450052, Henan, Peoples R China.

EM liujingeng888@163.com

CR Chen JQ, 2010, ANN THORAC SURG, V90, P435, DOI 10.1016/j.athoracsur.2010.04.002

Elias D, 2003, SURGERY, V133, P375, DOI 10.1067/msy.2003.114

Hanazaki K, 1998, HEPATO-GASTROENTEROL, V45, P201

Hsu PK, 2011, J GASTROINTEST SURG, V15, P558, DOI 10.1007/s11605-011-1458-1

Huddy JR, 2015, DIS ESOPHAGUS, V28, P483, DOI 10.1111/dote.12233

Ichida H, 2013, WORLD J SURG, V37, P398, DOI 10.1007/s00268-012-1830-7

Jiang YQ, 2010, ANN THORAC SURG, V90, P908, DOI 10.1016/j.athoracsur.2010.05.060

Khatri VP, 2005, J CLIN ONCOL, V23, P8490, DOI 10.1200/JCO.2004.00.6155

Li HH, 2009, J THORAC CARDIOV SUR, V137, P55, DOI 10.1016/j.jtcvs.2008.05.024

Mariette C, 2003, CANCER-AM CANCER SOC, V97, P1616, DOI 10.1002/cncr.11228

Mise Y, 2010, ANN SURG, V251, P902, DOI 10.1097/SLA.0b013e3181c9868a

Morita M, 2008, SURGERY, V143, P499, DOI 10.1016/j.surg.2007.12.007

Mudan SS, 2010, HIPPOKRATIA, V14, P291

Nagano H, 2001, Gan To Kagaku Ryoho, V28, P1628

Nakagawa S, 2004, J AM COLL SURGEONS, V198, P205, DOI 10.1016/j.jamcollsurg.2003.10.005

Nordlinger B, 2008, LANCET, V371, P1007, DOI 10.1016/S0140-6736(08)60455-9

Pawlik TM, 2007, J GASTROINTEST SURG, V11, P1057, DOI 10.1007/s11605-006-0061-3

Ponsner MC, 2005, CANC PRINCIPLES PRAC, P861

Sarmiento JM, 2003, J AM COLL SURGEONS, V197, P29, DOI 10.1016/S1072-7515(03)00230-8

Sato N, 1998, Gan To Kagaku Ryoho, V25, P314

Siewert JR, 2001, ANN SURG, V234, P360, DOI 10.1097/00000658-200109000-00010

Spolverato G, 2013, WORLD J GASTRO ONCOL, V5, P207, DOI 10.4251/wjgo.v5.i12.207

Stein HJ, 2005, ANN SURG, V242, P566, DOI 10.1097/01.sla.0000184211.75970.85

Tokairin Yutaka, 2009, Gan To Kagaku Ryoho, V36, P2462

Yong ELC, 2009, ANZ J SURG, V79, P724, DOI 10.1111/j.1445-2197.2009.05058.x

Younes M, 2002, SCAND J GASTROENTERO, V37, P1359, DOI 10.1080/003655202762671215

Zhang Dong-kun, 2008, Zhonghua Wai Ke Za Zhi, V46, P1333

NR 27

TC 7

Z9 7

U1 0

U2 1

PU WILEY

PI HOBOKEN

PA 111 RIVER ST, HOBOKEN 07030-5774, NJ USA

SN 1445-1433

EI 1445-2197

J9 ANZ J SURG

JI ANZ J. Surg.

PD APR

PY 2018

VL 88

IS 4

BP E252

EP E256

DI 10.1111/ans.13810

PG 5

WC Surgery

WE Science Citation Index Expanded (SCI-EXPANDED)

SC Surgery

GA GB3XS

UT WOS:000428994400006

PM 27764891

DA 2022-05-19

ER

PT J

AU Li, ZC

Huang, Y

Dong, F

Li, W

Ding, L

Yu, GS

Xu, D

Yang, YY

Xu, XG

Tong, DW

AF Li, Zhaocai

Huang, Yong

Dong, Feng

Li, Wei

Ding, Li

Yu, Gaoshui

Xu, Dan

Yang, Yuanyuan

Xu, Xingang

Tong, Dewen

TI Swainsonine promotes apoptosis in human oesophageal squamous cell

carcinoma cells in vitro and in vivo through activation of mitochondrial

pathway

SO JOURNAL OF BIOSCIENCES

LA English

DT Article

DE Apoptosis; caspase; Eca-109 cells; mitochondrial pathway; swainsonine

ID CANCER; GROWTH; INHIBITION; PROTEIN; STAT4; DATP

AB Swainsonine, a natural indolizidine alkaloid, has been reported to have antitumour effects, and can induce apoptosis in human gastric and lung cancer cells. In the present study, we evaluated the antitumour effects of swainsonine on several oesophageal squamous cell carcinoma cells and investigated relative molecular mechanisms. Swainsonine treatment inhibited the growth of Eca-109, TE-1 and TE-10 cells in a concentration-dependent manner as measured by MTT assay. Morphological observation, DNA laddering detection and flow cytometry analysis demonstrated that swainsonine treatment induced Eca-109 cell apoptosis in vitro. Further results showed that swainsonine treatment up-regulated Bax, down-regulated Bcl-2 expression, triggered Bax translocation to mitochondria, destructed mitochondria integrity and activated mitochondria-mediated apoptotic pathway, followed by the release of cytochrome c, which in turn activated caspase-9 and caspase-3, promoted the cleavage of PARP, resulting in Eca-109 cell apoptosis. Moreover, swainsonine treatment inhibited Bcl-2 expression, promoted Bax translocation, cytochrome c release and caspase-3 activation in xenograft tumour cells, resulting in a significant decrease of tumour volume and tumour weight in the swainsonine-treated xenograft mice groups compared with that in the control group. Taken together, this study demonstrated that swainsonine inhibited Eca-109 cells growth through activation of mitochondria-mediated caspase-dependent pathway. [Li Z, Huang Y Dong F, Li W, Ding L, Yu G, Xu D, Yang Y, Xu X and Tong D 2012 Swainsonine promotes apoptosis in human oesophageal squamous cell carcinoma cells in vitro and in vivo through activation of mitochondria! pathway. J. Biosci. 37 1005-1016] DOI 10.1007/s12038-012-9265-8

C1 [Li, Zhaocai; Huang, Yong; Dong, Feng; Li, Wei; Ding, Li; Yu, Gaoshui; Xu, Dan; Yang, Yuanyuan; Xu, Xingang; Tong, Dewen] NW A&F Univ, Coll Vet Med, Yangling 712100, Shaanxi, Peoples R China.

RP Tong, DW (corresponding author), NW A&F Univ, Coll Vet Med, Yangling 712100, Shaanxi, Peoples R China.

EM dwtong@nwsuaf.edu.cn

RI Li, Zhaocai/K-2599-2013; Huang, Yong/G-9365-2011

FU National Natural Science Foundation of China [31072108]; Doctoral

Program of Higher Education of China [20090204110016, 20110204110014];

Fundamental Research Funds for the Central Universities [QN2011064]

FX This work was supported by the National Natural Science Foundation of

China (Grant No. 31072108), the Doctoral Program of Higher Education of

China (Grant No. 20090204110016, 20110204110014) and the Fundamental

Research Funds for the Central Universities (QN2011064). We are grateful

to Yong Zhao and Kai Lei for their kind help in the animal experiments.

The authors also thank Xin He for flow cytometry assay, and Li Cao for

help in statistical analysis.

CR DENNIS JW, 1989, J NATL CANCER I, V81, P1028, DOI 10.1093/jnci/81.13.1028

DENNIS JW, 1989, CANCER CELL-MON REV, V1, P87

DENNIS JW, 1990, CANCER RES, V50, P1867

Ekman S, 2008, EXPERT REV ANTICANC, V8, P1433, DOI 10.1586/14737140.8.9.1433

Enzinger PC, 2003, NEW ENGL J MED, V349, P2241, DOI 10.1056/NEJMra035010

Ghobrial IM, 2005, CA-CANCER J CLIN, V55, P178, DOI 10.3322/canjclin.55.3.178

GOSS PE, 1994, CANCER RES, V54, P1450

Goss PE, 1997, CLIN CANCER RES, V3, P1077

Huang Y, 2011, BLOOD, V118, P6793, DOI 10.1182/blood-2011-05-356162

Huang Y, 2011, J LEUKOCYTE BIOL, V90, P87, DOI 10.1189/jlb.1210674

Huang Y, 2010, MOL IMMUNOL, V47, P2604, DOI 10.1016/j.molimm.2010.05.290

Huang YT, 2006, CANCER LETT, V231, P270, DOI 10.1016/j.canlet.2005.02.011

Janicke RU, 1998, J BIOL CHEM, V273, P9357, DOI 10.1074/jbc.273.16.9357

Li P, 1997, CELL, V91, P479, DOI 10.1016/S0092-8674(00)80434-1

Li ZC, 2012, INT J BIOL SCI, V8, P394, DOI 10.7150/ijbs.3882

Liu XS, 1996, CELL, V86, P147, DOI 10.1016/S0092-8674(00)80085-9

Livak KJ, 2001, METHODS, V25, P402, DOI 10.1006/meth.2001.1262

MOLYNEUX RJ, 1982, SCIENCE, V216, P190, DOI 10.1126/science.6801763

NOVIKOFF PM, 1985, J CELL BIOL, V101, P339, DOI 10.1083/jcb.101.2.339

Sharpe JC, 2004, BBA-MOL CELL RES, V1644, P107, DOI 10.1016/j.bbamcr.2003.10.016

Sun JY, 2007, PHYTOMEDICINE, V14, P353, DOI 10.1016/j.phymed.2006.08.003

Sun JY, 2009, PHYTOMEDICINE, V16, P1070, DOI 10.1016/j.phymed.2009.02.012

Surh YJ, 2003, NAT REV CANCER, V3, P768, DOI 10.1038/nrc1189

Susin SA, 1999, NATURE, V397, P441, DOI 10.1038/17135

Tong DW, 2008, ANIM FEED SCI TECH, V142, P74, DOI 10.1016/j.anifeedsci.2007.07.007

Verhagen AM, 2000, CELL, V102, P43, DOI 10.1016/S0092-8674(00)00009-X

Xu YF, 2009, CANCER LETT, V284, P229, DOI 10.1016/j.canlet.2009.04.028

YAGITA M, 1990, SCAND J IMMUNOL, V31, P275, DOI 10.1111/j.1365-3083.1990.tb02769.x

NR 28

TC 18

Z9 25

U1 2

U2 35

PU INDIAN ACAD SCIENCES

PI BANGALORE

PA C V RAMAN AVENUE, SADASHIVANAGAR, P B #8005, BANGALORE 560 080, INDIA

SN 0250-5991

EI 0973-7138

J9 J BIOSCIENCES

JI J. Biosci.

PD DEC

PY 2012

VL 37

IS 6

BP 1005

EP 1016

DI 10.1007/s12038-012-9265-8

PG 12

WC Biology

WE Science Citation Index Expanded (SCI-EXPANDED)

SC Life Sciences & Biomedicine - Other Topics

GA 050IC

UT WOS:000312046000012

PM 23151791

DA 2022-05-19

ER

PT J

AU You, ZB

Xu, DF

Ji, J

Guo, W

Zhu, WG

He, JD

AF You, Zhenbing

Xu, Dafu

Ji, Jian

Guo, Wei

Zhu, Weiguo

He, Jingdong

TI JAK/STAT signal pathway activation promotes progression and survival of

human oesophageal squamous cell carcinoma

SO CLINICAL & TRANSLATIONAL ONCOLOGY

LA English

DT Article

DE Oesophageal squamous cell carcinoma; JAK/STAT pathway; Prognosis

ID CONSTITUTIVE ACTIVATION; STAT3 ACTIVATION; EXPRESSION; PROSTATE;

PROGNOSIS; PROTEINS; GROWTH; BREAST

AB Objective Inappropriate activation of JAK/STAT pathway occurs with high frequency in human cancers and is associated with cancer cell survival and proliferation. However, its role in oesophageal squamous cell carcinoma (ESCC) is unknown.

Methods By immunohistochemistry, we analysed the expression of two components of this pathway, phosphorylated JAK-1 (pJAK-1) and phosphorylated STAT-3 (pSTAT-3), in 100 ESCC tumours and paired non-neoplastic oesophageal epithelia. Kaplan-Meier survival and Cox regression analyses were performed to evaluate the prognosis of patients.

Results We found that pJAK-1 and pSTAT-3 expression was not detectable in normal oesophageal squamous cells. Primary ESCC with pJAK-1-positive and pSTAT-3-positive expression was detected in the cancer cell nests of 78 and 72 cases, respectively. In addition, the Pearson's correlation coefficient between pJAK-1 and pSTAT-3 expression was 0.806 (p<0.001). Moreover, pJAK-1 and pSTAT-3 expression was correlated with N stage (lymph node metastasis, both p=0.01), pTNM stage (p=0.008 and 0.009, respectively) and metastatic status (both p=0.01). Furthermore, pJAK-1 and pSTAT-3 expression was associated with shorter overall survival (both p<0.001) and shorter disease-free survival (p=0.005 and 0.006, respectively). By multivariate analysis, TNM clinical classification (T, p<0.001; N, p=0.002; M, p=0.02), pJAK-1 (p=0.002) and pSTAT-3 (p=0.003) were independent prognosis predictors of ESCC.

Conclusion These results provide convincing evidence for the first time that the JAK/STAT pathway may participate in the progress of ESCC.

C1 [You, Zhenbing; Xu, Dafu; Ji, Jian; Guo, Wei] Nanjing Med Univ, Dept Chest Surg, Hosp Huaian 1, Huaian 223300, Peoples R China.

[Zhu, Weiguo; He, Jingdong] Nanjing Med Univ, Dept Oncol, Hosp Huaian 1, Huaian 223300, Peoples R China.

RP Guo, W (corresponding author), Nanjing Med Univ, Dept Chest Surg, Hosp Huaian 1, Huaian 223300, Peoples R China.

EM jshayzb@gmail.com

CR Berclaz G, 2001, INT J ONCOL, V19, P1155

Bolli R, 2003, TRENDS CARDIOVAS MED, V13, P72, DOI 10.1016/S1050-1738(02)00230-X

Booz GW, 2002, J MOL CELL CARDIOL, V34, P1443, DOI 10.1006/jmcc.2002.2076

Bromberg JF, 2001, BIOESSAYS, V23, P161, DOI 10.1002/1521-1878(200102)23:2<161::AID-BIES1023>3.0.CO;2-0

Calo V, 2003, J CELL PHYSIOL, V197, P157, DOI 10.1002/jcp.10364

Campbell CL, 2001, AM J PATHOL, V158, P25, DOI 10.1016/S0002-9440(10)63940-5

Darnell JE, 1997, SCIENCE, V277, P1630, DOI 10.1126/science.277.5332.1630

Delektorskaya VV, 2009, B EXP BIOL MED+, V148, P241, DOI 10.1007/s10517-009-0659-z

Gao B, 2001, FEBS LETT, V488, P179, DOI 10.1016/S0014-5793(00)02430-3

Gross ER, 2006, AM J PHYSIOL-HEART C, V291, pH827, DOI 10.1152/ajpheart.00003.2006

Hamilton SR, 2000, WHO CLASSIFICATION T

Haura EB, 2005, NAT CLIN PRACT ONCOL, V2, P315, DOI 10.1038/ncponc0195

Huang M, 2000, GYNECOL ONCOL, V79, P67, DOI 10.1006/gyno.2000.5931

Jiang H, 2010, DIS ESOPHAGUS, V23, P487, DOI 10.1111/j.1442-2050.2009.01043.x

Kandalam U, 2010, REGUL PEPTIDES, V159, P110, DOI 10.1016/j.regpep.2009.09.001

Kato H, 2007, DIGEST SURG, V24, P88, DOI 10.1159/000101894

Levy DE, 2002, NAT REV MOL CELL BIO, V3, P651, DOI 10.1038/nrm909

Niu GL, 2002, ONCOGENE, V21, P2000, DOI 10.1038/sj.onc.1205260

Page C, 2000, INT J ONCOL, V17, P23

Sano A, 2009, ANTICANCER RES, V29, P3375

Song JI, 2000, ONCOGENE, V19, P2489, DOI 10.1038/sj.onc.1203483

Tam L, 2007, BRIT J CANCER, V97, P378, DOI 10.1038/sj.bjc.6603871

Tu YY, 2011, MED ONCOL, V28, P15, DOI 10.1007/s12032-010-9435-1

Turkson J, 2004, EXPERT OPIN THER TAR, V8, P409, DOI 10.1517/14728222.8.5.409

Turkson J, 1998, MOL CELL BIOL, V18, P2545, DOI 10.1128/MCB.18.5.2545

Valdembri D, 2001, FASEB J, V15, P225, DOI 10.1096/fj.01-0633fje

Wang TH, 2004, NAT MED, V10, P48, DOI 10.1038/nm976

Wang WS, 2010, DIS ESOPHAGUS, V23, P512, DOI 10.1111/j.1442-2050.2009.01035.x

Wei LH, 2003, ONCOGENE, V22, P1517, DOI 10.1038/sj.onc.1206226

Williams JG, 2000, CURR OPIN GENET DEV, V10, P503, DOI 10.1016/S0959-437X(00)00119-2

NR 30

TC 36

Z9 41

U1 0

U2 17

PU SPRINGER INTERNATIONAL PUBLISHING AG

PI CHAM

PA GEWERBESTRASSE 11, CHAM, CH-6330, SWITZERLAND

SN 1699-048X

EI 1699-3055

J9 CLIN TRANSL ONCOL

JI Clin. Transl. Oncol.

PD FEB

PY 2012

VL 14

IS 2

BP 143

EP 149

DI 10.1007/s12094-012-0774-6

PG 7

WC Oncology

WE Science Citation Index Expanded (SCI-EXPANDED)

SC Oncology

GA 903NB

UT WOS:000301122200010

PM 22301404

DA 2022-05-19

ER

PT J

AU Mai, SJ

Liu, L

Jiang, JJ

Ren, PF

Diao, DW

Wang, HF

Cai, KC

AF Mai, Shijie

Liu, Le

Jiang, Jianjun

Ren, Pengfei

Diao, Dingwei

Wang, Haofei

Cai, Kaican

TI Oesophageal squamous cell carcinoma-associated IL-33 rewires macrophage

polarization towards M2 via activating ornithine decarboxylase

SO CELL PROLIFERATION

LA English

DT Article

DE IL&#8208; 33; macrophage; oesophageal squamous cell carcinoma; ornithine

decarboxylase

ID TUMOR-ASSOCIATED MACROPHAGES; ALTERNATIVE ACTIVATION; CANCER;

PROGRESSION; DIVERSITY; AXIS

AB Background The tumour microenvironment primarily constitutes macrophages in the form of an immunosuppressive M2 phenotype, which promotes tumour growth. Thus, the development of methodologies to rewire M2-like tumour-associated macrophages (TAMs) into the M1 phenotype, which inhibits tumour growth, might be a critical advancement in cancer immunotherapy research.

Methods The expressions of IL-33 and indicators related to macrophage polarization in oesophageal squamous cell carcinoma (ESCC) tissues and peripheral blood mononuclear cell (PBMC)-derived macrophages were determined. Inhibition of ornithine decarboxylase (ODC) with small interfering RNA was used to analyse the phenotype of macrophage polarization and polyamine secretory signals. CCK-8, wound-healing and Transwell assays were used to detect the proliferation and migration of ECA109 cells in vitro. The tumour xenograft assay in nude mice was used to examine the role of IL-33 in ESCC development in vivo.

Results This study showed the substantially elevated IL-33 expression in ESCC tissues compared with the normal tissues. Additionally, enhanced infiltration of M2-like macrophages into the ESCC tumour tissue was also observed. We observed a strong correlation between the IL-33 levels and the infiltration of M2-like macrophages in ESCC tumours locally. Mechanistically, IL-33 induces M2-like macrophage polarization by activating ODC, a key enzyme that catalyses the synthesis of polyamines. Inhibition of ODC suppressed M2-like macrophage polarization. Finally, in vivo, we confirmed that IL-33 promotes tumour progression.

Conclusions This study revealed an oncogenic role of IL-33 by actively inducing M2-like macrophage differentiation; thus, contributing to the formation of an immunosuppressive ESCC tumour microenvironment. Thus, IL-33 could act as a novel target for cancer immunotherapies.

C1 [Mai, Shijie; Jiang, Jianjun; Ren, Pengfei; Diao, Dingwei; Wang, Haofei; Cai, Kaican] Southern Med Univ, Nanfang Hosp, Dept Thorac Surg, Guangzhou 510515, Guangdong, Peoples R China.

[Liu, Le] Southern Med Univ, Nanfang Hosp, Dept Gastroenterol, Guangzhou, Peoples R China.

RP Cai, KC (corresponding author), Southern Med Univ, Nanfang Hosp, Dept Thorac Surg, Guangzhou 510515, Guangdong, Peoples R China.

EM caican@smu.edu.cn

OI Liu, Le/0000-0002-9270-9951

FU Science and Technology Planning Project of Guangdong Province, China

[2014A020212541]

FX This work was supported by the Science and Technology Planning Project

of Guangdong Province, China (2014A020212541).

CR Afferni C, 2018, FRONT IMMUNOL, V9, DOI 10.3389/fimmu.2018.02601

Anfray C, 2020, CELLS-BASEL, V9, DOI 10.3390/cells9010046

Balkwill F, 2004, NAT REV CANCER, V4, P540, DOI 10.1038/nrc1388

Bingle L, 2002, J PATHOL, V196, P254, DOI 10.1002/path.1027

BOTTAZZI B, 1983, SCIENCE, V220, P210, DOI 10.1126/science.6828888

Condeelis J, 2006, CELL, V124, P263, DOI 10.1016/j.cell.2006.01.007

Cui GL, 2019, VIRCHOWS ARCH, V475, P579, DOI 10.1007/s00428-019-02579-9

Cui GL, 2018, CANCER CELL INT, V18, DOI 10.1186/s12935-018-0700-2

De la Fuente M, 2015, CYTOKINE GROWTH F R, V26, P615, DOI 10.1016/j.cytogfr.2015.07.017

Fang M, 2017, CANCER RES, V77, P2735, DOI 10.1158/0008-5472.CAN-16-1602

Gordon S, 2003, NAT REV IMMUNOL, V3, P23, DOI 10.1038/nri978

Gordon S, 2010, IMMUNITY, V32, P593, DOI 10.1016/j.immuni.2010.05.007

Kurowska-Stolarska M, 2009, J IMMUNOL, V183, P6469, DOI 10.4049/jimmunol.0901575

Larsen KM, 2018, INT J MOL SCI, V19, DOI 10.3390/ijms19092676

Li A, 2019, CELL REP, V29, P2998, DOI 10.1016/j.celrep.2019.10.120

Long A, 2018, J IMMUNOL, V201, P3456, DOI 10.4049/jimmunol.1800173

Mantovani A, 2004, TRENDS IMMUNOL, V25, P677, DOI 10.1016/j.it.2004.09.015

Mantovani A, 2002, TRENDS IMMUNOL, V23, P549, DOI 10.1016/S1471-4906(02)02302-5

Mosser DM, 2003, J LEUKOCYTE BIOL, V73, P209, DOI 10.1189/jlb.0602325

Ostuni R, 2015, TRENDS IMMUNOL, V36, P229, DOI 10.1016/j.it.2015.02.004

Qian BZ, 2010, CELL, V141, P39, DOI 10.1016/j.cell.2010.03.014

Verreck FAW, 2004, P NATL ACAD SCI USA, V101, P4560, DOI 10.1073/pnas.0400983101

Wang C, 2017, J MOL CELL CARDIOL, V103, P22, DOI 10.1016/j.yjmcc.2016.12.010

Yue Y, 2020, CANCER SCI, V111, P795, DOI 10.1111/cas.14293

Zhang HF, 2017, EXP MOL MED, V49, DOI 10.1038/emm.2017.183

NR 25

TC 3

Z9 3

U1 4

U2 9

PU WILEY

PI HOBOKEN

PA 111 RIVER ST, HOBOKEN 07030-5774, NJ USA

SN 0960-7722

EI 1365-2184

J9 CELL PROLIFERAT

JI Cell Prolif.

PD FEB

PY 2021

VL 54

IS 2

AR e12960

DI 10.1111/cpr.12960

EA DEC 2020

PG 12

WC Cell Biology

WE Science Citation Index Expanded (SCI-EXPANDED)

SC Cell Biology

GA QF0BC

UT WOS:000597211100001

PM 33305406

OA Green Published, gold

DA 2022-05-19

ER

PT J

AU Wu, XC

Zheng, YF

Tang, M

Li, XF

Zeng, R

Zhang, JR

AF Wu, X. C.

Zheng, Y. F.

Tang, M.

Li, X. F.

Zeng, R.

Zhang, J. R.

TI Association Between Smoking and p53 Mutation in Oesophageal Squamous

Cell Carcinoma: A Meta-analysis

SO CLINICAL ONCOLOGY

LA English

DT Article

DE Carcinoma; meta-analysis; oesophageal squamous cell carcinoma; p53

mutation; risk factor; smoking

ID PAH O-QUINONES; CIGARETTE-SMOKING; CANCER INCIDENCE; REACTIVE OXYGEN;

GENE-MUTATIONS; 5 CONTINENTS; RISK-FACTORS; TP53 GENE; PROTEIN;

PREVALENCE

AB Aims: Several studies have evaluated the association between smoking and p53 mutation in oesophageal squamous cell carcinoma (ESCC), but the conclusions are inconsistent. The aim of the present study was to carry out a meta-analysis evaluating the relationship between smoking and p53 mutation in patients with ESCC.

Materials and methods: Eligible studies were identified through searches in PubMed and EMBASE. The odds ratio with 95% confidence interval was used to assess the association. In total, 20 studies were identified that met the selection criteria; these studies were analysed using STATA 12.0 software.

Results: The 20 studies identified comprised 1524 ESCC patients, of whom 72.97% were smokers and 27.03% were non-smokers. The pooled odds ratio of p53 mutation in ESCC for any cigarette smoking versus no smoking was 1.28 (95% confidence interval = 0.88-1.88). The estimated odds ratios were 1.06 (95% confidence interval =0.56-2.00, based on five studies, 129 light smokers and 70 non-smokers) for light smoking and 2.01 (95% confidence interval = 1.12-3.60, based on five studies, 223 heavy smokers and 73 non-smokers) for heavy smoking.

Conclusion: The results of our meta-analysis indicate an overall positive relationship between heavy smoking and p53 mutation in ESCC. Heavy smokers with ESCC have a higher risk for p53 mutation than non-smokers. Large-scale clinical studies are still needed to draw a more precise conclusion. (C) 2015 The Royal College of Radiologists. Published by Elsevier Ltd. All rights reserved.

C1 [Wu, X. C.; Zheng, Y. F.; Tang, M.; Li, X. F.; Zeng, R.; Zhang, J. R.] Southern Med Univ, Ctr Oncol, Zhujiang Hosp, Guangzhou, Guangdong, Peoples R China.

RP Zheng, YF (corresponding author), Southern Med Univ, Ctr Oncol, Zhujiang Hosp, Guangzhou, Guangdong, Peoples R China.

EM 18665000236@163.com

OI Zheng, Yanfang/0000-0002-5591-6425

FU National Natural Science Foundation of China [81202073]; Ministry of

Education [20114433120009]

FX This study was supported by a grant from the National Natural Science

Foundation of China (grant no. 81202073) and a grant from the Ministry

of Education (grant no. 20114433120009).

CR Abedi-Ardekani B, 2011, PLOS ONE, V6, DOI 10.1371/journal.pone.0029488

AUDREZET MP, 1993, CANCER RES, V53, P5745

BEGG CB, 1994, BIOMETRICS, V50, P1088, DOI 10.2307/2533446

Bellini MF, 2012, J BIOMED BIOTECHNOL, DOI 10.1155/2012/891961

Bennett WP, 1997, CANCER EPIDEM BIOMAR, V6, P963

BRENNAN JA, 1995, NEW ENGL J MED, V332, P712, DOI 10.1056/NEJM199503163321104

Chen ML, 2013, BMC CANCER, V13, DOI 10.1186/1471-2407-13-539

Corley DA, 2001, INT J EPIDEMIOL, V30, P1415, DOI 10.1093/ije/30.6.1415

DeMarini DM, 2004, MUTAT RES-REV MUTAT, V567, P447, DOI 10.1016/j.mrrev.2004.02.001

DERSIMONIAN R, 1986, CONTROL CLIN TRIALS, V7, P177, DOI 10.1016/0197-2456(86)90046-2

Egashira A, 2007, CANCER SCI, V98, P1152, DOI 10.1111/j.1349-7006.2007.00524.x

Egger M, 1997, BMJ-BRIT MED J, V315, P629, DOI 10.1136/bmj.315.7109.629

Gaur D, 1997, CLIN CANCER RES, V3, P2129

Higgins JPT, 2003, BRIT MED J, V327, P557, DOI 10.1136/bmj.327.7414.557

HOLLSTEIN M, 1991, SCIENCE, V253, P49, DOI 10.1126/science.1905840

HOLLSTEIN MC, 1991, CANCER RES, V51, P4102

Hu N, 2001, CLIN CANCER RES, V7, P883

Jemal A, 2011, CA-CANCER J CLIN, V61, P69, DOI [10.3322/caac.20107, 10.3322/caac.20115]

Kamangar F, 2006, J CLIN ONCOL, V24, P2137, DOI 10.1200/JCO.2005.05.2308

Kato H, 2001, CANCER LETT, V167, P65, DOI 10.1016/S0304-3835(01)00461-X

Lam KY, 1997, INT J CANCER, V74, P212, DOI 10.1002/(SICI)1097-0215(19970422)74:2<212::AID-IJC13>3.0.CO;2-F

Lea IA, 2007, CARCINOGENESIS, V28, P1851, DOI 10.1093/carcin/bgm176

Lee JM, 2006, MUTAT RES-FUND MOL M, V594, P181, DOI 10.1016/j.mrfmmm.2005.09.003

LEVINE AJ, 1991, NATURE, V351, P453, DOI 10.1038/351453a0

MANTEL N, 1959, J NATL CANCER I, V22, P719

Mir MM, 2005, INT J CANCER, V116, P62, DOI 10.1002/ijc.21002

Mitsudomi T, 2000, CLIN CANCER RES, V6, P4055

Mizobuchi S, 2000, JPN J CLIN ONCOL, V30, P423, DOI 10.1093/jjco/hyd114

Montesano R, 1996, INT J CANCER, V69, P225, DOI 10.1002/(SICI)1097-0215(19960621)69:3<225::AID-IJC13>3.0.CO;2-6

Montesano R, 1996, Ann Ist Super Sanita, V32, P73

Okuda E, 2001, CLIN CANCER RES, V7, P600

Pharoah PDP, 1999, BRIT J CANCER, V80, P1968, DOI 10.1038/sj.bjc.6690628

Pickens A, 2003, ANN THORAC SURG, V76, pS1367, DOI 10.1016/S0003-4975(03)01202-5

PUISIEUX A, 1991, CANCER RES, V51, P6185

Putz A, 2002, INT J CANCER, V98, P99, DOI 10.1002/ijc.10128

Roshandel G, 2012, ARCH IRAN MED, V15, P713, DOI 0121511/AIM.0013

Rossini A, 2010, MUTAT RES-GEN TOX EN, V696, P10, DOI 10.1016/j.mrgentox.2009.11.005

Saeki H, 2000, BRIT J CANCER, V82, P1892, DOI 10.1054/bjoc.1999.1212

Sengpiel C, 2009, CANCER INVEST, V27, P96, DOI 10.1080/07357900802161047

Shen YM, 2006, CHEM RES TOXICOL, V19, P1441, DOI 10.1021/tx0601206

Suwiwat S, 1997, INT J CANCER, V72, P23, DOI 10.1002/(SICI)1097-0215(19970703)72:1<23::AID-IJC2>3.0.CO;2-6

Szymanska K, 2010, CARCINOGENESIS, V31, P1054, DOI 10.1093/carcin/bgp212

Taghavi N, 2010, WORLD J GASTROENTERO, V16, P4958, DOI 10.3748/wjg.v16.i39.4958

Thongsuksai P, 2010, WORLD J GASTROENTERO, V16, P5359, DOI 10.3748/wjg.v16.i42.5359

WAGATA T, 1993, CANCER RES, V53, P846

Wang Xiao-li, 2004, Zhonghua Liu Xing Bing Xue Za Zhi, V25, P769

World Health Organization. International Agency for Research on Cancer, 2004, IARC MONOGR EVAL CAR, V83, P1

Yu DS, 2002, CHEM RES TOXICOL, V15, P832, DOI 10.1021/tx010177m

NR 48

TC 4

Z9 4

U1 1

U2 9

PU ELSEVIER SCIENCE LONDON

PI LONDON

PA 84 THEOBALDS RD, LONDON WC1X 8RR, ENGLAND

SN 0936-6555

EI 1433-2981

J9 CLIN ONCOL-UK

JI Clin. Oncol.

PD JUN

PY 2015

VL 27

IS 6

BP 337

EP 344

DI 10.1016/j.clon.2015.02.007

PG 8

WC Oncology

WE Science Citation Index Expanded (SCI-EXPANDED)

SC Oncology

GA CH2XB

UT WOS:000353887200020

PM 25736278

DA 2022-05-19

ER

PT J

AU Li, H

Chen, TW

Li, ZL

Zhang, XM

Chen, XL

Wang, LY

Zhou, L

Li, R

Li, CP

Huang, XH

AF Li, Hang

Chen, Tian-wu

Li, Zhen-lin

Zhang, Xiao-ming

Chen, Xiao-li

Wang, Li-ying

Zhou, Li

Li, Rui

Li, Chun-ping

Huang, Xiao-hua

TI Tumour size of resectable oesophageal squamous cell carcinoma measured

with multidetector computed tomography for predicting regional lymph

node metastasis and N stage

SO EUROPEAN RADIOLOGY

LA English

DT Article

DE Oesophagus; Squamous cell carcinoma; Lymph node metastasis; N stage;

Computed tomography

ID PROGNOSTIC-SIGNIFICANCE; THORACIC ESOPHAGUS; CANCER-PATIENTS; SURVIVAL;

VOLUME; TONGUE; LENGTH; THICKNESS; DIAGNOSIS; INVASION

AB To determine whether and how tumour size of resectable oesophageal squamous cell carcinoma (ESCC) measured with multidetector CT could predict regional lymph node metastasis (LNM) and N stage.

Two hundred five patients with ESCC underwent radical oesophagectomy with three-field lymphadenectomy less than 3 weeks after contrast-enhanced CT. Tumour size of the ESCC (tumour length, maximal thickness and gross tumour volume, GTV) was measured on CT. Statistical analyses were performed to identify whether tumour size could predict regional LNM and N stage, and to determine how to use the size of ESCC to predict N stage.

Univariate analysis showed that tumour size could predict regional LNM (all P < 0.05). Logistic regression analysis revealed that GTV could independently predict regional LNM (P = 0.021, odds ratio = 1.813). Mann-Whitney tests showed that tumour size could distinguish grouped N stages (all P < 0.05). GTV might be a differentiating indicator between N0 and N1-3 stages (cutoff, 14.4 cm(3)), between N0-1 and N2-3 (cutoff, 15.9 cm(3)), and between N0-2 and N3 (cutoff, 26.1 cm(3)), with sensitivity of 76%, 63% or 75%, and specificity of 75%, 61% and 81%, respectively.

The GTV of ESCC measured with CT could be an indicator for predicting regional LNM and grouped N stages.

aEuro cent Multidetector computed tomography (MDCT) allows accurate assessment of oesophageal tumour size

aEuro cent For resectable squamous cell tumours, size helped predict regional lymph node involvement

aEuro cent Gross tumour volume may predict the N stage of oesophageal squamous cell carcinoma.

C1 [Li, Hang; Chen, Tian-wu; Zhang, Xiao-ming; Chen, Xiao-li; Wang, Li-ying; Zhou, Li; Li, Rui; Li, Chun-ping; Huang, Xiao-hua] N Sichuan Med Coll, Sichuan Key Lab Med Imaging, Nanchong 637000, Sichuan, Peoples R China.

[Li, Hang; Chen, Tian-wu; Zhang, Xiao-ming; Chen, Xiao-li; Wang, Li-ying; Zhou, Li; Li, Rui; Li, Chun-ping; Huang, Xiao-hua] N Sichuan Med Coll, Dept Radiol, Affiliated Hosp, Nanchong 637000, Sichuan, Peoples R China.

[Li, Zhen-lin] Sichuan Univ, W China Hosp, Dept Radiol, Chengdu 610041, Sichuan, Peoples R China.

RP Chen, TW (corresponding author), N Sichuan Med Coll, Sichuan Key Lab Med Imaging, 63 Wenhua Rd, Nanchong 637000, Sichuan, Peoples R China.

EM twchenscu@yahoo.com.cn

RI Zhang, Xiao Ming/H-7104-2019

OI Zhang, Xiao Ming/0000-0001-5327-8506

FU Science Foundation for Distinguished Young Scholars of Sichuan Province

in China [2010JQ0039]; Scientific Research Project of the Sichuan

Province Health Office in China [100146]

FX This study was supported by the Science Foundation for Distinguished

Young Scholars of Sichuan Province in China (grant no. 2010JQ0039), and

the Scientific Research Project of the Sichuan Province Health Office in

China (grant no. 100146).

CR Bhutani MS, 1997, GASTROINTEST ENDOSC, V45, P474, DOI 10.1016/S0016-5107(97)70176-7

Crehange G, 2006, AM J CLIN ONCOL-CANC, V29, P583, DOI 10.1097/01.coc.0000242346.25229.48

DeMeester SR, 2006, ANN SURG ONCOL, V13, P12, DOI 10.1245/ASO.2005.12.025

Drudi F M, 2002, Radiol Med, V103, P344

Edge SB, 2010, AJCC CANC STAGING MA, V7th ed

Eloubeidi MA, 2002, CANCER-AM CANCER SOC, V95, P1434, DOI 10.1002/cncr.10868

Gaur P, 2010, ANN SURG, V252, P611, DOI 10.1097/SLA.0b013e3181f56419

Goldson Tove M, 2010, J Exp Ther Oncol, V8, P211

Greene FL, 2002, ESOPHAGUS AM JOINT C, P167

Griffiths EA, 2006, J SURG ONCOL, V93, P258, DOI 10.1002/jso.20449

JOHNSON CR, 1995, INT J RADIAT ONCOL, V32, P635, DOI 10.1016/0360-3016(95)00031-S

Khan OA, 2004, BRIT J SURG, V91, P1586, DOI 10.1002/bjs.4778

Kim DU, 2008, J GASTROEN HEPATOL, V23, P619, DOI 10.1111/j.1440-1746.2007.05259.x

Kimura Y, 2005, AM J NEURORADIOL, V26, P2384

Kobori D, 1999, CANCER-AM CANCER SOC, V86, P1638, DOI 10.1002/(SICI)1097-0142(19991101)86:9<1638::AID-CNCR4>3.0.CO;2-U

Kuriakose MA, 2000, BRIT J ORAL MAX SURG, V38, P460, DOI 10.1054/bjom.2000.0316

Li HC, 2007, EUR SURG RES, V39, P1, DOI 10.1159/000096925

Liang EY, 1996, BRIT J RADIOL, V69, P344, DOI 10.1259/0007-1285-69-820-344

LIEBERMANNMEFFE.D, 1996, SHACKELFORDS SURG AL, P3

MOSS AA, 1981, AM J ROENTGENOL, V136, P1051, DOI 10.2214/ajr.136.6.1051

MURAKAMI G, 1994, SURG RADIOL ANAT, V16, P399, DOI 10.1007/BF01627660

O-charoenrat P, 2003, ORAL ONCOL, V39, P386, DOI 10.1016/S1368-8375(02)00142-2

Okuda Itsuko, 2002, Nihon Geka Gakkai Zasshi, V103, P331

Okura M, 2008, AM J NEURORADIOL, V29, P45, DOI 10.3174/ajnr.A0749

Rice TW, 1998, ANN THORAC SURG, V65, P787, DOI 10.1016/S0003-4975(97)01387-8

Seok IH, 2012, CLIN NUCL MED, V37, P442, DOI 10.1097/RLU.0b013e318238f703

Sillah K, 2010, WORLD J GASTRO ONCOL, V2, P197, DOI 10.4251/wjgo.v2.i4.197

Tachibana M, 1999, ARCH SURG-CHICAGO, V134, P50, DOI 10.1001/archsurg.134.1.50

Tachibana M, 1999, AM J GASTROENTEROL, V94, P757

Tachibana M, 2001, DIGEST LIVER DIS, V33, P534, DOI 10.1016/S1590-8658(01)80103-1

Talback M, 2004, CANCER CAUSE CONTROL, V15, P967, DOI 10.1007/s10522-004-2475-1

Twine CP, 2010, SURG ENDOSC, V24, P870, DOI 10.1007/s00464-009-0681-2

Xiao ZF, 2005, INT J RADIAT ONCOL, V62, P82, DOI 10.1016/j.ijrobp.2004.08.046

Xu QR, 2011, WORLD J SURG, V35, P1303, DOI 10.1007/s00268-011-1015-9

Yoon YC, 2003, RADIOLOGY, V227, P764, DOI 10.1148/radiol.2281020423

NR 35

TC 16

Z9 16

U1 0

U2 3

PU SPRINGER

PI NEW YORK

PA 233 SPRING ST, NEW YORK, NY 10013 USA

SN 0938-7994

EI 1432-1084

J9 EUR RADIOL

JI Eur. Radiol.

PD NOV

PY 2012

VL 22

IS 11

BP 2487

EP 2493

DI 10.1007/s00330-012-2512-4

PG 7

WC Radiology, Nuclear Medicine & Medical Imaging

WE Science Citation Index Expanded (SCI-EXPANDED)

SC Radiology, Nuclear Medicine & Medical Imaging

GA 021EZ

UT WOS:000309869400024

PM 22653286

DA 2022-05-19

ER

PT J

AU Migita, K

Sho, M

Shimada, K

Yasuda, S

Yamato, I

Takayama, T

Matsumoto, S

Wakatsuki, K

Hotta, K

Tanaka, T

Ito, M

Konishi, N

Nakajima, Y

AF Migita, Kazuhiro

Sho, Masayuki

Shimada, Keiji

Yasuda, Satoshi

Yamato, Ichiro

Takayama, Tomoyoshi

Matsumoto, Sohei

Wakatsuki, Kohei

Hotta, Kiyohiko

Tanaka, Tetsuya

Ito, Masahiro

Konishi, Noboru

Nakajima, Yoshiyuki

TI Significant Involvement of Herpesvirus Entry Mediator in Human

Esophageal Squamous Cell Carcinoma

SO CANCER

LA English

DT Article

DE esophageal squamous cell carcinoma; herpesvirus entry mediator (HVEM);

prognosis; immunotherapy

ID CD8(+) T-CELLS; LYMPHOTOXIN-BETA-RECEPTOR; CLINICAL-SIGNIFICANCE;

B-LYMPHOCYTE; LIGHT; ACTIVATION; LIGAND; CANCER; HVEM; PROLIFERATION

AB BACKGROUNDHerpesvirus entry mediator (HVEM) is known to regulate immune response and to be expressed in several human malignancies. However, to the authors's knowledge, the precise role of HVEM in human cancer biology remains unknown. The objective of the current study was to clarify the clinical significance of HVEM in human esophageal squamous cell carcinoma as well as its in vivo functions.

METHODSHVEM expression was evaluated in 103 patients with esophageal squamous cell carcinoma to explore its clinical relevance and prognostic value. The functions of HVEM in tumors were analyzed in vitro and in vivo using the small interfering RNA (siRNA) silencing technique.

RESULTSHVEM expression was found to be significantly correlated with depth of tumor invasion and lymph node metastasis. Furthermore, it was found to be inversely correlated with tumor-infiltrating CD4(+), CD8(+), and CD45RO(+) lymphocytes. It is important to note that HVEM status was identified as an independent prognostic marker. HVEM gene silencing significantly inhibited cancer cell proliferation in vitro and cancer growth in vivo. This antitumor effect was associated with reduced cell proliferation activity. The effect was also correlated with the induction of CD8(+) cells and upregulation of local immune response.

CONCLUSIONSHVEM plays a critical role in both tumor progression and the evasion of host antitumor immune responses, possibly through direct and indirect mechanisms. Therefore, HVEM may be a promising therapeutic target for human esophageal cancer. Cancer 2014;120:808-817. (c) 2013 American Cancer Society.

Herpesvirus entry mediator plays a critical role in both tumor progression and the evasion of host antitumor immune responses in patients with esophageal cancer through direct and indirect mechanisms. Therefore, herpesvirus entry mediator may be a promising therapeutic target for human malignancy.

C1 [Migita, Kazuhiro; Sho, Masayuki; Yasuda, Satoshi; Yamato, Ichiro; Takayama, Tomoyoshi; Matsumoto, Sohei; Wakatsuki, Kohei; Hotta, Kiyohiko; Tanaka, Tetsuya; Ito, Masahiro; Nakajima, Yoshiyuki] Nara Med Univ, Dept Surg, Nara 634, Japan.

[Shimada, Keiji; Konishi, Noboru] Nara Med Univ, Dept Pathol, Nara 634, Japan.

RP Sho, M (corresponding author), Nara Med Univ, Dept Surg, 840 Shijo Cho, Kashihara, Nara 6348522, Japan.

EM m-sho@naramed-u.ac.jp

RI Yasuda, Satoshi/AAD-2497-2022

OI Yasuda, Satoshi/0000-0003-4323-7422

FU Ministry of Education, Culture, Sports, Science and Technology of Japan

[21591648, 24591887, 24791443]; Takeda Science Foundation; Nakayama

Cancer Research Institute

FX Supported by Grants-in-Aid for Scientific Research from the Ministry of

Education, Culture, Sports, Science and Technology of Japan (no.

21591648 and no. 24591887 to Dr. Sho and no. 24791443 to Dr. Migita); a

research grant from Takeda Science Foundation; and a research grant from

Nakayama Cancer Research Institute (to Dr. Sho).

CR Barnas C, 1997, INT J CANCER, V71, P79, DOI 10.1002/(SICI)1097-0215(19970328)71:1<79::AID-IJC14>3.0.CO;2-4

Brahmer JR, 2012, NEW ENGL J MED, V366, P2455, DOI 10.1056/NEJMoa1200694

Cai GF, 2008, NAT IMMUNOL, V9, P176, DOI 10.1038/ni1554

Cheung TC, 2009, P NATL ACAD SCI USA, V106, P6244, DOI 10.1073/pnas.0902115106

Cho Y, 2003, CANCER RES, V63, P1555

Cunningham D, 2006, NEW ENGL J MED, V355, P11, DOI 10.1056/NEJMoa055531

Derre L, 2010, J CLIN INVEST, V120, P157, DOI 10.1172/JCI40070

DiMenna L, 2010, J IMMUNOL, V184, P5475, DOI 10.4049/jimmunol.0903808

Dong HD, 2002, NAT MED, V8, P793, DOI 10.1038/nm730

Enomoto K, 2012, CLIN EXP IMMUNOL, V168, P186, DOI 10.1111/j.1365-2249.2012.04565.x

Han LF, 2009, J IMMUNOL, V183, P7842, DOI 10.4049/jimmunol.0804379

Harrop JA, 1998, J BIOL CHEM, V273, P27548, DOI 10.1074/jbc.273.42.27548

Hobo W, 2012, J IMMUNOL, V189, P39, DOI 10.4049/jimmunol.1102807

Iwai Y, 2002, P NATL ACAD SCI USA, V99, P12293, DOI 10.1073/pnas.192461099

Klein CA, 2009, CANCER RES, V69, P5285, DOI 10.1158/0008-5472.CAN-08-4586

Kleinberg L, 2007, J CLIN ONCOL, V25, DOI 10.1200/JCO.2007.12.0881

Krieg C, 2007, NAT IMMUNOL, V8, P162, DOI 10.1038/ni1418

Kwon BS, 1997, J BIOL CHEM, V272, P14272, DOI 10.1074/jbc.272.22.14272

Lasaro MO, 2008, NAT MED, V14, P205, DOI 10.1038/nm1704

Lasaro MO, 2011, MOL THER, V19, P1727, DOI 10.1038/mt.2011.88

Montgomery RI, 1996, CELL, V87, P427, DOI 10.1016/S0092-8674(00)81363-X

Nomi T, 2007, CLIN CANCER RES, V13, P2151, DOI 10.1158/1078-0432.CCR-06-2746

Ohigashi Y, 2005, CLIN CANCER RES, V11, P2947, DOI 10.1158/1078-0432.CCR-04-1469

Okines A, 2010, NAT REV CLIN ONCOL, V7, P231, DOI 10.1038/nrclinonc.2010.20

Pasero C, 2009, EUR J IMMUNOL, V39, P2502, DOI 10.1002/eji.200939069

Rauser S, 2010, BMC CANCER, V10, DOI 10.1186/1471-2407-10-608

Sakoda Y, 2011, BLOOD, V117, P2506, DOI 10.1182/blood-2010-08-301325

Sarrias MR, 2000, MOL IMMUNOL, V37, P665, DOI 10.1016/S0161-5890(00)00089-4

Sedy JR, 2005, NAT IMMUNOL, V6, P90, DOI 10.1038/ni1144

Sobin L, 2009, INT UNION CANC UICC

Soroosh P, 2011, J EXP MED, V208, P797, DOI 10.1084/jem.20101562

Steinberg MW, 2008, J EXP MED, V205, P1463, DOI 10.1084/jem.20071160

Tamada K, 2000, J IMMUNOL, V164, P4105, DOI 10.4049/jimmunol.164.8.4105

Thompson RH, 2006, CANCER RES, V66, P3381, DOI 10.1158/0008-5472.CAN-05-4303

Topalian SL, 2012, NEW ENGL J MED, V366, P2443, DOI 10.1056/NEJMoa1200690

Vogelstein B, 2000, NATURE, V408, P307, DOI 10.1038/35042675

Wang Y, 2005, J CLIN INVEST, V115, P711, DOI 10.1172/JCI200522982

Yamato I, 2012, CANCER RES, V72, P4829, DOI 10.1158/0008-5472.CAN-12-0328

Zhai YF, 1998, J CLIN INVEST, V102, P1142, DOI 10.1172/JCI3492

Zhang L, 2003, NEW ENGL J MED, V348, P203, DOI 10.1056/NEJMoa020177

NR 40

TC 22

Z9 23

U1 0

U2 6

PU WILEY

PI HOBOKEN

PA 111 RIVER ST, HOBOKEN 07030-5774, NJ USA

SN 0008-543X

EI 1097-0142

J9 CANCER-AM CANCER SOC

JI Cancer

PD MAR 15

PY 2014

VL 120

IS 6

BP 808

EP 817

DI 10.1002/cncr.28491

PG 10

WC Oncology

WE Science Citation Index Expanded (SCI-EXPANDED)

SC Oncology

GA AB9UB

UT WOS:000332140100008

PM 24249528

OA Green Submitted

DA 2022-05-19

ER

PT J

AU Guo, W

Dong, ZM

Lin, XW

Zhang, MH

Kuang, G

Zhu, T

AF Guo, Wei

Dong, Zhiming

Lin, Xinwen

Zhang, Minghui

Kuang, Gang

Zhu, Tienian

TI Decreased Expression and Aberrant Methylation of Raf Kinase Inhibitory

Protein Gene in Esophageal Squamous Cell Carcinoma

SO CANCER INVESTIGATION

LA English

DT Article

DE RKIP; Esophageal squamous cell carcinoma; Expression; Methylation

ID BREAST-CANCER; COLORECTAL-CANCER; METASTASIS; RKIP; PROGRESSION;

PROLIFERATION; SUPPRESSION; REDUCTION; PROSTATE

AB Raf kinase inhibitory protein (RKIP) gene is considered to be a suppressor of metastasis involved in various carcinomas. In the present study, we observed that promoter methylation repressed the expression of RKIP in TE-13 cell line. 5-Aza treatment and stable transfection of RKIP resulted in a significant inhibition of TE-13 cell proliferation. The promoter hypermethylation of RKIP was found to occur in dysplastic tissues and a close correlation was noted between RKIP methylation and the loss of mRNA and protein expression of the gene in ESCC specimens. In summary, RKIP may act as a tumor suppressor gene in esophageal cancer.

C1 [Guo, Wei; Zhu, Tienian] Bethune Int Peace Hosp, Tumor Treatment Dept, Shijiazhuang 050082, Hebei, Peoples R China.

[Dong, Zhiming; Lin, Xinwen; Zhang, Minghui; Kuang, Gang] Hebei Med Univ, Pathol Lab, Hebei Canc Inst, Hosp 4, Shijiazhuang, Hebei, Peoples R China.

RP Zhu, T (corresponding author), Bethune Int Peace Hosp, Tumor Treatment Dept, Zhongshanlu 398, Shijiazhuang 050082, Hebei, Peoples R China.

EM sjzgw73@yahoo.cn

CR Al-Mulla F, 2008, J CLIN PATHOL, V61, P524, DOI 10.1136/jcp.2007.046987

Al-Mulla F, 2006, J CLIN ONCOL, V24, P5672, DOI 10.1200/JCO.2006.07.5499

BERNIER I, 1986, BIOCHIM BIOPHYS ACTA, V871, P19, DOI 10.1016/0167-4838(86)90128-7

Bouras T, 2001, CANCER RES, V61, P903

Chatterjee D, 2004, J BIOL CHEM, V279, P17515, DOI 10.1074/jbc.M313816200

Fu Z, 2003, J NATL CANCER I, V95, P878, DOI 10.1093/jnci/95.12.878

Granovsky AE, 2008, CELL RES, V18, P452, DOI 10.1038/cr.2008.43

Guo W, 2008, J CANCER RES CLIN, V134, P263, DOI 10.1007/s00432-007-0283-0

Guo W, 2009, CANCER INVEST, V27, P459, DOI 10.1080/07357900802620828

Hagan S, 2005, CLIN CANCER RES, V11, P7392, DOI 10.1158/1078-0432.CCR-05-0283

Hagan S, 2006, METHOD ENZYMOL, V407, P248, DOI 10.1016/S0076-6879(05)07021-7

Keller ET, 2004, BIOCHEM PHARMACOL, V68, P1049, DOI 10.1016/j.bcp.2004.04.024

Klysik J, 2008, CELL SIGNAL, V20, P1, DOI 10.1016/j.cellsig.2007.07.003

Kolch W, 2000, BIOCHEM J, V351, P289, DOI 10.1042/0264-6021:3510289

Kolch W, 2005, NAT REV MOL CELL BIO, V6, P827, DOI 10.1038/nrm1743

Lee HC, 2006, GASTROENTEROLOGY, V131, P1208, DOI 10.1053/j.gastro.2006.07.012

Li HZ, 2008, MOL CANCER RES, V6, P917, DOI 10.1158/1541-7786.MCR-08-0093

Li HZ, 2009, MOL CANCER RES, V7, P832, DOI 10.1158/1541-7786.MCR-08-0403

Martinho O, 2009, VIRCHOWS ARCH, V455, P277, DOI 10.1007/s00428-009-0821-z

Minoo P, 2006, GUT, V55, DOI 10.1136/gut.2005.082859

Minoo P, 2007, AM J CLIN PATHOL, V127, P820, DOI 10.1309/5D7MM22DAVGDT1R8

Nguyen CT, 2002, CANCER RES, V62, P6456

Park S, 2005, ONCOGENE, V24, P3535, DOI 10.1038/sj.onc.1208435

Sarwar CMS, 2010, INT J SURG, V8, P417, DOI 10.1016/j.ijsu.2010.06.011

Schuierer MM, 2006, ONCOL REP, V16, P451

Schuierer MM, 2004, CANCER RES, V64, P5186, DOI 10.1158/0008-5472.CAN-03-3861

Wu DL, 2006, WORLD J GASTROENTERO, V12, P6933, DOI 10.3748/wjg.v12.i43.6933

Yeung K, 1999, NATURE, V401, P173, DOI 10.1038/43686

Yeung KC, 2001, MOL CELL BIOL, V21, P7207, DOI 10.1128/MCB.21.21.7207-7217.2001

Zhang LZ, 2004, SURGERY, V136, P708, DOI 10.1016/j.surg.2003.12.013

NR 30

TC 12

Z9 17

U1 0

U2 1

PU INFORMA HEALTHCARE

PI LONDON

PA TELEPHONE HOUSE, 69-77 PAUL STREET, LONDON EC2A 4LQ, ENGLAND

SN 0735-7907

J9 CANCER INVEST

JI Cancer Invest.

PD DEC

PY 2012

VL 30

IS 10

BP 703

EP 711

DI 10.3109/07357907.2012.732164

PG 9

WC Oncology

WE Science Citation Index Expanded (SCI-EXPANDED)

SC Oncology

GA 047KF

UT WOS:000311838600003

PM 23066640

DA 2022-05-19

ER

PT J

AU Isidro, RA

Dong, F

Hornick, JL

Wee, JO

Agoston, A

Patil, DT

Deshpande, V

Zhao, L

AF Isidro, Raymond A.

Dong, Fei

Hornick, Jason L.

Wee, Jon O.

Agoston, Agoston

Patil, Deepa T.

Deshpande, Vikram

Zhao, Lei

TI Verrucous carcinoma of the oesophagus is a genetically distinct subtype

of oesophageal squamous cell carcinoma

SO HISTOPATHOLOGY

LA English

DT Article

DE cancer genomics; oesophageal squamous cell carcinoma; verrucous

carcinoma

ID XL-ALPHA-S; G-PROTEIN; MUTATIONS; COMPLEXES; SUBUNIT; CANCER; TUMOR; DNA

AB Aims Oesophageal verrucous carcinoma (VSCC) is a rare and morphologically distinct type of oesophageal squamous cell carcinoma (SCC). Diagnosing VSCC on biopsy material is challenging, given the lack of significant atypia and the presence of keratinising epithelium and exophytic growth. The molecular pathogenesis of VSCC remains unclear. The aim of this study was to characterise the genomic landscape of VSCC in comparison to conventional oesophageal SCC. Methods and results Three cases of VSCC from the Brigham and Women's Hospital pathology archive were identified. Formalin-fixed, paraffin-embedded (FFPE) tumour tissue was used for p16 immunohistochemistry (IHC), high-risk human papillomavirus (HPV) in-situ mRNA hybridisation (ISH) and DNA isolation. Tumour DNA was sequenced using a targeted massively parallel sequencing assay enriched for cancer-associated genes. Three additional cases of VSCC were identified by image review of The Cancer Genome Atlas (TCGA) oesophageal SCC cohort. VSCC cases were negative for p16 IHC and high-risk HPV ISH. TP53 mutations (P < 0.001) and copy number variants (CNVs) for CDKN2A (P < 0.001), CDKN2B (P < 0.01) and CCND1 (P < 0.01) were absent in VSCC and significantly less frequent in comparison to conventional SCC. Five VSCC cases featured SMARCA4 missense mutations or in-frame deletions compared to only four of 88 conventional SCC cases (P < 0.001). VSCC featured driver mutations in PIK3CA, HRAS and GNAS. Recurrent CNVs were rare in VSCC. Conclusions VSCC is not only morphologically but also genetically distinct from conventional oesophageal SCC, featuring frequent SMARCA4 mutations and infrequent TP53 mutations or CDKN2A/B CNVs. Molecular findings may aid in establishing the challenging diagnosis of VSCC.

C1 [Isidro, Raymond A.; Dong, Fei; Hornick, Jason L.; Agoston, Agoston; Patil, Deepa T.; Zhao, Lei] Brigham & Womens Hosp, Dept Pathol, 75 Francis St, Boston, MA 02115 USA.

[Isidro, Raymond A.; Dong, Fei; Hornick, Jason L.; Wee, Jon O.; Agoston, Agoston; Patil, Deepa T.; Deshpande, Vikram; Zhao, Lei] Harvard Med Sch, Boston, MA 02115 USA.

[Wee, Jon O.] Brigham & Womens Hosp, Dept Surg, 75 Francis St, Boston, MA 02115 USA.

[Deshpande, Vikram] Massachusetts Gen Hosp, Dept Pathol, Boston, MA 02114 USA.

RP Zhao, L (corresponding author), Brigham & Womens Hosp, Dept Pathol, 75 Francis St, Boston, MA 02115 USA.

EM lzhao19@bwh.harvard.edu

OI zhao, lei/0000-0003-4322-6475

FU [2018P001007]

FX This study was approved by the institutional review board at BWH

(#2018P001007).

CR Abramowitz J, 2004, P NATL ACAD SCI USA, V101, P8366, DOI 10.1073/pnas.0308758101

Al-Shoha M, 2018, AM J GASTROENTEROL, V113, P919, DOI 10.1038/s41395-018-0065-0

Aydin C, 2009, ENDOCRINOLOGY, V150, P3567, DOI 10.1210/en.2009-0318

Bendell JC, 2012, J CLIN ONCOL, V30, P282, DOI 10.1200/JCO.2011.36.1360

Cappellesso R, 2019, HUM PATHOL, V85, P50, DOI 10.1016/j.humpath.2018.10.024

Chen XX, 2017, NAT COMMUN, V8, DOI 10.1038/s41467-017-00650-0

Davis H, 2014, GUT, V63, P792, DOI 10.1136/gutjnl-2013-304719

Davis RJ, 2014, CANCER CELL, V26, P455, DOI 10.1016/j.ccell.2014.09.013

Dykhuizen EC, 2013, NATURE, V497, P624, DOI 10.1038/nature12146

Ereno C, 2001, ENDOSCOPY, V33, P297, DOI 10.1055/s-2001-12806

Errichiello E, 2017, J PATHOL, V243, P9, DOI 10.1002/path.4926

Fecteau RE, 2014, PLOS ONE, V9, DOI 10.1371/journal.pone.0087966

Garcia EP, 2017, ARCH PATHOL LAB MED, V141, P751, DOI 10.5858/arpa.2016-0527-OA

Hodges HC, 2018, NAT STRUCT MOL BIOL, V25, P61, DOI 10.1038/s41594-017-0007-3

Howlader NR, 2020, BREAST CANCER RES TR, V182, P739, DOI 10.1007/s10549-020-05738-8

Husain A, 2016, NAT COMMUN, V7, DOI 10.1038/ncomms10549

Janku F, 2013, CANCER RES, V73, P276, DOI 10.1158/0008-5472.CAN-12-1726

Kang SY, 2005, P NATL ACAD SCI USA, V102, P802, DOI 10.1073/pnas.0408864102

Kiessling MK, 2015, ONCOTARGET, V6, P42183, DOI 10.18632/oncotarget.5619

Kim J, 2017, NATURE, V541, P169, DOI 10.1038/nature20805

Komatsu H, 2014, PLOS ONE, V9, DOI 10.1371/journal.pone.0087875

Macias-Garcia F, 2010, ENDOSCOPY, V42, pE137, DOI 10.1055/s-0029-1244051

Osborn NK, 2003, DIGEST DIS SCI, V48, P465, DOI 10.1023/A:1022572229285

Pouw RE, 2017, CLIN GASTROENTEROL H, V15, pE141, DOI 10.1016/j.cgh.2016.12.027

Shlien A, 2008, P NATL ACAD SCI USA, V105, P11264, DOI 10.1073/pnas.0802970105

SRIDHAR C, 1980, RADIOLOGY, V136, P614, DOI 10.1148/radiology.136.3.7403536

Tabuchi S, 2020, SURG CASE REP, V6, DOI 10.1186/s40792-020-0801-8

Tajiri H, 2000, AM J GASTROENTEROL, V95, P1076

Taki K, 2016, ONCOGENE, V35, P2407, DOI 10.1038/onc.2015.294

Urabe Yuji, 2019, Oncotarget, V10, P4139, DOI 10.18632/oncotarget.27014

Watkins JC, 2017, MODERN PATHOL, V30, P448, DOI 10.1038/modpathol.2016.187

Wilson BG, 2014, MOL CELL BIOL, V34, P1136, DOI 10.1128/MCB.01372-13

Wilson CH, 2010, ONCOGENE, V29, P4567, DOI 10.1038/onc.2010.202

NR 33

TC 1

Z9 1

U1 0

U2 0

PU WILEY

PI HOBOKEN

PA 111 RIVER ST, HOBOKEN 07030-5774, NJ USA

SN 0309-0167

EI 1365-2559

J9 HISTOPATHOLOGY

JI Histopathology

PD OCT

PY 2021

VL 79

IS 4

BP 642

EP 649

DI 10.1111/his.14395

EA JUL 2021

PG 8

WC Cell Biology; Pathology

WE Science Citation Index Expanded (SCI-EXPANDED)

SC Cell Biology; Pathology

GA US8PM

UT WOS:000670551100001

PM 33960520

DA 2022-05-19

ER

PT J

AU Zhu, JF

Feng, XY

Zhang, XW

Wen, YS

Lin, P

Cai, L

Zhang, LJ

AF Zhu, Jian-Fei

Feng, Xing-Yu

Zhang, Xue-Wen

Wen, Yin-Sheng

Lin, Peng

Cai, Ling

Zhang, Lan-Jun

TI Time distribution of recurrence risk of oesophageal squamous cell

carcinoma with complete resection (R0) in a Chinese population

SO EUROPEAN JOURNAL OF CARDIO-THORACIC SURGERY

LA English

DT Article

DE Recurrence risk-free survival; Oesophageal squamous cell carcinoma;

Surgery

ID LONG-TERM SURVIVAL; BREAST-CANCER; ESOPHAGOGASTRIC JUNCTION;

POSTOPERATIVE SURVIVAL; HAZARD RATES; SMOKING; DORMANCY; SURGERY;

ALCOHOL; PATTERN

AB OBJECTIVES: We aimed to clarify the correlation between clinic-pathological characteristics and the distribution of recurrence probability during follow-up of oesophageal squamous cell carcinoma (OSCC) patients with complete resection analysis by hazard function, and to try to provide evidence-based data for optimal timing for adjuvant therapy.

METHODS: A single-institution, retrospective study was conducted on 553 Chinese patients with OSCC who underwent R0 resection between January 2005 and October 2007. Survival curves were generated using the Kaplan-Meier method, and hazard function was used to analyse the annual recurrence hazard.

RESULTS: The median recurrence- free survival time of these patients was 3.4 years. In univariate analysis, the favourable prognostic factors were gender, smoking status, a tumour length of <= 4.0 cm, tumour invasion thickness, normal level of squamous cell carcinoma (SCC) antigen, pathological T category and pathological N category. In multivariate analysis, pathological T category and pathological N category were independent prognostic factors. Overall, the recurrence hazard curve for the entire cohort showed that the first major recurrence surge began to increase from the first year at 22.97% and peaked at 1.3 years at 27.4% during follow-up. The second recurrence surge peaked during the seventh year at 13.0%. A lower recurrence risk was observed in patients with the following clinic-pathological characteristics: gender, smoking status and N0.

CONCLUSIONS: We identify the presence of two peaks for recurrence risk in Chinese patients with resectable OSCC, which might contribute to choosing the optimal timing for adjuvant therapy after an operation to decrease or delay the recurrence hazard for patients with resectable OSCC.

C1 [Zhu, Jian-Fei; Wen, Yin-Sheng; Lin, Peng; Zhang, Lan-Jun] Sun Yat Sen Univ, Ctr Canc, Dept Thorac Surg, Guangzhou 510060, Guangdong, Peoples R China.

[Feng, Xing-Yu] Sun Yat Sen Univ, Ctr Canc, Dept Gastropancreat Surg, Guangzhou 510060, Guangdong, Peoples R China.

[Feng, Xing-Yu] Guangdong Gen Hosp, Dept Gastrointestinal Surg & Gen Surg, Guangzhou, Guangdong, Peoples R China.

[Zhang, Xue-Wen] Sun Yat Sen Univ, Ctr Canc, Dept Pathol, Guangzhou, Guangdong, Peoples R China.

[Wen, Yin-Sheng; Lin, Peng; Cai, Ling; Zhang, Lan-Jun] Sun Yat Sen Univ, Ctr Canc, State Key Lab Oncol South China, Guangzhou, Guangdong, Peoples R China.

[Cai, Ling] Sun Yat Sen Univ, Ctr Canc, Dept Radiat Oncol, Guangzhou, Guangdong, Peoples R China.

RP Zhang, LJ (corresponding author), Sun Yat Sen Univ, Ctr Canc, Dept Thorac Surg, Guangzhou 510060, Guangdong, Peoples R China.

EM zhanglj@sysucc.org.cn

RI Zhu, Jianfei/AAP-4683-2020

FU Ministry of Science and Technology of China [2012AA021503]

FX This research was supported by grants from the Ministry of Science and

Technology of China (no. 2012AA021503).

CR Ando N, 2012, ANN SURG ONCOL, V19, P68, DOI 10.1245/s10434-011-2049-9

[Anonymous], 2012, CA-CANCER J CLIN, V62, P215, DOI 10.3322/caac.21144

Cook MB, 2010, JNCI-J NATL CANCER I, V102, P1344, DOI 10.1093/jnci/djq289

Demicheli R, 2007, NAT CLIN PRACT ONCOL, V4, P699, DOI 10.1038/ncponc0999

EDWARDS JM, 1989, BRIT J CANCER, V59, P429, DOI 10.1038/bjc.1989.87

Enzinger PC, 2003, NEW ENGL J MED, V349, P2241, DOI 10.1056/NEJMra035010

Gao F, 2007, BREAST CANCER RES, V9, DOI 10.1186/bcr1658

Hou X, 2013, ANN SURG ONCOL, V20, P1653, DOI 10.1245/s10434-012-2794-4

Jatoi I, 2005, BREAST CANCER RES TR, V89, P173, DOI 10.1007/s10549-004-1722-0

Lin YB, 2012, CANCER SCI, V103, P1985, DOI 10.1111/cas.12000

Lu Siqi, 1999, Chinese Medical Journal (English Edition), V112, P767

NAIR MPN, 1990, CLIN IMMUNOL IMMUNOP, V54, P395, DOI 10.1016/0090-1229(90)90053-S

Osugi H, 2003, ONCOL REP, V10, P81

Rice TW, 2010, ANN SURG ONCOL, V17, P1721, DOI 10.1245/s10434-010-1024-1

Saphner T, 1996, J CLIN ONCOL, V14, P2738, DOI 10.1200/JCO.1996.14.10.2738

Shiozaki A, 2005, ONCOL REP, V13, P667

Shitara K, 2010, CANCER SCI, V101, P1001, DOI 10.1111/j.1349-7006.2009.01466.x

SIMES RJ, 1985, J CLIN ONCOL, V3, P1418, DOI 10.1200/JCO.1985.3.10.1418

Steevens J, 2011, CANCER EPIDEM BIOMAR, V20, P345, DOI 10.1158/1055-9965.EPI-10-0636

VARANI J, 1981, J SURG ONCOL, V17, P9, DOI 10.1002/jso.2930170103

Wu IC, 2013, PLOS ONE, V8, DOI 10.1371/journal.pone.0055834

Yang HX, 2012, ANN THORAC SURG, V93, P1861, DOI 10.1016/j.athoracsur.2012.03.002

Yang HX, 2011, ANN THORAC SURG, V92, P216, DOI 10.1016/j.athoracsur.2011.03.032

Yin WJ, 2009, BREAST CANCER RES TR, V114, P527, DOI 10.1007/s10549-008-0022-5

Zhu ZJ, 2011, ANN THORAC SURG, V91, P1502, DOI 10.1016/j.athoracsur.2011.01.007

NR 25

TC 2

Z9 2

U1 0

U2 4

PU OXFORD UNIV PRESS INC

PI CARY

PA JOURNALS DEPT, 2001 EVANS RD, CARY, NC 27513 USA

SN 1010-7940

EI 1873-734X

J9 EUR J CARDIO-THORAC

JI Eur. J. Cardio-Thorac. Surg.

PD DEC

PY 2015

VL 48

IS 6

BP 899

EP 905

DI 10.1093/ejcts/ezv147

PG 7

WC Cardiac & Cardiovascular Systems; Respiratory System; Surgery

WE Science Citation Index Expanded (SCI-EXPANDED)

SC Cardiovascular System & Cardiology; Respiratory System; Surgery

GA DB3OM

UT WOS:000368421000027

PM 25899517

OA Bronze

DA 2022-05-19

ER

PT J

AU Yang, HS

Liu, W

Zheng, SY

Cai, HY

Luo, HH

Feng, YF

Lei, YY

AF Yang, Hao-Shuai

Liu, Wei

Zheng, Shao-Yi

Cai, He-Yuan

Luo, Hong-He

Feng, Yan-Fen

Lei, Yi-Yan

TI A Novel Ras--Related Signature Improves Prognostic Capacity in

Oesophageal Squamous Cell Carcinoma

SO FRONTIERS IN GENETICS

LA English

DT Article

DE oesophageal squamous cell carcinoma; ras; prognosis; bioinformatics

analysis; TCGA; GTEx

AB Oesophageal squamous cell carcinoma (ESCC) remains a clinically challenging disease with high morbidity rates and poor prognosis. ESCC is also the most common pathological type of oesophageal cancer (EC) in China. Ras-related genes are one of the most frequently mutated gene families in cancer and regulate tumour development and progression. Given this, we investigated the Ras-related gene expression profiles and their values in ESCC prognosis, using data from the Genotype-Tissue Expression (GTEx) and The Cancer Genome Atlas (TCGA) databases. We found that we could identify three distinct oesophageal cancer clusters based on their unique expression profile for 11 differentially expressed Ras-related genes with each of these demonstrating some prognostic value when, evaluated using univariate Cox analysis. We then used multivariate Cox analysis to identify relevant independent prognostic indicators and used these to build a new prognostic prediction model for oesophageal cancer patients using these three Ras-related genes. These evaluations produced an area under the curve (AUC) of 0.932. We found that our Ras-related signatures could also act as independent factors in ESCC prognosis and that patients with low Ras scores showed a higher overall expression levels of various immune checkpoint genes, including TNFSF4, TNFRSF8, TNFRSF9, NRP1, CD28, CD70, CD200, CD276, METTL16, METTL14, ZC3H13, YTHDF3, VIRMA, FTO, and RBM15, as well as a higher CSMD3, FLG, DNAH5, MUC4, PLCO, EYS, and ZNF804B mutation rates, and better sensitivity to drugs such as erlotinib, paclitaxel, and gefitinib. In conclusion, we were able to use the unique expression profiles of several Ras-related genes to produce a novel disease signature which might facilitate improved prognosis in ESCC, providing new insight into both diagnosis and treatment in these cancers.

C1 [Yang, Hao-Shuai; Liu, Wei; Zheng, Shao-Yi; Cai, He-Yuan; Luo, Hong-He; Lei, Yi-Yan] Sun Yat Sen Univ, Affiliated Hosp 1, Dept Thorac Surg, Guangzhou, Peoples R China.

[Feng, Yan-Fen] Sun Yat Sen Univ, Ctr Canc, Collaborat Innovat Ctr Canc Med, State Key Lab Oncol South China, Guangzhou, Peoples R China.

[Feng, Yan-Fen] Sun Yat Sen Univ, Ctr Canc, Dept Pathol, Guangzhou, Peoples R China.

RP Lei, YY (corresponding author), Sun Yat Sen Univ, Affiliated Hosp 1, Dept Thorac Surg, Guangzhou, Peoples R China.; Feng, YF (corresponding author), Sun Yat Sen Univ, Ctr Canc, Collaborat Innovat Ctr Canc Med, State Key Lab Oncol South China, Guangzhou, Peoples R China.; Feng, YF (corresponding author), Sun Yat Sen Univ, Ctr Canc, Dept Pathol, Guangzhou, Peoples R China.

EM fengyf@sysucc.org.cn; leiyiyan@mail.sysu.edu.cn

OI Wei, Liu/0000-0002-5305-194X

FU Natural Science Foundation of Guangdong Province [2019A1515011329]

FX Funding This work was supported by Grants from the Natural Science

Foundation of Guangdong Province (No. 2019A1515011329).

CR Abnet CC, 2018, GASTROENTEROLOGY, V154, P360, DOI 10.1053/j.gastro.2017.08.023

Arnold M, 2015, GUT, V64, P381, DOI 10.1136/gutjnl-2014-308124

Bang YJ, 2010, LANCET, V376, P687, DOI 10.1016/S0140-6736(10)61121-X

Bray F, 2018, CA-CANCER J CLIN, V68, P394, DOI 10.3322/caac.21492

Chen DS, 2017, NATURE, V541, P321, DOI 10.1038/nature21349

Chen SJ, 2019, CURR PHARM DESIGN, V25, P1105, DOI 10.2174/1381612825666190506122228

Cui YP, 2020, CELL RES, V30, P902, DOI 10.1038/s41422-020-0333-6

Feng YF, 2018, CELL DEATH DIS, V9, DOI 10.1038/s41419-018-0979-x

Feng YF, 2020, BMC CANCER, V20, DOI 10.1186/s12885-020-07068-w

Ferrero S, 2020, HAEMATOLOGICA, V105, P1604, DOI 10.3324/haematol.2018.214056

Guo ZX, 2021, FRONT ONCOL, V11, DOI 10.3389/fonc.2021.603660

Hanzelmann S, 2013, BMC BIOINFORMATICS, V14, DOI 10.1186/1471-2105-14-7

Hartigan J. A., 1979, Applied Statistics, V28, P100, DOI 10.2307/2346830

Hazra A, 2016, INDIAN J DERMATOL, V61, P251, DOI 10.4103/0019-5154.182416

Kitano Y, 2018, J PATHOL, V244, P346, DOI 10.1002/path.5021

Li QF, 2019, ONCOL REP, V42, P1815, DOI 10.3892/or.2019.7309

Masliah-Planchon J, 2016, ONCOTARGET, V7, P38892, DOI 10.18632/oncotarget.6476

Mayakonda A, 2018, GENOME RES, V28, P1747, DOI 10.1101/gr.239244.118

Moehler M, 2020, ANN ONCOL, V31, P228, DOI 10.1016/j.annonc.2019.10.018

Moore AR, 2020, NAT REV DRUG DISCOV, V19, P533, DOI 10.1038/s41573-020-0068-6

Prior IA, 2020, CANCER RES, V80, P2969, DOI 10.1158/0008-5472.CAN-19-3682

Pylayeva-Gupta Y, 2011, NAT REV CANCER, V11, P761, DOI 10.1038/nrc3106

Ryan MB, 2018, NAT REV CLIN ONCOL, V15, P709, DOI 10.1038/s41571-018-0105-0

Simanshu DK, 2017, CELL, V170, P17, DOI 10.1016/j.cell.2017.06.009

Theiss L, 2019, SURG CLIN N AM, V99, P543, DOI 10.1016/j.suc.2019.02.012

Wilkerson MD, 2010, BIOINFORMATICS, V26, P1572, DOI 10.1093/bioinformatics/btq170

Yang YM, 2020, SIGNAL TRANSDUCT TAR, V5, DOI 10.1038/s41392-020-00323-3

Yoshihara K, 2013, NAT COMMUN, V4, DOI 10.1038/ncomms3612

Zhang ZT, 2018, CLIN EXP PHARMACOL P, V45, P720, DOI 10.1111/1440-1681.12926

Zheng BQ, 2021, FRONT MOL BIOSCI, V8, DOI 10.3389/fmolb.2021.688692

NR 30

TC 0

Z9 0

U1 1

U2 1

PU FRONTIERS MEDIA SA

PI LAUSANNE

PA AVENUE DU TRIBUNAL FEDERAL 34, LAUSANNE, CH-1015, SWITZERLAND

EI 1664-8021

J9 FRONT GENET

JI Front. Genet.

PD FEB 24

PY 2022

VL 13

AR 822966

DI 10.3389/fgene.2022.822966

PG 10

WC Genetics & Heredity

WE Science Citation Index Expanded (SCI-EXPANDED)

SC Genetics & Heredity

GA ZQ1SY

UT WOS:000766893300001

PM 35281814

OA Green Published, gold

DA 2022-05-19

ER

PT J

AU Hu, ZH

Sun, S

Zhao, XM

Yu, H

Wu, XH

Wang, JL

Chang, JH

Wang, HJ

AF Hu, Zhihuang

Sun, Si

Zhao, Xinmin

Yu, Hui

Wu, Xianghua

Wang, Jialei

Chang, Jianhua

Wang, Huijie

TI Rh-Endostatin Plus Irinotecan/Cisplatin as Second-Line Therapy for

Advanced Esophageal Squamous Cell Carcinoma: An Open-Label, Phase II

Study

SO ONCOLOGIST

LA English

DT Article

DE recombinant human endostatin (Rh-endostatin); irinotecan; cisplatin;

esophageal squamous cell carcinoma; phase II study

ID ANGIOGENESIS; EFFICACY

AB Accumulating evidence has shown that combination therapies with anti-angiogenics are a good strategy for advanced esophageal squamous cell carcinoma (ESCC). This prospective, open-label, phase II study provides the first analysis of the anti-tumor activity and safety for Rh-endostatin plus irinotecan/cisplatin (a commonly used regimen in ESCC) in patients with advanced ESCC.

Background This open-label, phase II study aimed to investigate the efficacy and safety of recombinant human endostatin (Rh-endostatin) plus irinotecan/cisplatin as second-line treatment in patients with advanced esophageal squamous cell carcinoma (ESCC). Methods Eligible patients received 15mg/m(2) Rh-endostatin as a continuous intravenous pump infusion (7 continuous days), 60mg/m(2) irinotecan (days 1 and 8), and 60mg/m(2) cisplatin (day 1) every 3 weeks. The primary endpoint was progression-free survival (PFS). Results A total of 50 patients were assessable for efficacy and safety analysis. The median follow-up was 10.97 months (95%CI: 7.03-19.42) as the data cutoff. Median PFS was 4.01 months (95% CI: 3.19-5.49), and median overall survival (OS) was 12.32 months (95% CI: 8.21-17.45); 13 (26%; 95% CI: 15.87-39.55) of 50 patients had an objective response, and 31 (62%; 95% CI: 48.15-74.14) had disease control. Grade 3 or greater treatment-related adverse events (AEs) occurred in 12 (24.0%) patients, and no deaths were reported. The common grade 3 or greater AEs were leucopenia (18.0%) and neutropenia (16.0%). Five (10%) patients discontinued treatment because of AEs. Conclusion Rh-endostatin plus irinotecan/cisplatin showed promising anti-tumor activity in advanced ESCC patients with a good safety profile in the second-line setting, which warrants further study in this population. (ClinicalTrials.gov identifier: NCT03797625).

C1 [Hu, Zhihuang; Sun, Si; Zhao, Xinmin; Yu, Hui; Wu, Xianghua; Wang, Jialei; Chang, Jianhua; Wang, Huijie] Fudan Univ, Shanghai Canc Ctr, Dept Med Oncol, Shanghai, Peoples R China.

[Hu, Zhihuang; Sun, Si; Zhao, Xinmin; Yu, Hui; Wu, Xianghua; Wang, Jialei; Wang, Huijie] Fudan Univ, Shanghai Med Coll, Dept Oncol, Shanghai, Peoples R China.

[Hu, Zhihuang; Sun, Si; Zhao, Xinmin; Yu, Hui; Wu, Xianghua; Wang, Jialei; Wang, Huijie] Fudan Univ, Inst Thorac Oncol, Shanghai, Peoples R China.

[Chang, Jianhua] Chinese Acad Med Sci, Shenzhen Ctr, Canc Hosp, Dept Med Oncol, Shenzhen, Peoples R China.

RP Chang, JH; Wang, HJ (corresponding author), Fudan Univ, Shanghai Canc Ctr, Dept Thorac Med Oncol, 270 Dong An Rd, Shanghai 200032, Peoples R China.

EM changjianhua@163.com; wanghi98@hotmail.com

CR Abraham P, 2020, ADV THER, V37, P3392, DOI 10.1007/s12325-020-01394-y

Chu L, 2021, ONCOLOGIST, V26, pE925, DOI 10.1002/onco.13668

Han BH, 2011, J THORAC ONCOL, V6, P1104, DOI 10.1097/JTO.0b013e3182166b6b

Huang WW, 2016, ONCOTARGET, V7, P31501, DOI 10.18632/oncotarget.8967

Kim M, 2017, CANCER RES TREAT, V49, P416, DOI 10.4143/crt.2016.121

Li JQ, 2017, ONCOTARGETS THER, V10, P3965, DOI 10.2147/OTT.S132756

Li N, 2012, J CANCER RES CLIN, V138, P1131, DOI 10.1007/s00432-012-1189-z

Li XY, 2013, MED ONCOL, V30, DOI 10.1007/s12032-013-0746-x

Lin H, 2017, ONCOTARGET, V8, P1141, DOI 10.18632/oncotarget.13588

Matsubara Y., 2021, J CLIN ONCOL, V39, pe16045

Noma K, 2008, GASTROENTEROLOGY, V134, P1981, DOI 10.1053/j.gastro.2008.02.061

Oshima Y, 2010, ANN THORAC CARDIOVAS, V16, P389

Rajabi M, 2017, BIOMEDICINES, V5, DOI 10.3390/biomedicines5020034

Russo M, 2018, THROMB RES, V164, pS3, DOI 10.1016/j.thromres.2018.01.030

Tian J, 2015, CANCER CHEMOTH PHARM, V76, P829, DOI 10.1007/s00280-015-2854-0

Wang ZQ, 2021, INVEST NEW DRUG, V39, P516, DOI 10.1007/s10637-020-01021-1

Yi M, 2019, MOL CANCER, V18, DOI 10.1186/s12943-019-0974-6

NR 17

TC 0

Z9 0

U1 0

U2 0

PU OXFORD UNIV PRESS

PI OXFORD

PA GREAT CLARENDON ST, OXFORD OX2 6DP, ENGLAND

SN 1083-7159

EI 1549-490X

J9 ONCOLOGIST

JI Oncologist

PD APR 5

PY 2022

VL 27

IS 4

BP 253

EP +

DI 10.1093/oncolo/oyab078

PG 10

WC Oncology

WE Science Citation Index Expanded (SCI-EXPANDED)

SC Oncology

GA 0G8RC

UT WOS:000778306200003

PM 35380726

OA Green Published

DA 2022-05-19

ER

PT J

AU Yu, R

Wang, WQ

Li, T

Li, JC

Zhao, KL

Wang, WH

Liang, L

Wu, HS

Ai, TS

Huang, W

Li, LY

Yu, WT

Wei, CL

Wang, YD

Shen, W

Xiao, ZF

AF Yu, Rong

Wang, Wenqing

Li, Tao

Li, Jiancheng

Zhao, Kuaile

Wang, Weihu

Liang, Long

Wu, Haishan

Ai, Tashan

Huang, Wei

Li, Liyun

Yu, Wentao

Wei, Chenlu

Wang, Yidi

Shen, Wei

Xiao, Zefen

TI RATIONALE 311: tislelizumab plus concurrent chemoradiotherapy for

localized esophageal squamous cell carcinoma

SO FUTURE ONCOLOGY

LA English

DT Article

DE concurrent chemoradiotherapy; esophageal squamous cell carcinoma; immune

checkpoint inhibitor; immunotherapy; Phase III clinical trial;

programmed cell death-1 (PD-1); tislelizumab

ID CANCER; CHEMORADIATION; RADIOTHERAPY; CISPLATIN; JUNCTION; THERAPY

AB Definitive chemoradiotherapy is the standard of care for inoperable locoregionally advanced esophageal squamous cell carcinoma (ESCC). Immune checkpoint inhibitors such as anti-PD-1/PD-L1 antibodies have led to a paradigm shift in advanced, metastatic ESCC treatment; however, the effect of incorporating checkpoint inhibitors in the definitive management of ESCC is unclear. Tislelizumab is an anti-PD-1 antibody specifically engineered to minimize Fc gamma R binding on macrophages to abrogate antibody-dependent phagocytosis, a mechanism of T-cell clearance and potential resistance to anti-PD-1 therapy. The RATIONALE 311 study described here (BGB-A317-311; NCT03957590) is a registrational multicenter, double-blind, placebo-controlled, randomized, Phase III clinical trial designed to evaluate the efficacy and safety of tislelizumab combined with concurrent chemoradiotherapy in patients with inoperable localized ESCC.

Lay abstract: Esophageal cancer is a challenging disease that seriously threatens patients' health and life. Esophageal squamous cell carcinoma (ESCC) is the most common type of esophageal cancer. Most patients who have inoperable stage II-IV ESCC are currently treated with a sequential combination of chemotherapy and radiation therapy, with the hopes of increasing the positive effects seen from either therapy alone. Immune checkpoint inhibitors such as anti-PD-1/PD-L1 antibodies have shown encouraging results in patients with ESCC, but it is not known if combining checkpoint inhibitors with simultaneous chemotherapy and radiation therapy will provide additional benefits. The safety and efficacy of tislelizumab, an anti-PD1 antibody specifically engineered to limit potential resistance to anti-PD-1 therapy, is being investigated in combination with simultaneous chemotherapy and radiation therapy in patients with inoperable stage II-IV ESCC in an actively enrolling clinical trial, RATIONALE 311 (NCT03957590). Our trial in progress article explains the reason RATIONALE 311 was started and provides important enrollment information for doctors.

C1 [Yu, Rong; Wang, Weihu] Peking Univ Canc Hosp, Dept Radiat Oncol, Beijing, Peoples R China.

[Wang, Wenqing; Xiao, Zefen] Chinese Acad Med Sci, Canc Hosp & Inst, Beijing, Peoples R China.

[Li, Tao; Liang, Long] Sichuan Canc Hosp, Dept Radiat Oncol, Chengdu, Peoples R China.

[Li, Jiancheng; Wu, Haishan] Fujian Canc Hosp, Dept Radiat Oncol, Fuzhou, Peoples R China.

[Zhao, Kuaile; Ai, Tashan] Fudan Univ, Shanghai Canc Ctr, Dept Radiat Oncol, Shanghai, Peoples R China.

[Huang, Wei; Li, Liyun; Yu, Wentao; Wei, Chenlu; Wang, Yidi; Shen, Wei] BeiGene Beijing Co Ltd, Beijing, Peoples R China.

RP Xiao, ZF (corresponding author), Chinese Acad Med Sci, Canc Hosp & Inst, Beijing, Peoples R China.

EM xiaozefen@sina.com

FU BeiGene, Ltd.

FX BeiGene, Ltd. provided financial support for this manuscript, including

writing and editorial assistance by S Lindsey and E Hermans, (Peloton

Advantage, LLC, an OPEN Health company, NJ, USA).

CR Abbas G, 2017, ANN CARDIOTHORAC SUR, V6, P131, DOI 10.21037/acs.2017.03.03

Ai D, 2020, INT J RADIAT ONCOL, V108, pS45

[Anonymous], 2019, CHINESE J CANCER RES, V31, P223, DOI 10.21147/j.issn.1000-9604.2019.02.01

Antonia SJ, 2017, NEW ENGL J MED, V377, P1919, DOI 10.1056/NEJMoa1709937

Arnold M, 2015, GUT, V64, P381, DOI 10.1136/gutjnl-2014-308124

Barbetta A, 2018, J THORAC CARDIOV SUR, V155, P2710, DOI 10.1016/j.jtcvs.2018.01.086

Chau I, 2021, J CLIN ONCOL, V39, DOI 10.1200/JCO.2021.39.15_suppl.LBA4001

Conroy T, 2014, LANCET ONCOL, V15, P305, DOI 10.1016/S1470-2045(14)70028-2

Cooper JS, 1999, JAMA-J AM MED ASSOC, V281, P1623, DOI 10.1001/jama.281.17.1623

Crosby T, 2013, LANCET ONCOL, V14, P627, DOI 10.1016/S1470-2045(13)70136-0

Dahan R, 2015, CANCER CELL, V28, P285, DOI 10.1016/j.ccell.2015.08.004

Desai J, 2020, J IMMUNOTHER CANCER, V8, DOI 10.1136/jitc-2019-000453

Gill S, 2011, CURR ONCOL, V18, pS5

Hong Y, 2021, FEBS OPEN BIO, V11, P782, DOI 10.1002/2211-5463.13102

International, 2020, OES FACT SHEET

Kato K, 2020, ANN ONCOL, V31, pS1192, DOI 10.1016/j.annonc.2020.08.2298

Kato K, 2019, LANCET ONCOL, V20, P1506, DOI 10.1016/S1470-2045(19)30626-6

Kelly RJ, 2020, ANN ONCOL, V31, pS1193, DOI 10.1016/j.annonc.2020.08.2299

Kelly RJ, 2019, J CLIN ONCOL, V37, DOI 10.1200/JCO.2019.37.4_suppl.142

Kojima T, 2020, J CLIN ONCOL, V38, DOI 10.1200/JCO.20.01888

Lee AN, 2020, DRUGS, V80, P617, DOI 10.1007/s40265-020-01286-z

Liao XY, 2019, ONCOL LETT, V18, P5011, DOI 10.3892/ol.2019.10893

Liu SY, 2020, EXPERT OPIN INV DRUG, V29, P1355, DOI 10.1080/13543784.2020.1833857

Oh P, 2016, INT J RADIAT ONCOL, V96, pS127, DOI 10.1016/j.ijrobp.2016.06.311

Qiu MJ, 2020, AGING-US, V12, P14736, DOI 10.18632/aging.103532

Rice TW, 2017, J THORAC ONCOL, V12, P36, DOI 10.1016/j.jtho.2016.10.016

Sasaki Y, 2016, JPN J CLIN ONCOL, V46, P805, DOI 10.1093/jjco/hyw082

Shen L, 2021, J CLIN ONCOL, V39, DOI 10.1200/JCO.2021.39.15_suppl.4012

Shen L, 2020, J IMMUNOTHER CANCER, V8, DOI 10.1136/jitc-2019-000437

Stahl M, 2017, J THORAC DIS, V9, pS792, DOI 10.21037/jtd.2017.05.05

Urba SG, 2003, CANCER, V98, P2177, DOI 10.1002/cncr.11759

Xu JM, 2020, CLIN CANCER RES, V26, P4542, DOI 10.1158/1078-0432.CCR-19-3561

Yamamoto S, 2020, FUTURE ONCOL, V16, P1351, DOI 10.2217/fon-2020-0189

Zhang T, 2018, CANCER IMMUNOL IMMUN, V67, P1079, DOI 10.1007/s00262-018-2160-x

NR 34

TC 1

Z9 1

U1 6

U2 6

PU FUTURE MEDICINE LTD

PI LONDON

PA UNITEC HOUSE, 3RD FLOOR, 2 ALBERT PLACE, FINCHLEY CENTRAL, LONDON, N3

1QB, ENGLAND

SN 1479-6694

EI 1744-8301

J9 FUTURE ONCOL

JI Future Oncol.

PD JUL

PY 2021

VL 17

IS 31

BP 4081

EP 4089

DI 10.2217/fon-2021-0632

EA JUL 2021

PG 9

WC Oncology

WE Science Citation Index Expanded (SCI-EXPANDED)

SC Oncology

GA WK0WL

UT WOS:000674048800001

PM 34269067

OA hybrid

DA 2022-05-19

ER

PT J

AU Takashi, S

Satoshi, Y

Akihiko, O

Naoya, Y

Yusuke, T

Kentaro, M

Yu, O

Yasuaki, N

Koichi, Y

Takashi, F

Ryo, O

Isamu, H

Chikara, K

Kosuke, N

Yasuhiro, T

Kazuhiko, Y

Hideaki, S

AF Takashi, Suzuki

Satoshi, Yajima

Akihiko, Okamura

Naoya, Yoshida

Yusuke, Taniyama

Kentaro, Murakami

Yu, Ohkura

Yasuaki, Nakajima

Koichi, Yagi

Takashi, Fukuda

Ryo, Ogawa

Isamu, Hoshino

Chikara, Kunisaki

Kosuke, Narumiya

Yasuhiro, Tsubosa

Kazuhiko, Yamada

Hideaki, Shimada

TI Clinical impact of preoperative serum p53 antibody titers in 1487

patients with surgically treated esophageal squamous cell carcinoma: a

multi-institutional study

SO ESOPHAGUS

LA English

DT Article

DE Serum p53 antibodies; Antibody titers; Esophageal squamous cell

carcinoma; Prognosis

ID CHEMOTHERAPY; RECURRENCE; SURGERY; CANCER

AB Background Although the clinicopathological significance of serum p53 antibodies (s-p53-Abs) in esophageal cancer have been evaluated previously, previous reports only analyzed around 100-200 patients. This study was a multi-institutional study promoted by the Japan Esophageal Society to evaluate the clinical significance of preoperative s-p53-Ab status and antibody titers in 1487 esophageal cancer patients without neoadjuvant therapy. Methods A total of 1487 patients with esophageal squamous cell carcinoma surgically treated between 2008 and 2016 in 15 hospitals in Japan were enrolled. The cut-off value to classify the patients into s-p53-Ab positive and negative groups was 1.30 U/ml. A receiver operating characteristic curve was constructed to assess the s-p53-Abs cut-off levels to differentiate poor prognosis among the s-p53-Ab positive group. Univariate and multivariate analyses were used to evaluate the clinicopathological and prognostic significance of s-p53-Ab status and titers. Results Although s-p53-Ab status was significantly associated with tumor depth (P = 0.002), nodal status (P = 0.027), and pathological stage (P = 0.002). The s-p53-Ab positive status was not significantly associated with poor overall survival (P = 0.699). Using 9.82 U/ml as a cut-off, the high s-p53-Ab titer group showed a significantly worse overall survival than the low s-p53-Ab titer group (P = 0.038). However, the difference was not significant in the multivariate analysis. Conclusion The presence of s-p53-Abs was associated with tumor progression. Although high s-p53-Ab titers more than 9.82 U/ml, might be associated with poor prognosis for patients with esophageal squamous cell carcinoma, it was not an independent risk factor.

C1 [Takashi, Suzuki; Satoshi, Yajima; Akihiko, Okamura; Naoya, Yoshida; Yusuke, Taniyama; Kentaro, Murakami; Yu, Ohkura; Yasuaki, Nakajima; Koichi, Yagi; Takashi, Fukuda; Ryo, Ogawa; Isamu, Hoshino; Chikara, Kunisaki; Kosuke, Narumiya; Yasuhiro, Tsubosa; Kazuhiko, Yamada; Hideaki, Shimada] Japan Esophageal Soc Promot Res, P53 Antibody Multictr Res Grp, Tokyo, Japan.

RP Hideaki, S (corresponding author), Japan Esophageal Soc Promot Res, P53 Antibody Multictr Res Grp, Tokyo, Japan.

EM hideaki.shimada@med.toho-u.ac.jp

FU JSPS KAKENHI [JP16K10520]

FX This work was supported by the JSPS KAKENHI Grant number JP16K10520.

CR Brierley MKGCW, 2017, UICC TNM CLASSIFICAT

Fisher OM, 2017, GUT, V66, P399, DOI 10.1136/gutjnl-2015-310888

Kanda Y, 2013, BONE MARROW TRANSPL, V48, P452, DOI 10.1038/bmt.2012.244

Kandioler D, 2014, J THORAC CARDIOV SUR, V148, P2280, DOI 10.1016/j.jtcvs.2014.06.079

Li XC, 2018, ANN ONCOL, V29, P938, DOI 10.1093/annonc/mdy011

Makino T, 2010, ANN SURG ONCOL, V17, P804, DOI 10.1245/s10434-009-0786-9

Oshima Y, 2020, SURG TODAY, V50, P1402, DOI 10.1007/s00595-020-02030-6

Shimada H, 2000, CANCER-AM CANCER SOC, V89, P1677, DOI 10.1002/1097-0142(20001015)89:8<1677::AID-CNCR5>3.0.CO;2-9

Shimada H, 2003, SURGERY, V133, P24, DOI 10.1067/msy.2003.31

Shimada H, 2002, SURGERY, V132, P41, DOI 10.1067/msy.2002.125307

Shimada H, 2018, ANN GASTROENT SURG, V2, P266, DOI 10.1002/ags3.12179

Suzuki T, 2018, ESOPHAGUS-TOKYO, V15, P294, DOI 10.1007/s10388-018-0629-5

Suzuki T, 2016, CLIN J GASTROENTEROL, V9, P55, DOI 10.1007/s12328-016-0633-z

Takahashi K, 2007, DIS ESOPHAGUS, V20, P117, DOI 10.1111/j.1442-2050.2007.00656.x

Ushigome M, 2020, INT J CLIN ONCOL, V25, P622, DOI 10.1007/s10147-019-01597-6

Yamasaki M, 2010, ANN SURG ONCOL, V17, P634, DOI 10.1245/s10434-009-0851-4

Yamashita K, 2017, WORLD J SURG, V41, P1566, DOI 10.1007/s00268-017-3894-x

NR 17

TC 7

Z9 8

U1 0

U2 0

PU SPRINGER JAPAN KK

PI TOKYO

PA SHIROYAMA TRUST TOWER 5F, 4-3-1 TORANOMON, MINATO-KU, TOKYO, 105-6005,

JAPAN

SN 1612-9059

EI 1612-9067

J9 ESOPHAGUS-TOKYO

JI Esophagus

PD JAN

PY 2021

VL 18

IS 1

BP 65

EP 71

DI 10.1007/s10388-020-00761-6

EA JUL 2020

PG 7

WC Gastroenterology & Hepatology

WE Science Citation Index Expanded (SCI-EXPANDED)

SC Gastroenterology & Hepatology

GA PR5SE

UT WOS:000552599300001

PM 32715348

DA 2022-05-19

ER

PT J

AU Chen, FJ

Luo, H

Xing, LG

Liang, N

Xie, J

Zhang, JD

AF Chen, Fangjie

Luo, Hui

Xing, Ligang

Liang, Ning

Xie, Jian

Zhang, Jiandong

TI Feasibility and efficiency of concurrent chemoradiotherapy with

capecitabine and cisplatin versus radiotherapy alone for elderly

patients with locally advanced esophageal squamous cell carcinoma:

Experience of two centers

SO THORACIC CANCER

LA English

DT Article

DE Concurrent chemoradiotherapy; elderly patient; esophageal squamous cell

carcinoma; radiotherapy

ID TERM-FOLLOW-UP; DEFINITIVE CHEMORADIOTHERAPY; PHASE-II; INDUCTION

CHEMOTHERAPY; RADIATION-THERAPY; SOLID TUMORS; CANCER; DOCETAXEL;

CHEMORADIATION; 5-FLUOROURACIL

AB Background: The purpose of this retrospective study was to evaluate the feasibility and efficacy of definitive concurrent chemoradiotherapy (CCRT) with capecitabine and cisplatin for elderly patients with locally advanced esophageal squamous cell carcinoma.

Methods: A total of 90 patients were included from two different centers. Forty-nine patients were treated with CCRT consisting of capecitabine (850 mg/m(2), oral, twice a day for 1-14 days) and cisplatin (20 mg/m(2)) weekly during radiotherapy (RT). The remaining 41 patients were treated with RT alone. The overall response, overall survival, progression-free survival, and toxicity rates were recorded.

Results: Compared to the RT group (51.2%; P = 0.029), the overall response rate in the CCRT group (73.5%) was obviously higher. A complete response was achieved in 34.7% and 14.6% of patients in the CCRT and RT groups, respectively (P = 0.030). Median progression-free and median overall survival rates were significantly higher in the CCRT group (24.7 and 30.6 months; P < 0.05) compared to the RT group (16.6 and 18.7 months). Acute toxic effects were more severe in the CCRT group, but no significant difference in grade 3 or 4 acute toxicities was observed between the groups.

Conclusion: Both CCRT with capecitabine and cisplatin and RT alone are feasible to treat elderly patients and yield a good performance status with locally advanced esophageal squamous cell carcinoma. CCRT improved the tumor response without increasing the side effects compared to RT alone. CCRT is recommended for patients over 65 with good performance status.

C1 [Chen, Fangjie] Weifang Med Coll, Dept Oncol, Weifang, Peoples R China.

[Luo, Hui] Henan Canc Hosp, Dept Radiat Oncol, Zhengzhou, Henan, Peoples R China.

[Xing, Ligang] Shandong Canc Hosp & Inst, Dept Radiat Oncol, Jinan, Shandong, Peoples R China.

[Liang, Ning; Xie, Jian; Zhang, Jiandong] Shandong Univ, Qianfoshan Hosp, Dept Radiat Oncol, 16766 Jingshi Rd, Jinan 250014, Shandong, Peoples R China.

RP Zhang, JD (corresponding author), Shandong Univ, Qianfoshan Hosp, Dept Radiat Oncol, 16766 Jingshi Rd, Jinan 250014, Shandong, Peoples R China.

EM zhangjiandongfl001@163.com

FU National Natural Science Foundation of China [81672974]

FX This work was supported by a grant from the National Natural Science

Foundation of China [No. 81672974].

CR Ahn D, 2013, MOL CLIN ONCOL, V1, P685, DOI 10.3892/mco.2013.113

AlSarraf M, 1997, J CLIN ONCOL, V15, P277, DOI 10.1200/JCO.1997.15.1.277

Bedenne L, 2007, J CLIN ONCOL, V25, P1160, DOI 10.1200/JCO.2005.04.7118

Cooper JS, 1999, JAMA-J AM MED ASSOC, V281, P1623, DOI 10.1001/jama.281.17.1623

Daly JM, 2000, J AM COLL SURGEONS, V190, P562, DOI 10.1016/S1072-7515(00)00238-6

Day FL, 2011, BRIT J CANCER, V104, P265, DOI 10.1038/sj.bjc.6606051

Font A, 2007, CLIN TRANSL ONCOL, V9, P177, DOI 10.1007/s12094-007-0032-5

Gupta S, 2013, DRUG DISCOV THER, V7, P36, DOI 10.5582/ddt.2013.v7.1.36

Higuchi K, 2008, RADIOTHER ONCOL, V87, P398, DOI 10.1016/j.radonc.2008.03.006

Ishikawa T, 1998, BIOCHEM PHARMACOL, V55, P1091, DOI 10.1016/S0006-2952(97)00682-5

Koo DH, 2012, CANCER CHEMOTH PHARM, V69, P655, DOI 10.1007/s00280-011-1750-5

Lee J, 2008, CANCER CHEMOTH PHARM, V62, P77, DOI 10.1007/s00280-007-0577-6

Lee SJ, 2009, J KOREAN MED SCI, V24, P120, DOI 10.3346/jkms.2009.24.1.120

Li BS, 2009, RADIOTHER ONCOL, V93, P458, DOI 10.1016/j.radonc.2009.08.002

Li QQ, 2010, DIS ESOPHAGUS, V23, P253, DOI 10.1111/j.1442-2050.2009.01003.x

Ruhstaller T, 2009, ANN ONCOL, V20, P1522, DOI 10.1093/annonc/mdp045

Schuller J, 2000, CANCER CHEMOTH PHARM, V45, P291, DOI 10.1007/s002800050043

Siegel R, 2013, CA-CANCER J CLIN, V63, P11, DOI 10.3322/caac.21166

Stahl M, 2005, J CLIN ONCOL, V23, P2310, DOI 10.1200/JCO.2005.00.034

Takeuchi S, 2007, AM J CLIN ONCOL-CANC, V30, P607, DOI 10.1097/COC.0b013e3180ca7c84

Therasse P, 2000, J NATL CANCER I, V92, P205, DOI 10.1093/jnci/92.3.205

Tougeron D, 2008, BRIT J CANCER, V99, P1586, DOI 10.1038/sj.bjc.6604749

Vaishampayan UN, 2002, INT J RADIAT ONCOL, V53, P675, DOI 10.1016/S0360-3016(02)02772-4

Wahl RL, 2009, J NUCL MED, V50, p122S, DOI 10.2967/jnumed.108.057307

Xing LG, 2014, J CANCER RES CLIN, V140, P867, DOI 10.1007/s00432-014-1615-5

Yamashita H, 2006, DIS ESOPHAGUS, V19, P15, DOI 10.1111/j.1442-2050.2006.00531.x

Yanasoot A, 2017, ASIAN CARDIOVASC THO

NR 27

TC 11

Z9 13

U1 1

U2 5

PU WILEY

PI HOBOKEN

PA 111 RIVER ST, HOBOKEN 07030-5774, NJ USA

SN 1759-7706

EI 1759-7714

J9 THORAC CANCER

JI Thorac. Cancer

PD JAN

PY 2018

VL 9

IS 1

BP 59

EP 65

DI 10.1111/1759-7714.12536

PG 7

WC Oncology; Respiratory System

WE Science Citation Index Expanded (SCI-EXPANDED)

SC Oncology; Respiratory System

GA FS0OU

UT WOS:000419474400009

PM 29024498

OA gold, Green Published

DA 2022-05-19

ER

PT J

AU Deng, YX

Bian, C

Tao, H

Zhang, HJ

AF Deng, Yuxia

Bian, Chao

Tao, Hua

Zhang, Haijun

TI Improved survival with higher radiation dose for esophageal squamous

cell carcinoma patients treated with definitive chemoradiotherapy

SO ONCOTARGET

LA English

DT Article

DE esophageal squamous cell carcinoma; radiotherapy; overall survival;

progression-free survival

ID ELECTIVE NODAL IRRADIATION; INVOLVED-FIELD RADIOTHERAPY; CONCURRENT

CHEMOTHERAPY; PREOPERATIVE CHEMORADIOTHERAPY; CANCER; PATTERNS; THERAPY;

CHEMORADIATION; SURGERY; TRIAL

AB Purpose: The optimal radiation dose for patients with esophageal squamous cell carcinoma (ESCC) has long been debated. We undertook the retrospective study to evaluate the survival impact of high dose vs standard dose in patients with stage II-III esophageal cancer treated with definitive chemoradiotherapy (CRT).

Results: A total of 137 patients were included in our study, 63 patients classified as standard-dose group and 74 as high-dose group. For the 63 patients in the standard-dose group, the median PFS and the 1-, 2-, and 3-year PFS rates were 12.6 months, 58.0%, 26.0% and 12.0%, respectively; for the 74 patients in the high-dose group, they were 20.0 months, 80.1%, 31.0% and 20.0%, respectively (P = 0.013). The median OS of the patients in the standard-dose group and high-dose group groups were 19.0 months and 26.6 months, respectively, and the 1-, 2- and 3-year survival rates were 78.0%, 39.0%, and 24.0%, and 89.0%, 61.0%, and 30.0%, respectively (P = 0.037). Besides the rate of grade 3 acute irradiation esophagitis in the high dose group (10.5% versus. 2.2%, P < 0.01), there were no significantly differ of treatment-related toxicities between the two groups.

Materials and Methods: According to the radiation dose, patients from 2010 to 2014 were allocated into either the standard-dose group (50-50.4 Gy) or the high dose group (>= 59.4 Gy). Overall survival (OS), progression-free survival (PFS) and treatment-related toxicities were assessed and compared between the two groups.

Conclusions: Our findings suggest that higher radiation dose could perform better outcomes for esophageal squamous cell carcinoma patients.

C1 [Deng, Yuxia; Zhang, Haijun] Southeast Univ, Dept Oncol, Zhongda Hosp, Med Sch, Nanjing, Jiangsu, Peoples R China.

[Bian, Chao] Ningxia Med Univ, Ningxia, Peoples R China.

[Tao, Hua] Jiangsu Canc Inst & Hosp, Dept Radiat Oncol, Nanjing, Jiangsu, Peoples R China.

RP Zhang, HJ (corresponding author), Southeast Univ, Dept Oncol, Zhongda Hosp, Med Sch, Nanjing, Jiangsu, Peoples R China.

EM zhanghaijunseu@163.com

CR Bedenne L, 2007, J CLIN ONCOL, V25, P1160, DOI 10.1200/JCO.2005.04.7118

Button MR, 2009, INT J RADIAT ONCOL, V73, P818, DOI 10.1016/j.ijrobp.2008.04.062

Cooper JS, 1999, JAMA-J AM MED ASSOC, V281, P1623, DOI 10.1001/jama.281.17.1623

Eisenhauer EA, 2009, EUR J CANCER, V45, P228, DOI 10.1016/j.ejca.2008.10.026

Ferlay J, 2010, INT J CANCER, V127, P2893, DOI 10.1002/ijc.25516

Gaspar LE, 2000, CANCER, V88, P988, DOI 10.1002/(SICI)1097-0142(20000301)88:5<988::AID-CNCR7>3.0.CO;2-U

He LR, 2014, J THORAC ONCOL, V9, P1398, DOI 10.1097/JTO.0000000000000267

HERSKOVIC A, 1992, NEW ENGL J MED, V326, P1593, DOI 10.1056/NEJM199206113262403

Hurmuzlu M, 2010, DIS ESOPHAGUS, V23, P244, DOI 10.1111/j.1442-2050.2009.00999.x

Kato K, 2013, JPN J CLIN ONCOL, V43, P608, DOI 10.1093/jjco/hyt048

Kawaguchi Y, 2011, JPN J CLIN ONCOL, V41, P1007, DOI 10.1093/jjco/hyr069

Kim HJ, 2016, CANC RES TREAT

Minsky BD, 2002, J CLIN ONCOL, V20, P1167, DOI 10.1200/JCO.20.5.1167

Pottgen C, 2012, CANCER TREAT REV, V38, P599, DOI 10.1016/j.ctrv.2011.10.005

Rawat Sheh, 2013, J Gastrointest Cancer, V44, P277, DOI 10.1007/s12029-013-9477-7

Rohatgi PR, 2005, CANCER-AM CANCER SOC, V104, P1349, DOI 10.1002/cncr.21346

Sarwar CMS, 2010, INT J SURG, V8, P417, DOI 10.1016/j.ijsu.2010.06.011

Suh YG, 2014, JPN J CLIN ONCOL, V44, P534, DOI 10.1093/jjco/hyu047

Torre LA, 2015, CA-CANCER J CLIN, V65, P87, DOI 10.3322/caac.21262

Trotti A, 2003, SEMIN RADIAT ONCOL, V13, P176, DOI 10.1016/S1053-4296(03)00031-6

van Hagen P, 2012, NEW ENGL J MED, V366, P2074, DOI 10.1056/NEJMoa1112088

Wang SL, 2006, J THORAC ONCOL, V1, P252, DOI 10.1016/S1556-0864(15)31576-8

Wheeler JB, 2012, SURG CLIN N AM, V92, P1077, DOI 10.1016/j.suc.2012.07.008

Yamashita H, 2015, RADIAT ONCOL, V10, DOI 10.1186/s13014-015-0482-9

Zhang XL, 2014, RADIAT ONCOL, V9, DOI 10.1186/1748-717X-9-64

Zhang Z, 2005, INT J RADIAT ONCOL, V61, P656, DOI 10.1016/j.ijrobp.2004.06.022

Zhao KL, 2010, INT J RADIAT ONCOL, V76, P446, DOI 10.1016/j.ijrobp.2009.02.078

NR 27

TC 9

Z9 9

U1 0

U2 5

PU IMPACT JOURNALS LLC

PI ORCHARD PARK

PA 6666 E QUAKER ST, STE 1, ORCHARD PARK, NY 14127 USA

EI 1949-2553

J9 ONCOTARGET

JI Oncotarget

PD OCT 3

PY 2017

VL 8

IS 45

BP 79662

EP 79669

DI 10.18632/oncotarget.19030

PG 8

WC Oncology; Cell Biology

WE Science Citation Index Expanded (SCI-EXPANDED)

SC Oncology; Cell Biology

GA FI6MT

UT WOS:000412111300130

PM 29108346

OA Green Published, gold

DA 2022-05-19

ER

PT J

AU Ansari, MH

Irani, S

Edalat, H

Amin, R

Roushandeh, AM

AF Ansari, Mohammad Hossein

Irani, Shiva

Edalat, Houri

Amin, Ruhul

Roushandeh, Amaneh Mohammadi

TI Deregulation of miR-93 and miR-143 in human esophageal cancer

SO TUMOR BIOLOGY

LA English

DT Article

DE Esophageal cancer; Squamous cell carcinoma; Adenocarcinoma of the

esophagus; miR-93; miR-143

ID SQUAMOUS-CELL CARCINOMA; DOWN-REGULATION; PROMOTES APOPTOSIS;

TUMOR-SUPPRESSOR; CERVICAL-CANCER; GASTRIC-CANCER; HUMAN MICRORNA;

EXPRESSION; PROGNOSIS; MIRNA

AB Esophageal squamous cell carcinoma (ESCC) is the second and third most common malignancy in Iranian males and females, respectively. Treatment of ESCC is largely ineffective due to lack of detection at early stages of the disease. In recent years, miRNA, a small RNA molecule, has drawn much attention to researchers as a potential biomarker for esophageal cancer. miR-93 and miR-143 are two miRNA molecules reported to be frequently deregulated in various cancers, including prostate, stomach, cervix, and etc. The purpose of this study was to investigate the expression levels of these miRNAs and evaluate their diagnostic and therapeutic potential in esophageal squamous cell carcinoma. In this study, total RNA was extracted from 30 tumor tissues and 30 nontumor tissues of esophageal tumor margins, using RNX-plus solution. After validating the quality and quantity of total RNA, cDNAs of interest were synthesized using microRNA-specific cDNA Synthesis Kit. The expression level of miR-93 and miR-143 was evaluated using quantitative real-time PCR with miRNA-specific primers. Finally, the obtained data was analyzed by SPSS ver.20 software and paired t test was performed to observe the significance of difference between groups. The expression level of miR-93 was significantly increased and of miR-143 was significantly decreased in most of the examined tumor tissues, compared to nontumor tissues. Also, our findings did not detect correlation between mir-93 and mir-143 expressions in regard to stage and grade of the samples. These findings suggest that the deregulation of these miRNAs may play an important role in esophageal squamous cell carcinoma. Both miR-93 and miR-143 might be used as potential biomarkers in esophageal squamous cell carcinoma. However, more studies with large population of samples are necessary.

C1 [Ansari, Mohammad Hossein; Edalat, Houri; Roushandeh, Amaneh Mohammadi] Hamadan Univ Med Sci, Fac Med, Res Ctr Mol Med, Hamadan, Iran.

[Irani, Shiva] Islamic Azad Univ, Sch Basic Sci, Dept Biol, Sci & Res Branch, Tehran, Iran.

[Amin, Ruhul] Tohoku Univ, IDAC, Dept Project Program, Sendai, Miyagi 980, Japan.

[Roushandeh, Amaneh Mohammadi] Hamadan Univ Med Sci, Fac Med, Dept Anat Sci, Hamadan, Iran.

RP Roushandeh, AM (corresponding author), Hamadan Univ Med Sci, Fac Med, Res Ctr Mol Med, Hamadan, Iran.; Roushandeh, AM (corresponding author), Hamadan Univ Med Sci, Fac Med, Dept Anat Sci, Hamadan, Iran.

EM a.mohammadiroshandeh@umsha.ac.ir

RI Mohammadi Roushandeh, Amaneh/F-3423-2017; Edalat, Houri/B-1957-2019;

Roushandeh, Amaneh Mohammadi/AAA-2860-2021; Ansari, Mohammad

Hossein/AAH-6470-2019; Irani, shiva/AAE-8167-2020

OI Mohammadi Roushandeh, Amaneh/0000-0002-3639-2291; Edalat,

Houri/0000-0003-4508-5514; Roushandeh, Amaneh

Mohammadi/0000-0002-3639-2291; Ansari, Mohammad

Hossein/0000-0002-1839-4314; Irani, Shiva/0000-0002-4202-9931; Amin, Md

Ruhul/0000-0001-8826-4040

CR Bartel DP, 2004, CELL, V116, P281, DOI 10.1016/S0092-8674(04)00045-5

Calin GA, 2004, P NATL ACAD SCI USA, V101, P2999, DOI 10.1073/pnas.0307323101

Chen HC, 2009, BRIT J CANCER, V100, P1002, DOI 10.1038/sj.bjc.6604948

Chen X, 2009, ONCOGENE, V28, P1385, DOI 10.1038/onc.2008.474

Chiam K, 2015, J GASTROINTEST SURG, V19, P1208, DOI 10.1007/s11605-015-2829-9

Cho WCS, 2010, INT J BIOCHEM CELL B, V42, P1273, DOI 10.1016/j.biocel.2009.12.014

Cho WCS, 2010, BBA-REV CANCER, V1805, P209, DOI 10.1016/j.bbcan.2009.11.003

Chu HY, 2016, ARCH TOXICOL, V90, P403, DOI 10.1007/s00204-014-1396-2

Clape C, 2009, PLOS ONE, V4, DOI 10.1371/journal.pone.0007542

Dews M, 2006, NAT GENET, V38, P1060, DOI 10.1038/ng1855

Enzinger PC, 2003, NEW ENGL J MED, V349, P2241, DOI 10.1056/NEJMra035010

Eslick GD, 2009, GASTROENTEROL CLIN N, V38, P17, DOI 10.1016/j.gtc.2009.01.008

Fang L, 2012, CELL CYCLE, V11, P4352, DOI 10.4161/cc.22670

Feber A, 2008, J THORAC CARDIOV SUR, V135, P255, DOI 10.1016/j.jtcvs.2007.08.055

Gao W, 2010, BIOMED PHARMACOTHER, V64, P399, DOI 10.1016/j.biopha.2010.01.018

Goto Y, 2015, INT J UROL, V22, P242, DOI 10.1111/iju.12700

Gu J, 2013, CURR PHARM DESIGN, V19, P1292

HALDAR S, 1994, CANCER RES, V54, P2095

Hemann MT, 2006, CELL DEATH DIFFER, V13, P1256, DOI 10.1038/sj.cdd.4401962

HENRIKSEN R, 1995, BRIT J CANCER, V72, P1324, DOI 10.1038/bjc.1995.509

Hu XX, 2010, CANCER RES, V70, P1441, DOI 10.1158/0008-5472.CAN-09-3289

Hu ZH, 2009, BMC GENOMICS, V10, DOI 10.1186/1471-2164-10-594

Hwang HW, 2006, BRIT J CANCER, V94, P776, DOI 10.1038/sj.bjc.6603023

Ilhan-Mutlu A, 2015, CANCER INVEST, V33, P246, DOI 10.3109/07357907.2015.1024319

Ivanovska I, 2008, MOL CELL BIOL, V28, P2167, DOI 10.1128/MCB.01977-07

Jay C, 2007, DNA CELL BIOL, V26, P293, DOI 10.1089/dna.2006.0554

Kamangar F, 2007, ARCH IRAN MED, V10, P70

Kano M, 2010, INT J CANCER, V127, P2804, DOI 10.1002/ijc.25284

Kim YK, 2007, EMBO J, V26, P775, DOI 10.1038/sj.emboj.7601512

Kojima S, 2014, J HUM GENET, V59, P78, DOI 10.1038/jhg.2013.121

Lardizabal MN, 2012, PLOS ONE, V7, DOI 10.1371/journal.pone.0036323

LEEK RD, 1994, BRIT J CANCER, V69, P135, DOI 10.1038/bjc.1994.22

Li G, 2015, TUMOR BIOL, V36, P3949, DOI 10.1007/s13277-015-3038-6

Lin S-L, 2013, PROTOCOLS, V342, P295

Lin YS, 2013, J EPIDEMIOL, V23, P233, DOI 10.2188/jea.JE20120162

Liu J, 2015, J EXP CLIN CANC RES, V34, DOI 10.1186/s13046-015-0154-5

Liu LP, 2012, MOL MED REP, V5, P753, DOI 10.3892/mmr.2011.696

Lotterman CD, 2008, CELL CYCLE, V7, P2493, DOI 10.4161/cc.7.16.6452

Lu M, 2008, PLOS ONE, V3, DOI 10.1371/journal.pone.0003420

Luthra R, 2008, ONCOGENE, V27, P6667, DOI 10.1038/onc.2008.256

Maru DM, 2009, AM J PATHOL, V174, P1940, DOI 10.2353/ajpath.2009.080718

Mattie MD, 2006, MOL CANCER, V5, DOI 10.1186/1476-4598-5-24

Nie JH, 2015, ONCOL REP, V34, P311, DOI 10.3892/or.2015.3962

Noguchi S, 2011, CANCER LETT, V307, P211, DOI 10.1016/j.canlet.2011.04.005

Pagliuca A, 2013, ONCOGENE, V32, P4806, DOI 10.1038/onc.2012.495

Petrocca F, 2008, CANCER CELL, V13, P272, DOI 10.1016/j.ccr.2008.02.013

Purwar P, 2015, ANN THORAC SURG, V99, P1488, DOI 10.1016/j.athoracsur.2014.11.029

Rajewsky N, 2006, NAT GENET, V38, pS8, DOI 10.1038/ng1798

Raponi M, 2009, CANCER RES, V69, P5776, DOI 10.1158/0008-5472.CAN-09-0587

Ross JS, 2007, AM J CLIN PATHOL, V128, P830, DOI 10.1309/2JK279BU2G743MWJ

Roth MJ, 1997, CANCER-AM CANCER SOC, V80, P2047, DOI 10.1002/(SICI)1097-0142(19971201)80:11<2047::AID-CNCR3>3.0.CO;2-U

Roush SF, 2006, ACS CHEM BIOL, V1, P132, DOI 10.1021/cb600138j

Sadjadi A, 2010, MIDDLE EAST J CANCER, V1, P5

Schou JV, 2014, PLOS ONE, V9, DOI 10.1371/journal.pone.0099886

Stewart BW, 2003, CANC WORLD CANC REPO, V57

Thai TH, 2010, DISCOV MED, V10, P184

Tricoli James V, 2007, Cancer Res, V67, P4553, DOI 10.1158/0008-5472.CAN-07-0563

Vasudevan S, 2012, WIRES RNA, V3, P311, DOI 10.1002/wrna.121

Vychytilova-Faltejskova P, 2015, DIAGN PATHOL, V10, DOI 10.1186/s13000-015-0272-6

Wang FJ, 2010, GYNECOL ONCOL, V119, P586, DOI 10.1016/j.ygyno.2010.07.021

Wszolek MF, 2011, UROL ONCOL-SEMIN ORI, V29, P794, DOI 10.1016/j.urolonc.2009.08.024

Wu BL, 2011, WORLD J GASTROENTERO, V17, P79, DOI 10.3748/wjg.v17.i1.79

Xu B, 2011, MOL CELL BIOCHEM, V350, P207, DOI 10.1007/s11010-010-0700-6

Yu D, 2014, LAB INVEST, V94, P1370, DOI 10.1038/labinvest.2014.125

Zehetner J, 2011, J THORAC CARDIOV SUR, V141, P39, DOI 10.1016/j.jtcvs.2010.08.058

Zhang H, 2010, ONCOL REP, V24, P1363, DOI 10.3892/or_00000994

Zhang J, 2013, ONCOGENE, V32, P61, DOI 10.1038/onc.2012.28

Zheng ST, 2010, EUR J GASTROEN HEPAT, V22, P917, DOI 10.1097/MEG.0b013e3283313d8b

NR 68

TC 38

Z9 43

U1 0

U2 8

PU SAGE PUBLICATIONS LTD

PI LONDON

PA 1 OLIVERS YARD, 55 CITY ROAD, LONDON EC1Y 1SP, ENGLAND

SN 1010-4283

EI 1423-0380

J9 TUMOR BIOL

JI Tumor Biol.

PD MAR

PY 2016

VL 37

IS 3

BP 3097

EP 3103

DI 10.1007/s13277-015-3987-9

PG 7

WC Oncology

WE Science Citation Index Expanded (SCI-EXPANDED)

SC Oncology

GA DK4QJ

UT WOS:000374903500034

PM 26427659

DA 2022-05-19

ER

PT J

AU Chen, YH

Li, SH

Chiu, YC

Lu, HI

Huang, CH

Rau, KM

Liu, CT

AF Chen, Yen-Hao

Li, Shau-Hsuan

Chiu, Yi-Chun

Lu, Hung-I

Huang, Cheng-Hua

Rau, Kun-Ming

Liu, Chien-Ting

TI Comparative Study of Esophageal Stent and Feeding

Gastrostomy/Jejunostomy for Tracheoesophageal Fistula Caused by

Esophageal Squamous Cell Carcinoma

SO PLOS ONE

LA English

DT Article

ID EXPANDING METAL STENTS; MALIGNANT ESOPHAGORESPIRATORY FISTULA; 20-YEAR

PERIOD; MANAGEMENT; CANCER; PROSTHESES; THERAPY; AIRWAY

AB Background: A malignant tracheoesophageal/bronchoesophageal fistula (TEF) is a life-threatening complication of esophageal squamous cell carcinoma. A feeding gastrostomy/jejunostomy had been the most common treatment method for patients with TEF before the era of stenting. The aim of this retrospective study is to compare the prognosis of esophageal squamous cell carcinoma patients with TEF treated with an esophageal metallic stent to those treated with a feeding gastrostomy/jejunostomy.

Methods: We retrospectively reviewed a total of 1011 patients with esophageal squamous cell carcinoma between 1996 and 2011 at Kaohsiung Chang Gung Memorial Hospital, and 86 patients with TEF (8.5%) were identified. The overall survival and other clinical data were compared between 30 patients treated with an esophageal metallic stent and 35 patients treated with a feeding gastrostomy/jejunostomy.

Results: Among the 65 patients receiving either an esophageal metallic stent or a feeding gastrostomy/jejunostomy, univariate analysis showed that treatment modality with an esophageal metallic stent (P = 0.007) and radiotherapy treatment after fistula diagnosis (P = 0.04) were predictive of superior overall survival. In the multivariate comparison, treatment modality with an esophageal metallic stent (P = 0.026, odds ratio: 1.859) represented the independent predictive factor of superior overall survival. There were no significant differences between groups in mean decrease in serum albumin or mean body weight loss. Compared to the feeding gastrostomy/jejunostomy group, a significantly higher proportion of patients in the stenting group (53% versus 14%, P = 0.001) were able to receive chemotherapy within 30 days after fistula diagnosis, indicating better infection control in the stenting group.

Conclusions: Compared with a feeding gastrostomy/jejunostomy, an esophageal metallic stent significantly improves overall survival in patients with malignant TEF in our retrospective analysis. Esophageal metallic stent placement may be considered the first-line of treatment for patients with malignant TEF.

C1 [Chen, Yen-Hao; Li, Shau-Hsuan; Huang, Cheng-Hua; Rau, Kun-Ming; Liu, Chien-Ting] Kaohsiung Chang Gung Mem Hosp, Dept Hematol Oncol, Kaohsiung, Taiwan.

[Chen, Yen-Hao; Li, Shau-Hsuan; Chiu, Yi-Chun; Lu, Hung-I; Huang, Cheng-Hua; Rau, Kun-Ming; Liu, Chien-Ting] Chang Gung Univ, Coll Med, Kaohsiung, Taiwan.

[Chiu, Yi-Chun] Kaohsiung Chang Gung Mem Hosp, Dept Hepatogastroenterol, Kaohsiung, Taiwan.

[Lu, Hung-I] Kaohsiung Chang Gung Mem Hosp, Dept Thorac & Cardiovasc Surg, Kaohsiung, Taiwan.

RP Chen, YH (corresponding author), Kaohsiung Chang Gung Mem Hosp, Dept Hematol Oncol, Kaohsiung, Taiwan.

EM lee.a0928@msa.hinet.net

FU National Science Council, Taiwan [NSC 100-2314-B-182A-044-MY3]; Chang

Gung Memorial Hospital [CMRPG8B0431]

FX This work was supported in part by grants from the National Science

Council, Taiwan (NSC 100-2314-B-182A-044-MY3) and Chang Gung Memorial

Hospital (CMRPG8B0431). The funders had no role in study design, data

collection and analysis, decision to publish, or preparation of the

manuscript

CR Balazs A, 2008, EUR J CARDIO-THORAC, V34, P1103, DOI 10.1016/j.ejcts.2008.06.025

Balazs A, 2009, WORLD J SURG, V33, P994, DOI 10.1007/s00268-009-9988-3

BURT M, 1991, ANN THORAC SURG, V52, P1222, DOI 10.1016/0003-4975(91)90005-B

Choi MK, 2010, MED ONCOL, V27, P1234, DOI 10.1007/s12032-009-9364-z

Freitag L, 1996, CHEST, V110, P1155, DOI 10.1378/chest.110.5.1155

GUDOVSKY LM, 1993, ANN THORAC SURG, V55, P868, DOI 10.1016/0003-4975(93)90108-T

Hu Y, 2009, DIS ESOPHAGUS, V22, P526, DOI 10.1111/j.1442-2050.2009.00950.x

Lazaraki G, 2011, SURG ENDOSC, V25, P628, DOI 10.1007/s00464-010-1236-2

LOLLEY DM, 1978, ANN THORAC SURG, V25, P516, DOI 10.1016/S0003-4975(10)63600-4

Low DE, 2003, SURG ENDOSC, V17, P990, DOI 10.1007/s00464-002-8528-0

Low DE, 1998, ANN THORAC SURG, V65, P919, DOI 10.1016/S0003-4975(98)00081-2

Margolis M, 2003, ANN THORAC SURG, V76, P1694, DOI 10.1016/S0003-4975(02)04890-7

MARTINI N, 1970, J THORAC CARDIOV SUR, V59, P319, DOI 10.1016/S0022-5223(19)42464-1

May A, 1996, J CLIN GASTROENTEROL, V22, P261, DOI 10.1097/00004836-199606000-00004

Morgan RA, 1997, RADIOLOGY, V204, P527, DOI 10.1148/radiology.204.2.9240548

Nomori H, 2000, ANN THORAC SURG, V70, P1803, DOI 10.1016/S0003-4975(00)02042-7

Paganin F, 2008, PLOS ONE, V3, DOI 10.1371/journal.pone.0003101

Shin JH, 2004, RADIOLOGY, V232, P252, DOI 10.1148/radiol.2321030733

Siddiqui AA, 2009, DIS ESOPHAGUS, V22, P216, DOI 10.1111/j.1442-2050.2008.00905.x

Siersema PD, 1998, GASTROINTEST ENDOSC, V47, P113, DOI 10.1016/S0016-5107(98)70342-6

Siewert JR, 2007, SEMIN RADIAT ONCOL, V17, P38, DOI 10.1016/j.semradonc.2006.09.007

Spivak H, 1996, J SURG ONCOL, V63, P65, DOI 10.1002/(SICI)1096-9098(199609)63:1<65::AID-JSO13>3.0.CO;2-O

Talreja JP, 2011, SURG ENDOSC	1216

Tomaselli F, 2001, EUR J CARDIO-THORAC, V20, P734, DOI 10.1016/S1010-7940(01)00867-3

WEIGERT N, 1995, GASTROINTEST ENDOSC, V41, P490, DOI 10.1016/S0016-5107(05)80009-4

NR 25

TC 32

Z9 40

U1 0

U2 9

PU PUBLIC LIBRARY SCIENCE

PI SAN FRANCISCO

PA 1160 BATTERY STREET, STE 100, SAN FRANCISCO, CA 94111 USA

SN 1932-6203

J9 PLOS ONE

JI PLoS One

PD AUG 13

PY 2012

VL 7

IS 8

AR e42766

DI 10.1371/journal.pone.0042766

PG 9

WC Multidisciplinary Sciences

WE Science Citation Index Expanded (SCI-EXPANDED)

SC Science & Technology - Other Topics

GA 988OJ

UT WOS:000307500100030

PM 22912737

OA gold, Green Submitted, Green Published

DA 2022-05-19

ER

PT J

AU Meng, P

Wei, JC

Geng, YQ

Chen, SB

Terpstra, MM

Huang, QY

Zhang, Q

Su, ZQ

Yu, WC

Su, M

Kok, K

van den Berg, A

Gu, J

AF Meng, Pei

Wei, Jiacong

Geng, Yiqun

Chen, Shaobin

Terpstra, Miente Martijn

Huang, Qiongyi

Zhang, Qian

Su, Zuoqing

Yu, Wanchun

Su, Min

Kok, Klaas

van den Berg, Anke

Gu, Jiang

TI Targeted sequencing of circulating cell-free DNA in stage II-III

resectable oesophageal squamous cell carcinoma patients

SO BMC CANCER

LA English

DT Article

DE Oesophageal squamous cell carcinoma; Circulating cell-free DNA;

Next-generation sequencing

ID TUMOR-DNA; MUTATIONS; PLASMA; HETEROGENEITY; FRAMEWORK; KRAS

AB Background The aim of this study was to investigate the potential of cell-free DNA (cfDNA) as a disease biomarker in oesophageal squamous cell carcinoma (ESCC) that can be used for treatment response evaluation and early detection of tumour recurrence. Methods Matched tumour tissue, pre- and post-surgery plasma and WBCs obtained from 17 ESCC patients were sequenced using a panel of 483 cancer-related genes. Results Somatic mutations were detected in 14 of 17 tumour tissues. Putative harmful mutations were observed in genes involved in well-known cancer-related pathways, including PI3K-Akt/mTOR signalling, Proteoglycans in cancer, FoxO signalling, Jak-STAT signalling, Chemokine signalling and Focal adhesion. Forty-six somatic mutations were found in pre-surgery cfDNA in 8 of 12 patients, with mutant allele frequencies (MAF) ranging from 0.24 to 4.91%. Three of the 8 patients with detectable circulating tumour DNA (ctDNA) had stage IIA disease, whereas the others had stage IIB-IIIB disease. Post-surgery cfDNA somatic mutations were detected in only 2 of 14 patients, with mutant allele frequencies of 0.28 and 0.36%. All other somatic mutations were undetectable in post-surgery cfDNA, even in samples collected within 3-4 h after surgery. Conclusion Our study shows that somatic mutations can be detected in pre-surgery cfDNA in stage IIA to IIIB patients, and at a lower frequency in post-surgery cfDNA. This indicates that cfDNA could potentially be used to monitor disease load, even in low disease-stage patients.

C1 [Meng, Pei; Wei, Jiacong; Geng, Yiqun; Huang, Qiongyi; Zhang, Qian; Su, Zuoqing; Yu, Wanchun; Gu, Jiang] Shantou Univ, Coll Med, Prov Key Lab Infect Dis & Mol Pathol, Dept Pathol & Pathophysiol,Collaborat & Creat Ctr, Shantou 515041, Guangdong, Peoples R China.

[Meng, Pei; van den Berg, Anke] Univ Groningen, Univ Med Ctr Groningen, Dept Pathol & Med Biol, NL-9700 RB Groningen, Netherlands.

[Wei, Jiacong; Terpstra, Miente Martijn; Kok, Klaas] Univ Groningen, Univ Med Ctr Groningen, Dept Genet, NL-9700 RB Groningen, Netherlands.

[Chen, Shaobin] Shantou Univ, Canc Hosp, Dept Thorac Surg, Shantou 515041, Guangdong, Peoples R China.

[Su, Min] Shantou Univ, Coll Med, Dept Pathol, Shantou 515041, Guangdong, Peoples R China.

[Su, Min] Shantou Univ, Coll Med, Inst Clin Pathol, Shantou 515041, Guangdong, Peoples R China.

[Gu, Jiang] Chengdu Jinjiang Hosp Maternal & Child Hlth Care, Jinxin Res Inst Reprod Med & Genet, 66 Jingxiu Rd, Chengdu 610066, Peoples R China.

RP Gu, J (corresponding author), Shantou Univ, Coll Med, Prov Key Lab Infect Dis & Mol Pathol, Dept Pathol & Pathophysiol,Collaborat & Creat Ctr, Shantou 515041, Guangdong, Peoples R China.; Gu, J (corresponding author), Chengdu Jinjiang Hosp Maternal & Child Hlth Care, Jinxin Res Inst Reprod Med & Genet, 66 Jingxiu Rd, Chengdu 610066, Peoples R China.

EM p.meng@umcg.nl; 2523381625@qq.com

RI Chen, Shao-bin/ABE-9239-2021; van den Berg, Anke/H-1718-2011

OI Gu, Jiang/0000-0002-0706-8329; Meng, Pei/0000-0002-2186-1622; van den

Berg, Anke/0000-0002-8894-2638

FU Li Ka Shing Foundation; National Natural Science Foundation of China

[81200712, 81872334]

FX This work is supported by Li Ka Shing Foundation and the National

Natural Science Foundation of China (81200712 and 81872334). The funders

played no role in research design, data collection, analysis, and

interpretation as well as manuscript writing.

CR Adleff V, 2017, SCI TRANSL MED, V9

Banki F, 2007, ARCH SURG-CHICAGO, V142, P533, DOI 10.1001/archsurg.142.6.533

Bettegowda C, 2014, SCI TRANSL MED, V6, DOI 10.1126/scitranslmed.3007094

Diehl F, 2005, P NATL ACAD SCI USA, V102, P16368, DOI 10.1073/pnas.0507904102

Diehl F, 2008, NAT MED, V14, P985, DOI 10.1038/nm.1789

Douillard JY, 2014, J THORAC ONCOL, V9, P1345, DOI 10.1097/JTO.0000000000000263

Forshew T, 2012, SCI TRANSL MED, V4, DOI 10.1126/scitranslmed.3003726

Heitzer E, 2015, CLIN CHEM, V61, P112, DOI 10.1373/clinchem.2014.222679

Huang DW, 2009, NAT PROTOC, V4, P44, DOI 10.1038/nprot.2008.211

Hye-Ryoun Kim SYL, 2014, J EXP CLIN CANC RES, V32, P50

Kircher M, 2014, NAT GENET, V46, P310, DOI 10.1038/ng.2892

Li LL, 2017, J CANCER, V8, P2051, DOI 10.7150/jca.18981

Liu Y, 2014, LEUKEMIA, V28, P2248, DOI 10.1038/leu.2014.201

Luo HL, 2016, BIOCHEM BIOPH RES CO, V471, P596, DOI 10.1016/j.bbrc.2016.02.011

Majure M, 2016, ANN TRANSL MED, V4, DOI 10.21037/atm.2016.11.77

McCormick RF, 2015, G3-GENES GENOM GENET, V5, P655, DOI 10.1534/g3.115.017012

McKenna A, 2010, GENOME RES, V20, P1297, DOI 10.1101/gr.107524.110

Newman AM, 2014, NAT MED, V20, P552, DOI 10.1038/nm.3519

Pennathur A, 2013, LANCET, V381, P400, DOI 10.1016/S0140-6736(12)60643-6

Qi YJ, 2012, J PROTEOMICS, V75, P3129, DOI 10.1016/j.jprot.2012.04.025

Qing T, 2017, SCI REP-UK, V7, DOI 10.1038/s41598-017-04221-7

Rostami A, 2017, RADIOTHER ONCOL, V124, P357, DOI 10.1016/j.radonc.2017.07.004

Rothe F, 2014, ANN ONCOL, V25, P1959, DOI 10.1093/annonc/mdu288

Saber A, 2017, CARCINOGENESIS, V38, P144, DOI 10.1093/carcin/bgw128

Sausen M, 2015, NAT COMMUN, V6, DOI 10.1038/ncomms8686

Shen WB, 2017, WORLD J SURG ONCOL, V15, DOI 10.1186/s12957-017-1259-4

Siravegna G, 2015, NAT MED, V21, P795, DOI 10.1038/nm.3870

Thierry AR, 2014, NAT MED, V20, P430, DOI 10.1038/nm.3511

Tie J, 2016, SCI TRANSL MED, V8, DOI 10.1126/scitranslmed.aaf6219

Wei JC, 2018, CANCERS, V10, DOI 10.3390/cancers10010010

Xiao ZF, 2003, ANN THORAC SURG, V75, P331, DOI 10.1016/S0003-4975(02)04401-6

Xu JM, 2014, J EXP CLIN CANC RES, V33, DOI 10.1186/s13046-014-0104-7

Xu S, 2016, CANCER LETT, V370, P324, DOI 10.1016/j.canlet.2015.11.005

Yu JY, 2016, CHIN J CANCER, V35, DOI 10.1186/s40880-016-0086-2

NR 34

TC 8

Z9 8

U1 1

U2 2

PU BMC

PI LONDON

PA CAMPUS, 4 CRINAN ST, LONDON N1 9XW, ENGLAND

EI 1471-2407

J9 BMC CANCER

JI BMC Cancer

PD AUG 20

PY 2019

VL 19

IS 1

AR 818

DI 10.1186/s12885-019-6025-2

PG 10

WC Oncology

WE Science Citation Index Expanded (SCI-EXPANDED)

SC Oncology

GA IT5PX

UT WOS:000482918300001

PM 31429737

OA gold, Green Published

DA 2022-05-19

ER

PT J

AU Okello, S

Churchill, C

Owori, R

Nasasira, B

Tumuhimbise, C

Abonga, CL

Mutiibwa, D

Christiani, DC

Corey, KE

AF Okello, Samson

Churchill, Cristina

Owori, Rogers

Nasasira, Benson

Tumuhimbise, Christine

Abonga, Charles Lagoro

Mutiibwa, David

Christiani, David C.

Corey, Kathleen E.

TI Population attributable fraction of Esophageal squamous cell carcinoma

due to smoking and alcohol in Uganda

SO BMC CANCER

LA English

DT Article

DE Population attributable fraction; Esophageal squamous cell carcinoma;

Alcohol; Smoking; Africa

ID RISK-FACTORS; HUMAN-PAPILLOMAVIRUS; TOBACCO SMOKING; CANCER; DRINKING;

LINXIAN; METHYLATION; COHORT; JOINT

AB Background: Despite the high rates and regional variation of esophageal squamous cell carcinoma (ESCC) in East Africa, the contributions of smoking and alcohol to the ESCC burden in the general population are unknown.

Methods: We conducted a case-control study of patients presenting for upper gastrointestinal endoscopic examination at Mbarara Regional Referral Hospital, Uganda. Sociodemographic data including smoking and alcohol intake were collected prior to endoscopy. Cases were those with histological diagnosis of ESCC and controls were participants with normal endoscopic examination and gastritis/duodentitis or normal histology. We used odds ratios associated with ESCC risk to determine the population attributable fractions for smoking, alcohol use, and a combination of smoking and alcohol use among adults aged 30 years or greater who underwent upper gastrointestinal endoscopy.

Results: Our study consisted of 67 cases and 142 controls. Median age was 51 years (IQR 40-64); and participants were predominantly male (59 %). Dysphagia and/or odynophagia as indications for endoscopy were significantly more in cases compared to controls (72 % vs 6 %, p < 0.0001). Male gender and increasing age were statistically associated with ESCC. In the unadjusted models, the population attributable fraction of ESCC due to male gender was 55 %, female gender - 49 %, smoking 20 %, alcohol 9 % and a combination of alcohol & smoking 15 %. After adjusting for gender and age, the population attributable fraction of ESCC due to smoking, alcohol intake and a combination of alcohol & smoking were 16, 10, and 13 % respectively.

Conclusion: In this population, 13 % of esophageal squamous cell carcinoma cases would be avoided if smoking and alcohol use were discontinued. These results suggest that other important risk factors for ESCC in southwestern Uganda remain unknown.

C1 [Okello, Samson; Nasasira, Benson; Tumuhimbise, Christine; Abonga, Charles Lagoro] Mbarara Univ Sci & Technol, Dept Internal Med, POB 1410, Mbarara, Uganda.

[Okello, Samson] Harvard TH Chan Sch Publ Hlth, Dept Epidemiol, Boston, MA USA.

[Churchill, Cristina] Univ Virginia, Dept Med, Charlottesville, VA USA.

[Owori, Rogers] Gwatiiro Hosp, Wakiso, Uganda.

[Mutiibwa, David] Mbarara Univ Sci & Technol, Dept Surg, Mbarara, Uganda.

[Christiani, David C.] Harvard TH Chan Sch Publ Hlth, Dept Environm Hlth, Boston, MA USA.

[Christiani, David C.; Corey, Kathleen E.] Massachusetts Gen Hosp, Harvard Med Sch, Boston, MA USA.

[Corey, Kathleen E.] Massachusetts Gen Hosp, Dept Med, Boston, MA USA.

RP Okello, S (corresponding author), Mbarara Univ Sci & Technol, Dept Internal Med, POB 1410, Mbarara, Uganda.; Okello, S (corresponding author), Harvard TH Chan Sch Publ Hlth, Dept Epidemiol, Boston, MA USA.

EM okello.samson@must.ac.ug

RI Okello, Samson/I-1877-2019

OI Okello, Samson/0000-0001-7377-6094

FU NATIONAL INSTITUTE OF DIABETES AND DIGESTIVE AND KIDNEY DISEASES

[K23DK099422] Funding Source: NIH RePORTER; NIDDK NIH HHS [K23 DK099422]

Funding Source: Medline

CR Alema ON, 2014, AFR HEALTH SCI, V14, P17, DOI 10.4314/ahs.v14i1.4

BENICHOU J, 1990, BIOMETRICS, V46, P991, DOI 10.2307/2532443

BROWN LM, 1994, J NATL CANCER I, V86, P1340, DOI 10.1093/jnci/86.17.1340

Castellsague X, 1999, INT J CANCER, V82, P657, DOI 10.1002/(SICI)1097-0215(19990827)82:5&lt;657::AID-IJC7&gt;3.0.CO;2-C

Castellsague X, 2000, INT J CANCER, V88, P658, DOI 10.1002/1097-0215(20001115)88:4&lt;658::AID-IJC22&gt;3.0.CO;2-T

Chen J, 2012, ASIAN PAC J CANCER P, V13, P5303, DOI 10.7314/APJCP.2012.13.10.5303

Dawsey SP, 2010, PLOS ONE, V5, DOI 10.1371/journal.pone.0014080

Dong J, 2013, ONCOL REP, V30, P2771, DOI 10.3892/or.2013.2770

Engel LS, 2003, J NATL CANCER I, V95, P1404, DOI 10.1093/jnci/djg047

Farhadi M, 2005, WORLD J GASTROENTERO, V11, P1200, DOI 10.3748/wjg.v11.i8.1200

Ferlay J, 2013, CANC INCIDENCE MORTA

Ferlay J, 2010, INT J CANCER, V127, P2893, DOI 10.1002/ijc.25516

Antunes JLF, 2013, PLOS ONE, V8, DOI 10.1371/journal.pone.0068132

Gibbons MC, 2007, J URBAN HEALTH, V84, P198, DOI 10.1007/s11524-006-9141-7

Hiripi E, 2012, ACTA ONCOL, V51, P906, DOI 10.3109/0284186X.2012.673732

Huang YP, 2011, INT J CANCER, V128, P2261, DOI 10.1002/ijc.25569

Jemal A, 2011, CA-CANCER J CLIN, V61, P69, DOI [10.3322/caac.20107, 10.3322/caac.20115]

Lofdahl HE, 2012, PLOS ONE, V7, DOI 10.1371/journal.pone.0046538

Mapstone N. P., 2007, DATASET HISTOPATHOLO, V2nd

Matejcic M, 2015, CRIT REV CL LAB SCI, V52, P211, DOI 10.3109/10408363.2015.1020358

Ocama P, 2008, AFR HEALTH SCI, V8, P8

Parker RK, 2010, DIS ESOPHAGUS, V23, P128, DOI 10.1111/j.1442-2050.2009.00977.x

Petrick JL, 2014, BRIT J CANCER, V110, P2369, DOI 10.1038/bjc.2014.96

Qiao YL, 2009, JNCI-J NATL CANCER I, V101, P507, DOI 10.1093/jnci/djp037

ROTHMAN KJ, 1980, PREV MED, V9, P174, DOI 10.1016/0091-7435(80)90072-9

SIEMIATYCKI J, 1995, INT J EPIDEMIOL, V24, P504, DOI 10.1093/ije/24.3.504

[Statistics UBo Statistics UBo], 2013, STAT ABSTR 2013

Tihan T, 2001, ANTICANCER RES, V21, P3107

Tran GD, 2005, INT J CANCER, V113, P456, DOI 10.1002/ijc.20616

VAUGHAN TL, 1995, CANCER EPIDEM BIOMAR, V4, P85

Wang GQ, 2004, ANN THORAC SURG, V77, P1740, DOI 10.1016/j.athoracsur.2003.10.098

YU Y, 1993, CANCER CAUSE CONTROL, V4, P195

Zhang H, 2004, WORLD J GASTROENTERO, V10, P1834

ZIEGLER RG, 1991, AM J CLIN NUTR, V53, pS251, DOI 10.1093/ajcn/53.1.251S

NR 34

TC 20

Z9 21

U1 0

U2 4

PU BMC

PI LONDON

PA CAMPUS, 4 CRINAN ST, LONDON N1 9XW, ENGLAND

EI 1471-2407

J9 BMC CANCER

JI BMC Cancer

PD JUL 11

PY 2016

VL 16

AR 446

DI 10.1186/s12885-016-2492-x

PG 6

WC Oncology

WE Science Citation Index Expanded (SCI-EXPANDED)

SC Oncology

GA DR6LZ

UT WOS:000380014700001

PM 27400987

OA Green Published, gold

DA 2022-05-19

ER

PT J

AU Yang, Z

Dai, HH

Lv, DX

Feng, AL

Shu, WB

Han, JQ

AF Yang, Zhe

Dai, Honghai

Lv, Dongxiao

Feng, A. Lei

Shu, Weibin

Han, Junqing

TI Clinical observation of gene expression-guided chemoradiation therapy

for nonsurgical esophageal squamous cell carcinoma patients: a

retrospective analysis of 36 cases

SO ONCOTARGETS AND THERAPY

LA English

DT Article

DE esophageal squamous cell carcinoma; gene expression; chemoradiation

therapy; survival

ID CISPLATIN-BASED CHEMOTHERAPY; MESSENGER-RNA EXPRESSION; PHASE-III TRIAL;

LUNG-CANCER; NEOADJUVANT RADIOCHEMOTHERAPY; COLORECTAL-CANCER; RRM1

EXPRESSION; SOLID TUMORS; CHEMORADIOTHERAPY; ERCC1

AB Objective: To make an informed choice of chemotherapy drugs according to the oncogene mRNA expression and to explore whether it could increase the survival rate of patients.

Patients and methods: The study retrospectively analyzed 36 cases of nonsurgical esophageal squamous cell carcinoma patients treated at the Center for Oncology of Shandong Provincial Hospital from December 1, 2010, to November 1, 2013. Intensity-modulated radiation therapy was used for the treatment with a conventional radiotherapy dose of 60-66 Gy. Chemotherapy started 1-5 weeks after radiation therapy. The selection of the chemotherapy drug was based on the mRNA expression levels of excision repair cross-complementation 1, thymidylate synthetase, ribonucleotide reductase M1, and beta-tubulin isotype III. The objective response rate, progression-free survival, and overall survival were observed.

Results: The reason for poor prognosis of patients with high expression of excision repair cross-complementation 1 was unknown. No correlation was observed between patient survival and expression of thymidylate synthetase, ribonucleotide reductase M1, and beta-tubulin isotype III. Complete response, partial response, stable disease, and progressive disease were observed in 25, five, three, and three patients, respectively. The objective response rate was 83.3%. The 1-year, 2-year, and 3-year progression-free survival rates were 79.8%, 58.9%, and 54.4%, respectively. The 1-year, 2-year, and 3-year overall survival rates were 83.3%, 68.1%, and 58.4%, respectively.

Conclusion: Selecting the chemotherapy drug according to the oncogene expression, combined with radiation therapy, could increase the 3-year survival rate in nonsurgical esophageal squamous cell carcinoma patients. Such conclusion needs to be further confirmed using a larger sample size.

C1 [Yang, Zhe; Dai, Honghai; Lv, Dongxiao; Feng, A. Lei; Shu, Weibin; Han, Junqing] Shandong Univ, Shandong Prov Hosp Affiliated, Tumor Res & Therapy Ctr, 324 Jingwu Weiqi Rd, Jinan 250021, Shandong, Peoples R China.

RP Han, JQ (corresponding author), Shandong Univ, Shandong Prov Hosp Affiliated, Tumor Res & Therapy Ctr, 324 Jingwu Weiqi Rd, Jinan 250021, Shandong, Peoples R China.

EM hanjq1960@126.com

CR Afzal S, 2011, PHARMACOGENOMICS, V12, P1257, DOI [10.2217/PGS.11.83, 10.2217/pgs.11.83]

Bepler G, 2013, J CLIN ONCOL, V31, P2404, DOI 10.1200/JCO.2012.46.9783

Chiu TJ, 2011, J TRANSL MED, V9, DOI 10.1186/1479-5876-9-31

Cooper JS, 1999, JAMA-J AM MED ASSOC, V281, P1623, DOI 10.1001/jama.281.17.1623

Edge S., 2010, AM JOINT COMMITTEE C, VSeventh

Eisenhauer EA, 2009, EUR J CANCER, V45, P228, DOI 10.1016/j.ejca.2008.10.026

Eloubeidi MA, 2002, CANCER-AM CANCER SOC, V95, P1434, DOI 10.1002/cncr.10868

Gong WY, 2012, LUNG CANCER, V75, P374, DOI 10.1016/j.lungcan.2011.08.003

Higuchi K, 2008, RADIOTHER ONCOL, V87, P398, DOI 10.1016/j.radonc.2008.03.006

Ji FZ, 2013, CHIN J GASTROINTEST, V19, P842

Kato K, 2011, INT J RADIAT ONCOL, V81, P684, DOI 10.1016/j.ijrobp.2010.06.033

Kelsey CR, 2007, INT J RADIAT ONCOL, V69, P770, DOI 10.1016/j.ijrobp.2007.03.035

Kim MK, 2008, CLIN CANCER RES, V14, P4225, DOI 10.1158/1078-0432.CCR-07-4848

Kim MK, 2008, EUR J CANCER, V44, P54, DOI 10.1016/j.ejca.2007.09.006

Lee JJ, 2010, LUNG CANCER, V70, P205, DOI 10.1016/j.lungcan.2010.02.005

[李巧巧 LI Qiao-qiao], 2009, [中华放射肿瘤学杂志, Chinese Journal of Radiation Oncology], V18, P379

Minsky BD, 2002, J CLIN ONCOL, V20, P1167, DOI 10.1200/JCO.20.5.1167

Miyazono F, 2004, BRIT J CANCER, V91, P666, DOI 10.1038/sj.bjc.6601976

Pottgen C, 2012, CANCER TREAT REV, V38, P599, DOI 10.1016/j.ctrv.2011.10.005

Sun Q, 2009, JPN J CLIN ONCOL, V39, P237, DOI 10.1093/jjco/hyp008

Tachibana M, 2008, EUR J CARDIO-THORAC, V34, P427, DOI 10.1016/j.ejcts.2008.04.022

Therasse P, 2000, J NATL CANCER I, V92, P205, DOI 10.1093/jnci/92.3.205

[王军 Wang Jun], 2013, [中华放射肿瘤学杂志, Chinese Journal of Radiation Oncology], V22, P446

Wang Y, 2011, ONCOL REP, V25, P1047, DOI 10.3892/or.2011.1170

Warnecke-Eberz U, 2004, CLIN CANCER RES, V10, P3794, DOI 10.1158/1078-0432.CCR-03-0079

Watson RG, 2010, EUR J CANCER, V46, P3358, DOI 10.1016/j.ejca.2010.07.011

Yu JW, 2012, MED ONCOL, V29, P3029, DOI 10.1007/s12032-012-0292-y

Zhang HL, 2012, LUNG CANCER, V77, P9, DOI 10.1016/j.lungcan.2012.01.005

Zhang Ting-rong, 2010, Zhonghua Zhong Liu Za Zhi, V32, P791

NR 29

TC 0

Z9 0

U1 0

U2 1

PU DOVE MEDICAL PRESS LTD

PI ALBANY

PA PO BOX 300-008, ALBANY, AUCKLAND 0752, NEW ZEALAND

SN 1178-6930

J9 ONCOTARGETS THER

JI OncoTargets Ther.

PY 2016

VL 9

BP 4561

EP 4568

DI 10.2147/OTT.S105221

PG 8

WC Biotechnology & Applied Microbiology; Oncology

WE Science Citation Index Expanded (SCI-EXPANDED)

SC Biotechnology & Applied Microbiology; Oncology

GA DR8QR

UT WOS:000380162900001

PM 27524911

OA Green Published, Green Submitted, gold

DA 2022-05-19

ER

PT J

AU Wang, JD

Ma, J

Dong, YC

Shen, Z

Ma, HH

Wang, X

Shi, SS

Wu, JR

Lu, GM

Peng, LB

Zhoud, XJ

AF Wang, Jiandong

Ma, Jie

Dong, Yingchun

Shen, Zhen

Ma, Henghui

Wang, Xuan

Shi, Shanshan

Wu, Jinrong

Lu, Guangming

Peng, Libo

Zhoud, Xiaojun

TI High expression of EphA1 in esophageal squamous cell carcinoma is

associated with lymph node metastasis and advanced disease

SO APMIS

LA English

DT Article

DE EphA1; esophageal squamous cell carcinoma; differentiation; metastasis

ID RECEPTOR TYROSINE KINASE; COLORECTAL-CANCER; DOWN-REGULATION; EPHRIN

LIGANDS; ANGIOGENESIS; SURVIVAL; GENE

AB Wang J, Ma J, Dong Y, Shen Z, Ma H, Wang X, Shi S, Wu J, Lu G, Peng L, Zhoud X. High expression of EphA1 in esophageal squamous cell carcinoma is associated with lymph node metastasis and advanced disease. APMIS 2013; 121: 30-7.

Eph receptors play important roles in the development of cancer. The present study aimed to investigate the expression of EphA1 and its clinicopathologic significance in esophageal squamous cell carcinoma (ESCC). The expression levels of the EphA1 transcript and protein were compared between ESCC and matched normal mucosa in the same patient. High expression levels of the EphA1 transcript and protein were detected in 25.6% (21/82) and 23.2% (19/82) of tumors compared with matched normal mucosa. The up-regulation of EphA1 transcript in tumors was positively associated with differentiation (p = 0.002), disease stage (p = 0.034), and lymph node metastasis (p = 0.042). Increased expression of EphA1 protein in tumors was more often observed in patients with well-differentiated tumors (p = 0.004), lymph node metastasis (p = 0.028), and advanced tumor stage (p = 0.008). EphA1 protein expression was detected in intraepithelial neoplasias as well. Decreased expression of EphA1 protein was detected in 65.2% (15/23) of samples with low-grade neoplasia and in 85.3% (8/15) of samples with high-grade intraepithelial neoplasia. Increased staining of EphA1 protein was not detected in low-grade intraepithelial neoplasia samples, but was detected in 21.3% (2/15) of high-grade intraepithelial neoplasia samples. Taken together, our results show that EphA1 appears to be a differentiation marker for esophageal squamous cells, and its increased expression is positively associated with lymph node metastasis and advanced disease stage.

C1 [Wang, Jiandong; Ma, Jie; Dong, Yingchun; Shen, Zhen; Ma, Henghui; Wang, Xuan; Shi, Shanshan; Wu, Jinrong; Peng, Libo; Zhoud, Xiaojun] Nanjing Univ, Coll Med, Sch Clin, Dept Pathol,Jinling Hosp, Nanjing 210002, Jiangsu, Peoples R China.

[Wang, Jiandong; Lu, Guangming] Nanjing Univ, Coll Med, Sch Clin, Dept Radiol,Jinling Hosp, Nanjing 210002, Jiangsu, Peoples R China.

RP Zhoud, XJ (corresponding author), Nanjing Univ, Coll Med, Sch Clin, Dept Pathol,Nanjing Jinling Hosp, Nanjing 210002, Jiangsu, Peoples R China.

EM nanjing_81@yahoo.com

FU National Natural Science Foundation of China [30970813, 30930028,

81171391]; Ministry of Science and Technology of China [2011CB707702]

FX This work was in part supported by the National Natural Science

Foundation of China (30970813, 30930028, 81171391), National Basic

Research Priorities Program 973 Project (2011CB707702) from the Ministry

of Science and Technology of China.

CR Antonsson A, 2010, CANCER EPIDEM BIOMAR, V19, P2080, DOI 10.1158/1055-9965.EPI-10-0033

Astin JW, 2010, NAT CELL BIOL, V12, P1194, DOI 10.1038/ncb2122

Brantley DM, 2002, ONCOGENE, V21, P7011, DOI 10.1038/sj.onc.1205679

Chen Jin, 2008, Cancer Res, V68, P10031, DOI 10.1158/0008-5472.CAN-08-3010

Dong YC, 2009, MODERN PATHOL, V22, P151, DOI 10.1038/modpathol.2008.188

Enzinger PC, 2003, NEW ENGL J MED, V349, P2241, DOI 10.1056/NEJMra035010

Genander M, 2010, CURR OPIN CELL BIOL, V22, P611, DOI 10.1016/j.ceb.2010.08.005

Hafner C, 2006, MODERN PATHOL, V19, P1369, DOI 10.1038/modpathol.3800660

Herath NI, 2009, BRIT J CANCER, V100, P1095, DOI 10.1038/sj.bjc.6604970

Herath NI, 2010, INT J CANCER, V126, P2003, DOI 10.1002/ijc.25147

HIRAI H, 1987, SCIENCE, V238, P1717, DOI 10.1126/science.2825356

Kano M, 2010, INT J CANCER, V127, P2804, DOI 10.1002/ijc.25284

Owshalimpur D, 1999, MOL CELL PROBE, V13, P169, DOI 10.1006/mcpr.1999.0228

Pasquale EB, 2010, NAT REV CANCER, V10, P165, DOI 10.1038/nrc2806

Vaught D, 2008, BREAST CANCER RES, V10, DOI 10.1186/bcr2207

Wang JD, 2005, ONCOGENE, V24, P5637, DOI 10.1038/sj.onc.1208720

Wang JD, 2007, HUM PATHOL, V38, P1649, DOI 10.1016/j.humpath.2007.01.030

Wang JD, 2010, ONCOL REP, V24, P1577, DOI 10.3892/or_00001020

Wu M, 2011, INT J CANCER, V128, P2147, DOI 10.1002/ijc.25532

Zhang J, 2006, J PATHOL, V208, P453, DOI 10.1002/path.1937

NR 20

TC 13

Z9 17

U1 1

U2 9

PU WILEY-BLACKWELL

PI HOBOKEN

PA 111 RIVER ST, HOBOKEN 07030-5774, NJ USA

SN 0903-4641

J9 APMIS

JI APMIS

PD JAN

PY 2013

VL 121

IS 1

BP 30

EP 37

DI 10.1111/j.1600-0463.2012.02941.x

PG 8

WC Immunology; Microbiology; Pathology

WE Science Citation Index Expanded (SCI-EXPANDED)

SC Immunology; Microbiology; Pathology

GA 074RK

UT WOS:000313830600004

PM 23030051

DA 2022-05-19

ER

PT J

AU Peng, J

Wang, WP

Yuan, Y

Wang, ZQ

Wang, Y

Chen, LQ

AF Peng, Jun

Wang, Wen-Ping

Yuan, Yong

Wang, Zhi-Qiang

Wang, Yun

Chen, Long-Qi

TI Adequate lymphadenectomy in patients with oesophageal squamous cell

carcinoma: resecting the minimal number of lymph node stations

SO EUROPEAN JOURNAL OF CARDIO-THORACIC SURGERY

LA English

DT Article

DE Oesophageal squamous cell carcinoma; Lymphadenectomy; Lymph node

ID PREDICTS SURVIVAL; CANCER; IMPACT; EXTENT; PREVALENCE; PATTERN; SPREAD

AB OBJECTIVES: The seventh edition of the American Joint Committee on Cancer (AJCC) tumour-node-metastasis (TNM) staging system for oesophageal cancer did not define the minimal requirement for an adequate extent of lymphadenectomy in patients with oesophageal cancer. This study aimed to define the minimum number of lymph node (LN) stations to be resected in patients with oesophageal squamous cell carcinoma (OSCC).

METHODS: We conducted a retrospective review of clinicopathological data from 2033 patients with OSCC undergoing complete resection between August 2005 and September 2013. An ordinal logistic regression analysis was used to identify the variables associated with revised N (rN) staging progression and indices that could be used in the stratified analysis. To determine the optimal number of resected LN stations, we compared the overall survival hazard ratio between groups with different numbers of LN stations using a Cox's proportional hazards regression model. The highest chi(2) value was deemed the cut-off point.

RESULTS: The progression of rN staging was influenced by T-staging, G-staging, tumour length (> 3 cm), lymphovascular invasion and number of resected LN stations. According to Cox's proportional hazards regression model, we recommended that at least six LN stations should be removed. Then, we conducted a stratified analysis by G status and tumour length. We found that at least seven LN stations should be removed in patients with G3 or tumour length > 3 cm.

CONCLUSIONS: For thoracic OSCC, at least six LN stations had to be removed. Furthermore, for patients with G3 or tumour length > 3 cm, the recommendation was seven.

C1 [Peng, Jun; Wang, Wen-Ping; Yuan, Yong; Wang, Zhi-Qiang; Wang, Yun; Chen, Long-Qi] Sichuan Univ, West China Hosp, Dept Thorac Surg, 37 Guoxue Alley, Chengdu 610041, Sichuan, Peoples R China.

RP Chen, LQ (corresponding author), Sichuan Univ, West China Hosp, Dept Thorac Surg, 37 Guoxue Alley, Chengdu 610041, Sichuan, Peoples R China.

EM drchenlq@gmail.com

OI Peng, Jun/0000-0001-5732-4899

CR Altorki NK, 2008, ANN SURG, V248, P221, DOI 10.1097/SLA.0b013e31817bbe59

Chen JH, 2014, J GASTROINTEST SURG, V18, P2080, DOI 10.1007/s11605-014-2663-5

Chen JQ, 2009, EUR J CARDIO-THORAC, V36, P480, DOI 10.1016/j.ejcts.2009.03.056

Chen YJ, 2009, J SURG ONCOL, V100, P127, DOI 10.1002/jso.21312

Dutkowski P, 2002, HEPATO-GASTROENTEROL, V49, P176

Edge S., 2010, AM JOINT COMMITTEE C, VSeventh

Gertler R, 2011, ANN SURG, V253, P689, DOI 10.1097/SLA.0b013e31821111b5

Greenstein AJ, 2008, CANCER-AM CANCER SOC, V112, P1239, DOI 10.1002/cncr.23309

Groth SS, 2010, J THORAC CARDIOV SUR, V139, P612, DOI 10.1016/j.jtcvs.2009.07.017

Kang CH, 2007, EUR J CARDIO-THORAC, V31, P154, DOI 10.1016/j.ejcts.2006.10.033

Li B, 2012, J THORAC CARDIOVASC, V144, P85

Li B, 2012, J THORAC CARDIOV SUR, V144, P778, DOI 10.1016/j.jtcvs.2012.07.002

Ma GW, 2014, WORLD J GASTROENTERO, V20, P18022, DOI 10.3748/wjg.v20.i47.18022

Network NCC, NCCN CLIN PRACT GUID

Peng J, 2016, ANN THORAC SURG, V101, P280, DOI 10.1016/j.athoracsur.2015.06.081

Peyre CG, 2008, ANN SURG, V248, P549, DOI 10.1097/SLA.0b013e318188c474

Rice TW, 2013, THORAC SURG CLIN, V23, P461, DOI 10.1016/j.thorsurg.2013.07.004

Rice TW, 2010, ANN SURG ONCOL, V17, P1721, DOI 10.1245/s10434-010-1024-1

Rizk NP, 2010, ANN SURG, V251, P46, DOI 10.1097/SLA.0b013e3181b2f6ee

Schwarz RE, 2007, J GASTROINTEST SURG, V11, P1384, DOI 10.1007/s11605-007-0264-2

Sobin LH., 2009, TNM CLASSIFICATION M, V7

Twine CP, 2009, HISTOPATHOLOGY, V55, P46, DOI 10.1111/j.1365-2559.2009.03332.x

Veeramachaneni NK, 2008, ANN THORAC SURG, V86, P418, DOI 10.1016/j.athoracsur.2008.04.043

Wang LX, 2014, LANCET, V383, P2057, DOI 10.1016/S0140-6736(13)62639-2

Xu QR, 2011, WORLD J SURG, V35, P1303, DOI 10.1007/s00268-011-1015-9

Yang HX, 2010, ANN SURG ONCOL, V17, P1901, DOI 10.1245/s10434-010-0948-9

NR 26

TC 9

Z9 14

U1 0

U2 4

PU OXFORD UNIV PRESS INC

PI CARY

PA JOURNALS DEPT, 2001 EVANS RD, CARY, NC 27513 USA

SN 1010-7940

EI 1873-734X

J9 EUR J CARDIO-THORAC

JI Eur. J. Cardio-Thorac. Surg.

PD MAY

PY 2016

VL 49

IS 5

BP E141

EP E146

DI 10.1093/ejcts/ezw015

PG 6

WC Cardiac & Cardiovascular Systems; Respiratory System; Surgery

WE Science Citation Index Expanded (SCI-EXPANDED)

SC Cardiovascular System & Cardiology; Respiratory System; Surgery

GA DP4XT

UT WOS:000378501300008

PM 26905182

OA Bronze

DA 2022-05-19

ER

PT J

AU Duan, JJ

Deng, T

Ying, GG

Huang, DZ

Zhang, HY

Zhou, LK

Bai, M

Li, HL

Yang, HM

Qu, YJ

Wang, X

Ba, Y

AF Duan, Jingjing

Deng, Ting

Ying, Guoguang

Huang, Dingzhi

Zhang, Haiyang

Zhou, Likun

Bai, Ming

Li, Hongli

Yang, Huimin

Qu, Yanjun

Wang, Xia

Ba, Yi

TI Prognostic nomogram for previously untreated patients with esophageal

squamous cell carcinoma after esophagectomy followed by adjuvant

chemotherapy

SO JAPANESE JOURNAL OF CLINICAL ONCOLOGY

LA English

DT Article

DE esophageal squamous cell carcinoma; nomogram; prognosis; esophagectomy;

adjuvant chemotherapy

ID 1,000 CONSECUTIVE RESECTIONS; CANCER STAGING SYSTEM; HISTOLOGIC TUMOR

TYPE; TNM CLASSIFICATION; POOR-PROGNOSIS; INTRAHEPATIC

CHOLANGIOCARCINOMA; CURRENT MANAGEMENT; RADIATION-THERAPY;

BREAST-CANCER; SINGLE-CENTER

AB The aim of the study was to establish an effective prognostic nomogram for esophageal squamous cell carcinoma after radical esophagectomy followed by adjuvant chemotherapy in those previously untreated patients.

The clinicopathological data from 328 patients who underwent radical esophagectomy followed by adjuvant chemotherapy or not at the Tianjin Medical University Cancer Institute and Hospital between 2006 and 2010 were retrospectively studied. Nomograms which predicted survival of esophageal squamous cell carcinoma were established based on the Cox proportional hazards regression model. To determine its predictive accuracy and discriminatory capacity, the concordance index and calibration curve were calculated after bootstrapping in the internal validation. An external validation of 76 patients in 2011 was prospectively studied at the same institution. To verify the performance of the nomogram, the comparison between the nomogram and Tumor-Node-Metastasis staging system was conducted.

The 5-year overall survival was 43.1% in the primary cohort. Based on multivariate analyses, five independent prognostic variables including gender, tumor length, T stage, N stage and chemotherapy cycles were selected to build the nomograms to predict disease-free survival and overall survival. The concordance index of the nomogram to predict overall survival was 0.71 (95% confidence interval, 0.63-0.79), which was superior to the predictive power of Tumor-Node-Metastasis staging system (0.64) in the primary cohort. Meanwhile, the calibration curve showed good accuracy between predictive and actual overall survival. In the validation cohort, the concordance index (0.77) and calibration plot displayed favorable performances. The other nomogram to predict disease-free survival also performed well.

The prognostic nomogram provided individualized risk estimate of survival in patients after esophagectomy followed by adjuvant chemotherapy.

C1 [Duan, Jingjing; Deng, Ting; Ying, Guoguang; Huang, Dingzhi; Zhang, Haiyang; Zhou, Likun; Bai, Ming; Li, Hongli; Yang, Huimin; Qu, Yanjun; Wang, Xia; Ba, Yi] Tianjin Med Univ Canc Inst & Hosp, Dept Gastrointestinal Oncol, Natl Clin Res Ctr Canc, Tianjin Key Lab Canc Prevent & Therapy, Tianjin, Peoples R China.

RP Ba, Y (corresponding author), Tianjin Med Univ Canc Inst & Hosp, Dept Gastrointestinal Oncol, Huan Huxi Rd, Tianjin 300060, Peoples R China.

EM bayi@tjmuch.com

FU National Natural Science Foundation of China [81201946, 81372394,

30772484, 81401257, 81171120]; National Research Platform of Clinical

Evaluation Technology for New Anticancer Drugs [2013ZX09303001]

FX This work was supported by grants from the National Natural Science

Foundation of China (Nos. 81201946, 81372394, 30772484, 81401257,

81171120) and National Research Platform of Clinical Evaluation

Technology for New Anticancer Drugs (No. 2013ZX09303001).

CR Ando N, 2003, J CLIN ONCOL, V21, P4592, DOI 10.1200/JCO.2003.12.095

Ando N, 2000, ANN SURG, V232, P225, DOI 10.1097/00000658-200008000-00013

Ando N, 2012, ANN SURG ONCOL, V19, P68, DOI 10.1245/s10434-011-2049-9

Berry MF, 2014, J THORAC DIS, V6, pS289, DOI 10.3978/j.issn.2072-1439.2014.03.11

Bi X, 2015, ONCOTARGETS THER, V8, P487, DOI 10.2147/OTT.S78903

Bohanes P, 2012, J CLIN ONCOL, V30, P2265, DOI 10.1200/JCO.2011.38.8751

Boutros C, 2015, BRIT J CANCER, V112, P992, DOI 10.1038/bjc.2015.34

Brennan MF, 2001, ANN SURG, V234, P368

Chen JY, 2015, TUMOR BIOL, V36, P6797, DOI 10.1007/s13277-015-3392-4

Chen K, 2015, ANN SURG ONCOL, V22, P3497, DOI 10.1245/s10434-015-4393-7

Chen SB, 2013, J THORAC ONCOL, V8, P495, DOI 10.1097/JTO.0b013e3182829e2c

Cools-Lartigue J, 2015, J GASTROINTEST SURG, V19, P964, DOI 10.1007/s11605-014-2701-3

D'Journo XB, 2014, J THORAC DIS, V6, pS253, DOI 10.3978/j.issn.2072-1439.2014.04.16

Derakhshan MH, 2009, GUT, V58, P16, DOI 10.1136/gut.2008.161331

Eloubeidi MA, 2002, CANCER-AM CANCER SOC, V95, P1434, DOI 10.1002/cncr.10868

Eom BW, 2015, PLOS ONE, V10, DOI 10.1371/journal.pone.0119671

Gao J, 2015, INT J CLIN EXP PATHO, V8, P345

Gaur P, 2010, ANN SURG, V252, P611, DOI 10.1097/SLA.0b013e3181f56419

Geng GJ, 2015, INT J CLIN EXP PATHO, V8, P423

Hamada T, 2014, BRIT J CANCER, V110, P1943, DOI 10.1038/bjc.2014.131

Han Li-Hui, 2015, Asian Pac J Cancer Prev, V16, P2245

Hou X, 2015, ANN SURG ONCOL, V22, P1008, DOI 10.1245/s10434-014-4067-x

Hou X, 2014, ANN SURG ONCOL, V21, P337, DOI 10.1245/s10434-013-3265-2

Huang ZL, 2015, ONCOTARGET, V6, P6850, DOI 10.18632/oncotarget.3190

Hyder O, 2014, JAMA SURG, V149, P432, DOI 10.1001/jamasurg.2013.5168

Lagarde SM, 2007, BRIT J SURG, V94, P1361, DOI 10.1002/bjs.5832

Li M, 2015, HUM PATHOL, V46, P707, DOI 10.1016/j.humpath.2015.01.009

Lin SH, 2015, J GASTROINTEST ONCOL, V6, P45, DOI 10.3978/j.issn.2078-6891.2014.054

Lyu X, 2014, J SURG ONCOL, V110, P864, DOI 10.1002/jso.23716

Markar SR, 2013, DIS ESOPHAGUS, V26, P250, DOI 10.1111/j.1442-2050.2012.01353.x

Pennathur A, 2013, LANCET, V381, P400, DOI 10.1016/S0140-6736(12)60643-6

Rustgi AK, 2014, NEW ENGL J MED, V371, P2499, DOI 10.1056/NEJMra1314530

Shiozaki A, 2005, ONCOL REP, V13, P667

Siewert JR, 2001, ANN SURG, V234, P360, DOI 10.1097/00000658-200109000-00010

Takeno S, 2007, ANN SURG ONCOL, V14, P1431, DOI 10.1245/s10434-006-9286-3

Tan H, 2014, BRIT J CANCER, V110, P2109

TENIERE P, 1991, SURG GYNECOL OBSTET, V173, P123

Tullavardhana T, 2015, ONCOL RES TREAT, V38, P110, DOI 10.1159/000380776

Wang J, 2014, WORLD J GASTROENTERO, V20, P18397, DOI 10.3748/wjg.v20.i48.18397

Wang JB, 2015, WORLD J SURG ONCOL, V13, DOI 10.1186/s12957-015-0543-4

Wang QM, 2011, MED ONCOL, V28, P188, DOI 10.1007/s12032-010-9457-8

Wang YZ, 2013, J CLIN ONCOL, V31, P1188, DOI 10.1200/JCO.2012.41.5984

Wu J, 2014, ANN THORAC SURG, V97, P446, DOI 10.1016/j.athoracsur.2013.10.043

Xiao ZF, 2003, ANN THORAC SURG, V75, P331, DOI 10.1016/S0003-4975(02)04401-6

Yamasaki M, 2014, ANN SURG ONCOL, V21, P2850, DOI 10.1245/s10434-014-3696-4

Yang Y, 2015, LEUKEMIA, V29, P1571, DOI 10.1038/leu.2015.44

Zhang L, 2015, AM J HUM GENET, V96, P597, DOI 10.1016/j.ajhg.2015.02.017

NR 47

TC 16

Z9 19

U1 1

U2 6

PU OXFORD UNIV PRESS

PI OXFORD

PA GREAT CLARENDON ST, OXFORD OX2 6DP, ENGLAND

SN 0368-2811

EI 1465-3621

J9 JPN J CLIN ONCOL

JI Jpn. J. Clin. Oncol.

PD APR

PY 2016

VL 46

IS 4

BP 336

EP 343

DI 10.1093/jjco/hyv206

PG 8

WC Oncology

WE Science Citation Index Expanded (SCI-EXPANDED)

SC Oncology

GA DJ4OW

UT WOS:000374187200008

PM 26819278

OA Green Published, Bronze

DA 2022-05-19

ER

PT J

AU Zakko, L

Lutzke, L

Wang, KK

AF Zakko, Liam

Lutzke, Lori

Wang, Kenneth K.

TI Screening and Preventive Strategies in Esophagogastric Cancer

SO SURGICAL ONCOLOGY CLINICS OF NORTH AMERICA

LA English

DT Article

DE Screening; Prevention; Gastric adenocarcinoma; Barrett esophagus;

Esophageal adenocarcinoma; Esophageal squamous cell carcinoma

ID GASTROESOPHAGEAL-REFLUX DISEASE; BARRETTS-ESOPHAGUS; GASTRIC-CANCER;

RADIOFREQUENCY ABLATION; NEOPLASTIC PROGRESSION; INTESTINAL METAPLASIA;

GRADE DYSPLASIA; FAMILY-HISTORY; COST-UTILITY; RISK-FACTORS

AB Gastric adenocarcinoma, esophageal adenocarcinoma, and esophageal squamous cell carcinoma are among the most prevalent and deadly of malignancies worldwide. Screening and prevention programs will be critical to finally improving outcomes in these diseases. For gastric adenocarcinoma, screening in high-risk populations has significantly reduced mortality. More research is needed on screening high-risk individuals in low-risk populations. For esophageal adenocarcinoma, work is needed to develop efficient and effective techniques in mass screening programs. For most Western populations, current screening is not cost effective. Avoiding environmental risk factors is critical to reducing the incidence of this deadly illness.

C1 [Zakko, Liam; Wang, Kenneth K.] Mayo Clin, Div Gastroenterol & Hepatol, 200 2nd St SW, Rochester, MN 55905 USA.

[Lutzke, Lori] Mayo Clin, Div Gastroenterol & Hepatol, Barretts Esophagus Unit, 200 First St Northwest, Rochester, MN USA.

RP Wang, KK (corresponding author), Mayo Clin, Div Gastroenterol & Hepatol, 200 2nd St SW, Rochester, MN 55905 USA.

EM wang.kenneth@mayo.edu

FU Nine Point Medical; C2 Therapeutic; CSA Medical; Boston Scientific;

Fujinon; National Cancer Institute [U01 CA182940, U54 CA163004];

NATIONAL CANCER INSTITUTE [U01CA182940, U54CA163004] Funding Source: NIH

RePORTER

FX Dr K.K. Wang discloses research support from Nine Point Medical, C2

Therapeutic, CSA Medical, Boston Scientific, and Fujinon. Funding

support provided by National Cancer Institute, U01 CA182940 and U54

CA163004. Drs L. Zakko and L. Lutzke have nothing to disclose.

CR Atkinson M, 2008, AM J GASTROENTEROL, V103, P92, DOI 10.1111/j.1572-0241.2007.01501.x

Auvinen MI, 2002, J CLIN ONCOL, V20, P2971, DOI 10.1200/JCO.2002.09.011

Benaglia T, 2013, GASTROENTEROLOGY, V144, P62, DOI 10.1053/j.gastro.2012.09.060

Bhat S, 2011, J NATL CANCER I, V103, P1049, DOI 10.1093/jnci/djr203

BOLLSCHWEILER E, 1993, CANCER-AM CANCER SOC, V71, P2918, DOI 10.1002/1097-0142(19930515)71:10<2918::AID-CNCR2820711006>3.0.CO;2-V

Bosetti C, 2013, ANN ONCOL, V24, P2657, DOI 10.1093/annonc/mdt301

Brown Linda Morris, 2002, Surg Oncol Clin N Am, V11, P235, DOI 10.1016/S1055-3207(02)00002-9

Camargo MC, 2012, CANCER EPIDEM BIOMAR, V21, P20, DOI 10.1158/1055-9965.EPI-11-0834

Cao Y, 2016, JAMA ONCOL, V2, P762, DOI 10.1001/jamaoncol.2015.6396

Chan DK, 2016, GASTROENTEROLOGY, V150, pS67, DOI 10.1016/S0016-5085(16)30345-6

Chandra S, 2013, CURR GASTROENTEROL R, V15, P32

Choi KS, 2009, CANCER EPIDEM BIOMAR, V18, P1390, DOI 10.1158/1055-9965.EPI-08-0940

Colvin H, 2015, SURG ONCOL CLIN N AM, V24, P765, DOI 10.1016/j.soc.2015.06.002

Cook MB, 2012, GASTROENTEROLOGY, V142, P744, DOI 10.1053/j.gastro.2011.12.049

Corley DA, 2003, GASTROENTEROLOGY, V124, P47, DOI 10.1053/gast.2003.50008

CORREA P, 1992, CANCER RES, V52, P6735

de Vries AC, 2008, GASTROENTEROLOGY, V134, P945, DOI 10.1053/j.gastro.2008.01.071

Derakhshan MH, 2009, GUT, V58, P16, DOI 10.1136/gut.2008.161331

Desai TK, 2012, GUT, V61, P970, DOI 10.1136/gutjnl-2011-300730

Dinis-Ribeiro M, 2004, NEOPLASIA, V6, P449, DOI 10.1593/neo.03505

Dinis-Ribeiro M, 2012, ENDOSCOPY, V44, P74, DOI 10.1055/s-0031-1291491

Arnal MJD, 2015, WORLD J GASTROENTERO, V21, P7933, DOI 10.3748/wjg.v21.i26.7933

Eloubeidi MA, 2001, J CLIN GASTROENTEROL, V33, P306, DOI 10.1097/00004836-200110000-00010

Evans JA, 2006, CLIN GASTROENTEROL H, V4, P38, DOI 10.1016/S1542-3565(05)00746-9

Evans JA, 2015, GASTROINTEST ENDOSC, V82, P1, DOI 10.1016/j.gie.2015.03.1967

Ferlay J., 2013, GLOBOCAN 2012 V10 CA

Ferlay J, 2015, INT J CANCER, V136, pE359, DOI 10.1002/ijc.29210

Ferlay J, 2010, INT J CANCER, V127, P2893, DOI 10.1002/ijc.25516

Fitzgerald RC, 2011, J MED GENET, V48, P216, DOI 10.1136/jmg.2009.074237corr1

Fitzgerald RC, 2014, GUT, V63, P7, DOI 10.1136/gutjnl-2013-305372

FUKAO A, 1995, INT J CANCER, V60, P45, DOI 10.1002/ijc.2910600106

Gammon MD, 2004, CANCER EPIDEM BIOMAR, V13, P34, DOI 10.1158/1055-9965.EPI-03-0198

Ge S, 2012, GASTROENT RES PRACT, V2012, DOI 10.1155/2012/808120

Gharahkhani P, 2016, HUM MOL GENET, V25, P828, DOI 10.1093/hmg/ddv512

Gora MJ, 2013, NAT MED, V19, P238, DOI 10.1038/nm.3052

Gupta N, 2011, GASTROINTEST ENDOSC, V74, P610, DOI 10.1016/j.gie.2011.05.001

Hansford S, 2015, JAMA ONCOL, V1, P23, DOI 10.1001/jamaoncol.2014.168

Hillman LC, 2004, MED J AUSTRALIA, V180, P387, DOI 10.5694/j.1326-5377.2004.tb05991.x

Hosokawa O., 2004, J GASTROENTEROL MASS, V42, P33

Inaba S, 1999, PREV MED, V29, P102

Isobe Y, 2011, GASTRIC CANCER, V14, P301, DOI 10.1007/s10120-011-0085-6

Jang JS, 2013, J GASTRIC CANCER, V13, P247, DOI 10.5230/jgc.2013.13.4.247

Juhasz A, 2011, J CLIN GASTROENTEROL, V45, P867, DOI 10.1097/MCG.0b013e31821f44a8

Kadri SR, 2010, BMJ-BRIT MED J, V341, P4372, DOI DOI 10.1136/BMJ.C4372

Kamangar F, 2005, ANTICANCER RES, V25, P425

Kastelein F, 2013, GUT, V62, P1676, DOI 10.1136/gutjnl-2012-303594

Kastelein F, 2013, CLIN GASTROENTEROL H, V11, P382, DOI 10.1016/j.cgh.2012.11.014

Kato M, 2012, JPN J CLIN ONCOL, V42, P987, DOI 10.1093/jjco/hys151

Kim GH, 2016, GASTROINTEST ENDOSC, V84, P18, DOI 10.1016/j.gie.2016.02.028

Koop H, 2000, ENDOSCOPY, V32, P101

Korean Statistical Information Service, CANC REG STAT 2012

Ladeiras-Lopes R, 2008, CANCER CAUSE CONTROL, V19, P689, DOI 10.1007/s10552-008-9132-y

Lao-Sirieix P, 2012, NAT REV CLIN ONCOL, V9, P278, DOI 10.1038/nrclinonc.2012.35

LAVECCHIA C, 1992, CANCER, V70, P50, DOI 10.1002/1097-0142(19920701)70:1<50::AID-CNCR2820700109>3.0.CO;2-I

Lee CH, 2005, INT J CANCER, V113, P475, DOI 10.1002/ijc.20619

Ley C, 2001, CANCER EPIDEM BIOMAR, V10, P107

Lin YS, 2013, J EPIDEMIOL, V23, P233, DOI 10.2188/jea.JE20120162

Liu KSH, 2016, WORLD J GASTROENTERO, V22, P1311, DOI 10.3748/wjg.v22.i3.1311

Lui FH, 2014, DIGEST DIS SCI, V59, P3027, DOI 10.1007/s10620-014-3275-3

Lunet N, 2007, EUR J CANCER PREV, V16, P312, DOI 10.1097/01.cej.0000236255.95769.22

Maskarinec G, 2004, ETHNIC DIS, V14, P431

Mlombe YB, 2015, MALAWI MED J, V27, P88, DOI 10.4314/mmj.v27i3.3

Nguyen DM, 2009, CLIN GASTROENTEROL H, V7, P1299, DOI 10.1016/j.cgh.2009.06.001

OTSUJI M, 1989, Stomach and Intestine (Tokyo), V24, P1291

PALLI D, 1994, CANCER EPIDEM BIOMAR, V3, P15

Pech O, 2011, ANN SURG, V254, P67, DOI 10.1097/SLA.0b013e31821d4bf6

Phoa KN, 2014, JAMA-J AM MED ASSOC, V311, P1209, DOI 10.1001/jama.2014.2511

Pohl H, 2010, CANCER EPIDEM BIOMAR, V19, P1468, DOI 10.1158/1055-9965.EPI-10-0012

Prasad GA, 2009, GASTROENTEROLOGY, V137, P815, DOI 10.1053/j.gastro.2009.05.059

Qumseya BJ, 2013, CLIN GASTROENTEROL H, V11, P1562, DOI 10.1016/j.cgh.2013.06.017

Reid BJ, 2015, GASTROENTEROLOGY, V149, P1142, DOI 10.1053/j.gastro.2015.07.010

Rubenstein JH, 2007, CLIN GASTROENTEROL H, V5, P312, DOI 10.1016/j.cgh.2006.12.008

Rubenstein JH, 2015, GASTROENTEROLOGY, V149, P302, DOI 10.1053/j.gastro.2015.04.053

Rubenstein JH, 2013, AM J GASTROENTEROL, V108, P353, DOI 10.1038/ajg.2012.446

Sami SS, 2015, CLIN GASTROENTEROL H, V13, P623, DOI 10.1016/j.cgh.2014.03.036

Sami SS, 2015, AM J GASTROENTEROL, V110, P148, DOI 10.1038/ajg.2014.362

Sami SS, 2013, SYSTEMATIC REV METAA, VUEG13-ABS-2547

Sauk J, 2013, DIGEST DIS SCI, V58, P2261, DOI 10.1007/s10620-013-2625-x

Shaheen NJ, 2016, AM J GASTROENTEROL, V111, P30, DOI 10.1038/ajg.2015.322

Shaheen NJ, 2009, NEW ENGL J MED, V360, P2277, DOI 10.1056/NEJMoa0808145

Sharma P, 2011, GASTROINTEST ENDOSC, V74, P465, DOI 10.1016/j.gie.2011.04.004

Sheh A, 2011, CANCER PREV RES, V4, P1426, DOI 10.1158/1940-6207.CAPR-11-0219

Shin Cheol Min, 2010, J Clin Gastroenterol, V44, pe34, DOI 10.1097/MCG.0b013e3181a159c4

Siegel RL, 2015, CA-CANCER J CLIN, V65, P5, DOI [10.3322/caac.21254, 10.1136/bmj.g1502, 10.1001/jamaoto.2014.2530, 10.3322/caac.21208]

Singh S, 2014, GUT, V63, P1229, DOI 10.1136/gutjnl-2013-305997

Thrift AP, 2014, CLIN GASTROENTEROL H, V12, P1267, DOI 10.1016/j.cgh.2013.12.014

Thrift AP, 2012, CANCER PREV RES, V5, P1115, DOI 10.1158/1940-6207.CAPR-12-0010

Wheeler JB, 2012, SURG CLIN N AM, V92, P1077, DOI 10.1016/j.suc.2012.07.008

Wong A, 2005, CLIN GASTROENTEROL H, V3, P1, DOI 10.1016/S1542-3565(04)00602-0

Yang J, 2012, WORLD J GASTROENTERO, V18, P2493, DOI 10.3748/wjg.v18.i20.2493

Yang P, 2009, EUR J CANCER, V45, P2867, DOI 10.1016/j.ejca.2009.04.019

Yang X, 2015, GENE, V556, P149, DOI 10.1016/j.gene.2014.11.049

Yeh JM, 2016, GUT, V65, P563, DOI 10.1136/gutjnl-2014-308588

Yoshihara M, 2007, SCAND J GASTROENTERO, V42, P760, DOI 10.1080/00365520601097351

Zhang HZ, 2012, CHIN J CANCER, V31, P281, DOI 10.5732/cjc.011.10390

NR 95

TC 10

Z9 11

U1 1

U2 6

PU W B SAUNDERS CO-ELSEVIER INC

PI PHILADELPHIA

PA 1600 JOHN F KENNEDY BOULEVARD, STE 1800, PHILADELPHIA, PA 19103-2899 USA

SN 1055-3207

EI 1558-5042

J9 SURG ONCOL CLIN N AM

JI Surg. Oncol. Clin. N. Am.

PD APR

PY 2017

VL 26

IS 2

BP 163

EP +

DI 10.1016/j.soc.2016.10.004

PG 17

WC Oncology; Surgery

WE Science Citation Index Expanded (SCI-EXPANDED)

SC Oncology; Surgery

GA EQ5PY

UT WOS:000398136300003

PM 28279462

OA Green Accepted

DA 2022-05-19

ER

PT J

AU Alsop, BR

Sharma, P

AF Alsop, Benjamin R.

Sharma, Prateek

TI Esophageal Cancer

SO GASTROENTEROLOGY CLINICS OF NORTH AMERICA

LA English

DT Article

DE Esophagus cancer; Esophageal squamous cell carcinoma; Esophageal

adenocarcinoma; Barrett's esophagus

ID HIGH-GRADE DYSPLASIA; SQUAMOUS-CELL CARCINOMA; SUCCESSFUL RADIOFREQUENCY

ABLATION; BARRETTS-ESOPHAGUS; UNITED-STATES; ENDOSCOPIC ULTRASOUND;

CONTROLLED-TRIAL; ADENOCARCINOMA; RISK; MANAGEMENT

AB Esophageal cancer carries a poor prognosis among gastrointestinal malignancies. Although esophageal squamous cell carcinoma predominates worldwide, Western nations have seen a marked rise in the incidence of esophageal adenocarcinoma that parallels the obesity epidemic. Efforts directed toward early detection have been difficult, given that dysplasia and early cancer are generally asymptomatic. However, significant advances have been made in the past 10 to 15 years that allow for endoscopic management and often cure in early stage esophageal malignancy. New diagnostic imaging technologies may provide a means by which cost-effective, early diagnosis of dysplasia allows for definitive therapy and ultimately improves the overall survival among patients.

C1 [Alsop, Benjamin R.; Sharma, Prateek] Kansas City VA Med Ctr, Dept Gastroenterol & Hepatol, 4801 Linwood Blvd, Kansas City, MO 64128 USA.

[Alsop, Benjamin R.; Sharma, Prateek] Univ Kansas, Med Ctr, Dept Gastroenterol & Hepatol, 3901 Rainbow Blvd,Mailstop 1023, Kansas City, KS 66160 USA.

RP Alsop, BR (corresponding author), Kansas City VA Med Ctr, Dept Gastroenterol & Hepatol, 4801 Linwood Blvd, Kansas City, MO 64128 USA.

EM benjamin.alsop@va.gov

CR Anaparthy R, 2013, CLIN GASTROENTEROL H, V11, P1430, DOI 10.1016/j.cgh.2013.05.007

Ardalan B, 2007, JPN J CLIN ONCOL, V37, P590, DOI 10.1093/jjco/hym076

Arnold M, 2015, GUT, V64, P381, DOI 10.1136/gutjnl-2014-308124

BARRETT NR, 1950, BRIT J SURG, V38, P175, DOI 10.1002/bjs.18003815005

Bruzzi JF, 2007, RADIOGRAPHICS, V27, P1635, DOI 10.1148/rg.276065742

Chou I, 2004, J CLIN ONCOL, V22, P2395, DOI 10.1200/JCO.2004.08.154

Cunningham D, 2008, NEW ENGL J MED, V358, P36, DOI 10.1056/NEJMoa073149

Dunbar KB, 2012, AM J GASTROENTEROL, V107, P850, DOI 10.1038/ajg.2012.78

El-Serag HB, 2007, GUT, V56, P749, DOI 10.1136/gut.2006.100263

ELLIS A, 1994, ORAL ONCOL, V30B, P102

Engel LS, 2003, J NATL CANCER I, V95, P1404, DOI 10.1093/jnci/djg047

Enzinger PC, 2003, NEW ENGL J MED, V349, P2241, DOI 10.1056/NEJMra035010

Evans JA, 2012, GASTROINTEST ENDOSC, V76, P1087, DOI 10.1016/j.gie.2012.08.004

Fitzmaurice C, 2015, JAMA ONCOL, V1, P505, DOI 10.1001/jamaoncol.2015.0735

GRAHAM DY, 1982, GASTROENTEROLOGY, V82, P228

Hayeck TJ, 2010, DIS ESOPHAGUS, V23, P451, DOI 10.1111/j.1442-2050.2010.01054.x

Hur C, 2013, CANCER-AM CANCER SOC, V119, P1149, DOI 10.1002/cncr.27834

Hvid-Jensen F, 2011, NEW ENGL J MED, V365, P1375, DOI 10.1056/NEJMoa1103042

Jain Rohit, 2010, J Gastrointest Cancer, V41, P93, DOI 10.1007/s12029-009-9121-8

Johanson JF, 2011, DIGEST DIS SCI, V56, P767, DOI 10.1007/s10620-010-1497-6

Kay M, 2009, CURR OPIN PEDIATR, V21, P651, DOI 10.1097/MOP.0b013e32832e2764

KNYRIM K, 1993, NEW ENGL J MED, V329, P1302, DOI 10.1056/NEJM199310283291803

Kroep S, 2015, GASTROENTEROLOGY, V149, P577, DOI 10.1053/j.gastro.2015.04.045

Larghi A, 2005, GASTROINTEST ENDOSC, V62, P16, DOI 10.1016/S0016-5107(05)00319-6

Leeuwenburgh I, 2010, AM J GASTROENTEROL, V105, P2144, DOI 10.1038/ajg.2010.263

Manner H, 2008, AM J GASTROENTEROL, V103, P2589, DOI 10.1111/j.1572-0241.2008.02083.x

Muto M, 2010, J CLIN ONCOL, V28, P1566, DOI 10.1200/JCO.2009.25.4680

Napier KJ, 2014, WORLD J GASTRO ONCOL, V6, P112, DOI 10.4251/wjgo.v6.i5.112

Network NCC, ES ES JUNCT CANC VER

Njei B, 2016, J GASTROEN HEPATOL, V31, P1141, DOI 10.1111/jgh.13289

Oppedijk V, 2014, J CLIN ONCOL, V32, P385, DOI 10.1200/JCO.2013.51.2186

Pasricha S, 2014, CLIN GASTROENTEROL H, V12, P1840, DOI 10.1016/j.cgh.2014.04.034

Pech O, 2014, GASTROENTEROLOGY, V146, P652, DOI 10.1053/j.gastro.2013.11.006

Pech O, 2011, ANN SURG, V254, P67, DOI 10.1097/SLA.0b013e31821d4bf6

Peery AF, 2015, GASTROENTEROLOGY, V149, P1731, DOI 10.1053/j.gastro.2015.08.045

Prabhu A, 2014, AM J GASTROENTEROL, V109, P821, DOI 10.1038/ajg.2014.71

Puli SR, 2008, WORLD J GASTROENTERO, V14, P1479, DOI 10.3748/wjg.14.1479

Rastogi A, 2008, GASTROINTEST ENDOSC, V67, P394, DOI 10.1016/j.gie.2007.07.019

Reid BJ, 2000, AM J GASTROENTEROL, V95, P3089, DOI 10.1111/j.1572-0241.2000.03182.x

Rubenstein JH, 2015, GASTROENTEROLOGY, V149, P302, DOI 10.1053/j.gastro.2015.04.053

Ryan AM, 2008, ANN SURG, V247, P909, DOI 10.1097/SLA.0b013e3181612cac

Seiwert TY, 2007, NAT CLIN PRACT ONCOL, V4, P86, DOI 10.1038/ncponc0714

Shaheen NJ, 2016, AM J GASTROENTEROL, V111, P30, DOI 10.1038/ajg.2015.322

Shaheen NJ, 2009, NEW ENGL J MED, V360, P2277, DOI 10.1056/NEJMoa0808145

Sharma P, 2006, GASTROENTEROLOGY, V131, P1392, DOI 10.1053/j.gastro.2006.08.032

Sharma P, 2016, GASTROENTEROLOGY, V150, P591, DOI 10.1053/j.gastro.2015.11.037

Singh S, 2014, GASTROINTEST ENDOSC, V79, P897, DOI 10.1016/j.gie.2014.01.009

Souza RF, 2008, AM J PHYSIOL-GASTR L, V295, pG211, DOI 10.1152/ajpgi.90250.2008

Spechler SJ, 2011, GASTROENTEROLOGY, V140, P1084, DOI 10.1053/j.gastro.2011.01.030

Spechler SJ, 2011, GASTROENTEROLOGY, V140, pE18, DOI 10.1053/j.gastro.2011.01.031

Stahl M, 2005, J CLIN ONCOL, V23, P2310, DOI 10.1200/JCO.2005.00.034

Stevens HP, 1996, ARCH DERMATOL, V132, P640, DOI 10.1001/archderm.132.6.640

Tileston W, 1906, AM J MED SCI, V132, P240, DOI 10.1097/00000441-190608000-00009

Titi M, 2012, GASTROENTEROLOGY, V143, P564, DOI 10.1053/j.gastro.2012.04.051

Trivers KF, 2008, INT J CANCER, V123, P1422, DOI 10.1002/ijc.23691

Tsujii Y, 2015, ENDOSCOPY, V47, P775, DOI 10.1055/s-0034-1391844

Vennalaganti PR, 2015, AM J GASTROENTEROL, V110, P1257, DOI 10.1038/ajg.2015.116

Wang KK, 2015, UNITED EUR GASTROENT, V3, P230, DOI 10.1177/2050640614566066

Wani S, 2011, GASTROENTEROLOGY, V141, P1179, DOI 10.1053/j.gastro.2011.06.055

Westhoff B, 2005, GASTROINTEST ENDOSC, V61, P226, DOI 10.1016/S0016-5107(04)02589-1

Wolfsen HC, 2015, GASTROINTEST ENDOSC, V82, P631, DOI 10.1016/j.gie.2015.03.1968

Wu AH, 2001, CANCER CAUSE CONTROL, V12, P721, DOI 10.1023/A:1011290704728

Young PE, 2010, CLIN GASTROENTEROL H, V8, P1037, DOI 10.1016/j.cgh.2010.08.020

NR 63

TC 96

Z9 98

U1 2

U2 25

PU W B SAUNDERS CO-ELSEVIER INC

PI PHILADELPHIA

PA 1600 JOHN F KENNEDY BOULEVARD, STE 1800, PHILADELPHIA, PA 19103-2899 USA

SN 0889-8553

EI 1558-1942

J9 GASTROENTEROL CLIN N

JI Gastroenterol. Clin. North Am.

PD SEP

PY 2016

VL 45

IS 3

BP 399

EP +

DI 10.1016/j.gtc.2016.04.001

PG 15

WC Gastroenterology & Hepatology

WE Science Citation Index Expanded (SCI-EXPANDED)

SC Gastroenterology & Hepatology

GA DW0BE

UT WOS:000383305100002

PM 27546839

DA 2022-05-19

ER

PT J

AU Chao, YK

Chen, HS

Wang, BY

Hsu, PK

Liu, CC

Wu, SC

AF Chao, Yin-Kai

Chen, Hui-Shan

Wang, Bing-Yen

Hsu, Po-Kuei

Liu, Chia-Chuan

Wu, Shiao-Chi

TI Prognosis of Patients With Pathologic T0 N+ Esophageal Squamous Cell

Carcinoma After Chemoradiotherapy and Surgical Resection: Results From a

Nationwide Study

SO ANNALS OF THORACIC SURGERY

LA English

DT Article

ID COMPLETE CLINICAL-RESPONSE; NEOADJUVANT CHEMORADIOTHERAPY; CANCER;

SURVIVAL; CHEMORADIATION; SURGERY; IMPROVEMENT; THERAPY; SYSTEM

AB Background. Few data are available on the survival outcomes of patients with esophageal squamous cell carcinoma who achieve complete response at the primary site but have residual nodal metastases after chemoradiotherapy. We sought to assess the survival of esophageal squamous cell carcinoma patients with ypT0 N+ disease.

Methods. Esophageal squamous cell carcinoma patients treated with chemoradiotherapy and esophagectomy were identified from the Taiwan Cancer Registry between 2008 and 2013. We compared the clinical and survival data of ypT0 N+ and ypT0 N0 patients. The median number of dissected nodes (n = 20) was used as the cutoff to classify the extent of lymph node dissection (LND). Survival data were analyzed with the Kaplan-Meier method and Cox proportional hazards regression models.

Results. The study included 369 ypT0 patients (50 ypT0 N+ [13.6%] and 319 ypT0 N0 [86.4%]). The 3-year overall survival was significantly lower in ypT0 N+ patients (30.1%) than in ypT0 N0 patients (55.9%, p < 0.001). Multivariate analysis showed that a higher number of positive lymph nodes (ypN2/N3 vs ypN1) was a strong adverse prognostic factor (hazard ratio, 3.76; p = 0.011) in ypT0 N+ patients. The extent of LND was identified as an independent predictor of survival in patients with ypT0 N0 disease (low vs high; hazard ratio, 1.49; p = 0.045). A stepwise decrease in 3-year overall survival rates was observed in the following groups: ypT0 N0 with high LND (61.2%), ypT0 N0 with low LND (50.3%), and ypT0 N+ (30.1%, p < 0.001).

Conclusions. At least 13.6% of ypT0 patients have lymph node metastases, which carry adverse prognostic implications. The number of positive nodes is the most important prognostic factor in this group. (C) 2016 by The Society of Thoracic Surgeons

C1 [Chao, Yin-Kai] Chang Gung Univ, Chang Gung Mem Hosp, Coll Med, Div Thorac Surg, Taoyuan, Taiwan.

[Chen, Hui-Shan; Wu, Shiao-Chi] Natl Yang Ming Univ, Inst Hlth & Welf Policy, Taipei 112, Taiwan.

[Wang, Bing-Yen] Chung Shan Med Univ, Changhua Christian Hosp, Dept Surg, Div Thorac Surg, Taichung, Taiwan.

[Wang, Bing-Yen] Chung Shan Med Univ, Inst Med, Taichung, Taiwan.

[Hsu, Po-Kuei] Taipei Vet Gen Hosp, Dept Surg, Div Thorac Surg, Taipei, Taiwan.

[Hsu, Po-Kuei] Natl Yang Ming Univ, Sch Med, Taipei 112, Taiwan.

[Liu, Chia-Chuan] Koo Fdn Sun Yat Sen Canc Ctr, Dept Surg, Div Thorac Surg, 125 Lih Der Rd, Taipei, Taiwan.

RP Liu, CC (corresponding author), Koo Fdn Sun Yat Sen Canc Ctr, Dept Surg, Div Thorac Surg, 125 Lih Der Rd, Taipei, Taiwan.

EM gcliu@kfsyscc.org

RI Chao, YinKai/ABE-7827-2020

OI Chao, Yin Kai/0000-0001-8649-8783

CR Cavallin F, 2015, ANN SURG, V262, pE101, DOI 10.1097/SLA.0000000000000666

Chao YK, 2015, MEDICINE, V94, DOI 10.1097/MD.0000000000000588

Chiang CJ, 2015, JPN J CLIN ONCOL, V45, P291, DOI 10.1093/jjco/hyu211

Cho HJ, 2012, ANN SURG ONCOL, V22, P1

Donahue JM, 2009, ANN THORAC SURG, V87, P392, DOI 10.1016/j.athoracsur.2008.11.001

Fyles AW, 1998, RADIOTHER ONCOL, V48, P149, DOI 10.1016/S0167-8140(98)00044-9

Kim JY, 2012, SEMIN THORAC CARDIOV, V24, P288, DOI 10.1053/j.semtcvs.2012.10.005

Kim MP, 2010, ANN THORAC SURG, V90, P884, DOI 10.1016/j.athoracsur.2010.03.116

Law S, 2003, ANN SURG, V238, P339, DOI 10.1097/01.sla.0000086545.45918.ee

MANDARD AM, 1994, CANCER, V73, P2680, DOI 10.1002/1097-0142(19940601)73:11<2680::AID-CNCR2820731105>3.0.CO;2-C

Meredith KL, 2010, ANN SURG ONCOL, V17, P1159, DOI 10.1245/s10434-009-0862-1

Piessen G, 2013, ANN SURG, V258, P793, DOI 10.1097/SLA.0000000000000228

Sjoquist KM, 2011, LANCET ONCOL, V12, P681, DOI 10.1016/S1470-2045(11)70142-5

Swisher SG, 2005, ANN SURG, V241, P810, DOI 10.1097/01.sla.0000161983.82345.85

Vallbohmer D, 2010, ANN SURG, V252, P744, DOI 10.1097/SLA.0b013e3181fb8dde

NR 15

TC 9

Z9 9

U1 0

U2 7

PU ELSEVIER SCIENCE INC

PI NEW YORK

PA 360 PARK AVE SOUTH, NEW YORK, NY 10010-1710 USA

SN 0003-4975

EI 1552-6259

J9 ANN THORAC SURG

JI Ann. Thorac. Surg.

PD MAY

PY 2016

VL 101

IS 5

BP 1897

EP 1902

DI 10.1016/j.athoracsur.2015.11.052

PG 6

WC Cardiac & Cardiovascular Systems; Respiratory System; Surgery

WE Science Citation Index Expanded (SCI-EXPANDED)

SC Cardiovascular System & Cardiology; Respiratory System; Surgery

GA DL8CY

UT WOS:000375868500055

PM 26912307

OA Bronze

DA 2022-05-19

ER

PT J

AU Chen, W

Zhao, L

Agoston, A

White, A

Mazzola, E

Boyle, PJ

Deshpande, V

Hornick, JL

Bueno, R

Bass, AJ

Enzinger, P

Mamon, H

Redston, M

Patil, DT

AF Chen, Wei

Zhao, Lei

Agoston, Agoston

White, Abby

Mazzola, Emanuele

Boyle, Patrick J.

Deshpande, Vikram

Hornick, Jason L.

Bueno, Raphael

Bass, Adam J.

Enzinger, Peter

Mamon, Harvey

Redston, Mark

Patil, Deepa T.

TI Florid Foreign Body-type Giant Cell Response to Keratin Is Associated

With Improved Overall Survival in Patients Receiving Preoperative

Therapy for Esophageal Squamous Cell Carcinoma

SO AMERICAN JOURNAL OF SURGICAL PATHOLOGY

LA English

DT Article

DE esophageal squamous cell carcinoma; keratin; giant cells; neoadjuvant

ID NEOADJUVANT RADIOCHEMOTHERAPY; TUMOR-REGRESSION; CANCER;

CHEMORADIOTHERAPY; CHEMORADIATION; CHEMOTHERAPY; GRANULOMAS

AB While most resection specimens from patients with neoadjuvantly treated esophageal squamous cell carcinoma show therapy-related changes in the form of inflammation and fibrosis, others harbor a florid foreign body-type giant cell response to keratin debris. The purpose of our study was to perform a detailed clinicopathologic analysis of these histologic types of treatment responses and correlate these findings with patient outcome. Clinical and pathologic parameters from 110 esophagogastrectomies were recorded and analyzed. Two main types of histologic responses were observed: inflammatory-predominant response (59%) and florid foreign body-type giant cell response to keratin (41%). Irrespective of cG, cTNM, and amount of residual cancer, florid foreign body-type giant cell reaction was predominantly noted deep within the esophageal wall, while the inflammatory response was restricted to the mucosa, submucosa, and inner half of muscularis propria. Patients with foreign body-type giant cell response showed significantly better overall survival compared with the inflammatory response group (log-rank test P=0.015). Florid foreign body-type giant cell response was the only factor associated with improved survival in a multivariable analysis for overall survival (hazard ratio=0.5; 95% confidence interval=0.3-1.0; P=0.038), but not in the model for disease-specific survival, whereas ypTNM stage II was the only significant risk factor for disease-specific survival in multivariable analysis (hazard ratio=3.4; 95% confidence interval=1.0-11.2; P=0.047). Our results suggest that in addition to the College of American Pathologists Tumor Regression Score and ypTNM stage, subtype of histologic response to therapy may represent another prognostic marker for neoadjuvantly treated esophageal squamous cell carcinoma.

C1 [Chen, Wei; Zhao, Lei; Agoston, Agoston; Hornick, Jason L.; Redston, Mark; Patil, Deepa T.] Brigham & Womens Hosp, Dept Pathol, 75 Francis St, Boston, MA 02115 USA.

[White, Abby; Bueno, Raphael] Brigham & Womens Hosp, Dept Thorac Surg, 75 Francis St, Boston, MA 02115 USA.

[Mazzola, Emanuele] Dana Farber Canc Inst, Dept Data Sci, Boston, MA 02115 USA.

[Bass, Adam J.; Enzinger, Peter] Dana Farber Canc Inst, Dept ofMedicine, Boston, MA 02115 USA.

[Boyle, Patrick J.; Mamon, Harvey] Brigham & Womens Hosp, Dana Farber Canc Inst, Dept Radiat Oncol, 75 Francis St, Boston, MA 02115 USA.

[Deshpande, Vikram] Massachusetts Gen Hosp, Dept Pathol, Boston, MA 02114 USA.

RP Patil, DT (corresponding author), Harvard Med Sch, Dept Pathol, 75 Francis St, Boston, MA 02115 USA.

EM chenw10@upmc.edu; lzhao19@bwh.harvard.edu; aagoston@bwh.harvard.edu;

awhite12@bwh.harvard.edu; mazzola@jimmy.harvard.edu;

pjboyle@dfci.harvard.edu; VDESHPANDE@mgh.harvard.edu;

jhornick@bwh.harvard.edu; rbueno@bwh.harvard.edu;

Adam_Bass@dfci.harvard.edu; Peter_Enzinger@dfci.harvard.edu;

HMAMON@BWH.HARVARD.EDU; MREDSTON@PARTNERS.ORG; dtpatil@bwh.harvard.edu

CR Brown LM, 2001, AM J EPIDEMIOL, V153, P114, DOI 10.1093/aje/153.2.114

Brucher BLDM, 2004, EJSO-EUR J SURG ONC, V30, P963, DOI 10.1016/S0748-7983(04)00149-0

Chang F, 2008, AM J CLIN PATHOL, V129, P252, DOI 10.1309/CCR3QN4874YJDJJ7

Chirieac LR, 2005, CANCER-AM CANCER SOC, V103, P1347, DOI 10.1002/cncr.20916

College of American Pathologists (CAP), TEMPL

Holscher AH, 2014, ANN SURG, V260, P779, DOI 10.1097/SLA.0000000000000964

Malaisrie SC, 2006, CANCER-AM CANCER SOC, V107, P967, DOI 10.1002/cncr.22077

Malhotra GK, 2017, J SURG ONCOL, V115, P564, DOI 10.1002/jso.24592

MANDARD AM, 1994, CANCER, V73, P2680, DOI 10.1002/1097-0142(19940601)73:11<2680::AID-CNCR2820731105>3.0.CO;2-C

National Comprehensive Cancer Network (NCCN), 2021, ES ES JUNCT CANC

Rice TW., 2017, AJCC CANC STAGING MA, V8th, P185

Rustgi AK, 2014, NEW ENGL J MED, V371, P2499, DOI 10.1056/NEJMra1314530

SAFAII H, 1966, CANCER RES, V26, P500

Schneider PM, 2005, ANN SURG, V242, P684, DOI 10.1097/01.sla.0000186170.38348.7b

Shapiro J, 2013, ANN SURG, V258, P678, DOI 10.1097/SLA.0b013e3182a6191d

Sjoquist KM, 2011, LANCET ONCOL, V12, P681, DOI 10.1016/S1470-2045(11)70142-5

Swisher SG, 2005, ANN SURG, V241, P810, DOI 10.1097/01.sla.0000161983.82345.85

Torre LA, 2015, CA-CANCER J CLIN, V65, P87, DOI 10.3322/caac.21262

Van Der Horst C, 2008, INT J GYNECOL CANCER, V18, P549, DOI 10.1111/j.1525-1438.2007.01033.x

van Rossum PSN, 2013, EUR RADIOL, V23, P1753, DOI 10.1007/s00330-013-2773-6

Wu TT, 2007, AM J SURG PATHOL, V31, P58, DOI 10.1097/01.pas.0000213312.36306.cc

Yamamoto M, 2000, DIS ESOPHAGUS, V13, P293, DOI 10.1046/j.1442-2050.2000.00136.x

Yang H, 2018, J CLIN ONCOL, V36, P2796, DOI 10.1200/JCO.2018.79.1483

NR 23

TC 0

Z9 0

U1 0

U2 0

PU LIPPINCOTT WILLIAMS & WILKINS

PI PHILADELPHIA

PA TWO COMMERCE SQ, 2001 MARKET ST, PHILADELPHIA, PA 19103 USA

SN 0147-5185

EI 1532-0979

J9 AM J SURG PATHOL

JI Am. J. Surg. Pathol.

PD DEC

PY 2021

VL 45

IS 12

BP 1648

EP 1660

DI 10.1097/PAS.0000000000001797

PG 13

WC Pathology; Surgery

WE Science Citation Index Expanded (SCI-EXPANDED)

SC Pathology; Surgery

GA WU5YK

UT WOS:000716620900009

PM 34469333

DA 2022-05-19

ER

PT J

AU Ainiwaer, J

Tuerhong, A

Hasim, A

Du, CS

Zhang, LW

Sheyhidin, I

AF Ainiwaer, Julaiti

Tuerhong, Abuduaini

Hasim, Ayshamgul

Du Chengsong

Zhang Liwei

Sheyhidin, Ilyar

TI Association of the plasma riboflavin levels and riboflavin transporter

(C20orf54) gene statuses in Kazak esophageal squamous cell carcinoma

patients

SO MOLECULAR BIOLOGY REPORTS

LA English

DT Article

DE Esophageal squamous cell carcinoma (ESCC); Riboflavin transporter

(C20orf54) gene; Riboflavin levels; Kazak ethnic

ID HIGH-INCIDENCE AREA; FUNCTIONAL-CHARACTERIZATION; NUTRITION

INTERVENTION; DEFICIENCY; CANCER; ADENOCARCINOMA; RISK; IDENTIFICATION;

CONSUMPTION; LINXIAN

AB To evaluate the association of the plasma riboflavin level in Kazak esophageal cancer patients and their riboflavin transporter (C20orf54) gene statuses. Plasma riboflavin levels were detected by high performance liquid chromatography in Kazak patients with esophageal squamous cell carcinoma (ESCC) and healthy controls. C20orf54 mRNA and protein expression were analyzed by real-time fluorogenic quantitative polymerase chain reaction and immunohistochemistry in samples from 61 ESCC patients consisting of both tumor and normal tissue, respectively. C20orf54 mRNA expression was decreased in ESCC (0.279 +/- A 0.102) than in normal counterpart tissue (0.479 +/- A 0.287; P = 0.049) significantly. Tumors exhibited low C20orf54 protein expression (42.6, 26.2, 18.0 and 13.1 % for no C20orf54 staining, weak staining, medium staining and strong staining, respectively), which was significantly lower than that in the normal mucous membrane (13.1, 26.2, 41.0 and 19.7 % for no C20orf54 staining, weak staining, medium staining and strong staining, respectively). Defective expression of C20orf54 in tumor cells was significantly associated with poor differentiation. However, other parameters such as depth of invasion and lymph node metastasis had no significant relationship with C20orf54 expression. The average blood concentration of riboflavin was 2.6468 +/- A 1.3474 ng/ml in ESCC patients lower than control group (4.2960 +/- A 3.2293 ng/ml, P = 0.015). A positive correlation of plasma riboflavin levels with defective expression of C20orf54 protein was found in ESCC patients (F = 8.626; P = 0.038). Defective expression of C20orf54 is associated with the development of Kazak esophageal squamous cell carcinoma and this may represent a mechanism underlying the decreased plasma riboflavin levels in ESCC.

C1 [Ainiwaer, Julaiti; Tuerhong, Abuduaini; Zhang Liwei; Sheyhidin, Ilyar] Med Univ Xinjiang, Affiliated Hosp 1, Dept Thorac Surg, Urumqi 830054, Xinjiang, Peoples R China.

[Hasim, Ayshamgul; Du Chengsong] Med Univ Xinjiang, Coll Basic Med, Dept Pathol, Urumqi 830011, Xinjiang, Peoples R China.

RP Sheyhidin, I (corresponding author), Med Univ Xinjiang, Affiliated Hosp 1, Dept Thorac Surg, Liyushan Rd 137, Urumqi 830054, Xinjiang, Peoples R China.

EM ilyarsha@hotmail.com

FU State Key Lab of Xinjiang Maior Disease Research Fund

[SKLIB-XJMDR-2012-4]

FX This study was supported by grants from the State Key Lab of Xinjiang

Maior Disease Research Fund (No. SKLIB-XJMDR-2012-4).

CR Abnet CC, 2010, NAT GENET, V42, P764, DOI 10.1038/ng.649

BLOT WJ, 1993, JNCI-J NATL CANCER I, V85, P1483, DOI 10.1093/jnci/85.18.1483

Cheung WY, 2009, GASTROENTEROL CLIN N, V38, P75, DOI 10.1016/j.gtc.2009.01.009

FOY H, 1984, J NATL CANCER I, V72, P941

FOY H, 1972, J NUTR, V102, P571, DOI 10.1093/jn/102.4.571

Fujimura M, 2010, J NUTR, V140, P1722, DOI 10.3945/jn.110.128330

Guohong Zhang, 2010, PLoS One, V5, pe9668, DOI 10.1371/journal.pone.0009668

He YT, 2009, ASIAN PAC J CANCER P, V10, P619

Ho G, 2011, HUM MUTAT, V32, pE1976, DOI 10.1002/humu.21399

KAIRAKBAEV MK, 1978, VOP ONKOL+, V24, P100

Ke L, 2000, INT J CANCER, V102, P271

LIU TP, 1993, CANCER EPIDEM BIOMAR, V2, P525

Manthey KC, 2005, J NUTR, V135, P978, DOI 10.1093/jn/135.5.978

Massey V, 2000, BIOCHEM SOC T, V28, P283, DOI 10.1042/0300-5127:0280283

Morita M, 2010, INT J CLIN ONCOL, V15, P126, DOI 10.1007/s10147-010-0056-7

Murphy SJ, 2010, J NUTR, V140, P1757, DOI 10.3945/jn.110.124362

Pandeya N, 2009, GASTROENTEROLOGY, V136, P1215, DOI 10.1053/j.gastro.2008.12.052

PANGREKAR J, 1993, FOOD CHEM TOXICOL, V31, P745, DOI 10.1016/0278-6915(93)90146-P

Petteys BJ, 2011, CLIN CHIM ACTA, V412, P38, DOI 10.1016/j.cca.2010.08.037

Pfaffl MW, 2001, NUCLEIC ACIDS RES, V29, DOI 10.1093/nar/29.9.e45

Powers HJ, 2003, AM J CLIN NUTR, V77, P1352

Siassi F, 2005, CANCER DETECT PREV, V29, P464, DOI 10.1016/j.cdp.2005.08.001

Wang LD, 2010, NAT GENET, V42, P759, DOI 10.1038/ng.648

Whelan S, 1990, CANCER RES, V102, P14

Ya MZ, 1988, XINJIANG YIXUEYUAN X, V20, P139

Yamamoto S, 2009, J BIOCHEM, V145, P437, DOI 10.1093/jb/mvn181

Yonezawa A, 2008, AM J PHYSIOL-CELL PH, V295, pC632, DOI 10.1152/ajpcell.00019.2008

Zhang WH, 2000, AM J HUM GENET, V67, P110, DOI 10.1086/302970

Zou XN, 2002, INT J VITAM NUTR RES, V72, P375, DOI 10.1024/0300-9831.72.6.375

纪爱芳, 2012, [肿瘤防治研究, Cancer Research on Prevention and Treatment], V39, P467

NR 30

TC 9

Z9 12

U1 0

U2 5

PU SPRINGER

PI DORDRECHT

PA VAN GODEWIJCKSTRAAT 30, 3311 GZ DORDRECHT, NETHERLANDS

SN 0301-4851

EI 1573-4978

J9 MOL BIOL REP

JI Mol. Biol. Rep.

PD MAY

PY 2013

VL 40

IS 5

BP 3769

EP 3775

DI 10.1007/s11033-012-2453-7

PG 7

WC Biochemistry & Molecular Biology

WE Science Citation Index Expanded (SCI-EXPANDED)

SC Biochemistry & Molecular Biology

GA 119CR

UT WOS:000317075300034

PM 23275236

DA 2022-05-19

ER

PT J

AU Lo, CM

Wang, YM

Chen, YH

Fang, FM

Huang, SC

Lu, HI

Li, SH

AF Lo, Chien-Ming

Wang, Yu-Ming

Chen, Yen-Hao

Fang, Fu-Min

Huang, Shun-Chen

Lu, Hung-, I

Li, Shau-Hsuan

TI The Impact of Radiotherapy Dose in Patients with Locally Advanced

Esophageal Squamous Cell Carcinoma Receiving Preoperative

Chemoradiotherapy

SO CURRENT ONCOLOGY

LA English

DT Article

DE esophageal squamous cell carcinoma; chemoradiotherapy; esophagectomy;

radiotherapy dose

ID PHASE-III TRIAL; NEOADJUVANT CHEMORADIATION; SURGERY; CANCER;

CHEMOTHERAPY; SURVIVAL; THERAPY

AB Objective: For patients with esophageal squamous cell carcinoma, preoperative chemoradiotherapy followed by planned esophagectomy is used as a curative treatment modality. However, the impact of radiotherapy dose remains undefined. Method: A total of 141 patients with stage III esophageal squamous cell carcinoma (ESCC; as defined by the 7th American Joint Committee on Cancer), receiving preoperative chemoradiotherapy followed by esophagectomy between 2000 and 2015 at Kaohsiung Chang Gung Memorial Hospital, Taiwan, were retrospectively reviewed. The radiotherapy dose of preoperative chemoradiotherapy (36 Gy before 2009 and 50-50.4 Gy after 2009) and other clinicopathological parameters were collected and correlated with the response to chemoradiotherapy and treatment outcome. Result: Of these 141 patients, the radiotherapy dose was 36 Gy in 59 (42%) patients and 50 Gy in 82 (58%) patients. A complete pathological response was noted in 12 (20%) of 59 patients receiving 36 Gy radiotherapy, and 37 (45%) of 82 patients receiving 50 Gy radiotherapy (p = 0.002). The three-year overall survival and disease-free survival rates were 31% and 25% in patients receiving 36 Gy radiotherapy, and 54% and 46% in patients receiving 50-50.4 Gy radiotherapy, respectively (p = 0.023 for overall survival; p = 0.047 for disease-free survival). Multivariate analysis showed that a higher radiotherapy dose was associated with increased pathological complete response (p = 0.003, hazard ratio: 3.215), better overall survival (p = 0.024, hazard ratio: 1.585), and superior disease-free survival (p = 0.049, hazard ratio: 1.493). However, higher radiotherapy doses revealed more surgical complications, including acute respiratory distress syndrome (p = 0.048) and anastomosis leaks (p = 0.004). Conclusion: For patients with locally advanced ESCC, preoperative chemoradiotherapy with higher radiotherapy doses led to increased pathologic complete response rates and improved survival.

C1 [Lo, Chien-Ming; Lu, Hung-, I] Chang Gung Univ, Coll Med, Kaohsiung Chang Gung Mem Hosp, Dept Thorac & Cardiovasc Surg, Kaohsiung 833401, Taiwan.

[Wang, Yu-Ming; Fang, Fu-Min] Chang Gung Univ, Coll Med, Kaohsiung Chang Gung Mem Hosp, Dept Radiat Oncol, Kaohsiung 833401, Taiwan.

[Chen, Yen-Hao; Li, Shau-Hsuan] Chang Gung Univ, Coll Med, Kaohsiung Chang Gung Mem Hosp, Dept Hematol Oncol, Kaohsiung 833401, Taiwan.

[Huang, Shun-Chen] Chang Gung Univ, Coll Med, Kaohsiung Chang Gung Mem Hosp, Dept Pathol, Kaohsiung 833401, Taiwan.

RP Lu, HI (corresponding author), Chang Gung Univ, Coll Med, Kaohsiung Chang Gung Mem Hosp, Dept Thorac & Cardiovasc Surg, Kaohsiung 833401, Taiwan.; Li, SH (corresponding author), Chang Gung Univ, Coll Med, Kaohsiung Chang Gung Mem Hosp, Dept Hematol Oncol, Kaohsiung 833401, Taiwan.

EM t123207424@cgmh.org.tw; scorpion@cgmh.org.tw; alex2999@cgmh.org.tw;

fang2569@cgmh.org.tw; shuang@cgmh.org.tw; luhungi@cgmh.org.tw;

lee.a0928@msa.hinet.net

OI Wang, Yu-Ming/0000-0002-2441-7551

FU Chang Gung Memorial Hospital [CMRPG8J0403, CMRPG8K0492, CMRPG8K1291]

FX This research was funded by grants from Chang Gung Memorial Hospital

(CMRPG8J0403, CMRPG8K0492, and CMRPG8K1291).

CR Bedenne L, 2007, J CLIN ONCOL, V25, P1160, DOI 10.1200/JCO.2005.04.7118

Bosset JF, 1997, NEW ENGL J MED, V337, P161, DOI 10.1056/NEJM199707173370304

Burmeister BH, 2005, LANCET ONCOL, V6, P659, DOI 10.1016/S1470-2045(05)70288-6

Engel S, 2019, J GASTROINTEST ONCOL, V10, P391, DOI 10.21037/jgo.2019.01.02

Fransen LFC, 2021, ANN SURG, V274, pE1129, DOI 10.1097/SLA.0000000000003772

Fujita H, 2005, J SURG ONCOL, V90, P209, DOI 10.1002/jso.20259

Gebski V, 2007, LANCET ONCOL, V8, P226, DOI 10.1016/S1470-2045(07)70039-6

Hofstetter W, 2002, ANN SURG, V236, P376, DOI 10.1097/00000658-200209000-00014

Koeter M, 2015, RADIAT ONCOL, V10, DOI 10.1186/s13014-015-0361-4

Lee JL, 2004, ANN ONCOL, V15, P947, DOI 10.1093/annonc/mdh219

Li SH, 2019, SURGERY, V165, P946, DOI 10.1016/j.surg.2018.11.015

Li ZH, 2021, CURR ONCOL, V28, P693, DOI 10.3390/curroncol28010068

Natsugoe S, 2006, DIS ESOPHAGUS, V19, P468, DOI 10.1111/j.1442-2050.2006.00615.x

NYGAARD K, 1992, WORLD J SURG, V16, P1104, DOI 10.1007/BF02067069

Prenzel KL, 2007, ANN SURG ONCOL, V14, P954, DOI 10.1245/s10434-006-9141-6

Shain AH, 2013, PLOS ONE, V8, DOI 10.1371/journal.pone.0055119

Sherman Carol A, 2002, Curr Treat Options Oncol, V3, P475, DOI 10.1007/s11864-002-0067-3

Stahl M, 2005, J CLIN ONCOL, V23, P2310, DOI 10.1200/JCO.2005.00.034

Stoner GD, 2001, CARCINOGENESIS, V22, P1737, DOI 10.1093/carcin/22.11.1737

Tepper J, 2008, J CLIN ONCOL, V26, P1086, DOI 10.1200/JCO.2007.12.9593

Thomas M, 2019, RADIOTHER ONCOL, V141, P33, DOI 10.1016/j.radonc.2019.09.015

Urba SG, 2001, J CLIN ONCOL, V19, P305, DOI 10.1200/JCO.2001.19.2.305

van Hagen P, 2012, NEW ENGL J MED, V366, P2074, DOI 10.1056/NEJMoa1112088

Walle CV, 2012, INT J RADIAT ONCOL, V82, pE513, DOI 10.1016/j.ijrobp.2011.05.071

Walsh TN, 1996, NEW ENGL J MED, V335, P462, DOI 10.1056/NEJM199608153350702

Yang H, 2018, J CLIN ONCOL, V36, P2796, DOI 10.1200/JCO.2018.79.1483

NR 26

TC 2

Z9 2

U1 0

U2 0

PU MDPI

PI BASEL

PA ST ALBAN-ANLAGE 66, CH-4052 BASEL, SWITZERLAND

SN 1198-0052

EI 1718-7729

J9 CURR ONCOL

JI Curr. Oncol.

PD APR

PY 2021

VL 28

IS 2

BP 1354

EP 1365

DI 10.3390/curroncol28020129

PG 12

WC Oncology

WE Science Citation Index Expanded (SCI-EXPANDED)

SC Oncology

GA RS5HJ

UT WOS:000643809800001

PM 33805318

OA Green Published, gold

DA 2022-05-19

ER

PT J

AU Sun, NN

Ge, XL

Liu, XS

Xu, LL

AF Sun, Na-Na

Ge, Xiao-Lin

Liu, Xi-Sheng

Xu, Lu-Lu

TI Histogram analysis of DCE-MRI for chemoradiotherapy response evaluation

in locally advanced esophageal squamous cell carcinoma

SO RADIOLOGIA MEDICA

LA English

DT Article

DE Dynamic contrast-enhanced MRI; Esophageal cancer; Chemoradiotherapy

response; Histogram analysis

ID CONTRAST-ENHANCED MRI; TUMOR RESPONSE; PARAMETRIC HISTOGRAM;

CERVICAL-CANCER; THERAPY; CHEMORADIATION; HETEROGENEITY; PREDICTION;

MICROCIRCULATION; BIOMARKERS

AB Aims The aim of the study was to predict and assess treatment response by histogram analysis of dynamic contrast-enhanced magnetic resonance imaging (DCE-MRI) to patients with locally advanced esophageal squamous cell carcinoma receiving chemoradiotherapy (CRT). Materials and methods Seventy-two patients with locally advanced esophageal squamous cell carcinoma who underwent DCE-MRI before and after chemoradiotherapy were enrolled and divided into the complete response (CR) group and the non-CR group based on RECIST. The histogram parameters (10th percentile, 90th percentile, median, mean, standard deviation, skewness, and kurtosis) of pre-CRT and post-CRT were compared using a paired Student's t test in the CR and non-CR groups, respectively. The histogram parameter differences between the CR and the non-CR groups were compared using an unpaired Student's t test. A receiver operating characteristic (ROC) analysis was performed to evaluate the diagnostic performance. Results The histogram parameters of K-trans values were observed to have significantly decreased after chemoradiotherapy in the CR group. The CR responders showed significantly higher median, mean, and 10th and 90th percentile of pre-K-trans values than those of the non-CR group. The histogram analysis indicated the decreased heterogeneity in the CR group after CRT. Esophageal cancer with higher pre-K-trans and lower post-K-trans values indicated a good treatment response to CRT. Pre-K-trans-10th showed the best diagnostic performance in predicting the chemoradiotherapy response. Conclusions The histogram parameters of K-trans are useful in the assessment and prediction of the chemoradiotherapy response in patients with advanced esophageal squamous cell carcinoma. DCE-MRI could serve as an adjunctive imaging technique for treatment planning.

C1 [Sun, Na-Na; Liu, Xi-Sheng; Xu, Lu-Lu] Nanjing Med Univ, Affiliated Hosp 1, Dept Radiol, 300 Guangzhou Rd, Nanjing 210000, Jiangsu, Peoples R China.

[Ge, Xiao-Lin] Nanjing Med Univ, Affiliated Hosp 1, Dept Radiotherapy, Nanjing 210000, Jiangsu, Peoples R China.

RP Liu, XS (corresponding author), Nanjing Med Univ, Affiliated Hosp 1, Dept Radiol, 300 Guangzhou Rd, Nanjing 210000, Jiangsu, Peoples R China.

EM liuxisheng2118@126.com

CR Allum WH, 2009, J CLIN ONCOL, V27, P5062, DOI 10.1200/JCO.2009.22.2083

Bollschweiler E, 2015, CHINESE J CANCER RES, V27, P221, DOI 10.3978/j.issn.1000-9604.2015.04.04

Chang YC, 2004, PHYS MED BIOL, V49, P3593, DOI 10.1088/0031-9155/49/16/007

Chawla S, 2013, AM J ROENTGENOL, V200, P35, DOI 10.2214/AJR.12.9432

Chikui T, 2012, J MAGN RESON IMAGING, V36, P589, DOI 10.1002/jmri.23704

Cooper RA, 2000, RADIOTHER ONCOL, V57, P53, DOI 10.1016/S0167-8140(00)00259-0

Crokart N, 2005, CANCER RES, V65, P7911, DOI 10.1158/0008-5472.CAN-05-1288

Davnall F, 2012, INSIGHTS IMAGING, V3, P573, DOI 10.1007/s13244-012-0196-6

De Lussanet QG, 2005, INT J RADIAT ONCOL, V63, P1309, DOI 10.1016/j.ijrobp.2005.04.052

Eisenhauer EA, 2009, EUR J CANCER, V45, P228, DOI 10.1016/j.ejca.2008.10.026

Falk A, 2014, NEURORADIOLOGY, V56, P1031, DOI 10.1007/s00234-014-1426-z

Hectors SJ, 2017, SCI REP-UK, V7, DOI 10.1038/s41598-017-02706-z

Heethuis SE, 2016, RADIOTHER ONCOL, V120, P128, DOI 10.1016/j.radonc.2016.05.009

Heo SH, 2013, KOREAN J RADIOL, V14, P616, DOI 10.3348/kjr.2013.14.4.616

Intven M, 2015, J MAGN RESON IMAGING, V41, P1646, DOI 10.1002/jmri.24718

Jackson A, 2007, CLIN CANCER RES, V13, P3449, DOI 10.1158/1078-0432.CCR-07-0238

Jemal A, 2011, CA-CANCER J CLIN, V61, P69, DOI [10.3322/caac.20107, 10.3322/caac.20115]

Jordan BF, 2005, CLIN CANCER RES, V11, P529

Just N, 2014, BRIT J CANCER, V111, P2205, DOI 10.1038/bjc.2014.512

Kim JH, 2012, EUR RADIOL, V22, P2533, DOI 10.1007/s00330-012-2504-4

Kim SH, 2014, J MAGN RESON IMAGING, V40, P730, DOI 10.1002/jmri.24387

King AD, 2016, CANCER IMAGING, V16, DOI 10.1186/s40644-016-0080-6

Kwee RM, 2014, PLOS ONE, V9, DOI 10.1371/journal.pone.0092211

Lee HY, 2016, DIAGN INTERV RADIOL, V22, P231, DOI 10.5152/dir.2015.15171

Lei J, 2015, ONCOL LETT, V10, P3607, DOI 10.3892/ol.2015.3779

Lerant G, 2015, PATHOL ONCOL RES, V21, P1091, DOI 10.1007/s12253-015-9942-7

Li ZW, 2018, J MAGN RESON IMAGING, V47, P91, DOI 10.1002/jmri.25762

Lim JS, 2012, EUR RADIOL, V22, P1693, DOI 10.1007/s00330-012-2416-3

Lin M, 2016, ONCOTARGETS THER, V9, P7029, DOI 10.2147/OTT.S112555

Liu S, 2016, ONCOTARGETS THER, V9, P5791, DOI 10.2147/OTT.S107466

Meng J, 2017, ONCOTARGET, V8, P92442, DOI 10.18632/oncotarget.21374

Murayama K, 2018, MAGN RESON MED SCI, V17, P42, DOI 10.2463/mrms.mp.2016-0113

Oberholzer K, 2008, J MAGN RESON IMAGING, V27, P1296, DOI 10.1002/jmri.21305

Park M, 2016, AM J ROENTGENOL, V206, P401, DOI 10.2214/AJR.15.14550

Peng SL, 2013, NMR BIOMED, V26, P443, DOI 10.1002/nbm.2882

Pham TT, 2017, BRIT J RADIOL, V90, DOI 10.1259/bjr.20151078

Rosenkrantz AB, 2015, ABDOM IMAGING, V40, P327, DOI 10.1007/s00261-014-0213-y

Suo ST, 2016, J MAGN RESON IMAGING, V43, P894, DOI 10.1002/jmri.25043

Tao CJ, 2015, J CANCER, V6, P1179, DOI 10.7150/jca.12346

Wang HY, 2016, SCI REP-UK, V6, DOI 10.1038/srep29146

Wu CJ, 2015, ABDOM IMAGING, V40, P3214, DOI 10.1007/s00261-015-0499-4

Xu XQ, 2016, DIAGN INTERV RADIOL, V22, P161, DOI 10.5152/dir.2015.15202

Zahra MA, 2007, LANCET ONCOL, V8, P63, DOI 10.1016/S1470-2045(06)71012-9

NR 43

TC 10

Z9 11

U1 2

U2 3

PU SPRINGER-VERLAG ITALIA SRL

PI MILAN

PA VIA DECEMBRIO, 28, MILAN, 20137, ITALY

SN 0033-8362

EI 1826-6983

J9 RADIOL MED

JI Radiol. Med.

PD FEB

PY 2020

VL 125

IS 2

BP 165

EP 176

DI 10.1007/s11547-019-01081-1

EA OCT 2019

PG 12

WC Radiology, Nuclear Medicine & Medical Imaging

WE Science Citation Index Expanded (SCI-EXPANDED)

SC Radiology, Nuclear Medicine & Medical Imaging

GA KJ5FL

UT WOS:000490380200002

PM 31605354

DA 2022-05-19

ER

PT J

AU Hsu, HS

Lin, MH

Jang, YH

Kuo, TT

Liu, CC

Cheng, TH

AF Hsu, Han-Shui

Lin, Ming-Hsien

Jang, Yi-Hua

Kuo, Ting-Ting

Liu, Chen-Chi

Cheng, Tzu-Hao

TI The 4E-BP1/eIF4E ratio is a determinant for rapamycin response in

esophageal cancer cells

SO JOURNAL OF THORACIC AND CARDIOVASCULAR SURGERY

LA English

DT Article

ID INITIATION-FACTOR 4E; TARGETED THERAPIES; SIGNALING PATHWAY;

PROSTATE-CANCER; PHASE-I; MTOR; EXPRESSION; TRANSLATION; ACTIVATION;

EVEROLIMUS

AB Objectives: Rapamycin inhibits products of molecular pathways in esophageal squamous cell carcinoma and limits tumor cell growth by targeting 4E-BP1- and eIF4E-dependent gene translation. In this study, we investigate the influence of 4E-BP1-to-eIF4E ratio on rapamycin response in esophageal squamous cell carcinoma cells, and the underlying mechanism is discussed.

Methods: The response to rapamycin treatment was examined in 6 esophageal cancer cell lines. Adjustment of the 4E-BP1/eIF4E ratio was carried out by knockdown or overexpression of 4E-BP1 and eIF4E. The relationship between Egr-1 and 4E-BP1 expression in esophageal cancer cells was also studied.

Results: The 4E-BP1/eIF4E ratio was adjusted to evaluate the response to rapamycin treatment in TE1 and TE2 esophageal cancer cells. TE2 cells are sensitized to rapamycin treatment after overexpression of 4E-BP1 or knockdown of eIF4E; TE1 cells become resistant to rapamycin after knockdown of 4E-BP1 or overexpression of eIF4E. These data suggest that the 4E-BP1/eIF4E ratio is a determinant for the response of TE1 and TE2 cells to rapamycin treatment. Egr-1 expression was higher in TE2 cells compared with other esophageal cancer cell lines, and its knockdown increased 4E-BP1 expression in TE2 cells, which became sensitive to rapamycin treatment.

Conclusions: The 4E-BP1/eIF4E ratio is a determinant of the response of rapamycin treatment in esophageal cancer cells. Egr-1 can reduce 4E-BP1 gene expression and render esophageal squamous cell carcinoma cells resistant to rapamycin with a relatively low 4E-BP1/eIF4E ratio. Thus, the 4E-BP1/eIF4E ratio may represent a therapeutic index for the prediction of clinical outcome of rapamycin treatment in patients with esophageal squamous cell carcinoma.

C1 [Hsu, Han-Shui] Natl Yang Ming Univ, Sch Med, Inst Emergency & Crit Care Med, Taipei 112, Taiwan.

[Hsu, Han-Shui] Taipei Vet Gen Hosp, Dept Surg, Div Thorac Surg, Taipei, Taiwan.

[Lin, Ming-Hsien] Natl Yang Ming Univ, Dept Biomed Imaging & Radiol Sci, Taipei 112, Taiwan.

[Lin, Ming-Hsien] Taipei City Hosp, Zhongxiao Branch, Div Nucl Med, Taipei, Taiwan.

[Jang, Yi-Hua; Kuo, Ting-Ting; Cheng, Tzu-Hao] Natl Yang Ming Univ, Inst Biochem & Mol Biol, Taipei 112, Taiwan.

[Liu, Chen-Chi] Natl Yang Ming Univ, Sch Med, Inst Clin Med, Taipei 112, Taiwan.

RP Cheng, TH (corresponding author), Natl Yang Ming Univ, Inst Biochem & Mol Biol, 155,Sec 2,Li Nong St, Taipei 112, Taiwan.

EM thcheng@ym.edu.tw

FU Taipei Veterans General Hospital [V100C-063, V101C-088, V101E1-016,

V102C-028, V103E8-004]; Lung Cancer Foundation; National Science Council

[97-2320-B-010-025, 102-2325-B-010-017]; "Aim for the Top University

Plan" grant from the Ministry of Education of the Republic of China

FX Han-Shui Hsu received grants from Taipei Veterans General Hospital

(V100C-063, V101C-088, V101E1-016, V102C-028, and V103E8-004) and was

supported partly by the Lung Cancer Foundation in Memory of Dr K. S. Lu.

Tzu-Hao Cheng received grants from the National Science Council

(97-2320-B-010-025 and 102-2325-B-010-017) and the "Aim for the Top

University Plan" grant from the Ministry of Education of the Republic of

China.

CR Adamson E, 2003, CANCER BIOL THER, V2, P617

Alain T, 2012, CANCER RES, V72, P6468, DOI 10.1158/0008-5472.CAN-12-2395

Avdulov S, 2004, CANCER CELL, V5, P553, DOI 10.1016/j.ccr.2004.05.024

De Benedetti A, 2004, ONCOGENE, V23, P3189, DOI 10.1038/sj.onc.1207545

Dilling MB, 2002, J BIOL CHEM, V277, P13907, DOI 10.1074/jbc.M110782200

Gao N, 2004, AM J PHYSIOL-CELL PH, V287, pC281, DOI 10.1152/ajpcell.00422.2003

Gingras AC, 1999, ANNU REV BIOCHEM, V68, P913, DOI 10.1146/annurev.biochem.68.1.913

Grosso S, 2011, PLOS ONE, V6, DOI 10.1371/journal.pone.0029136

Harada T, 2001, NAT CELL BIOL, V3, P453, DOI 10.1038/35074516

Hirashima K, 2012, BRIT J CANCER, V106, P876, DOI 10.1038/bjc.2012.36

Hou GQ, 2007, CANCER LETT, V253, P236, DOI 10.1016/j.canlet.2007.01.026

Hsu HS, 2011, CANCER-AM CANCER SOC, V117, P1516, DOI 10.1002/cncr.25599

Hsu HS, 2011, ANN SURG ONCOL, V18, P1469, DOI 10.1245/s10434-010-1428-y

Hsu PK, 2008, J THORAC CARDIOV SUR, V135, P1029, DOI 10.1016/j.jtcvs.2007.11.007

Kao CL, 2009, CANCER LETT, V286, P250, DOI 10.1016/j.canlet.2009.05.031

Kurmasheva RT, 2006, BRIT J CANCER, V95, P955, DOI 10.1038/sj.bjc.6603353

Leu SJJ, 2013, J CELL BIOCHEM, V114, P2082, DOI 10.1002/jcb.24557

Mamane Y, 2007, PLOS ONE, V2, DOI 10.1371/journal.pone.0000242

Mohamed A, 2014, CRIT REV ONCOL HEMAT, V91, P186, DOI 10.1016/j.critrevonc.2014.01.010

Noh WC, 2004, CLIN CANCER RES, V10, P1013, DOI 10.1158/1078-0432.CCR-03-0043

O'Reilly KE, 2009, CLIN CANCER RES, V15, P2872, DOI 10.1158/1078-0432.CCR-08-2336

Okamoto I, 2010, JPN J CLIN ONCOL, V40, P17, DOI 10.1093/jjco/hyp120

Populo H, 2012, INT J MOL SCI, V13, P1886, DOI 10.3390/ijms13021886

Rolli-Derkinderen M, 2003, J BIOL CHEM, V278, P18859, DOI 10.1074/jbc.M211696200

Salehi Z, 2006, CLIN BIOCHEM, V39, P404, DOI 10.1016/j.clinbiochem.2005.11.007

Seufferlein T, 1996, CANCER RES, V56, P3895

Shaw RJ, 2006, NATURE, V441, P424, DOI 10.1038/nature04869

She QB, 2010, CANCER CELL, V18, P39, DOI 10.1016/j.ccr.2010.05.023

Sonenberg N, 1998, CURR OPIN CELL BIOL, V10, P268, DOI 10.1016/S0955-0674(98)80150-6

van der Poel HG, 2003, UROL RES, V30, P380, DOI 10.1007/s00240-002-0282-1

Wang XM, 2007, METHOD ENZYMOL, V431, P113, DOI 10.1016/S0076-6879(07)31007-0

Werner D, 2013, CANCER MED-US, V2, P325, DOI 10.1002/cam4.77

WONG FH, 1994, CANCER INVEST, V12, P121, DOI 10.3109/07357909409024867

Yellen P, 2011, CELL CYCLE, V10, P3948, DOI 10.4161/cc.10.22.18124

NR 34

TC 4

Z9 4

U1 0

U2 3

PU MOSBY-ELSEVIER

PI NEW YORK

PA 360 PARK AVENUE SOUTH, NEW YORK, NY 10010-1710 USA

SN 0022-5223

EI 1097-685X

J9 J THORAC CARDIOV SUR

JI J. Thorac. Cardiovasc. Surg.

PD JAN

PY 2015

VL 149

IS 1

BP 378

EP 385

DI 10.1016/j.jtcvs.2014.09.047

PG 8

WC Cardiac & Cardiovascular Systems; Respiratory System; Surgery

WE Science Citation Index Expanded (SCI-EXPANDED)

SC Cardiovascular System & Cardiology; Respiratory System; Surgery

GA CC7LW

UT WOS:000350550100101

PM 25439783

OA Bronze

DA 2022-05-19

ER

PT J

AU Wu, X

Hu, XS

Chen, JR

He, L

AF Wu, Xun

Hu, Xingsheng

Chen, Junru

He, Lang

TI A re-irradiation dose of 55-60 Gy improves the survival rate of patients

with local recurrent esophageal squamous cell carcinoma after

radiotherapy

SO RADIATION ONCOLOGY

LA English

DT Article

DE Esophageal squamous cell carcinoma; Recurrence; Salvage chemoradiation

therapy; Salvage radiation therapy

ID SALVAGE SURGERY; DEFINITIVE CHEMORADIATION; CONCURRENT CHEMOTHERAPY;

RADIATION-THERAPY; CANCER; CHEMORADIOTHERAPY; MANAGEMENT; OUTCOMES

AB Introduction Local recurrence (LR) is clinical challenge in the treatment of esophageal squamous cell carcinoma (ESCC). The current study aimed to determine the optimal re-irradiation dose for local recurrent esophageal squamous cell carcinoma (LRESCC) following radical (chemo) radiotherapy. Methods We retrospectively analyzed 125 patients with LRESCC after receiving initial radiotherapy. For radiotherapy treatment, 58 patients were assigned to low-dose (LD) group (50-54 Gy) and 67 were assigned to the high-dose (HD) group (55-60 Gy). The response rate (complete + partial response), 1-, 2- and 3-year survival rate, and toxicity were recorded. We then analyzed the impact of different radiotherapy doses and combination chemotherapy on the survival of patients with LRESCC. Results After re-irradiation, the 1-, 2- and 3-year survival rates in the LD and HD groups were 48.3%, 24.1% and 10.3% and 61.2%, 34.3% and 19.4% in the HD group, respectively, and the difference in overall survival rate between the two groups were significant (P < 0.05). The median survival time of patients receiving radiotherapy alone was 9 months in the LD group and 15 months in the HD group (P < 0.05). The survival rate of patients treated with chemoradiotherapy was higher than that of patients treated with radiotherapy alone in the LD group. However, chemoradiotherapy showed no advantage over radiotherapy alone in the HD group. In addition, the incidence of radiation esophagitis, the most common toxicity, was higher in the HD group compared to the LD group (68.7% vs 58.6%). Multivariate analysis demonstrated that re-irradiation dose was an independent favorable prognostic factor in patients with LRESCC. Conclusion Higher re-irradiation dose (55-60 Gy) can improve the long-term survival of patients with LRESCC after radiotherapy, with tolerable toxicity.

C1 [Wu, Xun; Chen, Junru] Lanzhou Univ, Sch Clin Med 1, Lanzhou 730000, Gansu, Peoples R China.

[Wu, Xun; He, Lang] Chengdu Fifth Peoples Hosp, Dept Oncol, 33 Mashi St, Chengdu 611130, Sichuan, Peoples R China.

[Hu, Xingsheng] Cent South Univ, Dept Oncol, Xiangya Hosp 2, Changsha 410000, Hunan, Peoples R China.

RP He, L (corresponding author), Chengdu Fifth Peoples Hosp, Dept Oncol, 33 Mashi St, Chengdu 611130, Sichuan, Peoples R China.

EM helang729@163.com

CR Amini A, 2014, ANN SURG ONCOL, V21, P306, DOI 10.1245/s10434-013-3303-0

Chen Y, 2014, DIS ESOPHAGUS, V27, P134, DOI 10.1111/j.1442-2050.2012.01440.x

Cohen C, 2018, ANN SURG ONCOL, V25, P1277, DOI 10.1245/s10434-018-6365-1

D'Journo XB, 2014, J THORAC DIS, V6, pS253, DOI 10.3978/j.issn.2072-1439.2014.04.16

Faiz Z, 2019, EJSO-EUR J SURG ONC, V45, P931, DOI 10.1016/j.ejso.2018.11.002

Fakhrian K, 2012, STRAHLENTHER ONKOL, V188, P136, DOI 10.1007/s00066-011-0023-x

Harada H, 2020, LANGENBECK ARCH SURG, V405, P767, DOI 10.1007/s00423-020-01935-7

Ishihara R, 2010, INT J RADIAT ONCOL, V76, P123, DOI 10.1016/j.ijrobp.2009.01.038

Jingu K, 2017, RADIAT ONCOL, V12, DOI 10.1186/s13014-017-0882-0

Katano A, 2017, MOL CLIN ONCOL, V7, P681, DOI 10.3892/mco.2017.1384

Kim HW, 2014, DIGESTION, V90, P254, DOI 10.1159/000368983

Kim YS, 2012, RADIAT ONCOL J, V30, P182, DOI 10.3857/roj.2012.30.4.182

Kobayashi R, 2014, DIS ESOPHAGUS, V27, P72, DOI 10.1111/dote.12068

Li CC, 2018, MEDICINE, V97, DOI 10.1097/MD.0000000000010928

Markar S, 2015, J CLIN ONCOL, V33, P3866, DOI 10.1200/JCO.2014.59.9092

Pennathur A, 2013, LANCET, V381, P400, DOI 10.1016/S0140-6736(12)60643-6

Shridhar R, 2013, CANCER CONTROL, V20, P97, DOI 10.1177/107327481302000203

Sudo K, 2014, J CLIN ONCOL, V32, P3400, DOI 10.1200/JCO.2014.56.7156

Sun X, 2020, FRONT ONCOL, V10, DOI 10.3389/fonc.2020.01222

Werner-Wasik M, 2000, INT J RADIAT ONCOL, V48, P689, DOI 10.1016/S0360-3016(00)00699-4

Wu PP, 2017, ONCOTARGET, V8, P63871, DOI 10.18632/oncotarget.19151

Xiao LL, 2020, J CANCER, V11, P4605, DOI 10.7150/jca.44447

Xu XJ, 2019, RADIAT ONCOL, V14, DOI 10.1186/s13014-019-1402-1

Zhang W, 2018, CANCER MANAG RES, V10, P2675, DOI 10.2147/CMAR.S160909

Zhou ZG, 2015, RADIAT ONCOL, V10, DOI 10.1186/s13014-015-0358-z

NR 25

TC 0

Z9 0

U1 0

U2 2

PU BMC

PI LONDON

PA CAMPUS, 4 CRINAN ST, LONDON N1 9XW, ENGLAND

EI 1748-717X

J9 RADIAT ONCOL

JI Radiat. Oncol.

PD JUN 8

PY 2021

VL 16

IS 1

AR 100

DI 10.1186/s13014-021-01828-z

PG 9

WC Oncology; Radiology, Nuclear Medicine & Medical Imaging

WE Science Citation Index Expanded (SCI-EXPANDED)

SC Oncology; Radiology, Nuclear Medicine & Medical Imaging

GA ST8XO

UT WOS:000662722000002

PM 34103059

OA gold, Green Published

DA 2022-05-19

ER

PT J

AU Sobajima, J

Kumamoto, K

Haga, N

Tamaru, J

Takahashi, T

Miyazaki, T

Ishida, H

AF Sobajima, Jun

Kumamoto, Kensuke

Haga, Norihiro

Tamaru, Junichi

Takahashi, Takeo

Miyazaki, Tatsuya

Ishida, Hideyuki

TI Early evaluation of the apoptotic index ratio is useful in predicting

the efficacy of chemoradiotherapy in esophageal squamous cell carcinoma

SO ONCOLOGY LETTERS

LA English

DT Article

DE apoptotic index; chemoradiotherapy; esophageal squamous cell carcinoma

ID P53 PROTEIN EXPRESSION; NEOADJUVANT CHEMORADIOTHERAPY; CANCER;

RADIATION; RADIOTHERAPY; SENSITIVITY; METAANALYSIS; PROGNOSIS; THERAPY;

SURGERY

AB Chemoradiotherapy for advanced esophageal cancer is a standard treatment alongside surgical treatment. Although numerous investigators have attempted to identify the predictive markers for chemoradiosensitivity, there appear to be few candidates that can be applied in clinical use. Using biopsy specimens, we investigated the apoptotic index (AI) prior to treatment and following a radiation dose of 10 Gy to detect the early response to chemoradiotherapy in 28 patients with esophageal squamous cell carcinoma. Molecular markers, including p53, p21, bax, bcl-2, HSP27, HSP70, HSP90, Ku70, Ku86 and HIF-1 alpha, were also examined by immunohistochemical staining. The patients were divided into two groups depending on the response to chemoradiotherapy: a responder group (RG) (n=19) that included the patients with complete or partial response, and a non-responder group (NRG) (n=9), that included patients with stable or progressive disease In the RG and NRG, the AI of pretreatment was 4.7 +/- 5.3 (mean +/- SD, cells/1,000 cells) and 5.9 +/- 3.7, respectively. The apoptotic index ratio (AIR), which was determined by dividing the AI following 10 Gy radiation by the pretreatment AI, was higher in the RG compared to the NRG (4.7 +/- 4.5 versus 1.9 +/- 1.4, p=0.03). When the cut-off value of AIR was set at 2.4, the sensitivity, specificity and accuracy were 74,78 and 76%, respectively. Among the molecular markers we examined immunohistochemically, a positive p53 expression in the pretreatment evaluation was associated with the efficacy of chemoradiotherapy (p=0.08). Regarding the expression of other molecular markers, no significant correlations were found in RG and NRG. In the present study, the results indicated that AIR is useful for the prediction of chemoradiosensitivity in esophageal squamous cell carcinoma.

C1 [Sobajima, Jun; Kumamoto, Kensuke; Haga, Norihiro; Ishida, Hideyuki] Saitama Med Univ, Saitama Med Ctr, Dept Digest Tract & Gen Surg, Kawagoe, Saitama 3508550, Japan.

[Tamaru, Junichi] Saitama Med Univ, Saitama Med Ctr, Dept Pathol, Kawagoe, Saitama 3508550, Japan.

[Takahashi, Takeo] Saitama Med Univ, Saitama Med Ctr, Dept Radiat Oncol, Kawagoe, Saitama 3508550, Japan.

[Miyazaki, Tatsuya] Gunma Univ, Grad Sch, Grad Sch Med, Dept Gen Surg Sci, Gunma, Japan.

RP Ishida, H (corresponding author), Saitama Med Univ, Saitama Med Ctr, Dept Digest Tract & Gen Surg, 1981 Kamoda, Kawagoe, Saitama 3508550, Japan.

EM 05hishi@saitama-med.ac.jp

RI huang, simon/H-3756-2012

CR Fiorica F, 2004, GUT, V53, P925, DOI 10.1136/gut.2003.025080

Greer SE, 2005, SURGERY, V137, P172, DOI 10.1016/j.surg.2004.06.033

Gullo C, 2006, BBA-REV CANCER, V1765, P223, DOI 10.1016/j.bbcan.2006.01.001

Hefferin ML, 2005, DNA REPAIR, V4, P639, DOI 10.1016/j.dnarep.2004.12.005

Ito T, 2001, J GASTROENTEROL, V36, P303, DOI 10.1007/s005350170095

Japan Esophageal Society, 2007, JAP CLASS ES CANC, V10th

Japanese Society for Esophageal Diseases, 2004, ESOPHAGUS, V1, P61

JEGO G, 2010, CANC LETT	1113

Kitamura K, 2000, HEPATO-GASTROENTEROL, V47, P419

Lv J, 2009, WORLD J GASTROENTERO, V15, P4962, DOI 10.3748/wjg.15.4962

Matsubara H, 1999, INT J ONCOL, V14, P1081

Mitsuhashi N, 1996, INT J RADIAT BIOL, V69, P329, DOI 10.1080/095530096145887

Miyata H, 2000, CLIN CANCER RES, V6, P4859

Muro K, 1996, JPN J CLIN ONCOL, V26, P65, DOI 10.1093/oxfordjournals.jjco.a023186

Oka K, 2000, CANCER-AM CANCER SOC, V89, P1526

Raju U, 2006, RADIOTHER ONCOL, V80, P185, DOI 10.1016/j.radonc.2006.07.027

SARBIA M, 1994, CANCER, V74, P2218, DOI 10.1002/1097-0142(19941015)74:8<2218::AID-CNCR2820740803>3.0.CO;2-2

Shimoji H, 2000, DIS ESOPHAGUS, V13, P185, DOI 10.1046/j.1442-2050.2000.00097.x

Shimoyama S, 1998, HEPATO-GASTROENTEROL, V45, P1497

SOBIN LH, 2002, TNM CLASSIFICATION M, P72

Sohda M, 2004, INT J CANCER, V110, P838, DOI 10.1002/ijc.20215

Tepper J, 2008, J CLIN ONCOL, V26, P1086, DOI 10.1200/JCO.2007.12.9593

Theisen J, 2009, WORLD J GASTRO SURG, V1, P30, DOI 10.4240/wjgs.v1.i1.30

Therasse P, 2000, J NATL CANCER I, V92, P205, DOI 10.1093/jnci/92.3.205

Urschel JD, 2003, AM J SURG, V185, P538, DOI 10.1016/S0002-9610(03)00066-7

VALENTE G, 1994, J CLIN PATHOL, V47, P109, DOI 10.1136/jcp.47.2.109

NR 26

TC 1

Z9 1

U1 0

U2 1

PU SPANDIDOS PUBL LTD

PI ATHENS

PA POB 18179, ATHENS, 116 10, GREECE

SN 1792-1074

J9 ONCOL LETT

JI Oncol. Lett.

PD FEB

PY 2012

VL 3

IS 2

BP 287

EP 292

DI 10.3892/ol.2011.468

PG 6

WC Oncology

WE Science Citation Index Expanded (SCI-EXPANDED)

SC Oncology

GA 880KL

UT WOS:000299405900007

PM 22740896

OA Green Published, gold

DA 2022-05-19

ER

PT J

AU Chen, ZL

Li, JG

Tian, LQ

Zhou, CC

Gao, YB

Zhou, F

Shi, SS

Feng, XL

Sun, N

Yao, R

Shao, K

Li, N

Qiu, B

Tan, FW

He, J

AF Chen, Zhaoli

Li, Jiagen

Tian, Liqing

Zhou, Chengcheng

Gao, Yibo

Zhou, Fang

Shi, Susheng

Feng, Xiaoli

Sun, Nan

Yao, Ran

Shao, Kang

Li, Ning

Qiu, Bin

Tan, Fengwei

He, Jie

TI MiRNA expression profile reveals a prognostic signature for esophageal

squamous cell carcinoma

SO CANCER LETTERS

LA English

DT Article

DE Esophageal squamous cell carcinoma; MicroRNA; Signature; Prognosis;

Biomarker

ID MICRORNA SIGNATURE; TUMOR-SUPPRESSOR; GENE SIGNATURE; MIR-218;

INVASIVENESS; PROGRESSION; SURVIVAL; TARGETS; GROWTH

AB In this study, we analyzed the microRNA (miRNA) expression profile of 119 paired ESCC samples by microarray and identified a four-miRNA signature that predicted patient survival. The signature derived from the training set (n = 60) had a good prognostic value in the test set (n = 59) and the independent cohort (n = 58), indicating the replicability of its prognostic value. Furthermore, the stratified analysis showed that the signature could predict the survival of TNM stage II and stage III patients, indicating that the four-miRNA signature could help to more accurately predict ESCC patient survival. (C) 2014 Elsevier Ireland Ltd. All rights reserved.

C1 [Chen, Zhaoli; Li, Jiagen; Zhou, Chengcheng; Gao, Yibo; Zhou, Fang; Sun, Nan; Yao, Ran; Shao, Kang; Li, Ning; Qiu, Bin; Tan, Fengwei; He, Jie] Peking Union Med Coll, Canc Hosp & Inst, Dept Thorac Surg, Beijing 100021, Peoples R China.

[Chen, Zhaoli; Li, Jiagen; Zhou, Chengcheng; Gao, Yibo; Zhou, Fang; Shi, Susheng; Feng, Xiaoli; Sun, Nan; Yao, Ran; Shao, Kang; Li, Ning; Qiu, Bin; Tan, Fengwei; He, Jie] Chinese Acad Med Sci, Beijing 100730, Peoples R China.

[Tian, Liqing] Chinese Acad Sci, Inst Biophys, Bioinformat Lab, Beijing 100101, Peoples R China.

[Tian, Liqing] Chinese Acad Sci, Inst Biophys, Lab Noncoding RNA, Beijing 100101, Peoples R China.

[Shi, Susheng; Feng, Xiaoli] Peking Union Med Coll, Canc Hosp & Inst, Dept Pathol, Beijing 100021, Peoples R China.

RP He, J (corresponding author), Chinese Acad Med Sci, Canc Hosp & Inst, Dept Thorac Surg, Beijing 100021, Peoples R China.

EM prof.jiehe@gmail.com

RI Gao, Yi-Bo/AAP-4866-2020

OI Gao, Yi-Bo/0000-0002-0501-9814; Tian, Liqing/0000-0001-9921-0603

FU National High Technology Research and Development Program of China

[2012AA02A502, 2012AA02A503, 2012AA02A207]; National Natural Science

Foundation of China [81172336, 81372219, 81301851]; Government Health

Care Research Foundation [820098120]

FX This work was supported by the National High Technology Research and

Development Program of China (2012AA02A502, 2012AA02A503, 2012AA02A207),

the National Natural Science Foundation of China (81172336, 81372219,

81301851), and the Government Health Care Research Foundation for Senior

Officials (820098120).

CR Alajez NM, 2011, CANCER RES, V71, P2381, DOI 10.1158/0008-5472.CAN-10-2754

Bolstad BM, 2003, BIOINFORMATICS, V19, P185, DOI 10.1093/bioinformatics/19.2.185

Calin GA, 2005, NEW ENGL J MED, V353, P1793, DOI 10.1056/NEJMoa050995

Campbell PJ, 2008, P NATL ACAD SCI USA, V105, P13081, DOI 10.1073/pnas.0801523105

Chen DT, 2011, JNCI-J NATL CANCER I, V103, P1859, DOI 10.1093/jnci/djr420

Chen HY, 2007, NEW ENGL J MED, V356, P11, DOI 10.1056/NEJMoa060096

Chen ZL, 2011, J BIOL CHEM, V286, P10725, DOI 10.1074/jbc.M110.165654

Childs G, 2009, AM J PATHOL, V174, P736, DOI 10.2353/ajpath.2009.080731

Choudhury Y, 2012, J CLIN INVEST, V122, P4059, DOI 10.1172/JCI62925

Edge SB, 2010, ANN SURG ONCOL, V17, P1471, DOI 10.1245/s10434-010-0985-4

Gao CP, 2010, CANCER-AM CANCER SOC, V116, P41, DOI 10.1002/cncr.24743

Guo Y, 2008, CANCER RES, V68, P26, DOI 10.1158/0008-5472.CAN-06-4418

Huang DW, 2009, NAT PROTOC, V4, P44, DOI 10.1038/nprot.2008.211

Ji L, 2007, WORLD J GASTROENTERO, V13, P2622, DOI 10.3748/wjg.v13.i18.2622

Kong KL, 2012, GUT, V61, P33, DOI 10.1136/gutjnl-2011-300178

Krek A, 2005, NAT GENET, V37, P495, DOI 10.1038/ng1536

Lacson R, 2010, J BIOMED INFORM, V43, P709, DOI 10.1016/j.jbi.2010.04.007

Lewis BP, 2005, CELL, V120, P15, DOI 10.1016/j.cell.2004.12.035

Li J, 2014, GUT

Lin RJ, 2012, J SURG ONCOL, V105, P175, DOI 10.1002/jso.22066

Liu N, 2012, LANCET ONCOL, V13, DOI 10.1016/S1470-2045(12)70102-X

Liu YW, 2012, ONCOL REP, V28, P1013, DOI 10.3892/or.2012.1902

Lu J, 2005, NATURE, V435, P834, DOI 10.1038/nature03702

McShane LM, 2005, EUR J CANCER, V41, P1690, DOI 10.1016/j.ejca.2005.03.032

Schaefer A, 2010, INT J CANCER, V126, P1166, DOI 10.1002/ijc.24827

Schwarz DF, 2010, BIOINFORMATICS, V26, P1752, DOI 10.1093/bioinformatics/btq257

Srivastava SK, 2011, CARCINOGENESIS, V32, P1832, DOI 10.1093/carcin/bgr223

Su H, 2011, CLIN CANCER RES, V17, P2955, DOI 10.1158/1078-0432.CCR-10-2724

Tatarano S, 2011, INT J ONCOL, V39, P13, DOI 10.3892/ijo.2011.1012

Tong M, 2012, CANCER RES, V72, P6024, DOI 10.1158/0008-5472.CAN-12-1269

Uesugi A, 2011, CANCER RES, V71, P5765, DOI 10.1158/0008-5472.CAN-11-0368

Wang XW, 2008, RNA, V14, P1012, DOI 10.1261/rna.965408

Watanabe A, 2011, LEUKEMIA, V25, P1324, DOI 10.1038/leu.2011.81

Yang L, 2005, CANCER EPIDEM BIOMAR, V14, P243

Yu SL, 2008, CANCER CELL, V13, P48, DOI 10.1016/j.ccr.2007.12.008

Zhang J, 2012, INT J ONCOL, V40, P747, DOI 10.3892/ijo.2011.1242

Zhu CQ, 2010, J CLIN ONCOL, V28, P4417, DOI 10.1200/JCO.2009.26.4325

NR 37

TC 41

Z9 43

U1 2

U2 22

PU ELSEVIER IRELAND LTD

PI CLARE

PA ELSEVIER HOUSE, BROOKVALE PLAZA, EAST PARK SHANNON, CO, CLARE, 00000,

IRELAND

SN 0304-3835

EI 1872-7980

J9 CANCER LETT

JI Cancer Lett.

PD AUG 1

PY 2014

VL 350

IS 1-2

BP 34

EP 42

DI 10.1016/j.canlet.2014.04.013

PG 9

WC Oncology

WE Science Citation Index Expanded (SCI-EXPANDED)

SC Oncology

GA AJ7IS

UT WOS:000337871000005

PM 24769072

DA 2022-05-19

ER

PT J

AU Pan, XY

Wang, J

Guo, LJ

Na, FF

Du, JJ

Chen, XL

Zhong, AL

Zhao, L

Zhang, L

Zhang, MS

Wan, XD

Wang, ML

Liu, HY

Dai, SQ

Tan, P

Chen, JY

Liu, Y

Hu, B

Chen, C

AF Pan, Xiangyu

Wang, Jian

Guo, Linjie

Na, Feifei

Du, Jiajia

Chen, Xuelan

Zhong, Ailing

Zhao, Lei

Zhang, Lu

Zhang, Mengsha

Wan, Xudong

Wang, Manli

Liu, Hongyu

Dai, Siqi

Tan, Ping

Chen, Jingyao

Liu, Yu

Hu, Bing

Chen, Chong

TI Identifying a confused cell identity for esophageal squamous cell

carcinoma

SO SIGNAL TRANSDUCTION AND TARGETED THERAPY

LA English

DT Article

ID GENOME-WIDE ASSOCIATION; STEM-CELLS; LINEAGE PLASTICITY; CANCER; ORIGIN;

LUNG; HALLMARKS; SOX2; DIFFERENTIATION; HETEROGENEITY

AB The cell identity of malignant cells and how they acquire it are fundamental for our understanding of cancer. Here, we report that esophageal squamous cell carcinoma (ESCC) cells display molecular features equally similar but distinct to all three types of normal esophageal epithelial cells, which we term as confused cell identity (CCI). CCI is an independent prognostic marker associated with poor prognosis in ESCC. Further, we identify tropomyosin 4 (TPM4) as a critical CCI gene that promotes the aggressiveness of ESCC in vitro and in vivo. And TPM4 creates CCI through activating the Jak/STAT-SOX2 pathway. Thus, our study suggests an unrecognized feature of ESCC cells, which might be of value for clinic prognosis and potential interference.

C1 [Pan, Xiangyu; Wang, Jian; Guo, Linjie; Du, Jiajia; Chen, Xuelan; Zhong, Ailing; Zhao, Lei; Zhang, Lu; Zhang, Mengsha; Wan, Xudong; Wang, Manli; Liu, Hongyu; Dai, Siqi; Chen, Jingyao; Liu, Yu; Hu, Bing; Chen, Chong] Sichuan Univ, West China Hosp, State Key Lab Biotherapy, Dept Gastroenterol, Chengdu, Sichuan, Peoples R China.

[Pan, Xiangyu; Wang, Jian; Guo, Linjie; Na, Feifei; Du, Jiajia; Chen, Xuelan; Zhong, Ailing; Zhao, Lei; Zhang, Lu; Zhang, Mengsha; Wan, Xudong; Wang, Manli; Liu, Hongyu; Dai, Siqi; Chen, Jingyao; Liu, Yu; Hu, Bing; Chen, Chong] Sichuan Univ, West China Hosp, Canc Ctr, Chengdu 610041, Sichuan, Peoples R China.

[Na, Feifei] Sichuan Univ, West China Hosp, State Key Lab Biotherapy, Dept Thorac Oncol, Chengdu 610041, Sichuan, Peoples R China.

[Tan, Ping] Sichuan Univ, West China Hosp, Inst Urol, Dpartment Urol, Chengdu, Peoples R China.

RP Liu, Y; Hu, B; Chen, C (corresponding author), Sichuan Univ, West China Hosp, State Key Lab Biotherapy, Dept Gastroenterol, Chengdu, Sichuan, Peoples R China.; Liu, Y; Hu, B; Chen, C (corresponding author), Sichuan Univ, West China Hosp, Canc Ctr, Chengdu 610041, Sichuan, Peoples R China.

EM yuliuscu@scu.edu.cn; hubingnj@163.com; chongchen@scu.edu.cn

FU National Key R&D Program of China [2017YFA0505600]; National Natural

Science Foundation of China [81522003, 82170171, 81770157, 82003156];

Sichuan Science and Technology Program [2020YFQ0059, 2018JZ0077,

2017TJPT0005, 2022YFS0171, 2022YFS0205]; Project for Disciplines of

Excellence, West China Hospital, Sichuan University; innovation

initiative of Sichuan University [2018SCUH0060]

FX We thank the members of the CC-LY laboratory for their technical support

and suggestions, and the Core Facilities of West China Hospital. This

work was supported by the National Key R&D Program of China

(2017YFA0505600), the National Natural Science Foundation of China

(81522003, 82170171, 81770157, and 82003156), the Sichuan Science and

Technology Program (2020YFQ0059, 2018JZ0077, 2017TJPT0005, 2022YFS0171

and 2022YFS0205), 1.3.5. Project for Disciplines of Excellence, West

China Hospital, Sichuan University, and the innovation initiative of

Sichuan University, Grant no. 2018SCUH0060. We thank the Chengdu

OrganoidMed Medical Laboratory for their technical support. We also

thank BioRender (biorender. com) for providing items for drawing scheme

graphs.

CR Bass AJ, 2009, NAT GENET, V41, P1238, DOI 10.1038/ng.465

Batlle E, 2017, NAT MED, V23, P1124, DOI 10.1038/nm.4409

Beebe K, 2010, DEV BIOL, V338, P28, DOI 10.1016/j.ydbio.2009.10.045

Berenson JR, 1998, LEUKEMIA, V12, P121, DOI 10.1038/sj.leu.2400947

Blanpain C, 2007, CELL, V128, P445, DOI 10.1016/j.cell.2007.01.014

Blanpain C, 2013, NAT REV MOL CELL BIO, V14, P489, DOI 10.1038/nrm3625

Cao W, 2020, NAT COMMUN, V11, DOI 10.1038/s41467-020-17227-z

Chen C, 2014, CANCER CELL, V25, P652, DOI 10.1016/j.ccr.2014.03.016

Chen C, 2013, GENE DEV, V27, P1974, DOI 10.1101/gad.226613.113

Chen YK, 2016, SCI REP-UK, V6, DOI 10.1038/srep30057

DeWard AD, 2014, CELL REP, V9, P701, DOI 10.1016/j.celrep.2014.09.027

Dotto GP, 2016, CANCER CELL, V29, P622, DOI 10.1016/j.ccell.2016.04.004

Garraway LA, 2006, NAT REV CANCER, V6, P593, DOI 10.1038/nrc1947

Gen Y, 2013, CANCER SCI, V104, P810, DOI 10.1111/cas.12155

Han XK, 2014, NAT COMMUN, V5, DOI 10.1038/ncomms4261

Han XP, 2020, NATURE, V581, P303, DOI 10.1038/s41586-020-2157-4

Hanahan D, 2000, CELL, V100, P57, DOI 10.1016/S0092-8674(00)81683-9

Hanahan D, 2022, CANCER DISCOV, V12, P31, DOI 10.1158/2159-8290.CD-21-1059

Hanahan D, 2011, CELL, V144, P646, DOI 10.1016/j.cell.2011.02.013

Hu N, 2010, BMC GENOMICS, V11, DOI 10.1186/1471-2164-11-576

Hyland PL, 2016, INT J EPIDEMIOL, V45, P206, DOI 10.1093/ije/dyv294

Kalabis J, 2008, J CLIN INVEST, V118, P3860, DOI 10.1172/JCI35012

Kelly LM, 2002, ANNU REV GENOM HUM G, V3, P179, DOI 10.1146/annurev.genom.3.032802.115046

Kim J, 2017, NATURE, V541, P169, DOI 10.1038/nature20805

Koboldt DC, 2012, NATURE, V490, P61, DOI 10.1038/nature11412

Ku SY, 2017, SCIENCE, V355, P78, DOI 10.1126/science.aah4199

LaFave LM, 2020, CANCER CELL, V38, P212, DOI 10.1016/j.ccell.2020.06.006

Li JG, 2014, GUT, V63, P1700, DOI 10.1136/gutjnl-2013-305806

Li Y, 2021, SIGNAL TRANSDUCT TAR, V6, DOI 10.1038/s41392-021-00682-5

Lin JJC, 2008, ADV EXP MED BIOL, V644, P201

Lomnytska MI, 2011, BRIT J CANCER, V104, P110, DOI 10.1038/sj.bjc.6605992

Madissoon E, 2019, GENOME BIOL, V21, DOI 10.1186/s13059-019-1906-x

Magee JA, 2012, CANCER CELL, V21, P283, DOI 10.1016/j.ccr.2012.03.003

Marjanovic ND, 2020, CANCER CELL, V38, P229, DOI 10.1016/j.ccell.2020.06.012

Morrisey EE, 2018, TRENDS CELL BIOL, V28, P738, DOI 10.1016/j.tcb.2018.04.007

Mu P, 2017, SCIENCE, V355, P84, DOI 10.1126/science.aah4307

Nestorowa S, 2016, BLOOD, V128, pE20, DOI 10.1182/blood-2016-05-716480

Niederst MJ, 2015, NAT COMMUN, V6, DOI 10.1038/ncomms7377

Niwa H, 2009, NATURE, V460, P118, DOI 10.1038/nature08113

Pardal R, 2003, NAT REV CANCER, V3, P895, DOI 10.1038/nrc1232

Perez-Losada J, 2003, NAT REV CANCER, V3, P434, DOI 10.1038/nrc1095

Perou CM, 2000, NATURE, V406, P747, DOI 10.1038/35021093

Qi YJ, 2005, PROTEOMICS, V5, P2960, DOI 10.1002/pmic.200401175

Quintana E, 2008, NATURE, V456, P593, DOI 10.1038/nature07567

Quintanal-Villalonga A, 2020, NAT REV CLIN ONCOL, V17, P360, DOI 10.1038/s41571-020-0340-z

Reya T, 2001, NATURE, V414, P105, DOI 10.1038/35102167

Sanchez-Danes A, 2018, NAT REV CANCER, V18, P549, DOI 10.1038/s41568-018-0024-5

Sell S, 2004, CRIT REV ONCOL HEMAT, V51, P1, DOI 10.1016/j.critrevonc.2004.04.007

Song YM, 2014, NATURE, V509, P91, DOI 10.1038/nature13176

Su H, 2011, CLIN CANCER RES, V17, P2955, DOI 10.1158/1078-0432.CCR-10-2724

Thankamony AP, 2020, FRONT MOL BIOSCI, V7, DOI 10.3389/fmolb.2020.00079

Thomas SJ, 2015, BRIT J CANCER, V113, P365, DOI 10.1038/bjc.2015.233

Tirosh I, 2016, SCIENCE, V352, P189, DOI 10.1126/science.aad0501

Visvader JE, 2011, NATURE, V469, P314, DOI 10.1038/nature09781

Wu C, 2014, NAT GENET, V46, P1001, DOI 10.1038/ng.3064

Wu Z, 2021, NAT GENET, V53, P881, DOI 10.1038/s41588-021-00859-2

Zhang XN, 2021, NAT COMMUN, V12, DOI 10.1038/s41467-021-25539-x

Zhao XT, 2019, ONCOTARGETS THER, V12, P4055, DOI 10.2147/OTT.S198542

NR 58

TC 0

Z9 0

U1 1

U2 1

PU SPRINGERNATURE

PI LONDON

PA CAMPUS, 4 CRINAN ST, LONDON, N1 9XW, ENGLAND

SN 2095-9907

EI 2059-3635

J9 SIGNAL TRANSDUCT TAR

JI Signal Transduct. Target. Ther.

PD APR 13

PY 2022

VL 7

IS 1

AR 122

DI 10.1038/s41392-022-00946-8

PG 11

WC Biochemistry & Molecular Biology; Cell Biology

WE Science Citation Index Expanded (SCI-EXPANDED)

SC Biochemistry & Molecular Biology; Cell Biology

GA 0M9AU

UT WOS:000782440400001

PM 35418165

DA 2022-05-19

ER

PT J

AU Ou, YW

Ma, LY

Ma, L

Huang, Z

Zhou, W

Zhao, CL

Zhang, BL

Song, YM

Yu, CJ

Zhan, QM

AF Ou, Yunwei

Ma, Liying

Ma, Ling

Huang, Zhen

Zhou, Wei

Zhao, Chunling

Zhang, Bailin

Song, Yongmei

Yu, Chunjiang

Zhan, Qimin

TI Overexpression of cyclin B1 antagonizes chemotherapeutic-induced

apoptosis through PTEN/Akt pathway in human esophageal squamous cell

carcinoma cells

SO CANCER BIOLOGY & THERAPY

LA English

DT Article

ID CANCER-CELLS; ENDOMETRIAL CANCERS; PROSTATE-CANCER; DOWN-REGULATION;

OVARIAN-CANCER; TUMOR-CELLS; AKT; TAXOL; PHOSPHORYLATION; GROWTH

AB The role of cyclin B1 in the clinical therapeutic sensitivity of human esophageal squamous cell carcinoma (ESCC) remains to be defined. In this study, we found that elevated cyclin B1 expression attenuated the apoptosis induced by cisplatin or paclitaxel, while knockdown of cyclin B1 enhanced cisplatin or paclitaxel sensitivity in ESCC cells. Cyclin B1-mediated apoptosis may rely on the Bcl-2-dependent mitochondria-regulated intrinsic death pathway, and the antagonizing effect of cyclin B1 on chemotherapeutic agent-induced apoptosis was through PTEN/Akt pathway. Therefore, cyclin B1 might be a therapeutic target for the development of specific and efficient approaches in the treatment of ESCC.

C1 [Ou, Yunwei; Ma, Liying; Huang, Zhen; Zhou, Wei; Zhao, Chunling; Zhang, Bailin; Song, Yongmei; Zhan, Qimin] Chinese Acad Med Sci, Inst Canc, State Key Lab Mol Oncol, Beijing 100021, Peoples R China.

[Ou, Yunwei; Ma, Liying; Huang, Zhen; Zhou, Wei; Zhao, Chunling; Zhang, Bailin; Song, Yongmei; Zhan, Qimin] Chinese Acad Med Sci, Canc Hosp, Beijing 100021, Peoples R China.

[Ou, Yunwei; Ma, Liying; Huang, Zhen; Zhou, Wei; Zhao, Chunling; Zhang, Bailin; Song, Yongmei; Zhan, Qimin] Peking Union Med Coll, Beijing 100021, Peoples R China.

[Ou, Yunwei; Yu, Chunjiang] Capital Med Univ, Beijing Sanbo Brain Hosp, Dept Neurosurg, Beijing, Peoples R China.

[Ma, Ling] Capital Med Univ, Fu Xing Hosp, Dept Intens Care Unit, Beijing, Peoples R China.

RP Song, YM (corresponding author), Chinese Acad Med Sci, Inst Canc, State Key Lab Mol Oncol, Beijing 100021, Peoples R China.

EM ymsong.2008@gmail.com; cjyu1955@sina.com; zhanqimin@pumc.edu.cn

OI song, yongmei/0000-0002-7789-0158

FU 973 National Key Fundamental Research Program of China [2009CB521801];

National Natural Science Foundation of China [81071633, 81021061]

FX We thank Dr Shemada of Kyoto University for providing us with the

esophageal carcinoma cell line KYSE150. This work is supported by

funding from the 973 National Key Fundamental Research Program of China

(2009CB521801) and the National Natural Science Foundation of China

(81071633 and 81021061).

CR Akca H, 2011, LUNG CANCER, V73, P302, DOI 10.1016/j.lungcan.2011.01.012

Altomare DA, 2004, ONCOGENE, V23, P5853, DOI 10.1038/sj.onc.1207721

BELLACOSA A, 1991, SCIENCE, V254, P274, DOI 10.1126/science.1833819

Borgne A, 2006, ONCOGENE, V25, P7361, DOI 10.1038/sj.onc.1209718

Cantley LC, 1999, P NATL ACAD SCI USA, V96, P4240, DOI 10.1073/pnas.96.8.4240

Chae HD, 2011, STEM CELLS DEV, V20, P1277, DOI 10.1089/scd.2010.0465

Chang E, 2010, J THORAC CARDIOV SUR, V140, P225, DOI 10.1016/j.jtcvs.2009.11.038

Davies MA, 1999, CANCER RES, V59, P2551

Enzinger PC, 2003, NEW ENGL J MED, V349, P2241, DOI 10.1056/NEJMra035010

Gomez LA, 2007, MOL CANCER THER, V6, P1534, DOI 10.1158/1535-7163.MCT-06-0727

Han Yaling, 2002, Zhonghua Yi Xue Yi Chuan Xue Za Zhi, V19, P455

Hassan KA, 2002, CANCER RES, V62, P6414

Hayakawa J, 2000, CANCER RES, V60, P5988

HORWITZ SB, 1992, TRENDS PHARMACOL SCI, V13, P134

Itoh N, 2002, CANCER, V94, P3127, DOI 10.1002/cncr.10591

Jemal A, 2005, CA-CANCER J CLIN, V55, P10, DOI 10.3322/canjclin.55.1.10

Jin P, 1998, J CELL BIOL, V141, P875, DOI 10.1083/jcb.141.4.875

Jordan MA, 1996, CANCER RES, V56, P816

Jun DY, 2007, TOXICOL APPL PHARM, V222, P190, DOI 10.1016/j.taap.2007.04.011

Kantari C, 2011, BBA-MOL CELL RES, V1813, P558, DOI 10.1016/j.bbamcr.2011.01.026

Kim JY, 2010, CANCER CHEMOTH PHARM, V66, P31, DOI 10.1007/s00280-009-1130-6

KUMAR N, 1981, J BIOL CHEM, V256, P435

Li HL, 1998, CELL, V94, P491, DOI 10.1016/S0092-8674(00)81590-1

Li HF, 2011, J BIOMOL SCREEN, V16, P383, DOI 10.1177/1087057110397357

Li J, 1997, SCIENCE, V275, P1943, DOI 10.1126/science.275.5308.1943

Li K, 2010, CANCER INVEST, V28, P479, DOI 10.3109/07357900903476745

Lubin DJ, 2010, J MOL BIOL, V395, P705, DOI 10.1016/j.jmb.2009.11.013

Marsh DJ, 1998, J MED GENET, V35, P881, DOI 10.1136/jmg.35.11.881

McDonnell MA, 2003, CELL DEATH DIFFER, V10, P1005, DOI 10.1038/sj.cdd.4401271

MIYAHARA T, 1993, JPN J CANCER RES, V84, P336, DOI 10.1111/j.1349-7006.1993.tb02875.x

Reed JC, 1999, J CLIN ONCOL, V17, P2941, DOI 10.1200/JCO.1999.17.9.2941

Risinger JI, 1998, CLIN CANCER RES, V4, P3005

Risinger JI, 1997, CANCER RES, V57, P4736

SCHIFF PB, 1979, NATURE, V277, P665, DOI 10.1038/277665a0

Slee EA, 1999, J CELL BIOL, V144, P281, DOI 10.1083/jcb.144.2.281

Song YM, 2008, CARCINOGENESIS, V29, P307, DOI 10.1093/carcin/bgm269

Stephens L, 1998, SCIENCE, V279, P710, DOI 10.1126/science.279.5351.710

SZYMKOWSKI DE, 1992, P NATL ACAD SCI USA, V89, P10772, DOI 10.1073/pnas.89.22.10772

Tang L, 2009, REPROD BIOL ENDOCRIN, V7, DOI 10.1186/1477-7827-7-144

Tommasi S, 2011, ONCOGENE, V30, P690, DOI 10.1038/onc.2010.440

Tsai WS, 2006, MOL CANCER THER, V5, P2977, DOI 10.1158/1535-7163.MCT-05-0514

Vivanco I, 2002, NAT REV CANCER, V2, P489, DOI 10.1038/nrc839

Wan XL, 2003, ONCOGENE, V22, P8205, DOI 10.1038/sj.onc.1206878

Wang T, 2010, INT J CANCER, V126, P362, DOI 10.1002/ijc.24753

Wang XX, 2009, CANCER BIOL THER, V8, P1852, DOI 10.4161/cbt.8.19.9550

Xie MH, 2005, INT J CANCER, V116, P520, DOI 10.1002/ijc.21056

Yamada K, 2001, P NATL ACAD SCI USA, V98, P1442, DOI 10.1073/pnas.031470598

Yuan J, 2006, ONCOGENE, V25, P1753, DOI 10.1038/sj.onc.1209202

Yuan JP, 2004, ONCOGENE, V23, P5843, DOI 10.1038/sj.onc.1207757

NR 49

TC 31

Z9 34

U1 1

U2 10

PU TAYLOR & FRANCIS INC

PI PHILADELPHIA

PA 530 WALNUT STREET, STE 850, PHILADELPHIA, PA 19106 USA

SN 1538-4047

EI 1555-8576

J9 CANCER BIOL THER

JI Cancer Biol. Ther.

PD JAN

PY 2013

VL 14

IS 1

BP 45

EP 55

DI 10.4161/cbt.22627

PG 11

WC Oncology

WE Science Citation Index Expanded (SCI-EXPANDED)

SC Oncology

GA 064BQ

UT WOS:000313046300008

PM 23114644

OA Bronze, Green Published

DA 2022-05-19

ER

PT J

AU Liu, YP

Shan, BE

Wang, XL

Ma, L

AF Liu, Y. P.

Shan, B. E.

Wang, X. L.

Ma, L.

TI Correlation between MTA2 overexpression and tumour progression in

esophageal squamous cell carcinoma

SO EXPERIMENTAL AND THERAPEUTIC MEDICINE

LA English

DT Article

DE metastasis-associated gene 2; esophageal cancer; prognosis;

immunohistochemistry

ID BREAST-CANCER CELLS; MOUSE MODEL; METASTASIS; PROTEIN-2; GROWTH; GENE

AB The aim of this study was to clarify the clinicopathological outcome and prognostic significance of metastasis-associated gene 2 (MTA2) in esophageal squamous cell carcinoma (ESCC). lmmunohistochemical staining for MTA2 was performed on surgical specimens obtained from 162 patients with ESCC. Relationships between MTA2 expression and clinicopathological factors, including prognosis, were analyzed. Significant correlations were noted among MTA2 overexpression and TNM clinical classification staging, depth of invasion, presence of regional lymph node metastasis, presence of distant metastasis, lymphatic invasion, blood-vessel invasion and the 5-year survival rates. The expression of MTA2 protein was correlated with tumour progression. Patients with MTA2 overexpression tended to have poor prognosis compared to patients with MTA2 underexpression.

C1 [Ma, L.] Hebei Med Univ, Inst Hosp 4, Dept Surg, Shijiazhuang 050011, Hebei, Peoples R China.

[Liu, Y. P.; Wang, X. L.] Hebei Med Univ, Inst Hosp 4, Dept Pathol, Shijiazhuang 050011, Hebei, Peoples R China.

[Shan, B. E.] Hebei Med Univ, Inst Hosp 4, Ctr Sci Res, Shijiazhuang 050011, Hebei, Peoples R China.

RP Ma, L (corresponding author), Hebei Med Univ, Inst Hosp 4, Dept Surg, 12 Jiankangt Rd, Shijiazhuang 050011, Hebei, Peoples R China.

EM mali102l@126.com

FU Health Department of Hebei Province [07120]; Hebei Province Natural

Science Foundation of P.R. China [C2009001209]

FX This study was supported by a grant from the Health Department of Hebei

Province (No. 07120), and a grant from the Hebei Province Natural

Science Foundation of P.R. China (No. C2009001209).

CR Adjei AA, 2002, SEMIN ONCOL, V29, P50, DOI 10.1053/sonc.2002.30758

Cui YK, 2006, MOL ENDOCRINOL, V20, P2020, DOI 10.1210/me.2005-0063

Fujita N, 2004, MOL ENDOCRINOL, V18, P2937, DOI 10.1210/me.2004-0258

Kumar R, 2003, SEMIN ONCOL, V30, P30, DOI 10.1053/j.seminoncol.2003.08.005

Li W, 2007, CELL TISSUE RES, V329, P351, DOI 10.1007/s00441-007-0412-8

Liu YP, 2010, EJSO-EUR J SURG ONC, V36, P155, DOI 10.1016/j.ejso.2009.09.005

Manavathi Bramanandam, 2007, Nucl Recept Signal, V5, pe010, DOI 10.1621/nrs.05010

Nicolson GL, 2003, CLIN EXP METASTAS, V20, P19, DOI 10.1023/A:1022534217769

Park SI, 2008, CANCER RES, V68, P3323, DOI 10.1158/0008-5472.CAN-07-2997

Saito M, 2002, J BIOL CHEM, V277, P35434, DOI 10.1074/jbc.M203455200

Shappell SB, 2003, CANCER RES, V63, P2256

Toh Y, 2000, J EXP CLIN CANC RES, V19, P105

Wong JJL, 2007, GUT, V56, P140, DOI 10.1136/gut.2005.088799

Yao YL, 2003, J BIOL CHEM, V278, P42560, DOI 10.1074/jbc.M302955200

Zhang H, 2006, CLIN CANCER RES, V12, P1479, DOI 10.1158/1078-0432.CCR-05-1519

NR 15

TC 16

Z9 21

U1 0

U2 4

PU SPANDIDOS PUBL LTD

PI ATHENS

PA POB 18179, ATHENS, 116 10, GREECE

SN 1792-0981

J9 EXP THER MED

JI Exp. Ther. Med.

PD APR

PY 2012

VL 3

IS 4

BP 745

EP 749

DI 10.3892/etm.2012.475

PG 5

WC Medicine, Research & Experimental

WE Science Citation Index Expanded (SCI-EXPANDED)

SC Research & Experimental Medicine

GA 907GV

UT WOS:000301406700033

PM 22969963

OA Green Published, gold

DA 2022-05-19

ER

PT J

AU Diakowska, D

Nienartowicz, M

Grabowski, K

Rosinczuk, J

Krzystek-Korpacka, M

AF Diakowska, Dorota

Nienartowicz, Miroslaw

Grabowski, Krzysztof

Rosinczuk, Joanna

Krzystek-Korpacka, Malgorzata

TI Toll-like receptors TLR-2, TLR-4, TLR-7, and TLR-9 in tumor tissue and

serum of the patients with esophageal squamous cell carcinoma and

gastro-esophageal junction cancer

SO ADVANCES IN CLINICAL AND EXPERIMENTAL MEDICINE

LA English

DT Article

DE biomarkers; toll-like receptors; gastro-esophageal cancer

ID EXPRESSION; EPIDEMIOLOGY; PATHOGENESIS; PROGRESSION

AB Background. Stimulation of toll-like receptors (TLRs) has been linked to the development of esophageal and gastric cancers.

Objectives. The aim of the study was to evaluate the clinical significance of tissue expression and serum concentration of TLR-2, TLR-4, TLR-7, and TLR-9 in patients with esophageal squamous cell carcinoma and gastro-esophageal junction adenocarcinoma.

Material and methods. The study group consisted of 97 individuals: 32 with esophageal squamous cell carcinoma, 27 with gastro-esophageal junction cancer, and 38 age- and gender-matched controls. The mRNA expression and protein concentration of TLRs in tissues and sera were measured with reverse transcription polymerase chain reaction (RT-PCR) and enzyme-linked immunosorbent assay (ELISA) tests.

Results. In esophageal cancer patients, mRNA expressions of TLR-2, TLR-4 and TLR-7, and protein concentrations of all TLRs were significantly higher in tumor than in control tissue (p < 0.05). In esophageal cancer patients with lymph node metastasis, a tendency toward higher protein concentrations of tumor TLR-4 was observed. In gastro-esophageal junction adenocarcinoma subgroup, only the mRNA expression of TLR-7 and protein concentrations of TLR-4, TLR-7 and TLR-9 were significantly higher in tumors than in normal mucosa (p < 0.05). Protein concentration of TLR-9 was significantly higher in tumors of gastro-esophageal junction cancer with lymph node metastasis and depth of tumor invasion. Diagnostic potential of serum TLR-4 as a marker of gastro-esophageal junction cancer presence was reported.

Conclusions. We demonstrated differences in the expression patterns of TLRs between esophageal squamous cell carcinoma and adenocarcinoma of gastro-esophageal junction, and showed circulating TLR-4 to be a potential marker of gastro-esophageal junction cancer.

C1 [Diakowska, Dorota; Rosinczuk, Joanna] Wroclaw Med Univ, Fac Hlth Sci, Dept Nervous Syst Dis, Wroclaw, Poland.

[Nienartowicz, Miroslaw; Grabowski, Krzysztof] Wroclaw Med Univ, Dept Gastrointestinal & Gen Surg, Wroclaw, Poland.

[Krzystek-Korpacka, Malgorzata] Wroclaw Med Univ, Dept Med Biochem, Wroclaw, Poland.

RP Diakowska, D (corresponding author), Wroclaw Med Univ, Fac Hlth Sci, Dept Nervous Syst Dis, Wroclaw, Poland.

EM dorota.diakowska@umed.wroc.pl

RI Krzystek-Korpacka, Małgorzata/ABA-4556-2021

OI Diakowska, Dorota/0000-0002-1377-5601; Rosinczuk,

Joanna/0000-0001-7359-2287

CR Aggarwal R, 2015, GYNECOL ONCOL, V138, P358, DOI 10.1016/j.ygyno.2015.05.029

Avila M, 2011, IUBMB LIFE, V63, P873, DOI 10.1002/iub.555

Fernandez-Garcia B, 2014, J IMMUNOTHER, V37, P77, DOI 10.1097/CJI.0000000000000016

Ghosh A, 2015, EXP MOL PATHOL, V99, P720, DOI 10.1016/j.yexmp.2015.11.015

Helminen O, 2016, ONCOIMMUNOLOGY, V5, DOI 10.1080/2162402X.2015.1127495

Huhta H, 2016, ONCOTARGET, V7, P23658, DOI 10.18632/oncotarget.8151

Kauppila JH, 2014, FRONT IMMUNOL, V5, P1, DOI 10.3389/fimmu.2014.00200

Kauppila JH, 2011, HISTOPATHOLOGY, V59, P643, DOI 10.1111/j.1365-2559.2011.03991.x

Khan AA, 2016, ANN ONCOL, V27, P984, DOI 10.1093/annonc/mdw053

Lee CC, 2012, NAT REV IMMUNOL, V12, P168, DOI 10.1038/nri3151

Lin KJ, 2013, HUM PATHOL, V44, P534, DOI 10.1016/j.humpath.2012.06.015

Livak KJ, 2001, METHODS, V25, P402, DOI 10.1006/meth.2001.1262

Makinen LK, 2016, J ORAL PATHOL MED, V45, P338, DOI 10.1111/jop.12373

Makinen LK, 2015, ORAL ONCOL, V51, P96, DOI 10.1016/j.oraloncology.2014.08.017

Napier KJ, 2014, WORLD J GASTRO ONCOL, V6, P112, DOI 10.4251/wjgo.v6.i5.112

Ni YH, 2015, HISTOPATHOLOGY, V67, P730, DOI 10.1111/his.12703

Pandey S, 2015, CANCER GROWTH METAST, V8, P25, DOI 10.4137/CGM.S24314

Paulos CM, 2007, CLIN CANCER RES, V13, P5280, DOI 10.1158/1078-0432.CCR-07-1378

Pimentel-Nunes P, 2011, PATHOL ONCOL RES, V17, P677, DOI 10.1007/s12253-011-9368-9

Schmausser B, 2005, INT J MED MICROBIOL, V295, P179, DOI 10.1016/j.ijmm.2005.02.009

Sehdev A, 2013, DISCOV MED, V16, P103

Sheyhidin I, 2011, WORLD J GASTROENTERO, V17, P3745, DOI 10.3748/wjg.v17.i32.3745

Sobin LH, 2010, UICC INT UNION CANC, P63

Takala H, 2011, J INNATE IMMUN, V3, P631, DOI 10.1159/000329115

Vaz J, 2014, WORLD J GASTROENTERO, V20, P5808, DOI 10.3748/wjg.v20.i19.5808

Yang LY, 2012, CLIN CANCER RES, V18, P2138, DOI 10.1158/1078-0432.CCR-11-0934

Yuan X, 2013, CELL DEATH DIS, V4, DOI 10.1038/cddis.2013.334

Zaidi AH, 2016, BMC CANCER, V16, DOI 10.1186/s12885-016-2093-8

2010, MEDIAT INFLAMM

NR 29

TC 8

Z9 9

U1 1

U2 8

PU WROCLAW MEDICAL UNIV

PI WROCLAW

PA UL K MARCINKOWSKIEGO 2-6, WROCLAW, 50-368, POLAND

SN 1899-5276

EI 2451-2680

J9 ADV CLIN EXP MED

JI Adv. Clin. Exp. Med.

PD APR

PY 2019

VL 28

IS 4

BP 515

EP 522

DI 10.17219/acem/87012

PG 8

WC Medicine, Research & Experimental

WE Science Citation Index Expanded (SCI-EXPANDED)

SC Research & Experimental Medicine

GA HV9YM

UT WOS:000466338100010

PM 29968427

DA 2022-05-19

ER

PT J

AU Zhang, H

Ju, LL

Hu, PP

Ye, J

Yang, CL

Huang, JX

AF Zhang, Hao

Ju, Linling

Hu, Peipei

Ye, Jun

Yang, Canlin

Huang, Junxing

TI Circular RNA 0014715 Facilitates Cell Proliferation and Inhibits

Apoptosis in Esophageal Squamous Cell Carcinoma

SO CANCER MANAGEMENT AND RESEARCH

LA English

DT Article

DE circular RNA 0014715; circular RNAs; esophageal squamous cell carcinoma;

biological characteristics; cell proliferation

ID CANCER; METASTASIS

AB Background: Circular RNAs (circRNAs) have recently been verified to have multiple biological functions and participate in diverse biological processes in different malignant tumors, including esophageal squamous cell carcinoma (ESCC). Nonetheless, the function of circular RNA 0014715 (hsacirc_0014715, circ_0014715) in ESCC has not been described.

Materials and Methods: We investigated clinical data from sixty-seven patients undergoing surgery for esophageal cancer. The clinical data were collected. And we analyzed the correlation between the clinical characteristics of these patients and the expression of circ_0014715. Besides, we explored the expression of circ_0014715 in ESCC cell lines. We used cell counting kit-8, colony formation, transwell assay, and flow cytometry to detect changes in cell proliferation, migration, apoptosis, and cell cycle progression.

Results: We found that circ_0014715 was highly expressed in esophageal squamous cell carcinoma tissues and cell lines. The correlation analysis of clinicopathological features and gene expression revealed that high expression of circ_0014715 was related to nerve invasion, vascular invasion, more advanced tumor-node-metastasis (TNM) stage and poor differentiation grade. Receiver operating characteristic (ROC) curves revealed that circ_0014715 might have diagnostic value for ESCC. Experiments with cultured cells showed that knockdown of circ_0014715 significantly restrained cell proliferation, migration, invasion, wound healing and accelerated cell apoptosis. And cell cycle arrest at G2 phase was observed via flow cytometry. Overexpression of circ_0014715 caused the opposite effects. Collectively, these studies show that circ_0014715 is closely connected with the pathogenesis and development of ESCC. The excess expression of circ_0014715 may have promoting effects on the progression of esophageal cell carcinoma.

Conclusion: Our finding revealed that circ_0014715 promoted tumor growth and cell proliferation. All of these suggest that targeting circ_0014715 has potential therapeutic value in the treatment of ESCC.

C1 [Zhang, Hao; Yang, Canlin; Huang, Junxing] Nanjing Univ Chinese Med, Dept Oncol, Taizhou Peoples Hosp, 366 Taihu Rd, Taizhou 225300, Jiangsu, Peoples R China.

[Ju, Linling] Nantong Univ, Nantong Inst Liver Dis, Nantong Peoples Hosp 3, Nantong 226000, Jiangsu, Peoples R China.

[Hu, Peipei] Nanjing Univ Chinese Med, Nantong Hosp Tradit Chinese Med, Dept Pain Med, Nantong Hosp, Nantong 226000, Jiangsu, Peoples R China.

[Ye, Jun] Taizhou Peoples Hosp Affiliated Nanjing Univ Chin, Inst Clin Med, Taizhou 225300, Jiangsu, Peoples R China.

RP Huang, JX (corresponding author), Nanjing Univ Chinese Med, Dept Oncol, Taizhou Peoples Hosp, 366 Taihu Rd, Taizhou 225300, Jiangsu, Peoples R China.

EM hjxtz@sina.cn

FU Jiangsu Provincial Medical Innovation Team [CXTDA2017042]; Health

Committee of Nantong City [QB2020003, MA2020014]; Nantong Science and

Technology Bureau [JCZ20194]

FX This work is supported by the Jiangsu Provincial Medical Innovation Team

(Grant No. CXTDA2017042); the Health Committee of Nantong City (Grant

No. QB2020003; MA2020014); the Nantong Science and Technology Bureau

(Grant No. JCZ20194).

CR Arnaiz E, 2019, SEMIN CANCER BIOL, V58, P90, DOI 10.1016/j.semcancer.2018.12.002

Bray F, 2018, CA-CANCER J CLIN, V68, P394, DOI 10.3322/caac.21492

Chen J, 2015, DIS ESOPHAGUS, V28, P84, DOI 10.1111/dote.12088

Chen JL, 2019, THER ADV MED ONCOL, V11, DOI 10.1177/1758835919838958

Chen XQ, 2020, J EXP CLIN CANC RES, V39, DOI 10.1186/s13046-020-01640-9

Chen ZM, 2020, DIGEST DIS SCI, V65, P2272, DOI 10.1007/s10620-019-05973-0

Codipilly DC, 2018, GASTROINTEST ENDOSC, V88, P413, DOI 10.1016/j.gie.2018.04.2352

Hanahan D, 2011, CELL, V144, P646, DOI 10.1016/j.cell.2011.02.013

Hansen TB, 2013, NATURE, V495, P384, DOI 10.1038/nature11993

He YL, 2019, INT J BIOL MACROMOL, V125, P116, DOI 10.1016/j.ijbiomac.2018.11.273

Huang HR, 2019, CANCER BIOL THER, V20, P73, DOI 10.1080/15384047.2018.1507254

Jin G, 2020, NEOPLASMA, V67, P751, DOI 10.4149/neo_2020_190805N717

Kunzmann AT, 2018, EUR J GASTROEN HEPAT, V30, P263, DOI 10.1097/MEG.0000000000001029

Lin YH, 2018, INT J MOL SCI, V19, DOI 10.3390/ijms19123742

Liu L, 2019, HEPATOB PANCREAT DIS, V18, P580, DOI 10.1016/j.hbpd.2019.03.003

Rice TW, 2017, J THORAC ONCOL, V12, P36, DOI 10.1016/j.jtho.2016.10.016

Salzman J, 2013, PLOS GENET, V9, DOI 10.1371/journal.pgen.1003777

Shi F, 2019, BIOCHEM BIOPH RES CO, V510, P614, DOI 10.1016/j.bbrc.2019.02.019

Shi NM, 2019, J CELL BIOCHEM, V120, P10021, DOI 10.1002/jcb.28285

Siegel RL, 2018, CA-CANCER J CLIN, V68, P7, DOI 10.3322/caac.21442

Su HF, 2016, J TRANSL MED, V14, DOI 10.1186/s12967-016-0977-7

Teng HJ, 2018, THERANOSTICS, V8, P4345, DOI 10.7150/thno.26862

Ule J, 2019, MOL CELL, V76, P329, DOI 10.1016/j.molcel.2019.09.017

Wang JH, 2019, THORAC CANCER, V10, P78, DOI 10.1111/1759-7714.12904

Wang LQ, 2018, MOL CANCER, V17, DOI 10.1186/s12943-018-0889-7

Wei GH, 2021, GENE, V771, DOI 10.1016/j.gene.2020.145365

Wilusz JE, 2013, SCIENCE, V340, P440, DOI 10.1126/science.1238522

Xie J, 2019, J CELL MOL MED, V23, P3597, DOI 10.1111/jcmm.14260

Xu C, 2018, ONCOL REP, V40, P395, DOI 10.3892/or.2018.6450

Xu Y, 2019, AGING-US, V11, P1907, DOI 10.18632/aging.101872

Zhang HD, 2018, BREAST CANCER-TOKYO, V25, P1, DOI 10.1007/s12282-017-0793-9

NR 31

TC 1

Z9 1

U1 1

U2 1

PU DOVE MEDICAL PRESS LTD

PI ALBANY

PA PO BOX 300-008, ALBANY, AUCKLAND 0752, NEW ZEALAND

SN 1179-1322

J9 CANCER MANAG RES

JI Cancer Manag. Res.

PY 2021

VL 13

BP 4735

EP 4749

DI 10.2147/CMAR.S314882

PG 15

WC Oncology

WE Science Citation Index Expanded (SCI-EXPANDED)

SC Oncology

GA ST0PW

UT WOS:000662154300001

PM 34163248

OA Green Published, gold

DA 2022-05-19

ER

PT J

AU Chen, ZC

Zhang, CT

Pan, YQ

Xu, RP

Xu, CQ

Chen, ZP

Lu, ZM

Ke, Y

AF Chen, Zengchao

Zhang, Chaoting

Pan, Yaqi

Xu, Ruiping

Xu, Changqing

Chen, Ziping

Lu, Zheming

Ke, Yang

TI T cell receptor beta-chain repertoire analysis reveals intratumour

heterogeneity of tumour-infiltrating lymphocytes in oesophageal squamous

cell carcinoma

SO JOURNAL OF PATHOLOGY

LA English

DT Article

DE high-throughput sequencing; T cell receptors; intratumour heterogeneity;

tumour-infiltrating lymphocytes; oesophageal squamous cell carcinoma

ID CANCER; IMMUNOTHERAPY; EXPRESSION; BLOCKADE

AB Oesophageal squamous cell carcinoma (ESCC) has a generally poor prognosis, due to the lack of effective treatment methods. Immunotherapeutic approaches based on tumour-infiltrating lymphocytes (TILs) have demonstrated that durable responses are produced in some patients with solid tumours, which suggests the potential feasibility of clinical application of immunotherapy for ESCC. However, many of the basic characteristics of TILs in ESCC are poorly understood, including clonality, specificity and spatial heterogeneity of the response of TILs, which depends on the interaction between antigens and T cell receptors (TCRs). We used ultra-deep sequencing of rearranged genes in TCR beta-chain (TCR beta) to profile the basic characteristics of T cells in tumour tissues (four to six regions from each tumour) as well as matched adjacent normal tissue and peripheral blood from seven patients diagnosed with primary ESCC. We found that T cell clones within ESCCs were quite different from those of the peripheral blood and even the adjacent normal tissues in general. Although there was a relatively higher degree of overlap of intratumoural TCR beta repertoires than those between the tumour and other tissues, intratumoural TCR beta repertoires were spatially heterogeneous. Due to the restricted sampling, high-throughput TCR beta sequencing could characterize the diversity and composition of a limited (compartment-dependent) fraction of the respective T cell clones in any individual ESCC, expanding our understanding of immune behaviour and immune response and shedding more light on ESCC immunotherapy. Copyright (C) 2016 Pathological Society of Great Britain and Ireland. Published by John Wiley & Sons, Ltd.

C1 [Chen, Zengchao; Zhang, Chaoting; Pan, Yaqi; Lu, Zheming; Ke, Yang] Peking Univ, Key Lab Carcinogenesis & Translat Res, Minist Educ Beijing, Lab Genet,Canc Hosp & Inst, 52 Fucheng Rd, Beijing 100142, Peoples R China.

[Chen, Zengchao; Xu, Changqing; Chen, Ziping] Shandong Univ, Shandong Prov Qianfoshan Hosp, Dept Gastroenterol, Jinan, Peoples R China.

[Xu, Ruiping] Anyang Canc Hosp, Anyang, Henan, Peoples R China.

RP Lu, ZM; Ke, Y (corresponding author), Peking Univ, Key Lab Carcinogenesis & Translat Res, Minist Educ Beijing, Lab Genet,Canc Hosp & Inst, 52 Fucheng Rd, Beijing 100142, Peoples R China.

EM zheminglu@163.com; keyang@bjmu.edu.cn

FU Natural Science Foundation of China [81321003]; Beijing Municipal

Science and Technology [Z151100001615022, Z141100002114046]

FX This study was supported by grants from the Natural Science Foundation

of China (Grant No. 81321003) and Beijing Municipal Science and

Technology (Grant Nos Z151100001615022 and Z141100002114046).

CR Allum WH, 2009, J CLIN ONCOL, V27, P5062, DOI 10.1200/JCO.2009.22.2083

Chen WQ, 2015, CHINESE J CANCER RES, V27, P2, DOI 10.3978/j.issn.1000-9604.2015.01.06

Cho Y, 2003, CANCER RES, V63, P1555

Cooper ZA, 2013, ONCOIMMUNOLOGY, V2, DOI 10.4161/onci.26615

DAVIS MM, 1988, NATURE, V334, P395, DOI 10.1038/334395a0

Edge SB, 2010, ANN SURG ONCOL, V17, P1471, DOI 10.1245/s10434-010-0985-4

Emerson RO, 2013, J PATHOL, V231, P433, DOI 10.1002/path.4260

Gerlinger M, 2010, BRIT J CANCER, V103, P1139, DOI 10.1038/sj.bjc.6605912

Gerlinger M, 2013, J PATHOL, V231, P424, DOI 10.1002/path.4284

Gerlinger M, 2012, NEW ENGL J MED, V366, P883, DOI 10.1056/NEJMoa1113205

Gooden MJM, 2011, BRIT J CANCER, V105, P93, DOI 10.1038/bjc.2011.189

Han A, 2014, NAT BIOTECHNOL, V32, P684, DOI 10.1038/nbt.2938

Han YX, 2015, ONCOIMMUNOLOGY, V4, DOI 10.1080/2162402X.2015.1021537

Liaskou E, 2016, HEPATOLOGY, V63, P1608, DOI 10.1002/hep.28116

Mariette C, 2007, LANCET ONCOL, V8, P545, DOI 10.1016/S1470-2045(07)70172-9

Monod MY, 2004, BIOINFORMATICS, V20, P379, DOI 10.1093/bioinformatics/bth945

Ohigashi Y, 2005, CLIN CANCER RES, V11, P2947, DOI 10.1158/1078-0432.CCR-04-1469

Ott PA, 2013, CLIN CANCER RES, V19, P5300, DOI 10.1158/1078-0432.CCR-13-0143

Raufi AG, 2015, J GASTROINTEST ONCOL, V6, P561, DOI 10.3978/j.issn.2078-6891.2015.037

Robins H, 2012, J IMMUNOL METHODS, V375, P14, DOI 10.1016/j.jim.2011.09.001

Robins HS, 2009, BLOOD, V114, P4099, DOI 10.1182/blood-2009-04-217604

Rosenberg SA, 2015, SCIENCE, V348, P62, DOI 10.1126/science.aaa4967

Sherwood AM, 2013, CANCER IMMUNOL IMMUN, V62, P1453, DOI 10.1007/s00262-013-1446-2

Stevanovic S, 2015, J CLIN ONCOL, V33, P1543, DOI 10.1200/JCO.2014.58.9093

Swaika A, 2015, MOL IMMUNOL, V67, P4, DOI 10.1016/j.molimm.2015.02.009

TAMURA K, 1993, MOL BIOL EVOL, V10, P512, DOI [10.1093/molbev/mst197, 10.1093/oxfordjournals.molbev.a040023]

Torre LA, 2015, CA-CANCER J CLIN, V65, P87, DOI 10.3322/caac.21262

Tran E, 2014, SCIENCE, V344, P641, DOI 10.1126/science.1251102

Tsuchikawa T, 2011, CLIN EXP IMMUNOL, V164, P50, DOI 10.1111/j.1365-2249.2010.04311.x

Tumeh PC, 2014, NATURE, V515, P568, DOI 10.1038/nature13954

Verdegaal EME, 2011, CANCER IMMUNOL IMMUN, V60, P953, DOI 10.1007/s00262-011-1004-8

Warren RL, 2011, GENOME RES, V21, P790, DOI 10.1101/gr.115428.110

NR 32

TC 50

Z9 52

U1 0

U2 6

PU WILEY

PI HOBOKEN

PA 111 RIVER ST, HOBOKEN 07030-5774, NJ USA

SN 0022-3417

EI 1096-9896

J9 J PATHOL

JI J. Pathol.

PD AUG

PY 2016

VL 239

IS 4

BP 450

EP 458

DI 10.1002/path.4742

PG 9

WC Oncology; Pathology

WE Science Citation Index Expanded (SCI-EXPANDED)

SC Oncology; Pathology

GA DW4EL

UT WOS:000383595200008

PM 27171315

DA 2022-05-19

ER

PT J

AU Xu, ZJ

Zhuo, ZG

Song, TN

Alai, GH

Shen, X

Peng-Yao

Lin, YD

AF Xu, Zhi-Jie

Zhuo, Ze-Guo

Song, Tie-Niu

Alai, Gu-Ha

Shen, Xu

Peng-Yao

Lin, Yi-Dan

TI Role of nodal skip metastasis in patients with mid-thoracic oesophageal

squamous cell carcinoma: a propensity score matching study

SO EUROPEAN JOURNAL OF CARDIO-THORACIC SURGERY

LA English

DT Article

DE Nodal skip metastasis; Mid-thoracic oesophageal squamous cell carcinoma;

Prognosis; Propensity score matching

ID CANCER; PATTERN; CHINA

AB OBJECTIVES: Nodal skip metastasis (NSM) is a common phenomenon in mid-thoracic oesophageal squamous cell carcinoma (MT-OSCC); however, the prognostic implications of NSM in patients with MT-OSCC remain unclear.

METHODS: This retrospective study enrolled 300 patients with MT-OSCC who underwent radical oesophagectomy and who had pathologically confirmed lymph node metastasis from January 2014 to December 2016. The patients were divided into 2 groups according to the presence or absence of NSM. Propensity score matching was applied to minimize patient selection bias. The impact of NSM on overall survival (OS) was assessed by Kaplan-Meier and multiple Cox proportional hazards analyses. The median follow-up time was 57 months.

RESULTS: The NSM rate in the entire cohort was 22.0% (66/300). Pathological N (pN) stage (P < 0.001) and sex (P= 0.001) were identified as significant independent risk factors for NSM. NSM was more frequent in pN1 compared with pN2 patients (87.9% vs 12.1%, P < 0.001) and no NSM was found in pN3. NSM(+) patients had better prognoses than NSM(-) patients (Kaplan-Meier; 3-year OS, 62.1% vs 34.1%, P < 0.001). Propensity score matching produced 51 matched pairs, and the 3-year OS was still better in the NSM(+) compared with the NSM(-) group (66.7% vs 40.0%, P = 0.025). Multivariable Cox analysis confirmed NSM(+) as an independent factor favouring OS in patients with MT-OSCC.

CONCLUSIONS: NSM usually occurs at pN1 stage in patients with MT-OSCC, and is associated with a favourable prognosis.

C1 [Xu, Zhi-Jie; Zhuo, Ze-Guo; Song, Tie-Niu; Alai, Gu-Ha; Shen, Xu; Peng-Yao; Lin, Yi-Dan] Sichuan Univ, West China Hosp, Dept Thorac Surg, 37 Guoxue Alley, Chengdu 610041, Sichuan, Peoples R China.

RP Lin, YD (corresponding author), Sichuan Univ, West China Hosp, Dept Thorac Surg, 37 Guoxue Alley, Chengdu 610041, Sichuan, Peoples R China.

EM linyidan.academy@foxmail.com

FU National Natural Science Foundation of China [81672291, 31071210]

FX This work was supported by the National Natural Science Foundation of

China [81672291, 31071210 to Y.-D.L.].

CR Asamura H, 2015, J THORAC ONCOL, V10, P1675, DOI 10.1097/JTO.0000000000000678

Cavallin F, 2017, BMC SURG, V17, DOI 10.1186/s12893-017-0247-5

Chen JQ, 2009, EUR J CARDIO-THORAC, V36, P480, DOI 10.1016/j.ejcts.2009.03.056

Feng RM, 2019, CANCER COMMUN, V39, DOI 10.1186/s40880-019-0368-6

He SL, 2019, ANN THORAC SURG, V108, P1717, DOI 10.1016/j.athoracsur.2019.03.081

Ho FCH, 2012, BMC CANCER, V12, DOI 10.1186/1471-2407-12-98

Hu K, 2019, CANCER SCI, V110, P717, DOI 10.1111/cas.13891

Japan Esophageal Soc, 2017, ESOPHAGUS-TOKYO, V14, P1, DOI 10.1007/s10388-016-0551-7

Kuge K, 2003, J THORAC CARDIOV SUR, V125, P1343, DOI 10.1016/S0022-5223(03)00036-9

Kumakura Y, 2018, ANN SURG ONCOL, V25, P1221, DOI 10.1245/s10434-018-6390-0

Liu J, 2016, EJSO-EUR J SURG ONC, V42, P1202, DOI 10.1016/j.ejso.2016.05.025

Machens A, 2004, ARCH SURG-CHICAGO, V139, P43, DOI 10.1001/archsurg.139.1.43

Prenzel KL, 2010, ANN THORAC SURG, V90, P1662, DOI 10.1016/j.athoracsur.2010.07.008

Qiu ML, 2019, BMC CANCER, V19, DOI 10.1186/s12885-019-6191-2

Rice TW, 2017, J THORAC ONCOL, V12, P36, DOI 10.1016/j.jtho.2016.10.016

Sato Harunobu, 2019, Gan To Kagaku Ryoho, V46, P2048

Siegel RL, 2019, CA-CANCER J CLIN, V69, P7, DOI 10.3322/caac.21551

Song G, 2017, ONCOTARGETS THER, V10, P2729, DOI 10.2147/OTT.S132062

Tachimori Y, 2011, DIS ESOPHAGUS, V24, P33, DOI 10.1111/j.1442-2050.2010.01086.x

Wang F, 2017, ANN THORAC SURG, V104, P1187, DOI 10.1016/j.athoracsur.2017.03.081

Wu J, 2012, WORLD J SURG ONCOL, V10, DOI 10.1186/1477-7819-10-210

Yang YS, 2018, SURGERY, V164, P1130, DOI 10.1016/j.surg.2018.04.023

Zhu ZF, 2013, ANN SURG ONCOL, V20, P3052, DOI 10.1245/s10434-013-2987-5

NR 23

TC 3

Z9 3

U1 4

U2 9

PU OXFORD UNIV PRESS INC

PI CARY

PA JOURNALS DEPT, 2001 EVANS RD, CARY, NC 27513 USA

SN 1010-7940

EI 1873-734X

J9 EUR J CARDIO-THORAC

JI Eur. J. Cardio-Thorac. Surg.

PD APR

PY 2021

VL 59

IS 4

BP 799

EP 806

DI 10.1093/ejcts/ezaa371

PG 8

WC Cardiac & Cardiovascular Systems; Respiratory System; Surgery

WE Science Citation Index Expanded (SCI-EXPANDED)

SC Cardiovascular System & Cardiology; Respiratory System; Surgery

GA SE1LC

UT WOS:000651835600011

PM 33249483

OA Bronze

DA 2022-05-19

ER

PT J

AU Huang, J

Xiao, JX

Fang, WT

Lu, P

Fan, QX

Shu, YQ

Feng, JF

Zhang, S

Ba, Y

Zhao, Y

Liu, Y

Bai, CM

Bai, YX

Tang, Y

Song, Y

He, J

AF Huang, Jing

Xiao, Juxiang

Fang, Wentao

Lu, Ping

Fan, Qingxia

Shu, Yongqian

Feng, Jifeng

Zhang, Shu

Ba, Yi

Zhao, Yang

Liu, Ying

Bai, Chunmei

Bai, Yuxian

Tang, Yong

Song, Yan

He, Jie

TI Anlotinib for previously treated advanced or metastatic esophageal

squamous cell carcinoma: A double-blind randomized phase 2 trial

SO CANCER MEDICINE

LA English

DT Article

DE adverse events; anlotinib; esophageal squamous cell carcinoma;

metastatic; progression&#8208; free survival

AB Background Currently, there are no randomized trials on the effect of antiangiogenic therapy in patients with esophageal squamous cell carcinoma (ESCC). The following study investigated the efficacy and safety of anlotinib in patients with advanced ESCC who were previously treated with chemotherapy.

Methods This randomized, placebo-controlled, double-blind phase 2 trial (NCT02649361) was conducted in 13 Chinese hospitals. Eligible patients were adults with histologically confirmed recurrent or metastatic ESCC who were previously treated with chemotherapy, and were randomly assigned (2:1) to receive oral anlotinib 12 mg or placebo on days 1-14 (repeated every 21 days). The primary endpoint was progression-free survival (PFS).

Results One hundred and sixty-five patients were randomly assigned to the anlotinib (n = 110) or the placebo (n = 55) arm. Median PFS was 3.02 months (95% CI 2.63-3.65) in the anlotinib group and 1.41 months (95% CI 1.38-1.41) in the placebo group (hazard ratio 0.46 [95% CI 0.32-0.66]; p < 0.001). The most common treatment-related adverse events of grade 3 or 4 were hypertension (17 [16%] patients), decreased appetite (6 [6%] patients), and hyponatremia (4 [4%] patients) in the anlotinib group and decreased appetite (2 [4%] patients) in the placebo group. Three (3%) deaths in the anlotinib group were considered as drug related, while there were no treatment-related deaths in the placebo group.

Conclusions The use of anlotinib in previously treated, recurrent, or metastatic ESCC patients significantly improved PFS compared with placebo. Our findings suggest that antiangiogenesis might be an important therapeutic target in advanced ESCC.

Clinical Trials Registration Study of Anlotinib in Patients With Esophageal Squamous Cell Carcinoma (ALTER1102), NCT02649361.

C1 [Huang, Jing; Song, Yan] Chinese Acad Med Sci & Peking Union Med Coll, Canc Hosp, Natl Clin Res Ctr Canc, Dept Med Oncol,Natl Canc Ctr, Beijing 100021, Peoples R China.

[Xiao, Juxiang] Xi An Jiao Tong Univ, Affiliated Hosp 1, Dept Med Oncol, Xian, Peoples R China.

[Fang, Wentao] Shanghai Jiao Tong Univ, Shanghai Chest Hosp, Dept Thorac Surg, Shanghai, Peoples R China.

[Lu, Ping] Xinxiang Med Univ, Affiliated Hosp 1, Dept Oncol, Xinxiang, Henan, Peoples R China.

[Fan, Qingxia] Zhengzhou Univ, Affiliated Hosp 1, Dept Oncol, Zhengzhou, Peoples R China.

[Shu, Yongqian] Nanjing Med Univ, Affiliated Hosp 1, Dept Med Oncol, Nanjing, Peoples R China.

[Feng, Jifeng] Nanjing Med Univ, Affiliated Canc Hosp, Jiangsu Canc Hosp, Jiangsu Inst Canc Res,Dept Med Oncol, Nanjing, Peoples R China.

[Zhang, Shu] Shandong Canc Hosp, Dept Med Oncol, Jinan, Peoples R China.

[Ba, Yi] Tianjin Canc Hosp, Dept Med Oncol, Tianjin, Peoples R China.

[Zhao, Yang] Nanjing Med Univ, Sch Publ Hlth, Dept Biostat, Nanjing, Peoples R China.

[Liu, Ying] Henan Canc Hosp, Dept Med Oncol, Zhengzhou, Peoples R China.

[Bai, Chunmei] Chinese Acad Med Sci & Peking Union Med Coll, Peking Union Med Coll Hosp, Dept Med Oncol, Beijing, Peoples R China.

[Bai, Yuxian] Harbin Med Univ Canc Hosp, Dept Med Oncol, Harbin, Peoples R China.

[Tang, Yong] Xinjiang Med Univ, Affiliated Tumor Hosp, Dept Gastroenterol, Urumqi, Peoples R China.

[He, Jie] Chinese Acad Med Sci & Peking Union Med Coll, Canc Hosp, Natl Clin Res Ctr Canc, Natl Canc Ctr,Dept Thorac Surg Oncol, Beijing 100021, Peoples R China.

RP Huang, J (corresponding author), Chinese Acad Med Sci & Peking Union Med Coll, Canc Hosp, Natl Clin Res Ctr Canc, Dept Med Oncol,Natl Canc Ctr, Beijing 100021, Peoples R China.; He, J (corresponding author), Chinese Acad Med Sci & Peking Union Med Coll, Canc Hosp, Natl Clin Res Ctr Canc, Natl Canc Ctr,Dept Thorac Surg Oncol, Beijing 100021, Peoples R China.

EM huangjingwg@163.com; hejie@cicams.ac.cn

RI Shu, Yongqian/ABD-5698-2021

OI Huang, Jing/0000-0002-2167-8385

FU Chia Tai Tianqing Pharmaceutical Group Co., Ltd (Lianyungang, Jiangsu

Province, China)

FX This work was supported by Chia Tai Tianqing Pharmaceutical Group Co.,

Ltd (Lianyungang, Jiangsu Province, China). The sponsor provided the

study drugs. The sponsor had no role in the study design and conduct of

the study; collection, management, analysis, and interpretation of the

data; preparation, review, or approval of the manuscript; and decision

to submit the manuscript for publication.

CR Aghajanian C, 2012, J CLIN ONCOL, V30, P2039, DOI 10.1200/JCO.2012.42.0505

Argiris A, 2011, J CLIN ONCOL, V29, P1140, DOI 10.1200/JCO.2010.33.3591

Bray F, 2018, CA-CANCER J CLIN, V68, P394, DOI 10.3322/caac.21492

Chen DS, 2018, CANCER J, V24, P193, DOI 10.1097/PPO.0000000000000327

Chi YHBL, 2018, CLIN CANCER RES, V24, P5233, DOI 10.1158/1078-0432.CCR-17-3766

Di Pardo BJ, 2016, WORLD J SURG, V40, P395, DOI 10.1007/s00268-015-3356-2

Dutton SJ, 2014, LANCET ONCOL, V15, P894, DOI 10.1016/S1470-2045(14)70024-5

Han BH, 2018, JAMA ONCOL, V4, P1569, DOI 10.1001/jamaoncol.2018.3039

Han BH, 2018, BRIT J CANCER, V118, P654, DOI 10.1038/bjc.2017.478

Hayashi K, 2001, JPN J CLIN ONCOL, V31, P419, DOI 10.1093/jjco/hye090

Huang J, 2020, LANCET ONCOL, V21, P832, DOI 10.1016/S1470-2045(20)30110-8

Huang J, 2016, J THORAC ONCOL, V11, P910, DOI 10.1016/j.jtho.2016.02.020

Huang J, 2013, MED ONCOL, V30, DOI 10.1007/s12032-012-0343-4

Kato K, 2019, LANCET ONCOL, V20, P1506, DOI 10.1016/S1470-2045(19)30626-6

Keating GM, 2014, DRUGS, V74, P1891, DOI 10.1007/s40265-014-0302-9

Kojima T, 2019, J CLIN ONCOL, V37, DOI 10.1200/JCO.2019.37.4_suppl.2

Sun YK, 2018, THYROID, V28, P1455, DOI 10.1089/thy.2018.0022

Syed YY, 2018, DRUGS, V78, P1057, DOI 10.1007/s40265-018-0939-x

Tewari KS, 2014, NEW ENGL J MED, V370, P734, DOI 10.1056/NEJMoa1309748

NR 19

TC 6

Z9 7

U1 0

U2 1

PU WILEY

PI HOBOKEN

PA 111 RIVER ST, HOBOKEN 07030-5774, NJ USA

SN 2045-7634

J9 CANCER MED-US

JI Cancer Med.

PD MAR

PY 2021

VL 10

IS 5

BP 1681

EP 1689

DI 10.1002/cam4.3771

EA FEB 2021

PG 9

WC Oncology

WE Science Citation Index Expanded (SCI-EXPANDED)

SC Oncology

GA QT0GO

UT WOS:000617804500001

PM 33586360

OA Green Published, gold

DA 2022-05-19

ER

PT J

AU Liu, GP

Zeng, T

AF Liu, Guiping

Zeng, Tao

TI Sporoderm-Removed Ganoderma lucidum Spore Powder May Suppress the

Proliferation, Migration, and Invasion of Esophageal Squamous Cell

Carcinoma Cells Through PI3K/AKT/mTOR and Erk Pathway

SO INTEGRATIVE CANCER THERAPIES

LA English

DT Article

DE sporoderm-removed Ganoderma lucidum spore powder; esophageal squamous

cell carcinomas; cell proliferation; cell cycle; cell apoptosis; cell

migration; cell invasion

ID EPITHELIAL-MESENCHYMAL TRANSITION; CANCER-CELLS; BROKEN SPORES;

UP-REGULATION; CYCLE ARREST; LUNG-CANCER; APOPTOSIS; INHIBITION;

TRANSCRIPTION; ANGIOGENESIS

AB Tumor metastasis is a key factor of therapeutic failure in tumor patients, but the underlying molecular mechanism remains to be explored and novel effective curative strategies are urgently required. Emerging evidence suggests that sporoderm-removed Ganoderma lucidum spore powder can suppress tumor growth and metastasis. However, the molecular mechanisms of action remain elusive. In the present study, we investigated the effects and mechanisms of sporoderm-removed Ganoderma lucidum spore powder against esophageal squamous cell carcinomas (ESCC). The expression of MCP-1 in esophageal squamous cell carcinoma cells was detected by Western blotting. The MTS assay was used to assess the esophageal squamous cell carcinoma cells viability. The clone formation assay was used to evaluate to the proliferation ability of KYSE140 and KYSE510 cells. Apoptosis and the cell cycle were analyzed by flow cytometry. Wound healing and Transwell assays were used to analyze the migration of KYSE140 and KYSE510 cells. Invasion was also analyzed by the Transwell assay. The expressions of PI3K, AKT/p-AKT, Erk/p-Erk, JNK1, and mTOR were detected by Western blotting. We found that the MCP-1 protein was highly expressed in KYSE140 and KYSE510. In addition, sporoderm-removed Ganoderma lucidum spore powder treatment was found to inhibit esophageal squamous cell carcinoma cell proliferation, to block the cell cycle, to induce cell apoptosis and to inhibit cell migration and invasion. Finally, we found that sporoderm-removed Ganoderma lucidum spore powder decreased the expression of PI3K/AKT/mTOR and Erk signaling pathways. Taken together, these findings demonstrate that sporoderm-removed Ganoderma lucidum spore powder suppresses esophageal squamous cell carcinomas by involving MCP-1, regulated by PI3K/AKT/mTOR and Erk signal pathways.

C1 [Liu, Guiping; Zeng, Tao] Guangdong Med Univ, Dept Med Lab, Affiliated Hosp, 57 South Peoples Ave, Zhanjiang 524000, Guangdong, Peoples R China.

[Liu, Guiping] Peking Univ Shenzhen Hosp, Dept Lab Med, Shenzhen, Guangdong, Peoples R China.

RP Zeng, T (corresponding author), Guangdong Med Univ, Dept Med Lab, Affiliated Hosp, 57 South Peoples Ave, Zhanjiang 524000, Guangdong, Peoples R China.

EM zengt@smu.edu.cn

FU Start-up Fund for High-level Talents in Affiliated Hospital of Guangdong

Medical University [51301Z20200007]; Discipline construction project of

Guangdong Medical University [4SG21266P, 4SG21276P]

FX The author(s) disclosed receipt of the following financial support for

the research, authorship, and/or publication of this article: This work

was supported by the Start-up Fund for High-level Talents in Affiliated

Hospital of Guangdong Medical University (grant No. 51301Z20200007) and

Discipline construction project of Guangdong Medical University (grant

No. 4SG21266P and grant No. 4SG21276P) to TZ. The funders had no role in

study design, data collection and analysis, manuscript preparation, or

decision to publish.

CR Bianconi V, 2018, CURR OPIN HEMATOL, V25, P44, DOI 10.1097/MOH.0000000000000389

Bray F, 2018, CA-CANCER J CLIN, V68, P394, DOI 10.3322/caac.21492

Brown GT, 2015, J PATHOL, V237, P273, DOI 10.1002/path.4586

Chen YL, 2016, NUTR CANCER, V68, P1151, DOI 10.1080/01635581.2016.1208832

Chen Y, 2021, J ONCOL, V2021, DOI 10.1155/2021/1384289

Cichon MA, 2015, CANCER INFORM, V14, P1, DOI 10.4137/CIN.S18965

Costa C, 2015, PLOS ONE, V10, DOI 10.1371/journal.pone.0141253

Evan GI, 2001, NATURE, V411, P342, DOI 10.1038/35077213

Guo ZL, 2018, BMC CELL BIOL, V19, DOI 10.1186/s12860-018-0179-7

Houede N, 2015, PHARMACOL THERAPEUT, V145, P1, DOI 10.1016/j.pharmthera.2014.06.004

Huang T, 2019, J ANIM SCI BIOTECHNO, V10, DOI 10.1186/s40104-019-0361-6

International Agency for Research on Cancer, LAT GLOB CANC DAT CA

Ji WT, 2014, PLOS ONE, V9, DOI 10.1371/journal.pone.0088952

Jiang M, 1997, INT J CANCER, V74, P529, DOI 10.1002/(SICI)1097-0215(19971021)74:5<529::AID-IJC9>3.0.CO;2-5

Jin D, 2015, BRAIN RES, V1599, P158, DOI 10.1016/j.brainres.2014.12.043

Koga M, 2008, BIOCHEM BIOPH RES CO, V365, P279, DOI 10.1016/j.bbrc.2007.10.182

Lee CC, 2015, ANTICANCER RES, V35, P3299

Li DB, 2020, CANCER SCI, V111, P47, DOI 10.1111/cas.14230

Li K, 2017, ONCOL REP, V38, P2803, DOI 10.3892/or.2017.6010

Li L, 2016, PHYTOMEDICINE, V23, P491, DOI 10.1016/j.phymed.2016.02.019

Li ZH, 2020, FRONT PHARMACOL, V11, DOI 10.3389/fphar.2020.00287

Li ZL, 2021, CONNECT TISSUE RES, V62, P337, DOI 10.1080/03008207.2020.1736578

Liss C, 2002, INT J CANCER, V100, P283, DOI 10.1002/ijc.10507

Matsushima M, 2015, INT J ONCOL, V47, P377, DOI 10.3892/ijo.2015.2995

Nagoya H, 2014, AM J PHYSIOL-GASTR L, V306, pG183, DOI 10.1152/ajpgi.00057.2013

Pennathur A, 2013, LANCET, V381, P400, DOI 10.1016/S0140-6736(12)60643-6

Raza U, 2016, ONCOTARGET, V7, P49859, DOI 10.18632/oncotarget.10489

Shen H, 2019, GENE, V698, P50, DOI 10.1016/j.gene.2019.02.044

Singh SK, 2017, ONCOTARGET, V8, P17216, DOI 10.18632/oncotarget.15303

Talbert EE, 2018, J CACHEXIA SARCOPENI, V9, P358, DOI 10.1002/jcsm.12251

Thyagarajan A, 2010, NUTR CANCER, V62, P630, DOI 10.1080/01635580903532390

Timofeeva OA, 2013, P NATL ACAD SCI USA, V110, P1267, DOI 10.1073/pnas.1211805110

Trivedi R, 2014, CELL DEATH DIS, V5, DOI 10.1038/cddis.2014.429

Wang QH, 2016, EXP MOL PATHOL, V100, P514, DOI 10.1016/j.yexmp.2016.05.001

Wei Y, 2016, MOLECULES, V21, DOI 10.3390/molecules21060777

Wu GS, 2012, AM J CHINESE MED, V40, P631, DOI 10.1142/S0192415X12500474

Xie BW, 2019, CANCER LETT, V443, P56, DOI 10.1016/j.canlet.2018.11.019

Yang H, 2020, MOL CANCER, V19, DOI 10.1186/s12943-020-01165-x

Yoshimura T, 2013, PLOS ONE, V8, DOI 10.1371/journal.pone.0058791

Zhang H, 2010, J INT MED RES, V38, P1673, DOI 10.1177/147323001003800512

Zhang XQ, 2019, FOOD SCI NUTR, V7, P3403, DOI 10.1002/fsn3.1154

Zhao GY, 2016, AGING-US, V8, P2308, DOI 10.18632/aging.101038

Zhao ZH, 2017, MOL MED REP, V16, P2281, DOI 10.3892/mmr.2017.6841

Zhong JY, 2021, ONCOL LETT, V21, DOI 10.3892/ol.2021.12686

NR 44

TC 0

Z9 0

U1 5

U2 5

PU SAGE PUBLICATIONS INC

PI THOUSAND OAKS

PA 2455 TELLER RD, THOUSAND OAKS, CA 91320 USA

SN 1534-7354

EI 1552-695X

J9 INTEGR CANCER THER

JI Integr. Cancer Ther.

PD NOV

PY 2021

VL 20

AR 15347354211062157

DI 10.1177/15347354211062157

PG 13

WC Oncology; Integrative & Complementary Medicine

WE Science Citation Index Expanded (SCI-EXPANDED)

SC Oncology; Integrative & Complementary Medicine

GA XJ4CV

UT WOS:000726739100001

PM 34841952

OA Green Published, gold

DA 2022-05-19

ER

PT J

AU Wu, IC

Wang, YK

Chen, YH

Wu, CC

Wu, MC

Chen, WC

Wang, WL

Lin, HS

Chen, CC

Chou, SH

Liu, YP

Wu, MT

AF Wu, I-Chen

Wang, Yao-Kuang

Chen, Yi-Hsun

Wu, Chun-Chieh

Wu, Meng-Chieh

Chen, Wei-Chung

Wang, Wen-Lun

Lin, Hung-Shun

Chen, Chou-Cheng

Chou, Shah-Hwa

Liu, Yu-Peng

Wu, Ming-Tsang

TI High Serum Elafin Prediction of Poor Prognosis of Locoregional

Esophageal Squamous Cell Carcinoma

SO CANCERS

LA English

DT Article

DE esophageal squamous cell carcinoma; elafin; PI3; circulating markers

ID PROTEASE INHIBITOR; IMMUNOHISTOCHEMICAL EXPRESSION; ELASTASE INHIBITOR;

BREAST; SLPI; SKALP/ELAFIN

AB Simple Summary Conventional serum markers such as carcinoembryonic antigen (CEA), squamous cell carcinoma antigen (SCC), and tissue polypeptide antigen (TPA) have a low sensitivity in predicting the prognosis of locoregional esophageal squamous cell carcinoma cell (ESCC). In our clinical study, we found high serum elafin to be an independent outcome predictor for stage I-IIIA ESCC, considering T, N, overall stage, and treatment. In vitro experiments showed that adding recombinant elafin drove ESCC cell proliferation, migration and invasion, while shRNA attenuated elafin levels, abrogating those effects. Our results suggested serum elafin might be a noninvasive biomarker to predict the outcome of locoregional ESCC and could potentially be used as a therapeutic target. Esophageal squamous cell carcinoma (ESCC) is a highly aggressive tumor known to have locally advanced and metastatic features which cause a dismal prognosis. We sought to determine whether elafin, a non-invasive and secretory small-molecule marker, could be used to predict prognosis in locoregional ESCC patients in human and in vitro studies. In our human study, 119 subjects were identified as having incident and pathologically-proved ESCC with stage I-IIIA tumors from southern Taiwan between 2000 and 2016. We measured their serum elafin levels at baseline and followed them until the date of cancer death or until January 2020, the end of this study. Those with high serum elafin levels were found to have a 1.99-fold risk (95% confidence interval: 1.17-3.38) shorter survival than those who did not. In our in vitro experiments, elevated elafin levels were found to drive ESCC cell proliferation, migration and invasion, while attenuation of elafin level by shRNA abrogated those effects. We concluded that elafin promotes ESCC motility and invasion and leads to a worse clinical prognosis in ESCC patients without distant metastasis.

C1 [Wu, I-Chen; Wang, Yao-Kuang; Chen, Yi-Hsun] Kaohsiung Med Univ Hosp, Div Gastroenterol, Dept Internal Med, Kaohsiung 807, Taiwan.

[Wu, I-Chen; Wang, Yao-Kuang; Wu, Chun-Chieh; Chou, Shah-Hwa] Kaohsiung Med Univ, Dept Med, Coll Med, Fac Med, Kaohsiung 807, Taiwan.

[Wu, Chun-Chieh] Kaohsiung Med Univ, Kaohsiung Med Univ Hosp, Dept Pathol, Kaohsiung 807, Taiwan.

[Wu, Meng-Chieh] Kaohsiung Municipal Tatung Hosp, Dept Internal Med, Kaohsiung 807, Taiwan.

[Chen, Wei-Chung; Chen, Chou-Cheng; Liu, Yu-Peng; Wu, Ming-Tsang] Kaohsiung Med Univ, PhD Program Environm & Occupat Med, Kaohsiung 807, Taiwan.

[Chen, Wei-Chung; Wu, Ming-Tsang] Kaohsiung Med Univ, Res Ctr Environm Med, Kaohsiung 807, Taiwan.

[Wang, Wen-Lun] I Shou Univ, Div Gastroenterol & Hepatol, Dept Internal Med, E DA Hosp, Kaohsiung 824, Taiwan.

[Lin, Hung-Shun] Kaohsiung Med Univ, Kaohsiung Municipal Siaogang Hosp, Dept Lab Med, Kaohsiung 807, Taiwan.

[Lin, Hung-Shun] Kaohsiung Med Univ, Kaohsiung Municipal Siaogang Hosp, Dept Res Educ & Training, Kaohsiung 807, Taiwan.

[Lin, Hung-Shun; Wu, Ming-Tsang] Kaohsiung Med Univ, Dept Publ Hlth, Kaohsiung 807, Taiwan.

[Chou, Shah-Hwa] Kaohsiung Med Univ, Kaohsiung Med Univ Hosp, Div Chest Surg, Dept Surg, Kaohsiung 807, Taiwan.

[Liu, Yu-Peng; Wu, Ming-Tsang] Kaohsiung Med Univ, Grad Inst Clin Med, Kaohsiung 807, Taiwan.

[Wu, Ming-Tsang] Kaohsiung Med Univ, Kaohsiung Med Univ Hosp, Dept Family Med, Kaohsiung 807, Taiwan.

[Wu, Ming-Tsang] Natl Sun Yat Sen Univ, Rapid Screening Res Ctr Toxicol & Biomed, Kaohsiung 807, Taiwan.

RP Liu, YP; Wu, MT (corresponding author), Kaohsiung Med Univ, PhD Program Environm & Occupat Med, Kaohsiung 807, Taiwan.; Wu, MT (corresponding author), Kaohsiung Med Univ, Res Ctr Environm Med, Kaohsiung 807, Taiwan.; Wu, MT (corresponding author), Kaohsiung Med Univ, Dept Publ Hlth, Kaohsiung 807, Taiwan.; Liu, YP; Wu, MT (corresponding author), Kaohsiung Med Univ, Grad Inst Clin Med, Kaohsiung 807, Taiwan.; Wu, MT (corresponding author), Kaohsiung Med Univ, Kaohsiung Med Univ Hosp, Dept Family Med, Kaohsiung 807, Taiwan.; Wu, MT (corresponding author), Natl Sun Yat Sen Univ, Rapid Screening Res Ctr Toxicol & Biomed, Kaohsiung 807, Taiwan.

EM minica@kmu.edu.tw; 970395@kmuh.org.tw; 1020420@kmuh.org.tw;

wucchieh@kmu.edu.tw; 930293@mail.kmuh.org.tw; u103803001@kmu.edu.tw;

ed102807@edah.org.tw; 930570@kmhk.org.tw; new.purple@ctbc.edu.tw;

shhwch@kmu.edu.tw; ypliu@kmu.edu.tw; 960021@cc.kmuh.org.tw

OI Chen, Yi-Hsun/0000-0001-9981-5143; Wang, Yao-Kuang/0000-0002-5536-7542;

Liu, Yu-Peng/0000-0002-2716-0050

FU Kaohsiung Medical University Hospital in Taiwan [KMUH109-9R02];

Kaohsiung Medical University in Taiwan [NCTUKMU108-AI-06-2]; Featured

Areas Research Center Program in Taiwan [KMU-TC109A01]; Ministry of

Science and Technology in Taiwan [MOST109-2628-B-037-005-,

109-2314B-037-066, 109-2314-B-037-072, 110-2321-B-037-002]; National

Health Research Institutes in Taiwan [NHRI-EX110-10703PI]

FX This work was financially supported by the grants from Kaohsiung Medical

University Hospital (KMUH109-9R02), Kaohsiung Medical University

(NCTUKMU108-AI-06-2); from the Featured Areas Research Center Program

within the framework of the Higher Education Sprout Project by the

Ministry of Education (MOE), Kaohsiung Medical University Research

Center Grant (KMU-TC109A01); from Ministry of Science and Technology

(MOST109-2628-B-037-005-; 109-2314B-037-066; 109-2314-B-037-072-;

110-2321-B-037-002); from the National Health Research Institutes

(NHRI-EX110-10703PI), all in Taiwan. The authors thank the help from

members of the Taiwan Esophageal Consortium. None of these institutions

played any role in the study design, data collection and analysis,

decision to publish, or preparation of the manuscript.

CR Abnet CC, 2018, GASTROENTEROLOGY, V154, P360, DOI 10.1053/j.gastro.2017.08.023

Alam SR, 2012, BIOCHEM PHARMACOL, V83, P695, DOI 10.1016/j.bcp.2011.11.003

ALKEMADE HAC, 1993, AM J PATHOL, V143, P1679

Bouchard D, 2006, LANCET ONCOL, V7, P167, DOI 10.1016/S1470-2045(06)70579-4

Caruso JA, 2015, ONCOGENE, V34, P3556, DOI 10.1038/onc.2014.284

Caruso JA, 2014, BREAST CANCER RES, V16, DOI 10.1186/s13058-014-0497-4

Chen YK, 2010, ANTICANCER RES, V30, P727

Clauss A, 2010, NEOPLASIA, V12, P161, DOI 10.1593/neo.91542

Feng Yi, 2015, Oncoscience, V2, P793

Hunt KK, 2013, BREAST CANCER RES, V15, DOI 10.1186/bcr3374

KRAMPS JA, 1985, EXP LUNG RES, V9, P151, DOI 10.3109/01902148509061534

Kunzmann AT, 2018, EUR J GASTROEN HEPAT, V30, P263, DOI 10.1097/MEG.0000000000001029

Labidi-Galy SI, 2015, ONCOGENE, V34, P299, DOI 10.1038/onc.2013.562

Majchrzak-Gorecka M, 2016, CYTOKINE GROWTH F R, V28, P79, DOI 10.1016/j.cytogfr.2015.12.001

Mihaila A, 2001, Z NATURFORSCH C, V56, P291

O'Blenes SB, 2000, CIRCULATION, V102, P289

Pennathur A, 2013, LANCET, V381, P400, DOI 10.1016/S0140-6736(12)60643-6

Saidi A, 2008, INT J CANCER, V122, P2187, DOI 10.1002/ijc.23313

SALLENAVE JM, 1993, AM J RESP CELL MOL, V8, P439, DOI 10.1165/ajrcmb/8.4.439

Scott A, 2011, BIOCHEM SOC T, V39, P1437, DOI 10.1042/BST0391437

Shaw L, 2011, BIOCHEM SOC T, V39, P1450, DOI 10.1042/BST0391450

Singhi AD, 2015, HUM PATHOL, V46, P366, DOI 10.1016/j.humpath.2014.11.021

Verrier T, 2012, INT J BIOCHEM CELL B, V44, P1377, DOI 10.1016/j.biocel.2012.05.007

Wang QL, 2018, CLIN EPIDEMIOL, V10, P717, DOI 10.2147/CLEP.S166078

Wei HF, 2012, GYNECOL ONCOL, V125, P727, DOI 10.1016/j.ygyno.2012.03.018

Westin U, 2002, MEDIAT INFLAMM, V11, P7, DOI 10.1080/09629350210304

Yamamoto S, 1997, BRIT J CANCER, V76, P1081, DOI 10.1038/bjc.1997.511

Yazbeck R, 2016, WORLD J GASTROENTERO, V22, P10077, DOI 10.3748/wjg.v22.i46.10077

Yokota T, 2007, CANCER RES, V67, P11272, DOI 10.1158/0008-5472.CAN-07-2322

Yoshida N, 2002, ONCOL REP, V9, P495

Yu KS, 2010, INT J CANCER, V127, P1308, DOI 10.1002/ijc.25125

NR 31

TC 0

Z9 0

U1 2

U2 2

PU MDPI

PI BASEL

PA ST ALBAN-ANLAGE 66, CH-4052 BASEL, SWITZERLAND

EI 2072-6694

J9 CANCERS

JI Cancers

PD JUN

PY 2021

VL 13

IS 12

AR 3082

DI 10.3390/cancers13123082

PG 12

WC Oncology

WE Science Citation Index Expanded (SCI-EXPANDED)

SC Oncology

GA TA6WD

UT WOS:000667387800001

PM 34205756

OA Green Published, gold

DA 2022-05-19

ER

PT J

AU Luo, H

Quan, J

Xiao, H

Luo, J

Zhang, Q

Pi, GC

Ye, YF

He, R

Liu, Y

Su, XN

Zhao, LH

Wang, G

AF Luo, Hong

Quan, Jin

Xiao, He

Luo, Jia

Zhang, Qin

Pi, Guocheng

Ye, Yunfei

He, Rong

Liu, Yun

Su, Xiaona

Zhao, Lianhua

Wang, Ge

TI FGFR inhibitor AZD4547 can enhance sensitivity of esophageal squamous

cell carcinoma cells with epithelial-mesenchymal transition to gefitinib

SO ONCOLOGY REPORTS

LA English

DT Article

DE fibroblast growth factor receptor 1; epidermal growth factor receptor;

epithelial-mesenchymal transition; esophageal squamous cell carcinoma;

gefitinib

ID KINASE; AMPLIFICATION; SURVIVAL; AKT

AB Activation of fibroblast growth factor receptor (FGFR) signaling occurs in various cancers, including esophageal squamous cell carcinoma (ESCC), however, the effect of targeting FGFR in ESCC is not clear. Herein, we examined the phosphorylation level of FGFR1(Y654) (p-FGFR1) in ESCC cell lines and tumor tissues, as well as the cancer cell killing effects of gefitinib and FGFR inhibitor AZD4547 in combination form or alone in ESCC cells. Immunohistochemistry staining was used to detect the expression level of p-FGFR1 in 87 ESCC specimens. The effects of gefitinib and FGFR inhibitor AZD4547 on ESCC cells were analyzed by CCK-8 assay, flow cytometry and western blotting assays. Twenty-six patients diagnosed with esophageal squamous cell carcinoma (ESCC) (29.9%) were observed with a high level of p-FGFR1. The proportion of lesions located in the lower segment of the esophagus was significantly higher in the high p-FGFR1 level group (26.9 vs. 8.2%, P=0.003). The IC50 values of gefitinib alone and in combination with 500 nM AZD4547 were 22.9 +/- 2.1 and 4.13 +/- 0.12 mu M in TE10 cells, and 9.85 +/- 5.5 and 3.21 +/- 0.76 mu M in EC9706 cells, respectively. The combination of AZD4547 and gefitinib induced robust apoptosis and decreased clone formation ability compared to gefitinib monotherapy in the TE10 cells. TE10 cells exhibited a mesenchymal phenotype, with a higher level of p-FGFR1 and p-AKT than that in EC9706 cells. AZD4547 and gefitinib co-treatment resulted in a significant decrease in the level of p-AKT in TE10 cells and a complete inhibition of phosphorylation of ERK1/2 in EC9706 cells. Collectively, AZD4547 can improve sensitivity of ESCC cells to gefitinib.

C1 [Luo, Hong; Xiao, He; Luo, Jia; Zhang, Qin; Pi, Guocheng; Ye, Yunfei; He, Rong; Liu, Yun; Su, Xiaona; Wang, Ge] Third Mil Med Univ, Inst Surg Res, Daping Hosp, Ctr Canc, Chongqing 400042, Peoples R China.

[Zhao, Lianhua] Third Mil Med Univ, Inst Surg Res, Daping Hosp, Dept Pathol, Chongqing 400042, Peoples R China.

[Quan, Jin] Cent Hosp Jiangjin, Dept Oncol, Chongqing 402260, Peoples R China.

RP Wang, G (corresponding author), Third Med Univ, Inst Surg Res, Daping Hosp, Ctr Canc, 10 Changjiang Zhilu, Chongqing 400042, Peoples R China.

EM wangge70@hotmail.com

CR Ando N, 2000, ANN SURG, V232, P225, DOI 10.1097/00000658-200008000-00013

Ashton JC, 2015, CANCER RES, V75, P2400, DOI 10.1158/0008-5472.CAN-14-3763

Bandla S, 2012, ANN THORAC SURG, V93, P1101, DOI 10.1016/j.athoracsur.2012.01.064

Brown WS, 2016, ONCOTARGET, V7, P83424, DOI 10.18632/oncotarget.13117

Brown WS, 2016, MOL CANCER THER, V15, P2096, DOI 10.1158/1535-7163.MCT-16-0136

Costa R, 2016, ONCOTARGET, V7, P55924, DOI 10.18632/oncotarget.10482

de Hoon MJL, 2004, BIOINFORMATICS, V20, P1453, DOI 10.1093/bioinformatics/bth078

Dutton SJ, 2014, LANCET ONCOL, V15, P894, DOI 10.1016/S1470-2045(14)70024-5

Furdui CM, 2006, MOL CELL, V21, P711, DOI 10.1016/j.molcel.2006.01.022

Gavine PR, 2012, CANCER RES, V72, P2045, DOI 10.1158/0008-5472.CAN-11-3034

Gibault L, 2005, BRIT J CANCER, V93, P107, DOI 10.1038/sj.bjc.6602625

Hara F, 2005, CANCER LETT, V226, P37, DOI 10.1016/j.canlet.2004.12.025

He EH, 2015, PLOS ONE, V10, DOI 10.1371/journal.pone.0126149

Jo JC, 2015, MOL CANCER THER, V14, P2613, DOI 10.1158/1535-7163.MCT-14-0780

Kim HS, 2015, ONCOTARGET, V6, P2562, DOI 10.18632/oncotarget.2944

Kitai H, 2016, CANCER DISCOV, V6, P754, DOI 10.1158/2159-8290.CD-15-1377

Koole K, 2016, CLIN CANCER RES, V22, P3884, DOI 10.1158/1078-0432.CCR-15-1874

Nguyen PT, 2013, BRIT J CANCER, V109, P2248, DOI 10.1038/bjc.2013.550

Pande AU, 2007, ONCOLOGY-BASEL, V73, P281, DOI 10.1159/000132393

Petty RD, 2017, J CLIN ONCOL, V35, P2279, DOI 10.1200/JCO.2016.70.3934

Qian X, 2014, ONCOGENE, V33, P3411, DOI 10.1038/onc.2013.310

Saldanha AJ, 2004, BIOINFORMATICS, V20, P3246, DOI 10.1093/bioinformatics/bth349

Siegel RL, 2015, CA-CANCER J CLIN, V65, P5, DOI [10.3322/caac.21254, 10.1136/bmj.g1502, 10.1001/jamaoto.2014.2530, 10.3322/caac.21208]

Terry S, 2015, ONCOTARGET, V6, P11994, DOI 10.18632/oncotarget.2740

Thiery JP, 2009, CELL, V139, P871, DOI 10.1016/j.cell.2009.11.007

Tokunaga R, 2016, ONCOTARGET, V7, P19748, DOI 10.18632/oncotarget.7782

Xiong T, 2017, ONCOTARGET, V8, P49851, DOI 10.18632/oncotarget.17989

Xuan X, 2014, CANCER GENE THER, V21, P238, DOI 10.1038/cgt.2014.23

Zhang Y, 2014, J PHARMACOL SCI, V126, P77, DOI 10.1254/jphs.14150FP

NR 29

TC 10

Z9 11

U1 0

U2 8

PU SPANDIDOS PUBL LTD

PI ATHENS

PA POB 18179, ATHENS, 116 10, GREECE

SN 1021-335X

EI 1791-2431

J9 ONCOL REP

JI Oncol. Rep.

PD MAY

PY 2018

VL 39

IS 5

BP 2270

EP 2278

DI 10.3892/or.2018.6304

PG 9

WC Oncology

WE Science Citation Index Expanded (SCI-EXPANDED)

SC Oncology

GA GB6TG

UT WOS:000429205600024

PM 29517107

OA Bronze

DA 2022-05-19

ER

PT J

AU Xing, S

Zheng, X

Zeng, T

Zeng, MS

Zhong, Q

Cao, YS

Pan, KL

Wei, C

Hou, F

Liu, WL

AF Xing, Shan

Zheng, Xin

Zeng, Tao

Zeng, Mu-Sheng

Zhong, Qian

Cao, Yue-Song

Pan, Kai-Lu

Wei, Chu

Hou, Fan

Liu, Wan-Li

TI Chitinase 3-like 1 secreted by peritumoral macrophages in esophageal

squamous cell carcinoma is a favorable prognostic factor for survival

SO WORLD JOURNAL OF GASTROENTEROLOGY

LA English

DT Article

DE Esophageal squamous cell carcinoma; Prognostic biomarker; Chitinase

3-like 1; Macrophage; Esophageal squamous cell carcinoma

ID SERUM YKL-40; RHEUMATOID-ARTHRITIS; OVARIAN-CANCER; BREAST-CANCER;

PLASMA YKL-40; LUNG-CANCER; EXPRESSION; LEVEL; PROTEINS; DISEASE

AB AIM

To identify whether chitinase 3-like 1 (CHI3L1) serves as a suitable biomarker for the prognosis of esophageal squamous cell carcinoma (ESCC) and to analyze this protein's cellular source.

METHODS

An ELISA was conducted to detect the concentration of CHI3L1 in the serum of 150 ESCC patients diagnosed between January 2001 and February 2005. The prognostic relevance of CHI3L1 was evaluated by a Kaplan-Meier and Cox regression analysis. The immunohistochemistry was reanalyzed, and fluorescent staining was utilized to explore the cellular origins of CHI3L1. We stimulated monocyte-derived macrophages (MDMs) with either IL-6 or the supernatant of the ESCC cell line Eca-109 and later investigated the level of CHI3L1 by qPCR and ELISA.

RESULTS

The level of serum CHI3L1 was higher in older patients (>= 60) than in patients under the age of 60 (P = 0.001). The patients with higher levels of CHI3L1 had a significantly shorter overall survival, whereas the traditional markers, carcinoembryonic antigen and squamous cell carcinoma antigen, were less effective (P > 0.05). A multivariate Cox analysis (P = 0.001) indicated that CHI3L1 was an independent prognostic factor for ESCC patients. Peritumoral macrophages in ESCC exhibited high levels of CHI3L1. Interleukin-6 (IL-6) and the supernatant of Eca-109 containing IL-6 stimulated MDMs to secrete CHI3L1. The serum concentration of CHI3L1 in the ESCC patients showed a weak correlation with the laboratory inflammatory parameters neutrophil (NEU, P = 0.045), neutrophil/lymphocyte rate (NLR, P = 0.016), and C-reactive protein (CRP, P < 0.001).

CONCLUSION

Our study first established a connection between the pretreated CHI3L1 and patients with ESCC, and the serum CHI3L1 was primarily secreted by ESCC-surrounded macrophages.

C1 [Xing, Shan; Zheng, Xin; Zeng, Tao; Zeng, Mu-Sheng; Zhong, Qian; Hou, Fan; Liu, Wan-Li] Collaborat Innovat Ctr Canc Med, State Key Lab Oncol Southern China, Guangzhou 510060, Guangdong, Peoples R China.

[Xing, Shan; Zheng, Xin; Zeng, Tao; Hou, Fan; Liu, Wan-Li] Sun Yat Sen Univ, Dept Clin Lab, Canc Ctr, 651 Dongfeng Rd East, Guangzhou 510060, Guangdong, Peoples R China.

[Cao, Yue-Song] Gannan Med Univ, Dept Biotechnol, Ganzhou 341000, Jiangxi, Peoples R China.

[Pan, Kai-Lu; Wei, Chu] Guangdong Med Univ, Dept Clin Lab, Dongguan 523808, Guangdong, Peoples R China.

RP Liu, WL (corresponding author), Sun Yat Sen Univ, Dept Clin Lab, Canc Ctr, 651 Dongfeng Rd East, Guangzhou 510060, Guangdong, Peoples R China.

EM liuwl@sysucc.org.cn

CR Bleau G, 1999, EXS, V87, P211

Boot RG, 1999, ARTERIOSCL THROM VAS, V19, P687, DOI 10.1161/01.ATV.19.3.687

Cao HH, 2015, ONCOTARGET, V6, P5435, DOI 10.18632/oncotarget.3102

Chen GQ, 2014, MED ONCOL, V31, DOI 10.1007/s12032-014-0376-y

Daigneault M, 2010, PLOS ONE, V5, DOI 10.1371/journal.pone.0008668

Hempen M, 2009, OBES SURG, V19, P1557, DOI 10.1007/s11695-009-9917-4

Hogdall EVS, 2003, ONCOL REP, V10, P1535

Horbinski C, 2010, INT J CLIN EXP PATHO, V3, P226

Jensen BV, 2003, CLIN CANCER RES, V9, P4423

Ji L, 2007, WORLD J GASTROENTERO, V13, P2622, DOI 10.3748/wjg.v13.i18.2622

Johansen JS, 2008, CLIN EXP IMMUNOL, V151, P260, DOI 10.1111/j.1365-2249.2007.03561.x

Johansen JS, 2000, J HEPATOL, V32, P911, DOI 10.1016/S0168-8278(00)80095-1

JOHANSEN JS, 1993, BRIT J RHEUMATOL, V32, P949

Johansen JS, 1999, ARTHRITIS RHEUM-US, V42, P2624, DOI 10.1002/1529-0131(199912)42:12<2624::AID-ANR17>3.0.CO;2-K

JOHANSEN JS, 1992, J BONE MINER RES, V7, P501

Junker N, 2005, LUNG CANCER, V48, P223, DOI 10.1016/j.lungcan.2004.11.011

Kirkpatrick RB, 1997, EXP CELL RES, V237, P46, DOI 10.1006/excr.1997.3764

Kronborg G, 2002, SCAND J INFECT DIS, V34, P323, DOI 10.1080/00365540110080233

Lin YS, 2013, J EPIDEMIOL, V23, P233, DOI 10.2188/jea.JE20120162

Matsumoto T, 2001, CLIN EXP RHEUMATOL, V19, P655

Nielsen AR, 2011, CYTOKINE, V55, P152, DOI 10.1016/j.cyto.2011.03.014

Nojgaard C, 2003, J HEPATOL, V39, P179, DOI 10.1016/S0168-8278(03)00184-3

Nordenbaek C, 1999, J INFECT DIS, V180, P1722, DOI 10.1086/315050

Pennathur A, 2013, LANCET, V381, P400, DOI 10.1016/S0140-6736(12)60643-6

Rehli M, 2003, J BIOL CHEM, V278, P44058, DOI 10.1074/jbc.M306792200

Roslind A, 2008, INT J CANCER, V122, P857, DOI 10.1002/ijc.23152

Shao R, 2011, BRIT J CANCER, V105, P1203, DOI 10.1038/bjc.2011.347

Su H, 2011, CLIN CANCER RES, V17, P2955, DOI 10.1158/1078-0432.CCR-10-2724

Tanwar MK, 2002, CANCER RES, V62, P4364

Thom I, 2010, CANCER-AM CANCER SOC, V116, P4114, DOI 10.1002/cncr.25196

Tong M, 2012, CANCER RES, V72, P6024, DOI 10.1158/0008-5472.CAN-12-1269

Verhoeckx KCM, 2004, PROTEOMICS, V4, P1014, DOI 10.1002/pmic.200300669

Vind I, 2003, SCAND J GASTROENTERO, V38, P599, DOI 10.1080/00365520310000537

Volck B, 2001, OSTEOARTHR CARTILAGE, V9, P203, DOI 10.1053/joca.2000.0377

Yang L, 2005, CANCER EPIDEM BIOMAR, V14, P243

Zhang J, 2015, PLOS ONE, V10, DOI 10.1371/journal.pone.0116951

Zheng X, 2014, BMC CANCER, V14, DOI 10.1186/1471-2407-14-490

Zhu CB, 2012, ANN SURG ONCOL, V19, P817, DOI 10.1245/s10434-011-2026-3

Zou L, 2010, BRAZ J MED BIOL RES, V43, P1232, DOI 10.1590/S0100-879X2010007500133

NR 39

TC 8

Z9 8

U1 1

U2 6

PU BAISHIDENG PUBLISHING GROUP INC

PI PLEASANTON

PA 8226 REGENCY DR, PLEASANTON, CA 94588 USA

SN 1007-9327

EI 2219-2840

J9 WORLD J GASTROENTERO

JI World J. Gastroenterol.

PD NOV 21

PY 2017

VL 23

IS 43

BP 7693

EP 7704

DI 10.3748/wjg.v23.i43.7693

PG 12

WC Gastroenterology & Hepatology

WE Science Citation Index Expanded (SCI-EXPANDED)

SC Gastroenterology & Hepatology

GA FN0XE

UT WOS:000415703800005

PM 29209110

OA Green Submitted, Green Published, hybrid

DA 2022-05-19

ER

PT J

AU Aghcheli, K

Parsian, H

Qujeq, D

Talebi, M

Mosapour, A

Khalilipour, E

Islami, F

Semnani, S

Malekzadeh, R

AF Aghcheli, Karim

Parsian, Hadi

Qujeq, Durdi

Talebi, Mohammad

Mosapour, Abbas

Khalilipour, Elias

Islami, Farhad

Semnani, Shahryar

Malekzadeh, Reza

TI Serum hyaluronic acid and laminin as potential tumor markers for upper

gastrointestinal cancers

SO EUROPEAN JOURNAL OF INTERNAL MEDICINE

LA English

DT Article

DE Cardia cancer; Esophageal cancer; Esophageal squamous cell carcinoma;

Hyaluronic acid; Laminin

ID CARCINOMA; EXPRESSION; COLON; CD44

AB Background: Early diagnosis of patients with upper gastrointestinal cancer is important because many cases are diagnosed in advanced stages and have poor prognosis. Several studies have reported increased serum levels of hyaluronic acid and laminin in various cancers and the correlation of the levels with poor prognosis. However, little data on the use of serum hyaluronic acid and laminin levels for early detection of esophageal and gastric cancers are available.

Methods: We assessed serum hyaluronic acid and laminin levels using enzyme-linked immunosorbent assay in 20 gastric cardia cancer, 23 gastric noncardia cancer and 20 esophageal squamous cell carcinoma incident cases and 25 controls in the Golestan Province, northern Iran, a high risk area for upper gastrointestinal cancers.

Results: Mean serum hyaluronic acid and laminin concentrations in cancer cases were higher than in controls in crude analyses. Significant correlations were observed between hyaluronic acid levels and gastric noncardia cancer (Beta-coefficient=0.390; P=0.01) and esophageal squamous cell carcinoma (Betacoefficient=0.332; P=0.05) and between laminin levels and gastric cardia cancer (Beta-coefficient=0.454; P=0.003) in multivariate models. For esophageal squamous cell carcinoma, gastric cardia cancer, and gastric noncardia cancer, area under ROC curve (AUC) of hyaluronic acid was 0.708, 0.694, and 0.770, and of laminin was 0.706, 0.828, and 0.671.

Conclusions: Our study suggests that hyaluronic acid and laminin may be used to identify potentially high-risk groups of upper gastrointestinal cancers for further diagnostic work-ups, particularly in high incidence areas. Nevertheless, further studies with larger sample size and tumor staging information are warranted to clarify the clinical significance of hyaluronic acid and laminin in those cancers. (C) 2011 European Federation of Internal Medicine. Published by Elsevier B.V. All rights reserved.

C1 [Parsian, Hadi; Qujeq, Durdi; Mosapour, Abbas] Babol Univ Med Sci, Dept Biochem & Biophys, Babol Sar, Iran.

[Aghcheli, Karim; Khalilipour, Elias; Islami, Farhad; Malekzadeh, Reza] Univ Tehran Med Sci, Digest Dis Res Ctr, Tehran, Iran.

[Parsian, Hadi] Babol Univ Med Sci, Cellular & Mol Biol Res Ctr, Babol Sar, Iran.

[Talebi, Mohammad] Mazandaran Univ Med Sci, Dept Lab Sci, Sari, Iran.

[Islami, Farhad] Int Agcy Res Canc, F-69372 Lyon, France.

[Islami, Farhad] Kings Coll London, Thames Canc Registry, London WC2R 2LS, England.

[Semnani, Shahryar] Golestan Univ Med Sci, Golestan Res Ctr Gastroenterol & Hepatol, Gorgan, Iran.

RP Parsian, H (corresponding author), Babol Univ Med Sci, Dept Biochem & Biophys, Ganjafrooz Ave, Babol Sar, Iran.

EM hadiparsian@yahoo.com

RI Pour, Elias Khalili/AAT-5270-2021; Qujeq, Durdi/AAD-2318-2019; Semnani,

Shahryar/N-2270-2016; Mosapour, Abbas/Q-5418-2016; Malekzadeh,

Reza/U-1382-2017; Parsian, Hadi/H-7825-2017

OI Pour, Elias Khalili/0000-0001-8123-4375; Qujeq,

Durdi/0000-0003-1344-9344; Semnani, Shahryar/0000-0002-8768-6142;

Mosapour, Abbas/0000-0003-2599-5449; Malekzadeh,

Reza/0000-0002-9820-6335; Parsian, Hadi/0000-0002-3965-7566; Islami,

Farhad/0000-0002-7357-5994; Malekzadeh, Reza/0000-0003-1043-3814

FU Digestive Disease Research Centers of Tehran University of Medical

Sciences; Golestan University of Medical Sciences; Atrak clinic

FX We sincerely thank the staff of Atrak clinic, including Dr Haji-Amin

Marjani, Safora Kor, Bita Mohammadi, Maral Eskandar Nezhad, Ashur

Yolmeh, and also the Digestive Disease Research Centers of Tehran

University of Medical Sciences and Golestan University of Medical

Sciences for their financial support.

CR Belo JN, 2007, ARTHRIT RHEUM-ARTHR, V57, P13, DOI 10.1002/art.22475

Chu Yongli, 2002, J Huazhong Univ Sci Technolog Med Sci, V22, P58

COOPER EH, 1988, BRIT J CANCER, V58, P668, DOI 10.1038/bjc.1988.283

DELPECH B, 1990, INT J CANCER, V46, P388, DOI 10.1002/ijc.2910460309

Eissa S, 2005, CANCER-AM CANCER SOC, V103, P1356, DOI 10.1002/cncr.20902

Enzinger PC, 2003, NEW ENGL J MED, V349, P2241, DOI 10.1056/NEJMra035010

Franzmann EJ, 2003, INT J CANCER, V106, P438, DOI 10.1002/ijc.11252

FREBOURG T, 1987, CANCER, V59, P2104, DOI 10.1002/1097-0142(19870615)59:12<2104::AID-CNCR2820591224>3.0.CO;2-I

Gao ZL, 2007, HEPATO-GASTROENTEROL, V54, P1591

Genasetti A, 2008, CONNECT TISSUE RES, V49, P120, DOI 10.1080/03008200802148462

Gotte M, 2006, CANCER RES, V66, P10233, DOI 10.1158/0008-5472.CAN-06-1464

Islami F, 2004, BRIT J CANCER, V90, P1402, DOI 10.1038/sj.bjc.6601737

Islami F, 2009, EUR J CANCER, V45, P3156, DOI 10.1016/j.ejca.2009.09.018

Iwahashi M, 1997, J CLIN PATHOL, V50, P77, DOI 10.1136/jcp.50.1.77

Iyer RB, 2003, AM J ROENTGENOL, V181, P785, DOI 10.2214/ajr.181.3.1810785

KNUDSON W, 1984, P NATL ACAD SCI-BIOL, V81, P6767, DOI 10.1073/pnas.81.21.6767

KUMAR S, 1989, INT J CANCER, V44, P445, DOI 10.1002/ijc.2910440311

LAURENT TC, 1992, FASEB J, V6, P2397, DOI 10.1096/fasebj.6.7.1563592

Misra S, 2008, CONNECT TISSUE RES, V49, P219, DOI 10.1080/03008200802143356

Parkin DM, 2005, CA-CANCER J CLIN, V55, P74, DOI 10.3322/canjclin.55.2.74

Parsian H, 2010, ACTA CLIN CROAT, V49, P257

Parsian H, 2010, J GASTROINTEST LIVER, V19, P169

Parsian Hadi, 2009, New Zealand Journal of Medical Laboratory Science, V63, P12

Rosa H, 2008, WORLD J GASTROENTERO, V14, P3628, DOI 10.3748/wjg.14.3628

Ru-Dong X, 2008, CHINESE MED J-PEKING, V121, P327, DOI 10.1097/00029330-200802020-00009

Skubitz APN, 1996, AM J PATHOL, V148, P1445

Steevens J, 2010, EUR J GASTROEN HEPAT, V22, P669, DOI 10.1097/MEG.0b013e32832ca091

Tammi RH, 2008, SEMIN CANCER BIOL, V18, P288, DOI 10.1016/j.semcancer.2008.03.005

Trivers KF, 2008, INT J CANCER, V123, P1422, DOI 10.1002/ijc.23691

Vizoso FJ, 2004, EJSO-EUR J SURG ONC, V30, P318, DOI 10.1016/j.ejso.2003.11.007

Wang C, 1996, AM J PATHOL, V148, P1861

Whiting John, 2006, Gastric Cancer, V9, P74, DOI 10.1007/s10120-006-0360-0

Wu Chew-Wun, 2002, Expert Rev Mol Med, V4, P1, DOI 10.1017/S1462399402004337

ZWEIG MH, 1993, CLIN CHEM, V39, P561

NR 34

TC 16

Z9 17

U1 1

U2 8

PU ELSEVIER

PI AMSTERDAM

PA RADARWEG 29, 1043 NX AMSTERDAM, NETHERLANDS

SN 0953-6205

EI 1879-0828

J9 EUR J INTERN MED

JI Eur. J. Intern. Med.

PD JAN

PY 2012

VL 23

IS 1

BP 58

EP 64

DI 10.1016/j.ejim.2011.07.018

PG 7

WC Medicine, General & Internal

WE Science Citation Index Expanded (SCI-EXPANDED)

SC General & Internal Medicine

GA 863FS

UT WOS:000298148800022

PM 22153533

DA 2022-05-19

ER

PT J

AU Dura, P

Bregitha, CVV

Morsche, RHMT

Roelofs, HMJ

Kristinsson, JO

Wobbes, T

Witeman, BJM

Tan, ACITL

Drenth, JPM

Peters, WHM

AF Dura, Polat

Bregitha, Caro V. V.

Morsche, Rene H. M. Te

Roelofs, Hennie M. J.

Kristinsson, Jon O.

Wobbes, Theo

Witeman, Ben J. M.

Tan, Adriaan C. I. T. L.

Drenth, Joost P. M.

Peters, Wilbert H. M.

TI EPHX1 polymorphisms do not modify esophageal carcinoma susceptibility in

Dutch Caucasians

SO ONCOLOGY REPORTS

LA English

DT Article

DE microsomal epoxide hydrolase; genetic polymorphism; detoxification;

esophageal adenocarcinoma; esophageal squamous cell carcinoma

ID SQUAMOUS-CELL-CARCINOMA; EPOXIDE HYDROLASE GENOTYPES;

GLUTATHIONE-S-TRANSFERASE; GENOME-WIDE ASSOCIATION; GENETIC

POLYMORPHISMS; NORTH CHINA; CANCER; RISK; ADENOCARCINOMA; POPULATION

AB Esophageal cancer (EC) has a globally increasing incidence with poor curative treatment options and survival rates. Crucial risk factors are exposure to toxins or carcinogens. Microsomal epoxide hydrolase (mEH) is a biotransformation enzyme essential for the detoxification of xenobiotics. Polymorphisms in exon 3 and exon 4 of the microsomal epoxide hydrolase gene (EPHX1) modify catalytic activity of this enzyme and subsequently may play a role in EC etiology. This case-control study investigated whether these polymorphisms in the EPHX1 gene influence esophageal cancer susceptibility in a Dutch Caucasian population. A case-control study including 349 Caucasian EC patients and 581 Caucasian healthy controls was conducted and the polymorphisms Tyr113His (exon 3) and His139Arg (exon 4) in the EPHX1 gene were determined, using polymerase chain reaction. The distribution of exon 3 and exon 4 genotypes were compared between cases and controls. Analyses included a stratification according to tumor histology; esophageal adenocarcinoma (EAC) or squamous cell carcinoma (ESCC). Furthermore, on the basis of allelic in vitro enzyme activity assays, exon 3 and 4 genotypes were combined and categorized according to their predicted high, medium or low enzyme activity. Homozygosity and heterozygosity for both exon 3 and 4 polymorphisms were correlated with a decreased esophageal squamous cell carcinoma risk. Heterozygosity and homozygosity for both polymorphisms correlated with an increased and a decreased esophageal adenocarcinoma risk, respectively. Predicted inter-mediate and high activity genotypes were risk and protective factors for esophageal squamous cell carcinoma and esophageal adenocarcinoma, respectively. However, none of these associations were statistically significant. In conclusion, the polymorphisms in exon 3 and exon 4 of the EPHX1 gene do not seem to be modifiers of esophageal squamous cell carcinoma or esophageal adenocarcinoma risk in Dutch Caucasians.

C1 [Dura, Polat; Bregitha, Caro V. V.; Morsche, Rene H. M. Te; Roelofs, Hennie M. J.; Kristinsson, Jon O.; Drenth, Joost P. M.; Peters, Wilbert H. M.] Radboud Univ Nijmegen, Dept Gastroenterol, Med Ctr, NL-6500 HB Nijmegen, Netherlands.

[Wobbes, Theo] Radboud Univ Nijmegen, Dept Surg, Med Ctr, NL-6500 HB Nijmegen, Netherlands.

[Witeman, Ben J. M.] Hosp Gelderse Vallei, Dept Gastroenterol & Hepatol, Ede, Netherlands.

[Tan, Adriaan C. I. T. L.] Canisius Wilhelmina Hosp, Dept Gastroenterol & Hepatol, Nijmegen, Netherlands.

RP Dura, P (corresponding author), Radboud Univ Nijmegen, Dept Gastroenterol, Med Ctr, POB 9101, NL-6500 HB Nijmegen, Netherlands.

EM p.dura@mdl.umcn.nl

RI Drenth, J.P.H./H-8025-2014; te Morsche, R/P-9143-2015; Drenth,

Joost/V-7436-2019; Peters, W.H.M./L-4572-2015

CR Baxter SW, 2002, CANCER LETT, V177, P75, DOI 10.1016/S0304-3835(01)00782-0

Benhamou S, 1998, CANCER RES, V58, P5291

Casson AG, 2006, CANCER DETECT PREV, V30, P423, DOI 10.1016/j.cdp.2006.09.005

Chauhan PS, 2011, DNA CELL BIOL, V30, P39, DOI 10.1089/dna.2010.1092

Harrison DJ, 1999, BRIT J CANCER, V79, P168, DOI 10.1038/sj.bjc.6690028

Hiyama T, 2007, INT J CANCER, V121, P1643, DOI 10.1002/ijc.23044

Hongo M, 2009, J GASTROEN HEPATOL, V24, P729, DOI 10.1111/j.1440-1746.2009.05824.x

Huang WY, 2005, CANCER EPIDEM BIOMAR, V14, P152

Ihsan R, 2010, J GASTROEN HEPATOL, V25, P1456, DOI 10.1111/j.1440-1746.2010.06354.x

Jain M, 2008, TOXICOL APPL PHARM, V230, P247, DOI 10.1016/j.taap.2008.02.023

Kiran M, 2008, DNA CELL BIOL, V27, P687, DOI 10.1089/dna.2008.0805

Kristinsson JO, 2009, WORLD J GASTROENTERO, V15, P3493, DOI 10.3748/wjg.15.3493

Lacko M, 2008, HEAD NECK-J SCI SPEC, V30, P836, DOI 10.1002/hed.20781

Li ZQ, 2009, CELL RES, V19, P519, DOI 10.1038/cr.2009.33

Lin YC, 2006, CANCER LETT, V237, P281, DOI 10.1016/j.canlet.2005.06.010

Morisseau C, 2005, ANNU REV PHARMACOL, V45, P311, DOI 10.1146/annurev.pharmtox.45.120403.095920

Morisseau C, 2008, PEST MANAG SCI, V64, P594, DOI 10.1002/ps.1583

Newman JW, 2005, PROG LIPID RES, V44, P1, DOI 10.1016/j.plipres.2004.10.001

Peters WHM, 2005, GUT, V54, P1659, DOI 10.1136/gut.2005.076422

Peterson S, 2006, FOOD CHEM TOXICOL, V44, P1474, DOI 10.1016/j.fct.2006.04.010

Sant M, 2003, ANN ONCOL, V14, pV61, DOI 10.1093/annonc/mdg754

Smith TJ, 1998, CARCINOGENESIS, V19, P667, DOI 10.1093/carcin/19.4.667

Wang LD, 2003, WORLD J GASTROENTERO, V9, P1394, DOI 10.3748/wjg.v9.i7.1394

Wang LD, 2010, NAT GENET, V42, P759, DOI 10.1038/ng.648

Wang ZM, 2006, BMC CANCER, V6, DOI 10.1186/1471-2407-6-287

Ward MH, 1997, INT J CANCER, V71, P14, DOI 10.1002/(SICI)1097-0215(19970328)71:1&lt;14::AID-IJC4&gt;3.0.CO;2-6

Wiech T, 2009, LAB INVEST, V89, P385, DOI 10.1038/labinvest.2008.67

Wijnhoven BPL, 2002, EUR J GASTROEN HEPAT, V14, P115, DOI 10.1097/00042737-200202000-00004

Wu C, 2011, NAT GENET, V43, P679, DOI 10.1038/ng.849

Zhang JH, 2003, WORLD J GASTROENTERO, V9, P2654

NR 30

TC 14

Z9 14

U1 0

U2 0

PU SPANDIDOS PUBL LTD

PI ATHENS

PA POB 18179, ATHENS, 116 10, GREECE

SN 1021-335X

J9 ONCOL REP

JI Oncol. Rep.

PD JUN

PY 2012

VL 27

IS 6

BP 1710

EP 1716

DI 10.3892/or.2012.1734

PG 7

WC Oncology

WE Science Citation Index Expanded (SCI-EXPANDED)

SC Oncology

GA 939BY

UT WOS:000303783100002

PM 22447130

DA 2022-05-19

ER

PT J

AU Liu, JY

Li, F

Wang, LP

Chen, XF

Wang, D

Cao, L

Ping, Y

Zhao, S

Li, B

Thorne, SH

Zhang, B

Kalinski, P

Zhang, Y

AF Liu, J. Y.

Li, F.

Wang, L. P.

Chen, X. F.

Wang, D.

Cao, L.

Ping, Y.

Zhao, S.

Li, B.

Thorne, S. H.

Zhang, B.

Kalinski, P.

Zhang, Y.

TI CTL- vs T-reg lymphocyte-attracting chemokines, CCL4 and CCL20, are

strong reciprocal predictive markers for survival of patients with

oesophageal squamous cell carcinoma

SO BRITISH JOURNAL OF CANCER

LA English

DT Article

DE CCL4; CCL20; oesophageal squamous cell carcinoma; T lymphocyte;

chemotaxis; prognosis

ID TUMOR-INFILTRATING LYMPHOCYTES; COLORECTAL-CARCINOMA; OVARIAN-CANCER;

BREAST-CANCER; IMMUNE CELLS; SALVAGE STRATEGIES; RECRUITMENT;

EXPRESSION; MICROENVIRONMENT; METASTASES

AB Background: Tumoural infiltration of T lymphocytes is determined by local patterns of specific chemokine expression. In this report, we examined the roles of CCL4 and CCL20 in the accumulation of CD8(+) cytotoxic T lymphocytes (CTLs) and regulatory T (T-reg) lymphocytes in oesophageal squamous cell carcinoma (ESCC), and determined the correlations between chemokine expressions and ESCC patients' survival.

Methods: Reverse transcriptase-PCR and immunohistochemistry (IHC) staining were performed to examine the expressions of interested genes. Flow cytometry was adopted to check the expressions of CCL4- and CCL6-specific receptors, CCR5 and CCR6, on CTLs and T-reg cells. In addition, transwell assay was carried on.

Results: The CCL4 expression was significantly correlated with the expression of CTL markers (CD8 and Granzyme B), whereas CCL20 was positively correlated with T-reg markers (FoxP3 and IL-10). Consistently, CCR5 was found to be mainly expressed on CD8(+) T lymphocytes, while CCR6 showed prevalence on T-reg lymphocytes and the frequencies of CCR5(+)CD8(+) CTLs and CCR6(+) Treg cells were higher in TIL compared with PBMC. Respectively, CCL4 and CCL20 recruited CD8(+) and regulatory T cells in vitro. Importantly, high levels of CCL4 in the lesions of ESCC patients predicted prolonged survival. Furthermore, CCL4(high)/CCL20(low) group demonstrated better overall survival, whereas CCL4(low)/CCL20(low) and CCL4(low)/CCL20(high) groups showed the worst overall survival.

Conclusions: Our data showed that CCL4 and CCL20 recruit functionally different T lymphocyte subsets into oesophageal carcinoma, indicating CCL4 and CCL20 are potential predictors of ESCC patients' survival.

C1 [Liu, J. Y.; Li, F.; Chen, X. F.; Wang, D.; Cao, L.; Ping, Y.; Zhang, Y.] Zhengzhou Univ, Affiliated Hosp 1, Biotherapy Ctr, Zhengzhou 450052, Henan, Peoples R China.

[Liu, J. Y.; Ping, Y.; Zhang, Y.] Zhengzhou Univ, Sch Life Sci, Zhengzhou 450001, Henan, Peoples R China.

[Wang, L. P.; Chen, X. F.; Wang, D.; Cao, L.; Zhang, Y.] Zhengzhou Univ, Affiliated Hosp 1, Dept Oncol, Zhengzhou 450052, Henan, Peoples R China.

[Zhao, S.; Li, B.] Zhengzhou Univ, Affiliated Hosp 1, Dept Thorac Surg, Zhengzhou 450052, Henan, Peoples R China.

[Thorne, S. H.; Kalinski, P.] Univ Pittsburgh, Inst Canc, Dept Surg, Pittsburgh, PA 15232 USA.

[Zhang, B.] Northwestern Univ, Feinberg Sch Med, Div Hematol Oncol, Robert H Lurie Comprehens Canc Ctr,Dept Med, Chicago, IL 60611 USA.

[Zhang, Y.] Engn Key Lab Cell Therapy Henan Prov, Zhengzhou 450052, Henan, Peoples R China.

RP Zhang, Y (corresponding author), Zhengzhou Univ, Affiliated Hosp 1, Biotherapy Ctr, Zhengzhou 450052, Henan, Peoples R China.

EM yizhang@zzu.edu.cn

OI Zhang, Yi/0000-0001-9861-4681

FU China-US (NFSC-NIH) Program for Biomedical Collaborative Research

[812111102, P01CA132714-04S1]; National Natural Science Foundation of

China [81171986, 81271815]; NATIONAL CANCER INSTITUTE [P01CA132714]

Funding Source: NIH RePORTER

FX This study was supported by grants from the China-US (NFSC-NIH) Program

for Biomedical Collaborative Research (Grant Nos. 812111102 and

P01CA132714-04S1) and the National Natural Science Foundation of China

(Grant Nos. 81171986 and 81271815).

CR Bindea G, 2013, IMMUNITY, V39, P782, DOI 10.1016/j.immuni.2013.10.003

Chen DY, 2012, CELL IMMUNOL, V272, P166, DOI 10.1016/j.cellimm.2011.10.015

Chen KJ, 2011, PLOS ONE, V6, DOI 10.1371/journal.pone.0024671

Chew V, 2012, GUT, V61, P427, DOI 10.1136/gutjnl-2011-300509

Cho JW, 2014, CLIN ENDOSC, V47, P523, DOI 10.5946/ce.2014.47.6.523

Cook KW, 2014, GUT, V63, P1550, DOI 10.1136/gutjnl-2013-306253

Correale P, 2012, CLIN CANCER RES, V18, P850, DOI 10.1158/1078-0432.CCR-10-3186

Curiel TJ, 2004, NAT MED, V10, P942, DOI 10.1038/nm1093

Franciszkiewicz K, 2012, CANCER RES, V72, P6325, DOI 10.1158/0008-5472.CAN-12-2027

Gajewski TF, 2013, NAT IMMUNOL, V14, P1014, DOI 10.1038/ni.2703

Galon J, 2006, SCIENCE, V313, P1960, DOI 10.1126/science.1129139

Gu-Trantien C, 2013, J CLIN INVEST, V123, P2873, DOI 10.1172/JCI67428

Hamano R, 2011, CLIN CANCER RES, V17, P3029, DOI 10.1158/1078-0432.CCR-10-2532

Hannelien V, 2012, BBA-REV CANCER, V1825, P117, DOI 10.1016/j.bbcan.2011.10.008

Harlin H, 2009, CANCER RES, V69, P3077, DOI 10.1158/0008-5472.CAN-08-2281

Hermans C, 2014, PLOS ONE, V9, DOI 10.1371/journal.pone.0111757

Izzo Julie G, 2007, Gastrointest Cancer Res, V1, pS3

Junttila MR, 2013, NATURE, V501, P346, DOI 10.1038/nature12626

Katz SC, 2013, ANN SURG ONCOL, V20, P946, DOI 10.1245/s10434-012-2668-9

Liu JL, 2011, PLOS ONE, V6, DOI 10.1371/journal.pone.0019495

Mariette C, 2007, LANCET ONCOL, V8, P545, DOI 10.1016/S1470-2045(07)70172-9

Mariette C, 2014, J CLIN ONCOL, V32, P2416, DOI 10.1200/JCO.2013.53.6532

Martin-Orozco N, 2009, IMMUNITY, V31, P787, DOI 10.1016/j.immuni.2009.09.014

Mulligan AM, 2013, CLIN CANCER RES, V19, P336, DOI 10.1158/1078-0432.CCR-11-3314

Musha H, 2005, INT J CANCER, V116, P949, DOI 10.1002/ijc.21135

Oldham KA, 2012, EUR UROL, V61, P385, DOI 10.1016/j.eururo.2011.10.035

Oo YH, 2012, J HEPATOL, V57, P1044, DOI 10.1016/j.jhep.2012.07.008

Pages F, 2005, NEW ENGL J MED, V353, P2654, DOI 10.1056/NEJMoa051424

Parsonage G, 2012, AM J PATHOL, V180, P1215, DOI 10.1016/j.ajpath.2011.11.032

Popple A, 2012, BRIT J CANCER, V106, P1306, DOI 10.1038/bjc.2012.49

Roussos ET, 2011, NAT REV CANCER, V11, P573, DOI 10.1038/nrc3078

Rustgi AK, 2014, NEW ENGL J MED, V371, P2499, DOI 10.1056/NEJMra1314530

Salerno EP, 2014, INT J CANCER, V134, P563, DOI 10.1002/ijc.28391

Sato E, 2005, P NATL ACAD SCI USA, V102, P18538, DOI 10.1073/pnas.0509182102

Schlecker E, 2012, J IMMUNOL, V189, P5602, DOI 10.4049/jimmunol.1201018

Sehitogullari A, 2011, ASIAN PAC J CANCER P, V12, P599

Seo AN, 2013, BRIT J CANCER, V109, P2705, DOI 10.1038/bjc.2013.634

Sherwood AM, 2013, CANCER IMMUNOL IMMUN, V62, P1453, DOI 10.1007/s00262-013-1446-2

Sudo K, 2014, J CLIN ONCOL, V32, P3400, DOI 10.1200/JCO.2014.56.7156

Sudo K, 2013, J CLIN ONCOL, V31, P4306, DOI 10.1200/JCO.2013.51.7250

Sugiyama D, 2013, P NATL ACAD SCI USA, V110, P17945, DOI 10.1073/pnas.1316796110

Suzuki H, 2013, CLIN IMMUNOL, V146, P26, DOI 10.1016/j.clim.2012.10.007

Tachezy M, 2013, J TRANSL MED, V11, DOI 10.1186/1479-5876-11-238

Wehler TC, 2011, ANTICANCER RES, V31, P2797

Winkler AE, 2011, CANCER RES, V71, P5707, DOI 10.1158/0008-5472.CAN-11-0907

Wu C, 2014, NAT GENET, V46, P1001, DOI 10.1038/ng.3064

Yamazaki T, 2008, J IMMUNOL, V181, P8391, DOI 10.4049/jimmunol.181.12.8391

Yue DL, 2014, EXP CELL RES, V322, P149, DOI 10.1016/j.yexcr.2013.11.009

Zeng J, 2013, J TRANSL MED, V11, DOI 10.1186/1479-5876-11-6

Zhang L, 2003, NEW ENGL J MED, V348, P203, DOI 10.1056/NEJMoa020177

Zhang TQ, 2006, CANCER BIOL THER, V5, P1304, DOI 10.4161/cbt.5.10.3153

Zhao JJ, 2014, HEPATOLOGY, V59, P1331, DOI 10.1002/hep.26916

NR 52

TC 65

Z9 67

U1 1

U2 13

PU NATURE PUBLISHING GROUP

PI LONDON

PA MACMILLAN BUILDING, 4 CRINAN ST, LONDON N1 9XW, ENGLAND

SN 0007-0920

EI 1532-1827

J9 BRIT J CANCER

JI Br. J. Cancer

PD SEP 1

PY 2015

VL 113

IS 5

BP 747

EP 755

DI 10.1038/bjc.2015.290

PG 9

WC Oncology

WE Science Citation Index Expanded (SCI-EXPANDED)

SC Oncology

GA CQ6PU

UT WOS:000360727200008

PM 26284335

OA hybrid, Green Published

DA 2022-05-19

ER

PT J

AU Jo, YY

Yu, JS

Song, KJ

Jang, JY

Yoo, YJ

Kim, SB

Park, SR

Kim, YH

Kim, HR

Kim, JH

AF Jo, Yoon Young

Yu, Jesang

Song, Kye Jin

Jang, Jeong Yun

Yoo, Ye Jin

Kim, Sung-Bae

Park, Sook Ryun

Kim, Yong-Hee

Kim, Hyeong Ryul

Kim, Jong Hoon

TI Definitive chemoradiotherapy versus esophagectomy in patients with

clinical T1bN0M0 esophageal squamous cell carcinoma: A retrospective

study

SO RADIOTHERAPY AND ONCOLOGY

LA English

DT Article

DE Definitive chemoradiotherapy (DCRT); Radical esophagectomy; Esophageal

squamous cell carcinoma (ESCC); Comorbidity

ID CANCER; SURGERY; CHEMORADIATION; CHEMOTHERAPY; RADIOTHERAPY

AB Background and purpose: This study aimed to determine the equivalence between definitive chemoradiotherapy (DCRT) and radical esophagectomy in clinical T1bN0M0 esophageal squamous cell carcinoma (ESCC). Materials and methods: Among 282 patients with cT1bN0M0 ESCC, 238 underwent radical esophagectomy and 44 underwent DCRT. Both treatments were retrospectively compared overall survival (OS), progression-free survival (PFS), and complications. Results: The DCRT group exhibited poorer patient characteristics than the surgery group, especially with mean age (73 vs. 63 years), Eastern Cooperative Oncology Group (ECOG) status, and Charlson Comorbidity Index (p < 0.001, each). The median follow-up duration was 49.5 (range, 0.4-97.0) and 45.5 months (range, 5.0-112.0) in the surgery and DCRT groups, respectively. In the DCRT group, clinical complete response was achieved in 43 patients (97.7%) at 1 month after treatment. The 5-year OS rates were 75.8% and 68.8% (p = 0.135) and the 5-year PFS were 63.8% and 57.8% (p = 0.637) for the surgery and DCRT groups, respectively. Local recurrence rates were identical between the two groups (11.4% and 11.4%), but the distant metastasis rate was lower in the DCRT group (n = 1, 2.27% vs. n = 29, 12.15%). Grade 3-4 hematologic toxicities were observed in 11 patients (25%) of the DCRT group, and 56 patients (23.5%) in the surgery group showed grade 3-5 surgical complications, including mortality (n = 5). Conclusion: Based on the non-inferior survival rates, recurrence patterns, and complication rates without critical surgical mortality, DCRT was comparable to esophagectomy for cT1bN0 esophageal squamous cell carcinoma. (c) 2021 Published by Elsevier B.V. Radiotherapy and Oncology 162 (2021) 112-118

C1 [Jo, Yoon Young; Yu, Jesang; Song, Kye Jin; Jang, Jeong Yun; Yoo, Ye Jin; Kim, Jong Hoon] Univ Ulsan, Asan Med Ctr, Dept Radiat Oncol, Coll Med, 88 Olymp Ro 43 Gil, Seoul 05505, South Korea.

[Kim, Sung-Bae; Park, Sook Ryun] Univ Ulsan, Asan Med Ctr, Dept Med Oncol, Coll Med, Seoul, South Korea.

[Kim, Yong-Hee; Kim, Hyeong Ryul] Univ Ulsan, Asan Med Ctr, Dept Thorac & Cardiovasc Surg, Coll Med, Seoul, South Korea.

RP Kim, JH (corresponding author), Univ Ulsan, Asan Med Ctr, Dept Radiat Oncol, Coll Med, 88 Olymp Ro 43 Gil, Seoul 05505, South Korea.

EM jhkim2@amc.seoul.kr

CR AKIYAMA H, 1994, ANN SURG, V220, P364, DOI 10.1097/00000658-199409000-00012

Ariga H, 2009, INT J RADIAT ONCOL, V75, P348, DOI 10.1016/j.ijrobp.2009.02.086

Bedenne L, 2007, J CLIN ONCOL, V25, P1160, DOI 10.1200/JCO.2005.04.7118

Birkmeyer JD, 2002, NEW ENGL J MED, V346, P1128, DOI 10.1056/NEJMsa012337

Brierley J., 2017, TNM CLASSIFICATION M, V8

Chang AC, 2008, ANN THORAC SURG, V85, P424, DOI 10.1016/j.athoracsur.2007.10.007

Chen DW, 2019, JAMA ONCOL, V5, P1597, DOI 10.1001/jamaoncol.2019.2809

Chen HM, 2018, MED SCI MONITOR, V24, P4183, DOI 10.12659/MSM.910326

Chen WQ, 2016, CA-CANCER J CLIN, V66, P115, DOI 10.3322/caac.21338

Deng YX, 2017, ONCOTARGET, V8, P79662, DOI 10.18632/oncotarget.19030

Enzinger PC, 2003, NEW ENGL J MED, V349, P2241, DOI 10.1056/NEJMra035010

Ferlay J, 2019, INT J CANCER, V144, P1941, DOI 10.1002/ijc.31937

He LR, 2014, J THORAC ONCOL, V9, P1398, DOI 10.1097/JTO.0000000000000267

HERSKOVIC A, 1992, NEW ENGL J MED, V326, P1593, DOI 10.1056/NEJM199206113262403

Hironaka S, 2003, INT J RADIAT ONCOL, V57, P425, DOI 10.1016/S0360-3016(03)00585-6

Ishihara R, 2008, CANCER-AM CANCER SOC, V112, P2166, DOI 10.1002/cncr.23418

Ishikura S, 2003, J CLIN ONCOL, V21, P2697, DOI 10.1200/JCO.2003.03.055

Koide Y, 2017, JPN J CLIN ONCOL, V47, P393, DOI 10.1093/jjco/hyx021

Moro-Sibilot D, 2005, EUR RESPIR J, V26, P480, DOI 10.1183/09031936.05.00146004

Motoori M, 2012, ANN SURG ONCOL, V19, P2135, DOI 10.1245/s10434-012-2231-8

Murakami Y, 2015, INT J CLIN ONCOL, V20, P897, DOI 10.1007/s10147-015-0819-2

Ohtsu A, 1999, J CLIN ONCOL, V17, P2915, DOI 10.1200/JCO.1999.17.9.2915

Stahl M, 2005, J CLIN ONCOL, V23, P2310, DOI 10.1200/JCO.2005.00.034

Tachimori Y, 2019, ESOPHAGUS-TOKYO, V16, P221, DOI 10.1007/s10388-019-00674-z

Tachimori Y, 2016, ESOPHAGUS-TOKYO, V13, P110, DOI 10.1007/s10388-016-0531-y

Yamamoto S, 2011, AM J GASTROENTEROL, V106, P1048, DOI 10.1038/ajg.2011.42

Yamashita H, 2009, J SURG ONCOL, V100, P435, DOI 10.1002/jso.21361

Yoo C, 2012, ANN THORAC SURG, V94, P1862, DOI 10.1016/j.athoracsur.2012.07.042

NR 28

TC 0

Z9 0

U1 0

U2 0

PU ELSEVIER IRELAND LTD

PI CLARE

PA ELSEVIER HOUSE, BROOKVALE PLAZA, EAST PARK SHANNON, CO, CLARE, 00000,

IRELAND

SN 0167-8140

EI 1879-0887

J9 RADIOTHER ONCOL

JI Radiother. Oncol.

PD SEP

PY 2021

VL 162

BP 112

EP 118

DI 10.1016/j.radonc.2021.07.006

EA JUL 2021

PG 7

WC Oncology; Radiology, Nuclear Medicine & Medical Imaging

WE Science Citation Index Expanded (SCI-EXPANDED)

SC Oncology; Radiology, Nuclear Medicine & Medical Imaging

GA WC5ZQ

UT WOS:000704336600015

PM 34265356

DA 2022-05-19

ER

PT J

AU Zheng, JY

Xu, CS

Chu, DK

Zhang, XY

Li, JP

Ji, G

Hong, L

Feng, QX

Li, XH

Wu, GS

Du, JJ

Zhao, QC

AF Zheng Jianyong

Xu Chunsheng

Chu Dake

Zhang Xiaoying

Li Jipeng

Ji Gang

Hong Liu

Feng Quanxin

Li Xiaohua

Wu Guosheng

Du Jianjun

Zhao Qingchuan

TI Human leukocyte antigen G is associated with esophageal squamous cell

carcinoma progression and poor prognosis

SO IMMUNOLOGY LETTERS

LA English

DT Article

DE Esophageal squamous cell carcinoma; HLA-G; IL-10, Clinicopathological

parameters; Prognosis

ID HLA-G EXPRESSION; MONOCLONAL-ANTIBODIES; MOLECULES; CANCER; DIAGNOSIS

AB Human leukocyte antigen G (HLA-G) is a non-classical HLA class I molecule thought to play a key role in maternal-fetal tolerance and cancer immune evasion. This study aimed to investigate the HLA-G expression in lesion sections and plasma sHLA-G levels of primary esophageal squamous cell carcinoma (ESCC) patients and its clinical significance in diagnosis and prognosis of ESCC. 60 ESCC patients and 28 healthy controls were recruited, and the positive expression of HLA-G in ESCC lesions and adjacent normal tissues were 70% (42/60) and 8.6% (5/60) (P < 0.05), respectively, while no expression was found in normal controls. HLA-G1 and HLA-G5 were determined to be dominating isoforms measured by RT-PCR. There was a significant difference in plasma sHLA-G levels between patients with ESCC (15.04 U/ml, range 4.33-250.00 U/ml) and healthy controls (6.81 U/ml, range 0-29.27 U/ml) (P < 0.01). The plasma IL-10 level was higher in ESCC patients than the controls (23.86 pg/ml vs. 12.81 pg/ml, P < 0.01). HLA-G expression in lesion tissues was correlated with cancer cell differentiation (P = 0.033), lymph node metastasis (P = 0.035) of ESCC. However, no obvious correlations were demonstrated between the plasma sHLA-G levels and the clinicopathological parameters. There was a significant correlation between sHLA-G and IL-10 expression (r = 0.353, P = 0.006) in patients with Esophageal squamous cell carcinoma. HLA-G positive expression showed poorer prognosis of ESCC. HLA-G positive expression might serve as a potential marker in the diagnosis or prediction of ESCC. (C) 2014 Elsevier B.V. All rights reserved.

C1 [Zheng Jianyong; Xu Chunsheng; Chu Dake; Zhang Xiaoying; Li Jipeng; Ji Gang; Hong Liu; Feng Quanxin; Li Xiaohua; Wu Guosheng; Du Jianjun; Zhao Qingchuan] Fourth Mil Med Univ, Xijing Hosp Digest Dis, Dept Digest Surg, State Key Lab Canc Biol, Xian 710032, Shaanxi Provinc, Peoples R China.

RP Zhao, QC (corresponding author), Fourth Mil Med Univ, Xijing Hosp Digest Dis, Dept Digest Surg, State Key Lab Canc Biol, Changle Western Rd 17, Xian 710032, Shaanxi Provinc, Peoples R China.

EM zhaoqchuan@163.com

RI JI, Gang/AAG-9877-2020

OI JI, Gang/0000-0002-2415-6275

FU Natural Science Foundation of China [30771839]

FX The current research was supported by grant from Natural Science

Foundation of China (No. 30771839).

CR Amiot L, 2011, CELL MOL LIFE SCI, V68, P417, DOI 10.1007/s00018-010-0583-4

Cai EH, 2012, ASIAN PAC J CANCER P, V13, P1563, DOI 10.7314/APJCP.2012.13.4.1563

Carosella ED, 2003, ADV IMMUNOL, V81, P199, DOI 10.1016/S0065-2776(03)81006-4

Carosella ED, 2011, IMMUNOL LETT, V138, P22, DOI 10.1016/j.imlet.2011.02.011

Chen HX, 2010, HUM IMMUNOL, V71, P892, DOI 10.1016/j.humimm.2010.06.009

Ellyard JI, 2007, TISSUE ANTIGENS, V70, P1, DOI 10.1111/j.1399-0039.2007.00869.x

Fons P, 2006, BLOOD, V108, P2608, DOI 10.1182/blood-2005-12-019919

Gao GF, 2000, J BIOL CHEM, V275, P15232, DOI 10.1074/jbc.275.20.15232

GERAGHTY DE, 1987, P NATL ACAD SCI USA, V84, P9145, DOI 10.1073/pnas.84.24.9145

Gonzalez A, 2012, CRIT REV CL LAB SCI, V49, P63, DOI 10.3109/10408363.2012.677947

Ibrahim EC, 2001, CANCER RES, V61, P6838

Kashyap MK, 2009, CANCER BIOL THER, V8, P36, DOI 10.4161/cbt.8.1.7090

Khong HT, 2002, NAT IMMUNOL, V3, P999, DOI 10.1038/ni1102-999

Kurman RJ, 2006, DTIC DOCUMENT

Lin AF, 2011, INT J CANCER, V129, P1382, DOI 10.1002/ijc.25807

Lopez-Botet M, 1999, SEMIN CANCER BIOL, V9, P19, DOI 10.1006/scbi.1998.0107

Menier C, 2003, HUM IMMUNOL, V64, P315, DOI 10.1016/S0198-8859(02)00821-2

Moreau P, 1999, INT IMMUNOL, V11, P803, DOI 10.1093/intimm/11.5.803

Pistoia V, 2007, SEMIN CANCER BIOL, V17, P469, DOI 10.1016/j.semcancer.2007.07.004

Rizzo R, 2009, AM J REPROD IMMUNOL, V61, P368, DOI 10.1111/j.1600-0897.2009.00703.x

Rouas-Freiss N, 2005, CANCER RES, V65, P10139, DOI 10.1158/0008-5472.CAN-05-0097

Singer G, 2003, CLIN CANCER RES, V9, P4460

Song JH, 2012, GASTROENTEROLOGY, V143, P35, DOI 10.1053/j.gastro.2012.05.003

Yahyapour Y, 2012, ASIAN PAC J CANCER P, V13, P1261, DOI 10.7314/APJCP.2012.13.4.1261

Yan Wei-Hua, 2011, Endocrine Metabolic & Immune Disorders-Drug Targets, V11, P76

Yan WH, 2005, CELL RES, V15, P176, DOI 10.1038/sj.cr.7290283

Ye SR, 2007, MODERN PATHOL, V20, P375, DOI 10.1038/modpathol.3800751

Zhao L, 2012, TISSUE ANTIGENS, V80, P231, DOI 10.1111/j.1399-0039.2012.01922.x

Zidi I, 2011, TUMOR BIOL, V32, P1071, DOI 10.1007/s13277-011-0213-2

NR 29

TC 29

Z9 30

U1 1

U2 4

PU ELSEVIER SCIENCE BV

PI AMSTERDAM

PA PO BOX 211, 1000 AE AMSTERDAM, NETHERLANDS

SN 0165-2478

EI 1879-0542

J9 IMMUNOL LETT

JI Immunol. Lett.

PD SEP

PY 2014

VL 161

IS 1

BP 13

EP 19

DI 10.1016/j.imlet.2014.04.007

PG 7

WC Immunology

WE Science Citation Index Expanded (SCI-EXPANDED)

SC Immunology

GA AN6HU

UT WOS:000340696400003

PM 24768599

DA 2022-05-19

ER

PT J

AU Xu, XF

Mao, BN

Wu, LD

Liu, L

Rui, JP

Chen, GC

AF Xu, Xinfang

Mao, Boneng

Wu, Lingdong

Liu, Lei

Rui, Juping

Chen, Guochang

TI A118G Polymorphism in mu-Opioid Receptor Gene and Interactions with

Smoking and Drinking on Risk of Oesophageal Squamous Cell Carcinoma

SO JOURNAL OF CLINICAL LABORATORY ANALYSIS

LA English

DT Article

DE A118G; alcohol consumption; interaction; oesophageal squamous cell

carcinoma; polymorphism; smoking

ID CANCER; POPULATION; PREVALENCE; SMOKERS; SURGERY; HEALTH; US

AB Aim: To investigate the single nucleotide polymorphism (SNP) of A118G and its interaction with smoking and drinking on oesophageal squamous cell carcinoma (ESCC) risk. Methods: A total of 960 subjects (545 males and 415 females) with a mean age of 58.1 13.4 years were selected, including 490 ESCC patients and 470 normal control subjects. A logistic regression model was used to examine the association between A118G and ESCC and its interaction with A118G and current smoking and drinking. The odds ratio (OR) and 95% confident interval (95%CI) were calculated. Results: The frequency for the A allele of A118G was significantly higher in ESCC cases, OR (95%CI) = 1.22 (1.08-1.59). Logistic regression analysis showed a significant association between the A allele in A118G and increased ESCC risk. The ESCC risk was significantly higher in carriers of the A allele of the A118G polymorphism than those with GG (AG + AA vs. GG, adjusted OR (95%CI) = 1.20 (1.05-1.53)). We found that current smokers with AG or AA of the A118G genotype have the highest ESCC risk compared with never smokers with a GG genotype; the OR (95%CI) was 2.57 (1.66-3.33). Current drinkers with AG or AA of the A118G genotype have the highest ESCC risk compared with not currently drinking subjects with the GG genotype, OR (95%CI) = 2.36 (1.47-3.25), after adjusting for covariates. Conclusion: The A allele of A118G and ESCC and additional interaction between the A allele of A118G and smoking or drinking were associated with increased ESCC risk. (C) 2016 Wiley Periodicals, Inc.

C1 [Xu, Xinfang; Mao, Boneng; Wu, Lingdong; Liu, Lei; Rui, Juping; Chen, Guochang] Yixing Peoples Hosp, Dept Gastroenterol, Tong Zhen Guan Rd 75, Yixing 214200, Jiangsu, Peoples R China.

RP Xu, XF (corresponding author), Yixing Peoples Hosp, Dept Gastroenterol, Tong Zhen Guan Rd 75, Yixing 214200, Jiangsu, Peoples R China.

EM xuxinfang33@163.com

FU Department of Gastroenterology, Yixing People's Hospital

FX The writing of this study was supported by the Department of

Gastroenterology, Yixing People's Hospital. We thank all the partners

and staffs who helped us in the process of this study.

CR Afsharimani B, 2011, CANCER METAST REV, V30, P225, DOI 10.1007/s10555-011-9285-0

Bortsov AV, 2012, ANESTHESIOLOGY, V116, P896, DOI 10.1097/ALN.0b013e31824b96a1

Bovill JG, 2010, ANESTH ANALG, V110, P1524, DOI 10.1213/ANE.0b013e3181d8d183

China Nutrition Society, 2008, CHIN RES DIET GUID, P15

Cieliska A, 2015, TUMOR BIOL, V36, P4655

Eslick GD, 2009, GASTROENTEROL CLIN N, V38, P17, DOI 10.1016/j.gtc.2009.01.008

Freiberg MS, 2004, DIABETES CARE, V27, P2954, DOI 10.2337/diacare.27.12.2954

Gach K, 2011, N-S ARCH PHARMACOL, V384, P221, DOI 10.1007/s00210-011-0672-4

Iida H, 2009, J PUBLIC HEALTH DENT, V69, P231, DOI 10.1111/j.1752-7325.2009.00128.x

Islami F, 2011, INT J CANCER, V129, P2473, DOI 10.1002/ijc.25885

Jemal A, 2009, CA-CANCER J CLIN, V59, P225, DOI 10.3322/caac.20006

Kamangar F, 2006, J CLIN ONCOL, V24, P2137, DOI 10.1200/JCO.2005.05.2308

Kuwano H, 2005, SURG TODAY, V35, P7, DOI 10.1007/s00595-004-2885-3

Lennon FE, 2012, ANESTHESIOLOGY, V116, P940, DOI 10.1097/ALN.0b013e31824b9512

Lohman TG., 1988, ANTHROPOMETRIC STAND

Morita M, 2010, INT J CLIN ONCOL, V15, P126, DOI 10.1007/s10147-010-0056-7

Moss J, 2008, MAYO CLIN PROC, V83, P1116, DOI 10.4065/83.10.1116

Qin YR, 2008, WORLD J GASTROENTERO, V14, P1828, DOI 10.3748/wjg.14.1828

Ray R, 2011, P NATL ACAD SCI USA, V108, P9268, DOI 10.1073/pnas.1018699108

Shabalina SA, 2009, HUM MOL GENET, V18, P1037, DOI 10.1093/hmg/ddn439

Sobti RC, 2007, CANCER GENET CYTOGEN, V175, P91, DOI 10.1016/j.cancergencyto.2007.01.001

Sugimoto T, 2009, GENE CHROMOSOME CANC, V48, P132, DOI 10.1002/gcc.20626

Taghavi N, 2010, ARCH IRAN MED, V13, P235

Talukdar FR, 2013, PLOS ONE, V8, DOI 10.1371/journal.pone.0060996

Tavare AN, 2012, INT J CANCER, V130, P1237, DOI 10.1002/ijc.26448

Toh Y, 2010, INT J CLIN ONCOL, V15, P135, DOI 10.1007/s10147-010-0057-6

Tomar SL, 2000, J PERIODONTOL, V71, P743, DOI 10.1902/jop.2000.71.5.743

Wang S, 2013, INT J CLIN ONCOL, V18, P666, DOI 10.1007/s10147-012-0441-5

Zhang F, 2013, WORLD J SURG ONCOL, V11, DOI 10.1186/1477-7819-11-249

NR 29

TC 9

Z9 9

U1 0

U2 3

PU WILEY

PI HOBOKEN

PA 111 RIVER ST, HOBOKEN, NJ 07030 USA

SN 0887-8013

EI 1098-2825

J9 J CLIN LAB ANAL

JI J. Clin. Lab. Anal.

PD JAN

PY 2017

VL 31

IS 1

AR e22018

DI 10.1002/jcla.22018

PG 6

WC Medical Laboratory Technology

WE Science Citation Index Expanded (SCI-EXPANDED)

SC Medical Laboratory Technology

GA EK4CH

UT WOS:000393874200009

PM 27373278

OA Green Published

DA 2022-05-19

ER

PT J

AU Kong, J

Zhu, SC

Shi, GF

Liu, ZK

Zhang, J

Ren, JL

AF Kong, Jie

Zhu, Shuchai

Shi, Gaofeng

Liu, Zhikun

Zhang, Jun

Ren, Jialiang

TI Prediction of Locoregional Recurrence-Free Survival of Oesophageal

Squamous Cell Carcinoma After Chemoradiotherapy Based on an Enhanced

CT-Based Radiomics Model

SO FRONTIERS IN ONCOLOGY

LA English

DT Article

DE oesophageal squamous cell carcinoma; chemoradiotherapy; radiomics;

enhanced CT; locoregional recurrence-free survival

ID LYMPH-NODE METASTASIS; CANCER; PATTERNS; FEATURES; NOMOGRAM; SYSTEM

AB Background and Purpose

Chemoradiotherapy is the standard treatment for moderate and advanced oesophageal cancer. The aim of this study was to establish a predictive model based on enhanced computed tomography examination, and to evaluate its clinical value for detecting locoregional recurrence-free survival (LRFS) in cases of oesophageal squamous cell carcinoma after radiotherapy.</p> Materials and Methods

In total, 218 patients with pathologically diagnosed oesophageal squamous cell carcinoma who received radical chemoradiotherapy from July 2016 to December 2017 were collected in this study. Patients were randomly divided into either a training group (n=153) or a validation group (n=65) in a 7:3 ratio. Clinical patient information was then recorded. The enhanced computed tomography scan images of the patients were imported into 3D-slicer software (version 4.8.1), and the radiomic features were extracted by the Python programme package. In the training group, the dimensionality reduction of the radiomic features was implemented by Lasso regression, and then a radiological label, the model of predicting LRFS, was established and evaluated. To achieve a better prediction performance, the radiological label was combined with clinical risk factor information to construct a radiomics nomogram. A receiver operating characteristic curve was used to evaluate the efficacy of different models. Calibration curves were used to assess the consistency between the predicted and observed recurrence risk, and the Hosmer-Lemeshow method was used to test model fitness. The C-index evaluated the discriminating ability of the prediction model. Decision curve analysis was used to determine the clinical value of the constructed prediction model.</p> Results

Of the 218 patients followed up in this study, 44 patients (28.8%) in the training group and 21 patients (32.3%) in the validation group experienced recurrence. There was no difference in LRFS between the two groups (chi(2 =) 0.525, P=0.405). Lasso regression was used in the training group to select six significant radiomic features. The radiological label established using these six features had a satisfactory prediction performance. The C-index was 0.716 (95% CI: 0.645-0.787) in the training group and 0.718 (95% CI: 0.612-0.825) in the validation group. The radiomics nomogram, which included the radiological label and clinical risk factors, achieved a better prediction than the radiological label alone. The C-index was 0.742 (95% CI: 0.674-0.810) in the training group and 0.715 (95% CI: 0.609-0.820) in the validation group. The results of the calibration curve and decision curve analyses indicated that the radiomics nomogram was superior in predicting LRFS of oesophageal carcinoma after radiotherapy.</p> Conclusions

A radiological label was successfully established to predict the LRFS of oesophageal squamous cell carcinoma after radiotherapy. The radiomics nomogram was complementary to the clinical prognostic features and could improve the prediction of the LRFS after radiotherapy for oesophageal cancer.</p>

C1 [Kong, Jie; Zhu, Shuchai; Liu, Zhikun; Zhang, Jun] Hebei Med Univ, Dept Radiat Oncol, Hosp 4, Shijiazhuang, Hebei, Peoples R China.

[Shi, Gaofeng] Hebei Med Univ, Dept Computed Tomog & Magnet Resonance, Hosp 4, Shijiazhuang, Hebei, Peoples R China.

[Ren, Jialiang] GE Healthcare, Pharmaceut Diag, Beijing, Peoples R China.

RP Zhu, SC (corresponding author), Hebei Med Univ, Dept Radiat Oncol, Hosp 4, Shijiazhuang, Hebei, Peoples R China.

EM sczhu1965@163.com

FU National Natural Science Foundation of China [81872456]; Health

Innovation Special Fund of Hebei Province [213777104D]

FX This study was supported by the National Natural Science Foundation of

China (Grant No. 81872456) and the Health Innovation Special Fund of

Hebei Province (Grant No. 213777104D).

CR Anthony GJ, 2017, MED PHYS, V44, P3686, DOI 10.1002/mp.12282

Barbetta A, 2019, J THORAC CARDIOV SUR, V157, P1249, DOI 10.1016/j.jtcvs.2018.09.136

Bray F, 2018, CA-CANCER J CLIN, V68, P394, DOI 10.3322/caac.21492

Di Pardo BJ, 2016, WORLD J SURG, V40, P395, DOI 10.1007/s00268-015-3356-2

Dong XZ, 2017, ONCOTARGET, V8, P14969, DOI 10.18632/oncotarget.14743

Fried DV, 2016, RADIOLOGY, V278, P214, DOI 10.1148/radiol.2015142920

Ghaly G, 2018, ANN THORAC SURG, V105, P357, DOI 10.1016/j.athoracsur.2017.10.039

Hamai Y, 2018, SURGERY, V164, P40, DOI 10.1016/j.surg.2018.01.011

Huang KX, 2020, NEUROPSYCHOPHARMACOL, V45, P358, DOI 10.1038/s41386-019-0551-0

Huang RW, 2014, WORLD J SURG ONCOL, V12, DOI 10.1186/1477-7819-12-170

Huang YQ, 2016, J CLIN ONCOL, V34, P2157, DOI 10.1200/JCO.2015.65.9128

Jayaprakasam VS, 2020, AM J ROENTGENOL, V215, P1072, DOI 10.2214/AJR.20.22791

Ji GW, 2019, EBIOMEDICINE, V50, P156, DOI 10.1016/j.ebiom.2019.10.057

Ji GW, 2019, RADIOLOGY, V290, P90, DOI 10.1148/radiol.2018181408

Keek S, 2020, PLOS ONE, V15, DOI 10.1371/journal.pone.0232639

Lagergren J, 2017, LANCET, V390, P2383, DOI 10.1016/S0140-6736(17)31462-9

Lambin P, 2012, EUR J CANCER, V48, P441, DOI 10.1016/j.ejca.2011.11.036

Liang WJ, 2019, CLIN CANCER RES, V25, P584, DOI 10.1158/1078-0432.CCR-18-1305

Lou FR, 2013, J THORAC ONCOL, V8, P1558, DOI 10.1097/01.JTO.0000437420.38972.fb

Mattonen SA, 2014, MED PHYS, V41, DOI 10.1118/1.4866219

Oppedijk V, 2014, J CLIN ONCOL, V32, P385, DOI 10.1200/JCO.2013.51.2186

Parry K, 2015, ANN SURG ONCOL, V22, pS1292, DOI 10.1245/s10434-015-4840-5

Qiu QT, 2020, FRONT ONCOL, V10, DOI 10.3389/fonc.2020.01398

Rice TW, 2016, DIS ESOPHAGUS, V29, P913, DOI 10.1111/dote.12540

Shen C, 2018, TRANSL ONCOL, V11, P815, DOI 10.1016/j.tranon.2018.04.005

Tan ZH, 2018, J THORAC DIS, V10, P5254, DOI 10.21037/jtd.2018.08.88

Torre LA, 2015, CA-CANCER J CLIN, V65, P87, DOI 10.3322/caac.21262

van Griethuysen JJM, 2017, CANCER RES, V77, pE104, DOI 10.1158/0008-5472.CAN-17-0339

Weksler B, 2017, J THORAC CARDIOV SUR, V154, P1787, DOI 10.1016/j.jtcvs.2017.07.036

Welsh J, 2012, CANCER-AM CANCER SOC, V118, P2632, DOI 10.1002/cncr.26586

Wu SX, 2018, EBIOMEDICINE, V34, P76, DOI 10.1016/j.ebiom.2018.07.029

Wu SX, 2017, CLIN CANCER RES, V23, P6904, DOI 10.1158/1078-0432.CCR-17-1510

Xu XP, 2019, J MAGN RESON IMAGING, V50, P1893, DOI 10.1002/jmri.26749

Yang ZN, 2019, J RADIAT RES, V60, P538, DOI 10.1093/jrr/rrz027

Zhang LL, 2019, EBIOMEDICINE, V42, P270, DOI 10.1016/j.ebiom.2019.03.050

Zhang WY, 2018, BMC CANCER, V18, DOI 10.1186/s12885-018-4796-5

NR 36

TC 0

Z9 0

U1 4

U2 4

PU FRONTIERS MEDIA SA

PI LAUSANNE

PA AVENUE DU TRIBUNAL FEDERAL 34, LAUSANNE, CH-1015, SWITZERLAND

SN 2234-943X

J9 FRONT ONCOL

JI Front. Oncol.

PD SEP 24

PY 2021

VL 11

AR 739933

DI 10.3389/fonc.2021.739933

PG 12

WC Oncology

WE Science Citation Index Expanded (SCI-EXPANDED)

SC Oncology

GA WD3UT

UT WOS:000704870700001

PM 34631575

OA gold, Green Published

DA 2022-05-19

ER

PT J

AU Huang, J

Xu, JM

Chen, Y

Zhuang, W

Zhang, YP

Chen, ZD

Chen, J

Zhang, HL

Niu, ZX

Fan, QX

Lin, LZ

Gu, KS

Liu, Y

Ba, Y

Miao, ZH

Jiang, XD

Zeng, M

Chen, JH

Fu, ZC

Gan, L

Wang, J

Zhan, XB

Liu, TS

Li, ZP

Shen, L

Shu, YQ

Zhang, T

Yang, Q

Zou, JJ

AF Huang, Jing

Xu, Jianming

Chen, Yun

Zhuang, Wu

Zhang, Yiping

Chen, Zhendong

Chen, Jia

Zhang, Helong

Niu, Zuoxing

Fan, Qingxia

Lin, Lizhu

Gu, Kangsheng

Liu, Ying

Ba, Yi

Miao, Zhanhui

Jiang, Xiaodong

Zeng, Ming

Chen, Jianhua

Fu, Zhichao

Gan, Lu

Wang, Jun

Zhan, Xianbao

Liu, Tianshu

Li, Zhiping

Shen, Lin

Shu, Yongqian

Zhang, Tao

Yang, Qing

Zou, Jianjun

CA ESCORT Study Grp

TI Camrelizumab versus investigator's choice of chemotherapy as second-line

therapy for advanced or metastatic oesophageal squamous cell carcinoma

(ESCORT): a multicentre, randomised, open-label, phase 3 study

SO LANCET ONCOLOGY

LA English

DT Article

ID II TRIAL; DOCETAXEL; IRINOTECAN; CANCER; PACLITAXEL; SAFETY

AB Background Patients with advanced or metastatic oesophageal squamous cell carcinoma have poor prognosis and few treatment options after first-line therapy. We aimed to assess efficacy and safety of the anti-PD-1 antibody camrelizumab versus investigator's choice of chemotherapy in previously treated patients.

Methods ESCORT is a randomised, open-label, phase 3 study of patients aged 18 to 75 years with a histological or cytological diagnosis of advanced or metastatic oesophageal squamous cell carcinoma done at 43 hospitals in China. Eligible patients had an Eastern Cooperative Oncology Group (ECOG) performance status of 0 or 1, and had progressed on, or were intolerant to, first-line standard therapy. Patients were randomly assigned (1:1) to camrelizumab (200 mg every 2 weeks) or chemotherapy with docetaxel (75 mg/m(2) every 3 weeks) or irinotecan (180 mg/m(2) every 2 weeks), all given intravenously. Central randomisation was done using the Randomization and Trial Supply Management system with block size randomly generated as four or six and stratified by disease and ECOG performance status. The primary endpoint was overall survival, assessed in randomised patients who had received at least one dose of treatment. Safety was assessed in all treated patients. The trial is registered with ClinicalTrials.gov , NCT03099382, and is closed to new participants.

Findings From May 10,2017, to July 24,2018,457 (75%) of 607 screened patients were randomly assigned to treatment, of whom 228 received camrelizumab treatment and 220 received chemotherapy. As of data cutoff on May 6, 2019, with a median follow-up time of 8.3 months (IQR 4.1-12.8) in the camrelizumab group and 6.2 months (3.6-10.1) in the chemotherapy group, median overall survival was 8.3 months (95% CI 6.8-9.7) in the camrelizumab group and 6.2 months (5.7-6.9) in the chemotherapy group (hazard ratio 0.71 [95% CI 0.57-0.87]; two-sided p=0.0010). The most common treatment-related adverse events of grade 3 or worse were anaemia (camrelizumab vs chemotherapy: six [3%] vs 11 [5%]), abnormal hepatic function (four [2%] vs one [<1%]), and diarrhoea (three [1%] vs nine [4%]). Serious treatment-related adverse events occurred in 37 (16%) of 228 patients in the camrelizumab group, and in 32 (15%) of 220 patients in the chemotherapy group. Ten treatment-related deaths occurred, seven (3%) in the camrelizumab group (three deaths from unknown causes, one enterocolitis, one hepatic function abnormal, one pneumonitis, and one myocarditis) and three (1%) in the chemotherapy group (two deaths from unknown causes, and one gastrointestinal haemorrhage).

Interpretation Second-line camrelizumab significantly improved overall survival in patients with advanced or metastatic oesophageal squamous cell carcinoma compared with chemotherapy, with a manageable safety profile. It might represent a potential option of standard second-line treatment for patients with oesophageal squamous cell carcinoma in China. Copyright (C) 2020 Elsevier Ltd. All rights reserved.

C1 [Huang, Jing] Chinese Acad Med Sci & Peking Union Med Coll, Dept Med Oncol, Natl Canc Ctr, Natl Clin Res Ctr Canc, Beijing, Peoples R China.

[Xu, Jianming] PLA, Gen Hosp, Med Ctr 5, Dept Gastrointestinal Oncol,Canc Ctr, Beijing 100071, Peoples R China.

[Chen, Yun] Fudan Univ, Dept Radiat Oncol, Shanghai Canc Ctr, Shanghai, Peoples R China.

[Liu, Tianshu] Fudan Univ, Dept Oncol, Zhongshan Hosp, Shanghai, Peoples R China.

[Zhuang, Wu] Fujian Prov Canc Hosp, Dept Thorac Med Oncol, Fuzhou, Peoples R China.

[Zhang, Yiping] Zhejiang Canc Hosp, Dept Thorac Oncol, Hangzhou, Peoples R China.

[Chen, Zhendong] Anhui Med Univ, Affiliated Hosp 2, Dept Oncol, Hefei, Peoples R China.

[Gu, Kangsheng] Anhui Med Univ, Affiliated Hosp 1, Med Oncol, Hefei, Peoples R China.

[Chen, Jia] Jiangsu Canc Hosp, Dept Oncol, Nanjing, Peoples R China.

[Zhang, Helong] Fourth Mil Med Univ, Tangdu Hosp, Dept Oncol, Xian, Peoples R China.

[Niu, Zuoxing] Shandong Canc Hosp, Internal Med Ward 4, Jinan, Peoples R China.

[Fan, Qingxia] Zhengzhou Univ, Med Oncol, Affiliated Hosp 1, Zhengzhou, Peoples R China.

[Lin, Lizhu] Guangzhou Med Univ, Affiliated Hosp 1, Ctr Oncol, Guangzhou, Peoples R China.

[Liu, Ying; Liu, Tianshu] Zhengzhou Univ, Henan Canc Hosp, Med Oncol, Affiliated Canc Hosp, Zhengzhou, Peoples R China.

[Ba, Yi] Tianjin Canc Hosp, Med Oncol, Tianjin, Peoples R China.

[Miao, Zhanhui] Xinxiang Med Univ, Affiliated Hosp 1, Dept Oncol, Xinxiang, Henan, Peoples R China.

[Jiang, Xiaodong] First Peoples Hosp Lianyungang, Dept Oncol, Lianyungang, Peoples R China.

[Zeng, Ming] Sichuan Acad Med Sci & Sichuan Prov Peoples Hosp, Ctr Oncol, Chengdu, Peoples R China.

[Chen, Jianhua] Cent South Univ, Canc Hosp, Dept Med Oncol, Changsha, Peoples R China.

[Fu, Zhichao] 900 Hosp Joint Logist Team, Dept Radiotherapy, Fuzhou, Peoples R China.

[Gan, Lu] Chongqing Med Univ, Affiliated Hosp 1, Dept Oncol, Chongqing, Peoples R China.

[Wang, Jun] Hebei Med Univ, Dept Radiotherapy, Hosp 4, Shijiazhuang, Hebei, Peoples R China.

[Zhan, Xianbao] Second Mil Med Univ, Changhai Hosp, Dept Med Oncol, Shanghai, Peoples R China.

[Li, Zhiping] Jiangxi Canc Hosp, Integrated Chinese & Western Med Oncol, Nanchang, Jiangxi, Peoples R China.

[Shen, Lin] Peking Univ, Canc Hosp & Inst, Dept Gastrointestinal Oncol, Beijing, Peoples R China.

[Shu, Yongqian] Jiangsu Prov Hosp, Med Oncol, Nanjing, Peoples R China.

[Zhang, Tao; Yang, Qing; Zou, Jianjun] Jiangsu Hengrui Med, Shanghai, Peoples R China.

RP Xu, JM (corresponding author), PLA, Gen Hosp, Med Ctr 5, Dept Gastrointestinal Oncol,Canc Ctr, Beijing 100071, Peoples R China.

EM jmxu2003@yahoo.com

RI Shu, Yongqian/ABD-5698-2021

OI Zhang, Tao/0000-0002-9543-5474

FU Jiangsu Hengrui Medicine

FX This study was funded by Jiangsu Hengrui Medicine. We are grateful to

all patients and their families and all members of the collaborative

group in this trial. We thank Tao Wang (Jiangsu Hengrui Medicine) for

his input in study design, Yuanyuan Yu (Jiangsu Hengrui Medicine) for

her contribution to the collection of data, and Hanying Li (Jiangsu

Hengrui Medicine) for her input in statistical analyses. We would also

like to acknowledge PAREXEL International (MA, USA) for statistical

analyses (funded by Hengrui, Jiangsu, China). Medical writing support

was provided by Tengfei Zhang (Jiangsu Hengrui Medicine) according to

Good Publication Practice Guidelines.

CR [Anonymous], 2019, J CLIN ONCOL S

Bray F, 2018, CA-CANCER J CLIN, V68, P394, DOI 10.3322/caac.21492

BROOKMEYER R, 1982, BIOMETRICS, V38, P29, DOI 10.2307/2530286

Burkart C, 2007, ANTICANCER RES, V27, P2845

Chen J, 2019, ANN ONCOL, V30

Chen XL, 2019, CANCER BIOL MED, V16, P173, DOI 10.20892/j.issn.2095-3941.2018.0172

Chi YC, 2001, COMMUN STAT-SIMUL C, V30, P743, DOI 10.1081/SAC-100107779

Fang WF, 2018, LANCET ONCOL, V19, P1338, DOI 10.1016/S1470-2045(18)30495-9

Fayers PM., 2001, EORTC QLQ C30 SCORIN

Huang J, 2019, CANCER-AM CANCER SOC, V125, P742, DOI 10.1002/cncr.31855

Huang J, 2018, CLIN CANCER RES, V24, P1296, DOI 10.1158/1078-0432.CCR-17-2439

Kanekiyo S, 2016, ANTICANCER RES, V36, P1923

Kato K, 2019, LANCET ONCOL, V20, P1506, DOI 10.1016/S1470-2045(19)30626-6

Kudo T, 2017, LANCET ONCOL, V18, P631, DOI 10.1016/S1470-2045(17)30181-X

Kulangara K, 2019, ARCH PATHOL LAB MED, V143, P330, DOI 10.5858/arpa.2018-0043-OA

Lin YS, 2013, J EPIDEMIOL, V23, P233, DOI 10.2188/jea.JE20120162

Lordick F, 2003, BRIT J CANCER, V89, P630, DOI 10.1038/sj.bjc.6601168

Mizota A, 2011, ONCOLOGY-BASEL, V81, P237, DOI 10.1159/000334057

Muhr-Wilkenshoff F, 2003, INT J COLORECTAL DIS, V18, P330, DOI 10.1007/s00384-002-0464-x

Muro K, 2004, ANN ONCOL, V15, P955, DOI 10.1093/annonc/mdh231

Nie J, 2019, J CLIN ONCOL, V37, P1479, DOI 10.1200/JCO.18.02151

Shen L, 2018, ANN ONCOL, V29, P205

Shim HJ, 2010, AM J CLIN ONCOL-CANC, V33, P624, DOI 10.1097/COC.0b013e3181bead92

Shirakawa T, 2014, CANCER CHEMOTH PHARM, V74, P1207, DOI 10.1007/s00280-014-2597-3

US Food and Drug Administration, 2019, PRESCR INF KEYTRUDA

van Kleef JJ, 2020, JNCI-J NATL CANCER I, V112, P12, DOI 10.1093/jnci/djz133

Xu JM, 2019, CLIN CANCER RES, V25, P515, DOI 10.1158/1078-0432.CCR-18-2484

Zhang HZ, 2012, CHIN J CANCER, V31, P281, DOI 10.5732/cjc.011.10390

Zhou C., 2019, J CLIN ONCOL, V37, P9112, DOI [10.1200/JCO.2019.37.15_suppl.9112, DOI 10.1200/JCO.2019.37.15_SUPPL.9112]

NR 29

TC 108

Z9 127

U1 14

U2 33

PU ELSEVIER SCIENCE INC

PI NEW YORK

PA STE 800, 230 PARK AVE, NEW YORK, NY 10169 USA

SN 1470-2045

EI 1474-5488

J9 LANCET ONCOL

JI Lancet Oncol.

PD JUN

PY 2020

VL 21

IS 6

BP 832

EP 842

DI 10.1016/S1470-2045(20)30110-8

PG 11

WC Oncology

WE Science Citation Index Expanded (SCI-EXPANDED)

SC Oncology

GA MD6BT

UT WOS:000544057400034

PM 32416073

DA 2022-05-19

ER

PT J

AU Peng, L

He, WW

Ye, F

Song, YN

Shi, XY

Zhang, J

Li, QY

Fang, Q

Xiao, WG

Han, YT

AF Peng, Lin

He, Wenwu

Ye, Feng

Song, Yane

Shi, Xinying

Zhang, Jiao

Li, Qingyun

Fang, Qiang

Xiao, Wenguang

Han, Yongtao

TI Identification of tumor-infiltrating lymphocyte subpopulations

correlated with patient prognosis in esophageal squamous cell carcinoma

SO JOURNAL OF INTERNATIONAL MEDICAL RESEARCH

LA English

DT Article

DE Esophageal squamous cell carcinoma; DNA panel sequencing; transcriptome

sequencing; tumor-infiltrating lymphocyte; myeloid-derived suppressor

cell; T helper type 2 cell

ID CANCER; SURVIVAL; CHEMOTHERAPY; NIVOLUMAB; PREDICT; P53; DNA

AB Objective

To identify biomarkers related to esophageal squamous cell carcinoma (ESCC) prognosis by analyzing genetic variations and the infiltration levels of tumor-infiltrating lymphocytes (TILs) in patients.

Methods

The clinical features of 61 patients with ESCC were collected. DNA panel sequencing was performed to screen differentially expressed genes (DEGs). Transcriptome sequencing was performed to identify gene expression profiles, and subsequent enrichment analysis of DEGs was conducted using Metascape.

Results

We identified 488 DEGs between patients with ESCC with distinct prognoses that were mainly enriched in the human immune response, fibrinogen complex, and protein activation cascade pathways. Among patients with ESCC treated with postoperative chemotherapy, those with a high infiltration level of myeloid-derived suppressor cells (MDSCs) had longer overall survival (OS), and OS was positively correlated with the infiltration level of T helper type 2 (Th2) cells among patients treated without chemotherapy after surgery. Additionally, in the case of MDSCs >0.7059 or Th2 cells <0.6290, patients receiving postoperative chemotherapy had a longer OS than those treated without chemotherapy following surgery.

Conclusion

The level of MDSCs or Th2 cells can be used as a biomarker for assessing the prognosis of patients with ESCC treated with or without postoperative chemotherapy, respectively.

C1 [Peng, Lin; He, Wenwu; Fang, Qiang; Xiao, Wenguang; Han, Yongtao] Univ Elect Sci & Technol China, Sch Med, Sichuan Canc Hosp & Inst, Dept Thorac Surg,Sichuan Canc Ctr, 55,Sect 4,South Renmin Rd, Chengdu 610041, Peoples R China.

[Ye, Feng] Sichuan Univ, West China Hosp, Pathol Lab, Chengdu, Peoples R China.

[Song, Yane; Shi, Xinying; Zhang, Jiao; Li, Qingyun] Genecast Biotechnol Co Ltd, Wuxi, Jiangsu, Peoples R China.

RP Han, YT (corresponding author), Univ Elect Sci & Technol China, Sch Med, Sichuan Canc Hosp & Inst, Dept Thorac Surg,Sichuan Canc Ctr, 55,Sect 4,South Renmin Rd, Chengdu 610041, Peoples R China.

EM yongtao_han@126.com

FU Sichuan Science and Technology Program [2018JY0277, 2019JDRC0076];

Bethune Charitable Foundation [320.320.2730.1876]

FX The research was supported by the Sichuan Science and Technology Program

(Grant number 2018JY0277 and 2019JDRC0076) and the Bethune Charitable

Foundation (Grant number 320.320.2730.1876).

CR Brahmer J, 2015, NEW ENGL J MED, V373, P123, DOI 10.1056/NEJMoa1504627

Bray F, 2018, CA-CANCER J CLIN, V68, P394, DOI 10.3322/caac.21492

Chalmers ZR, 2017, GENOME MED, V9, DOI 10.1186/s13073-017-0424-2

Charoentong P, 2017, CELL REP, V18, P248, DOI 10.1016/j.celrep.2016.12.019

Chen HL, 2015, MED ONCOL, V32, DOI 10.1007/s12032-014-0417-6

Chen KY, 2017, MED SCI MONITOR, V23, P4619, DOI 10.12659/MSM.904154

Chen X, 2019, ONCOL REP, V41, P2529, DOI 10.3892/or.2019.6992

Cho Y, 2003, CANCER RES, V63, P1555

Dunn GP, 2004, ANNU REV IMMUNOL, V22, P329, DOI 10.1146/annurev.immunol.22.012703.104803

Enzinger PC, 2003, NEW ENGL J MED, V349, P2241, DOI 10.1056/NEJMra035010

Guo XF, 2015, THORAC CANCER, V6, P146, DOI 10.1111/1759-7714.12155

Hickman ES, 2002, CURR OPIN GENET DEV, V12, P60, DOI 10.1016/S0959-437X(01)00265-9

Jesinghaus M, 2017, ONCOTARGET, V8, P46756, DOI 10.18632/oncotarget.18606

Kennedy SR, 2012, MECH AGEING DEV, V133, P118, DOI 10.1016/j.mad.2011.10.009

Komatsu S, 2014, DIGEST DIS SCI, V59, P1152, DOI 10.1007/s10620-013-3005-2

Li SH, 2019, SURGERY, V165, P946, DOI 10.1016/j.surg.2018.11.015

Lin SF, 2019, INT J CLIN EXP PATHO, V12, P2657

Mroz EA, 2015, PLOS MED, V12, DOI 10.1371/journal.pmed.1001786

Nguyen N, 2016, HEAD NECK-J SCI SPEC, V38, P1074, DOI 10.1002/hed.24406

Ono T, 2017, ONCOTARGET, V8, P92699, DOI 10.18632/oncotarget.21564

Reck M, 2016, NEW ENGL J MED, V375, P1823, DOI 10.1056/NEJMoa1606774

Saeki H, 2011, CLIN CANCER RES, V17, P1731, DOI 10.1158/1078-0432.CCR-10-1996

Saleh R, 2020, FRONT ONCOL, V10, DOI 10.3389/fonc.2020.604906

Schreiber RD, 2011, SCIENCE, V331, P1565, DOI 10.1126/science.1203486

Talevich E, 2016, PLOS COMPUT BIOL, V12, DOI 10.1371/journal.pcbi.1004873

Wakasaki T, 2020, CANCERS, V12, DOI 10.3390/cancers12082299

Wang X, 2013, ASIAN PAC J CANCER P, V14, P2429, DOI 10.7314/APJCP.2013.14.4.2429

Wang YX, 2015, J CANCER RES THER, V11, pC16, DOI 10.4103/0973-1482.163833

Weiss GJ, 2017, CLIN CANCER RES, V23, P5074, DOI 10.1158/1078-0432.CCR-17-0231

Wolchok JD, 2017, NEW ENGL J MED, V377, P1345, DOI 10.1056/NEJMoa1709684

Yamaguchi T, 2021, CANCER REP-US, V4, DOI 10.1002/cnr2.1389

Yamasaki M, 2010, ANN SURG ONCOL, V17, P634, DOI 10.1245/s10434-009-0851-4

Yoshioka T, 2008, BRIT J CANCER, V98, P1258, DOI 10.1038/sj.bjc.6604294

Zhang LZ, 2017, CHINESE J CANCER RES, V29, P149, DOI 10.21147/j.issn.1000-9604.2017.02.08

Zingg U, 2010, EJSO-EUR J SURG ONC, V36, P670, DOI 10.1016/j.ejso.2010.05.012

NR 35

TC 1

Z9 1

U1 0

U2 0

PU SAGE PUBLICATIONS LTD

PI LONDON

PA 1 OLIVERS YARD, 55 CITY ROAD, LONDON EC1Y 1SP, ENGLAND

SN 0300-0605

EI 1473-2300

J9 J INT MED RES

JI J. Int. Med. Res.

PD MAY

PY 2021

VL 49

IS 5

AR 03000605211016206

DI 10.1177/03000605211016206

PG 16

WC Medicine, Research & Experimental; Pharmacology & Pharmacy

WE Science Citation Index Expanded (SCI-EXPANDED)

SC Research & Experimental Medicine; Pharmacology & Pharmacy

GA UW4AG

UT WOS:000700100300001

PM 34044599

OA Green Published, gold

DA 2022-05-19

ER

PT J

AU Cao, TH

Shen, JQ

Pan, WB

Li, CL

Qiao, ZG

AF Cao, Tinghua

Shen, Jiaqing

Pan, Wenbin

Li, Chunliang

Qiao, Zhenguo

TI Upregulation of long noncoding RNA ANRIL correlates with tumor

progression and poor prognosis in esophageal squamous cell carcinoma

SO JOURNAL OF BUON

LA English

DT Article

DE ANRIL; esophageal squamous cell carcinoma; long noncoding RNA; prognosis

ID MESENCHYMAL TRANSITION; INVASION; PROLIFERATION; METASTASIS; MIGRATION;

CANCER; OVEREXPRESSION; EXPRESSION; KNOCKDOWN

AB Purpose: Esophageal cancer (EC) is the 9th most common carcinoma worldwide with poor prognosis. Specific biomarkers can help predicting the development of esophageal squamous cell carcinoma (ESCC), which can improve the assessment of prognosis. This study aimed to explore long noncoding RNA (lncRNA) ANRIL expression and its potential value in ESCC prognosis.

Methods: Quantitative reverse transcriptase-polymerase chain reaction (qRT-PCR) was utilized to detect lncRNA ANRIL expression in 50 pairs of ESCC and matched normal samples in order to explore the role of lncRNA ANRIL in ESCC. Moreover, the association was investigated between clinical characteristics of ESCC and the expression level of ANRIL.

Results: Disease-free survival (DFS) and overall survival (OS) were significantly shorter in ESCC patients with higher expression level of lncRNA ANRIL. ESCC tissues examined showed an obvious increment in ANRIL expression when compared to normal tissues. Furthermore, ANRIL was positively related to lymph nodes metastasis, TNM stage and tumor clinical stage. Moreover, upregulated ANRIL expression was remarkably associated with shorter survival in ESCC patients,which was also an independent prognostic factor for both OS and DFS.

Conclusions: This study suggested that lncRNA ANRIL could be a potential oncogene of ESCC. ANRIL expression might be served as another potential therapeutic target and prognostic biomarker for ESCC.

C1 [Cao, Tinghua] First Peoples Hosp Wujiang Dist, Dept Oncol, Suzhou, Peoples R China.

[Shen, Jiaqing] Soochow Univ, Affiliated Hosp 1, Dept Gastroenterol, Suzhou, Peoples R China.

[Pan, Wenbin] Suzhou Yongding Hosp, Dept Gen Surg, Suzhou, Peoples R China.

[Li, Chunliang; Qiao, Zhenguo] First Peoples Hosp Wujiang Dist, Dept Gastroenterol, 169 Gongyuan Rd, Suzhou, Peoples R China.

RP Li, CL (corresponding author), First Peoples Hosp Wujiang Dist, Dept Gastroenterol, 169 Gongyuan Rd, Suzhou, Peoples R China.

EM docchunliang@163.com

OI qiao, zhen guo/0000-0002-9079-956X

CR Chen FJ, 2013, MOL CARCINOGEN, V52, P908, DOI 10.1002/mc.21944

Cheng S, 2017, EXP THER MED, V14, P5121, DOI 10.3892/etm.2017.5123

Dong XC, 2018, J CELL BIOCHEM, V119, P2708, DOI 10.1002/jcb.26437

Gao XF, 2017, ONCOTARGETS THER, V10, P1017, DOI 10.2147/OTT.S129343

Huang C, 2015, ONCOL LETT, V10, P291, DOI 10.3892/ol.2015.3165

Lin YS, 2013, J EPIDEMIOL, V23, P233, DOI 10.2188/jea.JE20120162

Luo M, 2013, CANCER LETT, V333, P213, DOI 10.1016/j.canlet.2013.01.033

Ma J, 2018, J CANCER RES CLIN, V144, P205, DOI 10.1007/s00432-017-2543-y

Siegel RL, 2015, CA-CANCER J CLIN, V65, P5, DOI [10.3322/caac.21254, 10.1136/bmj.g1502, 10.1001/jamaoto.2014.2530, 10.3322/caac.21208]

Wang PL, 2016, MOL MED REP, V13, P3074, DOI 10.3892/mmr.2016.4913

Wang W, 2016, ONCOTARGET, V7, P25668, DOI 10.18632/oncotarget.8257

Wang YF, 2017, MOL CANCER, V16, DOI 10.1186/s12943-017-0702-z

Wu JH, 2018, SCI REP-UK, V8, DOI 10.1038/s41598-018-21708-z

Yao GL, 2017, BIOMED PHARMACOTHER, V88, P778, DOI 10.1016/j.biopha.2017.01.124

Yu GY, 2018, J BONE ONCOL, V11, P51, DOI 10.1016/j.jbo.2018.02.002

Zhao B, 2018, CANCER BIOMARK, V21, P613, DOI 10.3233/CBM-170683

Zhao P, 2017, J BUON, V22, P265

NR 17

TC 9

Z9 9

U1 0

U2 3

PU IMPRIMATUR PUBLICATIONS

PI ATHENS

PA 30 DEM POLIORKETES ST, ATHENS, 136 76, GREECE

SN 1107-0625

EI 2241-6293

J9 J BUON

JI J. BUON

PD NOV-DEC

PY 2018

VL 23

IS 6

BP 1862

EP 1866

PG 5

WC Oncology

WE Science Citation Index Expanded (SCI-EXPANDED)

SC Oncology

GA HB4JC

UT WOS:000451018200037

PM 30610814

DA 2022-05-19

ER

PT J

AU Liv, JL

Han, S

Hu, RH

Huang, J

Zhang, RY

Li, KZ

Li, L

Zhang, GA

Zhaog, HL

AF Liv, Jianli

Han, Sha

Hu, Ronghang

Huang, Jian

Zhang, Renya

Li, Kaizhi

Li, Lu

Zhang, Guoan

Zhaog, Hongli

TI CD51 is an independent unfavorable prognostic factor in esophageal

squamous cell carcinoma

SO PATHOLOGY RESEARCH AND PRACTICE

LA English

DT Article

DE Esophageal squamous cell carcinoma; CD51; Prognosis

ID INTEGRIN ALPHA-V; TGF-BETA; OVEREXPRESSION; PROLIFERATION; EXPRESSION;

OSTEOPONTIN; PROGRESSION; APOPTOSIS; LARYNGEAL

AB Background: Esophageal squamous cell carcinoma (ESCC) is a common cancer in East Asia and some other parts of the world with a dismal prognosis. CD51 (integrin alpha v),a transmembrane glycoprotein responsible for cell-to-matrix binding has been found to enhance tumor progression. However, its expression and clinicopathological significance in ESCC tumors are not fully understood. The purpose of this study was to investigate the expression level of CD51 and to explore its clinicopathological significance in ESCC.

Methods: The expression of CD51 in 122 ESCC samples was examined by immunohistochemistry and its clinicopathological significance was evaluated.

Results: The expression of CD51 was observed in tumor cell membrane and/or cytoplasm, with a positive rate of 48.36% (59/122). High expression of CD51 was significantly associated with lymph node metastasis (P = 0.031), tumor size (P = 0.028) and invasive depth (P = 0.027). Kaplan-Meier analysis revealed that positive expression of CD51 was correlated with poor overall survival of ESCC patients (P = 0.015). Multivariate analysis suggested that CD51 was an independent prognositic factor for ESCC (hazard ration = 1.604; 95% CI, 1.086-2.368; P = 0.017).

Conclusion These data suggested CD51 was a predictor for the prognosis of ESCC patients.

C1 [Liv, Jianli; Zhang, Renya; Li, Kaizhi; Li, Lu] Jining Med Univ, Affiliated Hosp, Dept Pathol, Jining 272000, Shandong, Peoples R China.

[Han, Sha] Jining Med Univ, Life Sci Expt Ctr, Jining 272000, Shandong, Peoples R China.

[Hu, Ronghang] Jining Med Univ, Affiliated Hosp, Dept Thorac Surg, Jining 272000, Shandong, Peoples R China.

[Huang, Jian] Jining Med Univ, Affiliated Hosp, Cent Lab, Jining 272029, Shandong, Peoples R China.

[Zhang, Guoan] Jining Med Univ, Forens Sci Ctr, 16 Taibaihu Rd, Jining 272067, Shandong, Peoples R China.

[Zhang, Guoan] Jining Med Univ, Canc Pathol Inst, Jining 272000, Shandong, Peoples R China.

[Zhaog, Hongli] Shandong Control Ctr Digest Dis, Dept Gastroenterol, 11 Taibai Rd, Jining 272067, Shandong, Peoples R China.

RP Zhang, GA (corresponding author), Jining Med Univ, Forens Sci Ctr, 16 Taibaihu Rd, Jining 272067, Shandong, Peoples R China.; Zhaog, HL (corresponding author), Shandong Control Ctr Digest Dis, Dept Gastroenterol, 11 Taibai Rd, Jining 272067, Shandong, Peoples R China.

EM zga2007@126.com; xushulinfeng@163.com

OI Zhang, Guoan/0000-0002-9489-6033; Huang, Jian/0000-0003-0149-1799

FU Natural Science Foundation of Shandong province [2013ZRC03007,

ZR2016HL25]; Jining Medical University [JY2017KJ040]; Science and

Technology Development Project of Jining Science and Technology Bureau

[2014jnjc17]; Jining science and technology boost the old and new

kinetic energy conversion project [2017SMNS001]

FX This study is supported by Natural Science Foundation of Shandong

province (2013ZRC03007, ZR2016HL25), Supporting Fund of Jining Medical

University (JY2017KJ040), Science and Technology Development Project of

Jining Science and Technology Bureau (2014jnjc17) and Jining science and

technology boost the old and new kinetic energy conversion project

(2017SMNS001).

CR Allum WH, 2009, J CLIN ONCOL, V27, P5062, DOI 10.1200/JCO.2009.22.2083

Chen WQ, 2016, CA-CANCER J CLIN, V66, P115, DOI 10.3322/caac.21338

Desgrosellier JS, 2010, NAT REV CANCER, V10, P9, DOI 10.1038/nrc2748

Ha SY, 2014, J CLIN PATHOL, V67, P576, DOI 10.1136/jclinpath-2013-202163

Horioka K, 2016, INT J ONCOL, V48, P1499, DOI 10.3892/ijo.2016.3374

Hynes RO, 2002, CELL, V110, P673, DOI 10.1016/S0092-8674(02)00971-6

Jemal A, 2011, CA-CANCER J CLIN, V61, P69, DOI [10.3322/caac.20107, 10.3322/caac.20115]

Jemal A, 2010, CANCER EPIDEM BIOMAR, V19, P1893, DOI 10.1158/1055-9965.EPI-10-0437

Lu JG, 2011, J CANCER RES CLIN, V137, P1613, DOI 10.1007/s00432-011-1024-y

Lu JG, 2009, EUR ARCH OTO-RHINO-L, V266, P89, DOI 10.1007/s00405-008-0675-z

Luo ZP, 2016, CANCER BIOTHER RADIO, V31, P153, DOI 10.1089/cbr.2016.1995

Massague J, 2008, CELL, V134, P215, DOI 10.1016/j.cell.2008.07.001

Rustgi AK, 2014, NEW ENGL J MED, V371, P2499, DOI 10.1056/NEJMra1314530

Saleh S, 2016, BMC CANCER, V16, DOI 10.1186/s12885-016-2396-9

Seguin L, 2015, TRENDS CELL BIOL, V25, P234, DOI 10.1016/j.tcb.2014.12.006

Sui X, 2018, CELL DEATH DIS, V9, DOI 10.1038/s41419-018-0541-x

Vogetseder A, 2013, INT J CANCER, V133, P2362, DOI 10.1002/ijc.28267

Waisberg J, 2014, ANTICANCER RES, V34, P5599

Wang J, 2017, ONCOGENE, V36, P1351, DOI 10.1038/onc.2016.299

Weis S., 2011, COLD SPRING HARB PER, V1, P6478, DOI DOI 10.1101/CSHPERSPECT.A006478

Wipff PJ, 2008, EUR J CELL BIOL, V87, P601, DOI 10.1016/j.ejcb.2008.01.012

Xie JJ, 2016, HUM PATHOL, V48, P69, DOI 10.1016/j.humpath.2015.09.029

Zhang GA, 2013, ONCOL LETT, V5, P797, DOI 10.3892/ol.2013.1125

NR 23

TC 0

Z9 1

U1 0

U2 1

PU ELSEVIER GMBH

PI MUNICH

PA HACKERBRUCKE 6, 80335 MUNICH, GERMANY

SN 0344-0338

EI 1618-0631

J9 PATHOL RES PRACT

JI Pathol. Res. Pract.

PD SEP

PY 2018

VL 214

IS 9

BP 1352

EP 1355

DI 10.1016/j.prp.2018.07.005

PG 4

WC Pathology

WE Science Citation Index Expanded (SCI-EXPANDED)

SC Pathology

GA GV3DC

UT WOS:000445973500017

PM 30049512

DA 2022-05-19

ER

PT J

AU Wu, YB

Lu, D

He, ZF

Jin, CG

AF Wu, Yuan-Bo

Lu, Di

He, Zhi-Feng

Jin, Chan-Guan

TI PIM1 polymorphism and PIM1 expression as predisposing factors of

esophageal squamous cell carcinoma in the Asian population

SO ONCOTARGETS AND THERAPY

LA English

DT Article

DE PIM1; polymorphism; esophageal squamous cell carcinoma; -1 882 A > T;

clinicopathological features; proto-oncogene

ID PROTOONCOGENE PIM-1; POOR-PROGNOSIS; CANCER; KINASE; SURVIVAL; TARGET;

RISK; ADENOCARCINOMA; EPIDEMIOLOGY; DOCETAXEL

AB Our study aimed to identify the association between a PIM1 polymorphism and PIM1 expression levels with clinicopathological features of esophageal squamous cell carcinoma (ESCC). A total of 168 patients with ESCC were recruited as the case group, and 180 healthy individuals were included as the control group. Polymerase chain reaction-direct sequencing was employed to analyze all genotypes containing the PIM1 -1 882 A > T mutation. Immunohistochemistry was used to detect PIM1 expression. The distributions of genotype AA and allele A of PIM1 -1 882 A > T were higher in the case group than in the control group (both P < 0.05). AT + TT carriers had a lower risk of ESCC than AA carriers (P < 0.05). PIM1 polymorphism was related to the invasion depth, degree of differentiation, and lymphatic metastasis of ESCC (P < 0.05). PIM1 expression was associated with lymphatic metastasis of ESCC and PIM1 polymorphism (both P < 0.05). PIM1 -1 882 A. T and the overexpression of PIM1 were associated with the clinicopathological features of ESCC, and PIM1 -1 882 A > T may help to reduce the risk of ESCC in the Asian population.

C1 [Wu, Yuan-Bo; Lu, Di; He, Zhi-Feng] Wenzhou Med Univ, Dept Cardiothorac Surg, Affiliated Hosp 1, Wenzhou, Zhejiang, Peoples R China.

[Jin, Chan-Guan] Wenzhou Med Univ, Dept Hepatobiliary Surg, Affiliated Hosp 2, 109 Xueyuan West Rd, Wenzhou 325000, Zhejiang, Peoples R China.

RP Jin, CG (corresponding author), Wenzhou Med Univ, Dept Hepatobiliary Surg, Affiliated Hosp 2, 109 Xueyuan West Rd, Wenzhou 325000, Zhejiang, Peoples R China.

EM jinchanguan1229@163.com

CR Akagi T, 2009, HAEMATOL-HEMATOL J, V94, P1301, DOI 10.3324/haematol.2009.005744

Baron BW, 2014, LEUKEMIA LYMPHOMA, V55, P2658, DOI 10.3109/10428194.2014.893303

Bodelon C, 2011, CANCER PREV RES, V4, P840, DOI 10.1158/1940-6207.CAPR-10-0389

Choi JY, 2010, J OTOLARYNGOL-HEAD N, V39, P28, DOI 10.2310/7070.2009.080295

CUYPERS HT, 1984, CELL, V37, P141

Decker S, 2014, MOL CANCER THER, V13, P1231, DOI 10.1158/1535-7163.MCT-13-0575-T

Di Pietro F, 2011, MOL CELL PROBE, V25, P44, DOI 10.1016/j.mcp.2010.10.003

Gorogh T, 2015, EUR ARCH OTO-RHINO-L, V272, P1251, DOI 10.1007/s00405-014-3073-8

He XY, 2015, BMC CANCER, V15, DOI 10.1186/s12885-015-1581-6

Holder SL, 2014, CURR CANCER DRUG TAR, V14, P105, DOI 10.2174/1568009613666131126113854

Hongo M, 2009, J GASTROEN HEPATOL, V24, P729, DOI 10.1111/j.1440-1746.2009.05824.x

Jessri M, 2011, NUTR J, V10, DOI 10.1186/1475-2891-10-137

Kim Dae Sik, 2008, Cancer Res Treat, V40, P190, DOI 10.4143/crt.2008.40.4.190

Li J, 2009, EXP HEMATOL, V37, P1284, DOI 10.1016/j.exphem.2009.08.002

Li S, 2010, ONCOL REP, V24, P997, DOI 10.3892/or_00000947

Liu HT, 2010, J SURG ONCOL, V102, P683, DOI 10.1002/jso.21627

Liu NB, 2015, ONCOTARGETS THER, V8, P3449, DOI 10.2147/OTT.S92862

Magnuson NS, 2010, FUTURE ONCOL, V6, P1461, DOI [10.2217/fon.10.106, 10.2217/FON.10.106]

Malinen M, 2013, MOL CELL ENDOCRINOL, V365, P270, DOI 10.1016/j.mce.2012.10.028

Muraski JA, 2007, NAT MED, V13, P1467, DOI 10.1038/nm1671

Peng XE, 2015, BMC MED GENET, V16, DOI 10.1186/s12881-015-0156-3

Peng YH, 2013, PLOS ONE, V8, DOI 10.1371/journal.pone.0076693

Reiser-Erkan C, 2008, CANCER BIOL THER, V7, P1352, DOI 10.4161/cbt.7.9.6418

Rice Thomas W, 2015, Korean J Thorac Cardiovasc Surg, V48, P157, DOI 10.5090/kjtcs.2015.48.3.157

Santio NM, 2010, MOL CANCER, V9, DOI 10.1186/1476-4598-9-279

Willert M, 2010, BASIC RES CARDIOL, V105, P267, DOI 10.1007/s00395-009-0055-x

Xu X, DIS ESOPHAGUS

Yan B, 2012, GASTRIC CANCER, V15, P188, DOI 10.1007/s10120-011-0097-2

Zemskova M, 2008, J BIOL CHEM, V283, P20635, DOI 10.1074/jbc.M709479200

Zhang L, 2015, ANN CLIN LAB SCI, V45, P433

Zhang YW, 2013, WORLD J GASTROENTERO, V19, P5598, DOI 10.3748/wjg.v19.i34.5598

NR 31

TC 1

Z9 1

U1 0

U2 0

PU DOVE MEDICAL PRESS LTD

PI ALBANY

PA PO BOX 300-008, ALBANY, AUCKLAND 0752, NEW ZEALAND

SN 1178-6930

J9 ONCOTARGETS THER

JI OncoTargets Ther.

PY 2016

VL 9

BP 2919

EP 2925

DI 10.2147/OTT.S103392

PG 7

WC Biotechnology & Applied Microbiology; Oncology

WE Science Citation Index Expanded (SCI-EXPANDED)

SC Biotechnology & Applied Microbiology; Oncology

GA DM0DO

UT WOS:000376013200001

PM 27274285

OA Green Published, gold

DA 2022-05-19

ER

PT J

AU Shi, ZZ

Shang, L

Jiang, YY

Shi, F

Xu, X

Wang, MR

Hao, JJ

AF Shi, Zhi-Zhou

Shang, Li

Jiang, Yan-Yi

Shi, Feng

Xu, Xin

Wang, Ming-Rong

Hao, Jia-Jie

TI Identification of genomic biomarkers associated with the

clinicopathological parameters and prognosis of esophageal squamous cell

carcinoma

SO CANCER BIOMARKERS

LA English

DT Article

DE Esophageal squamous cell carcinoma; MAP3K7; prognosis; biomarker

ID CHROMOSOMAL-ABERRATIONS; PROSTATE-CANCER; POOR-PROGNOSIS; HYBRIDIZATION;

GENE; MAP3K7; INHIBITION; PROFILES; DELETION; CHINESE

AB BACKGROUND: At present no objective prognostic biomarkers have been established in esophageal squamous cell carcinoma (ESCC).

OBJECTIVE: To identify the genomic biomarkers associated with clinicopathological factors and prognosis of ESCCs.

METHODS: Real-time PCR was used to analyze the copy number change and mRNA expression of genes. The survival curves were plotted according to Kaplan-Meier method and checked by log-rank test.

RESULTS: We revealed the copy number increase of CACNA1C (12p13.33) and MRPL21 (11q13.2) as well as decrease of VIPR2 (7q36.3) and MAP3K7 (6q15) in ESCC by Real-time PCR, and also found that MRPL21 was significantly overexpressed and VIPR2 was underexpressed in ESCC. Gain of CACNA1C was significantly associated with differentiation (P = 0.043), and loss of VIPR2 was significantly linked with good prognosis (P = 0.016). Most importantly, we revealed that loss of MAP3K7 was significantly correlated with good prognosis in ESCC patients (n = 159, P = 0.004), especially in Grade II and pN0 patients (n = 76, P = 0.005 and n = 74, P = 0.024). Multivariate analysis confirmed that MAP3K7 loss provided prognostic information in ESCC (HR, 0.454; 95% CI: 0.208-0.995; P = 0.048).

CONCLUSIONS: Loss of MAP3K7 may be a candidate prognostic factor in ESCC.

C1 [Shi, Zhi-Zhou] Kunming Univ Sci & Technol, Fac Med, 727 South Jingming Rd, Kunming 650500, Yunnan, Peoples R China.

[Shang, Li; Jiang, Yan-Yi; Shi, Feng; Xu, Xin; Wang, Ming-Rong; Hao, Jia-Jie] Peking Union Med Coll, Canc Inst Hosp, State Key Lab Mol Oncol, Beijing 100021, Peoples R China.

[Shang, Li; Jiang, Yan-Yi; Shi, Feng; Xu, Xin; Wang, Ming-Rong; Hao, Jia-Jie] Chinese Acad Med Sci, Beijing 100021, Peoples R China.

RP Shi, ZZ (corresponding author), Kunming Univ Sci & Technol, Fac Med, 727 South Jingming Rd, Kunming 650500, Yunnan, Peoples R China.

EM zhizhoushi@126.com; hjj8173@126.com

FU National Natural Science Foundation of China [81460425]; National

High-Tech R&D Program of China [2012AA020206]; Yunnan Provincial

Research Foundation for Basic Research, China [2013FD012]; Foundation

for the Talents of Kunming University of Science and Technology

[KKSY201226099]

FX This study was funded by the National Natural Science Foundation of

China (Grant No. 81460425) and National High-Tech R&D Program of China

(Grant No. 2012AA020206) and Yunnan Provincial Research Foundation for

Basic Research, China (Grant No. 2013FD012) and Foundation for the

Talents of Kunming University of Science and Technology (Grant No.

KKSY201226099).

CR Bokaei PB, 2007, VIROLOGY, V362, P38, DOI 10.1016/j.virol.2006.12.012

Carneiro A, 2008, BMC CANCER, V8, DOI 10.1186/1471-2407-8-98

Davis S.J., 2015, MOL CANC THER

Ferlay J, 2015, INT J CANCER, V136, pE359, DOI 10.1002/ijc.29210

Giroux V, 2009, CANCER GENE THER, V16, P731, DOI 10.1038/cgt.2009.22

Hao J.J., 2014, J GASTROENTEROL

Hennessey JA, 2013, HEART RHYTHM, V10, P1886, DOI 10.1016/j.hrthm.2013.09.064

Hirasaki S, 2007, ONCOLOGIST, V12, P406, DOI 10.1634/theoncologist.12-4-406

Hou J, 2014, HUM PATHOL, V45, P352, DOI 10.1016/j.humpath.2013.09.011

Kim JC, 2008, WORLD J GASTROENTERO, V14, P6662, DOI 10.3748/wjg.14.6662

Kluth M, 2013, MODERN PATHOL, V26, P975, DOI 10.1038/modpathol.2012.236

Kwong D, 2004, HUM PATHOL, V35, P309, DOI 10.1016/j.humpath.2003.10.020

Light KC, 2013, PSYCHONEUROENDOCRINO, V38, P2983, DOI 10.1016/j.psyneuen.2013.08.008

Liu W, 2007, CLIN CANCER RES, V13, P5028, DOI 10.1158/1078-0432.CCR-07-0300

Martin SE, 2011, CURR CANCER DRUG TAR, V11, P976

Nieratschker V, 2011, EXPERT REV NEUROTHER, V11, P937, DOI [10.1586/ERN.11.84, 10.1586/ern.11.84]

Shi ZZ, 2013, CLIN CANCER RES, V19, P5867, DOI 10.1158/1078-0432.CCR-12-3753

Shi ZZ, 2011, GENE CHROMOSOME CANC, V50, P518, DOI 10.1002/gcc.20875

Singh A, 2012, CELL, V148, P639, DOI 10.1016/j.cell.2011.12.033

Sugimoto T, 2009, GENE CHROMOSOME CANC, V48, P132, DOI 10.1002/gcc.20626

Ueno T, 2002, CLIN CANCER RES, V8, P526

Vacic V, 2011, NATURE, V471, P499, DOI 10.1038/nature09884

Yen CC, 2001, CANCER, V92, P2769, DOI 10.1002/1097-0142(20011201)92:11<2769::AID-CNCR10118>3.0.CO;2-M

NR 23

TC 7

Z9 7

U1 1

U2 6

PU IOS PRESS

PI AMSTERDAM

PA NIEUWE HEMWEG 6B, 1013 BG AMSTERDAM, NETHERLANDS

SN 1574-0153

EI 1875-8592

J9 CANCER BIOMARK

JI Cancer Biomark.

PY 2015

VL 15

IS 6

BP 755

EP 761

DI 10.3233/CBM-150517

PG 7

WC Oncology

WE Science Citation Index Expanded (SCI-EXPANDED)

SC Oncology

GA DA4WJ

UT WOS:000367802400005

PM 26406417

DA 2022-05-19

ER

PT J

AU Fujihara, S

Kobara, H

Nishiyama, N

Hirose, K

Iwama, H

Masaki, T

AF Fujihara, Shintaro

Kobara, Hideki

Nishiyama, Noriko

Hirose, Kayo

Iwama, Hisakazu

Masaki, Tsutomu

TI MicroRNA Expression Profiles in Superficial Esophageal Squamous Cell

Carcinoma before Endoscopic Submucosal Dissection: A Pilot Study

SO INTERNATIONAL JOURNAL OF MOLECULAR SCIENCES

LA English

DT Article

DE esophageal squamous cell carcinoma; microRNA; endoscopy

ID INVASION; DIAGNOSIS; BIOMARKERS; MANAGEMENT; GROWTH; CANCER

AB Esophageal squamous cell carcinoma (ESCC) has a poor prognosis when diagnosed at an advanced stage, and early detection and treatment are essential to improve survival. However, intraobserver and interobserver variation make the diagnosis of superficial ESCC difficult, and suitable biomarkers are urgently needed. Here, we compared the microRNA (miRNA) expression profiles of superficial ESCC tissues and adjacent normal tissues obtained immediately before esophageal endoscopic submucosal dissection. We found that ESCC and normal tissues differed in their miRNA expression profiles. In particular, miR-21-5p and miR-146b-5p were significantly upregulated and miR-210-3p was significantly downregulated in tumor tissues compared with normal tissues. We also detected significant associations between miRNA expression and ESCC invasion depth and lymphovascular invasion. The same differential expression of miR-21-5p, miR-146b-5p, and miR-210-3p was detected in ESCC cell lines compared with normal esophageal epithelial cells in vitro. However, transfection of ESCC cells with miR-210-3p and miR-21-5p mimics or inhibitors had partial effects on cell proliferation and invasion in vitro. These results indicate that miRNA expression is significantly deregulated in superficial ESCC, and suggest that the potential contribution of differentially expressed miRNAs to the malignant phenotype should be further investigated.

C1 [Fujihara, Shintaro; Kobara, Hideki; Nishiyama, Noriko; Hirose, Kayo; Masaki, Tsutomu] Kagawa Univ, Grad Sch Med, Fac Med, Dept Gastroenterol & Neurol, 1750-1 Ikenobe, Miki, Kagawa 7610793, Japan.

[Iwama, Hisakazu] Kagawa Univ, Life Sci Res Ctr, 1750-1 Ikenobe, Miki, Kagawa 7610793, Japan.

RP Fujihara, S (corresponding author), Kagawa Univ, Grad Sch Med, Fac Med, Dept Gastroenterol & Neurol, 1750-1 Ikenobe, Miki, Kagawa 7610793, Japan.

EM joshin@med.kagawa-u.ac.jp; kobara@med.kagawa-u.ac.jp;

n-nori@med.kagawa-u.ac.jp; mizuyou1@med.kagawa-u.ac.jp;

iwama@med.kagawa-u.ac.jp; tmasaki@med.kagawa-u.ac.jp

RI Fujihara, Shintaro/AAW-6092-2021

OI Fujihara, Shintaro/0000-0002-1250-6012; Kobara,

Hideki/0000-0002-8508-827X

FU Japan Society for the Promotion of Science (JSPS) KAKENHI [20K17022]

FX This work was supported by the Japan Society for the Promotion of

Science (JSPS) KAKENHI (grant number 20K17022). In addition, this

research was supported by a donated fund laboratories established by

Kagawa Prefecture.

CR Boussios S, 2020, J PERS MED, V10, DOI 10.3390/jpm10020041

Boussios S, 2019, J PERS MED, V9, DOI 10.3390/jpm9010012

Bray F, 2018, CA-CANCER J CLIN, V68, P394, DOI 10.3322/caac.21492

Couch G, 2016, CANCER PREV RES, V9, P558, DOI 10.1158/1940-6207.CAPR-15-0379

di Pietro M, 2018, GASTROENTEROLOGY, V154, P421, DOI 10.1053/j.gastro.2017.07.041

Fu HL, 2013, CELL BIOCHEM BIOPHYS, V67, P657, DOI 10.1007/s12013-013-9554-3

Guo Y, 2008, CANCER RES, V68, P26, DOI 10.1158/0008-5472.CAN-06-4418

Hiyoshi Y, 2009, CLIN CANCER RES, V15, P1915, DOI 10.1158/1078-0432.CCR-08-2545

Hu YX, 2011, INT J CANCER, V128, P132, DOI 10.1002/ijc.25330

Huh CW, 2018, CLIN TRANSL GASTROEN, V9, DOI 10.1038/s41424-018-0024-5

Ishihara R, 2017, BMC GASTROENTEROL, V17, DOI 10.1186/s12876-017-0574-0

Katada C, 2019, J GASTROINTEST CANC, V50, P292, DOI 10.1007/s12029-018-0075-6

Kato M, 2020, JGH OPEN, V4, P178, DOI 10.1002/jgh3.12246

Kitagawa Y, 2019, ESOPHAGUS-TOKYO, V16, P1, DOI 10.1007/s10388-018-0641-9

Lao-Sirieix P, 2012, NAT REV CLIN ONCOL, V9, P278, DOI 10.1038/nrclinonc.2012.35

Li P, 2013, DIGEST DIS SCI, V58, P3483, DOI 10.1007/s10620-013-2854-z

Matsueda K, 2021, J CLIN MED, V10, DOI 10.3390/jcm10010013

Morishita A, 2021, CANCERS, V13, DOI 10.3390/cancers13030514

Morishita A, 2020, AM J PHYSIOL-GASTR L, V318, pG401, DOI 10.1152/ajpgi.00269.2019

Muto M, 2010, J CLIN ONCOL, V28, P1566, DOI 10.1200/JCO.2009.25.4680

Odenthal M, 2013, INT J CANCER, V133, P2454, DOI 10.1002/ijc.28253

Wang C, 2016, BRIT J CANCER, V114, P290, DOI 10.1038/bjc.2015.463

Wang GQ, 2004, ANN THORAC SURG, V77, P1740, DOI 10.1016/j.athoracsur.2003.10.098

Wang K, 2018, ONCOL LETT, V16, P1196, DOI 10.3892/ol.2018.8720

Wang N, 2013, DIGEST DIS SCI, V58, P1863, DOI 10.1007/s10620-013-2612-2

Wang WL, 2018, J GASTROEN HEPATOL, V33, P1248, DOI 10.1111/jgh.14071

Wang YK, 2021, CANCERS, V13, DOI 10.3390/cancers13020321

Xia Y, 2019, ANTI-CANCER DRUG, V30, P38, DOI 10.1097/CAD.0000000000000687

Zhang F, 2014, CANCER LETT, V342, P121, DOI 10.1016/j.canlet.2013.08.037

NR 29

TC 0

Z9 0

U1 0

U2 0

PU MDPI

PI BASEL

PA ST ALBAN-ANLAGE 66, CH-4052 BASEL, SWITZERLAND

EI 1422-0067

J9 INT J MOL SCI

JI Int. J. Mol. Sci.

PD MAY

PY 2021

VL 22

IS 9

AR 4789

DI 10.3390/ijms22094789

PG 11

WC Biochemistry & Molecular Biology; Chemistry, Multidisciplinary

WE Science Citation Index Expanded (SCI-EXPANDED)

SC Biochemistry & Molecular Biology; Chemistry

GA SC0QA

UT WOS:000650386200001

PM 33946439

OA gold, Green Published

DA 2022-05-19

ER

PT J

AU Yu, DH

Ruan, XL

Huang, JY

Hu, WD

Chen, C

Xu, Y

Hou, JX

Li, S

AF Yu, Donghu

Ruan, Xiaolan

Huang, Jingyu

Hu, Weidong

Chen, Chen

Xu, Yu

Hou, Jinxuan

Li, Sheng

TI Comprehensive Analysis of Competitive Endogenous RNAs Network, Being

Associated With Esophageal Squamous Cell Carcinoma and Its Emerging Role

in Head and Neck Squamous Cell Carcinoma

SO FRONTIERS IN ONCOLOGY

LA English

DT Article

DE esophageal squamous cell carcinoma; head and neck squamous cell

carcinoma; prognosis; weighted gene co-expression network analysis;

competitive endogenous RNAs network

ID LONG NONCODING RNA; CANCER; GENE; EXPRESSION; PROMOTES; SURVIVAL; MIRNA;

CHEMORADIOTHERAPY; PROGRESSION; METASTASIS

AB Esophageal squamous cell carcinoma (ESCC) is a common malignancy with poor prognosis and survival rate. To identify meaningful long non-coding RNA (lncRNA), microRNA (miRNA), and messenger RNA (mRNA) modules related to the ESCC prognosis, The Cancer Genome Atlas-ESCC was downloaded and processed, and then, a weighted gene co-expression network analysis was applied to construct lncRNA co-expression networks, miRNA co-expression networks, and mRNA co-expression networks. Twenty-one hub lncRNAs, seven hub miRNAs, and eight hub mRNAs were clarified. Additionally, a competitive endogenous RNAs network was constructed, and the emerging role of the network involved in head and neck squamous cell carcinoma (HNSCC) was also analyzed using several webtools. The expression levels of eight hub genes (TBC1D2, ATP6V0E1, SPI1, RNASE6, C1QB, C1QC, CSF1R, and C1QA) were different between normal esophageal tissues and HNSCC tissues. The expression levels of TBC1D2 and ATP6V0E1 were related to the survival time of HNSCC. The competitive endogenous RNAs network might provide common mechanisms involving in ESCC and HNSCC. More importantly, useful clues were provided for clinical treatments of both diseases based on novel molecular advances.

C1 [Yu, Donghu; Chen, Chen; Li, Sheng] Wuhan Univ, Zhongnan Hosp, Dept Biol Repositories, Wuhan, Peoples R China.

[Yu, Donghu; Chen, Chen; Li, Sheng] Human Genet Resource Preservat Ctr Hubei Prov, Wuhan, Peoples R China.

[Ruan, Xiaolan] Wuhan Univ, Renmin Hosp, Dept Hematol, Wuhan, Peoples R China.

[Huang, Jingyu; Hu, Weidong] Wuhan Univ, Zhongnan Hosp, Dept Thorac Surg, Wuhan, Peoples R China.

[Xu, Yu] Wuhan Univ, Zhongnan Hosp, Dept Radiat & Med Oncol, Wuhan, Peoples R China.

[Hou, Jinxuan] Wuhan Univ, Zhongnan Hosp, Dept Thyroid & Breast Surg, Wuhan, Peoples R China.

RP Li, S (corresponding author), Wuhan Univ, Zhongnan Hosp, Dept Biol Repositories, Wuhan, Peoples R China.; Li, S (corresponding author), Human Genet Resource Preservat Ctr Hubei Prov, Wuhan, Peoples R China.; Hou, JX (corresponding author), Wuhan Univ, Zhongnan Hosp, Dept Thyroid & Breast Surg, Wuhan, Peoples R China.

EM jhou@whu.edu.cn; lisheng-znyy@whu.edu.cn

OI Li, Sheng/0000-0003-4070-7345

FU Zhongnan Hospital of Wuhan University Science, Technology and Innovation

Cultivating Fund [znpy2018097]; 351 Talent Project of Wuhan University;

Young & Middle-aged Medical Key Talents Training Project of Wuhan

[WHQG201901]

FX This work was supported by Zhongnan Hospital of Wuhan University

Science, Technology and Innovation Cultivating Fund (znpy2018097) and

the 351 Talent Project of Wuhan University (Luojia Young Scholars: SL)

and The grant number of Young & Middle-aged Medical Key Talents Training

Project of Wuhan is WHQG201901.

CR Agarwal V, 2015, ELIFE, V4, DOI 10.7554/eLife.05005

Anaya J, 2016, PEERJ COMPUT SCI, DOI 10.7717/peerj-cs.67

Ashktorab H, 2017, GASTROENTEROLOGY, V153, P910, DOI 10.1053/j.gastro.2017.08.018

Botia J, 2015, HUM HERED, V80, P105

Bray F, 2018, CA-CANCER J CLIN, V68, P394, DOI 10.3322/caac.21492

BROWN CJ, 1991, NATURE, V349, P38, DOI 10.1038/349038a0

Chandrashekar DS, 2017, NEOPLASIA, V19, P649, DOI 10.1016/j.neo.2017.05.002

Chen DL, 2016, J EXP CLIN CANC RES, V35, DOI 10.1186/s13046-016-0420-1

Chen YH, 2017, SCI REP-UK, V7, DOI 10.1038/srep41785

Chen ZZ, 2019, BIOMED PHARMACOTHER, V109, P2228, DOI 10.1016/j.biopha.2018.11.049

Cheng J, 2017, ONCOTARGET, V8, P30265, DOI 10.18632/oncotarget.15754

Guo G, 2015, ONCOGENE, V34, P1768, DOI 10.1038/onc.2014.131

Han JDJ, 2004, NATURE, V430, P88, DOI 10.1038/nature02555

Higashijima A, 2013, PRENATAL DIAG, V33, P214, DOI 10.1002/pd.4045

Huang DW, 2009, NAT PROTOC, V4, P44, DOI 10.1038/nprot.2008.211

Iqbal J, 2015, JAMA-J AM MED ASSOC, V313, P165, DOI 10.1001/jama.2014.17322

Jiang H, 2017, MOL BIOSYST, V13, P1182, DOI 10.1039/c7mb00094d

Langfelder P, 2008, BIOINFORMATICS, V24, P719, DOI 10.1093/bioinformatics/btm563

Langfelder P, 2008, BMC BIOINFORMATICS, V9, DOI 10.1186/1471-2105-9-559

Li JG, 2014, GUT, V63, P1700, DOI 10.1136/gutjnl-2013-305806

Liang Y, 2017, BIOSCIENCE REP, V37, DOI 10.1042/BSR20171019

Lin CY, 2018, CLIN CANCER RES, V24, P486, DOI 10.1158/1078-0432.CCR-17-1851

Mimura K, 2018, FUKUSHIMA J MED SCI, V64, P46, DOI 10.5387/fms.2018-09

Natsuizaka M, 2017, NAT COMMUN, V8, DOI 10.1038/s41467-017-01500-9

Ohnuma H, 2018, CANCER SCI, V109, P3554, DOI 10.1111/cas.13772

Okamoto H, 2018, ESOPHAGUS-TOKYO, V15, P281, DOI 10.1007/s10388-018-0627-7

Paraskevopoulou MD, 2016, NUCLEIC ACIDS RES, V44, pD231, DOI 10.1093/nar/gkv1270

Paraskevopoulou MD, 2013, NUCLEIC ACIDS RES, V41, pW169, DOI 10.1093/nar/gkt393

Pardo OE, 2016, EMBO REP, V17, P570, DOI 10.15252/embr.201540970

Perou CM, 2000, NATURE, V406, P747, DOI 10.1038/35021093

Pinho FG, 2013, CANCER RES, V73, P5936, DOI 10.1158/0008-5472.CAN-13-0158

Ponting CP, 2009, CELL, V136, P629, DOI 10.1016/j.cell.2009.02.006

Rad E, 2016, SEMIN CELL DEV BIOL, V52, P39, DOI 10.1016/j.semcdb.2016.02.007

Razavi M, 2019, EXCLI J, V18, P422, DOI 10.17179/excli2018-1847

Rohrich M, 2016, ACTA NEUROPATHOL, V131, P877, DOI 10.1007/s00401-016-1540-6

Salmena L, 2011, CELL, V146, P353, DOI 10.1016/j.cell.2011.07.014

Shannon P, 2003, GENOME RES, V13, P2498, DOI 10.1101/gr.1239303

Shinoto M, 2011, AM J CLIN ONCOL-CANC, V34, P362, DOI 10.1097/COC.0b013e3181e84b4b

Stevens AS, 2016, CURR DRUG TARGETS, V17, P1414, DOI 10.2174/1389450116666150506112817

Subramanian A, 2005, P NATL ACAD SCI USA, V102, P15545, DOI 10.1073/pnas.0506580102

Szklarczyk D, 2019, NUCLEIC ACIDS RES, V47, pD607, DOI 10.1093/nar/gky1131

Tang H, 2019, ONCOGENE, V38, P3119, DOI 10.1038/s41388-018-0648-7

Tap WD, 2019, LANCET, V394, P478, DOI 10.1016/S0140-6736(19)30764-0

Vlachos IS, 2015, NUCLEIC ACIDS RES, V43, pW460, DOI 10.1093/nar/gkv403

Wang Q, 2019, ONCOL LETT, V18, P1199, DOI 10.3892/ol.2019.10440

Wang YK, 2017, INT J CANCER, V141, P1987, DOI 10.1002/ijc.30911

Wong NW, 2018, BIOINFORMATICS, V34, P713, DOI 10.1093/bioinformatics/btx627

Xue WH, 2018, WORLD J GASTROENTERO, V24, P23, DOI 10.3748/wjg.v24.i1.23

Yan X, 2019, FRONT GENET, V10, DOI 10.3389/fgene.2019.00408

Yan X, 2019, J CELL PHYSIOL, V234, P19073, DOI 10.1002/jcp.28546

Yang WL, 2017, EXPERT REV GASTROENT, V11, P1151, DOI 10.1080/17474124.2017.1372189

Yang WY, 2014, DRUG DEVELOP RES, V75, P331, DOI 10.1002/ddr.21216

Yoshioka M, 2017, J EXP CLIN CANC RES, V36, DOI 10.1186/s13046-017-0572-7

Zhang W, 2017, ONCOGENE, V36, P3890, DOI 10.1038/onc.2017.15

Zhao Z, 2015, DATABASE-OXFORD, DOI 10.1093/database/bav082

Zhou J, 2019, ONCOTARGETS THER, V12, P4595, DOI 10.2147/OTT.S198333

Zhu YJ, 2017, J CANCER, V8, P3657, DOI 10.7150/jca.20053

NR 57

TC 9

Z9 9

U1 2

U2 5

PU FRONTIERS MEDIA SA

PI LAUSANNE

PA AVENUE DU TRIBUNAL FEDERAL 34, LAUSANNE, CH-1015, SWITZERLAND

SN 2234-943X

J9 FRONT ONCOL

JI Front. Oncol.

PD JAN 21

PY 2020

VL 9

AR 1474

DI 10.3389/fonc.2019.01474

PG 15

WC Oncology

WE Science Citation Index Expanded (SCI-EXPANDED)

SC Oncology

GA KI2MV

UT WOS:000511184600001

PM 32038997

OA Green Published, gold

DA 2022-05-19

ER

PT J

AU Zhang, ZH

Shi, ZY

AF Zhang, Zhenhua

Shi, Zhengyuan

TI The pseudogene PTTG3P promotes cell migration and invasion in esophageal

squamous cell carcinoma

SO OPEN MEDICINE

LA English

DT Article

DE Pseudogene; PTTG3P; Esophageal squamous cell carcinoma; Regulation; Cell

metastasis

ID EXPRESSION; GROWTH; GENE; RNA

AB Pseudogenes are pivotal funtional non-coding RNAs in tumorigenesis. Cumulative evidences have shown that pituitary tumor-transforming 3, pseudogene (PTTG3P), serves as an oncogene in multiple human cancers. However, its expression pattern, biological function, and potential targets in esophageal squamous cell carcinoma (ESCC) remain unknown. Here, by quantitative real-time polymerise chain reaction (qRT-PCR) in 50 cases of ESCC, we found that the expression of PTTG3P, PTTG1 and PTTG2 in esophageal squamous cancer tissues and cell lines were significantly higher than their normal counterparts (P<0.01). Spearman correlation analysis showed that the PTTG3P expression was positively correlated with the PTTG1 and PTTG2 expression in ESCC tissue samples (P<0.05). Additionally, the high expression of PTTG3P in ESCC was significantly correlated with tumor depth, lymph node invasion and TNM stage (P<0.05). We also assessed the function of PTTG3P in vitro by gain-offunction studies. Results showed that enhanced expression of PTTG3P stimulated the migration and invasion of ESCC cells, and promoted the expression level of PTTG1 and PTTG2 in vitro. Furthermore, PTTG3P fulfilled its oncogenic functions by positively regulating its parent gene MG1 and PTTG2. Overall, our study indicated that PTTG3P is distinctly overexpressed and exhibited oncogenic role in a PTTG1 and PTTG2 mediated manner in ESCC.

C1 [Zhang, Zhenhua; Shi, Zhengyuan] Danyang Peoples Hosp, Dept Anesthesiol, Zhenjiang 212300, Jiangsu, Peoples R China.

RP Shi, ZY (corresponding author), Danyang Peoples Hosp, Dept Anesthesiol, Zhenjiang 212300, Jiangsu, Peoples R China.

EM shizhengyuan66@foxmail.com

CR Chan WL, 2013, NUCLEIC ACIDS RES, V41, P3734, DOI 10.1093/nar/gkt047

Chen LL, 2000, GENE, V248, P41, DOI 10.1016/S0378-1119(00)00096-2

Gong TT, 2017, MOL CARCINOGEN, V56, P2610, DOI 10.1002/mc.22705

Hayashi H, 2015, ONCOGENE, V34, P199, DOI 10.1038/onc.2013.547

Hu XL, 2018, CANCERS, V10, DOI 10.3390/cancers10080256

Huang JL, 2018, MOL CANCER, V17, DOI 10.1186/s12943-018-0841-x

Karreth FA, 2015, CELL, V161, P319, DOI 10.1016/j.cell.2015.02.043

Mattick JS, 2009, PLOS GENET, V5, DOI 10.1371/journal.pgen.1000459

Mattoo AR, 2014, CLIN CANCER RES, V20, P5446, DOI 10.1158/1078-0432.CCR-14-1134

Poliseno L, 2010, NATURE, V465, P1033, DOI 10.1038/nature09144

Puget N, 2002, AM J HUM GENET, V70, P858, DOI 10.1086/339434

Weng WW, 2017, J CELL MOL MED, V21, P3360, DOI 10.1111/jcmm.13239

Xu YC, 2018, J EXP CLIN CANC RES, V37, DOI 10.1186/s13046-018-0684-8

Zheng LL, 2018, NUCLEIC ACIDS RES, V46, pD85, DOI 10.1093/nar/gkx972

Zheng LF, 2015, BREAST CANCER RES TR, V150, P105, DOI 10.1007/s10549-015-3298-2

NR 15

TC 8

Z9 8

U1 0

U2 0

PU SCIENDO

PI WARSAW

PA DE GRUYTER POLAND SP Z O O, BOGUMILA ZUGA 32A STR, 01-811 WARSAW, POLAND

SN 2391-5463

J9 OPEN MED-WARSAW

JI Open Med.

PY 2019

VL 14

IS 1

BP 516

EP 522

DI 10.1515/med-2019-0057

PG 7

WC Medicine, General & Internal

WE Science Citation Index Expanded (SCI-EXPANDED)

SC General & Internal Medicine

GA IJ1OM

UT WOS:000475668000001

PM 31346548

OA Green Published, gold

DA 2022-05-19

ER

PT J

AU Okamura, A

Watanabe, M

Yamashita, K

Yuda, M

Hayami, M

Imamura, Y

Mine, S

AF Okamura, Akihiko

Watanabe, Masayuki

Yamashita, Kotaro

Yuda, Masami

Hayami, Masaru

Imamura, Yu

Mine, Shinji

TI Implication of visceral obesity in patients with esophageal squamous

cell carcinoma

SO LANGENBECKS ARCHIVES OF SURGERY

LA English

DT Article

DE Esophageal squamous cell carcinoma; Visceral obesity; Lymphatic

invasion; Lymph node metastasis; Response prediction

ID ADIPOSE-TISSUE; NEOADJUVANT CHEMORADIATION; COLORECTAL-CANCER;

COLON-CANCER; METASTASIS; RESISTANCE; ADENOCARCINOMA; CHEMOTHERAPY;

INFLAMMATION; PROGRESSION

AB Purpose Visceral obesity is considered to be associated not only with chronic systemic inflammation but also with aggressive cancer behavior. However, the implication of visceral obesity in patients with esophageal squamous cell carcinoma (ESCC) is unclear.

Methods Computed tomography volumetry was performed in 364 patients who underwent esophagectomy for ESCC. We calculated the ratio of the visceral fat area to the subcutaneous fat area (VS ratio), which is a valuable parameter of visceral obesity. Then, the clinicopathological characteristics were compared between patients with low VS ratio and those with high VS ratio.

Results Overall and disease-specific survivals of patients with high VS ratio were significantly worse than those with low VS ratio (P < 0.001 in both). Patients with high VS ratio had considerably more advanced pN factor, higher prevalence of lymphatic invasion, and more number of metastatic lymph nodes than those with low VS ratio (P = 0.044, < 0.001, and 0.006, respectively). Among patients who received preoperative treatment, high VS ratio correlated with poor response to preoperative treatment (P = 0.040).

Conclusions Visceral obesity was associated with lymphatic invasiveness and poor response to preoperative treatment in patients with ESCC, which may negatively influence their prognosis.

C1 [Okamura, Akihiko; Watanabe, Masayuki; Yamashita, Kotaro; Yuda, Masami; Hayami, Masaru; Imamura, Yu; Mine, Shinji] Japanese Fdn Canc Res, Canc Inst Hosp, Gastroenterol Ctr, Dept Gastroenterol Surg,Koto Ku, 3-8-31 Ariake, Tokyo 1358550, Japan.

RP Watanabe, M (corresponding author), Japanese Fdn Canc Res, Canc Inst Hosp, Gastroenterol Ctr, Dept Gastroenterol Surg,Koto Ku, 3-8-31 Ariake, Tokyo 1358550, Japan.

EM masayuki.watanabe@jfcr.or.jp

OI Okamura, Akihiko/0000-0002-3217-8888

FU Japan Society for the Promotion of Science (JSPS) [15K10122]

FX This work was supported in part by the Japan Society for the Promotion

of Science (JSPS) Grant-in-Aid for Scientific Research (M.W. grant

number 15K10122).

CR Cao Y, 2007, J CLIN INVEST, V117, P2362, DOI 10.1172/JCI32239

Clark W, 2013, J AM COLL SURGEONS, V216, P1070, DOI 10.1016/j.jamcollsurg.2013.01.007

Donohoe CL, 2011, DIABETOL METAB SYNDR, V3, DOI 10.1186/1758-5996-3-12

Doyle SL, 2012, P NUTR SOC, V71, P181, DOI 10.1017/S002966511100320X

Edge S., 2010, AM JOINT COMMITTEE C, VSeventh

Freese KE, 2015, CANCER RES, V75, P1161, DOI 10.1158/0008-5472.CAN-14-2744

Frezza EE, 2006, GUT, V55, P285, DOI 10.1136/gut.2005.073163

Fujiwara N, 2015, J HEPATOL, V63, P131, DOI 10.1016/j.jhep.2015.02.031

Guiu B, 2010, GUT, V59, P341, DOI 10.1136/gut.2009.188946

Hou X, 2013, ANN SURG ONCOL, V20, P1653, DOI 10.1245/s10434-012-2794-4

Incio J, 2016, CANCER DISCOV, V6, P852, DOI 10.1158/2159-8290.CD-15-1177

Kim B, 2016, NUTR CANCER, V68, P201, DOI 10.1080/01635581.2016.1134600

Kuwano H, 2015, ESOPHAGUS-TOKYO, V12, P1, DOI 10.1007/s10388-014-0465-1

Lashinger LM, 2014, CLIN PHARMACOL THER, V96, P458, DOI 10.1038/clpt.2014.136

Mariette C, 2008, ANN SURG, V247, P365, DOI 10.1097/SLA.0b013e31815aaadf

Moon HG, 2008, ANN SURG ONCOL, V15, P1918, DOI 10.1245/s10434-008-9891-4

Nieman KM, 2013, BBA-MOL CELL BIOL L, V1831, P1533, DOI 10.1016/j.bbalip.2013.02.010

Okamura A, 2016, ANN SURG ONCOL, V23, P1387, DOI 10.1245/s10434-015-5018-x

Park SW, 2015, J GASTROINTEST SURG, V19, P242, DOI 10.1007/s11605-014-2682-2

Sobin LH, 2010, TNM CLASSIFICATION M

Tran TT, 2008, CELL METAB, V7, P410, DOI 10.1016/j.cmet.2008.04.004

Trevellin E, 2015, ONCOTARGET, V6, P11203, DOI 10.18632/oncotarget.3587

van Kruijsdijk RCM, 2009, CANCER EPIDEM BIOMAR, V18, P2569, DOI 10.1158/1055-9965.EPI-09-0372

Ye S, 2016, BMC CANCER, V16, DOI 10.1186/s12885-016-2230-4

Zhang H., 2016, J CHEM, V2016, P1, DOI DOI 10.1016/J.FSIGEN.2016.01.005

2017, ESOPHAGUS PART 1, V14, P1, DOI DOI 10.1007/S10388-016-0551-7

NR 26

TC 9

Z9 9

U1 0

U2 1

PU SPRINGER

PI NEW YORK

PA 233 SPRING ST, NEW YORK, NY 10013 USA

SN 1435-2443

EI 1435-2451

J9 LANGENBECK ARCH SURG

JI Langenbecks Arch. Surg.

PD MAR

PY 2018

VL 403

IS 2

BP 245

EP 253

DI 10.1007/s00423-017-1643-0

PG 9

WC Surgery

WE Science Citation Index Expanded (SCI-EXPANDED)

SC Surgery

GA GB9EC

UT WOS:000429375900008

PM 29196841

DA 2022-05-19

ER

PT J

AU Yeo, SY

Ha, SY

Yu, EJ

Lee, KW

Kim, JH

Kim, SH

AF Yeo, So-Young

Ha, Sang Yun

Yu, Eun Ji

Lee, Keun-Woo

Kim, Jeong Hoon

Kim, Seok-Hyung

TI ZNF282 (Zinc finger protein 282), a novel E2F1 co-activator, promotes

esophageal squamous cell carcinoma

SO ONCOTARGET

LA English

DT Article

DE ZNF282; E2F1; Cell cycle; Esophageal squamous cell carcinoma; Prognosis

ID EXPRESSION; PROGNOSIS; OVEREXPRESSION; COMPLEX

AB Zinc finger protein 282 (ZNF282) is a newly identified transcription factor and little is known about its expression and function. Originally, ZNF282 is known to bind U5RE (U5 repressive element) of HLTV-1 (human T cell leukemia virus type 1) with a repressive effect. Recently we reported that ZNF282 functions as an estrogen receptor co-activator and plays an essential role in breast tumorigenesis. Although these results suggest the possible role of ZNF282 in cancers, clinical significance and function of ZNF282 are completely unknown in most of cancers. Here we found that ZNF282 was frequently overexpressed in esophageal squamous cell carcinoma (ESCC) (n=165) compared with normal esophageal epithelium and its overexpression was correlated with adverse clinical outcome. Multivariate survival analysis indicated that ZNF282 expression was an independent prognostic predictor for poor survival in ESCC (HR: 2.56 (95% CI 1.54-4.26), p<0.001). In addition, depletion of ZNF282 inhibited the cell cycle progression, migration, and invasion of ESCC cells and reduced the tumorigenicity of ESCC xenograft in nude mouse. We further showed that ZNF282 is required for E2F1-mediated gene expression in ESCC cells. Thus, ZNF282 is E2F1 co-activator involved in ESCC and elevated expression of ZNF282 is an independent adverse prognostic factor in ESCC.

C1 [Yeo, So-Young; Yu, Eun Ji; Lee, Keun-Woo; Kim, Jeong Hoon; Kim, Seok-Hyung] Sungkyunkwan Univ, Samsung Adv Inst Hlth Sci & Technol, Dept Hlth Sci & Technol, Seoul, South Korea.

[Ha, Sang Yun; Kim, Seok-Hyung] Sungkyunkwan Univ, Sch Med, Samsung Med Ctr, Dept Pathol, Seoul, South Korea.

[Kim, Jeong Hoon] Sungkyunkwan Univ, Sch Med, Samsung Med Ctr, Samsung Biomed Res Inst, Seoul, South Korea.

RP Kim, JH (corresponding author), Sungkyunkwan Univ, Samsung Adv Inst Hlth Sci & Technol, Dept Hlth Sci & Technol, Seoul, South Korea.

EM jeongkim@skku.edu; platoshkim@daum.net

FU Samsung Biomedical Research Institute [SS1B30131]; Basic Science

Research Program through the National Research Foundation of Korea -

Ministry of Education, Science and Technology [2010-0021428]; Korea

Health Technology R&D Project through the Korea Health Industry

Development Institute (KHIDI) - Ministry of Health & Welfare, Republic

of Korea [HI11C17620000]

FX This study was supported by Samsung Biomedical Research Institute grant

(SS1B30131) and by Basic Science Research Program through the National

Research Foundation of Korea funded by the Ministry of Education,

Science and Technology (2010-0021428). This research was also supported

by a grant of the Korea Health Technology R&D Project through the Korea

Health Industry Development Institute (KHIDI), funded by the Ministry of

Health & Welfare, Republic of Korea (grant number : HI11C17620000).

CR Bird-Lieberman EL, 2009, BRIT J CANCER, V101, P1, DOI 10.1038/sj.bjc.6605126

Biswas AK, 2012, CANCER RES, V72, P13, DOI 10.1158/0008-5472.CAN-11-2196

Ebihara Y, 2004, DIS ESOPHAGUS, V17, P150, DOI 10.1111/j.1442-2050.2004.00393.x

Engelmann D, 2012, CANCER RES, V72, P571, DOI 10.1158/0008-5472.CAN-11-2575

Fred T., 2009, WHO CLASSIFICATION T

He LR, 2009, CANCER SCI, V100, P1591, DOI 10.1111/j.1349-7006.2009.01224.x

Hongo M, 2009, J GASTROEN HEPATOL, V24, P729, DOI 10.1111/j.1440-1746.2009.05824.x

Hu N, 2010, BMC GENOMICS, V11, DOI 10.1186/1471-2164-11-576

Huang JX, 2012, ONCOL LETT, V4, P607, DOI 10.3892/ol.2012.814

Kim JH, 2008, MOL CELL, V31, P510, DOI 10.1016/j.molcel.2008.08.001

Kim SH, 2012, HISTOL HISTOPATHOL, V27, P49, DOI 10.14670/HH-27.49

Klein CA, 2009, CANCER RES, V69, P5285, DOI 10.1158/0008-5472.CAN-08-4586

Lao-Sirieix P, 2012, NAT REV CLIN ONCOL, V9, P278, DOI 10.1038/nrclinonc.2012.35

Lee KW, 2012, ANN SURG ONCOL, V19, P326, DOI 10.1245/s10434-011-1867-0

Lee TI, 2013, CELL, V152, P1237, DOI 10.1016/j.cell.2013.02.014

Li Y, 2011, ANN THORAC SURG, V91, P1531, DOI 10.1016/j.athoracsur.2010.12.012

Lin DC, 2009, DIS ESOPHAGUS, V22, P9, DOI 10.1111/j.1442-2050.2008.00845.x

Lv J, 2012, MED ONCOL, V29, P151, DOI 10.1007/s12032-010-9816-5

Mega S, 2005, DIS ESOPHAGUS, V18, P109, DOI 10.1111/j.1442-2050.2005.00463.x

Munro S, 2012, ONCOGENE, V31, P4343, DOI 10.1038/onc.2011.603

Okumura K, 1997, NUCLEIC ACIDS RES, V25, P5025, DOI 10.1093/nar/25.24.5025

Revenko AS, 2010, MOL CELL BIOL, V30, P5260, DOI 10.1128/MCB.00484-10

Sherr CJ, 1996, SCIENCE, V274, P1672, DOI 10.1126/science.274.5293.1672

SINICROPE FA, 1995, CANCER RES, V55, P237

Wong JV, 2011, CELL CYCLE, V10

Xiang J, 2012, EUR REV MED PHARMACO, V16, P2051

Xu FP, 2007, CANCER LETT, V245, P69, DOI 10.1016/j.canlet.2005.12.030

Yamazaki K, 2005, J CLIN PATHOL, V58, P904, DOI 10.1136/jcp.2004.023127

Yang CK, 2006, J BIOL CHEM, V281, P3389, DOI 10.1074/jbc.M510403200

Yu EJ, 2013, ONCOGENE, V32, P4160, DOI 10.1038/onc.2012.420

NR 30

TC 6

Z9 7

U1 0

U2 5

PU IMPACT JOURNALS LLC

PI ORCHARD PARK

PA 6666 E QUAKER ST, STE 1, ORCHARD PARK, NY 14127 USA

EI 1949-2553

J9 ONCOTARGET

JI Oncotarget

PD DEC 15

PY 2014

VL 5

IS 23

BP 12260

EP 12272

DI 10.18632/oncotarget.2630

PG 13

WC Oncology; Cell Biology

WE Science Citation Index Expanded (SCI-EXPANDED)

SC Oncology; Cell Biology

GA AZ2AT

UT WOS:000348037700037

PM 25373738

OA Green Published, Green Submitted, gold

DA 2022-05-19

ER

PT J

AU Lu, FY

Chen, WC

Jiang, TW

Cheng, CE

Wang, B

Lu, ZP

Huang, GJ

Qiu, JM

Wei, W

Yang, M

Huang, X

AF Lu, Fenying

Chen, Weichang

Jiang, Tingwang

Cheng, Cuie

Wang, Bin

Lu, Zhiping

Huang, Guojin

Qiu, Jiaming

Wei, Wei

Yang, Ming

Huang, Xia

TI Expression profile, clinical significance and biological functions of

IGF2BP2 in esophageal squamous cell carcinoma

SO EXPERIMENTAL AND THERAPEUTIC MEDICINE

LA English

DT Article

DE downregulation; esophageal-squamous cell carcinoma; insulin-like growth

factor 2 mRNA-binding protein 2; proliferation; migration

ID MESSENGER-RNA; CANCER; SURVIVAL; PROGRESSION; GENE; P62

AB The ectopic expression of insulin-like growth factor 2 mRNA-binding protein 2 (IGF2BP2) has been demonstrated to facilitate tumorigenesis and induce proliferation in a various types of cancer. However, the role of IGF2BP2 in esophageal squamous cell carcinoma (ESCC) has yet been fully elucidated. In this regard, the current study assessed the expression patterns and clinical significance of IGF2BP2 in 94 Chinese patients diagnosed with ESCC. Immunohistochemistry and reverse transcription-quantitative PCR assays were employed to assess IGF2BP2 expression in ESCC tissues compared with adjacent healthy tissues. The results revealed that the protein expression of IGF2BP2 was substantially upregulated in ESCC tissues compared with adjacent ESCC tissues. More specifically, higher IGF2BP2 expression strongly associated with tumor node metastasis stage, lymphatic infiltration and lymph node metastasis. Using two ESCC cell lines (TE-1 and TE-10), the inhibition of IGF2BP2 expression by small interfering RNA was proven to induce apoptosis and suppress proliferation, migration and cell cycle progression in vitro. Collectively, the present findings indicated that IGF2BP2 may serve a major role in the development of ESCC carcinogenesis. The present study may be helpful in the design of potential drug targets in the treatment of ESCC.

C1 [Lu, Fenying; Chen, Weichang] Soochow Univ, Dept Gastroenterol, Affiliated Hosp 1, 188 Shizi St, Suzhou 215006, Jiangsu, Peoples R China.

[Lu, Fenying; Cheng, Cuie; Wang, Bin; Lu, Zhiping; Huang, Guojin; Huang, Xia] Second Peoples Hosp Changshu, Dept Gastroenterol, Suzhou 215500, Jiangsu, Peoples R China.

[Jiang, Tingwang] Second Peoples Hosp Changshu, Sci & Technol Div, Suzhou 215500, Jiangsu, Peoples R China.

[Qiu, Jiaming; Wei, Wei] Second Peoples Hosp Changshu, Dept Pathol, Suzhou 215500, Jiangsu, Peoples R China.

[Yang, Ming] Second Peoples Hosp Changshu, Dept Thorac Surg, Suzhou 215500, Jiangsu, Peoples R China.

RP Chen, WC (corresponding author), Soochow Univ, Dept Gastroenterol, Affiliated Hosp 1, 188 Shizi St, Suzhou 215006, Jiangsu, Peoples R China.

EM weichangchen@126.com

FU Development Plant Projects of the Science and Technology at Suzhou

[SYS2019008, CS201915]

FX The present work was supported by the Development Plant Projects of the

Science and Technology at Suzhou (grant no. SYS2019008) and Changshu

(grant no. CS201915).

CR Albores-Saavedra J., 2010, WHO CLASSIFICATION T, ppp263

Alsop BR, 2016, GASTROENTEROL CLIN N, V45, P399, DOI 10.1016/j.gtc.2016.04.001

Barghash A, 2016, ONCOTARGET, V7, P49743, DOI 10.18632/oncotarget.10439

Bell JL, 2013, CELL MOL LIFE SCI, V70, P2657, DOI 10.1007/s00018-012-1186-z

Boudoukha S, 2010, MOL CELL BIOL, V30, P5710, DOI 10.1128/MCB.00665-10

Cao JG, 2018, STEM CELLS INT, V2018, DOI 10.1155/2018/4217259

Cleynen I, 2007, MOL CANCER RES, V5, P363, DOI 10.1158/1541-7786.MCR-06-0331

Dahlem C, 2019, INT J MOL SCI, V20, DOI 10.3390/ijms20133204

Dai N, 2017, ELIFE, V6, DOI [10.7554/eLife.27155.001, 10.7554/eLife.27155]

Dai N, 2015, CELL METAB, V21, P609, DOI 10.1016/j.cmet.2015.03.006

DETRE S, 1995, J CLIN PATHOL, V48, P876, DOI 10.1136/jcp.48.9.876

Gu TW, 2012, J DIABETES COMPLICAT, V26, P393, DOI 10.1016/j.jdiacomp.2012.05.012

He X, 2018, CELL PHYSIOL BIOCHEM, V51, P1945, DOI 10.1159/000495719

Hou PF, 2021, J EXP CLIN CANC RES, V40, DOI 10.1186/s13046-021-01857-2

Janiszewska M, 2012, GENE DEV, V26, P1926, DOI 10.1101/gad.188292.112

Li T., 2016, GUT, V68, pA1, DOI [10.1186/s12943-019-1038-7, DOI 10.1186/S12943-019-1038-7]

Li T, 2019, MOL CANCER, V18, DOI 10.1186/s12943-019-1038-7

Liu XX, 2014, J IMMUNOL RES, V2014, DOI 10.1155/2014/326593

Livak KJ, 2001, METHODS, V25, P402, DOI 10.1006/meth.2001.1262

Lu ML, 2001, AM J PATHOL, V159, P945, DOI 10.1016/S0002-9440(10)61770-1

McMullen ER, 2018, BREAST CANCER RES TR, V172, P577, DOI 10.1007/s10549-018-4960-2

Mu QC, 2015, CANCER BIOL THER, V16, P623, DOI 10.1080/15384047.2015.1019185

Nakajima M, 2013, EXPERT OPIN PHARMACO, V14, P1345, DOI 10.1517/14656566.2013.801454

Napier KJ, 2014, WORLD J GASTRO ONCOL, V6, P112, DOI 10.4251/wjgo.v6.i5.112

Pu J, 2020, FRONT ONCOL, V10, DOI 10.3389/fonc.2020.578816

Puschhof J, 2021, NAT PROTOC, V16, P1494, DOI 10.1038/s41596-020-00463-4

Quaas A, 2021, FRONT ONCOL, V11, DOI 10.3389/fonc.2021.569475

Short MW, 2017, AM FAM PHYSICIAN, V95, P22

Simon Y, 2014, GUT, V63, P861, DOI 10.1136/gutjnl-2013-305736

Sobin LH, 2010, CANCER-AM CANCER SOC, V116, P5336, DOI 10.1002/cncr.25537

Sung HH, 2022, CLIN EXP HYPERTENS, V44, P146, DOI 10.1080/10641963.2021.2007943

Tustumi F, 2016, ABCD-ARQ BRAS CIR DI, V29, P138, DOI 10.1590/0102-6720201600030003

Wu XD, 2021, FRONT ONCOL, V11, DOI 10.3389/fonc.2021.680642

Xu XD, 2019, J EXP CLIN CANC RES, V38, DOI 10.1186/s13046-019-1470-y

Yang HP, 2016, MOL MED REP, V13, P4143, DOI 10.3892/mmr.2016.5046

Ye S, 2016, FEBS LETT, V590, P1641, DOI 10.1002/1873-3468.12205

Zhang LP, 2011, AM J SURG PATHOL, V35, P868, DOI 10.1097/PAS.0b013e318219c6f9

Zhou SL, 2014, DIS ESOPHAGUS, V27, P790, DOI 10.1111/dote.12145

Zhu J, 2021, SCI REP-UK, V11, DOI 10.1038/s41598-021-96548-5

NR 39

TC 0

Z9 0

U1 2

U2 2

PU SPANDIDOS PUBL LTD

PI ATHENS

PA POB 18179, ATHENS, 116 10, GREECE

SN 1792-0981

EI 1792-1015

J9 EXP THER MED

JI Exp. Ther. Med.

PD APR

PY 2022

VL 23

IS 4

AR 252

DI 10.3892/etm.2022.11177

PG 11

WC Medicine, Research & Experimental

WE Science Citation Index Expanded (SCI-EXPANDED)

SC Research & Experimental Medicine

GA ZF6TR

UT WOS:000759698500001

PM 35261624

OA Green Published, gold

DA 2022-05-19

ER

PT J

AU Chai, TC

Shen, ZM

Zhang, ZY

Chen, S

Gao, L

Zhang, PP

Lin, WW

Kang, MQ

Lin, JB

AF Chai, Tianci

Shen, Zhimin

Zhang, Zhenyang

Chen, Sui

Gao, Lei

Zhang, Peipei

Lin, Wenwei

Kang, Mingqiang

Lin, Jiangbo

TI LGR6 is a potential diagnostic and prognostic marker for esophageal

squamous cell carcinoma

SO JOURNAL OF CLINICAL LABORATORY ANALYSIS

LA English

DT Article

DE esophageal squamous cell carcinoma; immunohistochemistry; LGR6;

prognosis

ID DRUG DISCOVERY; DYSREGULATION; RECEPTORS; TARGETS

AB Background Leucine-rich repeat-coupled receptor 6 (LGR6) is a marker of the skin, nails, and other types of adult tissue stem cells and has been widely found to be related to the development and progression of a variety of cancer types. The clinical significance and biological function of LGR6 in esophageal squamous cell carcinoma (ESCC) have not been determined. Methods The expression of LGR6 at the transcriptional level was analyzed by searching the TCGA and UCSC data sets. Immunohistochemistry, WB, and q-PCR were used to detect the expression of LGR6 in ESCC and adjacent normal tissues. LGR6 PPI networks and KEGG pathways were used to analyze the potential biological functions of LGR6. Results The expression of LGR6 in ESCC tissues was significantly higher than that in normal tissues and was negatively correlated with the differentiation degree of ESCC and the prognosis of the patients but not closely correlated with the TNM stage of ESCC. PPI networks showed that LGR6 had a close interaction with RSPO1, RSPO2, RSPO3, and RSPO4. KEGG pathway analysis showed that LGR6 activated the Wnt/beta-catenin signaling pathway by binding with RSPO ligands to promote the progression of ESCC. Conclusion LGR6 can serve as a potential diagnostic and prognostic marker for ESCC.

C1 [Chai, Tianci; Shen, Zhimin; Zhang, Zhenyang; Chen, Sui; Gao, Lei; Zhang, Peipei; Lin, Wenwei; Kang, Mingqiang; Lin, Jiangbo] Fujian Med Univ, Union Hosp, Dept Thorac Surg, 29 New Power Rd, Fuzhou 350004, Peoples R China.

[Chai, Tianci] Xinyi Peoples Hosp, Dept Anesthesiol, Xuzhou, Jiangsu, Peoples R China.

[Kang, Mingqiang] Fujian Med Univ, Minist Educ Gastrointestinal Canc, Key Lab, Fuzhou, Peoples R China.

RP Kang, MQ; Lin, JB (corresponding author), Fujian Med Univ, Union Hosp, Dept Thorac Surg, 29 New Power Rd, Fuzhou 350004, Peoples R China.

EM mingqiang_kang@126.cm; 135279779@qq.com

OI Shen, Zhimin/0000-0001-9294-4532

FU Joint Funds for the innovation of science and Technology,Fujian province

[2017Y9039]; Joint Funds for the innovation of science and Technology,

Fujian province [2018Y9034]; Natural Science Foundation of Fujian

Province [2016J01557]

FX Joint Funds for the innovation of science and Technology,Fujian

province, Grant/Award Number: 2017Y9039; Joint Funds for the innovation

of science and Technology, Fujian province, Grant/Award Number:

2018Y9034; Natural Science Foundation of Fujian Province, Grant/Award

Number: 2016J01557

CR Abnet CC, 2018, GASTROENTEROLOGY, V154, P360, DOI 10.1053/j.gastro.2017.08.023

Blaas L, 2016, NAT CELL BIOL, V18, P1346, DOI 10.1038/ncb3434

Bray F, 2018, CA-CANCER J CLIN, V68, P394, DOI 10.3322/caac.21492

de Lau W, 2011, NATURE, V476, P293, DOI 10.1038/nature10337

Dzobo K, 2019, OMICS, V23, P517, DOI 10.1089/omi.2019.0147

Garcia-Closas M, 2013, NAT GENET, V45, P392, DOI 10.1038/ng.2561

Guinot A, 2016, CANCER RES, V76, P4012, DOI 10.1158/0008-5472.CAN-15-3302

Haakonsen DL, 2017, J CLIN INVEST, V127, P4228, DOI 10.1172/JCI98262

Hao HX, 2016, CANCERS, V8, DOI 10.3390/cancers8060054

Hauser AS, 2017, NAT REV DRUG DISCOV, V16, P829, DOI 10.1038/nrd.2017.178

Jansson L, 2019, P NATL ACAD SCI USA, V116, P21054, DOI 10.1073/pnas.1910223116

Lappano R, 2011, NAT REV DRUG DISCOV, V10, P47, DOI 10.1038/nrd3320

Lehoczky JA, 2015, P NATL ACAD SCI USA, V112, P13249, DOI 10.1073/pnas.1518874112

Paola C, 2019, EXPERT REV MOL DIAGN, V19, P795

Park S, 2018, J BIOL CHEM, V293, P9759, DOI 10.1074/jbc.RA118.002743

Raslan AA, 2019, INT J BIOCHEM CELL B, V106, P26, DOI 10.1016/j.biocel.2018.11.005

Ruan XH, 2019, MOL THER-ONCOLYTICS, V14, P94, DOI 10.1016/j.omto.2019.04.002

Schindler AJ, 2018, ONCOTARGET, V9, P1346, DOI 10.18632/oncotarget.20178

Steffen JS, 2012, VIRCHOWS ARCH, V461, P355, DOI 10.1007/s00428-012-1292-1

Tan BH, 2018, CANCER RES, V78, P4929, DOI 10.1158/0008-5472.CAN-18-0152

Thrumurthy SG, 2019, BMJ-BRIT MED J, V366, DOI 10.1136/bmj.l4373

van de Glind GC, 2016, ONCOTARGET, V7, P86740, DOI 10.18632/oncotarget.13436

Wang WJ, 2019, FRONT MED-PRC, V13, P482, DOI 10.1007/s11684-018-0633-0

Xie Y, 2019, J PATHOL, V247, P228, DOI 10.1002/path.5186

Xu JG, 2015, J BIOL CHEM, V290, P2455, DOI 10.1074/jbc.M114.599134

Xu P, 2016, CANCER LETT, V383, P161, DOI 10.1016/j.canlet.2016.09.005

NR 26

TC 4

Z9 4

U1 0

U2 3

PU WILEY

PI HOBOKEN

PA 111 RIVER ST, HOBOKEN, NJ 07030 USA

SN 0887-8013

EI 1098-2825

J9 J CLIN LAB ANAL

JI J. Clin. Lab. Anal.

PD APR

PY 2020

VL 34

IS 4

AR e23121

DI 10.1002/jcla.23121

EA JAN 2020

PG 9

WC Medical Laboratory Technology

WE Science Citation Index Expanded (SCI-EXPANDED)

SC Medical Laboratory Technology

GA LI1KJ

UT WOS:000506264900001

PM 31917882

OA gold, Green Published

DA 2022-05-19

ER

PT J

AU Wu, SB

Wang, HQ

AF Wu, S-B

Wang, H-Q

TI RETRACTED: Upregulation of long noncoding RNA DLX6-AS1 promotes cell

growth and metastasis in esophageal squamous cell carcinoma via

targeting miR-577 (Retracted Article)

SO EUROPEAN REVIEW FOR MEDICAL AND PHARMACOLOGICAL SCIENCES

LA English

DT Article; Retracted Publication

DE Long noncoding RNA; DLX6-AS1; Esophageal squamous cell carcinoma;

MiR-577

ID CANCER STATISTICS; LNCRNA; PROLIFERATION; EXPRESSION; MIGRATION;

INVASION; CHINA

AB OBJECTIVE: Esophageal squamous cell carcinoma (ESCC) is the most malignant type of esophageal cancer. Although significant advances have been made in ESCC diagnosis and therapy, its poor pathogenesis and prognosis remain a life-threatening problem. Meanwhile, long noncoding RNAs (lncRNAs) exert a pivotal function in tumorigenesis. In this research, we aimed to explore the association between the aberrant expression of lncRNA DLX6-AS1 and the development and metastasis of ESCC.

PATIENTS AND METHODS: DLX6-AS1 expression was monitored by quantitative Real Time-Polymerase Chain Reaction (qRT-PCR) in ESCC specimens. Moreover, experiments were conducted to detect the effect of DLX6-AS1 on the cell proliferation and metastasis of ESCC. In addition, the underlying mechanism was further explored through luciferase assays and RNA immunoprecipitation assay (RIP).

RESULTS: DLX6-AS1 expression level was significantly higher in ESCC specimens. Moreover, cell proliferation and metastasis of ESCC cells could be inhibited via reducing DLX6-AS1 expression. Besides, DLX6-AS1 was regarded as an oncogene in ESCC. Furthermore, DLX6-AS1 acted as a competing endogenous RNA via sponging miR-577 in ESCC.

CONCLUSIONS: In summary, DLX6-AS1 promotes development and metastasis of ESCC by sponging miR-577 and could be a potential therapeutic target.

C1 [Wu, S-B; Wang, H-Q] Dalian Med Univ, Hosp 2, Dept Gastroenterol, Dalian, Peoples R China.

RP Wang, HQ (corresponding author), Dalian Med Univ, Hosp 2, Dept Gastroenterol, Dalian, Peoples R China.

EM whq96177@126.com

CR Alaei S, 2019, GENOMICS, V111, P76, DOI 10.1016/j.ygeno.2018.01.003

An Y, 2018, CANCER CELL INT, V18, DOI 10.1186/s12935-018-0643-7

Arnold M, 2015, GUT, V64, P381, DOI 10.1136/gutjnl-2014-308124

Chen WQ, 2014, THORAC CANCER, V5, P343, DOI 10.1111/1759-7714.12100

Deng YQ, 2018, CANCER BIOTHER RADIO, V33, P365, DOI 10.1089/cbr.2018.2503

Devesa SS, 1998, CANCER, V83, P2049, DOI 10.1002/(SICI)1097-0142(19981115)83:10<2049::AID-CNCR1>3.0.CO;2-2

Huang GL, 2016, SCI REP-UK, V6, DOI 10.1038/srep26524

Huang YJ, 2019, BIOMED PHARMACOTHER, V109, P1851, DOI 10.1016/j.biopha.2018.09.151

Sadeghpour S, 2019, MOL BIOL REP, V46, P991, DOI 10.1007/s11033-018-4556-2

Shi JX, 2019, CELL BIOCHEM FUNCT, V37, P11, DOI 10.1002/cbf.3368

Siegel RL, 2017, CA-CANCER J CLIN, V67, P177, DOI 10.3322/caac.21395

Song P, 2016, GENE, V592, P8, DOI 10.1016/j.gene.2016.07.055

Thin KZ, 2019, CLIN CHIM ACTA, V494, P38, DOI 10.1016/j.cca.2019.03.002

Vennin C, 2017, CANCER LETT, V385, P198, DOI 10.1016/j.canlet.2016.10.023

Xiong YY, 2017, CELL PHYSIOL BIOCHEM, V43, P405, DOI 10.1159/000480419

Xu J, 2017, CELL PHYSIOL BIOCHEM, V41, P635, DOI 10.1159/000457938

Xu XL, 2019, CELL DEATH DIS, V10, DOI 10.1038/s41419-019-1348-0

Yin CG, 2018, THORAC CANCER, V9, P472, DOI 10.1111/1759-7714.12612

Yuan X, 2013, INT J CLIN EXP PATHO, V6, P2651

Zeng HM, 2016, THORAC CANCER, V7, P232, DOI 10.1111/1759-7714.12322

Zeng X, 2017, CELL CYCLE, V16, P2212, DOI 10.1080/15384101.2017.1361072

Zhang RM, 2018, BIOCHEM BIOPH RES CO, V507, P260, DOI 10.1016/j.bbrc.2018.11.019

Zhang WG, 2016, MOL CARCINOGEN, V55, P575, DOI 10.1002/mc.22304

NR 23

TC 6

Z9 6

U1 2

U2 5

PU VERDUCI PUBLISHER

PI ROME

PA VIA GREGORIO VII, ROME, 186-00165, ITALY

SN 1128-3602

J9 EUR REV MED PHARMACO

JI Eur. Rev. Med. Pharmacol. Sci.

PY 2020

VL 24

IS 3

BP 1195

EP 1201

DI 10.26355/eurrev_202002_20171

PG 7

WC Pharmacology & Pharmacy

WE Science Citation Index Expanded (SCI-EXPANDED)

SC Pharmacology & Pharmacy

GA KP9TQ

UT WOS:000516575000023

PM 32096208

DA 2022-05-19

ER

PT J

AU Zhao, ZH

Wang, WW

Niu, LH

Zhang, JY

Jiang, GZ

AF Zhao Zhihua

Wang Weiwei

Niu Lihua

Zhang Jianying

Guozhong Jiang

TI p53-induced long non-coding RNA PGM5-AS1 inhibits the progression of

esophageal squamous cell carcinoma through regulating miR-466/PTEN axis

SO IUBMB LIFE

LA English

DT Article

DE biomarker; esophageal squamous cell carcinoma; PGM5-AS1; progression

ID CANCER; EXPRESSION

AB A growing body of evidence suggests that long non-coding RNA (lncRNA) is aberrantly expressed in human cancer and linked to cancer initiation and development. We previously identified Homo sapiens PGM5 antisense RNA 1 (PGM5-AS1) as a novel esophageal squamous cell carcinoma (ESCC)-related lncRNA by performing high-throughput RNA sequencing. However, its clinical implication and biological function in ESCC are still uncharacterized. In the present study, we found that PGM5-AS1 was frequently downregulated in ESCC tissues, plasma, and cell lines, and low PGM5-AS1 expression was positively correlated with poor differentiation, advanced tumor node metastasis (TNM) stage, and lymph node metastasis. Importantly, PGM5-AS1 was identified to be an effective diagnostic and prognostic biomarker for ESCC patients. Functional experiments revealed that exogenous expression of PGM5-AS1 significantly suppressed the proliferation, migration, and invasion of ESCC cells in vitro as well as tumor growth in vivo. Mechanistically, PGM5-AS1 was transcriptionally activated by p53 and it could directly interact with and sequester miR-466 to elevate PTEN expression, thereby inhibiting ESCC progression. Overall, our data indicate that PGM5-AS1 is a novel tumor suppressor in ESCC and restoration of PGM5-AS1 may be a promising avenue for treatment of ESCC patient.

C1 [Zhao Zhihua; Wang Weiwei; Niu Lihua; Zhang Jianying; Guozhong Jiang] Zhengzhou Univ, Affiliated Hosp 1, Dept Pathol, 1 Jianshe Dong Rd, Zhengzhou 450000, Henan, Peoples R China.

RP Jiang, GZ (corresponding author), Zhengzhou Univ, Affiliated Hosp 1, Dept Pathol, 1 Jianshe Dong Rd, Zhengzhou 450000, Henan, Peoples R China.

EM doc_jiang668@yeah.net

CR Abnet CC, 2018, GASTROENTEROLOGY, V154, P360, DOI 10.1053/j.gastro.2017.08.023

Bhan A, 2017, CANCER RES, V77, P3965, DOI 10.1158/0008-5472.CAN-16-2634

Boeke JD, 2016, SCIENCE, V353, P126, DOI 10.1126/science.aaf6850

Cao W, 2018, EXP THER MED, V16, P5117, DOI 10.3892/etm.2018.6888

Chan JJ, 2018, INT J MOL SCI, V19, DOI 10.3390/ijms19051310

Colden M, 2017, CELL DEATH DIS, V8, DOI 10.1038/cddis.2017.15

Davidovich C, 2013, NAT STRUCT MOL BIOL, V20, P1250, DOI 10.1038/nsmb.2679

Grossi E, 2016, BBA-GENE REGUL MECH, V1859, P200, DOI 10.1016/j.bbagrm.2015.07.011

Kopp F, 2018, CELL, V172, P393, DOI 10.1016/j.cell.2018.01.011

Lin C, 2017, ONCOGENE, V36, P5392, DOI 10.1038/onc.2017.133

Lin CR, 2018, TRENDS CELL BIOL, V28, P287, DOI 10.1016/j.tcb.2017.11.008

Liu T, 2019, IUBMB LIFE, V71, P254, DOI 10.1002/iub.1971

Lu W, 2017, ONCOTARGET, V8, P32384, DOI 10.18632/oncotarget.15660

Mazor G, 2019, CELL DEATH DIS, V10, DOI 10.1038/s41419-019-1477-5

Mello SS, 2018, CURR OPIN CELL BIOL, V51, P65, DOI 10.1016/j.ceb.2017.11.005

Murphy G, 2017, ANN ONCOL, V28, P2086, DOI 10.1093/annonc/mdx279

Qin XC, 2019, GENE, V690, P120, DOI 10.1016/j.gene.2018.12.047

Renganathan A, 2017, ADV EXP MED BIOL, V1008, P199, DOI 10.1007/978-981-10-5203-3_7

Serghiou S, 2016, MOL CANCER, V15, DOI 10.1186/s12943-016-0535-1

Smyth EC, 2017, NAT REV DIS PRIMERS, V3, DOI 10.1038/nrdp.2017.48

Sun P, 2017, INT J GYNECOL CANCER, V27, P339, DOI 10.1097/IGC.0000000000000863

Tai Y., 2019, IUBMB LIFE

Tan DSW, 2017, NAT MED, V23, P1167, DOI 10.1038/nm.4401

Tan JW, 2019, IUBMB LIFE, V71, P882, DOI 10.1002/iub.2012

Tong F, 2018, BOSNIAN J BASIC MED, V18, P252, DOI 10.17305/bjbms.2018.2376

Tsai MC, 2010, SCIENCE, V329, P689, DOI 10.1126/science.1192002

Wang C, 2019, ONCOGENE, V38, P1611, DOI 10.1038/s41388-018-0548-x

Wang M, 2019, CELL DEATH DIFFER, V26, P2329, DOI 10.1038/s41418-019-0304-y

Wang WW, 2018, GENE, V661, P169, DOI 10.1016/j.gene.2018.03.066

Xie JJ, 2018, GASTROENTEROLOGY, V154, P2137, DOI 10.1053/j.gastro.2018.02.018

Xing YH, 2016, IUBMB LIFE, V68, P887, DOI 10.1002/iub.1564

Xu M, 2019, J HEMATOL ONCOL, V12, DOI 10.1186/s13045-018-0690-5

Xu TP, 2018, ONCOGENE, V37, P5020, DOI 10.1038/s41388-018-0308-y

Yehia L, 2019, J CLIN INVEST, V129, P452, DOI 10.1172/JCI121277

Yu SJ, 2019, AGING-US, V11, P1389, DOI 10.18632/aging.101839

Zhang JW, 2018, HEPATOLOGY, V67, P171, DOI 10.1002/hep.29405

Zhuo W, 2019, GASTROENTEROLOGY, V156, P676, DOI 10.1053/j.gastro.2018.10.054

NR 37

TC 16

Z9 17

U1 1

U2 9

PU WILEY

PI HOBOKEN

PA 111 RIVER ST, HOBOKEN 07030-5774, NJ USA

SN 1521-6543

EI 1521-6551

J9 IUBMB LIFE

JI IUBMB Life

PD OCT

PY 2019

VL 71

IS 10

BP 1492

EP 1502

DI 10.1002/iub.2069

PG 11

WC Biochemistry & Molecular Biology; Cell Biology

WE Science Citation Index Expanded (SCI-EXPANDED)

SC Biochemistry & Molecular Biology; Cell Biology

GA IY2DD

UT WOS:000486199700008

PM 31185143

OA Bronze

DA 2022-05-19

ER

PT J

AU Song, Q

Wu, JZ

Wang, S

AF Song, Qian

Wu, Jun-Zhou

Wang, Sheng

TI Perioperative change in lymphocyte count and prognosis in esophageal

squamous cell carcinoma

SO JOURNAL OF THORACIC DISEASE

LA English

DT Article

DE Postoperative lymphocyte count change; esophageal squamous cell

carcinoma (ESCC); prognosis; disease-free survival (DFS)

ID COLORECTAL-CANCER; TUMOR-DEVELOPMENT; SURVIVAL; INFILTRATION;

LYMPHOPENIA

AB Background: Preoperative lymphocyte count (pre-LC) predicts the relapse and survival in patients with esophageal squamous cell carcinoma (ESCC), the clinical application of postoperative lymphocyte count (post-LC) change (LCc) remains unclear.

Methods: A retrospective analysis of patients with newly diagnosed ESCC who received curative resection from 2008 to 2015 was conducted. Complete blood counts from preoperative within seven days to postoperative within seven days were analyzed. LCc was defined as LCc increased (post-LC higher than pre-LC) and LCc decreased (post-LC lower than pre-LC). LCc was evaluated for an association with disease-free survival (DFS).

Results: A total of 677 patients who reach the standard were enrolled into the study. There were 579 (85.5%) male and 98 (14.5%) female patients with ESCC. The median age was 61 years (range, 39-84 years). In univariate analysis, LCc significantly correlated to DFS (P=0.006, HR =0.593, 95% CI: 0.409-0.862). In multivariate analysis (COX model), LCc was an independent prognostic factor for DES (P=0.011, RR =0.617, 95% Cl: 0.424-0.897).

Conclusions: The dynamic change of LC after surgery may serve as a simple and new prognostic factor in patients with newly diagnosed ESCC.

C1 [Song, Qian] Zhejiang Canc Hosp, Dept Clin Lab, Key Lab Head & Neck Canc Translat Res Zhejiang Pr, Hangzhou 310022, Zhejiang, Peoples R China.

[Wu, Jun-Zhou] Zhejiang Canc Hosp, Canc Res Inst, Hangzhou 310022, Zhejiang, Peoples R China.

[Wu, Jun-Zhou] Key Lab Diag & Treatment Technol Thorac Oncol Zhe, Hangzhou 310022, Zhejiang, Peoples R China.

[Wang, Sheng] Zhejiang Canc Hosp, Dept Clin Lab, 1 Banshan East Rd, Hangzhou 310022, Zhejiang, Peoples R China.

RP Wang, S (corresponding author), Zhejiang Canc Hosp, Dept Clin Lab, 1 Banshan East Rd, Hangzhou 310022, Zhejiang, Peoples R China.

EM wangsheng@zjcc.org.cn

FU National Natural Science Foundation of China [81502603]; General

research program of Health Department of Zhejiang Province [2016KYB048];

Zhejiang Provincial Medical Youth Talent [2019RC026]

FX This study was funded by National Natural Science Foundation of China

(contract/grant number: 81502603), General research program of Health

Department of Zhejiang Province (contract/grant number: 2016KYB048) and

Zhejiang Provincial Medical Youth Talent (2019RC026).

CR Arnold M, 2017, AM J GASTROENTEROL, V112, P1247, DOI 10.1038/ajg.2017.155

Bastid J, 2014, ONCOIMMUNOLOGY, V3, DOI 10.4161/onci.28273

Bray F, 2018, CA-CANCER J CLIN, V68, P394, DOI 10.3322/caac.21492

Dahlin AM, 2011, MODERN PATHOL, V24, P671, DOI 10.1038/modpathol.2010.234

Feng F, 2018, BMC GASTROENTEROL, V18, DOI 10.1186/s12876-018-0877-9

Feng JF, 2017, MEDICINE, V96, DOI 10.1097/MD.0000000000005886

Feng JF, 2014, MEDICINE, V93, DOI 10.1097/MD.0000000000000257

Gertler R, 2011, ANN SURG, V253, P689, DOI 10.1097/SLA.0b013e31821111b5

Hiraoka K, 2006, BRIT J CANCER, V94, P275, DOI 10.1038/sj.bjc.6602934

Huang SH, 2015, CANCER-AM CANCER SOC, V121, P545, DOI 10.1002/cncr.29100

Lohr J, 2011, CLIN CANCER RES, V17, P4296, DOI 10.1158/1078-0432.CCR-10-2557

Mahmoud SMA, 2011, J CLIN ONCOL, V29, P1949, DOI 10.1200/JCO.2010.30.5037

Martinez-Lostao L, 2015, CLIN CANCER RES, V21, P5047, DOI 10.1158/1078-0432.CCR-15-0685

Mehrazin R, 2015, UROL ONCOL, V33, P388

Nazir Taha, 2015, Breast Dis, V35, P1, DOI 10.3233/BD-140386

OWNBY HE, 1983, CANCER, V52, P126, DOI 10.1002/1097-0142(19830701)52:1<126::AID-CNCR2820520123>3.0.CO;2-Y

Ray-Coquard I, 2009, CANCER RES, V69, P5383, DOI 10.1158/0008-5472.CAN-08-3845

Shankaran V, 2001, NATURE, V410, P1107, DOI 10.1038/35074122

Smyth MJ, 2006, ADV IMMUNOL, V90, P1, DOI 10.1016/S0065-2776(06)90001-7

Tran GD, 2005, INT J CANCER, V113, P456, DOI 10.1002/ijc.20616

Wang L, 2017, J CANCER RES CLIN, V143, P2077, DOI 10.1007/s00432-017-2451-1

Wu ES, 2016, GYNECOL ONCOL, V140, P76, DOI 10.1016/j.ygyno.2015.11.013

Xie QK, 2018, CANCER MANAG RES, V10, P1365, DOI 10.2147/CMAR.S162147

Zhang HD, 2019, J CELL PHYSIOL, V234, P1794, DOI 10.1002/jcp.27052

Zhang J, 2013, MED ONCOL, V30, DOI 10.1007/s12032-012-0352-3

Zhao WW, 2017, BMC CANCER, V17, DOI 10.1186/s12885-017-3498-8

NR 26

TC 1

Z9 1

U1 2

U2 2

PU AME PUBL CO

PI SHATIN

PA FLAT-RM C 16F, KINGS WING PLAZA 1, NO 3 KWAN ST, SHATIN, HONG KONG

00000, PEOPLES R CHINA

SN 2072-1439

EI 2077-6624

J9 J THORAC DIS

JI J. Thorac. Dis.

PD JUN

PY 2019

VL 11

IS 6

BP 2332

EP 2339

DI 10.21037/jtd.2019.06.02

PG 8

WC Respiratory System

WE Science Citation Index Expanded (SCI-EXPANDED)

SC Respiratory System

GA IF7TN

UT WOS:000473287500028

PM 31372270

OA gold, Green Published

DA 2022-05-19

ER

PT J

AU Zhang, WC

Pang, QS

Yan, CH

Wang, QF

Yang, JS

Yu, SF

Liu, X

Yuan, ZY

Wang, P

Xiao, ZF

AF Zhang, Wencheng

Pang, Qingsong

Yan, Cihui

Wang, Qifeng

Yang, Jingsong

Yu, Shufei

Liu, Xiao

Yuan, Zhiyong

Wang, Ping

Xiao, Zefen

TI Induction of PD-L1 expression by epidermal growth factor

receptor-mediated signaling in esophageal squamous cell carcinoma

SO ONCOTARGETS AND THERAPY

LA English

DT Article

DE epidermal growth factor receptor; programmed death-ligand 1; esophageal

squamous cell carcinoma; immune checkpoint

ID LUNG-CANCER; IMMUNE ESCAPE; IFN-GAMMA; B7-H1 EXPRESSION; PROGNOSTIC

VALUE; OVARIAN-CANCER; LIGAND 1; EGFR; TUMOR; ACTIVATION

AB Purpose: The purpose of this study was to investigate the potential effect of activation of epidermal growth factor receptor (EGFR) signaling pathway on the expression of programmed death-ligand 1 (PD-L1) in esophageal squamous cell carcinoma (ESCC) cells with EGFR overexpression.

Methods: Flow cytometry and Western blot methods were used to assess PD-L1 expression on ESCC cells when EGFR signaling pathway was activated by epidermal growth factor (EGF) with or without EGFR-specific inhibitor AG-1478, and then EGFR signaling array was applied to analyze the potential signaling pathways involved.

Results: This study found that PD-L1 expression increased significantly in an EGFR-dependent manner by the activation of EGFR signaling and decreased sharply when EGFR signaling was blocked. The upregulated expression of PD-L1 was not associated with EGFR-STAT3 signaling pathway, but may be affected by EGFR-PI3K-AKT, EGFR-Ras-Raf-Erk, and EGR-PLC-gamma signaling pathways.

Conclusion: The expression of PD-L1 can be regulated by EGFR signaling activation in ESCC, which indicates an important role for EGFR-mediated immune escape and potential molecular pathways for EGFR-targeted therapy and immunotherapy.

C1 [Zhang, Wencheng; Pang, Qingsong; Yuan, Zhiyong; Wang, Ping] Tianjin Med Univ Canc Inst & Hosp, Natl Clin Res Ctr Canc, Dept Radiat Oncol, Tianjin, Peoples R China.

[Yan, Cihui] Tianjin Med Univ Canc Inst & Hosp, Natl Clin Res Ctr Canc, Dept Immunol, Key Lab Canc Prevent & Therapy, Tianjin, Peoples R China.

[Wang, Qifeng; Yang, Jingsong; Yu, Shufei; Liu, Xiao; Xiao, Zefen] Chinese Acad Med Sci, Canc Inst & Hosp, Dept Radiat Oncol, Pan Jia Yuan Nanli 17, Beijing 100021, Peoples R China.

[Wang, Qifeng; Yang, Jingsong; Yu, Shufei; Liu, Xiao; Xiao, Zefen] Peking Union Med Coll, Pan Jia Yuan Nanli 17, Beijing 100021, Peoples R China.

RP Xiao, ZF (corresponding author), Chinese Acad Med Sci, Canc Inst & Hosp, Dept Radiat Oncol, Pan Jia Yuan Nanli 17, Beijing 100021, Peoples R China.; Xiao, ZF (corresponding author), Peking Union Med Coll, Pan Jia Yuan Nanli 17, Beijing 100021, Peoples R China.; Wang, P (corresponding author), Tianjin Med Univ Canc Inst & Hosp, Natl Clin Res Ctr Canc, Dept Radiat Oncol, Key Lab Canc Prevent & Therapy, Huan Hu Xi Rd, Tianjin 300060, Peoples R China.

EM wangping@tjmuch.com; xiaozefen2013@163.com

RI Wang, Qifeng/AAX-7678-2021

OI Wang, Qifeng/0000-0001-8544-9007; Zhang, Wencheng/0000-0003-3730-5361

FU Science and Technology Project of Beijing [Z121107001012004]; Capital

Foundation for Medical Research and Development; National Natural

Science Foundation [81272512]; Chinese Hi-Tech RD Program [2012AA02A503]

FX This work was supported by the Science and Technology Project of Beijing

(Z121107001012004); the Capital Foundation for Medical Research and

Development (2007-2012); National Natural Science Foundation (81272512);

and Chinese Hi-Tech R&D Program (2012AA02A503).

CR Abiko K, 2015, BRIT J CANCER, V112, P1501, DOI 10.1038/bjc.2015.101

Akbay EA, 2013, CANCER DISCOV, V3, P1355, DOI 10.1158/2159-8290.CD-13-0310

Ayyappan S, 2013, ANTICANCER RES, V33, P4139

Azuma K, 2014, ANN ONCOL, V25, P1935, DOI 10.1093/annonc/mdu242

Borghaei H, 2015, NEW ENGL J MED, V373, P1627, DOI 10.1056/NEJMoa1507643

Butte MJ, 2007, IMMUNITY, V27, P111, DOI 10.1016/j.immuni.2007.05.016

Chen J, 2016, ANN ONCOL, V27, P409, DOI 10.1093/annonc/mdv615

Chen LJ, 2014, INT J CLIN EXP PATHO, V7, P6015

Chen MF, 2016, ONCOTARGET, V7, P7913, DOI 10.18632/oncotarget.6861

Chen N, 2015, J THORAC ONCOL, V10, P910, DOI 10.1097/JTO.0000000000000500

Chen R, 2016, TRANSL ONCOL, V9, P32, DOI 10.1016/j.tranon.2015.11.010

Concha-Benavente F, 2016, CANCER RES, V76, P1031, DOI 10.1158/0008-5472.CAN-15-2001

Crosby T, 2013, LANCET ONCOL, V14, P627, DOI 10.1016/S1470-2045(13)70136-0

Dayde D, 2016, ONCOGENE, V35, P3986, DOI 10.1038/onc.2015.480

Hamanishi J, 2016, INT J CLIN ONCOL, V21, P462, DOI 10.1007/s10147-016-0959-z

Herbst RS, 2016, LANCET, V387, P1540, DOI 10.1016/S0140-6736(15)01281-7

Iyer R, 2013, ONCOLOGY-BASEL, V85, P53, DOI 10.1159/000351617

Jiang DX, 2015, BMC CANCER, V15, DOI 10.1186/s12885-015-1393-8

Larkin J, 2015, NEW ENGL J MED, V373, P1270, DOI [10.1056/NEJMoa1504030, 10.1056/NEJMc1509660]

Lastwika KJ, 2016, CANCER RES, V76, P227, DOI 10.1158/0008-5472.CAN-14-3362

Leng CS, 2016, ONCOL REP, V35, P699, DOI 10.3892/or.2015.4435

Lienlaf M, 2016, MOL ONCOL, V10, P735, DOI 10.1016/j.molonc.2015.12.012

Luo Nian, 2015, Asian Pac J Cancer Prev, V16, P3509

Mandai M, 2016, CLIN CANCER RES, V22, P2329, DOI 10.1158/1078-0432.CCR-16-0224

Marzec M, 2008, P NATL ACAD SCI USA, V105, P20852, DOI 10.1073/pnas.0810958105

McDermott DF, 2013, CANCER MED-US, V2, P662, DOI 10.1002/cam4.106

Mellman I, 2011, NATURE, V480, P480, DOI 10.1038/nature10673

Mishra R, 2016, TISSUE ENG PART C-ME, V22, P904, DOI [10.1089/ten.tec.2016.0094, 10.1089/ten.TEC.2016.0094]

Mizukami T, 2015, INT J ONCOL, V47, P499, DOI 10.3892/ijo.2015.3050

Ota K, 2015, CLIN CANCER RES, V21, P4014, DOI 10.1158/1078-0432.CCR-15-0016

Parsa AT, 2007, NAT MED, V13, P84, DOI 10.1038/nm1517

Peng J, 2015, CANCER RES, V75, P5034, DOI 10.1158/0008-5472.CAN-14-3098

Pennathur A, 2013, LANCET, V381, P400, DOI 10.1016/S0140-6736(12)60643-6

Pivarcsi A, 2007, P NATL ACAD SCI USA, V104, P19055, DOI 10.1073/pnas.0705673104

Schalper KA, 2014, CLIN CANCER RES, V20, P2773, DOI 10.1158/1078-0432.CCR-13-2702

Sen M, 2015, NEOPLASIA, V17, P256, DOI 10.1016/j.neo.2015.01.003

Tanaka K, 2016, CANCER SCI, V107, P726, DOI 10.1111/cas.12938

Tang YN, 2015, ONCOTARGET, V6, P14209, DOI 10.18632/oncotarget.3694

Taube JM, 2012, SCI TRANSL MED, V4, DOI 10.1126/scitranslmed.3003689

Topalian SL, 2015, CANCER CELL, V27, P450, DOI 10.1016/j.ccell.2015.03.001

Velcheti V, 2014, LAB INVEST, V94, P107, DOI 10.1038/labinvest.2013.130

Wang J, 2014, ASIAN PAC J CANCER P, V15, P5889, DOI 10.7314/APJCP.2014.15.14.5889

Wang QF, 2013, WORLD J SURG ONCOL, V11, DOI 10.1186/1477-7819-11-278

Weber JS, 2015, LANCET ONCOL, V16, P375, DOI 10.1016/S1470-2045(15)70076-8

Zhang HH, 2016, CELL PHYSIOL BIOCHEM, V39, P360, DOI 10.1159/000445630

Zhang WC, 2014, ANN THORAC SURG, V98, P513, DOI 10.1016/j.athoracsur.2014.03.015

Zhang Y, 2014, ONCOTARGETS THER, V7, P567, DOI 10.2147/OTT.S59959

NR 47

TC 34

Z9 36

U1 2

U2 9

PU DOVE MEDICAL PRESS LTD

PI ALBANY

PA PO BOX 300-008, ALBANY, AUCKLAND 0752, NEW ZEALAND

SN 1178-6930

J9 ONCOTARGETS THER

JI OncoTargets Ther.

PY 2017

VL 10

BP 763

EP 771

DI 10.2147/OTT.S118982

PG 9

WC Biotechnology & Applied Microbiology; Oncology

WE Science Citation Index Expanded (SCI-EXPANDED)

SC Biotechnology & Applied Microbiology; Oncology

GA EK2GF

UT WOS:000393745100001

PM 28243112

OA Green Submitted, Green Published, gold

DA 2022-05-19

ER

PT J

AU Ichikawa, H

Kosugi, S

Kanda, T

Ishikawa, T

Yajima, K

Akazawa, K

Suzuki, T

Wakai, T

AF Ichikawa, Hiroshi

Kosugi, Shin-ichi

Kanda, Tatsuo

Ishikawa, Takashi

Yajima, Kazuhito

Akazawa, Kohei

Suzuki, Tsutomu

Wakai, Toshifumi

TI Prognostic Significance of Initial Recurrence Site in Hematogenous

Recurrence of Esophageal Squamous Cell Carcinoma

SO HEPATO-GASTROENTEROLOGY

LA English

DT Article

DE Esophageal squamous cell carcinoma; Hematogenous recurrence; Initial

recurrence site; Prognostic factor

ID EXTENDED RADICAL ESOPHAGECTOMY; CURATIVE RESECTION; CLINICAL-COURSE;

PATTERN; CANCER

AB Background/Aims: Hematogenous recurrences of esophageal squamous cell carcinoma (ESCC) have dismal prognoses, but prognostic heterogeneity exists in this disease. The objectives of this study were to clarify the prognosis in this disease with regard to the initial recurrence site and to define the prognostic factors. Methodology: We retrospectively reviewed the cases of 67 consecutive patients with hematogenous recurrence in major organs after esophagectomy for ESCC of the thoracic esophagus and the esophagogastric junction. We analyzed clinicopathological characteristics, survival probability and potential prognostic factors. Results: Lung, liver, bone, and multiple-organ metastases occurred in 24, 19, 14, and 10 patients, respectively. Twenty-seven patients simultaneously had locoregional recurrence (combined recurrence). Among all 67 patients, the median disease-free interval (DFI) was 9.7 months, and the median survival time after the initial recurrence was 4.9 months. The patients with initial lung metastasis had most favorable prognosis with the median survival time of 9.8 months. A multivariate analysis identified that initial recurrence site, DFI, combined recurrence, and anticancer therapy were independent prognostic factors. Conclusions: The initial recurrence site contributes to the prognostic heterogeneity of patients with hematogenous recurrence of ESCC. The prognostic factors identified in this study are useful to optimize the management of these patients.

C1 [Ichikawa, Hiroshi; Kosugi, Shin-ichi; Kanda, Tatsuo; Ishikawa, Takashi; Yajima, Kazuhito; Wakai, Toshifumi] Niigata Univ, Grad Sch Med & Dent Sci, Div Digest & Gen Surg, Niigata 9518510, Japan.

[Akazawa, Kohei] Niigata Univ, Med & Dent Hosp, Dept Med Informat, Niigata 9518510, Japan.

[Suzuki, Tsutomu] Niigata Univ, Sch Hlth Sci, Dept Nursing, Niigata 9518510, Japan.

RP Kosugi, S (corresponding author), Niigata Univ, Grad Sch Med & Dent Sci, Div Digest & Gen Surg, Chuo Ku, 1-757 Asahimachi Dori, Niigata 9518510, Japan.

EM sugishin@med.niigata-u.ac.jp

RI Kosugi, S./K-5142-2019

CR Bhansali MS, 1997, WORLD J SURG, V21, P275, DOI 10.1007/s002689900228

Carpizo DR, 2009, LANCET ONCOL, V10, P801, DOI 10.1016/S1470-2045(09)70081-6

Doki Y, 2005, WORLD J SURG, V29, P700, DOI 10.1007/s00268-005-7596-4

Dresner SM, 2000, BRIT J SURG, V87, P1426, DOI 10.1046/j.1365-2168.2000.01541.x

Gebski V, 2007, LANCET ONCOL, V8, P226, DOI 10.1016/S1470-2045(07)70039-6

Hofstetter W, 2002, ANN SURG, V236, P376, DOI 10.1097/00000658-200209000-00014

Hsu PK, 2011, J GASTROINTEST SURG, V15, P558, DOI 10.1007/s11605-011-1458-1

Hulscher JBF, 2000, J AM COLL SURGEONS, V191, P143, DOI 10.1016/S1072-7515(00)00349-5

Ichikawa H, 2011, SURGERY, V149, P164, DOI 10.1016/j.surg.2010.07.047

Mariette C, 2003, CANCER-AM CANCER SOC, V97, P1616, DOI 10.1002/cncr.11228

Miyata H, 2011, ANN SURG ONCOL, V18, P3353, DOI 10.1245/s10434-011-1747-7

Morita M, 2008, SURGERY, V143, P499, DOI 10.1016/j.surg.2007.12.007

Nakagawa S, 2004, J AM COLL SURGEONS, V198, P205, DOI 10.1016/j.jamcollsurg.2003.10.005

Natsugoe S, 2010, LANGENBECK ARCH SURG, V395, P341, DOI 10.1007/s00423-010-0592-7

Sobin LH., 2009, TNM CLASSIFICATION M, V7

Solomayer EF, 2000, BREAST CANCER RES TR, V59, P271, DOI 10.1023/A:1006308619659

Stein HJ, 2004, WORLD J SURG, V28, P520, DOI 10.1007/s00268-004-7417-1

Wagner AD, 2010, COCHRANE DB SYST REV, DOI 10.1002/14651858.CD004064.pub3

NR 18

TC 4

Z9 4

U1 0

U2 0

PU H G E UPDATE MEDICAL PUBLISHING S A

PI ATHENS

PA PO BOX 17257, ATHENS GR-10024, GREECE

SN 0172-6390

J9 HEPATO-GASTROENTEROL

JI Hepato-Gastroenterol.

PD NOV-DEC

PY 2014

VL 61

IS 136

BP 2241

EP 2246

DI 10.5754/hge13171

PG 6

WC Gastroenterology & Hepatology; Surgery

WE Science Citation Index Expanded (SCI-EXPANDED)

SC Gastroenterology & Hepatology; Surgery

GA AW5PK

UT WOS:000346326500019

PM 25699360

DA 2022-05-19

ER

PT J

AU Wu, KM

Wang, ZA

Huang, YL

Yao, L

Kang, NN

Ge, W

Zhang, RQ

He, W

AF Wu, Kaiming

Wang, Ziao

Huang, Yunlong

Yao, Long

Kang, Ningning

Ge, Wei

Zhang, Renquan

He, Wei

TI LncRNA PTPRG-AS1 facilitates glycolysis and stemness properties of

esophageal squamous cell carcinoma cells through miR-599/PDK1 axis

SO JOURNAL OF GASTROENTEROLOGY AND HEPATOLOGY

LA English

DT Article

DE Esophageal squamous cell carcinoma; Glycolysis; miR-599; PDK1; PTPRG-AS1

ID CANCER; PROLIFERATION; PROGRESSION; CERNA

AB Background and Aim Esophageal squamous cell carcinoma (ESCC) is the most significant subtype of esophageal cancer featured with high occurrence. Long noncoding RNAs (lncRNAs) have been proved to modulate the biological properties of cancer cells, including cell proliferation, invasion, migration, and apoptosis. LncRNA protein tyrosine phosphatase receptor type G-antisense RNA 1 (PTPRG-AS1) has been reported to play as an oncogene in diverse cancers. However, the detailed function PTPRG-AS1 may exert in ESCC is unclear. Methods PTPRG-AS1 expression in ESCC cells was investigated via quantitative reverse transcription real-time polymerase chain reaction (RT-qPCR). The effects of PTPRG-AS1 on ESCC cell proliferation, migration, glycolysis, and stemness were verified through functional assays. Mechanism assays including RIP assay, RNA pull down assay, and luciferase reporter assays were performed to verify the molecular mechanism of PTPRG-AS1. Results PTPRG-AS1 silencing hindered the proliferation, migration, glycolysis and stemness of ESCC cells. PTPRG-AS1 regulated pyruvate dehydrogenase kinase 1 (PDK1) expression via sponging miR-599. The PTPRG-AS1/miR-599/PDK1 axis was further verified to aggravate the progression of ESCC cells. Conclusion PTPRG-AS1 sponged miR-599 to up-regulate PDK1 expression, thereby promoting the proliferation and migration as well as glycolysis and stemness properties of ESCC cells.

C1 [Wu, Kaiming; Wang, Ziao; Huang, Yunlong; Yao, Long; Kang, Ningning; Ge, Wei; Zhang, Renquan] Anhui Med Univ, Affiliated Hosp 1, Dept Thorac Surg, 218 Ji Xi Rd, Hefei 230000, Anhui, Peoples R China.

[He, Wei] Anhui Med Univ, Sch Basic Med Sci, 81 Mei Shan Rd, Hefei 230032, Anhui, Peoples R China.

RP Zhang, RQ (corresponding author), Anhui Med Univ, Affiliated Hosp 1, Dept Thorac Surg, 218 Ji Xi Rd, Hefei 230000, Anhui, Peoples R China.; He, W (corresponding author), Anhui Med Univ, Sch Basic Med Sci, 81 Mei Shan Rd, Hefei 230032, Anhui, Peoples R China.

EM zhangrenquan@live.cn; weine@ahmu.edu.cn

CR Arnold M, 2015, GUT, V64, P381, DOI 10.1136/gutjnl-2014-308124

Baylin SB, 2016, CSH PERSPECT BIOL, V8, DOI 10.1101/cshperspect.a019505

Chang ZW, 2018, J EXP CLIN CANC RES, V37, DOI 10.1186/s13046-018-0724-4

Chen XX, 2018, CELL DEATH DIS, V9, DOI 10.1038/s41419-018-0962-6

Esquela-Kerscher A, 2006, NAT REV CANCER, V6, P259, DOI 10.1038/nrc1840

Ferlay J, 2015, INT J CANCER, V136, pE359, DOI 10.1002/ijc.29210

Ganapathy-Kanniappan S, 2013, MOL CANCER, V12, DOI 10.1186/1476-4598-12-152

Tallon AIG, 2012, MED CLIN-BARCELONA, V139, P588, DOI 10.1016/j.medcli.2012.05.024

Hu HB, 2018, EUR REV MED PHARMACO, V22, P1987, DOI 10.26355/eurrev_201804_14726

Iranpour M, 2016, TUMOR BIOL, V37, P2933, DOI 10.1007/s13277-015-4135-2

Jalali S, 2013, PLOS ONE, V8, DOI 10.1371/journal.pone.0053823

Kang M, 2018, J EXP CLIN CANC RES, V37, DOI 10.1186/s13046-018-0845-9

Li BG, 2018, LIFE SCI, V197, P122, DOI 10.1016/j.lfs.2018.02.006

Li X, 2018, INT J ONCOL, V53, P551, DOI 10.3892/ijo.2018.4418

Li Y, 2017, DIS MARKERS, V2017, DOI 10.1155/2017/5314649

Liang HH, 2018, MOL CANCER, V17, DOI 10.1186/s12943-018-0870-5

Liang Y, 2018, CELL DEATH DIFFER, V25, P1980, DOI 10.1038/s41418-018-0084-9

Liu H, 2018, J EXP CLIN CANC RES, V37, DOI 10.1186/s13046-018-0950-9

Liu YN, 2016, BIOINFORMATICS, V32, P1595, DOI 10.1093/bioinformatics/btw017

Ma F, 2017, J EXP CLIN CANC RES, V36, DOI 10.1186/s13046-017-0630-1

Najafi M, 2019, J CELL PHYSIOL, V234, P8381, DOI 10.1002/jcp.27740

Peng F, 2018, ONCOGENE, V37, P1062, DOI 10.1038/onc.2017.368

Pennathur A, 2013, LANCET, V381, P400, DOI 10.1016/S0140-6736(12)60643-6

Qi XL, 2015, J MED GENET, V52, P710, DOI 10.1136/jmedgenet-2015-103334

Qian HY, 2018, J MOL MED, V96, P753, DOI 10.1007/s00109-018-1654-5

Reid TD, 2012, CLIN ONCOL-UK, V24, P617, DOI 10.1016/j.clon.2012.02.001

Renganathan A, 2017, ADV EXP MED BIOL, V1008, P199, DOI 10.1007/978-981-10-5203-3_7

Semba H, 2016, NAT COMMUN, V7, DOI 10.1038/ncomms11635

Sugiura K., 2019, AM J PATHOL

Wang J, 2020, INT J CANCER, V146, P906, DOI 10.1002/ijc.32277

Wang Y, 2019, MOL CANCER, V18, DOI 10.1186/s12943-019-1105-0

Wei W, 2017, J CELL BIOCHEM, V118, P3349, DOI 10.1002/jcb.25988

Wu YY, 2017, MOL CANCER, V16, DOI 10.1186/s12943-017-0715-7

Yi L, 2019, J CELL PHYSIOL, V234, P19088, DOI 10.1002/jcp.28547

Yu J, 2019, MOL MED REP, V19, P5309, DOI 10.3892/mmr.2019.10213

Zhang M, 2018, J HEMATOL ONCOL, V11, DOI 10.1186/s13045-018-0656-7

Zhao LJ, 2017, CANCER RES, V77, P1369, DOI 10.1158/0008-5472.CAN-16-1615

Zhu H, 2018, BIOMED PHARMACOTHER, V105, P789, DOI 10.1016/j.biopha.2018.05.053

NR 38

TC 1

Z9 1

U1 5

U2 5

PU WILEY

PI HOBOKEN

PA 111 RIVER ST, HOBOKEN 07030-5774, NJ USA

SN 0815-9319

EI 1440-1746

J9 J GASTROEN HEPATOL

JI J. Gastroenterol. Hepatol.

PD MAR

PY 2022

VL 37

IS 3

BP 507

EP 517

DI 10.1111/jgh.15719

EA JAN 2022

PG 11

WC Gastroenterology & Hepatology

WE Science Citation Index Expanded (SCI-EXPANDED)

SC Gastroenterology & Hepatology

GA ZO6GA

UT WOS:000746511200001

PM 34676588

DA 2022-05-19

ER

PT J

AU Chen, M

Jin, DC

Wang, B

Gou, YJ

Dong, XC

AF Chen, Meng

Jin, Dacheng

Wang, Bing

Gou, Yunjiu

Dong, Xinchun

TI Identification of miRNAs as prognostic factors for esophageal squamous

cell carcinoma

SO MATHEMATICAL BIOSCIENCES AND ENGINEERING

LA English

DT Article

DE esophageal squamous cell carcinoma; microRNAs; prognosis; genes; tumor

ID GENOMIC ALTERATIONS; CANCER; MICRORNA; EXPRESSION; TROPHECTODERM;

TARGET; TEAD4

AB Objective: The prognostic value of microRNAs for esophageal squamous cell carcinoma (ESCC) is still not be well identified. Methods: The microRNA expression profiles of 119 paired ESCC tissue samples and para-carcinoma tissues from GEO database under accession number of GSE43732. A mutation information based feature selection method was applied to identify the discriminative microRNAs between paired ESCC tissues and para-carcinoma tissues. Results: In para-carcinoma tissues, patients had better survival with higher has-miR-410 (log-rank p = 0.0123), has-miR-411-5p (log-rank p = 0.0152), has-miR-193b-5p (log-rank p = 0.0188) and has-miR-4486 (log-rank p = 0.0307) expression levels. When compared with para-carcinoma tissues, there has more correlations between miRNA expression levels and survival in tumor tissues. We identified 20 potential miRNAs associated with prognosis. Besides, a heatmap was draw to explore miRNA expression levels in tumor tissues and survival. Conclusions: The present study identified 24 miRNAs in 119 paired ESCC tissue samples and para-carcinoma tissues, including 4 miRNAs in para-carcinoma tissues and 20 in tumor tissues, respectively. The dysregulation of these miRNAs were associated with different outcomes. We thought this study could provide novel noninvasive early biomarkers for ESCC patients.

C1 [Chen, Meng; Jin, Dacheng; Wang, Bing] Gansu Univ Chinese Med, Lanzhou 730000, Peoples R China.

[Chen, Meng; Jin, Dacheng; Wang, Bing; Gou, Yunjiu; Dong, Xinchun] Gansu Prov Hosp, Dept Thorac Surg, 204 Donggang West Rd, Lanzhou 730000, Peoples R China.

RP Gou, YJ; Dong, XC (corresponding author), Gansu Prov Hosp, Dept Thorac Surg, 204 Donggang West Rd, Lanzhou 730000, Peoples R China.

EM chuiji44751600@163.com; youzaibao8u@163.com

FU Hospital Research Foundation of Gansu Provincial people's Hospital;

Gansu Provincial Health Industry Plan

FX This work was supported by Hospital Research Foundation of Gansu

Provincial people's Hospital (No.l6gssy3-1); Gansu Provincial Health

Industry Plan (No.gswsky2017-56). Yunjiu Gou and Xinchun Dong designed

the study. Meng Chen was responsible for the collection and analysis of

the data. All authors drafted the manuscript and approved the final

version of the manuscript.

CR ALTMAN NS, 1992, AM STAT, V46, P175, DOI 10.2307/2685209

Chaudhry MA, 2014, J CELL BIOCHEM, V115, P436, DOI 10.1002/jcb.24694

Chen WQ, 2016, CA-CANCER J CLIN, V66, P115, DOI 10.3322/caac.21338

Chen ZL, 2014, CANCER LETT, V350, P34, DOI 10.1016/j.canlet.2014.04.013

Falzone L., 2019, SCI REP, V9, P6882

Falzone L, 2018, AGING-US, V10, P1000, DOI 10.18632/aging.101444

Fang R, 2019, FRONT PHYSIOL, V9, DOI 10.3389/fphys.2018.01879

Fesler A, 2014, ONCOTARGETS THER, V7, P7, DOI 10.2147/OTT.S65548

Guo Y, 2008, CANCER RES, V68, P26, DOI 10.1158/0008-5472.CAN-06-4418

Hu XH, 2019, CANCER BIOMARK, V25, P1, DOI 10.3233/CBM-180013

Kan CWS, 2015, GENE CHROMOSOME CANC, V54, P1, DOI 10.1002/gcc.22221

Kaneko KJ, 1998, DEV GENET, V22, P43, DOI 10.1002/(SICI)1520-6408(1998)22:1&lt;43::AID-DVG5&gt;3.0.CO;2-7

Li J, 2013, CANCER CELL INT, V13, DOI 10.1186/1475-2867-13-7

Liu T, 2019, J CELL BIOCHEM, V120, P7185, DOI 10.1002/jcb.27993

Liu Y, 2016, ONCOGENE, V35, P2789, DOI 10.1038/onc.2015.342

Martinez C, 2017, GUT, V66, P1597, DOI 10.1136/gutjnl-2016-311477

Nishioka N, 2008, MECH DEVELOP, V125, P270, DOI 10.1016/j.mod.2007.11.002

Nishioka N, 2009, DEV CELL, V16, P398, DOI 10.1016/j.devcel.2009.02.003

Rustgi AK, 2014, NEW ENGL J MED, V371, P2499, DOI 10.1056/NEJMra1314530

Shi Z, 2017, ONCOGENE, V36, P4362, DOI 10.1038/onc.2017.24

Slattery ML, 2015, INT J CANCER, V137, P428, DOI 10.1002/ijc.29384

Song YM, 2014, NATURE, V509, P91, DOI 10.1038/nature13176

Szpechcinski A., 2019, CELL MOL LIFE SCI, P1

Takeuchi S, 2017, BIOCHEM BIOPH RES CO, V486, P385, DOI 10.1016/j.bbrc.2017.03.050

Tan Y, 2019, CANCER EPIDEMIOL, V60, P67, DOI 10.1016/j.canep.2019.01.015

Tang J, 2019, J CELL PHYSL

van Schooneveld E, 2015, BREAST CANCER RES, V17, DOI 10.1186/s13058-015-0526-y

von Frowein J, 2018, LIVER INT, V38, P1280, DOI 10.1111/liv.13687

Wang SM, 2018, LEUKEMIA RES, V65, P61, DOI 10.1016/j.leukres.2018.01.004

Wang XS, 2019, J OVARIAN RES, V12, DOI 10.1186/s13048-019-0482-8

Wilke CM, 2018, INT J CANCER, V142, P573, DOI 10.1002/ijc.31072

Wong LL, 2019, J AM COLL CARDIOL, V73, P1300, DOI 10.1016/j.jacc.2018.11.060

Yang X, 2019, J CELL BIOCH

Zeybek A, 2019, CURR MOL MED, V19, P216, DOI 10.2174/1566524019666190314113800

Zhang HZ, 2012, CHIN J CANCER, V31, P281, DOI 10.5732/cjc.011.10390

Zhang Q, 2018, J CELL MOL MED, V22, P3560, DOI 10.1111/jcmm.13634

Zhao TH, 2013, BIOMED RES INT, V2013, DOI 10.1155/2013/414327

NR 37

TC 1

Z9 1

U1 0

U2 2

PU AMER INST MATHEMATICAL SCIENCES-AIMS

PI SPRINGFIELD

PA PO BOX 2604, SPRINGFIELD, MO 65801-2604 USA

SN 1547-1063

EI 1551-0018

J9 MATH BIOSCI ENG

JI Math. Biosci. Eng.

PY 2020

VL 17

IS 3

BP 2302

EP 2309

DI 10.3934/mbe.2020122

PG 8

WC Mathematical & Computational Biology

WE Science Citation Index Expanded (SCI-EXPANDED)

SC Mathematical & Computational Biology

GA KU7DN

UT WOS:000519873900022

PM 32233536

OA gold

DA 2022-05-19

ER

PT J

AU Fang, K

Chu, Y

Zhao, ZY

Li, QF

Li, HQ

Chen, T

Xu, MD

AF Fang, Kang

Chu, Yuan

Zhao, Ziying

Li, Qinfang

Li, Hongqi

Chen, Tao

Xu, Meidong

TI Enhanced expression of asparagine synthetase under glucose-deprived

conditions promotes esophageal squamous cell carcinoma development

SO INTERNATIONAL JOURNAL OF MEDICAL SCIENCES

LA English

DT Article

DE esophageal squamous cell carcinoma; cancer development; asparagine

synthetase; glucose-deprivation

ID CANCER; GENE; ACTIVATION; APOPTOSIS; NRF2

AB Background: Cancer cells survive and develop under nutrient deficient microenvironment caused by low blood supply. Although anaerobic metabolism could function through the enhanced uptake of glucose, other mechanisms of tolerance to glucose deficient conditions might be required.

Materials and Methods: Expression of asparagine synthetase (ASNS) under normal glucose and glucose-deprived conditions was examined. Cancer cell proliferation and migration were evaluated by in vitro and in vivo assays. In addition, the relationship between ASNS expression and cancer stages was also analyzed.

Results: Expression of ASNS was enhanced under glucose deficient conditions. In vitro assays indicated that ASNS could promote the proliferation and migration abilities of esophageal squamous cell carcinoma (ESCC) cells under glucose deficient condition. In mechanism, 2 critical effectors during nutrient deprivation, NRF2 and ATF4, were upregulated and demonstrated to promote ASNS expression. Clinically, high level of ASNS was significantly associated with ESCC with advanced stages and metastasis. In vivo, ASNS could promote tumor growth and metastasis in mouse xenograft models.

Conclusion: This study uncovered that glucose deprivation induces the overexpression of ASNS in ESCC cells, which in turn causes cancer cell tolerance to nutrient stress and promotes cancer development. The illustration of the mechanism sheds deep insight on how cell biology was regulated in response to the conditions of limited nutrient availability.

C1 [Fang, Kang; Chu, Yuan; Zhao, Ziying; Li, Qinfang; Li, Hongqi; Chen, Tao; Xu, Meidong] Tongji Univ, Shanghai East Hosp, Endoscopy Ctr, Sch Med, Shanghai, Peoples R China.

RP Chen, T; Xu, MD (corresponding author), Tongji Univ, East Hosp, Endoscopy Ctr, Sch Med, Shanghai 200120, Peoples R China.

EM chentao@tongji.edu.cn; xumeidong@aliyun.com

FU National Natural Science Foundation of China [81570595, 81872351];

Shanghai Science and Technology Committee [18140900100, 17140901100,

18140900101, 18140900102]

FX This work was supported by the [National Natural Science Foundation of

China #1] under Grant [number 81570595 and 81872351]; [Shanghai Science

and Technology Committee #2] under Grant [number 18140900100,

17140901100, 18140900101 and 18140900102].

CR Ameri K, 2008, INT J BIOCHEM CELL B, V40, P14, DOI 10.1016/j.biocel.2007.01.020

Barbosa-Tessmann IP, 1999, BIOCHEM J, V339, P151, DOI 10.1042/0264-6021:3390151

Barbosa-Tessmann IP, 1999, J BIOL CHEM, V274, P31139, DOI 10.1074/jbc.274.44.31139

Batool T, 2016, APPL BIOCHEM BIOTECH, V178, P900, DOI 10.1007/s12010-015-1917-3

Brown JM, 1998, CANCER RES, V58, P1408

Chen T, 2017, NAT COMMUN, V8, DOI 10.1038/s41467-017-01024-2

Cui HY, 2007, CANCER RES, V67, P3345, DOI 10.1158/0008-5472.CAN-06-2519

Dey S, 2015, J CLIN INVEST, V125, P2592, DOI 10.1172/JCI78031

Enzinger PC, 2003, NEW ENGL J MED, V349, P2241, DOI 10.1056/NEJMra035010

Fafournoux P, 2000, BIOCHEM J, V351, P1, DOI 10.1042/0264-6021:3510001

Greijer AE, 2004, J CLIN PATHOL, V57, P1009, DOI 10.1136/jcp.2003.015032

Gwinn DM, 2018, CANCER CELL, V33, P91, DOI 10.1016/j.ccell.2017.12.003

Hockel M, 1999, CANCER RES, V59, P4525

Lee KM, 2013, CANCER LETT, V330, P225, DOI 10.1016/j.canlet.2012.11.053

Malhotra R, 1999, J BIOL CHEM, V274, P12567, DOI 10.1074/jbc.274.18.12567

Papandreou I, 2005, CANCER RES, V65, P3171, DOI 10.1158/0008-5472.CAN-04-3395

Richards NGJ, 1998, ADV ENZYMOL RAMB, V72, P145

Sporn MB, 2012, NAT REV CANCER, V12, P564, DOI 10.1038/nrc3278

Zhang J, 2014, MOL CELL, V56, P205, DOI 10.1016/j.molcel.2014.08.018

NR 19

TC 0

Z9 0

U1 1

U2 3

PU IVYSPRING INT PUBL

PI LAKE HAVEN

PA PO BOX 4546, LAKE HAVEN, NSW 2263, AUSTRALIA

SN 1449-1907

J9 INT J MED SCI

JI Int. J. Med. Sci.

PY 2020

VL 17

IS 4

BP 510

EP 516

DI 10.7150/ijms.39557

PG 7

WC Medicine, General & Internal

WE Science Citation Index Expanded (SCI-EXPANDED)

SC General & Internal Medicine

GA KN5BF

UT WOS:000514851600010

PM 32174781

OA Green Published, gold

DA 2022-05-19

ER

PT J

AU Shen, L

Zhu, H

Ding, SG

Xu, YM

Zhong, CJ

Zheng, SY

AF Shen, Liang

Zhu, Hao

Ding, Shengguang

Xu, Yiming

Zhong, Chongjun

Zheng, Shiying

TI MiR-19b promotes growth and migration of human esophageal squamous cell

carcinoma Eca109 cells by negatively regulating PTEN

SO INTERNATIONAL JOURNAL OF CLINICAL AND EXPERIMENTAL MEDICINE

LA English

DT Article

DE miR-19b; proliferation and migration; ESCC; PTEN

ID TUMOR-SUPPRESSOR; CANCER CELLS; IN-SITU; PROLIFERATION; SURVIVAL;

OVEREXPRESSION; METASTASIS; BIOGENESIS; EXPRESSION; MIRNA

AB Esophageal cancer is one of the most common malignant tumors, and it makes a leading cause of death worldwide. Esophageal squamous cell carcinoma (ESCC) is the main histological characteristic of esophageal cancer in China, and it has a poor prognosis. Therefore, novel therapeutic strategies for ESCC are highly needed. MiR-19b is a critical regulator for development of tumors. However, its functional roles in ESCC are rarely elucidated. In our study, we reported that miR-19b accelerates growth and migration of ESCC cells by negatively regulating PTEN. First, Eca109 cells were transfected with miR-19b mimics, inhibitors or their respective negative controls. Second, proliferative role of cells was evaluated by Cell Counting Kit-8 (CCK-8) and EdU incorporation assay, while cell migration was determined by the width of the initial scratched area. Third, PTEN was confirmed as a target of miR 19b by bioinformatics analysis. Finally, knockdown of PTEN was able to promote cell multiplication and migration of Eca109 cells, while the growth inhibiting effect of miR-19b inhibitor in both proliferative and migratory roles of Eca109 cells could be hindered by PTEN siRNAs. Taken together, our present work indicates that inhibition of miR-19b might be a new therapeutic strategy for esophageal squamous cell carcinoma (ESCC).

C1 [Shen, Liang; Zheng, Shiying] Soochow Univ, Affiliated Hosp 1, Dept Thorac Surg, Shizi St 188, Suzhou 215006, Jiangsu, Peoples R China.

[Shen, Liang; Zhu, Hao; Ding, Shengguang; Xu, Yiming; Zhong, Chongjun] Nantong Univ, Affiliated Hosp 2, Dept Thorac & Cardiovasc Surg, Nantong 226001, Peoples R China.

RP Zheng, SY (corresponding author), Soochow Univ, Affiliated Hosp 1, Dept Thorac Surg, Shizi St 188, Suzhou 215006, Jiangsu, Peoples R China.

EM syzheng88@sina.com

FU Nantong municipal science and technology projects [HS2013066,

MS2015083]; Youth Fund Project of Nantong Health Bureau [WQ2016044];

Science and Technology Guiding Program of Nantong [YYZ17035]

FX The authors are grateful to the fund support by Nantong municipal

science and technology projects (Grant NO, HS2013066 to Dr C Zhong) and

(Grant NO, MS2015083 to Dr Y Xu). The present study was also supported

by the Youth Fund Project of Nantong Health Bureau (Grant No: WQ2016044

to S. Ding) and The Science and Technology Guiding Program of Nantong

(Project No: YYZ17035 to L. Shen).

CR Anderegg MCJ, 2014, BEST PRACT RES CL GA, V28, P41, DOI 10.1016/j.bpg.2013.11.002

Barr FG, 2009, GENE CHROMOSOME CANC, V48, P661, DOI 10.1002/gcc.20673

Bartel DP, 2004, CELL, V116, P281, DOI 10.1016/S0092-8674(04)00045-5

Berindan-Neagoe I, 2014, CA-CANCER J CLIN, V64, P311, DOI 10.3322/caac.21244

Calura E, 2013, CLIN CANCER RES, V19, P4114, DOI 10.1158/1078-0432.CCR-13-0360

Chen JQ, 2012, INT J RADIAT ONCOL, V82, P475, DOI 10.1016/j.ijrobp.2010.08.037

Chen WQ, 2016, CA-CANCER J CLIN, V66, P115, DOI 10.3322/caac.21338

de la Pena FA, 2011, J BIOL CHEM, V286, P20778, DOI 10.1074/jbc.M110.198069

Ding DP, 2011, CARCINOGENESIS, V32, P1025, DOI 10.1093/carcin/bgr078

Ferlay J, 2015, INT J CANCER, V136, pE359, DOI 10.1002/ijc.29210

Gu YY, 2017, PROTEIN CELL, V8, P455, DOI 10.1007/s13238-017-0393-7

Harata K, 2010, ONCOL LETT, V1, P685, DOI 10.3892/ol_00000120

Jia ZF, 2013, PATHOL ONCOL RES, V19, P847, DOI 10.1007/s12253-013-9653-x

Jiang T, 2017, J EXP CLIN CANC RES, V36, DOI 10.1186/s13046-017-0602-5

Jiang YQ, 2010, ANN THORAC SURG, V90, P908, DOI 10.1016/j.athoracsur.2010.05.060

Jiao XY, 2013, BREAST CANCER RES TR, V139, P717, DOI 10.1007/s10549-013-2607-x

Kong KL, 2012, GUT, V61, P33, DOI 10.1136/gutjnl-2011-300178

Kurashige J, 2012, BRIT J CANCER, V106, P182, DOI 10.1038/bjc.2011.509

Lee KH, 2009, EXP CELL RES, V315, P2529, DOI 10.1016/j.yexcr.2009.06.001

Li HP, 2014, J TRANSL MED, V12, DOI 10.1186/1479-5876-12-196

Li J, 2015, LAB INVEST, V95, P1056, DOI 10.1038/labinvest.2015.76

Li XT, 2014, PHYTOTHER RES, V28, P1553, DOI 10.1002/ptr.5167

Liu LP, 2015, GUT, V64, P26, DOI 10.1136/gutjnl-2013-306388

Liu YJ, 2016, ANN NY ACAD SCI, V1381, P66, DOI 10.1111/nyas.13144

MacFarlane LA, 2010, CURR GENOMICS, V11, P537, DOI 10.2174/138920210793175895

Medina PP, 2010, NATURE, V467, P86, DOI 10.1038/nature09284
[truncated: 43,504,604 more chars]
